# Identification of Methylation Signatures and Rules for Sarcoma Subtypes by Machine Learning Methods

**DOI:** 10.1155/2022/5297235

**Published:** 2022-12-28

**Authors:** Jingxin Ren, XianChao Zhou, Wei Guo, KaiYan Feng, Tao Huang, Yu-Dong Cai

**Affiliations:** ^1^School of Life Sciences, Shanghai University, Shanghai 200444, China; ^2^Center for Single-Cell Omics, School of Public Health, Shanghai Jiao Tong University School of Medicine, Shanghai, China; ^3^Key Laboratory of Stem Cell Biology, Shanghai Jiao Tong University School of Medicine (SJTUSM) & Shanghai Institutes for Biological Sciences (SIBS), Chinese Academy of Sciences (CAS), Shanghai 200030, China; ^4^Department of Computer Science, Guangdong AIB Polytechnic College, Guangzhou 510507, China; ^5^Bio-Med Big Data Center, CAS Key Laboratory of Computational Biology, Shanghai Institute of Nutrition and Health, University of Chinese Academy of Sciences, Chinese Academy of Sciences, Shanghai 200031, China; ^6^CAS Key Laboratory of Tissue Microenvironment and Tumor, Shanghai Institute of Nutrition and Health, University of Chinese Academy of Sciences, Chinese Academy of Sciences, Shanghai 200031, China

## Abstract

Sarcoma, the second common type of solid tumor in children and adolescents, has a wide variety of subtypes that are often not properly diagnosed at an early stage, leading to late metastases and causing serious loss of life and property to patients and families. It exhibits a high degree of heterogeneity at the cellular, molecular, and epigenetic levels, where DNA methylation has been proposed to play a role in the diagnosis of sarcoma subtypes. Thus, this study is aimed at finding potential biomarkers at the DNA methylation level to distinguish different sarcoma subtypes. A machine learning process was designed to analyse sarcoma samples, each of which was represented by lots of methylation sites. Irrelevant sites were removed using the Boruta method, and remaining sites related to the target variables were kept for further analyses. Afterward, three feature ranking methods (LASSO, LightGBM, and MCFS) were adopted to rank these features, and six classification models were constructed by combining incremental feature selection and two classification algorithms (decision tree and random forest). Among these models, the performance of RF model was higher than that of DT model under all three ranking conditions. The specific expression of genes obtained from the annotation of highly correlated methylation site features, such as PRKAR1B, INPP5A, and GLI3, was proven to be associated with sarcoma by publications. Moreover, the quantitative rules obtained by decision tree algorithm helped us to understand the essential differences between various sarcoma types and classify sarcoma subtypes, providing a new means of clinical identification and determining new therapeutic targets.

## 1. Introduction

Sarcomas are a heterogeneous group of mesenchymal neoplasms with a high incidence in children, and they can be divided into two categories: soft tissue sarcomas and primary osteosarcomas based on the anatomical site of occurrence [1]. A detailed taxonomic approach classifies sarcomas from hundreds of different bone and soft tissue types into more than 60 sarcoma subtypes based on clinical features, as well as genetic and molecular data [2]. For example, the traditional diagnosis of leiomyosarcoma has been based on the characteristic pathological features of hematoxylin and eosin staining [3]. Recent studies have revealed that leiomyosarcoma can be further classified into three molecular subtypes with different prognoses based on expression profiles [4]. Ewing sarcoma can be observed histologically as small round cells with high CD99 expression and a genetic signature of balanced chromosomal translocation, with EWSR1-FLI1 fusion occurring in approximately 85% of patients [5]. Synovial sarcoma was first defined as a fusion of the SS18 gene on chromosome 18 with several synovial sarcoma genes on chromosome 18, and it can be further classified into subtypes with different treatment responses and prognoses based on their histological features and gene expression characteristics [6]. However, the lack of evident symptoms in the early stage of sarcoma leads to delayed diagnosis and late metastasis, resulting in huge loss of life [7]. At present, about half of sarcomas lack significant tumor-specific pathological or marker changes. Thus, the correct diagnosis and effective treatment of sarcomas remain limited [8, 9].

DNA methylation is an important mechanism of transcriptional regulation in mammals, which appropriately regulates gene expression through epigenetic modifications in normal cells. However, considerable evidence indicates that DNA methylation plays an important role in carcinogenesis [10]. Researchers propose that DNA methylation can be used as a powerful biomarker of human cancer and applied in cancer diagnosis [11]. Several central nervous system (CNS) tumor types are identified, making standardization of the diagnostic process challenging. Studies have shown that DNA methylation profiles can improve the diagnostic accuracy of CNS tumors, indicating its great application potential [12]. Moreover, other studies have shown that DNA methylation profiles have an important classification and diagnostic or prognostic role in a variety of solid tumors [13, 14]. With the progress of research, a variety of commercially available DNA methylation biomarkers are identified, bringing new breakthroughs to cancer diagnosis [15]. In the field of sarcoma research, growing evidence shows that sarcomas are epigenetic diseases [16, 17]. DNA methylation, an extensively studied epigenetic alteration, has also played an important role in sarcoma. Recently, numerous studies have indicated that DNA methylation of soft tissue sarcoma and osteosarcoma subtypes has specific features and diagnostic potential [18–21]. Some histologically indistinguishable or indistinguishable sarcoma subtypes have specific methylation signatures; thus, the methylation signature of sarcoma is a potential tool for sarcoma classification and diagnosis [22].

In this study, an efficient machine learning based method was designed to investigate 59 sarcoma subtypes. The methylation profile on sarcoma samples retrieved from Gene Expression Omnibus (GEO) was deeply analysed by this method. In brief, the methylation features were first analysed by Boruta method [23] to exclude irrelevant features and select important features. Then, the selected features were investigated by three feature ranking algorithms (least absolute shrinkage and selection operator (LASSO) [24], light gradient boosting machine (LightGBM) [25], and Monte Carlo feature selection (MCFS) [26]). Three feature lists were generated, which were fed into incremental feature selection (IFS) method [27], incorporating decision tree (DT) [28], or random forest (RF) [29], to obtain essential methylation sites, efficient classification models and rules. Some genes (PRKAR1B, INPP5A, and GLI3), corresponding to essential methylation sites, and classification rules were discussed to confirm the reliability of the findings in this study. The results reported in this study can provide additional evidence for the specificity of DNA methylation of different sarcoma subtypes and highlight the potential application of methylation signatures in sarcoma diagnosis.

## 2. Materials and Methods

**Figure 1:**
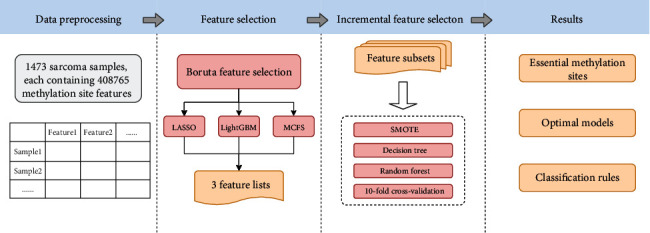
Flow chart of the whole analysis process. Methylation site features from sarcoma samples were analysed by Boruta, and remaining features were ranked in accordance with their relevance with three feature ranking algorithms, namely, LASSO, LightGBM, and MCFS. Subsequently, three ordered feature lists were fed into the incremental feature selection computational framework to access essential methylation sites, models with high performance, and quantitative classification rules.

The machine learning-based research process is shown in Figure 1. It can be summarized as follows: the methylation sites of the samples were used as features, feature screening was performed using the Boruta method, and feature ranking was performed using three methods. Finally, key biomarkers and quantitative classification rules were identified using IFS method.

### 2.1. Data

This study is aimed at accurately classifying different sarcoma types. We obtained gene methylation profile data from a total of 1,473 sarcoma samples from the GEO database with accession number GSE140686 [30]. These samples were classified into a total of 59 different sarcoma subtypes, and each sample was represented by 408,765 methylation sites. 59 sarcoma subtypes and their sample sizes are shown in Table S1. In this study, the subtypes were deemed as labels of samples, and methylation sites were considered as features. The novel findings can be identified by investigating such classification problem. The purpose was to extract essential methylation sites and patterns for different sarcoma subtypes. At the same time, efficient classification models were built to correctly classify sarcoma samples.

### 2.2. Boruta Feature Filtering

Removing redundant features that are less helpful to the identification could prevent noise in subsequent modelling. Lots of methylation features were involved in the investigated dataset. It is necessary to exclude irrelevant features. Here, the Boruta method was adopted [23].

Boruta method can filter out key features that are correlated with the dependent variable, regardless of its strong or weak correlation with the dependent variable. This method is based on the RF [23]. First, it shuffles the original feature list, introduces randomness, and generates a random combination of shadow features. The shadow feature list is merged with the original feature list and stitched into an expanded dataset to train a RF model, and features are assigned scores according to their importance. In each iteration, the score of an actual feature is checked to see if it outperforms the highest score of the shadow features. If a feature is positive for classification, then it must be more important than its random version. Therefore, an actual feature is marked as “important” if its score is significantly higher than the scores of shadow features. Then, all “important” variables and shadow features are removed. The updated data is fed into the next round. This procedure is repeated several times until all actual features are marked or a predetermined number of iterations are reached. The features marked as “important” are picked up as the output of the Boruta.

The Boruta program used in this study was obtained from https://github.com/scikit-learn-contrib/boruta_py [31] and was executed using default parameters.

### 2.3. Feature Ranking Algorithms

The Boruta method helps us extract important features. However, it cannot further identify which features are more important. We further employed three algorithms to rank remaining features, including LASSO [24], LightGBM [25], and MCFS [26].

#### 2.3.1. Least Absolute Shrinkage and Selection Operator

Based on the nonnegative garrote proposed by Breiman [32], Tibshirani first proposed the LASSO algorithm in 1996 [24]. As a regression analysis method, it exhibits feature selection and regularization, helping us to improve the accuracy and interpretability of statistical models. The method uses the L1-type regularization term or wavelength (*λ*) to obtain sparse results and determines the correlation by penalizing the coefficients of features. The coefficients of irrelevant and redundant features were zero, whereas those of relevant features are nonzero. Features with nonzero coefficients are retained. In addition, the magnitude of the absolute value of regression coefficients is proportional to the importance of the features, which is used to generate the feature ranking list. Such list was called LASSO feature list for convenience. We used LASSO program integrated in scikit-learn package in Python with default parameters.

#### 2.3.2. Light Gradient Boosting Machine

The LightGBM is a gradient boosting DT framework proposed by a research team from Microsoft and Peking University in 2017 [25]. LightGBM introduces gradient one-sided sampling (GOSS), exclusive feature bundling (EFB), and histogram algorithm. GOSS splits the sample based on the absolute value of the sample gradient, reduces the dimensionality of the sample features by bundling them with EFB, using a leaf-wise node splitting strategy different from that used in previous DTs, and finally calculates the importance of each feature. The developers describe various advantages of this algorithm, including faster training with high accuracy, smaller memory footprint, and support for parallel learning with direct feature classification, which are excellent when dealing with large-scale data. Features are ranked in a list according to their occurrence in DTs, which is called LightGBM feature list in this study. Here, we used LightGBM program implemented by Python, which can be obtained from https://lightgbm.readthedocs.io/en/latest/, and default parameters were adopted.

#### 2.3.3. Monte Carlo Feature Selection

MCFS was proposed in 2008 by Dramiński et al. [26]. It is based on the original dataset and several subsets of features that are randomly selected to form a number of DT classifiers. The importance of each feature is determined on the basis of its involvement in the tree classifiers. It is determined by a measurement, named, relative importance score (RI)

(1) $$\begin{array}{c} \text{R}{\text{I}}_{g}=\overset{p\times t}{\underset{\tau =1}{\sum }}{\left(\omega A_{CC}\right)}^{u}\underset{n_{g}\left(\tau \right)}{\sum }IG\left(n_{g}\left(\tau \right)\right){\left(\frac{no.\text{in }n_{g}\left(\tau \right)}{no.\text{in }\tau }\right)}^{v} \end{array}$$

where *ωA*_*CC*_ is the weighted accuracy of all samples; *n*_*g* (*τ*) is a feature node of the DT *τ* related to feature *g*, whose information acquisition is denoted as *IG*(*n*_*g* (*τ*)); *no*.in *n*_*g* (*τ*) and *no*.in *τ* denote the training sample size in *n*_*g* (*τ*) and the root of *τ*; *u* and *v* are conventional coefficients indicating the significance of the weights. By default, *u* and *v* are set to 1. Based on the results of the MCFS, the features can be ranked in accordance with the decreasing order of their RI values as a higher RI indicates that a feature is more important. Such list was called MCFS feature list. This study adopted the MCFS program from http://www.ipipan.eu/staff/m.draminski/mcfs.html with default parameters.

### 2.4. Incremental Feature Selection

Using the three above mentioned algorithms, a total of three feature lists were obtained, all of which represent the importance ranking of each feature under the corresponding method rule. However, the selection of most important features is still a problem. We do not known how many top features in each list can be selected. Thus, IFS method [27] was adopted to analyse each list.

In the IFS method, the feature list, denoted by *F* = [*f*_1_, *f*_2_, ⋯, *f*_*n*_], is transformed into a series of feature subsets, each of which has 10 more features than the previous subset. The first subset *F*_1_ contains the top 10 features in the list (i.e., *F*_1_ = {*f*_1_, *f*_2_, ⋯, *f*_10_}); the second subset *F*_2_ contains the top 20 features (i.e., *F*_2_ = {*f*_1_, *f*_2_, ⋯, *f*_20_}), and so on. For each subset, just the features from it are used to build a model based on one classification algorithm. Its performance is evaluated by cross-validation [33]. After all models have been tested, the model with best performance can be obtained. This model was termed as the optimal model and features used in this model constituted the optimal features.

### 2.5. Synthetic Minority Oversampling Technique

By checking the distribution of samples in 59 sarcoma subtypes (Table S1), the largest subtype contained much more samples than the smallest subtype. The large disparity in the number of samples of different sarcoma subtypes in the dataset can lead to biased results as the trained model develops a preference for some of the categories with a high number of samples. The synthetic minority oversampling technique (SMOTE) [34] was used in this study to balance the dataset.

The SMOTE determines the *k*-nearest neighbors for one sample, say *x*, in the minority class by calculating the Euclidean distance of that sample to other samples in the same minority class. A sample, say *y*, is randomly selected from the *k*-nearest neighbors. A point in the concatenation of *x* and *y* is identified as the new constructed sample, which putted into the same minority class. The process is repeated several times until the minority class has the same capacity as the majority class. After all minority classes have been considered, a balanced dataset can be obtained.

We used the SMOTE program downloaded from https://github.com/scikitlearn-contrib/imbalanced-learn, using the default parameters.

### 2.6. Classification Algorithm

In the present study, IFS was performed using two classification algorithms, namely, DT [28] and RF [29], which are widely used in life science [35–39].

#### 2.6.1. Decision Tree

The DT can be presented by a tree-like structure. Each internal node in this structure represents the judgment of one feature, and the different results are output in the form of tree branches, after which the next node is moved to a new feature. All samples starts from the root node, and the judgment is repeated until all samples reach the leaf node. The leaf nodes represent the final classification results for the sample categories. DT has various advantages, including the high classification accuracy, simplicity of the generated patterns, and ease of understanding and interpretation. In this study, we used the CART classification tree algorithm with node ranking by the Gini coefficient. The program was obtained from the scikit-learn package, and the default parameters were used for execution.

#### 2.6.2. Random Forest

The RF algorithm is an ensemble learning method based on DT, which introduces randomness for selecting samples and features. RF can handle high-dimensional data, and it has higher accuracy compared with a single classifier because it is an integrated algorithm. In addition, it prevents RF classifiers from overfitting and makes such classifier noise resistant because of the introduction of randomness. The RF program in the scikit-learn package was used in this study and performed using default parameters.

### 2.7. Performance Evaluation

The F1 score commonly used in machine learning evaluates the predictive ability of all models [40–42]. In this multiclassification problem, the first step is to calculate the precision and recall of each category. They can be computed as follows

(2) $$\begin{array}{c} {\text{Precision}}_{i}=\frac{{\text{TP}}_{i}}{{\text{TP}}_{i}+{\text{FP}}_{i}} \end{array}$$

(3) $$\begin{array}{c} {\text{Recall}}_{i}=\frac{{\text{TP}}_{i}}{{\text{TP}}_{i}+{\text{FN}}_{i}} \end{array}$$

where *TP*_*i*_, *FP*_*i*_ and *FN*_*i*_ represent true positives, false positives, and false negatives for the *i*-th category. Then the *F*1 score for the *i*-th category can be computed by

(4) $$\begin{array}{c} F1\text{ scor}{\text{e}}_{i}=\frac{2\times {\text{Precision}}_{i}\times {\text{Recall}}_{i}}{{\text{Precision}}_{i}+{\text{Recall}}_{i}} \end{array}$$

Aggregating the *F*1 scores of all categories can describe the overall performance of the classifier. If all *F*1 scores are equal, the macro *F*1 can be obtained. The weighted *F*1 further considers the weights of *F*1 scores on different categories. Clearly, weighted *F*1 can give a more objective evaluation of models' performance. Thus, it was selected as the major measurement.

In addition, other widely used measurements were also employed in this study, including overall accuracy (ACC) and Matthews' correlation coefficient (MCC) [43]. The ACC is defined as the proportion of correctly predicted samples and MCC can be calculated by

(5) $$\begin{array}{c} MCC=\frac{\mathrm{cov}\left(X,Y\right)}{\mathrm{cov}\left(X,X\right)\mathrm{cov}\left(Y,Y\right)} \end{array}$$

where *X* and *Y* are two binary matrices storing the actual and predicted classes of all samples, and cov(*X*, *Y*) stands for the covariance of *X* and *Y*.

## 3. Results

Based on the process shown in Figure 1, we screened and extracted key features that can characterize different sarcoma subtypes and established quantitative rules for their depictions and classification. The results of various stages of the entire computational process are summarized in the following section.

### 3.1. Boruta Feature Selection and Feature Ranking

Firstly, the methylation sites were streamlined using Boruta method. Irrelevant methylation sites were removed. Table S2 shows the final selection of 8954 features. Then, the features resulting from Boruta filtering were ranked using three algorithms, resulting in three feature lists: LASSO, LightGBM, and MCFS feature lists. These lists are also shown in Table S2.

### 3.2. IFS Results

**Figure 2:**
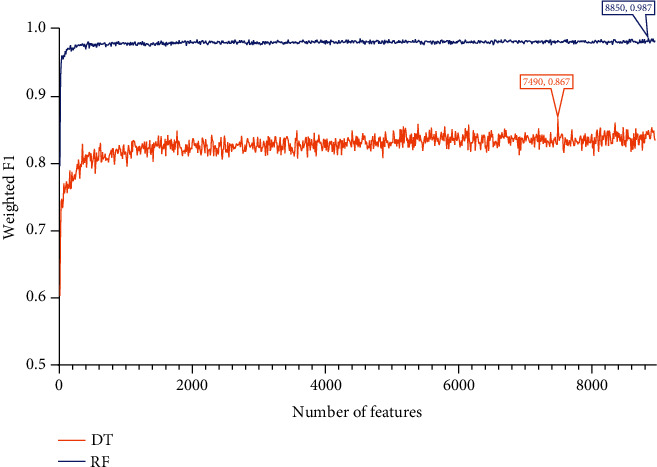
IFS curves showing the performance of decision tree (DT) and random forest (RF) based on the weighted *F*1 under different feature subsets derived from the LASSO feature list. The optimal DT/RF model yielded the weighted *F*1 of 0.867/0.987.

**Figure 3:**
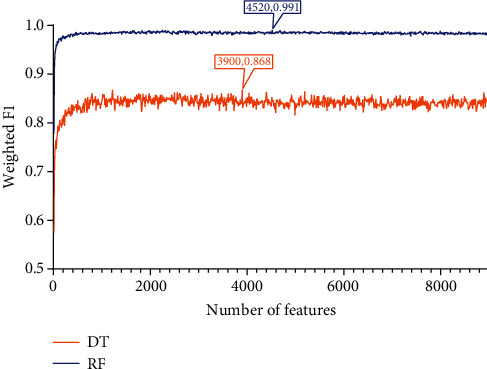
IFS curves showing the performance of decision tree (DT) and random forest (RF) based on the weighted *F*1 under different feature subsets derived from the LightGBM feature list. The optimal DT/RF model yielded the weighted F1 of 0.868/0.991.

**Figure 4:**
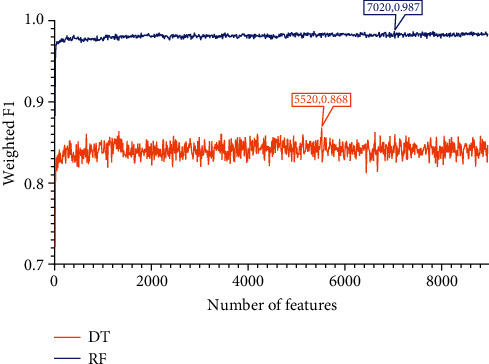
IFS curves showing the performance of decision tree (DT) and random forest (RF) based on the weighted *F*1 under different feature subsets derived from the MCFS feature list. The optimal DT/RF model yielded the weighted *F*1 of 0.868/0.987.

Apply IFS to each obtained list to construct a number of subsets with an interval of 10. Based on each feature subset, two models, based on DT and RF, were constructed. During this process, the number of minority class samples was supplemented using the SMOTE method, and the performance of all constructed models was evaluated using 10-fold cross-validation, yielding several measurements listed in Section 2.7. The detailed evaluation results are shown in Table S3. For easy observations, the IFS curves were plotted with the number of features as the horizontal coordinate and the weighted F1 as the vertical coordinate, as shown in Figures 2–4.

**Figure 5:**
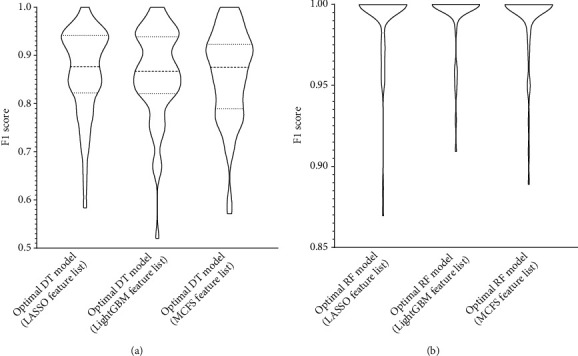
Violin plot showing the performance of the optimal models on sarcoma subtypes. (a) Performance of the optimal decision tree (DT) models. (b) Performance of the optimal random forest (RF) models.

For the feature subsets derived from the LASSO feature list, the performance of all models under these subsets is shown in Figure 2. RF can yield the highest weighted *F*1 of 0.987 when top 8850 methylation sites in the list were adopted. As for DT, its highest weighted *F*1 was 0.867 when top 7490 methylation sites were used. Accordingly, the top 8850 and 7490 methylation sites comprised the optimal features for RF and DT, respectively, based on which the optimal RF and DT models were constructed. Other overall measurements of these two models are listed in Table 1.  Figure 5 shows their detailed performance on 59 sarcoma subtypes. Clearly, the optimal RF model was much better than the optimal DT model.

The same arguments can be conducted for the LightGBM and MCFS feature lists. For the LightGBM feature list, the optimal RF model used the top 4520 methylation sites in the list and yielded the weighted *F*1 of 0.991 (Figure 3), whereas the optimal DT model yielded the weighted *F*1 of 0.868, which was obtained by using top 3900 methylation sites (Figure 3). Likewise, Table 1 lists other measurements of these two optimal models and Figure 5 shows their performance on all sarcoma subtypes. Also, the optimal RF model outperformed the optimal DT model. Finally, the optimal RF/DT model on the MCFS feature list generated the weighted *F*1 of 0.987/0.868 (Figure 4) when top 7020/5520 methylation sites were used. The performance of these two models is shown in Table 1 and Figure 5. Evidently, the optimal RF model also outperformed the optimal DT model.

### 3.3. Intersection of Essential Features Derived from Different Feature Lists

**Figure 6:**
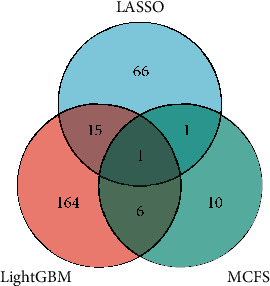
Venn diagram of three gene sets annotated by essential methylation sites extracted from three feature lists, which were generated by LASSO, LightGBM, and MCFS, respectively. The overlapping circles indicate genes that are identified by multiple feature ranking algorithms.

As the optimal RF model performs better than the optimal DT model for all feature lists, the optimal features for RF were selected as the optimal features on the corresponding feature list. However, there were too many optimal features, which was not easy to give further analyses. By checking the IFS results with RF on each feature list (Table S3), we can find that when top 110/30/310 features in the LASSO/LightGBM/MCFS feature list were used, the RF model can yield the weighted *F*1 of 0.971/0.940/0.978, which was only a little lower than that of the optimal RF model. The detailed performance of these models is listed in Table 1. Clearly, these models provided quite high performance. However, they adopted much less features than the optimal RF models, suggesting the extreme importance of these features. These methylation sites were annotated to genes, resulting in 83, 186, and 18 genes, respectively (Table S4), which constituted three gene sets. The intersection of these gene sets is shown in a Venn diagram (Figure 6). The detailed results of the intersection of the three sets are visible in Table S5. Some genes were included in multiple sets, which meant they were selected by multiple feature ranking algorithms. They may be highly relevant to the differentiation of different sarcoma subtypes. The specific analysis will be discussed in detail in subsequent sections.

### 3.4. Classification Rules

**Figure 7:**
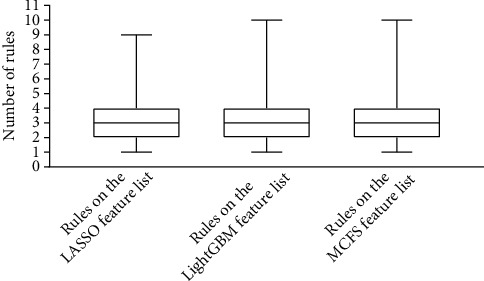
Boxplot showing the distribution of rules on sarcoma subtypes.

According to Figures 2–4, the performance of DT was evidently lower than that of RF on each feature list. However, DT has its own merit, which can make the classification procedures completely open. Such merit is helpful to understand its classification principle, providing more insights to figure out hidden information in the dataset. As mentioned above, the optimal DT models adopted top 7490 methylation sites in the LASSO list, top 3900 methylation sites in the LightGBM list, and top 5520 methylation sites in the MCFS list. With these features and all sarcoma samples, three big trees were obtained by DT. Accordingly, three sets of rules were summarized from these trees, which are provided in Table S6. 182, 190, and 198 rules, respectively, were included in three rule sets. In each rule set, all sarcoma subtypes were assigned some rules that could represent them. The rough distribution of rules in each set on 59 sarcoma subtypes is shown in Figure 7. It can be observed that most sarcoma subtypes were assigned 2-4 rules. Each rule indicated a special methylation pattern for its result (sarcoma subtype), which was a new way to investigate the essential differences between various sarcoma subtypes. Some of the important rules are discussed in detail in subsequent sections.

## 4. Discussion

The reliability of results obtained in this study was verified by existing studies. As some methylation sites have not been intensively studied, they may be new classification criteria and potential therapeutic targets for the corresponding sarcoma.

### 4.1. Analysis of the Decision Rules for Sarcoma Classification

Three rule sets were obtained by DT (Table S6). Here, we discussed some classification rules or criteria in different sets. We hypothesized that these methylation patterns, which are present as conditions in multiple rules, may be more important.

Based on the results, cg00982952 showed importance for Ewing (Ewing sarcoma) classification in all classifiers, and this methylation site could be annotated to the gene GLG1. Based on previous reports, GLG1 can be used as an auxiliary marker for the diagnosis of Ewing sarcoma by immunohistochemistry [44]. Although the detailed mechanism between GLG1 and Ewing sarcoma formation has not been clearly studied, the researchers found that GLG1 may be involved in the progression of multiple tumors by affecting the transport of key molecules involved in cell migration [45].

Some methylation sites play different roles in the decision rules of multiple sarcoma types. For example, our rules show that the AFF1 gene corresponding to the cg12109728 probe is hypermethylated in OS (HG)/high-grade conventional osteosarcoma but relatively hypomethylated in CHORD/chordoma. The transcript of AFF1 serves as a transcriptional regulator, and it can promote the expression of CD133, which is considered as a marker of normal or cancerous tissue [46]. At present, no studies have clearly pointed out the relationship between AFF1 methylation and OS (HG) or CHORD, but some studies have reported that the expression of CD133 in these two types of sarcomas changes specifically, which may serve as a potential therapeutic target [47]. High CD133 expression in CHORD may be related to its cancer stem-like cells and may enable CHORD to maintain self-renewal and resistance to chemotherapy [48].

The decision rules of different algorithms also have different criteria for some sarcoma types. For example, in the decision rules of alveolar rhabdomyosarcoma from the LASSO algorithm, we found that the FHL2 gene targeted by the cg02563156 probe requires hypermethylation. This finding has been indirectly confirmed by previous studies, that is, FHL2 is downregulated in rhabdomyosarcoma [49], which may be related to the hypermethylation of the FHL2 gene in this sarcoma type. In the MCFS algorithm, the classification is based on the methylation status of two sites, cg23157618 (ABCB9, hypomethylation) and cg23477348 (C21orf33, hypermethylation). By contrast, in the LightGBM algorithm, another gene, RNPEPL1 (cg16412000), is required to be hypomethylated, and the hypermethylation of this RNPEPL1 can be further used as a criterion for the decision rule of embryonal rhabdomyosarcoma. At present, the relationship among several methylation sites, including these genes and sarcoma, has not been studied, but these genes have been reported to be associated with sarcoma or other tumors [50–53]. We hypothesize that their methylation may serve as a basis for classification and provide reference for future sarcoma research and molecular pathology classification.

### 4.2. Analysis of the Predictive Features

Essential methylation sites extracted from three feature lists were mapped to genes, resulting in three gene sets. As shown in Figure 6, some genes occurred in multiple gene sets. These genes tended to be more important. We performed a preliminary analysis on these genes.

PRKAR1B is a key feature present in all three algorithms. Study has shown that circRNA circPRKAR1B promotes osteosarcoma progression, and it could be a potential therapeutic target [54]. At present, few studies have been conducted on the role of PRKAR1B gene in sarcoma, but it has been reported to play an important role in various tumors [55, 56]. This gene also encodes the regulatory subunit of gene PKA, and it is involved in the cAMP signaling pathway. In addition, PKA is involved in various sarcoma genesis and progression [57–59]. Therefore, the methylation of PRKAR1B gene may be very important for the identification or tumorigenesis of sarcoma, which deserve further investigation.

Methylation of INPP5A is a decisive feature in two algorithms, and our previous studies have shown the importance of gene methylation such as INPP5A for the diagnosis of multiple tumors [60]. Other studies have found that gene expression of INPP5A has an important role in sarcoma classification [61].

GLI3 is a transcription factor involved in the Hedgehog signaling pathway, and it plays an important role in development, immune system, and cancer [62]. Previous studies have found that GLI3 is highly expressed in embryonal rhabdomyosarcoma and some alveolar rhabdomyosarcoma, and it is associated with the prognosis of Ewing sarcoma [63, 64]. Therefore, expression changes caused by the aberrant methylation of GLI3 may serve as a basis for the classification of sarcomas.

In other previous studies, some genes (CCND1, CD109, NOS1, and ABLIM1) were found to be abnormally expressed in sarcomas, which can be used as prognostic markers or classification features for different sarcomas [65–69].

Some characteristically methylated genes are associated with bone lesions or osteocyte activity, but detailed investigation of their relationship with sarcoma is still lacking, for example, PHOSPHO1 and NFATC1. PHOSPHO1 is specifically expressed in bone lesions [70], and NFATC1 is associated with normal osteocyte function and osteosarcoma pathogenesis [71, 72]. We believe that further research of these genes may provide insights into sarcoma diagnosis and treatment.

## 5. Conclusions

Using a set of advanced machine learning methods, we designed a high-performance computational method to analyse sarcoma subtypes at the DNA methylation level. Through such method, genes highly associated with sarcoma subtypes, such as PRKAR1B, INPP5A, GLI3, and other genes, were obtained. The expression of these genes has been shown to be associated with sarcoma formation, demonstrating the robustness of our results. Furthermore, we combined IFS and two classification algorithms to build classification models with high performance. Three quantitative classification rule sets constructed by DT described the special patterns for different sarcoma subtypes. Our results provided scientific and theoretical guidance for clinical diagnosis and treatment of sarcoma.

## Figures and Tables

**Table 1:** Performance of random forest and decision tree under some feature subsets derived from three lists generated by three feature ranking algorithms.

Feature ranking algorithms	Classification algorithm	Number of features	Weighted *F*1	Macro *F*1	MCC	ACC
LASSO	RF	110	0.971	0.980	0.971	0.971
	RF	8850	0.987	0.990	0.987	0.987
	DT	7490	0.867	0.863	0.863	0.867
LightGBM	RF	30	0.940	0.948	0.939	0.940
	RF	4520	0.991	0.993	0.990	0.990
	DT	3900	0.868	0.861	0.864	0.868
MCFS	RF	310	0.978	0.982	0.978	0.978
	RF	7020	0.987	0.991	0.987	0.987
	DT	5520	0.868	0.860	0.863	0.866

## Data Availability

The original data used to support the findings of this study are available at Gene Expression Omnibus database (https://www.ncbi.nlm.nih.gov/geo/query/acc.cgi?acc=GSE140686).

## Supplementary Materials

Supplementary Materials Table S1: fifty-nine sarcoma subtypes and their sample sizes. Table S2: feature ranking results obtained using LASSO, LightGBM, and MCFS. Table S3: performance of IFS with different classification algorithms on three feature lists. Table S4: gene symbols obtained by annotating the most essential methylation sites derived from the LASSO, LightGBM, and MCFS feature lists. Table S5: intersection of three gene sets annotated by most essential methylation sites extracted from the LASSO, LightGBM, and MCFS feature lists. The genes that appear in the 3, 2, and 1 gene subsets are shown. Table S6: classification rules generated by decision tree using its optimal features on three feature lists.
