# Supplementary material for: Identification of Methylation Signatures and Rules for Sarcoma Subtypes by Machine Learning Methods
Source: Biomed Res Int. 2022 Dec 28;2022:5297235. doi: 10.1155/2022/5297235 (PMC9812612; doi:10.1155/2022/5297235)
Supplement: Supplementary Materials — Table S1: fifty-nine sarcoma subtypes and their sample sizes. Table S2: feature ranking results obtained using LASSO, LightGBM, and MCFS. Table S3: performance of IFS with different classification algorithms on three feature lists. Table S4: gene symbols obtained by annotating the most essential methylation sites derived from the LASSO, LightGBM, and MCFS feature lists. Table S5: intersection of three gene sets annotated by most essential methylation sites extracted from the LASSO, LightGBM, and MCFS feature lists. The genes that appear in the 3, 2, and 1 gene subsets are shown. Table S6: classification rules generated by decision tree using its optimal features on three feature lists. [file 5297235.f1.zip › Table S1 (1).pdf]

**Table S1:** Fifty-nine sarcoma subtypes and their sample sizes.
#

| Index | Class name (Abbreviation) | Sample size |
|---|---|---|
| 1 | angiomatoid fibrous histiocytoma (AFH) | 12 |
| 2 | atypical fibroxanthoma (AFX)/pleomorphic dermal sarcoma PDS | 15 |
| 3 | angioleiomyoma (ALMO)/myopericytoma (MPC) | 16 |
| 4 | angiosarcoma (AS) | 44 |
| 5 | alveolar soft part sarcoma (ASPS) | 27 |
| 6 | chondroblastoma (CB) | 10 |
| 7 | clear cell sarcoma of soft parts (CCS) | 12 |
| 8 | clear cell sarcoma of the kidney (CCSK) | 12 |
| 9 | chordoma (CHORD) | 74 |
| 10 | chondrosarcoma (CSA) | 69 |
| 11 | clear cell chondrosarcoma (CSA (CC)) | 8 |
| 12 | mesenchymal chondrosarcoma (CSA (MES)) | 11 |
| 13 | control (CTRL) | 10 |
| 14 | dermatofibrosarcoma protuberans (DFSP) | 39 |
| 15 | desmoplastic small round cell tumour (DSRCT) | 42 |
| 16 | desmoid-type fibromatosis (DTFM) | 13 |
| 17 | epithelioid haemangioendothelioma (EHE) | 10 |
| 18 | extraskeletal myxoid chondrosarcoma (EMCS) | 10 |
| 19 | epithelioid sarcoma (ES) | 32 |
| 20 | high-grade endometrial stromal sarcoma (ESS (HG)) | 11 |
| 21 | low-grade endometrial stromal sarcoma (ESS (LG)( | 18 |
| 22 | Ewing sarcoma (EWING) | 77 |
| 23 | fibrous dysplasia (FDY) | 14 |
| 24 | giant cell tumour of bone (GCTB) | 14 |
| 25 | gastrointestinal stromal tumour (GIST) | 52 |
| 26 | infantile fibrosarcoma (IFS) | 14 |
| 27 | inflammatory myofibroblastic tumour (IMT) | 13 |
| 28 | Kaposi sarcoma (KS) | 8 |
| 29 | Langerhans cell histiocytosis (LCH) | 12 |
| 30 | low-grade fibromyxoid sarcoma (LGFMS) | 8 |
| 31 | lipoma (LIPO) | 10 |
| 32 | leiomyoma (LMO) | 7 |
| 33 | leiomyosarcoma (LMS) | 69 |
| 34 | melanoma (MEL) | 12 |
| 35 | myxoid liposarcoma (MLS) | 38 |
| 36 | myositis ossificans (MO) | 8 |
| 37 | myositis proliferans (MP) | 8 |
| 38 | malignant peripheral nerve sheath tumour (MPNST) | 39 |
| 39 | malignant rhabdoid tumour (MRT) | 24 |

| | | |
|---|---|---|
| 40 | muscle tissue (MUS) | 8 |
| 41 | nodular fasciitis(NFA) | 10 |
| 42 | neurofibroma (NFB) | 9 |
| 43 | plexiform neurofibroma (NFB (PLEX)) | 7 |
| 44 | osteoblastoma (OB) | 8 |
| 45 | ossifying fibromyxoid tumour (OFMT) | 13 |
| 46 | high-grade conventional osteosarcoma (OS (HG)) | 86 |
| 47 | reactive tissue (REA) | 10 |
| 48 | rhabdomyosarcoma (RMS) | 10 |
| 49 | alveolar rhabdomyosarcoma (RMS (ALV)) | 67 |
| 50 | embryonal rhabdomyosarcoma (RMS (EMB)) | 49 |
| 51 | sarcoma (SARC) | 19 |
| 52 | small blue round cell tumour (SBRCT) | 23 |
| 53 | cutaneous squamous cell carcinoma (SCC (CUT)) | 10 |
| 54 | sclerosing epithelioid fibrosarcoma(SEF) | 10 |
| 55 | solitary fibrous tumour (SFT) | 31 |
| 56 | schwannoma (SWN) | 30 |
| 57 | synovial sarcoma (SYSA) | 59 |
| 58 | undifferentiated sarcoma (USARC) | 55 |
| 59 | well differentiated liposarcoma (WDLS)/dedifferentiated liposarcoma (DDLS) | 37 |

#
