# Supplementary material for: Identification of Methylation Signatures and Rules for Sarcoma Subtypes by Machine Learning Methods
Source: Biomed Res Int. 2022 Dec 28;2022:5297235. doi: 10.1155/2022/5297235 (PMC9812612; doi:10.1155/2022/5297235)
Supplement: Supplementary Materials — Table S1: fifty-nine sarcoma subtypes and their sample sizes. Table S2: feature ranking results obtained using LASSO, LightGBM, and MCFS. Table S3: performance of IFS with different classification algorithms on three feature lists. Table S4: gene symbols obtained by annotating the most essential methylation sites derived from the LASSO, LightGBM, and MCFS feature lists. Table S5: intersection of three gene sets annotated by most essential methylation sites extracted from the LASSO, LightGBM, and MCFS feature lists. The genes that appear in the 3, 2, and 1 gene subsets are shown. Table S6: classification rules generated by decision tree using its optimal features on three feature lists. [file 5297235.f1.zip › Table S2 (1).pdf]

Table S2: Feature ranking results obtained using LASSO, LightGBM, and MCFS.

Rank	LASSO feature list	LightGBM feature list	MCFS feature list
1	cg16452651	cg03359508	cg04703221
2	cg25339408	cg20560906	cg03438101
3	cg08325885	cg24613083	cg13453203
4	cg17056676	cg03988119	cg01013023
5	cg22799860	cg16071782	cg01727408
6	cg06509362	cg12895304	cg20415947
7	cg09604238	cg08304608	cg27065717
8	cg21726593	cg02619478	cg11854392
9	cg10457066	cg13810664	cg13734860
10	cg21307043	cg22706883	cg26152051
11	cg08573299	cg17886413	cg01084215
12	cg01356872	cg02315513	cg23902076
13	cg25373624	cg03639152	cg14839134
14	cg10370025	cg05555455	cg03223959
15	cg20065217	cg02202664	cg10131972
16	cg19287349	cg25461827	cg02650017
17	cg27336068	cg06827256	cg06814256
18	cg27403628	cg06950392	cg23075506
19	cg26607933	cg08913523	cg26929355
20	cg15532942	cg22161706	cg17758899
21	cg02734419	cg11843516	cg23311929
22	cg02471028	cg01863398	cg05951828
23	cg06950392	cg00765705	cg13473184
24	cg06323896	cg07333510	cg04815973
25	cg20943461	cg04007841	cg24202221
26	cg10960375	cg17171539	cg16461139
27	cg15346286	cg02628360	cg23519637
28	cg08240917	cg09925747	cg02816525
29	cg03614132	cg26543333	cg27495643
30	cg22731190	cg25834632	cg04867652
31	cg24073994	cg01664382	cg11285912
32	cg16269776	cg09123625	cg16232530
33	cg00042325	cg26018322	cg05376738
34	cg27143326	cg15639842	cg07324116
35	cg19849641	cg27010076	cg23723410
36	cg12266049	cg12744447	cg15577180
37	cg07727134	cg06659338	cg24283049
38	cg20218614	cg03971344	cg27301230
39	cg25134701	cg22663660	cg00651497
40	cg07066794	cg17428748	cg23986590
41	cg10546888	cg06967316	cg08132573
42	cg03840920	cg15317049	cg17367884
43	cg16023107	cg25453957	cg02665399
44	cg00706536	cg22204453	cg04658707
45	cg25245161	cg21163714	cg02061967
46	cg25556464	cg04708036	cg13808936
47	cg05760053	cg17938607	cg20592766
48	cg17497965	cg24852135	cg08209934
49	cg15553612	cg00001687	cg15230883
50	cg18273464	cg02829456	cg11311843

51	cg11088489	cg17469039	cg19857461
52	cg13734106	cg14410516	cg23539753
53	cg18847089	cg23719367	cg24499677
54	cg10532807	cg04774476	cg10722267
55	cg06584121	cg22977745	cg05813328
56	cg24926121	cg15540341	cg20690695
57	cg06562964	cg01439112	cg00657810
58	cg10327980	cg00885461	cg16073408
59	cg11143193	cg09786420	cg20899781
60	cg22958118	cg07891483	cg01323212
61	cg08343075	cg02148034	cg15342087
62	cg19313353	cg09494609	cg24024036
63	cg13922681	cg01818102	cg08061755
64	cg01837846	cg16700163	cg19259111
65	cg19745930	cg06752365	cg14505694
66	cg05364072	cg00783706	cg07946977
67	cg09982918	cg18556792	cg16700163
68	cg04665930	cg08097657	cg01007781
69	cg11748187	cg03234557	cg03077671
70	cg12865675	cg14005246	cg00133595
71	cg25815219	cg23237976	cg19262818
72	cg05960932	cg23208152	cg20669366
73	cg04254540	cg09558069	cg04147497
74	cg06098693	cg09326362	cg08779777
75	cg10457079	cg02353448	cg21386766
76	cg27067922	cg16602460	cg18749563
77	cg09425164	cg17459290	cg01262865
78	cg03017946	cg07266910	cg02753511
79	cg04457069	cg11412527	cg07197326
80	cg07824182	cg04140971	cg24859433
81	cg05116002	cg26847438	cg12690127
82	cg06340118	cg19138499	cg15028507
83	cg04440361	cg16819272	cg21757281
84	cg05205532	cg16770054	cg15161050
85	cg00610577	cg26321613	cg18055067
86	cg02528008	cg18852515	cg15262242
87	cg05139187	cg24358599	cg05482864
88	cg06772578	cg25403283	cg24987440
89	cg15959921	cg19536781	cg24386135
90	cg23171099	cg13358061	cg12308279
91	cg01723892	cg26880445	cg27226424
92	cg21171299	cg06193668	cg09335712
93	cg00187129	cg01968002	cg05229802
94	cg12178980	cg00785482	cg27161027
95	cg20285745	cg00436722	cg17508905
96	cg09861034	cg02793948	cg16185457
97	cg20528338	cg00216961	cg27449255
98	cg09141835	cg22575379	cg00158530
99	cg14129169	cg08825895	cg17193551
100	cg08390994	cg15715853	cg17736336
101	cg12700788	cg20592766	cg18503234
102	cg20256649	cg02926266	cg14789529
103	cg13624631	cg04963697	cg00900735

104	cg24866203	cg14024328	cg07294624
105	cg13148921	cg00259518	cg00070460
106	cg16535788	cg26720682	cg19849478
107	cg14467840	cg02306481	cg20664445
108	cg05976516	cg06943385	cg13957126
109	cg17860133	cg18752987	cg24002003
110	cg12210305	cg12121782	cg26011438
111	cg06884199	cg01683570	cg00586700
112	cg00341297	cg21850879	cg15679098
113	cg17297775	cg19779886	cg17610889
114	cg16723180	cg15899297	cg10142436
115	cg08897688	cg06263395	cg26064794
116	cg04141218	cg03148858	cg03549146
117	cg05229802	cg01260146	cg11444072
118	cg03212620	cg20329085	cg24186506
119	cg25028855	cg07571745	cg24824472
120	cg13745832	cg01338955	cg18231184
121	cg17329004	cg23690444	cg05824174
122	cg13414654	cg13298538	cg19508622
123	cg07012739	cg04716580	cg10012530
124	cg14462432	cg02983650	cg05109049
125	cg12939390	cg02660541	cg07802362
126	cg13215078	cg14081251	cg24866203
127	cg09932436	cg00982952	cg05650238
128	cg27395391	cg23971517	cg16402415
129	cg13461509	cg09685505	cg26757820
130	cg00299943	cg27505472	cg26566236
131	cg13071729	cg22990726	cg19536781
132	cg03131767	cg16217297	cg25138553
133	cg08806632	cg08331427	cg15934607
134	cg05181397	cg02462416	cg06562888
135	cg01448551	cg24420164	cg08002981
136	cg08894066	cg18461635	cg02108015
137	cg24747239	cg17036007	cg25431366
138	cg13309415	cg11935248	cg09897737
139	cg03498175	cg11762760	cg17796043
140	cg04629141	cg09318508	cg07363131
141	cg13470673	cg04311473	cg04195226
142	cg04505897	cg23393892	cg18758976
143	cg04907595	cg19057779	cg14189391
144	cg20019410	cg12939390	cg06323896
145	cg22957135	cg00131423	cg09141835
146	cg15174926	cg24314474	cg13598881
147	cg19354017	cg19857633	cg19244380
148	cg24386905	cg13808936	cg25947619
149	cg09327610	cg11080651	cg13753526
150	cg07907506	cg03006477	cg25510609
151	cg25154482	cg00757391	cg21648069
152	cg10000148	cg23357150	cg19698242
153	cg19244380	cg04353106	cg21557473
154	cg07187503	cg02034328	cg03545972
155	cg17477806	cg00626390	cg15881332
156	cg14584535	cg26954228	cg15843262

157	cg00680277	cg19239692	cg12017558
158	cg07329131	cg16996266	cg00569091
159	cg14065153	cg20415947	cg26034341
160	cg10841956	cg15020964	cg08240917
161	cg10518527	cg00810292	cg05664352
162	cg00887848	cg18090631	cg15715853
163	cg07598035	cg08092318	cg05341539
164	cg26156321	cg04438661	cg09230938
165	cg24059075	cg24259291	cg25590335
166	cg25236277	cg00607989	cg09186408
167	cg17437086	cg25189808	cg01349691
168	cg19101998	cg23172911	cg16306870
169	cg10107466	cg15720017	cg13721644
170	cg12734386	cg14420645	cg06186808
171	cg16889721	cg08750440	cg13800349
172	cg05339403	cg03538565	cg22731637
173	cg19109601	cg13708908	cg06174078
174	cg02817925	cg11444072	cg06611426
175	cg06783380	cg11234767	cg22451412
176	cg04724275	cg26963844	cg17434577
177	cg07607355	cg06143290	cg01044293
178	cg18382744	cg01661567	cg12832726
179	cg23413464	cg21698310	cg09701233
180	cg15464763	cg20285745	cg13366501
181	cg23181573	cg15545942	cg04963697
182	cg11468635	cg09623085	cg01400750
183	cg26105283	cg06937357	cg07536018
184	cg03811905	cg26891661	cg04884579
185	cg02827340	cg24982682	cg00820405
186	cg08364860	cg23222247	cg16572224
187	cg24404329	cg15929241	cg14565439
188	cg05661968	cg11538848	cg19130973
189	cg16617774	cg01937669	cg12681370
190	cg03515290	cg21189849	cg16752592
191	cg12113251	cg01012242	cg27306787
192	cg03888083	cg27302539	cg13681684
193	cg21658153	cg12113251	cg20293725
194	cg10626305	cg08572315	cg04956511
195	cg04015687	cg00051546	cg06989253
196	cg24706505	cg17266282	cg03538565
197	cg25573640	cg16489468	cg19374752
198	cg00559439	cg14104252	cg04213390
199	cg03446062	cg12762413	cg14286546
200	cg07131274	cg16721977	cg00353407
201	cg14698794	cg16102706	cg06368978
202	cg11068930	cg15247169	cg09825309
203	cg09123625	cg14514032	cg15702277
204	cg24613083	cg13663738	cg06547766
205	cg12741436	cg04972957	cg08447373
206	cg21863721	cg22202031	cg10017293
207	cg11270070	cg20195046	cg15686782
208	cg04612073	cg08033285	cg09006420
209	cg22805632	cg07927953	cg10408178

210	cg22584802	cg07377299	cg03013917
211	cg04883903	cg05482973	cg03431524
212	cg03779973	cg03779937	cg03376089
213	cg09164574	cg03139388	cg07021447
214	cg16067210	cg21979773	cg11041314
215	cg03131732	cg12944530	cg01606770
216	cg09537038	cg05076082	cg26184501
217	cg06126019	cg26124719	cg02998240
218	cg23477348	cg13470673	cg07507493
219	cg04254198	cg11994639	cg06795827
220	cg07512971	cg04473209	cg25730564
221	cg08779982	cg02197542	cg20076442
222	cg10062065	cg25520910	cg23485307
223	cg01678309	cg25310555	cg00967711
224	cg01828733	cg25013838	cg25281849
225	cg02633600	cg22809726	cg25556464
226	cg06204229	cg17384769	cg06880930
227	cg13846358	cg04095995	cg16081096
228	cg17196713	cg27437304	cg14482093
229	cg12595697	cg27351813	cg27025137
230	cg24015175	cg25364972	cg18878210
231	cg06765552	cg19772651	cg19776793
232	cg19137748	cg17367836	cg15487498
233	cg14081465	cg11977716	cg13948857
234	cg21450654	cg10976861	cg02968890
235	cg03417340	cg05650238	cg22459052
236	cg10071852	cg03017946	cg02339793
237	cg24976262	cg00567872	cg21546522
238	cg01677310	cg22803510	cg12727940
239	cg00927364	cg19051802	cg16205958
240	cg26638069	cg18854004	cg09304617
241	cg15226226	cg16185457	cg15089181
242	cg06460400	cg13488220	cg04232282
243	cg24426483	cg10886334	cg19856705
244	cg11432630	cg08091050	cg00944304
245	cg05800368	cg07529658	cg13447933
246	cg23750338	cg06748078	cg15582707
247	cg07349899	cg26180843	cg23058246
248	cg14843670	cg24073994	cg00673191
249	cg08007899	cg23687021	cg27390206
250	cg00841141	cg27226424	cg07281938
251	cg08613144	cg26882115	cg08726900
252	cg06436667	cg20934096	cg25287071
253	cg04771901	cg15063366	cg07793033
254	cg26267483	cg09421126	cg23910341
255	cg01260603	cg07818422	cg24750887
256	cg23929809	cg04555379	cg21870038
257	cg00051546	cg03840920	cg27092594
258	cg01483824	cg25430713	cg19784198
259	cg10667102	cg22644984	cg21351483
260	cg19643097	cg10713589	cg07921503
261	cg01696984	cg03444965	cg11960033
262	cg03640756	cg03216506	cg14655905

263	cg06344576	cg00058291	cg18645642
264	cg09796786	cg24787929	cg25846061
265	cg16056849	cg20139145	cg20027784
266	cg24773418	cg08775793	cg14571537
267	cg04774930	cg00542261	cg14628049
268	cg03812676	cg23920016	cg03400491
269	cg13093023	cg23664774	cg01103253
270	cg26927190	cg23300494	cg27331828
271	cg07433453	cg18765405	cg04601090
272	cg01613294	cg11553311	cg25061701
273	cg03659476	cg03938353	cg10227280
274	cg03077533	cg03861097	cg13838713
275	cg11450827	cg25692669	cg03614132
276	cg09185884	cg20122645	cg24635581
277	cg15712057	cg19220272	cg00994389
278	cg08749443	cg08377924	cg04403415
279	cg22605643	cg06436663	cg26215113
280	cg04196068	cg02124957	cg15862380
281	cg13637654	cg27489873	cg05552543
282	cg03953506	cg24408316	cg09421126
283	cg04716580	cg22286382	cg10037894
284	cg11135937	cg17778120	cg08577953
285	cg00949635	cg10907727	cg07275305
286	cg05547140	cg09829263	cg03606646
287	cg02622601	cg05982473	cg11320499
288	cg05305993	cg04804648	cg17578309
289	cg10091866	cg01303480	cg06200670
290	cg16459391	cg25270886	cg10899681
291	cg12843033	cg24647108	cg18369990
292	cg24319076	cg22593342	cg12302797
293	cg16812352	cg13496979	cg26572651
294	cg22152438	cg09665571	cg06746318
295	cg18766210	cg03999452	cg05116002
296	cg08257212	cg01753263	cg18766210
297	cg01697623	cg01013023	cg22161706
298	cg15363973	cg15553612	cg16366639
299	cg07917127	cg13734860	cg17152266
300	cg07099056	cg06400255	cg26726230
301	cg24970620	cg01824933	cg23973885
302	cg23237976	cg25900150	cg07044414
303	cg01385157	cg24870688	cg23207527
304	cg15127702	cg20700977	cg18244708
305	cg01546047	cg18375421	cg11869499
306	cg04188241	cg10409799	cg15451258
307	cg10439859	cg04009429	cg11685391
308	cg16516980	cg03285823	cg11051022
309	cg22118369	cg00732878	cg00512872
310	cg23301539	cg23058673	cg10607603
311	cg01462184	cg14419424	cg04110886
312	cg18965086	cg12948543	cg06579465
313	cg00478851	cg11958644	cg18207099
314	cg03291336	cg05468559	cg16026813
315	cg13070215	cg02926747	cg25102842

316	cg14003974	cg01345395	cg12312338
317	cg01640684	cg17693222	cg01357897
318	cg13439189	cg14935206	cg17130544
319	cg03401464	cg05196820	cg26169991
320	cg04867652	cg03393445	cg03023681
321	cg22370005	cg00412775	cg15299233
322	cg21613549	cg22871566	cg25723217
323	cg23024810	cg00290626	cg25941751
324	cg05931119	cg21035368	cg00903438
325	cg19706515	cg18238763	cg16625770
326	cg15081886	cg12389888	cg03161498
327	cg19767562	cg11391732	cg00223767
328	cg17539235	cg10202788	cg13916835
329	cg27540799	cg09322899	cg06752482
330	cg02047646	cg06618764	cg08527124
331	cg19702383	cg06084403	cg02137970
332	cg06891043	cg05547140	cg11637968
333	cg06591973	cg04914198	cg22854448
334	cg07295362	cg04819655	cg05403316
335	cg06297857	cg00651497	cg19308185
336	cg04324917	cg22376204	cg21756765
337	cg04398282	cg15225991	cg07720334
338	cg00358023	cg14890730	cg04425005
339	cg25940447	cg10979364	cg15250073
340	cg01602730	cg06675951	cg14558880
341	cg17518949	cg00806009	cg20354777
342	cg22173510	cg19313353	cg10512089
343	cg19382808	cg19047670	cg24706505
344	cg22472304	cg15506281	cg12847536
345	cg21887193	cg14742341	cg15815827
346	cg07811002	cg14101193	cg21118367
347	cg05445244	cg13397568	cg13352836
348	cg05755441	cg13374528	cg24982682
349	cg18203044	cg09425164	cg11650648
350	cg14231959	cg06819431	cg07155331
351	cg09646934	cg05951828	cg06407371
352	cg07281938	cg04729730	cg20540235
353	cg08476843	cg01306824	cg25005357
354	cg21569714	cg01170069	cg00904578
355	cg15460348	cg00549561	cg01589353
356	cg17046776	cg00095326	cg06385449
357	cg07010552	cg00095259	cg07385220
358	cg04568492	cg27007717	cg07630327
359	cg24697460	cg23873200	cg02767093
360	cg14207326	cg22451265	cg08847512
361	cg04060308	cg18965086	cg02817932
362	cg14468090	cg16167741	cg01166827
363	cg15941159	cg13849495	cg17939585
364	cg27643147	cg12413156	cg09548893
365	cg14375387	cg10059410	cg15646741
366	cg14507658	cg09777637	cg25310555
367	cg00994984	cg08841613	cg23779478
368	cg02068351	cg03315432	cg27381488

369	cg17654747	cg26784106	cg06225767
370	cg08727428	cg19827875	cg16204524
371	cg12245876	cg09534872	cg04604708
372	cg08002981	cg07041720	cg03452625
373	cg11360860	cg05386977	cg02013841
374	cg22987116	cg03482221	cg26552621
375	cg13411507	cg01487195	cg14213430
376	cg10997906	cg27095984	cg14195992
377	cg13170807	cg23533073	cg00876376
378	cg25364972	cg23493018	cg12416830
379	cg18766468	cg21658153	cg13611006
380	cg03743074	cg19770671	cg25312122
381	cg05201312	cg19340420	cg21642054
382	cg13035910	cg18560638	cg08571020
383	cg19702397	cg17024719	cg14599015
384	cg08273640	cg15592324	cg22666103
385	cg13367537	cg12700788	cg16996242
386	cg23539494	cg11960033	cg14753356
387	cg11319760	cg10664272	cg14583973
388	cg08843756	cg08619651	cg04381888
389	cg24407065	cg08248600	cg02937548
390	cg25650076	cg04727924	cg09892984
391	cg05797224	cg03779973	cg06943385
392	cg23486701	cg03667405	cg01456030
393	cg21616243	cg02003272	cg05201300
394	cg19418951	cg00860425	cg25084760
395	cg00767010	cg00001809	cg07552087
396	cg16976520	cg26566236	cg01749725
397	cg00360324	cg24590353	cg04967410
398	cg26888672	cg18120259	cg05798126
399	cg12669088	cg13808641	cg17174566
400	cg18617669	cg12688942	cg20338754
401	cg01780685	cg09331409	cg24212268
402	cg01983725	cg07324116	cg22016731
403	cg03439687	cg05832823	cg00711291
404	cg03163819	cg00248856	cg15899800
405	cg21650861	cg27073311	cg14811011
406	cg04784315	cg25471740	cg12422551
407	cg27076552	cg25287071	cg21235119
408	cg16936094	cg24993140	cg17036007
409	cg05575733	cg12494355	cg01581360
410	cg09725874	cg12210305	cg25249728
411	cg15411034	cg11631173	cg12657297
412	cg27278001	cg07344990	cg10237442
413	cg07654843	cg04944177	cg04707715
414	cg19714865	cg01437411	cg27202913
415	cg05139219	cg00967711	cg26362368
416	cg12293340	cg23005227	cg16523645
417	cg19784013	cg16597437	cg27498434
418	cg00089486	cg15416179	cg08824291
419	cg16158296	cg14870461	cg01824933
420	cg13299824	cg11231069	cg23939642
421	cg05488981	cg06927900	cg20252837

422	cg20387087	cg00277804	cg06212582
423	cg10244706	cg00223767	cg09510085
424	cg06024540	cg26705574	cg15543534
425	cg25019378	cg22964758	cg23887149
426	cg00232535	cg21163717	cg01653532
427	cg07818422	cg20197694	cg18854872
428	cg05274056	cg17903316	cg17865371
429	cg20154222	cg13863769	cg00810292
430	cg20383521	cg13798270	cg17781669
431	cg09335911	cg12186219	cg23835923
432	cg16625770	cg11248182	cg11772801
433	cg15991104	cg10149870	cg17185710
434	cg14425914	cg08345465	cg15862394
435	cg08092318	cg07630327	cg27549186
436	cg00688539	cg03380645	cg08825895
437	cg01077618	cg02827132	cg23339720
438	cg02452418	cg26995244	cg01871631
439	cg15963326	cg21526773	cg23313005
440	cg24870688	cg21450547	cg27200466
441	cg15326069	cg21292981	cg05041265
442	cg25568066	cg16564132	cg26697605
443	cg21475076	cg14602839	cg09015905
444	cg22256482	cg13297120	cg09912552
445	cg18819574	cg12669088	cg08777316
446	cg08863422	cg11405458	cg25984524
447	cg26754071	cg09142595	cg05825120
448	cg23748751	cg08469834	cg14879279
449	cg15082028	cg05185926	cg18710162
450	cg03030665	cg05088605	cg07412315
451	cg22422264	cg02878891	cg08566548
452	cg09746044	cg02157052	cg04312967
453	cg04260307	cg27177983	cg02372856
454	cg04195543	cg26529094	cg10174683
455	cg10458392	cg24783499	cg07888884
456	cg24960799	cg24747239	cg10963218
457	cg08877591	cg24215159	cg19745930
458	cg09603024	cg19573208	cg04911180
459	cg25684961	cg16161135	cg17721879
460	cg24899558	cg13366501	cg11046030
461	cg20494661	cg12832726	cg22021794
462	cg22463097	cg12072024	cg07038689
463	cg09278623	cg10546888	cg14412134
464	cg08642081	cg10239163	cg16102778
465	cg19549465	cg10129154	cg19988492
466	cg14553765	cg09861034	cg14919455
467	cg07622079	cg09009788	cg23100428
468	cg11390504	cg08094614	cg20861822
469	cg20924972	cg06900571	cg06849167
470	cg14320530	cg04911680	cg17787876
471	cg18583276	cg03038245	cg00863893
472	cg14431528	cg02607972	cg03999452
473	cg09279803	cg00916659	cg08258867
474	cg05226061	cg27110374	cg12167489

475	cg19621160	cg26932693	cg08141395
476	cg06943845	cg26929355	cg02806704
477	cg11016151	cg22528270	cg06389574
478	cg24990238	cg21393818	cg19783160
479	cg14935206	cg21105227	cg27565803
480	cg00748072	cg16570314	cg07094487
481	cg24939470	cg11661534	cg19736117
482	cg00259518	cg07283582	cg06098693
483	cg24018760	cg07021447	cg03036214
484	cg14095283	cg05477920	cg20449048
485	cg15351186	cg05364072	cg13782615
486	cg21698310	cg03512577	cg21252105
487	cg02658272	cg01349691	cg25881985
488	cg06927900	cg23525541	cg06344576
489	cg19432283	cg21735516	cg20792436
490	cg08386696	cg19747992	cg02835462
491	cg03044471	cg18527971	cg22731190
492	cg27302539	cg17901582	cg24233679
493	cg06853455	cg17853850	cg21670987
494	cg06390613	cg16820411	cg00581541
495	cg06037365	cg13165992	cg06853455
496	cg10225755	cg13115424	cg08390994
497	cg23953820	cg12245876	cg24175188
498	cg26397391	cg10676125	cg03991106
499	cg21105227	cg10611016	cg23854567
500	cg05966641	cg04386759	cg12076543
501	cg26308909	cg04234758	cg03621841
502	cg01716084	cg03604011	cg16636756
503	cg20341998	cg01467882	cg16812352
504	cg25921543	cg24738036	cg27566403
505	cg18761976	cg24002003	cg15033511
506	cg06547766	cg21648069	cg03872934
507	cg01872216	cg20951650	cg03818193
508	cg13216372	cg20138711	cg18327695
509	cg09028132	cg15959893	cg23925540
510	cg15353597	cg14978242	cg22700848
511	cg03495660	cg09957712	cg22786667
512	cg06443231	cg09631175	cg02644867
513	cg01675694	cg09604238	cg15188939
514	cg18888464	cg05999324	cg08304608
515	cg21546226	cg01501896	cg14350114
516	cg07990390	cg01380319	cg05251593
517	cg16349616	cg00610205	cg20316440
518	cg25013838	cg00083399	cg10820904
519	cg16205958	cg25888386	cg18061711
520	cg07050626	cg25355635	cg19621160
521	cg06186808	cg22798362	cg00937742
522	cg14360029	cg20010135	cg01442620
523	cg13951223	cg19063061	cg01818539
524	cg02935097	cg18763720	cg12233100
525	cg19016264	cg18465694	cg10149889
526	cg02882004	cg18397308	cg18120259
527	cg14628049	cg14523734	cg13488013

528	cg13681684	cg14330078	cg18233538
529	cg06887283	cg14003974	cg24365837
530	cg24335984	cg08966413	cg08913523
531	cg08412188	cg05798126	cg25596405
532	cg22451412	cg00421693	cg15259233
533	cg04838832	cg25227364	cg21268578
534	cg05269678	cg22365446	cg06660116
535	cg19182035	cg21377260	cg08113187
536	cg14569423	cg21246624	cg14521421
537	cg07927953	cg19921577	cg11546709
538	cg10152624	cg19382808	cg06422467
539	cg24630426	cg16700555	cg26927190
540	cg14565781	cg15451258	cg16936094
541	cg23732483	cg08897688	cg18011099
542	cg07104557	cg08866865	cg13223682
543	cg20323175	cg00631702	cg05705335
544	cg04070601	cg26638069	cg26326208
545	cg03654273	cg26315221	cg12762413
546	cg26171489	cg24987440	cg09950920
547	cg05986052	cg24175188	cg04143348
548	cg24631518	cg20418529	cg11143193
549	cg19921577	cg19450111	cg07716847
550	cg07027613	cg18474180	cg06618764
551	cg13343549	cg13556624	cg23468002
552	cg15889057	cg13070215	cg09029193
553	cg11253592	cg11700356	cg08934799
554	cg20591120	cg10884953	cg22255690
555	cg10472711	cg09725874	cg08110337
556	cg09597070	cg08236537	cg25841625
557	cg08695912	cg07917127	cg27571329
558	cg20609679	cg07706695	cg04993279
559	cg06925389	cg05977276	cg15121417
560	cg24164238	cg04926380	cg05288253
561	cg21628927	cg23777946	cg21048763
562	cg07925587	cg22397446	cg13694343
563	cg23415916	cg21818807	cg06817454
564	cg26745294	cg20256649	cg06833798
565	cg13582959	cg16794682	cg22700246
566	cg08205361	cg15063355	cg10461264
567	cg12846837	cg14971998	cg13721589
568	cg13620881	cg12595461	cg14884828
569	cg06116260	cg10642094	cg04273604
570	cg03798162	cg10052840	cg18280717
571	cg27483571	cg08559648	cg06267617
572	cg07038689	cg08489478	cg08774694
573	cg02102684	cg07749442	cg09901529
574	cg05399244	cg07309864	cg23974819
575	cg17696194	cg05204104	cg10859133
576	cg11902934	cg03040489	cg11786870
577	cg17221945	cg27067922	cg12459932
578	cg22920258	cg26379230	cg26810214
579	cg11861709	cg24059075	cg26884345
580	cg09036558	cg23682934	cg14330078

581	cg05330056	cg20686479	cg05977523
582	cg12612065	cg20227592	cg22330533
583	cg01710084	cg19702397	cg00901982
584	cg16965936	cg18601229	cg17250537
585	cg06259664	cg14671764	cg04074404
586	cg01185921	cg13915179	cg27295434
587	cg17307791	cg12448664	cg17755483
588	cg18645642	cg10897631	cg24298539
589	cg00035074	cg10091866	cg23545258
590	cg01788682	cg10089081	cg02838178
591	cg01012242	cg09669930	cg12106976
592	cg19756253	cg07900344	cg20090290
593	cg12503110	cg06255609	cg03318904
594	cg00106093	cg05705335	cg24065957
595	cg19295314	cg04260307	cg18938534
596	cg06890747	cg03589898	cg21936959
597	cg06363129	cg02595280	cg10109635
598	cg00216061	cg02579022	cg23881119
599	cg05750926	cg01691522	cg11307417
600	cg02961196	cg27044641	cg01791238
601	cg04624413	cg25138553	cg07265549
602	cg04685302	cg22700848	cg04248332
603	cg01835489	cg20586531	cg25634000
604	cg12878382	cg18253910	cg00444740
605	cg20137237	cg17825194	cg20418529
606	cg01239666	cg17638841	cg15275017
607	cg09646558	cg17475200	cg05202347
608	cg11367404	cg16863382	cg21442998
609	cg14437446	cg15016233	cg02697979
610	cg20461270	cg12183501	cg01154505
611	cg15161854	cg11215918	cg21546226
612	cg08124923	cg10921702	cg00548009
613	cg15410236	cg10472711	cg07483007
614	cg10860364	cg10299941	cg23049226
615	cg00372132	cg07379550	cg08476843
616	cg02346135	cg06856329	cg07938459
617	cg18905668	cg06257110	cg08687995
618	cg05664352	cg05623411	cg12143499
619	cg22663660	cg04025970	cg17415355
620	cg09900084	cg03962809	cg18109289
621	cg00656603	cg03800922	cg26835798
622	cg25883020	cg03421440	cg19553402
623	cg13517605	cg03031526	cg17884766
624	cg08625996	cg02829279	cg19802929
625	cg21925748	cg27495643	cg11728201
626	cg24365837	cg25463863	cg17444479
627	cg02725453	cg24158160	cg15226226
628	cg06815308	cg19956769	cg13332142
629	cg19240021	cg19444998	cg19133023
630	cg06825317	cg18435870	cg09534872
631	cg25521853	cg16205958	cg14313184
632	cg06513888	cg15988734	cg11025609
633	cg13287523	cg15074047	cg16060382

634	cg26929355	cg14789529	cg04091816
635	cg05376267	cg14202146	cg05032848
636	cg16074271	cg14190344	cg21156057
637	cg04213390	cg13348246	cg09631059
638	cg08110337	cg12642717	cg20608966
639	cg23052758	cg10508317	cg27627006
640	cg22866068	cg09279803	cg03006477
641	cg09450022	cg08364860	cg23748172
642	cg04091816	cg06989443	cg10493436
643	cg23235334	cg06012410	cg03218909
644	cg06483517	cg05017639	cg25658272
645	cg19525780	cg04158792	cg00937817
646	cg07124859	cg03571927	cg07844828
647	cg19081470	cg00039385	cg16712866
648	cg13274107	cg24706505	cg24941195
649	cg18532070	cg21201494	cg08806632
650	cg09666417	cg20757748	cg03604011
651	cg01441113	cg20133730	cg14747072
652	cg17854510	cg18957751	cg12895304
653	cg01399475	cg18533833	cg07749442
654	cg14651518	cg17920820	cg05139187
655	cg07438999	cg16681083	cg02066441
656	cg03892631	cg15547344	cg01411786
657	cg10654373	cg14865516	cg00123090
658	cg22846966	cg13680246	cg03417340
659	cg02829279	cg13655570	cg21377260
660	cg25445431	cg12395479	cg09837656
661	cg07541559	cg10865444	cg23378722
662	cg20999795	cg10802680	cg26689934
663	cg11423998	cg10380108	cg18801945
664	cg08661751	cg09165170	cg16602460
665	cg09165170	cg07733851	cg13618880
666	cg09745791	cg07555102	cg11490961
667	cg17375396	cg04919234	cg09537038
668	cg04149585	cg02418175	cg19683675
669	cg03101492	cg02247300	cg04771901
670	cg23304923	cg00948274	cg04607323
671	cg19216851	cg00257455	cg06827256
672	cg13413247	cg00118783	cg00449021
673	cg08910524	cg27506609	cg04397652
674	cg16060382	cg26092233	cg14820213
675	cg10990188	cg24424381	cg09665571
676	cg22822048	cg23818351	cg15536947
677	cg07520608	cg20435464	cg11818853
678	cg07398661	cg20005923	cg18652285
679	cg23348723	cg19849641	cg23663547
680	cg09307742	cg18280717	cg04972957
681	cg14839134	cg17152266	cg08115732
682	cg25498969	cg16996242	cg22780675
683	cg10925829	cg16488544	cg24158160
684	cg09526469	cg15613048	cg23936766
685	cg08836729	cg15127656	cg16411103
686	cg21450652	cg13425135	cg04140971

687	cg27222884	cg12614090	cg26313421
688	cg26124719	cg12552626	cg05242371
689	cg13971603	cg11030811	cg16766632
690	cg09520904	cg11010976	cg07236781
691	cg13877631	cg10977734	cg16213787
692	cg04508114	cg10803714	cg23172911
693	cg23244877	cg10634182	cg01399475
694	cg27051683	cg10109635	cg02138124
695	cg06292709	cg08115297	cg21497060
696	cg10809958	cg07346310	cg25532627
697	cg23632333	cg07197326	cg22918700
698	cg22257056	cg07044414	cg00699693
699	cg07915976	cg05041265	cg20386404
700	cg16649474	cg04661959	cg20230305
701	cg07156006	cg03495832	cg12710648
702	cg01993468	cg02867857	cg07577837
703	cg11722816	cg01448551	cg23019935
704	cg13385220	cg00968638	cg07713495
705	cg01783960	cg00904578	cg24993140
706	cg11497526	cg27641240	cg25552843
707	cg03379631	cg24845165	cg07088950
708	cg09276451	cg24594604	cg17327331
709	cg04819655	cg24024036	cg16078863
710	cg24541176	cg23344121	cg05279866
711	cg16519668	cg22099441	cg20679271
712	cg00713247	cg17945541	cg16829042
713	cg25028527	cg16759204	cg21196487
714	cg11490944	cg15684702	cg16277214
715	cg06792186	cg13251842	cg14606082
716	cg23019935	cg12866104	cg24148817
717	cg24034465	cg12093620	cg12728588
718	cg05718035	cg09330834	cg12204166
719	cg02522196	cg08688659	cg05204104
720	cg00158770	cg08605965	cg08850243
721	cg18539086	cg07795346	cg03573679
722	cg03784886	cg07079828	cg04058399
723	cg21945949	cg06755413	cg00559439
724	cg01414464	cg06377278	cg03131767
725	cg01380319	cg06142351	cg04157865
726	cg12053105	cg05595469	cg06070445
727	cg06813419	cg05109049	cg11815072
728	cg19513582	cg02862467	cg26899399
729	cg27117828	cg00322201	cg09036558
730	cg24462247	cg26960083	cg13068653
731	cg01207974	cg26547741	cg19541247
732	cg12927715	cg26383057	cg26734040
733	cg22180848	cg26064794	cg10082445
734	cg09935792	cg24738592	cg08677210
735	cg23125506	cg23217547	cg05376267
736	cg10677144	cg22076676	cg13657981
737	cg13863769	cg20418769	cg01272202
738	cg04919234	cg20167126	cg24044501
739	cg09230938	cg19799865	cg25974922

740	cg09175915	cg19683675	cg27042523
741	cg15891850	cg19190487	cg22979433
742	cg07385490	cg17601658	cg05380734
743	cg01077713	cg15364618	cg07165066
744	cg05091570	cg15016740	cg04805065
745	cg22946562	cg14218838	cg22380476
746	cg02928110	cg14159036	cg05945030
747	cg16826504	cg14001518	cg25095994
748	cg21108851	cg13008252	cg17169982
749	cg27409974	cg12312338	cg13724812
750	cg11772801	cg10223066	cg08185095
751	cg12117658	cg09334629	cg13857210
752	cg27394987	cg06937205	cg01356198
753	cg21973907	cg06825317	cg09884107
754	cg26828494	cg06363798	cg14096042
755	cg22890571	cg05242371	cg06251016
756	cg08154963	cg04482110	cg14700524
757	cg27578381	cg04384810	cg17107017
758	cg24249775	cg03538436	cg00464814
759	cg00001809	cg03053456	cg06812840
760	cg00008452	cg02882004	cg12727138
761	cg00013441	cg02522196	cg05900955
762	cg00017461	cg02226672	cg21095280
763	cg00027650	cg01663970	cg27521744
764	cg00027990	cg01079658	cg06786153
765	cg00028338	cg01014262	cg23337031
766	cg00035923	cg00627347	cg16640450
767	cg00038342	cg00449021	cg19815596
768	cg00039385	cg27339974	cg14758740
769	cg00043005	cg27051683	cg00549561
770	cg00044665	cg26873164	cg08276755
771	cg00047338	cg26308909	cg15756507
772	cg00050289	cg25950520	cg14096074
773	cg00054496	cg25408950	cg14242936
774	cg00058291	cg24899558	cg15104081
775	cg00059024	cg22529541	cg23999526
776	cg00060715	cg21397287	cg13472900
777	cg00061185	cg20675852	cg01356872
778	cg00070460	cg20230305	cg11982525
779	cg00074348	cg20147164	cg11576424
780	cg00083399	cg20072162	cg01691710
781	cg00085256	cg19332409	cg06607764
782	cg00086171	cg18653451	cg03074188
783	cg00091004	cg18247852	cg22108360
784	cg00095259	cg17599809	cg10239163
785	cg00095326	cg17528003	cg18939081
786	cg00096810	cg17444642	cg02235663
787	cg00103778	cg17378535	cg25568066
788	cg00118317	cg17107017	cg13447099
789	cg00118783	cg16776331	cg13496979
790	cg00123090	cg16412000	cg21854332
791	cg00127198	cg15844895	cg07613391
792	cg00131423	cg14826031	cg01761758

793	cg00133595	cg13282594	cg19439043
794	cg00134602	cg11205589	cg02249969
795	cg00137696	cg10780981	cg06856329
796	cg00143606	cg09410453	cg11703759
797	cg00144414	cg08833741	cg25654695
798	cg00145911	cg06500727	cg00127198
799	cg00147216	cg04576568	cg06376598
800	cg00147850	cg03879386	cg23344121
801	cg00149708	cg02935097	cg21893764
802	cg00158530	cg02281970	cg04784212
803	cg00166343	cg01675694	cg22663660
804	cg00169184	cg01553584	cg07612468
805	cg00175487	cg26280578	cg05194102
806	cg00175895	cg25575145	cg14024328
807	cg00180097	cg25521853	cg04145890
808	cg00184279	cg24775327	cg11263420
809	cg00185413	cg23976336	cg03038245
810	cg00187535	cg21450652	cg07308243
811	cg00201819	cg21145938	cg20000866
812	cg00204976	cg20647485	cg10617739
813	cg00206063	cg20637405	cg02793948
814	cg00209623	cg19569551	cg01352276
815	cg00209918	cg18851831	cg04113348
816	cg00210271	cg18677603	cg14406727
817	cg00216961	cg18639238	cg14136781
818	cg00218484	cg16640008	cg07925587
819	cg00226615	cg15815827	cg07268431
820	cg00232500	cg15035360	cg02481714
821	cg00236766	cg14406727	cg26978064
822	cg00236831	cg13931531	cg08400424
823	cg00244267	cg13634090	cg15618758
824	cg00244391	cg11848157	cg14362630
825	cg00245896	cg11844358	cg08784950
826	cg00248856	cg09975561	cg08149747
827	cg00257455	cg08764162	cg22782017
828	cg00260235	cg07577837	cg20566286
829	cg00266968	cg07508304	cg08701134
830	cg00268330	cg06241044	cg26639076
831	cg00270141	cg03286742	cg25807487
832	cg00270654	cg00828762	cg05590156
833	cg00271177	cg00704869	cg12452298
834	cg00271311	cg00680875	cg12669088
835	cg00275234	cg00271311	cg10439859
836	cg00275503	cg00035074	cg07824182
837	cg00277804	cg25587431	cg01767116
838	cg00285343	cg24727133	cg01559787
839	cg00286717	cg24293145	cg26573923
840	cg00289254	cg23998645	cg11046864
841	cg00296038	cg23408990	cg12005412
842	cg00308508	cg23040685	cg09989037
843	cg00311883	cg22622667	cg16529592
844	cg00314994	cg21329649	cg12448539
845	cg00322201	cg20382418	cg04293307

846	cg00330518	cg20322433	cg23777946
847	cg00333583	cg19912309	cg24541176
848	cg00334274	cg19598713	cg18120975
849	cg00335361	cg18645642	cg07410597
850	cg00352652	cg18544888	cg04706201
851	cg00353407	cg18369325	cg09801824
852	cg00355286	cg18231184	cg02238387
853	cg00358442	cg18118490	cg09650907
854	cg00359087	cg16883145	cg25352714
855	cg00359365	cg16442814	cg12503110
856	cg00362381	cg15574810	cg00590590
857	cg00366037	cg15082028	cg10803714
858	cg00371570	cg14992217	cg01837846
859	cg00373967	cg13827597	cg07900344
860	cg00374546	cg13515351	cg14553895
861	cg00380428	cg11671925	cg24453118
862	cg00386725	cg11069071	cg08682625
863	cg00392155	cg10827460	cg07662964
864	cg00400654	cg09646934	cg23903708
865	cg00400992	cg09230938	cg06741896
866	cg00401972	cg07561338	cg13643040
867	cg00405824	cg06093152	cg02227879
868	cg00408764	cg05341539	cg18560571
869	cg00409245	cg04819180	cg10338518
870	cg00410534	cg04707715	cg15425427
871	cg00412142	cg04046364	cg27365627
872	cg00412772	cg03837909	cg05150697
873	cg00412775	cg02711397	cg18677603
874	cg00414709	cg02144874	cg04070601
875	cg00415011	cg00949635	cg00651488
876	cg00418613	cg00900735	cg12744447
877	cg00421693	cg00869582	cg18703227
878	cg00423871	cg27604702	cg19770281
879	cg00426425	cg27543578	cg14231959
880	cg00428526	cg27302301	cg01546047
881	cg00431813	cg27161027	cg10588276
882	cg00436722	cg26071556	cg02369775
883	cg00442220	cg25828334	cg12070646
884	cg00444740	cg25510609	cg11288260
885	cg00446536	cg24812143	cg01361499
886	cg00449021	cg24804060	cg11363168
887	cg00452755	cg24137774	cg11609668
888	cg00454770	cg23949216	cg13431342
889	cg00460268	cg23261343	cg14833626
890	cg00464814	cg22370005	cg02901644
891	cg00469547	cg22022181	cg26231761
892	cg00470794	cg21736854	cg11961845
893	cg00470817	cg20242065	cg18960324
894	cg00472277	cg19244553	cg21658153
895	cg00473257	cg18857655	cg00998132
896	cg00484122	cg17616283	cg06556244
897	cg00488512	cg16812352	cg22189544
898	cg00488575	cg16803044	cg07990658

899	cg00488716	cg16734433	cg20072162
900	cg00489394	cg16572603	cg10811426
901	cg00500424	cg16133703	cg25623727
902	cg00501242	cg15396800	cg12058875
903	cg00503920	cg15353597	cg04034685
904	cg00512872	cg14553895	cg14201946
905	cg00523012	cg14094409	cg26800893
906	cg00528490	cg13840520	cg04356968
907	cg00542261	cg13514778	cg09437283
908	cg00545796	cg13488851	cg10248100
909	cg00547727	cg13274107	cg22151881
910	cg00547768	cg12727940	cg09457490
911	cg00548009	cg12120430	cg08883078
912	cg00549561	cg11815790	cg01385157
913	cg00549798	cg11351841	cg11341086
914	cg00550040	cg10857774	cg08749443
915	cg00552168	cg10255535	cg13840520
916	cg00554604	cg10024484	cg14178043
917	cg00556407	cg09832108	cg10709026
918	cg00562504	cg09775533	cg12075928
919	cg00563061	cg09524639	cg05982473
920	cg00567872	cg09164574	cg15364618
921	cg00569091	cg09038962	cg21374048
922	cg00578637	cg08571020	cg06784602
923	cg00581541	cg08005460	cg03040489
924	cg00585698	cg07743919	cg18935449
925	cg00585818	cg07531248	cg23049758
926	cg00586700	cg07512971	cg25854527
927	cg00589581	cg07410597	cg09811393
928	cg00590590	cg06504636	cg03501387
929	cg00592944	cg06376426	cg02344497
930	cg00594228	cg05873568	cg20067780
931	cg00594917	cg05229802	cg02836864
932	cg00595472	cg05030450	cg07803375
933	cg00604356	cg05013912	cg07041720
934	cg00606396	cg04685228	cg19799865
935	cg00607630	cg03817675	cg26177041
936	cg00607989	cg02785870	cg16077055
937	cg00610205	cg02150714	cg09256716
938	cg00618323	cg01303236	cg22871566
939	cg00622702	cg01259126	cg06033488
940	cg00623312	cg00920668	cg00334274
941	cg00624949	cg00118317	cg01120132
942	cg00626390	cg27604101	cg10604241
943	cg00627347	cg26828839	cg16479633
944	cg00631702	cg26808779	cg27560818
945	cg00637826	cg26169991	cg18436810
946	cg00639143	cg25203627	cg00216961
947	cg00639837	cg24247537	cg17722435
948	cg00647879	cg24194998	cg06340118
949	cg00651488	cg24064506	cg05909702
950	cg00651497	cg23999526	cg25461775
951	cg00656264	cg23173517	cg05426006

952	cg00656991	cg23144649	cg23037644
953	cg00657810	cg20386404	cg17216243
954	cg00663077	cg19691659	cg01441113
955	cg00665106	cg18929814	cg10921702
956	cg00667680	cg18668813	cg08044294
957	cg00669182	cg17898124	cg12999366
958	cg00669856	cg16823064	cg11492886
959	cg00670539	cg16680624	cg13586038
960	cg00673191	cg15968604	cg25189808
961	cg00674220	cg15765251	cg20322433
962	cg00675878	cg13953458	cg12669271
963	cg00676360	cg13851989	cg13402847
964	cg00677217	cg13500797	cg17548735
965	cg00681103	cg12609948	cg17920820
966	cg00686915	cg12467960	cg09119854
967	cg00694888	cg11546709	cg10434728
968	cg00699693	cg09358973	cg19774846
969	cg00701514	cg08843064	cg22153312
970	cg00701856	cg07328519	cg03829194
971	cg00704869	cg07133097	cg10498921
972	cg00706207	cg07099056	cg15417249
973	cg00707452	cg06566615	cg06290096
974	cg00710466	cg05503991	cg27554156
975	cg00711291	cg05388880	cg16151538
976	cg00711584	cg04961225	cg18163092
977	cg00713253	cg04681879	cg10907727
978	cg00713972	cg04547809	cg17827670
979	cg00715343	cg03888083	cg16874347
980	cg00718694	cg03812676	cg01303055
981	cg00718831	cg03143347	cg04719721
982	cg00729995	cg02767093	cg00610577
983	cg00732878	cg00830283	cg12659065
984	cg00738945	cg00711291	cg05591270
985	cg00742738	cg00488575	cg23058673
986	cg00747342	cg27571590	cg07795968
987	cg00750074	cg27567761	cg22798362
988	cg00753492	cg27410449	cg17943672
989	cg00755287	cg27127017	cg09280800
990	cg00757262	cg26894079	cg07965110
991	cg00757391	cg26127113	cg25408950
992	cg00757822	cg25865108	cg25589001
993	cg00760872	cg25479732	cg10979364
994	cg00765705	cg25189085	cg17749384
995	cg00776208	cg24600706	cg15988734
996	cg00777079	cg24297835	cg08089518
997	cg00784105	cg23723410	cg10418812
998	cg00785482	cg23619577	cg13857933
999	cg00786138	cg23375717	cg19974227
1000	cg00789416	cg23337031	cg08688659
1001	cg00802617	cg22058664	cg04505809
1002	cg00806009	cg19632842	cg03296204
1003	cg00808555	cg19508622	cg22964758
1004	cg00810292	cg19360685	cg13066703

1005	cg00816182	cg17650713	cg21244322
1006	cg00818557	cg17508905	cg09702334
1007	cg00820405	cg17216243	cg00405824
1008	cg00825317	cg15895197	cg18729681
1009	cg00827581	cg15639581	cg05966978
1010	cg00828762	cg13298379	cg01176028
1011	cg00845883	cg12522144	cg19287349
1012	cg00851981	cg12362980	cg06659338
1013	cg00852414	cg12072973	cg07270259
1014	cg00854594	cg11695684	cg08719095
1015	cg00857907	cg10584300	cg13550401
1016	cg00860425	cg10480329	cg14003974
1017	cg00863271	cg10371037	cg23273465
1018	cg00863893	cg10149889	cg25406657
1019	cg00864551	cg10099813	cg24812143
1020	cg00866186	cg09911316	cg21241424
1021	cg00869941	cg09529783	cg18544888
1022	cg00869989	cg09511662	cg05370752
1023	cg00870269	cg08920210	cg04743876
1024	cg00872170	cg08565675	cg26426334
1025	cg00873009	cg08329113	cg10102102
1026	cg00876376	cg08291302	cg07561547
1027	cg00876694	cg07617152	cg18918538
1028	cg00881300	cg07481273	cg02338778
1029	cg00885461	cg06561892	cg26947127
1030	cg00890378	cg05715751	cg21840948
1031	cg00892999	cg05099221	cg26460678
1032	cg00899463	cg05016508	cg02461114
1033	cg00899856	cg02704946	cg08293102
1034	cg00900735	cg02633600	cg06109284
1035	cg00901982	cg01848660	cg16723180
1036	cg00902427	cg26929394	cg12069547
1037	cg00903438	cg26551200	cg06580014
1038	cg00904122	cg26452091	cg22418565
1039	cg00904578	cg26368942	cg06656553
1040	cg00906241	cg26224624	cg02867857
1041	cg00912277	cg26070874	cg07467854
1042	cg00916659	cg26002713	cg08169341
1043	cg00918130	cg25938735	cg26350921
1044	cg00919689	cg24734735	cg01678309
1045	cg00920069	cg23539753	cg23322122
1046	cg00920668	cg23408104	cg04961225
1047	cg00927435	cg23079522	cg17985555
1048	cg00934066	cg22108360	cg19507725
1049	cg00935361	cg21242508	cg01108243
1050	cg00936895	cg21143086	cg23682934
1051	cg00936935	cg19755886	cg17394978
1052	cg00937742	cg18977727	cg27437304
1053	cg00938266	cg18617669	cg17475304
1054	cg00941576	cg18338925	cg09279803
1055	cg00941900	cg17758899	cg20829550
1056	cg00944304	cg17332023	cg18998442
1057	cg00945443	cg17099569	cg13408795

1058	cg00947413	cg16617774	cg16565294
1059	cg00948881	cg16337430	cg07795346
1060	cg00950038	cg15633699	cg04384810
1061	cg00950412	cg14691924	cg24090911
1062	cg00951857	cg14606082	cg13206932
1063	cg00952822	cg13608684	cg11879277
1064	cg00954841	cg13576994	cg16897462
1065	cg00961416	cg13066703	cg26605683
1066	cg00967711	cg12832565	cg12494355
1067	cg00968638	cg12219134	cg04717045
1068	cg00969565	cg12092201	cg20418769
1069	cg00978248	cg11585301	cg18352616
1070	cg00981003	cg11251006	cg05168368
1071	cg00981877	cg10691866	cg00748072
1072	cg00982952	cg10588276	cg10558233
1073	cg00989505	cg10152624	cg19289969
1074	cg00990874	cg09400566	cg05388880
1075	cg00991659	cg09368832	cg07260789
1076	cg00994389	cg09258240	cg16526755
1077	cg00998132	cg09075515	cg02570643
1078	cg00999267	cg08850243	cg23233631
1079	cg01000937	cg08450122	cg18637761
1080	cg01004063	cg08065374	cg26709300
1081	cg01007781	cg07306006	cg03035704
1082	cg01013023	cg07241090	cg04305601
1083	cg01014262	cg06241812	cg19640166
1084	cg01017228	cg06116260	cg14978242
1085	cg01017395	cg05116002	cg06061760
1086	cg01017397	cg04867652	cg11958644
1087	cg01023169	cg04688645	cg12865939
1088	cg01023808	cg04034685	cg07108443
1089	cg01032946	cg03655701	cg21964551
1090	cg01038640	cg03543120	cg25487008
1091	cg01040499	cg03538922	cg11425149
1092	cg01044293	cg03420242	cg07894983
1093	cg01044693	cg02289020	cg15888569
1094	cg01046905	cg01992684	cg01669185
1095	cg01048810	cg01872216	cg13225881
1096	cg01052428	cg01716084	cg04462774
1097	cg01055121	cg00647879	cg12417815
1098	cg01060040	cg27346937	cg26413192
1099	cg01062470	cg27120246	cg11996592
1100	cg01062651	cg26797297	cg22579075
1101	cg01063615	cg26692003	cg23398508
1102	cg01063813	cg26601559	cg11976592
1103	cg01065180	cg26152188	cg10388307
1104	cg01066494	cg25616055	cg01360115
1105	cg01069043	cg23421128	cg03529015
1106	cg01079658	cg22427797	cg20070852
1107	cg01084215	cg20725704	cg18050634
1108	cg01088579	cg20048529	cg18556792
1109	cg01089364	cg19827089	cg22325145
1110	cg01094351	cg18487916	cg17027476

1111	cg01096199	cg16453673	cg18485877
1112	cg01099855	cg15854022	cg10989175
1113	cg01101448	cg15702277	cg14487665
1114	cg01103253	cg14833626	cg09400566
1115	cg01104466	cg14078730	cg19638572
1116	cg01108243	cg13461509	cg13877974
1117	cg01110955	cg13431028	cg10734581
1118	cg01120132	cg12156838	cg09331409
1119	cg01122461	cg11843691	cg10532384
1120	cg01138652	cg09906558	cg10721220
1121	cg01139503	cg09666417	cg02572552
1122	cg01147665	cg09567735	cg02281970
1123	cg01150866	cg09356020	cg25268422
1124	cg01153620	cg08419373	cg12108265
1125	cg01154505	cg07872947	cg23415916
1126	cg01156295	cg07608094	cg23486701
1127	cg01158079	cg07456413	cg13871900
1128	cg01166827	cg07067993	cg06061092
1129	cg01170069	cg06790069	cg14992217
1130	cg01176028	cg06415302	cg16738646
1131	cg01176141	cg05539509	cg25638714
1132	cg01176823	cg04352763	cg08397968
1133	cg01188722	cg03269976	cg25341653
1134	cg01195672	cg02855309	cg23350812
1135	cg01197763	cg02473287	cg21133992
1136	cg01201519	cg02227879	cg06918474
1137	cg01204060	cg01788682	cg22343299
1138	cg01214847	cg27009448	cg17638841
1139	cg01227084	cg26695387	cg20197694
1140	cg01228193	cg26618058	cg00952822
1141	cg01246855	cg26152051	cg22655196
1142	cg01254644	cg24969303	cg03407184
1143	cg01256365	cg24893073	cg03393445
1144	cg01259126	cg24270629	cg08437802
1145	cg01260146	cg23908771	cg10095226
1146	cg01260820	cg23711760	cg18634690
1147	cg01262865	cg23507051	cg07102397
1148	cg01267373	cg23345038	cg23318523
1149	cg01274028	cg22946562	cg16711983
1150	cg01274233	cg22800332	cg21376658
1151	cg01277910	cg22786667	cg20080282
1152	cg01283625	cg22700246	cg08264481
1153	cg01288155	cg22512847	cg24691835
1154	cg01290421	cg21687003	cg00945443
1155	cg01293277	cg21457110	cg13099139
1156	cg01294717	cg21241424	cg03217729
1157	cg01296758	cg21238061	cg01827633
1158	cg01297744	cg20941528	cg08419373
1159	cg01298514	cg20713092	cg18304186
1160	cg01298912	cg20436707	cg14377596
1161	cg01303055	cg20323175	cg14524975
1162	cg01303236	cg19040077	cg13269555
1163	cg01303480	cg17291826	cg15587041

1164	cg01306747	cg17276624	cg19514469
1165	cg01306824	cg16283362	cg19090861
1166	cg01314034	cg15121364	cg14059665
1167	cg01314597	cg14506192	cg02522196
1168	cg01323212	cg13973594	cg11945474
1169	cg01324452	cg13881619	cg18905668
1170	cg01329151	cg12417362	cg11562411
1171	cg01330448	cg12108265	cg23956760
1172	cg01333205	cg11915444	cg22095128
1173	cg01337429	cg10237442	cg02449166
1174	cg01337736	cg09956907	cg24468934
1175	cg01338955	cg09544892	cg17289222
1176	cg01339351	cg09006420	cg10024583
1177	cg01342572	cg08351911	cg03773809
1178	cg01343045	cg08240917	cg21637392
1179	cg01343936	cg07612468	cg14769121
1180	cg01345395	cg07192772	cg23976499
1181	cg01349691	cg06784563	cg16407998
1182	cg01350190	cg05541267	cg24969303
1183	cg01352276	cg05139187	cg13264394
1184	cg01356198	cg04842880	cg03041650
1185	cg01357897	cg04838832	cg10639411
1186	cg01360115	cg04113348	cg07969918
1187	cg01362762	cg03991120	cg04868238
1188	cg01364674	cg03227104	cg04964845
1189	cg01369033	cg03212620	cg08681293
1190	cg01370334	cg02700194	cg02659431
1191	cg01377082	cg02665727	cg11964216
1192	cg01380884	cg01753176	cg16681436
1193	cg01388243	cg01716016	cg13148544
1194	cg01390647	cg27360231	cg08154963
1195	cg01398428	cg27241873	cg03769734
1196	cg01400750	cg26607933	cg15125438
1197	cg01402099	cg26441486	cg00637745
1198	cg01403055	cg26411080	cg12036877
1199	cg01403803	cg26204042	cg05826596
1200	cg01406317	cg25339408	cg26543333
1201	cg01411786	cg24479752	cg15420720
1202	cg01413698	cg23722792	cg12657361
1203	cg01415527	cg23207527	cg20663495
1204	cg01422009	cg22501449	cg05286252
1205	cg01422416	cg22402769	cg07012484
1206	cg01428849	cg22366375	cg18252102
1207	cg01428928	cg21532408	cg13586696
1208	cg01432405	cg21189146	cg03187301
1209	cg01436550	cg20999795	cg12183501
1210	cg01439112	cg20773915	cg05469118
1211	cg01442132	cg20692268	cg08236537
1212	cg01442620	cg20673255	cg08088222
1213	cg01443467	cg20000539	cg21850879
1214	cg01449168	cg19821361	cg12343881
1215	cg01449425	cg19589800	cg02804722
1216	cg01452115	cg19109601	cg02193283

1217	cg01454538	cg18917736	cg15657100
1218	cg01456030	cg18095041	cg20723705
1219	cg01456989	cg17781669	cg06509362
1220	cg01460382	cg17578309	cg24940701
1221	cg01467882	cg17009731	cg14878988
1222	cg01475325	cg16502866	cg16133703
1223	cg01481251	cg15963326	cg08897688
1224	cg01482620	cg14569423	cg15209369
1225	cg01483459	cg14426682	cg01579765
1226	cg01486610	cg13924326	cg15016624
1227	cg01486910	cg13815311	cg11935248
1228	cg01487195	cg13657511	cg08104146
1229	cg01493020	cg13215078	cg09863094
1230	cg01494593	cg12817154	cg01828733
1231	cg01497892	cg12692727	cg18818075
1232	cg01501018	cg11835347	cg19257111
1233	cg01503773	cg11588197	cg13867915
1234	cg01512113	cg10488777	cg25864762
1235	cg01514831	cg10095352	cg27655512
1236	cg01518755	cg09501516	cg16427420
1237	cg01519195	cg09474229	cg07125976
1238	cg01523759	cg08576185	cg08764162
1239	cg01527159	cg06212263	cg20165037
1240	cg01537847	cg06113801	cg03784886
1241	cg01543307	cg05651243	cg04458219
1242	cg01545140	cg05376267	cg21335012
1243	cg01553388	cg04843968	cg22986770
1244	cg01553584	cg02644867	cg14329833
1245	cg01553866	cg02566359	cg00330518
1246	cg01558195	cg02528008	cg00760872
1247	cg01559031	cg02393496	cg18459869
1248	cg01559356	cg02304427	cg10233454
1249	cg01559787	cg01519195	cg19961522
1250	cg01570589	cg01361499	cg17672035
1251	cg01576275	cg01246855	cg25684961
1252	cg01577298	cg01040499	cg06254425
1253	cg01578585	cg00663077	cg04684246
1254	cg01578875	cg00578637	cg02712546
1255	cg01579765	cg00437969	cg18657988
1256	cg01580176	cg00358442	cg25389863
1257	cg01581360	cg00218484	cg16387467
1258	cg01589353	cg27618145	cg04774476
1259	cg01589629	cg26710819	cg20166714
1260	cg01592387	cg26642540	cg08365638
1261	cg01596854	cg26313421	cg27618305
1262	cg01598009	cg25517015	cg21189849
1263	cg01604401	cg25117523	cg19643097
1264	cg01604946	cg25038926	cg24403487
1265	cg01606770	cg24260710	cg09719124
1266	cg01618851	cg24065957	cg01968525
1267	cg01628053	cg22737001	cg02243479
1268	cg01634146	cg22525688	cg17473727
1269	cg01637244	cg22459081	cg17587997

1270	cg01645401	cg22040672	cg26929394
1271	cg01653532	cg22021794	cg08523978
1272	cg01655150	cg21222559	cg22451265
1273	cg01656717	cg21159068	cg24004007
1274	cg01663970	cg20156139	cg18869485
1275	cg01664382	cg19970953	cg03044471
1276	cg01667978	cg19668990	cg06400255
1277	cg01669185	cg19441674	cg02688118
1278	cg01676623	cg19358589	cg22123915
1279	cg01691710	cg18566883	cg07085590
1280	cg01693662	cg17766055	cg15816464
1281	cg01705036	cg16778107	cg17820025
1282	cg01715499	cg16745596	cg02196592
1283	cg01716016	cg16572410	cg15022671
1284	cg01716827	cg16290996	cg05307141
1285	cg01719663	cg15944060	cg09925747
1286	cg01722498	cg15475080	cg09040552
1287	cg01723163	cg15420720	cg02317397
1288	cg01727408	cg14947411	cg17702736
1289	cg01730064	cg14783283	cg19317226
1290	cg01733438	cg14210790	cg15991082
1291	cg01741616	cg14035238	cg27039312
1292	cg01749725	cg13477354	cg10809958
1293	cg01753176	cg13447933	cg22427797
1294	cg01753263	cg11850468	cg23920016
1295	cg01758022	cg11786870	cg12266049
1296	cg01759870	cg11637968	cg18034859
1297	cg01761758	cg11576424	cg21439672
1298	cg01767116	cg10677144	cg09074223
1299	cg01770296	cg10512951	cg22430861
1300	cg01778908	cg10507281	cg19235974
1301	cg01778994	cg10215501	cg20780180
1302	cg01789743	cg09912552	cg10016358
1303	cg01791238	cg08954601	cg08947774
1304	cg01792640	cg08934799	cg27454102
1305	cg01793617	cg07965566	cg26828839
1306	cg01794853	cg07716847	cg05060686
1307	cg01794926	cg07295918	cg04654299
1308	cg01804278	cg07206497	cg13877631
1309	cg01809895	cg06890747	cg25568490
1310	cg01810186	cg06445533	cg10681992
1311	cg01810347	cg05261433	cg22171607
1312	cg01810684	cg05162523	cg21879102
1313	cg01818102	cg05051393	cg14741474
1314	cg01818539	cg04993279	cg00873009
1315	cg01820765	cg04550439	cg03430597
1316	cg01824284	cg04249605	cg13278004
1317	cg01824933	cg03727333	cg02061820
1318	cg01825806	cg03511735	cg05299486
1319	cg01838863	cg03344672	cg24429037
1320	cg01848660	cg03278299	cg20336472
1321	cg01849845	cg02979010	cg01514831
1322	cg01851970	cg02426072	cg04819655

1323	cg01852611	cg02278728	cg02512395
1324	cg01859228	cg02115911	cg25909064
1325	cg01859460	cg01559031	cg02607972
1326	cg01863398	cg00552168	cg00578637
1327	cg01866330	cg00050289	cg13107973
1328	cg01866959	cg27635271	cg05771342
1329	cg01869896	cg26915618	cg12555086
1330	cg01870865	cg26899399	cg21460582
1331	cg01870976	cg26668713	cg23539494
1332	cg01871631	cg26066560	cg05976168
1333	cg01876978	cg25993608	cg15317049
1334	cg01881549	cg25920512	cg19770671
1335	cg01885814	cg25389863	cg03141944
1336	cg01887374	cg25341653	cg05817833
1337	cg01902704	cg25264265	cg25117523
1338	cg01903305	cg24873093	cg07283859
1339	cg01914059	cg24212377	cg11822372
1340	cg01922697	cg23656110	cg17340779
1341	cg01924223	cg23630423	cg14467840
1342	cg01930756	cg23378722	cg20692268
1343	cg01930924	cg23366234	cg10427430
1344	cg01932663	cg23252698	cg01274028
1345	cg01937669	cg22430861	cg25197500
1346	cg01938025	cg21386766	cg03437713
1347	cg01942927	cg20780180	cg21643731
1348	cg01943414	cg20664445	cg10327980
1349	cg01945624	cg20218614	cg06702537
1350	cg01946510	cg19918057	cg11909137
1351	cg01949455	cg19289969	cg07866001
1352	cg01954438	cg18697143	cg01902704
1353	cg01957900	cg17724175	cg11919577
1354	cg01959071	cg17586345	cg26224624
1355	cg01959238	cg16826504	cg04133502
1356	cg01959262	cg16422492	cg22584802
1357	cg01968002	cg16234490	cg06422947
1358	cg01968525	cg16026813	cg19270309
1359	cg01971085	cg15911948	cg13626676
1360	cg01989275	cg14692453	cg01838863
1361	cg01991625	cg14244360	cg16446824
1362	cg01992684	cg14207326	cg23475725
1363	cg01997461	cg14095720	cg02873783
1364	cg02003272	cg13948956	cg19480117
1365	cg02003612	cg13784276	cg16889721
1366	cg02008511	cg13662628	cg04751761
1367	cg02010047	cg13537008	cg11828741
1368	cg02011409	cg12117658	cg17173086
1369	cg02013018	cg11909989	cg02250553
1370	cg02015219	cg11043092	cg02550308
1371	cg02017718	cg10626305	cg26026296
1372	cg02021485	cg10604241	cg25865108
1373	cg02025435	cg10472320	cg08576185
1374	cg02034328	cg09913449	cg11850468
1375	cg02040734	cg09727436	cg19623406

1376	cg02043994	cg09643151	cg00166343
1377	cg02048412	cg09307742	cg02469356
1378	cg02053092	cg09276451	cg23280294
1379	cg02055483	cg08929612	cg20677267
1380	cg02058267	cg08719095	cg20721135
1381	cg02060039	cg07835154	cg26495716
1382	cg02060434	cg07103201	cg13471336
1383	cg02061820	cg06787609	cg02386311
1384	cg02061967	cg06183287	cg21818807
1385	cg02062816	cg05898188	cg20309371
1386	cg02065141	cg05798436	cg13420004
1387	cg02066343	cg04397884	cg21251203
1388	cg02066441	cg04349839	cg10036892
1389	cg02067239	cg04198125	cg12692386
1390	cg02069095	cg04002957	cg13661703
1391	cg02069944	cg03168587	cg04716580
1392	cg02078525	cg03078718	cg18700940
1393	cg02083559	cg02998075	cg02528008
1394	cg02083676	cg02816367	cg07116732
1395	cg02084214	cg02648064	cg00954841
1396	cg02084834	cg02055483	cg09921821
1397	cg02084948	cg01576275	cg01101448
1398	cg02087101	cg01228193	cg24437859
1399	cg02102075	cg00990874	cg22413209
1400	cg02107173	cg00876376	cg01462184
1401	cg02108015	cg00512280	cg04431002
1402	cg02110527	cg00158770	cg01048810
1403	cg02112621	cg27573308	cg07103201
1404	cg02113385	cg27143326	cg16175077
1405	cg02113604	cg26656452	cg12232388
1406	cg02116283	cg26650973	cg02723533
1407	cg02119693	cg26166004	cg14041194
1408	cg02122052	cg25552705	cg24870662
1409	cg02124957	cg23304923	cg26311262
1410	cg02131465	cg23143093	cg13283765
1411	cg02133557	cg22971402	cg25369553
1412	cg02137970	cg22890896	cg02737507
1413	cg02138124	cg22605643	cg00941900
1414	cg02144518	cg22448292	cg17297775
1415	cg02144874	cg21757281	cg19582744
1416	cg02146941	cg21756355	cg06853492
1417	cg02147208	cg21475076	cg11438039
1418	cg02148034	cg20916646	cg00686915
1419	cg02150714	cg20899781	cg16452866
1420	cg02153814	cg20739726	cg24335984
1421	cg02153855	cg20462191	cg00442802
1422	cg02156952	cg20100049	cg12526923
1423	cg02157002	cg19992808	cg01818102
1424	cg02157052	cg19863040	cg16976520
1425	cg02157454	cg19793499	cg10950593
1426	cg02159643	cg18998365	cg08616182
1427	cg02162605	cg18772205	cg15507500
1428	cg02165692	cg18751958	cg24852135

1429	cg02172773	cg18738548	cg01983725
1430	cg02175033	cg18710162	cg04100595
1431	cg02190127	cg18634690	cg18729298
1432	cg02191483	cg18560571	cg05824594
1433	cg02192555	cg17634490	cg13560612
1434	cg02193283	cg17344813	cg09580592
1435	cg02193967	cg17115147	cg24288527
1436	cg02196592	cg17112695	cg06241812
1437	cg02197542	cg16306083	cg20516209
1438	cg02202664	cg16277214	cg05585379
1439	cg02212339	cg16239826	cg05888037
1440	cg02214441	cg16073408	cg10480329
1441	cg02216206	cg15941159	cg19006220
1442	cg02217035	cg15262242	cg06492744
1443	cg02225004	cg15249446	cg15556723
1444	cg02226672	cg14852276	cg00089486
1445	cg02227879	cg14694342	cg27399387
1446	cg02228688	cg14461852	cg01428849
1447	cg02235663	cg14437446	cg04438661
1448	cg02237875	cg14117392	cg10488777
1449	cg02238387	cg13079633	cg25317315
1450	cg02240030	cg12796332	cg11190278
1451	cg02240936	cg11009590	cg26092233
1452	cg02243479	cg10877385	cg22152438
1453	cg02244933	cg10740573	cg02882004
1454	cg02246916	cg10457066	cg06208158
1455	cg02247300	cg10063637	cg13556624
1456	cg02249969	cg09647797	cg26449178
1457	cg02250553	cg09631059	cg14651518
1458	cg02255721	cg09602138	cg20482280
1459	cg02262037	cg09100695	cg06719042
1460	cg02264288	cg08215532	cg26889953
1461	cg02272457	cg07986222	cg07425568
1462	cg02272751	cg07691914	cg14101687
1463	cg02273647	cg07665060	cg01096199
1464	cg02274236	cg07561547	cg08351911
1465	cg02278728	cg07236812	cg27273675
1466	cg02281970	cg06933824	cg11724511
1467	cg02283353	cg06765552	cg16564135
1468	cg02287325	cg05867158	cg09526469
1469	cg02297063	cg05854826	cg05054460
1470	cg02304222	cg05595142	cg06824199
1471	cg02304427	cg05483875	cg01422009
1472	cg02304580	cg05426006	cg19025193
1473	cg02306481	cg05002642	cg16222367
1474	cg02315513	cg04948014	cg08378932
1475	cg02317251	cg04944536	cg02574861
1476	cg02317397	cg03784886	cg20428133
1477	cg02323633	cg03701759	cg17384769
1478	cg02327654	cg03639170	cg05091570
1479	cg02331025	cg03573445	cg19725377
1480	cg02332902	cg03291336	cg13930682
1481	cg02338778	cg03217729	cg05515072

1482	cg02339793	cg02966841	cg11632592
1483	cg02340818	cg02928110	cg13475704
1484	cg02344497	cg02567151	cg21035368
1485	cg02348449	cg02374486	cg17094249
1486	cg02353448	cg01870865	cg02113385
1487	cg02358804	cg01545140	cg04454664
1488	cg02362505	cg01099855	cg23732483
1489	cg02363010	cg01089364	cg19018155
1490	cg02363969	cg00944304	cg21158528
1491	cg02364518	cg00818557	cg16927379
1492	cg02364610	cg00623312	cg25852019
1493	cg02366931	cg00334274	cg19016264
1494	cg02367992	cg27059537	cg15396800
1495	cg02369775	cg26552621	cg10364862
1496	cg02372856	cg25971741	cg15074047
1497	cg02374486	cg25878830	cg08864083
1498	cg02384859	cg25590335	cg19921577
1499	cg02386311	cg25373624	cg04046364
1500	cg02393496	cg25301103	cg09657114
1501	cg02399019	cg25048985	cg08869160
1502	cg02399294	cg24925163	cg27110374
1503	cg02402630	cg24859433	cg13821008
1504	cg02418175	cg24083817	cg05482973
1505	cg02421824	cg23903708	cg05051393
1506	cg02426072	cg23493119	cg05386977
1507	cg02435083	cg22240998	cg00777079
1508	cg02436098	cg22197033	cg04307274
1509	cg02438575	cg21854332	cg12866104
1510	cg02447268	cg21670987	cg07826642
1511	cg02447556	cg21460686	cg22488259
1512	cg02449166	cg21032292	cg06518233
1513	cg02451516	cg20210637	cg26448609
1514	cg02454890	cg20027331	cg20700977
1515	cg02455836	cg19513744	cg27212729
1516	cg02461114	cg19259111	cg12686055
1517	cg02462416	cg18938534	cg02722657
1518	cg02463970	cg18817654	cg22119466
1519	cg02468821	cg17327331	cg10677144
1520	cg02469356	cg17196448	cg25794830
1521	cg02469916	cg17013513	cg19354017
1522	cg02473287	cg16927379	cg15894581
1523	cg02480405	cg16636756	cg10488292
1524	cg02481307	cg16616370	cg14701108
1525	cg02481714	cg16366639	cg18148375
1526	cg02486181	cg16293569	cg08369014
1527	cg02492708	cg15336269	cg08833741
1528	cg02493905	cg15089806	cg03608520
1529	cg02495250	cg14835545	cg11966063
1530	cg02495395	cg14701108	cg09075515
1531	cg02495413	cg14558880	cg14210790
1532	cg02509027	cg14144513	cg01794853
1533	cg02512395	cg13716849	cg06755413
1534	cg02517473	cg13263472	cg26932693

1535	cg02524863	cg13036546	cg18250135
1536	cg02538557	cg12817352	cg23019125
1537	cg02550308	cg12786452	cg20210151
1538	cg02552250	cg12485685	cg01604401
1539	cg02556345	cg12163952	cg00359365
1540	cg02560388	cg12079279	cg26638069
1541	cg02563156	cg12076543	cg06172939
1542	cg02566359	cg11979837	cg14788686
1543	cg02567151	cg11698119	cg00592695
1544	cg02569718	cg11628781	cg20169015
1545	cg02570361	cg11583751	cg08293075
1546	cg02570643	cg11344729	cg21719704
1547	cg02572552	cg11018723	cg00232535
1548	cg02574861	cg10931901	cg08039592
1549	cg02581587	cg10515332	cg03667405
1550	cg02582619	cg10214757	cg22989033
1551	cg02583282	cg09863094	cg25588389
1552	cg02588098	cg09835239	cg03654273
1553	cg02592525	cg09807875	cg16521032
1554	cg02596645	cg09695261	cg01452115
1555	cg02597698	cg09159050	cg12944530
1556	cg02598618	cg08964948	cg07835154
1557	cg02604890	cg08737189	cg22442617
1558	cg02604995	cg08463775	cg07549381
1559	cg02605292	cg08185095	cg10278297
1560	cg02607544	cg07132183	cg27494100
1561	cg02607972	cg07012484	cg06749819
1562	cg02613295	cg06853455	cg01824603
1563	cg02613380	cg06272543	cg03576555
1564	cg02619478	cg06126019	cg17171539
1565	cg02621481	cg05966641	cg06272543
1566	cg02625138	cg05750926	cg21214613
1567	cg02627286	cg05634149	cg01298514
1568	cg02628107	cg05258102	cg25587431
1569	cg02628360	cg05004818	cg23309670
1570	cg02631921	cg04890137	cg05145297
1571	cg02633363	cg04458219	cg26645242
1572	cg02633409	cg04384031	cg14684068
1573	cg02635482	cg04381888	cg24212377
1574	cg02638755	cg04210082	cg19266014
1575	cg02643834	cg03631837	cg07478501
1576	cg02644867	cg03526625	cg03583746
1577	cg02645302	cg03316628	cg07904865
1578	cg02648064	cg03138206	cg16452651
1579	cg02648939	cg03074188	cg07109453
1580	cg02650017	cg03042971	cg20447038
1581	cg02654449	cg02667335	cg24896649
1582	cg02656049	cg02153855	cg01099855
1583	cg02659431	cg02102075	cg18537410
1584	cg02660541	cg01876978	cg07409629
1585	cg02665399	cg01640684	cg06791695
1586	cg02665727	cg00656264	cg02931058
1587	cg02667335	cg00007036	cg25688583

1588	cg02667880	cg27627006	cg14035238
1589	cg02685896	cg27280313	cg19410796
1590	cg02688118	cg27222884	cg21177165
1591	cg02693127	cg27117828	cg16318412
1592	cg02695969	cg26449178	cg15335728
1593	cg02697979	cg26423542	cg21364077
1594	cg02700194	cg26325286	cg25567938
1595	cg02704331	cg26034341	cg07048516
1596	cg02704946	cg25731074	cg27067922
1597	cg02707799	cg25479682	cg17213154
1598	cg02710481	cg25013910	cg03615933
1599	cg02712546	cg24875593	cg15408497
1600	cg02714192	cg24830876	cg27375286
1601	cg02718078	cg24257168	cg24463664
1602	cg02723533	cg22913843	cg12491114
1603	cg02725620	cg22834511	cg13981325
1604	cg02727423	cg22685009	cg06342954
1605	cg02729303	cg22418565	cg22982093
1606	cg02730804	cg22342925	cg17903316
1607	cg02733842	cg21973907	cg02365900
1608	cg02734600	cg21936959	cg27117828
1609	cg02737507	cg21893764	cg10514113
1610	cg02742533	cg21855211	cg08426200
1611	cg02743136	cg21727276	cg16668903
1612	cg02753511	cg21528710	cg22622505
1613	cg02753990	cg21108851	cg05700348
1614	cg02762546	cg20185083	cg12987059
1615	cg02764093	cg20154222	cg13414654
1616	cg02767093	cg19747744	cg23021584
1617	cg02776768	cg19271753	cg01937669
1618	cg02780210	cg18553570	cg04508114
1619	cg02785745	cg17670999	cg24073994
1620	cg02785870	cg17569754	cg13708908
1621	cg02793948	cg17256760	cg13477354
1622	cg02800817	cg16752592	cg18794809
1623	cg02804722	cg16510440	cg10273135
1624	cg02806032	cg15970457	cg09196257
1625	cg02806704	cg15661715	cg11952493
1626	cg02807008	cg15191744	cg21223341
1627	cg02816367	cg15188939	cg19233001
1628	cg02816525	cg15076811	cg10904740
1629	cg02817932	cg15033036	cg24766229
1630	cg02818775	cg14482093	cg13276704
1631	cg02821693	cg14467415	cg22730007
1632	cg02823137	cg14260162	cg26894079
1633	cg02825527	cg14189391	cg21779805
1634	cg02827132	cg13994897	cg03139388
1635	cg02829456	cg13821008	cg19107511
1636	cg02832357	cg13565718	cg06678137
1637	cg02832905	cg13525067	cg00585818
1638	cg02835462	cg13153796	cg25412310
1639	cg02836864	cg12948116	cg13171643
1640	cg02844593	cg12938917	cg15082028

1641	cg02855309	cg12865675	cg27196496
1642	cg02855558	cg12416830	cg02084948
1643	cg02857760	cg12267069	cg20505457
1644	cg02862467	cg12265604	cg07471052
1645	cg02867857	cg11589139	cg14082061
1646	cg02869364	cg10982692	cg12667792
1647	cg02872491	cg10583144	cg09326362
1648	cg02873783	cg09890597	cg03837909
1649	cg02875487	cg09548893	cg19325477
1650	cg02878891	cg08573299	cg16908123
1651	cg02886591	cg08527124	cg21759907
1652	cg02898721	cg08418375	cg06537652
1653	cg02901002	cg08351131	cg05651243
1654	cg02901644	cg08317243	cg01343045
1655	cg02907662	cg08205361	cg13948956
1656	cg02910959	cg07894983	cg16092370
1657	cg02914790	cg07691004	cg00869941
1658	cg02920396	cg07433905	cg21544633
1659	cg02926160	cg06784602	cg24550880
1660	cg02926266	cg06387842	cg02452418
1661	cg02926747	cg06208270	cg12271433
1662	cg02930033	cg06056558	cg01932663
1663	cg02931058	cg05615477	cg26953293
1664	cg02935024	cg05529123	cg02597698
1665	cg02937548	cg05384450	cg02961707
1666	cg02938429	cg04213390	cg04234758
1667	cg02940164	cg04196068	cg20634514
1668	cg02940562	cg03573679	cg19950556
1669	cg02951526	cg03485217	cg14021880
1670	cg02958180	cg03431067	cg10062065
1671	cg02958947	cg03358345	cg03562531
1672	cg02959112	cg03333149	cg10354880
1673	cg02961707	cg03318904	cg17692230
1674	cg02966841	cg02102684	cg19287817
1675	cg02968890	cg01938025	cg17266282
1676	cg02970384	cg01716827	cg06551007
1677	cg02972941	cg00901982	cg07565042
1678	cg02973735	cg00750074	cg15269394
1679	cg02975060	cg00669856	cg08095452
1680	cg02976723	cg00489394	cg13431028
1681	cg02979010	cg00355286	cg09226986
1682	cg02983650	cg00123317	cg01329151
1683	cg02993070	cg27663249	cg15540341
1684	cg02998075	cg27655716	cg24553775
1685	cg02998240	cg27410136	cg04349839
1686	cg02999082	cg27301230	cg03803102
1687	cg03006477	cg26872742	cg09483967
1688	cg03010561	cg26572651	cg00118783
1689	cg03013188	cg26503877	cg03198029
1690	cg03013917	cg25995420	cg08351131
1691	cg03017264	cg25964040	cg26888672
1692	cg03023681	cg25556464	cg20515846
1693	cg03029255	cg25424742	cg23476802

1694	cg03031073	cg25061701	cg04397884
1695	cg03031182	cg24614218	cg06096901
1696	cg03031526	cg24075760	cg10021749
1697	cg03035359	cg23085846	cg15346286
1698	cg03035704	cg23049226	cg14914440
1699	cg03036214	cg22937762	cg08915024
1700	cg03036398	cg22921096	cg05584950
1701	cg03038245	cg22118416	cg03227104
1702	cg03040489	cg21546522	cg05101437
1703	cg03042971	cg21513803	cg08384155
1704	cg03047420	cg21270847	cg24469719
1705	cg03049691	cg21000919	cg12785228
1706	cg03049782	cg20937296	cg06263395
1707	cg03050096	cg20822767	cg00001687
1708	cg03050965	cg20792436	cg16786144
1709	cg03051577	cg20761844	cg12832565
1710	cg03052760	cg20141509	cg02695969
1711	cg03054026	cg20019410	cg11567885
1712	cg03058664	cg19756253	cg10472320
1713	cg03063946	cg19709083	cg02436098
1714	cg03072692	cg19025193	cg06892907
1715	cg03074188	cg18766468	cg07267984
1716	cg03077492	cg18537410	cg20300776
1717	cg03077671	cg17589079	cg07733851
1718	cg03078718	cg17173086	cg01343936
1719	cg03082830	cg16953473	cg02366931
1720	cg03089923	cg16263152	cg26847438
1721	cg03091010	cg16106427	cg12728606
1722	cg03095814	cg16075266	cg18465694
1723	cg03098159	cg15853299	cg08644340
1724	cg03098837	cg15373257	cg01303480
1725	cg03101580	cg15329179	cg24259291
1726	cg03110167	cg15162922	cg26804423
1727	cg03114711	cg15012161	cg01881549
1728	cg03119088	cg14937059	cg00062282
1729	cg03119829	cg14058027	cg05621583
1730	cg03125400	cg13701991	cg18697143
1731	cg03125765	cg13654085	cg25189085
1732	cg03129134	cg13514824	cg24495350
1733	cg03137908	cg13491731	cg13943333
1734	cg03138206	cg13089661	cg23036171
1735	cg03139388	cg12774921	cg24804060
1736	cg03140766	cg12599569	cg07127410
1737	cg03142002	cg12503110	cg06294954
1738	cg03143333	cg12486287	cg07541559
1739	cg03143347	cg11728201	cg05185926
1740	cg03148858	cg11654662	cg01063813
1741	cg03158194	cg11492886	cg16890796
1742	cg03161498	cg11482422	cg02517473
1743	cg03162779	cg11439877	cg22422264
1744	cg03163783	cg11263420	cg18539461
1745	cg03168587	cg11253592	cg19295314
1746	cg03177023	cg10532384	cg09086087

1747	cg03178838	cg10338364	cg23258611
1748	cg03181382	cg10021122	cg19791606
1749	cg03181618	cg10018519	cg21422871
1750	cg03182608	cg08970648	cg25666403
1751	cg03187301	cg08159271	cg04324821
1752	cg03197830	cg08143701	cg16309127
1753	cg03198029	cg07048592	cg26692003
1754	cg03199926	cg06919693	cg00415011
1755	cg03215152	cg06898502	cg18998365
1756	cg03215657	cg06194960	cg07818422
1757	cg03216474	cg05833851	cg06553058
1758	cg03216729	cg05823589	cg13089599
1759	cg03217729	cg04813695	cg15626112
1760	cg03227104	cg04767697	cg04402828
1761	cg03229767	cg04287574	cg06132803
1762	cg03234557	cg04216070	cg09607915
1763	cg03242880	cg04091961	cg16174644
1764	cg03244438	cg04026354	cg00054496
1765	cg03244796	cg03977174	cg20008846
1766	cg03252568	cg03738365	cg19831403
1767	cg03253309	cg03582793	cg18595065
1768	cg03260744	cg03562531	cg14326472
1769	cg03267954	cg03355204	cg13298379
1770	cg03269976	cg03101492	cg25498969
1771	cg03270929	cg03036398	cg18272538
1772	cg03278299	cg02704331	cg26960083
1773	cg03284113	cg02695969	cg02764093
1774	cg03285823	cg02574861	cg02133557
1775	cg03286742	cg02481307	cg11758841
1776	cg03286774	cg02317397	cg11344729
1777	cg03287929	cg02146941	cg02068351
1778	cg03289072	cg02113604	cg12914733
1779	cg03294704	cg01809895	cg08094614
1780	cg03296204	cg01794926	cg07284476
1781	cg03300805	cg01678309	cg03956820
1782	cg03301025	cg01004063	cg00870269
1783	cg03307425	cg00353407	cg01922697
1784	cg03315432	cg00335361	cg24197051
1785	cg03316628	cg27493345	cg08955461
1786	cg03318904	cg27483571	cg10667102
1787	cg03318937	cg27449255	cg23916878
1788	cg03326128	cg27092594	cg14467415
1789	cg03326699	cg26685539	cg01602730
1790	cg03330678	cg26502610	cg23058194
1791	cg03333116	cg26466921	cg22846809
1792	cg03333149	cg26215113	cg07581623
1793	cg03339668	cg25589001	cg25364972
1794	cg03339817	cg25504217	cg04025970
1795	cg03344672	cg25346117	cg05141289
1796	cg03345925	cg24831140	cg02707799
1797	cg03358345	cg24387101	cg08805497
1798	cg03359508	cg24104241	cg04285855
1799	cg03367519	cg24018760	cg00656264

1800	cg03370270	cg23929809	cg07306006
1801	cg03379681	cg23244877	cg21286967
1802	cg03380645	cg23235334	cg14939765
1803	cg03380744	cg23039189	cg12351906
1804	cg03381007	cg22396663	cg10055950
1805	cg03384915	cg22289815	cg02951526
1806	cg03385114	cg21483216	cg19622474
1807	cg03389789	cg21273407	cg26504421
1808	cg03393445	cg20847114	cg04188241
1809	cg03400139	cg19594745	cg16163756
1810	cg03400491	cg19495444	cg24114708
1811	cg03407184	cg19462352	cg01012242
1812	cg03417342	cg19078037	cg02619478
1813	cg03419014	cg18203044	cg21171299
1814	cg03420242	cg17798749	cg00311883
1815	cg03421440	cg17450505	cg05781582
1816	cg03421964	cg17439009	cg10091866
1817	cg03424213	cg17241937	cg19170881
1818	cg03426226	cg16846503	cg02857760
1819	cg03426615	cg16517702	cg08605965
1820	cg03431067	cg16288579	cg16212219
1821	cg03431524	cg16265932	cg02009766
1822	cg03431903	cg16116632	cg04731861
1823	cg03434029	cg15933120	cg26261270
1824	cg03437713	cg15374133	cg01534871
1825	cg03438101	cg15232718	cg23783444
1826	cg03441279	cg14914440	cg02175033
1827	cg03444965	cg14373988	cg15168816
1828	cg03445151	cg14272573	cg01052428
1829	cg03452625	cg14122652	cg19589800
1830	cg03457485	cg13573244	cg01741616
1831	cg03469682	cg13408795	cg05004818
1832	cg03482221	cg13378934	cg13530474
1833	cg03484435	cg12266049	cg23259001
1834	cg03487040	cg11609668	cg10099813
1835	cg03488422	cg11270070	cg26862316
1836	cg03495832	cg11199137	cg24928546
1837	cg03501128	cg11025750	cg10714509
1838	cg03501387	cg10864794	cg19793499
1839	cg03512577	cg10856972	cg25517015
1840	cg03514843	cg10681992	cg07071036
1841	cg03515246	cg10644206	cg25713411
1842	cg03526625	cg10533694	cg09868556
1843	cg03528037	cg10024508	cg17854297
1844	cg03528118	cg10019018	cg27127017
1845	cg03529015	cg09729166	cg06125821
1846	cg03535239	cg09029193	cg20464155
1847	cg03538436	cg08805497	cg13008252
1848	cg03538565	cg08777316	cg04860563
1849	cg03538922	cg08218971	cg03047420
1850	cg03540589	cg07990390	cg10016364
1851	cg03543120	cg07661965	cg27302301
1852	cg03543319	cg07095945	cg22252999

1853	cg03545972	cg07083272	cg19995891
1854	cg03549146	cg06921552	cg17636309
1855	cg03550233	cg06489615	cg17860133
1856	cg03555203	cg06061760	cg01971590
1857	cg03556771	cg06036236	cg10543634
1858	cg03562531	cg05692420	cg09356020
1859	cg03566001	cg05360774	cg22429169
1860	cg03571927	cg05327192	cg16340152
1861	cg03573445	cg04958227	cg22685009
1862	cg03573679	cg04884579	cg10828599
1863	cg03575974	cg04762676	cg00410534
1864	cg03576555	cg04479713	cg00607630
1865	cg03582793	cg04360519	cg12497581
1866	cg03583746	cg04305601	cg02399019
1867	cg03589898	cg03829194	cg27250037
1868	cg03591954	cg03613003	cg15036118
1869	cg03596635	cg03543319	cg10976861
1870	cg03600605	cg03535239	cg26268125
1871	cg03604011	cg03431903	cg21474062
1872	cg03606646	cg03253309	cg02237875
1873	cg03608003	cg03049782	cg16009311
1874	cg03608520	cg03013917	cg14692453
1875	cg03613003	cg02486904	cg27351239
1876	cg03615933	cg02069095	cg27641240
1877	cg03618741	cg01837846	cg10864794
1878	cg03621841	cg01778994	cg07891483
1879	cg03627931	cg01359676	cg01017395
1880	cg03629926	cg01272202	cg25888386
1881	cg03630710	cg00260235	cg18804667
1882	cg03631837	cg00144414	cg18758281
1883	cg03639152	cg00028338	cg01260820
1884	cg03639170	cg00008452	cg00567872
1885	cg03639964	cg27650212	cg10034572
1886	cg03640215	cg27375286	cg18628371
1887	cg03644585	cg27287438	cg26204042
1888	cg03648611	cg27100436	cg24748868
1889	cg03649353	cg27040468	cg01388243
1890	cg03655701	cg26540302	cg03575974
1891	cg03657791	cg26366087	cg01664382
1892	cg03660377	cg26245697	cg15489196
1893	cg03661299	cg25701364	cg10215501
1894	cg03661844	cg25162533	cg13521018
1895	cg03666300	cg25134701	cg06616905
1896	cg03666500	cg24989405	cg00180097
1897	cg03667405	cg24969902	cg14118946
1898	cg03668470	cg24386135	cg01501896
1899	cg03669590	cg24097241	cg21962025
1900	cg03674127	cg23956760	cg04815334
1901	cg03680680	cg23946462	cg22058664
1902	cg03683994	cg23910098	cg03339668
1903	cg03684893	cg23902076	cg14290705
1904	cg03695693	cg23350558	cg04890137
1905	cg03696370	cg23021584	cg20383521

1906	cg03701759	cg22846611	cg15941159
1907	cg03726437	cg22521416	cg05529922
1908	cg03728898	cg22472360	cg09596336
1909	cg03730709	cg21770322	cg20637405
1910	cg03731348	cg21593628	cg16704889
1911	cg03736774	cg21408581	cg14168733
1912	cg03736944	cg21211480	cg01014262
1913	cg03737788	cg20679271	cg19357094
1914	cg03738365	cg19611817	cg02112621
1915	cg03761162	cg19277672	cg21756355
1916	cg03762393	cg19058262	cg22699768
1917	cg03763874	cg18729086	cg14577373
1918	cg03764767	cg18610958	cg06351520
1919	cg03765423	cg18444673	cg15770585
1920	cg03769734	cg17755483	cg07552868
1921	cg03773809	cg17721879	cg11150559
1922	cg03778594	cg17587997	cg06597413
1923	cg03779937	cg17021949	cg27010076
1924	cg03782662	cg16910448	cg05162523
1925	cg03785456	cg16421653	cg08692006
1926	cg03788408	cg16172837	cg27044591
1927	cg03789088	cg16030878	cg14217074
1928	cg03789507	cg15755154	cg03550233
1929	cg03793055	cg15637234	cg15302379
1930	cg03796321	cg15401523	cg11646638
1931	cg03800922	cg14645880	cg15368455
1932	cg03803102	cg14231959	cg07206676
1933	cg03808580	cg13681684	cg00701514
1934	cg03815917	cg13453203	cg20751706
1935	cg03817675	cg12878382	cg06933824
1936	cg03818193	cg12872329	cg16425726
1937	cg03824114	cg12866859	cg21145938
1938	cg03829194	cg12403778	cg07959741
1939	cg03834055	cg12371563	cg07054804
1940	cg03835755	cg11592677	cg19668990
1941	cg03837909	cg11400707	cg10705306
1942	cg03841376	cg10917602	cg04874782
1943	cg03851401	cg10632728	cg19445044
1944	cg03861097	cg10520924	cg01860459
1945	cg03872934	cg10330187	cg13054419
1946	cg03879386	cg10247864	cg00701856
1947	cg03881382	cg10233454	cg17789138
1948	cg03884079	cg09921821	cg20732160
1949	cg03884082	cg09781414	cg00981877
1950	cg03889044	cg09657114	cg03779937
1951	cg03899510	cg09627339	cg03669590
1952	cg03899978	cg09278623	cg01364674
1953	cg03918756	cg08932665	cg08920210
1954	cg03919694	cg07990658	cg18919478
1955	cg03933756	cg07937803	cg24247370
1956	cg03934478	cg07907506	cg04920385
1957	cg03938353	cg07824182	cg00951857
1958	cg03950492	cg07763497	cg13297120

1959	cg03954280	cg07102406	cg17818471
1960	cg03956820	cg06814256	cg09597070
1961	cg03958928	cg06212213	cg05185038
1962	cg03961998	cg06087826	cg27409974
1963	cg03962809	cg05899471	cg07508304
1964	cg03965207	cg05824174	cg07805999
1965	cg03970032	cg05816786	cg00784105
1966	cg03975834	cg05698228	cg04995521
1967	cg03977174	cg05678758	cg14817655
1968	cg03980268	cg05516390	cg03072692
1969	cg03980715	cg05403316	cg00048381
1970	cg03985415	cg05308970	cg14055379
1971	cg03988119	cg05168015	cg27073311
1972	cg03988952	cg05150641	cg20438460
1973	cg03991106	cg04920917	cg14175330
1974	cg03999452	cg04658841	cg16463044
1975	cg04002063	cg04601228	cg14444376
1976	cg04002957	cg04425005	cg26381919
1977	cg04003615	cg04077677	cg05308970
1978	cg04007841	cg03991106	cg12486498
1979	cg04009429	cg03391019	cg26711406
1980	cg04011474	cg03098837	cg20800216
1981	cg04023831	cg02495395	cg14281591
1982	cg04025970	cg02468821	cg02654449
1983	cg04026354	cg02447268	cg04791718
1984	cg04030444	cg02157002	cg11016151
1985	cg04034685	cg02043994	cg23039189
1986	cg04041283	cg01993468	cg19839325
1987	cg04046364	cg01972651	cg16023122
1988	cg04055053	cg01856887	cg10857774
1989	cg04058399	cg01294717	cg07888912
1990	cg04074404	cg01147665	cg22387323
1991	cg04077677	cg01065180	cg21897315
1992	cg04078658	cg00753492	cg19709083
1993	cg04080724	cg00554604	cg25765315
1994	cg04086327	cg00266968	cg12776287
1995	cg04088152	cg27406975	cg20852364
1996	cg04091961	cg27039312	cg21793437
1997	cg04095069	cg26939375	cg18560638
1998	cg04099803	cg26893743	cg08625996
1999	cg04100337	cg26867987	cg01406317
2000	cg04100595	cg26763380	cg14699728
2001	cg04107939	cg26268125	cg22305797
2002	cg04110886	cg26262482	cg01794929
2003	cg04113348	cg26187194	cg11915444
2004	cg04124361	cg26160492	cg26475911
2005	cg04125460	cg26143540	cg13700912
2006	cg04129282	cg25852019	cg23493018
2007	cg04133502	cg25617157	cg05564438
2008	cg04135242	cg25606046	cg13428516
2009	cg04140297	cg25573640	cg12117658
2010	cg04140971	cg25413575	cg05916989
2011	cg04143348	cg25372085	cg19631779

2012	cg04145522	cg25247887	cg19509663
2013	cg04145890	cg25197500	cg05637092
2014	cg04147497	cg25153629	cg05133706
2015	cg04147875	cg25135706	cg17854510
2016	cg04155280	cg25084760	cg01156295
2017	cg04156383	cg24875415	cg22845855
2018	cg04156643	cg24767368	cg20778199
2019	cg04157865	cg24617568	cg16316042
2020	cg04158792	cg24482246	cg00409245
2021	cg04175417	cg24288527	cg06218688
2022	cg04183638	cg23974819	cg01613294
2023	cg04186815	cg23939875	cg03668470
2024	cg04187433	cg23522522	cg01306824
2025	cg04194173	cg23475725	cg00027650
2026	cg04195226	cg23398508	cg03743074
2027	cg04198125	cg23366832	cg05750926
2028	cg04208466	cg23313005	cg26964426
2029	cg04210082	cg23250795	cg16711011
2030	cg04214430	cg23196346	cg21770322
2031	cg04216070	cg23028631	cg20741105
2032	cg04217556	cg22989033	cg26178184
2033	cg04220455	cg22638977	cg11714334
2034	cg04226256	cg22628240	cg26152017
2035	cg04226952	cg22550309	cg03339817
2036	cg04232282	cg22329743	cg07571745
2037	cg04234540	cg22095128	cg12121782
2038	cg04234758	cg21903817	cg00827581
2039	cg04236512	cg21847393	cg13162581
2040	cg04240456	cg21726593	cg00285343
2041	cg04248332	cg21650193	cg25946646
2042	cg04248364	cg21251203	cg09076584
2043	cg04249605	cg21242663	cg15354065
2044	cg04249706	cg20731213	cg04403423
2045	cg04251828	cg20140662	cg25617157
2046	cg04260676	cg20049727	cg00175487
2047	cg04261952	cg19988492	cg17966344
2048	cg04262465	cg19513582	cg17611093
2049	cg04269098	cg19482885	cg19713196
2050	cg04269351	cg19478343	cg25275372
2051	cg04282530	cg18526008	cg01553584
2052	cg04285855	cg18273464	cg02993070
2053	cg04287574	cg18051353	cg18325866
2054	cg04296187	cg18045658	cg01185921
2055	cg04304033	cg17783359	cg27241873
2056	cg04305601	cg17672035	cg16474791
2057	cg04307274	cg17400235	cg13282594
2058	cg04312967	cg17193551	cg17742459
2059	cg04315086	cg17101681	cg26934993
2060	cg04323313	cg17046776	cg06376426
2061	cg04324821	cg16734451	cg03965207
2062	cg04328729	cg16625770	cg19089328
2063	cg04334792	cg16392860	cg25350986
2064	cg04348080	cg16318412	cg10369955

2065	cg04349985	cg16312872	cg09494609
2066	cg04352404	cg16277922	cg18870258
2067	cg04352763	cg16159491	cg19702397
2068	cg04354126	cg16081499	cg18369325
2069	cg04356968	cg16023107	cg25019378
2070	cg04360147	cg14817655	cg25567021
2071	cg04360519	cg14778721	cg22877380
2072	cg04366815	cg14741474	cg04601228
2073	cg04372182	cg14711433	cg14947466
2074	cg04375492	cg14597504	cg20231900
2075	cg04381888	cg14326472	cg06194960
2076	cg04384031	cg14326263	cg15128510
2077	cg04386759	cg14242936	cg01122461
2078	cg04390667	cg14136214	cg06889272
2079	cg04392554	cg14125542	cg01759870
2080	cg04394390	cg13775299	cg14626525
2081	cg04397652	cg13728069	cg24960799
2082	cg04397884	cg13171643	cg08331427
2083	cg04398861	cg13149996	cg03543120
2084	cg04399647	cg13089599	cg00715343
2085	cg04402828	cg12308279	cg11700959
2086	cg04402948	cg12251803	cg01656717
2087	cg04403415	cg12104982	cg08606951
2088	cg04403423	cg11835806	cg00520380
2089	cg04410777	cg11438039	cg07802350
2090	cg04411052	cg11400143	cg01930756
2091	cg04415736	cg11275487	cg10664272
2092	cg04425005	cg10963218	cg02876276
2093	cg04437605	cg10617494	cg05253759
2094	cg04438661	cg10543634	cg17179557
2095	cg04454664	cg10514113	cg19063061
2096	cg04456245	cg10017293	cg15627593
2097	cg04458219	cg09858160	cg05865493
2098	cg04460041	cg09700054	cg04697056
2099	cg04462561	cg09674368	cg05670093
2100	cg04462774	cg09358422	cg03042971
2101	cg04465834	cg09333143	cg07217341
2102	cg04473654	cg08705647	cg13736131
2103	cg04476286	cg08458912	cg17972213
2104	cg04482110	cg08251036	cg01870976
2105	cg04493177	cg08173959	cg02959112
2106	cg04500730	cg07832950	cg06094523
2107	cg04505809	cg07574267	cg01481251
2108	cg04508340	cg06927337	cg06142351
2109	cg04510807	cg06880930	cg25642974
2110	cg04512603	cg06628679	cg05715751
2111	cg04517274	cg06580014	cg08506026
2112	cg04524477	cg06477663	cg08611376
2113	cg04529785	cg06259664	cg20909380
2114	cg04530852	cg06172939	cg26620147
2115	cg04534503	cg06017355	cg17695841
2116	cg04535391	cg05887890	cg01938025
2117	cg04536844	cg05836492	cg12384918

2118	cg04541146	cg05736906	cg18475969
2119	cg04545872	cg05267394	cg00594917
2120	cg04546061	cg05080811	cg06943845
2121	cg04547809	cg04899175	cg19495444
2122	cg04550439	cg04650653	cg23249399
2123	cg04555379	cg04622001	cg19821361
2124	cg04558973	cg04608722	cg02363010
2125	cg04568774	cg04410777	cg09386073
2126	cg04573078	cg04372182	cg12468255
2127	cg04576568	cg04183638	cg26723735
2128	cg04585669	cg04125460	cg00589581
2129	cg04591732	cg04060308	cg02569718
2130	cg04600641	cg04002063	cg02628360
2131	cg04601228	cg03884079	cg03119829
2132	cg04604708	cg03835755	cg00058291
2133	cg04607323	cg03788408	cg16422492
2134	cg04607844	cg03469682	cg09101894
2135	cg04608722	cg03439687	cg10644206
2136	cg04618903	cg03367519	cg12019806
2137	cg04623131	cg03287929	cg27143703
2138	cg04627183	cg03229767	cg04681879
2139	cg04628014	cg03182608	cg09309208
2140	cg04633661	cg03177023	cg04576568
2141	cg04654299	cg03004417	cg14596589
2142	cg04655520	cg02635482	cg19502812
2143	cg04658707	cg02481714	cg20675852
2144	cg04658841	cg02421824	cg10691866
2145	cg04663956	cg02304580	cg19084508
2146	cg04668631	cg02304222	cg08814268
2147	cg04674497	cg02097309	cg23693485
2148	cg04677227	cg02061967	cg17876645
2149	cg04678901	cg02009766	cg06275130
2150	cg04679114	cg01825806	cg11654662
2151	cg04681879	cg01824284	cg00187535
2152	cg04684246	cg01537847	cg04948014
2153	cg04687939	cg01454538	cg14016236
2154	cg04689023	cg01441113	cg24600706
2155	cg04696969	cg01120132	cg09368832
2156	cg04697056	cg00903308	cg11936643
2157	cg04699214	cg00688539	cg16489468
2158	cg04703221	cg00374717	cg13858803
2159	cg04703974	cg00371570	cg01104466
2160	cg04706201	cg00245896	cg23850277
2161	cg04707715	cg00236831	cg18238763
2162	cg04708036	cg00085256	cg25283432
2163	cg04717045	cg00042325	cg26143540
2164	cg04719721	cg27579131	cg08317243
2165	cg04724190	cg27549186	cg08740088
2166	cg04724387	cg27498434	cg07015803
2167	cg04727924	cg27454102	cg24052817
2168	cg04729730	cg27144884	cg25595431
2169	cg04731861	cg26868306	cg11840849
2170	cg04743876	cg26745294	cg24897108

2171	cg04744514	cg26446949	cg14037652
2172	cg04745820	cg26338030	cg06573945
2173	cg04751761	cg25607321	cg16885557
2174	cg04767697	cg25588826	cg02816367
2175	cg04774476	cg25502179	cg12747076
2176	cg04774496	cg25476129	cg21837323
2177	cg04777612	cg25283432	cg18720622
2178	cg04780481	cg24706981	cg01306747
2179	cg04784212	cg24673886	cg17291826
2180	cg04787317	cg24135645	cg03091010
2181	cg04791718	cg24035682	cg23623681
2182	cg04797170	cg23973885	cg02131465
2183	cg04800347	cg23925540	cg04550439
2184	cg04803994	cg22846809	cg05360774
2185	cg04804094	cg22584802	cg27320213
2186	cg04804648	cg22439641	cg09835239
2187	cg04805065	cg21171279	cg11190890
2188	cg04806664	cg20690695	cg08535779
2189	cg04813695	cg20679011	cg13761321
2190	cg04815334	cg20516209	cg10278394
2191	cg04815973	cg20333027	cg14476630
2192	cg04819180	cg19802929	cg02157052
2193	cg04820362	cg19791606	cg07236812
2194	cg04837783	cg19548479	cg18881778
2195	cg04839616	cg19201770	cg09812790
2196	cg04843690	cg18801945	cg11272874
2197	cg04843968	cg18729681	cg19494591
2198	cg04844977	cg18369990	cg25137314
2199	cg04846781	cg18153137	cg09233395
2200	cg04860563	cg18067847	cg02966841
2201	cg04868238	cg18032164	cg05161923
2202	cg04872027	cg18013789	cg09675604
2203	cg04873169	cg17680611	cg16236270
2204	cg04874782	cg17475304	cg27618145
2205	cg04880940	cg17442932	cg04505897
2206	cg04882759	cg17067528	cg26137103
2207	cg04884579	cg16998564	cg09612502
2208	cg04885333	cg16668903	cg16283362
2209	cg04890137	cg16452866	cg03050965
2210	cg04899175	cg16452651	cg16499415
2211	cg04902542	cg16285424	cg08364860
2212	cg04904609	cg16092370	cg05899471
2213	cg04910179	cg16078863	cg23812660
2214	cg04911180	cg15969277	cg24844449
2215	cg04911307	cg15874642	cg07547336
2216	cg04911680	cg15813460	cg27441486
2217	cg04914198	cg15582707	cg04402948
2218	cg04920385	cg15411034	cg02556345
2219	cg04920917	cg15368455	cg08458637
2220	cg04921335	cg15326069	cg19162768
2221	cg04924028	cg15117891	cg11080651
2222	cg04926380	cg14679558	cg23525541
2223	cg04929165	cg14588828	cg02844593

2224	cg04930596	cg14443041	cg08038054
2225	cg04944177	cg14438213	cg20018563
2226	cg04944536	cg14412134	cg18817831
2227	cg04945557	cg14228788	cg17319795
2228	cg04948014	cg13877974	cg22052672
2229	cg04955856	cg13708635	cg07818978
2230	cg04956511	cg13618969	cg00412772
2231	cg04958227	cg13385220	cg26939375
2232	cg04961225	cg13264394	cg02325128
2233	cg04963697	cg13148544	cg06919693
2234	cg04964845	cg13105904	cg16509173
2235	cg04966586	cg13058457	cg03543319
2236	cg04967009	cg12486498	cg25862117
2237	cg04967410	cg12293340	cg11585280
2238	cg04968127	cg12075928	cg14288326
2239	cg04972957	cg11457582	cg25652751
2240	cg04982190	cg11035122	cg05099063
2241	cg04983933	cg10904856	cg03380744
2242	cg04986373	cg10737521	cg23457862
2243	cg04990420	cg10705306	cg01946510
2244	cg04993279	cg10590657	cg20382418
2245	cg04995521	cg10558233	cg09859805
2246	cg04997235	cg10356060	cg10404601
2247	cg05002642	cg10338518	cg25386426
2248	cg05004818	cg10311754	cg04236512
2249	cg05013912	cg09950920	cg23301563
2250	cg05016508	cg09828346	cg03918756
2251	cg05017639	cg09712234	cg25383568
2252	cg05024916	cg09489567	cg08112616
2253	cg05030450	cg09464883	cg00999267
2254	cg05032848	cg09141835	cg19897003
2255	cg05037360	cg08993878	cg19991046
2256	cg05037556	cg08823975	cg27177779
2257	cg05040210	cg08812108	cg26828494
2258	cg05041061	cg08712631	cg08628233
2259	cg05041265	cg08703151	cg13737493
2260	cg05041871	cg08642081	cg13543915
2261	cg05051393	cg08276889	cg10827460
2262	cg05051734	cg08206881	cg14698794
2263	cg05054460	cg08151983	cg00074348
2264	cg05056653	cg07976390	cg02066343
2265	cg05057834	cg07880135	cg03573445
2266	cg05058103	cg07876450	cg20805475
2267	cg05060427	cg07410188	cg07346310
2268	cg05060686	cg07036799	cg14630692
2269	cg05065603	cg07012739	cg01158079
2270	cg05069728	cg06981182	cg25339612
2271	cg05070608	cg06868569	cg07050611
2272	cg05073386	cg06772578	cg02940562
2273	cg05076082	cg06705004	cg10888242
2274	cg05080811	cg06259557	cg15424477
2275	cg05088513	cg05917480	cg15546071
2276	cg05088605	cg05816006	cg05452742

2277	cg05088794	cg05664352	cg09358481
2278	cg05099063	cg05181397	cg16582732
2279	cg05099221	cg04982190	cg21619325
2280	cg05101437	cg04968127	cg22987116
2281	cg05109049	cg04791718	cg19048950
2282	cg05112986	cg04703974	cg09322899
2283	cg05133706	cg04582672	cg14126884
2284	cg05134769	cg04175292	cg25479732
2285	cg05141289	cg04133502	cg14360029
2286	cg05145297	cg03695693	cg18765405
2287	cg05150667	cg03657791	cg13818316
2288	cg05150697	cg03178838	cg04708036
2289	cg05159066	cg03044471	cg03640756
2290	cg05161923	cg02951526	cg07990390
2291	cg05162523	cg02931058	cg15968604
2292	cg05168015	cg02838178	cg21806750
2293	cg05179396	cg02366931	cg11844464
2294	cg05180954	cg02066441	cg16258545
2295	cg05185019	cg02011409	cg24394336
2296	cg05185038	cg02003612	cg26421308
2297	cg05185926	cg01971590	cg07310500
2298	cg05188868	cg01606770	cg01303236
2299	cg05190559	cg01486610	cg12179658
2300	cg05194102	cg01398428	cg05797224
2301	cg05196820	cg01293277	cg10296062
2302	cg05201300	cg00961932	cg05999324
2303	cg05202347	cg00707452	cg25476129
2304	cg05202424	cg00686915	cg24097241
2305	cg05204104	cg00677217	cg21332304
2306	cg05210671	cg00624949	cg04095069
2307	cg05218696	cg00592695	cg11284582
2308	cg05220083	cg00158530	cg16337430
2309	cg05223346	cg00061185	cg02317251
2310	cg05224498	cg00038342	cg13455439
2311	cg05229528	cg27585557	cg06927337
2312	cg05235248	cg27248741	cg08886695
2313	cg05242371	cg27177415	cg21155834
2314	cg05249271	cg27100471	cg03445151
2315	cg05251593	cg27096981	cg04234540
2316	cg05253759	cg27092248	cg19201144
2317	cg05258102	cg26672794	cg24479752
2318	cg05260236	cg26607528	cg10370305
2319	cg05267269	cg26341003	cg20000602
2320	cg05267394	cg26284638	cg14921326
2321	cg05270552	cg26068595	cg06649282
2322	cg05272587	cg26014373	cg03879386
2323	cg05272807	cg26007445	cg25488990
2324	cg05279172	cg25947619	cg05529123
2325	cg05279866	cg25714956	cg18440069
2326	cg05280797	cg25666403	cg02384859
2327	cg05283542	cg25625457	cg10921517
2328	cg05286252	cg25604994	cg06989443
2329	cg05288253	cg25352924	cg06328338

2330	cg05289897	cg25321935	cg15944060
2331	cg05291588	cg25221919	cg24783499
2332	cg05294455	cg24752354	cg03355204
2333	cg05299486	cg24502047	cg27186013
2334	cg05303899	cg24448421	cg01176141
2335	cg05307141	cg24425531	cg10338364
2336	cg05308970	cg24247370	cg11958668
2337	cg05309505	cg24197051	cg26420566
2338	cg05309989	cg24052817	cg20323175
2339	cg05317605	cg23916878	cg13542073
2340	cg05322217	cg23760873	cg11319760
2341	cg05324992	cg23415916	cg16142965
2342	cg05334190	cg23260554	cg07336123
2343	cg05336268	cg23251296	cg16828491
2344	cg05337637	cg22805632	cg02008511
2345	cg05339066	cg22750845	cg05468559
2346	cg05340240	cg22720481	cg21528927
2347	cg05340658	cg22371518	cg08097359
2348	cg05341539	cg22272218	cg24003508
2349	cg05346688	cg21889263	cg07044523
2350	cg05349039	cg21511816	cg11844358
2351	cg05350020	cg21434355	cg11802013
2352	cg05360477	cg21352006	cg02331025
2353	cg05360774	cg21331845	cg02725453
2354	cg05361262	cg21118367	cg22593342
2355	cg05364570	cg20971998	cg07582655
2356	cg05368595	cg20927312	cg26315985
2357	cg05368971	cg20767977	cg14437446
2358	cg05369142	cg20571592	cg13852730
2359	cg05370752	cg20341998	cg20027456
2360	cg05376738	cg20210151	cg22257056
2361	cg05380734	cg20078454	cg27478167
2362	cg05384450	cg19770281	cg14565781
2363	cg05386977	cg19698242	cg01197763
2364	cg05388880	cg19623406	cg06900571
2365	cg05390712	cg19584674	cg00663077
2366	cg05397809	cg19561607	cg05035470
2367	cg05400196	cg19514469	cg17030415
2368	cg05400252	cg19018155	cg17511604
2369	cg05403316	cg18869485	cg24191821
2370	cg05404117	cg18761894	cg07266910
2371	cg05404787	cg18758976	cg21863721
2372	cg05409945	cg18378662	cg01730064
2373	cg05415936	cg18325866	cg11547122
2374	cg05418508	cg18241094	cg20547131
2375	cg05418719	cg18225577	cg00920668
2376	cg05426006	cg17781958	cg20471017
2377	cg05434397	cg17636986	cg06109482
2378	cg05450563	cg17473727	cg09825670
2379	cg05452391	cg17250537	cg25236277
2380	cg05452742	cg17094249	cg04210082
2381	cg05459650	cg16694664	cg11998932
2382	cg05469118	cg16653700	cg19842026

2383	cg05477920	cg16649474	cg10492836
2384	cg05479174	cg16626731	cg24183575
2385	cg05482864	cg16349616	cg19646759
2386	cg05482973	cg16142965	cg07290269
2387	cg05483875	cg16126178	cg10149870
2388	cg05484458	cg16067210	cg14393609
2389	cg05494073	cg15637254	cg11496593
2390	cg05498905	cg15425427	cg18148488
2391	cg05503991	cg15111351	cg13500797
2392	cg05504354	cg15081886	cg02013018
2393	cg05506498	cg15033511	cg05776138
2394	cg05508315	cg14842237	cg06232205
2395	cg05515072	cg14839830	cg01523759
2396	cg05516390	cg14819399	cg11538848
2397	cg05517824	cg14641266	cg17056676
2398	cg05528280	cg14560240	cg05931119
2399	cg05529123	cg14550212	cg07344990
2400	cg05529922	cg14274896	cg01705036
2401	cg05535123	cg14264773	cg02829279
2402	cg05536457	cg14234732	cg07133097
2403	cg05539509	cg13947999	cg13982956
2404	cg05544807	cg13857210	cg03788408
2405	cg05552543	cg13737493	cg00927435
2406	cg05554592	cg13637654	cg02202664
2407	cg05555455	cg13572069	cg05544807
2408	cg05559648	cg13367537	cg17442683
2409	cg05561386	cg12914733	cg07487572
2410	cg05564438	cg12776287	cg23401796
2411	cg05579549	cg12732734	cg18279126
2412	cg05584950	cg12598178	cg22076676
2413	cg05585379	cg12526346	cg18397743
2414	cg05588341	cg12352896	cg06539449
2415	cg05590156	cg12195230	cg01518755
2416	cg05590948	cg12005412	cg27288127
2417	cg05591270	cg11936643	cg14312334
2418	cg05591701	cg11854392	cg17958516
2419	cg05595142	cg11743827	cg01810186
2420	cg05595469	cg11562411	cg07374490
2421	cg05603553	cg11321921	cg02958947
2422	cg05615230	cg11116288	cg06796435
2423	cg05615477	cg10799492	cg05862007
2424	cg05621583	cg10507988	cg12245876
2425	cg05623411	cg10461264	cg20494661
2426	cg05634149	cg10319905	cg00604356
2427	cg05637092	cg10225755	cg20210637
2428	cg05650238	cg09920043	cg06363798
2429	cg05651243	cg09597710	cg18526008
2430	cg05657292	cg09586198	cg08812108
2431	cg05658807	cg09553581	cg03469682
2432	cg05662839	cg09465065	cg00185413
2433	cg05670093	cg09373983	cg00656603
2434	cg05678758	cg09358481	cg23363971
2435	cg05680710	cg09119854	cg17821664

2436	cg05682319	cg08972190	cg02643834
2437	cg05692420	cg08824291	cg20334115
2438	cg05698228	cg08692006	cg16081499
2439	cg05703996	cg08677210	cg21811911
2440	cg05705335	cg08661751	cg23366832
2441	cg05711037	cg08613794	cg00131423
2442	cg05712931	cg08447373	cg04002063
2443	cg05714748	cg08378932	cg14890730
2444	cg05715422	cg08369014	cg09251291
2445	cg05715751	cg08132025	cg20662658
2446	cg05724271	cg08110337	cg10004897
2447	cg05727180	cg08043592	cg25588826
2448	cg05730150	cg07992340	cg11197908
2449	cg05736906	cg07965110	cg10147974
2450	cg05738470	cg07875385	cg21105227
2451	cg05738687	cg07802350	cg16030878
2452	cg05757474	cg07795968	cg15854022
2453	cg05757530	cg07447742	cg24037270
2454	cg05767015	cg07409629	cg02393496
2455	cg05767299	cg07398661	cg26955275
2456	cg05770240	cg07116732	cg06371044
2457	cg05771342	cg07109453	cg04784315
2458	cg05776138	cg07094993	cg14114546
2459	cg05777316	cg06864351	cg12579684
2460	cg05781582	cg06857516	cg09076960
2461	cg05798126	cg06758191	cg04882759
2462	cg05798436	cg06611426	cg11718965
2463	cg05801879	cg06579465	cg23388085
2464	cg05816006	cg06525127	cg23953820
2465	cg05816041	cg06422467	cg02119693
2466	cg05817833	cg06376598	cg09913449
2467	cg05821186	cg06204229	cg12442385
2468	cg05823589	cg06200092	cg13793145
2469	cg05824174	cg06109482	cg12678686
2470	cg05824594	cg05865493	cg12927715
2471	cg05825120	cg05380734	cg16572603
2472	cg05826596	cg05370752	cg24075760
2473	cg05832823	cg05168368	cg17113147
2474	cg05833851	cg04964845	cg02323633
2475	cg05836492	cg04910179	cg05397809
2476	cg05845879	cg04880940	cg24083817
2477	cg05846044	cg04847478	cg26740525
2478	cg05846633	cg04431002	cg03370270
2479	cg05851042	cg04234540	cg03426226
2480	cg05851887	cg04208466	cg21647035
2481	cg05854826	cg03934478	cg21494132
2482	cg05856230	cg03884082	cg00852414
2483	cg05858079	cg03785456	cg04819180
2484	cg05862007	cg03722184	cg02920396
2485	cg05865493	cg03683994	cg11035122
2486	cg05865548	cg03421964	cg23280999
2487	cg05867158	cg03376089	cg25202370
2488	cg05869732	cg03119088	cg12725520

2489	cg05874233	cg02857760	cg25903122
2490	cg05876246	cg02650017	cg24590353
2491	cg05887890	cg02452418	cg08892499
2492	cg05888037	cg02332902	cg16365663
2493	cg05894015	cg02262037	cg09554406
2494	cg05898188	cg02087101	cg11346522
2495	cg05899507	cg01902704	cg15104211
2496	cg05900955	cg01634146	cg04983933
2497	cg05909553	cg01579765	cg24926121
2498	cg05909702	cg01559787	cg01592387
2499	cg05915622	cg01512113	cg27168410
2500	cg05916989	cg01277910	cg22048683
2501	cg05917480	cg01156295	cg08613144
2502	cg05918682	cg01017228	cg00710466
2503	cg05934824	cg00820405	cg11847597
2504	cg05945030	cg00169184	cg05579549
2505	cg05949181	cg00133595	cg10129154
2506	cg05951828	cg00103778	cg10809282
2507	cg05966978	cg00074348	cg00941576
2508	cg05968917	cg27514730	cg20090162
2509	cg05970317	cg27247807	cg01362762
2510	cg05971891	cg27203917	cg04086327
2511	cg05975710	cg27192012	cg22908922
2512	cg05976168	cg27133959	cg13086402
2513	cg05977276	cg27096087	cg07433453
2514	cg05977523	cg27074355	cg21032564
2515	cg05978010	cg26955275	cg08904630
2516	cg05982473	cg26884345	cg27118035
2517	cg05989746	cg26862316	cg19020855
2518	cg05995260	cg26794830	cg07333510
2519	cg05996835	cg26668872	cg19444998
2520	cg05999324	cg26653360	cg06823034
2521	cg06008803	cg26503644	cg05058103
2522	cg06012410	cg26448137	cg13657511
2523	cg06012483	cg26144207	cg07132183
2524	cg06013127	cg26030804	cg20943461
2525	cg06017355	cg26013235	cg04462561
2526	cg06018095	cg25825612	cg17534540
2527	cg06028151	cg25629077	cg12075498
2528	cg06033488	cg25350986	cg08737189
2529	cg06036236	cg25350827	cg22803510
2530	cg06038180	cg25289803	cg00857907
2531	cg06056558	cg25167447	cg26882115
2532	cg06058262	cg24930851	cg17819085
2533	cg06061092	cg24866203	cg14403241
2534	cg06068392	cg24856699	cg19340420
2535	cg06070445	cg24825262	cg15391531
2536	cg06078337	cg24824472	cg13763482
2537	cg06084403	cg24773418	cg16560256
2538	cg06085713	cg24495350	cg06200092
2539	cg06087826	cg24233679	cg17361593
2540	cg06090895	cg24030630	cg04060308
2541	cg06093152	cg23754431	cg02729303

2542	cg06094523	cg23388085	cg16759204
2543	cg06096901	cg23288298	cg13654085
2544	cg06104859	cg22765117	cg11902934
2545	cg06109284	cg22730007	cg23630423
2546	cg06109482	cg22482278	cg04761077
2547	cg06113801	cg22461758	cg00622702
2548	cg06115826	cg22348713	cg08616663
2549	cg06120235	cg22325145	cg01723892
2550	cg06123891	cg22082745	cg05876246
2551	cg06124434	cg21906519	cg05887890
2552	cg06125821	cg21863499	cg14401746
2553	cg06132803	cg21696975	cg09524658
2554	cg06142351	cg21406967	cg25834632
2555	cg06143290	cg21255128	cg15541630
2556	cg06154633	cg20892492	cg18444673
2557	cg06159340	cg20852364	cg23408104
2558	cg06167719	cg20464155	cg23237976
2559	cg06172939	cg20165037	cg00500424
2560	cg06173481	cg20095124	cg16867086
2561	cg06174078	cg19949955	cg06752365
2562	cg06179971	cg19849478	cg24775327
2563	cg06194960	cg19765175	cg11037787
2564	cg06198069	cg19565757	cg09637963
2565	cg06200092	cg19016264	cg14642045
2566	cg06200670	cg18863119	cg15374133
2567	cg06208158	cg18857588	cg26315221
2568	cg06208229	cg18795383	cg19827875
2569	cg06208270	cg18285813	cg04573078
2570	cg06212213	cg18201671	cg05261433
2571	cg06212263	cg18108009	cg09293488
2572	cg06212582	cg18083415	cg08740008
2573	cg06213876	cg17817350	cg02272751
2574	cg06219670	cg17683336	cg04399631
2575	cg06219732	cg17442683	cg21735516
2576	cg06225767	cg17367901	cg13763232
2577	cg06232205	cg17364872	cg06646728
2578	cg06233301	cg17202331	cg12609948
2579	cg06237774	cg17174566	cg09109383
2580	cg06241044	cg17156862	cg07005960
2581	cg06241812	cg16897800	cg20379239
2582	cg06246000	cg16711084	cg25761326
2583	cg06251016	cg16666964	cg10731848
2584	cg06252645	cg16640450	cg01661567
2585	cg06254425	cg16629076	cg15693937
2586	cg06255601	cg16621790	cg25484139
2587	cg06255609	cg16402415	cg24916358
2588	cg06257110	cg16238993	cg09375892
2589	cg06264060	cg16081096	cg03977174
2590	cg06267617	cg15250073	cg17901582
2591	cg06272543	cg15228983	cg18699466
2592	cg06275130	cg14983540	cg21329649
2593	cg06290096	cg14878988	cg24137511
2594	cg06292891	cg14601444	cg03345925

2595	cg06294856	cg14571537	cg08860136
2596	cg06294954	cg14389425	cg08307171
2597	cg06309980	cg14337844	cg00542261
2598	cg06313479	cg14199827	cg27548256
2599	cg06321304	cg14055379	cg17156862
2600	cg06328338	cg13910681	cg22921096
2601	cg06333135	cg13700912	cg12079279
2602	cg06338958	cg13517605	cg05309989
2603	cg06342954	cg13508949	cg22525688
2604	cg06351520	cg13414654	cg00675878
2605	cg06355908	cg13287523	cg21230793
2606	cg06363798	cg13278004	cg10190898
2607	cg06368978	cg13154622	cg10101600
2608	cg06369532	cg12549211	cg01545140
2609	cg06371044	cg12179658	cg15264708
2610	cg06376426	cg11912239	cg00400992
2611	cg06377278	cg11761615	cg20065217
2612	cg06378490	cg11703759	cg17017404
2613	cg06379876	cg11650648	cg01578875
2614	cg06385449	cg11530995	cg06864351
2615	cg06386880	cg11423998	cg03181382
2616	cg06387204	cg11311843	cg04612073
2617	cg06387842	cg11115867	cg07394799
2618	cg06389574	cg11108115	cg16609966
2619	cg06397381	cg11068930	cg03125765
2620	cg06400255	cg10898310	cg27485845
2621	cg06406157	cg10813908	cg09180239
2622	cg06407371	cg10724774	cg20773915
2623	cg06407441	cg10174683	cg00562504
2624	cg06415302	cg10082445	cg07048592
2625	cg06419732	cg09634481	cg13996750
2626	cg06422467	cg09524658	cg04842880
2627	cg06422947	cg09486967	cg11888747
2628	cg06432200	cg09375892	cg08186671
2629	cg06436663	cg09326135	cg16242615
2630	cg06439736	cg09293488	cg04787317
2631	cg06445533	cg09198138	cg24155123
2632	cg06456125	cg08986727	cg00729995
2633	cg06463230	cg08847512	cg13162609
2634	cg06474428	cg08695912	cg05934824
2635	cg06477663	cg08681293	cg11691844
2636	cg06485166	cg08628233	cg24093182
2637	cg06489615	cg08463581	cg07965566
2638	cg06492744	cg08400424	cg09631175
2639	cg06497934	cg08342966	cg15661715
2640	cg06500727	cg08193650	cg25382652
2641	cg06504636	cg08186671	cg08412215
2642	cg06506523	cg08113187	cg25430713
2643	cg06518233	cg08108655	cg23429698
2644	cg06521357	cg08095452	cg09389557
2645	cg06522179	cg07915976	cg05970317
2646	cg06524531	cg07888912	cg10107330
2647	cg06525127	cg07826642	cg00501242

2648	cg06528340	cg07811002	cg15460348
2649	cg06530983	cg07720334	cg05917419
2650	cg06532257	cg07374490	cg02043994
2651	cg06537333	cg07270259	cg00899463
2652	cg06537652	cg07187503	cg00158770
2653	cg06537744	cg07127410	cg02306481
2654	cg06539449	cg07085590	cg23990272
2655	cg06541938	cg07071036	cg06219732
2656	cg06546521	cg06947205	cg03488422
2657	cg06549228	cg06809295	cg13171107
2658	cg06551007	cg06808089	cg08098420
2659	cg06553058	cg06760467	cg07624888
2660	cg06553975	cg06646728	cg00209623
2661	cg06556244	cg06642012	cg05602356
2662	cg06561892	cg06551007	cg14758812
2663	cg06562888	cg06013127	cg10496150
2664	cg06566615	cg05995260	cg07740306
2665	cg06568783	cg05851887	cg20049727
2666	cg06572904	cg05479174	cg09425164
2667	cg06573945	cg05210671	cg15991104
2668	cg06577703	cg05159066	cg05017639
2669	cg06579465	cg05101437	cg06840243
2670	cg06580014	cg04658707	cg11390504
2671	cg06584329	cg04324821	cg22461758
2672	cg06586578	cg04315086	cg01634146
2673	cg06597413	cg04307274	cg06537333
2674	cg06599914	cg04131010	cg21552014
2675	cg06604690	cg04058399	cg19548524
2676	cg06607764	cg04030444	cg25545917
2677	cg06610254	cg03684893	cg06525127
2678	cg06611426	cg03583746	cg01792640
2679	cg06614534	cg03488422	cg14615152
2680	cg06616905	cg03330678	cg25053164
2681	cg06617528	cg03326699	cg02710481
2682	cg06621620	cg03244438	cg13817905
2683	cg06622892	cg02898721	cg00732878
2684	cg06628679	cg02340818	cg23910098
2685	cg06632214	cg02331025	cg16778107
2686	cg06632821	cg02263260	cg10457079
2687	cg06633739	cg02216206	cg17898124
2688	cg06641607	cg02107173	cg14313576
2689	cg06642012	cg01903305	cg12925881
2690	cg06646728	cg01691710	cg13475822
2691	cg06647026	cg01581360	cg01997461
2692	cg06649282	cg01546047	cg02069944
2693	cg06650364	cg01518755	cg00143606
2694	cg06656553	cg01413698	cg19604110
2695	cg06659338	cg01339351	cg26837766
2696	cg06660116	cg01314034	cg04100337
2697	cg06665485	cg01267373	cg27378814
2698	cg06671298	cg01214847	cg23115933
2699	cg06673840	cg01158079	cg25424742
2700	cg06675951	cg00981877	cg14671764

2701	cg06678137	cg00887848	cg09185884
2702	cg06689039	cg00869941	cg25346117
2703	cg06694381	cg00710466	cg14062119
2704	cg06702537	cg00699693	cg05657292
2705	cg06705004	cg00594917	cg21740826
2706	cg06706550	cg00523012	cg10904856
2707	cg06710741	cg00409245	cg12019990
2708	cg06711298	cg00352031	cg25372085
2709	cg06719042	cg00232500	cg03252568
2710	cg06723357	cg27612166	cg23464284
2711	cg06741399	cg27611699	cg00341297
2712	cg06741896	cg27578381	cg22575379
2713	cg06746318	cg27566403	cg04156383
2714	cg06746774	cg27532130	cg04328729
2715	cg06748078	cg27234648	cg06372475
2716	cg06750802	cg27207616	cg15351186
2717	cg06753050	cg27202913	cg00936935
2718	cg06753827	cg27073349	cg00356131
2719	cg06753918	cg26977086	cg00785482
2720	cg06754496	cg26818159	cg10737521
2721	cg06755413	cg26740525	cg11523661
2722	cg06758191	cg26660115	cg08115297
2723	cg06760467	cg26637901	cg18225577
2724	cg06768437	cg26120842	cg06894011
2725	cg06769510	cg26097051	cg09028383
2726	cg06776976	cg25707005	cg03730709
2727	cg06784563	cg25644380	cg23707719
2728	cg06784602	cg25271479	cg05309505
2729	cg06786153	cg25116615	cg02658272
2730	cg06787104	cg24940701	cg17046776
2731	cg06787609	cg24940138	cg05874233
2732	cg06790069	cg24897108	cg13058457
2733	cg06791695	cg24896649	cg18311516
2734	cg06795827	cg24750887	cg09890339
2735	cg06796435	cg24657347	cg20540306
2736	cg06797069	cg24630383	cg05037360
2737	cg06799533	cg24544986	cg27247807
2738	cg06807837	cg24474622	cg17470627
2739	cg06808089	cg24100115	cg01576275
2740	cg06809295	cg23850277	cg11043092
2741	cg06812836	cg23812679	cg19572849
2742	cg06812840	cg23397909	cg07531248
2743	cg06814256	cg22990430	cg23173517
2744	cg06817454	cg22885000	cg24659758
2745	cg06819431	cg22780675	cg23887746
2746	cg06823034	cg22743617	cg02855558
2747	cg06824199	cg22039204	cg13470673
2748	cg06827256	cg21500064	cg20548182
2749	cg06833798	cg21442998	cg19100996
2750	cg06836544	cg21235119	cg07398661
2751	cg06840243	cg20800216	cg09586198
2752	cg06846976	cg20784591	cg19317433
2753	cg06849167	cg20459543	cg13808641

2754	cg06851844	cg20316440	cg20069939
2755	cg06853492	cg20226512	cg27514730
2756	cg06856329	cg20166714	cg17470198
2757	cg06856570	cg20018563	cg02627286
2758	cg06857516	cg19974227	cg23691781
2759	cg06861572	cg19892287	cg17285883
2760	cg06862949	cg19874640	cg13385220
2761	cg06863830	cg19603100	cg04473654
2762	cg06864351	cg19303748	cg09932436
2763	cg06868569	cg18696900	cg05229528
2764	cg06869755	cg18436810	cg21370924
2765	cg06870740	cg18384190	cg11777290
2766	cg06880930	cg18304186	cg12614090
2767	cg06881421	cg18149689	cg17450505
2768	cg06889272	cg18120975	cg21089789
2769	cg06892815	cg18108716	cg08701675
2770	cg06892907	cg17796593	cg05536457
2771	cg06894011	cg17791936	cg02928110
2772	cg06895946	cg17550566	cg00489394
2773	cg06898502	cg17537493	cg14044465
2774	cg06899970	cg17431079	cg07067993
2775	cg06900571	cg17307791	cg11176525
2776	cg06900843	cg17304712	cg16263152
2777	cg06907347	cg16995299	cg20459543
2778	cg06914693	cg16711011	cg10925829
2779	cg06916574	cg16704889	cg11482422
2780	cg06918474	cg16660312	cg12552626
2781	cg06919312	cg16572224	cg09259595
2782	cg06919693	cg16514214	cg01770296
2783	cg06921552	cg16407998	cg21903817
2784	cg06924233	cg16265707	cg23460369
2785	cg06926306	cg16114651	cg11977716
2786	cg06927337	cg16031980	cg18854004
2787	cg06931444	cg16027775	cg15464763
2788	cg06937201	cg15770585	cg05832823
2789	cg06937205	cg15576082	cg09175915
2790	cg06937357	cg15402232	cg23610994
2791	cg06938890	cg15354065	cg10126181
2792	cg06943385	cg15121417	cg05698228
2793	cg06943835	cg15028232	cg26965897
2794	cg06947205	cg14916108	cg02078525
2795	cg06967316	cg14870682	cg18048309
2796	cg06967828	cg14657458	cg06642012
2797	cg06969777	cg14524553	cg16516980
2798	cg06981182	cg14393609	cg09320595
2799	cg06984255	cg13927454	cg17329004
2800	cg06987369	cg13642149	cg24875593
2801	cg06989253	cg13618372	cg09006487
2802	cg06989443	cg13522118	cg08524717
2803	cg06995299	cg13483248	cg27346937
2804	cg06996029	cg13422164	cg04249605
2805	cg06996896	cg13216372	cg26574247
2806	cg07005960	cg13207250	cg15264991

2807	cg07008193	cg13071729	cg09520904
2808	cg07012484	cg12692386	cg24738592
2809	cg07012725	cg12616177	cg25520910
2810	cg07014438	cg12367789	cg16823064
2811	cg07015803	cg12109728	cg00469547
2812	cg07016298	cg12019990	cg23834919
2813	cg07020987	cg11998932	cg11535638
2814	cg07021447	cg11864076	cg25714956
2815	cg07022478	cg11822372	cg02613295
2816	cg07025242	cg11818853	cg14125542
2817	cg07026910	cg10876003	cg21546950
2818	cg07034004	cg10859358	cg20822767
2819	cg07036799	cg10759972	cg19584674
2820	cg07038822	cg10434728	cg27302539
2821	cg07041214	cg10404601	cg16034562
2822	cg07041720	cg10095226	cg22286382
2823	cg07041883	cg09897737	cg23393892
2824	cg07043952	cg09702334	cg27635271
2825	cg07044414	cg09647352	cg07214314
2826	cg07044523	cg09256716	cg17049328
2827	cg07046047	cg09238162	cg12786452
2828	cg07048516	cg09175915	cg06723357
2829	cg07048592	cg09171093	cg02742533
2830	cg07050599	cg09036558	cg24215159
2831	cg07050611	cg09028132	cg19055390
2832	cg07051221	cg08978399	cg27503499
2833	cg07054804	cg08973281	cg05400196
2834	cg07057687	cg08892499	cg08004045
2835	cg07058109	cg08864083	cg22186291
2836	cg07061582	cg08739576	cg06784563
2837	cg07064592	cg08607612	cg10626305
2838	cg07067280	cg08577953	cg12109728
2839	cg07067993	cg08407014	cg22932649
2840	cg07068045	cg08282578	cg27026202
2841	cg07070824	cg08134301	cg07377299
2842	cg07071036	cg07997682	cg16274890
2843	cg07071809	cg07925615	cg13050716
2844	cg07077978	cg07588779	cg04678901
2845	cg07078758	cg07581623	cg10378795
2846	cg07079828	cg07447845	cg17181336
2847	cg07083272	cg07347148	cg07520608
2848	cg07085590	cg07041214	cg17095737
2849	cg07086112	cg07038689	cg20679011
2850	cg07088950	cg06892907	cg12764441
2851	cg07090980	cg06892815	cg12474695
2852	cg07091062	cg06873590	cg24523322
2853	cg07094487	cg06812840	cg04847386
2854	cg07094993	cg06812836	cg26942121
2855	cg07095945	cg06647026	cg00982952
2856	cg07097367	cg06492744	cg04685228
2857	cg07097374	cg06378490	cg04800347
2858	cg07102397	cg06309980	cg09152259
2859	cg07102406	cg06208158	cg03287929

2860	cg07103201	cg06070445	cg24163668
2861	cg07104086	cg05976516	cg23990942
2862	cg07108443	cg05865548	cg01824284
2863	cg07109453	cg05826596	cg22986999
2864	cg07109788	cg05730150	cg14359798
2865	cg07116732	cg05536457	cg20840795
2866	cg07122529	cg05434397	cg09970023
2867	cg07123022	cg05283542	cg20195046
2868	cg07125976	cg05251593	cg20382493
2869	cg07127410	cg05037360	cg20927312
2870	cg07129714	cg04883903	cg16803044
2871	cg07129769	cg04847386	cg03439687
2872	cg07133097	cg04774930	cg10810290
2873	cg07139329	cg04724190	cg00401972
2874	cg07146723	cg04687939	cg13149996
2875	cg07148207	cg04375492	cg24881255
2876	cg07152894	cg04304033	cg26005197
2877	cg07155331	cg04015687	cg17431079
2878	cg07156484	cg03988952	cg12367789
2879	cg07160118	cg03669590	cg24873093
2880	cg07162085	cg03661844	cg09745791
2881	cg07165066	cg03627931	cg07057687
2882	cg07166333	cg03596635	cg16326073
2883	cg07171780	cg03575245	cg00017461
2884	cg07178379	cg03417342	cg07283582
2885	cg07181257	cg03379631	cg13393917
2886	cg07192047	cg03197830	cg03389789
2887	cg07192772	cg03098159	cg23998645
2888	cg07197326	cg03049691	cg05846633
2889	cg07204803	cg02938429	cg25232510
2890	cg07206497	cg02725620	cg19007948
2891	cg07206676	cg02451516	cg24123198
2892	cg07207742	cg02399019	cg01604946
2893	cg07217341	cg02325128	cg25403283
2894	cg07221518	cg02131465	cg18375421
2895	cg07230078	cg02068351	cg27213925
2896	cg07236781	cg01859228	cg09911316
2897	cg07236812	cg01818539	cg09124230
2898	cg07241090	cg01789743	cg09790523
2899	cg07260789	cg01705036	cg12528056
2900	cg07261940	cg01656717	cg26919387
2901	cg07265549	cg01580176	cg02153814
2902	cg07266910	cg01442620	cg08831077
2903	cg07267984	cg01357897	cg16499416
2904	cg07268431	cg01256365	cg18772205
2905	cg07270259	cg01000937	cg12450391
2906	cg07275305	cg00994389	cg03419014
2907	cg07279281	cg00927364	cg07835443
2908	cg07283859	cg00872170	cg23827284
2909	cg07284476	cg00808555	cg08097657
2910	cg07284519	cg00760872	cg12650685
2911	cg07285148	cg00757822	cg02926266
2912	cg07290269	cg00590590	cg06204229

2913	cg07290652	cg00184279	cg16234490
2914	cg07294624	cg27522078	cg12491594
2915	cg07295918	cg27349909	cg19951269
2916	cg07296854	cg27306243	cg18989491
2917	cg07306006	cg27239280	cg19238415
2918	cg07308243	cg27073066	cg09494646
2919	cg07309864	cg27065717	cg00386725
2920	cg07310500	cg26937267	cg11351841
2921	cg07324116	cg26837766	cg06809295
2922	cg07333510	cg26767198	cg26441230
2923	cg07336123	cg26744332	cg16863382
2924	cg07336964	cg26645242	cg07608094
2925	cg07337025	cg26526953	cg17797898
2926	cg07338476	cg26516446	cg08151983
2927	cg07344990	cg26467269	cg18327762
2928	cg07346310	cg26410484	cg11391732
2929	cg07347148	cg26332285	cg09829263
2930	cg07352757	cg26231761	cg27089352
2931	cg07353266	cg26177041	cg00245896
2932	cg07363131	cg26156321	cg26514080
2933	cg07365071	cg26132163	cg04011474
2934	cg07368061	cg25952596	cg21569714
2935	cg07374490	cg25831522	cg17683336
2936	cg07379550	cg25801976	cg02244933
2937	cg07384019	cg25677394	cg04623131
2938	cg07385220	cg25588389	cg15889057
2939	cg07391023	cg25586305	cg03666300
2940	cg07394799	cg25582924	cg04945557
2941	cg07396047	cg25390635	cg08787401
2942	cg07410597	cg25281849	cg19765175
2943	cg07412315	cg25115276	cg16776981
2944	cg07416344	cg25086418	cg18847089
2945	cg07420867	cg24926121	cg02621481
2946	cg07425568	cg24918798	cg02473287
2947	cg07425646	cg24738346	cg16139912
2948	cg07431229	cg24659758	cg00380428
2949	cg07433905	cg24589334	cg03899510
2950	cg07447742	cg24275626	cg19747992
2951	cg07447845	cg24148817	cg14468090
2952	cg07449447	cg23936766	cg05201312
2953	cg07456413	cg23900422	cg13637552
2954	cg07464578	cg23756143	cg11894474
2955	cg07467854	cg23712594	cg10273072
2956	cg07468956	cg23620047	cg14005246
2957	cg07471052	cg23538755	cg14514032
2958	cg07475178	cg23530850	cg23547892
2959	cg07477924	cg23468002	cg06881421
2960	cg07478111	cg23166740	cg05065603
2961	cg07478501	cg23010693	cg23024810
2962	cg07481273	cg22699768	cg20284937
2963	cg07483007	cg22558061	cg23032993
2964	cg07483266	cg22542420	cg16084313
2965	cg07486170	cg22255690	cg24998879

2966	cg07487572	cg22154992	cg06143290
2967	cg07495357	cg22013790	cg19137748
2968	cg07503211	cg21947590	cg26995244
2969	cg07505515	cg21573345	cg13798679
2970	cg07505901	cg21528927	cg08407014
2971	cg07506407	cg21335012	cg02102075
2972	cg07508304	cg21332304	cg10213353
2973	cg07511723	cg21304766	cg10361005
2974	cg07529658	cg21165519	cg11025750
2975	cg07531248	cg20925383	cg00299943
2976	cg07536018	cg20703928	cg16880392
2977	cg07545858	cg20663495	cg24926689
2978	cg07547336	cg20515846	cg00335361
2979	cg07549381	cg20505457	cg03446062
2980	cg07549715	cg20441046	cg08059361
2981	cg07552087	cg20329303	cg20197756
2982	cg07552868	cg20254251	cg10811485
2983	cg07555102	cg19995891	cg03049691
2984	cg07560287	cg19841423	cg11971852
2985	cg07561547	cg19593878	cg06489615
2986	cg07565042	cg19548524	cg14414154
2987	cg07571745	cg19262818	cg03514843
2988	cg07572251	cg19244380	cg24973993
2989	cg07574267	cg19178876	cg00968638
2990	cg07574854	cg19130973	cg02727423
2991	cg07577837	cg19084508	cg05205532
2992	cg07577934	cg19083914	cg25932290
2993	cg07581623	cg18978531	cg19293468
2994	cg07582655	cg18931850	cg12379145
2995	cg07584734	cg18878210	cg04366815
2996	cg07585257	cg18732855	cg09140274
2997	cg07586345	cg18657988	cg24670927
2998	cg07588779	cg18440069	cg09623085
2999	cg07605062	cg18047920	cg18371976
3000	cg07608094	cg17980999	cg08650647
3001	cg07612468	cg17799760	cg22979083
3002	cg07613391	cg17775332	cg09184899
3003	cg07617152	cg17695841	cg01403055
3004	cg07620571	cg17454920	cg06937357
3005	cg07624888	cg17319795	cg20567148
3006	cg07630327	cg17285177	cg01819707
3007	cg07638689	cg17113147	cg02552250
3008	cg07642499	cg17056676	cg15959921
3009	cg07661899	cg16829042	cg17206596
3010	cg07662964	cg16797656	cg05360477
3011	cg07665060	cg16706038	cg25373624
3012	cg07673230	cg16608596	cg02255721
3013	cg07674095	cg16523645	cg02462416
3014	cg07685601	cg16511831	cg03540589
3015	cg07691004	cg16421359	cg10798745
3016	cg07691914	cg16222790	cg10425513
3017	cg07701911	cg16158296	cg13637654
3018	cg07706695	cg16089233	cg00204976

3019	cg07713495	cg15934607	cg10301695
3020	cg07714708	cg15843262	cg09537448
3021	cg07716287	cg15726557	cg26041493
3022	cg07716847	cg15408497	cg00582671
3023	cg07720334	cg15164881	cg15034300
3024	cg07731639	cg15141238	cg07184807
3025	cg07732097	cg15016481	cg10692363
3026	cg07733800	cg14937228	cg20591120
3027	cg07733851	cg14715030	cg07642499
3028	cg07736115	cg14701122	cg27573308
3029	cg07740306	cg14553765	cg11139446
3030	cg07741192	cg14428166	cg09926389
3031	cg07743919	cg14362630	cg00550040
3032	cg07746532	cg14201387	cg06984255
3033	cg07748741	cg14143441	cg23811289
3034	cg07749442	cg14129169	cg10464467
3035	cg07754347	cg14081465	cg16031980
3036	cg07763497	cg14065153	cg26361671
3037	cg07770222	cg13991631	cg27040468
3038	cg07793033	cg13920768	cg11951169
3039	cg07795346	cg13852730	cg08843064
3040	cg07795968	cg13842262	cg07099056
3041	cg07800359	cg13543915	cg05415936
3042	cg07802350	cg13495850	cg02930033
3043	cg07802362	cg13461247	cg04003615
3044	cg07803375	cg13455439	cg07665060
3045	cg07804434	cg13169796	cg15532942
3046	cg07805999	cg13148921	cg14334310
3047	cg07810967	cg12921473	cg10607485
3048	cg07811634	cg12764441	cg04384031
3049	cg07813622	cg12710648	cg06159340
3050	cg07818978	cg12220376	cg27110054
3051	cg07826642	cg12185975	cg01296758
3052	cg07828612	cg12141218	cg15970457
3053	cg07832950	cg12017558	cg21450547
3054	cg07833472	cg11971852	cg04674497
3055	cg07834408	cg11897120	cg17333042
3056	cg07835154	cg11781564	cg21973907
3057	cg07835443	cg11731626	cg04777612
3058	cg07836226	cg11469540	cg04689023
3059	cg07841877	cg11197908	cg14058027
3060	cg07844828	cg10980495	cg14692854
3061	cg07849237	cg10774480	cg13278105
3062	cg07850832	cg10726559	cg23217547
3063	cg07858195	cg10654373	cg03727333
3064	cg07862575	cg10555744	cg12734386
3065	cg07866001	cg10492836	cg19400179
3066	cg07867517	cg10332704	cg02573234
3067	cg07874353	cg10298992	cg00137696
3068	cg07876450	cg10294836	cg17724627
3069	cg07880135	cg10288921	cg13710553
3070	cg07888884	cg10021749	cg23181573
3071	cg07894983	cg10005273	cg11327857

3072	cg07899389	cg09687417	cg10146514
3073	cg07900344	cg09580592	cg25736617
3074	cg07904865	cg09555736	cg25582924
3075	cg07910945	cg09541576	cg07309864
3076	cg07912144	cg09500421	cg16159491
3077	cg07921503	cg09385972	cg06907347
3078	cg07925615	cg09370702	cg16391182
3079	cg07927540	cg09335712	cg17454920
3080	cg07929112	cg09086087	cg16221927
3081	cg07937803	cg09063434	cg13148921
3082	cg07938459	cg09035930	cg18181891
3083	cg07941953	cg09028383	cg14428166
3084	cg07946977	cg08954277	cg24629431
3085	cg07951978	cg08883485	cg25940447
3086	cg07959741	cg08818094	cg08823837
3087	cg07962882	cg08774694	cg09499482
3088	cg07964527	cg08613144	cg14920846
3089	cg07965110	cg08611376	cg22222413
3090	cg07965566	cg08437576	cg25629077
3091	cg07969918	cg08412215	cg13029400
3092	cg07976390	cg08276755	cg10255535
3093	cg07986222	cg08242475	cg00262446
3094	cg07990198	cg08098420	cg07447845
3095	cg07990658	cg08037719	cg19372504
3096	cg07992340	cg07969918	cg18760055
3097	cg08003887	cg07862575	cg15633699
3098	cg08004045	cg07802362	cg05898188
3099	cg08005460	cg07748741	cg16892393
3100	cg08033285	cg07665929	cg12939390
3101	cg08037719	cg07620571	cg02832357
3102	cg08039592	cg07584734	cg15174926
3103	cg08043592	cg07131274	cg19937288
3104	cg08044294	cg07122529	cg12178980
3105	cg08059361	cg07027613	cg23514135
3106	cg08061755	cg07010552	cg09534489
3107	cg08062713	cg06749819	cg07463059
3108	cg08065374	cg06673840	cg06813419
3109	cg08067991	cg06641607	cg04843968
3110	cg08070235	cg06532257	cg23726802
3111	cg08073527	cg06390613	cg02055483
3112	cg08088222	cg06254425	cg16770054
3113	cg08089518	cg06219670	cg17893426
3114	cg08091050	cg06123891	cg12832282
3115	cg08091706	cg06028151	cg24129923
3116	cg08094614	cg05816041	cg05150641
3117	cg08097359	cg05750323	cg10589745
3118	cg08098420	cg05657292	cg16616765
3119	cg08101977	cg05602356	cg12419932
3120	cg08103691	cg05516020	cg03591954
3121	cg08104146	cg05494073	cg19944656
3122	cg08104310	cg05235248	cg19220272
3123	cg08105005	cg05056653	cg27133959
3124	cg08106635	cg05041061	cg22532155

3125	cg08108655	cg04995521	cg05272587
3126	cg08110688	cg04967410	cg02976723
3127	cg08112616	cg04967009	cg18761894
3128	cg08113187	cg04872027	cg21168101
3129	cg08115297	cg04839616	cg22376204
3130	cg08115732	cg04706201	cg14059835
3131	cg08119777	cg04655520	cg24030630
3132	cg08132025	cg04558973	cg05575733
3133	cg08132525	cg04394390	cg26097051
3134	cg08132573	cg04261952	cg24097814
3135	cg08134301	cg04135242	cg09142595
3136	cg08135904	cg04100337	cg26164879
3137	cg08137602	cg03956820	cg20952105
3138	cg08141395	cg03769734	cg02622601
3139	cg08143701	cg03731348	cg20051177
3140	cg08148618	cg03501128	cg13728131
3141	cg08149747	cg03431524	cg10965996
3142	cg08151983	cg03407184	cg18829162
3143	cg08157446	cg03333776	cg00257455
3144	cg08168529	cg03260744	cg14706807
3145	cg08169341	cg03036214	cg12595697
3146	cg08173709	cg02936315	cg16766325
3147	cg08173959	cg02807008	cg01259126
3148	cg08180831	cg02696830	cg23386779
3149	cg08183125	cg02665399	cg09330834
3150	cg08185095	cg02654449	cg19931529
3151	cg08193650	cg02435083	cg12395479
3152	cg08197880	cg02240030	cg12817352
3153	cg08198711	cg02058267	cg10810026
3154	cg08206098	cg02040734	cg08251036
3155	cg08206156	cg01930756	cg08509383
3156	cg08206881	cg01770296	cg13953458
3157	cg08208595	cg01589629	cg06890747
3158	cg08209934	cg01493020	cg04394390
3159	cg08215532	cg01462184	cg12948543
3160	cg08218971	cg01456989	cg10513161
3161	cg08236537	cg01415527	cg19244553
3162	cg08242475	cg01260820	cg01512113
3163	cg08248600	cg00991659	cg27526774
3164	cg08250738	cg00961416	cg02886591
3165	cg08251036	cg00950038	cg05334190
3166	cg08258506	cg00676360	cg23674358
3167	cg08258867	cg00484122	cg19605931
3168	cg08259083	cg00444740	cg09780231
3169	cg08261841	cg00428526	cg01794926
3170	cg08264519	cg00210271	cg20519665
3171	cg08276755	cg00043005	cg18929814
3172	cg08276889	cg27588653	cg27655716
3173	cg08277216	cg27560818	cg25600823
3174	cg08281590	cg27441486	cg14104252
3175	cg08282578	cg27143703	cg15600176
3176	cg08288016	cg27105598	cg13583088
3177	cg08291302	cg27020028	cg03385114

3178	cg08293075	cg26835798	cg03367519
3179	cg08293102	cg26810214	cg11108115
3180	cg08293536	cg26614264	cg23348723
3181	cg08304608	cg26605683	cg19714865
3182	cg08307171	cg26381919	cg15726557
3183	cg08313842	cg26365854	cg22809726
3184	cg08329113	cg26365254	cg05917480
3185	cg08331427	cg26053832	cg06706550
3186	cg08334780	cg25866889	cg11225330
3187	cg08342966	cg25684961	cg14650228
3188	cg08345465	cg25552843	cg04921335
3189	cg08351131	cg25484139	cg09358422
3190	cg08351911	cg25442652	cg20208009
3191	cg08362308	cg25235326	cg24247537
3192	cg08365638	cg25136955	cg04262465
3193	cg08365802	cg25028855	cg12549211
3194	cg08369014	cg24648594	cg16421653
3195	cg08378932	cg24499677	cg01290421
3196	cg08381596	cg24426483	cg02605292
3197	cg08397968	cg24342377	cg21727276
3198	cg08400424	cg24249775	cg01359676
3199	cg08407014	cg24136288	cg12251803
3200	cg08409074	cg23921860	cg01274233
3201	cg08411881	cg23812660	cg03815917
3202	cg08412215	cg23674358	cg16514214
3203	cg08418375	cg23484234	cg16328007
3204	cg08419373	cg23385847	cg08215532
3205	cg08426200	cg23250528	cg25153629
3206	cg08428985	cg23133355	cg25441338
3207	cg08433725	cg23093846	cg02581587
3208	cg08436467	cg23019935	cg24969902
3209	cg08437576	cg22904096	cg16700555
3210	cg08437802	cg22619784	cg05452391
3211	cg08441269	cg22315164	cg10363202
3212	cg08450122	cg22186291	cg25557739
3213	cg08457169	cg22180848	cg11476985
3214	cg08458912	cg21789440	cg11530995
3215	cg08463581	cg21642054	cg04095995
3216	cg08463775	cg21637392	cg07010552
3217	cg08469834	cg21464492	cg08329113
3218	cg08480739	cg21404476	cg08705647
3219	cg08489478	cg20840795	cg17009731
3220	cg08494755	cg20781516	cg18191688
3221	cg08506026	cg20699157	cg25953130
3222	cg08507725	cg20662658	cg12865675
3223	cg08509383	cg20595846	cg18172013
3224	cg08510718	cg20461270	cg25613180
3225	cg08521178	cg20061812	cg18487916
3226	cg08523978	cg20000866	cg23029159
3227	cg08524717	cg19995595	cg24902250
3228	cg08527124	cg19774846	cg08288016
3229	cg08535779	cg19730379	cg14095720
3230	cg08537794	cg19582744	cg19138499

3231	cg08544606	cg19541247	cg15598425
3232	cg08545132	cg19502812	cg03613003
3233	cg08550729	cg19253339	cg15963326
3234	cg08552167	cg19190016	cg15408640
3235	cg08552290	cg19162768	cg16550656
3236	cg08552446	cg19120897	cg15545942
3237	cg08553857	cg18905668	cg01559356
3238	cg08559648	cg18854872	cg07070824
3239	cg08565675	cg18729298	cg23812679
3240	cg08566548	cg18704218	cg07041214
3241	cg08571020	cg18628371	cg02471028
3242	cg08572315	cg18467406	cg21205855
3243	cg08576185	cg18259487	cg14960869
3244	cg08577953	cg18259342	cg10617494
3245	cg08590257	cg18259003	cg05289897
3246	cg08591538	cg18163092	cg04517274
3247	cg08603083	cg18148375	cg15089806
3248	cg08604505	cg17796043	cg01246855
3249	cg08605347	cg17747635	cg13794839
3250	cg08605965	cg17703629	cg03401464
3251	cg08606951	cg17692230	cg24706981
3252	cg08607612	cg17534540	cg02538557
3253	cg08611376	cg17400113	cg16168321
3254	cg08613794	cg17340779	cg26668713
3255	cg08616182	cg17221941	cg07910945
3256	cg08616663	cg17213154	cg05865548
3257	cg08619515	cg17199010	cg06530983
3258	cg08622923	cg16897462	cg24301288
3259	cg08625990	cg16695570	cg14601444
3260	cg08628233	cg16516980	cg07638689
3261	cg08631328	cg16436210	cg13444374
3262	cg08636203	cg16309127	cg06456125
3263	cg08644106	cg16144562	cg06995299
3264	cg08650647	cg15888569	cg14202146
3265	cg08665249	cg15850851	cg13400018
3266	cg08677140	cg15806038	cg03934478
3267	cg08681293	cg15678662	cg23679798
3268	cg08682625	cg15627593	cg07585257
3269	cg08687995	cg15487498	cg01959238
3270	cg08688659	cg15450139	cg10082525
3271	cg08692006	cg15260437	cg00927364
3272	cg08692104	cg15031106	cg00757822
3273	cg08694923	cg14462432	cg21225667
3274	cg08701134	cg14156441	cg05493407
3275	cg08701675	cg14114546	cg25785495
3276	cg08703151	cg14064268	cg07090980
3277	cg08712631	cg14016236	cg09807875
3278	cg08719095	cg13908988	cg25413575
3279	cg08726900	cg13800349	cg21698310
3280	cg08732879	cg13750566	cg03889044
3281	cg08733553	cg13736131	cg03608003
3282	cg08737189	cg13734106	cg06237774
3283	cg08739576	cg13731108	cg24617568

3284	cg08740008	cg13726456	cg16496462
3285	cg08740088	cg13661703	cg05260236
3286	cg08741843	cg13611006	cg00201819
3287	cg08749599	cg13549638	cg03824114
3288	cg08750440	cg13473184	cg09214456
3289	cg08764162	cg13383819	cg11852646
3290	cg08767627	cg13236409	cg03938110
3291	cg08768904	cg13093023	cg02926747
3292	cg08771923	cg13035910	cg01953240
3293	cg08775774	cg12982283	cg16861209
3294	cg08775793	cg12887832	cg14070108
3295	cg08777316	cg12640387	cg12195230
3296	cg08779777	cg12499572	cg03812676
3297	cg08782674	cg12240761	cg22304399
3298	cg08784950	cg12240637	cg06937205
3299	cg08787401	cg12231088	cg02304580
3300	cg08805497	cg12112434	cg22890896
3301	cg08812108	cg11995490	cg08250738
3302	cg08812784	cg11894474	cg25573640
3303	cg08814268	cg11802013	cg26053832
3304	cg08818094	cg11748187	cg04375492
3305	cg08823837	cg11620716	cg10077985
3306	cg08823975	cg11198094	cg25594550
3307	cg08824291	cg11084269	cg14939096
3308	cg08825895	cg10734581	cg02862467
3309	cg08826080	cg10721220	cg12403778
3310	cg08829140	cg10607603	cg02348449
3311	cg08831077	cg10571824	cg21247923
3312	cg08831531	cg10513161	cg00244391
3313	cg08833741	cg10494703	cg06255601
3314	cg08836353	cg10354880	cg11553311
3315	cg08841613	cg10352104	cg08552167
3316	cg08847512	cg10146514	cg02144874
3317	cg08850243	cg10146112	cg05168015
3318	cg08860136	cg10071852	cg09712234
3319	cg08864042	cg10062065	cg25148456
3320	cg08864083	cg10057853	cg14523734
3321	cg08866865	cg10004897	cg01859228
3322	cg08869160	cg09939191	cg04304033
3323	cg08883078	cg09845604	cg03796321
3324	cg08883485	cg09782637	cg15383141
3325	cg08886695	cg09754828	cg09688726
3326	cg08892499	cg09701233	cg01486610
3327	cg08894401	cg09379188	cg00044665
3328	cg08899531	cg09186408	cg00210271
3329	cg08904630	cg09076584	cg18994446
3330	cg08912841	cg09057885	cg08437576
3331	cg08913523	cg08986340	cg10550245
3332	cg08914150	cg08644340	cg19736226
3333	cg08915024	cg08625990	cg10262770
3334	cg08920210	cg08263571	cg23117583
3335	cg08924256	cg08206098	cg02451516
3336	cg08924374	cg08141395	cg03197830

3337	cg08932665	cg08135904	cg11497526
3338	cg08933276	cg08105005	cg07058109
3339	cg08934318	cg08073527	cg08727428
3340	cg08934799	cg07951978	cg05603553
3341	cg08945920	cg07941953	cg14875394
3342	cg08947774	cg07834408	cg00355286
3343	cg08955461	cg07552087	cg25463863
3344	cg08964948	cg07545858	cg03391019
3345	cg08966188	cg07541559	cg00275503
3346	cg08966413	cg07337025	cg07034004
3347	cg08970648	cg07171780	cg27278001
3348	cg08972190	cg07044523	cg07661899
3349	cg08973281	cg07026910	cg11945095
3350	cg08974450	cg06967828	cg02246916
3351	cg08975850	cg06919312	cg27021553
3352	cg08976646	cg06870740	cg26794830
3353	cg08978399	cg06861572	cg26053547
3354	cg08983215	cg06851844	cg04440361
3355	cg08986727	cg06678137	cg14227418
3356	cg08993878	cg06573945	cg12160664
3357	cg09004351	cg06556244	cg18907942
3358	cg09006420	cg06530983	cg14935206
3359	cg09006487	cg06407441	cg26937267
3360	cg09007161	cg06323896	cg14351526
3361	cg09009788	cg06251016	cg10052840
3362	cg09015905	cg06232205	cg06012410
3363	cg09018739	cg06198069	cg06474428
3364	cg09020697	cg06098693	cg08782674
3365	cg09029046	cg05971891	cg06522179
3366	cg09029193	cg05900955	cg03441279
3367	cg09033641	cg05817833	cg11093939
3368	cg09035930	cg05813328	cg01138652
3369	cg09037712	cg05757530	cg21070669
3370	cg09038962	cg05621583	cg18237191
3371	cg09040552	cg05584950	cg18978531
3372	cg09041614	cg05309989	cg09278623
3373	cg09043524	cg05201300	cg07899389
3374	cg09057885	cg05032848	cg25878830
3375	cg09063434	cg05024916	cg08091050
3376	cg09073443	cg04930596	cg00830283
3377	cg09074223	cg04906603	cg14081465
3378	cg09075515	cg04815973	cg23493119
3379	cg09076584	cg04803994	cg18601229
3380	cg09076960	cg04787317	cg01945624
3381	cg09078622	cg04697056	cg20951650
3382	cg09086087	cg04677227	cg14074351
3383	cg09100373	cg04633661	cg16201762
3384	cg09100695	cg04573078	cg08966188
3385	cg09101894	cg04415736	cg21242508
3386	cg09109383	cg04392554	cg05851042
3387	cg09109553	cg04269098	cg26156321
3388	cg09115960	cg04248332	cg04249706
3389	cg09118048	cg04195226	cg20793665

3390	cg09119967	cg04188241	cg09541576
3391	cg09123760	cg04091816	cg27230140
3392	cg09123961	cg04086327	cg10786098
3393	cg09124230	cg03980268	cg11468635
3394	cg09126859	cg03630710	cg06439736
3395	cg09139451	cg03528118	cg25136955
3396	cg09140274	cg03426615	cg23894219
3397	cg09142117	cg03339817	cg15658793
3398	cg09142595	cg03218909	cg05483875
3399	cg09152259	cg03125400	cg10546888
3400	cg09163478	cg03035704	cg13458561
3401	cg09163745	cg02970384	cg07833472
3402	cg09171093	cg02959112	cg10512951
3403	cg09172973	cg02832905	cg24630426
3404	cg09174690	cg02707799	cg12901650
3405	cg09180239	cg02604995	cg14833024
3406	cg09184899	cg02512395	cg26687830
3407	cg09186408	cg02348449	cg12240637
3408	cg09196257	cg02214441	cg02827132
3409	cg09198138	cg02162605	cg10021122
3410	cg09198283	cg02133557	cg12688942
3411	cg09198360	cg02067239	cg19402179
3412	cg09213124	cg02017718	cg19113668
3413	cg09214456	cg02010686	cg11024450
3414	cg09216143	cg01968525	cg14088181
3415	cg09218398	cg01810186	cg08986727
3416	cg09226986	cg01486910	cg24549289
3417	cg09228051	cg01442132	cg25032991
3418	cg09233395	cg01414464	cg18193482
3419	cg09238162	cg01399475	cg13786171
3420	cg09246253	cg01377082	cg21929969
3421	cg09248660	cg01296758	cg02869364
3422	cg09249800	cg01150866	cg23987336
3423	cg09251291	cg01048810	cg24639863
3424	cg09253696	cg00952822	cg23510131
3425	cg09255505	cg00784105	cg05270552
3426	cg09256716	cg00757262	cg08342966
3427	cg09258509	cg00694888	cg19849641
3428	cg09259595	cg00673191	cg04508340
3429	cg09262882	cg00669182	cg16359657
3430	cg09267483	cg00488716	cg15016481
3431	cg09268338	cg00372132	cg16766036
3432	cg09280800	cg00289254	cg16885096
3433	cg09293488	cg00262446	cg10457066
3434	cg09300185	cg00204976	cg08343075
3435	cg09309208	cg00187129	cg03566001
3436	cg09318508	cg00048381	cg26214948
3437	cg09320595	cg27616751	cg20524889
3438	cg09322899	cg27540799	cg01543307
3439	cg09326135	cg27478167	cg05190559
3440	cg09326362	cg27469606	cg08976646
3441	cg09327804	cg27068650	cg00367649
3442	cg09330834	cg27042523	cg19772651

3443	cg09331409	cg26947127	cg00050289
3444	cg09333143	cg26941073	cg22990726
3445	cg09334629	cg26871875	cg12508392
3446	cg09335314	cg26798772	cg10730148
3447	cg09335712	cg26714514	cg17477806
3448	cg09342325	cg26709309	cg07654843
3449	cg09353705	cg26709300	cg14156650
3450	cg09356020	cg26367275	cg18030799
3451	cg09358422	cg26350921	cg00333583
3452	cg09358481	cg26184501	cg14502713
3453	cg09358961	cg25909064	cg06808089
3454	cg09358973	cg25846061	cg21617218
3455	cg09368832	cg25722029	cg19382808
3456	cg09370299	cg25668093	cg16145211
3457	cg09370702	cg25623727	cg09597710
3458	cg09371084	cg25613180	cg26550337
3459	cg09372617	cg25595641	cg09814231
3460	cg09373983	cg25591418	cg20322837
3461	cg09375892	cg25535708	cg16831894
3462	cg09379188	cg25441338	cg22221847
3463	cg09385972	cg25382652	cg15164881
3464	cg09386073	cg25199850	cg17696194
3465	cg09389557	cg24990238	cg15081886
3466	cg09391399	cg24881558	cg07560287
3467	cg09397692	cg24468934	cg13549638
3468	cg09400566	cg24423806	cg27578381
3469	cg09406921	cg24164238	cg21292981
3470	cg09407273	cg24073352	cg20757748
3471	cg09410453	cg23989207	cg03424213
3472	cg09421126	cg23735646	cg00912277
3473	cg09437283	cg23626908	cg25504217
3474	cg09444036	cg23617947	cg07433905
3475	cg09457490	cg23362032	cg08545132
3476	cg09459955	cg23280999	cg04688645
3477	cg09464883	cg23273465	cg14113353
3478	cg09465065	cg23262213	cg23166740
3479	cg09473315	cg23225637	cg09126859
3480	cg09474229	cg23181573	cg20262021
3481	cg09483967	cg23037644	cg06038180
3482	cg09486967	cg22516162	cg23260554
3483	cg09489567	cg22304399	cg12612065
3484	cg09494609	cg22253401	cg10824354
3485	cg09494646	cg22221847	cg19577548
3486	cg09495643	cg22133366	cg18032164
3487	cg09499482	cg21831649	cg02604995
3488	cg09500421	cg21756765	cg24426483
3489	cg09501516	cg21439672	cg08954601
3490	cg09510085	cg21282907	cg16680624
3491	cg09511351	cg21268578	cg17103856
3492	cg09511662	cg21205855	cg00359087
3493	cg09524639	cg21196487	cg15684702
3494	cg09524658	cg21176488	cg26877336
3495	cg09526129	cg21171299	cg16293569

3496	cg09529783	cg21168101	cg12511749
3497	cg09534489	cg21158737	cg17525357
3498	cg09534872	cg21063361	cg01369033
3499	cg09536516	cg21048763	cg09358961
3500	cg09537448	cg21043481	cg23085846
3501	cg09540676	cg21032564	cg10000148
3502	cg09541576	cg21011702	cg02278728
3503	cg09544892	cg20985758	cg04026354
3504	cg09548893	cg20916068	cg17844448
3505	cg09551019	cg20881888	cg26934881
3506	cg09553581	cg20812052	cg22998873
3507	cg09554406	cg20540235	cg10467217
3508	cg09555736	cg20426710	cg14966346
3509	cg09558069	cg20354777	cg24773493
3510	cg09567735	cg20349199	cg01859460
3511	cg09569347	cg20334115	cg09579081
3512	cg09579081	cg20300794	cg04622001
3513	cg09580592	cg20143111	cg10317175
3514	cg09580859	cg20000602	cg20937296
3515	cg09581065	cg19931529	cg00563061
3516	cg09586198	cg19865472	cg21845869
3517	cg09596336	cg19598875	cg14983540
3518	cg09597710	cg19439043	cg11860238
3519	cg09602138	cg19238415	cg16533510
3520	cg09607915	cg19233001	cg24260710
3521	cg09623085	cg19144954	cg22060153
3522	cg09627339	cg19062489	cg13054523
3523	cg09631059	cg19006220	cg15450139
3524	cg09631175	cg18965684	cg21004924
3525	cg09634481	cg18960324	cg23222247
3526	cg09637963	cg18817444	cg26889437
3527	cg09641077	cg18568570	cg06093152
3528	cg09643151	cg18539086	cg14870682
3529	cg09647352	cg18442286	cg16027775
3530	cg09647797	cg18181891	cg24674087
3531	cg09649196	cg18172013	cg16016960
3532	cg09650907	cg18158033	cg06783380
3533	cg09659803	cg18142262	cg19032492
3534	cg09665571	cg17966344	cg24102222
3535	cg09669930	cg17868840	cg27192012
3536	cg09672200	cg17742459	cg25066857
3537	cg09674368	cg17649815	cg06662568
3538	cg09674500	cg17482237	cg10768996
3539	cg09675604	cg17130544	cg19357195
3540	cg09680719	cg16718902	cg27271532
3541	cg09685505	cg16584688	cg23196346
3542	cg09687417	cg16529592	cg07139329
3543	cg09688726	cg16522491	cg04476286
3544	cg09695261	cg16521443	cg15518113
3545	cg09697259	cg16425726	cg07733800
3546	cg09700054	cg16084313	cg13730600
3547	cg09701233	cg16056849	cg01640684
3548	cg09702334	cg15899800	cg00674220

3549	cg09703323	cg15817440	cg20329303
3550	cg09711421	cg15658793	cg11523799
3551	cg09712234	cg15460348	cg08104310
3552	cg09719124	cg15410236	cg15561453
3553	cg09727436	cg15352671	cg22974467
3554	cg09729166	cg15209369	cg22414151
3555	cg09742751	cg15161050	cg07736115
3556	cg09743906	cg14992771	cg13856126
3557	cg09754828	cg14788686	cg11275487
3558	cg09776314	cg14753356	cg11815790
3559	cg09777637	cg14658346	cg13555689
3560	cg09780231	cg14638315	cg00296038
3561	cg09781414	cg14519777	cg07665929
3562	cg09782637	cg14487665	cg06116260
3563	cg09786420	cg14286546	cg27216597
3564	cg09789315	cg14100888	cg09889848
3565	cg09790512	cg14096042	cg16519668
3566	cg09790523	cg14087401	cg13153540
3567	cg09801824	cg13996750	cg23546343
3568	cg09807875	cg13964068	cg13933043
3569	cg09809922	cg13962153	cg01578585
3570	cg09811393	cg13832457	cg17202331
3571	cg09812790	cg13765417	cg23192604
3572	cg09814231	cg13744954	cg25154482
3573	cg09824023	cg13560612	cg00706536
3574	cg09825670	cg13499923	cg07295918
3575	cg09829263	cg13458561	cg04955856
3576	cg09832108	cg13171107	cg14842237
3577	cg09835239	cg13135376	cg21831649
3578	cg09837656	cg13099829	cg27349909
3579	cg09845604	cg13070650	cg14425960
3580	cg09848749	cg12900489	cg26441486
3581	cg09849774	cg12832282	cg27051683
3582	cg09856367	cg12822242	cg04574046
3583	cg09858160	cg12799862	cg15402232
3584	cg09859805	cg12734386	cg18397308
3585	cg09863094	cg12500891	cg06338958
3586	cg09868556	cg12417815	cg12141218
3587	cg09869286	cg12371263	cg00371570
3588	cg09875326	cg12343881	cg12179884
3589	cg09884107	cg12166802	cg08954277
3590	cg09886931	cg12087627	cg14574066
3591	cg09889848	cg12069547	cg02700194
3592	cg09890339	cg11961845	cg16726374
3593	cg09890597	cg11700959	cg01869896
3594	cg09890746	cg11646638	cg19724698
3595	cg09891393	cg11496593	cg17929859
3596	cg09892984	cg11401278	cg00145911
3597	cg09896120	cg11307417	cg01866330
3598	cg09896867	cg11288260	cg17322655
3599	cg09897002	cg11254317	cg19348272
3600	cg09897737	cg11103999	cg26905281
3601	cg09901529	cg11060194	cg19479935

3602	cg09901532	cg10921517	cg15970890
3603	cg09905732	cg10893014	cg15089272
3604	cg09906558	cg10885338	cg10298992
3605	cg09906780	cg10809282	cg20218614
3606	cg09911316	cg10796022	cg22628240
3607	cg09911521	cg10790470	cg13571319
3608	cg09912552	cg10756252	cg03495660
3609	cg09913449	cg10517096	cg12774921
3610	cg09915232	cg10276623	cg01293277
3611	cg09915883	cg10273072	cg02053092
3612	cg09920043	cg10213353	cg14266952
3613	cg09921821	cg10121745	cg21887193
3614	cg09925620	cg09989037	cg04315086
3615	cg09925747	cg09926389	cg10860364
3616	cg09926389	cg09892984	cg09142117
3617	cg09939191	cg09814231	cg11526020
3618	cg09950920	cg09743906	cg23492249
3619	cg09956907	cg09327804	cg11701615
3620	cg09972881	cg09041614	cg01923724
3621	cg09975093	cg08974450	cg02435083
3622	cg09975561	cg08877591	cg18604419
3623	cg09976716	cg08787401	cg11524428
3624	cg09989037	cg08665249	cg03215152
3625	cg09996971	cg08604505	cg18581669
3626	cg10004897	cg08603083	cg03301025
3627	cg10005273	cg08433725	cg10780981
3628	cg10006956	cg08390994	cg17400113
3629	cg10009207	cg08313842	cg22047282
3630	cg10009737	cg08281590	cg02495250
3631	cg10012530	cg08198711	cg09988676
3632	cg10016358	cg08183125	cg24890045
3633	cg10016364	cg08157446	cg27489873
3634	cg10017293	cg08148618	cg19130981
3635	cg10018519	cg07804434	cg12220376
3636	cg10019018	cg07727134	cg03512577
3637	cg10021122	cg07605062	cg27073066
3638	cg10021749	cg07486170	cg00236766
3639	cg10024484	cg07425646	cg13390372
3640	cg10024508	cg07349899	cg08642081
3641	cg10024583	cg07296260	cg18382744
3642	cg10034572	cg07162085	cg25677394
3643	cg10036892	cg07090980	cg02685896
3644	cg10037894	cg07038822	cg11036962
3645	cg10052840	cg06891043	cg26280578
3646	cg10053473	cg06884199	cg21591452
3647	cg10055950	cg06849167	cg18696900
3648	cg10057853	cg06792186	cg23828593
3649	cg10059410	cg06689039	cg11252765
3650	cg10060631	cg06584329	cg23976336
3651	cg10063407	cg06562964	cg26767198
3652	cg10063637	cg06546521	cg06633739
3653	cg10065736	cg06474428	cg03660377
3654	cg10077985	cg06407371	cg03618741

3655	cg10082445	cg06269419	cg18527971
3656	cg10082525	cg06132803	cg16585619
3657	cg10088715	cg06018095	cg22501449
3658	cg10089081	cg05918682	cg12927785
3659	cg10095352	cg05884394	cg23301539
3660	cg10099813	cg05797224	cg05272807
3661	cg10101600	cg05590156	cg09385972
3662	cg10102102	cg05544807	cg07555102
3663	cg10107322	cg05508315	cg24925163
3664	cg10107330	cg05334190	cg25247887
3665	cg10109146	cg05299486	cg02263260
3666	cg10115918	cg05279172	cg18856214
3667	cg10119288	cg05150697	cg00991659
3668	cg10121745	cg05088513	cg15874302
3669	cg10126181	cg05060427	cg13849495
3670	cg10129154	cg04774496	cg13522118
3671	cg10131972	cg04743876	cg07770222
3672	cg10142436	cg04714939	cg09100373
3673	cg10146112	cg04628014	cg07129714
3674	cg10146514	cg04529785	cg02047646
3675	cg10147974	cg04402948	cg23003961
3676	cg10149870	cg04402828	cg04217556
3677	cg10149889	cg04293307	cg15974867
3678	cg10168009	cg04145890	cg01716827
3679	cg10174191	cg04129282	cg21979773
3680	cg10174683	cg04100595	cg00637826
3681	cg10190898	cg03958928	cg20989443
3682	cg10193870	cg03954280	cg24825262
3683	cg10202788	cg03555203	cg11912239
3684	cg10208609	cg03528037	cg03950492
3685	cg10213353	cg03216474	cg12860109
3686	cg10213762	cg03041650	cg01663970
3687	cg10214757	cg02993070	cg24152000
3688	cg10215501	cg02935024	cg13491731
3689	cg10218733	cg02835462	cg26383138
3690	cg10223066	cg02785745	cg26873164
3691	cg10227280	cg02727423	cg11697861
3692	cg10227312	cg02688118	cg23202053
3693	cg10233454	cg02631921	cg04392554
3694	cg10237442	cg02581587	cg02113604
3695	cg10239163	cg02447556	cg01428928
3696	cg10247864	cg02365900	cg03036398
3697	cg10248100	cg02346135	cg00989505
3698	cg10248148	cg02287325	cg19949955
3699	cg10255535	cg02272457	cg13061648
3700	cg10257671	cg02015219	cg03199926
3701	cg10262770	cg01804278	cg23626908
3702	cg10263684	cg01761758	cg25451660
3703	cg10273072	cg01733438	cg08836729
3704	cg10273135	cg01730064	cg25963540
3705	cg10276623	cg01523759	cg09524639
3706	cg10277646	cg01400750	cg23484234
3707	cg10278297	cg01333205	cg00266968

3708	cg10278394	cg01329151	cg07425646
3709	cg10288921	cg01166827	cg08604505
3710	cg10294836	cg01103253	cg05546878
3711	cg10296062	cg01069043	cg05399244
3712	cg10301695	cg00998132	cg00347938
3713	cg10311315	cg00978248	cg21361322
3714	cg10311754	cg00802617	cg07572251
3715	cg10317175	cg00729995	cg22345063
3716	cg10319905	cg00637745	cg13093285
3717	cg10330187	cg00562504	cg11390978
3718	cg10332437	cg00503920	cg13488851
3719	cg10332704	cg00410600	cg25490527
3720	cg10333594	cg00380428	cg04260676
3721	cg10335736	cg00268330	cg07673230
3722	cg10338364	cg00106093	cg20010135
3723	cg10338518	cg00062282	cg21997465
3724	cg10352104	cg00060715	cg17459290
3725	cg10354880	cg27618305	cg11381282
3726	cg10356060	cg27494100	cg26613811
3727	cg10357989	cg27424692	cg05873568
3728	cg10360139	cg27399387	cg12120430
3729	cg10361005	cg27378814	cg01667978
3730	cg10363202	cg27269190	cg04568774
3731	cg10364862	cg27231912	cg24253714
3732	cg10369955	cg27212729	cg07577934
3733	cg10370305	cg27196496	cg09674500
3734	cg10371037	cg27110054	cg03763874
3735	cg10378795	cg27044591	cg00841141
3736	cg10380108	cg26977644	cg00421693
3737	cg10388307	cg26964426	cg20971998
3738	cg10392840	cg26889437	cg22295745
3739	cg10408178	cg26796443	cg11748187
3740	cg10409799	cg26648465	cg13048437
3741	cg10418812	cg26609642	cg00864551
3742	cg10421435	cg26597727	cg05337637
3743	cg10425513	cg26574247	cg21947590
3744	cg10427430	cg26462488	cg07230078
3745	cg10434728	cg26413192	cg05235248
3746	cg10453420	cg26383138	cg22154992
3747	cg10461264	cg26207909	cg08550729
3748	cg10464467	cg26187123	cg17616283
3749	cg10467217	cg26133909	cg26262482
3750	cg10472263	cg26104640	cg21158737
3751	cg10472320	cg25953130	cg04482110
3752	cg10480329	cg25854527	cg01170069
3753	cg10488292	cg25815219	cg07674095
3754	cg10492836	cg25652751	cg18930928
3755	cg10493436	cg25567938	cg06765552
3756	cg10494703	cg25557739	cg00277804
3757	cg10495754	cg25405238	cg08092318
3758	cg10496150	cg25390243	cg00096810
3759	cg10504150	cg25178749	cg21696975
3760	cg10507281	cg25066857	cg22482278

3761	cg10507988	cg25007422	cg26510473
3762	cg10508317	cg24960799	cg08607612
3763	cg10510707	cg24857399	cg27100436
3764	cg10512089	cg24711397	cg07574854
3765	cg10512408	cg24639863	cg20143111
3766	cg10512951	cg24453118	cg01487195
3767	cg10513161	cg24304880	cg11835347
3768	cg10514113	cg24114708	cg16554164
3769	cg10515332	cg24004007	cg25187161
3770	cg10517096	cg23989757	cg17796593
3771	cg10519271	cg23945793	cg22786631
3772	cg10520924	cg23906872	cg00665106
3773	cg10531372	cg23844904	cg27105598
3774	cg10532384	cg23811289	cg18047920
3775	cg10533694	cg23797615	cg23477348
3776	cg10543634	cg23782083	cg04998447
3777	cg10545738	cg23463186	cg05715422
3778	cg10550245	cg23457862	cg17341136
3779	cg10552275	cg23206032	cg25321935
3780	cg10555744	cg23117583	cg26308909
3781	cg10558233	cg23089261	cg07748741
3782	cg10563645	cg23087358	cg14287742
3783	cg10571824	cg23052758	cg15910594
3784	cg10580341	cg23024810	cg05678758
3785	cg10583144	cg22974467	cg10799492
3786	cg10584300	cg22957135	cg12734852
3787	cg10585257	cg22944823	cg21211480
3788	cg10588276	cg22888023	cg06927900
3789	cg10589235	cg22861317	cg23906687
3790	cg10589745	cg22764700	cg24586870
3791	cg10590657	cg22752049	cg26463171
3792	cg10601476	cg22584582	cg18566883
3793	cg10604241	cg22562003	cg01501018
3794	cg10607485	cg22466400	cg18565130
3795	cg10607603	cg22422264	cg17264541
3796	cg10611016	cg22379668	cg24809845
3797	cg10617494	cg22173510	cg19271753
3798	cg10617739	cg22083335	cg17021949
3799	cg10623198	cg22054793	cg04175292
3800	cg10632728	cg22047338	cg20977024
3801	cg10634182	cg21897315	cg13422164
3802	cg10639411	cg21887193	cg08257212
3803	cg10642330	cg21870038	cg13706365
3804	cg10644206	cg21863721	cg00713972
3805	cg10664162	cg21750915	cg15241074
3806	cg10664272	cg21719704	cg23939875
3807	cg10664618	cg21593030	cg14122652
3808	cg10667207	cg21552014	cg25887811
3809	cg10673740	cg21230793	cg00472277
3810	cg10676125	cg21089789	cg24696473
3811	cg10681992	cg21069814	cg03098837
3812	cg10689512	cg21017775	cg00216061
3813	cg10691866	cg20828052	cg16563454

3814	cg10692363	cg20771808	cg11828470
3815	cg10705306	cg20600205	cg08894066
3816	cg10713589	cg20476021	cg27406975
3817	cg10714160	cg20438460	cg03528118
3818	cg10714509	cg20336472	cg01723163
3819	cg10716356	cg20158671	cg27076552
3820	cg10721220	cg20069939	cg15627277
3821	cg10722267	cg19901381	cg04088152
3822	cg10724774	cg19745930	cg08932665
3823	cg10726559	cg19736226	cg24845165
3824	cg10730148	cg19714865	cg18885289
3825	cg10731848	cg19621160	cg27599376
3826	cg10734581	cg19618438	cg15127702
3827	cg10737521	cg19604110	cg14431528
3828	cg10738865	cg19567295	cg17439009
3829	cg10740573	cg19528797	cg14413378
3830	cg10754670	cg19514230	cg16617141
3831	cg10756252	cg19503351	cg24525461
3832	cg10759972	cg19415091	cg04524477
3833	cg10762613	cg19288921	cg27643147
3834	cg10768996	cg19048950	cg13731636
3835	cg10774480	cg18885289	cg27078464
3836	cg10780981	cg18819574	cg14065153
3837	cg10786098	cg18809729	cg12817154
3838	cg10790470	cg18798744	cg11843516
3839	cg10796022	cg18749563	cg16052388
3840	cg10796068	cg18677278	cg10504150
3841	cg10798745	cg18595065	cg17036641
3842	cg10799492	cg18352616	cg04311473
3843	cg10802680	cg18323018	cg00103778
3844	cg10805676	cg18233538	cg04254540
3845	cg10810026	cg18177819	cg17497965
3846	cg10810290	cg18035571	cg16949378
3847	cg10811426	cg17860133	cg24630383
3848	cg10811485	cg17838182	cg23203730
3849	cg10813908	cg17780565	cg20847114
3850	cg10818702	cg17732044	cg15175632
3851	cg10820904	cg17537252	cg03307425
3852	cg10824354	cg17517442	cg00436722
3853	cg10827094	cg17415355	cg19909613
3854	cg10827460	cg17361593	cg20300651
3855	cg10828599	cg17254904	cg14064268
3856	cg10829693	cg17211223	cg02362505
3857	cg10844382	cg17169982	cg00767010
3858	cg10856972	cg16976520	cg01959262
3859	cg10857774	cg16965936	cg10227312
3860	cg10859133	cg16892393	cg13707189
3861	cg10859358	cg16738646	cg04352404
3862	cg10862981	cg16717225	cg14140717
3863	cg10872457	cg16642938	cg18069174
3864	cg10884953	cg16525330	cg02806032
3865	cg10886334	cg16499956	cg23079522
3866	cg10888242	cg16366809	cg06387842

3867	cg10890199	cg16106903	cg08945920
3868	cg10893014	cg16034562	cg18940830
3869	cg10897631	cg16023122	cg03234557
3870	cg10898310	cg15980914	cg14132388
3871	cg10899681	cg15892617	cg02901002
3872	cg10902107	cg15878619	cg22616343
3873	cg10902667	cg15776300	cg19450111
3874	cg10904740	cg15756507	cg22946562
3875	cg10904856	cg15693937	cg16684117
3876	cg10907727	cg15532236	cg19847601
3877	cg10917602	cg15518113	cg13411507
3878	cg10921517	cg15498306	cg00374717
3879	cg10921702	cg15407257	cg22013790
3880	cg10931901	cg14947466	cg00881300
3881	cg10937494	cg14919455	cg13499600
3882	cg10950593	cg14839134	cg24875415
3883	cg10959933	cg14583973	cg22150680
3884	cg10963218	cg14543285	cg01866959
3885	cg10965164	cg14475381	cg14916108
3886	cg10965996	cg14468090	cg27180994
3887	cg10968396	cg14455781	cg02704946
3888	cg10976861	cg14304073	cg08412188
3889	cg10977734	cg14297023	cg27092248
3890	cg10979364	cg14281591	cg20320158
3891	cg10980293	cg14270348	cg11468233
3892	cg10980495	cg14244878	cg10876003
3893	cg10982692	cg14149492	cg26609642
3894	cg10982964	cg14118946	cg05884394
3895	cg10989175	cg13947385	cg13236409
3896	cg11007190	cg13943333	cg16960291
3897	cg11009590	cg13885965	cg06087826
3898	cg11010976	cg13798679	cg13956443
3899	cg11018723	cg13753526	cg14681171
3900	cg11024450	cg13629563	cg19528797
3901	cg11025609	cg13586696	cg01696984
3902	cg11025750	cg13441142	cg22861317
3903	cg11027058	cg13414581	cg09558069
3904	cg11028291	cg13360638	cg07475178
3905	cg11030811	cg13332783	cg11490944
3906	cg11035122	cg13299824	cg14190344
3907	cg11036962	cg13274014	cg14852276
3908	cg11037787	cg13269555	cg00845883
3909	cg11041314	cg13151645	cg26601559
3910	cg11043092	cg13107973	cg27344022
3911	cg11046578	cg13068653	cg20579480
3912	cg11046864	cg12925881	cg17868840
3913	cg11051022	cg12847536	cg03739877
3914	cg11060194	cg12747076	cg10102736
3915	cg11069071	cg12659065	cg03701759
3916	cg11079426	cg12528056	cg22539584
3917	cg11080651	cg12508392	cg03163783
3918	cg11081826	cg12491114	cg11313862
3919	cg11084269	cg12452298	cg19514230

3920	cg11087939	cg12429455	cg23385847
3921	cg11092157	cg12279175	cg20713092
3922	cg11095122	cg12176815	cg00552168
3923	cg11101316	cg12156167	cg09604238
3924	cg11103999	cg12142346	cg07811002
3925	cg11108115	cg11973602	cg09464883
3926	cg11115867	cg11794120	cg06967316
3927	cg11116288	cg11706790	cg18583329
3928	cg11124652	cg11699517	cg05057834
3929	cg11139446	cg11425149	cg21613549
3930	cg11148096	cg11272705	cg12412384
3931	cg11150559	cg11093939	cg10019018
3932	cg11151524	cg10982964	cg25719378
3933	cg11152884	cg10968396	cg23522522
3934	cg11155016	cg10937494	cg05516390
3935	cg11161142	cg10844382	cg23470262
3936	cg11174847	cg10824354	cg03779973
3937	cg11176525	cg10768996	cg14210001
3938	cg11189177	cg10716356	cg02155048
3939	cg11190278	cg10504150	cg04774930
3940	cg11190890	cg10208609	cg21185187
3941	cg11197908	cg10171557	cg25142010
3942	cg11198094	cg10126181	cg01277910
3943	cg11199137	cg10088715	cg01088579
3944	cg11199862	cg09889848	cg11007190
3945	cg11203377	cg09875326	cg09647352
3946	cg11205589	cg09851241	cg09795809
3947	cg11207081	cg09649196	cg04907595
3948	cg11210357	cg09646558	cg11719457
3949	cg11215918	cg09596336	cg22215392
3950	cg11218175	cg09579081	cg24989405
3951	cg11225330	cg09554406	cg02936315
3952	cg11227822	cg09519954	cg18817444
3953	cg11231069	cg09444036	cg01260603
3954	cg11234767	cg09391399	cg22619784
3955	cg11241684	cg09335911	cg27492839
3956	cg11248182	cg09262882	cg27074355
3957	cg11251006	cg09152259	cg01436550
3958	cg11252765	cg09123760	cg17574471
3959	cg11254317	cg08945920	cg00916659
3960	cg11258489	cg08914150	cg27053040
3961	cg11263420	cg08904630	cg08124986
3962	cg11272705	cg08863422	cg24518609
3963	cg11272874	cg08831077	cg03980268
3964	cg11275487	cg08784950	cg18294610
3965	cg11284582	cg08740088	cg11199137
3966	cg11285912	cg08727428	cg18484068
3967	cg11288260	cg08682625	cg13680246
3968	cg11307417	cg08644106	cg22885000
3969	cg11311843	cg08566548	cg03655701
3970	cg11313862	cg08552446	cg01475325
3971	cg11321921	cg08365802	cg16290996
3972	cg11327857	cg08365638	cg01753263

3973	cg11341086	cg08365388	cg13251842
3974	cg11343506	cg08258506	cg01676623
3975	cg11344729	cg08137602	cg08345465
3976	cg11346522	cg07833472	cg10886334
3977	cg11351841	cg07805999	cg20784591
3978	cg11363168	cg07662964	cg27082076
3979	cg11372818	cg07503211	cg20963020
3980	cg11381282	cg07495357	cg05755441
3981	cg11390978	cg07477924	cg23408990
3982	cg11391732	cg07336964	cg11671308
3983	cg11400143	cg07285148	cg13439189
3984	cg11400707	cg07268431	cg16051954
3985	cg11401278	cg07236781	cg10263684
3986	cg11405458	cg07192047	cg17877600
3987	cg11409060	cg07156484	cg17680611
3988	cg11415402	cg07155331	cg03431067
3989	cg11425149	cg07097367	cg07079828
3990	cg11436027	cg07094487	cg00903308
3991	cg11438039	cg07034004	cg03661844
3992	cg11439877	cg07014438	cg05339403
3993	cg11444072	cg06938890	cg00707452
3994	cg11449408	cg06889272	cg00892999
3995	cg11457582	cg06824199	cg20158671
3996	cg11459133	cg06797069	cg02638755
3997	cg11462099	cg06591973	cg10495754
3998	cg11469540	cg06539449	cg02389084
3999	cg11476985	cg06368978	cg02598618
4000	cg11482422	cg06237774	cg12855331
4001	cg11490961	cg06225767	cg02122052
4002	cg11492886	cg06218688	cg17518949
4003	cg11496593	cg06173481	cg13558912
4004	cg11497017	cg06159340	cg09972881
4005	cg11512026	cg06125821	cg18624777
4006	cg11517528	cg06115826	cg21611093
4007	cg11519725	cg06058262	cg12049462
4008	cg11523191	cg06037365	cg19548479
4009	cg11523661	cg05996835	cg20609679
4010	cg11524428	cg05845879	cg00854594
4011	cg11526020	cg05825120	cg26709309
4012	cg11530995	cg05781582	cg03980715
4013	cg11535638	cg05700348	cg27171605
4014	cg11538848	cg05668807	cg20703928
4015	cg11544882	cg05559648	cg07262506
4016	cg11546709	cg05376738	cg25116615
4017	cg11547122	cg05368971	cg07148207
4018	cg11553311	cg05339066	cg01598009
4019	cg11564792	cg05309505	cg17977723
4020	cg11567885	cg05088794	cg14062899
4021	cg11575912	cg05041871	cg19883388
4022	cg11576424	cg04955856	cg14701122
4023	cg11576988	cg04911180	cg16650096
4024	cg11583751	cg04885333	cg19047670
4025	cg11585280	cg04800347	cg01778908

4026	cg11585301	cg04780481	cg23144649
4027	cg11588197	cg04623131	cg21183664
4028	cg11589139	cg04604708	cg10009207
4029	cg11592377	cg04585669	cg11497017
4030	cg11592677	cg04534503	cg12087627
4031	cg11601967	cg04398861	cg00358442
4032	cg11609668	cg04360147	cg06919312
4033	cg11612905	cg04352404	cg18761976
4034	cg11628781	cg04328729	cg00147160
4035	cg11628880	cg04262465	cg11940177
4036	cg11631173	cg04226256	cg14207326
4037	cg11632592	cg04175417	cg22599005
4038	cg11637968	cg04088152	cg11401278
4039	cg11646638	cg04080724	cg09809922
4040	cg11650648	cg03970032	cg12113251
4041	cg11654662	cg03918756	cg04411052
4042	cg11661534	cg03899510	cg07941953
4043	cg11662712	cg03881382	cg03101580
4044	cg11668876	cg03789507	cg21201494
4045	cg11671308	cg03778594	cg02711397
4046	cg11671925	cg03660377	cg15710638
4047	cg11672338	cg03640756	cg27275022
4048	cg11685391	cg03606646	cg10370025
4049	cg11691844	cg03515246	cg01943414
4050	cg11695684	cg03457485	cg03631837
4051	cg11697433	cg03254566	cg24738346
4052	cg11697861	cg03131767	cg12389888
4053	cg11698119	cg03129134	cg10235845
4054	cg11699517	cg03077671	cg03400139
4055	cg11700356	cg03050965	cg08377924
4056	cg11700959	cg03047420	cg23332610
4057	cg11703722	cg03035359	cg18222083
4058	cg11703759	cg03023681	cg24628866
4059	cg11706540	cg02940562	cg26744332
4060	cg11706790	cg02855558	cg21316772
4061	cg11717552	cg02710481	cg11873161
4062	cg11718965	cg02638755	cg05088605
4063	cg11719457	cg02625138	cg18090631
4064	cg11724511	cg02607544	cg23736055
4065	cg11725852	cg02552250	cg19593878
4066	cg11728201	cg02495250	cg12866859
4067	cg11731626	cg02358804	cg18386876
4068	cg11743827	cg02339793	cg09342325
4069	cg11753157	cg02338778	cg17799760
4070	cg11758841	cg02228688	cg02567151
4071	cg11761615	cg02192555	cg15083845
4072	cg11762760	cg02172773	cg05484458
4073	cg11777290	cg02137970	cg03379631
4074	cg11786870	cg02108015	cg18095041
4075	cg11792186	cg02078525	cg13089661
4076	cg11794120	cg02041470	cg20161791
4077	cg11802013	cg01997461	cg18741439
4078	cg11804414	cg01991625	cg16385941

4079	cg11806749	cg01942927	cg09037712
4080	cg11809476	cg01794853	cg01792229
4081	cg11815072	cg01618851	cg03380645
4082	cg11815790	cg01570589	cg18201671
4083	cg11816841	cg01422416	cg22582721
4084	cg11818853	cg01323212	cg25875209
4085	cg11819469	cg01298912	cg08768904
4086	cg11822372	cg01262865	cg05539509
4087	cg11828470	cg01227084	cg16797656
4088	cg11828741	cg01176141	cg08463775
4089	cg11828820	cg01101448	cg22197033
4090	cg11834956	cg01062470	cg16762085
4091	cg11835347	cg00927435	cg03031526
4092	cg11840849	cg00904122	cg20139145
4093	cg11843516	cg00903438	cg19702703
4094	cg11843691	cg00899463	cg04555379
4095	cg11843948	cg00786138	cg07907506
4096	cg11844358	cg00767010	cg18240528
4097	cg11844464	cg00755287	cg21755755
4098	cg11847597	cg00520380	cg00314994
4099	cg11848157	cg00473257	cg13298538
4100	cg11850468	cg00472277	cg00828762
4101	cg11852646	cg00367649	cg24244437
4102	cg11854392	cg00333583	cg04627183
4103	cg11860238	cg00330518	cg11009590
4104	cg11864076	cg00086171	cg17475200
4105	cg11865296	cg00044665	cg02116283
4106	cg11869499	cg27526774	cg24648594
4107	cg11873161	cg27478020	cg01254644
4108	cg11888359	cg27395391	cg04041283
4109	cg11888747	cg27344022	cg27007717
4110	cg11894474	cg27333269	cg20078454
4111	cg11897120	cg27306787	cg16745596
4112	cg11898009	cg27277104	cg16488544
4113	cg11908294	cg27186013	cg04585669
4114	cg11909137	cg27158462	cg06378490
4115	cg11909989	cg27156542	cg21159068
4116	cg11912239	cg26970113	cg13139630
4117	cg11915444	cg26951000	cg00001809
4118	cg11919577	cg26905281	cg27333269
4119	cg11929154	cg26721908	cg20767977
4120	cg11935248	cg26677892	cg17850498
4121	cg11936643	cg26585452	cg09511662
4122	cg11936817	cg26460678	cg22315164
4123	cg11940177	cg26448609	cg17284725
4124	cg11940726	cg26421308	cg09123760
4125	cg11945095	cg26394825	cg05516020
4126	cg11945474	cg26392980	cg08924374
4127	cg11951169	cg26261873	cg07349899
4128	cg11952493	cg26165908	cg07468956
4129	cg11952839	cg26034516	cg09139451
4130	cg11952908	cg26011438	cg19931596
4131	cg11958644	cg26001655	cg11697433

4132	cg11958668	cg25963540	cg05280797
4133	cg11960033	cg25945499	cg11254847
4134	cg11961845	cg25852715	cg06379876
4135	cg11964216	cg25835936	cg13753460
4136	cg11966063	cg25729585	cg18091245
4137	cg11971852	cg25638714	cg10982692
4138	cg11973602	cg25634000	cg08510718
4139	cg11976592	cg25596405	cg02021485
4140	cg11977716	cg25532627	cg04658841
4141	cg11979837	cg25488990	cg24271718
4142	cg11980500	cg25296804	cg16554534
4143	cg11982525	cg25268422	cg12136716
4144	cg11990296	cg25118879	cg25827936
4145	cg11994639	cg25033220	cg09725874
4146	cg11995490	cg25019378	cg07337025
4147	cg11996592	cg24976262	cg14841828
4148	cg11997603	cg24970620	cg15035360
4149	cg11998932	cg24960639	cg07545858
4150	cg12005412	cg24939470	cg26872742
4151	cg12017558	cg24732123	cg06815308
4152	cg12019806	cg24724937	cg03384915
4153	cg12019990	cg24437408	cg23362032
4154	cg12025243	cg24386905	cg12708994
4155	cg12036877	cg24319076	cg13618969
4156	cg12049462	cg24244437	cg16649474
4157	cg12051710	cg24191821	cg26503877
4158	cg12054698	cg24186506	cg06566615
4159	cg12058043	cg24107411	cg12819011
4160	cg12058875	cg24008544	cg14790609
4161	cg12061219	cg24003508	cg20668718
4162	cg12062088	cg23881119	cg21667939
4163	cg12064373	cg23834688	cg19441674
4164	cg12068791	cg23783444	cg00308508
4165	cg12069132	cg23626961	cg27351813
4166	cg12069547	cg23510131	cg19486804
4167	cg12070646	cg23490822	cg04944177
4168	cg12071544	cg23413464	cg15318570
4169	cg12072024	cg23401741	cg02524863
4170	cg12072973	cg23386779	cg20141509
4171	cg12075498	cg23202053	cg22192454
4172	cg12075928	cg23003961	cg15879620
4173	cg12076543	cg22992258	cg05134769
4174	cg12077685	cg22920258	cg05037556
4175	cg12079279	cg22814801	cg04780481
4176	cg12081291	cg22788065	cg11695684
4177	cg12087627	cg22622505	cg27571590
4178	cg12092201	cg22532194	cg00091004
4179	cg12093220	cg22459052	cg16794682
4180	cg12093620	cg22356839	cg26127166
4181	cg12106976	cg22169866	cg25286715
4182	cg12108265	cg22123915	cg09555736
4183	cg12109728	cg22011888	cg11251006
4184	cg12112434	cg21597937	cg02067239

4185	cg12119133	cg21546226	cg03600605
4186	cg12120430	cg21535761	cg27170206
4187	cg12121782	cg21504064	cg19771975
4188	cg12129983	cg21379004	cg10654373
4189	cg12136716	cg21279756	cg05777316
4190	cg12141218	cg21228259	cg00175895
4191	cg12142346	cg21045072	cg23951305
4192	cg12143499	cg20952105	cg06937201
4193	cg12149319	cg20826610	cg04500730
4194	cg12153709	cg20808227	cg17589890
4195	cg12156167	cg20668718	cg16704938
4196	cg12160664	cg20397614	cg19040077
4197	cg12161625	cg20349803	cg15026277
4198	cg12163781	cg20190649	cg07083272
4199	cg12163952	cg20173014	cg26467269
4200	cg12166802	cg20137441	cg09038962
4201	cg12167489	cg19998675	cg09118048
4202	cg12176815	cg19987219	cg25722029
4203	cg12179884	cg19897003	cg09353705
4204	cg12183501	cg19713140	cg01537847
4205	cg12185975	cg19525780	cg00757262
4206	cg12186219	cg19418951	cg13397568
4207	cg12195230	cg19287349	cg19083914
4208	cg12204166	cg19241089	cg01679473
4209	cg12219134	cg19170881	cg21562750
4210	cg12220376	cg19123356	cg27130493
4211	cg12231088	cg19055390	cg08933276
4212	cg12232388	cg18919478	cg05851887
4213	cg12233100	cg18855096	cg08912841
4214	cg12240637	cg18829162	cg04714939
4215	cg12240761	cg18820914	cg07129769
4216	cg12250561	cg18817459	cg07162085
4217	cg12251803	cg18793661	cg17601658
4218	cg12265604	cg18549386	cg17537493
4219	cg12267069	cg18386876	cg22463097
4220	cg12271433	cg18348566	cg02264288
4221	cg12279175	cg17822706	cg20100049
4222	cg12284521	cg17702736	cg12064373
4223	cg12302797	cg17477806	cg01722498
4224	cg12308279	cg17343483	cg14117392
4225	cg12312338	cg17243540	cg01683570
4226	cg12339425	cg17226348	cg11628880
4227	cg12343881	cg17201542	cg12077685
4228	cg12351906	cg17185710	cg00373967
4229	cg12352896	cg16902190	cg02898721
4230	cg12362980	cg16867086	cg24990238
4231	cg12367539	cg16741602	cg13745832
4232	cg12367789	cg16723180	cg10885338
4233	cg12371263	cg16707423	cg13957538
4234	cg12371563	cg16461139	cg25711239
4235	cg12379145	cg16387467	cg18378662
4236	cg12384918	cg16328007	cg17781958
4237	cg12389888	cg16326073	cg06790069

4238	cg12392429	cg16269144	cg07097367
4239	cg12395479	cg16221927	cg01972651
4240	cg12397802	cg16168321	cg22909085
4241	cg12412384	cg16163756	cg06386880
4242	cg12413156	cg16163535	cg01297744
4243	cg12416830	cg16102778	cg14162011
4244	cg12417362	cg16060382	cg06647026
4245	cg12417815	cg16030972	cg06632214
4246	cg12417871	cg16006963	cg18318722
4247	cg12419932	cg15710638	cg26225655
4248	cg12422551	cg15617847	cg01038640
4249	cg12429455	cg15382580	cg07014438
4250	cg12433926	cg15269148	cg17221945
4251	cg12442385	cg15241074	cg07336964
4252	cg12448539	cg14884828	cg20102955
4253	cg12448664	cg14830485	cg09300185
4254	cg12450391	cg14769121	cg26162025
4255	cg12452298	cg14763173	cg09896867
4256	cg12456379	cg14758812	cg09465065
4257	cg12459932	cg14758740	cg26411080
4258	cg12467090	cg14744022	cg15988204
4259	cg12467435	cg14699728	cg13700939
4260	cg12467960	cg14681171	cg20731213
4261	cg12468255	cg14524975	cg16572410
4262	cg12469964	cg14497697	cg21282907
4263	cg12474695	cg14476630	cg14966074
4264	cg12481567	cg14328761	cg18013789
4265	cg12485685	cg14313184	cg09444036
4266	cg12486287	cg14136781	cg08993878
4267	cg12486498	cg14132388	cg15904939
4268	cg12491114	cg14126884	cg01778994
4269	cg12491594	cg14102880	cg04727924
4270	cg12494355	cg14092259	cg17112949
4271	cg12497581	cg14070108	cg26763380
4272	cg12499572	cg14001914	cg12702354
4273	cg12500891	cg13971603	cg24321688
4274	cg12508392	cg13919369	cg15817440
4275	cg12511749	cg13916835	cg06123891
4276	cg12514933	cg13856126	cg09333143
4277	cg12522144	cg13849999	cg10472711
4278	cg12526346	cg13747876	cg07152894
4279	cg12526923	cg13727957	cg05224498
4280	cg12528056	cg13721644	cg26100137
4281	cg12529228	cg13571319	cg27020028
4282	cg12550496	cg13499600	cg08712631
4283	cg12551582	cg13411507	cg14875081
4284	cg12552626	cg13332142	cg10740573
4285	cg12555086	cg13048437	cg17322118
4286	cg12569216	cg13028113	cg00808555
4287	cg12579684	cg12955277	cg07858195
4288	cg12583325	cg12467435	cg06787609
4289	cg12590668	cg12448539	cg25521853
4290	cg12591125	cg12422551	cg00554604

4291	cg12591689	cg12302797	cg08703151
4292	cg12595461	cg12081291	cg22133366
4293	cg12598178	cg12068791	cg04745820
4294	cg12599569	cg12058875	cg13545717
4295	cg12604490	cg12058043	cg12156838
4296	cg12609948	cg11964216	cg13520715
4297	cg12614090	cg11919577	cg08258506
4298	cg12616177	cg11861709	cg21528710
4299	cg12629008	cg11806749	cg15063366
4300	cg12640387	cg11722816	cg23053961
4301	cg12642717	cg11718965	cg09358973
4302	cg12650685	cg11714334	cg00147216
4303	cg12657297	cg11703722	cg27160524
4304	cg12657361	cg11691844	cg24244854
4305	cg12659065	cg11668876	cg19865472
4306	cg12667792	cg11190278	cg08694923
4307	cg12669271	cg11161142	cg08043592
4308	cg12678686	cg11152884	cg04944536
4309	cg12680106	cg10888242	cg11703722
4310	cg12681370	cg10722267	cg25591418
4311	cg12681784	cg10667102	cg22644984
4312	cg12686055	cg10457079	cg26784300
4313	cg12688942	cg10453420	cg25661792
4314	cg12692386	cg10425513	cg05188868
4315	cg12692727	cg10333594	cg13910681
4316	cg12694261	cg10194844	cg26607528
4317	cg12702354	cg10055950	cg03049782
4318	cg12705212	cg10037894	cg21450654
4319	cg12708994	cg09976716	cg03571927
4320	cg12710648	cg09906780	cg16580681
4321	cg12725520	cg09869286	cg15126957
4322	cg12727138	cg09849774	cg02625138
4323	cg12727940	cg09837656	cg19603100
4324	cg12728588	cg09824023	cg06198069
4325	cg12728606	cg09688726	cg07486170
4326	cg12729518	cg09675604	cg13038544
4327	cg12732734	cg09641077	cg09326135
4328	cg12734852	cg09537038	cg17649815
4329	cg12736760	cg09259595	cg06926306
4330	cg12737285	cg09174690	cg00969565
4331	cg12742209	cg09172973	cg23709782
4332	cg12743638	cg09163745	cg05498905
4333	cg12744447	cg09163478	cg26886188
4334	cg12747076	cg09040552	cg13039539
4335	cg12762413	cg09004351	cg19144954
4336	cg12764441	cg08985282	cg07503211
4337	cg12766383	cg08924374	cg26367275
4338	cg12774921	cg08869160	cg03315432
4339	cg12778183	cg08771923	cg23908771
4340	cg12785228	cg08694923	cg25418363
4341	cg12786452	cg08619515	cg10805676
4342	cg12787036	cg08591538	cg06313479
4343	cg12790592	cg08523978	cg20173014

4344	cg12796332	cg08510718	cg23632333
4345	cg12797609	cg08506026	cg13810664
4346	cg12799862	cg08384155	cg09996971
4347	cg12806353	cg08334780	cg04910179
4348	cg12806728	cg07925587	cg14715030
4349	cg12817154	cg07733800	cg04195543
4350	cg12817352	cg07431229	cg01872216
4351	cg12819011	cg07394799	cg15892617
4352	cg12822242	cg07265549	cg02855309
4353	cg12832282	cg07261940	cg27522078
4354	cg12832565	cg07146723	cg11069071
4355	cg12832726	cg07129714	cg01801191
4356	cg12835599	cg07109788	cg02780210
4357	cg12842409	cg07104086	cg00484122
4358	cg12847536	cg07097374	cg00134602
4359	cg12855331	cg07051221	cg11135937
4360	cg12856760	cg07046047	cg22022181
4361	cg12860109	cg07012725	cg00750074
4362	cg12865939	cg06984255	cg26765599
4363	cg12866104	cg06914693	cg26105283
4364	cg12866859	cg06786153	cg03058664
4365	cg12887832	cg06754496	cg17176209
4366	cg12900489	cg06753918	cg07498879
4367	cg12901038	cg06741896	cg03765423
4368	cg12901650	cg06656553	cg25045972
4369	cg12905673	cg06547766	cg03063946
4370	cg12914733	cg06518233	cg18817459
4371	cg12921473	cg06506523	cg13799838
4372	cg12927785	cg06485166	cg03434029
4373	cg12936779	cg06422947	cg16383573
4374	cg12938917	cg06372475	cg18142262
4375	cg12944530	cg06208229	cg24100115
4376	cg12948116	cg06096901	cg12210305
4377	cg12948543	cg05978010	cg09920043
4378	cg12955277	cg05945030	cg27469606
4379	cg12964187	cg05724271	cg00950038
4380	cg12971523	cg05658807	cg09371084
4381	cg12987059	cg05591701	cg14553765
4382	cg12995933	cg05498905	cg11717552
4383	cg12999366	cg05369142	cg17367836
4384	cg13008252	cg05330056	cg14156441
4385	cg13028113	cg05303899	cg13215078
4386	cg13029400	cg05286252	cg18689614
4387	cg13036546	cg05274056	cg06390613
4388	cg13038544	cg05229528	cg18568570
4389	cg13039539	cg05218696	cg18018990
4390	cg13048437	cg05179396	cg08929612
4391	cg13050716	cg05069728	cg05112986
4392	cg13054419	cg05065603	cg02588098
4393	cg13054523	cg05057834	cg19382919
4394	cg13055385	cg05040210	cg17550566
4395	cg13058457	cg04929165	cg18857588
4396	cg13061648	cg04902542	cg07205670

4397	cg13063335	cg04854911	cg14470121
4398	cg13066703	cg04629141	cg00366037
4399	cg13068653	cg04607323	cg18532070
4400	cg13070650	cg04568492	cg05384450
4401	cg13079633	cg04273604	cg08843756
4402	cg13080151	cg04254540	cg04216070
4403	cg13086402	cg04186815	cg14129169
4404	cg13089599	cg04003615	cg22957135
4405	cg13089661	cg03789088	cg11400707
4406	cg13093285	cg03666500	cg07850832
4407	cg13096278	cg03640215	cg02212339
4408	cg13099139	cg03591954	cg26016985
4409	cg13099829	cg03550233	cg18862502
4410	cg13105904	cg03545972	cg01283625
4411	cg13107973	cg03515290	cg20256649
4412	cg13109865	cg03498175	cg21026566
4413	cg13115424	cg03307425	cg04536844
4414	cg13116061	cg03091010	cg26891661
4415	cg13119578	cg03082830	cg18226382
4416	cg13129380	cg03077492	cg14331609
4417	cg13135376	cg03068497	cg04679114
4418	cg13139630	cg02998240	cg09406921
4419	cg13148544	cg02926160	cg16642938
4420	cg13149996	cg02873783	cg05196820
4421	cg13151645	cg02844593	cg04885333
4422	cg13153540	cg02825527	cg00934066
4423	cg13153796	cg02800817	cg06898502
4424	cg13154622	cg02573234	cg09790512
4425	cg13162609	cg02570643	cg21406967
4426	cg13165390	cg02563156	cg22490722
4427	cg13165992	cg02486181	cg06036236
4428	cg13169796	cg02461114	cg07875385
4429	cg13171107	cg02363969	cg14011387
4430	cg13171643	cg02250553	cg01618851
4431	cg13175060	cg02138124	cg23873200
4432	cg13177873	cg02083559	cg07506407
4433	cg13189671	cg02066343	cg24276624
4434	cg13190879	cg01953240	cg16776331
4435	cg13200640	cg01870976	cg09258509
4436	cg13206932	cg01828733	cg03970032
4437	cg13207250	cg01667978	cg25588387
4438	cg13210470	cg01558195	cg22686523
4439	cg13210578	cg01553866	cg10333594
4440	cg13222945	cg01543307	cg16920538
4441	cg13223682	cg01443467	cg27073349
4442	cg13225881	cg01369033	cg08883485
4443	cg13236409	cg01306662	cg06436667
4444	cg13248406	cg01274028	cg15720017
4445	cg13251842	cg01094351	cg17505642
4446	cg13263472	cg01088579	cg23114772
4447	cg13264394	cg00989505	cg03589898
4448	cg13269555	cg00938266	cg14050395
4449	cg13274014	cg00934066	cg23844904

4450	cg13278004	cg00892999	cg03882967
4451	cg13278105	cg00718831	cg26434328
4452	cg13282594	cg00713253	cg01069043
4453	cg13297120	cg00606396	cg13701991
4454	cg13298379	cg00604356	cg04529785
4455	cg13319468	cg00595472	cg20785796
4456	cg13320257	cg00442220	cg01930924
4457	cg13332142	cg00405824	cg08325885
4458	cg13348246	cg00360324	cg04847478
4459	cg13352836	cg00299943	cg18253910
4460	cg13358061	cg00275503	cg26426564
4461	cg13360638	cg00236766	cg04030444
4462	cg13369099	cg00149708	cg13972711
4463	cg13374528	cg00147216	cg23989757
4464	cg13377839	cg00017461	cg00757391
4465	cg13378789	cg27641072	cg25086418
4466	cg13378934	cg27631766	cg19713140
4467	cg13381243	cg27554156	cg00676360
4468	cg13383819	cg27511289	cg13685294
4469	cg13390372	cg27485845	cg16288579
4470	cg13393917	cg27470978	cg21585138
4471	cg13397568	cg27294008	cg10738865
4472	cg13400018	cg27228210	cg21031345
4473	cg13402847	cg27227317	cg09489567
4474	cg13408795	cg27025137	cg27540799
4475	cg13409409	cg27004760	cg03785456
4476	cg13411951	cg26974441	cg03316628
4477	cg13420004	cg26769720	cg22436253
4478	cg13420647	cg26765599	cg04919234
4479	cg13422164	cg26757820	cg13378934
4480	cg13425135	cg26690672	cg03050096
4481	cg13429555	cg26661623	cg18259342
4482	cg13433644	cg26510473	cg21945949
4483	cg13434216	cg26499286	cg05730150
4484	cg13441142	cg26463171	cg13620881
4485	cg13444374	cg26426334	cg13980079
4486	cg13446070	cg26328589	cg26529655
4487	cg13447099	cg26309261	cg14473030
4488	cg13447933	cg26289871	cg09163745
4489	cg13453203	cg26281621	cg19769521
4490	cg13455439	cg26181840	cg13904806
4491	cg13456321	cg26171489	cg09391399
4492	cg13457217	cg26164879	cg25114693
4493	cg13458561	cg26147269	cg00147850
4494	cg13461247	cg25998584	cg13927454
4495	cg13461718	cg25984524	cg07795082
4496	cg13464157	cg25887811	cg23755969
4497	cg13467292	cg25841625	cg22908423
4498	cg13470557	cg25785495	cg00872170
4499	cg13471336	cg25780982	cg07876450
4500	cg13472900	cg25763426	cg10208609
4501	cg13473184	cg25754933	cg05736906
4502	cg13475822	cg25730564	cg27228210

4503	cg13476204	cg25650076	cg23300494
4504	cg13477354	cg25600823	cg27134342
4505	cg13483248	cg25567021	cg13062396
4506	cg13488013	cg25559625	cg27009448
4507	cg13488220	cg25236277	cg08091706
4508	cg13488851	cg25161092	cg15422033
4509	cg13490227	cg25154482	cg00755287
4510	cg13491731	cg25122824	cg01617074
4511	cg13495850	cg25114693	cg16144562
4512	cg13496979	cg25011577	cg20808227
4513	cg13499600	cg25010788	cg03800922
4514	cg13499923	cg24998879	cg26796443
4515	cg13500797	cg24928546	cg16004738
4516	cg13508949	cg24902250	cg15012161
4517	cg13509456	cg24844449	cg13369099
4518	cg13514824	cg24825937	cg14092259
4519	cg13515351	cg24730224	cg00454770
4520	cg13520715	cg24728456	cg00624949
4521	cg13521002	cg24628866	cg15656769
4522	cg13521018	cg24429081	cg13915179
4523	cg13522118	cg24407065	cg24407065
4524	cg13525067	cg24403487	cg26737223
4525	cg13530474	cg24118850	cg03840920
4526	cg13533142	cg24044501	cg22516162
4527	cg13537940	cg23834427	cg05327192
4528	cg13542073	cg23780635	cg15980914
4529	cg13543915	cg23755969	cg18766468
4530	cg13545717	cg23726802	cg02604211
4531	cg13549638	cg23632333	cg06746774
4532	cg13550401	cg23466118	cg24940138
4533	cg13554136	cg23464284	cg14529170
4534	cg13556624	cg23439917	cg19219672
4535	cg13558912	cg23372092	cg19057916
4536	cg13560612	cg23280294	cg14524553
4537	cg13565718	cg23192604	cg10563645
4538	cg13567403	cg23171099	cg16772023
4539	cg13571319	cg23127694	cg26608486
4540	cg13572069	cg23108580	cg03010561
4541	cg13573036	cg23053961	cg25077823
4542	cg13576994	cg23046918	cg04208466
4543	cg13583088	cg22916722	cg01968002
4544	cg13584383	cg22845855	cg06292891
4545	cg13586038	cg22819824	cg00488575
4546	cg13586696	cg22799860	cg17971649
4547	cg13587802	cg22748815	cg13169796
4548	cg13589431	cg22679474	cg21189146
4549	cg13593436	cg22546818	cg03485217
4550	cg13598881	cg22539584	cg06415302
4551	cg13601605	cg22451412	cg12743638
4552	cg13608684	cg22442617	cg19730379
4553	cg13610622	cg22323067	cg02097309
4554	cg13611006	cg22305797	cg11592677
4555	cg13618372	cg22222413	cg20560906

4556	cg13618880	cg22153312	cg18888464
4557	cg13618969	cg22122013	cg25221919
4558	cg13626676	cg22078934	cg07077978
4559	cg13629563	cg22052672	cg21176488
4560	cg13630560	cg22049753	cg06584121
4561	cg13630646	cg22016731	cg27491887
4562	cg13634090	cg21962953	cg19532212
4563	cg13636986	cg21962025	cg23820560
4564	cg13637552	cg21759907	cg10352104
4565	cg13639244	cg21747549	cg03229767
4566	cg13639866	cg21667939	cg05330056
4567	cg13642149	cg21616243	cg13565718
4568	cg13642849	cg21437521	cg10759972
4569	cg13643040	cg21385606	cg13846358
4570	cg13653963	cg21183664	cg10223066
4571	cg13655570	cg21057429	cg24297835
4572	cg13657004	cg21040069	cg14204266
4573	cg13657511	cg20849855	cg13608684
4574	cg13657981	cg20805475	cg21618273
4575	cg13658093	cg20798249	cg07068045
4576	cg13662225	cg20667709	cg03029255
4577	cg13662628	cg20650515	cg02633600
4578	cg13663738	cg20593887	cg00392155
4579	cg13668823	cg20547131	cg14865516
4580	cg13672348	cg20544406	cg21015266
4581	cg13675389	cg20528338	cg15975598
4582	cg13676538	cg20519665	cg02613380
4583	cg13680188	cg20513080	cg11088489
4584	cg13683219	cg20370991	cg05073386
4585	cg13685294	cg20300651	cg24517989
4586	cg13691622	cg20099458	cg05223346
4587	cg13694343	cg20060759	cg14610776
4588	cg13700912	cg19789753	cg21400851
4589	cg13706365	cg19784198	cg01870865
4590	cg13707189	cg19750606	cg26214074
4591	cg13708635	cg19702383	cg11848157
4592	cg13708908	cg19646759	cg09078622
4593	cg13710553	cg19640166	cg14095283
4594	cg13716849	cg19638572	cg17825194
4595	cg13721589	cg19577054	cg02607544
4596	cg13721644	cg19553402	cg27533454
4597	cg13724379	cg19549465	cg04904609
4598	cg13724812	cg19532212	cg01350190
4599	cg13726456	cg19375196	cg25492112
4600	cg13727957	cg19372504	cg14023774
4601	cg13728069	cg19354017	cg14644865
4602	cg13728131	cg19284767	cg06895946
4603	cg13730600	cg19182279	cg09861034
4604	cg13731108	cg18940830	cg00092644
4605	cg13731636	cg18856214	cg05390712
4606	cg13734860	cg18758281	cg00042325
4607	cg13736131	cg18703721	cg23128056
4608	cg13744954	cg18698681	cg01835489

4609	cg13747145	cg18690282	cg17468543
4610	cg13747876	cg18302629	cg03140766
4611	cg13753460	cg18252102	cg26551200
4612	cg13753526	cg18207099	cg24773418
4613	cg13758724	cg18048309	cg15516314
4614	cg13763232	cg18034859	cg00352652
4615	cg13763482	cg18011099	cg02015219
4616	cg13765417	cg17854297	cg20745684
4617	cg13774184	cg17849972	cg15170442
4618	cg13774342	cg17842918	cg03101492
4619	cg13775299	cg17836842	cg20019410
4620	cg13782615	cg17793608	cg18153137
4621	cg13784276	cg17470627	cg00681103
4622	cg13786171	cg17434577	cg08636203
4623	cg13793145	cg17385318	cg12729518
4624	cg13794839	cg17329004	cg00869989
4625	cg13798270	cg17285883	cg17099569
4626	cg13798679	cg17181336	cg05631399
4627	cg13799838	cg17027476	cg13589431
4628	cg13800349	cg17017404	cg18763720
4629	cg13808180	cg16936094	cg02633363
4630	cg13808641	cg16885557	cg07051221
4631	cg13808936	cg16812626	cg19078853
4632	cg13810664	cg16582732	cg20048529
4633	cg13815311	cg16550656	cg08007899
4634	cg13817905	cg16474791	cg13888853
4635	cg13818316	cg16465939	cg17429870
4636	cg13821008	cg16446824	cg16074271
4637	cg13821287	cg16411103	cg23945793
4638	cg13823144	cg16395892	cg00861010
4639	cg13827597	cg16391347	cg10511902
4640	cg13832457	cg16359657	cg17241937
4641	cg13836318	cg16204524	cg21492137
4642	cg13838713	cg16151538	cg12250561
4643	cg13842262	cg16139912	cg15373257
4644	cg13849495	cg16077055	cg12072024
4645	cg13849999	cg16004738	cg20441046
4646	cg13850871	cg15974867	cg13784276
4647	cg13852730	cg15862380	cg10119288
4648	cg13856126	cg15834460	cg01228193
4649	cg13857210	cg15587041	cg23719367
4650	cg13857933	cg15577180	cg05692420
4651	cg13858803	cg15532942	cg22638977
4652	cg13860981	cg15424477	cg17853850
4653	cg13864546	cg15422033	cg11929154
4654	cg13867915	cg15346286	cg10311315
4655	cg13871900	cg15275017	cg13063335
4656	cg13877452	cg15230883	cg12469964
4657	cg13877974	cg15161854	cg02003612
4658	cg13881619	cg15065169	cg18027683
4659	cg13885965	cg15028507	cg22433862
4660	cg13888853	cg15022671	cg25595641
4661	cg13904806	cg14920846	cg26564172

4662	cg13908988	cg14781242	cg03286774
4663	cg13910681	cg14780990	cg07464578
4664	cg13915179	cg14764357	cg00290626
4665	cg13916835	cg14734994	cg16106903
4666	cg13916961	cg14584535	cg25134701
4667	cg13919369	cg14574066	cg24064506
4668	cg13920768	cg14565408	cg23711760
4669	cg13924326	cg14547067	cg16030972
4670	cg13927454	cg14529170	cg24723883
4671	cg13931531	cg14414154	cg19399165
4672	cg13933043	cg14342817	cg27177296
4673	cg13936383	cg14328115	cg20967819
4674	cg13940218	cg14313576	cg01239666
4675	cg13943333	cg14311523	cg05682319
4676	cg13947385	cg14290705	cg10623198
4677	cg13948857	cg14162011	cg14480449
4678	cg13948956	cg14096074	cg25815219
4679	cg13953458	cg14050395	cg19313353
4680	cg13956443	cg14044465	cg22083335
4681	cg13957538	cg14037937	cg02462933
4682	cg13962153	cg14037250	cg06617528
4683	cg13964068	cg14021880	cg17208278
4684	cg13972711	cg13948857	cg07027613
4685	cg13973594	cg13936383	cg01004063
4686	cg13977320	cg13794839	cg10917602
4687	cg13980079	cg13761321	cg26171489
4688	cg13981325	cg13680188	cg12799862
4689	cg13982956	cg13676538	cg08823975
4690	cg13991631	cg13637552	cg14100888
4691	cg13994897	cg13624631	cg19901381
4692	cg13996750	cg13550401	cg26668872
4693	cg14001518	cg13431342	cg22788065
4694	cg14001914	cg13428516	cg16269144
4695	cg14003211	cg13200640	cg11589139
4696	cg14005246	cg12999366	cg27336068
4697	cg14010305	cg12936779	cg04873169
4698	cg14010805	cg12865939	cg17670999
4699	cg14011387	cg12856760	cg18833140
4700	cg14016236	cg12835599	cg13515351
4701	cg14016879	cg12785228	cg23989207
4702	cg14021880	cg12725520	cg05202424
4703	cg14023774	cg12694261	cg24787929
4704	cg14024328	cg12669271	cg22762745
4705	cg14025149	cg12657297	cg13170807
4706	cg14030593	cg12579684	cg08741843
4707	cg14035238	cg12551582	cg07937803
4708	cg14037250	cg12397802	cg06546521
4709	cg14037652	cg12351906	cg04360519
4710	cg14037937	cg12232388	cg27579131
4711	cg14041194	cg12178980	cg05846044
4712	cg14044465	cg12153709	cg15813460
4713	cg14050395	cg11952493	cg11706790
4714	cg14051367	cg11873161	cg05303899

4715	cg14055379	cg11717552	cg23262213
4716	cg14058027	cg11628880	cg25242498
4717	cg14059665	cg11547122	cg15895197
4718	cg14059835	cg11517528	cg27483571
4719	cg14062119	cg11490961	cg11030811
4720	cg14062899	cg11459133	cg02998075
4721	cg14064268	cg11285912	cg06899970
4722	cg14070108	cg11258489	cg04349985
4723	cg14074351	cg11199862	cg12598178
4724	cg14078730	cg11037787	cg19418951
4725	cg14082061	cg10899681	cg02150714
4726	cg14087401	cg10890199	cg21080655
4727	cg14088181	cg10812717	cg23250795
4728	cg14092259	cg10762613	cg11231069
4729	cg14093018	cg10714160	cg03627931
4730	cg14093663	cg10617739	cg05409945
4731	cg14096042	cg10580341	cg10937494
4732	cg14100888	cg10518527	cg09198360
4733	cg14101193	cg10512408	cg25028855
4734	cg14101687	cg10498921	cg14329059
4735	cg14102880	cg10439859	cg09255505
4736	cg14104252	cg10335736	cg22247495
4737	cg14113353	cg10278394	cg20826610
4738	cg14114546	cg10248100	cg06931444
4739	cg14117392	cg10227280	cg08970648
4740	cg14118424	cg10102736	cg14645880
4741	cg14118946	cg10063407	cg10960375
4742	cg14119680	cg10023624	cg07295362
4743	cg14125542	cg10009207	cg14304073
4744	cg14126884	cg09911521	cg16989380
4745	cg14127396	cg09896120	cg09519954
4746	cg14132388	cg09890339	cg24528693
4747	cg14136214	cg09868556	cg00373245
4748	cg14136781	cg09796786	cg08613794
4749	cg14140717	cg09745791	cg21105175
4750	cg14143441	cg09674500	cg26648465
4751	cg14144513	cg09526129	cg09228051
4752	cg14149492	cg09473315	cg24900927
4753	cg14156441	cg09437283	cg22920258
4754	cg14156650	cg09267483	cg18482303
4755	cg14159036	cg09126859	cg09248660
4756	cg14162011	cg09118048	cg08767627
4757	cg14165633	cg09115960	cg09407273
4758	cg14166377	cg09100373	cg26423824
4759	cg14168733	cg09078622	cg10218733
4760	cg14175330	cg09029046	cg24893073
4761	cg14178043	cg09018739	cg10472263
4762	cg14178294	cg08970011	cg03379681
4763	cg14189391	cg08910524	cg22020227
4764	cg14190128	cg08860136	cg06115826
4765	cg14190344	cg08749443	cg13309415
4766	cg14195992	cg08687995	cg12011299
4767	cg14199827	cg08544606	cg13639244

4768	cg14201387	cg08409074	cg04186815
4769	cg14201946	cg08259083	cg27396760
4770	cg14202146	cg08097359	cg07178379
4771	cg14204738	cg08038054	cg24220178
4772	cg14206983	cg07899389	cg15504666
4773	cg14208133	cg07866001	cg10827094
4774	cg14210001	cg07828612	cg14227460
4775	cg14210790	cg07741192	cg25555068
4776	cg14213430	cg07536018	cg14149492
4777	cg14217074	cg07483266	cg04968127
4778	cg14218838	cg07475178	cg21730805
4779	cg14219236	cg07463059	cg04015687
4780	cg14227418	cg07438999	cg14715383
4781	cg14227460	cg07416344	cg01483459
4782	cg14228788	cg07284476	cg12129983
4783	cg14234732	cg07283859	cg07146723
4784	cg14239346	cg07165066	cg13639866
4785	cg14242936	cg07015803	cg27410136
4786	cg14244360	cg06924233	cg20918463
4787	cg14244878	cg06846976	cg09982918
4788	cg14249876	cg06840243	cg16998564
4789	cg14260162	cg06719042	cg04838832
4790	cg14262433	cg06671298	cg24059075
4791	cg14264773	cg06616905	cg20776543
4792	cg14264901	cg06604690	cg00512280
4793	cg14266952	cg06599914	cg00949635
4794	cg14272573	cg06584121	cg14602839
4795	cg14274896	cg06436667	cg10508317
4796	cg14281591	cg06419732	cg04479713
4797	cg14282937	cg06385449	cg24092282
4798	cg14285365	cg06379876	cg12569216
4799	cg14286546	cg06344576	cg14337655
4800	cg14287742	cg06297857	cg15033036
4801	cg14290705	cg06186808	cg04353106
4802	cg14294646	cg06124434	cg08565675
4803	cg14297546	cg06104859	cg14462432
4804	cg14304073	cg06008803	cg10898310
4805	cg14311523	cg05988219	cg27470978
4806	cg14312334	cg05970317	cg09276451
4807	cg14313184	cg05821186	cg13537008
4808	cg14313576	cg05767015	cg03798162
4809	cg14317513	cg05760053	cg15891850
4810	cg14321412	cg05603553	cg13129380
4811	cg14321522	cg05585379	cg17226348
4812	cg14323199	cg05554592	cg21304766
4813	cg14325029	cg05400196	cg17348667
4814	cg14326263	cg05339403	cg09569347
4815	cg14326472	cg05322217	cg16265707
4816	cg14328115	cg05317605	cg04930596
4817	cg14328761	cg05260236	cg21593030
4818	cg14329059	cg05223346	cg14455781
4819	cg14329833	cg05139219	cg05056653
4820	cg14330078	cg05134769	cg22731981

4821	cg14331206	cg05060686	cg07481273
4822	cg14331609	cg05051734	cg04990420
4823	cg14334310	cg04941831	cg12161625
4824	cg14337655	cg04815334	cg19594745
4825	cg14337844	cg04806664	cg25552705
4826	cg14340089	cg04804094	cg21397287
4827	cg14342817	cg04678901	cg10005273
4828	cg14350114	cg04668631	cg20147164
4829	cg14351526	cg04591732	cg05750323
4830	cg14359798	cg04462561	cg20476021
4831	cg14373988	cg04457069	cg08362308
4832	cg14377596	cg04403423	cg03851401
4833	cg14389425	cg04269351	cg26240231
4834	cg14393609	cg04249706	cg00206063
4835	cg14401746	cg04217556	cg25838968
4836	cg14403241	cg04145522	cg24705818
4837	cg14405528	cg04132177	cg02493905
4838	cg14406727	cg04011474	cg10520924
4839	cg14410516	cg03961998	cg24420089
4840	cg14412134	cg03953506	cg14204738
4841	cg14413378	cg03919694	cg03861097
4842	cg14414154	cg03892631	cg26328589
4843	cg14419424	cg03674127	cg16023896
4844	cg14420645	cg03666300	cg09848749
4845	cg14425960	cg03600605	cg05479174
4846	cg14426682	cg03566001	cg10063407
4847	cg14428166	cg03430597	cg26547741
4848	cg14429457	cg03389789	cg06694381
4849	cg14438213	cg03223959	cg11612905
4850	cg14444376	cg03181618	cg25285041
4851	cg14455139	cg03140766	cg20227592
4852	cg14455781	cg03050096	cg26871875
4853	cg14461852	cg02875487	cg02240936
4854	cg14462369	cg02806704	cg01519195
4855	cg14467415	cg02780210	cg26784106
4856	cg14469436	cg02645302	cg22822599
4857	cg14470121	cg02621481	cg18653451
4858	cg14473030	cg02592525	cg00358023
4859	cg14473062	cg02572552	cg04650653
4860	cg14474966	cg02560388	cg24831140
4861	cg14475381	cg02469916	cg08281590
4862	cg14476630	cg02469356	cg05030450
4863	cg14480449	cg02351425	cg26332285
4864	cg14482093	cg02191483	cg20595846
4865	cg14487665	cg02053092	cg11436027
4866	cg14497697	cg01827633	cg15181351
4867	cg14500614	cg01801191	cg14455139
4868	cg14502713	cg01767116	cg16734451
4869	cg14505694	cg01759870	cg17843418
4870	cg14506192	cg01604401	cg01443467
4871	cg14514032	cg01559356	cg17112695
4872	cg14516350	cg01475325	cg00232500
4873	cg14519294	cg01432405	cg26840755

4874	cg14519777	cg01139503	cg13153796
4875	cg14521421	cg01038640	cg00851981
4876	cg14523734	cg00977384	cg23466118
4877	cg14524553	cg00951857	cg04607844
4878	cg14524975	cg00947413	cg26798772
4879	cg14529170	cg00937817	cg10248148
4880	cg14543285	cg00937742	cg06900843
4881	cg14547067	cg00863271	cg25316172
4882	cg14547599	cg00785831	cg02017718
4883	cg14550212	cg00742738	cg08619651
4884	cg14553895	cg00738945	cg00035074
4885	cg14558880	cg00607630	cg16163535
4886	cg14560240	cg00594228	cg18815879
4887	cg14565408	cg00585818	cg01641136
4888	cg14565439	cg00569091	cg00478851
4889	cg14567593	cg00549798	cg22591040
4890	cg14570652	cg00548009	cg05638821
4891	cg14571537	cg00500424	cg09961689
4892	cg14574066	cg00478851	cg10713589
4893	cg14575741	cg00454770	cg15592324
4894	cg14577373	cg00452755	cg23093846
4895	cg14578363	cg00359087	cg25616055
4896	cg14583686	cg00356131	cg06943835
4897	cg14583973	cg00308508	cg23366234
4898	cg14588828	cg00232535	cg22173510
4899	cg14596589	cg27637521	cg24901995
4900	cg14597504	cg27613016	cg22562003
4901	cg14598025	cg27503499	cg18668813
4902	cg14599015	cg27491887	cg25828334
4903	cg14601444	cg27396760	cg11631173
4904	cg14602839	cg27382164	cg02447556
4905	cg14606082	cg27381488	cg27201457
4906	cg14610776	cg27365627	cg06753827
4907	cg14615152	cg27320213	cg01887374
4908	cg14616881	cg27295434	cg14500614
4909	cg14620138	cg27233847	cg24706522
4910	cg14626525	cg27201457	cg17467243
4911	cg14630692	cg27160524	cg16465939
4912	cg14638315	cg27082292	cg18857655
4913	cg14641266	cg27070508	cg03326128
4914	cg14642045	cg27053040	cg08277216
4915	cg14644865	cg27011829	cg25825612
4916	cg14645880	cg26978064	cg04398282
4917	cg14650228	cg26965897	cg13411951
4918	cg14655044	cg26934993	cg10836809
4919	cg14655905	cg26919387	cg02327654
4920	cg14657458	cg26890480	cg14127396
4921	cg14658346	cg26889953	cg08450122
4922	cg14671764	cg26828494	cg11415402
4923	cg14679558	cg26778630	cg21942532
4924	cg14681171	cg26564172	cg25502179
4925	cg14684068	cg26434328	cg14317513
4926	cg14684166	cg26405979	cg12339425

4927	cg14692453	cg26397391	cg06436663
4928	cg14692854	cg26326208	cg23876072
4929	cg14699728	cg26313976	cg10550166
4930	cg14700524	cg25978487	cg27011829
4931	cg14701108	cg25977879	cg06710741
4932	cg14701122	cg25946646	cg26160492
4933	cg14706807	cg25940447	cg07530947
4934	cg14711433	cg25827936	cg20733077
4935	cg14715030	cg25825970	cg02214441
4936	cg14715383	cg25765315	cg06868569
4937	cg14732540	cg25761326	cg00446536
4938	cg14734994	cg25749254	cg05185019
4939	cg14738348	cg25688583	cg24293145
4940	cg14741474	cg25654695	cg00887848
4941	cg14742341	cg25642974	cg03068497
4942	cg14744022	cg25637473	cg04254198
4943	cg14747072	cg25591106	cg18313342
4944	cg14753356	cg25568745	cg11101316
4945	cg14758740	cg25506288	cg08110688
4946	cg14758812	cg25477334	cg03244796
4947	cg14763173	cg25451660	cg10319905
4948	cg14764357	cg25409944	cg19375196
4949	cg14772050	cg25323005	cg20781516
4950	cg14777056	cg25242498	cg09334629
4951	cg14778721	cg25146042	cg19561607
4952	cg14780990	cg25137314	cg10997906
4953	cg14781242	cg25006254	cg02193967
4954	cg14782266	cg24926689	cg07622079
4955	cg14785464	cg24881255	cg01390647
4956	cg14788686	cg24691835	cg00187129
4957	cg14789529	cg24606594	cg12417871
4958	cg14790609	cg24586870	cg22040672
4959	cg14811011	cg24362347	cg00184279
4960	cg14817655	cg24349804	cg06857516
4961	cg14820213	cg24154271	cg17700443
4962	cg14826031	cg24137511	cg18553570
4963	cg14829271	cg24045498	cg15225991
4964	cg14830485	cg24009250	cg20699157
4965	cg14833626	cg23996800	cg13630560
4966	cg14835545	cg23947450	cg09246253
4967	cg14839830	cg23887746	cg24760753
4968	cg14841828	cg23827284	cg04034290
4969	cg14842237	cg23779478	cg20118577
4970	cg14864276	cg23732483	cg10859358
4971	cg14870461	cg23696210	cg02975060
4972	cg14870682	cg23691781	cg13808180
4973	cg14875081	cg23690480	cg08242475
4974	cg14875394	cg23610994	cg21500064
4975	cg14878988	cg23601586	cg17783359
4976	cg14879279	cg23485307	cg06259664
4977	cg14879569	cg23318523	cg17343483
4978	cg14884828	cg23316807	cg08665249
4979	cg14899960	cg23301979	cg11092157

4980	cg14914440	cg23299804	cg22743617
4981	cg14916108	cg23244464	cg20654462
4982	cg14917100	cg23100428	cg27156542
4983	cg14918743	cg22998873	cg00149708
4984	cg14919455	cg22932649	cg13151645
4985	cg14920846	cg22908423	cg06297857
4986	cg14921326	cg22414151	cg21242663
4987	cg14923274	cg22343299	cg00860425
4988	cg14923398	cg22257056	cg16566899
4989	cg14937059	cg22150680	cg08552446
4990	cg14937228	cg22118369	cg05459650
4991	cg14939096	cg22097225	cg03575245
4992	cg14939765	cg22020227	cg13425135
4993	cg14947411	cg21942532	cg15121364
4994	cg14947466	cg21845869	cg12185975
4995	cg14960869	cg21840948	cg27616751
4996	cg14966074	cg21837323	cg11835806
4997	cg14966346	cg21740826	cg10584300
4998	cg14971998	cg21613549	cg04260307
4999	cg14978242	cg21611093	cg15031106
5000	cg14983540	cg21574349	cg27248741
5001	cg14992217	cg21549497	cg12629008
5002	cg14992771	cg21544633	cg18248273
5003	cg14995235	cg21450654	cg17334762
5004	cg15012161	cg21422871	cg19817882
5005	cg15014006	cg21400851	cg26893743
5006	cg15016233	cg21185187	cg21504064
5007	cg15016481	cg21133992	cg20934096
5008	cg15016624	cg21006686	cg27434984
5009	cg15016740	cg20910352	cg26338030
5010	cg15020964	cg20785796	cg05909553
5011	cg15022671	cg20745684	cg12900489
5012	cg15026277	cg20741105	cg16200325
5013	cg15028232	cg20731110	cg04586928
5014	cg15028507	cg20567148	cg25945499
5015	cg15031106	cg20566286	cg22532194
5016	cg15033036	cg20524889	cg15676677
5017	cg15033511	cg20390500	cg09674368
5018	cg15034300	cg20383521	cg12375903
5019	cg15035360	cg20300776	cg26597727
5020	cg15036118	cg20193802	cg01176823
5021	cg15048900	cg20080282	cg07832950
5022	cg15063355	cg20076442	cg26750188
5023	cg15063366	cg20051177	cg22888023
5024	cg15065169	cg20027784	cg20060759
5025	cg15072038	cg20008846	cg00776208
5026	cg15074047	cg19991046	cg09373983
5027	cg15076811	cg19879265	cg25013838
5028	cg15083845	cg19771975	cg23619577
5029	cg15086474	cg19761272	cg21750915
5030	cg15089181	cg19622474	cg23212751
5031	cg15089272	cg19577082	cg11700356
5032	cg15089806	cg19529732	cg14923274

5033	cg15104081	cg19515081	cg23818351
5034	cg15111351	cg19494591	cg14780990
5035	cg15117891	cg19479935	cg21560157
5036	cg15121364	cg19445044	cg05899507
5037	cg15121417	cg19348272	cg10893014
5038	cg15126957	cg19295314	cg20297670
5039	cg15127656	cg19270309	cg04804094
5040	cg15128510	cg19113668	cg17010895
5041	cg15141238	cg18888464	cg15111351
5042	cg15142430	cg18811860	cg27378358
5043	cg15155463	cg18517266	cg26720682
5044	cg15161050	cg18505837	cg17214107
5045	cg15162922	cg18279126	cg08866865
5046	cg15164881	cg18226382	cg10519271
5047	cg15168816	cg18061711	cg13119578
5048	cg15170442	cg18018990	cg27143695
5049	cg15171237	cg17977723	cg11753157
5050	cg15175632	cg17971649	cg25167447
5051	cg15188939	cg17907628	cg25975823
5052	cg15209369	cg17893426	cg09695261
5053	cg15214284	cg17884766	cg13274014
5054	cg15225991	cg17865371	cg19120897
5055	cg15228983	cg17819085	cg01201519
5056	cg15230883	cg17736336	cg03244438
5057	cg15231872	cg17700633	cg20329085
5058	cg15232718	cg17700443	cg22999786
5059	cg15241074	cg17497965	cg13618372
5060	cg15247169	cg17341136	cg11189177
5061	cg15249446	cg17234201	cg13332783
5062	cg15250073	cg17202706	cg18795383
5063	cg15259233	cg17176209	cg05218696
5064	cg15260437	cg17153432	cg24768094
5065	cg15262242	cg16975812	cg25650076
5066	cg15264708	cg16908123	cg08732879
5067	cg15264991	cg16889721	cg26127113
5068	cg15267890	cg16796135	cg02289020
5069	cg15269394	cg16712866	cg04530852
5070	cg15275017	cg16711983	cg21238061
5071	cg15277378	cg16609966	cg12905673
5072	cg15283164	cg16585619	cg00718831
5073	cg15299233	cg16554164	cg03420242
5074	cg15302379	cg16201762	cg11897120
5075	cg15318570	cg15862394	cg13821287
5076	cg15322667	cg15744108	cg02402630
5077	cg15329179	cg15679098	cg00677217
5078	cg15335728	cg15626112	cg04009429
5079	cg15336269	cg15618758	cg10507988
5080	cg15342087	cg15582126	cg10288921
5081	cg15352671	cg15498121	cg10714160
5082	cg15354065	cg15489196	cg23400222
5083	cg15355859	cg15417249	cg26218835
5084	cg15363996	cg15214284	cg15755154
5085	cg15364618	cg15083845	cg00061185

5086	cg15367698	cg14820213	cg22118416
5087	cg15368455	cg14732540	cg13207250
5088	cg15373257	cg14715383	cg04147875
5089	cg15374133	cg14655905	cg11079426
5090	cg15383141	cg14620138	cg10171557
5091	cg15389749	cg14567593	cg25445431
5092	cg15391531	cg14480449	cg13634090
5093	cg15396800	cg14474966	cg19047283
5094	cg15401523	cg14360029	cg04806664
5095	cg15402232	cg14351526	cg20892492
5096	cg15407257	cg14325029	cg05041061
5097	cg15408497	cg14312334	cg16239826
5098	cg15408640	cg14227460	cg24034465
5099	cg15416179	cg14195992	cg16207263
5100	cg15420720	cg14165633	cg26147269
5101	cg15422033	cg14156650	cg08197880
5102	cg15424477	cg14088181	cg13036546
5103	cg15425427	cg14082061	cg17685579
5104	cg15444149	cg14011387	cg13567403
5105	cg15446884	cg13984005	cg10902107
5106	cg15450139	cg13916961	cg24215635
5107	cg15451258	cg13818316	cg24033503
5108	cg15459715	cg13795838	cg09915232
5109	cg15465367	cg13730600	cg03294704
5110	cg15473017	cg13728131	cg25649889
5111	cg15475080	cg13706365	cg07716287
5112	cg15485665	cg13691622	cg06363129
5113	cg15487498	cg13630646	cg21238609
5114	cg15488570	cg13488013	cg17667773
5115	cg15489196	cg13461718	cg14389425
5116	cg15498121	cg13411951	cg19062489
5117	cg15498306	cg13377839	cg25993608
5118	cg15504666	cg13309415	cg00963675
5119	cg15506281	cg13177873	cg21535761
5120	cg15507500	cg13165390	cg02062816
5121	cg15516314	cg13119578	cg02157454
5122	cg15518113	cg13063335	cg01256365
5123	cg15520941	cg13062396	cg18860847
5124	cg15532236	cg13003178	cg26934759
5125	cg15536489	cg12901038	cg07124859
5126	cg15536947	cg12846837	cg21558509
5127	cg15540341	cg12842409	cg21434355
5128	cg15541630	cg12766383	cg14918743
5129	cg15543534	cg12734852	cg04226952
5130	cg15545942	cg12708994	cg22039204
5131	cg15546071	cg12705212	cg01993468
5132	cg15547344	cg12595697	cg15411034
5133	cg15548656	cg12555086	cg13470557
5134	cg15556723	cg12250561	cg11731626
5135	cg15561453	cg12143499	cg02084214
5136	cg15574810	cg12129983	cg05080811
5137	cg15576082	cg12119133	cg03985415
5138	cg15577180	cg12071544	cg06294856

5139	cg15582126	cg12036877	cg05070608
5140	cg15582707	cg11996592	cg25390635
5141	cg15587041	cg11936817	cg05995260
5142	cg15592324	cg11860238	cg15629568
5143	cg15598425	cg11828741	cg15616400
5144	cg15600176	cg11685391	cg06753918
5145	cg15603885	cg11672338	cg23566503
5146	cg15613048	cg11662712	cg22371518
5147	cg15616400	cg11450827	cg21573345
5148	cg15617336	cg11432630	cg06856570
5149	cg15617847	cg11320499	cg14469436
5150	cg15618758	cg11176525	cg23760873
5151	cg15626112	cg11025609	cg26133909
5152	cg15627277	cg10692363	cg00977384
5153	cg15627593	cg10607485	cg00260235
5154	cg15629568	cg10369955	cg13610622
5155	cg15632675	cg10307940	cg16694664
5156	cg15633699	cg10168009	cg04132177
5157	cg15636859	cg10102102	cg02226672
5158	cg15637234	cg09975093	cg22805632
5159	cg15637254	cg09900084	cg11124652
5160	cg15639581	cg09856367	cg04911680
5161	cg15646741	cg09825670	cg27178714
5162	cg15656769	cg09659803	cg26867987
5163	cg15657100	cg09526469	cg09218398
5164	cg15658793	cg09495643	cg15576082
5165	cg15661715	cg09457490	cg14272573
5166	cg15676677	cg09386073	cg06863830
5167	cg15678662	cg09358961	cg12855271
5168	cg15679098	cg09304617	cg04665930
5169	cg15684702	cg09251291	cg02495395
5170	cg15686782	cg09198283	cg02958180
5171	cg15693937	cg09185884	cg07561338
5172	cg15702277	cg09101894	cg05294455
5173	cg15709435	cg09043524	cg14262433
5174	cg15712304	cg08779777	cg12778183
5175	cg15715853	cg08701134	cg16067210
5176	cg15720017	cg08650647	cg08625990
5177	cg15726557	cg08631328	cg07483266
5178	cg15744108	cg08625996	cg17034088
5179	cg15755154	cg08458637	cg13573244
5180	cg15756507	cg08412188	cg01460382
5181	cg15765251	cg08325885	cg22666223
5182	cg15770585	cg08293102	cg27049344
5183	cg15772924	cg08250738	cg02762546
5184	cg15777335	cg08209934	cg05623411
5185	cg15802323	cg08180831	cg18809729
5186	cg15806038	cg08132525	cg08005460
5187	cg15813460	cg08124923	cg22528270
5188	cg15815827	cg08110688	cg07979388
5189	cg15816464	cg08106635	cg19658812
5190	cg15817440	cg08104310	cg07036799
5191	cg15819921	cg08103691	cg03778594

5192	cg15834460	cg08070235	cg21726593
5193	cg15843262	cg08044294	cg21006686
5194	cg15844895	cg07844828	cg27227317
5195	cg15850851	cg07793033	cg16116632
5196	cg15854022	cg07740306	cg24771349
5197	cg15862380	cg07586345	cg04618903
5198	cg15862394	cg07560287	cg16626731
5199	cg15874302	cg07505901	cg00095326
5200	cg15879620	cg07498879	cg14405528
5201	cg15881332	cg07363131	cg24008544
5202	cg15888569	cg07353266	cg25866889
5203	cg15888693	cg07290652	cg01783960
5204	cg15892617	cg07275305	cg02315513
5205	cg15895197	cg07262506	cg14917100
5206	cg15899800	cg07217341	cg13727957
5207	cg15904939	cg07115952	cg06987369
5208	cg15910594	cg07108443	cg26261873
5209	cg15911948	cg07070824	cg17636986
5210	cg15933120	cg07068045	cg21628927
5211	cg15934607	cg07054804	cg19895894
5212	cg15935860	cg07041883	cg23299804
5213	cg15937073	cg06943845	cg25606046
5214	cg15943602	cg06900843	cg12921473
5215	cg15944060	cg06899970	cg02340818
5216	cg15956469	cg06887283	cg10024484
5217	cg15957394	cg06826908	cg22049753
5218	cg15959893	cg06807837	cg25939647
5219	cg15968604	cg06752482	cg17791936
5220	cg15969277	cg06746744	cg15632675
5221	cg15970457	cg06632214	cg12616177
5222	cg15970890	cg06617528	cg07997682
5223	cg15974867	cg06597413	cg14342817
5224	cg15975598	cg06562888	cg08168529
5225	cg15980914	cg06537333	cg12583325
5226	cg15988204	cg06351520	cg02287325
5227	cg15988734	cg06292891	cg07115952
5228	cg15988812	cg06290096	cg06246999
5229	cg15991082	cg06275130	cg15020964
5230	cg16004738	cg06167719	cg15161854
5231	cg16006963	cg06085713	cg03108347
5232	cg16009311	cg05977523	cg17196713
5233	cg16016960	cg05894015	cg06500727
5234	cg16023122	cg05888037	cg00765705
5235	cg16023896	cg05718035	cg23656110
5236	cg16024937	cg05670093	cg15765251
5237	cg16026212	cg05638821	cg09536516
5238	cg16026813	cg05469118	cg22277106
5239	cg16027775	cg05324992	cg09020697
5240	cg16030878	cg05270552	cg03138206
5241	cg16030972	cg05194102	cg07605062
5242	cg16031980	cg05145297	cg02935024
5243	cg16034562	cg04945557	cg20005923
5244	cg16051954	cg04904609	cg17563229

5245	cg16052388	cg04882759	cg21925748
5246	cg16071082	cg04868238	cg17285177
5247	cg16071782	cg04860563	cg23143093
5248	cg16073408	cg04844977	cg00271311
5249	cg16075266	cg04784315	cg22019569
5250	cg16077055	cg04685302	cg07449447
5251	cg16078836	cg04663956	cg26915618
5252	cg16078863	cg04586928	cg21906519
5253	cg16081096	cg04530852	cg21464492
5254	cg16081499	cg04524477	cg21483216
5255	cg16084313	cg04510807	cg26797297
5256	cg16089233	cg04493177	cg10357989
5257	cg16092370	cg04465834	cg06711298
5258	cg16102706	cg04462774	cg00286014
5259	cg16102778	cg04454664	cg10667207
5260	cg16106427	cg04403415	cg20140662
5261	cg16106903	cg04341806	cg00594228
5262	cg16114651	cg04312967	cg26019124
5263	cg16116632	cg04240456	cg23005227
5264	cg16117028	cg04195543	cg12054698
5265	cg16125397	cg04137490	cg15844895
5266	cg16126178	cg04074404	cg14331206
5267	cg16133703	cg04070601	cg24970620
5268	cg16139912	cg04041283	cg25971741
5269	cg16142965	cg03872934	cg22916722
5270	cg16144562	cg03743074	cg07123022
5271	cg16145211	cg03739877	cg04655520
5272	cg16151538	cg03737788	cg22169866
5273	cg16161135	cg03668470	cg26660115
5274	cg16163535	cg03649353	cg24482246
5275	cg16163756	cg03614132	cg20495333
5276	cg16167741	cg03472610	cg24706188
5277	cg16168321	cg03452625	cg22958118
5278	cg16172837	cg03446062	cg07521789
5279	cg16174644	cg03400491	cg00322201
5280	cg16175077	cg03242880	cg14764357
5281	cg16183741	cg03198029	cg26365254
5282	cg16184131	cg03181382	cg11010976
5283	cg16185457	cg03135948	cg12499572
5284	cg16195091	cg03054026	cg16344173
5285	cg16200325	cg02940164	cg00286717
5286	cg16201762	cg02869364	cg24647108
5287	cg16204524	cg02817932	cg03582793
5288	cg16207263	cg02762546	cg14311523
5289	cg16212219	cg02737507	cg18330203
5290	cg16213787	cg02729303	cg12025243
5291	cg16221927	cg02722657	cg22472304
5292	cg16222367	cg02712546	cg03736774
5293	cg16222790	cg02697979	cg22563907
5294	cg16223967	cg02693127	cg02821693
5295	cg16232530	cg02648939	cg05662839
5296	cg16234490	cg02622601	cg08559648
5297	cg16236270	cg02600978	cg26068595

5298	cg16237297	cg02569718	cg26245697
5299	cg16238993	cg02517473	cg18852515
5300	cg16239826	cg02384859	cg24314474
5301	cg16245035	cg02272751	cg08135904
5302	cg16255804	cg02196592	cg10330187
5303	cg16258545	cg02165692	cg04145522
5304	cg16263152	cg02119693	cg14340089
5305	cg16265707	cg02113385	cg12872329
5306	cg16265932	cg02060039	cg18716503
5307	cg16269144	cg02048412	cg24728456
5308	cg16274890	cg02008511	cg11781564
5309	cg16277922	cg01959238	cg26124719
5310	cg16283362	cg01914059	cg12901038
5311	cg16285424	cg01859460	cg22822048
5312	cg16288579	cg01810347	cg11321921
5313	cg16289101	cg01794929	cg13509456
5314	cg16290996	cg01758022	cg09119967
5315	cg16292933	cg01676623	cg10494703
5316	cg16293569	cg01553388	cg07410188
5317	cg16306083	cg01501018	cg26079864
5318	cg16306870	cg01481251	cg04457069
5319	cg16309127	cg01456030	cg09643151
5320	cg16311946	cg01428849	cg17469039
5321	cg16312872	cg01411786	cg02961196
5322	cg16316042	cg01403055	cg20513080
5323	cg16318412	cg01343936	cg09327804
5324	cg16326073	cg01298514	cg04107939
5325	cg16328007	cg01297744	cg22054793
5326	cg16329509	cg01239666	cg12887832
5327	cg16337430	cg01153620	cg17836842
5328	cg16340152	cg01063813	cg00488716
5329	cg16344173	cg01063615	cg15943602
5330	cg16359657	cg00941576	cg20000539
5331	cg16365663	cg00869989	cg20544097
5332	cg16366639	cg00863893	cg11990296
5333	cg16366809	cg00776208	cg09551019
5334	cg16381321	cg00706536	cg17199010
5335	cg16385941	cg00701514	cg02060434
5336	cg16387467	cg00675878	cg23250528
5337	cg16387532	cg00656991	cg02718078
5338	cg16391182	cg00585698	cg06028151
5339	cg16391347	cg00550617	cg19063768
5340	cg16392729	cg00547727	cg05269678
5341	cg16392860	cg00488512	cg19940729
5342	cg16395892	cg00358023	cg03487040
5343	cg16397629	cg00314994	cg22240998
5344	cg16402415	cg00270654	cg22971402
5345	cg16407998	cg00175895	cg15162922
5346	cg16411103	cg00059024	cg03053456
5347	cg16412000	cg00035923	cg07836226
5348	cg16419526	cg27643147	cg06632821
5349	cg16421359	cg27572074	cg03962809
5350	cg16421653	cg27533454	cg05370136

5351	cg16422492	cg27492839	cg22370005
5352	cg16425726	cg27438218	cg08934318
5353	cg16427420	cg27409974	cg23316807
5354	cg16436210	cg27399753	cg03284113
5355	cg16442814	cg27278961	cg25007422
5356	cg16446824	cg27181295	cg09646558
5357	cg16452866	cg27177296	cg00513981
5358	cg16453673	cg27170206	cg12604490
5359	cg16461139	cg27130493	cg04843690
5360	cg16463044	cg27117439	cg08033285
5361	cg16465939	cg26953293	cg12948116
5362	cg16474791	cg26942121	cg01204060
5363	cg16475712	cg26877336	cg01414464
5364	cg16476700	cg26856771	cg18247852
5365	cg16479633	cg26804423	cg05561386
5366	cg16488544	cg26803670	cg11994639
5367	cg16489468	cg26711406	cg18692382
5368	cg16496024	cg26613811	cg00043005
5369	cg16496462	cg26495716	cg01214847
5370	cg16499415	cg26426564	cg09372617
5371	cg16499416	cg26311262	cg06167719
5372	cg16499956	cg26261270	cg27287438
5373	cg16502866	cg26178184	cg26448137
5374	cg16509072	cg26177754	cg01559031
5375	cg16509173	cg26105658	cg15933120
5376	cg16510440	cg26100137	cg14264901
5377	cg16511831	cg26060535	cg02907662
5378	cg16514214	cg26019124	cg25668093
5379	cg16517702	cg25939647	cg09216143
5380	cg16521032	cg25883020	cg05767015
5381	cg16521443	cg25881985	cg01914059
5382	cg16522491	cg25807487	cg02102684
5383	cg16523645	cg25713411	cg08864042
5384	cg16525330	cg25661792	cg13672348
5385	cg16526755	cg25649889	cg27400447
5386	cg16529592	cg25619703	cg27278961
5387	cg16530165	cg25588387	cg10168009
5388	cg16532399	cg25531618	cg04129282
5389	cg16533510	cg25498969	cg04465834
5390	cg16537245	cg25492112	cg03125400
5391	cg16550656	cg25406657	cg13521002
5392	cg16563454	cg25383568	cg20156139
5393	cg16564132	cg25311162	cg03526625
5394	cg16564135	cg25275372	cg04386759
5395	cg16565294	cg25261191	cg10115918
5396	cg16566899	cg25249728	cg22379668
5397	cg16570314	cg25148456	cg01558195
5398	cg16572224	cg25095994	cg26120842
5399	cg16572410	cg25082361	cg16741602
5400	cg16572603	cg25066224	cg25270886
5401	cg16580681	cg25053164	cg23750338
5402	cg16582732	cg24973993	cg16217297
5403	cg16584688	cg24773493	cg10370426

5404	cg16585619	cg24748868	cg04941831
5405	cg16597437	cg24723883	cg21650861
5406	cg16602460	cg24674087	cg23108126
5407	cg16608596	cg24469719	cg06332666
5408	cg16609966	cg24463664	cg01733438
5409	cg16616370	cg24462247	cg14830485
5410	cg16616765	cg24425316	cg06671298
5411	cg16617141	cg24372256	cg26224791
5412	cg16621790	cg24365837	cg13499923
5413	cg16626731	cg24299913	cg21334198
5414	cg16629076	cg24294013	cg11952839
5415	cg16636756	cg24194413	cg05858079
5416	cg16640008	cg24163616	cg06126019
5417	cg16640450	cg24093176	cg26222498
5418	cg16653700	cg23976499	cg15637254
5419	cg16660312	cg23951305	cg27612166
5420	cg16664095	cg23895846	cg06355908
5421	cg16666964	cg23887149	cg04140297
5422	cg16668534	cg23876072	cg13850871
5423	cg16668903	cg23834919	cg12705212
5424	cg16672557	cg23707719	cg00610205
5425	cg16680624	cg23614979	cg01345395
5426	cg16681083	cg23539494	cg24270629
5427	cg16681436	cg23529249	cg21805118
5428	cg16694664	cg23509272	cg01825806
5429	cg16695570	cg23476802	cg18881978
5430	cg16697024	cg23339720	cg27632471
5431	cg16700163	cg23301539	cg09697259
5432	cg16700555	cg23261102	cg19781117
5433	cg16704889	cg23212751	cg04353997
5434	cg16704938	cg23019125	cg04685302
5435	cg16706038	cg22979433	cg21460686
5436	cg16707423	cg22908922	cg13624631
5437	cg16711011	cg22854448	cg00549798
5438	cg16711084	cg22846966	cg13135376
5439	cg16712866	cg22838354	cg26614264
5440	cg16718902	cg22831256	cg27117439
5441	cg16721977	cg22762745	cg14425914
5442	cg16726039	cg22761077	cg13734106
5443	cg16726374	cg22731981	cg24752354
5444	cg16734433	cg22731190	cg21170978
5445	cg16734451	cg22706186	cg22366375
5446	cg16738646	cg22655196	cg18813518
5447	cg16745596	cg22555262	cg10642094
5448	cg16747567	cg22463097	cg06584329
5449	cg16752592	cg22380476	cg27399753
5450	cg16759204	cg22171607	cg19055828
5451	cg16762085	cg22047282	cg27100471
5452	cg16766036	cg21998505	cg22237707
5453	cg16766325	cg21879102	cg09474229
5454	cg16772023	cg21735384	cg22011888
5455	cg16773043	cg21725701	cg18168520
5456	cg16776981	cg21620968	cg24423806

5457	cg16778107	cg21569714	cg05040210
5458	cg16786144	cg21557473	cg00028338
5459	cg16794682	cg21546950	cg05770240
5460	cg16796135	cg21445553	cg23935261
5461	cg16797656	cg21374048	cg22679890
5462	cg16803044	cg21364077	cg14569423
5463	cg16812626	cg21286967	cg19044462
5464	cg16819272	cg21252105	cg25856632
5465	cg16820411	cg21158528	cg20512713
5466	cg16823064	cg21067905	cg12371563
5467	cg16824603	cg21031345	cg06406157
5468	cg16828491	cg20989443	cg05062820
5469	cg16829042	cg20967819	cg21497164
5470	cg16846503	cg20943461	cg20412217
5471	cg16861209	cg20924972	cg09956907
5472	cg16863382	cg20861822	cg14952488
5473	cg16867086	cg20733739	cg14328115
5474	cg16874347	cg20677267	cg07990198
5475	cg16876192	cg20609679	cg06673840
5476	cg16880392	cg20548182	cg05076082
5477	cg16883145	cg20540306	cg02304222
5478	cg16885096	cg20494661	cg20571592
5479	cg16885557	cg20490386	cg07071809
5480	cg16890796	cg20449048	cg04248364
5481	cg16892393	cg20396385	cg13096278
5482	cg16897462	cg20338075	cg22811647
5483	cg16897800	cg20262021	cg07867517
5484	cg16902190	cg20252837	cg03538436
5485	cg16908123	cg20070852	cg13514778
5486	cg16908801	cg20017253	cg07706695
5487	cg16910448	cg19783160	cg17849972
5488	cg16919420	cg19725377	cg03333776
5489	cg16920538	cg19713196	cg03884082
5490	cg16927379	cg19658812	cg05615477
5491	cg16929793	cg19629843	cg22963109
5492	cg16949378	cg19539481	cg21276022
5493	cg16953473	cg19536407	cg09935792
5494	cg16962557	cg19480117	cg06532257
5495	cg16975812	cg19478410	cg14723032
5496	cg16989380	cg19455322	cg23635789
5497	cg16995299	cg19382919	cg10512408
5498	cg16996242	cg19308185	cg07691004
5499	cg16996266	cg19287817	cg07206497
5500	cg16996571	cg19270505	cg18817654
5501	cg16998564	cg19257111	cg23413464
5502	cg17009731	cg19125081	cg13947999
5503	cg17010895	cg19107511	cg26745294
5504	cg17013513	cg19011826	cg00789416
5505	cg17017404	cg18918538	cg12700788
5506	cg17018661	cg18862502	cg05986052
5507	cg17021949	cg18760055	cg11940726
5508	cg17024719	cg18751203	cg16387532
5509	cg17027476	cg18716503	cg16629076

5510	cg17030415	cg18475969	cg07379550
5511	cg17032372	cg18371976	cg16511831
5512	cg17034088	cg18327695	cg23157618
5513	cg17036007	cg18243853	cg22521416
5514	cg17036641	cg18228456	cg24562682
5515	cg17049328	cg18191688	cg09900084
5516	cg17054783	cg18019825	cg25409944
5517	cg17067528	cg17961835	cg10552275
5518	cg17074958	cg17956079	cg06193668
5519	cg17094249	cg17844448	cg27506609
5520	cg17095737	cg17792616	cg06241044
5521	cg17095936	cg17749384	cg13860981
5522	cg17099569	cg17722435	cg11698119
5523	cg17101681	cg17696194	cg22367886
5524	cg17103856	cg17673769	cg16329509
5525	cg17107017	cg17645528	cg11450827
5526	cg17112404	cg17563229	cg21385606
5527	cg17112695	cg17524821	cg08259083
5528	cg17112949	cg17429870	cg03659476
5529	cg17113147	cg17348667	cg02999082
5530	cg17115147	cg17322118	cg19182279
5531	cg17130544	cg17221945	cg08836353
5532	cg17152266	cg17206596	cg26379230
5533	cg17153432	cg17112949	cg00978248
5534	cg17156862	cg17034088	cg02818775
5535	cg17169982	cg17030415	cg24881558
5536	cg17171539	cg17010895	cg21438602
5537	cg17172949	cg16920538	cg23087358
5538	cg17173086	cg16766325	cg07095945
5539	cg17174566	cg16726374	cg13367537
5540	cg17176209	cg16684117	cg02107173
5541	cg17179557	cg16560256	cg06925389
5542	cg17181336	cg16526755	cg25355635
5543	cg17185710	cg16519668	cg25515317
5544	cg17196448	cg16498681	cg02358804
5545	cg17199010	cg16475712	cg24462247
5546	cg17201542	cg16397629	cg24009250
5547	cg17202331	cg16392729	cg18517266
5548	cg17202706	cg16344173	cg18051353
5549	cg17203290	cg16306870	cg18917736
5550	cg17206596	cg16175077	cg21177940
5551	cg17208278	cg16009311	cg15737302
5552	cg17211223	cg15891850	cg13003178
5553	cg17213154	cg15816464	cg18704218
5554	cg17214107	cg15636859	cg01077713
5555	cg17216243	cg15541630	cg12163781
5556	cg17221941	cg15516314	cg23372092
5557	cg17226348	cg15444149	cg11151524
5558	cg17234201	cg15355859	cg06219670
5559	cg17241937	cg15346619	cg23261343
5560	cg17243540	cg15318570	cg24362347
5561	cg17250537	cg15267890	cg01139503
5562	cg17254904	cg15259233	cg09004351

5563	cg17256760	cg15230743	cg18933685
5564	cg17264541	cg15034300	cg26268565
5565	cg17266282	cg14875081	cg18751958
5566	cg17276624	cg14833024	cg03511735
5567	cg17278466	cg14829271	cg12081291
5568	cg17284725	cg14747072	cg02153855
5569	cg17285177	cg14650228	cg23714019
5570	cg17285883	cg14642045	cg09975561
5571	cg17289222	cg14630692	cg14443041
5572	cg17291826	cg14596589	cg17054783
5573	cg17297354	cg14583686	cg02486181
5574	cg17304712	cg14444376	cg15853299
5575	cg17315679	cg14377596	cg05595142
5576	cg17319576	cg14331609	cg13093023
5577	cg17319795	cg14329833	cg19750606
5578	cg17321561	cg14321412	cg15353597
5579	cg17322118	cg14113353	cg12061219
5580	cg17322655	cg14074351	cg25567674
5581	cg17327331	cg14016879	cg09370702
5582	cg17332023	cg13981325	cg08973281
5583	cg17333042	cg13930682	cg22288012
5584	cg17334762	cg13598881	cg16706038
5585	cg17340779	cg13586038	cg10107322
5586	cg17341136	cg13584383	cg13115424
5587	cg17343483	cg13583088	cg24594604
5588	cg17344813	cg13567403	cg16183741
5589	cg17361593	cg13558912	cg16812626
5590	cg17364872	cg13545717	cg19912309
5591	cg17367836	cg13475822	cg16616370
5592	cg17367884	cg13400018	cg08264519
5593	cg17367901	cg13378789	cg14190128
5594	cg17378535	cg13278105	cg00669856
5595	cg17384769	cg13210470	cg00547727
5596	cg17385318	cg13189671	cg23397909
5597	cg17394978	cg13162609	cg00742738
5598	cg17400113	cg13029400	cg03216506
5599	cg17400235	cg12905673	cg22459081
5600	cg17415355	cg12819011	cg26234644
5601	cg17418956	cg12797609	cg02566359
5602	cg17426166	cg12737285	cg07241090
5603	cg17428748	cg12736760	cg05559648
5604	cg17431079	cg12727138	cg09249800
5605	cg17434577	cg12686055	cg07447742
5606	cg17439009	cg12657361	cg20300794
5607	cg17442683	cg12583325	cg24619276
5608	cg17442932	cg12550496	cg21806238
5609	cg17444479	cg12526923	cg14516350
5610	cg17444642	cg12450391	cg13798270
5611	cg17450505	cg12433926	cg01000937
5612	cg17454920	cg12419932	cg13655570
5613	cg17459290	cg12392429	cg02486904
5614	cg17467243	cg12233100	cg04668631
5615	cg17468543	cg12077685	cg03426615

5616	cg17469039	cg12011299	cg19081470
5617	cg17470198	cg11902934	cg05504354
5618	cg17470627	cg11828820	cg07743919
5619	cg17475200	cg11758841	cg19422138
5620	cg17475304	cg11575912	cg14218838
5621	cg17482237	cg11567885	cg01339351
5622	cg17505642	cg11526020	cg17522727
5623	cg17508905	cg11512026	cg11068930
5624	cg17511604	cg11476985	cg18931850
5625	cg17517442	cg11367404	cg03333149
5626	cg17522727	cg11346522	cg04929165
5627	cg17524821	cg11150559	cg11828820
5628	cg17525357	cg11139446	cg19478343
5629	cg17528003	cg11092157	cg15712057
5630	cg17537252	cg11081826	cg08863422
5631	cg17537493	cg11079426	cg16089233
5632	cg17548735	cg10965996	cg18543610
5633	cg17550566	cg10950593	cg26325286
5634	cg17560283	cg10809958	cg22764700
5635	cg17563229	cg10639411	cg26070874
5636	cg17569754	cg10545738	cg02274236
5637	cg17574471	cg10532807	cg06269419
5638	cg17578309	cg10493436	cg04183638
5639	cg17586345	cg10296062	cg18302629
5640	cg17587997	cg10257671	cg10601476
5641	cg17589079	cg10235845	cg06549824
5642	cg17589890	cg10142436	cg03630710
5643	cg17599809	cg10060631	cg27317493
5644	cg17601658	cg10006956	cg11517528
5645	cg17610889	cg09996971	cg16784943
5646	cg17611093	cg09961689	cg26398024
5647	cg17634490	cg09897002	cg05976516
5648	cg17638841	cg09848749	cg06477663
5649	cg17645528	cg09811393	cg21163717
5650	cg17649815	cg09795809	cg12156167
5651	cg17650713	cg09534489	cg11367404
5652	cg17667773	cg09483967	cg03017264
5653	cg17670999	cg09389557	cg18435870
5654	cg17671199	cg09370299	cg19618438
5655	cg17672035	cg09309208	cg24437408
5656	cg17673769	cg09233395	cg01194444
5657	cg17678224	cg09228051	cg07352757
5658	cg17680611	cg09213124	cg11671925
5659	cg17683336	cg09119967	cg15744108
5660	cg17685579	cg09109383	cg13908988
5661	cg17692230	cg09015905	cg02785870
5662	cg17695841	cg08983215	cg14937059
5663	cg17700633	cg08943374	cg07607355
5664	cg17702736	cg08899531	cg23690444
5665	cg17703629	cg08886695	cg14584535
5666	cg17720554	cg08732879	cg17032372
5667	cg17721879	cg08726900	cg07008193
5668	cg17722435	cg08552167	cg19632842

5669	cg17724175	cg08411881	cg15142430
5670	cg17724627	cg08293075	cg06017355
5671	cg17732044	cg08176056	cg18639238
5672	cg17732877	cg08132573	cg05517824
5673	cg17736336	cg08101977	cg08101977
5674	cg17740401	cg08003887	cg02025435
5675	cg17742459	cg07990198	cg22999502
5676	cg17743939	cg07938459	cg13747876
5677	cg17747635	cg07929112	cg18751203
5678	cg17749384	cg07907315	cg06851844
5679	cg17755483	cg07888884	cg24885442
5680	cg17758899	cg07849237	cg25915512
5681	cg17775332	cg07836226	cg05800368
5682	cg17780565	cg07835443	cg15506281
5683	cg17781958	cg07810967	cg06861572
5684	cg17783359	cg07713495	cg02346135
5685	cg17787876	cg07642499	cg21165519
5686	cg17789138	cg07638689	cg03841376
5687	cg17791936	cg07607355	cg10533694
5688	cg17792616	cg07574854	cg00938266
5689	cg17793608	cg07521789	cg01023808
5690	cg17796043	cg07464578	cg05279172
5691	cg17796593	cg07412315	cg04986373
5692	cg17797898	cg07336123	cg07192772
5693	cg17798749	cg07260789	cg04187433
5694	cg17799760	cg07221518	cg26714514
5695	cg17803589	cg07205670	cg26104640
5696	cg17817350	cg07178379	cg00473257
5697	cg17818471	cg07064592	cg08386696
5698	cg17819085	cg07057687	cg05258102
5699	cg17820025	cg07025242	cg03835755
5700	cg17825194	cg07022478	cg26187123
5701	cg17827670	cg06995299	cg10796022
5702	cg17830810	cg06969777	cg02144518
5703	cg17830812	cg06937201	cg06008803
5704	cg17836842	cg06926306	cg00680875
5705	cg17838182	cg06916574	cg24502047
5706	cg17842918	cg06862949	cg23399577
5707	cg17843418	cg06833798	cg25554340
5708	cg17844448	cg06815308	cg16419526
5709	cg17846497	cg06787104	cg04997235
5710	cg17849972	cg06783380	cg09511351
5711	cg17850498	cg06741399	cg16717225
5712	cg17851345	cg06696911	cg05977276
5713	cg17853850	cg06650364	cg03657791
5714	cg17854297	cg06633739	cg00943124
5715	cg17857870	cg06614534	cg01309081
5716	cg17865371	cg06572904	cg25594106
5717	cg17868840	cg06568783	cg10299941
5718	cg17876645	cg06528340	cg13222945
5719	cg17877600	cg06509598	cg05041871
5720	cg17884766	cg06456125	cg01437411
5721	cg17886413	cg06432200	cg10754670

5722	cg17893426	cg06386880	cg14583686
5723	cg17901582	cg06371044	cg16306083
5724	cg17903316	cg06363129	cg06018095
5725	cg17920820	cg06332339	cg27053299
5726	cg17929859	cg06233301	cg02596645
5727	cg17931529	cg06213876	cg10856972
5728	cg17938607	cg06174078	cg26484017
5729	cg17939585	cg06109284	cg16694397
5730	cg17943672	cg06012483	cg06628679
5731	cg17945541	cg05949181	cg17400235
5732	cg17947599	cg05909702	cg15959893
5733	cg17956079	cg05776138	cg15498306
5734	cg17958516	cg05767299	cg18592315
5735	cg17966344	cg05715422	cg01693662
5736	cg17971649	cg05703996	cg22472360
5737	cg17972213	cg05682319	cg21043481
5738	cg17976731	cg05631399	cg03817675
5739	cg17977133	cg05418508	cg02983650
5740	cg17977723	cg05409945	cg10642330
5741	cg17980999	cg05404117	cg05404117
5742	cg17985555	cg05368595	cg06846976
5743	cg17993073	cg05361262	cg16158296
5744	cg18000764	cg05291588	cg13035910
5745	cg18011099	cg05267269	cg22905571
5746	cg18013789	cg05226061	cg14118424
5747	cg18018990	cg05224498	cg12467090
5748	cg18019825	cg05201312	cg27144884
5749	cg18027683	cg05190559	cg13971603
5750	cg18030799	cg05185038	cg09123625
5751	cg18032164	cg05185019	cg10884953
5752	cg18034859	cg05161923	cg03971344
5753	cg18035571	cg05112986	cg13200640
5754	cg18036710	cg05099063	cg16366809
5755	cg18045658	cg04983933	cg19101998
5756	cg18047920	cg04956511	cg09174690
5757	cg18048309	cg04920385	cg06090895
5758	cg18050634	cg04846781	cg20600205
5759	cg18051353	cg04843690	cg01283300
5760	cg18051825	cg04797170	cg26185621
5761	cg18055067	cg04751761	cg10053473
5762	cg18058895	cg04674497	cg03728898
5763	cg18061711	cg04545872	cg06012483
5764	cg18067847	cg04541146	cg22622667
5765	cg18069174	cg04517274	cg23350558
5766	cg18083415	cg04508114	cg06862949
5767	cg18090631	cg04290396	cg26579379
5768	cg18091245	cg04282530	cg03482221
5769	cg18095041	cg04260676	cg19241089
5770	cg18098187	cg04251828	cg14870461
5771	cg18108009	cg04232282	cg22213752
5772	cg18108716	cg04187433	cg00271177
5773	cg18109289	cg04147875	cg27403628
5774	cg18116267	cg04107939	cg14297023

5775	cg18118490	cg04095069	cg05712931
5776	cg18120975	cg04055053	cg20185083
5777	cg18132690	cg03980715	cg14923398
5778	cg18142262	cg03975834	cg16075266
5779	cg18148375	cg03841376	cg23028631
5780	cg18148488	cg03796321	cg14206983
5781	cg18153137	cg03736774	cg10257671
5782	cg18158033	cg03728898	cg24336730
5783	cg18163092	cg03615933	cg25535708
5784	cg18168520	cg03437713	cg09556902
5785	cg18172013	cg03333116	cg27096981
5786	cg18174089	cg03137908	cg15929241
5787	cg18177819	cg03095814	cg14475381
5788	cg18181891	cg03030665	cg04240456
5789	cg18191688	cg02972475	cg18465945
5790	cg18193482	cg02886591	cg26868306
5791	cg18201671	cg02883198	cg26618058
5792	cg18207099	cg02753990	cg14094409
5793	cg18222083	cg02658272	cg10225755
5794	cg18225577	cg02598618	cg08733553
5795	cg18226382	cg02524863	cg20122645
5796	cg18228456	cg02492708	cg06892815
5797	cg18231184	cg02389084	cg06665485
5798	cg18233538	cg02323633	cg25162533
5799	cg18237191	cg02297063	cg03498175
5800	cg18238763	cg02110527	cg17297354
5801	cg18241094	cg02062816	cg13525067
5802	cg18243853	cg02061820	cg24595704
5803	cg18244708	cg02010047	cg07050626
5804	cg18247852	cg01959262	cg13533142
5805	cg18248273	cg01943414	cg20329153
5806	cg18249380	cg01922697	cg10193870
5807	cg18250135	cg01887374	cg15367698
5808	cg18252102	cg01866959	cg06208270
5809	cg18253787	cg01783960	cg00670539
5810	cg18253910	cg01641136	cg17018661
5811	cg18259003	cg01602730	cg00523012
5812	cg18259342	cg01599714	cg21143086
5813	cg18259487	cg01436550	cg26661623
5814	cg18264298	cg01422009	cg00352031
5815	cg18266458	cg01404873	cg08148618
5816	cg18272538	cg01390647	cg26526953
5817	cg18279126	cg01375259	cg13189671
5818	cg18280717	cg01362762	cg09100695
5819	cg18285813	cg01356872	cg13495850
5820	cg18294610	cg01356198	cg04341806
5821	cg18302629	cg01306747	cg26502610
5822	cg18304186	cg01283300	cg25178749
5823	cg18311516	cg01207974	cg10585257
5824	cg18313342	cg01201519	cg18384190
5825	cg18317492	cg01176823	cg05668807
5826	cg18323018	cg01060040	cg13881619
5827	cg18325866	cg01052428	cg03881382

5828	cg18327695	cg00950412	cg26118698
5829	cg18327762	cg00918130	cg08985282
5830	cg18330203	cg00881300	cg19755886
5831	cg18338925	cg00877758	cg27401116
5832	cg18340416	cg00864551	cg03278299
5833	cg18348566	cg00841141	cg04774496
5834	cg18348731	cg00713972	cg18965684
5835	cg18352616	cg00667680	cg25783610
5836	cg18369325	cg00651488	cg17792616
5837	cg18369990	cg00620733	cg12068791
5838	cg18371976	cg00556407	cg15548656
5839	cg18375421	cg00464814	cg01483824
5840	cg18378662	cg00446536	cg19529732
5841	cg18386876	cg00442802	cg10802680
5842	cg18397308	cg00392155	cg06310294
5843	cg18397743	cg00347938	cg03648611
5844	cg18400889	cg00296038	cg03737788
5845	cg18406924	cg00206063	cg27177415
5846	cg18428888	cg00175487	cg25059445
5847	cg18435870	cg00166343	cg07927540
5848	cg18436810	cg00145911	cg14426682
5849	cg18440069	cg00127198	cg03286742
5850	cg18442286	cg00091004	cg06068392
5851	cg18444673	cg00070460	cg03661299
5852	cg18459869	cg27634115	cg06561892
5853	cg18465945	cg27599376	cg06741399
5854	cg18467406	cg27521744	cg20594316
5855	cg18474180	cg27440150	cg13194638
5856	cg18475969	cg27336068	cg14321412
5857	cg18482303	cg27263448	cg09397692
5858	cg18484068	cg27178714	cg24295561
5859	cg18485872	cg27177779	cg26152188
5860	cg18485877	cg27143695	cg23536830
5861	cg18487916	cg27101023	cg16071082
5862	cg18493371	cg27026202	cg02563156
5863	cg18503234	cg27000120	cg05002642
5864	cg18505837	cg26987720	cg00431813
5865	cg18514164	cg26819427	cg20265951
5866	cg18519450	cg26787096	cg21513803
5867	cg18526008	cg26739233	cg25825970
5868	cg18527971	cg26579379	cg23954066
5869	cg18533833	cg26524387	cg05274056
5870	cg18537410	cg26504421	cg03289072
5871	cg18539461	cg26428962	cg26354947
5872	cg18543610	cg26342552	cg21574855
5873	cg18544888	cg26323752	cg08205361
5874	cg18549386	cg26224791	cg20396385
5875	cg18553570	cg25954194	cg11210357
5876	cg18556792	cg25934198	cg07328519
5877	cg18560638	cg25890936	cg27231912
5878	cg18565130	cg25782041	cg11046578
5879	cg18566883	cg25736617	cg13190879
5880	cg18568570	cg25552768	cg07661965

5881	cg18581669	cg25487008	cg26323752
5882	cg18583329	cg25433222	cg15326069
5883	cg18595065	cg25431366	cg02102456
5884	cg18601229	cg25386426	cg14051367
5885	cg18604419	cg25316172	cg09213124
5886	cg18610958	cg25306579	cg01398428
5887	cg18624777	cg25285041	cg22734014
5888	cg18628371	cg25246084	cg17747635
5889	cg18634690	cg25202370	cg00595472
5890	cg18636829	cg25142010	cg09544892
5891	cg18637761	cg25104512	cg15228983
5892	cg18645910	cg25097308	cg21388753
5893	cg18649632	cg25059445	cg04165857
5894	cg18652285	cg24997330	cg21408581
5895	cg18653451	cg24957532	cg25433222
5896	cg18657988	cg24915915	cg05966641
5897	cg18659450	cg24903767	cg22911054
5898	cg18668813	cg24847636	cg14843670
5899	cg18673163	cg24709718	cg25831522
5900	cg18677278	cg24701232	cg02650728
5901	cg18677603	cg24625084	cg24709718
5902	cg18689614	cg24450180	cg08173709
5903	cg18690282	cg24321688	cg21549497
5904	cg18692382	cg24301288	cg07505901
5905	cg18693479	cg24295561	cg01589629
5906	cg18697143	cg24209874	cg22289815
5907	cg18698681	cg24123198	cg18177819
5908	cg18700940	cg24097814	cg01060040
5909	cg18703227	cg24090911	cg25978487
5910	cg18703721	cg24047905	cg12741436
5911	cg18704218	cg23986590	cg15459715
5912	cg18704595	cg23954066	cg26377276
5913	cg18708810	cg23953820	cg21606405
5914	cg18710162	cg23939642	cg11439877
5915	cg18716503	cg23910341	cg07025242
5916	cg18720622	cg23894219	cg18338925
5917	cg18729086	cg23854567	cg26499286
5918	cg18729681	cg23820770	cg24118850
5919	cg18732855	cg23738548	cg00268330
5920	cg18738548	cg23547892	cg03052760
5921	cg18741439	cg23546343	cg20798249
5922	cg18749563	cg23514135	cg20390500
5923	cg18751203	cg23455778	cg20756600
5924	cg18751958	cg23350812	cg11095122
5925	cg18752987	cg23348723	cg08132525
5926	cg18758281	cg23339482	cg18406924
5927	cg18758976	cg23259001	cg21650193
5928	cg18760055	cg23233631	cg17634490
5929	cg18761894	cg23125506	cg02753990
5930	cg18763720	cg22982093	cg26860182
5931	cg18765405	cg22906113	cg00470817
5932	cg18771839	cg22905571	cg14228788
5933	cg18772205	cg22731637	cg16537245

5934	cg18793661	cg22660619	cg16707423
5935	cg18794809	cg22591040	cg12982283
5936	cg18795383	cg22576033	cg20586971
5937	cg18798744	cg22515589	cg27478020
5938	cg18801945	cg22433862	cg06497934
5939	cg18804667	cg22413209	cg08661751
5940	cg18809729	cg22330533	cg02110527
5941	cg18811860	cg22304169	cg15269148
5942	cg18813518	cg22288012	cg08418375
5943	cg18815879	cg22247495	cg24903767
5944	cg18817444	cg22215392	cg00680277
5945	cg18817654	cg22189544	cg06568783
5946	cg18817831	cg22151881	cg08537794
5947	cg18818075	cg22060153	cg16071782
5948	cg18820914	cg22048683	cg05364570
5949	cg18829162	cg22019569	cg12456379
5950	cg18833140	cg21987921	cg02262037
5951	cg18834729	cg21751147	cg25350827
5952	cg18842598	cg21647035	cg07549715
5953	cg18851831	cg21643731	cg14410516
5954	cg18852515	cg21591452	cg21069814
5955	cg18854004	cg21562750	cg03163819
5956	cg18854872	cg21492137	cg06792186
5957	cg18855096	cg21409449	cg21309147
5958	cg18856214	cg21370924	cg23455778
5959	cg18857588	cg21311736	cg15363996
5960	cg18857655	cg21307043	cg00144414
5961	cg18860847	cg21177165	cg09777637
5962	cg18862502	cg21105175	cg21331845
5963	cg18863119	cg21080655	cg09869286
5964	cg18869485	cg21015266	cg25559625
5965	cg18870258	cg20794957	cg14655044
5966	cg18881778	cg20778199	cg27458631
5967	cg18881978	cg20738406	cg02148034
5968	cg18885289	cg20733077	cg23666829
5969	cg18907942	cg20732160	cg09906780
5970	cg18912160	cg20685600	cg02069095
5971	cg18918538	cg20608966	cg23092218
5972	cg18925548	cg20591120	cg12680106
5973	cg18930928	cg20588285	cg06610254
5974	cg18931850	cg20586971	cg12550496
5975	cg18933685	cg20579480	cg22515589
5976	cg18935449	cg20342460	cg13920768
5977	cg18939081	cg20329153	cg19995595
5978	cg18940830	cg20320158	cg24730224
5979	cg18941831	cg20118577	cg01306662
5980	cg18957751	cg20090162	cg04535391
5981	cg18960324	cg20067272	cg01628053
5982	cg18965684	cg19931596	cg22558061
5983	cg18968623	cg19878200	cg20738406
5984	cg18977727	cg19842026	cg16349616
5985	cg18978531	cg19776793	cg17332023
5986	cg18994446	cg19605931	cg10059410

5987	cg18998365	cg19410796	cg15574810
5988	cg18998442	cg19293468	cg13175060
5989	cg19005763	cg19240021	cg15613048
5990	cg19006220	cg19235974	cg25048985
5991	cg19007948	cg19216851	cg07102406
5992	cg19011826	cg19133023	cg14373988
5993	cg19018155	cg19090861	cg24625084
5994	cg19020855	cg19049734	cg08818094
5995	cg19032492	cg19044462	cg03640215
5996	cg19040077	cg19034028	cg17211223
5997	cg19047283	cg19005763	cg16718902
5998	cg19047670	cg18989491	cg24544986
5999	cg19049734	cg18881978	cg00412775
6000	cg19051802	cg18881778	cg05418719
6001	cg19055390	cg18842598	cg07731639
6002	cg19055828	cg18834729	cg17798749
6003	cg19057779	cg18833140	cg13409409
6004	cg19057916	cg18817831	cg26013235
6005	cg19058262	cg18720622	cg26105658
6006	cg19062489	cg18708810	cg25013910
6007	cg19063061	cg18700940	cg26540925
6008	cg19063768	cg18692382	cg10931901
6009	cg19078037	cg18592315	cg09500421
6010	cg19078853	cg18532070	cg09603024
6011	cg19083914	cg18493371	cg11400143
6012	cg19084508	cg18492803	cg23906872
6013	cg19089328	cg18465945	cg23490822
6014	cg19090861	cg18340416	cg05400252
6015	cg19100996	cg18313342	cg04251828
6016	cg19107511	cg18294610	cg16392860
6017	cg19113668	cg18249380	cg11253592
6018	cg19116924	cg18237191	cg25323005
6019	cg19123356	cg18168520	cg19779886
6020	cg19125081	cg17853089	cg09486967
6021	cg19130973	cg17803589	cg16570314
6022	cg19130981	cg17743939	cg00607989
6023	cg19133023	cg17740401	cg16929793
6024	cg19138499	cg17732877	cg03696370
6025	cg19144954	cg17724627	cg03330678
6026	cg19161559	cg17720554	cg18793661
6027	cg19162768	cg17667773	cg17517442
6028	cg19170881	cg17611093	cg03501128
6029	cg19178876	cg17610889	cg27505472
6030	cg19181160	cg17560283	cg24976262
6031	cg19182279	cg17539235	cg04512603
6032	cg19190016	cg17522727	cg23439917
6033	cg19190487	cg17518949	cg02463970
6034	cg19200440	cg17467243	cg04546061
6035	cg19201144	cg17418956	cg17857870
6036	cg19201770	cg17375396	cg23333146
6037	cg19219672	cg17322655	cg26365854
6038	cg19235974	cg17319576	cg10872457
6039	cg19238415	cg17297775	cg21385052

6040	cg19239692	cg17297354	cg21784096
6041	cg19242568	cg17284725	cg16564132
6042	cg19244553	cg17018661	cg06891043
6043	cg19253339	cg16919420	cg16381321
6044	cg19257111	cg16831894	cg26129303
6045	cg19259111	cg16773043	cg02272457
6046	cg19262818	cg16672557	cg07284519
6047	cg19266014	cg16664095	cg23825482
6048	cg19270309	cg16650096	cg16023107
6049	cg19270505	cg16563454	cg21032292
6050	cg19271753	cg16535788	cg03148858
6051	cg19277672	cg16521032	cg14119680
6052	cg19284767	cg16459391	cg17375396
6053	cg19287817	cg16391182	cg00706207
6054	cg19288921	cg16292933	cg24035682
6055	cg19289969	cg16242615	cg08193650
6056	cg19293468	cg16213787	cg08771923
6057	cg19296501	cg16117028	cg02633409
6058	cg19303748	cg16078836	cg06760467
6059	cg19308185	cg15959921	cg22651048
6060	cg19317226	cg15935860	cg21978195
6061	cg19317433	cg15772924	cg24518264
6062	cg19319067	cg15737302	cg26739233
6063	cg19325477	cg15686782	cg09041614
6064	cg19340420	cg15646741	cg02058267
6065	cg19348272	cg15556723	cg25784372
6066	cg19353949	cg15485665	cg18259487
6067	cg19357094	cg15478081	cg19549465
6068	cg19357195	cg15470344	cg22866998
6069	cg19360685	cg15446884	cg22439641
6070	cg19372504	cg15408640	cg24307703
6071	cg19374752	cg15389749	cg05508315
6072	cg19375196	cg15283164	cg19987219
6073	cg19382919	cg15277378	cg24342377
6074	cg19397321	cg15264708	cg16796135
6075	cg19399165	cg15181351	cg21752525
6076	cg19400179	cg15174926	cg23712594
6077	cg19402179	cg15127702	cg01294717
6078	cg19404500	cg15104081	cg06825317
6079	cg19410796	cg15016624	cg04080724
6080	cg19415091	cg14966346	cg01413698
6081	cg19422138	cg14960869	cg25235326
6082	cg19437281	cg14875394	cg11462099
6083	cg19439043	cg14843670	cg13936383
6084	cg19443475	cg14706807	cg19404500
6085	cg19444998	cg14644865	cg27543578
6086	cg19450111	cg14547599	cg16195091
6087	cg19455322	cg14521421	cg24899558
6088	cg19462352	cg14505694	cg19178876
6089	cg19478343	cg14502713	cg14578363
6090	cg19478410	cg14455139	cg01402099
6091	cg19479935	cg14425960	cg03054026
6092	cg19480117	cg14413378	cg16522491

6093	cg19482885	cg14375387	cg13378789
6094	cg19486437	cg14359798	cg24154271
6095	cg19486804	cg14323199	cg10311754
6096	cg19489699	cg14266952	cg02560388
6097	cg19500393	cg14264901	cg27294008
6098	cg19502812	cg14217074	cg00372132
6099	cg19503351	cg14210001	cg21574349
6100	cg19507725	cg14093663	cg16995299
6101	cg19508622	cg14059835	cg14543285
6102	cg19509663	cg14051367	cg12219134
6103	cg19512961	cg14030593	cg05591701
6104	cg19513744	cg14023774	cg19956769
6105	cg19514230	cg13982956	cg20333027
6106	cg19514469	cg13956443	cg07290652
6107	cg19515081	cg13933043	cg23696210
6108	cg19528797	cg13877631	cg27100149
6109	cg19529732	cg13867915	cg26832915
6110	cg19532212	cg13864546	cg18539086
6111	cg19536407	cg13860981	cg26313976
6112	cg19536781	cg13846358	cg11888359
6113	cg19539481	cg13836318	cg13724379
6114	cg19541247	cg13774342	cg19049734
6115	cg19548479	cg13774184	cg21856603
6116	cg19548524	cg13758724	cg02734600
6117	cg19553402	cg13747145	cg14375387
6118	cg19560210	cg13710553	cg12736760
6119	cg19561607	cg13626676	cg08573299
6120	cg19565757	cg13593436	cg14781242
6121	cg19567295	cg13471336	cg24135293
6122	cg19572849	cg13467292	cg26778630
6123	cg19573208	cg13457217	cg06013127
6124	cg19577054	cg13420004	cg15878619
6125	cg19577082	cg13153540	cg07104086
6126	cg19577548	cg13061648	cg08619515
6127	cg19582744	cg12971523	cg12732734
6128	cg19584674	cg12927785	cg05267269
6129	cg19585100	cg12860109	cg07915976
6130	cg19589800	cg12807855	cg19478410
6131	cg19593878	cg12806728	cg25934198
6132	cg19594745	cg12806353	cg26585452
6133	cg19598713	cg12787036	cg20242065
6134	cg19598875	cg12728606	cg02534048
6135	cg19604110	cg12702354	cg14093018
6136	cg19605931	cg12690127	cg08970011
6137	cg19611817	cg12681370	cg26546882
6138	cg19618438	cg12667792	cg12964187
6139	cg19622474	cg12591125	cg27611699
6140	cg19623406	cg12497581	cg01055121
6141	cg19629843	cg12367539	cg18241094
6142	cg19631779	cg12179884	cg26656452
6143	cg19632842	cg12167489	cg06591973
6144	cg19638572	cg12051710	cg15247169
6145	cg19646759	cg11945474	cg19125081

6146	cg19658812	cg11940726	cg17074958
6147	cg19668990	cg11908294	cg15639581
6148	cg19683675	cg11869499	cg10006956
6149	cg19691659	cg11816841	cg18863119
6150	cg19698242	cg11564792	cg13320257
6151	cg19702703	cg11535638	cg27585557
6152	cg19709083	cg11497526	cg02940164
6153	cg19713140	cg11468635	cg21915998
6154	cg19713196	cg11436027	cg21987921
6155	cg19724698	cg11360860	cg23780635
6156	cg19725377	cg11313862	cg00704869
6157	cg19730379	cg11284582	cg24366425
6158	cg19736117	cg11237495	cg01227084
6159	cg19736226	cg11218175	cg18698681
6160	cg19747992	cg11174847	cg18264298
6161	cg19761272	cg11151524	cg09450022
6162	cg19763281	cg11101316	cg05718035
6163	cg19765175	cg11095122	cg09231120
6164	cg19765378	cg11046030	cg16695570
6165	cg19769521	cg11016151	cg02168436
6166	cg19770281	cg10990188	cg22895198
6167	cg19771975	cg10904740	cg15407257
6168	cg19772651	cg10859133	cg18323018
6169	cg19774846	cg10836406	cg10332704
6170	cg19776793	cg10820904	cg00218484
6171	cg19779886	cg10811426	cg05267394
6172	cg19781117	cg10805676	cg23756143
6173	cg19783160	cg10731848	cg06257110
6174	cg19784198	cg10730148	cg20267732
6175	cg19786422	cg10550245	cg23375717
6176	cg19789753	cg10529994	cg25782041
6177	cg19791606	cg10496150	cg17067528
6178	cg19799865	cg10408178	cg24701662
6179	cg19802929	cg10370025	cg01191157
6180	cg19815596	cg10361005	cg13747145
6181	cg19817882	cg10360139	cg06183287
6182	cg19821361	cg10357989	cg11343506
6183	cg19822716	cg10278297	cg12806353
6184	cg19824396	cg10227312	cg19058262
6185	cg19827089	cg10218733	cg05226061
6186	cg19831403	cg10213762	cg13490227
6187	cg19839325	cg10147974	cg23538755
6188	cg19841423	cg10053473	cg27323343
6189	cg19842026	cg10024583	cg16510440
6190	cg19847601	cg10000148	cg16006963
6191	cg19849478	cg09970023	cg04149585
6192	cg19856705	cg09801824	cg16660312
6193	cg19857461	cg09703323	cg01959071
6194	cg19857633	cg09685601	cg24408316
6195	cg19863040	cg09650907	cg13154622
6196	cg19865472	cg09607915	cg26034516
6197	cg19865916	cg09581065	cg27181295
6198	cg19874640	cg09551019	cg19560210

6199	cg19878200	cg09511351	cg06869755
6200	cg19879265	cg09371084	cg25264265
6201	cg19892287	cg09353705	cg06504636
6202	cg19895894	cg09196257	cg07384019
6203	cg19909613	cg09109553	cg02872491
6204	cg19912309	cg09037712	cg26015115
6205	cg19918057	cg09020697	cg03035359
6206	cg19925801	cg08976646	cg10611016
6207	cg19931529	cg08947774	cg01835854
6208	cg19931596	cg08933276	cg15410236
6209	cg19937288	cg08883078	cg01338955
6210	cg19940729	cg08836353	cg18645910
6211	cg19944656	cg08741843	cg14003211
6212	cg19949955	cg08740008	cg00095259
6213	cg19950556	cg08636203	cg24448421
6214	cg19961522	cg08622923	cg07714708
6215	cg19970953	cg08605347	cg26890480
6216	cg19974227	cg08509383	cg15127656
6217	cg19975759	cg08494755	cg03143347
6218	cg19987219	cg08457169	cg02878891
6219	cg19988492	cg08437802	cg17482237
6220	cg19991046	cg08428985	cg18492803
6221	cg19992808	cg08381596	cg09335314
6222	cg19995595	cg08362308	cg01449425
6223	cg20000539	cg08197880	cg14219236
6224	cg20000602	cg08169341	cg09109553
6225	cg20000866	cg08124986	cg17654747
6226	cg20005923	cg08104146	cg25749254
6227	cg20008846	cg07946977	cg12240761
6228	cg20010135	cg07800359	cg23046918
6229	cg20011326	cg07714708	cg19629843
6230	cg20017253	cg07654843	cg10865444
6231	cg20018563	cg07622079	cg19565757
6232	cg20027456	cg07520608	cg12806728
6233	cg20027784	cg07507493	cg20731110
6234	cg20030934	cg07478501	cg16265932
6235	cg20048529	cg07420867	cg22272218
6236	cg20049727	cg07385490	cg12790592
6237	cg20051177	cg07384019	cg16517702
6238	cg20060759	cg07365071	cg01810684
6239	cg20061812	cg07352757	cg27637521
6240	cg20067272	cg07308243	cg14037937
6241	cg20067780	cg07104557	cg19123356
6242	cg20068058	cg07048516	cg06887283
6243	cg20069939	cg07005960	cg14001518
6244	cg20070852	cg06817454	cg14294646
6245	cg20076442	cg06769510	cg26909797
6246	cg20078454	cg06746318	cg21307043
6247	cg20080282	cg06706550	cg13070215
6248	cg20090162	cg06649282	cg08180831
6249	cg20090290	cg06541938	cg17276624
6250	cg20095124	cg06537744	cg04196068
6251	cg20099458	cg06463230	cg18583276

6252	cg20100049	cg06460400	cg14620138
6253	cg20102955	cg06439736	cg06321304
6254	cg20118577	cg06340118	cg19270505
6255	cg20133730	cg06328338	cg00528490
6256	cg20137441	cg06310294	cg21735384
6257	cg20138711	cg06292709	cg09905732
6258	cg20139145	cg06252645	cg06604690
6259	cg20140662	cg06120235	cg18000764
6260	cg20141509	cg06094523	cg23900422
6261	cg20143111	cg05931119	cg09856367
6262	cg20147164	cg05915622	cg26071556
6263	cg20149780	cg05851042	cg23529249
6264	cg20152304	cg05755441	cg18158033
6265	cg20156139	cg05738687	cg15231872
6266	cg20158671	cg05727180	cg14575741
6267	cg20161791	cg05662839	cg27003787
6268	cg20165037	cg05615230	cg10109146
6269	cg20166714	cg05601844	cg02083676
6270	cg20167126	cg05517824	cg26304689
6271	cg20169015	cg05515072	cg05069728
6272	cg20173014	cg05493407	cg06264060
6273	cg20185083	cg05364570	cg19190487
6274	cg20188546	cg05350020	cg07122529
6275	cg20189937	cg05346688	cg17426166
6276	cg20193802	cg05305993	cg01330448
6277	cg20195046	cg05289897	cg06799533
6278	cg20197694	cg05220083	cg22550309
6279	cg20197756	cg05141289	cg12265604
6280	cg20208009	cg05091570	cg06445533
6281	cg20210151	cg05073386	cg16277922
6282	cg20210637	cg05062820	cg01758022
6283	cg20226512	cg04966586	cg00667680
6284	cg20227592	cg04874782	cg18108009
6285	cg20229025	cg04837783	cg20229025
6286	cg20230305	cg04777612	cg01637244
6287	cg20231900	cg04745820	cg11662712
6288	cg20241952	cg04717045	cg06772578
6289	cg20242065	cg04679114	cg03031182
6290	cg20252837	cg04612073	cg19034028
6291	cg20254251	cg04601090	cg07732097
6292	cg20262021	cg04590974	cg20349803
6293	cg20265951	cg04568774	cg23345038
6294	cg20267732	cg04512603	cg17196448
6295	cg20283716	cg04505897	cg25146042
6296	cg20284937	cg04437605	cg18729086
6297	cg20293725	cg04399631	cg07156484
6298	cg20297053	cg04390667	cg26666292
6299	cg20297670	cg04353997	cg15937073
6300	cg20300776	cg04349985	cg21017775
6301	cg20300794	cg04334792	cg20137237
6302	cg20309339	cg04194173	cg19216851
6303	cg20309371	cg04156643	cg03216474
6304	cg20316440	cg04149585	cg23797615

6305	cg20320158	cg04141218	cg07204803
6306	cg20322433	cg04140297	cg08914150
6307	cg20322837	cg04110886	cg17673769
6308	cg20329303	cg03965207	cg16391347
6309	cg20333027	cg03938110	cg16167741
6310	cg20334115	cg03818193	cg14078730
6311	cg20336472	cg03808580	cg03231163
6312	cg20338075	cg03793055	cg06179971
6313	cg20338754	cg03773809	cg19841423
6314	cg20342460	cg03761162	cg23171099
6315	cg20349803	cg03736944	cg05978010
6316	cg20354777	cg03680680	cg10018519
6317	cg20370991	cg03654273	cg23252698
6318	cg20379239	cg03639964	cg12467435
6319	cg20382418	cg03608003	cg11527930
6320	cg20382493	cg03529015	cg13420647
6321	cg20390500	cg03484435	cg03177023
6322	cg20396385	cg03438101	cg09746044
6323	cg20397614	cg03400139	cg11995490
6324	cg20407868	cg03244796	cg19573208
6325	cg20412217	cg03215657	cg18659450
6326	cg20415947	cg03199926	cg04703974
6327	cg20418529	cg03108347	cg23202545
6328	cg20418769	cg03089923	cg07872947
6329	cg20426710	cg03029255	cg13973594
6330	cg20428133	cg03017264	cg09007161
6331	cg20435464	cg02958947	cg10841956
6332	cg20436707	cg02910959	cg01467882
6333	cg20437892	cg02742455	cg00606396
6334	cg20438460	cg02733842	cg12742209
6335	cg20441046	cg02730804	cg05757474
6336	cg20447038	cg02725453	cg25780982
6337	cg20449048	cg02659431	cg18266458
6338	cg20459543	cg02627286	cg27158462
6339	cg20462191	cg02613295	cg02875487
6340	cg20463808	cg02588098	cg23949216
6341	cg20464155	cg02509027	cg16056849
6342	cg20471017	cg02493905	cg01809895
6343	cg20476021	cg02454890	cg06212213
6344	cg20482280	cg02244933	cg25289803
6345	cg20490386	cg02225004	cg12284521
6346	cg20495333	cg02157454	cg27233847
6347	cg20505457	cg02102456	cg13931531
6348	cg20512713	cg02084948	cg04269351
6349	cg20513080	cg02084214	cg11743827
6350	cg20515846	cg02060434	cg16908801
6351	cg20516209	cg02025435	cg06521357
6352	cg20519665	cg02021485	cg17304712
6353	cg20524889	cg01959071	cg01599714
6354	cg20540235	cg01946510	cg03051577
6355	cg20540306	cg01932663	cg16024937
6356	cg20544097	cg01924223	cg13319468
6357	cg20544406	cg01885814	cg23617947

6358	cg20548182	cg01852611	cg11360860
6359	cg20560906	cg01810684	cg10664162
6360	cg20566286	cg01792229	cg19765378
6361	cg20566450	cg01791238	cg27263448
6362	cg20567148	cg01741616	cg02197542
6363	cg20571592	cg01719663	cg05869732
6364	cg20579480	cg01645401	cg13728069
6365	cg20586971	cg01578585	cg02807008
6366	cg20588285	cg01534871	cg20193802
6367	cg20592766	cg01370334	cg14419424
6368	cg20593887	cg01364674	cg09258240
6369	cg20594316	cg01360115	cg04804648
6370	cg20595846	cg01337736	cg10453420
6371	cg20600205	cg01324452	cg17838182
6372	cg20602417	cg01314597	cg25271479
6373	cg20608966	cg01303055	cg21736854
6374	cg20634514	cg01287012	cg26653360
6375	cg20637405	cg01260603	cg00947413
6376	cg20647485	cg01195672	cg12367539
6377	cg20650515	cg01154505	cg25920512
6378	cg20654462	cg01110955	cg07353266
6379	cg20658711	cg01096199	cg10063637
6380	cg20662658	cg01077713	cg13668823
6381	cg20663495	cg01066494	cg22243439
6382	cg20664445	cg01062651	cg18474180
6383	cg20667709	cg01044293	cg24673886
6384	cg20668447	cg01007781	cg22280406
6385	cg20668718	cg00999267	cg03142002
6386	cg20669366	cg00941900	cg24678812
6387	cg20675852	cg00906241	cg20138711
6388	cg20677267	cg00827581	cg16820411
6389	cg20677814	cg00825317	cg27440150
6390	cg20679011	cg00816182	cg12642717
6391	cg20679271	cg00748072	cg14166377
6392	cg20679659	cg00718694	cg26721908
6393	cg20686479	cg00665106	cg05767299
6394	cg20690695	cg00656603	cg05757530
6395	cg20692268	cg00622702	cg08134301
6396	cg20700977	cg00586700	cg03129134
6397	cg20703928	cg00513981	cg14201387
6398	cg20713092	cg00469547	cg11218175
6399	cg20723705	cg00460268	cg01580176
6400	cg20725704	cg00431813	cg24657347
6401	cg20731110	cg00418613	cg16734433
6402	cg20732160	cg00373245	cg26207909
6403	cg20733077	cg00341297	cg14081251
6404	cg20733739	cg00286717	cg21171279
6405	cg20738406	cg00271177	cg20188546
6406	cg20739726	cg00209623	cg19598713
6407	cg20741105	cg00201819	cg04007841
6408	cg20745684	cg00185413	cg26970113
6409	cg20751706	cg00147160	cg20095124
6410	cg20756600	cg00089486	cg08365802

6411	cg20757748	cg00013441	cg14839830
6412	cg20758651	cg27662093	cg07391023
6413	cg20761844	cg27655512	cg00877758
6414	cg20767977	cg27571329	cg20668447
6415	cg20771808	cg27565803	cg08894401
6416	cg20773915	cg27434984	cg05680710
6417	cg20776543	cg27403628	cg21067905
6418	cg20779414	cg27290103	cg08818866
6419	cg20780180	cg27273675	cg00360324
6420	cg20781516	cg27216597	cg10818702
6421	cg20792436	cg27146477	cg07022478
6422	cg20793665	cg27082076	cg04575609
6423	cg20794957	cg27049344	cg08360599
6424	cg20798249	cg26974415	cg24474622
6425	cg20805475	cg26974276	cg02972941
6426	cg20806143	cg26969840	cg15401523
6427	cg20808227	cg26931296	cg00086171
6428	cg20812052	cg26927190	cg05088513
6429	cg20822767	cg26909797	cg13210578
6430	cg20826610	cg26888672	cg14438213
6431	cg20828052	cg26767085	cg22679474
6432	cg20840795	cg26726230	cg16311946
6433	cg20847114	cg26697605	cg10632728
6434	cg20849855	cg26673975	cg09781414
6435	cg20852364	cg26666292	cg26180843
6436	cg20861822	cg26585320	cg16953473
6437	cg20863668	cg26573923	cg14328761
6438	cg20881888	cg26543243	cg17172949
6439	cg20892492	cg26540925	cg14136214
6440	cg20899781	cg26529655	cg02665727
6441	cg20909380	cg26419962	cg10796068
6442	cg20910352	cg26398024	cg20739726
6443	cg20916068	cg26361671	cg19415091
6444	cg20916646	cg26315985	cg02730804
6445	cg20918463	cg26267483	cg00626390
6446	cg20925383	cg26234644	cg26642540
6447	cg20934096	cg26222498	cg06541938
6448	cg20937296	cg26218835	cg07078758
6449	cg20941528	cg26214948	cg21063361
6450	cg20951650	cg26172211	cg14947411
6451	cg20952105	cg26152017	cg25190718
6452	cg20958959	cg26129303	cg26769720
6453	cg20963020	cg26079864	cg17830812
6454	cg20967819	cg26026296	cg17938607
6455	cg20971998	cg25945504	cg03110167
6456	cg20977024	cg25921543	cg08521178
6457	cg20985758	cg25903122	cg16102706
6458	cg20989443	cg25892761	cg06419732
6459	cg20997320	cg25875209	cg11725852
6460	cg21000919	cg25784372	cg02468821
6461	cg21004924	cg25765819	cg07438999
6462	cg21006686	cg25739142	cg27014538
6463	cg21010028	cg25568490	cg24433265

6464	cg21011702	cg25568066	cg00437969
6465	cg21011883	cg25556841	cg04914198
6466	cg21015266	cg25545917	cg27177983
6467	cg21017775	cg25515317	cg23197939
6468	cg21031345	cg25513535	cg03078718
6469	cg21032292	cg25461775	cg14899960
6470	cg21032564	cg25418363	cg25011577
6471	cg21035368	cg25352714	cg27395391
6472	cg21040069	cg25077823	cg26060535
6473	cg21043481	cg24996315	cg24349804
6474	cg21045072	cg24706522	cg17101681
6475	cg21048763	cg24697460	cg26002713
6476	cg21057429	cg24635581	cg05816041
6477	cg21063361	cg24631518	cg04287574
6478	cg21067905	cg24630426	cg20068058
6479	cg21069814	cg24553775	cg04628014
6480	cg21070669	cg24523322	cg23133355
6481	cg21080655	cg24518609	cg05949181
6482	cg21089789	cg24517989	cg21108851
6483	cg21092828	cg24453600	cg08841613
6484	cg21095280	cg24433265	cg04547809
6485	cg21105175	cg24360745	cg04023831
6486	cg21118367	cg24336730	cg14616881
6487	cg21133992	cg24307703	cg15336269
6488	cg21143086	cg24283049	cg23598378
6489	cg21145938	cg24244854	cg20528338
6490	cg21155834	cg24225455	cg16106427
6491	cg21156057	cg24202221	cg02162605
6492	cg21158528	cg24181389	cg03535239
6493	cg21158737	cg24163668	cg24319076
6494	cg21159068	cg24155123	cg22450662
6495	cg21163714	cg24129923	cg09580859
6496	cg21163717	cg24096323	cg08037719
6497	cg21165519	cg24092282	cg24194413
6498	cg21168101	cg24031446	cg16975812
6499	cg21170978	cg23935261	cg03381007
6500	cg21171279	cg23824902	cg10877385
6501	cg21176488	cg23820560	cg19512961
6502	cg21177165	cg23800573	cg19827089
6503	cg21177940	cg23709782	cg25938735
6504	cg21185187	cg23400222	cg22136038
6505	cg21189849	cg23363971	cg20099458
6506	cg21196487	cg23309670	cg18058895
6507	cg21201494	cg23301563	cg13070650
6508	cg21205855	cg23202545	cg26194092
6509	cg21211480	cg23128056	cg06870740
6510	cg21214613	cg23075506	cg11174847
6511	cg21222559	cg23049758	cg23681616
6512	cg21223341	cg22979083	cg13537940
6513	cg21225667	cg22782017	cg03961998
6514	cg21228259	cg22735889	cg00229868
6515	cg21230793	cg22734014	cg25952596
6516	cg21235119	cg22679890	cg21222559

6517	cg21238609	cg22666223	cg18819574
6518	cg21241424	cg22651048	cg12058043
6519	cg21242508	cg22616343	cg25203085
6520	cg21242663	cg22599005	cg20309339
6521	cg21244322	cg22584580	cg05180954
6522	cg21246624	cg22582721	cg15957394
6523	cg21247923	cg22532155	cg26037660
6524	cg21251203	cg22490722	cg21511816
6525	cg21252105	cg22295745	cg00785831
6526	cg21255128	cg22280406	cg22906113
6527	cg21268578	cg22252999	cg25352924
6528	cg21268659	cg22237707	cg10121745
6529	cg21269763	cg22213752	cg09033641
6530	cg21270847	cg22192454	cg09891393
6531	cg21273407	cg22119466	cg17254904
6532	cg21274136	cg22046535	cg26030804
6533	cg21276022	cg21964551	cg13446070
6534	cg21279756	cg21945949	cg13662225
6535	cg21282907	cg21925748	cg12362980
6536	cg21286967	cg21794816	cg09581065
6537	cg21292981	cg21787369	cg01716084
6538	cg21304766	cg21779805	cg12835599
6539	cg21309147	cg21730805	cg06208229
6540	cg21311736	cg21676780	cg20771808
6541	cg21316772	cg21617218	cg02667880
6542	cg21329649	cg21585138	cg17851345
6543	cg21331845	cg21535670	cg08065374
6544	cg21332304	cg21404878	cg26637901
6545	cg21334198	cg21388753	cg08124923
6546	cg21335012	cg21316772	cg20586531
6547	cg21351483	cg21309147	cg04813695
6548	cg21352006	cg21274136	cg10146112
6549	cg21361322	cg21244322	cg06884199
6550	cg21364077	cg21223341	cg08779982
6551	cg21370924	cg21214613	cg14879569
6552	cg21373806	cg21156057	cg15470344
6553	cg21374048	cg21026566	cg03135948
6554	cg21376658	cg21010028	cg24453600
6555	cg21377260	cg20909380	cg10897631
6556	cg21379004	cg20776543	cg18693479
6557	cg21385052	cg20721135	cg05738687
6558	cg21385606	cg20634514	cg14429457
6559	cg21386766	cg20512713	cg24092444
6560	cg21388753	cg20482280	cg00248856
6561	cg21389456	cg20463808	cg14694342
6562	cg21393818	cg20387087	cg05368595
6563	cg21397287	cg20338754	cg18285813
6564	cg21400851	cg20284937	cg24455383
6565	cg21404476	cg20189937	cg01949455
6566	cg21404878	cg20161791	cg27239280
6567	cg21406967	cg20102955	cg18771839
6568	cg21409449	cg20090290	cg03666500
6569	cg21422871	cg20067780	cg13984005

6570	cg21434355	cg20027456	cg11258489
6571	cg21437521	cg20011326	cg01288155
6572	cg21438602	cg19940729	cg00786138
6573	cg21439672	cg19937288	cg13413247
6574	cg21442998	cg19895894	cg07754347
6575	cg21445553	cg19883388	cg09687417
6576	cg21457110	cg19865916	cg06443231
6577	cg21460582	cg19856705	cg23108580
6578	cg21460686	cg19847601	cg22099441
6579	cg21464492	cg19824396	cg05317605
6580	cg21474062	cg19815596	cg24126880
6581	cg21475781	cg19767562	cg23687021
6582	cg21492137	cg19702703	cg10057853
6583	cg21497060	cg19577548	cg25122824
6584	cg21497164	cg19507725	cg11706540
6585	cg21500064	cg19443475	cg22961292
6586	cg21504064	cg19432283	cg24747427
6587	cg21513803	cg19374752	cg00585698
6588	cg21526773	cg19357094	cg03933756
6589	cg21528710	cg19317433	cg17203290
6590	cg21528927	cg19201144	cg00259518
6591	cg21532408	cg19181160	cg12267069
6592	cg21535670	cg19100996	cg03538922
6593	cg21535761	cg19078853	cg09685601
6594	cg21544633	cg19032492	cg02696830
6595	cg21546522	cg18968623	cg11116288
6596	cg21546950	cg18925548	cg24104241
6597	cg21552014	cg18870258	cg04872027
6598	cg21557473	cg18818075	cg14598025
6599	cg21558509	cg18804667	cg17589079
6600	cg21560157	cg18771839	cg23206032
6601	cg21562750	cg18766210	cg00869582
6602	cg21574349	cg18703227	cg08480739
6603	cg21585138	cg18659450	cg05711037
6604	cg21591452	cg18624777	cg04541146
6605	cg21593030	cg18583329	cg00226615
6606	cg21593628	cg18519450	cg10977734
6607	cg21597937	cg18484068	cg13708635
6608	cg21611093	cg18400889	cg24925945
6609	cg21617218	cg18318722	cg24606594
6610	cg21618273	cg18240528	cg27101023
6611	cg21619325	cg18051825	cg11459133
6612	cg21620968	cg18036710	cg20341998
6613	cg21637392	cg17976731	cg18118490
6614	cg21642054	cg17947599	cg24047905
6615	cg21643731	cg17943672	cg08826080
6616	cg21647035	cg17876645	cg04924028
6617	cg21648069	cg17854510	cg02353448
6618	cg21650193	cg17830812	cg18243853
6619	cg21667939	cg17830810	cg26132163
6620	cg21676780	cg17636309	cg09035930
6621	cg21687003	cg17367884	cg26071135
6622	cg21697252	cg17334762	cg12093220

6623	cg21719704	cg17315679	cg01804278
6624	cg21725701	cg17289222	cg12166802
6625	cg21727276	cg17264541	cg08775774
6626	cg21730805	cg17095737	cg01942927
6627	cg21735384	cg17074958	cg13055385
6628	cg21736854	cg17036641	cg26529094
6629	cg21740826	cg16989380	cg05016508
6630	cg21747549	cg16960291	cg14260162
6631	cg21747652	cg16949378	cg26977086
6632	cg21751147	cg16874347	cg04099803
6633	cg21752525	cg16828491	cg09043524
6634	cg21756355	cg16776981	cg27423208
6635	cg21756765	cg16726039	cg27146036
6636	cg21757281	cg16681436	cg14811014
6637	cg21759907	cg16580681	cg07586345
6638	cg21768835	cg16566899	cg01697623
6639	cg21770322	cg16533510	cg17315679
6640	cg21779805	cg16532399	cg20999795
6641	cg21784096	cg16476700	cg18977727
6642	cg21787369	cg16289101	cg00906241
6643	cg21789440	cg16269776	cg00545796
6644	cg21793437	cg16212219	cg00423871
6645	cg21794816	cg16145211	cg03260744
6646	cg21805118	cg16052388	cg19513582
6647	cg21806238	cg16026212	cg03991120
6648	cg21806750	cg16016960	cg20285745
6649	cg21811911	cg15970890	cg13483248
6650	cg21818807	cg15937073	cg25556841
6651	cg21831649	cg15888693	cg12938917
6652	cg21837323	cg15879620	cg27538859
6653	cg21840948	cg15874302	cg06969777
6654	cg21845869	cg15657100	cg26007445
6655	cg21847393	cg15656769	cg26419962
6656	cg21850879	cg15632675	cg25311162
6657	cg21854332	cg15603885	cg22122013
6658	cg21855211	cg15473017	cg03472610
6659	cg21856603	cg15363996	cg07020987
6660	cg21863499	cg15363973	cg02157002
6661	cg21870038	cg15351186	cg00688539
6662	cg21874862	cg15299233	cg20027331
6663	cg21879102	cg15264991	cg09540676
6664	cg21881364	cg15231872	cg01856887
6665	cg21889263	cg15170442	cg22748815
6666	cg21893764	cg15142430	cg01298912
6667	cg21897315	cg15048900	cg00008452
6668	cg21903817	cg14952488	cg19051802
6669	cg21906519	cg14923398	cg23782083
6670	cg21915998	cg14879279	cg17724175
6671	cg21927420	cg14790609	cg20149780
6672	cg21929969	cg14785464	cg07038822
6673	cg21936959	cg14700524	cg01432405
6674	cg21942532	cg14692854	cg02481307
6675	cg21947590	cg14628049	cg11633461

6676	cg21962025	cg14610776	cg20725704
6677	cg21962953	cg14578363	cg24257168
6678	cg21978195	cg14577373	cg01971085
6679	cg21979773	cg14469436	cg25245161
6680	cg21987921	cg14403241	cg08591538
6681	cg21997465	cg14350114	cg06058262
6682	cg21998505	cg14340089	cg05975710
6683	cg22011888	cg14337655	cg25082361
6684	cg22013790	cg14334310	cg25835936
6685	cg22016731	cg14331206	cg04696969
6686	cg22019569	cg14329059	cg00902427
6687	cg22020227	cg14320530	cg25399818
6688	cg22021794	cg14288326	cg01370334
6689	cg22022181	cg14287742	cg05150667
6690	cg22024295	cg14282937	cg05013912
6691	cg22039204	cg14249876	cg08159271
6692	cg22040672	cg14204738	cg13731108
6693	cg22046535	cg14204266	cg27475586
6694	cg22047282	cg14178294	cg20152304
6695	cg22047338	cg14178043	cg17778120
6696	cg22047766	cg14119680	cg11804414
6697	cg22048683	cg14095283	cg00035923
6698	cg22049753	cg14059665	cg07691914
6699	cg22052672	cg14010305	cg03013188
6700	cg22058664	cg14003211	cg00503920
6701	cg22060153	cg13980079	cg16509072
6702	cg22069262	cg13957126	cg13726456
6703	cg22078934	cg13951223	cg21311736
6704	cg22082745	cg13857933	cg02187822
6705	cg22083335	cg13823144	cg22584582
6706	cg22095128	cg13786171	cg03782662
6707	cg22097225	cg13763482	cg08103691
6708	cg22099441	cg13672348	cg03215657
6709	cg22108360	cg13658093	cg12293340
6710	cg22118416	cg13653963	cg03267954
6711	cg22119466	cg13642849	cg22944823
6712	cg22122013	cg13639866	cg22660619
6713	cg22123915	cg13630560	cg00059024
6714	cg22133366	cg13542073	cg00783706
6715	cg22150680	cg13533142	cg22838354
6716	cg22151881	cg13509456	cg02367992
6717	cg22153312	cg13490227	cg16919420
6718	cg22154992	cg13476204	cg09198138
6719	cg22161706	cg13475704	cg23601586
6720	cg22169866	cg13472900	cg08119777
6721	cg22171607	cg13429555	cg25627714
6722	cg22186291	cg13420647	cg10088715
6723	cg22189544	cg13413247	cg17886413
6724	cg22192454	cg13409409	cg17524821
6725	cg22194948	cg13223682	cg26685539
6726	cg22197033	cg13210578	cg02192555
6727	cg22202031	cg13190879	cg26144207
6728	cg22204453	cg13175060	cg02010686

6729	cg22213752	cg13116061	cg25575145
6730	cg22215392	cg13096278	cg26969840
6731	cg22217449	cg13086402	cg24613083
6732	cg22221847	cg13080151	cg08590257
6733	cg22237707	cg13054419	cg18019825
6734	cg22240998	cg13050716	cg25625457
6735	cg22243439	cg13039539	cg02469916
6736	cg22245494	cg13038544	cg25921543
6737	cg22247495	cg12964187	cg24424381
6738	cg22251048	cg12843033	cg12069132
6739	cg22252999	cg12590668	cg03984919
6740	cg22253401	cg12456379	cg14165633
6741	cg22255690	cg12204166	cg17817350
6742	cg22272218	cg12136716	cg12417362
6743	cg22277106	cg12075498	cg13716849
6744	cg22280406	cg12049462	cg09459955
6745	cg22288012	cg12025243	cg05415131
6746	cg22289815	cg11976592	cg09018739
6747	cg22295745	cg11844464	cg16681083
6748	cg22304169	cg11706540	cg08062713
6749	cg22305797	cg11671308	cg11601967
6750	cg22315164	cg11632592	cg11583751
6751	cg22323067	cg11601967	cg16902190
6752	cg22329743	cg11524428	cg08975850
6753	cg22330533	cg11497017	cg08365388
6754	cg22342925	cg11490944	cg14016879
6755	cg22343299	cg11468233	cg07511723
6756	cg22345063	cg11462099	cg26974441
6757	cg22356839	cg11409060	cg13517605
6758	cg22365446	cg11341086	cg10065736
6759	cg22366375	cg11319760	cg07841877
6760	cg22371518	cg11272874	cg13823144
6761	cg22376204	cg11252765	cg21269763
6762	cg22379668	cg11087939	cg19503351
6763	cg22380476	cg11046578	cg23784046
6764	cg22387323	cg11041314	cg21676780
6765	cg22396663	cg10997906	cg03555203
6766	cg22397446	cg10925829	cg24711397
6767	cg22402769	cg10841956	cg04456245
6768	cg22413209	cg10828599	cg10571824
6769	cg22414151	cg10810290	cg26172211
6770	cg22418565	cg10810026	cg12526346
6771	cg22427797	cg10709026	cg10089081
6772	cg22429169	cg10673740	cg12413156
6773	cg22430861	cg10664618	cg14474966
6774	cg22433862	cg10563645	cg04558973
6775	cg22436253	cg10511902	cg19432283
6776	cg22439641	cg10488292	cg14547599
6777	cg22442617	cg10472263	cg19332409
6778	cg22448292	cg10458392	cg03344672
6779	cg22449085	cg10427430	cg09782637
6780	cg22450662	cg10392840	cg10555744
6781	cg22451265	cg10378795	cg24249775

6782	cg22459052	cg10370426	cg21616243
6783	cg22459081	cg10370305	cg21962953
6784	cg22461758	cg10332437	cg24209874
6785	cg22466400	cg10327980	cg17775332
6786	cg22472360	cg10273135	cg15488570
6787	cg22482278	cg10131972	cg11864076
6788	cg22488259	cg10107322	cg04473209
6789	cg22490722	cg10036892	cg00919689
6790	cg22501449	cg10016364	cg04880940
6791	cg22512847	cg09982918	cg08877591
6792	cg22515589	cg09932436	cg10194844
6793	cg22516162	cg09915232	cg14864276
6794	cg22521416	cg09905732	cg16668534
6795	cg22525688	cg09901532	cg03417342
6796	cg22528270	cg09901529	cg25104512
6797	cg22532155	cg09896867	cg03683994
6798	cg22532194	cg09891393	cg01454538
6799	cg22539584	cg09886931	cg12591125
6800	cg22542420	cg09812790	cg01017397
6801	cg22546818	cg09809922	cg03031073
6802	cg22550309	cg09790512	cg24939470
6803	cg22555262	cg09742751	cg26815121
6804	cg22558061	cg09711421	cg06407441
6805	cg22562003	cg09569347	cg19585100
6806	cg22563907	cg09540676	cg02693127
6807	cg22575379	cg09510085	cg10213762
6808	cg22576033	cg09397692	cg19822716
6809	cg22579075	cg09300185	cg07741192
6810	cg22582721	cg09249800	cg11699517
6811	cg22584580	cg09248660	cg01267373
6812	cg22584582	cg09216143	cg13832457
6813	cg22591040	cg09142117	cg00920069
6814	cg22593342	cg09139451	cg02648064
6815	cg22599005	cg09033641	cg13584383
6816	cg22616343	cg09007161	cg04398861
6817	cg22619784	cg08975850	cg11661534
6818	cg22622164	cg08912841	cg10202788
6819	cg22622505	cg08749599	cg17256760
6820	cg22622667	cg08550729	cg06981182
6821	cg22628240	cg08545132	cg00550617
6822	cg22639325	cg08524717	cg21847393
6823	cg22644984	cg08480739	cg26408861
6824	cg22651048	cg08476843	cg25301103
6825	cg22655196	cg08343075	cg24429081
6826	cg22657772	cg08307171	cg00085256
6827	cg22660619	cg08288016	cg11248182
6828	cg22666103	cg08277216	cg15678662
6829	cg22666223	cg08273640	cg15712304
6830	cg22679474	cg08261841	cg03457485
6831	cg22679890	cg08149747	cg09602138
6832	cg22685009	cg08119777	cg04372182
6833	cg22686523	cg07959741	cg10980293
6834	cg22699768	cg07921503	cg12807855

6835	cg22700246	cg07910945	cg22118369
6836	cg22700848	cg07904865	cg26421310
6837	cg22706186	cg07867517	cg06622892
6838	cg22720481	cg07803375	cg10335736
6839	cg22730007	cg07736115	cg26923754
6840	cg22731637	cg07582655	cg08218971
6841	cg22731981	cg07552868	cg15776300
6842	cg22734014	cg07549715	cg01677310
6843	cg22735889	cg07547336	cg17732877
6844	cg22737001	cg07530947	cg03988952
6845	cg22743617	cg07478111	cg23351271
6846	cg22748815	cg07471052	cg08108655
6847	cg22750845	cg07396047	cg22323067
6848	cg22752049	cg07338476	cg01675694
6849	cg22761077	cg07329131	cg16608596
6850	cg22762745	cg07267984	cg14001914
6851	cg22764700	cg07166333	cg03131732
6852	cg22765117	cg07148207	cg01439112
6853	cg22780675	cg07077978	cg04582672
6854	cg22782017	cg07067280	cg11205589
6855	cg22786631	cg07058109	cg13885965
6856	cg22786667	cg07043952	cg03162779
6857	cg22788065	cg06943835	cg22097225
6858	cg22798362	cg06931444	cg01596854
6859	cg22800332	cg06907347	cg13582959
6860	cg22803510	cg06894011	cg23010693
6861	cg22809726	cg06863830	cg25439867
6862	cg22811647	cg06799533	cg19303748
6863	cg22814801	cg06776976	cg14102880
6864	cg22819824	cg06753827	cg04194173
6865	cg22822599	cg06753050	cg27234648
6866	cg22831256	cg06746774	cg24697460
6867	cg22834511	cg06711298	cg00209918
6868	cg22838354	cg06702537	cg17931529
6869	cg22845855	cg06660116	cg19078037
6870	cg22846498	cg06607764	cg22512847
6871	cg22846611	cg06522179	cg02283353
6872	cg22861317	cg06355908	cg25141943
6873	cg22866998	cg06246999	cg01992684
6874	cg22867608	cg06090895	cg08206098
6875	cg22871566	cg06038180	cg17678224
6876	cg22877380	cg06033488	cg22365446
6877	cg22888023	cg06024540	cg04839616
6878	cg22890896	cg05976168	cg25950520
6879	cg22895198	cg05968917	cg19161559
6880	cg22904096	cg05966978	cg21863499
6881	cg22905571	cg05960932	cg25552768
6882	cg22906113	cg05846633	cg08489478
6883	cg22908423	cg05801879	cg19397321
6884	cg22908922	cg05800368	cg22448292
6885	cg22909085	cg05771342	cg06996896
6886	cg22911054	cg05770240	cg18738548
6887	cg22913843	cg05714748	cg20370991

6888	cg22916722	cg05661968	cg18461635
6889	cg22918700	cg05564438	cg07329131
6890	cg22921096	cg05546878	cg06793277
6891	cg22932649	cg05529922	cg15444149
6892	cg22937762	cg05504354	cg10980495
6893	cg22942245	cg05418719	cg24391931
6894	cg22944823	cg05336268	cg23454517
6895	cg22961292	cg05288253	cg06292709
6896	cg22964758	cg05280797	cg15473017
6897	cg22971402	cg05279866	cg25883020
6898	cg22974467	cg05205532	cg13433644
6899	cg22977745	cg05070608	cg11628781
6900	cg22979083	cg05054460	cg22253401
6901	cg22979433	cg05014846	cg16824603
6902	cg22982093	cg04990420	cg09672200
6903	cg22986770	cg04907595	cg01404873
6904	cg22986999	cg04820362	cg16395892
6905	cg22987457	cg04744514	cg27143326
6906	cg22989033	cg04731861	cg20555305
6907	cg22990430	cg04684246	cg24073352
6908	cg22990726	cg04607844	cg05541267
6909	cg22992258	cg04574046	cg19611817
6910	cg22998873	cg04560531	cg07221518
6911	cg22999502	cg04546061	cg14285365
6912	cg22999786	cg04500730	cg23125506
6913	cg23003961	cg04397652	cg23626961
6914	cg23010693	cg04354126	cg27269190
6915	cg23019125	cg04254198	cg06078337
6916	cg23021584	cg04248364	cg19640754
6917	cg23028631	cg04236512	cg16773043
6918	cg23029159	cg04156383	cg18340416
6919	cg23032993	cg04143348	cg21274136
6920	cg23036171	cg04023831	cg11432630
6921	cg23037644	cg03882967	cg24586376
6922	cg23039189	cg03803102	cg07347148
6923	cg23040685	cg03782662	cg20189937
6924	cg23046918	cg03760490	cg02595280
6925	cg23049226	cg03730709	cg09526129
6926	cg23049758	cg03696370	cg10332437
6927	cg23053961	cg03661299	cg09335911
6928	cg23058194	cg03426226	cg09073443
6929	cg23058246	cg03380744	cg12062088
6930	cg23058673	cg03379681	cg21246624
6931	cg23066057	cg03345925	cg06124434
6932	cg23072051	cg03318937	cg25203627
6933	cg23075506	cg03215152	cg13374528
6934	cg23085846	cg03163819	cg09729166
6935	cg23087239	cg03161498	cg02817925
6936	cg23087358	cg03141944	cg14519777
6937	cg23089261	cg03125765	cg15214284
6938	cg23092218	cg03119829	cg14010805
6939	cg23093846	cg03114711	cg22078934
6940	cg23100081	cg03101580	cg13758724

6941	cg23100428	cg03052760	cg10518527
6942	cg23104930	cg03031182	cg22977745
6943	cg23108580	cg03031073	cg16621790
6944	cg23114772	cg02958180	cg06754496
6945	cg23115933	cg02872491	cg00713247
6946	cg23117250	cg02832357	cg08775793
6947	cg23117583	cg02734419	cg00639837
6948	cg23119433	cg02471028	cg07365071
6949	cg23127694	cg02274236	cg10023624
6950	cg23128056	cg02255721	cg01094351
6951	cg23133355	cg02193967	cg15498121
6952	cg23143093	cg02187822	cg19489699
6953	cg23144649	cg02153814	cg23251296
6954	cg23157618	cg02116283	cg02973735
6955	cg23166740	cg02084834	cg00738945
6956	cg23172911	cg01983725	cg17378535
6957	cg23192604	cg01971085	cg27438218
6958	cg23196346	cg01849845	cg24386905
6959	cg23197939	cg01838863	cg10360139
6960	cg23202053	cg01835854	cg15532236
6961	cg23202545	cg01819707	cg00660140
6962	cg23203730	cg01710084	cg10583144
6963	cg23206032	cg01655150	cg09727436
6964	cg23207527	cg01637244	cg21725701
6965	cg23208152	cg01628053	cg05488981
6966	cg23212751	cg01617074	cg11523191
6967	cg23216122	cg01494593	cg02003272
6968	cg23217547	cg01483824	cg22846498
6969	cg23222247	cg01460382	cg24135645
6970	cg23225637	cg01449168	cg23946462
6971	cg23233631	cg01330448	cg13775299
6972	cg23244464	cg01204060	cg13441142
6973	cg23249399	cg01188722	cg06432200
6974	cg23250528	cg01104466	cg21768835
6975	cg23250795	cg01084215	cg18957751
6976	cg23251296	cg01077618	cg18708810
6977	cg23252698	cg01044693	cg08206881
6978	cg23254569	cg00912277	cg02190127
6979	cg23258611	cg00899856	cg15709435
6980	cg23259001	cg00876694	cg05450563
6981	cg23260554	cg00870269	cg09906558
6982	cg23261102	cg00861010	cg02600978
6983	cg23261343	cg00851981	cg05996835
6984	cg23262213	cg00639143	cg23399441
6985	cg23273465	cg00592944	cg06776976
6986	cg23280999	cg00582671	cg09911521
6987	cg23288298	cg00581541	cg05249271
6988	cg23299804	cg00414709	cg02363969
6989	cg23300494	cg00412772	cg26754071
6990	cg23301563	cg00410534	cg22180848
6991	cg23301979	cg00285343	cg18549386
6992	cg23309670	cg00187535	cg02191483
6993	cg23311929	cg00147850	cg24732123

6994	cg23313005	cg00134602	cg08924256
6995	cg23316807	cg00096810	cg06524531
6996	cg23322122	cg00092644	cg00460268
6997	cg23332610	cg27475586	cg17956079
6998	cg23333146	cg27401116	cg01493020
6999	cg23337031	cg27343801	cg04156643
7000	cg23339482	cg27331828	cg02832905
7001	cg23339720	cg27287951	cg05350020
7002	cg23344121	cg27250037	cg02785745
7003	cg23345038	cg27200466	cg03789507
7004	cg23350558	cg27163630	cg24299913
7005	cg23350812	cg27100149	cg10756252
7006	cg23357150	cg27089352	cg06120235
7007	cg23362032	cg27076552	cg23475109
7008	cg23363971	cg26934759	cg05445244
7009	cg23366234	cg26932586	cg08105005
7010	cg23366832	cg26923754	cg04966586
7011	cg23372092	cg26886188	cg16398832
7012	cg23375717	cg26840755	cg06562964
7013	cg23378722	cg26748583	cg07181257
7014	cg23385847	cg26689934	cg14239346
7015	cg23386779	cg26484017	cg06173481
7016	cg23388085	cg26450275	cg06812836
7017	cg23393892	cg26423824	cg16653700
7018	cg23397909	cg26421310	cg16392729
7019	cg23398508	cg26420566	cg06797069
7020	cg23399441	cg26365784	cg01207974
7021	cg23399577	cg26354947	cg07685601
7022	cg23400222	cg26343721	cg16237297
7023	cg23401796	cg26214074	cg19360685
7024	cg23408104	cg26185621	cg14420645
7025	cg23429698	cg26180006	cg19274401
7026	cg23439917	cg26127166	cg19874640
7027	cg23454517	cg26118698	cg22639325
7028	cg23455778	cg26071135	cg11865296
7029	cg23460369	cg25974922	cg00556407
7030	cg23463186	cg25941751	cg13572069
7031	cg23464284	cg25915512	cg22449085
7032	cg23466118	cg25856632	cg25729585
7033	cg23468002	cg25838968	cg17700633
7034	cg23470262	cg25826644	cg00866186
7035	cg23475109	cg25711239	cg08176056
7036	cg23475725	cg25658272	cg13573036
7037	cg23476802	cg25594106	cg06377278
7038	cg23484234	cg25554340	cg13071729
7039	cg23485307	cg25412310	cg17793608
7040	cg23490822	cg25315148	cg20349199
7041	cg23492249	cg25214584	cg00418613
7042	cg23493018	cg25171014	cg04926380
7043	cg23493119	cg25112643	cg01570589
7044	cg23507051	cg25102842	cg03379270
7045	cg23509272	cg25032991	cg19462352
7046	cg23510131	cg25028527	cg13576994

7047	cg23515921	cg24925945	cg15547344
7048	cg23519637	cg24900927	cg03254566
7049	cg23522522	cg24771349	cg02509027
7050	cg23525541	cg24768094	cg10531372
7051	cg23529249	cg24747427	cg25745246
7052	cg23530850	cg24705818	cg06485166
7053	cg23533073	cg24701662	cg08622923
7054	cg23536830	cg24586376	cg05210671
7055	cg23538755	cg24530000	cg07156006
7056	cg23539753	cg24528693	cg17569754
7057	cg23545258	cg24391931	cg02914790
7058	cg23546343	cg24299136	cg02648939
7059	cg23547892	cg24298539	cg04324917
7060	cg23566503	cg24287189	cg20387087
7061	cg23598378	cg24212268	cg12640387
7062	cg23601586	cg24093182	cg23301979
7063	cg23610994	cg24015175	cg25890936
7064	cg23614979	cg23990942	cg09307742
7065	cg23617947	cg23987336	cg05434397
7066	cg23619577	cg23750338	cg03484435
7067	cg23620047	cg23681616	cg20849855
7068	cg23623681	cg23666829	cg16584688
7069	cg23626908	cg23623681	cg25701364
7070	cg23626961	cg23492249	cg20602417
7071	cg23635789	cg23475109	cg02733842
7072	cg23656110	cg23470262	cg13642849
7073	cg23663547	cg23399441	cg25339408
7074	cg23664774	cg23258611	cg01089364
7075	cg23666829	cg23216122	cg04762676
7076	cg23674358	cg23197939	cg16726039
7077	cg23677881	cg23115933	cg01380884
7078	cg23679798	cg23087239	cg12142346
7079	cg23681616	cg23032993	cg12429455
7080	cg23682934	cg22987457	cg06950392
7081	cg23687021	cg22987116	cg26540302
7082	cg23690480	cg22963109	cg21526773
7083	cg23691781	cg22961292	cg05220083
7084	cg23693485	cg22911054	cg25171014
7085	cg23696210	cg22786631	cg05404787
7086	cg23704085	cg22429169	cg18505837
7087	cg23707719	cg22277106	cg17720554
7088	cg23709782	cg22251048	cg25707005
7089	cg23711760	cg22136038	cg20067272
7090	cg23712594	cg22047766	cg25466396
7091	cg23714019	cg22024295	cg13630646
7092	cg23722792	cg22009923	cg02812189
7093	cg23723410	cg21997465	cg06553975
7094	cg23726802	cg21755755	cg04141218
7095	cg23735646	cg21752525	cg18690282
7096	cg23736055	cg21650861	cg24225455
7097	cg23738548	cg21619325	cg23339482
7098	cg23748172	cg21606405	cg16672557
7099	cg23754431	cg21497164	cg20647485

7100	cg23755969	cg21497060	cg02970384
7101	cg23760873	cg21494132	cg10510707
7102	cg23777946	cg21351483	cg00694888
7103	cg23779478	cg21268659	cg03853328
7104	cg23780635	cg21247923	cg14249876
7105	cg23782083	cg21225667	cg12694261
7106	cg23783444	cg21170978	cg06705004
7107	cg23784046	cg21155834	cg01789743
7108	cg23797615	cg21095280	cg19242568
7109	cg23800573	cg21070669	cg23087239
7110	cg23811289	cg21011883	cg23664774
7111	cg23812660	cg20977024	cg27008230
7112	cg23812679	cg20958959	cg09627339
7113	cg23818351	cg20918463	cg26181840
7114	cg23820560	cg20806143	cg08282578
7115	cg23824902	cg20756600	cg12692727
7116	cg23825482	cg20751706	cg13381243
7117	cg23827284	cg20544097	cg18259003
7118	cg23828593	cg20489239	cg23690480
7119	cg23834427	cg20437892	cg01023169
7120	cg23834688	cg20428133	cg08381596
7121	cg23835923	cg20382493	cg07026910
7122	cg23844904	cg20297670	cg01017228
7123	cg23850277	cg20241952	cg19005763
7124	cg23854567	cg20231900	cg25964040
7125	cg23873200	cg20208009	cg21000919
7126	cg23881119	cg20149780	cg03731348
7127	cg23886515	cg20068058	cg05989746
7128	cg23887149	cg20030934	cg17732044
7129	cg23894219	cg19961522	cg15065169
7130	cg23895846	cg19944656	cg25115276
7131	cg23900422	cg19909613	cg12051710
7132	cg23902076	cg19822716	cg09473315
7133	cg23903708	cg19817882	cg13864546
7134	cg23906687	cg19784013	cg02823137
7135	cg23906872	cg19736117	cg02421824
7136	cg23908771	cg19706515	cg08994061
7137	cg23910098	cg19640754	cg16184131
7138	cg23910341	cg19489699	cg27059537
7139	cg23916878	cg19435621	cg20924972
7140	cg23920016	cg19357195	cg04560531
7141	cg23925540	cg19325477	cg09775533
7142	cg23935261	cg19319067	cg02041470
7143	cg23936766	cg19266014	cg06787104
7144	cg23939642	cg19182035	cg01066494
7145	cg23939875	cg18941831	cg10676125
7146	cg23945793	cg18933685	cg20283716
7147	cg23946462	cg18813518	cg17740401
7148	cg23947450	cg18741439	cg21163714
7149	cg23949216	cg18673163	cg13456321
7150	cg23951305	cg18645910	cg02979010
7151	cg23954066	cg18637761	cg20241952
7152	cg23956760	cg18583276	cg11909989

7153	cg23959115	cg18543610	cg24614218
7154	cg23971517	cg18539461	cg05139219
7155	cg23973885	cg18503234	cg12737285
7156	cg23974819	cg18485877	cg00123317
7157	cg23976499	cg18482303	cg13977320
7158	cg23986590	cg18406924	cg00688979
7159	cg23987336	cg18348731	cg06819431
7160	cg23989207	cg18272538	cg10726559
7161	cg23989757	cg18250135	cg09700054
7162	cg23990272	cg18222083	cg03114711
7163	cg23990942	cg18132690	cg10812717
7164	cg23996800	cg18069174	cg15536489
7165	cg23998645	cg18030799	cg17202706
7166	cg23999526	cg18027683	cg13461247
7167	cg24002003	cg17985555	cg23748751
7168	cg24003508	cg17972213	cg10969245
7169	cg24004007	cg17850498	cg00408764
7170	cg24008544	cg17827670	cg27588653
7171	cg24009250	cg17818471	cg22304169
7172	cg24024036	cg17787876	cg18051825
7173	cg24030630	cg17671199	cg16525330
7174	cg24031446	cg17574471	cg06233301
7175	cg24033503	cg17525357	cg01497892
7176	cg24035682	cg17444479	cg26450275
7177	cg24037270	cg17179557	cg11843948
7178	cg24044501	cg16962557	cg02418175
7179	cg24045498	cg16876192	cg13916961
7180	cg24047905	cg16824603	cg17703629
7181	cg24052817	cg16747567	cg12231088
7182	cg24064506	cg16694397	cg15636859
7183	cg24065957	cg16616765	cg19201770
7184	cg24073352	cg16509173	cg04797170
7185	cg24075760	cg16496462	cg02592525
7186	cg24083817	cg16496024	cg25531618
7187	cg24090911	cg16381321	cg10276623
7188	cg24092282	cg16340152	cg11234767
7189	cg24092444	cg16316042	cg07338476
7190	cg24093176	cg16274890	cg23800573
7191	cg24093182	cg16258545	cg10277646
7192	cg24096323	cg16255804	cg06924233
7193	cg24097241	cg16237297	cg11423998
7194	cg24097814	cg16223967	cg09567735
7195	cg24100115	cg16222367	cg15446884
7196	cg24102222	cg16207263	cg26462488
7197	cg24104241	cg16195091	cg14025149
7198	cg24107411	cg16183741	cg08261841
7199	cg24114708	cg16074271	cg13099829
7200	cg24118850	cg16071082	cg23040685
7201	cg24123198	cg16023896	cg26284638
7202	cg24126880	cg15991082	cg03793055
7203	cg24129923	cg15975598	cg06947205
7204	cg24135645	cg15957394	cg24918798
7205	cg24137511	cg15904939	cg09941367

7206	cg24137774	cg15894581	cg25443991
7207	cg24148817	cg15802323	cg21457110
7208	cg24152000	cg15777335	cg20462191
7209	cg24154271	cg15709435	cg00400654
7210	cg24155123	cg15629568	cg04220455
7211	cg24158160	cg15617336	cg25453957
7212	cg24163616	cg15598425	cg11215918
7213	cg24163668	cg15504666	cg08692104
7214	cg24175188	cg15367698	cg27068650
7215	cg24181389	cg15302379	cg22622164
7216	cg24183575	cg15128510	cg26281621
7217	cg24186506	cg15125438	cg02495413
7218	cg24191821	cg15104211	cg14244878
7219	cg24194413	cg15072038	cg02010047
7220	cg24194998	cg15036118	cg07964527
7221	cg24197051	cg15014006	cg00428526
7222	cg24202221	cg14939765	cg17947599
7223	cg24209874	cg14923274	cg16499956
7224	cg24212268	cg14918743	cg00270141
7225	cg24212377	cg14917100	cg05547140
7226	cg24215159	cg14864276	cg16535788
7227	cg24215635	cg14841828	cg05661968
7228	cg24220178	cg14811011	cg20828052
7229	cg24225455	cg14772050	cg01046905
7230	cg24233679	cg14684166	cg07104557
7231	cg24244437	cg14655044	cg07042489
7232	cg24247370	cg14575741	cg08293536
7233	cg24247537	cg14321522	cg24930851
7234	cg24253714	cg14227418	cg07962882
7235	cg24257168	cg14219236	cg14734994
7236	cg24259291	cg14213430	cg24957532
7237	cg24260710	cg14208133	cg26951000
7238	cg24264451	cg14206983	cg07160118
7239	cg24268698	cg14190128	cg04982190
7240	cg24270629	cg14127396	cg04460041
7241	cg24271718	cg14062119	cg17766055
7242	cg24275626	cg14010805	cg17803589
7243	cg24276624	cg13957538	cg06753050
7244	cg24283049	cg13940218	cg24450180
7245	cg24287189	cg13871900	cg19486437
7246	cg24288527	cg13858803	cg22761077
7247	cg24289452	cg13850871	cg00885461
7248	cg24293145	cg13817905	cg02216206
7249	cg24294013	cg13724379	cg02910959
7250	cg24295561	cg13685294	cg15141238
7251	cg24297835	cg13683219	cg13133492
7252	cg24298539	cg13675389	cg04125460
7253	cg24299136	cg13657981	cg19515081
7254	cg24299913	cg13657004	cg26516446
7255	cg24301288	cg13639244	cg23208152
7256	cg24304880	cg13636986	cg00904122
7257	cg24307703	cg13618880	cg05051734
7258	cg24314474	cg13589431	cg07192047

7259	cg24321688	cg13573036	cg10830021
7260	cg24336730	cg13555689	cg04354126
7261	cg24342377	cg13554136	cg13919369
7262	cg24349804	cg13526766	cg13924326
7263	cg24358599	cg13447099	cg16721977
7264	cg24360745	cg13446070	cg06252645
7265	cg24362347	cg13402847	cg19525780
7266	cg24366425	cg13392861	cg08457169
7267	cg24372256	cg13352836	cg09080909
7268	cg24387101	cg13283765	cg06938890
7269	cg24391931	cg13276704	cg16530165
7270	cg24394336	cg13232249	cg14826031
7271	cg24403487	cg13194638	cg15260437
7272	cg24408200	cg13170807	cg17961835
7273	cg24408316	cg13139630	cg01079658
7274	cg24420089	cg12927715	cg13383819
7275	cg24420164	cg12901650	cg09666417
7276	cg24423806	cg12778183	cg21255128
7277	cg24424381	cg12742209	cg26710819
7278	cg24425316	cg12728588	cg07012725
7279	cg24429037	cg12650685	cg11792186
7280	cg24429081	cg12629008	cg03253309
7281	cg24433265	cg12481567	cg25118879
7282	cg24437408	cg12469964	cg06577703
7283	cg24437859	cg12459932	cg09828346
7284	cg24448421	cg12375903	cg05324992
7285	cg24450180	cg12356870	cg23244877
7286	cg24453118	cg12339425	cg18514164
7287	cg24453600	cg12161625	cg02399294
7288	cg24455383	cg12106976	cg13287523
7289	cg24463664	cg12069132	cg14588828
7290	cg24469719	cg11966063	cg17442932
7291	cg24474622	cg11958668	cg26452091
7292	cg24479752	cg11898009	cg27226927
7293	cg24482246	cg11888747	cg06572904
7294	cg24495350	cg11879277	cg01876978
7295	cg24499677	cg11843948	cg26423542
7296	cg24502047	cg11834956	cg04352763
7297	cg24517989	cg11828470	cg19892287
7298	cg24518264	cg11819469	cg01062470
7299	cg24518609	cg11809476	cg21445553
7300	cg24523322	cg11804414	cg02829456
7301	cg24525461	cg11777290	cg11544882
7302	cg24528693	cg11719457	cg18752987
7303	cg24530000	cg11592377	cg24289452
7304	cg24544986	cg11576988	cg26321613
7305	cg24549289	cg11523661	cg04214430
7306	cg24550880	cg11381282	cg05856230
7307	cg24553775	cg11363168	cg23304923
7308	cg24562682	cg11143193	cg09159050
7309	cg24586376	cg11088489	cg06131879
7310	cg24586870	cg11046864	cg05060427
7311	cg24589334	cg11028291	cg11952908

7312	cg24590353	cg11007190	cg13508949
7313	cg24595704	cg10989175	cg18428888
7314	cg24606594	cg10960375	cg27380631
7315	cg24614218	cg10959933	cg18485872
7316	cg24617568	cg10862981	cg16125397
7317	cg24619276	cg10860364	cg16965936
7318	cg24625084	cg10836809	cg13177873
7319	cg24628866	cg10829693	cg01503773
7320	cg24629431	cg10827094	cg19057779
7321	cg24630383	cg10798745	cg00618323
7322	cg24635581	cg10786098	cg08507725
7323	cg24639863	cg10754670	cg19925801
7324	cg24647108	cg10738865	cg25230305
7325	cg24648594	cg10589745	cg19865916
7326	cg24657347	cg10585257	cg04334792
7327	cg24659758	cg10531372	cg13360638
7328	cg24670927	cg10512089	cg17560283
7329	cg24673886	cg10418812	cg26410484
7330	cg24674087	cg10388307	cg01449168
7331	cg24678812	cg10277646	cg26018322
7332	cg24691835	cg10248148	cg18098187
7333	cg24701232	cg10082525	cg03318937
7334	cg24701662	cg10034572	cg23620047
7335	cg24705818	cg09988676	cg00647879
7336	cg24706188	cg09935792	cg08334780
7337	cg24706522	cg09925620	cg08276889
7338	cg24706981	cg09790523	cg16161135
7339	cg24709718	cg09776314	cg17980999
7340	cg24711397	cg09746044	cg02883198
7341	cg24723883	cg09697259	cg25637473
7342	cg24724937	cg09672200	cg15086474
7343	cg24727133	cg09580859	cg08899531
7344	cg24728456	cg09499482	cg17112404
7345	cg24730224	cg09342325	cg16078836
7346	cg24732123	cg09335314	cg06996029
7347	cg24734735	cg09327610	cg03182608
7348	cg24738036	cg09320595	cg18798744
7349	cg24738346	cg09231120	cg11898009
7350	cg24738592	cg09214456	cg05555455
7351	cg24747427	cg09140274	cg09851241
7352	cg24748448	cg09076960	cg14270348
7353	cg24748868	cg08966188	cg25214584
7354	cg24750887	cg08934318	cg03761162
7355	cg24752354	cg08864042	cg15117891
7356	cg24760753	cg08836729	cg25007781
7357	cg24766229	cg08812784	cg18677278
7358	cg24767368	cg08782674	cg23463186
7359	cg24768094	cg08768904	cg15834460
7360	cg24771349	cg08767627	cg18732855
7361	cg24775327	cg08692104	cg07285148
7362	cg24783499	cg08616663	cg11060194
7363	cg24787929	cg08507725	cg13815311
7364	cg24804060	cg08436467	cg27631766

7365	cg24809845	cg08435157	cg20733739
7366	cg24824472	cg08397968	cg05738470
7367	cg24825262	cg08360599	cg21697252
7368	cg24825937	cg08091706	cg16962557
7369	cg24830876	cg08067991	cg13554136
7370	cg24831140	cg08062713	cg04767697
7371	cg24844449	cg08007899	cg04629141
7372	cg24845165	cg07912144	cg08070235
7373	cg24847636	cg07850832	cg27663249
7374	cg24852135	cg07770222	cg00452755
7375	cg24856699	cg07731639	cg20910352
7376	cg24857399	cg07716287	cg20916068
7377	cg24859433	cg07701911	cg10844382
7378	cg24870662	cg07661899	cg13774342
7379	cg24873093	cg07624888	cg15355859
7380	cg24875415	cg07613391	cg09939191
7381	cg24875593	cg07549381	cg23895846
7382	cg24881255	cg07505515	cg11564792
7383	cg24881558	cg07468956	cg25900150
7384	cg24885442	cg07467854	cg24299136
7385	cg24890045	cg07391023	cg06333135
7386	cg24893073	cg07290269	cg03082830
7387	cg24896649	cg07281938	cg25731074
7388	cg24897108	cg07279281	cg10174191
7389	cg24900927	cg07230078	cg05368971
7390	cg24901995	cg07156006	cg02351425
7391	cg24902250	cg07152894	cg01863398
7392	cg24903767	cg07139329	cg05099221
7393	cg24915915	cg07129769	cg03216729
7394	cg24916358	cg07125976	cg19569551
7395	cg24918798	cg07050626	cg09890746
7396	cg24925163	cg07042489	cg04677227
7397	cg24925945	cg06996896	cg09957712
7398	cg24926689	cg06895946	cg21040069
7399	cg24928546	cg06856570	cg07016298
7400	cg24930851	cg06823034	cg07529658
7401	cg24940138	cg06710741	cg26672794
7402	cg24940701	cg06586578	cg02060039
7403	cg24941195	cg06549228	cg01793617
7404	cg24957532	cg06524531	cg08695912
7405	cg24964215	cg06509362	cg27070508
7406	cg24969902	cg06483517	cg26748583
7407	cg24973993	cg06406157	cg13216372
7408	cg24982682	cg06342954	cg07385490
7409	cg24987440	cg06294954	cg05678033
7410	cg24989405	cg06267617	cg04493177
7411	cg24993140	cg06212582	cg09786420
7412	cg24996315	cg06154633	cg07131274
7413	cg24997330	cg05909553	cg24275626
7414	cg24998879	cg05899507	cg24164238
7415	cg25005357	cg05874233	cg20030934
7416	cg25006254	cg05862007	cg01044693
7417	cg25007422	cg05858079	cg08677140

7418	cg25007781	cg05680710	cg24738036
7419	cg25010788	cg05678033	cg02156952
7420	cg25013910	cg05588341	cg09505809
7421	cg25032991	cg05552543	cg02034328
7422	cg25033220	cg05535123	cg13642149
7423	cg25038926	cg05484458	cg13210470
7424	cg25045972	cg05452742	cg06309980
7425	cg25048985	cg05445244	cg22009923
7426	cg25053164	cg05415936	cg07296854
7427	cg25059445	cg05399244	cg11241684
7428	cg25061701	cg05272807	cg05658807
7429	cg25066224	cg05150667	cg03954280
7430	cg25066857	cg05035470	cg16421359
7431	cg25077823	cg04997235	cg10421435
7432	cg25084760	cg04835685	cg18649632
7433	cg25086418	cg04724275	cg25471740
7434	cg25095994	cg04696969	cg15969277
7435	cg25097308	cg04665930	cg22866068
7436	cg25102842	cg04654299	cg06506523
7437	cg25104512	cg04627183	cg11834956
7438	cg25114693	cg04600641	cg17385318
7439	cg25115276	cg04575609	cg05833851
7440	cg25116615	cg04536844	cg03098159
7441	cg25117523	cg04476286	cg11449408
7442	cg25118879	cg04440361	cg24964215
7443	cg25122824	cg04366815	cg25765819
7444	cg25135706	cg04348080	cg01553866
7445	cg25136955	cg04324917	cg10836406
7446	cg25137314	cg04323313	cg16126178
7447	cg25138553	cg04285855	cg09410453
7448	cg25142010	cg04226952	cg22706186
7449	cg25146042	cg04157865	cg14143441
7450	cg25148456	cg04078658	cg11148096
7451	cg25153629	cg03985415	cg05179396
7452	cg25161092	cg03984919	cg17671199
7453	cg25162533	cg03950492	cg07043952
7454	cg25167447	cg03933756	cg19513744
7455	cg25171014	cg03899978	cg19918057
7456	cg25187161	cg03815917	cg22937762
7457	cg25189085	cg03798162	cg11816841
7458	cg25189808	cg03765423	cg14320530
7459	cg25190718	cg03763874	cg10009737
7460	cg25197500	cg03644585	cg19975759
7461	cg25199850	cg03608520	cg00169184
7462	cg25202370	cg03576555	cg09634481
7463	cg25203085	cg03549146	cg18228456
7464	cg25203627	cg03417340	cg14995235
7465	cg25214584	cg03384915	cg04803994
7466	cg25221919	cg03370270	cg15772924
7467	cg25223552	cg03339668	cg19767562
7468	cg25227364	cg03296204	cg24194998
7469	cg25235326	cg03286774	cg18519450
7470	cg25242498	cg03284113	cg05283542

7471	cg25246084	cg03267954	cg05291588
7472	cg25247887	cg03231163	cg15322667
7473	cg25249728	cg03187301	cg18442286
7474	cg25261191	cg03163783	cg02660541
7475	cg25264265	cg03077533	cg11980500
7476	cg25268422	cg03063946	cg01032946
7477	cg25270886	cg02999082	cg18703721
7478	cg25271479	cg02975060	cg13593436
7479	cg25281849	cg02961707	cg01188722
7480	cg25283432	cg02937548	cg25739142
7481	cg25285041	cg02930033	cg02725620
7482	cg25286715	cg02914790	cg13657004
7483	cg25287071	cg02901002	cg26159074
7484	cg25289803	cg02827340	cg25692669
7485	cg25296804	cg02816525	cg10107466
7486	cg25301103	cg02804722	cg08552290
7487	cg25306579	cg02734600	cg27146477
7488	cg25310555	cg02720600	cg05971891
7489	cg25311162	cg02714192	cg21404878
7490	cg25312122	cg02633409	cg14087401
7491	cg25315148	cg02583282	cg19239692
7492	cg25316172	cg02582619	cg14638315
7493	cg25317315	cg02550308	cg17234201
7494	cg25321935	cg02538557	cg20588285
7495	cg25323005	cg02495413	cg00876694
7496	cg25341653	cg02462933	cg19539481
7497	cg25346117	cg02449166	cg09646934
7498	cg25350827	cg02438575	cg26808779
7499	cg25350986	cg02402630	cg00747342
7500	cg25352714	cg02399294	cg24589334
7501	cg25352924	cg02364610	cg13028113
7502	cg25355635	cg02362505	cg25223552
7503	cg25363336	cg02264288	cg18820914
7504	cg25369553	cg02246916	cg14461852
7505	cg25372085	cg02240936	cg03975834
7506	cg25372722	cg02238387	cg05364072
7507	cg25382652	cg02221422	cg12787036
7508	cg25383568	cg02212339	cg04600641
7509	cg25386426	cg02175033	cg02645302
7510	cg25389863	cg02159643	cg15465367
7511	cg25390243	cg02112621	cg01153620
7512	cg25399818	cg02069944	cg19288921
7513	cg25403283	cg02047646	cg23515921
7514	cg25405238	cg02013841	cg05634149
7515	cg25406657	cg01930924	cg10724774
7516	cg25408950	cg01871631	cg06914693
7517	cg25409944	cg01866330	cg27410449
7518	cg25412310	cg01824603	cg06085713
7519	cg25413575	cg01820765	cg21475781
7520	cg25418363	cg01792640	cg18851831
7521	cg25424742	cg01749725	cg15553612
7522	cg25430713	cg01723163	cg00955867
7523	cg25431366	cg01697623	cg03528037

7524	cg25433222	cg01693662	cg27424692
7525	cg25439867	cg01589353	cg05339066
7526	cg25441338	cg01577298	cg13079633
7527	cg25442652	cg01514831	cg02972475
7528	cg25443991	cg01483459	cg02570361
7529	cg25451660	cg01482620	cg15478081
7530	cg25453957	cg01452115	cg25995420
7531	cg25461775	cg01449425	cg13662628
7532	cg25461827	cg01428928	cg11237495
7533	cg25463863	cg01402099	cg06549228
7534	cg25466396	cg01337429	cg26787096
7535	cg25471740	cg01309081	cg05821186
7536	cg25476129	cg01254644	cg12352896
7537	cg25477334	cg01194444	cg24701232
7538	cg25479682	cg01138652	cg19786422
7539	cg25479732	cg01023808	cg20226512
7540	cg25481201	cg01017397	cg07207742
7541	cg25484139	cg00959308	cg27421679
7542	cg25488990	cg00955867	cg14567593
7543	cg25490527	cg00954841	cg27604101
7544	cg25492112	cg00936895	cg07456413
7545	cg25502179	cg00919689	cg05915622
7546	cg25504217	cg00902427	cg13836318
7547	cg25506288	cg00857907	cg20881888
7548	cg25510609	cg00852414	cg19789753
7549	cg25515317	cg00845883	cg25405238
7550	cg25517015	cg00777079	cg03326699
7551	cg25517187	cg00715343	cg22529541
7552	cg25519921	cg00670539	cg13827597
7553	cg25520910	cg00657810	cg13663738
7554	cg25531618	cg00648573	cg04590974
7555	cg25532627	cg00589581	cg19756253
7556	cg25535708	cg00550040	cg22799860
7557	cg25545917	cg00423871	cg10545738
7558	cg25552705	cg00408764	cg08441269
7559	cg25552768	cg00374546	cg26673975
7560	cg25552843	cg00366037	cg22202031
7561	cg25554340	cg00286014	cg05590948
7562	cg25555068	cg00244391	cg04835685
7563	cg25556841	cg00244267	cg04198125
7564	cg25557739	cg00143606	cg13232249
7565	cg25559625	cg00137696	cg10634182
7566	cg25567021	cg27548256	cg16910448
7567	cg25567674	cg27538859	cg10060631
7568	cg25567938	cg27458631	cg20461270
7569	cg25568490	cg27421679	cg02582619
7570	cg25568745	cg27390206	cg25644380
7571	cg25575145	cg27380631	cg04899175
7572	cg25582924	cg27378358	cg16747567
7573	cg25587431	cg27271532	cg23824902
7574	cg25588387	cg27221053	cg00236831
7575	cg25588389	cg27213925	cg01989275
7576	cg25588826	cg27146036	cg14835545

7577	cg25589001	cg27021553	cg13248406
7578	cg25591106	cg27014538	cg11936817
7579	cg25591418	cg26860182	cg26607933
7580	cg25594106	cg26800893	cg08073527
7581	cg25594550	cg26750188	cg07598035
7582	cg25595641	cg26737223	cg12279175
7583	cg25596405	cg26734040	cg09897002
7584	cg25600823	cg26723735	cg12371263
7585	cg25604994	cg26639076	cg23704085
7586	cg25606046	cg26608486	cg18467406
7587	cg25607321	cg26444806	cg14208133
7588	cg25613180	cg26377276	cg04902542
7589	cg25616055	cg26297299	cg24425316
7590	cg25617157	cg26268565	cg08248600
7591	cg25619703	cg26240231	cg27364916
7592	cg25623727	cg26162025	cg00013441
7593	cg25627714	cg26137103	cg08143701
7594	cg25629077	cg26074140	cg26932586
7595	cg25634000	cg26053547	cg22942245
7596	cg25635000	cg26016985	cg02426072
7597	cg25637473	cg26015115	cg00936895
7598	cg25638714	cg26005197	cg06332339
7599	cg25642974	cg25862117	cg01494593
7600	cg25644380	cg25783610	cg03158194
7601	cg25649889	cg25635000	cg25591106
7602	cg25652751	cg25627714	cg25517187
7603	cg25654695	cg25567674	cg04958227
7604	cg25658272	cg25519921	cg07097374
7605	cg25661792	cg25466396	cg04633661
7606	cg25666403	cg25372722	cg24107411
7607	cg25668093	cg25363336	cg13922681
7608	cg25688583	cg25312122	cg08964948
7609	cg25692669	cg25286715	cg15389749
7610	cg25701364	cg25232510	cg18253787
7611	cg25707005	cg25230305	cg13488220
7612	cg25711239	cg25223552	cg26342552
7613	cg25713411	cg25045972	cg14297546
7614	cg25714956	cg25007781	cg05322217
7615	cg25719378	cg24964215	cg12514933
7616	cg25722029	cg24941195	cg21450652
7617	cg25723217	cg24901995	cg00007036
7618	cg25729585	cg24890045	cg07041883
7619	cg25730564	cg24809845	cg02635482
7620	cg25731074	cg24766229	cg03834055
7621	cg25739142	cg24760753	cg01314597
7622	cg25739943	cg24748448	cg08603083
7623	cg25745246	cg24595704	cg07261940
7624	cg25754933	cg24562682	cg20985758
7625	cg25761326	cg24518264	cg16496024
7626	cg25763426	cg24437859	cg05823589
7627	cg25765315	cg24408200	cg00935361
7628	cg25765819	cg24404329	cg14199827
7629	cg25780982	cg24335984	cg08544606

7630	cg25782041	cg24276624	cg07505515
7631	cg25783610	cg24253714	cg07368061
7632	cg25785495	cg24220178	cg02492708
7633	cg25793812	cg24215635	cg16397629
7634	cg25794830	cg24183575	cg02604890
7635	cg25801976	cg24126880	cg12072973
7636	cg25825612	cg24092444	cg26585320
7637	cg25826644	cg24033503	cg27222884
7638	cg25827936	cg23957850	cg27630540
7639	cg25828334	cg23835923	cg20677814
7640	cg25831522	cg23828593	cg22348713
7641	cg25835936	cg23736055	cg00547768
7642	cg25838968	cg23714019	cg26705574
7643	cg25841625	cg23704085	cg08208595
7644	cg25846061	cg23693485	cg07834408
7645	cg25852019	cg23663547	cg26343721
7646	cg25852715	cg23635789	cg08553857
7647	cg25854527	cg23598378	cg21789440
7648	cg25856632	cg23545258	cg08966413
7649	cg25862117	cg23515921	cg20812052
7650	cg25864762	cg23486701	cg05601844
7651	cg25875209	cg23401796	cg22204453
7652	cg25878830	cg23332610	cg08829140
7653	cg25881985	cg23203730	cg26690672
7654	cg25887811	cg23157618	cg19702383
7655	cg25888386	cg23108126	cg01924223
7656	cg25890936	cg23100081	cg05088794
7657	cg25892761	cg23066057	cg24136288
7658	cg25903122	cg23029159	cg01077618
7659	cg25909064	cg22999786	cg09164574
7660	cg25909885	cg22918700	cg06528340
7661	cg25915512	cg22909085	cg23127694
7662	cg25920512	cg22890571	cg08313842
7663	cg25932290	cg22877380	cg09163478
7664	cg25934198	cg22822599	cg07094993
7665	cg25938735	cg22822048	cg17107501
7666	cg25945499	cg22686523	cg25315148
7667	cg25945504	cg22488259	cg23117250
7668	cg25946646	cg22450662	cg27221053
7669	cg25947619	cg22449085	cg05845879
7670	cg25950520	cg22387323	cg06650364
7671	cg25952596	cg22367886	cg17977133
7672	cg25953130	cg22243439	cg08436467
7673	cg25954194	cg22217449	cg25826644
7674	cg25963540	cg22152438	cg22737001
7675	cg25964040	cg22069262	cg02332902
7676	cg25971741	cg21874862	cg26677892
7677	cg25974922	cg21768835	cg03726437
7678	cg25975823	cg21570597	cg22402769
7679	cg25977879	cg21476203	cg04699214
7680	cg25978487	cg21389456	cg20426710
7681	cg25984524	cg21361322	cg09529783
7682	cg25995420	cg21334198	cg07086112

7683	cg25998584	cg20997320	cg16711084
7684	cg26001655	cg20829550	cg00669182
7685	cg26002713	cg20779414	cg23421128
7686	cg26005197	cg20723705	cg25261191
7687	cg26007445	cg20669366	cg19011826
7688	cg26011438	cg20654462	cg01422416
7689	cg26013235	cg20555305	cg02217035
7690	cg26014373	cg20495333	cg26856771
7691	cg26015115	cg20471017	cg15016740
7692	cg26016985	cg20447038	cg24137774
7693	cg26018322	cg20407868	cg25481201
7694	cg26019124	cg20379239	cg21352006
7695	cg26026296	cg20322837	cg24724937
7696	cg26030804	cg20309371	cg25801976
7697	cg26034341	cg20309339	cg26428962
7698	cg26034516	cg20300032	cg06371291
7699	cg26041493	cg20297053	cg14772050
7700	cg26053547	cg20267732	cg24372256
7701	cg26053832	cg20188546	cg07917127
7702	cg26060535	cg20169015	cg08157446
7703	cg26064794	cg20137237	cg23244464
7704	cg26066560	cg19857461	cg24825937
7705	cg26068595	cg19765378	cg11519725
7706	cg26070874	cg19724698	cg11270070
7707	cg26071135	cg19631779	cg02124957
7708	cg26071556	cg19572849	cg04624413
7709	cg26074140	cg19509663	cg27466615
7710	cg26075905	cg19500393	cg15232718
7711	cg26079864	cg19397321	cg18108716
7712	cg26092233	cg19317226	cg05960932
7713	cg26097051	cg19296662	cg20679659
7714	cg26104640	cg19242568	cg13765417
7715	cg26105658	cg19200440	cg05968917
7716	cg26118698	cg19161559	cg00374546
7717	cg26120842	cg19130981	cg03629926
7718	cg26127113	cg19092603	cg08469834
7719	cg26127166	cg19081470	cg03358345
7720	cg26129303	cg19057916	cg07109788
7721	cg26132163	cg18998442	cg25739943
7722	cg26133909	cg18939081	cg06586578
7723	cg26137103	cg18935449	cg23947450
7724	cg26143540	cg18907942	cg05714748
7725	cg26144207	cg18860847	cg25033220
7726	cg26147269	cg18847089	cg04724275
7727	cg26152017	cg18815879	cg21228259
7728	cg26152051	cg18761976	cg09915883
7729	cg26152188	cg18689614	cg04410777
7730	cg26159074	cg18649632	cg24181389
7731	cg26160492	cg18636829	cg26446949
7732	cg26162025	cg18604419	cg15911948
7733	cg26166004	cg18459869	cg14778721
7734	cg26169991	cg18428888	cg17743939
7735	cg26172211	cg18382744	cg03736944

7736	cg26177041	cg18264298	cg05760053
7737	cg26177754	cg18253787	cg14763173
7738	cg26178184	cg18248273	cg22047766
7739	cg26180843	cg18091245	cg15014006
7740	cg26181840	cg18055067	cg01852611
7741	cg26184501	cg17993073	cg11084269
7742	cg26185621	cg17958516	cg21747549
7743	cg26187123	cg17857870	cg07187503
7744	cg26187194	cg17846497	cg05801879
7745	cg26194092	cg17843418	cg02734419
7746	cg26204042	cg17820025	cg05503991
7747	cg26207909	cg17797898	cg05724271
7748	cg26214074	cg17678224	cg06154633
7749	cg26214948	cg17548735	cg11592377
7750	cg26215113	cg17511604	cg05477920
7751	cg26218835	cg17470198	cg17830810
7752	cg26222498	cg17214107	cg13601605
7753	cg26224624	cg17203290	cg14732540
7754	cg26224791	cg17196713	cg19761272
7755	cg26231761	cg17112404	cg06513888
7756	cg26234644	cg17107501	cg24767368
7757	cg26240231	cg17032372	cg25754933
7758	cg26245697	cg16996571	cg27203917
7759	cg26261270	cg16908801	cg18132690
7760	cg26261873	cg16890796	cg25998584
7761	cg26262482	cg16885096	cg24747239
7762	cg26268125	cg16784943	cg16726435
7763	cg26268565	cg16762085	cg16436210
7764	cg26284638	cg16704938	cg04846781
7765	cg26289871	cg16565294	cg14992771
7766	cg26297299	cg16554534	cg20190649
7767	cg26304689	cg16499415	cg24268698
7768	cg26309261	cg16479633	cg27128001
7769	cg26313421	cg16463044	cg02704331
7770	cg26313976	cg16365663	cg11672338
7771	cg26315221	cg16329509	cg11843691
7772	cg26315985	cg16174644	cg26428825
7773	cg26321613	cg15943602	cg11588197
7774	cg26323752	cg15889057	cg11762760
7775	cg26325286	cg15798050	cg04415736
7776	cg26326208	cg15712304	cg02225004
7777	cg26328589	cg15561453	cg23929809
7778	cg26332285	cg15546071	cg03680680
7779	cg26341003	cg15536947	cg00442220
7780	cg26342552	cg15536489	cg18617669
7781	cg26343721	cg15507500	cg20673255
7782	cg26350921	cg15391531	cg22584580
7783	cg26354947	cg15342087	cg22256482
7784	cg26362368	cg15269394	cg18045658
7785	cg26365254	cg15171237	cg06212263
7786	cg26365784	cg15155463	cg12186219
7787	cg26365854	cg14966074	cg10982964
7788	cg26366087	cg14921326	cg07912144

7789	cg26367275	cg14811014	cg19443475
7790	cg26377276	cg14698794	cg12681784
7791	cg26379230	cg14570652	cg16876192
7792	cg26381919	cg14473030	cg04137490
7793	cg26383057	cg14462369	cg22904096
7794	cg26383138	cg14405528	cg13877452
7795	cg26392980	cg14297546	cg02159643
7796	cg26394825	cg14262433	cg27290103
7797	cg26398024	cg14201946	cg17645528
7798	cg26408861	cg14200357	cg18834729
7799	cg26410484	cg14175330	cg11152884
7800	cg26411080	cg14168733	cg27662093
7801	cg26413192	cg14093018	cg17221941
7802	cg26419962	cg13972711	cg13795838
7803	cg26420566	cg13904806	cg10580341
7804	cg26421308	cg13877452	cg14506192
7805	cg26421310	cg13799838	cg03649353
7806	cg26423542	cg13793145	cg08983215
7807	cg26423824	cg13782615	cg05349039
7808	cg26426334	cg13753460	cg20925383
7809	cg26426564	cg13731636	cg01903305
7810	cg26428825	cg13721589	cg26503644
7811	cg26428962	cg13707189	cg14337844
7812	cg26434328	cg13668823	cg13991631
7813	cg26441230	cg13662225	cg10862981
7814	cg26441486	cg13601605	cg03515246
7815	cg26446949	cg13587802	cg12595461
7816	cg26448137	cg13537940	cg20958959
7817	cg26448609	cg13464157	cg13629563
7818	cg26449178	cg13456321	cg01820765
7819	cg26450275	cg13444374	cg21535670
7820	cg26452091	cg13439189	cg02926160
7821	cg26460678	cg13433644	cg03722184
7822	cg26462488	cg13099139	cg13526766
7823	cg26466921	cg13093285	cg04883903
7824	cg26467269	cg13055385	cg03285823
7825	cg26475911	cg13054523	cg03760490
7826	cg26484017	cg12995933	cg01454947
7827	cg26495716	cg12729518	cg04348080
7828	cg26499286	cg12612065	cg26074140
7829	cg26502610	cg12569216	cg03212620
7830	cg26503644	cg12442385	cg14178294
7831	cg26503877	cg12379145	cg21011883
7832	cg26504421	cg12070646	cg12467960
7833	cg26510473	cg12064373	cg23507051
7834	cg26514080	cg12054698	cg09057885
7835	cg26516446	cg12053105	cg17846497
7836	cg26526953	cg12019806	cg01527159
7837	cg26529655	cg11990296	cg16172837
7838	cg26540302	cg11952908	cg24870688
7839	cg26543243	cg11951169	cg12433926
7840	cg26547741	cg11945095	cg10716356
7841	cg26550337	cg11847597	cg09262882

7842	cg26551200	cg11840849	cg15363973
7843	cg26552621	cg11815072	cg16640008
7844	cg26564172	cg11772801	cg27594157
7845	cg26566236	cg11753157	cg07810967
7846	cg26572651	cg11697433	cg00981003
7847	cg26573923	cg11585280	cg14570652
7848	cg26574247	cg11527930	cg13274107
7849	cg26579379	cg11523799	cg20435464
7850	cg26585320	cg11449408	cg19536407
7851	cg26585452	cg11225330	cg03181618
7852	cg26597727	cg11207081	cg24358599
7853	cg26601559	cg11155016	cg17539235
7854	cg26605683	cg11036962	cg12053105
7855	cg26607528	cg11027058	cg06246000
7856	cg26608486	cg10902107	cg03431903
7857	cg26609642	cg10872457	cg19970953
7858	cg26613811	cg10818702	cg14658346
7859	cg26614264	cg10601476	cg06641607
7860	cg26620147	cg10552275	cg21279756
7861	cg26637901	cg10550166	cg14093663
7862	cg26645242	cg10495754	cg04155280
7863	cg26648465	cg10467217	cg02228688
7864	cg26650973	cg10464467	cg23971517
7865	cg26653360	cg10193870	cg00948881
7866	cg26660115	cg10174191	cg17307791
7867	cg26661623	cg10119288	cg22706883
7868	cg26666292	cg10115918	cg12590668
7869	cg26668713	cg10107466	cg25066224
7870	cg26668872	cg10107330	cg11207081
7871	cg26672794	cg10101600	cg16117028
7872	cg26673975	cg10012530	cg01753176
7873	cg26677892	cg09884107	cg26963844
7874	cg26687830	cg09859805	cg23996800
7875	cg26689934	cg09789315	cg00994984
7876	cg26690672	cg09612502	cg01885814
7877	cg26692003	cg09597070	cg20863668
7878	cg26695387	cg09556902	cg12599569
7879	cg26697605	cg09520904	cg24304880
7880	cg26705574	cg09505809	cg19567295
7881	cg26709309	cg09450022	cg24960639
7882	cg26710819	cg09407273	cg06696911
7883	cg26711406	cg09406921	cg19181160
7884	cg26720682	cg09253696	cg08137602
7885	cg26721908	cg09246253	cg08572315
7886	cg26723735	cg09226986	cg19435621
7887	cg26726230	cg09218398	cg21010028
7888	cg26734040	cg09198360	cg05703996
7889	cg26737223	cg09123961	cg19182035
7890	cg26740525	cg09080909	cg14657458
7891	cg26744332	cg09006487	cg08435157
7892	cg26748583	cg08955461	cg02247300
7893	cg26750188	cg08924256	cg10095352
7894	cg26757820	cg08894401	cg14597504

7895	cg26765599	cg08806632	cg20061812
7896	cg26767085	cg08775774	cg27044641
7897	cg26767198	cg08733553	cg17367901
7898	cg26769720	cg08590257	cg26767085
7899	cg26778630	cg08521178	cg13299824
7900	cg26784106	cg08264519	cg03639964
7901	cg26784300	cg08257212	cg07828612
7902	cg26787096	cg08206156	cg24096323
7903	cg26794830	cg08115732	cg11018723
7904	cg26796443	cg08061755	cg14507427
7905	cg26797297	cg08059361	cg17528003
7906	cg26798772	cg08004045	cg00470794
7907	cg26800893	cg08002981	cg17437086
7908	cg26803670	cg07818978	cg13744954
7909	cg26808779	cg07795082	cg27207616
7910	cg26810214	cg07746532	cg06807837
7911	cg26815121	cg07685601	cg19691659
7912	cg26818159	cg07674095	cg00816182
7913	cg26819427	cg07673230	cg01849845
7914	cg26828839	cg07585257	cg02084834
7915	cg26832915	cg07572251	cg19109601
7916	cg26835798	cg07565042	cg13851989
7917	cg26837766	cg07506407	cg01788682
7918	cg26840755	cg07487572	cg09875326
7919	cg26853057	cg07449447	cg07061582
7920	cg26856771	cg07425568	cg16996266
7921	cg26860182	cg07368061	cg03644585
7922	cg26862316	cg07296854	cg05535123
7923	cg26867987	cg07295362	cg07171780
7924	cg26871875	cg07284519	cg15874642
7925	cg26873164	cg07207742	cg00718694
7926	cg26877336	cg07181257	cg27318635
7927	cg26880445	cg07071809	cg25112643
7928	cg26882115	cg07061582	cg25479682
7929	cg26884345	cg07016298	cg11575912
7930	cg26886188	cg07008193	cg15277378
7931	cg26889437	cg06989253	cg04661959
7932	cg26889953	cg06925389	cg12486287
7933	cg26890480	cg06918474	cg09659803
7934	cg26891661	cg06813419	cg16476700
7935	cg26893743	cg06795827	cg03017946
7936	cg26894079	cg06793277	cg14274896
7937	cg26899399	cg06791695	cg26853057
7938	cg26905281	cg06768437	cg08986340
7939	cg26909797	cg06723357	cg09267483
7940	cg26915618	cg06694381	cg03639152
7941	cg26919387	cg06665485	cg24015175
7942	cg26923754	cg06662568	cg05816786
7943	cg26929394	cg06610254	cg04131010
7944	cg26931296	cg06553975	cg12856760
7945	cg26932586	cg06537652	cg05816006
7946	cg26932693	cg06521357	cg01448551
7947	cg26934759	cg06497934	cg02165692

7948	cg26934881	cg06387204	cg26014373
7949	cg26937267	cg06369532	cg12846837
7950	cg26939375	cg06332666	cg04837783
7951	cg26942121	cg06294856	cg04608722
7952	cg26947127	cg06255601	cg05528280
7953	cg26951000	cg06200670	cg08739576
7954	cg26954228	cg06179971	cg27306243
7955	cg26955275	cg05986052	cg11794120
7956	cg26960083	cg05917419	cg06873590
7957	cg26963844	cg05916989	cg06689039
7958	cg26964426	cg05856230	cg07813622
7959	cg26965897	cg05824594	cg08173959
7960	cg26969840	cg05712931	cg00047338
7961	cg26970113	cg05591270	cg16996571
7962	cg26974276	cg05579549	cg10458392
7963	cg26974415	cg05450563	cg24997330
7964	cg26974441	cg05340240	cg15076811
7965	cg26977086	cg05307141	cg06614534
7966	cg26977644	cg05253759	cg21620968
7967	cg26978064	cg05249271	cg21273407
7968	cg26987720	cg05202424	cg08273640
7969	cg26995244	cg05202347	cg14782266
7970	cg27000120	cg05133706	cg13263472
7971	cg27003787	cg05058103	cg26177754
7972	cg27004760	cg05037556	cg07927953
7973	cg27007717	cg04998447	cg01645401
7974	cg27008230	cg04924028	cg08605347
7975	cg27009448	cg04873169	cg15603885
7976	cg27010076	cg04761077	cg13358061
7977	cg27011829	cg04724387	cg22867608
7978	cg27014538	cg04689023	cg17201542
7979	cg27020028	cg04508340	cg17115147
7980	cg27021553	cg04473654	cg13658093
7981	cg27025137	cg04296187	cg09553581
7982	cg27026202	cg04220455	cg00118317
7983	cg27039312	cg04214430	cg07804434
7984	cg27040468	cg04165857	cg04854911
7985	cg27042523	cg04155280	cg07416344
7986	cg27044591	cg04034290	cg02040734
7987	cg27044641	cg03851401	cg19577054
7988	cg27049344	cg03762393	cg07992340
7989	cg27053040	cg03659476	cg27280313
7990	cg27053299	cg03629926	cg14323199
7991	cg27059537	cg03621841	cg01957900
7992	cg27065717	cg03618741	cg26394825
7993	cg27068650	cg03575974	cg01147665
7994	cg27070508	cg03501387	cg27641072
7995	cg27073066	cg03487040	cg05836492
7996	cg27073311	cg03445151	cg25909885
7997	cg27073349	cg03434029	cg19577082
7998	cg27074355	cg03424213	cg01567615
7999	cg27078464	cg03401464	cg16475712
8000	cg27082076	cg03381007	cg13464157

8001	cg27082292	cg03301025	cg22814801
8002	cg27089352	cg03294704	cg23288298
8003	cg27092594	cg03289072	cg15819921
8004	cg27095984	cg03162779	cg08106635
8005	cg27096087	cg03158194	cg07279281
8006	cg27096981	cg03143333	cg08433725
8007	cg27100149	cg02968890	cg07617152
8008	cg27100436	cg02920396	cg12955277
8009	cg27100471	cg02901644	cg21476203
8010	cg27101023	cg02817925	cg12149319
8011	cg27105598	cg02812189	cg27277104
8012	cg27110054	cg02764093	cg06024540
8013	cg27110374	cg02753511	cg05595469
8014	cg27117439	cg02742533	cg25513535
8015	cg27118035	cg02650728	cg24287189
8016	cg27120246	cg02643834	cg10829693
8017	cg27128001	cg02633363	cg27082292
8018	cg27133959	cg02613380	cg11405458
8019	cg27143695	cg02605292	cg16846503
8020	cg27143703	cg02604890	cg06213876
8021	cg27144884	cg02596645	cg19857633
8022	cg27146036	cg02570361	cg11372818
8023	cg27146477	cg02534048	cg20437892
8024	cg27156542	cg02369775	cg15016233
8025	cg27160524	cg02364518	cg26180006
8026	cg27161027	cg02344497	cg02447268
8027	cg27163630	cg02243479	cg26650973
8028	cg27168410	cg02156952	cg19284767
8029	cg27170206	cg02122052	cg07727134
8030	cg27171605	cg02013018	cg07420867
8031	cg27177415	cg01949455	cg03515290
8032	cg27177779	cg01923724	cg10214757
8033	cg27177983	cg01869896	cg00027990
8034	cg27178714	cg01835489	cg13842262
8035	cg27180994	cg01722498	cg04724387
8036	cg27181295	cg01715499	cg08067991
8037	cg27186013	cg01677310	cg23052758
8038	cg27192012	cg01604946	cg12163952
8039	cg27196496	cg01596854	cg24163616
8040	cg27200466	cg01567615	cg14507658
8041	cg27201457	cg01527159	cg03077533
8042	cg27202913	cg01503773	cg07849237
8043	cg27203917	cg01497892	cg14200357
8044	cg27207616	cg01454947	cg10532807
8045	cg27212729	cg01406317	cg22542420
8046	cg27213925	cg01403803	cg16238993
8047	cg27216597	cg01352276	cg26075905
8048	cg27221053	cg01350190	cg00950412
8049	cg27226424	cg01191157	cg20593887
8050	cg27226927	cg01176028	cg14777056
8051	cg27227317	cg01122461	cg02273647
8052	cg27228210	cg01017395	cg01719663
8053	cg27230140	cg00963675	cg25306579

8054	cg27233847	cg00945443	cg00412142
8055	cg27234648	cg00935361	cg15352671
8056	cg27239280	cg00890378	cg05340240
8057	cg27241873	cg00873009	cg25372722
8058	cg27250037	cg00866186	cg04568492
8059	cg27263448	cg00789416	cg26383057
8060	cg27269190	cg00701856	cg17907628
8061	cg27273675	cg00660140	cg13964068
8062	cg27275022	cg00563061	cg02011409
8063	cg27277104	cg00559439	cg14282937
8064	cg27280313	cg00547768	cg22752049
8065	cg27287438	cg00528490	cg00051546
8066	cg27287951	cg00470817	cg02827340
8067	cg27288127	cg00415011	cg13683219
8068	cg27290103	cg00401972	cg00631702
8069	cg27294008	cg00400654	cg09890597
8070	cg27295434	cg00386725	cg09172973
8071	cg27301230	cg00373967	cg08978399
8072	cg27302301	cg00359365	cg03300805
8073	cg27306243	cg00311883	cg08631328
8074	cg27306787	cg00270141	cg27120246
8075	cg27318635	cg00229868	cg03291336
8076	cg27320213	cg00216061	cg00038342
8077	cg27331828	cg00123090	cg03421440
8078	cg27333269	cg00027990	cg05588341
8079	cg27339974	cg00027650	cg16697024
8080	cg27343801	cg27632471	cg13636986
8081	cg27344022	cg27400447	cg20463808
8082	cg27346937	cg27364916	cg08494755
8083	cg27349909	cg27351239	cg09115960
8084	cg27351239	cg27318635	cg14550212
8085	cg27351813	cg27317493	cg12843033
8086	cg27360231	cg27275022	cg25586305
8087	cg27364916	cg27230140	cg02631921
8088	cg27365627	cg27171605	cg15230743
8089	cg27375286	cg27168410	cg12092201
8090	cg27378358	cg27134342	cg14783283
8091	cg27378814	cg27053299	cg19968041
8092	cg27380631	cg27003787	cg15617847
8093	cg27381488	cg26934881	cg15329179
8094	cg27382164	cg26832915	cg20397614
8095	cg27390206	cg26784300	cg16292933
8096	cg27396760	cg26754071	cg07296260
8097	cg27399387	cg26687830	cg16285424
8098	cg27399753	cg26550337	cg10517096
8099	cg27400447	cg26546882	cg07588779
8100	cg27401116	cg26514080	cg04091961
8101	cg27410136	cg26475911	cg15777335
8102	cg27410449	cg26428825	cg14738348
8103	cg27421679	cg26304689	cg13863769
8104	cg27424692	cg26194092	cg05506498
8105	cg27434984	cg26159074	cg20806143
8106	cg27437304	cg26075905	cg26166004

8107	cg27438218	cg26041493	cg19482885
8108	cg27441486	cg26037660	cg15171237
8109	cg27449255	cg25975823	cg14744022
8110	cg27454102	cg25909885	cg23089261
8111	cg27458631	cg25864762	cg11761615
8112	cg27466615	cg25793812	cg07431229
8113	cg27469606	cg25739943	cg09327610
8114	cg27475586	cg25723217	cg19092603
8115	cg27478167	cg25719378	cg24404329
8116	cg27485845	cg25699199	cg02935097
8117	cg27489873	cg25594550	cg26397391
8118	cg27491887	cg25445431	cg12481567
8119	cg27492839	cg25443991	cg19763281
8120	cg27493345	cg25399818	cg15382580
8121	cg27494100	cg25369553	cg05024916
8122	cg27495643	cg25339612	cg06750802
8123	cg27498434	cg25317315	cg23100081
8124	cg27503499	cg25245161	cg08644106
8125	cg27505472	cg25190718	cg01403803
8126	cg27506609	cg24916358	cg11469540
8127	cg27511289	cg24696473	cg22396663
8128	cg27521744	cg24678812	cg01716016
8129	cg27522078	cg24629431	cg13691622
8130	cg27526774	cg24619276	cg04744514
8131	cg27532130	cg24550880	cg09647797
8132	cg27533454	cg24549289	cg10959933
8133	cg27543578	cg24420089	cg15806038
8134	cg27548256	cg24394336	cg08428985
8135	cg27549186	cg24289452	cg27287951
8136	cg27554156	cg24268698	cg02115911
8137	cg27560818	cg24152000	cg04282530
8138	cg27565803	cg24034465	cg01065180
8139	cg27566403	cg23990272	cg18116267
8140	cg27567761	cg23906687	cg25390243
8141	cg27571329	cg23886515	cg18273464
8142	cg27571590	cg23748172	cg21379004
8143	cg27573308	cg23566503	cg24996315
8144	cg27579131	cg23536830	cg04545872
8145	cg27585557	cg23519637	cg00106093
8146	cg27594157	cg23477348	cg06113801
8147	cg27599376	cg23460369	cg21475076
8148	cg27604101	cg23454517	cg15475080
8149	cg27611699	cg23429698	cg02579022
8150	cg27612166	cg23399577	cg00825317
8151	cg27613016	cg23351271	cg22735889
8152	cg27616751	cg23333146	cg15208689
8153	cg27618145	cg23311929	cg07763497
8154	cg27618305	cg23249399	cg20997320
8155	cg27627006	cg23119433	cg22800332
8156	cg27630540	cg23092218	cg13461509
8157	cg27631766	cg23058194	cg18174089
8158	cg27634115	cg23036171	cg03359508
8159	cg27635271	cg22986999	cg24045498

8160	cg27637521	cg22986770	cg21687003
8161	cg27641072	cg22958118	cg05014846
8162	cg27641240	cg22942245	cg21270847
8163	cg27650212	cg22866998	cg19878200
8164	cg27655512	cg22866068	cg12112434
8165	cg27655716	cg22666103	cg03030665
8166	cg27662093	cg22639325	cg17976731
8167	cg06933824	cg22579075	cg10409799
8168	cg25230305	cg22563907	cg11155016
8169	cg26872742	cg22436253	cg00711584
8170	cg15899297	cg22245494	cg16617774
8171	cg04353106	cg22194948	cg13653963
8172	cg08954277	cg21978195	cg15798050
8173	cg01191157	cg21929969	cg09896120
8174	cg01283300	cg21927420	cg01150866
8175	cg01794929	cg21915998	cg26267483
8176	cg01683570	cg21811911	cg21268659
8177	cg22885000	cg21697252	cg25246084
8178	cg20778199	cg21628927	cg22605643
8179	cg15269148	cg21560157	cg17243540
8180	cg00229868	cg21558509	cg14244360
8181	cg02221422	cg21474062	cg26974276
8182	cg27423208	cg21460582	cg00289254
8183	cg05541267	cg21438602	cg00899856
8184	cg08384155	cg21385052	cg03495832
8185	cg20300032	cg21373806	cg25619703
8186	cg10404601	cg21269763	cg00713253
8187	cg01972651	cg21092828	cg21881364
8188	cg06752365	cg21004924	cg18035571
8189	cg14852276	cg20793665	cg25363336
8190	cg20555305	cg20758651	cg15639842
8191	cg15798050	cg20679659	cg21855211
8192	cg16277214	cg20677814	cg09641077
8193	cg13428516	cg20668447	cg04663956
8194	cg19044462	cg20602417	cg11081826
8195	cg10095226	cg20197756	cg12397802
8196	cg22638977	cg20152304	cg22069262
8197	cg07377299	cg20065217	cg08198711
8198	cg25900150	cg19968041	cg09832108
8199	cg14865516	cg19951269	cg11861709
8200	cg04685228	cg19950556	cg00890378
8201	cg06371291	cg19781117	cg25038926
8202	cg09851241	cg19769521	cg07050599
8203	cg16766632	cg19585100	cg26803670
8204	cg09685601	cg19560210	cg25699199
8205	cg22348713	cg19512961	cg21874862
8206	cg07328519	cg19486437	cg07811634
8207	cg00977384	cg19422138	cg22356839
8208	cg19569551	cg19402179	cg08263571
8209	cg27470978	cg19353949	cg09703323
8210	cg09970023	cg19296501	cg03764767
8211	cg08970011	cg19274401	cg06967828
8212	cg01819707	cg19137748	cg01216078

8213	cg19755886	cg19116924	cg02800817
8214	cg22854448	cg19101998	cg02742455
8215	cg25941751	cg19089328	cg11199862
8216	cg06310294	cg19063768	cg18965086
8217	cg25011577	cg19007948	cg05418508
8218	cg17778120	cg18994446	cg14325029
8219	cg19025193	cg18912160	cg17153432
8220	cg04688645	cg18794809	cg10247864
8221	cg15104211	cg18693479	cg24915915
8222	cg04714939	cg18514164	cg08749599
8223	cg08377924	cg18397743	cg23834688
8224	cg21696975	cg18330203	cg22329743
8225	cg25586305	cg18327762	cg25604994
8226	cg16726435	cg18311516	cg20011326
8227	cg21670987	cg18244708	cg02240030
8228	cg18696900	cg18193482	cg15063355
8229	cg04835685	cg18148488	cg12104982
8230	cg22136038	cg18116267	cg07800359
8231	cg27231912	cg18109289	cg06768437
8232	cg12375903	cg18000764	cg02083559
8233	cg12179658	cg17939585	cg08458912
8234	cg00442802	cg17929859	cg16222790
8235	cg20731213	cg17851345	cg19998675
8236	cg26953293	cg17685579	cg01991625
8237	cg01827633	cg17654747	cg17945541
8238	cg08124986	cg17589890	cg07396047
8239	cg03485217	cg17505642	cg17842918
8240	cg21549497	cg17468543	cg14829271
8241	cg08176056	cg17437086	cg21787369
8242	cg17907628	cg17394978	cg13434216
8243	cg09556902	cg17333042	cg23357150
8244	cg14288326	cg17321561	cg13343549
8245	cg12872329	cg17208278	cg07862575
8246	cg20673255	cg17172949	cg22834511
8247	cg01617074	cg17103856	cg07046047
8248	cg04601090	cg17095936	cg01062651
8249	cg20685600	cg16929793	cg04390667
8250	cg23005227	cg16786144	cg09495643
8251	cg06259557	cg16697024	cg01380319
8252	cg11237495	cg16564135	cg25097308
8253	cg17429870	cg16537245	cg04820362
8254	cg10885338	cg16509072	cg27613016
8255	cg06269419	cg16419526	cg06599914
8256	cg08994061	cg16398832	cg09849774
8257	cg23318523	cg16387532	cg09370299
8258	cg16684117	cg16385941	cg22546818
8259	cg25339612	cg16232530	cg18704595
8260	cg01923724	cg16184131	cg04967009
8261	cg18929814	cg16024937	cg21794816
8262	cg26763380	cg15988204	cg10762613
8263	cg17107501	cg15910594	cg07874353
8264	cg17961835	cg15819921	cg10590657
8265	cg10876003	cg15616400	cg01810347

8266	cg00592695	cg15600176	cg21373806
8267	cg12855271	cg15520941	cg11668876
8268	cg19901381	cg15488570	cg19296501
8269	cg02102456	cg15464763	cg03899978
8270	cg00637745	cg15459715	cg09028132
8271	cg10299941	cg15335728	cg14819399
8272	cg24696473	cg15322667	cg25006254
8273	cg04431002	cg15226226	cg26974415
8274	cg04762676	cg15208689	cg04269098
8275	cg13003178	cg15175632	cg27511289
8276	cg14769121	cg15168816	cg15582126
8277	cg10803714	cg15126957	cg19353949
8278	cg17766055	cg15089272	cg24847636
8279	cg08954601	cg15086474	cg21597937
8280	cg07205670	cg15026277	cg13774184
8281	cg22325145	cg14899960	cg21437521
8282	cg05638821	cg14777056	cg16312872
8283	cg10023624	cg14738348	cg14684166
8284	cg08677210	cg14723032	cg22397446
8285	cg03379270	cg14684068	cg03811905
8286	cg13537008	cg14626525	cg12842409
8287	cg25082361	cg14615152	cg04437605
8288	cg01194444	cg14565781	cg25519921
8289	cg15230743	cg14519294	cg07574267
8290	cg08435157	cg14516350	cg08409074
8291	cg11714334	cg14507427	cg27000120
8292	cg19770671	cg14470121	cg22342925
8293	cg15776300	cg14467840	cg26931296
8294	cg00374717	cg14425914	cg01342572
8295	cg06376598	cg14317513	cg11819469
8296	cg04574046	cg14285365	cg24387101
8297	cg01835854	cg14239346	cg11412527
8298	cg22706883	cg14140717	cg09165170
8299	cg12807855	cg14118424	cg22990430
8300	cg02972475	cg14025149	cg06509598
8301	cg24960639	cg13977320	cg26444806
8302	cg10511902	cg13922681	cg25635000
8303	cg00620733	cg13888853	cg15485665
8304	cg21735516	cg13745832	cg16502866
8305	cg19296662	cg13724812	cg17095936
8306	cg20386404	cg13700939	cg16819272
8307	cg07042489	cg13643040	cg01063615
8308	cg13700939	cg13620881	cg05494073
8309	cg13795838	cg13610622	cg07012739
8310	cg15417249	cg13530474	cg07064592
8311	cg20329153	cg13521018	cg19455322
8312	cg08264481	cg13521002	cg26543243
8313	cg19048950	cg13520715	cg13947385
8314	cg14507427	cg13470557	cg12119133
8315	cg09825309	cg13434216	cg26954228
8316	cg05816786	cg13390372	cg04920917
8317	cg00410600	cg13381243	cg00270654
8318	cg13555689	cg13369099	cg23254569

8319	cg02604211	cg13343549	cg01691522
8320	cg20489239	cg13320257	cg20916646
8321	cg07907315	cg13225881	cg10507281
8322	cg02263260	cg13206932	cg17319576
8323	cg00680875	cg13162581	cg05346688
8324	cg04349839	cg13133492	cg12093620
8325	cg08458637	cg13129380	cg25477334
8326	cg05150641	cg13109865	cg19824396
8327	cg22009923	cg12987059	cg27096087
8328	cg17473727	cg12855331	cg15249446
8329	cg02155048	cg12855271	cg06259557
8330	cg10836809	cg12790592	cg15899297
8331	cg02600978	cg12741436	cg10380108
8332	cg02883198	cg12680106	cg19190016
8333	cg08365388	cg12604490	cg12551582
8334	cg05601844	cg12591689	cg20686479
8335	cg15382580	cg12514933	cg03421964
8336	cg05700348	cg12163781	cg02065141
8337	cg27177296	cg12160664	cg04226256
8338	cg19750606	cg12093220	cg07091062
8339	cg08843064	cg12061219	cg17278466
8340	cg04131010	cg11997603	cg22846966
8341	cg25390635	cg11982525	cg11115867
8342	cg07410188	cg11940177	cg11227822
8343	cg20721135	cg11929154	cg16459391
8344	cg23408990	cg11888359	cg00948274
8345	cg07979388	cg11792186	cg23959115
8346	cg15639842	cg11701615	cg19879265
8347	cg22076676	cg11544882	cg24420164
8348	cg25807487	cg11523191	cg27493345
8349	cg10298992	cg11519725	cg17344813
8350	cg09231120	cg11415402	cg02087101
8351	cg01484266	cg11343506	cg03004417
8352	cg24425531	cg11327857	cg19319067
8353	cg15191744	cg11227822	cg25296804
8354	cg21183664	cg11210357	cg03596635
8355	cg13514778	cg11190890	cg15028232
8356	cg21606405	cg11189177	cg06537744
8357	cg00062282	cg10980293	cg09685505
8358	cg21238061	cg10969245	cg14711433
8359	cg05168368	cg10965164	cg26366087
8360	cg18149689	cg10811485	cg01484266
8361	cg12925881	cg10714509	cg20779414
8362	cg06131879	cg10689512	cg00648573
8363	cg00262446	cg10664162	cg10529994
8364	cg23173517	cg10642330	cg22987457
8365	cg08097657	cg10623198	cg16412000
8366	cg08644340	cg10519271	cg10244706
8367	cg24136288	cg10421435	cg01848660
8368	cg05899471	cg10364862	cg14497697
8369	cg00877758	cg10301695	cg20758651
8370	cg21189146	cg10263684	cg03888083
8371	cg02115911	cg10190898	cg00656991

8372	cg03391019	cg10065736	cg27572074
8373	cg21408581	cg10016358	cg23921860
8374	cg19034028	cg10009737	cg00426425
8375	cg04761077	cg09972881	cg01337429
8376	cg07262506	cg09951320	cg26977644
8377	cg21450547	cg09915883	cg01715499
8378	cg07498879	cg09890746	cg06037365
8379	cg26546882	cg09825309	cg16269776
8380	cg16717225	cg09780231	cg11103999
8381	cg13750566	cg09719124	cg13392861
8382	cg05988219	cg09603024	cg06104859
8383	cg13654085	cg09537448	cg22024295
8384	cg09775533	cg09536516	cg02628107
8385	cg02720600	cg09280800	cg14037250
8386	cg10529994	cg09258509	cg13476204
8387	cg22846809	cg09180239	cg12529228
8388	cg03991120	cg08994061	cg24425531
8389	cg01216078	cg08915024	cg22245494
8390	cg04399631	cg08829140	cg17013513
8391	cg20300651	cg08823837	cg20300032
8392	cg01641136	cg08814268	cg09171093
8393	cg26618058	cg08779982	cg21092828
8394	cg17821664	cg08677140	cg01375259
8395	cg20547131	cg08606951	cg09901532
8396	cg07795082	cg08552290	cg08003887
8397	cg17636309	cg08293536	cg03958928
8398	cg03041650	cg08264481	cg20167126
8399	cg09951320	cg08208595	cg06746744
8400	cg21494132	cg08173709	cg08411881
8401	cg02289020	cg08154963	cg26309261
8402	cg08360599	cg08088222	cg10024508
8403	cg00367649	cg08039592	cg18941831
8404	cg07561338	cg07964527	cg06758191
8405	cg03004417	cg07927540	cg02480405
8406	cg06218688	cg07874353	cg05798436
8407	cg00660140	cg07858195	cg06463230
8408	cg27514730	cg07811634	cg01577298
8409	cg07409629	cg07754347	cg15267890
8410	cg25141943	cg07577934	cg22992258
8411	cg27271532	cg07511723	cg26695387
8412	cg16383573	cg07385220	cg11620716
8413	cg20829550	cg07310500	cg05780637
8414	cg03053456	cg07294624	cg07584734
8415	cg08943374	cg07214314	cg03242880
8416	cg01272202	cg07184807	cg13080151
8417	cg07661965	cg07124859	cg10307940
8418	cg03722184	cg07123022	cg03119088
8419	cg03984919	cg07088950	cg12500891
8420	cg13984005	cg07066794	cg03939897
8421	cg02041470	cg07050611	cg10589235
8422	cg14122652	cg06996029	cg22831256
8423	cg18917736	cg06987369	cg13377839
8424	cg23820770	cg06869755	cg07478111

8425	cg00147160	cg06853492	cg05305993
8426	cg15878619	cg06577703	cg14691924
8427	cg26225655	cg06553058	cg11908294
8428	cg08317243	cg06443231	cg24857399
8429	cg01599714	cg06389574	cg24631518
8430	cg27134342	cg06131879	cg19784013
8431	cg12011299	cg06068392	cg04323313
8432	cg14811014	cg05934824	cg16897800
8433	cg13930682	cg05738470	cg01482620
8434	cg25699199	cg05637092	cg23261102
8435	cg16898124	cg05575733	cg13105904
8436	cg07521789	cg05561386	cg26392980
8437	cg19747744	cg05506498	cg03178838
8438	cg25993608	cg05415131	cg22657772
8439	cg00048381	cg05397809	cg21889263
8440	cg22304399	cg05390712	cg06255609
8441	cg18817459	cg05370136	cg05854826
8442	cg27158462	cg05360477	cg00083399
8443	cg16694397	cg05340658	cg27163630
8444	cg03223959	cg05294455	cg01456989
8445	cg27317493	cg05180954	cg14785464
8446	cg12690127	cg04911307	cg14679558
8447	cg00943124	cg04784212	cg04911307
8448	cg04661959	cg04771901	cg10790470
8449	cg24600706	cg04699214	cg20297053
8450	cg18240528	cg04456245	cg18811860
8451	cg01437411	cg04411052	cg02776768
8452	cg04273604	cg04399647	cg01040499
8453	cg07507493	cg04147497	cg24031446
8454	cg14297023	cg04124361	cg02374486
8455	cg13283765	cg03889044	cg18317492
8456	cg18919478	cg03824114	cg01851970
8457	cg16831894	cg03540589	cg14473062
8458	cg14691924	cg03419014	cg26289871
8459	cg15208689	cg03326128	cg00961932
8460	cg11620716	cg03300805	cg00627347
8461	cg04586928	cg03252568	cg00959308
8462	cg25625457	cg03058664	cg15956469
8463	cg02168436	cg03010561	cg22720481
8464	cg20072162	cg02973735	cg03270929
8465	cg20349199	cg02907662	cg24748448
8466	cg24244854	cg02876276	cg23072051
8467	cg16498681	cg02836864	cg13680188
8468	cg25677394	cg02823137	cg17321561
8469	cg04941831	cg02821693	cg02438575
8470	cg14096074	cg02776768	cg04290396
8471	cg05917419	cg02743136	cg03762393
8472	cg03430597	cg02718078	cg20133730
8473	cg14443041	cg02667880	cg09501516
8474	cg18517266	cg02628107	cg09669930
8475	cg15346619	cg02604211	cg04078658
8476	cg00373245	cg02597698	cg18912160
8477	cg26405979	cg02455836	cg04135242

8478	cg16554534	cg02436098	cg13940218
8479	cg09080909	cg02386311	cg03143333
8480	cg17348667	cg02367992	cg22750845
8481	cg10836406	cg02273647	cg20338075
8482	cg19092603	cg02249969	cg21045072
8483	cg13332783	cg02235663	cg05336268
8484	cg09941367	cg02217035	cg21927420
8485	cg00648573	cg02168436	cg05894015
8486	cg19968041	cg02155048	cg00806009
8487	cg25487008	cg02144518	cg00639143
8488	cg27278961	cg02083676	cg11203377
8489	cg24468934	cg01989275	cg25793812
8490	cg26444806	cg01957900	cg26187194
8491	cg01534871	cg01954438	cg08463581
8492	cg00961932	cg01945624	cg06675951
8493	cg02650728	cg01885080	cg08831531
8494	cg19897003	cg01860459	cg16883145
8495	cg13431342	cg01793617	cg00592944
8496	cg05493407	cg01778908	cg13414581
8497	cg01885080	cg01727408	cg23533073
8498	cg23834919	cg01679473	cg03095814
8499	cg24386135	cg01669185	cg09845604
8500	cg03938110	cg01653532	cg13116061
8501	cg00001687	cg01592387	cg18673163
8502	cg04906603	cg01484266	cg23509272
8503	cg26847438	cg01380884	cg09198283
8504	cg11523799	cg01288155	cg15346619
8505	cg18699466	cg01283625	cg09925620
8506	cg01661567	cg01216078	cg21404476
8507	cg02462933	cg01108243	cg27360231
8508	cg02010686	cg00948881	cg06621620
8509	cg04095995	cg00936935	cg02455836
8510	cg01501896	cg00920069	cg15637234
8511	cg15929241	cg00854594	cg25442652
8512	cg27538859	cg00713247	cg12591689
8513	cg06193668	cg00711584	cg17780565
8514	cg23756143	cg00706207	cg20566450
8515	cg11835806	cg00681103	cg01195672
8516	cg26934993	cg00674220	cg17428748
8517	cg23079522	cg00639837	cg15155463
8518	cg06752482	cg00618323	cg02221422
8519	cg24135293	cg00545796	cg02938429
8520	cg12982283	cg00426425	cg26818159
8521	cg05546878	cg00362381	cg17853089
8522	cg15317049	cg00275234	cg26880445
8523	cg13761321	cg00054496	cg05369142
8524	cg13392861	cg27594157	cg13849999
8525	cg25513535	cg27466615	cg12176815
8526	cg16776331	cg27278001	cg19992808
8527	cg00347938	cg27180994	cg10356060
8528	cg06246999	cg27118035	cg20941528
8529	cg01679473	cg27054948	cg23530850
8530	cg25112643	cg27008230	cg17586345

8531	cg07530947	cg26853057	cg03884079
8532	cg03472610	cg26815121	cg20761844
8533	cg19441674	cg26620147	cg24727133
8534	cg00955867	cg26441230	cg01260146
8535	cg27247807	cg26362368	cg12766383
8536	cg07214314	cg26225655	cg03077492
8537	cg09795809	cg26105283	cg09789315
8538	cg01801191	cg25932290	cg24018760
8539	cg27478020	cg25794830	cg16289101
8540	cg04847386	cg25745246	cg23066057
8541	cg14694342	cg25595431	cg00362381
8542	cg04800715	cg25555068	cg21747652
8543	cg03216506	cg25517187	cg05181397
8544	cg14095720	cg25203085	cg03444965
8545	cg05873568	cg25187161	cg03168587
8546	cg11412527	cg25141943	cg19437281
8547	cg03355204	cg25005357	cg27054948
8548	cg14833024	cg24870662	cg20436707
8549	cg16650096	cg24670927	cg12485685
8550	cg03141944	cg24541176	cg14264773
8551	cg05678033	cg24525461	cg01710084
8552	cg10307940	cg24455383	cg18855096
8553	cg21570597	cg24429037	cg15416179
8554	cg13475704	cg24366425	cg22251048
8555	cg05780637	cg24271718	cg11973602
8556	cg14362630	cg24264451	cg02172773
8557	cg17853089	cg24135293	cg17693222
8558	cg03333776	cg24102222	cg00275234
8559	cg21755755	cg23959115	cg23225637
8560	cg25595431	cg23784046	cg07067280
8561	cg13298538	cg23679798	cg10689512
8562	cg15737302	cg23114772	cg02146941
8563	cg25866889	cg23104930	cg02667335
8564	cg04293307	cg23058246	cg15935860
8565	cg03853328	cg22895198	cg09009788
8566	cg26543333	cg22846498	cg07746532
8567	cg01792229	cg22811647	cg24734735
8568	cg06618764	cg22657772	cg03953506
8569	cg12403778	cg22345063	cg17650713
8570	cg16741602	cg22256482	cg18610958
8571	cg00520380	cg21881364	cg18968623
8572	cg11320499	cg21856603	cg14971998
8573	cg00286014	cg21806238	cg25763426
8574	cg08159271	cg21805118	cg09742751
8575	cg02742455	cg21793437	cg20489239
8576	cg02696830	cg21784096	cg04591732
8577	cg03511735	cg21618273	cg27095984
8578	cg20927312	cg21574855	cg09754828
8579	cg05261433	cg21475781	cg00623312
8580	cg04165857	cg21376658	cg07951978
8581	cg20027331	cg21276022	cg12936779
8582	cg07997682	cg21238609	cg10664618
8583	cg26709300	cg21177940	cg07620571

8584	cg25825970	cg20963020	cg16521443
8585	cg18492803	cg20594316	cg10371037
8586	cg02711397	cg20293725	cg13676538
8587	cg03231163	cg20283716	cg13951223
8588	cg25590335	cg20265951	cg09743906
8589	cg10830021	cg19925801	cg26165908
8590	cg02187822	cg19839325	cg01486910
8591	cg13062396	cg19831403	cg20544406
8592	cg08774694	cg19786422	cg06836544
8593	cg03760490	cg19763281	cg26524387
8594	cg11633461	cg19486804	cg08812784
8595	cg16960291	cg19404500	cg04055053
8596	cg02595280	cg19400179	cg09976716
8597	cg06826908	cg19399165	cg02743136
8598	cg23457862	cg19219672	cg21393818
8599	cg18639238	cg19020855	cg23886515
8600	cg27440150	cg18704595	cg14519294
8601	cg27054948	cg18699466	cg27634115
8602	cg06061760	cg18317492	cg02297063
8603	cg21511816	cg18174089	cg05867158
8604	cg14890730	cg18098187	cg21532408
8605	cg26361671	cg18050634	cg18533833
8606	cg26540925	cg17931529	cg07477924
8607	cg00092644	cg17877600	cg04175417
8608	cg06332339	cg17821664	cg09029046
8609	cg26529094	cg17426166	cg18083415
8610	cg20586531	cg17054783	cg04534503
8611	cg05468559	cg17049328	cg05988219
8612	cg26281621	cg16898124	cg00620733
8613	cg01856887	cg16880392	cg01780685
8614	cg16642938	cg16861209	cg00863271
8615	cg26656452	cg16726435	cg08132025
8616	cg21750915	cg16617141	cg18249380
8617	cg21026566	cg16530165	cg23235334
8618	cg10171557	cg16499416	cg20685600
8619	cg04582672	cg16427420	cg11027058
8620	cg08447373	cg16383573	cg10902667
8621	cg16554164	cg16311946	cg16532399
8622	cg10812717	cg16245035	cg27567761
8623	cg08095452	cg16236270	cg27532130
8624	cg07872947	cg16200325	cg08291302
8625	cg25232510	cg16051954	cg15888693
8626	cg01404873	cg15991104	cg22217449
8627	cg27572074	cg15956469	cg02364518
8628	cg25736617	cg15712057	cg04687939
8629	cg23976336	cg15676677	cg25135706
8630	cg03882967	cg15627277	cg25199850
8631	cg14723032	cg15548656	cg13750566
8632	cg16217297	cg15543534	cg01553388
8633	cg05370136	cg15465367	cg04158792
8634	cg01567615	cg14879569	cg26297299
8635	cg01361499	cg14651518	cg25161092
8636	cg19241089	cg14599015	cg17537252

8637	cg14081251	cg14473062	cg03684893
8638	cg04473209	cg14431528	cg11576988
8639	cg13957126	cg14401746	cg18348566
8640	cg11781564	cg14294646	cg01885080
8641	cg23887746	cg14166377	cg13467292
8642	cg26941073	cg14101687	cg12392429
8643	cg13737493	cg14062899	cg19296662
8644	cg05062820	cg14037652	cg07701911
8645	cg09828346	cg13821287	cg06387204
8646	cg05327192	cg13808180	cg16245035
8647	cg18384190	cg13763232	cg10774480
8648	cg09612502	cg13582959	cg00818557
8649	cg03575245	cg13393917	cg05159066
8650	cg13414581	cg13319468	cg01337736
8651	cg02876276	cg13248406	cg14641266
8652	cg16784943	cg12743638	cg11585301
8653	cg15710638	cg12681784	cg21389456
8654	cg14204266	cg12511749	cg26987720
8655	cg27323343	cg12491594	cg15988812
8656	cg01953240	cg12474695	cg24530000
8657	cg23876072	cg12468255	cg09238162
8658	cg04650653	cg12417871	cg05340658
8659	cg17822706	cg12384918	cg21998505
8660	cg01454947	cg12284521	cg04077677
8661	cg08705647	cg12149319	cg13429555
8662	cg13276704	cg12062088	cg25506288
8663	cg06873590	cg11980500	cg12522144
8664	cg14819399	cg11952839	cg02720600
8665	cg26338030	cg11909137	cg01377082
8666	cg26868306	cg11865296	cg21011702
8667	cg27588653	cg11697861	cg07929112
8668	cg10498921	cg11633461	cg01415527
8669	cg13431028	cg11612905	cg13165390
8670	cg00869582	cg11390978	cg11457582
8671	cg12104982	cg11390504	cg09824023
8672	cg23921860	cg11372818	cg23754431
8673	cg02534048	cg11254847	cg20342460
8674	cg05668807	cg11241684	cg11979837
8675	cg06509598	cg11203377	cg14326263
8676	cg07132183	cg11148096	cg23735646
8677	cg24594604	cg11135937	cg10152624
8678	cg04132177	cg11124652	cg23820770
8679	cg10235845	cg11051022	cg16597437
8680	cg18465694	cg10902667	cg18348731
8681	cg27604702	cg10796068	cg21751147
8682	cg10865444	cg10589235	cg01442132
8683	cg04175292	cg10510707	cg18149689
8684	cg22054793	cg10317175	cg22576033
8685	cg09961689	cg10109146	cg13461718
8686	cg08929612	cg10077985	cg05361262
8687	cg18729298	cg09637963	cg03695693
8688	cg17700443	cg09494646	cg09886931
8689	cg02389084	cg09459955	cg09951320

8690	cg14952488	cg09372617	cg22082745
8691	cg20800216	cg09184899	cg04360147
8692	cg09258240	cg09124230	cg11254317
8693	cg18318722	cg09074223	cg22846611
8694	cg15478081	cg08831531	cg10294836
8695	cg19332409	cg08826080	cg03089923
8696	cg26714514	cg08818866	cg20794957
8697	cg25939647	cg08701675	cg06397381
8698	cg12356870	cg08616182	cg15048900
8699	cg27130493	cg08553857	cg09711421
8700	cg26639076	cg08537794	cg23119433
8701	cg11527930	cg08441269	cg25852715
8702	cg18878210	cg08426200	cg16442814
8703	cg19640754	cg08386696	cg12995933
8704	cg01309081	cg08258867	cg03556771
8705	cg06662568	cg08168529	cg03892631
8706	cg00550617	cg08089518	cg01314034
8707	cg10864794	cg07979388	cg24856699
8708	cg11046030	cg07962882	cg27650212
8709	cg09657114	cg07841877	cg23401741
8710	cg04290396	cg07813622	cg21593628
8711	cg11879277	cg07732097	cg21057429
8712	cg24812143	cg07598035	cg22819824
8713	cg10709026	cg07483007	cg03738365
8714	cg08818866	cg07433453	cg26341003
8715	cg06696911	cg07206676	cg02583282
8716	cg21476203	cg07204803	cg16453673
8717	cg25865108	cg07091062	cg23738548
8718	cg03108347	cg07050599	cg14462369
8719	cg13133492	cg07020987	cg22890571
8720	cg06183287	cg06881421	cg01110955
8721	cg18560571	cg06836544	cg14937228
8722	cg25178749	cg06622892	cg06084403
8723	cg10488777	cg06549824	cg01287012
8724	cg09988676	cg06513888	cg10890199
8725	cg01860459	cg06371291	cg13457217
8726	cg07665929	cg06333135	cg24264451
8727	cg07875385	cg06321304	cg02454890
8728	cg02838178	cg06313479	cg20137441
8729	cg08985282	cg06219732	cg26001655
8730	cg23514135	cg06078337	cg09123961
8731	cg01306662	cg06061092	cg09776314
8732	cg00963675	cg05975710	cg08750440
8733	cg02351425	cg05780637	cg17364872
8734	cg23108126	cg05711037	cg18842598
8735	cg13701991	cg05528280	cg22466400
8736	cg09028383	cg05488981	cg10965164
8737	cg00903308	cg05482864	cg00410600
8738	cg06263395	cg05459650	cg14560240
8739	cg16770054	cg05452391	cg11809476
8740	cg27663249	cg05400252	cg22194948
8741	cg01691522	cg05349039	cg12071544
8742	cg19603100	cg05337637	cg06921552

8743	cg25749254	cg05272587	cg01324452
8744	cg07891483	cg05269678	cg24830876
8745	cg02722657	cg04986373	cg20658711
8746	cg08619651	cg04921335	cg20407868
8747	cg02936315	cg04805065	cg27382164
8748	cg07184807	cg04800715	cg02048412
8749	cg07888912	cg04719721	cg19200440
8750	cg10550166	cg04703221	cg10990188
8751	cg26037660	cg04624413	cg07880135
8752	cg27092248	cg04618903	cg20490386
8753	cg20190649	cg04535391	cg27343801
8754	cg00830283	cg04505809	cg07986222
8755	cg17898124	cg04398282	cg13962153
8756	cg05516020	cg04356968	cg02304427
8757	cg07283582	cg03939897	cg13994897
8758	cg10809282	cg03811905	cg10813908
8759	cg20785796	cg03764767	cg12822242
8760	cg17193551	cg03556771	cg02825527
8761	cg09957712	cg03514843	cg13348246
8762	cg00948274	cg03441279	cg06369532
8763	cg00582671	cg03385114	cg01954438
8764	cg27406975	cg03379270	cg15520941
8765	cg26311262	cg03270929	cg16114651
8766	cg19120897	cg03216729	cg23614979
8767	cg26685539	cg03131732	cg10968396
8768	cg25834632	cg03110167	cg04296187
8769	cg19951269	cg03072692	cg06483517
8770	cg02097309	cg02976723	cg04906603
8771	cg10102736	cg02961196	cg12878382
8772	cg23351271	cg02818775	cg12971523
8773	cg22367886	cg02806032	cg23957850
8774	cg20699157	cg02723533	cg08183125
8775	cg04854911	cg02685896	cg27604702
8776	cg26642540	cg02656049	cg15802323
8777	cg05014846	cg02556345	cg08943374
8778	cg13851989	cg02480405	cg00753492
8779	cg26368942	cg02463970	cg26066560
8780	cg04560531	cg02363010	cg14144513
8781	cg19233001	cg02283353	cg11198094
8782	cg02579022	cg02237875	cg02714192
8783	cg04353997	cg02193283	cg07066794
8784	cg08186671	cg02190127	cg25977879
8785	cg00688979	cg02065141	cg11409060
8786	cg04137490	cg01881549	cg04724190
8787	cg13573244	cg01780685	cg18493371
8788	cg12549211	cg01723892	cg09680719
8789	cg10370426	cg01696984	cg17822706
8790	cg19495444	cg01613294	cg23216122
8791	cg07296260	cg01598009	cg07976390
8792	cg00959308	cg01578875	cg07925615
8793	cg13162581	cg01388243	cg13675389
8794	cg00513981	cg01343045	cg19706515
8795	cg23719367	cg01290421	cg13109865

8796	cg19220272	cg01274233	cg03137908
8797	cg14270348	cg01185921	cg23834427
8798	cg26165908	cg01055121	cg18067847
8799	cg04479713	cg01046905	cg02147208
8800	cg15125438	cg01023169	cg00990874
8801	cg19274401	cg00969565	cg06826908
8802	cg02573234	cg00943124	cg12153709
8803	cg19827875	cg00680277	cg21570597
8804	cg13947999	cg00610577	cg09379188
8805	cg23957850	cg00512872	cg00039385
8806	cg09159050	cg00501242	cg14321522
8807	cg23630423	cg00470794	cg17599809
8808	cg26463171	cg00412142	cg19358589
8809	cg10109635	cg00400992	cg24360745
8810	cg00356131	cg00226615	cg14159036
8811	cg09304617	cg00209918	cg04729730
8812	cg00861010	cg00180097	cg25227364
8813	cg13366501	cg00047338	cg06056558
8814	cg23690444	cg27630540	cg07512971
8815	cg06746744	cg27423208	cg16498681
8816	cg00123317	cg27394987	cg03269976
8817	cg07115952	cg27323343	cg20017253
8818	cg14094409	cg27288127	cg20667709
8819	cg00437969	cg27226927	cg19863040
8820	cg14783283	cg27128001	cg07495357
8821	cg24969303	cg27078464	cg08972190
8822	cg18461635	cg26408861	cg00918130
8823	cg25784372	cg25490527	cg11028291
8824	cg19445044	cg25481201	cg00802617
8825	cg13194638	cg25439867	cg11512026
8826	cg22529541	cg24885442	cg23722792
8827	cg00007036	cg24706188	cg04002957
8828	cg00290626	cg24037270	cg25028527
8829	cg22286382	cg23825482	cg07166333
8830	cg00352031	cg23748751	cg09253696
8831	cg05415131	cg23677881	cg09796786
8832	cg21574855	cg23322122	cg00488512
8833	cg03939897	cg23254569	cg25607321
8834	cg08263571	cg23117250	cg25954194
8835	cg04847478	cg23072051	cg24093176
8836	cg03376089	cg22999502	cg20650515
8837	cg06372475	cg22867608	cg16826504
8838	cg10194844	cg22622164	cg19253339
8839	cg20784591	cg22472304	cg09975093
8840	cg04998447	cg21806750	cg18203044
8841	cg11093939	cg21747652	cg24408200
8842	cg17636986	cg20863668	cg20254251
8843	cg05602356	cg20658711	cg25892761
8844	cg00937817	cg20566450	cg11806749
8845	cg26739233	cg20412217	cg00244267
8846	cg24773493	cg20229025	cg07907315
8847	cg14200357	cg19975759	cg15283164
8848	cg23401741	cg19643097	cg16666964

8849	cg11701615	cg19437281	cg10515332
8850	cg05035470	cg19055828	cg19747744
8851	cg19793499	cg19047283	cg18636829
8852	cg26524387	cg18930928	cg02656049
8853	cg17616283	cg18652285	cg19500393
8854	cg22963109	cg18581669	cg14010305
8855	cg01824603	cg18565130	cg14565408
8856	cg15181351	cg18485872	cg00060715
8857	cg13840520	cg18266458	cg25945504
8858	cg19998675	cg18058895	cg03919694
8859	cg02325128	cg17977133	cg20154222
8860	cg05750323	cg17789138	cg25010788
8861	cg27127017	cg17278466	cg14030593
8862	cg05813328	cg16772023	cg11272705
8863	cg03218909	cg16766632	cg04510807
8864	cg07463059	cg16766036	cg22555262
8865	cg19883388	cg16668534	cg22913843
8866	cg03068497	cg16125397	cg14547067
8867	cg17693222	cg15988812	cg14234732
8868	cg26100137	cg15881332	cg26365784
8869	cg20122645	cg15383141	cg09649196
8870	cg02812189	cg15089181	cg17444642
8871	cg10642094	cg14995235	cg26405979
8872	cg17534540	cg14939096	cg15850851
8873	cg00785831	cg14782266	cg09318508
8874	cg02486904	cg14616881	cg16664095
8875	cg02013841	cg14598025	cg08206156
8876	cg06549824	cg14565439	cg22046535
8877	cg05884394	cg14507658	cg15191744
8878	cg04590974	cg14500614	cg04399647
8879	cg01375259	cg14429457	cg08910524
8880	cg13232249	cg14041194	cg17418956
8881	cg03254566	cg13838713	cg04124361
8882	cg18989491	cg13694343	cg14742341
8883	cg18938534	cg13222945	cg16223967
8884	cg09519954	cg12678686	cg22765117
8885	cg16711983	cg12529228	cg18400889
8886	cg11254847	cg12467090	cg13514824
8887	cg15894581	cg12412384	cg08974450
8888	cg26804423	cg12271433	cg11997603
8889	cg04622001	cg11852646	cg00961416
8890	cg01359676	cg11725852	cg25568745
8891	cg19956769	cg11724511	cg12796332
8892	cg16242615	cg11024450	cg19598875
8893	cg00223767	cg10830021	cg25461827
8894	cg19640166	cg10667207	cg04261952
8895	cg04341806	cg10363202	cg24294013
8896	cg16560256	cg10311315	cg04844977
8897	cg25275372	cg10262770	cg18036710
8898	cg21964551	cg10244706	cg06916574
8899	cg21483216	cg09941367	cg03639170
8900	cg19494591	cg09680719	cg01655150
8901	cg26280578	cg09268338	cg22047338

8902	cg04034290	cg09255505	cg11087939
8903	cg03135948	cg09073443	cg03674127
8904	cg10969245	cg08894066	cg19240021
8905	cg04842880	cg08843756	cg19277672
8906	cg13526766	cg08535779	cg21409449
8907	cg12895304	cg08112616	cg06748078
8908	cg04384810	cg07160118	cg13587802
8909	cg01287012	cg07102397	cg04800715
8910	cg06749819	cg07086112	cg09268338
8911	cg22222413	cg07078758	cg01333205
8912	cg02365900	cg06796435	cg19116924
8913	cg19358589	cg06750802	cg11722816
8914	cg13680246	cg06632821	cg23677881
8915	cg09505809	cg06621620	cg11161142
8916	cg03971344	cg06397381	cg26466921
8917	cg12776287	cg06338958	cg13165992
8918	cg05631399	cg06264060	cg03938353
8919	cg26180006	cg06246000	cg17993073
8920	cg01971590	cg05989746	cg03988119
8921	cg11468233	cg05876246	cg15617336
8922	cg13661703	cg05869732	cg03333116
8923	cg21573345	cg05846044	cg17024719
8924	cg15874642	cg05777316	cg10673740
8925	cg09119854	cg05757474	cg12797609
8926	cg11562411	cg05590948	cg14101193
8927	cg27632471	cg05404787	cg05615230
8928	cg03739877	cg05188868	cg05918682
8929	cg23421128	cg04460041	cg16898124
8930	cg06332666	cg04099803	cg15072038
8931	cg26164879	cg03853328	cg26819427
8932	cg19435621	cg03834055	cg06460400
8933	cg23280294	cg03726437	cg09858160
8934	cg16159491	cg03648611	cg03808580
8935	cg18592315	cg03495660	cg02364610
8936	cg15470344	cg03142002	cg16255804
8937	cg04311473	cg03051577	cg23104930
8938	cg18120259	cg03013188	cg26368942
8939	cg08038054	cg02972941	cg06769510
8940	cg06793277	cg02372856	cg10071852
8941	cg08986340	cg02327654	cg03789088
8942	cg04575609	cg02317251	cg10392840
8943	cg16398832	cg02147208	cg27004760
8944	cg12156838	cg01851970	cg12448664
8945	cg03727333	cg01385157	cg18925548
8946	cg27248741	cg01342572	cg26941073
8947	cg19995891	cg01197763	cg05554592
8948	cg00512280	cg01032946	cg16026212
8949	cg02009766	cg00994984	cg05727180
8950	cg20329085	cg00981003	cg00414709
8951	cg00783706	cg00747342	cg12356870
8952	cg10877385	cg00688979	cg27339974
8953	cg17781669	cg00637826	cg09063434
8954	cg15853299	cg00352652	cg27394987
