# Supplementary material for: Identification of Methylation Signatures and Rules for Sarcoma Subtypes by Machine Learning Methods
Source: Biomed Res Int. 2022 Dec 28;2022:5297235. doi: 10.1155/2022/5297235 (PMC9812612; doi:10.1155/2022/5297235)
Supplement: Supplementary Materials — Table S1: fifty-nine sarcoma subtypes and their sample sizes. Table S2: feature ranking results obtained using LASSO, LightGBM, and MCFS. Table S3: performance of IFS with different classification algorithms on three feature lists. Table S4: gene symbols obtained by annotating the most essential methylation sites derived from the LASSO, LightGBM, and MCFS feature lists. Table S5: intersection of three gene sets annotated by most essential methylation sites extracted from the LASSO, LightGBM, and MCFS feature lists. The genes that appear in the 3, 2, and 1 gene subsets are shown. Table S6: classification rules generated by decision tree using its optimal features on three feature lists. [file 5297235.f1.zip › Table S3 (1).pdf]

Table S3: Performance of IFS with different classification algorithms on three feature lists.

(1) IFS results with RF on the LASSO feature list

Number of features	ACC	MCC	Macro F1	Weighted F1
10	0.802	0.797	0.830	0.797
20	0.929	0.927	0.942	0.928
30	0.950	0.948	0.960	0.949
40	0.962	0.961	0.971	0.962
50	0.961	0.960	0.969	0.960
60	0.957	0.956	0.968	0.956
70	0.963	0.962	0.971	0.962
80	0.961	0.960	0.970	0.960
90	0.969	0.968	0.977	0.968
100	0.964	0.963	0.974	0.963
110	0.971	0.971	0.980	0.971
120	0.970	0.969	0.977	0.969
130	0.974	0.973	0.980	0.973
140	0.972	0.971	0.980	0.971
150	0.971	0.971	0.980	0.971
160	0.973	0.972	0.979	0.972
170	0.978	0.977	0.986	0.977
180	0.969	0.968	0.978	0.968
190	0.973	0.972	0.979	0.972
200	0.974	0.973	0.981	0.973
210	0.971	0.970	0.981	0.970
220	0.974	0.973	0.981	0.973
230	0.973	0.972	0.980	0.972
240	0.978	0.977	0.984	0.977
250	0.978	0.977	0.983	0.977
260	0.974	0.973	0.980	0.974
270	0.976	0.976	0.982	0.976
280	0.977	0.976	0.984	0.976
290	0.978	0.978	0.985	0.978
300	0.977	0.976	0.983	0.977
310	0.977	0.976	0.983	0.976
320	0.978	0.978	0.985	0.978
330	0.977	0.976	0.984	0.977
340	0.978	0.977	0.984	0.977
350	0.980	0.979	0.987	0.979
360	0.977	0.976	0.985	0.977
370	0.976	0.975	0.984	0.975
380	0.979	0.978	0.985	0.979

390	0.976	0.976	0.981	0.976
400	0.971	0.971	0.980	0.971
410	0.978	0.977	0.985	0.977
420	0.979	0.978	0.984	0.978
430	0.982	0.981	0.986	0.981
440	0.982	0.982	0.987	0.982
450	0.973	0.972	0.980	0.972
460	0.980	0.980	0.986	0.980
470	0.978	0.977	0.983	0.977
480	0.980	0.980	0.986	0.980
490	0.977	0.976	0.983	0.976
500	0.980	0.979	0.986	0.979
510	0.976	0.975	0.983	0.975
520	0.977	0.976	0.983	0.976
530	0.979	0.978	0.984	0.978
540	0.976	0.976	0.982	0.976
550	0.980	0.979	0.986	0.979
560	0.974	0.973	0.982	0.973
570	0.977	0.976	0.984	0.976
580	0.980	0.980	0.987	0.980
590	0.980	0.980	0.986	0.980
600	0.976	0.976	0.981	0.976
610	0.980	0.980	0.986	0.980
620	0.980	0.980	0.986	0.980
630	0.979	0.978	0.984	0.978
640	0.978	0.978	0.985	0.978
650	0.980	0.979	0.983	0.979
660	0.979	0.978	0.984	0.979
670	0.980	0.979	0.984	0.979
680	0.981	0.980	0.987	0.981
690	0.980	0.979	0.984	0.979
700	0.975	0.974	0.981	0.974
710	0.978	0.978	0.984	0.978
720	0.978	0.977	0.983	0.977
730	0.978	0.977	0.983	0.977
740	0.978	0.978	0.984	0.978
750	0.980	0.979	0.987	0.979
760	0.981	0.980	0.987	0.980
770	0.979	0.978	0.984	0.978
780	0.976	0.975	0.985	0.975
790	0.982	0.981	0.988	0.981
800	0.981	0.980	0.987	0.981
810	0.980	0.980	0.986	0.980

820	0.979	0.978	0.986	0.978
830	0.981	0.980	0.987	0.980
840	0.981	0.980	0.986	0.980
850	0.976	0.976	0.984	0.976
860	0.982	0.982	0.986	0.982
870	0.980	0.979	0.985	0.979
880	0.980	0.980	0.985	0.980
890	0.983	0.983	0.987	0.983
900	0.978	0.978	0.984	0.978
910	0.981	0.980	0.987	0.981
920	0.979	0.978	0.985	0.979
930	0.979	0.978	0.986	0.978
940	0.980	0.980	0.985	0.980
950	0.980	0.980	0.986	0.980
960	0.981	0.980	0.986	0.981
970	0.980	0.980	0.985	0.980
980	0.979	0.978	0.985	0.978
990	0.981	0.980	0.987	0.980
1000	0.980	0.980	0.986	0.980
1010	0.980	0.979	0.985	0.979
1020	0.977	0.976	0.984	0.976
1030	0.981	0.980	0.986	0.981
1040	0.979	0.978	0.984	0.979
1050	0.980	0.979	0.986	0.980
1060	0.977	0.976	0.982	0.976
1070	0.982	0.981	0.987	0.981
1080	0.978	0.977	0.985	0.977
1090	0.980	0.979	0.985	0.979
1100	0.981	0.980	0.987	0.981
1110	0.977	0.976	0.983	0.977
1120	0.979	0.978	0.985	0.979
1130	0.982	0.981	0.986	0.981
1140	0.982	0.982	0.987	0.982
1150	0.978	0.977	0.984	0.977
1160	0.978	0.978	0.984	0.978
1170	0.977	0.976	0.984	0.976
1180	0.980	0.979	0.985	0.979
1190	0.981	0.980	0.987	0.981
1200	0.979	0.978	0.985	0.978
1210	0.980	0.979	0.985	0.980
1220	0.982	0.981	0.987	0.981
1230	0.978	0.978	0.984	0.978
1240	0.978	0.978	0.985	0.978

1250	0.983	0.983	0.988	0.983
1260	0.980	0.979	0.986	0.979
1270	0.977	0.976	0.984	0.976
1280	0.981	0.980	0.986	0.981
1290	0.976	0.976	0.983	0.976
1300	0.978	0.977	0.982	0.977
1310	0.980	0.979	0.984	0.979
1320	0.980	0.979	0.986	0.979
1330	0.978	0.978	0.984	0.978
1340	0.979	0.978	0.983	0.978
1350	0.977	0.976	0.984	0.976
1360	0.976	0.976	0.983	0.976
1370	0.976	0.976	0.983	0.976
1380	0.980	0.979	0.983	0.980
1390	0.979	0.978	0.985	0.979
1400	0.978	0.978	0.984	0.978
1410	0.981	0.980	0.986	0.981
1420	0.980	0.980	0.987	0.980
1430	0.978	0.978	0.984	0.978
1440	0.977	0.976	0.983	0.976
1450	0.980	0.979	0.984	0.979
1460	0.979	0.978	0.985	0.979
1470	0.981	0.980	0.986	0.981
1480	0.978	0.977	0.984	0.977
1490	0.983	0.983	0.988	0.983
1500	0.978	0.977	0.984	0.977
1510	0.979	0.978	0.986	0.979
1520	0.980	0.979	0.985	0.980
1530	0.984	0.983	0.988	0.984
1540	0.981	0.980	0.987	0.981
1550	0.978	0.978	0.984	0.978
1560	0.982	0.981	0.987	0.981
1570	0.978	0.978	0.985	0.978
1580	0.978	0.977	0.984	0.977
1590	0.980	0.980	0.986	0.980
1600	0.976	0.975	0.982	0.975
1610	0.982	0.981	0.987	0.982
1620	0.978	0.978	0.983	0.978
1630	0.978	0.978	0.986	0.978
1640	0.980	0.980	0.986	0.980
1650	0.980	0.980	0.983	0.980
1660	0.980	0.979	0.984	0.979
1670	0.982	0.981	0.987	0.982

1680	0.977	0.976	0.983	0.977
1690	0.982	0.982	0.986	0.982
1700	0.979	0.978	0.985	0.979
1710	0.982	0.981	0.987	0.981
1720	0.979	0.978	0.986	0.979
1730	0.980	0.980	0.985	0.980
1740	0.981	0.980	0.987	0.981
1750	0.979	0.978	0.985	0.979
1760	0.982	0.982	0.988	0.982
1770	0.980	0.979	0.985	0.980
1780	0.982	0.981	0.988	0.981
1790	0.979	0.978	0.984	0.979
1800	0.984	0.984	0.988	0.984
1810	0.980	0.979	0.986	0.979
1820	0.981	0.980	0.988	0.981
1830	0.980	0.980	0.986	0.980
1840	0.980	0.980	0.987	0.980
1850	0.983	0.983	0.987	0.983
1860	0.982	0.981	0.988	0.981
1870	0.982	0.981	0.986	0.981
1880	0.982	0.981	0.987	0.982
1890	0.984	0.983	0.988	0.984
1900	0.982	0.982	0.987	0.982
1910	0.976	0.976	0.983	0.976
1920	0.982	0.981	0.988	0.981
1930	0.983	0.983	0.989	0.983
1940	0.982	0.982	0.988	0.982
1950	0.980	0.979	0.983	0.980
1960	0.980	0.980	0.986	0.980
1970	0.984	0.984	0.989	0.984
1980	0.981	0.980	0.986	0.981
1990	0.981	0.980	0.985	0.981
2000	0.982	0.981	0.987	0.981
2010	0.980	0.979	0.986	0.979
2020	0.981	0.980	0.988	0.981
2030	0.982	0.982	0.986	0.982
2040	0.982	0.981	0.987	0.981
2050	0.981	0.980	0.986	0.981
2060	0.982	0.981	0.986	0.981
2070	0.986	0.985	0.990	0.986
2080	0.980	0.980	0.987	0.980
2090	0.982	0.981	0.987	0.981
2100	0.980	0.979	0.986	0.979

2110	0.983	0.983	0.989	0.983
2120	0.978	0.978	0.985	0.978
2130	0.982	0.982	0.988	0.982
2140	0.982	0.982	0.988	0.982
2150	0.984	0.984	0.989	0.984
2160	0.983	0.983	0.987	0.983
2170	0.980	0.979	0.985	0.979
2180	0.981	0.980	0.987	0.981
2190	0.981	0.980	0.986	0.981
2200	0.984	0.983	0.987	0.984
2210	0.980	0.980	0.987	0.980
2220	0.980	0.979	0.985	0.979
2230	0.978	0.977	0.984	0.977
2240	0.980	0.979	0.986	0.979
2250	0.982	0.981	0.987	0.981
2260	0.984	0.983	0.988	0.984
2270	0.981	0.980	0.988	0.981
2280	0.982	0.982	0.987	0.982
2290	0.983	0.983	0.988	0.983
2300	0.982	0.982	0.988	0.982
2310	0.980	0.980	0.986	0.980
2320	0.982	0.981	0.988	0.981
2330	0.982	0.981	0.987	0.982
2340	0.979	0.978	0.986	0.979
2350	0.981	0.980	0.986	0.981
2360	0.980	0.979	0.986	0.979
2370	0.980	0.980	0.987	0.980
2380	0.983	0.983	0.987	0.983
2390	0.982	0.981	0.987	0.981
2400	0.983	0.983	0.988	0.983
2410	0.981	0.980	0.986	0.981
2420	0.979	0.978	0.986	0.979
2430	0.979	0.978	0.987	0.979
2440	0.984	0.983	0.989	0.983
2450	0.981	0.980	0.986	0.981
2460	0.984	0.983	0.988	0.984
2470	0.981	0.980	0.986	0.981
2480	0.980	0.980	0.986	0.980
2490	0.981	0.980	0.987	0.981
2500	0.980	0.980	0.985	0.980
2510	0.982	0.981	0.986	0.981
2520	0.980	0.980	0.986	0.980
2530	0.980	0.979	0.986	0.979

2540	0.982	0.982	0.986	0.982
2550	0.983	0.983	0.988	0.983
2560	0.980	0.979	0.985	0.979
2570	0.980	0.980	0.985	0.980
2580	0.980	0.979	0.986	0.979
2590	0.984	0.983	0.988	0.984
2600	0.981	0.980	0.987	0.981
2610	0.984	0.983	0.989	0.984
2620	0.980	0.979	0.986	0.979
2630	0.981	0.980	0.986	0.981
2640	0.985	0.985	0.989	0.985
2650	0.983	0.983	0.988	0.983
2660	0.981	0.980	0.985	0.981
2670	0.978	0.978	0.984	0.978
2680	0.982	0.981	0.987	0.982
2690	0.981	0.980	0.986	0.981
2700	0.982	0.981	0.987	0.982
2710	0.984	0.984	0.990	0.984
2720	0.981	0.980	0.988	0.981
2730	0.982	0.981	0.988	0.982
2740	0.984	0.984	0.989	0.984
2750	0.982	0.981	0.987	0.982
2760	0.981	0.980	0.986	0.981
2770	0.982	0.981	0.987	0.981
2780	0.984	0.983	0.990	0.984
2790	0.980	0.980	0.988	0.980
2800	0.980	0.980	0.984	0.980
2810	0.980	0.980	0.987	0.980
2820	0.978	0.977	0.985	0.977
2830	0.981	0.980	0.987	0.981
2840	0.981	0.980	0.986	0.981
2850	0.985	0.985	0.990	0.985
2860	0.978	0.977	0.984	0.977
2870	0.980	0.979	0.986	0.980
2880	0.978	0.978	0.985	0.978
2890	0.982	0.982	0.988	0.982
2900	0.980	0.980	0.986	0.980
2910	0.978	0.977	0.984	0.977
2920	0.984	0.984	0.989	0.984
2930	0.979	0.978	0.985	0.979
2940	0.981	0.980	0.987	0.981
2950	0.981	0.980	0.987	0.981
2960	0.979	0.978	0.985	0.979

2970	0.982	0.982	0.986	0.982
2980	0.980	0.980	0.986	0.980
2990	0.979	0.978	0.983	0.979
3000	0.981	0.980	0.985	0.981
3010	0.981	0.980	0.988	0.981
3020	0.978	0.977	0.985	0.977
3030	0.979	0.978	0.985	0.978
3040	0.980	0.979	0.985	0.979
3050	0.978	0.978	0.986	0.978
3060	0.983	0.983	0.987	0.983
3070	0.980	0.980	0.984	0.980
3080	0.982	0.982	0.986	0.982
3090	0.978	0.978	0.986	0.978
3100	0.982	0.982	0.987	0.982
3110	0.982	0.981	0.987	0.982
3120	0.980	0.979	0.986	0.979
3130	0.982	0.982	0.986	0.982
3140	0.981	0.980	0.987	0.981
3150	0.980	0.980	0.986	0.980
3160	0.978	0.977	0.984	0.977
3170	0.980	0.980	0.986	0.980
3180	0.983	0.983	0.987	0.983
3190	0.982	0.981	0.987	0.981
3200	0.980	0.980	0.986	0.980
3210	0.980	0.979	0.986	0.979
3220	0.982	0.981	0.986	0.982
3230	0.982	0.981	0.986	0.981
3240	0.981	0.980	0.986	0.981
3250	0.982	0.982	0.987	0.982
3260	0.980	0.980	0.987	0.980
3270	0.981	0.980	0.985	0.981
3280	0.984	0.984	0.989	0.984
3290	0.983	0.983	0.988	0.983
3300	0.982	0.981	0.987	0.982
3310	0.982	0.982	0.987	0.982
3320	0.981	0.980	0.986	0.981
3330	0.981	0.980	0.987	0.981
3340	0.982	0.982	0.988	0.982
3350	0.980	0.980	0.986	0.980
3360	0.980	0.980	0.985	0.980
3370	0.978	0.978	0.985	0.978
3380	0.982	0.981	0.988	0.981
3390	0.979	0.978	0.983	0.979

3400	0.981	0.980	0.986	0.981
3410	0.982	0.981	0.987	0.982
3420	0.980	0.979	0.986	0.979
3430	0.982	0.982	0.988	0.982
3440	0.985	0.985	0.989	0.985
3450	0.980	0.980	0.986	0.980
3460	0.981	0.980	0.989	0.981
3470	0.980	0.979	0.986	0.980
3480	0.981	0.980	0.987	0.981
3490	0.981	0.980	0.986	0.981
3500	0.981	0.980	0.985	0.981
3510	0.982	0.982	0.988	0.982
3520	0.980	0.979	0.985	0.979
3530	0.982	0.981	0.986	0.982
3540	0.980	0.980	0.984	0.980
3550	0.982	0.982	0.988	0.982
3560	0.980	0.980	0.986	0.980
3570	0.981	0.980	0.986	0.981
3580	0.979	0.978	0.984	0.979
3590	0.981	0.980	0.988	0.981
3600	0.980	0.979	0.986	0.979
3610	0.982	0.982	0.988	0.982
3620	0.983	0.983	0.988	0.983
3630	0.982	0.982	0.987	0.982
3640	0.983	0.983	0.988	0.983
3650	0.982	0.981	0.987	0.982
3660	0.985	0.985	0.987	0.985
3670	0.983	0.983	0.988	0.983
3680	0.982	0.982	0.986	0.982
3690	0.981	0.980	0.987	0.981
3700	0.980	0.980	0.987	0.980
3710	0.983	0.983	0.989	0.983
3720	0.980	0.979	0.986	0.979
3730	0.980	0.979	0.985	0.979
3740	0.983	0.983	0.988	0.983
3750	0.984	0.983	0.988	0.984
3760	0.982	0.981	0.988	0.981
3770	0.981	0.980	0.986	0.981
3780	0.980	0.979	0.985	0.979
3790	0.984	0.983	0.989	0.984
3800	0.982	0.982	0.987	0.982
3810	0.982	0.982	0.988	0.982
3820	0.982	0.982	0.988	0.982

3830	0.981	0.980	0.988	0.981
3840	0.981	0.980	0.986	0.981
3850	0.984	0.984	0.989	0.984
3860	0.982	0.981	0.986	0.981
3870	0.985	0.985	0.990	0.985
3880	0.982	0.982	0.989	0.982
3890	0.982	0.981	0.987	0.982
3900	0.980	0.980	0.986	0.980
3910	0.981	0.980	0.987	0.981
3920	0.982	0.982	0.987	0.982
3930	0.985	0.985	0.989	0.985
3940	0.981	0.980	0.987	0.981
3950	0.984	0.983	0.988	0.984
3960	0.981	0.980	0.988	0.981
3970	0.983	0.983	0.988	0.983
3980	0.980	0.979	0.984	0.979
3990	0.984	0.983	0.988	0.984
4000	0.979	0.978	0.986	0.978
4010	0.984	0.984	0.989	0.984
4020	0.982	0.982	0.988	0.982
4030	0.980	0.979	0.985	0.980
4040	0.982	0.982	0.986	0.982
4050	0.981	0.980	0.986	0.981
4060	0.982	0.982	0.987	0.982
4070	0.978	0.977	0.984	0.977
4080	0.985	0.985	0.989	0.985
4090	0.983	0.983	0.988	0.983
4100	0.982	0.982	0.988	0.982
4110	0.983	0.983	0.987	0.983
4120	0.982	0.982	0.986	0.982
4130	0.980	0.980	0.986	0.980
4140	0.980	0.980	0.985	0.980
4150	0.982	0.982	0.988	0.982
4160	0.982	0.981	0.987	0.982
4170	0.981	0.980	0.987	0.981
4180	0.980	0.979	0.986	0.979
4190	0.981	0.980	0.986	0.981
4200	0.984	0.984	0.988	0.984
4210	0.980	0.980	0.984	0.980
4220	0.980	0.980	0.986	0.980
4230	0.982	0.981	0.987	0.982
4240	0.983	0.983	0.988	0.983
4250	0.984	0.984	0.988	0.984

4260	0.982	0.982	0.988	0.982
4270	0.981	0.980	0.985	0.981
4280	0.981	0.980	0.986	0.981
4290	0.982	0.982	0.986	0.982
4300	0.982	0.981	0.986	0.982
4310	0.985	0.985	0.989	0.985
4320	0.983	0.983	0.986	0.983
4330	0.980	0.979	0.986	0.979
4340	0.985	0.985	0.989	0.985
4350	0.981	0.980	0.987	0.981
4360	0.982	0.982	0.988	0.982
4370	0.982	0.982	0.988	0.982
4380	0.980	0.980	0.986	0.980
4390	0.983	0.983	0.988	0.983
4400	0.984	0.983	0.988	0.984
4410	0.982	0.982	0.987	0.982
4420	0.980	0.980	0.986	0.980
4430	0.984	0.983	0.988	0.983
4440	0.983	0.983	0.987	0.983
4450	0.984	0.983	0.989	0.984
4460	0.982	0.982	0.987	0.982
4470	0.981	0.980	0.987	0.981
4480	0.980	0.980	0.986	0.980
4490	0.982	0.981	0.985	0.982
4500	0.982	0.981	0.986	0.982
4510	0.980	0.980	0.984	0.980
4520	0.987	0.987	0.992	0.987
4530	0.982	0.982	0.986	0.982
4540	0.982	0.981	0.986	0.981
4550	0.984	0.983	0.988	0.984
4560	0.984	0.984	0.990	0.984
4570	0.980	0.979	0.985	0.979
4580	0.983	0.983	0.989	0.983
4590	0.978	0.978	0.983	0.978
4600	0.981	0.980	0.986	0.981
4610	0.984	0.983	0.988	0.984
4620	0.982	0.981	0.987	0.982
4630	0.982	0.982	0.986	0.982
4640	0.982	0.982	0.987	0.982
4650	0.982	0.982	0.987	0.982
4660	0.979	0.978	0.985	0.979
4670	0.985	0.985	0.989	0.985
4680	0.982	0.981	0.987	0.981

4690	0.979	0.978	0.984	0.979
4700	0.982	0.982	0.987	0.982
4710	0.983	0.983	0.989	0.983
4720	0.983	0.983	0.988	0.983
4730	0.979	0.978	0.985	0.979
4740	0.982	0.982	0.986	0.982
4750	0.983	0.983	0.988	0.983
4760	0.982	0.982	0.987	0.982
4770	0.984	0.984	0.988	0.984
4780	0.983	0.983	0.987	0.983
4790	0.984	0.984	0.989	0.984
4800	0.980	0.979	0.985	0.979
4810	0.983	0.983	0.988	0.983
4820	0.980	0.980	0.985	0.980
4830	0.980	0.979	0.984	0.979
4840	0.980	0.980	0.987	0.980
4850	0.984	0.983	0.987	0.984
4860	0.983	0.983	0.988	0.983
4870	0.980	0.979	0.984	0.980
4880	0.978	0.978	0.984	0.978
4890	0.985	0.985	0.990	0.985
4900	0.980	0.980	0.986	0.980
4910	0.982	0.982	0.988	0.982
4920	0.983	0.983	0.988	0.983
4930	0.982	0.981	0.988	0.981
4940	0.980	0.980	0.986	0.980
4950	0.986	0.985	0.990	0.986
4960	0.982	0.981	0.987	0.981
4970	0.981	0.980	0.987	0.981
4980	0.982	0.981	0.986	0.982
4990	0.981	0.980	0.986	0.981
5000	0.980	0.980	0.985	0.980
5010	0.984	0.983	0.988	0.984
5020	0.982	0.982	0.988	0.982
5030	0.984	0.984	0.989	0.984
5040	0.980	0.980	0.984	0.980
5050	0.980	0.979	0.985	0.979
5060	0.981	0.980	0.985	0.981
5070	0.980	0.980	0.987	0.980
5080	0.982	0.982	0.988	0.982
5090	0.981	0.980	0.985	0.981
5100	0.984	0.984	0.989	0.984
5110	0.981	0.980	0.986	0.981

5120	0.983	0.983	0.988	0.983
5130	0.982	0.981	0.987	0.982
5140	0.981	0.980	0.987	0.981
5150	0.980	0.980	0.986	0.980
5160	0.985	0.985	0.990	0.985
5170	0.981	0.980	0.987	0.981
5180	0.982	0.982	0.987	0.982
5190	0.981	0.980	0.986	0.981
5200	0.984	0.984	0.989	0.984
5210	0.984	0.983	0.987	0.984
5220	0.982	0.981	0.987	0.982
5230	0.980	0.980	0.987	0.980
5240	0.983	0.983	0.987	0.983
5250	0.982	0.982	0.988	0.982
5260	0.982	0.982	0.986	0.982
5270	0.982	0.982	0.988	0.982
5280	0.982	0.981	0.987	0.982
5290	0.982	0.981	0.987	0.982
5300	0.982	0.981	0.987	0.982
5310	0.984	0.984	0.989	0.984
5320	0.982	0.981	0.987	0.981
5330	0.982	0.981	0.985	0.981
5340	0.982	0.981	0.987	0.982
5350	0.986	0.985	0.990	0.986
5360	0.981	0.980	0.985	0.981
5370	0.982	0.981	0.985	0.982
5380	0.982	0.981	0.986	0.982
5390	0.983	0.983	0.988	0.983
5400	0.984	0.984	0.989	0.984
5410	0.982	0.982	0.987	0.982
5420	0.982	0.982	0.987	0.982
5430	0.978	0.978	0.983	0.978
5440	0.983	0.983	0.987	0.983
5450	0.981	0.980	0.987	0.981
5460	0.979	0.978	0.985	0.979
5470	0.980	0.980	0.985	0.980
5480	0.978	0.977	0.983	0.977
5490	0.982	0.982	0.986	0.982
5500	0.983	0.983	0.987	0.983
5510	0.983	0.983	0.988	0.983
5520	0.983	0.983	0.988	0.983
5530	0.982	0.982	0.987	0.982
5540	0.982	0.981	0.987	0.982

5550	0.982	0.982	0.987	0.982
5560	0.982	0.981	0.986	0.982
5570	0.982	0.982	0.986	0.982
5580	0.983	0.983	0.987	0.983
5590	0.979	0.978	0.986	0.979
5600	0.979	0.978	0.984	0.979
5610	0.983	0.983	0.989	0.983
5620	0.983	0.983	0.988	0.983
5630	0.982	0.981	0.987	0.982
5640	0.978	0.978	0.984	0.978
5650	0.981	0.980	0.986	0.981
5660	0.982	0.982	0.987	0.982
5670	0.979	0.978	0.985	0.979
5680	0.983	0.983	0.988	0.983
5690	0.982	0.982	0.987	0.982
5700	0.982	0.981	0.988	0.982
5710	0.980	0.980	0.985	0.980
5720	0.979	0.978	0.985	0.979
5730	0.984	0.984	0.989	0.984
5740	0.983	0.983	0.988	0.983
5750	0.985	0.985	0.989	0.985
5760	0.980	0.980	0.986	0.980
5770	0.983	0.983	0.988	0.983
5780	0.983	0.983	0.988	0.983
5790	0.981	0.980	0.986	0.981
5800	0.982	0.982	0.988	0.982
5810	0.982	0.982	0.987	0.982
5820	0.984	0.983	0.988	0.984
5830	0.984	0.983	0.987	0.984
5840	0.984	0.983	0.988	0.984
5850	0.979	0.978	0.985	0.979
5860	0.984	0.984	0.989	0.984
5870	0.983	0.983	0.988	0.983
5880	0.978	0.978	0.983	0.978
5890	0.983	0.983	0.987	0.983
5900	0.979	0.978	0.985	0.979
5910	0.981	0.980	0.985	0.981
5920	0.983	0.983	0.986	0.983
5930	0.980	0.980	0.985	0.980
5940	0.982	0.981	0.986	0.981
5950	0.980	0.980	0.985	0.980
5960	0.980	0.980	0.986	0.980
5970	0.983	0.983	0.988	0.983

5980	0.980	0.980	0.986	0.980
5990	0.984	0.983	0.988	0.983
6000	0.982	0.982	0.986	0.982
6010	0.984	0.984	0.989	0.984
6020	0.984	0.984	0.989	0.984
6030	0.983	0.983	0.988	0.983
6040	0.980	0.980	0.986	0.980
6050	0.979	0.978	0.985	0.979
6060	0.982	0.982	0.986	0.982
6070	0.980	0.979	0.986	0.979
6080	0.980	0.980	0.985	0.980
6090	0.982	0.982	0.988	0.982
6100	0.985	0.985	0.990	0.985
6110	0.982	0.982	0.987	0.982
6120	0.980	0.980	0.985	0.980
6130	0.982	0.981	0.986	0.982
6140	0.981	0.980	0.986	0.981
6150	0.981	0.980	0.985	0.981
6160	0.982	0.982	0.987	0.982
6170	0.981	0.980	0.985	0.981
6180	0.981	0.980	0.987	0.981
6190	0.984	0.983	0.988	0.984
6200	0.981	0.980	0.987	0.981
6210	0.982	0.982	0.986	0.982
6220	0.980	0.979	0.984	0.980
6230	0.984	0.983	0.988	0.984
6240	0.982	0.982	0.987	0.982
6250	0.980	0.980	0.985	0.980
6260	0.980	0.980	0.984	0.980
6270	0.980	0.980	0.984	0.980
6280	0.980	0.979	0.985	0.980
6290	0.982	0.981	0.987	0.982
6300	0.984	0.984	0.989	0.984
6310	0.982	0.981	0.987	0.981
6320	0.980	0.980	0.985	0.980
6330	0.984	0.983	0.987	0.984
6340	0.982	0.982	0.988	0.982
6350	0.982	0.982	0.987	0.982
6360	0.981	0.980	0.986	0.981
6370	0.982	0.981	0.986	0.981
6380	0.980	0.980	0.986	0.980
6390	0.982	0.981	0.986	0.982
6400	0.984	0.983	0.989	0.984

6410	0.982	0.982	0.986	0.982
6420	0.981	0.980	0.986	0.981
6430	0.980	0.980	0.987	0.980
6440	0.982	0.981	0.987	0.982
6450	0.982	0.981	0.987	0.981
6460	0.980	0.979	0.985	0.980
6470	0.980	0.979	0.985	0.980
6480	0.982	0.982	0.989	0.982
6490	0.984	0.983	0.988	0.984
6500	0.983	0.983	0.987	0.983
6510	0.982	0.982	0.988	0.982
6520	0.978	0.977	0.984	0.977
6530	0.980	0.979	0.985	0.979
6540	0.985	0.985	0.989	0.985
6550	0.984	0.983	0.988	0.984
6560	0.984	0.984	0.989	0.984
6570	0.982	0.981	0.987	0.982
6580	0.982	0.982	0.988	0.982
6590	0.980	0.980	0.986	0.980
6600	0.984	0.983	0.988	0.984
6610	0.982	0.982	0.988	0.982
6620	0.982	0.982	0.988	0.982
6630	0.982	0.981	0.987	0.982
6640	0.985	0.985	0.990	0.985
6650	0.981	0.980	0.985	0.981
6660	0.981	0.980	0.986	0.981
6670	0.982	0.981	0.987	0.982
6680	0.982	0.982	0.987	0.982
6690	0.985	0.985	0.989	0.985
6700	0.985	0.985	0.989	0.985
6710	0.984	0.983	0.988	0.984
6720	0.982	0.982	0.987	0.982
6730	0.982	0.982	0.987	0.982
6740	0.982	0.981	0.985	0.982
6750	0.984	0.983	0.988	0.984
6760	0.983	0.983	0.986	0.983
6770	0.984	0.984	0.989	0.984
6780	0.984	0.984	0.988	0.984
6790	0.983	0.983	0.988	0.983
6800	0.982	0.981	0.987	0.981
6810	0.983	0.983	0.987	0.983
6820	0.980	0.980	0.985	0.980
6830	0.983	0.983	0.988	0.983

6840	0.983	0.983	0.987	0.983
6850	0.982	0.982	0.988	0.982
6860	0.984	0.983	0.988	0.984
6870	0.982	0.982	0.987	0.982
6880	0.982	0.981	0.987	0.982
6890	0.980	0.979	0.986	0.980
6900	0.984	0.983	0.988	0.984
6910	0.984	0.983	0.989	0.984
6920	0.982	0.981	0.988	0.982
6930	0.982	0.981	0.987	0.982
6940	0.983	0.983	0.987	0.983
6950	0.983	0.983	0.988	0.983
6960	0.981	0.980	0.987	0.981
6970	0.982	0.981	0.986	0.982
6980	0.979	0.978	0.985	0.979
6990	0.982	0.982	0.987	0.982
7000	0.983	0.983	0.988	0.983
7010	0.984	0.983	0.987	0.984
7020	0.984	0.983	0.989	0.983
7030	0.982	0.981	0.985	0.982
7040	0.983	0.983	0.988	0.983
7050	0.983	0.983	0.988	0.983
7060	0.984	0.983	0.988	0.984
7070	0.983	0.983	0.988	0.983
7080	0.982	0.982	0.987	0.982
7090	0.982	0.982	0.986	0.982
7100	0.979	0.978	0.985	0.979
7110	0.982	0.981	0.988	0.982
7120	0.982	0.982	0.988	0.982
7130	0.982	0.981	0.986	0.982
7140	0.982	0.982	0.987	0.982
7150	0.983	0.983	0.987	0.983
7160	0.983	0.983	0.987	0.983
7170	0.981	0.980	0.986	0.981
7180	0.983	0.983	0.986	0.983
7190	0.980	0.979	0.986	0.979
7200	0.984	0.983	0.989	0.984
7210	0.982	0.981	0.987	0.982
7220	0.982	0.981	0.986	0.982
7230	0.982	0.981	0.986	0.981
7240	0.983	0.983	0.989	0.983
7250	0.984	0.983	0.988	0.984
7260	0.981	0.980	0.985	0.981

7270	0.979	0.978	0.983	0.979
7280	0.982	0.982	0.988	0.982
7290	0.983	0.983	0.987	0.983
7300	0.980	0.980	0.985	0.980
7310	0.984	0.984	0.988	0.984
7320	0.984	0.983	0.988	0.984
7330	0.982	0.982	0.988	0.982
7340	0.984	0.983	0.988	0.984
7350	0.981	0.980	0.987	0.981
7360	0.980	0.980	0.986	0.980
7370	0.984	0.984	0.988	0.984
7380	0.985	0.985	0.989	0.985
7390	0.978	0.977	0.984	0.977
7400	0.982	0.981	0.986	0.982
7410	0.982	0.982	0.987	0.982
7420	0.983	0.983	0.987	0.983
7430	0.981	0.980	0.984	0.981
7440	0.984	0.983	0.987	0.984
7450	0.980	0.979	0.985	0.980
7460	0.982	0.982	0.986	0.982
7470	0.982	0.981	0.986	0.982
7480	0.982	0.981	0.987	0.982
7490	0.984	0.984	0.988	0.984
7500	0.982	0.982	0.987	0.982
7510	0.982	0.982	0.987	0.982
7520	0.982	0.981	0.988	0.981
7530	0.981	0.980	0.987	0.981
7540	0.981	0.980	0.986	0.981
7550	0.982	0.982	0.988	0.982
7560	0.981	0.980	0.986	0.981
7570	0.982	0.982	0.987	0.982
7580	0.983	0.983	0.987	0.983
7590	0.984	0.984	0.988	0.984
7600	0.982	0.982	0.988	0.982
7610	0.985	0.985	0.989	0.985
7620	0.981	0.980	0.987	0.981
7630	0.986	0.985	0.989	0.986
7640	0.982	0.981	0.987	0.982
7650	0.980	0.979	0.987	0.979
7660	0.981	0.980	0.987	0.981
7670	0.982	0.982	0.988	0.982
7680	0.982	0.981	0.987	0.982
7690	0.983	0.983	0.987	0.983

7700	0.981	0.980	0.986	0.981
7710	0.983	0.983	0.988	0.983
7720	0.984	0.983	0.989	0.984
7730	0.978	0.978	0.984	0.978
7740	0.982	0.982	0.988	0.982
7750	0.984	0.984	0.989	0.984
7760	0.979	0.978	0.985	0.979
7770	0.981	0.980	0.986	0.981
7780	0.981	0.980	0.985	0.981
7790	0.983	0.983	0.988	0.983
7800	0.983	0.983	0.987	0.983
7810	0.983	0.983	0.988	0.983
7820	0.982	0.981	0.987	0.982
7830	0.983	0.983	0.988	0.983
7840	0.984	0.983	0.987	0.984
7850	0.982	0.981	0.987	0.982
7860	0.983	0.983	0.989	0.983
7870	0.984	0.983	0.988	0.984
7880	0.982	0.982	0.987	0.982
7890	0.981	0.980	0.986	0.981
7900	0.984	0.984	0.989	0.984
7910	0.982	0.982	0.988	0.982
7920	0.981	0.980	0.987	0.981
7930	0.981	0.980	0.986	0.981
7940	0.980	0.980	0.987	0.980
7950	0.982	0.982	0.987	0.982
7960	0.981	0.980	0.985	0.981
7970	0.981	0.980	0.987	0.981
7980	0.980	0.980	0.985	0.980
7990	0.980	0.980	0.987	0.980
8000	0.980	0.980	0.984	0.980
8010	0.983	0.983	0.987	0.983
8020	0.981	0.980	0.986	0.981
8030	0.980	0.980	0.985	0.980
8040	0.982	0.981	0.985	0.982
8050	0.984	0.984	0.988	0.984
8060	0.983	0.983	0.988	0.983
8070	0.982	0.982	0.987	0.982
8080	0.980	0.980	0.986	0.980
8090	0.982	0.982	0.987	0.982
8100	0.984	0.984	0.989	0.984
8110	0.982	0.982	0.987	0.982
8120	0.982	0.982	0.986	0.982

8130	0.982	0.981	0.986	0.982
8140	0.986	0.985	0.989	0.986
8150	0.982	0.982	0.987	0.982
8160	0.982	0.982	0.988	0.982
8170	0.980	0.980	0.985	0.980
8180	0.982	0.982	0.988	0.982
8190	0.983	0.983	0.986	0.983
8200	0.982	0.982	0.988	0.982
8210	0.980	0.980	0.985	0.980
8220	0.982	0.981	0.987	0.982
8230	0.978	0.978	0.984	0.978
8240	0.981	0.980	0.987	0.981
8250	0.983	0.983	0.987	0.983
8260	0.981	0.980	0.985	0.981
8270	0.982	0.981	0.986	0.982
8280	0.982	0.981	0.988	0.982
8290	0.982	0.982	0.986	0.982
8300	0.983	0.983	0.988	0.983
8310	0.978	0.977	0.984	0.977
8320	0.981	0.980	0.986	0.981
8330	0.986	0.985	0.990	0.986
8340	0.984	0.984	0.989	0.984
8350	0.985	0.985	0.989	0.985
8360	0.982	0.981	0.986	0.982
8370	0.982	0.982	0.987	0.982
8380	0.981	0.980	0.985	0.981
8390	0.981	0.980	0.985	0.981
8400	0.982	0.981	0.985	0.982
8410	0.982	0.981	0.986	0.982
8420	0.980	0.979	0.984	0.980
8430	0.982	0.982	0.987	0.982
8440	0.982	0.981	0.987	0.982
8450	0.980	0.980	0.986	0.980
8460	0.980	0.980	0.986	0.980
8470	0.984	0.983	0.986	0.984
8480	0.984	0.983	0.987	0.984
8490	0.982	0.981	0.986	0.982
8500	0.981	0.980	0.986	0.981
8510	0.982	0.982	0.988	0.982
8520	0.984	0.983	0.988	0.984
8530	0.978	0.978	0.984	0.978
8540	0.983	0.983	0.987	0.983
8550	0.980	0.979	0.985	0.980

8560	0.985	0.985	0.989	0.985
8570	0.986	0.985	0.989	0.986
8580	0.981	0.980	0.984	0.981
8590	0.984	0.984	0.988	0.984
8600	0.986	0.985	0.990	0.986
8610	0.985	0.985	0.989	0.985
8620	0.982	0.982	0.986	0.982
8630	0.982	0.981	0.984	0.982
8640	0.982	0.982	0.987	0.982
8650	0.982	0.982	0.987	0.982
8660	0.981	0.980	0.987	0.981
8670	0.982	0.981	0.985	0.982
8680	0.982	0.981	0.987	0.981
8690	0.979	0.978	0.983	0.979
8700	0.981	0.980	0.987	0.981
8710	0.980	0.980	0.986	0.980
8720	0.985	0.985	0.990	0.985
8730	0.980	0.980	0.987	0.980
8740	0.983	0.983	0.988	0.983
8750	0.980	0.980	0.984	0.980
8760	0.986	0.985	0.989	0.986
8770	0.981	0.980	0.986	0.981
8780	0.982	0.982	0.987	0.982
8790	0.986	0.985	0.989	0.986
8800	0.980	0.979	0.984	0.980
8810	0.982	0.982	0.988	0.982
8820	0.983	0.983	0.988	0.983
8830	0.983	0.983	0.986	0.983
8840	0.982	0.982	0.986	0.982
8850	0.987	0.987	0.990	0.987
8860	0.984	0.984	0.989	0.984
8870	0.980	0.980	0.983	0.980
8880	0.982	0.982	0.987	0.982
8890	0.984	0.984	0.988	0.984
8900	0.986	0.986	0.990	0.986
8910	0.983	0.983	0.988	0.983
8920	0.986	0.986	0.990	0.986
8930	0.982	0.982	0.987	0.982
8940	0.982	0.981	0.987	0.981
8950	0.984	0.983	0.988	0.984

(2) IFS results with DT on the LASSO feature list

Number of features	ACC	MCC	Macro F1	Weighted F1

10	0.606	0.596	0.651	0.603
20	0.705	0.697	0.709	0.700
30	0.743	0.737	0.747	0.743
40	0.747	0.740	0.752	0.748
50	0.735	0.728	0.738	0.734
60	0.747	0.740	0.755	0.744
70	0.769	0.763	0.765	0.771
80	0.755	0.748	0.749	0.756
90	0.764	0.758	0.767	0.763
100	0.767	0.761	0.757	0.767
110	0.772	0.766	0.775	0.774
120	0.756	0.749	0.756	0.756
130	0.771	0.765	0.772	0.770
140	0.762	0.756	0.763	0.763
150	0.766	0.759	0.758	0.766
160	0.788	0.782	0.789	0.790
170	0.762	0.756	0.768	0.763
180	0.763	0.757	0.756	0.764
190	0.788	0.782	0.793	0.788
200	0.776	0.770	0.782	0.776
210	0.777	0.771	0.784	0.774
220	0.778	0.772	0.780	0.779
230	0.785	0.780	0.788	0.784
240	0.778	0.772	0.784	0.776
250	0.783	0.778	0.782	0.784
260	0.790	0.784	0.788	0.790
270	0.800	0.794	0.795	0.799
280	0.792	0.787	0.788	0.792
290	0.806	0.801	0.799	0.806
300	0.793	0.787	0.788	0.793
310	0.778	0.772	0.778	0.778
320	0.800	0.794	0.798	0.800
330	0.794	0.788	0.789	0.795
340	0.804	0.799	0.797	0.805
350	0.830	0.826	0.829	0.830
360	0.798	0.793	0.793	0.799
370	0.809	0.803	0.802	0.809
380	0.802	0.797	0.806	0.801
390	0.810	0.805	0.803	0.811
400	0.813	0.808	0.809	0.814
410	0.795	0.790	0.790	0.794
420	0.812	0.807	0.801	0.813
430	0.810	0.805	0.802	0.811

440	0.810	0.805	0.808	0.811
450	0.818	0.813	0.815	0.817
460	0.815	0.810	0.808	0.815
470	0.802	0.796	0.797	0.804
480	0.815	0.811	0.805	0.815
490	0.794	0.788	0.787	0.793
500	0.802	0.796	0.785	0.803
510	0.807	0.802	0.804	0.807
520	0.815	0.810	0.808	0.815
530	0.821	0.816	0.815	0.822
540	0.785	0.779	0.772	0.785
550	0.802	0.797	0.792	0.804
560	0.816	0.811	0.807	0.816
570	0.804	0.799	0.801	0.803
580	0.809	0.803	0.805	0.810
590	0.806	0.801	0.799	0.805
600	0.823	0.818	0.818	0.825
610	0.819	0.815	0.811	0.819
620	0.832	0.827	0.832	0.831
630	0.802	0.797	0.796	0.802
640	0.804	0.798	0.802	0.804
650	0.817	0.812	0.816	0.817
660	0.807	0.802	0.801	0.807
670	0.809	0.803	0.812	0.809
680	0.811	0.805	0.807	0.810
690	0.814	0.809	0.801	0.815
700	0.804	0.799	0.796	0.804
710	0.807	0.802	0.797	0.808
720	0.805	0.800	0.799	0.806
730	0.809	0.804	0.810	0.808
740	0.802	0.796	0.807	0.802
750	0.811	0.805	0.813	0.811
760	0.815	0.810	0.817	0.814
770	0.817	0.812	0.815	0.816
780	0.821	0.816	0.822	0.821
790	0.808	0.803	0.809	0.809
800	0.823	0.818	0.829	0.822
810	0.821	0.817	0.823	0.823
820	0.813	0.808	0.812	0.812
830	0.798	0.792	0.802	0.799
840	0.813	0.808	0.809	0.812
850	0.819	0.814	0.818	0.820
860	0.821	0.817	0.820	0.822

870	0.820	0.815	0.818	0.821
880	0.816	0.811	0.813	0.815
890	0.817	0.812	0.814	0.818
900	0.828	0.823	0.835	0.828
910	0.822	0.817	0.820	0.822
920	0.822	0.817	0.824	0.822
930	0.811	0.805	0.814	0.812
940	0.817	0.812	0.810	0.817
950	0.807	0.801	0.800	0.807
960	0.827	0.822	0.828	0.825
970	0.829	0.824	0.823	0.829
980	0.813	0.808	0.807	0.814
990	0.819	0.815	0.820	0.818
1000	0.817	0.812	0.814	0.817
1010	0.805	0.800	0.800	0.807
1020	0.798	0.792	0.806	0.796
1030	0.815	0.810	0.817	0.816
1040	0.828	0.824	0.831	0.828
1050	0.822	0.817	0.825	0.821
1060	0.815	0.810	0.810	0.814
1070	0.818	0.813	0.817	0.819
1080	0.807	0.802	0.805	0.808
1090	0.836	0.832	0.835	0.834
1100	0.816	0.811	0.819	0.818
1110	0.835	0.831	0.831	0.835
1120	0.832	0.827	0.826	0.834
1130	0.830	0.826	0.823	0.833
1140	0.828	0.823	0.825	0.828
1150	0.826	0.821	0.820	0.824
1160	0.826	0.821	0.827	0.827
1170	0.814	0.809	0.803	0.813
1180	0.819	0.815	0.813	0.822
1190	0.826	0.821	0.837	0.826
1200	0.819	0.814	0.824	0.819
1210	0.827	0.822	0.818	0.826
1220	0.813	0.808	0.807	0.814
1230	0.823	0.818	0.826	0.822
1240	0.825	0.820	0.827	0.826
1250	0.830	0.826	0.827	0.830
1260	0.822	0.817	0.821	0.823
1270	0.822	0.817	0.822	0.822
1280	0.834	0.829	0.834	0.833
1290	0.813	0.808	0.797	0.815

1300	0.822	0.817	0.814	0.822
1310	0.830	0.826	0.826	0.833
1320	0.809	0.804	0.806	0.810
1330	0.821	0.817	0.824	0.821
1340	0.824	0.819	0.817	0.824
1350	0.822	0.817	0.829	0.823
1360	0.834	0.829	0.827	0.835
1370	0.816	0.811	0.812	0.817
1380	0.813	0.808	0.816	0.814
1390	0.825	0.820	0.829	0.823
1400	0.829	0.824	0.822	0.829
1410	0.802	0.797	0.788	0.803
1420	0.833	0.828	0.836	0.832
1430	0.825	0.820	0.828	0.824
1440	0.827	0.822	0.817	0.827
1450	0.834	0.829	0.829	0.833
1460	0.828	0.823	0.828	0.828
1470	0.827	0.822	0.823	0.826
1480	0.828	0.823	0.817	0.828
1490	0.841	0.837	0.836	0.842
1500	0.824	0.819	0.817	0.824
1510	0.823	0.818	0.816	0.823
1520	0.830	0.825	0.816	0.829
1530	0.840	0.836	0.837	0.841
1540	0.815	0.810	0.811	0.814
1550	0.824	0.819	0.821	0.826
1560	0.821	0.816	0.810	0.822
1570	0.830	0.825	0.820	0.829
1580	0.823	0.819	0.817	0.823
1590	0.838	0.833	0.830	0.837
1600	0.811	0.805	0.803	0.812
1610	0.833	0.828	0.825	0.834
1620	0.828	0.823	0.828	0.828
1630	0.843	0.839	0.843	0.843
1640	0.827	0.822	0.823	0.828
1650	0.832	0.828	0.834	0.832
1660	0.835	0.831	0.819	0.835
1670	0.822	0.817	0.814	0.822
1680	0.838	0.833	0.834	0.837
1690	0.830	0.826	0.822	0.832
1700	0.819	0.815	0.811	0.820
1710	0.829	0.824	0.822	0.829
1720	0.817	0.812	0.815	0.817

1730	0.835	0.831	0.828	0.836
1740	0.820	0.815	0.815	0.821
1750	0.832	0.827	0.835	0.833
1760	0.830	0.826	0.822	0.831
1770	0.849	0.845	0.845	0.849
1780	0.812	0.807	0.806	0.813
1790	0.834	0.829	0.827	0.834
1800	0.833	0.829	0.831	0.833
1810	0.826	0.822	0.825	0.827
1820	0.825	0.820	0.822	0.823
1830	0.823	0.819	0.815	0.825
1840	0.821	0.816	0.809	0.821
1850	0.814	0.809	0.815	0.811
1860	0.819	0.814	0.809	0.819
1870	0.817	0.812	0.807	0.818
1880	0.821	0.816	0.822	0.821
1890	0.816	0.811	0.812	0.815
1900	0.829	0.824	0.820	0.829
1910	0.819	0.815	0.813	0.818
1920	0.828	0.823	0.829	0.828
1930	0.821	0.817	0.821	0.821
1940	0.811	0.805	0.812	0.811
1950	0.836	0.832	0.827	0.836
1960	0.817	0.812	0.819	0.817
1970	0.819	0.815	0.812	0.821
1980	0.823	0.818	0.816	0.824
1990	0.822	0.817	0.833	0.822
2000	0.833	0.829	0.831	0.834
2010	0.838	0.834	0.832	0.838
2020	0.837	0.833	0.834	0.836
2030	0.828	0.823	0.824	0.827
2040	0.830	0.825	0.831	0.830
2050	0.826	0.822	0.821	0.827
2060	0.823	0.818	0.816	0.823
2070	0.832	0.827	0.825	0.831
2080	0.817	0.812	0.809	0.816
2090	0.834	0.830	0.825	0.834
2100	0.823	0.819	0.820	0.822
2110	0.826	0.821	0.826	0.827
2120	0.817	0.812	0.822	0.818
2130	0.813	0.808	0.807	0.813
2140	0.819	0.814	0.807	0.819
2150	0.840	0.836	0.827	0.839

2160	0.836	0.831	0.834	0.836
2170	0.832	0.827	0.833	0.830
2180	0.825	0.820	0.814	0.825
2190	0.815	0.810	0.803	0.816
2200	0.837	0.833	0.825	0.837
2210	0.826	0.822	0.824	0.826
2220	0.828	0.824	0.818	0.828
2230	0.811	0.805	0.798	0.811
2240	0.821	0.816	0.820	0.820
2250	0.820	0.815	0.817	0.820
2260	0.821	0.816	0.814	0.822
2270	0.813	0.808	0.809	0.813
2280	0.823	0.818	0.820	0.822
2290	0.844	0.840	0.838	0.843
2300	0.835	0.831	0.833	0.835
2310	0.834	0.829	0.836	0.833
2320	0.811	0.806	0.798	0.812
2330	0.823	0.818	0.822	0.823
2340	0.827	0.822	0.829	0.828
2350	0.823	0.819	0.812	0.824
2360	0.812	0.807	0.808	0.811
2370	0.822	0.817	0.821	0.823
2380	0.818	0.813	0.824	0.818
2390	0.815	0.810	0.814	0.814
2400	0.834	0.829	0.821	0.832
2410	0.826	0.822	0.828	0.825
2420	0.829	0.824	0.827	0.829
2430	0.840	0.835	0.826	0.840
2440	0.814	0.809	0.820	0.813
2450	0.807	0.801	0.807	0.806
2460	0.822	0.817	0.818	0.821
2470	0.823	0.818	0.811	0.822
2480	0.842	0.838	0.840	0.842
2490	0.836	0.831	0.839	0.836
2500	0.819	0.815	0.821	0.817
2510	0.823	0.819	0.819	0.821
2520	0.816	0.811	0.810	0.817
2530	0.832	0.827	0.826	0.832
2540	0.819	0.814	0.825	0.819
2550	0.812	0.807	0.805	0.810
2560	0.830	0.826	0.829	0.830
2570	0.827	0.822	0.811	0.826
2580	0.830	0.826	0.829	0.829

2590	0.819	0.814	0.811	0.818
2600	0.830	0.826	0.825	0.829
2610	0.828	0.824	0.825	0.828
2620	0.823	0.818	0.818	0.822
2630	0.824	0.819	0.822	0.825
2640	0.823	0.818	0.809	0.823
2650	0.824	0.819	0.813	0.825
2660	0.830	0.826	0.817	0.830
2670	0.829	0.824	0.827	0.829
2680	0.825	0.820	0.818	0.827
2690	0.828	0.823	0.827	0.828
2700	0.828	0.824	0.834	0.829
2710	0.832	0.828	0.828	0.833
2720	0.825	0.820	0.823	0.825
2730	0.829	0.824	0.828	0.829
2740	0.843	0.839	0.838	0.842
2750	0.827	0.822	0.822	0.828
2760	0.813	0.808	0.808	0.815
2770	0.821	0.816	0.813	0.820
2780	0.817	0.812	0.820	0.818
2790	0.828	0.823	0.822	0.828
2800	0.818	0.813	0.817	0.818
2810	0.822	0.817	0.825	0.822
2820	0.834	0.829	0.825	0.834
2830	0.838	0.834	0.825	0.838
2840	0.833	0.828	0.833	0.832
2850	0.831	0.826	0.828	0.832
2860	0.826	0.821	0.825	0.827
2870	0.826	0.821	0.822	0.824
2880	0.828	0.823	0.831	0.827
2890	0.829	0.824	0.828	0.829
2900	0.831	0.826	0.823	0.831
2910	0.821	0.817	0.818	0.821
2920	0.840	0.835	0.838	0.840
2930	0.829	0.824	0.819	0.830
2940	0.821	0.817	0.828	0.819
2950	0.817	0.812	0.825	0.817
2960	0.825	0.820	0.831	0.825
2970	0.827	0.822	0.830	0.826
2980	0.840	0.835	0.845	0.840
2990	0.826	0.821	0.816	0.826
3000	0.823	0.819	0.807	0.824
3010	0.824	0.819	0.826	0.824

3020	0.838	0.833	0.834	0.837
3030	0.819	0.814	0.812	0.818
3040	0.820	0.815	0.816	0.819
3050	0.824	0.819	0.829	0.824
3060	0.826	0.821	0.821	0.826
3070	0.834	0.829	0.833	0.834
3080	0.831	0.826	0.841	0.830
3090	0.826	0.821	0.814	0.825
3100	0.812	0.807	0.812	0.812
3110	0.833	0.829	0.831	0.832
3120	0.836	0.832	0.830	0.834
3130	0.813	0.808	0.808	0.813
3140	0.840	0.836	0.838	0.841
3150	0.827	0.822	0.811	0.828
3160	0.838	0.834	0.840	0.839
3170	0.819	0.814	0.825	0.819
3180	0.822	0.817	0.820	0.823
3190	0.836	0.832	0.842	0.836
3200	0.825	0.820	0.820	0.825
3210	0.830	0.826	0.824	0.830
3220	0.833	0.829	0.818	0.833
3230	0.825	0.820	0.829	0.825
3240	0.840	0.836	0.841	0.840
3250	0.840	0.836	0.838	0.841
3260	0.831	0.826	0.829	0.832
3270	0.832	0.827	0.833	0.831
3280	0.816	0.811	0.817	0.815
3290	0.828	0.824	0.832	0.827
3300	0.820	0.815	0.823	0.819
3310	0.831	0.826	0.821	0.832
3320	0.838	0.834	0.832	0.840
3330	0.813	0.808	0.822	0.813
3340	0.826	0.821	0.813	0.827
3350	0.823	0.818	0.813	0.822
3360	0.819	0.814	0.823	0.820
3370	0.836	0.831	0.828	0.836
3380	0.838	0.833	0.829	0.839
3390	0.815	0.810	0.809	0.815
3400	0.836	0.831	0.835	0.837
3410	0.836	0.832	0.833	0.838
3420	0.842	0.838	0.840	0.841
3430	0.837	0.833	0.836	0.838
3440	0.834	0.829	0.835	0.835

3450	0.826	0.822	0.828	0.826
3460	0.819	0.814	0.814	0.819
3470	0.831	0.826	0.829	0.830
3480	0.813	0.808	0.809	0.814
3490	0.826	0.821	0.820	0.825
3500	0.824	0.819	0.815	0.823
3510	0.816	0.811	0.818	0.816
3520	0.817	0.812	0.813	0.817
3530	0.812	0.807	0.805	0.811
3540	0.821	0.816	0.825	0.821
3550	0.838	0.834	0.833	0.838
3560	0.835	0.830	0.831	0.834
3570	0.851	0.846	0.851	0.850
3580	0.809	0.803	0.809	0.808
3590	0.828	0.823	0.836	0.828
3600	0.834	0.829	0.839	0.834
3610	0.826	0.821	0.818	0.826
3620	0.831	0.826	0.829	0.829
3630	0.824	0.819	0.819	0.823
3640	0.819	0.814	0.816	0.819
3650	0.821	0.817	0.811	0.823
3660	0.835	0.831	0.839	0.835
3670	0.823	0.819	0.823	0.823
3680	0.828	0.823	0.823	0.827
3690	0.830	0.825	0.825	0.831
3700	0.838	0.834	0.834	0.838
3710	0.839	0.835	0.848	0.840
3720	0.822	0.817	0.819	0.823
3730	0.811	0.805	0.803	0.810
3740	0.840	0.836	0.834	0.841
3750	0.827	0.822	0.836	0.827
3760	0.838	0.833	0.830	0.838
3770	0.822	0.817	0.823	0.820
3780	0.831	0.826	0.825	0.829
3790	0.838	0.834	0.841	0.837
3800	0.828	0.823	0.827	0.828
3810	0.828	0.823	0.834	0.827
3820	0.824	0.819	0.826	0.822
3830	0.845	0.840	0.846	0.846
3840	0.815	0.810	0.800	0.818
3850	0.834	0.829	0.826	0.834
3860	0.811	0.805	0.815	0.811
3870	0.832	0.827	0.831	0.833

3880	0.836	0.831	0.829	0.835
3890	0.832	0.827	0.827	0.832
3900	0.844	0.840	0.836	0.843
3910	0.835	0.831	0.831	0.834
3920	0.831	0.827	0.831	0.830
3930	0.834	0.829	0.840	0.833
3940	0.826	0.821	0.826	0.827
3950	0.818	0.813	0.809	0.819
3960	0.825	0.820	0.815	0.824
3970	0.837	0.833	0.836	0.837
3980	0.835	0.830	0.830	0.835
3990	0.823	0.818	0.816	0.824
4000	0.834	0.830	0.833	0.836
4010	0.820	0.815	0.823	0.820
4020	0.836	0.832	0.825	0.838
4030	0.830	0.825	0.820	0.831
4040	0.817	0.813	0.814	0.818
4050	0.821	0.816	0.809	0.822
4060	0.832	0.828	0.832	0.832
4070	0.834	0.830	0.825	0.833
4080	0.829	0.824	0.814	0.829
4090	0.836	0.831	0.835	0.834
4100	0.818	0.813	0.805	0.818
4110	0.823	0.819	0.826	0.825
4120	0.832	0.828	0.825	0.832
4130	0.814	0.809	0.815	0.814
4140	0.827	0.822	0.816	0.828
4150	0.840	0.836	0.834	0.838
4160	0.830	0.825	0.829	0.829
4170	0.825	0.820	0.814	0.825
4180	0.813	0.808	0.805	0.813
4190	0.832	0.827	0.831	0.833
4200	0.822	0.817	0.810	0.823
4210	0.831	0.826	0.830	0.832
4220	0.838	0.833	0.827	0.837
4230	0.833	0.828	0.834	0.834
4240	0.813	0.808	0.806	0.813
4250	0.835	0.831	0.836	0.835
4260	0.823	0.819	0.821	0.824
4270	0.823	0.819	0.815	0.825
4280	0.839	0.835	0.839	0.838
4290	0.823	0.818	0.814	0.823
4300	0.831	0.826	0.833	0.831

4310	0.832	0.828	0.830	0.832
4320	0.826	0.822	0.818	0.826
4330	0.837	0.833	0.830	0.837
4340	0.840	0.835	0.836	0.839
4350	0.835	0.831	0.836	0.835
4360	0.835	0.831	0.830	0.835
4370	0.832	0.827	0.829	0.832
4380	0.827	0.822	0.828	0.828
4390	0.824	0.820	0.818	0.825
4400	0.849	0.844	0.843	0.850
4410	0.835	0.831	0.836	0.835
4420	0.828	0.823	0.819	0.829
4430	0.834	0.829	0.824	0.834
4440	0.820	0.815	0.812	0.819
4450	0.834	0.830	0.832	0.834
4460	0.842	0.838	0.840	0.842
4470	0.828	0.824	0.829	0.828
4480	0.840	0.836	0.831	0.841
4490	0.826	0.822	0.821	0.825
4500	0.836	0.831	0.832	0.837
4510	0.828	0.823	0.821	0.829
4520	0.823	0.819	0.824	0.824
4530	0.832	0.827	0.835	0.832
4540	0.828	0.824	0.833	0.830
4550	0.838	0.834	0.849	0.837
4560	0.847	0.842	0.838	0.846
4570	0.836	0.832	0.828	0.836
4580	0.842	0.838	0.841	0.842
4590	0.840	0.836	0.837	0.841
4600	0.828	0.823	0.826	0.829
4610	0.832	0.828	0.833	0.833
4620	0.831	0.826	0.817	0.831
4630	0.822	0.817	0.819	0.821
4640	0.825	0.820	0.815	0.827
4650	0.823	0.818	0.821	0.824
4660	0.834	0.829	0.821	0.835
4670	0.824	0.819	0.820	0.825
4680	0.819	0.814	0.822	0.819
4690	0.835	0.830	0.826	0.835
4700	0.833	0.829	0.823	0.834
4710	0.839	0.835	0.823	0.841
4720	0.822	0.817	0.819	0.823
4730	0.830	0.826	0.831	0.829

4740	0.846	0.842	0.848	0.845
4750	0.830	0.826	0.819	0.830
4760	0.826	0.821	0.819	0.827
4770	0.832	0.827	0.820	0.833
4780	0.830	0.825	0.818	0.830
4790	0.825	0.820	0.816	0.825
4800	0.840	0.835	0.829	0.839
4810	0.834	0.829	0.836	0.834
4820	0.839	0.835	0.841	0.841
4830	0.823	0.819	0.816	0.824
4840	0.823	0.819	0.805	0.823
4850	0.834	0.829	0.836	0.834
4860	0.807	0.802	0.802	0.808
4870	0.838	0.833	0.829	0.839
4880	0.828	0.824	0.829	0.828
4890	0.824	0.819	0.816	0.825
4900	0.848	0.844	0.839	0.848
4910	0.838	0.833	0.823	0.838
4920	0.832	0.827	0.828	0.831
4930	0.834	0.830	0.832	0.835
4940	0.830	0.825	0.819	0.831
4950	0.822	0.817	0.814	0.823
4960	0.830	0.825	0.823	0.830
4970	0.834	0.829	0.828	0.833
4980	0.826	0.821	0.822	0.827
4990	0.830	0.825	0.828	0.831
5000	0.833	0.828	0.822	0.832
5010	0.831	0.826	0.825	0.831
5020	0.849	0.844	0.835	0.849
5030	0.845	0.840	0.838	0.844
5040	0.829	0.824	0.817	0.830
5050	0.832	0.828	0.827	0.832
5060	0.838	0.833	0.832	0.837
5070	0.827	0.822	0.822	0.826
5080	0.836	0.832	0.841	0.836
5090	0.831	0.826	0.828	0.830
5100	0.836	0.832	0.829	0.836
5110	0.822	0.817	0.820	0.823
5120	0.853	0.849	0.852	0.852
5130	0.832	0.827	0.824	0.832
5140	0.850	0.846	0.841	0.851
5150	0.834	0.829	0.821	0.836
5160	0.823	0.819	0.813	0.824

5170	0.855	0.851	0.854	0.856
5180	0.829	0.824	0.832	0.830
5190	0.848	0.844	0.838	0.848
5200	0.853	0.849	0.843	0.853
5210	0.847	0.842	0.841	0.847
5220	0.834	0.830	0.831	0.834
5230	0.838	0.834	0.835	0.838
5240	0.826	0.821	0.824	0.824
5250	0.838	0.834	0.839	0.836
5260	0.823	0.818	0.811	0.825
5270	0.842	0.837	0.841	0.843
5280	0.830	0.825	0.821	0.830
5290	0.845	0.841	0.840	0.845
5300	0.833	0.828	0.838	0.834
5310	0.821	0.817	0.818	0.820
5320	0.838	0.834	0.832	0.840
5330	0.824	0.820	0.826	0.824
5340	0.846	0.842	0.842	0.846
5350	0.828	0.824	0.822	0.827
5360	0.845	0.841	0.842	0.845
5370	0.842	0.838	0.839	0.842
5380	0.837	0.833	0.830	0.836
5390	0.859	0.856	0.862	0.859
5400	0.838	0.833	0.835	0.838
5410	0.834	0.830	0.827	0.834
5420	0.844	0.840	0.844	0.843
5430	0.837	0.833	0.829	0.837
5440	0.833	0.828	0.826	0.833
5450	0.831	0.826	0.821	0.832
5460	0.819	0.815	0.810	0.819
5470	0.828	0.824	0.818	0.828
5480	0.834	0.829	0.825	0.835
5490	0.843	0.839	0.833	0.841
5500	0.828	0.824	0.826	0.830
5510	0.847	0.843	0.841	0.847
5520	0.836	0.832	0.826	0.838
5530	0.834	0.829	0.825	0.832
5540	0.839	0.835	0.832	0.839
5550	0.832	0.827	0.825	0.833
5560	0.849	0.845	0.848	0.850
5570	0.836	0.831	0.828	0.836
5580	0.840	0.836	0.834	0.841
5590	0.853	0.849	0.852	0.854

5600	0.833	0.828	0.824	0.835
5610	0.840	0.835	0.829	0.839
5620	0.835	0.831	0.825	0.837
5630	0.842	0.838	0.838	0.844
5640	0.827	0.822	0.822	0.827
5650	0.834	0.830	0.832	0.834
5660	0.847	0.843	0.847	0.849
5670	0.836	0.831	0.821	0.837
5680	0.832	0.828	0.829	0.833
5690	0.836	0.832	0.826	0.838
5700	0.830	0.825	0.833	0.830
5710	0.828	0.823	0.826	0.828
5720	0.835	0.830	0.832	0.835
5730	0.842	0.838	0.837	0.843
5740	0.848	0.844	0.838	0.847
5750	0.838	0.834	0.825	0.837
5760	0.836	0.832	0.826	0.835
5770	0.846	0.842	0.838	0.846
5780	0.832	0.828	0.823	0.834
5790	0.855	0.851	0.849	0.856
5800	0.845	0.840	0.841	0.844
5810	0.838	0.833	0.831	0.838
5820	0.823	0.818	0.820	0.822
5830	0.837	0.833	0.828	0.837
5840	0.845	0.840	0.839	0.845
5850	0.821	0.816	0.820	0.822
5860	0.832	0.828	0.827	0.834
5870	0.840	0.836	0.837	0.840
5880	0.832	0.828	0.828	0.831
5890	0.837	0.833	0.840	0.837
5900	0.850	0.846	0.839	0.852
5910	0.835	0.831	0.826	0.835
5920	0.837	0.833	0.844	0.837
5930	0.837	0.833	0.829	0.838
5940	0.828	0.824	0.816	0.828
5950	0.847	0.843	0.848	0.849
5960	0.842	0.837	0.841	0.842
5970	0.834	0.830	0.831	0.835
5980	0.835	0.831	0.830	0.836
5990	0.848	0.844	0.841	0.846
6000	0.840	0.836	0.830	0.839
6010	0.834	0.829	0.829	0.835
6020	0.825	0.820	0.812	0.825

6030	0.829	0.824	0.824	0.830
6040	0.835	0.831	0.831	0.835
6050	0.827	0.822	0.820	0.827
6060	0.842	0.838	0.841	0.844
6070	0.840	0.835	0.834	0.841
6080	0.834	0.829	0.832	0.834
6090	0.835	0.831	0.827	0.834
6100	0.852	0.848	0.844	0.853
6110	0.839	0.835	0.839	0.838
6120	0.849	0.844	0.842	0.849
6130	0.847	0.842	0.837	0.847
6140	0.821	0.816	0.820	0.821
6150	0.858	0.854	0.853	0.859
6160	0.836	0.832	0.831	0.837
6170	0.829	0.824	0.825	0.830
6180	0.818	0.813	0.810	0.818
6190	0.831	0.826	0.827	0.832
6200	0.830	0.826	0.830	0.830
6210	0.832	0.827	0.830	0.832
6220	0.851	0.847	0.846	0.850
6230	0.834	0.829	0.825	0.835
6240	0.845	0.840	0.844	0.845
6250	0.819	0.814	0.805	0.820
6260	0.841	0.837	0.833	0.841
6270	0.838	0.833	0.832	0.838
6280	0.845	0.841	0.835	0.845
6290	0.840	0.835	0.843	0.841
6300	0.832	0.827	0.827	0.831
6310	0.830	0.825	0.817	0.830
6320	0.830	0.826	0.816	0.832
6330	0.821	0.816	0.818	0.820
6340	0.831	0.826	0.826	0.832
6350	0.843	0.839	0.844	0.843
6360	0.831	0.826	0.828	0.830
6370	0.838	0.834	0.833	0.840
6380	0.842	0.837	0.846	0.843
6390	0.840	0.836	0.828	0.841
6400	0.822	0.817	0.819	0.823
6410	0.828	0.823	0.819	0.827
6420	0.817	0.812	0.820	0.817
6430	0.833	0.829	0.835	0.832
6440	0.840	0.836	0.826	0.842
6450	0.820	0.815	0.813	0.820

6460	0.847	0.842	0.837	0.848
6470	0.835	0.831	0.829	0.834
6480	0.841	0.837	0.838	0.841
6490	0.849	0.845	0.846	0.850
6500	0.839	0.835	0.830	0.840
6510	0.845	0.840	0.843	0.844
6520	0.825	0.820	0.828	0.825
6530	0.833	0.829	0.827	0.833
6540	0.826	0.821	0.818	0.827
6550	0.832	0.827	0.828	0.832
6560	0.835	0.831	0.832	0.834
6570	0.829	0.824	0.822	0.830
6580	0.823	0.818	0.824	0.823
6590	0.830	0.826	0.825	0.831
6600	0.845	0.841	0.842	0.845
6610	0.826	0.822	0.817	0.826
6620	0.842	0.838	0.831	0.843
6630	0.838	0.833	0.833	0.840
6640	0.838	0.834	0.842	0.838
6650	0.847	0.842	0.837	0.847
6660	0.838	0.834	0.825	0.840
6670	0.840	0.836	0.830	0.841
6680	0.838	0.833	0.833	0.838
6690	0.853	0.849	0.852	0.852
6700	0.840	0.836	0.837	0.840
6710	0.850	0.846	0.850	0.850
6720	0.839	0.835	0.837	0.840
6730	0.842	0.838	0.839	0.840
6740	0.840	0.836	0.833	0.840
6750	0.829	0.824	0.825	0.830
6760	0.841	0.837	0.840	0.841
6770	0.836	0.831	0.835	0.835
6780	0.828	0.823	0.828	0.827
6790	0.843	0.839	0.841	0.842
6800	0.836	0.831	0.835	0.837
6810	0.838	0.834	0.830	0.838
6820	0.835	0.831	0.836	0.833
6830	0.836	0.832	0.828	0.837
6840	0.838	0.834	0.834	0.839
6850	0.844	0.840	0.837	0.845
6860	0.836	0.831	0.837	0.836
6870	0.841	0.837	0.825	0.843
6880	0.836	0.831	0.835	0.837

6890	0.822	0.817	0.824	0.823
6900	0.840	0.835	0.831	0.840
6910	0.812	0.807	0.808	0.813
6920	0.840	0.835	0.837	0.841
6930	0.819	0.814	0.813	0.820
6940	0.837	0.833	0.830	0.838
6950	0.838	0.834	0.829	0.839
6960	0.838	0.833	0.838	0.837
6970	0.830	0.826	0.823	0.831
6980	0.831	0.826	0.823	0.832
6990	0.839	0.835	0.835	0.840
7000	0.845	0.840	0.841	0.847
7010	0.833	0.828	0.824	0.834
7020	0.838	0.834	0.835	0.840
7030	0.842	0.838	0.835	0.843
7040	0.840	0.835	0.827	0.840
7050	0.838	0.834	0.830	0.838
7060	0.835	0.830	0.838	0.834
7070	0.832	0.828	0.821	0.831
7080	0.832	0.828	0.825	0.833
7090	0.840	0.835	0.839	0.838
7100	0.828	0.823	0.820	0.829
7110	0.836	0.832	0.836	0.836
7120	0.843	0.839	0.842	0.844
7130	0.837	0.833	0.830	0.837
7140	0.824	0.820	0.812	0.825
7150	0.840	0.835	0.835	0.840
7160	0.839	0.835	0.831	0.840
7170	0.845	0.840	0.839	0.845
7180	0.834	0.829	0.836	0.835
7190	0.832	0.828	0.822	0.833
7200	0.852	0.848	0.847	0.853
7210	0.821	0.816	0.817	0.821
7220	0.843	0.839	0.830	0.843
7230	0.833	0.829	0.831	0.833
7240	0.833	0.828	0.823	0.834
7250	0.843	0.839	0.843	0.843
7260	0.837	0.833	0.834	0.837
7270	0.828	0.824	0.825	0.830
7280	0.842	0.838	0.838	0.843
7290	0.831	0.826	0.825	0.832
7300	0.832	0.828	0.825	0.833
7310	0.839	0.835	0.827	0.839

7320	0.823	0.818	0.816	0.825
7330	0.834	0.829	0.842	0.834
7340	0.832	0.828	0.827	0.834
7350	0.832	0.828	0.825	0.832
7360	0.828	0.823	0.818	0.830
7370	0.825	0.820	0.817	0.826
7380	0.847	0.843	0.845	0.847
7390	0.830	0.826	0.825	0.831
7400	0.834	0.830	0.831	0.835
7410	0.830	0.826	0.823	0.831
7420	0.824	0.819	0.826	0.825
7430	0.836	0.832	0.826	0.837
7440	0.834	0.829	0.832	0.835
7450	0.841	0.837	0.846	0.840
7460	0.831	0.826	0.826	0.832
7470	0.829	0.824	0.829	0.829
7480	0.829	0.824	0.819	0.829
7490	0.867	0.863	0.863	0.867
7500	0.843	0.839	0.841	0.845
7510	0.834	0.830	0.831	0.835
7520	0.839	0.835	0.838	0.840
7530	0.838	0.833	0.833	0.839
7540	0.841	0.837	0.826	0.842
7550	0.844	0.840	0.837	0.845
7560	0.843	0.839	0.843	0.843
7570	0.823	0.818	0.817	0.822
7580	0.816	0.811	0.809	0.816
7590	0.830	0.825	0.827	0.829
7600	0.839	0.835	0.833	0.839
7610	0.836	0.831	0.823	0.834
7620	0.828	0.823	0.816	0.828
7630	0.836	0.831	0.824	0.836
7640	0.826	0.822	0.820	0.827
7650	0.830	0.825	0.825	0.829
7660	0.834	0.830	0.835	0.835
7670	0.831	0.826	0.819	0.832
7680	0.825	0.820	0.811	0.826
7690	0.836	0.831	0.823	0.837
7700	0.850	0.846	0.840	0.851
7710	0.834	0.829	0.833	0.835
7720	0.834	0.829	0.827	0.834
7730	0.828	0.823	0.824	0.827
7740	0.826	0.821	0.825	0.827

7750	0.839	0.835	0.843	0.838
7760	0.835	0.830	0.836	0.836
7770	0.832	0.828	0.819	0.833
7780	0.832	0.828	0.833	0.834
7790	0.828	0.824	0.825	0.829
7800	0.831	0.826	0.827	0.832
7810	0.832	0.827	0.829	0.831
7820	0.836	0.832	0.830	0.838
7830	0.842	0.838	0.841	0.842
7840	0.836	0.832	0.835	0.837
7850	0.819	0.815	0.807	0.819
7860	0.836	0.831	0.832	0.837
7870	0.830	0.825	0.821	0.829
7880	0.842	0.838	0.837	0.842
7890	0.834	0.830	0.826	0.834
7900	0.845	0.841	0.837	0.845
7910	0.825	0.820	0.819	0.825
7920	0.833	0.828	0.826	0.832
7930	0.839	0.835	0.830	0.841
7940	0.828	0.823	0.825	0.827
7950	0.841	0.837	0.838	0.840
7960	0.832	0.828	0.828	0.832
7970	0.832	0.828	0.838	0.832
7980	0.832	0.827	0.823	0.831
7990	0.840	0.835	0.848	0.839
8000	0.843	0.839	0.844	0.844
8010	0.824	0.819	0.816	0.823
8020	0.834	0.829	0.833	0.834
8030	0.811	0.806	0.794	0.812
8040	0.839	0.835	0.836	0.839
8050	0.831	0.826	0.833	0.830
8060	0.829	0.824	0.819	0.830
8070	0.847	0.843	0.840	0.847
8080	0.850	0.846	0.839	0.851
8090	0.840	0.836	0.831	0.841
8100	0.819	0.815	0.820	0.818
8110	0.833	0.828	0.830	0.833
8120	0.850	0.846	0.843	0.850
8130	0.853	0.849	0.845	0.854
8140	0.830	0.825	0.820	0.829
8150	0.824	0.819	0.812	0.826
8160	0.842	0.837	0.837	0.842
8170	0.834	0.830	0.823	0.836

8180	0.838	0.834	0.828	0.840
8190	0.849	0.845	0.843	0.850
8200	0.842	0.837	0.839	0.843
8210	0.826	0.822	0.821	0.827
8220	0.837	0.833	0.834	0.837
8230	0.847	0.843	0.840	0.847
8240	0.847	0.843	0.838	0.849
8250	0.826	0.822	0.819	0.827
8260	0.850	0.846	0.848	0.850
8270	0.846	0.842	0.840	0.847
8280	0.837	0.833	0.830	0.838
8290	0.830	0.826	0.829	0.830
8300	0.833	0.829	0.827	0.832
8310	0.831	0.826	0.829	0.830
8320	0.845	0.841	0.831	0.846
8330	0.838	0.833	0.831	0.837
8340	0.849	0.844	0.847	0.849
8350	0.862	0.858	0.855	0.861
8360	0.840	0.836	0.843	0.841
8370	0.849	0.845	0.848	0.848
8380	0.833	0.829	0.828	0.833
8390	0.848	0.844	0.843	0.848
8400	0.838	0.834	0.831	0.839
8410	0.845	0.841	0.844	0.845
8420	0.832	0.828	0.825	0.834
8430	0.834	0.829	0.835	0.833
8440	0.843	0.839	0.838	0.844
8450	0.828	0.824	0.825	0.829
8460	0.827	0.822	0.824	0.827
8470	0.853	0.849	0.849	0.855
8480	0.838	0.834	0.835	0.839
8490	0.827	0.822	0.827	0.828
8500	0.834	0.830	0.827	0.835
8510	0.823	0.819	0.818	0.825
8520	0.852	0.848	0.849	0.851
8530	0.834	0.830	0.830	0.834
8540	0.851	0.847	0.851	0.851
8550	0.836	0.832	0.839	0.837
8560	0.837	0.833	0.834	0.839
8570	0.837	0.833	0.836	0.838
8580	0.836	0.832	0.831	0.837
8590	0.817	0.812	0.808	0.818
8600	0.836	0.832	0.831	0.838

8610	0.820	0.815	0.818	0.821
8620	0.823	0.819	0.829	0.823
8630	0.845	0.841	0.841	0.845
8640	0.827	0.822	0.820	0.827
8650	0.821	0.817	0.811	0.823
8660	0.853	0.849	0.848	0.853
8670	0.835	0.831	0.837	0.836
8680	0.844	0.839	0.843	0.844
8690	0.845	0.841	0.841	0.844
8700	0.828	0.823	0.824	0.828
8710	0.828	0.824	0.822	0.828
8720	0.839	0.835	0.829	0.841
8730	0.843	0.839	0.834	0.843
8740	0.834	0.829	0.825	0.835
8750	0.843	0.839	0.844	0.843
8760	0.819	0.815	0.817	0.820
8770	0.848	0.844	0.847	0.848
8780	0.830	0.826	0.824	0.832
8790	0.829	0.824	0.828	0.831
8800	0.845	0.840	0.843	0.844
8810	0.838	0.834	0.831	0.837
8820	0.848	0.844	0.850	0.848
8830	0.847	0.842	0.842	0.846
8840	0.843	0.839	0.835	0.843
8850	0.850	0.846	0.844	0.850
8860	0.845	0.840	0.835	0.845
8870	0.836	0.832	0.829	0.837
8880	0.837	0.833	0.829	0.838
8890	0.847	0.843	0.841	0.846
8900	0.853	0.849	0.844	0.854
8910	0.847	0.842	0.837	0.848
8920	0.846	0.842	0.842	0.846
8930	0.847	0.843	0.847	0.849
8940	0.838	0.833	0.826	0.839
8950	0.834	0.830	0.832	0.835

(3) IFS results with RF on the LightGBM feature list

Number of features	ACC	MCC	Macro F1	Weighted F1
10	0.788	0.783	0.816	0.778
20	0.911	0.909	0.926	0.909
30	0.940	0.939	0.948	0.940
40	0.946	0.945	0.952	0.946
50	0.954	0.953	0.958	0.954

60	0.962	0.961	0.968	0.962
70	0.957	0.956	0.966	0.957
80	0.967	0.966	0.972	0.967
90	0.965	0.964	0.970	0.965
100	0.964	0.963	0.970	0.964
110	0.973	0.972	0.979	0.973
120	0.971	0.970	0.975	0.971
130	0.971	0.970	0.976	0.971
140	0.973	0.972	0.978	0.972
150	0.975	0.974	0.979	0.975
160	0.967	0.966	0.972	0.967
170	0.970	0.969	0.974	0.970
180	0.976	0.975	0.979	0.975
190	0.977	0.976	0.981	0.977
200	0.979	0.978	0.983	0.979
210	0.974	0.973	0.979	0.973
220	0.977	0.976	0.983	0.977
230	0.976	0.976	0.982	0.976
240	0.972	0.971	0.977	0.972
250	0.977	0.976	0.982	0.977
260	0.977	0.976	0.983	0.977
270	0.975	0.974	0.982	0.975
280	0.978	0.978	0.984	0.978
290	0.978	0.978	0.982	0.978
300	0.978	0.978	0.985	0.978
310	0.981	0.980	0.986	0.981
320	0.979	0.978	0.983	0.979
330	0.977	0.976	0.982	0.977
340	0.976	0.975	0.982	0.975
350	0.981	0.980	0.985	0.981
360	0.982	0.982	0.985	0.982
370	0.982	0.981	0.984	0.982
380	0.982	0.981	0.988	0.982
390	0.980	0.979	0.984	0.980
400	0.977	0.976	0.982	0.977
410	0.982	0.981	0.985	0.982
420	0.982	0.982	0.987	0.982
430	0.982	0.982	0.986	0.982
440	0.979	0.978	0.984	0.979
450	0.982	0.981	0.985	0.982
460	0.985	0.985	0.988	0.985
470	0.986	0.985	0.990	0.986
480	0.982	0.982	0.986	0.982

490	0.980	0.979	0.983	0.979
500	0.984	0.983	0.987	0.984
510	0.982	0.982	0.987	0.982
520	0.981	0.980	0.985	0.981
530	0.983	0.983	0.986	0.983
540	0.984	0.983	0.988	0.984
550	0.984	0.983	0.988	0.984
560	0.986	0.985	0.990	0.986
570	0.983	0.983	0.986	0.983
580	0.984	0.984	0.987	0.984
590	0.984	0.984	0.989	0.984
600	0.982	0.981	0.986	0.981
610	0.982	0.981	0.986	0.982
620	0.982	0.982	0.987	0.982
630	0.984	0.984	0.987	0.984
640	0.982	0.981	0.985	0.982
650	0.982	0.982	0.987	0.982
660	0.984	0.984	0.988	0.984
670	0.986	0.985	0.990	0.986
680	0.985	0.985	0.990	0.985
690	0.982	0.982	0.986	0.982
700	0.982	0.981	0.987	0.981
710	0.982	0.982	0.989	0.982
720	0.983	0.983	0.987	0.983
730	0.985	0.985	0.987	0.985
740	0.982	0.982	0.986	0.982
750	0.986	0.986	0.991	0.986
760	0.983	0.983	0.987	0.983
770	0.984	0.984	0.989	0.984
780	0.984	0.984	0.988	0.984
790	0.983	0.983	0.988	0.983
800	0.986	0.986	0.992	0.986
810	0.984	0.983	0.988	0.984
820	0.986	0.985	0.990	0.986
830	0.982	0.982	0.987	0.982
840	0.983	0.983	0.988	0.983
850	0.984	0.983	0.988	0.984
860	0.983	0.983	0.987	0.983
870	0.985	0.985	0.988	0.985
880	0.984	0.983	0.987	0.984
890	0.984	0.983	0.988	0.984
900	0.982	0.981	0.985	0.981
910	0.982	0.982	0.987	0.982

920	0.984	0.983	0.988	0.983
930	0.981	0.980	0.987	0.981
940	0.984	0.984	0.988	0.984
950	0.985	0.985	0.989	0.985
960	0.981	0.980	0.987	0.981
970	0.984	0.984	0.988	0.984
980	0.983	0.983	0.988	0.983
990	0.983	0.983	0.987	0.983
1000	0.983	0.983	0.988	0.983
1010	0.982	0.982	0.989	0.982
1020	0.984	0.983	0.987	0.984
1030	0.984	0.984	0.987	0.984
1040	0.983	0.983	0.988	0.983
1050	0.982	0.982	0.987	0.982
1060	0.986	0.985	0.989	0.986
1070	0.982	0.981	0.987	0.982
1080	0.983	0.983	0.988	0.983
1090	0.984	0.984	0.989	0.984
1100	0.986	0.986	0.990	0.986
1110	0.984	0.983	0.988	0.983
1120	0.983	0.983	0.989	0.983
1130	0.984	0.983	0.988	0.984
1140	0.984	0.984	0.987	0.984
1150	0.984	0.983	0.988	0.983
1160	0.984	0.983	0.988	0.983
1170	0.986	0.985	0.990	0.986
1180	0.982	0.982	0.987	0.982
1190	0.987	0.987	0.990	0.987
1200	0.986	0.985	0.988	0.986
1210	0.984	0.984	0.988	0.984
1220	0.984	0.983	0.988	0.984
1230	0.984	0.984	0.988	0.984
1240	0.986	0.985	0.990	0.986
1250	0.984	0.984	0.990	0.984
1260	0.986	0.986	0.991	0.986
1270	0.984	0.984	0.989	0.984
1280	0.984	0.983	0.989	0.984
1290	0.984	0.984	0.989	0.984
1300	0.986	0.985	0.991	0.986
1310	0.984	0.984	0.987	0.984
1320	0.985	0.985	0.990	0.985
1330	0.986	0.985	0.989	0.986
1340	0.984	0.983	0.987	0.984

1350	0.986	0.986	0.989	0.987
1360	0.986	0.986	0.989	0.986
1370	0.985	0.985	0.989	0.985
1380	0.986	0.985	0.988	0.986
1390	0.986	0.986	0.990	0.986
1400	0.987	0.987	0.990	0.987
1410	0.982	0.981	0.986	0.981
1420	0.985	0.985	0.989	0.985
1430	0.987	0.987	0.990	0.987
1440	0.987	0.987	0.990	0.987
1450	0.985	0.985	0.989	0.985
1460	0.986	0.986	0.991	0.986
1470	0.988	0.988	0.991	0.988
1480	0.987	0.987	0.991	0.987
1490	0.984	0.983	0.988	0.984
1500	0.986	0.985	0.990	0.986
1510	0.984	0.984	0.988	0.984
1520	0.988	0.987	0.991	0.988
1530	0.986	0.985	0.988	0.986
1540	0.986	0.985	0.990	0.986
1550	0.984	0.984	0.987	0.984
1560	0.985	0.985	0.988	0.985
1570	0.984	0.983	0.988	0.984
1580	0.984	0.983	0.988	0.984
1590	0.988	0.987	0.991	0.988
1600	0.987	0.987	0.990	0.987
1610	0.983	0.983	0.988	0.983
1620	0.986	0.985	0.990	0.986
1630	0.986	0.986	0.991	0.986
1640	0.990	0.990	0.992	0.990
1650	0.986	0.986	0.990	0.986
1660	0.986	0.985	0.989	0.986
1670	0.989	0.989	0.991	0.989
1680	0.984	0.983	0.987	0.983
1690	0.986	0.985	0.988	0.986
1700	0.984	0.983	0.988	0.984
1710	0.982	0.982	0.986	0.982
1720	0.984	0.984	0.989	0.984
1730	0.986	0.985	0.990	0.986
1740	0.986	0.985	0.990	0.985
1750	0.986	0.985	0.988	0.986
1760	0.988	0.987	0.991	0.988
1770	0.986	0.985	0.990	0.986

1780	0.984	0.983	0.987	0.984
1790	0.984	0.984	0.989	0.984
1800	0.983	0.983	0.988	0.983
1810	0.986	0.986	0.991	0.986
1820	0.988	0.987	0.990	0.988
1830	0.989	0.989	0.992	0.989
1840	0.986	0.986	0.990	0.986
1850	0.984	0.984	0.988	0.984
1860	0.986	0.986	0.989	0.986
1870	0.984	0.983	0.988	0.984
1880	0.986	0.985	0.989	0.986
1890	0.984	0.983	0.989	0.984
1900	0.987	0.987	0.991	0.987
1910	0.986	0.985	0.989	0.985
1920	0.982	0.981	0.986	0.982
1930	0.986	0.986	0.990	0.986
1940	0.987	0.987	0.990	0.987
1950	0.986	0.986	0.990	0.986
1960	0.987	0.987	0.991	0.987
1970	0.990	0.990	0.993	0.990
1980	0.988	0.987	0.991	0.988
1990	0.987	0.987	0.991	0.987
2000	0.984	0.983	0.988	0.983
2010	0.988	0.988	0.991	0.988
2020	0.984	0.983	0.987	0.984
2030	0.985	0.985	0.989	0.985
2040	0.987	0.987	0.990	0.987
2050	0.984	0.983	0.989	0.983
2060	0.988	0.987	0.990	0.988
2070	0.987	0.987	0.989	0.987
2080	0.987	0.987	0.992	0.987
2090	0.986	0.986	0.990	0.986
2100	0.985	0.985	0.990	0.985
2110	0.986	0.986	0.990	0.986
2120	0.983	0.983	0.988	0.983
2130	0.988	0.987	0.992	0.988
2140	0.988	0.988	0.991	0.988
2150	0.987	0.987	0.991	0.987
2160	0.986	0.985	0.989	0.986
2170	0.988	0.987	0.990	0.988
2180	0.988	0.987	0.991	0.988
2190	0.984	0.983	0.988	0.984
2200	0.986	0.985	0.989	0.986

2210	0.986	0.986	0.989	0.986
2220	0.988	0.987	0.991	0.988
2230	0.987	0.987	0.991	0.987
2240	0.988	0.987	0.990	0.988
2250	0.990	0.990	0.993	0.990
2260	0.986	0.986	0.989	0.986
2270	0.986	0.985	0.989	0.986
2280	0.987	0.987	0.991	0.987
2290	0.987	0.987	0.989	0.987
2300	0.988	0.987	0.992	0.988
2310	0.990	0.990	0.993	0.990
2320	0.986	0.986	0.990	0.986
2330	0.985	0.985	0.989	0.985
2340	0.987	0.987	0.990	0.987
2350	0.985	0.985	0.990	0.985
2360	0.989	0.989	0.991	0.989
2370	0.988	0.988	0.991	0.988
2380	0.988	0.987	0.991	0.988
2390	0.985	0.985	0.988	0.985
2400	0.986	0.986	0.990	0.986
2410	0.984	0.984	0.988	0.984
2420	0.984	0.984	0.989	0.984
2430	0.984	0.983	0.989	0.983
2440	0.984	0.984	0.988	0.984
2450	0.981	0.980	0.985	0.981
2460	0.986	0.986	0.990	0.986
2470	0.988	0.987	0.990	0.988
2480	0.988	0.987	0.991	0.988
2490	0.986	0.985	0.989	0.986
2500	0.985	0.985	0.988	0.985
2510	0.985	0.985	0.987	0.985
2520	0.984	0.983	0.988	0.983
2530	0.984	0.984	0.988	0.984
2540	0.985	0.985	0.989	0.985
2550	0.982	0.981	0.987	0.981
2560	0.985	0.985	0.989	0.985
2570	0.988	0.987	0.990	0.988
2580	0.982	0.981	0.988	0.982
2590	0.986	0.986	0.991	0.986
2600	0.987	0.987	0.989	0.987
2610	0.989	0.989	0.991	0.989
2620	0.988	0.988	0.991	0.988
2630	0.985	0.985	0.988	0.985

2640	0.988	0.988	0.992	0.988
2650	0.986	0.985	0.988	0.986
2660	0.985	0.985	0.989	0.985
2670	0.986	0.986	0.989	0.986
2680	0.988	0.987	0.992	0.988
2690	0.988	0.988	0.992	0.988
2700	0.986	0.985	0.989	0.986
2710	0.986	0.986	0.989	0.986
2720	0.989	0.989	0.993	0.989
2730	0.982	0.981	0.985	0.981
2740	0.986	0.986	0.990	0.986
2750	0.988	0.988	0.992	0.988
2760	0.984	0.984	0.989	0.984
2770	0.988	0.988	0.992	0.988
2780	0.988	0.987	0.991	0.988
2790	0.982	0.982	0.987	0.982
2800	0.986	0.985	0.988	0.986
2810	0.987	0.987	0.990	0.987
2820	0.986	0.986	0.991	0.986
2830	0.986	0.985	0.988	0.986
2840	0.985	0.985	0.988	0.985
2850	0.985	0.985	0.989	0.985
2860	0.986	0.986	0.990	0.986
2870	0.988	0.987	0.991	0.988
2880	0.985	0.985	0.988	0.985
2890	0.988	0.988	0.991	0.988
2900	0.982	0.982	0.987	0.982
2910	0.986	0.985	0.990	0.986
2920	0.985	0.985	0.988	0.985
2930	0.984	0.984	0.989	0.984
2940	0.981	0.980	0.986	0.981
2950	0.982	0.981	0.987	0.981
2960	0.982	0.981	0.987	0.981
2970	0.982	0.982	0.987	0.982
2980	0.986	0.986	0.990	0.986
2990	0.986	0.985	0.990	0.986
3000	0.984	0.984	0.988	0.984
3010	0.986	0.986	0.989	0.986
3020	0.986	0.985	0.990	0.986
3030	0.988	0.987	0.991	0.988
3040	0.983	0.983	0.987	0.983
3050	0.985	0.985	0.987	0.985
3060	0.984	0.984	0.989	0.984

3070	0.982	0.982	0.988	0.982
3080	0.986	0.986	0.989	0.986
3090	0.988	0.987	0.991	0.988
3100	0.986	0.986	0.990	0.986
3110	0.986	0.985	0.989	0.986
3120	0.984	0.984	0.990	0.984
3130	0.986	0.985	0.990	0.986
3140	0.986	0.986	0.989	0.986
3150	0.984	0.983	0.986	0.984
3160	0.984	0.984	0.988	0.984
3170	0.984	0.983	0.988	0.984
3180	0.986	0.986	0.989	0.986
3190	0.984	0.984	0.989	0.984
3200	0.986	0.985	0.990	0.986
3210	0.985	0.985	0.989	0.985
3220	0.987	0.987	0.991	0.987
3230	0.987	0.987	0.991	0.987
3240	0.985	0.985	0.990	0.985
3250	0.986	0.986	0.991	0.986
3260	0.986	0.986	0.991	0.986
3270	0.980	0.979	0.982	0.979
3280	0.980	0.980	0.985	0.980
3290	0.985	0.985	0.989	0.985
3300	0.984	0.984	0.988	0.984
3310	0.986	0.985	0.990	0.986
3320	0.988	0.988	0.992	0.988
3330	0.986	0.985	0.989	0.986
3340	0.984	0.984	0.989	0.984
3350	0.987	0.987	0.990	0.987
3360	0.984	0.983	0.987	0.983
3370	0.983	0.983	0.989	0.983
3380	0.986	0.986	0.991	0.986
3390	0.986	0.986	0.990	0.986
3400	0.982	0.981	0.986	0.982
3410	0.985	0.985	0.988	0.985
3420	0.985	0.985	0.988	0.985
3430	0.988	0.987	0.991	0.988
3440	0.989	0.989	0.992	0.989
3450	0.984	0.983	0.987	0.984
3460	0.984	0.983	0.988	0.984
3470	0.984	0.984	0.989	0.984
3480	0.984	0.984	0.988	0.984
3490	0.981	0.980	0.986	0.981

3500	0.984	0.984	0.988	0.984
3510	0.982	0.982	0.988	0.982
3520	0.986	0.986	0.990	0.986
3530	0.985	0.985	0.988	0.985
3540	0.986	0.985	0.988	0.986
3550	0.986	0.986	0.990	0.986
3560	0.987	0.987	0.991	0.987
3570	0.984	0.984	0.988	0.984
3580	0.984	0.983	0.987	0.984
3590	0.982	0.981	0.985	0.981
3600	0.988	0.987	0.991	0.988
3610	0.984	0.984	0.989	0.984
3620	0.984	0.983	0.986	0.984
3630	0.985	0.985	0.991	0.985
3640	0.984	0.984	0.989	0.984
3650	0.987	0.987	0.990	0.987
3660	0.985	0.985	0.989	0.985
3670	0.986	0.985	0.989	0.986
3680	0.983	0.983	0.989	0.983
3690	0.980	0.979	0.985	0.979
3700	0.985	0.985	0.989	0.985
3710	0.985	0.985	0.988	0.985
3720	0.981	0.980	0.985	0.981
3730	0.984	0.983	0.989	0.984
3740	0.984	0.984	0.988	0.984
3750	0.983	0.983	0.988	0.983
3760	0.986	0.985	0.988	0.986
3770	0.985	0.985	0.988	0.985
3780	0.988	0.987	0.991	0.988
3790	0.985	0.985	0.989	0.985
3800	0.983	0.983	0.988	0.983
3810	0.984	0.984	0.988	0.984
3820	0.986	0.985	0.991	0.986
3830	0.984	0.984	0.988	0.984
3840	0.986	0.985	0.990	0.986
3850	0.982	0.981	0.985	0.981
3860	0.986	0.986	0.990	0.986
3870	0.988	0.987	0.991	0.988
3880	0.985	0.985	0.989	0.985
3890	0.985	0.985	0.988	0.985
3900	0.984	0.984	0.988	0.984
3910	0.984	0.984	0.988	0.984
3920	0.985	0.985	0.987	0.985

3930	0.984	0.983	0.987	0.984
3940	0.987	0.987	0.989	0.987
3950	0.984	0.984	0.989	0.984
3960	0.984	0.983	0.988	0.984
3970	0.984	0.983	0.988	0.984
3980	0.986	0.985	0.989	0.986
3990	0.984	0.984	0.988	0.984
4000	0.984	0.984	0.988	0.984
4010	0.985	0.985	0.990	0.985
4020	0.982	0.982	0.987	0.982
4030	0.987	0.987	0.989	0.987
4040	0.984	0.984	0.989	0.984
4050	0.982	0.981	0.988	0.981
4060	0.986	0.985	0.989	0.986
4070	0.986	0.985	0.989	0.986
4080	0.982	0.982	0.987	0.982
4090	0.986	0.985	0.990	0.986
4100	0.984	0.983	0.989	0.983
4110	0.982	0.982	0.986	0.982
4120	0.985	0.985	0.989	0.985
4130	0.984	0.983	0.987	0.984
4140	0.984	0.983	0.989	0.984
4150	0.988	0.987	0.990	0.988
4160	0.986	0.986	0.990	0.986
4170	0.982	0.982	0.987	0.982
4180	0.984	0.984	0.987	0.984
4190	0.986	0.985	0.987	0.986
4200	0.984	0.984	0.988	0.984
4210	0.986	0.985	0.989	0.986
4220	0.986	0.986	0.989	0.986
4230	0.986	0.986	0.990	0.986
4240	0.986	0.986	0.991	0.986
4250	0.988	0.987	0.991	0.988
4260	0.986	0.986	0.989	0.986
4270	0.985	0.985	0.988	0.985
4280	0.984	0.984	0.989	0.984
4290	0.984	0.984	0.987	0.984
4300	0.986	0.986	0.989	0.986
4310	0.984	0.984	0.988	0.984
4320	0.986	0.986	0.989	0.986
4330	0.984	0.984	0.988	0.984
4340	0.985	0.985	0.988	0.985
4350	0.984	0.984	0.988	0.984

4360	0.981	0.980	0.984	0.981
4370	0.984	0.983	0.987	0.983
4380	0.985	0.985	0.988	0.985
4390	0.984	0.984	0.989	0.984
4400	0.986	0.986	0.989	0.987
4410	0.984	0.983	0.986	0.984
4420	0.986	0.985	0.988	0.986
4430	0.986	0.986	0.989	0.986
4440	0.985	0.985	0.989	0.985
4450	0.985	0.985	0.988	0.985
4460	0.982	0.982	0.987	0.982
4470	0.987	0.987	0.991	0.987
4480	0.986	0.986	0.989	0.986
4490	0.986	0.985	0.988	0.986
4500	0.983	0.983	0.986	0.983
4510	0.984	0.984	0.988	0.984
4520	0.990	0.990	0.993	0.991
4530	0.983	0.983	0.988	0.983
4540	0.985	0.985	0.987	0.985
4550	0.983	0.983	0.986	0.983
4560	0.985	0.985	0.988	0.985
4570	0.984	0.983	0.987	0.984
4580	0.984	0.983	0.988	0.983
4590	0.986	0.986	0.989	0.986
4600	0.985	0.985	0.988	0.985
4610	0.986	0.985	0.989	0.986
4620	0.987	0.987	0.992	0.987
4630	0.986	0.986	0.989	0.986
4640	0.984	0.983	0.988	0.983
4650	0.986	0.986	0.989	0.986
4660	0.986	0.986	0.989	0.986
4670	0.988	0.987	0.991	0.988
4680	0.990	0.990	0.993	0.990
4690	0.986	0.986	0.990	0.986
4700	0.986	0.985	0.989	0.986
4710	0.984	0.983	0.989	0.984
4720	0.987	0.987	0.991	0.987
4730	0.983	0.983	0.988	0.983
4740	0.983	0.983	0.986	0.983
4750	0.984	0.984	0.987	0.984
4760	0.986	0.985	0.989	0.986
4770	0.988	0.988	0.992	0.988
4780	0.984	0.983	0.988	0.984

4790	0.986	0.985	0.988	0.986
4800	0.986	0.986	0.990	0.986
4810	0.984	0.983	0.989	0.984
4820	0.986	0.985	0.988	0.986
4830	0.983	0.983	0.988	0.983
4840	0.984	0.984	0.987	0.984
4850	0.984	0.984	0.988	0.984
4860	0.984	0.984	0.988	0.984
4870	0.986	0.986	0.988	0.986
4880	0.986	0.985	0.989	0.986
4890	0.986	0.986	0.989	0.986
4900	0.984	0.983	0.989	0.983
4910	0.987	0.987	0.990	0.987
4920	0.983	0.983	0.986	0.983
4930	0.986	0.986	0.989	0.986
4940	0.984	0.984	0.988	0.984
4950	0.987	0.987	0.989	0.987
4960	0.984	0.984	0.988	0.984
4970	0.987	0.987	0.991	0.987
4980	0.986	0.986	0.990	0.986
4990	0.984	0.984	0.988	0.984
5000	0.982	0.982	0.987	0.982
5010	0.986	0.986	0.990	0.986
5020	0.985	0.985	0.988	0.985
5030	0.986	0.985	0.990	0.986
5040	0.986	0.985	0.988	0.986
5050	0.987	0.987	0.991	0.987
5060	0.986	0.986	0.990	0.986
5070	0.987	0.987	0.990	0.987
5080	0.982	0.982	0.987	0.982
5090	0.985	0.985	0.989	0.985
5100	0.986	0.986	0.990	0.986
5110	0.985	0.985	0.989	0.985
5120	0.985	0.985	0.988	0.985
5130	0.984	0.983	0.988	0.984
5140	0.985	0.985	0.989	0.985
5150	0.984	0.984	0.989	0.984
5160	0.986	0.986	0.989	0.986
5170	0.987	0.987	0.990	0.987
5180	0.984	0.984	0.988	0.984
5190	0.988	0.988	0.991	0.988
5200	0.985	0.985	0.987	0.985
5210	0.986	0.985	0.989	0.986

5220	0.986	0.985	0.990	0.986
5230	0.985	0.985	0.988	0.985
5240	0.984	0.984	0.988	0.984
5250	0.986	0.985	0.989	0.986
5260	0.985	0.985	0.989	0.985
5270	0.984	0.983	0.988	0.983
5280	0.986	0.986	0.991	0.986
5290	0.984	0.983	0.989	0.983
5300	0.982	0.982	0.987	0.982
5310	0.988	0.987	0.991	0.988
5320	0.984	0.984	0.988	0.984
5330	0.984	0.984	0.988	0.984
5340	0.987	0.987	0.989	0.987
5350	0.986	0.986	0.989	0.986
5360	0.986	0.986	0.990	0.986
5370	0.985	0.985	0.989	0.985
5380	0.984	0.984	0.988	0.984
5390	0.986	0.986	0.989	0.986
5400	0.984	0.984	0.987	0.984
5410	0.986	0.985	0.989	0.986
5420	0.986	0.985	0.988	0.986
5430	0.983	0.983	0.985	0.983
5440	0.983	0.983	0.987	0.983
5450	0.984	0.984	0.987	0.984
5460	0.986	0.985	0.988	0.986
5470	0.982	0.982	0.987	0.982
5480	0.985	0.985	0.988	0.985
5490	0.983	0.983	0.988	0.983
5500	0.984	0.984	0.989	0.984
5510	0.986	0.986	0.988	0.986
5520	0.986	0.986	0.989	0.986
5530	0.988	0.987	0.991	0.988
5540	0.985	0.985	0.990	0.985
5550	0.986	0.985	0.989	0.986
5560	0.985	0.985	0.989	0.985
5570	0.985	0.985	0.988	0.985
5580	0.983	0.983	0.988	0.983
5590	0.984	0.984	0.988	0.984
5600	0.985	0.985	0.987	0.985
5610	0.984	0.984	0.987	0.984
5620	0.985	0.985	0.988	0.985
5630	0.984	0.984	0.988	0.984
5640	0.986	0.985	0.989	0.986

5650	0.986	0.985	0.988	0.986
5660	0.985	0.985	0.989	0.985
5670	0.985	0.985	0.989	0.985
5680	0.984	0.983	0.988	0.984
5690	0.988	0.987	0.991	0.988
5700	0.986	0.985	0.988	0.986
5710	0.986	0.986	0.989	0.986
5720	0.984	0.983	0.989	0.983
5730	0.984	0.984	0.988	0.984
5740	0.986	0.985	0.988	0.986
5750	0.987	0.987	0.990	0.987
5760	0.984	0.983	0.988	0.984
5770	0.984	0.983	0.987	0.983
5780	0.984	0.984	0.988	0.984
5790	0.984	0.983	0.988	0.983
5800	0.982	0.981	0.985	0.981
5810	0.986	0.986	0.989	0.986
5820	0.984	0.983	0.987	0.984
5830	0.985	0.985	0.988	0.985
5840	0.986	0.985	0.989	0.985
5850	0.984	0.984	0.988	0.984
5860	0.982	0.981	0.985	0.981
5870	0.984	0.984	0.989	0.984
5880	0.986	0.985	0.988	0.986
5890	0.982	0.982	0.986	0.982
5900	0.986	0.986	0.990	0.986
5910	0.984	0.984	0.988	0.984
5920	0.982	0.982	0.988	0.982
5930	0.986	0.985	0.988	0.986
5940	0.988	0.987	0.990	0.988
5950	0.984	0.983	0.989	0.984
5960	0.982	0.982	0.987	0.982
5970	0.983	0.983	0.987	0.983
5980	0.987	0.987	0.991	0.987
5990	0.984	0.984	0.988	0.984
6000	0.981	0.980	0.985	0.981
6010	0.985	0.985	0.988	0.985
6020	0.985	0.985	0.988	0.985
6030	0.984	0.984	0.987	0.984
6040	0.988	0.987	0.991	0.988
6050	0.984	0.983	0.988	0.984
6060	0.984	0.984	0.988	0.984
6070	0.983	0.983	0.988	0.983

6080	0.984	0.984	0.989	0.984
6090	0.984	0.983	0.989	0.984
6100	0.981	0.980	0.984	0.981
6110	0.985	0.985	0.988	0.985
6120	0.986	0.986	0.990	0.986
6130	0.981	0.980	0.985	0.981
6140	0.987	0.987	0.990	0.987
6150	0.986	0.986	0.990	0.986
6160	0.984	0.984	0.989	0.984
6170	0.983	0.983	0.987	0.983
6180	0.984	0.984	0.988	0.984
6190	0.984	0.983	0.988	0.983
6200	0.986	0.986	0.991	0.986
6210	0.982	0.982	0.988	0.982
6220	0.985	0.985	0.988	0.985
6230	0.986	0.985	0.987	0.986
6240	0.984	0.983	0.989	0.984
6250	0.986	0.985	0.990	0.986
6260	0.986	0.985	0.990	0.986
6270	0.982	0.981	0.987	0.981
6280	0.984	0.984	0.988	0.984
6290	0.984	0.983	0.989	0.983
6300	0.986	0.986	0.991	0.986
6310	0.986	0.985	0.989	0.986
6320	0.985	0.985	0.989	0.985
6330	0.984	0.983	0.988	0.984
6340	0.984	0.983	0.988	0.984
6350	0.984	0.983	0.988	0.984
6360	0.984	0.983	0.988	0.983
6370	0.984	0.983	0.988	0.984
6380	0.982	0.982	0.986	0.982
6390	0.984	0.983	0.987	0.984
6400	0.983	0.983	0.987	0.983
6410	0.984	0.984	0.988	0.984
6420	0.983	0.983	0.987	0.983
6430	0.988	0.987	0.992	0.988
6440	0.985	0.985	0.988	0.985
6450	0.982	0.981	0.987	0.981
6460	0.983	0.983	0.987	0.983
6470	0.983	0.983	0.987	0.983
6480	0.984	0.984	0.988	0.984
6490	0.985	0.985	0.988	0.985
6500	0.984	0.984	0.987	0.984

6510	0.984	0.983	0.989	0.983
6520	0.983	0.983	0.986	0.983
6530	0.986	0.985	0.988	0.986
6540	0.984	0.983	0.987	0.984
6550	0.984	0.983	0.987	0.984
6560	0.986	0.985	0.990	0.986
6570	0.984	0.984	0.988	0.984
6580	0.984	0.983	0.988	0.984
6590	0.985	0.985	0.989	0.985
6600	0.985	0.985	0.989	0.985
6610	0.980	0.980	0.986	0.980
6620	0.982	0.981	0.987	0.981
6630	0.984	0.983	0.988	0.984
6640	0.985	0.985	0.988	0.985
6650	0.982	0.981	0.986	0.982
6660	0.982	0.982	0.987	0.982
6670	0.987	0.987	0.990	0.987
6680	0.983	0.983	0.988	0.983
6690	0.985	0.985	0.988	0.985
6700	0.982	0.982	0.987	0.982
6710	0.984	0.984	0.988	0.984
6720	0.986	0.985	0.988	0.986
6730	0.982	0.981	0.985	0.982
6740	0.984	0.983	0.989	0.984
6750	0.982	0.982	0.986	0.982
6760	0.984	0.983	0.988	0.984
6770	0.983	0.983	0.987	0.983
6780	0.980	0.980	0.983	0.980
6790	0.986	0.985	0.990	0.986
6800	0.984	0.984	0.989	0.984
6810	0.981	0.980	0.985	0.981
6820	0.984	0.983	0.988	0.984
6830	0.984	0.984	0.989	0.984
6840	0.988	0.987	0.991	0.988
6850	0.985	0.985	0.988	0.985
6860	0.985	0.985	0.989	0.985
6870	0.984	0.983	0.989	0.984
6880	0.983	0.983	0.987	0.983
6890	0.984	0.983	0.987	0.984
6900	0.987	0.987	0.990	0.987
6910	0.986	0.986	0.989	0.986
6920	0.985	0.985	0.988	0.985
6930	0.984	0.984	0.988	0.984

6940	0.982	0.982	0.987	0.982
6950	0.982	0.982	0.987	0.982
6960	0.982	0.982	0.986	0.982
6970	0.986	0.985	0.989	0.986
6980	0.986	0.985	0.990	0.986
6990	0.982	0.981	0.987	0.981
7000	0.983	0.983	0.988	0.983
7010	0.985	0.985	0.989	0.985
7020	0.984	0.984	0.987	0.984
7030	0.984	0.983	0.987	0.984
7040	0.982	0.982	0.987	0.982
7050	0.983	0.983	0.986	0.983
7060	0.986	0.986	0.990	0.986
7070	0.986	0.986	0.990	0.986
7080	0.984	0.983	0.987	0.984
7090	0.984	0.983	0.989	0.984
7100	0.984	0.983	0.988	0.983
7110	0.984	0.984	0.988	0.984
7120	0.986	0.986	0.989	0.986
7130	0.984	0.984	0.988	0.984
7140	0.983	0.983	0.987	0.983
7150	0.984	0.983	0.989	0.983
7160	0.987	0.987	0.992	0.987
7170	0.985	0.985	0.990	0.985
7180	0.980	0.980	0.986	0.980
7190	0.984	0.984	0.987	0.984
7200	0.982	0.982	0.986	0.982
7210	0.984	0.983	0.988	0.984
7220	0.981	0.980	0.985	0.981
7230	0.982	0.982	0.985	0.982
7240	0.984	0.983	0.989	0.983
7250	0.984	0.984	0.988	0.984
7260	0.985	0.985	0.989	0.985
7270	0.982	0.982	0.988	0.982
7280	0.982	0.982	0.988	0.982
7290	0.982	0.982	0.987	0.982
7300	0.982	0.982	0.988	0.982
7310	0.982	0.982	0.987	0.982
7320	0.984	0.984	0.988	0.984
7330	0.983	0.983	0.987	0.983
7340	0.984	0.983	0.988	0.983
7350	0.983	0.983	0.989	0.983
7360	0.986	0.986	0.990	0.986

7370	0.985	0.985	0.989	0.985
7380	0.978	0.977	0.982	0.977
7390	0.986	0.985	0.989	0.986
7400	0.986	0.985	0.988	0.986
7410	0.986	0.986	0.990	0.986
7420	0.986	0.985	0.990	0.986
7430	0.984	0.983	0.988	0.984
7440	0.984	0.984	0.988	0.984
7450	0.982	0.981	0.986	0.982
7460	0.984	0.984	0.988	0.984
7470	0.987	0.987	0.990	0.987
7480	0.982	0.981	0.986	0.981
7490	0.982	0.982	0.986	0.982
7500	0.984	0.983	0.987	0.983
7510	0.984	0.984	0.988	0.984
7520	0.984	0.984	0.988	0.984
7530	0.984	0.983	0.989	0.984
7540	0.986	0.985	0.989	0.986
7550	0.985	0.985	0.987	0.985
7560	0.986	0.986	0.990	0.986
7570	0.986	0.985	0.989	0.986
7580	0.988	0.988	0.992	0.988
7590	0.986	0.985	0.991	0.986
7600	0.984	0.983	0.986	0.984
7610	0.984	0.983	0.986	0.984
7620	0.982	0.982	0.986	0.982
7630	0.981	0.980	0.985	0.981
7640	0.981	0.980	0.987	0.981
7650	0.986	0.985	0.990	0.986
7660	0.984	0.983	0.988	0.984
7670	0.987	0.987	0.990	0.987
7680	0.987	0.987	0.990	0.987
7690	0.984	0.983	0.987	0.984
7700	0.986	0.986	0.989	0.986
7710	0.982	0.982	0.986	0.982
7720	0.984	0.984	0.988	0.984
7730	0.982	0.982	0.987	0.982
7740	0.982	0.981	0.986	0.982
7750	0.986	0.986	0.990	0.986
7760	0.983	0.983	0.986	0.983
7770	0.986	0.985	0.989	0.986
7780	0.984	0.984	0.988	0.984
7790	0.984	0.983	0.987	0.984

7800	0.984	0.984	0.988	0.984
7810	0.983	0.983	0.988	0.983
7820	0.986	0.985	0.990	0.986
7830	0.985	0.985	0.989	0.985
7840	0.984	0.983	0.988	0.984
7850	0.984	0.984	0.988	0.984
7860	0.982	0.981	0.985	0.982
7870	0.984	0.983	0.988	0.984
7880	0.984	0.984	0.989	0.984
7890	0.985	0.985	0.988	0.985
7900	0.986	0.986	0.990	0.986
7910	0.983	0.983	0.987	0.983
7920	0.983	0.983	0.987	0.983
7930	0.985	0.985	0.989	0.985
7940	0.982	0.981	0.987	0.981
7950	0.983	0.983	0.988	0.983
7960	0.982	0.982	0.987	0.982
7970	0.984	0.983	0.988	0.984
7980	0.984	0.984	0.988	0.984
7990	0.988	0.988	0.991	0.988
8000	0.984	0.984	0.988	0.984
8010	0.982	0.982	0.986	0.982
8020	0.984	0.984	0.989	0.984
8030	0.985	0.985	0.988	0.985
8040	0.983	0.983	0.988	0.983
8050	0.982	0.981	0.986	0.982
8060	0.985	0.985	0.988	0.985
8070	0.982	0.982	0.988	0.982
8080	0.980	0.980	0.984	0.980
8090	0.986	0.985	0.991	0.986
8100	0.984	0.984	0.989	0.984
8110	0.985	0.985	0.987	0.985
8120	0.985	0.985	0.989	0.985
8130	0.986	0.985	0.988	0.986
8140	0.982	0.981	0.984	0.982
8150	0.984	0.984	0.988	0.984
8160	0.984	0.983	0.988	0.984
8170	0.983	0.983	0.988	0.983
8180	0.984	0.984	0.988	0.984
8190	0.982	0.981	0.987	0.981
8200	0.983	0.983	0.986	0.983
8210	0.986	0.986	0.989	0.986
8220	0.984	0.984	0.989	0.984

8230	0.984	0.983	0.988	0.984
8240	0.982	0.981	0.987	0.982
8250	0.984	0.984	0.987	0.984
8260	0.984	0.983	0.987	0.984
8270	0.983	0.983	0.989	0.983
8280	0.982	0.981	0.986	0.981
8290	0.981	0.980	0.987	0.981
8300	0.982	0.981	0.987	0.981
8310	0.984	0.984	0.987	0.984
8320	0.983	0.983	0.988	0.983
8330	0.984	0.983	0.987	0.984
8340	0.982	0.981	0.986	0.982
8350	0.982	0.981	0.987	0.982
8360	0.982	0.982	0.987	0.982
8370	0.985	0.985	0.988	0.985
8380	0.987	0.987	0.990	0.987
8390	0.984	0.984	0.988	0.984
8400	0.985	0.985	0.988	0.985
8410	0.982	0.981	0.986	0.982
8420	0.982	0.981	0.986	0.982
8430	0.986	0.985	0.989	0.986
8440	0.984	0.984	0.989	0.984
8450	0.982	0.982	0.988	0.982
8460	0.984	0.984	0.988	0.984
8470	0.983	0.983	0.988	0.983
8480	0.982	0.982	0.987	0.982
8490	0.984	0.983	0.988	0.984
8500	0.983	0.983	0.986	0.983
8510	0.984	0.983	0.988	0.984
8520	0.984	0.983	0.987	0.984
8530	0.982	0.982	0.987	0.982
8540	0.985	0.985	0.988	0.985
8550	0.985	0.985	0.988	0.985
8560	0.983	0.983	0.987	0.983
8570	0.984	0.984	0.989	0.984
8580	0.984	0.984	0.988	0.984
8590	0.987	0.987	0.991	0.987
8600	0.980	0.980	0.986	0.980
8610	0.982	0.982	0.987	0.982
8620	0.985	0.985	0.989	0.985
8630	0.982	0.982	0.987	0.982
8640	0.982	0.982	0.987	0.982
8650	0.983	0.983	0.987	0.983

8660	0.985	0.985	0.989	0.985
8670	0.982	0.982	0.987	0.982
8680	0.982	0.982	0.986	0.982
8690	0.986	0.985	0.989	0.986
8700	0.983	0.983	0.988	0.983
8710	0.980	0.980	0.986	0.980
8720	0.986	0.985	0.990	0.986
8730	0.982	0.981	0.987	0.982
8740	0.983	0.983	0.987	0.983
8750	0.983	0.983	0.987	0.983
8760	0.984	0.984	0.988	0.984
8770	0.982	0.982	0.988	0.982
8780	0.984	0.983	0.988	0.984
8790	0.982	0.982	0.986	0.982
8800	0.984	0.984	0.989	0.984
8810	0.985	0.985	0.989	0.985
8820	0.983	0.983	0.989	0.983
8830	0.984	0.983	0.987	0.984
8840	0.982	0.981	0.986	0.982
8850	0.983	0.983	0.987	0.983
8860	0.982	0.982	0.988	0.982
8870	0.985	0.985	0.989	0.985
8880	0.982	0.981	0.986	0.981
8890	0.984	0.984	0.990	0.984
8900	0.983	0.983	0.988	0.983
8910	0.984	0.983	0.987	0.984
8920	0.980	0.980	0.985	0.980
8930	0.985	0.985	0.990	0.985
8940	0.984	0.983	0.988	0.984
8950	0.980	0.980	0.985	0.980

(4) IFS results with DT on the LightGBM feature list

**Number of features**	**ACC**	**MCC**	**Macro_F1**	**Weighted_F1**
10	0.580	0.570	0.613	0.575
20	0.695	0.687	0.711	0.697
30	0.740	0.733	0.746	0.740
40	0.749	0.743	0.760	0.751
50	0.766	0.760	0.778	0.765
60	0.750	0.744	0.767	0.747
70	0.770	0.764	0.777	0.769
80	0.773	0.767	0.789	0.773
90	0.794	0.788	0.796	0.795
100	0.783	0.777	0.795	0.782

110	0.791	0.786	0.799	0.790
120	0.784	0.778	0.783	0.784
130	0.807	0.802	0.806	0.806
140	0.798	0.793	0.804	0.798
150	0.797	0.791	0.812	0.796
160	0.796	0.791	0.810	0.796
170	0.791	0.785	0.798	0.790
180	0.809	0.804	0.815	0.809
190	0.796	0.790	0.804	0.796
200	0.811	0.806	0.822	0.811
210	0.816	0.811	0.817	0.816
220	0.805	0.800	0.810	0.806
230	0.795	0.790	0.802	0.794
240	0.824	0.819	0.836	0.825
250	0.827	0.822	0.839	0.827
260	0.818	0.813	0.829	0.818
270	0.813	0.808	0.828	0.813
280	0.822	0.817	0.828	0.823
290	0.818	0.813	0.824	0.819
300	0.817	0.812	0.817	0.818
310	0.832	0.828	0.841	0.830
320	0.832	0.827	0.836	0.831
330	0.819	0.814	0.821	0.818
340	0.824	0.820	0.833	0.824
350	0.816	0.811	0.813	0.816
360	0.813	0.808	0.821	0.814
370	0.836	0.831	0.841	0.837
380	0.823	0.818	0.822	0.824
390	0.817	0.813	0.822	0.817
400	0.836	0.831	0.841	0.837
410	0.834	0.830	0.829	0.834
420	0.823	0.819	0.824	0.824
430	0.829	0.824	0.822	0.830
440	0.832	0.827	0.822	0.832
450	0.833	0.828	0.841	0.834
460	0.833	0.828	0.830	0.834
470	0.839	0.835	0.835	0.838
480	0.826	0.821	0.825	0.826
490	0.822	0.817	0.821	0.821
500	0.845	0.841	0.842	0.846
510	0.827	0.822	0.839	0.827
520	0.830	0.825	0.840	0.831
530	0.834	0.829	0.838	0.835

540	0.828	0.823	0.834	0.828
550	0.832	0.828	0.836	0.833
560	0.823	0.819	0.823	0.824
570	0.819	0.814	0.826	0.819
580	0.828	0.823	0.838	0.827
590	0.820	0.815	0.832	0.821
600	0.832	0.828	0.830	0.834
610	0.833	0.828	0.835	0.833
620	0.840	0.836	0.833	0.841
630	0.821	0.817	0.826	0.822
640	0.825	0.820	0.826	0.826
650	0.842	0.838	0.839	0.843
660	0.849	0.845	0.857	0.851
670	0.847	0.842	0.849	0.848
680	0.840	0.836	0.833	0.842
690	0.821	0.816	0.818	0.821
700	0.826	0.821	0.827	0.825
710	0.853	0.849	0.849	0.853
720	0.836	0.832	0.831	0.838
730	0.829	0.824	0.818	0.828
740	0.831	0.826	0.827	0.830
750	0.846	0.842	0.847	0.848
760	0.837	0.833	0.830	0.837
770	0.844	0.840	0.838	0.845
780	0.853	0.849	0.864	0.852
790	0.858	0.854	0.856	0.857
800	0.861	0.857	0.857	0.861
810	0.834	0.830	0.828	0.836
820	0.840	0.835	0.835	0.840
830	0.848	0.844	0.848	0.848
840	0.842	0.837	0.833	0.841
850	0.845	0.841	0.835	0.844
860	0.852	0.848	0.861	0.853
870	0.853	0.849	0.851	0.854
880	0.826	0.821	0.821	0.825
890	0.843	0.839	0.829	0.843
900	0.847	0.843	0.846	0.847
910	0.836	0.831	0.827	0.836
920	0.849	0.845	0.846	0.850
930	0.846	0.842	0.843	0.847
940	0.836	0.832	0.830	0.837
950	0.842	0.837	0.833	0.841
960	0.840	0.835	0.843	0.839

970	0.830	0.826	0.829	0.829
980	0.836	0.831	0.831	0.838
990	0.837	0.833	0.834	0.837
1000	0.846	0.842	0.844	0.846
1010	0.849	0.845	0.849	0.849
1020	0.859	0.856	0.856	0.859
1030	0.855	0.851	0.864	0.855
1040	0.851	0.847	0.841	0.852
1050	0.858	0.854	0.856	0.858
1060	0.836	0.832	0.832	0.837
1070	0.850	0.846	0.853	0.849
1080	0.830	0.825	0.831	0.832
1090	0.850	0.846	0.853	0.851
1100	0.844	0.840	0.837	0.845
1110	0.833	0.828	0.833	0.833
1120	0.844	0.840	0.842	0.845
1130	0.845	0.841	0.847	0.845
1140	0.846	0.842	0.836	0.848
1150	0.845	0.840	0.835	0.846
1160	0.842	0.838	0.845	0.842
1170	0.853	0.849	0.851	0.851
1180	0.849	0.844	0.850	0.847
1190	0.836	0.832	0.834	0.837
1200	0.843	0.839	0.832	0.844
1210	0.854	0.850	0.857	0.853
1220	0.868	0.864	0.868	0.868
1230	0.847	0.843	0.840	0.847
1240	0.849	0.844	0.848	0.848
1250	0.850	0.846	0.844	0.850
1260	0.832	0.828	0.836	0.833
1270	0.824	0.819	0.826	0.824
1280	0.847	0.842	0.852	0.848
1290	0.848	0.844	0.840	0.847
1300	0.842	0.838	0.835	0.843
1310	0.841	0.837	0.840	0.841
1320	0.845	0.841	0.837	0.846
1330	0.855	0.851	0.850	0.856
1340	0.839	0.835	0.840	0.839
1350	0.838	0.834	0.834	0.840
1360	0.843	0.839	0.839	0.844
1370	0.834	0.830	0.832	0.834
1380	0.841	0.837	0.847	0.842
1390	0.853	0.849	0.853	0.854

1400	0.837	0.833	0.839	0.837
1410	0.846	0.842	0.852	0.845
1420	0.846	0.842	0.843	0.847
1430	0.834	0.829	0.830	0.834
1440	0.842	0.838	0.843	0.841
1450	0.851	0.847	0.852	0.852
1460	0.845	0.840	0.832	0.846
1470	0.843	0.839	0.840	0.844
1480	0.855	0.851	0.847	0.855
1490	0.847	0.843	0.851	0.849
1500	0.847	0.842	0.842	0.847
1510	0.830	0.825	0.826	0.832
1520	0.849	0.845	0.848	0.850
1530	0.831	0.826	0.824	0.832
1540	0.838	0.833	0.825	0.839
1550	0.850	0.846	0.840	0.849
1560	0.832	0.828	0.826	0.832
1570	0.837	0.833	0.826	0.838
1580	0.846	0.842	0.842	0.845
1590	0.849	0.844	0.850	0.850
1600	0.841	0.837	0.834	0.843
1610	0.843	0.839	0.837	0.844
1620	0.849	0.844	0.848	0.847
1630	0.841	0.837	0.840	0.841
1640	0.838	0.834	0.833	0.838
1650	0.846	0.842	0.849	0.847
1660	0.846	0.842	0.848	0.847
1670	0.842	0.838	0.834	0.843
1680	0.853	0.849	0.852	0.853
1690	0.851	0.846	0.848	0.851
1700	0.842	0.838	0.844	0.844
1710	0.840	0.836	0.836	0.842
1720	0.856	0.852	0.858	0.858
1730	0.848	0.844	0.848	0.846
1740	0.855	0.851	0.853	0.855
1750	0.849	0.845	0.858	0.849
1760	0.858	0.854	0.848	0.860
1770	0.855	0.851	0.858	0.855
1780	0.848	0.844	0.847	0.847
1790	0.859	0.855	0.853	0.858
1800	0.853	0.849	0.840	0.854
1810	0.838	0.834	0.833	0.840
1820	0.824	0.819	0.818	0.822

1830	0.830	0.826	0.828	0.830
1840	0.843	0.839	0.837	0.844
1850	0.860	0.856	0.856	0.859
1860	0.847	0.843	0.842	0.846
1870	0.845	0.840	0.841	0.844
1880	0.852	0.848	0.845	0.853
1890	0.832	0.828	0.837	0.833
1900	0.845	0.841	0.844	0.844
1910	0.856	0.852	0.846	0.857
1920	0.847	0.842	0.835	0.848
1930	0.849	0.844	0.844	0.848
1940	0.844	0.840	0.844	0.844
1950	0.841	0.837	0.836	0.841
1960	0.840	0.835	0.833	0.841
1970	0.855	0.852	0.842	0.856
1980	0.860	0.856	0.853	0.860
1990	0.845	0.840	0.832	0.845
2000	0.855	0.851	0.850	0.855
2010	0.842	0.837	0.840	0.842
2020	0.839	0.835	0.836	0.841
2030	0.838	0.833	0.840	0.838
2040	0.851	0.847	0.839	0.850
2050	0.838	0.833	0.827	0.838
2060	0.842	0.838	0.838	0.843
2070	0.851	0.847	0.849	0.852
2080	0.840	0.836	0.823	0.841
2090	0.836	0.832	0.826	0.837
2100	0.849	0.844	0.855	0.849
2110	0.856	0.852	0.855	0.858
2120	0.844	0.840	0.835	0.844
2130	0.856	0.852	0.855	0.856
2140	0.853	0.849	0.846	0.853
2150	0.848	0.844	0.843	0.849
2160	0.828	0.823	0.820	0.828
2170	0.836	0.832	0.829	0.836
2180	0.848	0.844	0.838	0.849
2190	0.853	0.849	0.848	0.853
2200	0.837	0.832	0.839	0.838
2210	0.855	0.851	0.851	0.857
2220	0.842	0.838	0.843	0.840
2230	0.843	0.839	0.844	0.843
2240	0.858	0.854	0.857	0.858
2250	0.842	0.838	0.836	0.843

2260	0.849	0.844	0.844	0.848
2270	0.850	0.846	0.845	0.850
2280	0.852	0.848	0.845	0.853
2290	0.845	0.840	0.829	0.844
2300	0.842	0.837	0.837	0.843
2310	0.848	0.844	0.849	0.849
2320	0.845	0.840	0.837	0.845
2330	0.842	0.837	0.835	0.843
2340	0.851	0.847	0.840	0.853
2350	0.847	0.843	0.835	0.848
2360	0.849	0.845	0.835	0.849
2370	0.847	0.842	0.835	0.848
2380	0.851	0.847	0.847	0.850
2390	0.849	0.845	0.842	0.850
2400	0.849	0.845	0.843	0.851
2410	0.848	0.844	0.843	0.847
2420	0.845	0.840	0.837	0.845
2430	0.847	0.843	0.846	0.848
2440	0.857	0.853	0.857	0.858
2450	0.856	0.852	0.854	0.855
2460	0.845	0.840	0.834	0.846
2470	0.846	0.842	0.847	0.846
2480	0.853	0.849	0.850	0.852
2490	0.843	0.839	0.831	0.842
2500	0.861	0.857	0.853	0.862
2510	0.840	0.836	0.829	0.841
2520	0.837	0.833	0.830	0.839
2530	0.839	0.835	0.829	0.839
2540	0.847	0.843	0.829	0.847
2550	0.847	0.843	0.842	0.847
2560	0.864	0.860	0.859	0.863
2570	0.847	0.842	0.845	0.847
2580	0.848	0.844	0.840	0.849
2590	0.849	0.845	0.838	0.849
2600	0.857	0.853	0.849	0.857
2610	0.859	0.855	0.843	0.861
2620	0.858	0.854	0.856	0.858
2630	0.852	0.848	0.846	0.851
2640	0.847	0.843	0.839	0.849
2650	0.842	0.838	0.836	0.842
2660	0.834	0.829	0.828	0.833
2670	0.850	0.846	0.852	0.851
2680	0.853	0.849	0.849	0.854

2690	0.852	0.848	0.849	0.853
2700	0.846	0.842	0.845	0.846
2710	0.851	0.847	0.846	0.852
2720	0.857	0.853	0.859	0.858
2730	0.845	0.840	0.830	0.845
2740	0.845	0.841	0.837	0.844
2750	0.848	0.844	0.841	0.847
2760	0.849	0.845	0.843	0.848
2770	0.856	0.852	0.847	0.855
2780	0.842	0.838	0.834	0.844
2790	0.836	0.831	0.827	0.836
2800	0.845	0.840	0.834	0.845
2810	0.836	0.831	0.832	0.838
2820	0.834	0.829	0.822	0.834
2830	0.845	0.841	0.848	0.846
2840	0.856	0.852	0.841	0.857
2850	0.840	0.835	0.831	0.839
2860	0.841	0.837	0.826	0.842
2870	0.850	0.846	0.843	0.852
2880	0.845	0.841	0.835	0.846
2890	0.840	0.836	0.844	0.840
2900	0.833	0.828	0.827	0.833
2910	0.841	0.837	0.831	0.841
2920	0.855	0.851	0.842	0.856
2930	0.844	0.840	0.831	0.844
2940	0.855	0.851	0.842	0.856
2950	0.849	0.845	0.844	0.850
2960	0.857	0.853	0.849	0.858
2970	0.842	0.837	0.832	0.841
2980	0.839	0.835	0.828	0.839
2990	0.844	0.840	0.838	0.844
3000	0.826	0.821	0.819	0.826
3010	0.839	0.835	0.837	0.839
3020	0.849	0.845	0.846	0.850
3030	0.834	0.829	0.832	0.834
3040	0.850	0.846	0.845	0.851
3050	0.859	0.856	0.855	0.861
3060	0.839	0.835	0.836	0.840
3070	0.851	0.847	0.847	0.851
3080	0.852	0.848	0.838	0.853
3090	0.849	0.845	0.832	0.849
3100	0.830	0.826	0.825	0.831
3110	0.842	0.838	0.834	0.843

3120	0.832	0.828	0.826	0.832
3130	0.840	0.835	0.828	0.840
3140	0.846	0.842	0.838	0.846
3150	0.845	0.841	0.844	0.846
3160	0.831	0.826	0.819	0.832
3170	0.849	0.845	0.840	0.849
3180	0.840	0.835	0.838	0.840
3190	0.857	0.853	0.855	0.858
3200	0.847	0.842	0.843	0.847
3210	0.835	0.830	0.826	0.835
3220	0.835	0.830	0.836	0.834
3230	0.844	0.840	0.836	0.843
3240	0.840	0.836	0.834	0.841
3250	0.847	0.843	0.846	0.846
3260	0.851	0.847	0.841	0.850
3270	0.847	0.842	0.840	0.845
3280	0.860	0.856	0.861	0.860
3290	0.845	0.840	0.828	0.846
3300	0.846	0.842	0.847	0.845
3310	0.845	0.841	0.841	0.846
3320	0.844	0.840	0.838	0.844
3330	0.851	0.846	0.840	0.850
3340	0.843	0.839	0.833	0.845
3350	0.834	0.829	0.826	0.836
3360	0.844	0.840	0.839	0.845
3370	0.850	0.846	0.846	0.851
3380	0.856	0.852	0.841	0.857
3390	0.840	0.835	0.823	0.840
3400	0.838	0.834	0.836	0.839
3410	0.848	0.844	0.841	0.848
3420	0.839	0.835	0.842	0.841
3430	0.824	0.820	0.811	0.824
3440	0.851	0.847	0.835	0.852
3450	0.838	0.833	0.822	0.838
3460	0.847	0.842	0.840	0.846
3470	0.859	0.855	0.856	0.860
3480	0.849	0.844	0.848	0.849
3490	0.841	0.837	0.823	0.842
3500	0.851	0.847	0.834	0.852
3510	0.831	0.826	0.830	0.833
3520	0.837	0.833	0.829	0.836
3530	0.842	0.838	0.837	0.841
3540	0.851	0.847	0.849	0.852

3550	0.836	0.831	0.830	0.836
3560	0.840	0.836	0.840	0.843
3570	0.823	0.819	0.822	0.824
3580	0.848	0.844	0.848	0.848
3590	0.851	0.847	0.842	0.852
3600	0.843	0.839	0.826	0.844
3610	0.859	0.855	0.851	0.860
3620	0.840	0.836	0.832	0.841
3630	0.851	0.847	0.843	0.852
3640	0.851	0.847	0.850	0.851
3650	0.846	0.842	0.835	0.846
3660	0.848	0.844	0.849	0.846
3670	0.860	0.856	0.857	0.861
3680	0.853	0.849	0.848	0.854
3690	0.847	0.843	0.844	0.848
3700	0.838	0.833	0.838	0.838
3710	0.852	0.848	0.850	0.852
3720	0.856	0.852	0.855	0.857
3730	0.823	0.819	0.816	0.824
3740	0.836	0.832	0.836	0.837
3750	0.850	0.846	0.848	0.848
3760	0.844	0.840	0.840	0.843
3770	0.853	0.849	0.845	0.853
3780	0.844	0.840	0.834	0.845
3790	0.840	0.836	0.831	0.841
3800	0.838	0.834	0.827	0.839
3810	0.843	0.839	0.838	0.845
3820	0.834	0.829	0.819	0.834
3830	0.842	0.838	0.838	0.842
3840	0.838	0.833	0.834	0.837
3850	0.836	0.831	0.826	0.837
3860	0.847	0.842	0.837	0.848
3870	0.837	0.832	0.832	0.839
3880	0.840	0.836	0.836	0.842
3890	0.841	0.837	0.837	0.840
3900	0.868	0.864	0.861	0.868
3910	0.830	0.826	0.819	0.831
3920	0.836	0.832	0.828	0.838
3930	0.838	0.834	0.822	0.838
3940	0.842	0.838	0.837	0.842
3950	0.840	0.835	0.835	0.840
3960	0.846	0.842	0.841	0.846
3970	0.840	0.836	0.841	0.840

3980	0.849	0.845	0.841	0.850
3990	0.833	0.828	0.826	0.833
4000	0.851	0.847	0.843	0.850
4010	0.851	0.847	0.849	0.852
4020	0.840	0.835	0.832	0.841
4030	0.848	0.844	0.860	0.847
4040	0.860	0.856	0.845	0.861
4050	0.836	0.832	0.835	0.838
4060	0.846	0.842	0.829	0.846
4070	0.851	0.847	0.839	0.853
4080	0.839	0.835	0.827	0.840
4090	0.855	0.851	0.846	0.854
4100	0.847	0.842	0.851	0.847
4110	0.845	0.841	0.832	0.846
4120	0.830	0.825	0.829	0.831
4130	0.833	0.829	0.831	0.834
4140	0.849	0.844	0.843	0.848
4150	0.834	0.830	0.824	0.835
4160	0.843	0.839	0.838	0.844
4170	0.843	0.839	0.834	0.843
4180	0.839	0.835	0.834	0.840
4190	0.827	0.822	0.831	0.827
4200	0.851	0.847	0.846	0.850
4210	0.847	0.843	0.846	0.848
4220	0.844	0.840	0.832	0.844
4230	0.836	0.831	0.830	0.837
4240	0.844	0.840	0.841	0.844
4250	0.839	0.835	0.833	0.838
4260	0.838	0.834	0.833	0.839
4270	0.841	0.837	0.831	0.840
4280	0.844	0.840	0.839	0.844
4290	0.841	0.837	0.838	0.842
4300	0.840	0.836	0.841	0.842
4310	0.845	0.841	0.842	0.846
4320	0.843	0.839	0.833	0.844
4330	0.828	0.823	0.810	0.829
4340	0.838	0.834	0.834	0.838
4350	0.832	0.827	0.826	0.831
4360	0.845	0.840	0.838	0.845
4370	0.847	0.842	0.845	0.845
4380	0.842	0.838	0.834	0.841
4390	0.853	0.849	0.844	0.853
4400	0.862	0.858	0.856	0.863

4410	0.850	0.846	0.849	0.850
4420	0.840	0.836	0.839	0.842
4430	0.836	0.831	0.828	0.836
4440	0.850	0.846	0.851	0.849
4450	0.839	0.835	0.842	0.840
4460	0.846	0.842	0.846	0.845
4470	0.838	0.833	0.841	0.839
4480	0.842	0.838	0.837	0.841
4490	0.832	0.828	0.825	0.835
4500	0.834	0.829	0.823	0.835
4510	0.851	0.847	0.843	0.853
4520	0.848	0.844	0.843	0.847
4530	0.844	0.840	0.841	0.844
4540	0.847	0.843	0.846	0.847
4550	0.836	0.831	0.832	0.834
4560	0.841	0.837	0.841	0.841
4570	0.828	0.823	0.824	0.828
4580	0.841	0.837	0.835	0.841
4590	0.821	0.816	0.816	0.820
4600	0.839	0.835	0.841	0.840
4610	0.847	0.843	0.844	0.848
4620	0.845	0.841	0.848	0.846
4630	0.845	0.841	0.841	0.847
4640	0.840	0.836	0.836	0.841
4650	0.838	0.833	0.828	0.839
4660	0.832	0.828	0.823	0.833
4670	0.845	0.840	0.839	0.846
4680	0.848	0.844	0.841	0.849
4690	0.836	0.832	0.824	0.837
4700	0.839	0.835	0.825	0.840
4710	0.840	0.836	0.830	0.843
4720	0.842	0.837	0.841	0.841
4730	0.837	0.833	0.835	0.839
4740	0.841	0.837	0.845	0.841
4750	0.838	0.834	0.840	0.838
4760	0.832	0.827	0.831	0.830
4770	0.828	0.823	0.827	0.829
4780	0.838	0.834	0.829	0.840
4790	0.836	0.832	0.825	0.837
4800	0.840	0.836	0.831	0.841
4810	0.834	0.830	0.827	0.834
4820	0.842	0.838	0.834	0.843
4830	0.837	0.833	0.826	0.837

4840	0.831	0.826	0.822	0.831
4850	0.842	0.838	0.839	0.842
4860	0.828	0.823	0.829	0.828
4870	0.836	0.831	0.841	0.834
4880	0.831	0.826	0.818	0.833
4890	0.846	0.842	0.844	0.845
4900	0.850	0.846	0.843	0.850
4910	0.844	0.840	0.840	0.844
4920	0.859	0.855	0.857	0.858
4930	0.841	0.837	0.829	0.842
4940	0.847	0.843	0.846	0.849
4950	0.841	0.837	0.836	0.842
4960	0.843	0.839	0.841	0.844
4970	0.845	0.840	0.840	0.845
4980	0.837	0.833	0.826	0.838
4990	0.815	0.810	0.808	0.815
5000	0.841	0.837	0.837	0.841
5010	0.830	0.826	0.823	0.831
5020	0.830	0.826	0.822	0.832
5030	0.836	0.831	0.830	0.836
5040	0.838	0.833	0.823	0.840
5050	0.836	0.832	0.832	0.837
5060	0.836	0.831	0.828	0.837
5070	0.851	0.847	0.844	0.852
5080	0.835	0.830	0.833	0.834
5090	0.845	0.840	0.843	0.846
5100	0.849	0.844	0.844	0.849
5110	0.856	0.852	0.854	0.856
5120	0.845	0.841	0.834	0.846
5130	0.844	0.840	0.840	0.844
5140	0.830	0.826	0.824	0.832
5150	0.842	0.838	0.829	0.843
5160	0.836	0.832	0.817	0.838
5170	0.837	0.833	0.837	0.837
5180	0.845	0.840	0.843	0.845
5190	0.866	0.862	0.865	0.865
5200	0.841	0.837	0.851	0.841
5210	0.838	0.834	0.831	0.837
5220	0.847	0.842	0.844	0.847
5230	0.841	0.837	0.833	0.842
5240	0.846	0.842	0.842	0.845
5250	0.845	0.840	0.836	0.844
5260	0.841	0.837	0.834	0.841

5270	0.837	0.833	0.827	0.840
5280	0.838	0.834	0.837	0.839
5290	0.841	0.837	0.841	0.840
5300	0.834	0.830	0.827	0.836
5310	0.843	0.839	0.835	0.843
5320	0.840	0.836	0.831	0.840
5330	0.856	0.852	0.850	0.858
5340	0.844	0.840	0.838	0.844
5350	0.841	0.837	0.840	0.840
5360	0.832	0.828	0.828	0.832
5370	0.835	0.831	0.825	0.835
5380	0.843	0.839	0.836	0.845
5390	0.857	0.853	0.848	0.857
5400	0.838	0.834	0.829	0.838
5410	0.847	0.842	0.841	0.847
5420	0.842	0.838	0.829	0.843
5430	0.830	0.826	0.828	0.831
5440	0.844	0.839	0.840	0.843
5450	0.854	0.850	0.851	0.855
5460	0.836	0.831	0.833	0.838
5470	0.830	0.826	0.818	0.830
5480	0.857	0.853	0.854	0.855
5490	0.836	0.831	0.828	0.837
5500	0.842	0.838	0.846	0.842
5510	0.837	0.833	0.823	0.838
5520	0.835	0.830	0.835	0.836
5530	0.843	0.839	0.836	0.844
5540	0.842	0.838	0.837	0.842
5550	0.845	0.840	0.843	0.846
5560	0.831	0.826	0.819	0.832
5570	0.853	0.849	0.851	0.853
5580	0.854	0.850	0.854	0.854
5590	0.856	0.852	0.854	0.857
5600	0.831	0.826	0.822	0.832
5610	0.845	0.840	0.835	0.845
5620	0.851	0.847	0.841	0.852
5630	0.841	0.837	0.838	0.841
5640	0.843	0.839	0.836	0.843
5650	0.823	0.819	0.816	0.825
5660	0.842	0.838	0.838	0.844
5670	0.853	0.849	0.848	0.853
5680	0.832	0.828	0.833	0.833
5690	0.842	0.838	0.832	0.842

5700	0.833	0.829	0.826	0.835
5710	0.850	0.846	0.842	0.850
5720	0.838	0.834	0.829	0.839
5730	0.853	0.849	0.839	0.853
5740	0.842	0.837	0.844	0.842
5750	0.847	0.843	0.834	0.847
5760	0.837	0.833	0.838	0.838
5770	0.845	0.841	0.838	0.846
5780	0.845	0.841	0.836	0.845
5790	0.828	0.824	0.826	0.830
5800	0.848	0.844	0.836	0.849
5810	0.845	0.840	0.843	0.845
5820	0.832	0.827	0.820	0.832
5830	0.836	0.832	0.834	0.838
5840	0.840	0.836	0.839	0.840
5850	0.840	0.836	0.834	0.841
5860	0.845	0.840	0.835	0.845
5870	0.847	0.843	0.841	0.847
5880	0.840	0.835	0.831	0.840
5890	0.841	0.837	0.839	0.842
5900	0.845	0.841	0.846	0.847
5910	0.842	0.838	0.841	0.843
5920	0.825	0.820	0.814	0.825
5930	0.845	0.840	0.837	0.845
5940	0.834	0.830	0.830	0.835
5950	0.844	0.840	0.836	0.842
5960	0.840	0.835	0.839	0.839
5970	0.837	0.833	0.833	0.837
5980	0.854	0.850	0.846	0.854
5990	0.839	0.835	0.840	0.839
6000	0.844	0.840	0.840	0.845
6010	0.857	0.853	0.840	0.857
6020	0.832	0.828	0.824	0.832
6030	0.830	0.826	0.823	0.831
6040	0.834	0.829	0.829	0.834
6050	0.840	0.835	0.829	0.841
6060	0.837	0.833	0.837	0.837
6070	0.851	0.847	0.849	0.851
6080	0.842	0.838	0.836	0.841
6090	0.837	0.833	0.834	0.838
6100	0.847	0.842	0.835	0.848
6110	0.838	0.834	0.832	0.838
6120	0.840	0.836	0.836	0.841

6130	0.847	0.842	0.842	0.848
6140	0.838	0.834	0.838	0.839
6150	0.855	0.851	0.850	0.855
6160	0.838	0.833	0.839	0.837
6170	0.847	0.842	0.845	0.847
6180	0.835	0.831	0.829	0.836
6190	0.847	0.842	0.849	0.849
6200	0.838	0.833	0.836	0.838
6210	0.834	0.829	0.832	0.836
6220	0.840	0.836	0.830	0.841
6230	0.833	0.828	0.829	0.832
6240	0.834	0.830	0.833	0.834
6250	0.827	0.822	0.824	0.827
6260	0.849	0.844	0.849	0.849
6270	0.853	0.849	0.847	0.853
6280	0.844	0.840	0.837	0.844
6290	0.855	0.851	0.850	0.856
6300	0.834	0.830	0.835	0.835
6310	0.844	0.840	0.842	0.845
6320	0.851	0.847	0.846	0.851
6330	0.835	0.831	0.847	0.836
6340	0.840	0.836	0.840	0.841
6350	0.839	0.835	0.830	0.840
6360	0.837	0.833	0.827	0.837
6370	0.840	0.835	0.831	0.841
6380	0.831	0.826	0.834	0.832
6390	0.847	0.842	0.829	0.848
6400	0.819	0.815	0.820	0.822
6410	0.835	0.830	0.827	0.837
6420	0.830	0.826	0.827	0.832
6430	0.853	0.849	0.847	0.854
6440	0.848	0.844	0.847	0.847
6450	0.846	0.842	0.843	0.846
6460	0.847	0.843	0.843	0.848
6470	0.845	0.840	0.843	0.844
6480	0.842	0.837	0.839	0.842
6490	0.847	0.843	0.843	0.847
6500	0.849	0.845	0.849	0.848
6510	0.849	0.844	0.836	0.849
6520	0.836	0.832	0.821	0.836
6530	0.847	0.843	0.835	0.847
6540	0.851	0.847	0.848	0.851
6550	0.851	0.847	0.850	0.851

6560	0.841	0.837	0.832	0.841
6570	0.849	0.844	0.847	0.850
6580	0.858	0.854	0.855	0.858
6590	0.844	0.840	0.841	0.844
6600	0.847	0.843	0.837	0.848
6610	0.848	0.844	0.851	0.849
6620	0.830	0.825	0.834	0.830
6630	0.840	0.836	0.837	0.841
6640	0.839	0.835	0.841	0.840
6650	0.830	0.826	0.824	0.831
6660	0.849	0.845	0.851	0.849
6670	0.829	0.824	0.811	0.830
6680	0.832	0.827	0.829	0.830
6690	0.839	0.835	0.833	0.839
6700	0.838	0.833	0.832	0.838
6710	0.836	0.831	0.827	0.836
6720	0.837	0.833	0.834	0.837
6730	0.834	0.829	0.833	0.834
6740	0.836	0.832	0.836	0.838
6750	0.841	0.837	0.835	0.841
6760	0.855	0.851	0.862	0.855
6770	0.849	0.845	0.836	0.850
6780	0.823	0.819	0.816	0.825
6790	0.833	0.829	0.831	0.833
6800	0.838	0.833	0.830	0.839
6810	0.838	0.834	0.836	0.839
6820	0.836	0.831	0.829	0.836
6830	0.826	0.821	0.828	0.826
6840	0.834	0.829	0.831	0.834
6850	0.840	0.835	0.835	0.839
6860	0.828	0.824	0.825	0.828
6870	0.837	0.833	0.829	0.838
6880	0.832	0.828	0.830	0.833
6890	0.840	0.836	0.839	0.840
6900	0.841	0.837	0.837	0.841
6910	0.849	0.845	0.840	0.850
6920	0.840	0.835	0.836	0.840
6930	0.842	0.838	0.844	0.842
6940	0.836	0.832	0.827	0.836
6950	0.833	0.829	0.830	0.835
6960	0.830	0.825	0.835	0.830
6970	0.832	0.828	0.826	0.833
6980	0.844	0.840	0.836	0.844

6990	0.837	0.833	0.840	0.836
7000	0.838	0.833	0.825	0.839
7010	0.836	0.832	0.834	0.837
7020	0.837	0.833	0.832	0.837
7030	0.841	0.837	0.832	0.844
7040	0.849	0.845	0.840	0.849
7050	0.849	0.845	0.840	0.850
7060	0.842	0.838	0.842	0.842
7070	0.846	0.842	0.848	0.845
7080	0.854	0.850	0.852	0.856
7090	0.836	0.831	0.825	0.837
7100	0.833	0.828	0.832	0.833
7110	0.846	0.842	0.838	0.847
7120	0.848	0.844	0.841	0.849
7130	0.836	0.831	0.828	0.835
7140	0.849	0.845	0.844	0.851
7150	0.834	0.829	0.831	0.835
7160	0.857	0.853	0.863	0.857
7170	0.847	0.843	0.850	0.847
7180	0.837	0.833	0.837	0.838
7190	0.836	0.832	0.828	0.837
7200	0.837	0.833	0.833	0.838
7210	0.831	0.826	0.829	0.831
7220	0.847	0.843	0.843	0.848
7230	0.846	0.842	0.839	0.847
7240	0.838	0.834	0.837	0.839
7250	0.842	0.838	0.826	0.844
7260	0.844	0.840	0.837	0.844
7270	0.850	0.846	0.849	0.850
7280	0.834	0.829	0.821	0.835
7290	0.834	0.829	0.832	0.835
7300	0.834	0.829	0.826	0.835
7310	0.842	0.838	0.833	0.844
7320	0.837	0.833	0.832	0.837
7330	0.838	0.833	0.832	0.840
7340	0.851	0.847	0.846	0.851
7350	0.836	0.832	0.834	0.836
7360	0.854	0.850	0.851	0.855
7370	0.838	0.834	0.831	0.841
7380	0.845	0.840	0.839	0.846
7390	0.846	0.842	0.848	0.846
7400	0.838	0.834	0.832	0.839
7410	0.845	0.841	0.848	0.846

7420	0.823	0.818	0.815	0.826
7430	0.844	0.840	0.837	0.845
7440	0.844	0.840	0.840	0.845
7450	0.828	0.824	0.827	0.827
7460	0.845	0.841	0.847	0.845
7470	0.839	0.835	0.843	0.839
7480	0.827	0.822	0.819	0.827
7490	0.841	0.837	0.834	0.842
7500	0.849	0.845	0.842	0.850
7510	0.831	0.826	0.819	0.831
7520	0.836	0.831	0.826	0.836
7530	0.834	0.830	0.836	0.835
7540	0.845	0.841	0.835	0.845
7550	0.833	0.829	0.825	0.833
7560	0.836	0.832	0.832	0.838
7570	0.853	0.849	0.843	0.853
7580	0.833	0.829	0.826	0.835
7590	0.847	0.842	0.842	0.848
7600	0.834	0.830	0.826	0.835
7610	0.855	0.852	0.852	0.855
7620	0.835	0.831	0.831	0.837
7630	0.856	0.852	0.861	0.855
7640	0.836	0.832	0.835	0.836
7650	0.845	0.841	0.844	0.844
7660	0.840	0.835	0.838	0.841
7670	0.835	0.831	0.825	0.836
7680	0.837	0.833	0.831	0.837
7690	0.845	0.841	0.834	0.846
7700	0.834	0.829	0.832	0.835
7710	0.828	0.823	0.825	0.829
7720	0.840	0.835	0.825	0.841
7730	0.843	0.839	0.841	0.844
7740	0.823	0.818	0.827	0.822
7750	0.838	0.834	0.837	0.840
7760	0.833	0.828	0.829	0.833
7770	0.830	0.825	0.821	0.830
7780	0.823	0.818	0.814	0.825
7790	0.837	0.833	0.829	0.837
7800	0.843	0.839	0.844	0.844
7810	0.841	0.837	0.827	0.841
7820	0.839	0.835	0.826	0.841
7830	0.842	0.838	0.839	0.843
7840	0.849	0.844	0.844	0.848

7850	0.840	0.836	0.828	0.842
7860	0.841	0.837	0.832	0.840
7870	0.842	0.838	0.833	0.844
7880	0.834	0.830	0.825	0.835
7890	0.836	0.831	0.837	0.836
7900	0.834	0.830	0.831	0.836
7910	0.835	0.831	0.836	0.834
7920	0.838	0.834	0.834	0.838
7930	0.832	0.827	0.815	0.832
7940	0.842	0.838	0.831	0.843
7950	0.840	0.836	0.838	0.841
7960	0.840	0.836	0.840	0.840
7970	0.842	0.838	0.835	0.843
7980	0.846	0.842	0.846	0.845
7990	0.838	0.833	0.835	0.841
8000	0.840	0.836	0.832	0.841
8010	0.845	0.841	0.842	0.846
8020	0.843	0.839	0.836	0.843
8030	0.834	0.829	0.818	0.834
8040	0.845	0.840	0.833	0.845
8050	0.836	0.831	0.831	0.835
8060	0.827	0.822	0.826	0.829
8070	0.845	0.840	0.853	0.845
8080	0.847	0.842	0.842	0.849
8090	0.847	0.843	0.834	0.847
8100	0.842	0.838	0.832	0.844
8110	0.836	0.832	0.833	0.837
8120	0.827	0.822	0.821	0.829
8130	0.836	0.831	0.824	0.837
8140	0.838	0.833	0.836	0.839
8150	0.845	0.841	0.840	0.846
8160	0.834	0.829	0.820	0.832
8170	0.850	0.846	0.851	0.851
8180	0.838	0.833	0.839	0.839
8190	0.853	0.849	0.839	0.853
8200	0.845	0.840	0.841	0.845
8210	0.849	0.845	0.848	0.848
8220	0.837	0.833	0.831	0.838
8230	0.839	0.835	0.829	0.840
8240	0.834	0.829	0.831	0.834
8250	0.860	0.856	0.848	0.859
8260	0.846	0.842	0.836	0.847
8270	0.840	0.835	0.836	0.842

8280	0.847	0.843	0.835	0.847
8290	0.848	0.844	0.845	0.849
8300	0.829	0.824	0.833	0.830
8310	0.845	0.841	0.839	0.847
8320	0.838	0.834	0.833	0.838
8330	0.848	0.844	0.840	0.848
8340	0.833	0.828	0.832	0.834
8350	0.848	0.844	0.840	0.848
8360	0.847	0.842	0.842	0.846
8370	0.845	0.841	0.842	0.846
8380	0.838	0.834	0.830	0.840
8390	0.835	0.831	0.823	0.835
8400	0.833	0.829	0.824	0.834
8410	0.830	0.826	0.825	0.830
8420	0.840	0.835	0.832	0.841
8430	0.849	0.845	0.850	0.850
8440	0.833	0.828	0.823	0.833
8450	0.841	0.837	0.841	0.841
8460	0.836	0.832	0.836	0.836
8470	0.841	0.837	0.840	0.842
8480	0.842	0.838	0.841	0.842
8490	0.853	0.849	0.842	0.854
8500	0.843	0.839	0.840	0.844
8510	0.844	0.839	0.847	0.845
8520	0.849	0.844	0.831	0.849
8530	0.842	0.838	0.838	0.843
8540	0.840	0.835	0.833	0.841
8550	0.841	0.837	0.839	0.842
8560	0.842	0.838	0.842	0.840
8570	0.828	0.823	0.821	0.827
8580	0.832	0.827	0.823	0.832
8590	0.829	0.824	0.825	0.829
8600	0.836	0.831	0.827	0.835
8610	0.843	0.839	0.839	0.843
8620	0.840	0.835	0.838	0.841
8630	0.836	0.831	0.837	0.836
8640	0.847	0.842	0.840	0.847
8650	0.834	0.830	0.828	0.835
8660	0.828	0.824	0.812	0.829
8670	0.847	0.843	0.852	0.848
8680	0.848	0.844	0.847	0.847
8690	0.836	0.831	0.826	0.837
8700	0.838	0.833	0.831	0.838

8710	0.847	0.843	0.834	0.849
8720	0.845	0.841	0.838	0.845
8730	0.822	0.817	0.827	0.823
8740	0.824	0.820	0.821	0.825
8750	0.833	0.828	0.833	0.832
8760	0.834	0.830	0.829	0.836
8770	0.845	0.840	0.841	0.845
8780	0.839	0.835	0.837	0.839
8790	0.845	0.841	0.841	0.845
8800	0.839	0.835	0.833	0.839
8810	0.847	0.842	0.836	0.848
8820	0.840	0.836	0.837	0.842
8830	0.833	0.828	0.833	0.834
8840	0.832	0.828	0.822	0.834
8850	0.846	0.842	0.832	0.847
8860	0.846	0.842	0.848	0.847
8870	0.846	0.842	0.839	0.846
8880	0.828	0.824	0.818	0.829
8890	0.862	0.858	0.851	0.863
8900	0.854	0.850	0.855	0.854
8910	0.847	0.842	0.841	0.847
8920	0.830	0.826	0.834	0.832
8930	0.850	0.846	0.845	0.851
8940	0.845	0.841	0.845	0.846
8950	0.842	0.838	0.843	0.844

(5) IFS results with RF on the MCFS feature list

**Number of features**	**ACC**	**MCC**	**Macro F1**	**Weighted F1**
10	0.884	0.881	0.902	0.883
20	0.954	0.953	0.959	0.954
30	0.971	0.971	0.977	0.971
40	0.970	0.969	0.975	0.970
50	0.975	0.974	0.981	0.974
60	0.972	0.971	0.978	0.972
70	0.974	0.973	0.976	0.973
80	0.971	0.971	0.976	0.971
90	0.975	0.974	0.979	0.975
100	0.973	0.972	0.976	0.973
110	0.976	0.975	0.979	0.975
120	0.976	0.975	0.979	0.975
130	0.974	0.973	0.978	0.973
140	0.977	0.976	0.979	0.977
150	0.978	0.978	0.982	0.978

160	0.974	0.973	0.979	0.974
170	0.976	0.975	0.978	0.976
180	0.978	0.977	0.983	0.977
190	0.974	0.973	0.978	0.973
200	0.974	0.973	0.976	0.973
210	0.974	0.973	0.978	0.974
220	0.977	0.976	0.980	0.977
230	0.978	0.977	0.981	0.977
240	0.979	0.978	0.984	0.979
250	0.978	0.978	0.982	0.978
260	0.982	0.981	0.985	0.982
270	0.976	0.976	0.980	0.976
280	0.980	0.979	0.984	0.980
290	0.978	0.977	0.980	0.977
300	0.976	0.975	0.980	0.975
310	0.978	0.978	0.982	0.978
320	0.978	0.977	0.982	0.977
330	0.980	0.979	0.983	0.980
340	0.976	0.976	0.980	0.976
350	0.976	0.975	0.982	0.975
360	0.978	0.977	0.982	0.977
370	0.976	0.976	0.981	0.976
380	0.981	0.980	0.986	0.981
390	0.980	0.979	0.983	0.980
400	0.982	0.982	0.987	0.982
410	0.978	0.978	0.983	0.978
420	0.978	0.978	0.982	0.978
430	0.978	0.978	0.985	0.978
440	0.978	0.978	0.982	0.978
450	0.978	0.977	0.979	0.977
460	0.978	0.977	0.982	0.977
470	0.979	0.978	0.985	0.979
480	0.979	0.978	0.982	0.979
490	0.980	0.980	0.985	0.980
500	0.976	0.976	0.982	0.976
510	0.978	0.977	0.982	0.978
520	0.978	0.977	0.982	0.978
530	0.976	0.975	0.982	0.975
540	0.980	0.979	0.983	0.979
550	0.973	0.972	0.978	0.972
560	0.976	0.976	0.982	0.976
570	0.977	0.976	0.982	0.977
580	0.976	0.976	0.981	0.976

590	0.976	0.975	0.981	0.975
600	0.974	0.973	0.979	0.974
610	0.978	0.977	0.981	0.978
620	0.976	0.975	0.981	0.975
630	0.979	0.978	0.983	0.979
640	0.978	0.977	0.981	0.978
650	0.977	0.976	0.983	0.977
660	0.976	0.976	0.981	0.976
670	0.977	0.976	0.982	0.977
680	0.974	0.973	0.979	0.974
690	0.978	0.978	0.984	0.978
700	0.979	0.978	0.985	0.979
710	0.978	0.977	0.981	0.977
720	0.977	0.976	0.980	0.977
730	0.978	0.978	0.984	0.978
740	0.977	0.976	0.982	0.977
750	0.977	0.976	0.982	0.977
760	0.976	0.976	0.979	0.976
770	0.978	0.978	0.981	0.978
780	0.978	0.978	0.983	0.978
790	0.977	0.976	0.980	0.977
800	0.978	0.978	0.983	0.978
810	0.976	0.976	0.982	0.976
820	0.976	0.976	0.981	0.976
830	0.978	0.978	0.983	0.978
840	0.978	0.978	0.982	0.978
850	0.975	0.974	0.982	0.975
860	0.978	0.977	0.982	0.977
870	0.975	0.974	0.980	0.975
880	0.982	0.981	0.985	0.982
890	0.976	0.975	0.981	0.975
900	0.977	0.976	0.982	0.977
910	0.976	0.976	0.980	0.976
920	0.976	0.975	0.982	0.975
930	0.977	0.976	0.982	0.977
940	0.976	0.976	0.982	0.976
950	0.978	0.977	0.982	0.978
960	0.980	0.979	0.984	0.980
970	0.978	0.978	0.982	0.978
980	0.978	0.978	0.983	0.978
990	0.977	0.976	0.982	0.977
1000	0.976	0.975	0.980	0.975
1010	0.976	0.975	0.981	0.976

1020	0.980	0.980	0.986	0.980
1030	0.978	0.977	0.983	0.977
1040	0.980	0.980	0.985	0.980
1050	0.979	0.978	0.983	0.979
1060	0.982	0.982	0.986	0.982
1070	0.980	0.980	0.985	0.980
1080	0.980	0.979	0.984	0.979
1090	0.982	0.982	0.986	0.982
1100	0.976	0.976	0.981	0.976
1110	0.980	0.979	0.984	0.979
1120	0.982	0.981	0.986	0.982
1130	0.982	0.981	0.985	0.982
1140	0.980	0.979	0.982	0.980
1150	0.980	0.979	0.983	0.980
1160	0.980	0.979	0.984	0.979
1170	0.978	0.978	0.983	0.978
1180	0.978	0.978	0.982	0.978
1190	0.981	0.980	0.987	0.981
1200	0.980	0.979	0.985	0.980
1210	0.981	0.980	0.986	0.981
1220	0.978	0.978	0.982	0.978
1230	0.981	0.980	0.984	0.981
1240	0.976	0.976	0.980	0.976
1250	0.979	0.978	0.983	0.979
1260	0.984	0.983	0.987	0.984
1270	0.978	0.978	0.983	0.978
1280	0.982	0.982	0.988	0.982
1290	0.982	0.982	0.987	0.982
1300	0.976	0.976	0.982	0.976
1310	0.980	0.980	0.983	0.980
1320	0.976	0.976	0.981	0.976
1330	0.976	0.976	0.981	0.976
1340	0.980	0.980	0.984	0.980
1350	0.981	0.980	0.985	0.981
1360	0.980	0.980	0.985	0.980
1370	0.982	0.981	0.985	0.982
1380	0.980	0.979	0.983	0.979
1390	0.978	0.978	0.982	0.978
1400	0.982	0.981	0.985	0.982
1410	0.981	0.980	0.984	0.981
1420	0.982	0.981	0.985	0.981
1430	0.979	0.978	0.983	0.979
1440	0.980	0.979	0.984	0.980

1450	0.981	0.980	0.984	0.981
1460	0.981	0.980	0.985	0.981
1470	0.980	0.980	0.984	0.980
1480	0.982	0.982	0.986	0.982
1490	0.978	0.978	0.981	0.978
1500	0.979	0.978	0.984	0.979
1510	0.980	0.980	0.985	0.980
1520	0.982	0.981	0.986	0.982
1530	0.979	0.978	0.983	0.979
1540	0.980	0.980	0.983	0.980
1550	0.980	0.980	0.983	0.980
1560	0.980	0.979	0.985	0.979
1570	0.982	0.981	0.986	0.982
1580	0.980	0.979	0.983	0.980
1590	0.982	0.981	0.986	0.982
1600	0.980	0.979	0.983	0.979
1610	0.983	0.983	0.985	0.983
1620	0.980	0.980	0.984	0.980
1630	0.982	0.982	0.985	0.982
1640	0.979	0.978	0.982	0.979
1650	0.981	0.980	0.985	0.981
1660	0.982	0.981	0.985	0.981
1670	0.982	0.981	0.986	0.981
1680	0.982	0.982	0.986	0.982
1690	0.978	0.978	0.983	0.978
1700	0.981	0.980	0.985	0.981
1710	0.979	0.978	0.984	0.979
1720	0.981	0.980	0.986	0.981
1730	0.982	0.982	0.986	0.982
1740	0.980	0.979	0.983	0.980
1750	0.980	0.980	0.984	0.980
1760	0.982	0.981	0.986	0.982
1770	0.980	0.980	0.984	0.980
1780	0.978	0.978	0.985	0.978
1790	0.985	0.985	0.988	0.985
1800	0.980	0.979	0.982	0.980
1810	0.981	0.980	0.985	0.981
1820	0.982	0.982	0.985	0.982
1830	0.980	0.980	0.984	0.980
1840	0.981	0.980	0.985	0.981
1850	0.982	0.981	0.985	0.982
1860	0.982	0.982	0.986	0.982
1870	0.982	0.981	0.986	0.981

1880	0.982	0.982	0.987	0.982
1890	0.982	0.981	0.986	0.982
1900	0.982	0.981	0.984	0.982
1910	0.982	0.982	0.986	0.982
1920	0.983	0.983	0.986	0.983
1930	0.981	0.980	0.986	0.981
1940	0.984	0.984	0.987	0.984
1950	0.982	0.981	0.986	0.982
1960	0.982	0.982	0.986	0.982
1970	0.981	0.980	0.986	0.981
1980	0.980	0.980	0.985	0.980
1990	0.980	0.979	0.982	0.980
2000	0.980	0.979	0.982	0.980
2010	0.982	0.981	0.987	0.982
2020	0.980	0.980	0.982	0.980
2030	0.981	0.980	0.984	0.981
2040	0.981	0.980	0.984	0.981
2050	0.980	0.980	0.984	0.980
2060	0.984	0.983	0.987	0.984
2070	0.979	0.978	0.984	0.979
2080	0.982	0.981	0.984	0.981
2090	0.981	0.980	0.984	0.981
2100	0.982	0.982	0.986	0.982
2110	0.984	0.983	0.987	0.984
2120	0.980	0.980	0.985	0.980
2130	0.984	0.983	0.987	0.984
2140	0.981	0.980	0.985	0.981
2150	0.982	0.981	0.984	0.982
2160	0.985	0.985	0.988	0.985
2170	0.981	0.980	0.985	0.981
2180	0.983	0.983	0.987	0.983
2190	0.983	0.983	0.987	0.983
2200	0.982	0.982	0.985	0.982
2210	0.981	0.980	0.985	0.981
2220	0.983	0.983	0.987	0.983
2230	0.982	0.982	0.986	0.982
2240	0.979	0.978	0.985	0.979
2250	0.982	0.982	0.986	0.982
2260	0.980	0.979	0.983	0.980
2270	0.978	0.977	0.982	0.977
2280	0.980	0.979	0.984	0.980
2290	0.981	0.980	0.985	0.981
2300	0.982	0.981	0.985	0.982

2310	0.981	0.980	0.987	0.981
2320	0.981	0.980	0.985	0.981
2330	0.981	0.980	0.985	0.981
2340	0.979	0.978	0.982	0.979
2350	0.980	0.979	0.983	0.979
2360	0.979	0.978	0.983	0.979
2370	0.977	0.976	0.981	0.977
2380	0.976	0.976	0.982	0.976
2390	0.980	0.980	0.984	0.980
2400	0.981	0.980	0.985	0.981
2410	0.982	0.981	0.985	0.982
2420	0.982	0.982	0.985	0.982
2430	0.982	0.982	0.988	0.982
2440	0.978	0.978	0.983	0.978
2450	0.983	0.983	0.988	0.983
2460	0.982	0.981	0.985	0.982
2470	0.980	0.980	0.983	0.980
2480	0.980	0.980	0.985	0.980
2490	0.982	0.981	0.984	0.982
2500	0.983	0.983	0.986	0.983
2510	0.980	0.980	0.984	0.980
2520	0.982	0.982	0.986	0.982
2530	0.982	0.982	0.987	0.982
2540	0.981	0.980	0.983	0.981
2550	0.978	0.978	0.983	0.978
2560	0.981	0.980	0.984	0.981
2570	0.981	0.980	0.985	0.981
2580	0.980	0.979	0.985	0.980
2590	0.982	0.982	0.985	0.982
2600	0.980	0.979	0.984	0.979
2610	0.979	0.978	0.984	0.979
2620	0.977	0.976	0.983	0.977
2630	0.981	0.980	0.984	0.981
2640	0.981	0.980	0.986	0.981
2650	0.980	0.980	0.983	0.980
2660	0.978	0.977	0.984	0.977
2670	0.978	0.978	0.983	0.978
2680	0.983	0.983	0.987	0.983
2690	0.982	0.982	0.986	0.982
2700	0.979	0.978	0.982	0.979
2710	0.982	0.981	0.986	0.982
2720	0.982	0.981	0.986	0.982
2730	0.983	0.983	0.987	0.983

2740	0.980	0.979	0.985	0.979
2750	0.979	0.978	0.985	0.979
2760	0.983	0.983	0.986	0.983
2770	0.982	0.981	0.986	0.982
2780	0.983	0.983	0.987	0.983
2790	0.982	0.981	0.985	0.981
2800	0.980	0.980	0.984	0.980
2810	0.984	0.983	0.988	0.984
2820	0.977	0.976	0.982	0.977
2830	0.981	0.980	0.985	0.981
2840	0.980	0.980	0.984	0.980
2850	0.978	0.978	0.983	0.978
2860	0.982	0.981	0.984	0.981
2870	0.980	0.979	0.985	0.979
2880	0.982	0.981	0.986	0.982
2890	0.985	0.985	0.989	0.985
2900	0.981	0.980	0.985	0.981
2910	0.982	0.981	0.985	0.982
2920	0.982	0.982	0.986	0.982
2930	0.980	0.980	0.984	0.980
2940	0.979	0.978	0.985	0.979
2950	0.980	0.980	0.983	0.980
2960	0.981	0.980	0.985	0.981
2970	0.980	0.980	0.985	0.980
2980	0.983	0.983	0.988	0.983
2990	0.982	0.981	0.985	0.982
3000	0.980	0.979	0.983	0.980
3010	0.980	0.980	0.984	0.980
3020	0.976	0.976	0.981	0.976
3030	0.981	0.980	0.986	0.981
3040	0.978	0.978	0.982	0.978
3050	0.981	0.980	0.986	0.981
3060	0.981	0.980	0.986	0.981
3070	0.983	0.983	0.986	0.983
3080	0.980	0.980	0.983	0.980
3090	0.980	0.980	0.984	0.980
3100	0.982	0.982	0.986	0.982
3110	0.980	0.980	0.984	0.980
3120	0.978	0.978	0.982	0.978
3130	0.980	0.980	0.983	0.980
3140	0.983	0.983	0.986	0.983
3150	0.982	0.981	0.985	0.981
3160	0.980	0.980	0.985	0.980

3170	0.982	0.982	0.986	0.982
3180	0.980	0.980	0.984	0.980
3190	0.982	0.982	0.986	0.982
3200	0.981	0.980	0.985	0.981
3210	0.979	0.978	0.984	0.979
3220	0.982	0.981	0.986	0.982
3230	0.982	0.981	0.985	0.982
3240	0.982	0.981	0.986	0.982
3250	0.982	0.982	0.987	0.982
3260	0.982	0.981	0.986	0.982
3270	0.979	0.978	0.983	0.979
3280	0.982	0.981	0.986	0.982
3290	0.980	0.979	0.984	0.979
3300	0.984	0.983	0.988	0.984
3310	0.980	0.980	0.984	0.980
3320	0.982	0.981	0.986	0.982
3330	0.978	0.977	0.982	0.977
3340	0.980	0.979	0.984	0.980
3350	0.982	0.981	0.986	0.982
3360	0.978	0.978	0.984	0.978
3370	0.981	0.980	0.986	0.981
3380	0.980	0.979	0.983	0.980
3390	0.982	0.981	0.985	0.982
3400	0.979	0.978	0.983	0.979
3410	0.982	0.981	0.985	0.982
3420	0.982	0.981	0.986	0.982
3430	0.980	0.980	0.984	0.980
3440	0.980	0.979	0.985	0.979
3450	0.982	0.981	0.986	0.982
3460	0.984	0.983	0.988	0.984
3470	0.978	0.977	0.982	0.977
3480	0.980	0.980	0.985	0.980
3490	0.984	0.983	0.987	0.984
3500	0.981	0.980	0.985	0.981
3510	0.982	0.982	0.987	0.982
3520	0.985	0.985	0.989	0.985
3530	0.983	0.983	0.988	0.983
3540	0.979	0.978	0.983	0.979
3550	0.982	0.982	0.987	0.982
3560	0.981	0.980	0.985	0.981
3570	0.984	0.983	0.987	0.984
3580	0.984	0.983	0.987	0.984
3590	0.982	0.982	0.986	0.982

3600	0.980	0.980	0.985	0.980
3610	0.982	0.981	0.985	0.982
3620	0.980	0.979	0.983	0.980
3630	0.981	0.980	0.986	0.981
3640	0.980	0.980	0.984	0.980
3650	0.980	0.980	0.986	0.980
3660	0.982	0.982	0.986	0.982
3670	0.982	0.981	0.987	0.982
3680	0.981	0.980	0.986	0.981
3690	0.982	0.982	0.987	0.982
3700	0.979	0.978	0.983	0.979
3710	0.980	0.980	0.984	0.980
3720	0.982	0.981	0.986	0.982
3730	0.982	0.982	0.985	0.982
3740	0.981	0.980	0.986	0.981
3750	0.981	0.980	0.985	0.981
3760	0.983	0.983	0.987	0.983
3770	0.979	0.978	0.983	0.979
3780	0.978	0.978	0.985	0.978
3790	0.980	0.979	0.983	0.980
3800	0.980	0.979	0.985	0.980
3810	0.979	0.978	0.984	0.979
3820	0.982	0.982	0.986	0.982
3830	0.981	0.980	0.985	0.981
3840	0.982	0.981	0.987	0.982
3850	0.980	0.980	0.985	0.980
3860	0.982	0.982	0.987	0.982
3870	0.982	0.981	0.985	0.982
3880	0.984	0.983	0.989	0.984
3890	0.984	0.983	0.988	0.984
3900	0.983	0.983	0.986	0.983
3910	0.982	0.982	0.986	0.982
3920	0.984	0.983	0.988	0.984
3930	0.980	0.980	0.985	0.980
3940	0.982	0.982	0.986	0.982
3950	0.981	0.980	0.987	0.981
3960	0.982	0.982	0.986	0.982
3970	0.978	0.978	0.982	0.978
3980	0.982	0.981	0.987	0.982
3990	0.982	0.981	0.986	0.982
4000	0.983	0.983	0.988	0.983
4010	0.982	0.982	0.988	0.982
4020	0.978	0.977	0.983	0.977

4030	0.983	0.983	0.987	0.983
4040	0.979	0.978	0.984	0.979
4050	0.980	0.979	0.986	0.979
4060	0.983	0.983	0.987	0.983
4070	0.981	0.980	0.986	0.981
4080	0.981	0.980	0.985	0.981
4090	0.982	0.981	0.986	0.982
4100	0.982	0.982	0.986	0.982
4110	0.979	0.978	0.984	0.979
4120	0.978	0.977	0.982	0.977
4130	0.984	0.983	0.987	0.984
4140	0.979	0.978	0.983	0.979
4150	0.983	0.983	0.987	0.983
4160	0.982	0.982	0.987	0.982
4170	0.981	0.980	0.986	0.981
4180	0.981	0.980	0.985	0.981
4190	0.982	0.981	0.986	0.982
4200	0.981	0.980	0.986	0.981
4210	0.981	0.980	0.986	0.981
4220	0.980	0.980	0.986	0.980
4230	0.984	0.983	0.988	0.984
4240	0.980	0.979	0.985	0.980
4250	0.981	0.980	0.986	0.981
4260	0.981	0.980	0.986	0.981
4270	0.980	0.980	0.986	0.980
4280	0.981	0.980	0.986	0.981
4290	0.982	0.982	0.987	0.982
4300	0.982	0.982	0.985	0.982
4310	0.981	0.980	0.986	0.981
4320	0.982	0.982	0.987	0.982
4330	0.981	0.980	0.986	0.981
4340	0.984	0.983	0.987	0.984
4350	0.980	0.979	0.985	0.979
4360	0.978	0.977	0.982	0.978
4370	0.982	0.981	0.986	0.982
4380	0.982	0.981	0.985	0.982
4390	0.980	0.979	0.985	0.979
4400	0.984	0.983	0.988	0.984
4410	0.980	0.980	0.983	0.980
4420	0.982	0.982	0.985	0.982
4430	0.980	0.980	0.985	0.980
4440	0.979	0.978	0.982	0.979
4450	0.982	0.982	0.987	0.982

4460	0.980	0.979	0.983	0.979
4470	0.977	0.976	0.982	0.977
4480	0.982	0.982	0.987	0.982
4490	0.980	0.979	0.986	0.979
4500	0.978	0.977	0.983	0.977
4510	0.980	0.980	0.985	0.980
4520	0.983	0.983	0.987	0.983
4530	0.982	0.981	0.986	0.981
4540	0.980	0.980	0.986	0.980
4550	0.981	0.980	0.985	0.981
4560	0.980	0.979	0.986	0.979
4570	0.979	0.978	0.984	0.979
4580	0.981	0.980	0.985	0.981
4590	0.978	0.978	0.984	0.978
4600	0.980	0.980	0.985	0.980
4610	0.980	0.979	0.984	0.979
4620	0.980	0.980	0.984	0.980
4630	0.981	0.980	0.986	0.981
4640	0.984	0.983	0.988	0.984
4650	0.980	0.980	0.986	0.980
4660	0.980	0.980	0.985	0.980
4670	0.983	0.983	0.987	0.983
4680	0.984	0.984	0.989	0.984
4690	0.982	0.982	0.986	0.982
4700	0.981	0.980	0.986	0.981
4710	0.984	0.983	0.988	0.984
4720	0.984	0.983	0.987	0.984
4730	0.983	0.983	0.988	0.983
4740	0.983	0.983	0.987	0.983
4750	0.983	0.983	0.988	0.983
4760	0.980	0.979	0.984	0.980
4770	0.979	0.978	0.985	0.979
4780	0.978	0.978	0.984	0.978
4790	0.980	0.980	0.986	0.980
4800	0.983	0.983	0.988	0.983
4810	0.980	0.980	0.985	0.980
4820	0.979	0.978	0.984	0.979
4830	0.985	0.985	0.988	0.985
4840	0.982	0.981	0.986	0.982
4850	0.982	0.982	0.986	0.982
4860	0.984	0.984	0.989	0.984
4870	0.980	0.980	0.984	0.980
4880	0.984	0.983	0.988	0.984

4890	0.984	0.984	0.989	0.984
4900	0.986	0.985	0.990	0.986
4910	0.982	0.982	0.986	0.982
4920	0.984	0.984	0.988	0.984
4930	0.981	0.980	0.985	0.981
4940	0.982	0.982	0.987	0.982
4950	0.983	0.983	0.987	0.983
4960	0.984	0.984	0.988	0.984
4970	0.980	0.979	0.983	0.980
4980	0.981	0.980	0.985	0.981
4990	0.985	0.985	0.988	0.985
5000	0.984	0.984	0.988	0.984
5010	0.985	0.985	0.989	0.985
5020	0.982	0.981	0.986	0.982
5030	0.984	0.984	0.989	0.984
5040	0.981	0.980	0.984	0.981
5050	0.982	0.982	0.986	0.982
5060	0.983	0.983	0.988	0.983
5070	0.983	0.983	0.987	0.983
5080	0.982	0.981	0.985	0.982
5090	0.985	0.985	0.989	0.985
5100	0.982	0.981	0.985	0.982
5110	0.979	0.978	0.982	0.979
5120	0.979	0.978	0.986	0.979
5130	0.983	0.983	0.986	0.983
5140	0.979	0.978	0.985	0.979
5150	0.984	0.984	0.989	0.984
5160	0.985	0.985	0.989	0.985
5170	0.982	0.982	0.986	0.982
5180	0.982	0.982	0.987	0.982
5190	0.984	0.984	0.987	0.984
5200	0.982	0.981	0.986	0.982
5210	0.982	0.982	0.985	0.982
5220	0.985	0.985	0.988	0.985
5230	0.982	0.981	0.985	0.981
5240	0.985	0.985	0.989	0.985
5250	0.984	0.983	0.988	0.984
5260	0.984	0.983	0.988	0.984
5270	0.984	0.983	0.988	0.983
5280	0.984	0.984	0.988	0.984
5290	0.980	0.980	0.986	0.980
5300	0.980	0.979	0.983	0.979
5310	0.981	0.980	0.984	0.981

5320	0.984	0.984	0.989	0.984
5330	0.984	0.983	0.987	0.984
5340	0.983	0.983	0.987	0.983
5350	0.981	0.980	0.985	0.981
5360	0.987	0.987	0.991	0.987
5370	0.983	0.983	0.987	0.983
5380	0.984	0.983	0.988	0.984
5390	0.984	0.983	0.989	0.984
5400	0.982	0.981	0.985	0.982
5410	0.980	0.979	0.985	0.979
5420	0.982	0.982	0.986	0.982
5430	0.981	0.980	0.984	0.981
5440	0.982	0.981	0.987	0.981
5450	0.984	0.983	0.989	0.983
5460	0.982	0.981	0.986	0.982
5470	0.984	0.984	0.988	0.984
5480	0.983	0.983	0.988	0.983
5490	0.981	0.980	0.985	0.981
5500	0.984	0.984	0.989	0.984
5510	0.979	0.978	0.983	0.979
5520	0.983	0.983	0.988	0.983
5530	0.984	0.984	0.987	0.984
5540	0.984	0.984	0.988	0.984
5550	0.982	0.981	0.985	0.982
5560	0.984	0.984	0.989	0.984
5570	0.983	0.983	0.986	0.983
5580	0.985	0.985	0.989	0.985
5590	0.983	0.983	0.985	0.983
5600	0.980	0.979	0.984	0.979
5610	0.984	0.983	0.987	0.984
5620	0.983	0.983	0.986	0.983
5630	0.984	0.983	0.988	0.984
5640	0.984	0.984	0.987	0.984
5650	0.982	0.982	0.986	0.982
5660	0.980	0.980	0.985	0.980
5670	0.982	0.982	0.987	0.982
5680	0.984	0.984	0.988	0.984
5690	0.981	0.980	0.986	0.981
5700	0.983	0.983	0.986	0.983
5710	0.984	0.983	0.988	0.984
5720	0.984	0.984	0.989	0.984
5730	0.982	0.982	0.986	0.982
5740	0.985	0.985	0.989	0.985

5750	0.983	0.983	0.985	0.983
5760	0.980	0.980	0.986	0.980
5770	0.982	0.981	0.987	0.982
5780	0.982	0.982	0.986	0.982
5790	0.982	0.982	0.988	0.982
5800	0.980	0.980	0.985	0.980
5810	0.982	0.981	0.985	0.981
5820	0.984	0.983	0.987	0.984
5830	0.984	0.983	0.986	0.984
5840	0.982	0.981	0.985	0.982
5850	0.985	0.985	0.988	0.985
5860	0.983	0.983	0.986	0.983
5870	0.982	0.981	0.986	0.982
5880	0.982	0.981	0.987	0.981
5890	0.981	0.980	0.986	0.981
5900	0.984	0.984	0.988	0.984
5910	0.981	0.980	0.986	0.981
5920	0.983	0.983	0.987	0.983
5930	0.984	0.984	0.987	0.984
5940	0.982	0.982	0.986	0.982
5950	0.984	0.984	0.988	0.984
5960	0.984	0.983	0.986	0.984
5970	0.984	0.984	0.990	0.984
5980	0.983	0.983	0.988	0.983
5990	0.983	0.983	0.986	0.983
6000	0.982	0.982	0.986	0.982
6010	0.984	0.983	0.988	0.984
6020	0.982	0.982	0.986	0.982
6030	0.981	0.980	0.987	0.981
6040	0.984	0.983	0.987	0.984
6050	0.984	0.983	0.987	0.984
6060	0.982	0.982	0.986	0.982
6070	0.982	0.982	0.987	0.982
6080	0.984	0.984	0.987	0.984
6090	0.983	0.983	0.986	0.983
6100	0.982	0.981	0.985	0.982
6110	0.982	0.982	0.986	0.982
6120	0.982	0.982	0.986	0.982
6130	0.983	0.983	0.986	0.983
6140	0.984	0.983	0.988	0.983
6150	0.982	0.982	0.987	0.982
6160	0.982	0.982	0.987	0.982
6170	0.981	0.980	0.986	0.981

6180	0.982	0.982	0.987	0.982
6190	0.983	0.983	0.986	0.983
6200	0.982	0.982	0.986	0.982
6210	0.982	0.981	0.984	0.982
6220	0.980	0.980	0.985	0.980
6230	0.981	0.980	0.986	0.981
6240	0.984	0.984	0.987	0.984
6250	0.984	0.983	0.987	0.983
6260	0.984	0.983	0.987	0.984
6270	0.980	0.979	0.985	0.979
6280	0.981	0.980	0.986	0.981
6290	0.983	0.983	0.987	0.983
6300	0.982	0.981	0.986	0.982
6310	0.980	0.980	0.985	0.980
6320	0.986	0.985	0.990	0.986
6330	0.984	0.983	0.989	0.983
6340	0.983	0.983	0.987	0.983
6350	0.985	0.985	0.989	0.985
6360	0.984	0.983	0.988	0.984
6370	0.982	0.982	0.988	0.982
6380	0.983	0.983	0.988	0.983
6390	0.982	0.982	0.987	0.982
6400	0.985	0.985	0.990	0.985
6410	0.986	0.985	0.989	0.986
6420	0.982	0.981	0.986	0.982
6430	0.983	0.983	0.986	0.983
6440	0.979	0.978	0.983	0.979
6450	0.981	0.980	0.987	0.981
6460	0.982	0.982	0.986	0.982
6470	0.982	0.981	0.985	0.981
6480	0.982	0.982	0.986	0.982
6490	0.980	0.980	0.985	0.980
6500	0.980	0.980	0.984	0.980
6510	0.984	0.983	0.987	0.984
6520	0.982	0.981	0.986	0.982
6530	0.981	0.980	0.985	0.981
6540	0.984	0.983	0.988	0.984
6550	0.981	0.980	0.987	0.981
6560	0.981	0.980	0.986	0.981
6570	0.982	0.982	0.987	0.982
6580	0.981	0.980	0.985	0.981
6590	0.982	0.982	0.987	0.982
6600	0.983	0.983	0.987	0.983

6610	0.983	0.983	0.987	0.983
6620	0.982	0.982	0.987	0.982
6630	0.982	0.981	0.986	0.981
6640	0.984	0.983	0.987	0.984
6650	0.980	0.980	0.985	0.980
6660	0.982	0.981	0.986	0.982
6670	0.981	0.980	0.986	0.981
6680	0.982	0.981	0.985	0.982
6690	0.982	0.982	0.987	0.982
6700	0.985	0.985	0.989	0.985
6710	0.986	0.985	0.989	0.986
6720	0.979	0.978	0.984	0.979
6730	0.982	0.982	0.987	0.982
6740	0.982	0.981	0.986	0.982
6750	0.981	0.980	0.985	0.981
6760	0.984	0.983	0.987	0.984
6770	0.982	0.982	0.987	0.982
6780	0.984	0.984	0.987	0.984
6790	0.982	0.982	0.986	0.982
6800	0.980	0.980	0.985	0.980
6810	0.985	0.985	0.989	0.985
6820	0.985	0.985	0.988	0.985
6830	0.984	0.984	0.988	0.984
6840	0.986	0.985	0.989	0.986
6850	0.979	0.978	0.984	0.979
6860	0.983	0.983	0.987	0.983
6870	0.982	0.981	0.987	0.981
6880	0.982	0.982	0.987	0.982
6890	0.981	0.980	0.985	0.981
6900	0.981	0.980	0.986	0.981
6910	0.983	0.983	0.987	0.983
6920	0.986	0.985	0.988	0.986
6930	0.979	0.978	0.983	0.979
6940	0.984	0.983	0.987	0.984
6950	0.984	0.983	0.987	0.984
6960	0.984	0.983	0.987	0.984
6970	0.980	0.980	0.986	0.980
6980	0.982	0.982	0.987	0.982
6990	0.980	0.980	0.984	0.980
7000	0.984	0.983	0.989	0.984
7010	0.983	0.983	0.987	0.983
7020	0.987	0.987	0.991	0.987
7030	0.978	0.978	0.983	0.978

7040	0.982	0.981	0.986	0.981
7050	0.983	0.983	0.986	0.983
7060	0.984	0.983	0.987	0.984
7070	0.985	0.985	0.989	0.985
7080	0.982	0.982	0.986	0.982
7090	0.984	0.984	0.988	0.984
7100	0.979	0.978	0.984	0.979
7110	0.984	0.983	0.989	0.984
7120	0.985	0.985	0.987	0.985
7130	0.982	0.981	0.986	0.981
7140	0.983	0.983	0.986	0.983
7150	0.983	0.983	0.986	0.983
7160	0.984	0.984	0.988	0.984
7170	0.983	0.983	0.986	0.983
7180	0.983	0.983	0.988	0.983
7190	0.983	0.983	0.986	0.983
7200	0.980	0.979	0.985	0.979
7210	0.981	0.980	0.986	0.981
7220	0.984	0.983	0.987	0.984
7230	0.982	0.982	0.988	0.982
7240	0.986	0.985	0.989	0.986
7250	0.982	0.982	0.986	0.982
7260	0.981	0.980	0.986	0.981
7270	0.981	0.980	0.986	0.981
7280	0.982	0.981	0.987	0.982
7290	0.982	0.982	0.986	0.982
7300	0.984	0.983	0.989	0.984
7310	0.982	0.982	0.987	0.982
7320	0.980	0.980	0.985	0.980
7330	0.984	0.983	0.988	0.983
7340	0.984	0.984	0.986	0.984
7350	0.981	0.980	0.986	0.981
7360	0.982	0.981	0.986	0.981
7370	0.982	0.982	0.988	0.982
7380	0.982	0.982	0.986	0.982
7390	0.981	0.980	0.985	0.981
7400	0.981	0.980	0.985	0.981
7410	0.986	0.986	0.990	0.986
7420	0.984	0.983	0.988	0.983
7430	0.982	0.981	0.987	0.982
7440	0.982	0.982	0.986	0.982
7450	0.984	0.984	0.989	0.984
7460	0.980	0.980	0.986	0.980

7470	0.983	0.983	0.987	0.983
7480	0.984	0.983	0.988	0.984
7490	0.986	0.985	0.989	0.986
7500	0.984	0.983	0.988	0.984
7510	0.984	0.983	0.989	0.984
7520	0.981	0.980	0.986	0.981
7530	0.984	0.983	0.987	0.984
7540	0.984	0.983	0.989	0.984
7550	0.984	0.983	0.988	0.984
7560	0.984	0.983	0.988	0.984
7570	0.979	0.978	0.985	0.979
7580	0.982	0.982	0.988	0.982
7590	0.984	0.984	0.989	0.984
7600	0.986	0.986	0.989	0.986
7610	0.982	0.981	0.986	0.982
7620	0.982	0.982	0.987	0.982
7630	0.982	0.982	0.986	0.982
7640	0.985	0.985	0.990	0.985
7650	0.981	0.980	0.985	0.981
7660	0.981	0.980	0.986	0.981
7670	0.985	0.985	0.988	0.985
7680	0.984	0.984	0.988	0.984
7690	0.978	0.978	0.984	0.978
7700	0.982	0.981	0.986	0.982
7710	0.984	0.984	0.988	0.984
7720	0.983	0.983	0.988	0.983
7730	0.984	0.984	0.988	0.984
7740	0.982	0.981	0.986	0.982
7750	0.985	0.985	0.989	0.985
7760	0.981	0.980	0.987	0.981
7770	0.986	0.985	0.989	0.986
7780	0.984	0.983	0.988	0.984
7790	0.984	0.983	0.989	0.983
7800	0.983	0.983	0.988	0.983
7810	0.985	0.985	0.989	0.985
7820	0.987	0.987	0.991	0.987
7830	0.982	0.981	0.987	0.981
7840	0.980	0.980	0.984	0.980
7850	0.986	0.985	0.990	0.986
7860	0.982	0.981	0.986	0.981
7870	0.982	0.982	0.987	0.982
7880	0.982	0.981	0.986	0.982
7890	0.984	0.983	0.988	0.984

7900	0.984	0.983	0.988	0.984
7910	0.981	0.980	0.985	0.981
7920	0.980	0.980	0.985	0.980
7930	0.982	0.982	0.987	0.982
7940	0.982	0.982	0.986	0.982
7950	0.984	0.983	0.988	0.984
7960	0.981	0.980	0.985	0.981
7970	0.984	0.983	0.988	0.984
7980	0.978	0.978	0.985	0.978
7990	0.982	0.981	0.986	0.982
8000	0.984	0.983	0.987	0.984
8010	0.982	0.981	0.986	0.982
8020	0.983	0.983	0.987	0.983
8030	0.984	0.984	0.990	0.984
8040	0.981	0.980	0.986	0.981
8050	0.981	0.980	0.986	0.981
8060	0.983	0.983	0.988	0.983
8070	0.982	0.982	0.987	0.982
8080	0.982	0.981	0.987	0.981
8090	0.982	0.982	0.987	0.982
8100	0.984	0.984	0.988	0.984
8110	0.980	0.980	0.986	0.980
8120	0.982	0.982	0.986	0.982
8130	0.982	0.981	0.986	0.982
8140	0.983	0.983	0.986	0.983
8150	0.984	0.984	0.987	0.984
8160	0.980	0.979	0.985	0.979
8170	0.984	0.983	0.988	0.984
8180	0.985	0.985	0.989	0.985
8190	0.981	0.980	0.986	0.981
8200	0.982	0.982	0.986	0.982
8210	0.981	0.980	0.985	0.981
8220	0.982	0.981	0.986	0.981
8230	0.984	0.984	0.988	0.984
8240	0.982	0.982	0.986	0.982
8250	0.985	0.985	0.988	0.985
8260	0.982	0.982	0.987	0.982
8270	0.981	0.980	0.984	0.981
8280	0.980	0.980	0.986	0.980
8290	0.982	0.981	0.987	0.981
8300	0.982	0.982	0.986	0.982
8310	0.985	0.985	0.989	0.985
8320	0.981	0.980	0.986	0.981

8330	0.983	0.983	0.986	0.983
8340	0.984	0.983	0.987	0.984
8350	0.984	0.984	0.989	0.984
8360	0.980	0.980	0.984	0.980
8370	0.982	0.982	0.987	0.982
8380	0.982	0.982	0.986	0.982
8390	0.980	0.979	0.986	0.980
8400	0.984	0.984	0.989	0.984
8410	0.984	0.983	0.988	0.984
8420	0.986	0.985	0.991	0.986
8430	0.982	0.981	0.987	0.982
8440	0.982	0.982	0.987	0.982
8450	0.985	0.985	0.988	0.985
8460	0.984	0.984	0.989	0.984
8470	0.982	0.981	0.986	0.982
8480	0.984	0.984	0.989	0.984
8490	0.983	0.983	0.985	0.983
8500	0.985	0.985	0.989	0.985
8510	0.985	0.985	0.990	0.985
8520	0.983	0.983	0.987	0.983
8530	0.982	0.982	0.986	0.982
8540	0.984	0.984	0.987	0.984
8550	0.983	0.983	0.986	0.983
8560	0.985	0.985	0.989	0.985
8570	0.982	0.982	0.987	0.982
8580	0.984	0.984	0.989	0.984
8590	0.986	0.985	0.990	0.986
8600	0.983	0.983	0.988	0.983
8610	0.983	0.983	0.987	0.983
8620	0.986	0.986	0.990	0.986
8630	0.984	0.983	0.987	0.984
8640	0.981	0.980	0.986	0.981
8650	0.984	0.983	0.988	0.984
8660	0.986	0.985	0.990	0.986
8670	0.984	0.983	0.988	0.984
8680	0.983	0.983	0.987	0.983
8690	0.984	0.983	0.988	0.984
8700	0.984	0.983	0.987	0.984
8710	0.984	0.984	0.987	0.984
8720	0.983	0.983	0.988	0.983
8730	0.983	0.983	0.988	0.983
8740	0.981	0.980	0.986	0.981
8750	0.985	0.985	0.988	0.985

8760	0.983	0.983	0.987	0.983
8770	0.982	0.981	0.986	0.981
8780	0.983	0.983	0.987	0.983
8790	0.984	0.983	0.988	0.984
8800	0.983	0.983	0.987	0.983
8810	0.983	0.983	0.987	0.983
8820	0.986	0.985	0.989	0.986
8830	0.982	0.981	0.986	0.982
8840	0.984	0.983	0.987	0.984
8850	0.984	0.983	0.988	0.984
8860	0.984	0.983	0.988	0.984
8870	0.982	0.982	0.987	0.982
8880	0.983	0.983	0.988	0.983
8890	0.983	0.983	0.986	0.983
8900	0.984	0.984	0.989	0.984
8910	0.986	0.986	0.990	0.986
8920	0.983	0.983	0.986	0.983
8930	0.984	0.984	0.988	0.984
8940	0.981	0.980	0.985	0.981
8950	0.984	0.983	0.987	0.984

(6) IFS results with DT on the MCFS feature list

**Number of features**	**ACC**	**MCC**	**Macro F1**	**Weighted F1**
10	0.724	0.717	0.735	0.722
20	0.800	0.794	0.789	0.801
30	0.822	0.817	0.817	0.823
40	0.831	0.826	0.830	0.831
50	0.814	0.809	0.815	0.815
60	0.834	0.830	0.839	0.834
70	0.821	0.816	0.825	0.819
80	0.836	0.831	0.837	0.836
90	0.827	0.822	0.833	0.825
100	0.837	0.833	0.826	0.841
110	0.825	0.820	0.828	0.826
120	0.836	0.832	0.838	0.837
130	0.832	0.827	0.827	0.831
140	0.824	0.820	0.829	0.825
150	0.826	0.822	0.822	0.827
160	0.823	0.818	0.823	0.824
170	0.832	0.828	0.837	0.833
180	0.839	0.835	0.840	0.840
190	0.832	0.827	0.836	0.832
200	0.842	0.838	0.847	0.842

210	0.847	0.842	0.845	0.847
220	0.840	0.835	0.836	0.841
230	0.823	0.818	0.817	0.824
240	0.839	0.835	0.842	0.842
250	0.844	0.840	0.843	0.843
260	0.841	0.837	0.839	0.841
270	0.847	0.842	0.847	0.846
280	0.831	0.826	0.834	0.831
290	0.831	0.826	0.838	0.831
300	0.832	0.827	0.829	0.832
310	0.851	0.847	0.847	0.852
320	0.851	0.847	0.856	0.852
330	0.834	0.829	0.828	0.835
340	0.849	0.844	0.850	0.849
350	0.836	0.832	0.838	0.837
360	0.826	0.821	0.830	0.827
370	0.835	0.830	0.835	0.834
380	0.823	0.818	0.827	0.825
390	0.838	0.833	0.834	0.838
400	0.832	0.827	0.839	0.833
410	0.834	0.829	0.831	0.834
420	0.823	0.819	0.826	0.825
430	0.828	0.823	0.829	0.828
440	0.834	0.829	0.831	0.833
450	0.847	0.843	0.850	0.848
460	0.826	0.822	0.831	0.827
470	0.819	0.814	0.821	0.820
480	0.838	0.834	0.838	0.839
490	0.851	0.847	0.855	0.851
500	0.819	0.814	0.819	0.819
510	0.839	0.835	0.838	0.839
520	0.835	0.831	0.826	0.835
530	0.842	0.838	0.832	0.844
540	0.832	0.828	0.831	0.833
550	0.841	0.837	0.837	0.841
560	0.838	0.833	0.833	0.838
570	0.823	0.819	0.822	0.824
580	0.840	0.836	0.841	0.841
590	0.831	0.826	0.830	0.831
600	0.830	0.825	0.833	0.830
610	0.841	0.837	0.833	0.841
620	0.843	0.839	0.841	0.843
630	0.838	0.834	0.833	0.839

640	0.830	0.826	0.828	0.831
650	0.843	0.839	0.847	0.843
660	0.861	0.857	0.854	0.861
670	0.825	0.820	0.825	0.825
680	0.835	0.831	0.843	0.835
690	0.840	0.836	0.838	0.842
700	0.823	0.819	0.837	0.823
710	0.827	0.822	0.814	0.827
720	0.829	0.824	0.828	0.827
730	0.848	0.844	0.847	0.849
740	0.836	0.831	0.841	0.836
750	0.845	0.840	0.838	0.845
760	0.846	0.842	0.842	0.847
770	0.838	0.834	0.839	0.839
780	0.840	0.835	0.835	0.838
790	0.831	0.826	0.823	0.831
800	0.841	0.837	0.845	0.841
810	0.841	0.837	0.845	0.841
820	0.849	0.844	0.849	0.848
830	0.840	0.835	0.845	0.841
840	0.831	0.826	0.830	0.830
850	0.843	0.839	0.841	0.842
860	0.849	0.845	0.851	0.849
870	0.832	0.828	0.830	0.833
880	0.828	0.823	0.836	0.826
890	0.838	0.833	0.825	0.837
900	0.838	0.833	0.840	0.838
910	0.844	0.840	0.847	0.842
920	0.840	0.836	0.826	0.841
930	0.849	0.844	0.857	0.850
940	0.848	0.844	0.846	0.849
950	0.846	0.842	0.847	0.847
960	0.845	0.841	0.841	0.847
970	0.851	0.847	0.854	0.853
980	0.836	0.832	0.834	0.837
990	0.836	0.831	0.838	0.837
1000	0.838	0.834	0.846	0.838
1010	0.837	0.833	0.847	0.837
1020	0.843	0.839	0.839	0.844
1030	0.851	0.847	0.850	0.852
1040	0.847	0.843	0.840	0.846
1050	0.837	0.833	0.833	0.837
1060	0.840	0.835	0.845	0.839

1070	0.829	0.824	0.827	0.829
1080	0.849	0.845	0.852	0.850
1090	0.838	0.834	0.844	0.838
1100	0.819	0.814	0.821	0.819
1110	0.849	0.844	0.848	0.849
1120	0.852	0.848	0.858	0.852
1130	0.845	0.841	0.850	0.846
1140	0.856	0.852	0.853	0.857
1150	0.850	0.846	0.851	0.850
1160	0.851	0.847	0.844	0.851
1170	0.846	0.842	0.853	0.848
1180	0.826	0.821	0.829	0.826
1190	0.855	0.851	0.858	0.856
1200	0.851	0.846	0.854	0.850
1210	0.834	0.830	0.837	0.836
1220	0.846	0.842	0.846	0.845
1230	0.847	0.843	0.853	0.848
1240	0.856	0.852	0.855	0.855
1250	0.849	0.844	0.848	0.848
1260	0.855	0.851	0.859	0.853
1270	0.854	0.850	0.852	0.853
1280	0.841	0.837	0.835	0.842
1290	0.841	0.837	0.845	0.843
1300	0.859	0.855	0.859	0.858
1310	0.847	0.843	0.846	0.849
1320	0.840	0.835	0.839	0.839
1330	0.862	0.858	0.856	0.864
1340	0.842	0.838	0.843	0.842
1350	0.851	0.847	0.856	0.852
1360	0.853	0.849	0.848	0.855
1370	0.836	0.832	0.832	0.838
1380	0.854	0.850	0.854	0.854
1390	0.851	0.847	0.841	0.853
1400	0.837	0.833	0.833	0.838
1410	0.849	0.845	0.847	0.849
1420	0.832	0.828	0.826	0.832
1430	0.840	0.836	0.840	0.840
1440	0.836	0.831	0.828	0.835
1450	0.834	0.830	0.831	0.835
1460	0.834	0.829	0.826	0.835
1470	0.834	0.829	0.831	0.835
1480	0.830	0.826	0.823	0.831
1490	0.840	0.835	0.836	0.841

1500	0.851	0.847	0.850	0.852
1510	0.832	0.828	0.831	0.834
1520	0.831	0.826	0.826	0.830
1530	0.849	0.845	0.850	0.847
1540	0.836	0.831	0.828	0.837
1550	0.834	0.829	0.829	0.835
1560	0.836	0.832	0.831	0.838
1570	0.834	0.830	0.827	0.833
1580	0.842	0.837	0.836	0.843
1590	0.832	0.828	0.830	0.832
1600	0.849	0.845	0.850	0.851
1610	0.836	0.831	0.826	0.835
1620	0.834	0.830	0.835	0.836
1630	0.843	0.839	0.840	0.843
1640	0.851	0.847	0.845	0.848
1650	0.825	0.820	0.823	0.825
1660	0.826	0.822	0.821	0.825
1670	0.832	0.828	0.835	0.833
1680	0.842	0.838	0.839	0.843
1690	0.837	0.833	0.831	0.836
1700	0.836	0.832	0.832	0.837
1710	0.837	0.833	0.838	0.837
1720	0.848	0.844	0.838	0.848
1730	0.838	0.834	0.837	0.840
1740	0.837	0.833	0.834	0.837
1750	0.832	0.828	0.829	0.833
1760	0.848	0.844	0.842	0.849
1770	0.840	0.836	0.836	0.840
1780	0.826	0.821	0.834	0.826
1790	0.832	0.828	0.827	0.832
1800	0.838	0.834	0.830	0.838
1810	0.843	0.839	0.831	0.843
1820	0.839	0.835	0.839	0.840
1830	0.838	0.833	0.841	0.838
1840	0.839	0.835	0.838	0.840
1850	0.833	0.828	0.830	0.833
1860	0.840	0.836	0.832	0.842
1870	0.837	0.833	0.839	0.838
1880	0.838	0.834	0.836	0.838
1890	0.842	0.838	0.848	0.843
1900	0.855	0.851	0.858	0.854
1910	0.830	0.825	0.829	0.830
1920	0.835	0.830	0.845	0.835

1930	0.851	0.847	0.847	0.852
1940	0.831	0.826	0.834	0.832
1950	0.843	0.839	0.844	0.843
1960	0.845	0.840	0.846	0.845
1970	0.828	0.823	0.826	0.830
1980	0.836	0.832	0.831	0.836
1990	0.847	0.843	0.848	0.847
2000	0.850	0.846	0.853	0.849
2010	0.822	0.817	0.819	0.822
2020	0.834	0.830	0.847	0.835
2030	0.840	0.835	0.841	0.838
2040	0.851	0.847	0.858	0.852
2050	0.842	0.838	0.842	0.842
2060	0.828	0.823	0.830	0.828
2070	0.845	0.841	0.842	0.846
2080	0.834	0.830	0.834	0.835
2090	0.842	0.838	0.830	0.844
2100	0.834	0.829	0.832	0.833
2110	0.851	0.847	0.857	0.851
2120	0.838	0.833	0.839	0.836
2130	0.836	0.831	0.831	0.836
2140	0.843	0.839	0.846	0.843
2150	0.831	0.826	0.826	0.831
2160	0.842	0.837	0.849	0.842
2170	0.832	0.828	0.837	0.833
2180	0.835	0.831	0.837	0.836
2190	0.841	0.837	0.846	0.842
2200	0.833	0.828	0.831	0.832
2210	0.835	0.831	0.832	0.836
2220	0.827	0.822	0.829	0.827
2230	0.837	0.833	0.832	0.837
2240	0.851	0.847	0.856	0.852
2250	0.854	0.850	0.848	0.854
2260	0.826	0.821	0.835	0.826
2270	0.832	0.827	0.836	0.831
2280	0.836	0.831	0.840	0.836
2290	0.844	0.840	0.843	0.843
2300	0.831	0.826	0.830	0.831
2310	0.851	0.847	0.852	0.851
2320	0.840	0.836	0.839	0.839
2330	0.846	0.842	0.839	0.846
2340	0.835	0.831	0.832	0.834
2350	0.834	0.829	0.832	0.834

2360	0.833	0.828	0.832	0.833
2370	0.843	0.839	0.838	0.844
2380	0.831	0.826	0.819	0.830
2390	0.853	0.849	0.850	0.852
2400	0.838	0.834	0.835	0.836
2410	0.841	0.837	0.835	0.841
2420	0.847	0.842	0.843	0.848
2430	0.832	0.828	0.835	0.831
2440	0.821	0.817	0.822	0.820
2450	0.849	0.845	0.850	0.848
2460	0.856	0.852	0.854	0.858
2470	0.842	0.838	0.848	0.843
2480	0.837	0.833	0.843	0.836
2490	0.847	0.843	0.851	0.847
2500	0.854	0.850	0.857	0.854
2510	0.828	0.824	0.827	0.828
2520	0.853	0.849	0.851	0.852
2530	0.839	0.835	0.843	0.840
2540	0.838	0.833	0.840	0.837
2550	0.842	0.838	0.849	0.843
2560	0.839	0.835	0.837	0.839
2570	0.850	0.846	0.855	0.849
2580	0.851	0.847	0.853	0.850
2590	0.828	0.823	0.836	0.827
2600	0.834	0.829	0.835	0.833
2610	0.836	0.831	0.837	0.835
2620	0.838	0.834	0.838	0.839
2630	0.832	0.827	0.828	0.831
2640	0.845	0.841	0.846	0.845
2650	0.829	0.824	0.833	0.829
2660	0.835	0.831	0.843	0.834
2670	0.833	0.829	0.839	0.833
2680	0.845	0.841	0.845	0.845
2690	0.847	0.842	0.849	0.847
2700	0.849	0.845	0.849	0.850
2710	0.828	0.824	0.829	0.829
2720	0.851	0.847	0.851	0.851
2730	0.838	0.834	0.841	0.837
2740	0.844	0.840	0.845	0.844
2750	0.840	0.835	0.841	0.839
2760	0.839	0.835	0.844	0.838
2770	0.845	0.841	0.844	0.845
2780	0.850	0.846	0.858	0.849

2790	0.842	0.838	0.842	0.843
2800	0.856	0.852	0.858	0.855
2810	0.851	0.847	0.855	0.849
2820	0.849	0.845	0.857	0.849
2830	0.843	0.839	0.850	0.841
2840	0.835	0.830	0.834	0.835
2850	0.837	0.833	0.841	0.837
2860	0.830	0.825	0.827	0.829
2870	0.834	0.829	0.836	0.833
2880	0.850	0.846	0.847	0.850
2890	0.840	0.836	0.840	0.839
2900	0.847	0.843	0.841	0.846
2910	0.845	0.840	0.843	0.844
2920	0.836	0.831	0.826	0.836
2930	0.836	0.831	0.844	0.835
2940	0.842	0.838	0.843	0.842
2950	0.829	0.824	0.831	0.826
2960	0.852	0.848	0.848	0.852
2970	0.844	0.840	0.840	0.845
2980	0.833	0.828	0.836	0.832
2990	0.857	0.854	0.861	0.856
3000	0.837	0.833	0.836	0.835
3010	0.838	0.834	0.831	0.839
3020	0.818	0.813	0.819	0.820
3030	0.842	0.838	0.841	0.842
3040	0.830	0.825	0.828	0.829
3050	0.848	0.844	0.853	0.846
3060	0.837	0.833	0.840	0.837
3070	0.835	0.831	0.834	0.834
3080	0.837	0.833	0.840	0.835
3090	0.840	0.836	0.839	0.840
3100	0.854	0.850	0.852	0.854
3110	0.845	0.840	0.843	0.844
3120	0.838	0.834	0.841	0.839
3130	0.838	0.833	0.844	0.837
3140	0.840	0.836	0.836	0.841
3150	0.838	0.834	0.832	0.838
3160	0.832	0.828	0.830	0.832
3170	0.840	0.835	0.839	0.840
3180	0.822	0.817	0.819	0.823
3190	0.830	0.826	0.825	0.830
3200	0.853	0.849	0.851	0.852
3210	0.832	0.828	0.824	0.832

3220	0.838	0.833	0.839	0.836
3230	0.840	0.836	0.841	0.839
3240	0.832	0.827	0.829	0.830
3250	0.852	0.848	0.852	0.852
3260	0.834	0.829	0.827	0.832
3270	0.840	0.836	0.844	0.840
3280	0.826	0.821	0.823	0.827
3290	0.830	0.825	0.829	0.830
3300	0.845	0.841	0.851	0.844
3310	0.842	0.838	0.845	0.842
3320	0.848	0.844	0.851	0.848
3330	0.845	0.841	0.849	0.844
3340	0.840	0.835	0.835	0.840
3350	0.851	0.847	0.850	0.850
3360	0.839	0.835	0.831	0.842
3370	0.849	0.845	0.853	0.847
3380	0.845	0.840	0.839	0.845
3390	0.840	0.836	0.846	0.839
3400	0.853	0.849	0.851	0.852
3410	0.851	0.847	0.856	0.851
3420	0.836	0.832	0.833	0.836
3430	0.841	0.837	0.839	0.840
3440	0.859	0.856	0.860	0.860
3450	0.860	0.856	0.854	0.860
3460	0.828	0.824	0.834	0.829
3470	0.850	0.846	0.855	0.849
3480	0.845	0.840	0.842	0.844
3490	0.836	0.832	0.830	0.836
3500	0.845	0.840	0.844	0.845
3510	0.855	0.851	0.859	0.853
3520	0.829	0.824	0.835	0.827
3530	0.839	0.835	0.836	0.839
3540	0.838	0.834	0.842	0.839
3550	0.834	0.830	0.835	0.833
3560	0.828	0.824	0.830	0.827
3570	0.839	0.835	0.837	0.839
3580	0.841	0.837	0.835	0.839
3590	0.828	0.823	0.826	0.829
3600	0.840	0.836	0.839	0.839
3610	0.853	0.849	0.857	0.853
3620	0.835	0.830	0.835	0.836
3630	0.825	0.820	0.821	0.824
3640	0.840	0.836	0.845	0.840

3650	0.855	0.851	0.856	0.855
3660	0.839	0.835	0.831	0.838
3670	0.850	0.846	0.848	0.849
3680	0.840	0.836	0.834	0.840
3690	0.847	0.843	0.841	0.848
3700	0.828	0.823	0.821	0.827
3710	0.843	0.839	0.841	0.843
3720	0.826	0.822	0.828	0.826
3730	0.836	0.831	0.835	0.836
3740	0.856	0.852	0.858	0.853
3750	0.851	0.847	0.842	0.851
3760	0.845	0.840	0.844	0.845
3770	0.832	0.827	0.829	0.832
3780	0.856	0.852	0.857	0.856
3790	0.844	0.840	0.844	0.842
3800	0.835	0.831	0.827	0.835
3810	0.835	0.831	0.829	0.832
3820	0.846	0.842	0.843	0.845
3830	0.855	0.851	0.850	0.857
3840	0.834	0.830	0.828	0.835
3850	0.829	0.824	0.823	0.830
3860	0.853	0.849	0.844	0.854
3870	0.845	0.841	0.842	0.845
3880	0.842	0.837	0.838	0.840
3890	0.843	0.839	0.842	0.842
3900	0.853	0.849	0.853	0.852
3910	0.851	0.847	0.848	0.850
3920	0.834	0.830	0.836	0.833
3930	0.838	0.833	0.835	0.837
3940	0.857	0.853	0.853	0.857
3950	0.850	0.846	0.845	0.851
3960	0.840	0.835	0.832	0.841
3970	0.825	0.820	0.819	0.825
3980	0.839	0.835	0.838	0.837
3990	0.827	0.822	0.825	0.827
4000	0.843	0.839	0.849	0.843
4010	0.840	0.836	0.843	0.839
4020	0.840	0.836	0.837	0.839
4030	0.830	0.826	0.822	0.830
4040	0.857	0.853	0.856	0.858
4050	0.847	0.843	0.850	0.848
4060	0.852	0.848	0.858	0.851
4070	0.839	0.835	0.836	0.841

4080	0.840	0.836	0.839	0.839
4090	0.855	0.851	0.853	0.853
4100	0.834	0.830	0.834	0.834
4110	0.838	0.834	0.844	0.838
4120	0.844	0.840	0.836	0.843
4130	0.856	0.852	0.851	0.855
4140	0.845	0.840	0.848	0.846
4150	0.847	0.843	0.843	0.845
4160	0.853	0.849	0.851	0.853
4170	0.836	0.831	0.828	0.836
4180	0.846	0.842	0.849	0.845
4190	0.860	0.856	0.860	0.860
4200	0.856	0.852	0.862	0.856
4210	0.838	0.833	0.835	0.836
4220	0.840	0.836	0.835	0.840
4230	0.842	0.838	0.844	0.842
4240	0.842	0.837	0.841	0.843
4250	0.843	0.839	0.845	0.843
4260	0.840	0.836	0.835	0.840
4270	0.846	0.842	0.846	0.846
4280	0.842	0.838	0.840	0.843
4290	0.848	0.844	0.841	0.848
4300	0.847	0.843	0.852	0.848
4310	0.835	0.831	0.838	0.836
4320	0.854	0.850	0.847	0.856
4330	0.834	0.830	0.836	0.832
4340	0.828	0.824	0.830	0.828
4350	0.847	0.842	0.844	0.847
4360	0.857	0.854	0.848	0.858
4370	0.839	0.835	0.847	0.840
4380	0.840	0.835	0.835	0.839
4390	0.847	0.843	0.850	0.846
4400	0.844	0.840	0.855	0.845
4410	0.841	0.837	0.836	0.841
4420	0.847	0.842	0.842	0.846
4430	0.835	0.831	0.835	0.834
4440	0.821	0.817	0.826	0.821
4450	0.843	0.839	0.839	0.844
4460	0.836	0.832	0.838	0.837
4470	0.838	0.834	0.826	0.839
4480	0.841	0.837	0.835	0.842
4490	0.852	0.848	0.849	0.852
4500	0.831	0.826	0.825	0.832

4510	0.856	0.852	0.856	0.857
4520	0.835	0.831	0.843	0.835
4530	0.851	0.847	0.853	0.851
4540	0.855	0.851	0.856	0.855
4550	0.847	0.843	0.844	0.847
4560	0.838	0.834	0.833	0.837
4570	0.855	0.851	0.851	0.855
4580	0.849	0.845	0.850	0.850
4590	0.851	0.847	0.848	0.851
4600	0.855	0.851	0.855	0.854
4610	0.839	0.835	0.841	0.840
4620	0.858	0.854	0.856	0.858
4630	0.845	0.840	0.838	0.845
4640	0.843	0.839	0.837	0.844
4650	0.850	0.846	0.846	0.849
4660	0.849	0.844	0.841	0.848
4670	0.859	0.855	0.864	0.859
4680	0.840	0.835	0.840	0.839
4690	0.840	0.836	0.845	0.841
4700	0.826	0.822	0.816	0.828
4710	0.847	0.842	0.853	0.846
4720	0.852	0.848	0.850	0.853
4730	0.845	0.840	0.841	0.844
4740	0.844	0.840	0.841	0.843
4750	0.855	0.851	0.857	0.856
4760	0.841	0.837	0.849	0.842
4770	0.836	0.831	0.843	0.836
4780	0.845	0.840	0.836	0.847
4790	0.835	0.830	0.827	0.835
4800	0.840	0.835	0.845	0.840
4810	0.853	0.849	0.851	0.854
4820	0.853	0.849	0.857	0.853
4830	0.840	0.835	0.827	0.840
4840	0.832	0.828	0.829	0.832
4850	0.840	0.836	0.842	0.840
4860	0.845	0.840	0.848	0.845
4870	0.853	0.849	0.861	0.853
4880	0.839	0.835	0.844	0.838
4890	0.848	0.844	0.846	0.848
4900	0.826	0.821	0.831	0.826
4910	0.842	0.838	0.845	0.843
4920	0.842	0.837	0.836	0.842
4930	0.838	0.833	0.837	0.838

4940	0.859	0.855	0.864	0.858
4950	0.828	0.823	0.823	0.826
4960	0.832	0.827	0.839	0.832
4970	0.846	0.842	0.843	0.847
4980	0.851	0.847	0.848	0.851
4990	0.849	0.845	0.847	0.850
5000	0.836	0.831	0.832	0.836
5010	0.843	0.839	0.843	0.843
5020	0.831	0.826	0.822	0.831
5030	0.834	0.829	0.838	0.834
5040	0.837	0.833	0.835	0.837
5050	0.836	0.831	0.832	0.835
5060	0.846	0.842	0.845	0.845
5070	0.845	0.840	0.835	0.844
5080	0.849	0.845	0.847	0.849
5090	0.829	0.824	0.820	0.829
5100	0.848	0.844	0.849	0.848
5110	0.842	0.838	0.835	0.844
5120	0.847	0.843	0.856	0.847
5130	0.861	0.857	0.857	0.860
5140	0.855	0.851	0.868	0.855
5150	0.836	0.831	0.837	0.836
5160	0.836	0.831	0.838	0.836
5170	0.837	0.833	0.828	0.839
5180	0.838	0.834	0.842	0.839
5190	0.842	0.838	0.841	0.842
5200	0.853	0.849	0.852	0.853
5210	0.842	0.838	0.839	0.842
5220	0.848	0.844	0.841	0.849
5230	0.837	0.833	0.836	0.838
5240	0.849	0.844	0.851	0.848
5250	0.840	0.836	0.845	0.841
5260	0.838	0.834	0.844	0.839
5270	0.850	0.846	0.837	0.852
5280	0.845	0.840	0.835	0.846
5290	0.850	0.846	0.847	0.852
5300	0.844	0.840	0.841	0.844
5310	0.836	0.831	0.834	0.835
5320	0.846	0.842	0.853	0.844
5330	0.849	0.844	0.849	0.850
5340	0.832	0.828	0.835	0.832
5350	0.851	0.847	0.857	0.851
5360	0.840	0.835	0.836	0.840

5370	0.838	0.833	0.832	0.838
5380	0.828	0.823	0.820	0.828
5390	0.850	0.846	0.847	0.851
5400	0.851	0.847	0.857	0.852
5410	0.836	0.832	0.838	0.838
5420	0.839	0.835	0.843	0.838
5430	0.845	0.841	0.839	0.846
5440	0.854	0.850	0.858	0.853
5450	0.830	0.826	0.836	0.830
5460	0.846	0.842	0.842	0.845
5470	0.842	0.837	0.842	0.842
5480	0.841	0.837	0.839	0.842
5490	0.847	0.843	0.840	0.848
5500	0.855	0.851	0.850	0.856
5510	0.856	0.852	0.855	0.855
5520	0.866	0.863	0.860	0.868
5530	0.826	0.821	0.820	0.826
5540	0.831	0.826	0.826	0.832
5550	0.841	0.837	0.844	0.840
5560	0.840	0.835	0.844	0.839
5570	0.848	0.844	0.846	0.849
5580	0.857	0.853	0.858	0.856
5590	0.849	0.845	0.844	0.849
5600	0.840	0.835	0.831	0.840
5610	0.855	0.851	0.848	0.856
5620	0.842	0.838	0.838	0.841
5630	0.840	0.835	0.847	0.839
5640	0.838	0.834	0.831	0.839
5650	0.833	0.828	0.821	0.834
5660	0.840	0.836	0.832	0.842
5670	0.845	0.841	0.849	0.845
5680	0.840	0.835	0.835	0.841
5690	0.856	0.852	0.849	0.855
5700	0.847	0.842	0.849	0.847
5710	0.847	0.842	0.850	0.847
5720	0.845	0.841	0.841	0.845
5730	0.833	0.828	0.832	0.834
5740	0.847	0.843	0.839	0.847
5750	0.853	0.849	0.856	0.854
5760	0.836	0.831	0.837	0.837
5770	0.832	0.827	0.825	0.832
5780	0.849	0.845	0.851	0.848
5790	0.840	0.835	0.831	0.840

5800	0.844	0.840	0.847	0.844
5810	0.847	0.843	0.846	0.847
5820	0.840	0.836	0.835	0.841
5830	0.845	0.841	0.846	0.844
5840	0.841	0.837	0.839	0.841
5850	0.838	0.834	0.842	0.839
5860	0.843	0.839	0.836	0.844
5870	0.837	0.833	0.840	0.837
5880	0.827	0.822	0.819	0.827
5890	0.853	0.849	0.844	0.854
5900	0.841	0.837	0.837	0.841
5910	0.834	0.830	0.842	0.833
5920	0.842	0.838	0.841	0.845
5930	0.839	0.835	0.837	0.839
5940	0.838	0.833	0.835	0.838
5950	0.851	0.847	0.849	0.851
5960	0.845	0.841	0.846	0.848
5970	0.834	0.830	0.832	0.834
5980	0.830	0.826	0.832	0.830
5990	0.829	0.824	0.835	0.828
6000	0.841	0.837	0.843	0.839
6010	0.850	0.846	0.856	0.850
6020	0.842	0.838	0.843	0.842
6030	0.851	0.847	0.851	0.852
6040	0.847	0.843	0.840	0.848
6050	0.831	0.826	0.835	0.832
6060	0.844	0.840	0.836	0.845
6070	0.840	0.835	0.839	0.841
6080	0.842	0.838	0.839	0.843
6090	0.835	0.831	0.822	0.836
6100	0.849	0.844	0.846	0.848
6110	0.826	0.822	0.819	0.827
6120	0.849	0.845	0.847	0.848
6130	0.840	0.836	0.842	0.838
6140	0.849	0.845	0.845	0.850
6150	0.835	0.831	0.832	0.835
6160	0.842	0.838	0.837	0.842
6170	0.839	0.835	0.831	0.839
6180	0.846	0.842	0.840	0.847
6190	0.835	0.831	0.833	0.836
6200	0.845	0.841	0.846	0.845
6210	0.831	0.826	0.824	0.831
6220	0.839	0.835	0.827	0.839

6230	0.829	0.824	0.821	0.829
6240	0.840	0.835	0.836	0.842
6250	0.853	0.849	0.852	0.854
6260	0.842	0.838	0.838	0.844
6270	0.838	0.834	0.834	0.838
6280	0.848	0.844	0.846	0.850
6290	0.847	0.843	0.843	0.848
6300	0.832	0.827	0.825	0.834
6310	0.844	0.840	0.847	0.844
6320	0.841	0.837	0.840	0.840
6330	0.837	0.833	0.833	0.836
6340	0.842	0.838	0.842	0.843
6350	0.836	0.832	0.833	0.836
6360	0.845	0.841	0.841	0.846
6370	0.847	0.843	0.848	0.846
6380	0.845	0.840	0.846	0.844
6390	0.839	0.835	0.835	0.840
6400	0.848	0.844	0.853	0.848
6410	0.843	0.839	0.846	0.844
6420	0.842	0.838	0.836	0.843
6430	0.850	0.846	0.848	0.849
6440	0.812	0.807	0.813	0.812
6450	0.838	0.833	0.833	0.839
6460	0.842	0.838	0.839	0.842
6470	0.853	0.849	0.843	0.853
6480	0.850	0.846	0.837	0.851
6490	0.842	0.838	0.837	0.843
6500	0.840	0.836	0.838	0.841
6510	0.838	0.833	0.825	0.840
6520	0.824	0.820	0.817	0.826
6530	0.838	0.834	0.833	0.837
6540	0.832	0.827	0.825	0.832
6550	0.826	0.821	0.816	0.828
6560	0.837	0.833	0.837	0.837
6570	0.842	0.838	0.838	0.843
6580	0.834	0.830	0.836	0.834
6590	0.827	0.822	0.818	0.827
6600	0.848	0.844	0.846	0.849
6610	0.839	0.835	0.834	0.839
6620	0.861	0.857	0.855	0.863
6630	0.836	0.832	0.831	0.837
6640	0.834	0.830	0.835	0.834
6650	0.838	0.834	0.835	0.839

6660	0.840	0.836	0.843	0.841
6670	0.815	0.810	0.820	0.814
6680	0.829	0.824	0.825	0.830
6690	0.845	0.840	0.836	0.845
6700	0.838	0.834	0.826	0.840
6710	0.843	0.839	0.839	0.844
6720	0.838	0.834	0.834	0.839
6730	0.858	0.854	0.853	0.858
6740	0.837	0.833	0.827	0.837
6750	0.847	0.842	0.841	0.846
6760	0.842	0.838	0.836	0.841
6770	0.838	0.834	0.836	0.838
6780	0.849	0.845	0.838	0.850
6790	0.837	0.833	0.829	0.836
6800	0.851	0.847	0.843	0.851
6810	0.835	0.831	0.832	0.834
6820	0.835	0.831	0.830	0.836
6830	0.829	0.824	0.826	0.827
6840	0.847	0.842	0.855	0.847
6850	0.849	0.845	0.847	0.847
6860	0.845	0.841	0.846	0.845
6870	0.843	0.839	0.844	0.844
6880	0.827	0.822	0.812	0.828
6890	0.839	0.835	0.826	0.839
6900	0.847	0.842	0.842	0.847
6910	0.849	0.845	0.840	0.851
6920	0.830	0.825	0.827	0.831
6930	0.858	0.854	0.860	0.858
6940	0.837	0.833	0.833	0.837
6950	0.845	0.840	0.833	0.846
6960	0.845	0.841	0.837	0.844
6970	0.830	0.825	0.835	0.830
6980	0.837	0.833	0.838	0.836
6990	0.838	0.834	0.841	0.837
7000	0.847	0.842	0.848	0.846
7010	0.838	0.833	0.846	0.837
7020	0.845	0.840	0.842	0.843
7030	0.861	0.857	0.864	0.860
7040	0.845	0.840	0.848	0.845
7050	0.849	0.844	0.840	0.850
7060	0.826	0.821	0.820	0.825
7070	0.845	0.841	0.844	0.846
7080	0.832	0.828	0.829	0.832

7090	0.842	0.838	0.837	0.843
7100	0.838	0.834	0.840	0.838
7110	0.834	0.830	0.836	0.835
7120	0.837	0.833	0.832	0.837
7130	0.855	0.851	0.854	0.855
7140	0.845	0.840	0.844	0.845
7150	0.853	0.849	0.848	0.853
7160	0.849	0.845	0.846	0.849
7170	0.838	0.833	0.834	0.838
7180	0.831	0.826	0.821	0.833
7190	0.826	0.822	0.827	0.827
7200	0.839	0.835	0.836	0.839
7210	0.834	0.829	0.830	0.833
7220	0.850	0.846	0.845	0.850
7230	0.835	0.830	0.830	0.836
7240	0.849	0.845	0.837	0.850
7250	0.846	0.842	0.843	0.846
7260	0.859	0.855	0.855	0.858
7270	0.830	0.825	0.821	0.829
7280	0.849	0.845	0.842	0.851
7290	0.830	0.825	0.822	0.828
7300	0.850	0.846	0.844	0.851
7310	0.843	0.839	0.828	0.844
7320	0.857	0.853	0.866	0.856
7330	0.841	0.837	0.832	0.840
7340	0.846	0.842	0.843	0.847
7350	0.847	0.843	0.848	0.848
7360	0.834	0.829	0.822	0.835
7370	0.830	0.826	0.824	0.831
7380	0.843	0.839	0.840	0.844
7390	0.835	0.831	0.830	0.836
7400	0.845	0.840	0.850	0.843
7410	0.847	0.843	0.847	0.848
7420	0.838	0.834	0.828	0.838
7430	0.850	0.846	0.846	0.850
7440	0.834	0.830	0.829	0.835
7450	0.848	0.844	0.844	0.848
7460	0.846	0.842	0.835	0.846
7470	0.842	0.838	0.847	0.843
7480	0.846	0.842	0.837	0.847
7490	0.841	0.837	0.838	0.841
7500	0.836	0.832	0.840	0.837
7510	0.840	0.836	0.840	0.840

7520	0.840	0.836	0.831	0.842
7530	0.831	0.826	0.827	0.831
7540	0.835	0.831	0.832	0.836
7550	0.842	0.838	0.841	0.843
7560	0.832	0.828	0.833	0.834
7570	0.844	0.840	0.843	0.843
7580	0.836	0.832	0.836	0.837
7590	0.835	0.831	0.825	0.836
7600	0.835	0.831	0.829	0.834
7610	0.840	0.836	0.833	0.841
7620	0.840	0.835	0.831	0.839
7630	0.845	0.840	0.833	0.844
7640	0.842	0.838	0.834	0.844
7650	0.848	0.844	0.842	0.848
7660	0.844	0.840	0.848	0.844
7670	0.835	0.831	0.828	0.835
7680	0.838	0.834	0.834	0.838
7690	0.842	0.838	0.839	0.842
7700	0.841	0.837	0.843	0.842
7710	0.849	0.845	0.840	0.850
7720	0.845	0.841	0.835	0.847
7730	0.837	0.833	0.837	0.838
7740	0.823	0.818	0.812	0.824
7750	0.842	0.837	0.839	0.842
7760	0.849	0.845	0.840	0.849
7770	0.855	0.851	0.853	0.856
7780	0.848	0.844	0.846	0.847
7790	0.845	0.840	0.829	0.843
7800	0.843	0.839	0.839	0.844
7810	0.834	0.830	0.833	0.835
7820	0.844	0.840	0.839	0.846
7830	0.838	0.833	0.842	0.837
7840	0.843	0.839	0.845	0.844
7850	0.832	0.827	0.824	0.831
7860	0.846	0.842	0.847	0.845
7870	0.826	0.821	0.820	0.824
7880	0.845	0.841	0.845	0.845
7890	0.856	0.852	0.852	0.857
7900	0.836	0.831	0.840	0.835
7910	0.818	0.813	0.807	0.820
7920	0.838	0.834	0.841	0.839
7930	0.834	0.830	0.833	0.834
7940	0.828	0.823	0.825	0.829

7950	0.840	0.835	0.831	0.840
7960	0.845	0.841	0.844	0.845
7970	0.845	0.840	0.840	0.845
7980	0.828	0.824	0.817	0.830
7990	0.843	0.839	0.839	0.844
8000	0.836	0.831	0.823	0.837
8010	0.845	0.840	0.840	0.845
8020	0.837	0.833	0.833	0.838
8030	0.821	0.817	0.828	0.822
8040	0.839	0.835	0.835	0.838
8050	0.856	0.852	0.844	0.858
8060	0.837	0.833	0.831	0.837
8070	0.829	0.824	0.825	0.830
8080	0.850	0.846	0.848	0.851
8090	0.844	0.840	0.834	0.845
8100	0.836	0.832	0.827	0.838
8110	0.840	0.836	0.835	0.840
8120	0.847	0.843	0.842	0.849
8130	0.836	0.831	0.828	0.838
8140	0.837	0.833	0.831	0.839
8150	0.840	0.836	0.829	0.840
8160	0.855	0.851	0.851	0.854
8170	0.830	0.825	0.829	0.830
8180	0.849	0.845	0.844	0.849
8190	0.832	0.827	0.825	0.833
8200	0.857	0.853	0.863	0.856
8210	0.836	0.832	0.838	0.837
8220	0.847	0.843	0.839	0.849
8230	0.851	0.847	0.851	0.851
8240	0.841	0.837	0.826	0.842
8250	0.837	0.833	0.834	0.837
8260	0.849	0.845	0.835	0.850
8270	0.844	0.840	0.841	0.844
8280	0.837	0.833	0.826	0.837
8290	0.840	0.836	0.839	0.840
8300	0.824	0.820	0.816	0.826
8310	0.838	0.833	0.830	0.840
8320	0.840	0.835	0.837	0.841
8330	0.832	0.828	0.827	0.834
8340	0.857	0.853	0.849	0.857
8350	0.845	0.841	0.842	0.845
8360	0.842	0.837	0.837	0.842
8370	0.849	0.844	0.842	0.849

8380	0.834	0.830	0.835	0.833
8390	0.836	0.832	0.829	0.837
8400	0.847	0.842	0.851	0.847
8410	0.836	0.832	0.833	0.837
8420	0.835	0.831	0.830	0.837
8430	0.828	0.823	0.817	0.829
8440	0.823	0.818	0.817	0.823
8450	0.842	0.837	0.840	0.842
8460	0.841	0.837	0.836	0.842
8470	0.844	0.840	0.843	0.846
8480	0.847	0.842	0.844	0.847
8490	0.849	0.845	0.845	0.849
8500	0.844	0.840	0.838	0.845
8510	0.848	0.844	0.851	0.848
8520	0.835	0.831	0.830	0.837
8530	0.842	0.837	0.836	0.843
8540	0.841	0.837	0.842	0.840
8550	0.830	0.825	0.825	0.830
8560	0.831	0.826	0.825	0.832
8570	0.847	0.842	0.840	0.847
8580	0.845	0.840	0.843	0.845
8590	0.839	0.835	0.833	0.840
8600	0.844	0.839	0.844	0.845
8610	0.849	0.845	0.841	0.850
8620	0.833	0.828	0.831	0.833
8630	0.839	0.835	0.824	0.839
8640	0.856	0.852	0.852	0.856
8650	0.844	0.840	0.845	0.843
8660	0.853	0.849	0.855	0.854
8670	0.852	0.848	0.844	0.852
8680	0.842	0.838	0.826	0.843
8690	0.833	0.828	0.829	0.835
8700	0.819	0.815	0.814	0.820
8710	0.844	0.840	0.838	0.845
8720	0.842	0.838	0.842	0.844
8730	0.832	0.827	0.826	0.834
8740	0.841	0.837	0.840	0.842
8750	0.834	0.829	0.827	0.835
8760	0.830	0.825	0.823	0.830
8770	0.837	0.833	0.831	0.837
8780	0.834	0.829	0.831	0.834
8790	0.842	0.837	0.838	0.843
8800	0.857	0.854	0.843	0.858

8810	0.843	0.839	0.840	0.845
8820	0.834	0.830	0.829	0.836
8830	0.838	0.833	0.839	0.839
8840	0.853	0.849	0.838	0.854
8850	0.834	0.829	0.828	0.832
8860	0.834	0.829	0.829	0.835
8870	0.851	0.847	0.841	0.851
8880	0.841	0.837	0.838	0.843
8890	0.840	0.836	0.841	0.841
8900	0.850	0.846	0.847	0.851
8910	0.840	0.835	0.829	0.840
8920	0.834	0.829	0.830	0.833
8930	0.840	0.836	0.831	0.843
8940	0.825	0.820	0.821	0.826
8950	0.845	0.841	0.845	0.847
