# Supplementary material for: Identification of Methylation Signatures and Rules for Sarcoma Subtypes by Machine Learning Methods
Source: Biomed Res Int. 2022 Dec 28;2022:5297235. doi: 10.1155/2022/5297235 (PMC9812612; doi:10.1155/2022/5297235)
Supplement: Supplementary Materials — Table S1: fifty-nine sarcoma subtypes and their sample sizes. Table S2: feature ranking results obtained using LASSO, LightGBM, and MCFS. Table S3: performance of IFS with different classification algorithms on three feature lists. Table S4: gene symbols obtained by annotating the most essential methylation sites derived from the LASSO, LightGBM, and MCFS feature lists. Table S5: intersection of three gene sets annotated by most essential methylation sites extracted from the LASSO, LightGBM, and MCFS feature lists. The genes that appear in the 3, 2, and 1 gene subsets are shown. Table S6: classification rules generated by decision tree using its optimal features on three feature lists. [file 5297235.f1.zip › Table S4 (1).pdf]

Table S4: Gene symbols obtained by annotating the most essential methylation sites derived from the LASSO, LightGBM, and MCFS feature lists.

(1) Genes extracted from the LASSO feature list

Index	Gene symbol
1	ITSN1
2	LOC100133985
3	PPP3CC
4	ABLIM1
5	CHD1
6	WWP2
7	NOTCH3
8	NAT8L
9	RAB40B
10	MSI2
11	ABR
12	TPM1
13	ADARB2
14	BANF2
15	DSTYK
16	NFATC1
17	EEPD1
18	LDLR
19	ZBTB47
20	STK39
21	ADAMTSL2
22	RARA
23	LOC728743
24	ABHD6
25	CCND1
26	CIT
27	FAM83A
28	NET1
29	PTPN21
30	DDX17
31	SNX29
32	EI24
33	ATP6V0A1
34	ARHGEF4
35	RBM24
36	TBX15
37	TNRC18
38	SEPT9

39	EBF3
40	PRKAR1B
41	IL13
42	WASF2
43	RELL1
44	FITM1
45	DBNDD1
46	BTBD11
47	LRP3
48	KLHL29
49	MAP7D1
50	MYO18B
51	POLRMT
52	TCF7L2
53	CANX
54	CDGAP
55	KIAA0174
56	ANO1
57	STXBP5
58	PDE8A
59	NPFFR2
60	FLNB
61	CD109
62	LITAF
63	ETV6
64	MCC
65	ITSN2
66	PPARGC1A
67	MAMSTR
68	TK2
69	CUGBP2
70	NOS1
71	OSBPL1A
72	CBFA2T3
73	ZNF788
74	APOL3
75	KIF19
76	MYT1L
77	DHRS7
78	JAK1
79	ARNT2
80	HEXDC
81	CHD9

82	FBXW8
83	USP39

(2) Genes extracted from the LightGBM feature list

**Index**	**Gene symbol**
1	ANXA11
2	SCAP
3	TBX4
4	USP3
5	PAPOLA
6	PCCA
7	TUBB1
8	RBPMS
9	FAM178B
10	C15orf61
11	PCGF3
12	MGMT
13	HLTF
14	TIAM2
15	LDLR
16	BBS9
17	ESPNP
18	NCOR2
19	ENO3
20	LTBP3
21	NFIC
22	ZC3H3
23	BAHCC1
24	MCC
25	C1orf83
26	ANKRD11
27	LIPC
28	IL13
29	FLJ43663
30	VRK3
31	NFATC1
32	AUTS2
33	CDK10
34	CFLAR
35	MAFK
36	PDS5A
37	LONRF1
38	FBXL7

39	CTDSPL
40	C6orf129
41	ADAR
42	TRIM26
43	TENC1
44	C16orf45
45	SEMA3B
46	TSPAN5
47	GPR137B
48	ZMYND10
49	S100A5
50	PCOLCE
51	PBX2
52	LGALS8
53	ZMAT3
54	WDR27
55	MACROD1
56	SP140L
57	REPS1
58	PITRM1
59	MAD1L1
60	WDR60
61	NSMCE2
62	ACACA
63	MSRA
64	NCRNA00171
65	SNX33
66	LRP5
67	ANO2
68	NUDT16P
69	SND1
70	SH3RF3
71	PARD3B
72	CLUAP1
73	CLPTM1L
74	GRB2
75	KRT18
76	SFRS8
77	PRKAR1B
78	TLE2
79	ASXL3
80	LCK
81	INPP5A

82	ST6GALNAC4
83	GLG1
84	CTBP2
85	LRRC27
86	RALB
87	PIGZ
88	RAD51L1
89	ECSCR
90	HIRA
91	CCDC12
92	MEF2D
93	CDYL
94	KIF25
95	DYSF
96	AGPAT3
97	SRBD1
98	IER2
99	ROPN1L
100	CD109
101	SIN3B
102	RGS5
103	TPO
104	RPTOR
105	ARTN
106	NELL2
107	TBC1D4
108	ZNFX1
109	13-Sep
110	CUL3
111	MMP11
112	RAB11FIP3
113	MXI1
114	ASAP2
115	CCND1
116	CCNY
117	LETM1
118	PPP1R9B
119	NOS1
120	PACS2
121	ZMIZ1
122	PHOSPHO1
123	XPR1
124	HAPLN2

125	C9orf3
126	SLC37A3
127	RGS3
128	RHOU
129	ABLIM1
130	LYST
131	LBX2
132	PPP2R5E
133	CHFR
134	N4BP1
135	C5orf52
136	ITGA6
137	SLC39A11
138	ZBTB20
139	NCRNA00162
140	IRS2
141	MYO18B
142	COL1A1
143	UST
144	APLP2
145	SHANK1
146	RDH16
147	SORCS2
148	HES6
149	NPFFR2
150	CUX1
151	FNBP1L
152	BRE
153	MAPKAP1
154	ST3GAL3
155	FGFRL1
156	RARA
157	C7orf50
158	WDFY2
159	SNX29
160	RBM5
161	MNT
162	APOL1
163	HK1
164	NOS1AP
165	ZNRF3
166	LOC100129637
167	DOCK1

168	MEOX1
169	VGLL4
170	SLC17A3
171	RBP2
172	SYNCRIP
173	RCBTB2
174	OSBPL5
175	KIF19
176	RIPK4
177	CBX2
178	TSPAN14
179	TBCD
180	SH3PXD2B
181	GLI3
182	TBX15
183	ALDOA
184	PCSK7
185	FIS1
186	TNRC18

(3) Genes extracted from the MCFS feature list

**Index**	**Gene symbol**
1	INPP5A
2	PKP4
3	GLI3
4	TXNRD2
5	NHEJ1
6	TBC1D16
7	ASAP2
8	RAB2A
9	PHOSPHO1
10	WWP2
11	LOC728264
12	CTBP2
13	OTUD7B
14	PMVK
15	C7orf50
16	ADAM10
17	PRKAR1B
18	SEC14L1
