# Supplementary material for: Identification of Methylation Signatures and Rules for Sarcoma Subtypes by Machine Learning Methods
Source: Biomed Res Int. 2022 Dec 28;2022:5297235. doi: 10.1155/2022/5297235 (PMC9812612; doi:10.1155/2022/5297235)
Supplement: Supplementary Materials — Table S1: fifty-nine sarcoma subtypes and their sample sizes. Table S2: feature ranking results obtained using LASSO, LightGBM, and MCFS. Table S3: performance of IFS with different classification algorithms on three feature lists. Table S4: gene symbols obtained by annotating the most essential methylation sites derived from the LASSO, LightGBM, and MCFS feature lists. Table S5: intersection of three gene sets annotated by most essential methylation sites extracted from the LASSO, LightGBM, and MCFS feature lists. The genes that appear in the 3, 2, and 1 gene subsets are shown. Table S6: classification rules generated by decision tree using its optimal features on three feature lists. [file 5297235.f1.zip › Table S5 (1).pdf]

Table S5: Intersection of three gene sets annotated by most essential methylation sites extracted from the LASSO, LightGBM, and MCFS feature lists. The genes that appear in the 3, 2, and 1 gene subsets are shown.

Genes in three sets	Genes in two sets	Genes in one set
PRKAR1B	INPP5A	PKP4
	CTBP2	TXNRD2
	ASAP2	NHEJ1
	PHOSPHO1	TBC1D16
	C7orf50	RAB2A
	GLI3	LOC728264
	WWP2	OTUD7B
	ABLIM1	PMVK
	NFATC1	ADAM10
	LDLR	SEC14L1
	RARA	ANXA11
	CCND1	SCAP
	SNX29	TBX4
	TBX15	USP3
	TNRC18	PAPOLA
	IL13	PCCA
	MYO18B	TUBB1
	NPFFR2	RBPMS
	CD109	FAM178B
	MCC	C15orf61
	NOS1	PCGF3
	KIF19	MGMT
		HLTF
		TIAM2
		BBS9
		ESPNP
		NCOR2
		ENO3
		LTBP3
		NFIC
		ZC3H3
		BAHCC1
		C1orf83
		ANKRD11
		LIPC
		FLJ43663
		VRK3
		AUTS2

		CDK10
		CFLAR
		MAFK
		PDS5A
		LONRF1
		FBXL7
		CTDSPL
		C6orf129
		ADAR
		TRIM26
		TENC1
		C16orf45
		SEMA3B
		TSPAN5
		GPR137B
		ZMYND10
		S100A5
		PCOLCE
		PBX2
		LGALS8
		ZMAT3
		WDR27
		MACROD1
		SP140L
		REPS1
		PITRM1
		MAD1L1
		WDR60
		NSMCE2
		ACACA
		MSRA
		NCRNA00171
		SNX33
		LRP5
		ANO2
		NUDT16P
		SND1
		SH3RF3
		PARD3B
		CLUAP1
		CLPTM1L
		GRB2
		KRT18

		SFRS8
		TLE2
		ASXL3
		LCK
		ST6GALNAC4
		GLG1
		LRRC27
		RALB
		PIGZ
		RAD51L1
		ECSCR
		HIRA
		CCDC12
		MEF2D
		CDYL
		KIF25
		DYSF
		AGPAT3
		SRBD1
		IER2
		ROPN1L
		SIN3B
		RGS5
		TPO
		RPTOR
		ARTN
		NELL2
		TBC1D4
		ZNFX1
		SEPT13
		CUL3
		MMP11
		RAB11FIP3
		MXI1
		CCNY
		LETM1
		PPP1R9B
		PACS2
		ZMIZ1
		XPR1
		HAPLN2
		C9orf3
		SLC37A3

		RGS3
		RHOU
		LYST
		LBX2
		PPP2R5E
		CHFR
		N4BP1
		C5orf52
		ITGA6
		SLC39A11
		ZBTB20
		NCRNA00162
		IRS2
		COL1A1
		UST
		APLP2
		SHANK1
		RDH16
		SORCS2
		HES6
		CUX1
		FNBP1L
		BRE
		MAPKAP1
		ST3GAL3
		FGFRL1
		WDFY2
		RBM5
		MNT
		APOL1
		HK1
		NOS1AP
		ZNRF3
		LOC100129637
		DOCK1
		MEOX1
		VGLL4
		SLC17A3
		RBP2
		SYNCRIP
		RCBTB2
		OSBPL5
		RIPK4

		CBX2
		TSPAN14
		TBCD
		SH3PXD2B
		ALDOA
		PCSK7
		FIS1
		ITSN1
		LOC100133985
		PPP3CC
		CHD1
		NOTCH3
		NAT8L
		RAB40B
		MSI2
		ABR
		TPM1
		ADARB2
		BANF2
		DSTYK
		EEPD1
		ZBTB47
		STK39
		ADAMTSL2
		LOC728743
		ABHD6
		CIT
		FAM83A
		NET1
		PTPN21
		DDX17
		EI24
		ATP6V0A1
		ARHGEF4
		RBM24
		SEPT9
		EBF3
		WASF2
		RELL1
		FITM1
		DBNDD1
		BTBD11
		LRP3

		KLHL29
		MAP7D1
		POLRMT
		TCF7L2
		CANX
		CDGAP
		KIAA0174
		ANO1
		STXBP5
		PDE8A
		FLNB
		LITAF
		ETV6
		ITSN2
		PPARGC1A
		MAMSTR
		TK2
		CUGBP2
		OSBPL1A
		CBFA2T3
		ZNF788
		APOL3
		MYT1L
		DHRS7
		JAK1
		ARNT2
		HEXDC
		CHD9
		FBXW8
		USP39
