# Supplementary material for: Identification of Methylation Signatures and Rules for Sarcoma Subtypes by Machine Learning Methods
Source: Biomed Res Int. 2022 Dec 28;2022:5297235. doi: 10.1155/2022/5297235 (PMC9812612; doi:10.1155/2022/5297235)
Supplement: Supplementary Materials — Table S1: fifty-nine sarcoma subtypes and their sample sizes. Table S2: feature ranking results obtained using LASSO, LightGBM, and MCFS. Table S3: performance of IFS with different classification algorithms on three feature lists. Table S4: gene symbols obtained by annotating the most essential methylation sites derived from the LASSO, LightGBM, and MCFS feature lists. Table S5: intersection of three gene sets annotated by most essential methylation sites extracted from the LASSO, LightGBM, and MCFS feature lists. The genes that appear in the 3, 2, and 1 gene subsets are shown. Table S6: classification rules generated by decision tree using its optimal features on three feature lists. [file 5297235.f1.zip › Table S6 (1).pdf]

Table S6: Classification rules generated by decision tree using its optimal features on three feature lists.

(1) Rules on the LASSO feature list

Rules_0	passed counts:72
node_0: feature_name=cg11915444	feature_id[4116].value <= threshold=0.3402601182460785
node_1: feature_name=cg00982952	feature_id[1071].value <= threshold=0.7386334538459778
Class: Ewing sarcoma (EWING)	
Rules_1	passed counts:70
node_0: feature_name=cg11915444	feature_id[4116].value > threshold=0.3402601182460785
node_10: feature_name=cg12109728	feature_id[4182].value > threshold=0.7068270146846771
node_20: feature_name=cg10844382	feature_id[3856].value > threshold=0.5988894104957581
node_24: feature_name=cg00631702	feature_id[943].value > threshold=0.37470583617687225
node_352: feature_name=cg07027613	feature_id[549].value > threshold=0.22842608392238617
node_360: feature_name=cg18406924	feature_id[5844].value > threshold=0.3586410731077194
Class: high-grade conventional osteosarcoma (OS (HG))	
Rules_2	passed counts:67
node_0: feature_name=cg11915444	feature_id[4116].value > threshold=0.3402601182460785
node_10: feature_name=cg12109728	feature_id[4182].value <= threshold=0.7068270146846771
node_11: feature_name=cg23902076	feature_id[7131].value <= threshold=0.8691235482692719
Class: chordoma (CHORD)	
Rules_3	passed counts:64
node_0: feature_name=cg11915444	feature_id[4116].value > threshold=0.3402601182460785
node_10: feature_name=cg12109728	feature_id[4182].value > threshold=0.7068270146846771
node_20: feature_name=cg10844382	feature_id[3856].value > threshold=0.5988894104957581

node_24: feature_name=cg00631702	feature_id[943].value <= threshold=0.37470583617687225
node_25: feature_name=cg07912144	feature_id[3075].value > threshold=0.5578519105911255
node_29: feature_name=cg24407065	feature_id[388].value > threshold=0.5364363789558411
node_33: feature_name=cg15275017	feature_id[5069].value > threshold=0.4538573920726776
node_51: feature_name=cg07281938	feature_id[351].value <= threshold=0.7506992518901825
node_52: feature_name=cg21189849	feature_id[6504].value <= threshold=0.7545044124126434
node_53: feature_name=cg11717552	feature_id[4060].value > threshold=0.8407479226589203
Class: chondrosarcoma (CSA)	
Rules_4	passed counts:62
node_0: feature_name=cg11915444	feature_id[4116].value > threshold=0.3402601182460785
node_10: feature_name=cg12109728	feature_id[4182].value > threshold=0.7068270146846771
node_20: feature_name=cg10844382	feature_id[3856].value > threshold=0.5988894104957581
node_24: feature_name=cg00631702	feature_id[943].value <= threshold=0.37470583617687225
node_25: feature_name=cg07912144	feature_id[3075].value > threshold=0.5578519105911255
node_29: feature_name=cg24407065	feature_id[388].value > threshold=0.5364363789558411
node_33: feature_name=cg15275017	feature_id[5069].value <= threshold=0.4538573920726776
node_34: feature_name=cg08236537	feature_id[3160].value > threshold=0.4830351173877716
node_46: feature_name=cg13287523	feature_id[632].value > threshold=0.6652203798294067
Class: leiomyosarcoma (LMS)	
Rules_5	passed counts:62
node_0: feature_name=cg11915444	feature_id[4116].value > threshold=0.3402601182460785
node_10: feature_name=cg12109728	feature_id[4182].value > threshold=0.7068270146846771

node_20: feature_name=cg10844382	feature_id[3856].value <= threshold=0.5988894104957581
node_21: feature_name=cg02563156	feature_id[1540].value > threshold=0.6083262264728546
Class: alveolar rhabdomyosarcoma (RMS (ALV))	
Rules_6	passed counts:56
node_0: feature_name=cg11915444	feature_id[4116].value > threshold=0.3402601182460785
node_10: feature_name=cg12109728	feature_id[4182].value > threshold=0.7068270146846771
node_20: feature_name=cg10844382	feature_id[3856].value > threshold=0.5988894104957581
node_24: feature_name=cg00631702	feature_id[943].value <= threshold=0.37470583617687225
node_25: feature_name=cg07912144	feature_id[3075].value <= threshold=0.5578519105911255
node_26: feature_name=cg07478111	feature_id[2959].value > threshold=0.7341253161430359
Class: synovial sarcoma (SYSA)	
Rules_7	passed counts:49
node_0: feature_name=cg11915444	feature_id[4116].value > threshold=0.3402601182460785
node_10: feature_name=cg12109728	feature_id[4182].value > threshold=0.7068270146846771
node_20: feature_name=cg10844382	feature_id[3856].value > threshold=0.5988894104957581
node_24: feature_name=cg00631702	feature_id[943].value <= threshold=0.37470583617687225
node_25: feature_name=cg07912144	feature_id[3075].value > threshold=0.5578519105911255
node_29: feature_name=cg24407065	feature_id[388].value <= threshold=0.5364363789558411
node_30: feature_name=cg03300805	feature_id[1780].value > threshold=0.03215229045599699
Class: gastrointestinal stromal tumour (GIST)	
Rules_8	passed counts:42
node_0: feature_name=cg11915444	feature_id[4116].value > threshold=0.3402601182460785
node_10: feature_name=cg12109728	feature_id[4182].value > threshold=0.7068270146846771

node_20: feature_name=cg10844382	feature_id[3856].value > threshold=0.5988894104957581
node_24: feature_name=cg00631702	feature_id[943].value <= threshold=0.37470583617687225
node_25: feature_name=cg07912144	feature_id[3075].value > threshold=0.5578519105911255
node_29: feature_name=cg24407065	feature_id[388].value > threshold=0.5364363789558411
node_33: feature_name=cg15275017	feature_id[5069].value > threshold=0.4538573920726776
node_51: feature_name=cg07281938	feature_id[351].value > threshold=0.7506992518901825
node_73: feature_name=cg17524821	feature_id[5626].value <= threshold=0.4504699558019638
Class: desmoplastic small round cell tumour (DSRCT)	
Rules_9	passed counts:40
node_0: feature_name=cg11915444	feature_id[4116].value > threshold=0.3402601182460785
node_10: feature_name=cg12109728	feature_id[4182].value > threshold=0.7068270146846771
node_20: feature_name=cg10844382	feature_id[3856].value > threshold=0.5988894104957581
node_24: feature_name=cg00631702	feature_id[943].value <= threshold=0.37470583617687225
node_25: feature_name=cg07912144	feature_id[3075].value > threshold=0.5578519105911255
node_29: feature_name=cg24407065	feature_id[388].value > threshold=0.5364363789558411
node_33: feature_name=cg15275017	feature_id[5069].value > threshold=0.4538573920726776
node_51: feature_name=cg07281938	feature_id[351].value > threshold=0.7506992518901825
node_73: feature_name=cg17524821	feature_id[5626].value > threshold=0.4504699558019638
node_75: feature_name=cg02973735	feature_id[1677].value > threshold=0.29383400082588196
node_77: feature_name=cg23345038	feature_id[7002].value > threshold=0.6255057156085968
node_79: feature_name=cg17537493	feature_id[5630].value <= threshold=0.5825372636318207
node_80: feature_name=cg07990390	feature_id[515].value <= threshold=0.7988054752349854

Class: angiosarcoma (AS)	
Rules_10	passed counts:39
node_0: feature_name=cg11915444	feature_id[4116].value > threshold=0.3402601182460785
node_10: feature_name=cg12109728	feature_id[4182].value > threshold=0.7068270146846771
node_20: feature_name=cg10844382	feature_id[3856].value > threshold=0.5988894104957581
node_24: feature_name=cg00631702	feature_id[943].value <= threshold=0.37470583617687225
node_25: feature_name=cg07912144	feature_id[3075].value > threshold=0.5578519105911255
node_29: feature_name=cg24407065	feature_id[388].value > threshold=0.5364363789558411
node_33: feature_name=cg15275017	feature_id[5069].value > threshold=0.4538573920726776
node_51: feature_name=cg07281938	feature_id[351].value > threshold=0.7506992518901825
node_73: feature_name=cg17524821	feature_id[5626].value > threshold=0.4504699558019638
node_75: feature_name=cg02973735	feature_id[1677].value <= threshold=0.29383400082588196
Class: dermatofibrosarcoma protuberans (DFSP)	
Rules_11	passed counts:38
node_0: feature_name=cg11915444	feature_id[4116].value > threshold=0.3402601182460785
node_10: feature_name=cg12109728	feature_id[4182].value > threshold=0.7068270146846771
node_20: feature_name=cg10844382	feature_id[3856].value > threshold=0.5988894104957581
node_24: feature_name=cg00631702	feature_id[943].value <= threshold=0.37470583617687225
node_25: feature_name=cg07912144	feature_id[3075].value > threshold=0.5578519105911255
node_29: feature_name=cg24407065	feature_id[388].value > threshold=0.5364363789558411
node_33: feature_name=cg15275017	feature_id[5069].value > threshold=0.4538573920726776
node_51: feature_name=cg07281938	feature_id[351].value > threshold=0.7506992518901825

node_73: feature_name=cg17524821	feature_id[5626].value > threshold=0.4504699558019638
node_75: feature_name=cg02973735	feature_id[1677].value > threshold=0.29383400082588196
node_77: feature_name=cg23345038	feature_id[7002].value > threshold=0.6255057156085968
node_79: feature_name=cg17537493	feature_id[5630].value > threshold=0.5825372636318207
node_85: feature_name=cg12948116	feature_id[4375].value > threshold=0.5658791363239288
node_347: feature_name=cg15262242	feature_id[5064].value <= threshold=0.36207813024520874
Class: embryonal rhabdomyosarcoma (RMS (EMB))	
Rules_12	passed counts:38
node_0: feature_name=cg11915444	feature_id[4116].value > threshold=0.3402601182460785
node_10: feature_name=cg12109728	feature_id[4182].value > threshold=0.7068270146846771
node_20: feature_name=cg10844382	feature_id[3856].value > threshold=0.5988894104957581
node_24: feature_name=cg00631702	feature_id[943].value <= threshold=0.37470583617687225
node_25: feature_name=cg07912144	feature_id[3075].value > threshold=0.5578519105911255
node_29: feature_name=cg24407065	feature_id[388].value > threshold=0.5364363789558411
node_33: feature_name=cg15275017	feature_id[5069].value > threshold=0.4538573920726776
node_51: feature_name=cg07281938	feature_id[351].value > threshold=0.7506992518901825
node_73: feature_name=cg17524821	feature_id[5626].value > threshold=0.4504699558019638
node_75: feature_name=cg02973735	feature_id[1677].value > threshold=0.29383400082588196
node_77: feature_name=cg23345038	feature_id[7002].value <= threshold=0.6255057156085968
Class: myxoid liposarcoma (MLS)	
Rules_13	passed counts:34
node_0: feature_name=cg11915444	feature_id[4116].value > threshold=0.3402601182460785

node_10: feature_name=cg12109728	feature_id[4182].value > threshold=0.7068270146846771
node_20: feature_name=cg10844382	feature_id[3856].value > threshold=0.5988894104957581
node_24: feature_name=cg00631702	feature_id[943].value <= threshold=0.37470583617687225
node_25: feature_name=cg07912144	feature_id[3075].value > threshold=0.5578519105911255
node_29: feature_name=cg24407065	feature_id[388].value > threshold=0.5364363789558411
node_33: feature_name=cg15275017	feature_id[5069].value > threshold=0.4538573920726776
node_51: feature_name=cg07281938	feature_id[351].value > threshold=0.7506992518901825
node_73: feature_name=cg17524821	feature_id[5626].value > threshold=0.4504699558019638
node_75: feature_name=cg02973735	feature_id[1677].value > threshold=0.29383400082588196
node_77: feature_name=cg23345038	feature_id[7002].value > threshold=0.6255057156085968
node_79: feature_name=cg17537493	feature_id[5630].value > threshold=0.5825372636318207
node_85: feature_name=cg12948116	feature_id[4375].value <= threshold=0.5658791363239288
node_86: feature_name=cg06038180	feature_id[2529].value > threshold=0.43877437710762024
node_88: feature_name=cg15720017	feature_id[5175].value > threshold=0.7009969055652618
node_90: feature_name=cg13070215	feature_id[314].value <= threshold=0.14645694941282272
node_91: feature_name=cg01014262	feature_id[1082].value <= threshold=0.5737917125225067
node_92: feature_name=cg16863382	feature_id[5471].value > threshold=0.7200668156147003
node_102: feature_name=cg06633739	feature_id[2686].value > threshold=0.47115950286388397
node_126: feature_name=cg22946562	feature_id[744].value <= threshold=0.6194809079170227
node_127: feature_name=cg23920016	feature_id[7139].value <= threshold=0.7449243068695068
node_128: feature_name=cg16521032	feature_id[5379].value > threshold=0.8948074877262115
node_136: feature_name=cg22345063	feature_id[6755].value <= threshold=0.38597847521305084

node_137: feature_name=cg24033503	feature_id[7174].value > threshold=0.9229673147201538
Class: undifferentiated sarcoma (USARC)	
Rules_14	passed counts:30
node_0: feature_name=cg11915444	feature_id[4116].value > threshold=0.3402601182460785
node_10: feature_name=cg12109728	feature_id[4182].value > threshold=0.7068270146846771
node_20: feature_name=cg10844382	feature_id[3856].value > threshold=0.5988894104957581
node_24: feature_name=cg00631702	feature_id[943].value <= threshold=0.37470583617687225
node_25: feature_name=cg07912144	feature_id[3075].value > threshold=0.5578519105911255
node_29: feature_name=cg24407065	feature_id[388].value > threshold=0.5364363789558411
node_33: feature_name=cg15275017	feature_id[5069].value > threshold=0.4538573920726776
node_51: feature_name=cg07281938	feature_id[351].value > threshold=0.7506992518901825
node_73: feature_name=cg17524821	feature_id[5626].value > threshold=0.4504699558019638
node_75: feature_name=cg02973735	feature_id[1677].value > threshold=0.29383400082588196
node_77: feature_name=cg23345038	feature_id[7002].value > threshold=0.6255057156085968
node_79: feature_name=cg17537493	feature_id[5630].value > threshold=0.5825372636318207
node_85: feature_name=cg12948116	feature_id[4375].value <= threshold=0.5658791363239288
node_86: feature_name=cg06038180	feature_id[2529].value > threshold=0.43877437710762024
node_88: feature_name=cg15720017	feature_id[5175].value <= threshold=0.7009969055652618
Class: solitary fibrous tumour (SFT)	
Rules_15	passed counts:30
node_0: feature_name=cg11915444	feature_id[4116].value > threshold=0.3402601182460785
node_10: feature_name=cg12109728	feature_id[4182].value > threshold=0.7068270146846771

node_20: feature_name=cg10844382	feature_id[3856].value > threshold=0.5988894104957581
node_24: feature_name=cg00631702	feature_id[943].value <= threshold=0.37470583617687225
node_25: feature_name=cg07912144	feature_id[3075].value > threshold=0.5578519105911255
node_29: feature_name=cg24407065	feature_id[388].value > threshold=0.5364363789558411
node_33: feature_name=cg15275017	feature_id[5069].value > threshold=0.4538573920726776
node_51: feature_name=cg07281938	feature_id[351].value > threshold=0.7506992518901825
node_73: feature_name=cg17524821	feature_id[5626].value > threshold=0.4504699558019638
node_75: feature_name=cg02973735	feature_id[1677].value > threshold=0.29383400082588196
node_77: feature_name=cg23345038	feature_id[7002].value > threshold=0.6255057156085968
node_79: feature_name=cg17537493	feature_id[5630].value > threshold=0.5825372636318207
node_85: feature_name=cg12948116	feature_id[4375].value <= threshold=0.5658791363239288
node_86: feature_name=cg06038180	feature_id[2529].value <= threshold=0.43877437710762024
Class: schwannoma (SWN)	
Rules_16	passed counts:29
node_0: feature_name=cg11915444	feature_id[4116].value > threshold=0.3402601182460785
node_10: feature_name=cg12109728	feature_id[4182].value > threshold=0.7068270146846771
node_20: feature_name=cg10844382	feature_id[3856].value > threshold=0.5988894104957581
node_24: feature_name=cg00631702	feature_id[943].value <= threshold=0.37470583617687225
node_25: feature_name=cg07912144	feature_id[3075].value > threshold=0.5578519105911255
node_29: feature_name=cg24407065	feature_id[388].value > threshold=0.5364363789558411
node_33: feature_name=cg15275017	feature_id[5069].value > threshold=0.4538573920726776
node_51: feature_name=cg07281938	feature_id[351].value > threshold=0.7506992518901825

node_73: feature_name=cg17524821	feature_id[5626].value > threshold=0.4504699558019638
node_75: feature_name=cg02973735	feature_id[1677].value > threshold=0.29383400082588196
node_77: feature_name=cg23345038	feature_id[7002].value > threshold=0.6255057156085968
node_79: feature_name=cg17537493	feature_id[5630].value > threshold=0.5825372636318207
node_85: feature_name=cg12948116	feature_id[4375].value <= threshold=0.5658791363239288
node_86: feature_name=cg06038180	feature_id[2529].value > threshold=0.43877437710762024
node_88: feature_name=cg15720017	feature_id[5175].value > threshold=0.7009969055652618
node_90: feature_name=cg13070215	feature_id[314].value <= threshold=0.14645694941282272
node_91: feature_name=cg01014262	feature_id[1082].value <= threshold=0.5737917125225067
node_92: feature_name=cg16863382	feature_id[5471].value > threshold=0.7200668156147003
node_102: feature_name=cg06633739	feature_id[2686].value > threshold=0.47115950286388397
node_126: feature_name=cg22946562	feature_id[744].value > threshold=0.6194809079170227
node_170: feature_name=cg12120430	feature_id[4185].value > threshold=0.5775820016860962
node_172: feature_name=cg02137970	feature_id[1411].value <= threshold=0.6705857813358307
node_173: feature_name=cg16185457	feature_id[5282].value > threshold=0.5055856853723526
node_183: feature_name=cg04993279	feature_id[2243].value <= threshold=0.46021124720573425
node_184: feature_name=cg04655520	feature_id[2141].value > threshold=0.08191334456205368
Class: well differentiated liposarcoma (WDLS)/dedifferentiated liposarcoma (DDLS)	
Rules_17	passed counts:28
node_0: feature_name=cg11915444	feature_id[4116].value > threshold=0.3402601182460785
node_10: feature_name=cg12109728	feature_id[4182].value > threshold=0.7068270146846771
node_20: feature_name=cg10844382	feature_id[3856].value > threshold=0.5988894104957581

node_24: feature_name=cg00631702	feature_id[943].value <= threshold=0.37470583617687225
node_25: feature_name=cg07912144	feature_id[3075].value > threshold=0.5578519105911255
node_29: feature_name=cg24407065	feature_id[388].value > threshold=0.5364363789558411
node_33: feature_name=cg15275017	feature_id[5069].value > threshold=0.4538573920726776
node_51: feature_name=cg07281938	feature_id[351].value > threshold=0.7506992518901825
node_73: feature_name=cg17524821	feature_id[5626].value > threshold=0.4504699558019638
node_75: feature_name=cg02973735	feature_id[1677].value > threshold=0.29383400082588196
node_77: feature_name=cg23345038	feature_id[7002].value > threshold=0.6255057156085968
node_79: feature_name=cg17537493	feature_id[5630].value > threshold=0.5825372636318207
node_85: feature_name=cg12948116	feature_id[4375].value <= threshold=0.5658791363239288
node_86: feature_name=cg06038180	feature_id[2529].value > threshold=0.43877437710762024
node_88: feature_name=cg15720017	feature_id[5175].value > threshold=0.7009969055652618
node_90: feature_name=cg13070215	feature_id[314].value > threshold=0.14645694941282272
node_340: feature_name=cg06272543	feature_id[2590].value > threshold=0.07370422407984734
Class: epithelioid sarcoma (ES)	
Rules_18	passed counts:26
node_0: feature_name=cg11915444	feature_id[4116].value > threshold=0.3402601182460785
node_10: feature_name=cg12109728	feature_id[4182].value > threshold=0.7068270146846771
node_20: feature_name=cg10844382	feature_id[3856].value > threshold=0.5988894104957581
node_24: feature_name=cg00631702	feature_id[943].value <= threshold=0.37470583617687225
node_25: feature_name=cg07912144	feature_id[3075].value > threshold=0.5578519105911255
node_29: feature_name=cg24407065	feature_id[388].value > threshold=0.5364363789558411

node_33: feature_name=cg15275017	feature_id[5069].value > threshold=0.4538573920726776
node_51: feature_name=cg07281938	feature_id[351].value > threshold=0.7506992518901825
node_73: feature_name=cg17524821	feature_id[5626].value > threshold=0.4504699558019638
node_75: feature_name=cg02973735	feature_id[1677].value > threshold=0.29383400082588196
node_77: feature_name=cg23345038	feature_id[7002].value > threshold=0.6255057156085968
node_79: feature_name=cg17537493	feature_id[5630].value > threshold=0.5825372636318207
node_85: feature_name=cg12948116	feature_id[4375].value <= threshold=0.5658791363239288
node_86: feature_name=cg06038180	feature_id[2529].value > threshold=0.43877437710762024
node_88: feature_name=cg15720017	feature_id[5175].value > threshold=0.7009969055652618
node_90: feature_name=cg13070215	feature_id[314].value <= threshold=0.14645694941282272
node_91: feature_name=cg01014262	feature_id[1082].value <= threshold=0.5737917125225067
node_92: feature_name=cg16863382	feature_id[5471].value > threshold=0.7200668156147003
node_102: feature_name=cg06633739	feature_id[2686].value <= threshold=0.47115950286388397
node_103: feature_name=cg03861097	feature_id[1943].value > threshold=0.24196631461381912
node_123: feature_name=cg11468635	feature_id[181].value <= threshold=0.9009453654289246
Class: malignant peripheral nerve sheath tumour (MPNST)	
Rules_19	passed counts:23
node_0: feature_name=cg11915444	feature_id[4116].value > threshold=0.3402601182460785
node_10: feature_name=cg12109728	feature_id[4182].value > threshold=0.7068270146846771
node_20: feature_name=cg10844382	feature_id[3856].value > threshold=0.5988894104957581
node_24: feature_name=cg00631702	feature_id[943].value <= threshold=0.37470583617687225
node_25: feature_name=cg07912144	feature_id[3075].value > threshold=0.5578519105911255

node_29: feature_name=cg24407065	feature_id[388].value > threshold=0.5364363789558411
node_33: feature_name=cg15275017	feature_id[5069].value > threshold=0.4538573920726776
node_51: feature_name=cg07281938	feature_id[351].value > threshold=0.7506992518901825
node_73: feature_name=cg17524821	feature_id[5626].value > threshold=0.4504699558019638
node_75: feature_name=cg02973735	feature_id[1677].value > threshold=0.29383400082588196
node_77: feature_name=cg23345038	feature_id[7002].value > threshold=0.6255057156085968
node_79: feature_name=cg17537493	feature_id[5630].value > threshold=0.5825372636318207
node_85: feature_name=cg12948116	feature_id[4375].value <= threshold=0.5658791363239288
node_86: feature_name=cg06038180	feature_id[2529].value > threshold=0.43877437710762024
node_88: feature_name=cg15720017	feature_id[5175].value > threshold=0.7009969055652618
node_90: feature_name=cg13070215	feature_id[314].value <= threshold=0.14645694941282272
node_91: feature_name=cg01014262	feature_id[1082].value <= threshold=0.5737917125225067
node_92: feature_name=cg16863382	feature_id[5471].value <= threshold=0.7200668156147003
node_93: feature_name=cg18601229	feature_id[5883].value > threshold=0.7762220203876495
Class: alveolar soft part sarcoma (ASPS)	
Rules_20	passed counts:22
node_0: feature_name=cg11915444	feature_id[4116].value > threshold=0.3402601182460785
node_10: feature_name=cg12109728	feature_id[4182].value > threshold=0.7068270146846771
node_20: feature_name=cg10844382	feature_id[3856].value > threshold=0.5988894104957581
node_24: feature_name=cg00631702	feature_id[943].value <= threshold=0.37470583617687225
node_25: feature_name=cg07912144	feature_id[3075].value > threshold=0.5578519105911255
node_29: feature_name=cg24407065	feature_id[388].value > threshold=0.5364363789558411

node_33: feature_name=cg15275017	feature_id[5069].value > threshold=0.4538573920726776
node_51: feature_name=cg07281938	feature_id[351].value > threshold=0.7506992518901825
node_73: feature_name=cg17524821	feature_id[5626].value > threshold=0.4504699558019638
node_75: feature_name=cg02973735	feature_id[1677].value > threshold=0.29383400082588196
node_77: feature_name=cg23345038	feature_id[7002].value > threshold=0.6255057156085968
node_79: feature_name=cg17537493	feature_id[5630].value > threshold=0.5825372636318207
node_85: feature_name=cg12948116	feature_id[4375].value <= threshold=0.5658791363239288
node_86: feature_name=cg06038180	feature_id[2529].value > threshold=0.43877437710762024
node_88: feature_name=cg15720017	feature_id[5175].value > threshold=0.7009969055652618
node_90: feature_name=cg13070215	feature_id[314].value <= threshold=0.14645694941282272
node_91: feature_name=cg01014262	feature_id[1082].value <= threshold=0.5737917125225067
node_92: feature_name=cg16863382	feature_id[5471].value > threshold=0.7200668156147003
node_102: feature_name=cg06633739	feature_id[2686].value > threshold=0.47115950286388397
node_126: feature_name=cg22946562	feature_id[744].value > threshold=0.6194809079170227
node_170: feature_name=cg12120430	feature_id[4185].value > threshold=0.5775820016860962
node_172: feature_name=cg02137970	feature_id[1411].value > threshold=0.6705857813358307
node_196: feature_name=cg03393445	feature_id[1807].value <= threshold=0.4260125905275345
node_197: feature_name=cg01297744	feature_id[1157].value > threshold=0.8005762100219727
node_211: feature_name=cg14401746	feature_id[4834].value <= threshold=0.3708716481924057
Class: small blue round cell tumour (SBRCT)	
Rules_21	passed counts:21
node_0: feature_name=cg11915444	feature_id[4116].value > threshold=0.3402601182460785

node_10: feature_name=cg12109728	feature_id[4182].value > threshold=0.7068270146846771
node_20: feature_name=cg10844382	feature_id[3856].value > threshold=0.5988894104957581
node_24: feature_name=cg00631702	feature_id[943].value <= threshold=0.37470583617687225
node_25: feature_name=cg07912144	feature_id[3075].value > threshold=0.5578519105911255
node_29: feature_name=cg24407065	feature_id[388].value > threshold=0.5364363789558411
node_33: feature_name=cg15275017	feature_id[5069].value > threshold=0.4538573920726776
node_51: feature_name=cg07281938	feature_id[351].value > threshold=0.7506992518901825
node_73: feature_name=cg17524821	feature_id[5626].value > threshold=0.4504699558019638
node_75: feature_name=cg02973735	feature_id[1677].value > threshold=0.29383400082588196
node_77: feature_name=cg23345038	feature_id[7002].value > threshold=0.6255057156085968
node_79: feature_name=cg17537493	feature_id[5630].value > threshold=0.5825372636318207
node_85: feature_name=cg12948116	feature_id[4375].value <= threshold=0.5658791363239288
node_86: feature_name=cg06038180	feature_id[2529].value > threshold=0.43877437710762024
node_88: feature_name=cg15720017	feature_id[5175].value > threshold=0.7009969055652618
node_90: feature_name=cg13070215	feature_id[314].value <= threshold=0.14645694941282272
node_91: feature_name=cg01014262	feature_id[1082].value > threshold=0.5737917125225067
node_337: feature_name=cg19909613	feature_id[6202].value > threshold=0.8301863670349121
Class: malignant rhabdoid tumour (MRT)	
Rules_22	passed counts:14
node_0: feature_name=cg11915444	feature_id[4116].value > threshold=0.3402601182460785
node_10: feature_name=cg12109728	feature_id[4182].value > threshold=0.7068270146846771
node_20: feature_name=cg10844382	feature_id[3856].value > threshold=0.5988894104957581

node_24: feature_name=cg00631702	feature_id[943].value <= threshold=0.37470583617687225
node_25: feature_name=cg07912144	feature_id[3075].value > threshold=0.5578519105911255
node_29: feature_name=cg24407065	feature_id[388].value > threshold=0.5364363789558411
node_33: feature_name=cg15275017	feature_id[5069].value > threshold=0.4538573920726776
node_51: feature_name=cg07281938	feature_id[351].value > threshold=0.7506992518901825
node_73: feature_name=cg17524821	feature_id[5626].value > threshold=0.4504699558019638
node_75: feature_name=cg02973735	feature_id[1677].value > threshold=0.29383400082588196
node_77: feature_name=cg23345038	feature_id[7002].value > threshold=0.6255057156085968
node_79: feature_name=cg17537493	feature_id[5630].value > threshold=0.5825372636318207
node_85: feature_name=cg12948116	feature_id[4375].value <= threshold=0.5658791363239288
node_86: feature_name=cg06038180	feature_id[2529].value > threshold=0.43877437710762024
node_88: feature_name=cg15720017	feature_id[5175].value > threshold=0.7009969055652618
node_90: feature_name=cg13070215	feature_id[314].value <= threshold=0.14645694941282272
node_91: feature_name=cg01014262	feature_id[1082].value <= threshold=0.5737917125225067
node_92: feature_name=cg16863382	feature_id[5471].value > threshold=0.7200668156147003
node_102: feature_name=cg06633739	feature_id[2686].value > threshold=0.47115950286388397
node_126: feature_name=cg22946562	feature_id[744].value > threshold=0.6194809079170227
node_170: feature_name=cg12120430	feature_id[4185].value <= threshold=0.5775820016860962
Class: angioleiomyoma (ALMO)/myopericytoma (MPC)	
Rules_23	passed counts:13
node_0: feature_name=cg11915444	feature_id[4116].value > threshold=0.3402601182460785
node_10: feature_name=cg12109728	feature_id[4182].value > threshold=0.7068270146846771

node_20: feature_name=cg10844382	feature_id[3856].value > threshold=0.5988894104957581
node_24: feature_name=cg00631702	feature_id[943].value <= threshold=0.37470583617687225
node_25: feature_name=cg07912144	feature_id[3075].value > threshold=0.5578519105911255
node_29: feature_name=cg24407065	feature_id[388].value > threshold=0.5364363789558411
node_33: feature_name=cg15275017	feature_id[5069].value > threshold=0.4538573920726776
node_51: feature_name=cg07281938	feature_id[351].value > threshold=0.7506992518901825
node_73: feature_name=cg17524821	feature_id[5626].value > threshold=0.4504699558019638
node_75: feature_name=cg02973735	feature_id[1677].value > threshold=0.29383400082588196
node_77: feature_name=cg23345038	feature_id[7002].value > threshold=0.6255057156085968
node_79: feature_name=cg17537493	feature_id[5630].value > threshold=0.5825372636318207
node_85: feature_name=cg12948116	feature_id[4375].value <= threshold=0.5658791363239288
node_86: feature_name=cg06038180	feature_id[2529].value > threshold=0.43877437710762024
node_88: feature_name=cg15720017	feature_id[5175].value > threshold=0.7009969055652618
node_90: feature_name=cg13070215	feature_id[314].value <= threshold=0.14645694941282272
node_91: feature_name=cg01014262	feature_id[1082].value <= threshold=0.5737917125225067
node_92: feature_name=cg16863382	feature_id[5471].value > threshold=0.7200668156147003
node_102: feature_name=cg06633739	feature_id[2686].value > threshold=0.47115950286388397
node_126: feature_name=cg22946562	feature_id[744].value > threshold=0.6194809079170227
node_170: feature_name=cg12120430	feature_id[4185].value > threshold=0.5775820016860962
node_172: feature_name=cg02137970	feature_id[1411].value > threshold=0.6705857813358307
node_196: feature_name=cg03393445	feature_id[1807].value > threshold=0.4260125905275345
node_218: feature_name=cg23525541	feature_id[7049].value <= threshold=0.4485483318567276

node_219: feature_name=cg24964215	feature_id[7404].value > threshold=0.04320882819592953
Class: fibrous dysplasia (FDY)	
Rules_24	passed counts:13
node_0: feature_name=cg11915444	feature_id[4116].value > threshold=0.3402601182460785
node_10: feature_name=cg12109728	feature_id[4182].value > threshold=0.7068270146846771
node_20: feature_name=cg10844382	feature_id[3856].value > threshold=0.5988894104957581
node_24: feature_name=cg00631702	feature_id[943].value <= threshold=0.37470583617687225
node_25: feature_name=cg07912144	feature_id[3075].value > threshold=0.5578519105911255
node_29: feature_name=cg24407065	feature_id[388].value > threshold=0.5364363789558411
node_33: feature_name=cg15275017	feature_id[5069].value > threshold=0.4538573920726776
node_51: feature_name=cg07281938	feature_id[351].value > threshold=0.7506992518901825
node_73: feature_name=cg17524821	feature_id[5626].value > threshold=0.4504699558019638
node_75: feature_name=cg02973735	feature_id[1677].value > threshold=0.29383400082588196
node_77: feature_name=cg23345038	feature_id[7002].value > threshold=0.6255057156085968
node_79: feature_name=cg17537493	feature_id[5630].value > threshold=0.5825372636318207
node_85: feature_name=cg12948116	feature_id[4375].value <= threshold=0.5658791363239288
node_86: feature_name=cg06038180	feature_id[2529].value > threshold=0.43877437710762024
node_88: feature_name=cg15720017	feature_id[5175].value > threshold=0.7009969055652618
node_90: feature_name=cg13070215	feature_id[314].value <= threshold=0.14645694941282272
node_91: feature_name=cg01014262	feature_id[1082].value <= threshold=0.5737917125225067
node_92: feature_name=cg16863382	feature_id[5471].value > threshold=0.7200668156147003
node_102: feature_name=cg06633739	feature_id[2686].value > threshold=0.47115950286388397

node_126: feature_name=cg22946562	feature_id[744].value > threshold=0.6194809079170227
node_170: feature_name=cg12120430	feature_id[4185].value > threshold=0.5775820016860962
node_172: feature_name=cg02137970	feature_id[1411].value <= threshold=0.6705857813358307
node_173: feature_name=cg16185457	feature_id[5282].value <= threshold=0.5055856853723526
node_174: feature_name=cg09040552	feature_id[3370].value <= threshold=0.596051037311554
Class: desmoid-type fibromatosis (DTFM)	
Rules_25	passed counts:12
node_0: feature_name=cg11915444	feature_id[4116].value > threshold=0.3402601182460785
node_10: feature_name=cg12109728	feature_id[4182].value > threshold=0.7068270146846771
node_20: feature_name=cg10844382	feature_id[3856].value > threshold=0.5988894104957581
node_24: feature_name=cg00631702	feature_id[943].value <= threshold=0.37470583617687225
node_25: feature_name=cg07912144	feature_id[3075].value > threshold=0.5578519105911255
node_29: feature_name=cg24407065	feature_id[388].value > threshold=0.5364363789558411
node_33: feature_name=cg15275017	feature_id[5069].value > threshold=0.4538573920726776
node_51: feature_name=cg07281938	feature_id[351].value > threshold=0.7506992518901825
node_73: feature_name=cg17524821	feature_id[5626].value > threshold=0.4504699558019638
node_75: feature_name=cg02973735	feature_id[1677].value > threshold=0.29383400082588196
node_77: feature_name=cg23345038	feature_id[7002].value > threshold=0.6255057156085968
node_79: feature_name=cg17537493	feature_id[5630].value > threshold=0.5825372636318207
node_85: feature_name=cg12948116	feature_id[4375].value <= threshold=0.5658791363239288
node_86: feature_name=cg06038180	feature_id[2529].value > threshold=0.43877437710762024
node_88: feature_name=cg15720017	feature_id[5175].value > threshold=0.7009969055652618

node_90: feature_name=cg13070215	feature_id[314].value <= threshold=0.14645694941282272
node_91: feature_name=cg01014262	feature_id[1082].value <= threshold=0.5737917125225067
node_92: feature_name=cg16863382	feature_id[5471].value > threshold=0.7200668156147003
node_102: feature_name=cg06633739	feature_id[2686].value > threshold=0.47115950286388397
node_126: feature_name=cg22946562	feature_id[744].value > threshold=0.6194809079170227
node_170: feature_name=cg12120430	feature_id[4185].value > threshold=0.5775820016860962
node_172: feature_name=cg02137970	feature_id[1411].value > threshold=0.6705857813358307
node_196: feature_name=cg03393445	feature_id[1807].value > threshold=0.4260125905275345
node_218: feature_name=cg23525541	feature_id[7049].value > threshold=0.4485483318567276
node_222: feature_name=cg10543634	feature_id[3775].value > threshold=0.29505227506160736
node_224: feature_name=cg07344990	feature_id[2926].value > threshold=0.5750180780887604
node_226: feature_name=cg10558233	feature_id[3780].value <= threshold=0.7217690348625183
node_227: feature_name=cg08097359	feature_id[3116].value > threshold=0.3598180413246155
node_229: feature_name=cg22737001	feature_id[6843].value <= threshold=0.46281898021698
node_230: feature_name=cg24276624	feature_id[7242].value > threshold=0.6169979274272919
node_232: feature_name=cg04026354	feature_id[1982].value > threshold=0.42332448065280914
node_316: feature_name=cg23693485	feature_id[7083].value <= threshold=0.7056963741779327
node_317: feature_name=cg14583686	feature_id[4895].value > threshold=0.70423823595047
Class: inflammatory myofibroblastic tumour (IMT)	
Rules_26	passed counts:12
node_0: feature_name=cg11915444	feature_id[4116].value > threshold=0.3402601182460785
node_10: feature_name=cg12109728	feature_id[4182].value > threshold=0.7068270146846771

node_20:<br>feature_name=cg10844382	feature_id[3856].value ><br>threshold=0.5988894104957581
node_24:<br>feature_name=cg00631702	feature_id[943].value <=<br>threshold=0.37470583617687225
node_25:<br>feature_name=cg07912144	feature_id[3075].value ><br>threshold=0.5578519105911255
node_29:<br>feature_name=cg24407065	feature_id[388].value ><br>threshold=0.5364363789558411
node_33:<br>feature_name=cg15275017	feature_id[5069].value ><br>threshold=0.4538573920726776
node_51:<br>feature_name=cg07281938	feature_id[351].value ><br>threshold=0.7506992518901825
node_73:<br>feature_name=cg17524821	feature_id[5626].value ><br>threshold=0.4504699558019638
node_75:<br>feature_name=cg02973735	feature_id[1677].value ><br>threshold=0.29383400082588196
node_77:<br>feature_name=cg23345038	feature_id[7002].value ><br>threshold=0.6255057156085968
node_79:<br>feature_name=cg17537493	feature_id[5630].value ><br>threshold=0.5825372636318207
node_85:<br>feature_name=cg12948116	feature_id[4375].value <=<br>threshold=0.5658791363239288
node_86:<br>feature_name=cg06038180	feature_id[2529].value ><br>threshold=0.43877437710762024
node_88:<br>feature_name=cg15720017	feature_id[5175].value ><br>threshold=0.7009969055652618
node_90:<br>feature_name=cg13070215	feature_id[314].value <=<br>threshold=0.14645694941282272
node_91:<br>feature_name=cg01014262	feature_id[1082].value <=<br>threshold=0.5737917125225067
node_92:<br>feature_name=cg16863382	feature_id[5471].value ><br>threshold=0.7200668156147003
node_102:<br>feature_name=cg06633739	feature_id[2686].value ><br>threshold=0.47115950286388397
node_126:<br>feature_name=cg22946562	feature_id[744].value ><br>threshold=0.6194809079170227
node_170:<br>feature_name=cg12120430	feature_id[4185].value ><br>threshold=0.5775820016860962
node_172:<br>feature_name=cg02137970	feature_id[1411].value ><br>threshold=0.6705857813358307
node_196:<br>feature_name=cg03393445	feature_id[1807].value ><br>threshold=0.4260125905275345
node_218:<br>feature_name=cg23525541	feature_id[7049].value ><br>threshold=0.4485483318567276

node_222:<br>feature_name=cg10543634	feature_id[3775].value <=<br>threshold=0.29505227506160736
Class: ossifying fibromyxoid tumour (OFMT)	
Rules_27	passed counts:12
node_0:<br>feature_name=cg11915444	feature_id[4116].value ><br>threshold=0.3402601182460785
node_10:<br>feature_name=cg12109728	feature_id[4182].value ><br>threshold=0.7068270146846771
node_20:<br>feature_name=cg10844382	feature_id[3856].value ><br>threshold=0.5988894104957581
node_24:<br>feature_name=cg00631702	feature_id[943].value <=<br>threshold=0.37470583617687225
node_25:<br>feature_name=cg07912144	feature_id[3075].value ><br>threshold=0.5578519105911255
node_29:<br>feature_name=cg24407065	feature_id[388].value ><br>threshold=0.5364363789558411
node_33:<br>feature_name=cg15275017	feature_id[5069].value ><br>threshold=0.4538573920726776
node_51:<br>feature_name=cg07281938	feature_id[351].value ><br>threshold=0.7506992518901825
node_73:<br>feature_name=cg17524821	feature_id[5626].value ><br>threshold=0.4504699558019638
node_75:<br>feature_name=cg02973735	feature_id[1677].value ><br>threshold=0.29383400082588196
node_77:<br>feature_name=cg23345038	feature_id[7002].value ><br>threshold=0.6255057156085968
node_79:<br>feature_name=cg17537493	feature_id[5630].value ><br>threshold=0.5825372636318207
node_85:<br>feature_name=cg12948116	feature_id[4375].value <=<br>threshold=0.5658791363239288
node_86:<br>feature_name=cg06038180	feature_id[2529].value ><br>threshold=0.43877437710762024
node_88:<br>feature_name=cg15720017	feature_id[5175].value ><br>threshold=0.7009969055652618
node_90:<br>feature_name=cg13070215	feature_id[314].value <=<br>threshold=0.14645694941282272
node_91:<br>feature_name=cg01014262	feature_id[1082].value <=<br>threshold=0.5737917125225067
node_92:<br>feature_name=cg16863382	feature_id[5471].value ><br>threshold=0.7200668156147003
node_102:<br>feature_name=cg06633739	feature_id[2686].value ><br>threshold=0.47115950286388397

node_126: feature_name=cg22946562	feature_id[744].value > threshold=0.6194809079170227
node_170: feature_name=cg12120430	feature_id[4185].value > threshold=0.5775820016860962
node_172: feature_name=cg02137970	feature_id[1411].value > threshold=0.6705857813358307
node_196: feature_name=cg03393445	feature_id[1807].value <= threshold=0.4260125905275345
node_197: feature_name=cg01297744	feature_id[1157].value <= threshold=0.8005762100219727
node_198: feature_name=cg08831077	feature_id[3310].value > threshold=0.4210805594921112
Class: clear cell sarcoma of the kidney (CCSK)	
Rules_28	passed counts:11
node_0: feature_name=cg11915444	feature_id[4116].value > threshold=0.3402601182460785
node_10: feature_name=cg12109728	feature_id[4182].value > threshold=0.7068270146846771
node_20: feature_name=cg10844382	feature_id[3856].value > threshold=0.5988894104957581
node_24: feature_name=cg00631702	feature_id[943].value <= threshold=0.37470583617687225
node_25: feature_name=cg07912144	feature_id[3075].value > threshold=0.5578519105911255
node_29: feature_name=cg24407065	feature_id[388].value > threshold=0.5364363789558411
node_33: feature_name=cg15275017	feature_id[5069].value > threshold=0.4538573920726776
node_51: feature_name=cg07281938	feature_id[351].value > threshold=0.7506992518901825
node_73: feature_name=cg17524821	feature_id[5626].value > threshold=0.4504699558019638
node_75: feature_name=cg02973735	feature_id[1677].value > threshold=0.29383400082588196
node_77: feature_name=cg23345038	feature_id[7002].value > threshold=0.6255057156085968
node_79: feature_name=cg17537493	feature_id[5630].value > threshold=0.5825372636318207
node_85: feature_name=cg12948116	feature_id[4375].value <= threshold=0.5658791363239288
node_86: feature_name=cg06038180	feature_id[2529].value > threshold=0.43877437710762024

node_88: feature_name=cg15720017	feature_id[5175].value > threshold=0.7009969055652618
node_90: feature_name=cg13070215	feature_id[314].value <= threshold=0.14645694941282272
node_91: feature_name=cg01014262	feature_id[1082].value <= threshold=0.5737917125225067
node_92: feature_name=cg16863382	feature_id[5471].value > threshold=0.7200668156147003
node_102: feature_name=cg06633739	feature_id[2686].value > threshold=0.47115950286388397
node_126: feature_name=cg22946562	feature_id[744].value > threshold=0.6194809079170227
node_170: feature_name=cg12120430	feature_id[4185].value > threshold=0.5775820016860962
node_172: feature_name=cg02137970	feature_id[1411].value > threshold=0.6705857813358307
node_196: feature_name=cg03393445	feature_id[1807].value > threshold=0.4260125905275345
node_218: feature_name=cg23525541	feature_id[7049].value > threshold=0.4485483318567276
node_222: feature_name=cg10543634	feature_id[3775].value > threshold=0.29505227506160736
node_224: feature_name=cg07344990	feature_id[2926].value > threshold=0.5750180780887604
node_226: feature_name=cg10558233	feature_id[3780].value <= threshold=0.7217690348625183
node_227: feature_name=cg08097359	feature_id[3116].value > threshold=0.3598180413246155
node_229: feature_name=cg22737001	feature_id[6843].value <= threshold=0.46281898021698
node_230: feature_name=cg24276624	feature_id[7242].value > threshold=0.6169979274272919
node_232: feature_name=cg04026354	feature_id[1982].value > threshold=0.42332448065280914
node_316: feature_name=cg23693485	feature_id[7083].value > threshold=0.7056963741779327
node_320: feature_name=cg16027775	feature_id[5238].value <= threshold=0.17370137572288513
Class: angiomatoid fibrous histiocytoma (AFH)	
Rules_29	passed counts:11
node_0: feature_name=cg11915444	feature_id[4116].value > threshold=0.3402601182460785

node_10: feature_name=cg12109728	feature_id[4182].value > threshold=0.7068270146846771
node_20: feature_name=cg10844382	feature_id[3856].value > threshold=0.5988894104957581
node_24: feature_name=cg00631702	feature_id[943].value <= threshold=0.37470583617687225
node_25: feature_name=cg07912144	feature_id[3075].value > threshold=0.5578519105911255
node_29: feature_name=cg24407065	feature_id[388].value > threshold=0.5364363789558411
node_33: feature_name=cg15275017	feature_id[5069].value > threshold=0.4538573920726776
node_51: feature_name=cg07281938	feature_id[351].value > threshold=0.7506992518901825
node_73: feature_name=cg17524821	feature_id[5626].value > threshold=0.4504699558019638
node_75: feature_name=cg02973735	feature_id[1677].value > threshold=0.29383400082588196
node_77: feature_name=cg23345038	feature_id[7002].value > threshold=0.6255057156085968
node_79: feature_name=cg17537493	feature_id[5630].value > threshold=0.5825372636318207
node_85: feature_name=cg12948116	feature_id[4375].value <= threshold=0.5658791363239288
node_86: feature_name=cg06038180	feature_id[2529].value > threshold=0.43877437710762024
node_88: feature_name=cg15720017	feature_id[5175].value > threshold=0.7009969055652618
node_90: feature_name=cg13070215	feature_id[314].value <= threshold=0.14645694941282272
node_91: feature_name=cg01014262	feature_id[1082].value <= threshold=0.5737917125225067
node_92: feature_name=cg16863382	feature_id[5471].value > threshold=0.7200668156147003
node_102: feature_name=cg06633739	feature_id[2686].value > threshold=0.47115950286388397
node_126: feature_name=cg22946562	feature_id[744].value > threshold=0.6194809079170227
node_170: feature_name=cg12120430	feature_id[4185].value > threshold=0.5775820016860962
node_172: feature_name=cg02137970	feature_id[1411].value > threshold=0.6705857813358307
node_196: feature_name=cg03393445	feature_id[1807].value > threshold=0.4260125905275345

node_218: feature_name=cg23525541	feature_id[7049].value > threshold=0.4485483318567276
node_222: feature_name=cg10543634	feature_id[3775].value > threshold=0.29505227506160736
node_224: feature_name=cg07344990	feature_id[2926].value <= threshold=0.5750180780887604
Class: low-grade endometrial stromal sarcoma (ESS (LG))	
Rules_30	passed counts:10
node_0: feature_name=cg11915444	feature_id[4116].value > threshold=0.3402601182460785
node_10: feature_name=cg12109728	feature_id[4182].value > threshold=0.7068270146846771
node_20: feature_name=cg10844382	feature_id[3856].value > threshold=0.5988894104957581
node_24: feature_name=cg00631702	feature_id[943].value <= threshold=0.37470583617687225
node_25: feature_name=cg07912144	feature_id[3075].value > threshold=0.5578519105911255
node_29: feature_name=cg24407065	feature_id[388].value > threshold=0.5364363789558411
node_33: feature_name=cg15275017	feature_id[5069].value > threshold=0.4538573920726776
node_51: feature_name=cg07281938	feature_id[351].value > threshold=0.7506992518901825
node_73: feature_name=cg17524821	feature_id[5626].value > threshold=0.4504699558019638
node_75: feature_name=cg02973735	feature_id[1677].value > threshold=0.29383400082588196
node_77: feature_name=cg23345038	feature_id[7002].value > threshold=0.6255057156085968
node_79: feature_name=cg17537493	feature_id[5630].value > threshold=0.5825372636318207
node_85: feature_name=cg12948116	feature_id[4375].value <= threshold=0.5658791363239288
node_86: feature_name=cg06038180	feature_id[2529].value > threshold=0.43877437710762024
node_88: feature_name=cg15720017	feature_id[5175].value > threshold=0.7009969055652618
node_90: feature_name=cg13070215	feature_id[314].value <= threshold=0.14645694941282272
node_91: feature_name=cg01014262	feature_id[1082].value <= threshold=0.5737917125225067

node_92: feature_name=cg16863382	feature_id[5471].value > threshold=0.7200668156147003
node_102: feature_name=cg06633739	feature_id[2686].value > threshold=0.47115950286388397
node_126: feature_name=cg22946562	feature_id[744].value > threshold=0.6194809079170227
node_170: feature_name=cg12120430	feature_id[4185].value > threshold=0.5775820016860962
node_172: feature_name=cg02137970	feature_id[1411].value > threshold=0.6705857813358307
node_196: feature_name=cg03393445	feature_id[1807].value > threshold=0.4260125905275345
node_218: feature_name=cg23525541	feature_id[7049].value > threshold=0.4485483318567276
node_222: feature_name=cg10543634	feature_id[3775].value > threshold=0.29505227506160736
node_224: feature_name=cg07344990	feature_id[2926].value > threshold=0.5750180780887604
node_226: feature_name=cg10558233	feature_id[3780].value > threshold=0.7217690348625183
Class: control (CTRL)	
Rules_31	passed counts:10
node_0: feature_name=cg11915444	feature_id[4116].value > threshold=0.3402601182460785
node_10: feature_name=cg12109728	feature_id[4182].value > threshold=0.7068270146846771
node_20: feature_name=cg10844382	feature_id[3856].value > threshold=0.5988894104957581
node_24: feature_name=cg00631702	feature_id[943].value <= threshold=0.37470583617687225
node_25: feature_name=cg07912144	feature_id[3075].value > threshold=0.5578519105911255
node_29: feature_name=cg24407065	feature_id[388].value > threshold=0.5364363789558411
node_33: feature_name=cg15275017	feature_id[5069].value > threshold=0.4538573920726776
node_51: feature_name=cg07281938	feature_id[351].value > threshold=0.7506992518901825
node_73: feature_name=cg17524821	feature_id[5626].value > threshold=0.4504699558019638
node_75: feature_name=cg02973735	feature_id[1677].value > threshold=0.29383400082588196

node_77: feature_name=cg23345038	feature_id[7002].value > threshold=0.6255057156085968
node_79: feature_name=cg17537493	feature_id[5630].value > threshold=0.5825372636318207
node_85: feature_name=cg12948116	feature_id[4375].value <= threshold=0.5658791363239288
node_86: feature_name=cg06038180	feature_id[2529].value > threshold=0.43877437710762024
node_88: feature_name=cg15720017	feature_id[5175].value > threshold=0.7009969055652618
node_90: feature_name=cg13070215	feature_id[314].value <= threshold=0.14645694941282272
node_91: feature_name=cg01014262	feature_id[1082].value <= threshold=0.5737917125225067
node_92: feature_name=cg16863382	feature_id[5471].value > threshold=0.7200668156147003
node_102: feature_name=cg06633739	feature_id[2686].value > threshold=0.47115950286388397
node_126: feature_name=cg22946562	feature_id[744].value > threshold=0.6194809079170227
node_170: feature_name=cg12120430	feature_id[4185].value > threshold=0.5775820016860962
node_172: feature_name=cg02137970	feature_id[1411].value > threshold=0.6705857813358307
node_196: feature_name=cg03393445	feature_id[1807].value > threshold=0.4260125905275345
node_218: feature_name=cg23525541	feature_id[7049].value > threshold=0.4485483318567276
node_222: feature_name=cg10543634	feature_id[3775].value > threshold=0.29505227506160736
node_224: feature_name=cg07344990	feature_id[2926].value > threshold=0.5750180780887604
node_226: feature_name=cg10558233	feature_id[3780].value <= threshold=0.7217690348625183
node_227: feature_name=cg08097359	feature_id[3116].value > threshold=0.3598180413246155
node_229: feature_name=cg22737001	feature_id[6843].value > threshold=0.46281898021698
node_333: feature_name=cg17775332	feature_id[5680].value <= threshold=0.38904982805252075
Class: giant cell tumour of bone (GCTB)	
Rules_32	passed counts:10

node_0: feature_name=cg11915444	feature_id[4116].value > threshold=0.3402601182460785
node_10: feature_name=cg12109728	feature_id[4182].value > threshold=0.7068270146846771
node_20: feature_name=cg10844382	feature_id[3856].value > threshold=0.5988894104957581
node_24: feature_name=cg00631702	feature_id[943].value <= threshold=0.37470583617687225
node_25: feature_name=cg07912144	feature_id[3075].value > threshold=0.5578519105911255
node_29: feature_name=cg24407065	feature_id[388].value > threshold=0.5364363789558411
node_33: feature_name=cg15275017	feature_id[5069].value > threshold=0.4538573920726776
node_51: feature_name=cg07281938	feature_id[351].value > threshold=0.7506992518901825
node_73: feature_name=cg17524821	feature_id[5626].value > threshold=0.4504699558019638
node_75: feature_name=cg02973735	feature_id[1677].value > threshold=0.29383400082588196
node_77: feature_name=cg23345038	feature_id[7002].value > threshold=0.6255057156085968
node_79: feature_name=cg17537493	feature_id[5630].value > threshold=0.5825372636318207
node_85: feature_name=cg12948116	feature_id[4375].value <= threshold=0.5658791363239288
node_86: feature_name=cg06038180	feature_id[2529].value > threshold=0.43877437710762024
node_88: feature_name=cg15720017	feature_id[5175].value > threshold=0.7009969055652618
node_90: feature_name=cg13070215	feature_id[314].value <= threshold=0.14645694941282272
node_91: feature_name=cg01014262	feature_id[1082].value <= threshold=0.5737917125225067
node_92: feature_name=cg16863382	feature_id[5471].value > threshold=0.7200668156147003
node_102: feature_name=cg06633739	feature_id[2686].value > threshold=0.47115950286388397
node_126: feature_name=cg22946562	feature_id[744].value > threshold=0.6194809079170227
node_170: feature_name=cg12120430	feature_id[4185].value > threshold=0.5775820016860962
node_172: feature_name=cg02137970	feature_id[1411].value > threshold=0.6705857813358307

node_196: feature_name=cg03393445	feature_id[1807].value > threshold=0.4260125905275345
node_218: feature_name=cg23525541	feature_id[7049].value > threshold=0.4485483318567276
node_222: feature_name=cg10543634	feature_id[3775].value > threshold=0.29505227506160736
node_224: feature_name=cg07344990	feature_id[2926].value > threshold=0.5750180780887604
node_226: feature_name=cg10558233	feature_id[3780].value <= threshold=0.7217690348625183
node_227: feature_name=cg08097359	feature_id[3116].value > threshold=0.3598180413246155
node_229: feature_name=cg22737001	feature_id[6843].value <= threshold=0.46281898021698
node_230: feature_name=cg24276624	feature_id[7242].value > threshold=0.6169979274272919
node_232: feature_name=cg04026354	feature_id[1982].value <= threshold=0.42332448065280914
node_233: feature_name=cg06038180	feature_id[2529].value > threshold=0.6430551409721375
node_235: feature_name=cg14104252	feature_id[4735].value > threshold=0.5213949382305145
node_239: feature_name=cg06711298	feature_id[2707].value > threshold=0.36770085990428925
node_241: feature_name=cg11703722	feature_id[4056].value > threshold=0.5125315636396408
node_243: feature_name=cg21230793	feature_id[6514].value > threshold=0.27807849645614624
node_245: feature_name=cg06225767	feature_id[2575].value > threshold=0.18034886568784714
node_247: feature_name=cg00941576	feature_id[1053].value > threshold=0.2476368322968483
node_249: feature_name=cg10149889	feature_id[3676].value > threshold=0.633601188659668
node_257: feature_name=cg07608094	feature_id[2999].value > threshold=0.7820693254470825
node_259: feature_name=cg03315432	feature_id[1783].value > threshold=0.6790387332439423
node_261: feature_name=cg07894983	feature_id[3070].value > threshold=0.3577418476343155
node_263: feature_name=cg15646741	feature_id[5160].value <= threshold=0.2826688587665558
node_264: feature_name=cg05400196	feature_id[2366].value > threshold=0.666697084903717

Class: reactive tissue (REA)	
Rules_33	passed counts:10
node_0: feature_name=cg11915444	feature_id[4116].value > threshold=0.3402601182460785
node_10: feature_name=cg12109728	feature_id[4182].value > threshold=0.7068270146846771
node_20: feature_name=cg10844382	feature_id[3856].value > threshold=0.5988894104957581
node_24: feature_name=cg00631702	feature_id[943].value <= threshold=0.37470583617687225
node_25: feature_name=cg07912144	feature_id[3075].value > threshold=0.5578519105911255
node_29: feature_name=cg24407065	feature_id[388].value > threshold=0.5364363789558411
node_33: feature_name=cg15275017	feature_id[5069].value > threshold=0.4538573920726776
node_51: feature_name=cg07281938	feature_id[351].value > threshold=0.7506992518901825
node_73: feature_name=cg17524821	feature_id[5626].value > threshold=0.4504699558019638
node_75: feature_name=cg02973735	feature_id[1677].value > threshold=0.29383400082588196
node_77: feature_name=cg23345038	feature_id[7002].value > threshold=0.6255057156085968
node_79: feature_name=cg17537493	feature_id[5630].value > threshold=0.5825372636318207
node_85: feature_name=cg12948116	feature_id[4375].value <= threshold=0.5658791363239288
node_86: feature_name=cg06038180	feature_id[2529].value > threshold=0.43877437710762024
node_88: feature_name=cg15720017	feature_id[5175].value > threshold=0.7009969055652618
node_90: feature_name=cg13070215	feature_id[314].value <= threshold=0.14645694941282272
node_91: feature_name=cg01014262	feature_id[1082].value <= threshold=0.5737917125225067
node_92: feature_name=cg16863382	feature_id[5471].value > threshold=0.7200668156147003
node_102: feature_name=cg06633739	feature_id[2686].value > threshold=0.47115950286388397
node_126: feature_name=cg22946562	feature_id[744].value > threshold=0.6194809079170227

node_170: feature_name=cg12120430	feature_id[4185].value > threshold=0.5775820016860962
node_172: feature_name=cg02137970	feature_id[1411].value > threshold=0.6705857813358307
node_196: feature_name=cg03393445	feature_id[1807].value > threshold=0.4260125905275345
node_218: feature_name=cg23525541	feature_id[7049].value > threshold=0.4485483318567276
node_222: feature_name=cg10543634	feature_id[3775].value > threshold=0.29505227506160736
node_224: feature_name=cg07344990	feature_id[2926].value > threshold=0.5750180780887604
node_226: feature_name=cg10558233	feature_id[3780].value <= threshold=0.7217690348625183
node_227: feature_name=cg08097359	feature_id[3116].value <= threshold=0.3598180413246155
Class: Langerhans cell histiocytosis (LCH)	
Rules_34	passed counts:10
node_0: feature_name=cg11915444	feature_id[4116].value > threshold=0.3402601182460785
node_10: feature_name=cg12109728	feature_id[4182].value > threshold=0.7068270146846771
node_20: feature_name=cg10844382	feature_id[3856].value > threshold=0.5988894104957581
node_24: feature_name=cg00631702	feature_id[943].value <= threshold=0.37470583617687225
node_25: feature_name=cg07912144	feature_id[3075].value > threshold=0.5578519105911255
node_29: feature_name=cg24407065	feature_id[388].value > threshold=0.5364363789558411
node_33: feature_name=cg15275017	feature_id[5069].value > threshold=0.4538573920726776
node_51: feature_name=cg07281938	feature_id[351].value > threshold=0.7506992518901825
node_73: feature_name=cg17524821	feature_id[5626].value > threshold=0.4504699558019638
node_75: feature_name=cg02973735	feature_id[1677].value > threshold=0.29383400082588196
node_77: feature_name=cg23345038	feature_id[7002].value > threshold=0.6255057156085968
node_79: feature_name=cg17537493	feature_id[5630].value > threshold=0.5825372636318207

node_85: feature_name=cg12948116	feature_id[4375].value <= threshold=0.5658791363239288
node_86: feature_name=cg06038180	feature_id[2529].value > threshold=0.43877437710762024
node_88: feature_name=cg15720017	feature_id[5175].value > threshold=0.7009969055652618
node_90: feature_name=cg13070215	feature_id[314].value <= threshold=0.14645694941282272
node_91: feature_name=cg01014262	feature_id[1082].value <= threshold=0.5737917125225067
node_92: feature_name=cg16863382	feature_id[5471].value > threshold=0.7200668156147003
node_102: feature_name=cg06633739	feature_id[2686].value > threshold=0.47115950286388397
node_126: feature_name=cg22946562	feature_id[744].value > threshold=0.6194809079170227
node_170: feature_name=cg12120430	feature_id[4185].value > threshold=0.5775820016860962
node_172: feature_name=cg02137970	feature_id[1411].value > threshold=0.6705857813358307
node_196: feature_name=cg03393445	feature_id[1807].value <= threshold=0.4260125905275345
node_197: feature_name=cg01297744	feature_id[1157].value <= threshold=0.8005762100219727
node_198: feature_name=cg08831077	feature_id[3310].value <= threshold=0.4210805594921112
node_199: feature_name=cg11526020	feature_id[4010].value > threshold=0.8567313253879547
Class: high-grade endometrial stromal sarcoma (ESS (HG))	
Rules_35	passed counts:10
node_0: feature_name=cg11915444	feature_id[4116].value > threshold=0.3402601182460785
node_10: feature_name=cg12109728	feature_id[4182].value > threshold=0.7068270146846771
node_20: feature_name=cg10844382	feature_id[3856].value > threshold=0.5988894104957581
node_24: feature_name=cg00631702	feature_id[943].value <= threshold=0.37470583617687225
node_25: feature_name=cg07912144	feature_id[3075].value > threshold=0.5578519105911255
node_29: feature_name=cg24407065	feature_id[388].value > threshold=0.5364363789558411

node_33: feature_name=cg15275017	feature_id[5069].value > threshold=0.4538573920726776
node_51: feature_name=cg07281938	feature_id[351].value > threshold=0.7506992518901825
node_73: feature_name=cg17524821	feature_id[5626].value > threshold=0.4504699558019638
node_75: feature_name=cg02973735	feature_id[1677].value > threshold=0.29383400082588196
node_77: feature_name=cg23345038	feature_id[7002].value > threshold=0.6255057156085968
node_79: feature_name=cg17537493	feature_id[5630].value > threshold=0.5825372636318207
node_85: feature_name=cg12948116	feature_id[4375].value <= threshold=0.5658791363239288
node_86: feature_name=cg06038180	feature_id[2529].value > threshold=0.43877437710762024
node_88: feature_name=cg15720017	feature_id[5175].value > threshold=0.7009969055652618
node_90: feature_name=cg13070215	feature_id[314].value <= threshold=0.14645694941282272
node_91: feature_name=cg01014262	feature_id[1082].value <= threshold=0.5737917125225067
node_92: feature_name=cg16863382	feature_id[5471].value > threshold=0.7200668156147003
node_102: feature_name=cg06633739	feature_id[2686].value > threshold=0.47115950286388397
node_126: feature_name=cg22946562	feature_id[744].value <= threshold=0.6194809079170227
node_127: feature_name=cg23920016	feature_id[7139].value > threshold=0.7449243068695068
node_151: feature_name=cg21617218	feature_id[6608].value <= threshold=0.8795748353004456
Class: high-grade conventional osteosarcoma (OS (HG))	
Rules_36	passed counts:10
node_0: feature_name=cg11915444	feature_id[4116].value > threshold=0.3402601182460785
node_10: feature_name=cg12109728	feature_id[4182].value > threshold=0.7068270146846771
node_20: feature_name=cg10844382	feature_id[3856].value > threshold=0.5988894104957581
node_24: feature_name=cg00631702	feature_id[943].value <= threshold=0.37470583617687225

node_25: feature_name=cg07912144	feature_id[3075].value > threshold=0.5578519105911255
node_29: feature_name=cg24407065	feature_id[388].value > threshold=0.5364363789558411
node_33: feature_name=cg15275017	feature_id[5069].value > threshold=0.4538573920726776
node_51: feature_name=cg07281938	feature_id[351].value > threshold=0.7506992518901825
node_73: feature_name=cg17524821	feature_id[5626].value > threshold=0.4504699558019638
node_75: feature_name=cg02973735	feature_id[1677].value > threshold=0.29383400082588196
node_77: feature_name=cg23345038	feature_id[7002].value > threshold=0.6255057156085968
node_79: feature_name=cg17537493	feature_id[5630].value > threshold=0.5825372636318207
node_85: feature_name=cg12948116	feature_id[4375].value <= threshold=0.5658791363239288
node_86: feature_name=cg06038180	feature_id[2529].value > threshold=0.43877437710762024
node_88: feature_name=cg15720017	feature_id[5175].value > threshold=0.7009969055652618
node_90: feature_name=cg13070215	feature_id[314].value <= threshold=0.14645694941282272
node_91: feature_name=cg01014262	feature_id[1082].value <= threshold=0.5737917125225067
node_92: feature_name=cg16863382	feature_id[5471].value > threshold=0.7200668156147003
node_102: feature_name=cg06633739	feature_id[2686].value > threshold=0.47115950286388397
node_126: feature_name=cg22946562	feature_id[744].value <= threshold=0.6194809079170227
node_127: feature_name=cg23920016	feature_id[7139].value <= threshold=0.7449243068695068
node_128: feature_name=cg16521032	feature_id[5379].value <= threshold=0.8948074877262115
node_129: feature_name=cg09597070	feature_id[555].value > threshold=0.67006054520607
Class: atypical fibroxanthoma (AFX)/pleomorphic dermal sarcoma (PDS)	
Rules_37	passed counts:9
node_0: feature_name=cg11915444	feature_id[4116].value > threshold=0.3402601182460785

node_10: feature_name=cg12109728	feature_id[4182].value > threshold=0.7068270146846771
node_20: feature_name=cg10844382	feature_id[3856].value > threshold=0.5988894104957581
node_24: feature_name=cg00631702	feature_id[943].value <= threshold=0.37470583617687225
node_25: feature_name=cg07912144	feature_id[3075].value > threshold=0.5578519105911255
node_29: feature_name=cg24407065	feature_id[388].value > threshold=0.5364363789558411
node_33: feature_name=cg15275017	feature_id[5069].value > threshold=0.4538573920726776
node_51: feature_name=cg07281938	feature_id[351].value > threshold=0.7506992518901825
node_73: feature_name=cg17524821	feature_id[5626].value > threshold=0.4504699558019638
node_75: feature_name=cg02973735	feature_id[1677].value > threshold=0.29383400082588196
node_77: feature_name=cg23345038	feature_id[7002].value > threshold=0.6255057156085968
node_79: feature_name=cg17537493	feature_id[5630].value > threshold=0.5825372636318207
node_85: feature_name=cg12948116	feature_id[4375].value <= threshold=0.5658791363239288
node_86: feature_name=cg06038180	feature_id[2529].value > threshold=0.43877437710762024
node_88: feature_name=cg15720017	feature_id[5175].value > threshold=0.7009969055652618
node_90: feature_name=cg13070215	feature_id[314].value <= threshold=0.14645694941282272
node_91: feature_name=cg01014262	feature_id[1082].value <= threshold=0.5737917125225067
node_92: feature_name=cg16863382	feature_id[5471].value > threshold=0.7200668156147003
node_102: feature_name=cg06633739	feature_id[2686].value > threshold=0.47115950286388397
node_126: feature_name=cg22946562	feature_id[744].value > threshold=0.6194809079170227
node_170: feature_name=cg12120430	feature_id[4185].value > threshold=0.5775820016860962
node_172: feature_name=cg02137970	feature_id[1411].value > threshold=0.6705857813358307
node_196: feature_name=cg03393445	feature_id[1807].value > threshold=0.4260125905275345

node_218: feature_name=cg23525541	feature_id[7049].value > threshold=0.4485483318567276
node_222: feature_name=cg10543634	feature_id[3775].value > threshold=0.29505227506160736
node_224: feature_name=cg07344990	feature_id[2926].value > threshold=0.5750180780887604
node_226: feature_name=cg10558233	feature_id[3780].value <= threshold=0.7217690348625183
node_227: feature_name=cg08097359	feature_id[3116].value > threshold=0.3598180413246155
node_229: feature_name=cg22737001	feature_id[6843].value <= threshold=0.46281898021698
node_230: feature_name=cg24276624	feature_id[7242].value > threshold=0.6169979274272919
node_232: feature_name=cg04026354	feature_id[1982].value <= threshold=0.42332448065280914
node_233: feature_name=cg06038180	feature_id[2529].value > threshold=0.6430551409721375
node_235: feature_name=cg14104252	feature_id[4735].value > threshold=0.5213949382305145
node_239: feature_name=cg06711298	feature_id[2707].value > threshold=0.36770085990428925
node_241: feature_name=cg11703722	feature_id[4056].value > threshold=0.5125315636396408
node_243: feature_name=cg21230793	feature_id[6514].value > threshold=0.27807849645614624
node_245: feature_name=cg06225767	feature_id[2575].value > threshold=0.18034886568784714
node_247: feature_name=cg00941576	feature_id[1053].value > threshold=0.2476368322968483
node_249: feature_name=cg10149889	feature_id[3676].value <= threshold=0.633601188659668
node_250: feature_name=cg03631837	feature_id[1881].value > threshold=0.8389041423797607
Class: nodular fasciitis(NFA)	
Rules_38	passed counts:9
node_0: feature_name=cg11915444	feature_id[4116].value > threshold=0.3402601182460785
node_10: feature_name=cg12109728	feature_id[4182].value > threshold=0.7068270146846771
node_20: feature_name=cg10844382	feature_id[3856].value > threshold=0.5988894104957581

node_24: feature_name=cg00631702	feature_id[943].value <= threshold=0.37470583617687225
node_25: feature_name=cg07912144	feature_id[3075].value > threshold=0.5578519105911255
node_29: feature_name=cg24407065	feature_id[388].value > threshold=0.5364363789558411
node_33: feature_name=cg15275017	feature_id[5069].value > threshold=0.4538573920726776
node_51: feature_name=cg07281938	feature_id[351].value > threshold=0.7506992518901825
node_73: feature_name=cg17524821	feature_id[5626].value > threshold=0.4504699558019638
node_75: feature_name=cg02973735	feature_id[1677].value > threshold=0.29383400082588196
node_77: feature_name=cg23345038	feature_id[7002].value > threshold=0.6255057156085968
node_79: feature_name=cg17537493	feature_id[5630].value > threshold=0.5825372636318207
node_85: feature_name=cg12948116	feature_id[4375].value <= threshold=0.5658791363239288
node_86: feature_name=cg06038180	feature_id[2529].value > threshold=0.43877437710762024
node_88: feature_name=cg15720017	feature_id[5175].value > threshold=0.7009969055652618
node_90: feature_name=cg13070215	feature_id[314].value <= threshold=0.14645694941282272
node_91: feature_name=cg01014262	feature_id[1082].value <= threshold=0.5737917125225067
node_92: feature_name=cg16863382	feature_id[5471].value > threshold=0.7200668156147003
node_102: feature_name=cg06633739	feature_id[2686].value > threshold=0.47115950286388397
node_126: feature_name=cg22946562	feature_id[744].value > threshold=0.6194809079170227
node_170: feature_name=cg12120430	feature_id[4185].value > threshold=0.5775820016860962
node_172: feature_name=cg02137970	feature_id[1411].value > threshold=0.6705857813358307
node_196: feature_name=cg03393445	feature_id[1807].value > threshold=0.4260125905275345
node_218: feature_name=cg23525541	feature_id[7049].value > threshold=0.4485483318567276
node_222: feature_name=cg10543634	feature_id[3775].value > threshold=0.29505227506160736

node_224: feature_name=cg07344990	feature_id[2926].value > threshold=0.5750180780887604
node_226: feature_name=cg10558233	feature_id[3780].value <= threshold=0.7217690348625183
node_227: feature_name=cg08097359	feature_id[3116].value > threshold=0.3598180413246155
node_229: feature_name=cg22737001	feature_id[6843].value <= threshold=0.46281898021698
node_230: feature_name=cg24276624	feature_id[7242].value > threshold=0.6169979274272919
node_232: feature_name=cg04026354	feature_id[1982].value <= threshold=0.42332448065280914
node_233: feature_name=cg06038180	feature_id[2529].value > threshold=0.6430551409721375
node_235: feature_name=cg14104252	feature_id[4735].value <= threshold=0.5213949382305145
node_236: feature_name=cg12163781	feature_id[4197].value > threshold=0.6278624832630157
Class: epithelioid haemangioendothelioma (EHE)	
Rules_39	passed counts:9
node_0: feature_name=cg11915444	feature_id[4116].value > threshold=0.3402601182460785
node_10: feature_name=cg12109728	feature_id[4182].value > threshold=0.7068270146846771
node_20: feature_name=cg10844382	feature_id[3856].value > threshold=0.5988894104957581
node_24: feature_name=cg00631702	feature_id[943].value <= threshold=0.37470583617687225
node_25: feature_name=cg07912144	feature_id[3075].value > threshold=0.5578519105911255
node_29: feature_name=cg24407065	feature_id[388].value > threshold=0.5364363789558411
node_33: feature_name=cg15275017	feature_id[5069].value > threshold=0.4538573920726776
node_51: feature_name=cg07281938	feature_id[351].value > threshold=0.7506992518901825
node_73: feature_name=cg17524821	feature_id[5626].value > threshold=0.4504699558019638
node_75: feature_name=cg02973735	feature_id[1677].value > threshold=0.29383400082588196
node_77: feature_name=cg23345038	feature_id[7002].value > threshold=0.6255057156085968

node_79: feature_name=cg17537493	feature_id[5630].value > threshold=0.5825372636318207
node_85: feature_name=cg12948116	feature_id[4375].value <= threshold=0.5658791363239288
node_86: feature_name=cg06038180	feature_id[2529].value > threshold=0.43877437710762024
node_88: feature_name=cg15720017	feature_id[5175].value > threshold=0.7009969055652618
node_90: feature_name=cg13070215	feature_id[314].value <= threshold=0.14645694941282272
node_91: feature_name=cg01014262	feature_id[1082].value <= threshold=0.5737917125225067
node_92: feature_name=cg16863382	feature_id[5471].value > threshold=0.7200668156147003
node_102: feature_name=cg06633739	feature_id[2686].value > threshold=0.47115950286388397
node_126: feature_name=cg22946562	feature_id[744].value > threshold=0.6194809079170227
node_170: feature_name=cg12120430	feature_id[4185].value > threshold=0.5775820016860962
node_172: feature_name=cg02137970	feature_id[1411].value > threshold=0.6705857813358307
node_196: feature_name=cg03393445	feature_id[1807].value > threshold=0.4260125905275345
node_218: feature_name=cg23525541	feature_id[7049].value > threshold=0.4485483318567276
node_222: feature_name=cg10543634	feature_id[3775].value > threshold=0.29505227506160736
node_224: feature_name=cg07344990	feature_id[2926].value > threshold=0.5750180780887604
node_226: feature_name=cg10558233	feature_id[3780].value <= threshold=0.7217690348625183
node_227: feature_name=cg08097359	feature_id[3116].value > threshold=0.3598180413246155
node_229: feature_name=cg22737001	feature_id[6843].value <= threshold=0.46281898021698
node_230: feature_name=cg24276624	feature_id[7242].value > threshold=0.6169979274272919
node_232: feature_name=cg04026354	feature_id[1982].value <= threshold=0.42332448065280914
node_233: feature_name=cg06038180	feature_id[2529].value <= threshold=0.6430551409721375
Class: neurofibroma (NFB)	

Rules_40	passed counts:9
node_0: feature_name=cg11915444	feature_id[4116].value > threshold=0.3402601182460785
node_10: feature_name=cg12109728	feature_id[4182].value > threshold=0.7068270146846771
node_20: feature_name=cg10844382	feature_id[3856].value > threshold=0.5988894104957581
node_24: feature_name=cg00631702	feature_id[943].value <= threshold=0.37470583617687225
node_25: feature_name=cg07912144	feature_id[3075].value > threshold=0.5578519105911255
node_29: feature_name=cg24407065	feature_id[388].value > threshold=0.5364363789558411
node_33: feature_name=cg15275017	feature_id[5069].value > threshold=0.4538573920726776
node_51: feature_name=cg07281938	feature_id[351].value > threshold=0.7506992518901825
node_73: feature_name=cg17524821	feature_id[5626].value > threshold=0.4504699558019638
node_75: feature_name=cg02973735	feature_id[1677].value > threshold=0.29383400082588196
node_77: feature_name=cg23345038	feature_id[7002].value > threshold=0.6255057156085968
node_79: feature_name=cg17537493	feature_id[5630].value > threshold=0.5825372636318207
node_85: feature_name=cg12948116	feature_id[4375].value <= threshold=0.5658791363239288
node_86: feature_name=cg06038180	feature_id[2529].value > threshold=0.43877437710762024
node_88: feature_name=cg15720017	feature_id[5175].value > threshold=0.7009969055652618
node_90: feature_name=cg13070215	feature_id[314].value <= threshold=0.14645694941282272
node_91: feature_name=cg01014262	feature_id[1082].value <= threshold=0.5737917125225067
node_92: feature_name=cg16863382	feature_id[5471].value > threshold=0.7200668156147003
node_102: feature_name=cg06633739	feature_id[2686].value > threshold=0.47115950286388397
node_126: feature_name=cg22946562	feature_id[744].value > threshold=0.6194809079170227

node_170: feature_name=cg12120430	feature_id[4185].value > threshold=0.5775820016860962
node_172: feature_name=cg02137970	feature_id[1411].value > threshold=0.6705857813358307
node_196: feature_name=cg03393445	feature_id[1807].value > threshold=0.4260125905275345
node_218: feature_name=cg23525541	feature_id[7049].value > threshold=0.4485483318567276
node_222: feature_name=cg10543634	feature_id[3775].value > threshold=0.29505227506160736
node_224: feature_name=cg07344990	feature_id[2926].value > threshold=0.5750180780887604
node_226: feature_name=cg10558233	feature_id[3780].value <= threshold=0.7217690348625183
node_227: feature_name=cg08097359	feature_id[3116].value > threshold=0.3598180413246155
node_229: feature_name=cg22737001	feature_id[6843].value <= threshold=0.46281898021698
node_230: feature_name=cg24276624	feature_id[7242].value <= threshold=0.6169979274272919
Class: cutaneous squamous cell carcinoma (SCC (CUT))	
Rules_41	passed counts:8
node_0: feature_name=cg11915444	feature_id[4116].value > threshold=0.3402601182460785
node_10: feature_name=cg12109728	feature_id[4182].value > threshold=0.7068270146846771
node_20: feature_name=cg10844382	feature_id[3856].value > threshold=0.5988894104957581
node_24: feature_name=cg00631702	feature_id[943].value <= threshold=0.37470583617687225
node_25: feature_name=cg07912144	feature_id[3075].value > threshold=0.5578519105911255
node_29: feature_name=cg24407065	feature_id[388].value > threshold=0.5364363789558411
node_33: feature_name=cg15275017	feature_id[5069].value > threshold=0.4538573920726776
node_51: feature_name=cg07281938	feature_id[351].value > threshold=0.7506992518901825
node_73: feature_name=cg17524821	feature_id[5626].value > threshold=0.4504699558019638
node_75: feature_name=cg02973735	feature_id[1677].value > threshold=0.29383400082588196

node_77: feature_name=cg23345038	feature_id[7002].value > threshold=0.6255057156085968
node_79: feature_name=cg17537493	feature_id[5630].value > threshold=0.5825372636318207
node_85: feature_name=cg12948116	feature_id[4375].value <= threshold=0.5658791363239288
node_86: feature_name=cg06038180	feature_id[2529].value > threshold=0.43877437710762024
node_88: feature_name=cg15720017	feature_id[5175].value > threshold=0.7009969055652618
node_90: feature_name=cg13070215	feature_id[314].value <= threshold=0.14645694941282272
node_91: feature_name=cg01014262	feature_id[1082].value <= threshold=0.5737917125225067
node_92: feature_name=cg16863382	feature_id[5471].value > threshold=0.7200668156147003
node_102: feature_name=cg06633739	feature_id[2686].value > threshold=0.47115950286388397
node_126: feature_name=cg22946562	feature_id[744].value > threshold=0.6194809079170227
node_170: feature_name=cg12120430	feature_id[4185].value > threshold=0.5775820016860962
node_172: feature_name=cg02137970	feature_id[1411].value > threshold=0.6705857813358307
node_196: feature_name=cg03393445	feature_id[1807].value > threshold=0.4260125905275345
node_218: feature_name=cg23525541	feature_id[7049].value > threshold=0.4485483318567276
node_222: feature_name=cg10543634	feature_id[3775].value > threshold=0.29505227506160736
node_224: feature_name=cg07344990	feature_id[2926].value > threshold=0.5750180780887604
node_226: feature_name=cg10558233	feature_id[3780].value <= threshold=0.7217690348625183
node_227: feature_name=cg08097359	feature_id[3116].value > threshold=0.3598180413246155
node_229: feature_name=cg22737001	feature_id[6843].value <= threshold=0.46281898021698
node_230: feature_name=cg24276624	feature_id[7242].value > threshold=0.6169979274272919
node_232: feature_name=cg04026354	feature_id[1982].value <= threshold=0.42332448065280914
node_233: feature_name=cg06038180	feature_id[2529].value > threshold=0.6430551409721375

node_235: feature_name=cg14104252	feature_id[4735].value > threshold=0.5213949382305145
node_239: feature_name=cg06711298	feature_id[2707].value > threshold=0.36770085990428925
node_241: feature_name=cg11703722	feature_id[4056].value > threshold=0.5125315636396408
node_243: feature_name=cg21230793	feature_id[6514].value > threshold=0.27807849645614624
node_245: feature_name=cg06225767	feature_id[2575].value > threshold=0.18034886568784714
node_247: feature_name=cg00941576	feature_id[1053].value > threshold=0.2476368322968483
node_249: feature_name=cg10149889	feature_id[3676].value <= threshold=0.633601188659668
node_250: feature_name=cg03631837	feature_id[1881].value <= threshold=0.8389041423797607
node_251: feature_name=cg18201671	feature_id[5790].value <= threshold=0.7097389698028564
Class: myositis ossificans (MO)	
Rules_42	passed counts:8
node_0: feature_name=cg11915444	feature_id[4116].value > threshold=0.3402601182460785
node_10: feature_name=cg12109728	feature_id[4182].value > threshold=0.7068270146846771
node_20: feature_name=cg10844382	feature_id[3856].value > threshold=0.5988894104957581
node_24: feature_name=cg00631702	feature_id[943].value <= threshold=0.37470583617687225
node_25: feature_name=cg07912144	feature_id[3075].value > threshold=0.5578519105911255
node_29: feature_name=cg24407065	feature_id[388].value > threshold=0.5364363789558411
node_33: feature_name=cg15275017	feature_id[5069].value > threshold=0.4538573920726776
node_51: feature_name=cg07281938	feature_id[351].value > threshold=0.7506992518901825
node_73: feature_name=cg17524821	feature_id[5626].value > threshold=0.4504699558019638
node_75: feature_name=cg02973735	feature_id[1677].value > threshold=0.29383400082588196
node_77: feature_name=cg23345038	feature_id[7002].value > threshold=0.6255057156085968

node_79: feature_name=cg17537493	feature_id[5630].value > threshold=0.5825372636318207
node_85: feature_name=cg12948116	feature_id[4375].value <= threshold=0.5658791363239288
node_86: feature_name=cg06038180	feature_id[2529].value > threshold=0.43877437710762024
node_88: feature_name=cg15720017	feature_id[5175].value > threshold=0.7009969055652618
node_90: feature_name=cg13070215	feature_id[314].value <= threshold=0.14645694941282272
node_91: feature_name=cg01014262	feature_id[1082].value <= threshold=0.5737917125225067
node_92: feature_name=cg16863382	feature_id[5471].value > threshold=0.7200668156147003
node_102: feature_name=cg06633739	feature_id[2686].value > threshold=0.47115950286388397
node_126: feature_name=cg22946562	feature_id[744].value > threshold=0.6194809079170227
node_170: feature_name=cg12120430	feature_id[4185].value > threshold=0.5775820016860962
node_172: feature_name=cg02137970	feature_id[1411].value > threshold=0.6705857813358307
node_196: feature_name=cg03393445	feature_id[1807].value > threshold=0.4260125905275345
node_218: feature_name=cg23525541	feature_id[7049].value > threshold=0.4485483318567276
node_222: feature_name=cg10543634	feature_id[3775].value > threshold=0.29505227506160736
node_224: feature_name=cg07344990	feature_id[2926].value > threshold=0.5750180780887604
node_226: feature_name=cg10558233	feature_id[3780].value <= threshold=0.7217690348625183
node_227: feature_name=cg08097359	feature_id[3116].value > threshold=0.3598180413246155
node_229: feature_name=cg22737001	feature_id[6843].value <= threshold=0.46281898021698
node_230: feature_name=cg24276624	feature_id[7242].value > threshold=0.6169979274272919
node_232: feature_name=cg04026354	feature_id[1982].value <= threshold=0.42332448065280914
node_233: feature_name=cg06038180	feature_id[2529].value > threshold=0.6430551409721375
node_235: feature_name=cg14104252	feature_id[4735].value > threshold=0.5213949382305145

node_239: feature_name=cg06711298	feature_id[2707].value > threshold=0.36770085990428925
node_241: feature_name=cg11703722	feature_id[4056].value > threshold=0.5125315636396408
node_243: feature_name=cg21230793	feature_id[6514].value > threshold=0.27807849645614624
node_245: feature_name=cg06225767	feature_id[2575].value > threshold=0.18034886568784714
node_247: feature_name=cg00941576	feature_id[1053].value <= threshold=0.2476368322968483
Class: low-grade fibromyxoid sarcoma (LGFMS)	
Rules_43	passed counts:8
node_0: feature_name=cg11915444	feature_id[4116].value > threshold=0.3402601182460785
node_10: feature_name=cg12109728	feature_id[4182].value > threshold=0.7068270146846771
node_20: feature_name=cg10844382	feature_id[3856].value > threshold=0.5988894104957581
node_24: feature_name=cg00631702	feature_id[943].value <= threshold=0.37470583617687225
node_25: feature_name=cg07912144	feature_id[3075].value > threshold=0.5578519105911255
node_29: feature_name=cg24407065	feature_id[388].value > threshold=0.5364363789558411
node_33: feature_name=cg15275017	feature_id[5069].value > threshold=0.4538573920726776
node_51: feature_name=cg07281938	feature_id[351].value > threshold=0.7506992518901825
node_73: feature_name=cg17524821	feature_id[5626].value > threshold=0.4504699558019638
node_75: feature_name=cg02973735	feature_id[1677].value > threshold=0.29383400082588196
node_77: feature_name=cg23345038	feature_id[7002].value > threshold=0.6255057156085968
node_79: feature_name=cg17537493	feature_id[5630].value > threshold=0.5825372636318207
node_85: feature_name=cg12948116	feature_id[4375].value <= threshold=0.5658791363239288
node_86: feature_name=cg06038180	feature_id[2529].value > threshold=0.43877437710762024
node_88: feature_name=cg15720017	feature_id[5175].value > threshold=0.7009969055652618

node_90: feature_name=cg13070215	feature_id[314].value <= threshold=0.14645694941282272
node_91: feature_name=cg01014262	feature_id[1082].value <= threshold=0.5737917125225067
node_92: feature_name=cg16863382	feature_id[5471].value > threshold=0.7200668156147003
node_102: feature_name=cg06633739	feature_id[2686].value > threshold=0.47115950286388397
node_126: feature_name=cg22946562	feature_id[744].value > threshold=0.6194809079170227
node_170: feature_name=cg12120430	feature_id[4185].value > threshold=0.5775820016860962
node_172: feature_name=cg02137970	feature_id[1411].value > threshold=0.6705857813358307
node_196: feature_name=cg03393445	feature_id[1807].value > threshold=0.4260125905275345
node_218: feature_name=cg23525541	feature_id[7049].value > threshold=0.4485483318567276
node_222: feature_name=cg10543634	feature_id[3775].value > threshold=0.29505227506160736
node_224: feature_name=cg07344990	feature_id[2926].value > threshold=0.5750180780887604
node_226: feature_name=cg10558233	feature_id[3780].value <= threshold=0.7217690348625183
node_227: feature_name=cg08097359	feature_id[3116].value > threshold=0.3598180413246155
node_229: feature_name=cg22737001	feature_id[6843].value <= threshold=0.46281898021698
node_230: feature_name=cg24276624	feature_id[7242].value > threshold=0.6169979274272919
node_232: feature_name=cg04026354	feature_id[1982].value <= threshold=0.42332448065280914
node_233: feature_name=cg06038180	feature_id[2529].value > threshold=0.6430551409721375
node_235: feature_name=cg14104252	feature_id[4735].value > threshold=0.5213949382305145
node_239: feature_name=cg06711298	feature_id[2707].value > threshold=0.36770085990428925
node_241: feature_name=cg11703722	feature_id[4056].value > threshold=0.5125315636396408
node_243: feature_name=cg21230793	feature_id[6514].value > threshold=0.27807849645614624
node_245: feature_name=cg06225767	feature_id[2575].value <= threshold=0.18034886568784714

Class: infantile fibrosarcoma (IFS)	
Rules_44	passed counts:8
node_0: feature_name=cg11915444	feature_id[4116].value > threshold=0.3402601182460785
node_10: feature_name=cg12109728	feature_id[4182].value > threshold=0.7068270146846771
node_20: feature_name=cg10844382	feature_id[3856].value > threshold=0.5988894104957581
node_24: feature_name=cg00631702	feature_id[943].value <= threshold=0.37470583617687225
node_25: feature_name=cg07912144	feature_id[3075].value > threshold=0.5578519105911255
node_29: feature_name=cg24407065	feature_id[388].value > threshold=0.5364363789558411
node_33: feature_name=cg15275017	feature_id[5069].value > threshold=0.4538573920726776
node_51: feature_name=cg07281938	feature_id[351].value > threshold=0.7506992518901825
node_73: feature_name=cg17524821	feature_id[5626].value > threshold=0.4504699558019638
node_75: feature_name=cg02973735	feature_id[1677].value > threshold=0.29383400082588196
node_77: feature_name=cg23345038	feature_id[7002].value > threshold=0.6255057156085968
node_79: feature_name=cg17537493	feature_id[5630].value > threshold=0.5825372636318207
node_85: feature_name=cg12948116	feature_id[4375].value <= threshold=0.5658791363239288
node_86: feature_name=cg06038180	feature_id[2529].value > threshold=0.43877437710762024
node_88: feature_name=cg15720017	feature_id[5175].value > threshold=0.7009969055652618
node_90: feature_name=cg13070215	feature_id[314].value <= threshold=0.14645694941282272
node_91: feature_name=cg01014262	feature_id[1082].value <= threshold=0.5737917125225067
node_92: feature_name=cg16863382	feature_id[5471].value > threshold=0.7200668156147003
node_102: feature_name=cg06633739	feature_id[2686].value > threshold=0.47115950286388397
node_126: feature_name=cg22946562	feature_id[744].value > threshold=0.6194809079170227

node_170: feature_name=cg12120430	feature_id[4185].value > threshold=0.5775820016860962
node_172: feature_name=cg02137970	feature_id[1411].value > threshold=0.6705857813358307
node_196: feature_name=cg03393445	feature_id[1807].value > threshold=0.4260125905275345
node_218: feature_name=cg23525541	feature_id[7049].value > threshold=0.4485483318567276
node_222: feature_name=cg10543634	feature_id[3775].value > threshold=0.29505227506160736
node_224: feature_name=cg07344990	feature_id[2926].value > threshold=0.5750180780887604
node_226: feature_name=cg10558233	feature_id[3780].value <= threshold=0.7217690348625183
node_227: feature_name=cg08097359	feature_id[3116].value > threshold=0.3598180413246155
node_229: feature_name=cg22737001	feature_id[6843].value <= threshold=0.46281898021698
node_230: feature_name=cg24276624	feature_id[7242].value > threshold=0.6169979274272919
node_232: feature_name=cg04026354	feature_id[1982].value <= threshold=0.42332448065280914
node_233: feature_name=cg06038180	feature_id[2529].value > threshold=0.6430551409721375
node_235: feature_name=cg14104252	feature_id[4735].value > threshold=0.5213949382305145
node_239: feature_name=cg06711298	feature_id[2707].value > threshold=0.36770085990428925
node_241: feature_name=cg11703722	feature_id[4056].value > threshold=0.5125315636396408
node_243: feature_name=cg21230793	feature_id[6514].value <= threshold=0.27807849645614624
Class: clear cell sarcoma of soft parts (CCS)	
Rules_45	passed counts:8
node_0: feature_name=cg11915444	feature_id[4116].value > threshold=0.3402601182460785
node_10: feature_name=cg12109728	feature_id[4182].value > threshold=0.7068270146846771
node_20: feature_name=cg10844382	feature_id[3856].value > threshold=0.5988894104957581
node_24: feature_name=cg00631702	feature_id[943].value <= threshold=0.37470583617687225

node_25: feature_name=cg07912144	feature_id[3075].value > threshold=0.5578519105911255
node_29: feature_name=cg24407065	feature_id[388].value > threshold=0.5364363789558411
node_33: feature_name=cg15275017	feature_id[5069].value > threshold=0.4538573920726776
node_51: feature_name=cg07281938	feature_id[351].value > threshold=0.7506992518901825
node_73: feature_name=cg17524821	feature_id[5626].value > threshold=0.4504699558019638
node_75: feature_name=cg02973735	feature_id[1677].value > threshold=0.29383400082588196
node_77: feature_name=cg23345038	feature_id[7002].value > threshold=0.6255057156085968
node_79: feature_name=cg17537493	feature_id[5630].value > threshold=0.5825372636318207
node_85: feature_name=cg12948116	feature_id[4375].value <= threshold=0.5658791363239288
node_86: feature_name=cg06038180	feature_id[2529].value > threshold=0.43877437710762024
node_88: feature_name=cg15720017	feature_id[5175].value > threshold=0.7009969055652618
node_90: feature_name=cg13070215	feature_id[314].value <= threshold=0.14645694941282272
node_91: feature_name=cg01014262	feature_id[1082].value <= threshold=0.5737917125225067
node_92: feature_name=cg16863382	feature_id[5471].value > threshold=0.7200668156147003
node_102: feature_name=cg06633739	feature_id[2686].value > threshold=0.47115950286388397
node_126: feature_name=cg22946562	feature_id[744].value > threshold=0.6194809079170227
node_170: feature_name=cg12120430	feature_id[4185].value > threshold=0.5775820016860962
node_172: feature_name=cg02137970	feature_id[1411].value > threshold=0.6705857813358307
node_196: feature_name=cg03393445	feature_id[1807].value > threshold=0.4260125905275345
node_218: feature_name=cg23525541	feature_id[7049].value > threshold=0.4485483318567276
node_222: feature_name=cg10543634	feature_id[3775].value > threshold=0.29505227506160736
node_224: feature_name=cg07344990	feature_id[2926].value > threshold=0.5750180780887604

node_226: feature_name=cg10558233	feature_id[3780].value <= threshold=0.7217690348625183
node_227: feature_name=cg08097359	feature_id[3116].value > threshold=0.3598180413246155
node_229: feature_name=cg22737001	feature_id[6843].value <= threshold=0.46281898021698
node_230: feature_name=cg24276624	feature_id[7242].value > threshold=0.6169979274272919
node_232: feature_name=cg04026354	feature_id[1982].value <= threshold=0.42332448065280914
node_233: feature_name=cg06038180	feature_id[2529].value > threshold=0.6430551409721375
node_235: feature_name=cg14104252	feature_id[4735].value > threshold=0.5213949382305145
node_239: feature_name=cg06711298	feature_id[2707].value > threshold=0.36770085990428925
node_241: feature_name=cg11703722	feature_id[4056].value <= threshold=0.5125315636396408
Class: muscle tissue (MUS)	
Rules_46	passed counts:8
node_0: feature_name=cg11915444	feature_id[4116].value > threshold=0.3402601182460785
node_10: feature_name=cg12109728	feature_id[4182].value > threshold=0.7068270146846771
node_20: feature_name=cg10844382	feature_id[3856].value > threshold=0.5988894104957581
node_24: feature_name=cg00631702	feature_id[943].value <= threshold=0.37470583617687225
node_25: feature_name=cg07912144	feature_id[3075].value > threshold=0.5578519105911255
node_29: feature_name=cg24407065	feature_id[388].value > threshold=0.5364363789558411
node_33: feature_name=cg15275017	feature_id[5069].value > threshold=0.4538573920726776
node_51: feature_name=cg07281938	feature_id[351].value > threshold=0.7506992518901825
node_73: feature_name=cg17524821	feature_id[5626].value > threshold=0.4504699558019638
node_75: feature_name=cg02973735	feature_id[1677].value > threshold=0.29383400082588196
node_77: feature_name=cg23345038	feature_id[7002].value > threshold=0.6255057156085968

node_79: feature_name=cg17537493	feature_id[5630].value > threshold=0.5825372636318207
node_85: feature_name=cg12948116	feature_id[4375].value <= threshold=0.5658791363239288
node_86: feature_name=cg06038180	feature_id[2529].value > threshold=0.43877437710762024
node_88: feature_name=cg15720017	feature_id[5175].value > threshold=0.7009969055652618
node_90: feature_name=cg13070215	feature_id[314].value <= threshold=0.14645694941282272
node_91: feature_name=cg01014262	feature_id[1082].value <= threshold=0.5737917125225067
node_92: feature_name=cg16863382	feature_id[5471].value > threshold=0.7200668156147003
node_102: feature_name=cg06633739	feature_id[2686].value > threshold=0.47115950286388397
node_126: feature_name=cg22946562	feature_id[744].value > threshold=0.6194809079170227
node_170: feature_name=cg12120430	feature_id[4185].value > threshold=0.5775820016860962
node_172: feature_name=cg02137970	feature_id[1411].value > threshold=0.6705857813358307
node_196: feature_name=cg03393445	feature_id[1807].value > threshold=0.4260125905275345
node_218: feature_name=cg23525541	feature_id[7049].value > threshold=0.4485483318567276
node_222: feature_name=cg10543634	feature_id[3775].value > threshold=0.29505227506160736
node_224: feature_name=cg07344990	feature_id[2926].value > threshold=0.5750180780887604
node_226: feature_name=cg10558233	feature_id[3780].value <= threshold=0.7217690348625183
node_227: feature_name=cg08097359	feature_id[3116].value > threshold=0.3598180413246155
node_229: feature_name=cg22737001	feature_id[6843].value <= threshold=0.46281898021698
node_230: feature_name=cg24276624	feature_id[7242].value > threshold=0.6169979274272919
node_232: feature_name=cg04026354	feature_id[1982].value <= threshold=0.42332448065280914
node_233: feature_name=cg06038180	feature_id[2529].value > threshold=0.6430551409721375
node_235: feature_name=cg14104252	feature_id[4735].value > threshold=0.5213949382305145

node_239: feature_name=cg06711298	feature_id[2707].value <= threshold=0.36770085990428925
Class: extraskeletal myxoid chondrosarcoma (EMCS)	
Rules_47	passed counts:8
node_0: feature_name=cg11915444	feature_id[4116].value > threshold=0.3402601182460785
node_10: feature_name=cg12109728	feature_id[4182].value > threshold=0.7068270146846771
node_20: feature_name=cg10844382	feature_id[3856].value > threshold=0.5988894104957581
node_24: feature_name=cg00631702	feature_id[943].value <= threshold=0.37470583617687225
node_25: feature_name=cg07912144	feature_id[3075].value > threshold=0.5578519105911255
node_29: feature_name=cg24407065	feature_id[388].value > threshold=0.5364363789558411
node_33: feature_name=cg15275017	feature_id[5069].value > threshold=0.4538573920726776
node_51: feature_name=cg07281938	feature_id[351].value > threshold=0.7506992518901825
node_73: feature_name=cg17524821	feature_id[5626].value > threshold=0.4504699558019638
node_75: feature_name=cg02973735	feature_id[1677].value > threshold=0.29383400082588196
node_77: feature_name=cg23345038	feature_id[7002].value > threshold=0.6255057156085968
node_79: feature_name=cg17537493	feature_id[5630].value > threshold=0.5825372636318207
node_85: feature_name=cg12948116	feature_id[4375].value <= threshold=0.5658791363239288
node_86: feature_name=cg06038180	feature_id[2529].value > threshold=0.43877437710762024
node_88: feature_name=cg15720017	feature_id[5175].value > threshold=0.7009969055652618
node_90: feature_name=cg13070215	feature_id[314].value <= threshold=0.14645694941282272
node_91: feature_name=cg01014262	feature_id[1082].value <= threshold=0.5737917125225067
node_92: feature_name=cg16863382	feature_id[5471].value > threshold=0.7200668156147003
node_102: feature_name=cg06633739	feature_id[2686].value > threshold=0.47115950286388397

node_126: feature_name=cg22946562	feature_id[744].value <= threshold=0.6194809079170227
node_127: feature_name=cg23920016	feature_id[7139].value > threshold=0.7449243068695068
node_151: feature_name=cg21617218	feature_id[6608].value > threshold=0.8795748353004456
node_153: feature_name=cg01663970	feature_id[1273].value <= threshold=0.8442256152629852
Class: melanoma (MEL)	
Rules_48	passed counts:7
node_0: feature_name=cg11915444	feature_id[4116].value > threshold=0.3402601182460785
node_10: feature_name=cg12109728	feature_id[4182].value > threshold=0.7068270146846771
node_20: feature_name=cg10844382	feature_id[3856].value > threshold=0.5988894104957581
node_24: feature_name=cg00631702	feature_id[943].value <= threshold=0.37470583617687225
node_25: feature_name=cg07912144	feature_id[3075].value > threshold=0.5578519105911255
node_29: feature_name=cg24407065	feature_id[388].value > threshold=0.5364363789558411
node_33: feature_name=cg15275017	feature_id[5069].value > threshold=0.4538573920726776
node_51: feature_name=cg07281938	feature_id[351].value > threshold=0.7506992518901825
node_73: feature_name=cg17524821	feature_id[5626].value > threshold=0.4504699558019638
node_75: feature_name=cg02973735	feature_id[1677].value > threshold=0.29383400082588196
node_77: feature_name=cg23345038	feature_id[7002].value > threshold=0.6255057156085968
node_79: feature_name=cg17537493	feature_id[5630].value > threshold=0.5825372636318207
node_85: feature_name=cg12948116	feature_id[4375].value <= threshold=0.5658791363239288
node_86: feature_name=cg06038180	feature_id[2529].value > threshold=0.43877437710762024
node_88: feature_name=cg15720017	feature_id[5175].value > threshold=0.7009969055652618
node_90: feature_name=cg13070215	feature_id[314].value <= threshold=0.14645694941282272

node_91: feature_name=cg01014262	feature_id[1082].value <= threshold=0.5737917125225067
node_92: feature_name=cg16863382	feature_id[5471].value > threshold=0.7200668156147003
node_102: feature_name=cg06633739	feature_id[2686].value > threshold=0.47115950286388397
node_126: feature_name=cg22946562	feature_id[744].value > threshold=0.6194809079170227
node_170: feature_name=cg12120430	feature_id[4185].value > threshold=0.5775820016860962
node_172: feature_name=cg02137970	feature_id[1411].value > threshold=0.6705857813358307
node_196: feature_name=cg03393445	feature_id[1807].value > threshold=0.4260125905275345
node_218: feature_name=cg23525541	feature_id[7049].value > threshold=0.4485483318567276
node_222: feature_name=cg10543634	feature_id[3775].value > threshold=0.29505227506160736
node_224: feature_name=cg07344990	feature_id[2926].value > threshold=0.5750180780887604
node_226: feature_name=cg10558233	feature_id[3780].value <= threshold=0.7217690348625183
node_227: feature_name=cg08097359	feature_id[3116].value > threshold=0.3598180413246155
node_229: feature_name=cg22737001	feature_id[6843].value <= threshold=0.46281898021698
node_230: feature_name=cg24276624	feature_id[7242].value > threshold=0.6169979274272919
node_232: feature_name=cg04026354	feature_id[1982].value <= threshold=0.42332448065280914
node_233: feature_name=cg06038180	feature_id[2529].value > threshold=0.6430551409721375
node_235: feature_name=cg14104252	feature_id[4735].value > threshold=0.5213949382305145
node_239: feature_name=cg06711298	feature_id[2707].value > threshold=0.36770085990428925
node_241: feature_name=cg11703722	feature_id[4056].value > threshold=0.5125315636396408
node_243: feature_name=cg21230793	feature_id[6514].value > threshold=0.27807849645614624
node_245: feature_name=cg06225767	feature_id[2575].value > threshold=0.18034886568784714
node_247: feature_name=cg00941576	feature_id[1053].value > threshold=0.2476368322968483

node_249: feature_name=cg10149889	feature_id[3676].value > threshold=0.633601188659668
node_257: feature_name=cg07608094	feature_id[2999].value > threshold=0.7820693254470825
node_259: feature_name=cg03315432	feature_id[1783].value > threshold=0.6790387332439423
node_261: feature_name=cg07894983	feature_id[3070].value <= threshold=0.3577418476343155
Class: rhabdomyosarcoma (RMS)	
Rules_49	passed counts:7
node_0: feature_name=cg11915444	feature_id[4116].value > threshold=0.3402601182460785
node_10: feature_name=cg12109728	feature_id[4182].value > threshold=0.7068270146846771
node_20: feature_name=cg10844382	feature_id[3856].value > threshold=0.5988894104957581
node_24: feature_name=cg00631702	feature_id[943].value <= threshold=0.37470583617687225
node_25: feature_name=cg07912144	feature_id[3075].value > threshold=0.5578519105911255
node_29: feature_name=cg24407065	feature_id[388].value > threshold=0.5364363789558411
node_33: feature_name=cg15275017	feature_id[5069].value > threshold=0.4538573920726776
node_51: feature_name=cg07281938	feature_id[351].value > threshold=0.7506992518901825
node_73: feature_name=cg17524821	feature_id[5626].value > threshold=0.4504699558019638
node_75: feature_name=cg02973735	feature_id[1677].value > threshold=0.29383400082588196
node_77: feature_name=cg23345038	feature_id[7002].value > threshold=0.6255057156085968
node_79: feature_name=cg17537493	feature_id[5630].value > threshold=0.5825372636318207
node_85: feature_name=cg12948116	feature_id[4375].value <= threshold=0.5658791363239288
node_86: feature_name=cg06038180	feature_id[2529].value > threshold=0.43877437710762024
node_88: feature_name=cg15720017	feature_id[5175].value > threshold=0.7009969055652618
node_90: feature_name=cg13070215	feature_id[314].value <= threshold=0.14645694941282272

node_91: feature_name=cg01014262	feature_id[1082].value <= threshold=0.5737917125225067
node_92: feature_name=cg16863382	feature_id[5471].value > threshold=0.7200668156147003
node_102: feature_name=cg06633739	feature_id[2686].value > threshold=0.47115950286388397
node_126: feature_name=cg22946562	feature_id[744].value > threshold=0.6194809079170227
node_170: feature_name=cg12120430	feature_id[4185].value > threshold=0.5775820016860962
node_172: feature_name=cg02137970	feature_id[1411].value > threshold=0.6705857813358307
node_196: feature_name=cg03393445	feature_id[1807].value > threshold=0.4260125905275345
node_218: feature_name=cg23525541	feature_id[7049].value > threshold=0.4485483318567276
node_222: feature_name=cg10543634	feature_id[3775].value > threshold=0.29505227506160736
node_224: feature_name=cg07344990	feature_id[2926].value > threshold=0.5750180780887604
node_226: feature_name=cg10558233	feature_id[3780].value <= threshold=0.7217690348625183
node_227: feature_name=cg08097359	feature_id[3116].value > threshold=0.3598180413246155
node_229: feature_name=cg22737001	feature_id[6843].value <= threshold=0.46281898021698
node_230: feature_name=cg24276624	feature_id[7242].value > threshold=0.6169979274272919
node_232: feature_name=cg04026354	feature_id[1982].value <= threshold=0.42332448065280914
node_233: feature_name=cg06038180	feature_id[2529].value > threshold=0.6430551409721375
node_235: feature_name=cg14104252	feature_id[4735].value > threshold=0.5213949382305145
node_239: feature_name=cg06711298	feature_id[2707].value > threshold=0.36770085990428925
node_241: feature_name=cg11703722	feature_id[4056].value > threshold=0.5125315636396408
node_243: feature_name=cg21230793	feature_id[6514].value > threshold=0.27807849645614624
node_245: feature_name=cg06225767	feature_id[2575].value > threshold=0.18034886568784714
node_247: feature_name=cg00941576	feature_id[1053].value > threshold=0.2476368322968483

node_249: feature_name=cg10149889	feature_id[3676].value > threshold=0.633601188659668
node_257: feature_name=cg07608094	feature_id[2999].value > threshold=0.7820693254470825
node_259: feature_name=cg03315432	feature_id[1783].value <= threshold=0.6790387332439423
Class: sclerosing epithelioid fibrosarcoma(SEF)	
Rules_50	passed counts:7
node_0: feature_name=cg11915444	feature_id[4116].value > threshold=0.3402601182460785
node_10: feature_name=cg12109728	feature_id[4182].value > threshold=0.7068270146846771
node_20: feature_name=cg10844382	feature_id[3856].value > threshold=0.5988894104957581
node_24: feature_name=cg00631702	feature_id[943].value <= threshold=0.37470583617687225
node_25: feature_name=cg07912144	feature_id[3075].value > threshold=0.5578519105911255
node_29: feature_name=cg24407065	feature_id[388].value > threshold=0.5364363789558411
node_33: feature_name=cg15275017	feature_id[5069].value > threshold=0.4538573920726776
node_51: feature_name=cg07281938	feature_id[351].value > threshold=0.7506992518901825
node_73: feature_name=cg17524821	feature_id[5626].value > threshold=0.4504699558019638
node_75: feature_name=cg02973735	feature_id[1677].value > threshold=0.29383400082588196
node_77: feature_name=cg23345038	feature_id[7002].value > threshold=0.6255057156085968
node_79: feature_name=cg17537493	feature_id[5630].value > threshold=0.5825372636318207
node_85: feature_name=cg12948116	feature_id[4375].value <= threshold=0.5658791363239288
node_86: feature_name=cg06038180	feature_id[2529].value > threshold=0.43877437710762024
node_88: feature_name=cg15720017	feature_id[5175].value > threshold=0.7009969055652618
node_90: feature_name=cg13070215	feature_id[314].value <= threshold=0.14645694941282272
node_91: feature_name=cg01014262	feature_id[1082].value <= threshold=0.5737917125225067

node_92: feature_name=cg16863382	feature_id[5471].value > threshold=0.7200668156147003
node_102: feature_name=cg06633739	feature_id[2686].value > threshold=0.47115950286388397
node_126: feature_name=cg22946562	feature_id[744].value > threshold=0.6194809079170227
node_170: feature_name=cg12120430	feature_id[4185].value > threshold=0.5775820016860962
node_172: feature_name=cg02137970	feature_id[1411].value > threshold=0.6705857813358307
node_196: feature_name=cg03393445	feature_id[1807].value > threshold=0.4260125905275345
node_218: feature_name=cg23525541	feature_id[7049].value > threshold=0.4485483318567276
node_222: feature_name=cg10543634	feature_id[3775].value > threshold=0.29505227506160736
node_224: feature_name=cg07344990	feature_id[2926].value > threshold=0.5750180780887604
node_226: feature_name=cg10558233	feature_id[3780].value <= threshold=0.7217690348625183
node_227: feature_name=cg08097359	feature_id[3116].value > threshold=0.3598180413246155
node_229: feature_name=cg22737001	feature_id[6843].value <= threshold=0.46281898021698
node_230: feature_name=cg24276624	feature_id[7242].value > threshold=0.6169979274272919
node_232: feature_name=cg04026354	feature_id[1982].value <= threshold=0.42332448065280914
node_233: feature_name=cg06038180	feature_id[2529].value > threshold=0.6430551409721375
node_235: feature_name=cg14104252	feature_id[4735].value > threshold=0.5213949382305145
node_239: feature_name=cg06711298	feature_id[2707].value > threshold=0.36770085990428925
node_241: feature_name=cg11703722	feature_id[4056].value > threshold=0.5125315636396408
node_243: feature_name=cg21230793	feature_id[6514].value > threshold=0.27807849645614624
node_245: feature_name=cg06225767	feature_id[2575].value > threshold=0.18034886568784714
node_247: feature_name=cg00941576	feature_id[1053].value > threshold=0.2476368322968483
node_249: feature_name=cg10149889	feature_id[3676].value > threshold=0.633601188659668

node_257: feature_name=cg07608094	feature_id[2999].value <= threshold=0.7820693254470825
Class: plexiform neurofibroma (NFB (PLEX))	
Rules_51	passed counts:7
node_0: feature_name=cg11915444	feature_id[4116].value > threshold=0.3402601182460785
node_10: feature_name=cg12109728	feature_id[4182].value > threshold=0.7068270146846771
node_20: feature_name=cg10844382	feature_id[3856].value > threshold=0.5988894104957581
node_24: feature_name=cg00631702	feature_id[943].value <= threshold=0.37470583617687225
node_25: feature_name=cg07912144	feature_id[3075].value > threshold=0.5578519105911255
node_29: feature_name=cg24407065	feature_id[388].value > threshold=0.5364363789558411
node_33: feature_name=cg15275017	feature_id[5069].value > threshold=0.4538573920726776
node_51: feature_name=cg07281938	feature_id[351].value > threshold=0.7506992518901825
node_73: feature_name=cg17524821	feature_id[5626].value > threshold=0.4504699558019638
node_75: feature_name=cg02973735	feature_id[1677].value > threshold=0.29383400082588196
node_77: feature_name=cg23345038	feature_id[7002].value > threshold=0.6255057156085968
node_79: feature_name=cg17537493	feature_id[5630].value > threshold=0.5825372636318207
node_85: feature_name=cg12948116	feature_id[4375].value <= threshold=0.5658791363239288
node_86: feature_name=cg06038180	feature_id[2529].value > threshold=0.43877437710762024
node_88: feature_name=cg15720017	feature_id[5175].value > threshold=0.7009969055652618
node_90: feature_name=cg13070215	feature_id[314].value <= threshold=0.14645694941282272
node_91: feature_name=cg01014262	feature_id[1082].value <= threshold=0.5737917125225067
node_92: feature_name=cg16863382	feature_id[5471].value > threshold=0.7200668156147003
node_102: feature_name=cg06633739	feature_id[2686].value > threshold=0.47115950286388397

node_126: feature_name=cg22946562	feature_id[744].value > threshold=0.6194809079170227
node_170: feature_name=cg12120430	feature_id[4185].value > threshold=0.5775820016860962
node_172: feature_name=cg02137970	feature_id[1411].value <= threshold=0.6705857813358307
node_173: feature_name=cg16185457	feature_id[5282].value <= threshold=0.5055856853723526
node_174: feature_name=cg09040552	feature_id[3370].value > threshold=0.596051037311554
node_176: feature_name=cg12064373	feature_id[4162].value > threshold=0.10608945414423943
Class: undifferentiated sarcoma (USARC)	
Rules_52	passed counts:7
node_0: feature_name=cg11915444	feature_id[4116].value > threshold=0.3402601182460785
node_10: feature_name=cg12109728	feature_id[4182].value > threshold=0.7068270146846771
node_20: feature_name=cg10844382	feature_id[3856].value > threshold=0.5988894104957581
node_24: feature_name=cg00631702	feature_id[943].value <= threshold=0.37470583617687225
node_25: feature_name=cg07912144	feature_id[3075].value > threshold=0.5578519105911255
node_29: feature_name=cg24407065	feature_id[388].value > threshold=0.5364363789558411
node_33: feature_name=cg15275017	feature_id[5069].value > threshold=0.4538573920726776
node_51: feature_name=cg07281938	feature_id[351].value > threshold=0.7506992518901825
node_73: feature_name=cg17524821	feature_id[5626].value > threshold=0.4504699558019638
node_75: feature_name=cg02973735	feature_id[1677].value > threshold=0.29383400082588196
node_77: feature_name=cg23345038	feature_id[7002].value > threshold=0.6255057156085968
node_79: feature_name=cg17537493	feature_id[5630].value > threshold=0.5825372636318207
node_85: feature_name=cg12948116	feature_id[4375].value <= threshold=0.5658791363239288
node_86: feature_name=cg06038180	feature_id[2529].value > threshold=0.43877437710762024

node_88: feature_name=cg15720017	feature_id[5175].value > threshold=0.7009969055652618
node_90: feature_name=cg13070215	feature_id[314].value <= threshold=0.14645694941282272
node_91: feature_name=cg01014262	feature_id[1082].value <= threshold=0.5737917125225067
node_92: feature_name=cg16863382	feature_id[5471].value > threshold=0.7200668156147003
node_102: feature_name=cg06633739	feature_id[2686].value > threshold=0.47115950286388397
node_126: feature_name=cg22946562	feature_id[744].value > threshold=0.6194809079170227
node_170: feature_name=cg12120430	feature_id[4185].value > threshold=0.5775820016860962
node_172: feature_name=cg02137970	feature_id[1411].value <= threshold=0.6705857813358307
node_173: feature_name=cg16185457	feature_id[5282].value <= threshold=0.5055856853723526
node_174: feature_name=cg09040552	feature_id[3370].value > threshold=0.596051037311554
node_176: feature_name=cg12064373	feature_id[4162].value <= threshold=0.10608945414423943
node_177: feature_name=cg04874782	feature_id[2203].value > threshold=0.24745557457208633
Class: myositis proliferans (MP)	
Rules_53	passed counts:7
node_0: feature_name=cg11915444	feature_id[4116].value > threshold=0.3402601182460785
node_10: feature_name=cg12109728	feature_id[4182].value > threshold=0.7068270146846771
node_20: feature_name=cg10844382	feature_id[3856].value > threshold=0.5988894104957581
node_24: feature_name=cg00631702	feature_id[943].value <= threshold=0.37470583617687225
node_25: feature_name=cg07912144	feature_id[3075].value > threshold=0.5578519105911255
node_29: feature_name=cg24407065	feature_id[388].value > threshold=0.5364363789558411
node_33: feature_name=cg15275017	feature_id[5069].value > threshold=0.4538573920726776
node_51: feature_name=cg07281938	feature_id[351].value > threshold=0.7506992518901825

node_73: feature_name=cg17524821	feature_id[5626].value > threshold=0.4504699558019638
node_75: feature_name=cg02973735	feature_id[1677].value > threshold=0.29383400082588196
node_77: feature_name=cg23345038	feature_id[7002].value > threshold=0.6255057156085968
node_79: feature_name=cg17537493	feature_id[5630].value > threshold=0.5825372636318207
node_85: feature_name=cg12948116	feature_id[4375].value <= threshold=0.5658791363239288
node_86: feature_name=cg06038180	feature_id[2529].value > threshold=0.43877437710762024
node_88: feature_name=cg15720017	feature_id[5175].value > threshold=0.7009969055652618
node_90: feature_name=cg13070215	feature_id[314].value <= threshold=0.14645694941282272
node_91: feature_name=cg01014262	feature_id[1082].value <= threshold=0.5737917125225067
node_92: feature_name=cg16863382	feature_id[5471].value > threshold=0.7200668156147003
node_102: feature_name=cg06633739	feature_id[2686].value <= threshold=0.47115950286388397
node_103: feature_name=cg03861097	feature_id[1943].value <= threshold=0.24196631461381912
node_104: feature_name=cg05145297	feature_id[2285].value <= threshold=0.5227190256118774
Class: sarcoma (SARC)	
Rules_54	passed counts:7
node_0: feature_name=cg11915444	feature_id[4116].value > threshold=0.3402601182460785
node_10: feature_name=cg12109728	feature_id[4182].value > threshold=0.7068270146846771
node_20: feature_name=cg10844382	feature_id[3856].value > threshold=0.5988894104957581
node_24: feature_name=cg00631702	feature_id[943].value <= threshold=0.37470583617687225
node_25: feature_name=cg07912144	feature_id[3075].value > threshold=0.5578519105911255
node_29: feature_name=cg24407065	feature_id[388].value > threshold=0.5364363789558411
node_33: feature_name=cg15275017	feature_id[5069].value > threshold=0.4538573920726776

node_51: feature_name=cg07281938	feature_id[351].value <= threshold=0.7506992518901825
node_52: feature_name=cg21189849	feature_id[6504].value > threshold=0.7545044124126434
node_58: feature_name=cg02867857	feature_id[1644].value <= threshold=0.5450765490531921
Class: mesenchymal chondrosarcoma (CSA (MES))	
Rules_55	passed counts:7
node_0: feature_name=cg11915444	feature_id[4116].value > threshold=0.3402601182460785
node_10: feature_name=cg12109728	feature_id[4182].value > threshold=0.7068270146846771
node_20: feature_name=cg10844382	feature_id[3856].value > threshold=0.5988894104957581
node_24: feature_name=cg00631702	feature_id[943].value <= threshold=0.37470583617687225
node_25: feature_name=cg07912144	feature_id[3075].value > threshold=0.5578519105911255
node_29: feature_name=cg24407065	feature_id[388].value > threshold=0.5364363789558411
node_33: feature_name=cg15275017	feature_id[5069].value <= threshold=0.4538573920726776
node_34: feature_name=cg08236537	feature_id[3160].value <= threshold=0.4830351173877716
node_35: feature_name=cg21591452	feature_id[6603].value > threshold=0.878025621175766
Class: leiomyoma (LMO)	
Rules_56	passed counts:6
node_0: feature_name=cg11915444	feature_id[4116].value > threshold=0.3402601182460785
node_10: feature_name=cg12109728	feature_id[4182].value > threshold=0.7068270146846771
node_20: feature_name=cg10844382	feature_id[3856].value > threshold=0.5988894104957581
node_24: feature_name=cg00631702	feature_id[943].value <= threshold=0.37470583617687225
node_25: feature_name=cg07912144	feature_id[3075].value > threshold=0.5578519105911255
node_29: feature_name=cg24407065	feature_id[388].value > threshold=0.5364363789558411

node_33: feature_name=cg15275017	feature_id[5069].value > threshold=0.4538573920726776
node_51: feature_name=cg07281938	feature_id[351].value > threshold=0.7506992518901825
node_73: feature_name=cg17524821	feature_id[5626].value > threshold=0.4504699558019638
node_75: feature_name=cg02973735	feature_id[1677].value > threshold=0.29383400082588196
node_77: feature_name=cg23345038	feature_id[7002].value > threshold=0.6255057156085968
node_79: feature_name=cg17537493	feature_id[5630].value > threshold=0.5825372636318207
node_85: feature_name=cg12948116	feature_id[4375].value <= threshold=0.5658791363239288
node_86: feature_name=cg06038180	feature_id[2529].value > threshold=0.43877437710762024
node_88: feature_name=cg15720017	feature_id[5175].value > threshold=0.7009969055652618
node_90: feature_name=cg13070215	feature_id[314].value <= threshold=0.14645694941282272
node_91: feature_name=cg01014262	feature_id[1082].value <= threshold=0.5737917125225067
node_92: feature_name=cg16863382	feature_id[5471].value > threshold=0.7200668156147003
node_102: feature_name=cg06633739	feature_id[2686].value > threshold=0.47115950286388397
node_126: feature_name=cg22946562	feature_id[744].value > threshold=0.6194809079170227
node_170: feature_name=cg12120430	feature_id[4185].value > threshold=0.5775820016860962
node_172: feature_name=cg02137970	feature_id[1411].value > threshold=0.6705857813358307
node_196: feature_name=cg03393445	feature_id[1807].value > threshold=0.4260125905275345
node_218: feature_name=cg23525541	feature_id[7049].value > threshold=0.4485483318567276
node_222: feature_name=cg10543634	feature_id[3775].value > threshold=0.29505227506160736
node_224: feature_name=cg07344990	feature_id[2926].value > threshold=0.5750180780887604
node_226: feature_name=cg10558233	feature_id[3780].value <= threshold=0.7217690348625183
node_227: feature_name=cg08097359	feature_id[3116].value > threshold=0.3598180413246155

node_229: feature_name=cg22737001	feature_id[6843].value <= threshold=0.46281898021698
node_230: feature_name=cg24276624	feature_id[7242].value > threshold=0.6169979274272919
node_232: feature_name=cg04026354	feature_id[1982].value <= threshold=0.42332448065280914
node_233: feature_name=cg06038180	feature_id[2529].value > threshold=0.6430551409721375
node_235: feature_name=cg14104252	feature_id[4735].value > threshold=0.5213949382305145
node_239: feature_name=cg06711298	feature_id[2707].value > threshold=0.36770085990428925
node_241: feature_name=cg11703722	feature_id[4056].value > threshold=0.5125315636396408
node_243: feature_name=cg21230793	feature_id[6514].value > threshold=0.27807849645614624
node_245: feature_name=cg06225767	feature_id[2575].value > threshold=0.18034886568784714
node_247: feature_name=cg00941576	feature_id[1053].value > threshold=0.2476368322968483
node_249: feature_name=cg10149889	feature_id[3676].value > threshold=0.633601188659668
node_257: feature_name=cg07608094	feature_id[2999].value > threshold=0.7820693254470825
node_259: feature_name=cg03315432	feature_id[1783].value > threshold=0.6790387332439423
node_261: feature_name=cg07894983	feature_id[3070].value > threshold=0.3577418476343155
node_263: feature_name=cg15646741	feature_id[5160].value > threshold=0.2826688587665558
node_273: feature_name=cg09701233	feature_id[3546].value <= threshold=0.5189756155014038
Class: embryonal rhabdomyosarcoma (RMS (EMB))	
Rules_57	passed counts:6
node_0: feature_name=cg11915444	feature_id[4116].value > threshold=0.3402601182460785
node_10: feature_name=cg12109728	feature_id[4182].value > threshold=0.7068270146846771
node_20: feature_name=cg10844382	feature_id[3856].value > threshold=0.5988894104957581
node_24: feature_name=cg00631702	feature_id[943].value <= threshold=0.37470583617687225

node_25: feature_name=cg07912144	feature_id[3075].value > threshold=0.5578519105911255
node_29: feature_name=cg24407065	feature_id[388].value > threshold=0.5364363789558411
node_33: feature_name=cg15275017	feature_id[5069].value > threshold=0.4538573920726776
node_51: feature_name=cg07281938	feature_id[351].value > threshold=0.7506992518901825
node_73: feature_name=cg17524821	feature_id[5626].value > threshold=0.4504699558019638
node_75: feature_name=cg02973735	feature_id[1677].value > threshold=0.29383400082588196
node_77: feature_name=cg23345038	feature_id[7002].value > threshold=0.6255057156085968
node_79: feature_name=cg17537493	feature_id[5630].value > threshold=0.5825372636318207
node_85: feature_name=cg12948116	feature_id[4375].value <= threshold=0.5658791363239288
node_86: feature_name=cg06038180	feature_id[2529].value > threshold=0.43877437710762024
node_88: feature_name=cg15720017	feature_id[5175].value > threshold=0.7009969055652618
node_90: feature_name=cg13070215	feature_id[314].value <= threshold=0.14645694941282272
node_91: feature_name=cg01014262	feature_id[1082].value <= threshold=0.5737917125225067
node_92: feature_name=cg16863382	feature_id[5471].value > threshold=0.7200668156147003
node_102: feature_name=cg06633739	feature_id[2686].value > threshold=0.47115950286388397
node_126: feature_name=cg22946562	feature_id[744].value > threshold=0.6194809079170227
node_170: feature_name=cg12120430	feature_id[4185].value > threshold=0.5775820016860962
node_172: feature_name=cg02137970	feature_id[1411].value <= threshold=0.6705857813358307
node_173: feature_name=cg16185457	feature_id[5282].value > threshold=0.5055856853723526
node_183: feature_name=cg04993279	feature_id[2243].value > threshold=0.46021124720573425
node_191: feature_name=cg09043524	feature_id[3372].value > threshold=0.7900851368904114
Class: lipoma (LIPO)	

Rules_58	passed counts:6
node_0: feature_name=cg11915444	feature_id[4116].value > threshold=0.3402601182460785
node_10: feature_name=cg12109728	feature_id[4182].value > threshold=0.7068270146846771
node_20: feature_name=cg10844382	feature_id[3856].value > threshold=0.5988894104957581
node_24: feature_name=cg00631702	feature_id[943].value <= threshold=0.37470583617687225
node_25: feature_name=cg07912144	feature_id[3075].value > threshold=0.5578519105911255
node_29: feature_name=cg24407065	feature_id[388].value > threshold=0.5364363789558411
node_33: feature_name=cg15275017	feature_id[5069].value > threshold=0.4538573920726776
node_51: feature_name=cg07281938	feature_id[351].value <= threshold=0.7506992518901825
node_52: feature_name=cg21189849	feature_id[6504].value > threshold=0.7545044124126434
node_58: feature_name=cg02867857	feature_id[1644].value > threshold=0.5450765490531921
node_60: feature_name=cg00863893	feature_id[1017].value > threshold=0.5313664376735687
Class: chordoma (CHORD)	
Rules_59	passed counts:6
node_0: feature_name=cg11915444	feature_id[4116].value > threshold=0.3402601182460785
node_10: feature_name=cg12109728	feature_id[4182].value > threshold=0.7068270146846771
node_20: feature_name=cg10844382	feature_id[3856].value > threshold=0.5988894104957581
node_24: feature_name=cg00631702	feature_id[943].value <= threshold=0.37470583617687225
node_25: feature_name=cg07912144	feature_id[3075].value > threshold=0.5578519105911255
node_29: feature_name=cg24407065	feature_id[388].value > threshold=0.5364363789558411
node_33: feature_name=cg15275017	feature_id[5069].value <= threshold=0.4538573920726776
node_34: feature_name=cg08236537	feature_id[3160].value <= threshold=0.4830351173877716

node_35: feature_name=cg21591452	feature_id[6603].value <= threshold=0.878025621175766
node_36: feature_name=cg07368061	feature_id[2933].value > threshold=0.6850627958774567
Class: low-grade endometrial stromal sarcoma (ESS (LG))	
Rules_60	passed counts:5
node_0: feature_name=cg11915444	feature_id[4116].value > threshold=0.3402601182460785
node_10: feature_name=cg12109728	feature_id[4182].value > threshold=0.7068270146846771
node_20: feature_name=cg10844382	feature_id[3856].value > threshold=0.5988894104957581
node_24: feature_name=cg00631702	feature_id[943].value <= threshold=0.37470583617687225
node_25: feature_name=cg07912144	feature_id[3075].value > threshold=0.5578519105911255
node_29: feature_name=cg24407065	feature_id[388].value > threshold=0.5364363789558411
node_33: feature_name=cg15275017	feature_id[5069].value > threshold=0.4538573920726776
node_51: feature_name=cg07281938	feature_id[351].value > threshold=0.7506992518901825
node_73: feature_name=cg17524821	feature_id[5626].value > threshold=0.4504699558019638
node_75: feature_name=cg02973735	feature_id[1677].value > threshold=0.29383400082588196
node_77: feature_name=cg23345038	feature_id[7002].value > threshold=0.6255057156085968
node_79: feature_name=cg17537493	feature_id[5630].value > threshold=0.5825372636318207
node_85: feature_name=cg12948116	feature_id[4375].value <= threshold=0.5658791363239288
node_86: feature_name=cg06038180	feature_id[2529].value > threshold=0.43877437710762024
node_88: feature_name=cg15720017	feature_id[5175].value > threshold=0.7009969055652618
node_90: feature_name=cg13070215	feature_id[314].value <= threshold=0.14645694941282272
node_91: feature_name=cg01014262	feature_id[1082].value <= threshold=0.5737917125225067
node_92: feature_name=cg16863382	feature_id[5471].value > threshold=0.7200668156147003

node_102: feature_name=cg06633739	feature_id[2686].value > threshold=0.47115950286388397
node_126: feature_name=cg22946562	feature_id[744].value > threshold=0.6194809079170227
node_170: feature_name=cg12120430	feature_id[4185].value > threshold=0.5775820016860962
node_172: feature_name=cg02137970	feature_id[1411].value > threshold=0.6705857813358307
node_196: feature_name=cg03393445	feature_id[1807].value > threshold=0.4260125905275345
node_218: feature_name=cg23525541	feature_id[7049].value > threshold=0.4485483318567276
node_222: feature_name=cg10543634	feature_id[3775].value > threshold=0.29505227506160736
node_224: feature_name=cg07344990	feature_id[2926].value > threshold=0.5750180780887604
node_226: feature_name=cg10558233	feature_id[3780].value <= threshold=0.7217690348625183
node_227: feature_name=cg08097359	feature_id[3116].value > threshold=0.3598180413246155
node_229: feature_name=cg22737001	feature_id[6843].value <= threshold=0.46281898021698
node_230: feature_name=cg24276624	feature_id[7242].value > threshold=0.6169979274272919
node_232: feature_name=cg04026354	feature_id[1982].value <= threshold=0.42332448065280914
node_233: feature_name=cg06038180	feature_id[2529].value > threshold=0.6430551409721375
node_235: feature_name=cg14104252	feature_id[4735].value > threshold=0.5213949382305145
node_239: feature_name=cg06711298	feature_id[2707].value > threshold=0.36770085990428925
node_241: feature_name=cg11703722	feature_id[4056].value > threshold=0.5125315636396408
node_243: feature_name=cg21230793	feature_id[6514].value > threshold=0.27807849645614624
node_245: feature_name=cg06225767	feature_id[2575].value > threshold=0.18034886568784714
node_247: feature_name=cg00941576	feature_id[1053].value > threshold=0.2476368322968483
node_249: feature_name=cg10149889	feature_id[3676].value > threshold=0.633601188659668
node_257: feature_name=cg07608094	feature_id[2999].value > threshold=0.7820693254470825

node_259: feature_name=cg03315432	feature_id[1783].value > threshold=0.6790387332439423
node_261: feature_name=cg07894983	feature_id[3070].value > threshold=0.3577418476343155
node_263: feature_name=cg15646741	feature_id[5160].value > threshold=0.2826688587665558
node_273: feature_name=cg09701233	feature_id[3546].value > threshold=0.5189756155014038
node_275: feature_name=cg09230938	feature_id[738].value > threshold=0.3108274042606354
node_277: feature_name=cg05334190	feature_id[2341].value <= threshold=0.2764553725719452
Class: malignant peripheral nerve sheath tumour (MPNST)	
Rules_61	passed counts:5
node_0: feature_name=cg11915444	feature_id[4116].value > threshold=0.3402601182460785
node_10: feature_name=cg12109728	feature_id[4182].value > threshold=0.7068270146846771
node_20: feature_name=cg10844382	feature_id[3856].value > threshold=0.5988894104957581
node_24: feature_name=cg00631702	feature_id[943].value <= threshold=0.37470583617687225
node_25: feature_name=cg07912144	feature_id[3075].value > threshold=0.5578519105911255
node_29: feature_name=cg24407065	feature_id[388].value > threshold=0.5364363789558411
node_33: feature_name=cg15275017	feature_id[5069].value > threshold=0.4538573920726776
node_51: feature_name=cg07281938	feature_id[351].value > threshold=0.7506992518901825
node_73: feature_name=cg17524821	feature_id[5626].value > threshold=0.4504699558019638
node_75: feature_name=cg02973735	feature_id[1677].value > threshold=0.29383400082588196
node_77: feature_name=cg23345038	feature_id[7002].value > threshold=0.6255057156085968
node_79: feature_name=cg17537493	feature_id[5630].value > threshold=0.5825372636318207
node_85: feature_name=cg12948116	feature_id[4375].value <= threshold=0.5658791363239288
node_86: feature_name=cg06038180	feature_id[2529].value > threshold=0.43877437710762024

node_88: feature_name=cg15720017	feature_id[5175].value > threshold=0.7009969055652618
node_90: feature_name=cg13070215	feature_id[314].value <= threshold=0.14645694941282272
node_91: feature_name=cg01014262	feature_id[1082].value <= threshold=0.5737917125225067
node_92: feature_name=cg16863382	feature_id[5471].value > threshold=0.7200668156147003
node_102: feature_name=cg06633739	feature_id[2686].value > threshold=0.47115950286388397
node_126: feature_name=cg22946562	feature_id[744].value > threshold=0.6194809079170227
node_170: feature_name=cg12120430	feature_id[4185].value > threshold=0.5775820016860962
node_172: feature_name=cg02137970	feature_id[1411].value > threshold=0.6705857813358307
node_196: feature_name=cg03393445	feature_id[1807].value > threshold=0.4260125905275345
node_218: feature_name=cg23525541	feature_id[7049].value > threshold=0.4485483318567276
node_222: feature_name=cg10543634	feature_id[3775].value > threshold=0.29505227506160736
node_224: feature_name=cg07344990	feature_id[2926].value > threshold=0.5750180780887604
node_226: feature_name=cg10558233	feature_id[3780].value <= threshold=0.7217690348625183
node_227: feature_name=cg08097359	feature_id[3116].value > threshold=0.3598180413246155
node_229: feature_name=cg22737001	feature_id[6843].value <= threshold=0.46281898021698
node_230: feature_name=cg24276624	feature_id[7242].value > threshold=0.6169979274272919
node_232: feature_name=cg04026354	feature_id[1982].value <= threshold=0.42332448065280914
node_233: feature_name=cg06038180	feature_id[2529].value > threshold=0.6430551409721375
node_235: feature_name=cg14104252	feature_id[4735].value > threshold=0.5213949382305145
node_239: feature_name=cg06711298	feature_id[2707].value > threshold=0.36770085990428925
node_241: feature_name=cg11703722	feature_id[4056].value > threshold=0.5125315636396408
node_243: feature_name=cg21230793	feature_id[6514].value > threshold=0.27807849645614624

node_245: feature_name=cg06225767	feature_id[2575].value > threshold=0.18034886568784714
node_247: feature_name=cg00941576	feature_id[1053].value > threshold=0.2476368322968483
node_249: feature_name=cg10149889	feature_id[3676].value > threshold=0.633601188659668
node_257: feature_name=cg07608094	feature_id[2999].value > threshold=0.7820693254470825
node_259: feature_name=cg03315432	feature_id[1783].value > threshold=0.6790387332439423
node_261: feature_name=cg07894983	feature_id[3070].value > threshold=0.3577418476343155
node_263: feature_name=cg15646741	feature_id[5160].value > threshold=0.2826688587665558
node_273: feature_name=cg09701233	feature_id[3546].value > threshold=0.5189756155014038
node_275: feature_name=cg09230938	feature_id[738].value <= threshold=0.3108274042606354
Class: osteoblastoma (OB)	
Rules_62	passed counts:5
node_0: feature_name=cg11915444	feature_id[4116].value > threshold=0.3402601182460785
node_10: feature_name=cg12109728	feature_id[4182].value > threshold=0.7068270146846771
node_20: feature_name=cg10844382	feature_id[3856].value > threshold=0.5988894104957581
node_24: feature_name=cg00631702	feature_id[943].value <= threshold=0.37470583617687225
node_25: feature_name=cg07912144	feature_id[3075].value > threshold=0.5578519105911255
node_29: feature_name=cg24407065	feature_id[388].value > threshold=0.5364363789558411
node_33: feature_name=cg15275017	feature_id[5069].value > threshold=0.4538573920726776
node_51: feature_name=cg07281938	feature_id[351].value > threshold=0.7506992518901825
node_73: feature_name=cg17524821	feature_id[5626].value > threshold=0.4504699558019638
node_75: feature_name=cg02973735	feature_id[1677].value > threshold=0.29383400082588196
node_77: feature_name=cg23345038	feature_id[7002].value > threshold=0.6255057156085968

node_79: feature_name=cg17537493	feature_id[5630].value > threshold=0.5825372636318207
node_85: feature_name=cg12948116	feature_id[4375].value <= threshold=0.5658791363239288
node_86: feature_name=cg06038180	feature_id[2529].value > threshold=0.43877437710762024
node_88: feature_name=cg15720017	feature_id[5175].value > threshold=0.7009969055652618
node_90: feature_name=cg13070215	feature_id[314].value <= threshold=0.14645694941282272
node_91: feature_name=cg01014262	feature_id[1082].value <= threshold=0.5737917125225067
node_92: feature_name=cg16863382	feature_id[5471].value > threshold=0.7200668156147003
node_102: feature_name=cg06633739	feature_id[2686].value <= threshold=0.47115950286388397
node_103: feature_name=cg03861097	feature_id[1943].value <= threshold=0.24196631461381912
node_104: feature_name=cg05145297	feature_id[2285].value > threshold=0.5227190256118774
node_106: feature_name=cg16797656	feature_id[5460].value <= threshold=0.4279162883758545
Class: infantile fibrosarcoma (IFS)	
Rules_63	passed counts:4
node_0: feature_name=cg11915444	feature_id[4116].value > threshold=0.3402601182460785
node_10: feature_name=cg12109728	feature_id[4182].value > threshold=0.7068270146846771
node_20: feature_name=cg10844382	feature_id[3856].value > threshold=0.5988894104957581
node_24: feature_name=cg00631702	feature_id[943].value <= threshold=0.37470583617687225
node_25: feature_name=cg07912144	feature_id[3075].value > threshold=0.5578519105911255
node_29: feature_name=cg24407065	feature_id[388].value > threshold=0.5364363789558411
node_33: feature_name=cg15275017	feature_id[5069].value > threshold=0.4538573920726776
node_51: feature_name=cg07281938	feature_id[351].value > threshold=0.7506992518901825
node_73: feature_name=cg17524821	feature_id[5626].value > threshold=0.4504699558019638

node_75: feature_name=cg02973735	feature_id[1677].value > threshold=0.29383400082588196
node_77: feature_name=cg23345038	feature_id[7002].value > threshold=0.6255057156085968
node_79: feature_name=cg17537493	feature_id[5630].value > threshold=0.5825372636318207
node_85: feature_name=cg12948116	feature_id[4375].value <= threshold=0.5658791363239288
node_86: feature_name=cg06038180	feature_id[2529].value > threshold=0.43877437710762024
node_88: feature_name=cg15720017	feature_id[5175].value > threshold=0.7009969055652618
node_90: feature_name=cg13070215	feature_id[314].value > threshold=0.14645694941282272
node_340: feature_name=cg06272543	feature_id[2590].value <= threshold=0.07370422407984734
node_341: feature_name=cg24268698	feature_id[7238].value > threshold=0.9151104986667633
Class: undifferentiated sarcoma (USARC)	
Rules_64	passed counts:4
node_0: feature_name=cg11915444	feature_id[4116].value > threshold=0.3402601182460785
node_10: feature_name=cg12109728	feature_id[4182].value > threshold=0.7068270146846771
node_20: feature_name=cg10844382	feature_id[3856].value > threshold=0.5988894104957581
node_24: feature_name=cg00631702	feature_id[943].value <= threshold=0.37470583617687225
node_25: feature_name=cg07912144	feature_id[3075].value > threshold=0.5578519105911255
node_29: feature_name=cg24407065	feature_id[388].value > threshold=0.5364363789558411
node_33: feature_name=cg15275017	feature_id[5069].value > threshold=0.4538573920726776
node_51: feature_name=cg07281938	feature_id[351].value > threshold=0.7506992518901825
node_73: feature_name=cg17524821	feature_id[5626].value > threshold=0.4504699558019638
node_75: feature_name=cg02973735	feature_id[1677].value > threshold=0.29383400082588196
node_77: feature_name=cg23345038	feature_id[7002].value > threshold=0.6255057156085968

node_79: feature_name=cg17537493	feature_id[5630].value > threshold=0.5825372636318207
node_85: feature_name=cg12948116	feature_id[4375].value <= threshold=0.5658791363239288
node_86: feature_name=cg06038180	feature_id[2529].value > threshold=0.43877437710762024
node_88: feature_name=cg15720017	feature_id[5175].value > threshold=0.7009969055652618
node_90: feature_name=cg13070215	feature_id[314].value <= threshold=0.14645694941282272
node_91: feature_name=cg01014262	feature_id[1082].value <= threshold=0.5737917125225067
node_92: feature_name=cg16863382	feature_id[5471].value > threshold=0.7200668156147003
node_102: feature_name=cg06633739	feature_id[2686].value > threshold=0.47115950286388397
node_126: feature_name=cg22946562	feature_id[744].value > threshold=0.6194809079170227
node_170: feature_name=cg12120430	feature_id[4185].value > threshold=0.5775820016860962
node_172: feature_name=cg02137970	feature_id[1411].value > threshold=0.6705857813358307
node_196: feature_name=cg03393445	feature_id[1807].value > threshold=0.4260125905275345
node_218: feature_name=cg23525541	feature_id[7049].value > threshold=0.4485483318567276
node_222: feature_name=cg10543634	feature_id[3775].value > threshold=0.29505227506160736
node_224: feature_name=cg07344990	feature_id[2926].value > threshold=0.5750180780887604
node_226: feature_name=cg10558233	feature_id[3780].value <= threshold=0.7217690348625183
node_227: feature_name=cg08097359	feature_id[3116].value > threshold=0.3598180413246155
node_229: feature_name=cg22737001	feature_id[6843].value <= threshold=0.46281898021698
node_230: feature_name=cg24276624	feature_id[7242].value > threshold=0.6169979274272919
node_232: feature_name=cg04026354	feature_id[1982].value <= threshold=0.42332448065280914
node_233: feature_name=cg06038180	feature_id[2529].value > threshold=0.6430551409721375
node_235: feature_name=cg14104252	feature_id[4735].value > threshold=0.5213949382305145

node_239: feature_name=cg06711298	feature_id[2707].value > threshold=0.36770085990428925
node_241: feature_name=cg11703722	feature_id[4056].value > threshold=0.5125315636396408
node_243: feature_name=cg21230793	feature_id[6514].value > threshold=0.27807849645614624
node_245: feature_name=cg06225767	feature_id[2575].value > threshold=0.18034886568784714
node_247: feature_name=cg00941576	feature_id[1053].value > threshold=0.2476368322968483
node_249: feature_name=cg10149889	feature_id[3676].value > threshold=0.633601188659668
node_257: feature_name=cg07608094	feature_id[2999].value > threshold=0.7820693254470825
node_259: feature_name=cg03315432	feature_id[1783].value > threshold=0.6790387332439423
node_261: feature_name=cg07894983	feature_id[3070].value > threshold=0.3577418476343155
node_263: feature_name=cg15646741	feature_id[5160].value > threshold=0.2826688587665558
node_273: feature_name=cg09701233	feature_id[3546].value > threshold=0.5189756155014038
node_275: feature_name=cg09230938	feature_id[738].value > threshold=0.3108274042606354
node_277: feature_name=cg05334190	feature_id[2341].value > threshold=0.2764553725719452
node_279: feature_name=cg15617847	feature_id[5148].value > threshold=0.05914711393415928
Class: chondrosarcoma (CSA)	
Rules_65	passed counts:4
node_0: feature_name=cg11915444	feature_id[4116].value > threshold=0.3402601182460785
node_10: feature_name=cg12109728	feature_id[4182].value > threshold=0.7068270146846771
node_20: feature_name=cg10844382	feature_id[3856].value > threshold=0.5988894104957581
node_24: feature_name=cg00631702	feature_id[943].value <= threshold=0.37470583617687225
node_25: feature_name=cg07912144	feature_id[3075].value > threshold=0.5578519105911255
node_29: feature_name=cg24407065	feature_id[388].value > threshold=0.5364363789558411

node_33: feature_name=cg15275017	feature_id[5069].value > threshold=0.4538573920726776
node_51: feature_name=cg07281938	feature_id[351].value > threshold=0.7506992518901825
node_73: feature_name=cg17524821	feature_id[5626].value > threshold=0.4504699558019638
node_75: feature_name=cg02973735	feature_id[1677].value > threshold=0.29383400082588196
node_77: feature_name=cg23345038	feature_id[7002].value > threshold=0.6255057156085968
node_79: feature_name=cg17537493	feature_id[5630].value > threshold=0.5825372636318207
node_85: feature_name=cg12948116	feature_id[4375].value <= threshold=0.5658791363239288
node_86: feature_name=cg06038180	feature_id[2529].value > threshold=0.43877437710762024
node_88: feature_name=cg15720017	feature_id[5175].value > threshold=0.7009969055652618
node_90: feature_name=cg13070215	feature_id[314].value <= threshold=0.14645694941282272
node_91: feature_name=cg01014262	feature_id[1082].value <= threshold=0.5737917125225067
node_92: feature_name=cg16863382	feature_id[5471].value > threshold=0.7200668156147003
node_102: feature_name=cg06633739	feature_id[2686].value > threshold=0.47115950286388397
node_126: feature_name=cg22946562	feature_id[744].value > threshold=0.6194809079170227
node_170: feature_name=cg12120430	feature_id[4185].value > threshold=0.5775820016860962
node_172: feature_name=cg02137970	feature_id[1411].value > threshold=0.6705857813358307
node_196: feature_name=cg03393445	feature_id[1807].value > threshold=0.4260125905275345
node_218: feature_name=cg23525541	feature_id[7049].value > threshold=0.4485483318567276
node_222: feature_name=cg10543634	feature_id[3775].value > threshold=0.29505227506160736
node_224: feature_name=cg07344990	feature_id[2926].value > threshold=0.5750180780887604
node_226: feature_name=cg10558233	feature_id[3780].value <= threshold=0.7217690348625183
node_227: feature_name=cg08097359	feature_id[3116].value > threshold=0.3598180413246155

node_229: feature_name=cg22737001	feature_id[6843].value <= threshold=0.46281898021698
node_230: feature_name=cg24276624	feature_id[7242].value > threshold=0.6169979274272919
node_232: feature_name=cg04026354	feature_id[1982].value <= threshold=0.42332448065280914
node_233: feature_name=cg06038180	feature_id[2529].value > threshold=0.6430551409721375
node_235: feature_name=cg14104252	feature_id[4735].value > threshold=0.5213949382305145
node_239: feature_name=cg06711298	feature_id[2707].value > threshold=0.36770085990428925
node_241: feature_name=cg11703722	feature_id[4056].value > threshold=0.5125315636396408
node_243: feature_name=cg21230793	feature_id[6514].value > threshold=0.27807849645614624
node_245: feature_name=cg06225767	feature_id[2575].value > threshold=0.18034886568784714
node_247: feature_name=cg00941576	feature_id[1053].value > threshold=0.2476368322968483
node_249: feature_name=cg10149889	feature_id[3676].value > threshold=0.633601188659668
node_257: feature_name=cg07608094	feature_id[2999].value > threshold=0.7820693254470825
node_259: feature_name=cg03315432	feature_id[1783].value > threshold=0.6790387332439423
node_261: feature_name=cg07894983	feature_id[3070].value > threshold=0.3577418476343155
node_263: feature_name=cg15646741	feature_id[5160].value > threshold=0.2826688587665558
node_273: feature_name=cg09701233	feature_id[3546].value > threshold=0.5189756155014038
node_275: feature_name=cg09230938	feature_id[738].value > threshold=0.3108274042606354
node_277: feature_name=cg05334190	feature_id[2341].value > threshold=0.2764553725719452
node_279: feature_name=cg15617847	feature_id[5148].value <= threshold=0.05914711393415928
node_280: feature_name=cg00216961	feature_id[816].value > threshold=0.42650654911994934
Class: lipoma (LIPO)	
Rules_66	passed counts:4

node_0: feature_name=cg11915444	feature_id[4116].value > threshold=0.3402601182460785
node_10: feature_name=cg12109728	feature_id[4182].value > threshold=0.7068270146846771
node_20: feature_name=cg10844382	feature_id[3856].value > threshold=0.5988894104957581
node_24: feature_name=cg00631702	feature_id[943].value <= threshold=0.37470583617687225
node_25: feature_name=cg07912144	feature_id[3075].value > threshold=0.5578519105911255
node_29: feature_name=cg24407065	feature_id[388].value > threshold=0.5364363789558411
node_33: feature_name=cg15275017	feature_id[5069].value > threshold=0.4538573920726776
node_51: feature_name=cg07281938	feature_id[351].value > threshold=0.7506992518901825
node_73: feature_name=cg17524821	feature_id[5626].value > threshold=0.4504699558019638
node_75: feature_name=cg02973735	feature_id[1677].value > threshold=0.29383400082588196
node_77: feature_name=cg23345038	feature_id[7002].value > threshold=0.6255057156085968
node_79: feature_name=cg17537493	feature_id[5630].value > threshold=0.5825372636318207
node_85: feature_name=cg12948116	feature_id[4375].value <= threshold=0.5658791363239288
node_86: feature_name=cg06038180	feature_id[2529].value > threshold=0.43877437710762024
node_88: feature_name=cg15720017	feature_id[5175].value > threshold=0.7009969055652618
node_90: feature_name=cg13070215	feature_id[314].value <= threshold=0.14645694941282272
node_91: feature_name=cg01014262	feature_id[1082].value <= threshold=0.5737917125225067
node_92: feature_name=cg16863382	feature_id[5471].value > threshold=0.7200668156147003
node_102: feature_name=cg06633739	feature_id[2686].value > threshold=0.47115950286388397
node_126: feature_name=cg22946562	feature_id[744].value > threshold=0.6194809079170227
node_170: feature_name=cg12120430	feature_id[4185].value > threshold=0.5775820016860962
node_172: feature_name=cg02137970	feature_id[1411].value > threshold=0.6705857813358307

node_196: feature_name=cg03393445	feature_id[1807].value > threshold=0.4260125905275345
node_218: feature_name=cg23525541	feature_id[7049].value > threshold=0.4485483318567276
node_222: feature_name=cg10543634	feature_id[3775].value > threshold=0.29505227506160736
node_224: feature_name=cg07344990	feature_id[2926].value > threshold=0.5750180780887604
node_226: feature_name=cg10558233	feature_id[3780].value <= threshold=0.7217690348625183
node_227: feature_name=cg08097359	feature_id[3116].value > threshold=0.3598180413246155
node_229: feature_name=cg22737001	feature_id[6843].value <= threshold=0.46281898021698
node_230: feature_name=cg24276624	feature_id[7242].value > threshold=0.6169979274272919
node_232: feature_name=cg04026354	feature_id[1982].value <= threshold=0.42332448065280914
node_233: feature_name=cg06038180	feature_id[2529].value > threshold=0.6430551409721375
node_235: feature_name=cg14104252	feature_id[4735].value > threshold=0.5213949382305145
node_239: feature_name=cg06711298	feature_id[2707].value > threshold=0.36770085990428925
node_241: feature_name=cg11703722	feature_id[4056].value > threshold=0.5125315636396408
node_243: feature_name=cg21230793	feature_id[6514].value > threshold=0.27807849645614624
node_245: feature_name=cg06225767	feature_id[2575].value > threshold=0.18034886568784714
node_247: feature_name=cg00941576	feature_id[1053].value > threshold=0.2476368322968483
node_249: feature_name=cg10149889	feature_id[3676].value > threshold=0.633601188659668
node_257: feature_name=cg07608094	feature_id[2999].value > threshold=0.7820693254470825
node_259: feature_name=cg03315432	feature_id[1783].value > threshold=0.6790387332439423
node_261: feature_name=cg07894983	feature_id[3070].value > threshold=0.3577418476343155
node_263: feature_name=cg15646741	feature_id[5160].value > threshold=0.2826688587665558
node_273: feature_name=cg09701233	feature_id[3546].value > threshold=0.5189756155014038

node_275: feature_name=cg09230938	feature_id[738].value > threshold=0.3108274042606354
node_277: feature_name=cg05334190	feature_id[2341].value > threshold=0.2764553725719452
node_279: feature_name=cg15617847	feature_id[5148].value <= threshold=0.05914711393415928
node_280: feature_name=cg00216961	feature_id[816].value <= threshold=0.42650654911994934
node_281: feature_name=cg23946462	feature_id[7146].value > threshold=0.9469821751117706
Class: undifferentiated sarcoma (USARC)	
Rules_67	passed counts:4
node_0: feature_name=cg11915444	feature_id[4116].value > threshold=0.3402601182460785
node_10: feature_name=cg12109728	feature_id[4182].value > threshold=0.7068270146846771
node_20: feature_name=cg10844382	feature_id[3856].value > threshold=0.5988894104957581
node_24: feature_name=cg00631702	feature_id[943].value <= threshold=0.37470583617687225
node_25: feature_name=cg07912144	feature_id[3075].value > threshold=0.5578519105911255
node_29: feature_name=cg24407065	feature_id[388].value > threshold=0.5364363789558411
node_33: feature_name=cg15275017	feature_id[5069].value > threshold=0.4538573920726776
node_51: feature_name=cg07281938	feature_id[351].value > threshold=0.7506992518901825
node_73: feature_name=cg17524821	feature_id[5626].value > threshold=0.4504699558019638
node_75: feature_name=cg02973735	feature_id[1677].value > threshold=0.29383400082588196
node_77: feature_name=cg23345038	feature_id[7002].value > threshold=0.6255057156085968
node_79: feature_name=cg17537493	feature_id[5630].value > threshold=0.5825372636318207
node_85: feature_name=cg12948116	feature_id[4375].value <= threshold=0.5658791363239288
node_86: feature_name=cg06038180	feature_id[2529].value > threshold=0.43877437710762024
node_88: feature_name=cg15720017	feature_id[5175].value > threshold=0.7009969055652618

node_90: feature_name=cg13070215	feature_id[314].value <= threshold=0.14645694941282272
node_91: feature_name=cg01014262	feature_id[1082].value <= threshold=0.5737917125225067
node_92: feature_name=cg16863382	feature_id[5471].value > threshold=0.7200668156147003
node_102: feature_name=cg06633739	feature_id[2686].value > threshold=0.47115950286388397
node_126: feature_name=cg22946562	feature_id[744].value > threshold=0.6194809079170227
node_170: feature_name=cg12120430	feature_id[4185].value > threshold=0.5775820016860962
node_172: feature_name=cg02137970	feature_id[1411].value > threshold=0.6705857813358307
node_196: feature_name=cg03393445	feature_id[1807].value > threshold=0.4260125905275345
node_218: feature_name=cg23525541	feature_id[7049].value > threshold=0.4485483318567276
node_222: feature_name=cg10543634	feature_id[3775].value > threshold=0.29505227506160736
node_224: feature_name=cg07344990	feature_id[2926].value > threshold=0.5750180780887604
node_226: feature_name=cg10558233	feature_id[3780].value <= threshold=0.7217690348625183
node_227: feature_name=cg08097359	feature_id[3116].value > threshold=0.3598180413246155
node_229: feature_name=cg22737001	feature_id[6843].value <= threshold=0.46281898021698
node_230: feature_name=cg24276624	feature_id[7242].value > threshold=0.6169979274272919
node_232: feature_name=cg04026354	feature_id[1982].value <= threshold=0.42332448065280914
node_233: feature_name=cg06038180	feature_id[2529].value > threshold=0.6430551409721375
node_235: feature_name=cg14104252	feature_id[4735].value > threshold=0.5213949382305145
node_239: feature_name=cg06711298	feature_id[2707].value > threshold=0.36770085990428925
node_241: feature_name=cg11703722	feature_id[4056].value > threshold=0.5125315636396408
node_243: feature_name=cg21230793	feature_id[6514].value > threshold=0.27807849645614624
node_245: feature_name=cg06225767	feature_id[2575].value > threshold=0.18034886568784714

node_247: feature_name=cg00941576	feature_id[1053].value > threshold=0.2476368322968483
node_249: feature_name=cg10149889	feature_id[3676].value > threshold=0.633601188659668
node_257: feature_name=cg07608094	feature_id[2999].value > threshold=0.7820693254470825
node_259: feature_name=cg03315432	feature_id[1783].value > threshold=0.6790387332439423
node_261: feature_name=cg07894983	feature_id[3070].value > threshold=0.3577418476343155
node_263: feature_name=cg15646741	feature_id[5160].value > threshold=0.2826688587665558
node_273: feature_name=cg09701233	feature_id[3546].value > threshold=0.5189756155014038
node_275: feature_name=cg09230938	feature_id[738].value > threshold=0.3108274042606354
node_277: feature_name=cg05334190	feature_id[2341].value > threshold=0.2764553725719452
node_279: feature_name=cg15617847	feature_id[5148].value <= threshold=0.05914711393415928
node_280: feature_name=cg00216961	feature_id[816].value <= threshold=0.42650654911994934
node_281: feature_name=cg23946462	feature_id[7146].value <= threshold=0.9469821751117706
node_282: feature_name=cg06143290	feature_id[2554].value > threshold=0.9263531863689423
Class: well differentiated liposarcoma (WDLS)/dedifferentiated liposarcoma (DDLS)	
Rules_68	passed counts:4
node_0: feature_name=cg11915444	feature_id[4116].value > threshold=0.3402601182460785
node_10: feature_name=cg12109728	feature_id[4182].value > threshold=0.7068270146846771
node_20: feature_name=cg10844382	feature_id[3856].value > threshold=0.5988894104957581
node_24: feature_name=cg00631702	feature_id[943].value <= threshold=0.37470583617687225
node_25: feature_name=cg07912144	feature_id[3075].value > threshold=0.5578519105911255
node_29: feature_name=cg24407065	feature_id[388].value > threshold=0.5364363789558411
node_33: feature_name=cg15275017	feature_id[5069].value > threshold=0.4538573920726776

node_51: feature_name=cg07281938	feature_id[351].value > threshold=0.7506992518901825
node_73: feature_name=cg17524821	feature_id[5626].value > threshold=0.4504699558019638
node_75: feature_name=cg02973735	feature_id[1677].value > threshold=0.29383400082588196
node_77: feature_name=cg23345038	feature_id[7002].value > threshold=0.6255057156085968
node_79: feature_name=cg17537493	feature_id[5630].value > threshold=0.5825372636318207
node_85: feature_name=cg12948116	feature_id[4375].value <= threshold=0.5658791363239288
node_86: feature_name=cg06038180	feature_id[2529].value > threshold=0.43877437710762024
node_88: feature_name=cg15720017	feature_id[5175].value > threshold=0.7009969055652618
node_90: feature_name=cg13070215	feature_id[314].value <= threshold=0.14645694941282272
node_91: feature_name=cg01014262	feature_id[1082].value <= threshold=0.5737917125225067
node_92: feature_name=cg16863382	feature_id[5471].value > threshold=0.7200668156147003
node_102: feature_name=cg06633739	feature_id[2686].value > threshold=0.47115950286388397
node_126: feature_name=cg22946562	feature_id[744].value > threshold=0.6194809079170227
node_170: feature_name=cg12120430	feature_id[4185].value > threshold=0.5775820016860962
node_172: feature_name=cg02137970	feature_id[1411].value <= threshold=0.6705857813358307
node_173: feature_name=cg16185457	feature_id[5282].value <= threshold=0.5055856853723526
node_174: feature_name=cg09040552	feature_id[3370].value > threshold=0.596051037311554
node_176: feature_name=cg12064373	feature_id[4162].value <= threshold=0.10608945414423943
node_177: feature_name=cg04874782	feature_id[2203].value <= threshold=0.24745557457208633
node_178: feature_name=cg10963218	feature_id[3883].value <= threshold=0.6643033921718597
Class: chondroblastoma (CB)	
Rules_69	passed counts:4

node_0: feature_name=cg11915444	feature_id[4116].value > threshold=0.3402601182460785
node_10: feature_name=cg12109728	feature_id[4182].value > threshold=0.7068270146846771
node_20: feature_name=cg10844382	feature_id[3856].value > threshold=0.5988894104957581
node_24: feature_name=cg00631702	feature_id[943].value <= threshold=0.37470583617687225
node_25: feature_name=cg07912144	feature_id[3075].value > threshold=0.5578519105911255
node_29: feature_name=cg24407065	feature_id[388].value > threshold=0.5364363789558411
node_33: feature_name=cg15275017	feature_id[5069].value > threshold=0.4538573920726776
node_51: feature_name=cg07281938	feature_id[351].value > threshold=0.7506992518901825
node_73: feature_name=cg17524821	feature_id[5626].value > threshold=0.4504699558019638
node_75: feature_name=cg02973735	feature_id[1677].value > threshold=0.29383400082588196
node_77: feature_name=cg23345038	feature_id[7002].value > threshold=0.6255057156085968
node_79: feature_name=cg17537493	feature_id[5630].value > threshold=0.5825372636318207
node_85: feature_name=cg12948116	feature_id[4375].value <= threshold=0.5658791363239288
node_86: feature_name=cg06038180	feature_id[2529].value > threshold=0.43877437710762024
node_88: feature_name=cg15720017	feature_id[5175].value > threshold=0.7009969055652618
node_90: feature_name=cg13070215	feature_id[314].value <= threshold=0.14645694941282272
node_91: feature_name=cg01014262	feature_id[1082].value <= threshold=0.5737917125225067
node_92: feature_name=cg16863382	feature_id[5471].value > threshold=0.7200668156147003
node_102: feature_name=cg06633739	feature_id[2686].value > threshold=0.47115950286388397
node_126: feature_name=cg22946562	feature_id[744].value <= threshold=0.6194809079170227
node_127: feature_name=cg23920016	feature_id[7139].value > threshold=0.7449243068695068
node_151: feature_name=cg21617218	feature_id[6608].value > threshold=0.8795748353004456

node_153: feature_name=cg01663970	feature_id[1273].value > threshold=0.8442256152629852
node_155: feature_name=cg00686915	feature_id[965].value <= threshold=0.20000632852315903
Class: clear cell sarcoma of soft parts (CCS)	
Rules_70	passed counts:4
node_0: feature_name=cg11915444	feature_id[4116].value > threshold=0.3402601182460785
node_10: feature_name=cg12109728	feature_id[4182].value > threshold=0.7068270146846771
node_20: feature_name=cg10844382	feature_id[3856].value > threshold=0.5988894104957581
node_24: feature_name=cg00631702	feature_id[943].value <= threshold=0.37470583617687225
node_25: feature_name=cg07912144	feature_id[3075].value > threshold=0.5578519105911255
node_29: feature_name=cg24407065	feature_id[388].value > threshold=0.5364363789558411
node_33: feature_name=cg15275017	feature_id[5069].value > threshold=0.4538573920726776
node_51: feature_name=cg07281938	feature_id[351].value > threshold=0.7506992518901825
node_73: feature_name=cg17524821	feature_id[5626].value > threshold=0.4504699558019638
node_75: feature_name=cg02973735	feature_id[1677].value > threshold=0.29383400082588196
node_77: feature_name=cg23345038	feature_id[7002].value > threshold=0.6255057156085968
node_79: feature_name=cg17537493	feature_id[5630].value <= threshold=0.5825372636318207
node_80: feature_name=cg07990390	feature_id[515].value > threshold=0.7988054752349854
node_82: feature_name=cg10006956	feature_id[3627].value <= threshold=0.9051482081413269
Class: Kaposi sarcoma (KS)	
Rules_71	passed counts:4
node_0: feature_name=cg11915444	feature_id[4116].value > threshold=0.3402601182460785
node_10: feature_name=cg12109728	feature_id[4182].value > threshold=0.7068270146846771

node_20: feature_name=cg10844382	feature_id[3856].value > threshold=0.5988894104957581
node_24: feature_name=cg00631702	feature_id[943].value <= threshold=0.37470583617687225
node_25: feature_name=cg07912144	feature_id[3075].value > threshold=0.5578519105911255
node_29: feature_name=cg24407065	feature_id[388].value > threshold=0.5364363789558411
node_33: feature_name=cg15275017	feature_id[5069].value > threshold=0.4538573920726776
node_51: feature_name=cg07281938	feature_id[351].value <= threshold=0.7506992518901825
node_52: feature_name=cg21189849	feature_id[6504].value > threshold=0.7545044124126434
node_58: feature_name=cg02867857	feature_id[1644].value > threshold=0.5450765490531921
node_60: feature_name=cg00863893	feature_id[1017].value <= threshold=0.5313664376735687
node_61: feature_name=cg22397446	feature_id[6765].value > threshold=0.02860488835722208
Class: clear cell chondrosarcoma (CSA (CC))	
Rules_72	passed counts:3
node_0: feature_name=cg11915444	feature_id[4116].value > threshold=0.3402601182460785
node_10: feature_name=cg12109728	feature_id[4182].value > threshold=0.7068270146846771
node_20: feature_name=cg10844382	feature_id[3856].value > threshold=0.5988894104957581
node_24: feature_name=cg00631702	feature_id[943].value > threshold=0.37470583617687225
node_352: feature_name=cg07027613	feature_id[549].value <= threshold=0.22842608392238617
node_353: feature_name=cg04356968	feature_id[2068].value > threshold=0.728985995054245
Class: malignant peripheral nerve sheath tumour (MPNST)	
Rules_73	passed counts:3
node_0: feature_name=cg11915444	feature_id[4116].value > threshold=0.3402601182460785
node_10: feature_name=cg12109728	feature_id[4182].value > threshold=0.7068270146846771

node_20: feature_name=cg10844382	feature_id[3856].value > threshold=0.5988894104957581
node_24: feature_name=cg00631702	feature_id[943].value <= threshold=0.37470583617687225
node_25: feature_name=cg07912144	feature_id[3075].value > threshold=0.5578519105911255
node_29: feature_name=cg24407065	feature_id[388].value > threshold=0.5364363789558411
node_33: feature_name=cg15275017	feature_id[5069].value > threshold=0.4538573920726776
node_51: feature_name=cg07281938	feature_id[351].value > threshold=0.7506992518901825
node_73: feature_name=cg17524821	feature_id[5626].value > threshold=0.4504699558019638
node_75: feature_name=cg02973735	feature_id[1677].value > threshold=0.29383400082588196
node_77: feature_name=cg23345038	feature_id[7002].value > threshold=0.6255057156085968
node_79: feature_name=cg17537493	feature_id[5630].value > threshold=0.5825372636318207
node_85: feature_name=cg12948116	feature_id[4375].value <= threshold=0.5658791363239288
node_86: feature_name=cg06038180	feature_id[2529].value > threshold=0.43877437710762024
node_88: feature_name=cg15720017	feature_id[5175].value > threshold=0.7009969055652618
node_90: feature_name=cg13070215	feature_id[314].value <= threshold=0.14645694941282272
node_91: feature_name=cg01014262	feature_id[1082].value <= threshold=0.5737917125225067
node_92: feature_name=cg16863382	feature_id[5471].value > threshold=0.7200668156147003
node_102: feature_name=cg06633739	feature_id[2686].value > threshold=0.47115950286388397
node_126: feature_name=cg22946562	feature_id[744].value > threshold=0.6194809079170227
node_170: feature_name=cg12120430	feature_id[4185].value > threshold=0.5775820016860962
node_172: feature_name=cg02137970	feature_id[1411].value > threshold=0.6705857813358307
node_196: feature_name=cg03393445	feature_id[1807].value > threshold=0.4260125905275345
node_218: feature_name=cg23525541	feature_id[7049].value > threshold=0.4485483318567276

node_222: feature_name=cg10543634	feature_id[3775].value > threshold=0.29505227506160736
node_224: feature_name=cg07344990	feature_id[2926].value > threshold=0.5750180780887604
node_226: feature_name=cg10558233	feature_id[3780].value <= threshold=0.7217690348625183
node_227: feature_name=cg08097359	feature_id[3116].value > threshold=0.3598180413246155
node_229: feature_name=cg22737001	feature_id[6843].value <= threshold=0.46281898021698
node_230: feature_name=cg24276624	feature_id[7242].value > threshold=0.6169979274272919
node_232: feature_name=cg04026354	feature_id[1982].value <= threshold=0.42332448065280914
node_233: feature_name=cg06038180	feature_id[2529].value > threshold=0.6430551409721375
node_235: feature_name=cg14104252	feature_id[4735].value > threshold=0.5213949382305145
node_239: feature_name=cg06711298	feature_id[2707].value > threshold=0.36770085990428925
node_241: feature_name=cg11703722	feature_id[4056].value > threshold=0.5125315636396408
node_243: feature_name=cg21230793	feature_id[6514].value > threshold=0.27807849645614624
node_245: feature_name=cg06225767	feature_id[2575].value > threshold=0.18034886568784714
node_247: feature_name=cg00941576	feature_id[1053].value > threshold=0.2476368322968483
node_249: feature_name=cg10149889	feature_id[3676].value > threshold=0.633601188659668
node_257: feature_name=cg07608094	feature_id[2999].value > threshold=0.7820693254470825
node_259: feature_name=cg03315432	feature_id[1783].value > threshold=0.6790387332439423
node_261: feature_name=cg07894983	feature_id[3070].value > threshold=0.3577418476343155
node_263: feature_name=cg15646741	feature_id[5160].value > threshold=0.2826688587665558
node_273: feature_name=cg09701233	feature_id[3546].value > threshold=0.5189756155014038
node_275: feature_name=cg09230938	feature_id[738].value > threshold=0.3108274042606354
node_277: feature_name=cg05334190	feature_id[2341].value > threshold=0.2764553725719452

node_279: feature_name=cg15617847	feature_id[5148].value <= threshold=0.05914711393415928
node_280: feature_name=cg00216961	feature_id[816].value <= threshold=0.42650654911994934
node_281: feature_name=cg23946462	feature_id[7146].value <= threshold=0.9469821751117706
node_282: feature_name=cg06143290	feature_id[2554].value <= threshold=0.9263531863689423
node_283: feature_name=cg21528710	feature_id[6588].value > threshold=0.8718287348747253
Class: atypical fibroxanthoma (AFX)/pleomorphic dermal sarcoma (PDS)	
Rules_74	passed counts:3
node_0: feature_name=cg11915444	feature_id[4116].value > threshold=0.3402601182460785
node_10: feature_name=cg12109728	feature_id[4182].value > threshold=0.7068270146846771
node_20: feature_name=cg10844382	feature_id[3856].value > threshold=0.5988894104957581
node_24: feature_name=cg00631702	feature_id[943].value <= threshold=0.37470583617687225
node_25: feature_name=cg07912144	feature_id[3075].value > threshold=0.5578519105911255
node_29: feature_name=cg24407065	feature_id[388].value > threshold=0.5364363789558411
node_33: feature_name=cg15275017	feature_id[5069].value > threshold=0.4538573920726776
node_51: feature_name=cg07281938	feature_id[351].value > threshold=0.7506992518901825
node_73: feature_name=cg17524821	feature_id[5626].value > threshold=0.4504699558019638
node_75: feature_name=cg02973735	feature_id[1677].value > threshold=0.29383400082588196
node_77: feature_name=cg23345038	feature_id[7002].value > threshold=0.6255057156085968
node_79: feature_name=cg17537493	feature_id[5630].value > threshold=0.5825372636318207
node_85: feature_name=cg12948116	feature_id[4375].value <= threshold=0.5658791363239288
node_86: feature_name=cg06038180	feature_id[2529].value > threshold=0.43877437710762024
node_88: feature_name=cg15720017	feature_id[5175].value > threshold=0.7009969055652618

node_90: feature_name=cg13070215	feature_id[314].value <= threshold=0.14645694941282272
node_91: feature_name=cg01014262	feature_id[1082].value <= threshold=0.5737917125225067
node_92: feature_name=cg16863382	feature_id[5471].value > threshold=0.7200668156147003
node_102: feature_name=cg06633739	feature_id[2686].value > threshold=0.47115950286388397
node_126: feature_name=cg22946562	feature_id[744].value > threshold=0.6194809079170227
node_170: feature_name=cg12120430	feature_id[4185].value > threshold=0.5775820016860962
node_172: feature_name=cg02137970	feature_id[1411].value > threshold=0.6705857813358307
node_196: feature_name=cg03393445	feature_id[1807].value > threshold=0.4260125905275345
node_218: feature_name=cg23525541	feature_id[7049].value > threshold=0.4485483318567276
node_222: feature_name=cg10543634	feature_id[3775].value > threshold=0.29505227506160736
node_224: feature_name=cg07344990	feature_id[2926].value > threshold=0.5750180780887604
node_226: feature_name=cg10558233	feature_id[3780].value <= threshold=0.7217690348625183
node_227: feature_name=cg08097359	feature_id[3116].value > threshold=0.3598180413246155
node_229: feature_name=cg22737001	feature_id[6843].value <= threshold=0.46281898021698
node_230: feature_name=cg24276624	feature_id[7242].value > threshold=0.6169979274272919
node_232: feature_name=cg04026354	feature_id[1982].value <= threshold=0.42332448065280914
node_233: feature_name=cg06038180	feature_id[2529].value > threshold=0.6430551409721375
node_235: feature_name=cg14104252	feature_id[4735].value > threshold=0.5213949382305145
node_239: feature_name=cg06711298	feature_id[2707].value > threshold=0.36770085990428925
node_241: feature_name=cg11703722	feature_id[4056].value > threshold=0.5125315636396408
node_243: feature_name=cg21230793	feature_id[6514].value > threshold=0.27807849645614624
node_245: feature_name=cg06225767	feature_id[2575].value > threshold=0.18034886568784714

node_247: feature_name=cg00941576	feature_id[1053].value > threshold=0.2476368322968483
node_249: feature_name=cg10149889	feature_id[3676].value > threshold=0.633601188659668
node_257: feature_name=cg07608094	feature_id[2999].value > threshold=0.7820693254470825
node_259: feature_name=cg03315432	feature_id[1783].value > threshold=0.6790387332439423
node_261: feature_name=cg07894983	feature_id[3070].value > threshold=0.3577418476343155
node_263: feature_name=cg15646741	feature_id[5160].value <= threshold=0.2826688587665558
node_264: feature_name=cg05400196	feature_id[2366].value <= threshold=0.666697084903717
node_265: feature_name=cg11936643	feature_id[4120].value <= threshold=0.19320054352283478
Class: chondroblastoma (CB)	
Rules_75	passed counts:3
node_0: feature_name=cg11915444	feature_id[4116].value > threshold=0.3402601182460785
node_10: feature_name=cg12109728	feature_id[4182].value > threshold=0.7068270146846771
node_20: feature_name=cg10844382	feature_id[3856].value > threshold=0.5988894104957581
node_24: feature_name=cg00631702	feature_id[943].value <= threshold=0.37470583617687225
node_25: feature_name=cg07912144	feature_id[3075].value > threshold=0.5578519105911255
node_29: feature_name=cg24407065	feature_id[388].value > threshold=0.5364363789558411
node_33: feature_name=cg15275017	feature_id[5069].value > threshold=0.4538573920726776
node_51: feature_name=cg07281938	feature_id[351].value > threshold=0.7506992518901825
node_73: feature_name=cg17524821	feature_id[5626].value > threshold=0.4504699558019638
node_75: feature_name=cg02973735	feature_id[1677].value > threshold=0.29383400082588196
node_77: feature_name=cg23345038	feature_id[7002].value > threshold=0.6255057156085968
node_79: feature_name=cg17537493	feature_id[5630].value > threshold=0.5825372636318207

node_85: feature_name=cg12948116	feature_id[4375].value <= threshold=0.5658791363239288
node_86: feature_name=cg06038180	feature_id[2529].value > threshold=0.43877437710762024
node_88: feature_name=cg15720017	feature_id[5175].value > threshold=0.7009969055652618
node_90: feature_name=cg13070215	feature_id[314].value <= threshold=0.14645694941282272
node_91: feature_name=cg01014262	feature_id[1082].value <= threshold=0.5737917125225067
node_92: feature_name=cg16863382	feature_id[5471].value > threshold=0.7200668156147003
node_102: feature_name=cg06633739	feature_id[2686].value > threshold=0.47115950286388397
node_126: feature_name=cg22946562	feature_id[744].value <= threshold=0.6194809079170227
node_127: feature_name=cg23920016	feature_id[7139].value <= threshold=0.7449243068695068
node_128: feature_name=cg16521032	feature_id[5379].value <= threshold=0.8948074877262115
node_129: feature_name=cg09597070	feature_id[555].value <= threshold=0.67006054520607
node_130: feature_name=cg08411881	feature_id[3200].value > threshold=0.7572305798530579
Class: sarcoma (SARC)	
Rules_76	passed counts:3
node_0: feature_name=cg11915444	feature_id[4116].value > threshold=0.3402601182460785
node_10: feature_name=cg12109728	feature_id[4182].value > threshold=0.7068270146846771
node_20: feature_name=cg10844382	feature_id[3856].value > threshold=0.5988894104957581
node_24: feature_name=cg00631702	feature_id[943].value <= threshold=0.37470583617687225
node_25: feature_name=cg07912144	feature_id[3075].value > threshold=0.5578519105911255
node_29: feature_name=cg24407065	feature_id[388].value > threshold=0.5364363789558411
node_33: feature_name=cg15275017	feature_id[5069].value > threshold=0.4538573920726776
node_51: feature_name=cg07281938	feature_id[351].value > threshold=0.7506992518901825

node_73: feature_name=cg17524821	feature_id[5626].value > threshold=0.4504699558019638
node_75: feature_name=cg02973735	feature_id[1677].value > threshold=0.29383400082588196
node_77: feature_name=cg23345038	feature_id[7002].value > threshold=0.6255057156085968
node_79: feature_name=cg17537493	feature_id[5630].value > threshold=0.5825372636318207
node_85: feature_name=cg12948116	feature_id[4375].value <= threshold=0.5658791363239288
node_86: feature_name=cg06038180	feature_id[2529].value > threshold=0.43877437710762024
node_88: feature_name=cg15720017	feature_id[5175].value > threshold=0.7009969055652618
node_90: feature_name=cg13070215	feature_id[314].value <= threshold=0.14645694941282272
node_91: feature_name=cg01014262	feature_id[1082].value <= threshold=0.5737917125225067
node_92: feature_name=cg16863382	feature_id[5471].value <= threshold=0.7200668156147003
node_93: feature_name=cg18601229	feature_id[5883].value <= threshold=0.7762220203876495
node_94: feature_name=cg04819180	feature_id[2191].value > threshold=0.8672285974025726
Class: alveolar rhabdomyosarcoma (RMS (ALV))	
Rules_77	passed counts:3
node_0: feature_name=cg11915444	feature_id[4116].value > threshold=0.3402601182460785
node_10: feature_name=cg12109728	feature_id[4182].value > threshold=0.7068270146846771
node_20: feature_name=cg10844382	feature_id[3856].value > threshold=0.5988894104957581
node_24: feature_name=cg00631702	feature_id[943].value <= threshold=0.37470583617687225
node_25: feature_name=cg07912144	feature_id[3075].value > threshold=0.5578519105911255
node_29: feature_name=cg24407065	feature_id[388].value > threshold=0.5364363789558411
node_33: feature_name=cg15275017	feature_id[5069].value <= threshold=0.4538573920726776
node_34: feature_name=cg08236537	feature_id[3160].value <= threshold=0.4830351173877716

node_35: feature_name=cg21591452	feature_id[6603].value <= threshold=0.878025621175766
node_36: feature_name=cg07368061	feature_id[2933].value <= threshold=0.6850627958774567
node_37: feature_name=cg21562750	feature_id[6600].value <= threshold=0.680396169424057
Class: sarcoma (SARC)	
Rules_78	passed counts:2
node_0: feature_name=cg11915444	feature_id[4116].value > threshold=0.3402601182460785
node_10: feature_name=cg12109728	feature_id[4182].value > threshold=0.7068270146846771
node_20: feature_name=cg10844382	feature_id[3856].value > threshold=0.5988894104957581
node_24: feature_name=cg00631702	feature_id[943].value > threshold=0.37470583617687225
node_352: feature_name=cg07027613	feature_id[549].value <= threshold=0.22842608392238617
node_353: feature_name=cg04356968	feature_id[2068].value <= threshold=0.728985995054245
node_354: feature_name=cg18259342	feature_id[5811].value <= threshold=0.1999226212501526
Class: giant cell tumour of bone (GCTB)	
Rules_79	passed counts:2
node_0: feature_name=cg11915444	feature_id[4116].value > threshold=0.3402601182460785
node_10: feature_name=cg12109728	feature_id[4182].value > threshold=0.7068270146846771
node_20: feature_name=cg10844382	feature_id[3856].value > threshold=0.5988894104957581
node_24: feature_name=cg00631702	feature_id[943].value <= threshold=0.37470583617687225
node_25: feature_name=cg07912144	feature_id[3075].value > threshold=0.5578519105911255
node_29: feature_name=cg24407065	feature_id[388].value > threshold=0.5364363789558411
node_33: feature_name=cg15275017	feature_id[5069].value > threshold=0.4538573920726776
node_51: feature_name=cg07281938	feature_id[351].value > threshold=0.7506992518901825

node_73: feature_name=cg17524821	feature_id[5626].value > threshold=0.4504699558019638
node_75: feature_name=cg02973735	feature_id[1677].value > threshold=0.29383400082588196
node_77: feature_name=cg23345038	feature_id[7002].value > threshold=0.6255057156085968
node_79: feature_name=cg17537493	feature_id[5630].value > threshold=0.5825372636318207
node_85: feature_name=cg12948116	feature_id[4375].value <= threshold=0.5658791363239288
node_86: feature_name=cg06038180	feature_id[2529].value > threshold=0.43877437710762024
node_88: feature_name=cg15720017	feature_id[5175].value > threshold=0.7009969055652618
node_90: feature_name=cg13070215	feature_id[314].value > threshold=0.14645694941282272
node_340: feature_name=cg06272543	feature_id[2590].value <= threshold=0.07370422407984734
node_341: feature_name=cg24268698	feature_id[7238].value <= threshold=0.9151104986667633
node_342: feature_name=cg12669088	feature_id[398].value > threshold=0.5310345143079758
Class: leiomyosarcoma (LMS)	
Rules_80	passed counts:2
node_0: feature_name=cg11915444	feature_id[4116].value > threshold=0.3402601182460785
node_10: feature_name=cg12109728	feature_id[4182].value > threshold=0.7068270146846771
node_20: feature_name=cg10844382	feature_id[3856].value > threshold=0.5988894104957581
node_24: feature_name=cg00631702	feature_id[943].value <= threshold=0.37470583617687225
node_25: feature_name=cg07912144	feature_id[3075].value > threshold=0.5578519105911255
node_29: feature_name=cg24407065	feature_id[388].value > threshold=0.5364363789558411
node_33: feature_name=cg15275017	feature_id[5069].value > threshold=0.4538573920726776
node_51: feature_name=cg07281938	feature_id[351].value > threshold=0.7506992518901825
node_73: feature_name=cg17524821	feature_id[5626].value > threshold=0.4504699558019638

node_75: feature_name=cg02973735	feature_id[1677].value > threshold=0.29383400082588196
node_77: feature_name=cg23345038	feature_id[7002].value > threshold=0.6255057156085968
node_79: feature_name=cg17537493	feature_id[5630].value > threshold=0.5825372636318207
node_85: feature_name=cg12948116	feature_id[4375].value <= threshold=0.5658791363239288
node_86: feature_name=cg06038180	feature_id[2529].value > threshold=0.43877437710762024
node_88: feature_name=cg15720017	feature_id[5175].value > threshold=0.7009969055652618
node_90: feature_name=cg13070215	feature_id[314].value <= threshold=0.14645694941282272
node_91: feature_name=cg01014262	feature_id[1082].value > threshold=0.5737917125225067
node_337: feature_name=cg19909613	feature_id[6202].value <= threshold=0.8301863670349121
Class: Langerhans cell histiocytosis (LCH)	
Rules_81	passed counts:2
node_0: feature_name=cg11915444	feature_id[4116].value > threshold=0.3402601182460785
node_10: feature_name=cg12109728	feature_id[4182].value > threshold=0.7068270146846771
node_20: feature_name=cg10844382	feature_id[3856].value > threshold=0.5988894104957581
node_24: feature_name=cg00631702	feature_id[943].value <= threshold=0.37470583617687225
node_25: feature_name=cg07912144	feature_id[3075].value > threshold=0.5578519105911255
node_29: feature_name=cg24407065	feature_id[388].value > threshold=0.5364363789558411
node_33: feature_name=cg15275017	feature_id[5069].value > threshold=0.4538573920726776
node_51: feature_name=cg07281938	feature_id[351].value > threshold=0.7506992518901825
node_73: feature_name=cg17524821	feature_id[5626].value > threshold=0.4504699558019638
node_75: feature_name=cg02973735	feature_id[1677].value > threshold=0.29383400082588196
node_77: feature_name=cg23345038	feature_id[7002].value > threshold=0.6255057156085968

node_79: feature_name=cg17537493	feature_id[5630].value > threshold=0.5825372636318207
node_85: feature_name=cg12948116	feature_id[4375].value <= threshold=0.5658791363239288
node_86: feature_name=cg06038180	feature_id[2529].value > threshold=0.43877437710762024
node_88: feature_name=cg15720017	feature_id[5175].value > threshold=0.7009969055652618
node_90: feature_name=cg13070215	feature_id[314].value <= threshold=0.14645694941282272
node_91: feature_name=cg01014262	feature_id[1082].value <= threshold=0.5737917125225067
node_92: feature_name=cg16863382	feature_id[5471].value > threshold=0.7200668156147003
node_102: feature_name=cg06633739	feature_id[2686].value > threshold=0.47115950286388397
node_126: feature_name=cg22946562	feature_id[744].value > threshold=0.6194809079170227
node_170: feature_name=cg12120430	feature_id[4185].value > threshold=0.5775820016860962
node_172: feature_name=cg02137970	feature_id[1411].value > threshold=0.6705857813358307
node_196: feature_name=cg03393445	feature_id[1807].value > threshold=0.4260125905275345
node_218: feature_name=cg23525541	feature_id[7049].value > threshold=0.4485483318567276
node_222: feature_name=cg10543634	feature_id[3775].value > threshold=0.29505227506160736
node_224: feature_name=cg07344990	feature_id[2926].value > threshold=0.5750180780887604
node_226: feature_name=cg10558233	feature_id[3780].value <= threshold=0.7217690348625183
node_227: feature_name=cg08097359	feature_id[3116].value > threshold=0.3598180413246155
node_229: feature_name=cg22737001	feature_id[6843].value <= threshold=0.46281898021698
node_230: feature_name=cg24276624	feature_id[7242].value > threshold=0.6169979274272919
node_232: feature_name=cg04026354	feature_id[1982].value > threshold=0.42332448065280914
node_316: feature_name=cg23693485	feature_id[7083].value > threshold=0.7056963741779327
node_320: feature_name=cg16027775	feature_id[5238].value > threshold=0.17370137572288513

node_322: feature_name=cg16820411	feature_id[5464].value > threshold=0.9055586755275726
node_324: feature_name=cg07990658	feature_id[3094].value <= threshold=0.9101644456386566
Class: Kaposi sarcoma (KS)	
Rules_82	passed counts:2
node_0: feature_name=cg11915444	feature_id[4116].value > threshold=0.3402601182460785
node_10: feature_name=cg12109728	feature_id[4182].value > threshold=0.7068270146846771
node_20: feature_name=cg10844382	feature_id[3856].value > threshold=0.5988894104957581
node_24: feature_name=cg00631702	feature_id[943].value <= threshold=0.37470583617687225
node_25: feature_name=cg07912144	feature_id[3075].value > threshold=0.5578519105911255
node_29: feature_name=cg24407065	feature_id[388].value > threshold=0.5364363789558411
node_33: feature_name=cg15275017	feature_id[5069].value > threshold=0.4538573920726776
node_51: feature_name=cg07281938	feature_id[351].value > threshold=0.7506992518901825
node_73: feature_name=cg17524821	feature_id[5626].value > threshold=0.4504699558019638
node_75: feature_name=cg02973735	feature_id[1677].value > threshold=0.29383400082588196
node_77: feature_name=cg23345038	feature_id[7002].value > threshold=0.6255057156085968
node_79: feature_name=cg17537493	feature_id[5630].value > threshold=0.5825372636318207
node_85: feature_name=cg12948116	feature_id[4375].value <= threshold=0.5658791363239288
node_86: feature_name=cg06038180	feature_id[2529].value > threshold=0.43877437710762024
node_88: feature_name=cg15720017	feature_id[5175].value > threshold=0.7009969055652618
node_90: feature_name=cg13070215	feature_id[314].value <= threshold=0.14645694941282272
node_91: feature_name=cg01014262	feature_id[1082].value <= threshold=0.5737917125225067
node_92: feature_name=cg16863382	feature_id[5471].value > threshold=0.7200668156147003

node_102: feature_name=cg06633739	feature_id[2686].value > threshold=0.47115950286388397
node_126: feature_name=cg22946562	feature_id[744].value > threshold=0.6194809079170227
node_170: feature_name=cg12120430	feature_id[4185].value > threshold=0.5775820016860962
node_172: feature_name=cg02137970	feature_id[1411].value > threshold=0.6705857813358307
node_196: feature_name=cg03393445	feature_id[1807].value > threshold=0.4260125905275345
node_218: feature_name=cg23525541	feature_id[7049].value > threshold=0.4485483318567276
node_222: feature_name=cg10543634	feature_id[3775].value > threshold=0.29505227506160736
node_224: feature_name=cg07344990	feature_id[2926].value > threshold=0.5750180780887604
node_226: feature_name=cg10558233	feature_id[3780].value <= threshold=0.7217690348625183
node_227: feature_name=cg08097359	feature_id[3116].value > threshold=0.3598180413246155
node_229: feature_name=cg22737001	feature_id[6843].value <= threshold=0.46281898021698
node_230: feature_name=cg24276624	feature_id[7242].value > threshold=0.6169979274272919
node_232: feature_name=cg04026354	feature_id[1982].value > threshold=0.42332448065280914
node_316: feature_name=cg23693485	feature_id[7083].value > threshold=0.7056963741779327
node_320: feature_name=cg16027775	feature_id[5238].value > threshold=0.17370137572288513
node_322: feature_name=cg16820411	feature_id[5464].value <= threshold=0.9055586755275726
Class: melanoma (MEL)	
Rules_83	passed counts:2
node_0: feature_name=cg11915444	feature_id[4116].value > threshold=0.3402601182460785
node_10: feature_name=cg12109728	feature_id[4182].value > threshold=0.7068270146846771
node_20: feature_name=cg10844382	feature_id[3856].value > threshold=0.5988894104957581
node_24: feature_name=cg00631702	feature_id[943].value <= threshold=0.37470583617687225

node_25: feature_name=cg07912144	feature_id[3075].value > threshold=0.5578519105911255
node_29: feature_name=cg24407065	feature_id[388].value > threshold=0.5364363789558411
node_33: feature_name=cg15275017	feature_id[5069].value > threshold=0.4538573920726776
node_51: feature_name=cg07281938	feature_id[351].value > threshold=0.7506992518901825
node_73: feature_name=cg17524821	feature_id[5626].value > threshold=0.4504699558019638
node_75: feature_name=cg02973735	feature_id[1677].value > threshold=0.29383400082588196
node_77: feature_name=cg23345038	feature_id[7002].value > threshold=0.6255057156085968
node_79: feature_name=cg17537493	feature_id[5630].value > threshold=0.5825372636318207
node_85: feature_name=cg12948116	feature_id[4375].value <= threshold=0.5658791363239288
node_86: feature_name=cg06038180	feature_id[2529].value > threshold=0.43877437710762024
node_88: feature_name=cg15720017	feature_id[5175].value > threshold=0.7009969055652618
node_90: feature_name=cg13070215	feature_id[314].value <= threshold=0.14645694941282272
node_91: feature_name=cg01014262	feature_id[1082].value <= threshold=0.5737917125225067
node_92: feature_name=cg16863382	feature_id[5471].value > threshold=0.7200668156147003
node_102: feature_name=cg06633739	feature_id[2686].value > threshold=0.47115950286388397
node_126: feature_name=cg22946562	feature_id[744].value > threshold=0.6194809079170227
node_170: feature_name=cg12120430	feature_id[4185].value > threshold=0.5775820016860962
node_172: feature_name=cg02137970	feature_id[1411].value > threshold=0.6705857813358307
node_196: feature_name=cg03393445	feature_id[1807].value > threshold=0.4260125905275345
node_218: feature_name=cg23525541	feature_id[7049].value > threshold=0.4485483318567276
node_222: feature_name=cg10543634	feature_id[3775].value > threshold=0.29505227506160736
node_224: feature_name=cg07344990	feature_id[2926].value > threshold=0.5750180780887604

node_226: feature_name=cg10558233	feature_id[3780].value <= threshold=0.7217690348625183
node_227: feature_name=cg08097359	feature_id[3116].value > threshold=0.3598180413246155
node_229: feature_name=cg22737001	feature_id[6843].value <= threshold=0.46281898021698
node_230: feature_name=cg24276624	feature_id[7242].value > threshold=0.6169979274272919
node_232: feature_name=cg04026354	feature_id[1982].value <= threshold=0.42332448065280914
node_233: feature_name=cg06038180	feature_id[2529].value > threshold=0.6430551409721375
node_235: feature_name=cg14104252	feature_id[4735].value > threshold=0.5213949382305145
node_239: feature_name=cg06711298	feature_id[2707].value > threshold=0.36770085990428925
node_241: feature_name=cg11703722	feature_id[4056].value > threshold=0.5125315636396408
node_243: feature_name=cg21230793	feature_id[6514].value > threshold=0.27807849645614624
node_245: feature_name=cg06225767	feature_id[2575].value > threshold=0.18034886568784714
node_247: feature_name=cg00941576	feature_id[1053].value > threshold=0.2476368322968483
node_249: feature_name=cg10149889	feature_id[3676].value > threshold=0.633601188659668
node_257: feature_name=cg07608094	feature_id[2999].value > threshold=0.7820693254470825
node_259: feature_name=cg03315432	feature_id[1783].value > threshold=0.6790387332439423
node_261: feature_name=cg07894983	feature_id[3070].value > threshold=0.3577418476343155
node_263: feature_name=cg15646741	feature_id[5160].value > threshold=0.2826688587665558
node_273: feature_name=cg09701233	feature_id[3546].value > threshold=0.5189756155014038
node_275: feature_name=cg09230938	feature_id[738].value > threshold=0.3108274042606354
node_277: feature_name=cg05334190	feature_id[2341].value > threshold=0.2764553725719452
node_279: feature_name=cg15617847	feature_id[5148].value <= threshold=0.05914711393415928
node_280: feature_name=cg00216961	feature_id[816].value <= threshold=0.42650654911994934

node_281: feature_name=cg23946462	feature_id[7146].value <= threshold=0.9469821751117706
node_282: feature_name=cg06143290	feature_id[2554].value <= threshold=0.9263531863689423
node_283: feature_name=cg21528710	feature_id[6588].value <= threshold=0.8718287348747253
node_284: feature_name=cg14178043	feature_id[4760].value > threshold=0.6852438449859619
Class: high-grade conventional osteosarcoma (OS (HG))	
Rules_84	passed counts:2
node_0: feature_name=cg11915444	feature_id[4116].value > threshold=0.3402601182460785
node_10: feature_name=cg12109728	feature_id[4182].value > threshold=0.7068270146846771
node_20: feature_name=cg10844382	feature_id[3856].value > threshold=0.5988894104957581
node_24: feature_name=cg00631702	feature_id[943].value <= threshold=0.37470583617687225
node_25: feature_name=cg07912144	feature_id[3075].value > threshold=0.5578519105911255
node_29: feature_name=cg24407065	feature_id[388].value > threshold=0.5364363789558411
node_33: feature_name=cg15275017	feature_id[5069].value > threshold=0.4538573920726776
node_51: feature_name=cg07281938	feature_id[351].value > threshold=0.7506992518901825
node_73: feature_name=cg17524821	feature_id[5626].value > threshold=0.4504699558019638
node_75: feature_name=cg02973735	feature_id[1677].value > threshold=0.29383400082588196
node_77: feature_name=cg23345038	feature_id[7002].value > threshold=0.6255057156085968
node_79: feature_name=cg17537493	feature_id[5630].value > threshold=0.5825372636318207
node_85: feature_name=cg12948116	feature_id[4375].value <= threshold=0.5658791363239288
node_86: feature_name=cg06038180	feature_id[2529].value > threshold=0.43877437710762024
node_88: feature_name=cg15720017	feature_id[5175].value > threshold=0.7009969055652618
node_90: feature_name=cg13070215	feature_id[314].value <= threshold=0.14645694941282272

node_91: feature_name=cg01014262	feature_id[1082].value <= threshold=0.5737917125225067
node_92: feature_name=cg16863382	feature_id[5471].value > threshold=0.7200668156147003
node_102: feature_name=cg06633739	feature_id[2686].value > threshold=0.47115950286388397
node_126: feature_name=cg22946562	feature_id[744].value > threshold=0.6194809079170227
node_170: feature_name=cg12120430	feature_id[4185].value > threshold=0.5775820016860962
node_172: feature_name=cg02137970	feature_id[1411].value > threshold=0.6705857813358307
node_196: feature_name=cg03393445	feature_id[1807].value > threshold=0.4260125905275345
node_218: feature_name=cg23525541	feature_id[7049].value > threshold=0.4485483318567276
node_222: feature_name=cg10543634	feature_id[3775].value > threshold=0.29505227506160736
node_224: feature_name=cg07344990	feature_id[2926].value > threshold=0.5750180780887604
node_226: feature_name=cg10558233	feature_id[3780].value <= threshold=0.7217690348625183
node_227: feature_name=cg08097359	feature_id[3116].value > threshold=0.3598180413246155
node_229: feature_name=cg22737001	feature_id[6843].value <= threshold=0.46281898021698
node_230: feature_name=cg24276624	feature_id[7242].value > threshold=0.6169979274272919
node_232: feature_name=cg04026354	feature_id[1982].value <= threshold=0.42332448065280914
node_233: feature_name=cg06038180	feature_id[2529].value > threshold=0.6430551409721375
node_235: feature_name=cg14104252	feature_id[4735].value > threshold=0.5213949382305145
node_239: feature_name=cg06711298	feature_id[2707].value > threshold=0.36770085990428925
node_241: feature_name=cg11703722	feature_id[4056].value > threshold=0.5125315636396408
node_243: feature_name=cg21230793	feature_id[6514].value > threshold=0.27807849645614624
node_245: feature_name=cg06225767	feature_id[2575].value > threshold=0.18034886568784714
node_247: feature_name=cg00941576	feature_id[1053].value > threshold=0.2476368322968483

node_249: feature_name=cg10149889	feature_id[3676].value > threshold=0.633601188659668
node_257: feature_name=cg07608094	feature_id[2999].value > threshold=0.7820693254470825
node_259: feature_name=cg03315432	feature_id[1783].value > threshold=0.6790387332439423
node_261: feature_name=cg07894983	feature_id[3070].value > threshold=0.3577418476343155
node_263: feature_name=cg15646741	feature_id[5160].value > threshold=0.2826688587665558
node_273: feature_name=cg09701233	feature_id[3546].value > threshold=0.5189756155014038
node_275: feature_name=cg09230938	feature_id[738].value > threshold=0.3108274042606354
node_277: feature_name=cg05334190	feature_id[2341].value > threshold=0.2764553725719452
node_279: feature_name=cg15617847	feature_id[5148].value <= threshold=0.05914711393415928
node_280: feature_name=cg00216961	feature_id[816].value <= threshold=0.42650654911994934
node_281: feature_name=cg23946462	feature_id[7146].value <= threshold=0.9469821751117706
node_282: feature_name=cg06143290	feature_id[2554].value <= threshold=0.9263531863689423
node_283: feature_name=cg21528710	feature_id[6588].value <= threshold=0.8718287348747253
node_284: feature_name=cg14178043	feature_id[4760].value <= threshold=0.6852438449859619
node_285: feature_name=cg23906687	feature_id[7133].value > threshold=0.5761600136756897
node_287: feature_name=cg13838713	feature_id[4641].value > threshold=0.6983235478401184
node_289: feature_name=cg10239163	feature_id[3694].value <= threshold=0.49343886971473694
Class: sarcoma (SARC)	
Rules_85	passed counts:2
node_0: feature_name=cg11915444	feature_id[4116].value > threshold=0.3402601182460785
node_10: feature_name=cg12109728	feature_id[4182].value > threshold=0.7068270146846771
node_20: feature_name=cg10844382	feature_id[3856].value > threshold=0.5988894104957581

node_24: feature_name=cg00631702	feature_id[943].value <= threshold=0.37470583617687225
node_25: feature_name=cg07912144	feature_id[3075].value > threshold=0.5578519105911255
node_29: feature_name=cg24407065	feature_id[388].value > threshold=0.5364363789558411
node_33: feature_name=cg15275017	feature_id[5069].value > threshold=0.4538573920726776
node_51: feature_name=cg07281938	feature_id[351].value > threshold=0.7506992518901825
node_73: feature_name=cg17524821	feature_id[5626].value > threshold=0.4504699558019638
node_75: feature_name=cg02973735	feature_id[1677].value > threshold=0.29383400082588196
node_77: feature_name=cg23345038	feature_id[7002].value > threshold=0.6255057156085968
node_79: feature_name=cg17537493	feature_id[5630].value > threshold=0.5825372636318207
node_85: feature_name=cg12948116	feature_id[4375].value <= threshold=0.5658791363239288
node_86: feature_name=cg06038180	feature_id[2529].value > threshold=0.43877437710762024
node_88: feature_name=cg15720017	feature_id[5175].value > threshold=0.7009969055652618
node_90: feature_name=cg13070215	feature_id[314].value <= threshold=0.14645694941282272
node_91: feature_name=cg01014262	feature_id[1082].value <= threshold=0.5737917125225067
node_92: feature_name=cg16863382	feature_id[5471].value > threshold=0.7200668156147003
node_102: feature_name=cg06633739	feature_id[2686].value > threshold=0.47115950286388397
node_126: feature_name=cg22946562	feature_id[744].value > threshold=0.6194809079170227
node_170: feature_name=cg12120430	feature_id[4185].value > threshold=0.5775820016860962
node_172: feature_name=cg02137970	feature_id[1411].value > threshold=0.6705857813358307
node_196: feature_name=cg03393445	feature_id[1807].value > threshold=0.4260125905275345
node_218: feature_name=cg23525541	feature_id[7049].value > threshold=0.4485483318567276
node_222: feature_name=cg10543634	feature_id[3775].value > threshold=0.29505227506160736

node_224: feature_name=cg07344990	feature_id[2926].value > threshold=0.5750180780887604
node_226: feature_name=cg10558233	feature_id[3780].value <= threshold=0.7217690348625183
node_227: feature_name=cg08097359	feature_id[3116].value > threshold=0.3598180413246155
node_229: feature_name=cg22737001	feature_id[6843].value <= threshold=0.46281898021698
node_230: feature_name=cg24276624	feature_id[7242].value > threshold=0.6169979274272919
node_232: feature_name=cg04026354	feature_id[1982].value <= threshold=0.42332448065280914
node_233: feature_name=cg06038180	feature_id[2529].value > threshold=0.6430551409721375
node_235: feature_name=cg14104252	feature_id[4735].value > threshold=0.5213949382305145
node_239: feature_name=cg06711298	feature_id[2707].value > threshold=0.36770085990428925
node_241: feature_name=cg11703722	feature_id[4056].value > threshold=0.5125315636396408
node_243: feature_name=cg21230793	feature_id[6514].value > threshold=0.27807849645614624
node_245: feature_name=cg06225767	feature_id[2575].value > threshold=0.18034886568784714
node_247: feature_name=cg00941576	feature_id[1053].value > threshold=0.2476368322968483
node_249: feature_name=cg10149889	feature_id[3676].value > threshold=0.633601188659668
node_257: feature_name=cg07608094	feature_id[2999].value > threshold=0.7820693254470825
node_259: feature_name=cg03315432	feature_id[1783].value > threshold=0.6790387332439423
node_261: feature_name=cg07894983	feature_id[3070].value > threshold=0.3577418476343155
node_263: feature_name=cg15646741	feature_id[5160].value > threshold=0.2826688587665558
node_273: feature_name=cg09701233	feature_id[3546].value > threshold=0.5189756155014038
node_275: feature_name=cg09230938	feature_id[738].value > threshold=0.3108274042606354
node_277: feature_name=cg05334190	feature_id[2341].value > threshold=0.2764553725719452
node_279: feature_name=cg15617847	feature_id[5148].value <= threshold=0.05914711393415928

node_280: feature_name=cg00216961	feature_id[816].value <= threshold=0.42650654911994934
node_281: feature_name=cg23946462	feature_id[7146].value <= threshold=0.9469821751117706
node_282: feature_name=cg06143290	feature_id[2554].value <= threshold=0.9263531863689423
node_283: feature_name=cg21528710	feature_id[6588].value <= threshold=0.8718287348747253
node_284: feature_name=cg14178043	feature_id[4760].value <= threshold=0.6852438449859619
node_285: feature_name=cg23906687	feature_id[7133].value > threshold=0.5761600136756897
node_287: feature_name=cg13838713	feature_id[4641].value <= threshold=0.6983235478401184
Class: clear cell chondrosarcoma (CSA (CC))	
Rules_86	passed counts:2
node_0: feature_name=cg11915444	feature_id[4116].value > threshold=0.3402601182460785
node_10: feature_name=cg12109728	feature_id[4182].value > threshold=0.7068270146846771
node_20: feature_name=cg10844382	feature_id[3856].value > threshold=0.5988894104957581
node_24: feature_name=cg00631702	feature_id[943].value <= threshold=0.37470583617687225
node_25: feature_name=cg07912144	feature_id[3075].value > threshold=0.5578519105911255
node_29: feature_name=cg24407065	feature_id[388].value > threshold=0.5364363789558411
node_33: feature_name=cg15275017	feature_id[5069].value > threshold=0.4538573920726776
node_51: feature_name=cg07281938	feature_id[351].value > threshold=0.7506992518901825
node_73: feature_name=cg17524821	feature_id[5626].value > threshold=0.4504699558019638
node_75: feature_name=cg02973735	feature_id[1677].value > threshold=0.29383400082588196
node_77: feature_name=cg23345038	feature_id[7002].value > threshold=0.6255057156085968
node_79: feature_name=cg17537493	feature_id[5630].value > threshold=0.5825372636318207
node_85: feature_name=cg12948116	feature_id[4375].value <= threshold=0.5658791363239288

node_86: feature_name=cg06038180	feature_id[2529].value > threshold=0.43877437710762024
node_88: feature_name=cg15720017	feature_id[5175].value > threshold=0.7009969055652618
node_90: feature_name=cg13070215	feature_id[314].value <= threshold=0.14645694941282272
node_91: feature_name=cg01014262	feature_id[1082].value <= threshold=0.5737917125225067
node_92: feature_name=cg16863382	feature_id[5471].value > threshold=0.7200668156147003
node_102: feature_name=cg06633739	feature_id[2686].value > threshold=0.47115950286388397
node_126: feature_name=cg22946562	feature_id[744].value > threshold=0.6194809079170227
node_170: feature_name=cg12120430	feature_id[4185].value > threshold=0.5775820016860962
node_172: feature_name=cg02137970	feature_id[1411].value > threshold=0.6705857813358307
node_196: feature_name=cg03393445	feature_id[1807].value > threshold=0.4260125905275345
node_218: feature_name=cg23525541	feature_id[7049].value > threshold=0.4485483318567276
node_222: feature_name=cg10543634	feature_id[3775].value > threshold=0.29505227506160736
node_224: feature_name=cg07344990	feature_id[2926].value > threshold=0.5750180780887604
node_226: feature_name=cg10558233	feature_id[3780].value <= threshold=0.7217690348625183
node_227: feature_name=cg08097359	feature_id[3116].value > threshold=0.3598180413246155
node_229: feature_name=cg22737001	feature_id[6843].value <= threshold=0.46281898021698
node_230: feature_name=cg24276624	feature_id[7242].value > threshold=0.6169979274272919
node_232: feature_name=cg04026354	feature_id[1982].value <= threshold=0.42332448065280914
node_233: feature_name=cg06038180	feature_id[2529].value > threshold=0.6430551409721375
node_235: feature_name=cg14104252	feature_id[4735].value > threshold=0.5213949382305145
node_239: feature_name=cg06711298	feature_id[2707].value > threshold=0.36770085990428925
node_241: feature_name=cg11703722	feature_id[4056].value > threshold=0.5125315636396408

node_243: feature_name=cg21230793	feature_id[6514].value > threshold=0.27807849645614624
node_245: feature_name=cg06225767	feature_id[2575].value > threshold=0.18034886568784714
node_247: feature_name=cg00941576	feature_id[1053].value > threshold=0.2476368322968483
node_249: feature_name=cg10149889	feature_id[3676].value > threshold=0.633601188659668
node_257: feature_name=cg07608094	feature_id[2999].value > threshold=0.7820693254470825
node_259: feature_name=cg03315432	feature_id[1783].value > threshold=0.6790387332439423
node_261: feature_name=cg07894983	feature_id[3070].value > threshold=0.3577418476343155
node_263: feature_name=cg15646741	feature_id[5160].value > threshold=0.2826688587665558
node_273: feature_name=cg09701233	feature_id[3546].value > threshold=0.5189756155014038
node_275: feature_name=cg09230938	feature_id[738].value > threshold=0.3108274042606354
node_277: feature_name=cg05334190	feature_id[2341].value > threshold=0.2764553725719452
node_279: feature_name=cg15617847	feature_id[5148].value <= threshold=0.05914711393415928
node_280: feature_name=cg00216961	feature_id[816].value <= threshold=0.42650654911994934
node_281: feature_name=cg23946462	feature_id[7146].value <= threshold=0.9469821751117706
node_282: feature_name=cg06143290	feature_id[2554].value <= threshold=0.9263531863689423
node_283: feature_name=cg21528710	feature_id[6588].value <= threshold=0.8718287348747253
node_284: feature_name=cg14178043	feature_id[4760].value <= threshold=0.6852438449859619
node_285: feature_name=cg23906687	feature_id[7133].value <= threshold=0.5761600136756897
Class: mesenchymal chondrosarcoma (CSA (MES))	
Rules_87	passed counts:2
node_0: feature_name=cg11915444	feature_id[4116].value > threshold=0.3402601182460785
node_10: feature_name=cg12109728	feature_id[4182].value > threshold=0.7068270146846771

node_20: feature_name=cg10844382	feature_id[3856].value > threshold=0.5988894104957581
node_24: feature_name=cg00631702	feature_id[943].value <= threshold=0.37470583617687225
node_25: feature_name=cg07912144	feature_id[3075].value > threshold=0.5578519105911255
node_29: feature_name=cg24407065	feature_id[388].value > threshold=0.5364363789558411
node_33: feature_name=cg15275017	feature_id[5069].value > threshold=0.4538573920726776
node_51: feature_name=cg07281938	feature_id[351].value > threshold=0.7506992518901825
node_73: feature_name=cg17524821	feature_id[5626].value > threshold=0.4504699558019638
node_75: feature_name=cg02973735	feature_id[1677].value > threshold=0.29383400082588196
node_77: feature_name=cg23345038	feature_id[7002].value > threshold=0.6255057156085968
node_79: feature_name=cg17537493	feature_id[5630].value > threshold=0.5825372636318207
node_85: feature_name=cg12948116	feature_id[4375].value <= threshold=0.5658791363239288
node_86: feature_name=cg06038180	feature_id[2529].value > threshold=0.43877437710762024
node_88: feature_name=cg15720017	feature_id[5175].value > threshold=0.7009969055652618
node_90: feature_name=cg13070215	feature_id[314].value <= threshold=0.14645694941282272
node_91: feature_name=cg01014262	feature_id[1082].value <= threshold=0.5737917125225067
node_92: feature_name=cg16863382	feature_id[5471].value > threshold=0.7200668156147003
node_102: feature_name=cg06633739	feature_id[2686].value > threshold=0.47115950286388397
node_126: feature_name=cg22946562	feature_id[744].value > threshold=0.6194809079170227
node_170: feature_name=cg12120430	feature_id[4185].value > threshold=0.5775820016860962
node_172: feature_name=cg02137970	feature_id[1411].value > threshold=0.6705857813358307
node_196: feature_name=cg03393445	feature_id[1807].value <= threshold=0.4260125905275345
node_197: feature_name=cg01297744	feature_id[1157].value > threshold=0.8005762100219727

node_211: feature_name=cg14401746	feature_id[4834].value > threshold=0.3708716481924057
node_213: feature_name=cg22060153	feature_id[6700].value > threshold=0.7927507758140564
Class: Ewing sarcoma (EWING)	
Rules_88	passed counts:2
node_0: feature_name=cg11915444	feature_id[4116].value > threshold=0.3402601182460785
node_10: feature_name=cg12109728	feature_id[4182].value > threshold=0.7068270146846771
node_20: feature_name=cg10844382	feature_id[3856].value > threshold=0.5988894104957581
node_24: feature_name=cg00631702	feature_id[943].value <= threshold=0.37470583617687225
node_25: feature_name=cg07912144	feature_id[3075].value > threshold=0.5578519105911255
node_29: feature_name=cg24407065	feature_id[388].value > threshold=0.5364363789558411
node_33: feature_name=cg15275017	feature_id[5069].value > threshold=0.4538573920726776
node_51: feature_name=cg07281938	feature_id[351].value > threshold=0.7506992518901825
node_73: feature_name=cg17524821	feature_id[5626].value > threshold=0.4504699558019638
node_75: feature_name=cg02973735	feature_id[1677].value > threshold=0.29383400082588196
node_77: feature_name=cg23345038	feature_id[7002].value > threshold=0.6255057156085968
node_79: feature_name=cg17537493	feature_id[5630].value > threshold=0.5825372636318207
node_85: feature_name=cg12948116	feature_id[4375].value <= threshold=0.5658791363239288
node_86: feature_name=cg06038180	feature_id[2529].value > threshold=0.43877437710762024
node_88: feature_name=cg15720017	feature_id[5175].value > threshold=0.7009969055652618
node_90: feature_name=cg13070215	feature_id[314].value <= threshold=0.14645694941282272
node_91: feature_name=cg01014262	feature_id[1082].value <= threshold=0.5737917125225067
node_92: feature_name=cg16863382	feature_id[5471].value > threshold=0.7200668156147003

node_102: feature_name=cg06633739	feature_id[2686].value > threshold=0.47115950286388397
node_126: feature_name=cg22946562	feature_id[744].value > threshold=0.6194809079170227
node_170: feature_name=cg12120430	feature_id[4185].value > threshold=0.5775820016860962
node_172: feature_name=cg02137970	feature_id[1411].value <= threshold=0.6705857813358307
node_173: feature_name=cg16185457	feature_id[5282].value > threshold=0.5055856853723526
node_183: feature_name=cg04993279	feature_id[2243].value <= threshold=0.46021124720573425
node_184: feature_name=cg04655520	feature_id[2141].value <= threshold=0.08191334456205368
node_185: feature_name=cg11103999	feature_id[3923].value <= threshold=0.8651433885097504
Class: rhabdomyosarcoma (RMS)	
Rules_89	passed counts:2
node_0: feature_name=cg11915444	feature_id[4116].value > threshold=0.3402601182460785
node_10: feature_name=cg12109728	feature_id[4182].value > threshold=0.7068270146846771
node_20: feature_name=cg10844382	feature_id[3856].value > threshold=0.5988894104957581
node_24: feature_name=cg00631702	feature_id[943].value <= threshold=0.37470583617687225
node_25: feature_name=cg07912144	feature_id[3075].value > threshold=0.5578519105911255
node_29: feature_name=cg24407065	feature_id[388].value > threshold=0.5364363789558411
node_33: feature_name=cg15275017	feature_id[5069].value > threshold=0.4538573920726776
node_51: feature_name=cg07281938	feature_id[351].value > threshold=0.7506992518901825
node_73: feature_name=cg17524821	feature_id[5626].value > threshold=0.4504699558019638
node_75: feature_name=cg02973735	feature_id[1677].value > threshold=0.29383400082588196
node_77: feature_name=cg23345038	feature_id[7002].value > threshold=0.6255057156085968
node_79: feature_name=cg17537493	feature_id[5630].value > threshold=0.5825372636318207

node_85: feature_name=cg12948116	feature_id[4375].value <= threshold=0.5658791363239288
node_86: feature_name=cg06038180	feature_id[2529].value > threshold=0.43877437710762024
node_88: feature_name=cg15720017	feature_id[5175].value > threshold=0.7009969055652618
node_90: feature_name=cg13070215	feature_id[314].value <= threshold=0.14645694941282272
node_91: feature_name=cg01014262	feature_id[1082].value <= threshold=0.5737917125225067
node_92: feature_name=cg16863382	feature_id[5471].value > threshold=0.7200668156147003
node_102: feature_name=cg06633739	feature_id[2686].value > threshold=0.47115950286388397
node_126: feature_name=cg22946562	feature_id[744].value <= threshold=0.6194809079170227
node_127: feature_name=cg23920016	feature_id[7139].value > threshold=0.7449243068695068
node_151: feature_name=cg21617218	feature_id[6608].value > threshold=0.8795748353004456
node_153: feature_name=cg01663970	feature_id[1273].value > threshold=0.8442256152629852
node_155: feature_name=cg00686915	feature_id[965].value > threshold=0.20000632852315903
node_157: feature_name=cg11628781	feature_id[4033].value > threshold=0.9032271206378937
Class: sclerosing epithelioid fibrosarcoma(SEF)	
Rules_90	passed counts:2
node_0: feature_name=cg11915444	feature_id[4116].value > threshold=0.3402601182460785
node_10: feature_name=cg12109728	feature_id[4182].value > threshold=0.7068270146846771
node_20: feature_name=cg10844382	feature_id[3856].value > threshold=0.5988894104957581
node_24: feature_name=cg00631702	feature_id[943].value <= threshold=0.37470583617687225
node_25: feature_name=cg07912144	feature_id[3075].value > threshold=0.5578519105911255
node_29: feature_name=cg24407065	feature_id[388].value > threshold=0.5364363789558411
node_33: feature_name=cg15275017	feature_id[5069].value > threshold=0.4538573920726776

node_51: feature_name=cg07281938	feature_id[351].value > threshold=0.7506992518901825
node_73: feature_name=cg17524821	feature_id[5626].value > threshold=0.4504699558019638
node_75: feature_name=cg02973735	feature_id[1677].value > threshold=0.29383400082588196
node_77: feature_name=cg23345038	feature_id[7002].value > threshold=0.6255057156085968
node_79: feature_name=cg17537493	feature_id[5630].value > threshold=0.5825372636318207
node_85: feature_name=cg12948116	feature_id[4375].value <= threshold=0.5658791363239288
node_86: feature_name=cg06038180	feature_id[2529].value > threshold=0.43877437710762024
node_88: feature_name=cg15720017	feature_id[5175].value > threshold=0.7009969055652618
node_90: feature_name=cg13070215	feature_id[314].value <= threshold=0.14645694941282272
node_91: feature_name=cg01014262	feature_id[1082].value <= threshold=0.5737917125225067
node_92: feature_name=cg16863382	feature_id[5471].value > threshold=0.7200668156147003
node_102: feature_name=cg06633739	feature_id[2686].value > threshold=0.47115950286388397
node_126: feature_name=cg22946562	feature_id[744].value <= threshold=0.6194809079170227
node_127: feature_name=cg23920016	feature_id[7139].value <= threshold=0.7449243068695068
node_128: feature_name=cg16521032	feature_id[5379].value > threshold=0.8948074877262115
node_136: feature_name=cg22345063	feature_id[6755].value > threshold=0.38597847521305084
node_144: feature_name=cg05650238	feature_id[2427].value > threshold=0.7126822769641876
Class: epithelioid sarcoma (ES)	
Rules_91	passed counts:2
node_0: feature_name=cg11915444	feature_id[4116].value > threshold=0.3402601182460785
node_10: feature_name=cg12109728	feature_id[4182].value > threshold=0.7068270146846771
node_20: feature_name=cg10844382	feature_id[3856].value > threshold=0.5988894104957581

node_24: feature_name=cg00631702	feature_id[943].value <= threshold=0.37470583617687225
node_25: feature_name=cg07912144	feature_id[3075].value > threshold=0.5578519105911255
node_29: feature_name=cg24407065	feature_id[388].value > threshold=0.5364363789558411
node_33: feature_name=cg15275017	feature_id[5069].value > threshold=0.4538573920726776
node_51: feature_name=cg07281938	feature_id[351].value > threshold=0.7506992518901825
node_73: feature_name=cg17524821	feature_id[5626].value > threshold=0.4504699558019638
node_75: feature_name=cg02973735	feature_id[1677].value > threshold=0.29383400082588196
node_77: feature_name=cg23345038	feature_id[7002].value > threshold=0.6255057156085968
node_79: feature_name=cg17537493	feature_id[5630].value > threshold=0.5825372636318207
node_85: feature_name=cg12948116	feature_id[4375].value <= threshold=0.5658791363239288
node_86: feature_name=cg06038180	feature_id[2529].value > threshold=0.43877437710762024
node_88: feature_name=cg15720017	feature_id[5175].value > threshold=0.7009969055652618
node_90: feature_name=cg13070215	feature_id[314].value <= threshold=0.14645694941282272
node_91: feature_name=cg01014262	feature_id[1082].value <= threshold=0.5737917125225067
node_92: feature_name=cg16863382	feature_id[5471].value <= threshold=0.7200668156147003
node_93: feature_name=cg18601229	feature_id[5883].value <= threshold=0.7762220203876495
node_94: feature_name=cg04819180	feature_id[2191].value <= threshold=0.8672285974025726
node_95: feature_name=cg09370702	feature_id[3456].value > threshold=0.8020930886268616
node_97: feature_name=cg08625990	feature_id[3258].value <= threshold=0.8129918575286865
Class: embryonal rhabdomyosarcoma (RMS (EMB))	
Rules_92	passed counts:2
node_0: feature_name=cg11915444	feature_id[4116].value > threshold=0.3402601182460785

node_10: feature_name=cg12109728	feature_id[4182].value > threshold=0.7068270146846771
node_20: feature_name=cg10844382	feature_id[3856].value > threshold=0.5988894104957581
node_24: feature_name=cg00631702	feature_id[943].value <= threshold=0.37470583617687225
node_25: feature_name=cg07912144	feature_id[3075].value > threshold=0.5578519105911255
node_29: feature_name=cg24407065	feature_id[388].value > threshold=0.5364363789558411
node_33: feature_name=cg15275017	feature_id[5069].value > threshold=0.4538573920726776
node_51: feature_name=cg07281938	feature_id[351].value > threshold=0.7506992518901825
node_73: feature_name=cg17524821	feature_id[5626].value > threshold=0.4504699558019638
node_75: feature_name=cg02973735	feature_id[1677].value > threshold=0.29383400082588196
node_77: feature_name=cg23345038	feature_id[7002].value > threshold=0.6255057156085968
node_79: feature_name=cg17537493	feature_id[5630].value > threshold=0.5825372636318207
node_85: feature_name=cg12948116	feature_id[4375].value <= threshold=0.5658791363239288
node_86: feature_name=cg06038180	feature_id[2529].value > threshold=0.43877437710762024
node_88: feature_name=cg15720017	feature_id[5175].value > threshold=0.7009969055652618
node_90: feature_name=cg13070215	feature_id[314].value <= threshold=0.14645694941282272
node_91: feature_name=cg01014262	feature_id[1082].value <= threshold=0.5737917125225067
node_92: feature_name=cg16863382	feature_id[5471].value <= threshold=0.7200668156147003
node_93: feature_name=cg18601229	feature_id[5883].value <= threshold=0.7762220203876495
node_94: feature_name=cg04819180	feature_id[2191].value <= threshold=0.8672285974025726
node_95: feature_name=cg09370702	feature_id[3456].value <= threshold=0.8020930886268616
Class: undifferentiated sarcoma (USARC)	
Rules_93	passed counts:2

node_0: feature_name=cg11915444	feature_id[4116].value > threshold=0.3402601182460785
node_10: feature_name=cg12109728	feature_id[4182].value > threshold=0.7068270146846771
node_20: feature_name=cg10844382	feature_id[3856].value > threshold=0.5988894104957581
node_24: feature_name=cg00631702	feature_id[943].value <= threshold=0.37470583617687225
node_25: feature_name=cg07912144	feature_id[3075].value > threshold=0.5578519105911255
node_29: feature_name=cg24407065	feature_id[388].value > threshold=0.5364363789558411
node_33: feature_name=cg15275017	feature_id[5069].value > threshold=0.4538573920726776
node_51: feature_name=cg07281938	feature_id[351].value <= threshold=0.7506992518901825
node_52: feature_name=cg21189849	feature_id[6504].value <= threshold=0.7545044124126434
node_53: feature_name=cg11717552	feature_id[4060].value <= threshold=0.8407479226589203
node_54: feature_name=cg07043952	feature_id[2823].value > threshold=0.8715284764766693
Class: clear cell chondrosarcoma (CSA (CC))	
Rules_94	passed counts:2
node_0: feature_name=cg11915444	feature_id[4116].value > threshold=0.3402601182460785
node_10: feature_name=cg12109728	feature_id[4182].value <= threshold=0.7068270146846771
node_11: feature_name=cg23902076	feature_id[7131].value > threshold=0.8691235482692719
node_13: feature_name=cg20099458	feature_id[6250].value > threshold=0.7871655821800232
Class: synovial sarcoma (SYSA)	
Rules_95	passed counts:2
node_0: feature_name=cg11915444	feature_id[4116].value <= threshold=0.3402601182460785
node_1: feature_name=cg00982952	feature_id[1071].value > threshold=0.7386334538459778
node_3: feature_name=cg22315164	feature_id[6749].value <= threshold=0.35902709513902664
Class: alveolar rhabdomyosarcoma (RMS (ALV))	

Rules_96	passed counts:1
node_0: feature_name=cg11915444	feature_id[4116].value > threshold=0.3402601182460785
node_10: feature_name=cg12109728	feature_id[4182].value > threshold=0.7068270146846771
node_20: feature_name=cg10844382	feature_id[3856].value > threshold=0.5988894104957581
node_24: feature_name=cg00631702	feature_id[943].value > threshold=0.37470583617687225
node_352: feature_name=cg07027613	feature_id[549].value > threshold=0.22842608392238617
node_360: feature_name=cg18406924	feature_id[5844].value <= threshold=0.3586410731077194
Class: epithelioid sarcoma (ES)	
Rules_97	passed counts:1
node_0: feature_name=cg11915444	feature_id[4116].value > threshold=0.3402601182460785
node_10: feature_name=cg12109728	feature_id[4182].value > threshold=0.7068270146846771
node_20: feature_name=cg10844382	feature_id[3856].value > threshold=0.5988894104957581
node_24: feature_name=cg00631702	feature_id[943].value > threshold=0.37470583617687225
node_352: feature_name=cg07027613	feature_id[549].value <= threshold=0.22842608392238617
node_353: feature_name=cg04356968	feature_id[2068].value <= threshold=0.728985995054245
node_354: feature_name=cg18259342	feature_id[5811].value > threshold=0.1999226212501526
node_356: feature_name=cg04091816	feature_id[641].value > threshold=0.7511296272277832
Class: undifferentiated sarcoma (USARC)	
Rules_98	passed counts:1
node_0: feature_name=cg11915444	feature_id[4116].value > threshold=0.3402601182460785
node_10: feature_name=cg12109728	feature_id[4182].value > threshold=0.7068270146846771
node_20: feature_name=cg10844382	feature_id[3856].value > threshold=0.5988894104957581

node_24: feature_name=cg00631702	feature_id[943].value > threshold=0.37470583617687225
node_352: feature_name=cg07027613	feature_id[549].value <= threshold=0.22842608392238617
node_353: feature_name=cg04356968	feature_id[2068].value <= threshold=0.728985995054245
node_354: feature_name=cg18259342	feature_id[5811].value > threshold=0.1999226212501526
node_356: feature_name=cg04091816	feature_id[641].value <= threshold=0.7511296272277832
Class: chondrosarcoma (CSA)	
Rules_99	passed counts:1
node_0: feature_name=cg11915444	feature_id[4116].value > threshold=0.3402601182460785
node_10: feature_name=cg12109728	feature_id[4182].value > threshold=0.7068270146846771
node_20: feature_name=cg10844382	feature_id[3856].value > threshold=0.5988894104957581
node_24: feature_name=cg00631702	feature_id[943].value <= threshold=0.37470583617687225
node_25: feature_name=cg07912144	feature_id[3075].value > threshold=0.5578519105911255
node_29: feature_name=cg24407065	feature_id[388].value > threshold=0.5364363789558411
node_33: feature_name=cg15275017	feature_id[5069].value > threshold=0.4538573920726776
node_51: feature_name=cg07281938	feature_id[351].value > threshold=0.7506992518901825
node_73: feature_name=cg17524821	feature_id[5626].value > threshold=0.4504699558019638
node_75: feature_name=cg02973735	feature_id[1677].value > threshold=0.29383400082588196
node_77: feature_name=cg23345038	feature_id[7002].value > threshold=0.6255057156085968
node_79: feature_name=cg17537493	feature_id[5630].value > threshold=0.5825372636318207
node_85: feature_name=cg12948116	feature_id[4375].value > threshold=0.5658791363239288
node_347: feature_name=cg15262242	feature_id[5064].value > threshold=0.36207813024520874
node_349: feature_name=cg13658093	feature_id[4574].value > threshold=0.6347142904996872

Class: malignant peripheral nerve sheath tumour (MPNST)	
Rules_100	passed counts:1
node_0: feature_name=cg11915444	feature_id[4116].value > threshold=0.3402601182460785
node_10: feature_name=cg12109728	feature_id[4182].value > threshold=0.7068270146846771
node_20: feature_name=cg10844382	feature_id[3856].value > threshold=0.5988894104957581
node_24: feature_name=cg00631702	feature_id[943].value <= threshold=0.37470583617687225
node_25: feature_name=cg07912144	feature_id[3075].value > threshold=0.5578519105911255
node_29: feature_name=cg24407065	feature_id[388].value > threshold=0.5364363789558411
node_33: feature_name=cg15275017	feature_id[5069].value > threshold=0.4538573920726776
node_51: feature_name=cg07281938	feature_id[351].value > threshold=0.7506992518901825
node_73: feature_name=cg17524821	feature_id[5626].value > threshold=0.4504699558019638
node_75: feature_name=cg02973735	feature_id[1677].value > threshold=0.29383400082588196
node_77: feature_name=cg23345038	feature_id[7002].value > threshold=0.6255057156085968
node_79: feature_name=cg17537493	feature_id[5630].value > threshold=0.5825372636318207
node_85: feature_name=cg12948116	feature_id[4375].value > threshold=0.5658791363239288
node_347: feature_name=cg15262242	feature_id[5064].value > threshold=0.36207813024520874
node_349: feature_name=cg13658093	feature_id[4574].value <= threshold=0.6347142904996872
Class: malignant rhabdoid tumour (MRT)	
Rules_101	passed counts:1
node_0: feature_name=cg11915444	feature_id[4116].value > threshold=0.3402601182460785
node_10: feature_name=cg12109728	feature_id[4182].value > threshold=0.7068270146846771
node_20: feature_name=cg10844382	feature_id[3856].value > threshold=0.5988894104957581

node_24: feature_name=cg00631702	feature_id[943].value <= threshold=0.37470583617687225
node_25: feature_name=cg07912144	feature_id[3075].value > threshold=0.5578519105911255
node_29: feature_name=cg24407065	feature_id[388].value > threshold=0.5364363789558411
node_33: feature_name=cg15275017	feature_id[5069].value > threshold=0.4538573920726776
node_51: feature_name=cg07281938	feature_id[351].value > threshold=0.7506992518901825
node_73: feature_name=cg17524821	feature_id[5626].value > threshold=0.4504699558019638
node_75: feature_name=cg02973735	feature_id[1677].value > threshold=0.29383400082588196
node_77: feature_name=cg23345038	feature_id[7002].value > threshold=0.6255057156085968
node_79: feature_name=cg17537493	feature_id[5630].value > threshold=0.5825372636318207
node_85: feature_name=cg12948116	feature_id[4375].value <= threshold=0.5658791363239288
node_86: feature_name=cg06038180	feature_id[2529].value > threshold=0.43877437710762024
node_88: feature_name=cg15720017	feature_id[5175].value > threshold=0.7009969055652618
node_90: feature_name=cg13070215	feature_id[314].value > threshold=0.14645694941282272
node_340: feature_name=cg06272543	feature_id[2590].value <= threshold=0.07370422407984734
node_341: feature_name=cg24268698	feature_id[7238].value <= threshold=0.9151104986667633
node_342: feature_name=cg12669088	feature_id[398].value <= threshold=0.5310345143079758
Class: epithelioid sarcoma (ES)	
Rules_102	passed counts:1
node_0: feature_name=cg11915444	feature_id[4116].value > threshold=0.3402601182460785
node_10: feature_name=cg12109728	feature_id[4182].value > threshold=0.7068270146846771
node_20: feature_name=cg10844382	feature_id[3856].value > threshold=0.5988894104957581
node_24: feature_name=cg00631702	feature_id[943].value <= threshold=0.37470583617687225

node_25: feature_name=cg07912144	feature_id[3075].value > threshold=0.5578519105911255
node_29: feature_name=cg24407065	feature_id[388].value > threshold=0.5364363789558411
node_33: feature_name=cg15275017	feature_id[5069].value > threshold=0.4538573920726776
node_51: feature_name=cg07281938	feature_id[351].value > threshold=0.7506992518901825
node_73: feature_name=cg17524821	feature_id[5626].value > threshold=0.4504699558019638
node_75: feature_name=cg02973735	feature_id[1677].value > threshold=0.29383400082588196
node_77: feature_name=cg23345038	feature_id[7002].value > threshold=0.6255057156085968
node_79: feature_name=cg17537493	feature_id[5630].value > threshold=0.5825372636318207
node_85: feature_name=cg12948116	feature_id[4375].value <= threshold=0.5658791363239288
node_86: feature_name=cg06038180	feature_id[2529].value > threshold=0.43877437710762024
node_88: feature_name=cg15720017	feature_id[5175].value > threshold=0.7009969055652618
node_90: feature_name=cg13070215	feature_id[314].value <= threshold=0.14645694941282272
node_91: feature_name=cg01014262	feature_id[1082].value <= threshold=0.5737917125225067
node_92: feature_name=cg16863382	feature_id[5471].value > threshold=0.7200668156147003
node_102: feature_name=cg06633739	feature_id[2686].value > threshold=0.47115950286388397
node_126: feature_name=cg22946562	feature_id[744].value > threshold=0.6194809079170227
node_170: feature_name=cg12120430	feature_id[4185].value > threshold=0.5775820016860962
node_172: feature_name=cg02137970	feature_id[1411].value > threshold=0.6705857813358307
node_196: feature_name=cg03393445	feature_id[1807].value > threshold=0.4260125905275345
node_218: feature_name=cg23525541	feature_id[7049].value > threshold=0.4485483318567276
node_222: feature_name=cg10543634	feature_id[3775].value > threshold=0.29505227506160736
node_224: feature_name=cg07344990	feature_id[2926].value > threshold=0.5750180780887604

node_226: feature_name=cg10558233	feature_id[3780].value <= threshold=0.7217690348625183
node_227: feature_name=cg08097359	feature_id[3116].value > threshold=0.3598180413246155
node_229: feature_name=cg22737001	feature_id[6843].value > threshold=0.46281898021698
node_333: feature_name=cg17775332	feature_id[5680].value > threshold=0.3890498280525207 5
Class: high-grade conventional osteosarcoma (OS (HG))	
Rules_103	passed counts:1
node_0: feature_name=cg11915444	feature_id[4116].value > threshold=0.3402601182460785
node_10: feature_name=cg12109728	feature_id[4182].value > threshold=0.7068270146846771
node_20: feature_name=cg10844382	feature_id[3856].value > threshold=0.5988894104957581
node_24: feature_name=cg00631702	feature_id[943].value <= threshold=0.37470583617687225
node_25: feature_name=cg07912144	feature_id[3075].value > threshold=0.5578519105911255
node_29: feature_name=cg24407065	feature_id[388].value > threshold=0.5364363789558411
node_33: feature_name=cg15275017	feature_id[5069].value > threshold=0.4538573920726776
node_51: feature_name=cg07281938	feature_id[351].value > threshold=0.7506992518901825
node_73: feature_name=cg17524821	feature_id[5626].value > threshold=0.4504699558019638
node_75: feature_name=cg02973735	feature_id[1677].value > threshold=0.29383400082588196
node_77: feature_name=cg23345038	feature_id[7002].value > threshold=0.6255057156085968
node_79: feature_name=cg17537493	feature_id[5630].value > threshold=0.5825372636318207
node_85: feature_name=cg12948116	feature_id[4375].value <= threshold=0.5658791363239288
node_86: feature_name=cg06038180	feature_id[2529].value > threshold=0.43877437710762024
node_88: feature_name=cg15720017	feature_id[5175].value > threshold=0.7009969055652618
node_90: feature_name=cg13070215	feature_id[314].value <= threshold=0.14645694941282272

node_91: feature_name=cg01014262	feature_id[1082].value <= threshold=0.5737917125225067
node_92: feature_name=cg16863382	feature_id[5471].value > threshold=0.7200668156147003
node_102: feature_name=cg06633739	feature_id[2686].value > threshold=0.47115950286388397
node_126: feature_name=cg22946562	feature_id[744].value > threshold=0.6194809079170227
node_170: feature_name=cg12120430	feature_id[4185].value > threshold=0.5775820016860962
node_172: feature_name=cg02137970	feature_id[1411].value > threshold=0.6705857813358307
node_196: feature_name=cg03393445	feature_id[1807].value > threshold=0.4260125905275345
node_218: feature_name=cg23525541	feature_id[7049].value > threshold=0.4485483318567276
node_222: feature_name=cg10543634	feature_id[3775].value > threshold=0.29505227506160736
node_224: feature_name=cg07344990	feature_id[2926].value > threshold=0.5750180780887604
node_226: feature_name=cg10558233	feature_id[3780].value <= threshold=0.7217690348625183
node_227: feature_name=cg08097359	feature_id[3116].value > threshold=0.3598180413246155
node_229: feature_name=cg22737001	feature_id[6843].value <= threshold=0.46281898021698
node_230: feature_name=cg24276624	feature_id[7242].value > threshold=0.6169979274272919
node_232: feature_name=cg04026354	feature_id[1982].value > threshold=0.42332448065280914
node_316: feature_name=cg23693485	feature_id[7083].value > threshold=0.7056963741779327
node_320: feature_name=cg16027775	feature_id[5238].value > threshold=0.17370137572288513
node_322: feature_name=cg16820411	feature_id[5464].value > threshold=0.9055586755275726
node_324: feature_name=cg07990658	feature_id[3094].value > threshold=0.9101644456386566
node_326: feature_name=cg12389888	feature_id[4236].value > threshold=0.6996647417545319
node_328: feature_name=cg06397381	feature_id[2618].value > threshold=0.6873181164264679
node_330: feature_name=cg21352006	feature_id[6547].value > threshold=0.09681446850299835

Class: well differentiated liposarcoma (WDLS)/dedifferentiated liposarcoma (DDLS)	
Rules_104	passed counts:1
node_0: feature_name=cg11915444	feature_id[4116].value > threshold=0.3402601182460785
node_10: feature_name=cg12109728	feature_id[4182].value > threshold=0.7068270146846771
node_20: feature_name=cg10844382	feature_id[3856].value > threshold=0.5988894104957581
node_24: feature_name=cg00631702	feature_id[943].value <= threshold=0.37470583617687225
node_25: feature_name=cg07912144	feature_id[3075].value > threshold=0.5578519105911255
node_29: feature_name=cg24407065	feature_id[388].value > threshold=0.5364363789558411
node_33: feature_name=cg15275017	feature_id[5069].value > threshold=0.4538573920726776
node_51: feature_name=cg07281938	feature_id[351].value > threshold=0.7506992518901825
node_73: feature_name=cg17524821	feature_id[5626].value > threshold=0.4504699558019638
node_75: feature_name=cg02973735	feature_id[1677].value > threshold=0.29383400082588196
node_77: feature_name=cg23345038	feature_id[7002].value > threshold=0.6255057156085968
node_79: feature_name=cg17537493	feature_id[5630].value > threshold=0.5825372636318207
node_85: feature_name=cg12948116	feature_id[4375].value <= threshold=0.5658791363239288
node_86: feature_name=cg06038180	feature_id[2529].value > threshold=0.43877437710762024
node_88: feature_name=cg15720017	feature_id[5175].value > threshold=0.7009969055652618
node_90: feature_name=cg13070215	feature_id[314].value <= threshold=0.14645694941282272
node_91: feature_name=cg01014262	feature_id[1082].value <= threshold=0.5737917125225067
node_92: feature_name=cg16863382	feature_id[5471].value > threshold=0.7200668156147003
node_102: feature_name=cg06633739	feature_id[2686].value > threshold=0.47115950286388397
node_126: feature_name=cg22946562	feature_id[744].value > threshold=0.6194809079170227

node_170: feature_name=cg12120430	feature_id[4185].value > threshold=0.5775820016860962
node_172: feature_name=cg02137970	feature_id[1411].value > threshold=0.6705857813358307
node_196: feature_name=cg03393445	feature_id[1807].value > threshold=0.4260125905275345
node_218: feature_name=cg23525541	feature_id[7049].value > threshold=0.4485483318567276
node_222: feature_name=cg10543634	feature_id[3775].value > threshold=0.29505227506160736
node_224: feature_name=cg07344990	feature_id[2926].value > threshold=0.5750180780887604
node_226: feature_name=cg10558233	feature_id[3780].value <= threshold=0.7217690348625183
node_227: feature_name=cg08097359	feature_id[3116].value > threshold=0.3598180413246155
node_229: feature_name=cg22737001	feature_id[6843].value <= threshold=0.46281898021698
node_230: feature_name=cg24276624	feature_id[7242].value > threshold=0.6169979274272919
node_232: feature_name=cg04026354	feature_id[1982].value > threshold=0.42332448065280914
node_316: feature_name=cg23693485	feature_id[7083].value > threshold=0.7056963741779327
node_320: feature_name=cg16027775	feature_id[5238].value > threshold=0.17370137572288513
node_322: feature_name=cg16820411	feature_id[5464].value > threshold=0.9055586755275726
node_324: feature_name=cg07990658	feature_id[3094].value > threshold=0.9101644456386566
node_326: feature_name=cg12389888	feature_id[4236].value > threshold=0.6996647417545319
node_328: feature_name=cg06397381	feature_id[2618].value > threshold=0.6873181164264679
node_330: feature_name=cg21352006	feature_id[6547].value <= threshold=0.09681446850299835
Class: malignant rhabdoid tumour (MRT)	
Rules_105	passed counts:1
node_0: feature_name=cg11915444	feature_id[4116].value > threshold=0.3402601182460785
node_10: feature_name=cg12109728	feature_id[4182].value > threshold=0.7068270146846771

node_20: feature_name=cg10844382	feature_id[3856].value > threshold=0.5988894104957581
node_24: feature_name=cg00631702	feature_id[943].value <= threshold=0.37470583617687225
node_25: feature_name=cg07912144	feature_id[3075].value > threshold=0.5578519105911255
node_29: feature_name=cg24407065	feature_id[388].value > threshold=0.5364363789558411
node_33: feature_name=cg15275017	feature_id[5069].value > threshold=0.4538573920726776
node_51: feature_name=cg07281938	feature_id[351].value > threshold=0.7506992518901825
node_73: feature_name=cg17524821	feature_id[5626].value > threshold=0.4504699558019638
node_75: feature_name=cg02973735	feature_id[1677].value > threshold=0.29383400082588196
node_77: feature_name=cg23345038	feature_id[7002].value > threshold=0.6255057156085968
node_79: feature_name=cg17537493	feature_id[5630].value > threshold=0.5825372636318207
node_85: feature_name=cg12948116	feature_id[4375].value <= threshold=0.5658791363239288
node_86: feature_name=cg06038180	feature_id[2529].value > threshold=0.43877437710762024
node_88: feature_name=cg15720017	feature_id[5175].value > threshold=0.7009969055652618
node_90: feature_name=cg13070215	feature_id[314].value <= threshold=0.14645694941282272
node_91: feature_name=cg01014262	feature_id[1082].value <= threshold=0.5737917125225067
node_92: feature_name=cg16863382	feature_id[5471].value > threshold=0.7200668156147003
node_102: feature_name=cg06633739	feature_id[2686].value > threshold=0.47115950286388397
node_126: feature_name=cg22946562	feature_id[744].value > threshold=0.6194809079170227
node_170: feature_name=cg12120430	feature_id[4185].value > threshold=0.5775820016860962
node_172: feature_name=cg02137970	feature_id[1411].value > threshold=0.6705857813358307
node_196: feature_name=cg03393445	feature_id[1807].value > threshold=0.4260125905275345
node_218: feature_name=cg23525541	feature_id[7049].value > threshold=0.4485483318567276

node_222: feature_name=cg10543634	feature_id[3775].value > threshold=0.29505227506160736
node_224: feature_name=cg07344990	feature_id[2926].value > threshold=0.5750180780887604
node_226: feature_name=cg10558233	feature_id[3780].value <= threshold=0.7217690348625183
node_227: feature_name=cg08097359	feature_id[3116].value > threshold=0.3598180413246155
node_229: feature_name=cg22737001	feature_id[6843].value <= threshold=0.46281898021698
node_230: feature_name=cg24276624	feature_id[7242].value > threshold=0.6169979274272919
node_232: feature_name=cg04026354	feature_id[1982].value > threshold=0.42332448065280914
node_316: feature_name=cg23693485	feature_id[7083].value > threshold=0.7056963741779327
node_320: feature_name=cg16027775	feature_id[5238].value > threshold=0.17370137572288513
node_322: feature_name=cg16820411	feature_id[5464].value > threshold=0.9055586755275726
node_324: feature_name=cg07990658	feature_id[3094].value > threshold=0.9101644456386566
node_326: feature_name=cg12389888	feature_id[4236].value > threshold=0.6996647417545319
node_328: feature_name=cg06397381	feature_id[2618].value <= threshold=0.6873181164264679
Class: alveolar soft part sarcoma (ASPS)	
Rules_106	passed counts:1
node_0: feature_name=cg11915444	feature_id[4116].value > threshold=0.3402601182460785
node_10: feature_name=cg12109728	feature_id[4182].value > threshold=0.7068270146846771
node_20: feature_name=cg10844382	feature_id[3856].value > threshold=0.5988894104957581
node_24: feature_name=cg00631702	feature_id[943].value <= threshold=0.37470583617687225
node_25: feature_name=cg07912144	feature_id[3075].value > threshold=0.5578519105911255
node_29: feature_name=cg24407065	feature_id[388].value > threshold=0.5364363789558411
node_33: feature_name=cg15275017	feature_id[5069].value > threshold=0.4538573920726776

node_51: feature_name=cg07281938	feature_id[351].value > threshold=0.7506992518901825
node_73: feature_name=cg17524821	feature_id[5626].value > threshold=0.4504699558019638
node_75: feature_name=cg02973735	feature_id[1677].value > threshold=0.29383400082588196
node_77: feature_name=cg23345038	feature_id[7002].value > threshold=0.6255057156085968
node_79: feature_name=cg17537493	feature_id[5630].value > threshold=0.5825372636318207
node_85: feature_name=cg12948116	feature_id[4375].value <= threshold=0.5658791363239288
node_86: feature_name=cg06038180	feature_id[2529].value > threshold=0.43877437710762024
node_88: feature_name=cg15720017	feature_id[5175].value > threshold=0.7009969055652618
node_90: feature_name=cg13070215	feature_id[314].value <= threshold=0.14645694941282272
node_91: feature_name=cg01014262	feature_id[1082].value <= threshold=0.5737917125225067
node_92: feature_name=cg16863382	feature_id[5471].value > threshold=0.7200668156147003
node_102: feature_name=cg06633739	feature_id[2686].value > threshold=0.47115950286388397
node_126: feature_name=cg22946562	feature_id[744].value > threshold=0.6194809079170227
node_170: feature_name=cg12120430	feature_id[4185].value > threshold=0.5775820016860962
node_172: feature_name=cg02137970	feature_id[1411].value > threshold=0.6705857813358307
node_196: feature_name=cg03393445	feature_id[1807].value > threshold=0.4260125905275345
node_218: feature_name=cg23525541	feature_id[7049].value > threshold=0.4485483318567276
node_222: feature_name=cg10543634	feature_id[3775].value > threshold=0.29505227506160736
node_224: feature_name=cg07344990	feature_id[2926].value > threshold=0.5750180780887604
node_226: feature_name=cg10558233	feature_id[3780].value <= threshold=0.7217690348625183
node_227: feature_name=cg08097359	feature_id[3116].value > threshold=0.3598180413246155
node_229: feature_name=cg22737001	feature_id[6843].value <= threshold=0.46281898021698

node_230: feature_name=cg24276624	feature_id[7242].value > threshold=0.6169979274272919
node_232: feature_name=cg04026354	feature_id[1982].value > threshold=0.42332448065280914
node_316: feature_name=cg23693485	feature_id[7083].value > threshold=0.7056963741779327
node_320: feature_name=cg16027775	feature_id[5238].value > threshold=0.17370137572288513
node_322: feature_name=cg16820411	feature_id[5464].value > threshold=0.9055586755275726
node_324: feature_name=cg07990658	feature_id[3094].value > threshold=0.9101644456386566
node_326: feature_name=cg12389888	feature_id[4236].value <= threshold=0.6996647417545319
Class: angiosarcoma (AS)	
Rules_107	passed counts:1
node_0: feature_name=cg11915444	feature_id[4116].value > threshold=0.3402601182460785
node_10: feature_name=cg12109728	feature_id[4182].value > threshold=0.7068270146846771
node_20: feature_name=cg10844382	feature_id[3856].value > threshold=0.5988894104957581
node_24: feature_name=cg00631702	feature_id[943].value <= threshold=0.37470583617687225
node_25: feature_name=cg07912144	feature_id[3075].value > threshold=0.5578519105911255
node_29: feature_name=cg24407065	feature_id[388].value > threshold=0.5364363789558411
node_33: feature_name=cg15275017	feature_id[5069].value > threshold=0.4538573920726776
node_51: feature_name=cg07281938	feature_id[351].value > threshold=0.7506992518901825
node_73: feature_name=cg17524821	feature_id[5626].value > threshold=0.4504699558019638
node_75: feature_name=cg02973735	feature_id[1677].value > threshold=0.29383400082588196
node_77: feature_name=cg23345038	feature_id[7002].value > threshold=0.6255057156085968
node_79: feature_name=cg17537493	feature_id[5630].value > threshold=0.5825372636318207
node_85: feature_name=cg12948116	feature_id[4375].value <= threshold=0.5658791363239288

node_86: feature_name=cg06038180	feature_id[2529].value > threshold=0.43877437710762024
node_88: feature_name=cg15720017	feature_id[5175].value > threshold=0.7009969055652618
node_90: feature_name=cg13070215	feature_id[314].value <= threshold=0.14645694941282272
node_91: feature_name=cg01014262	feature_id[1082].value <= threshold=0.5737917125225067
node_92: feature_name=cg16863382	feature_id[5471].value > threshold=0.7200668156147003
node_102: feature_name=cg06633739	feature_id[2686].value > threshold=0.47115950286388397
node_126: feature_name=cg22946562	feature_id[744].value > threshold=0.6194809079170227
node_170: feature_name=cg12120430	feature_id[4185].value > threshold=0.5775820016860962
node_172: feature_name=cg02137970	feature_id[1411].value > threshold=0.6705857813358307
node_196: feature_name=cg03393445	feature_id[1807].value > threshold=0.4260125905275345
node_218: feature_name=cg23525541	feature_id[7049].value > threshold=0.4485483318567276
node_222: feature_name=cg10543634	feature_id[3775].value > threshold=0.29505227506160736
node_224: feature_name=cg07344990	feature_id[2926].value > threshold=0.5750180780887604
node_226: feature_name=cg10558233	feature_id[3780].value <= threshold=0.7217690348625183
node_227: feature_name=cg08097359	feature_id[3116].value > threshold=0.3598180413246155
node_229: feature_name=cg22737001	feature_id[6843].value <= threshold=0.46281898021698
node_230: feature_name=cg24276624	feature_id[7242].value > threshold=0.6169979274272919
node_232: feature_name=cg04026354	feature_id[1982].value > threshold=0.42332448065280914
node_316: feature_name=cg23693485	feature_id[7083].value <= threshold=0.7056963741779327
node_317: feature_name=cg14583686	feature_id[4895].value <= threshold=0.70423823595047
Class: sarcoma (SARC)	
Rules_108	passed counts:1

node_0: feature_name=cg11915444	feature_id[4116].value > threshold=0.3402601182460785
node_10: feature_name=cg12109728	feature_id[4182].value > threshold=0.7068270146846771
node_20: feature_name=cg10844382	feature_id[3856].value > threshold=0.5988894104957581
node_24: feature_name=cg00631702	feature_id[943].value <= threshold=0.37470583617687225
node_25: feature_name=cg07912144	feature_id[3075].value > threshold=0.5578519105911255
node_29: feature_name=cg24407065	feature_id[388].value > threshold=0.5364363789558411
node_33: feature_name=cg15275017	feature_id[5069].value > threshold=0.4538573920726776
node_51: feature_name=cg07281938	feature_id[351].value > threshold=0.7506992518901825
node_73: feature_name=cg17524821	feature_id[5626].value > threshold=0.4504699558019638
node_75: feature_name=cg02973735	feature_id[1677].value > threshold=0.29383400082588196
node_77: feature_name=cg23345038	feature_id[7002].value > threshold=0.6255057156085968
node_79: feature_name=cg17537493	feature_id[5630].value > threshold=0.5825372636318207
node_85: feature_name=cg12948116	feature_id[4375].value <= threshold=0.5658791363239288
node_86: feature_name=cg06038180	feature_id[2529].value > threshold=0.43877437710762024
node_88: feature_name=cg15720017	feature_id[5175].value > threshold=0.7009969055652618
node_90: feature_name=cg13070215	feature_id[314].value <= threshold=0.14645694941282272
node_91: feature_name=cg01014262	feature_id[1082].value <= threshold=0.5737917125225067
node_92: feature_name=cg16863382	feature_id[5471].value > threshold=0.7200668156147003
node_102: feature_name=cg06633739	feature_id[2686].value > threshold=0.47115950286388397
node_126: feature_name=cg22946562	feature_id[744].value > threshold=0.6194809079170227
node_170: feature_name=cg12120430	feature_id[4185].value > threshold=0.5775820016860962
node_172: feature_name=cg02137970	feature_id[1411].value > threshold=0.6705857813358307

node_196: feature_name=cg03393445	feature_id[1807].value > threshold=0.4260125905275345
node_218: feature_name=cg23525541	feature_id[7049].value > threshold=0.4485483318567276
node_222: feature_name=cg10543634	feature_id[3775].value > threshold=0.29505227506160736
node_224: feature_name=cg07344990	feature_id[2926].value > threshold=0.5750180780887604
node_226: feature_name=cg10558233	feature_id[3780].value <= threshold=0.7217690348625183
node_227: feature_name=cg08097359	feature_id[3116].value > threshold=0.3598180413246155
node_229: feature_name=cg22737001	feature_id[6843].value <= threshold=0.46281898021698
node_230: feature_name=cg24276624	feature_id[7242].value > threshold=0.6169979274272919
node_232: feature_name=cg04026354	feature_id[1982].value <= threshold=0.42332448065280914
node_233: feature_name=cg06038180	feature_id[2529].value > threshold=0.6430551409721375
node_235: feature_name=cg14104252	feature_id[4735].value > threshold=0.5213949382305145
node_239: feature_name=cg06711298	feature_id[2707].value > threshold=0.36770085990428925
node_241: feature_name=cg11703722	feature_id[4056].value > threshold=0.5125315636396408
node_243: feature_name=cg21230793	feature_id[6514].value > threshold=0.27807849645614624
node_245: feature_name=cg06225767	feature_id[2575].value > threshold=0.18034886568784714
node_247: feature_name=cg00941576	feature_id[1053].value > threshold=0.2476368322968483
node_249: feature_name=cg10149889	feature_id[3676].value > threshold=0.633601188659668
node_257: feature_name=cg07608094	feature_id[2999].value > threshold=0.7820693254470825
node_259: feature_name=cg03315432	feature_id[1783].value > threshold=0.6790387332439423
node_261: feature_name=cg07894983	feature_id[3070].value > threshold=0.3577418476343155
node_263: feature_name=cg15646741	feature_id[5160].value > threshold=0.2826688587665558
node_273: feature_name=cg09701233	feature_id[3546].value > threshold=0.5189756155014038

node_275: feature_name=cg09230938	feature_id[738].value > threshold=0.3108274042606354
node_277: feature_name=cg05334190	feature_id[2341].value > threshold=0.2764553725719452
node_279: feature_name=cg15617847	feature_id[5148].value <= threshold=0.05914711393415928
node_280: feature_name=cg00216961	feature_id[816].value <= threshold=0.42650654911994934
node_281: feature_name=cg23946462	feature_id[7146].value <= threshold=0.9469821751117706
node_282: feature_name=cg06143290	feature_id[2554].value <= threshold=0.9263531863689423
node_283: feature_name=cg21528710	feature_id[6588].value <= threshold=0.8718287348747253
node_284: feature_name=cg14178043	feature_id[4760].value <= threshold=0.6852438449859619
node_285: feature_name=cg23906687	feature_id[7133].value > threshold=0.5761600136756897
node_287: feature_name=cg13838713	feature_id[4641].value > threshold=0.6983235478401184
node_289: feature_name=cg10239163	feature_id[3694].value > threshold=0.49343886971473694
node_291: feature_name=cg01197763	feature_id[1134].value > threshold=0.5575756281614304
node_293: feature_name=cg13210470	feature_id[4437].value > threshold=0.08023958280682564
node_295: feature_name=cg03731348	feature_id[1909].value > threshold=0.8963189721107483
node_297: feature_name=cg26929355	feature_id[633].value > threshold=0.7743391394615173
node_299: feature_name=cg02196592	feature_id[1435].value > threshold=0.4457027018070221
node_301: feature_name=cg07849237	feature_id[3060].value > threshold=0.8495026230812073
node_303: feature_name=cg03424213	feature_id[1816].value > threshold=0.6150504946708679
node_305: feature_name=cg06862949	feature_id[2759].value > threshold=0.7639872133731842
node_307: feature_name=cg19865472	feature_id[6195].value > threshold=0.9426173269748688
Class: gastrointestinal stromal tumour (GIST)	
Rules_109	passed counts:1

node_0: feature_name=cg11915444	feature_id[4116].value > threshold=0.3402601182460785
node_10: feature_name=cg12109728	feature_id[4182].value > threshold=0.7068270146846771
node_20: feature_name=cg10844382	feature_id[3856].value > threshold=0.5988894104957581
node_24: feature_name=cg00631702	feature_id[943].value <= threshold=0.37470583617687225
node_25: feature_name=cg07912144	feature_id[3075].value > threshold=0.5578519105911255
node_29: feature_name=cg24407065	feature_id[388].value > threshold=0.5364363789558411
node_33: feature_name=cg15275017	feature_id[5069].value > threshold=0.4538573920726776
node_51: feature_name=cg07281938	feature_id[351].value > threshold=0.7506992518901825
node_73: feature_name=cg17524821	feature_id[5626].value > threshold=0.4504699558019638
node_75: feature_name=cg02973735	feature_id[1677].value > threshold=0.29383400082588196
node_77: feature_name=cg23345038	feature_id[7002].value > threshold=0.6255057156085968
node_79: feature_name=cg17537493	feature_id[5630].value > threshold=0.5825372636318207
node_85: feature_name=cg12948116	feature_id[4375].value <= threshold=0.5658791363239288
node_86: feature_name=cg06038180	feature_id[2529].value > threshold=0.43877437710762024
node_88: feature_name=cg15720017	feature_id[5175].value > threshold=0.7009969055652618
node_90: feature_name=cg13070215	feature_id[314].value <= threshold=0.14645694941282272
node_91: feature_name=cg01014262	feature_id[1082].value <= threshold=0.5737917125225067
node_92: feature_name=cg16863382	feature_id[5471].value > threshold=0.7200668156147003
node_102: feature_name=cg06633739	feature_id[2686].value > threshold=0.47115950286388397
node_126: feature_name=cg22946562	feature_id[744].value > threshold=0.6194809079170227
node_170: feature_name=cg12120430	feature_id[4185].value > threshold=0.5775820016860962
node_172: feature_name=cg02137970	feature_id[1411].value > threshold=0.6705857813358307

node_196: feature_name=cg03393445	feature_id[1807].value > threshold=0.4260125905275345
node_218: feature_name=cg23525541	feature_id[7049].value > threshold=0.4485483318567276
node_222: feature_name=cg10543634	feature_id[3775].value > threshold=0.29505227506160736
node_224: feature_name=cg07344990	feature_id[2926].value > threshold=0.5750180780887604
node_226: feature_name=cg10558233	feature_id[3780].value <= threshold=0.7217690348625183
node_227: feature_name=cg08097359	feature_id[3116].value > threshold=0.3598180413246155
node_229: feature_name=cg22737001	feature_id[6843].value <= threshold=0.46281898021698
node_230: feature_name=cg24276624	feature_id[7242].value > threshold=0.6169979274272919
node_232: feature_name=cg04026354	feature_id[1982].value <= threshold=0.42332448065280914
node_233: feature_name=cg06038180	feature_id[2529].value > threshold=0.6430551409721375
node_235: feature_name=cg14104252	feature_id[4735].value > threshold=0.5213949382305145
node_239: feature_name=cg06711298	feature_id[2707].value > threshold=0.36770085990428925
node_241: feature_name=cg11703722	feature_id[4056].value > threshold=0.5125315636396408
node_243: feature_name=cg21230793	feature_id[6514].value > threshold=0.27807849645614624
node_245: feature_name=cg06225767	feature_id[2575].value > threshold=0.18034886568784714
node_247: feature_name=cg00941576	feature_id[1053].value > threshold=0.2476368322968483
node_249: feature_name=cg10149889	feature_id[3676].value > threshold=0.633601188659668
node_257: feature_name=cg07608094	feature_id[2999].value > threshold=0.7820693254470825
node_259: feature_name=cg03315432	feature_id[1783].value > threshold=0.6790387332439423
node_261: feature_name=cg07894983	feature_id[3070].value > threshold=0.3577418476343155
node_263: feature_name=cg15646741	feature_id[5160].value > threshold=0.2826688587665558
node_273: feature_name=cg09701233	feature_id[3546].value > threshold=0.5189756155014038

node_275: feature_name=cg09230938	feature_id[738].value > threshold=0.3108274042606354
node_277: feature_name=cg05334190	feature_id[2341].value > threshold=0.2764553725719452
node_279: feature_name=cg15617847	feature_id[5148].value <= threshold=0.05914711393415928
node_280: feature_name=cg00216961	feature_id[816].value <= threshold=0.42650654911994934
node_281: feature_name=cg23946462	feature_id[7146].value <= threshold=0.9469821751117706
node_282: feature_name=cg06143290	feature_id[2554].value <= threshold=0.9263531863689423
node_283: feature_name=cg21528710	feature_id[6588].value <= threshold=0.8718287348747253
node_284: feature_name=cg14178043	feature_id[4760].value <= threshold=0.6852438449859619
node_285: feature_name=cg23906687	feature_id[7133].value > threshold=0.5761600136756897
node_287: feature_name=cg13838713	feature_id[4641].value > threshold=0.6983235478401184
node_289: feature_name=cg10239163	feature_id[3694].value > threshold=0.49343886971473694
node_291: feature_name=cg01197763	feature_id[1134].value > threshold=0.5575756281614304
node_293: feature_name=cg13210470	feature_id[4437].value > threshold=0.08023958280682564
node_295: feature_name=cg03731348	feature_id[1909].value > threshold=0.8963189721107483
node_297: feature_name=cg26929355	feature_id[633].value > threshold=0.7743391394615173
node_299: feature_name=cg02196592	feature_id[1435].value > threshold=0.4457027018070221
node_301: feature_name=cg07849237	feature_id[3060].value > threshold=0.8495026230812073
node_303: feature_name=cg03424213	feature_id[1816].value > threshold=0.6150504946708679
node_305: feature_name=cg06862949	feature_id[2759].value > threshold=0.7639872133731842
node_307: feature_name=cg19865472	feature_id[6195].value <= threshold=0.9426173269748688
Class: Kaposi sarcoma (KS)	
Rules_110	passed counts:1

node_0: feature_name=cg11915444	feature_id[4116].value > threshold=0.3402601182460785
node_10: feature_name=cg12109728	feature_id[4182].value > threshold=0.7068270146846771
node_20: feature_name=cg10844382	feature_id[3856].value > threshold=0.5988894104957581
node_24: feature_name=cg00631702	feature_id[943].value <= threshold=0.37470583617687225
node_25: feature_name=cg07912144	feature_id[3075].value > threshold=0.5578519105911255
node_29: feature_name=cg24407065	feature_id[388].value > threshold=0.5364363789558411
node_33: feature_name=cg15275017	feature_id[5069].value > threshold=0.4538573920726776
node_51: feature_name=cg07281938	feature_id[351].value > threshold=0.7506992518901825
node_73: feature_name=cg17524821	feature_id[5626].value > threshold=0.4504699558019638
node_75: feature_name=cg02973735	feature_id[1677].value > threshold=0.29383400082588196
node_77: feature_name=cg23345038	feature_id[7002].value > threshold=0.6255057156085968
node_79: feature_name=cg17537493	feature_id[5630].value > threshold=0.5825372636318207
node_85: feature_name=cg12948116	feature_id[4375].value <= threshold=0.5658791363239288
node_86: feature_name=cg06038180	feature_id[2529].value > threshold=0.43877437710762024
node_88: feature_name=cg15720017	feature_id[5175].value > threshold=0.7009969055652618
node_90: feature_name=cg13070215	feature_id[314].value <= threshold=0.14645694941282272
node_91: feature_name=cg01014262	feature_id[1082].value <= threshold=0.5737917125225067
node_92: feature_name=cg16863382	feature_id[5471].value > threshold=0.7200668156147003
node_102: feature_name=cg06633739	feature_id[2686].value > threshold=0.47115950286388397
node_126: feature_name=cg22946562	feature_id[744].value > threshold=0.6194809079170227
node_170: feature_name=cg12120430	feature_id[4185].value > threshold=0.5775820016860962
node_172: feature_name=cg02137970	feature_id[1411].value > threshold=0.6705857813358307

node_196: feature_name=cg03393445	feature_id[1807].value > threshold=0.4260125905275345
node_218: feature_name=cg23525541	feature_id[7049].value > threshold=0.4485483318567276
node_222: feature_name=cg10543634	feature_id[3775].value > threshold=0.29505227506160736
node_224: feature_name=cg07344990	feature_id[2926].value > threshold=0.5750180780887604
node_226: feature_name=cg10558233	feature_id[3780].value <= threshold=0.7217690348625183
node_227: feature_name=cg08097359	feature_id[3116].value > threshold=0.3598180413246155
node_229: feature_name=cg22737001	feature_id[6843].value <= threshold=0.46281898021698
node_230: feature_name=cg24276624	feature_id[7242].value > threshold=0.6169979274272919
node_232: feature_name=cg04026354	feature_id[1982].value <= threshold=0.42332448065280914
node_233: feature_name=cg06038180	feature_id[2529].value > threshold=0.6430551409721375
node_235: feature_name=cg14104252	feature_id[4735].value > threshold=0.5213949382305145
node_239: feature_name=cg06711298	feature_id[2707].value > threshold=0.36770085990428925
node_241: feature_name=cg11703722	feature_id[4056].value > threshold=0.5125315636396408
node_243: feature_name=cg21230793	feature_id[6514].value > threshold=0.27807849645614624
node_245: feature_name=cg06225767	feature_id[2575].value > threshold=0.18034886568784714
node_247: feature_name=cg00941576	feature_id[1053].value > threshold=0.2476368322968483
node_249: feature_name=cg10149889	feature_id[3676].value > threshold=0.633601188659668
node_257: feature_name=cg07608094	feature_id[2999].value > threshold=0.7820693254470825
node_259: feature_name=cg03315432	feature_id[1783].value > threshold=0.6790387332439423
node_261: feature_name=cg07894983	feature_id[3070].value > threshold=0.3577418476343155
node_263: feature_name=cg15646741	feature_id[5160].value > threshold=0.2826688587665558
node_273: feature_name=cg09701233	feature_id[3546].value > threshold=0.5189756155014038

node_275: feature_name=cg09230938	feature_id[738].value > threshold=0.3108274042606354
node_277: feature_name=cg05334190	feature_id[2341].value > threshold=0.2764553725719452
node_279: feature_name=cg15617847	feature_id[5148].value <= threshold=0.05914711393415928
node_280: feature_name=cg00216961	feature_id[816].value <= threshold=0.42650654911994934
node_281: feature_name=cg23946462	feature_id[7146].value <= threshold=0.9469821751117706
node_282: feature_name=cg06143290	feature_id[2554].value <= threshold=0.9263531863689423
node_283: feature_name=cg21528710	feature_id[6588].value <= threshold=0.8718287348747253
node_284: feature_name=cg14178043	feature_id[4760].value <= threshold=0.6852438449859619
node_285: feature_name=cg23906687	feature_id[7133].value > threshold=0.5761600136756897
node_287: feature_name=cg13838713	feature_id[4641].value > threshold=0.6983235478401184
node_289: feature_name=cg10239163	feature_id[3694].value > threshold=0.49343886971473694
node_291: feature_name=cg01197763	feature_id[1134].value > threshold=0.5575756281614304
node_293: feature_name=cg13210470	feature_id[4437].value > threshold=0.08023958280682564
node_295: feature_name=cg03731348	feature_id[1909].value > threshold=0.8963189721107483
node_297: feature_name=cg26929355	feature_id[633].value > threshold=0.7743391394615173
node_299: feature_name=cg02196592	feature_id[1435].value > threshold=0.4457027018070221
node_301: feature_name=cg07849237	feature_id[3060].value > threshold=0.8495026230812073
node_303: feature_name=cg03424213	feature_id[1816].value > threshold=0.6150504946708679
node_305: feature_name=cg06862949	feature_id[2759].value <= threshold=0.7639872133731842
Class: Ewing sarcoma (EWING)	
Rules_111	passed counts:1
node_0: feature_name=cg11915444	feature_id[4116].value > threshold=0.3402601182460785

node_10: feature_name=cg12109728	feature_id[4182].value > threshold=0.7068270146846771
node_20: feature_name=cg10844382	feature_id[3856].value > threshold=0.5988894104957581
node_24: feature_name=cg00631702	feature_id[943].value <= threshold=0.37470583617687225
node_25: feature_name=cg07912144	feature_id[3075].value > threshold=0.5578519105911255
node_29: feature_name=cg24407065	feature_id[388].value > threshold=0.5364363789558411
node_33: feature_name=cg15275017	feature_id[5069].value > threshold=0.4538573920726776
node_51: feature_name=cg07281938	feature_id[351].value > threshold=0.7506992518901825
node_73: feature_name=cg17524821	feature_id[5626].value > threshold=0.4504699558019638
node_75: feature_name=cg02973735	feature_id[1677].value > threshold=0.29383400082588196
node_77: feature_name=cg23345038	feature_id[7002].value > threshold=0.6255057156085968
node_79: feature_name=cg17537493	feature_id[5630].value > threshold=0.5825372636318207
node_85: feature_name=cg12948116	feature_id[4375].value <= threshold=0.5658791363239288
node_86: feature_name=cg06038180	feature_id[2529].value > threshold=0.43877437710762024
node_88: feature_name=cg15720017	feature_id[5175].value > threshold=0.7009969055652618
node_90: feature_name=cg13070215	feature_id[314].value <= threshold=0.14645694941282272
node_91: feature_name=cg01014262	feature_id[1082].value <= threshold=0.5737917125225067
node_92: feature_name=cg16863382	feature_id[5471].value > threshold=0.7200668156147003
node_102: feature_name=cg06633739	feature_id[2686].value > threshold=0.47115950286388397
node_126: feature_name=cg22946562	feature_id[744].value > threshold=0.6194809079170227
node_170: feature_name=cg12120430	feature_id[4185].value > threshold=0.5775820016860962
node_172: feature_name=cg02137970	feature_id[1411].value > threshold=0.6705857813358307
node_196: feature_name=cg03393445	feature_id[1807].value > threshold=0.4260125905275345

node_218: feature_name=cg23525541	feature_id[7049].value > threshold=0.4485483318567276
node_222: feature_name=cg10543634	feature_id[3775].value > threshold=0.29505227506160736
node_224: feature_name=cg07344990	feature_id[2926].value > threshold=0.5750180780887604
node_226: feature_name=cg10558233	feature_id[3780].value <= threshold=0.7217690348625183
node_227: feature_name=cg08097359	feature_id[3116].value > threshold=0.3598180413246155
node_229: feature_name=cg22737001	feature_id[6843].value <= threshold=0.46281898021698
node_230: feature_name=cg24276624	feature_id[7242].value > threshold=0.6169979274272919
node_232: feature_name=cg04026354	feature_id[1982].value <= threshold=0.42332448065280914
node_233: feature_name=cg06038180	feature_id[2529].value > threshold=0.6430551409721375
node_235: feature_name=cg14104252	feature_id[4735].value > threshold=0.5213949382305145
node_239: feature_name=cg06711298	feature_id[2707].value > threshold=0.36770085990428925
node_241: feature_name=cg11703722	feature_id[4056].value > threshold=0.5125315636396408
node_243: feature_name=cg21230793	feature_id[6514].value > threshold=0.27807849645614624
node_245: feature_name=cg06225767	feature_id[2575].value > threshold=0.18034886568784714
node_247: feature_name=cg00941576	feature_id[1053].value > threshold=0.2476368322968483
node_249: feature_name=cg10149889	feature_id[3676].value > threshold=0.633601188659668
node_257: feature_name=cg07608094	feature_id[2999].value > threshold=0.7820693254470825
node_259: feature_name=cg03315432	feature_id[1783].value > threshold=0.6790387332439423
node_261: feature_name=cg07894983	feature_id[3070].value > threshold=0.3577418476343155
node_263: feature_name=cg15646741	feature_id[5160].value > threshold=0.2826688587665558
node_273: feature_name=cg09701233	feature_id[3546].value > threshold=0.5189756155014038
node_275: feature_name=cg09230938	feature_id[738].value > threshold=0.3108274042606354

node_277: feature_name=cg05334190	feature_id[2341].value > threshold=0.2764553725719452
node_279: feature_name=cg15617847	feature_id[5148].value <= threshold=0.05914711393415928
node_280: feature_name=cg00216961	feature_id[816].value <= threshold=0.42650654911994934
node_281: feature_name=cg23946462	feature_id[7146].value <= threshold=0.9469821751117706
node_282: feature_name=cg06143290	feature_id[2554].value <= threshold=0.9263531863689423
node_283: feature_name=cg21528710	feature_id[6588].value <= threshold=0.8718287348747253
node_284: feature_name=cg14178043	feature_id[4760].value <= threshold=0.6852438449859619
node_285: feature_name=cg23906687	feature_id[7133].value > threshold=0.5761600136756897
node_287: feature_name=cg13838713	feature_id[4641].value > threshold=0.6983235478401184
node_289: feature_name=cg10239163	feature_id[3694].value > threshold=0.49343886971473694
node_291: feature_name=cg01197763	feature_id[1134].value > threshold=0.5575756281614304
node_293: feature_name=cg13210470	feature_id[4437].value > threshold=0.08023958280682564
node_295: feature_name=cg03731348	feature_id[1909].value > threshold=0.8963189721107483
node_297: feature_name=cg26929355	feature_id[633].value > threshold=0.7743391394615173
node_299: feature_name=cg02196592	feature_id[1435].value > threshold=0.4457027018070221
node_301: feature_name=cg07849237	feature_id[3060].value > threshold=0.8495026230812073
node_303: feature_name=cg03424213	feature_id[1816].value <= threshold=0.6150504946708679
Class: well differentiated liposarcoma (WDLS)/dedifferentiated liposarcoma (DDLS)	
Rules_112	passed counts:1
node_0: feature_name=cg11915444	feature_id[4116].value > threshold=0.3402601182460785
node_10: feature_name=cg12109728	feature_id[4182].value > threshold=0.7068270146846771
node_20: feature_name=cg10844382	feature_id[3856].value > threshold=0.5988894104957581

node_24: feature_name=cg00631702	feature_id[943].value <= threshold=0.37470583617687225
node_25: feature_name=cg07912144	feature_id[3075].value > threshold=0.5578519105911255
node_29: feature_name=cg24407065	feature_id[388].value > threshold=0.5364363789558411
node_33: feature_name=cg15275017	feature_id[5069].value > threshold=0.4538573920726776
node_51: feature_name=cg07281938	feature_id[351].value > threshold=0.7506992518901825
node_73: feature_name=cg17524821	feature_id[5626].value > threshold=0.4504699558019638
node_75: feature_name=cg02973735	feature_id[1677].value > threshold=0.29383400082588196
node_77: feature_name=cg23345038	feature_id[7002].value > threshold=0.6255057156085968
node_79: feature_name=cg17537493	feature_id[5630].value > threshold=0.5825372636318207
node_85: feature_name=cg12948116	feature_id[4375].value <= threshold=0.5658791363239288
node_86: feature_name=cg06038180	feature_id[2529].value > threshold=0.43877437710762024
node_88: feature_name=cg15720017	feature_id[5175].value > threshold=0.7009969055652618
node_90: feature_name=cg13070215	feature_id[314].value <= threshold=0.14645694941282272
node_91: feature_name=cg01014262	feature_id[1082].value <= threshold=0.5737917125225067
node_92: feature_name=cg16863382	feature_id[5471].value > threshold=0.7200668156147003
node_102: feature_name=cg06633739	feature_id[2686].value > threshold=0.47115950286388397
node_126: feature_name=cg22946562	feature_id[744].value > threshold=0.6194809079170227
node_170: feature_name=cg12120430	feature_id[4185].value > threshold=0.5775820016860962
node_172: feature_name=cg02137970	feature_id[1411].value > threshold=0.6705857813358307
node_196: feature_name=cg03393445	feature_id[1807].value > threshold=0.4260125905275345
node_218: feature_name=cg23525541	feature_id[7049].value > threshold=0.4485483318567276
node_222: feature_name=cg10543634	feature_id[3775].value > threshold=0.29505227506160736

node_224: feature_name=cg07344990	feature_id[2926].value > threshold=0.5750180780887604
node_226: feature_name=cg10558233	feature_id[3780].value <= threshold=0.7217690348625183
node_227: feature_name=cg08097359	feature_id[3116].value > threshold=0.3598180413246155
node_229: feature_name=cg22737001	feature_id[6843].value <= threshold=0.46281898021698
node_230: feature_name=cg24276624	feature_id[7242].value > threshold=0.6169979274272919
node_232: feature_name=cg04026354	feature_id[1982].value <= threshold=0.42332448065280914
node_233: feature_name=cg06038180	feature_id[2529].value > threshold=0.6430551409721375
node_235: feature_name=cg14104252	feature_id[4735].value > threshold=0.5213949382305145
node_239: feature_name=cg06711298	feature_id[2707].value > threshold=0.36770085990428925
node_241: feature_name=cg11703722	feature_id[4056].value > threshold=0.5125315636396408
node_243: feature_name=cg21230793	feature_id[6514].value > threshold=0.27807849645614624
node_245: feature_name=cg06225767	feature_id[2575].value > threshold=0.18034886568784714
node_247: feature_name=cg00941576	feature_id[1053].value > threshold=0.2476368322968483
node_249: feature_name=cg10149889	feature_id[3676].value > threshold=0.633601188659668
node_257: feature_name=cg07608094	feature_id[2999].value > threshold=0.7820693254470825
node_259: feature_name=cg03315432	feature_id[1783].value > threshold=0.6790387332439423
node_261: feature_name=cg07894983	feature_id[3070].value > threshold=0.3577418476343155
node_263: feature_name=cg15646741	feature_id[5160].value > threshold=0.2826688587665558
node_273: feature_name=cg09701233	feature_id[3546].value > threshold=0.5189756155014038
node_275: feature_name=cg09230938	feature_id[738].value > threshold=0.3108274042606354
node_277: feature_name=cg05334190	feature_id[2341].value > threshold=0.2764553725719452
node_279: feature_name=cg15617847	feature_id[5148].value <= threshold=0.05914711393415928

node_280: feature_name=cg00216961	feature_id[816].value <= threshold=0.42650654911994934
node_281: feature_name=cg23946462	feature_id[7146].value <= threshold=0.9469821751117706
node_282: feature_name=cg06143290	feature_id[2554].value <= threshold=0.9263531863689423
node_283: feature_name=cg21528710	feature_id[6588].value <= threshold=0.8718287348747253
node_284: feature_name=cg14178043	feature_id[4760].value <= threshold=0.6852438449859619
node_285: feature_name=cg23906687	feature_id[7133].value > threshold=0.5761600136756897
node_287: feature_name=cg13838713	feature_id[4641].value > threshold=0.6983235478401184
node_289: feature_name=cg10239163	feature_id[3694].value > threshold=0.49343886971473694
node_291: feature_name=cg01197763	feature_id[1134].value > threshold=0.5575756281614304
node_293: feature_name=cg13210470	feature_id[4437].value > threshold=0.08023958280682564
node_295: feature_name=cg03731348	feature_id[1909].value > threshold=0.8963189721107483
node_297: feature_name=cg26929355	feature_id[633].value > threshold=0.7743391394615173
node_299: feature_name=cg02196592	feature_id[1435].value > threshold=0.4457027018070221
node_301: feature_name=cg07849237	feature_id[3060].value <= threshold=0.8495026230812073
Class: myositis proliferans (MP)	
Rules_113	passed counts:1
node_0: feature_name=cg11915444	feature_id[4116].value > threshold=0.3402601182460785
node_10: feature_name=cg12109728	feature_id[4182].value > threshold=0.7068270146846771
node_20: feature_name=cg10844382	feature_id[3856].value > threshold=0.5988894104957581
node_24: feature_name=cg00631702	feature_id[943].value <= threshold=0.37470583617687225
node_25: feature_name=cg07912144	feature_id[3075].value > threshold=0.5578519105911255
node_29: feature_name=cg24407065	feature_id[388].value > threshold=0.5364363789558411

node_33: feature_name=cg15275017	feature_id[5069].value > threshold=0.4538573920726776
node_51: feature_name=cg07281938	feature_id[351].value > threshold=0.7506992518901825
node_73: feature_name=cg17524821	feature_id[5626].value > threshold=0.4504699558019638
node_75: feature_name=cg02973735	feature_id[1677].value > threshold=0.29383400082588196
node_77: feature_name=cg23345038	feature_id[7002].value > threshold=0.6255057156085968
node_79: feature_name=cg17537493	feature_id[5630].value > threshold=0.5825372636318207
node_85: feature_name=cg12948116	feature_id[4375].value <= threshold=0.5658791363239288
node_86: feature_name=cg06038180	feature_id[2529].value > threshold=0.43877437710762024
node_88: feature_name=cg15720017	feature_id[5175].value > threshold=0.7009969055652618
node_90: feature_name=cg13070215	feature_id[314].value <= threshold=0.14645694941282272
node_91: feature_name=cg01014262	feature_id[1082].value <= threshold=0.5737917125225067
node_92: feature_name=cg16863382	feature_id[5471].value > threshold=0.7200668156147003
node_102: feature_name=cg06633739	feature_id[2686].value > threshold=0.47115950286388397
node_126: feature_name=cg22946562	feature_id[744].value > threshold=0.6194809079170227
node_170: feature_name=cg12120430	feature_id[4185].value > threshold=0.5775820016860962
node_172: feature_name=cg02137970	feature_id[1411].value > threshold=0.6705857813358307
node_196: feature_name=cg03393445	feature_id[1807].value > threshold=0.4260125905275345
node_218: feature_name=cg23525541	feature_id[7049].value > threshold=0.4485483318567276
node_222: feature_name=cg10543634	feature_id[3775].value > threshold=0.29505227506160736
node_224: feature_name=cg07344990	feature_id[2926].value > threshold=0.5750180780887604
node_226: feature_name=cg10558233	feature_id[3780].value <= threshold=0.7217690348625183
node_227: feature_name=cg08097359	feature_id[3116].value > threshold=0.3598180413246155

node_229: feature_name=cg22737001	feature_id[6843].value <= threshold=0.46281898021698
node_230: feature_name=cg24276624	feature_id[7242].value > threshold=0.6169979274272919
node_232: feature_name=cg04026354	feature_id[1982].value <= threshold=0.42332448065280914
node_233: feature_name=cg06038180	feature_id[2529].value > threshold=0.6430551409721375
node_235: feature_name=cg14104252	feature_id[4735].value > threshold=0.5213949382305145
node_239: feature_name=cg06711298	feature_id[2707].value > threshold=0.36770085990428925
node_241: feature_name=cg11703722	feature_id[4056].value > threshold=0.5125315636396408
node_243: feature_name=cg21230793	feature_id[6514].value > threshold=0.27807849645614624
node_245: feature_name=cg06225767	feature_id[2575].value > threshold=0.18034886568784714
node_247: feature_name=cg00941576	feature_id[1053].value > threshold=0.2476368322968483
node_249: feature_name=cg10149889	feature_id[3676].value > threshold=0.633601188659668
node_257: feature_name=cg07608094	feature_id[2999].value > threshold=0.7820693254470825
node_259: feature_name=cg03315432	feature_id[1783].value > threshold=0.6790387332439423
node_261: feature_name=cg07894983	feature_id[3070].value > threshold=0.3577418476343155
node_263: feature_name=cg15646741	feature_id[5160].value > threshold=0.2826688587665558
node_273: feature_name=cg09701233	feature_id[3546].value > threshold=0.5189756155014038
node_275: feature_name=cg09230938	feature_id[738].value > threshold=0.3108274042606354
node_277: feature_name=cg05334190	feature_id[2341].value > threshold=0.2764553725719452
node_279: feature_name=cg15617847	feature_id[5148].value <= threshold=0.05914711393415928
node_280: feature_name=cg00216961	feature_id[816].value <= threshold=0.42650654911994934
node_281: feature_name=cg23946462	feature_id[7146].value <= threshold=0.9469821751117706
node_282: feature_name=cg06143290	feature_id[2554].value <= threshold=0.9263531863689423

node_283: feature_name=cg21528710	feature_id[6588].value <= threshold=0.8718287348747253
node_284: feature_name=cg14178043	feature_id[4760].value <= threshold=0.6852438449859619
node_285: feature_name=cg23906687	feature_id[7133].value > threshold=0.5761600136756897
node_287: feature_name=cg13838713	feature_id[4641].value > threshold=0.6983235478401184
node_289: feature_name=cg10239163	feature_id[3694].value > threshold=0.49343886971473694
node_291: feature_name=cg01197763	feature_id[1134].value > threshold=0.5575756281614304
node_293: feature_name=cg13210470	feature_id[4437].value > threshold=0.08023958280682564
node_295: feature_name=cg03731348	feature_id[1909].value > threshold=0.8963189721107483
node_297: feature_name=cg26929355	feature_id[633].value > threshold=0.7743391394615173
node_299: feature_name=cg02196592	feature_id[1435].value <= threshold=0.4457027018070221
Class: malignant peripheral nerve sheath tumour (MPNST)	
Rules_114	passed counts:1
node_0: feature_name=cg11915444	feature_id[4116].value > threshold=0.3402601182460785
node_10: feature_name=cg12109728	feature_id[4182].value > threshold=0.7068270146846771
node_20: feature_name=cg10844382	feature_id[3856].value > threshold=0.5988894104957581
node_24: feature_name=cg00631702	feature_id[943].value <= threshold=0.37470583617687225
node_25: feature_name=cg07912144	feature_id[3075].value > threshold=0.5578519105911255
node_29: feature_name=cg24407065	feature_id[388].value > threshold=0.5364363789558411
node_33: feature_name=cg15275017	feature_id[5069].value > threshold=0.4538573920726776
node_51: feature_name=cg07281938	feature_id[351].value > threshold=0.7506992518901825
node_73: feature_name=cg17524821	feature_id[5626].value > threshold=0.4504699558019638
node_75: feature_name=cg02973735	feature_id[1677].value > threshold=0.29383400082588196

node_77: feature_name=cg23345038	feature_id[7002].value > threshold=0.6255057156085968
node_79: feature_name=cg17537493	feature_id[5630].value > threshold=0.5825372636318207
node_85: feature_name=cg12948116	feature_id[4375].value <= threshold=0.5658791363239288
node_86: feature_name=cg06038180	feature_id[2529].value > threshold=0.43877437710762024
node_88: feature_name=cg15720017	feature_id[5175].value > threshold=0.7009969055652618
node_90: feature_name=cg13070215	feature_id[314].value <= threshold=0.14645694941282272
node_91: feature_name=cg01014262	feature_id[1082].value <= threshold=0.5737917125225067
node_92: feature_name=cg16863382	feature_id[5471].value > threshold=0.7200668156147003
node_102: feature_name=cg06633739	feature_id[2686].value > threshold=0.47115950286388397
node_126: feature_name=cg22946562	feature_id[744].value > threshold=0.6194809079170227
node_170: feature_name=cg12120430	feature_id[4185].value > threshold=0.5775820016860962
node_172: feature_name=cg02137970	feature_id[1411].value > threshold=0.6705857813358307
node_196: feature_name=cg03393445	feature_id[1807].value > threshold=0.4260125905275345
node_218: feature_name=cg23525541	feature_id[7049].value > threshold=0.4485483318567276
node_222: feature_name=cg10543634	feature_id[3775].value > threshold=0.29505227506160736
node_224: feature_name=cg07344990	feature_id[2926].value > threshold=0.5750180780887604
node_226: feature_name=cg10558233	feature_id[3780].value <= threshold=0.7217690348625183
node_227: feature_name=cg08097359	feature_id[3116].value > threshold=0.3598180413246155
node_229: feature_name=cg22737001	feature_id[6843].value <= threshold=0.46281898021698
node_230: feature_name=cg24276624	feature_id[7242].value > threshold=0.6169979274272919
node_232: feature_name=cg04026354	feature_id[1982].value <= threshold=0.42332448065280914
node_233: feature_name=cg06038180	feature_id[2529].value > threshold=0.6430551409721375

node_235: feature_name=cg14104252	feature_id[4735].value > threshold=0.5213949382305145
node_239: feature_name=cg06711298	feature_id[2707].value > threshold=0.36770085990428925
node_241: feature_name=cg11703722	feature_id[4056].value > threshold=0.5125315636396408
node_243: feature_name=cg21230793	feature_id[6514].value > threshold=0.27807849645614624
node_245: feature_name=cg06225767	feature_id[2575].value > threshold=0.18034886568784714
node_247: feature_name=cg00941576	feature_id[1053].value > threshold=0.2476368322968483
node_249: feature_name=cg10149889	feature_id[3676].value > threshold=0.633601188659668
node_257: feature_name=cg07608094	feature_id[2999].value > threshold=0.7820693254470825
node_259: feature_name=cg03315432	feature_id[1783].value > threshold=0.6790387332439423
node_261: feature_name=cg07894983	feature_id[3070].value > threshold=0.3577418476343155
node_263: feature_name=cg15646741	feature_id[5160].value > threshold=0.2826688587665558
node_273: feature_name=cg09701233	feature_id[3546].value > threshold=0.5189756155014038
node_275: feature_name=cg09230938	feature_id[738].value > threshold=0.3108274042606354
node_277: feature_name=cg05334190	feature_id[2341].value > threshold=0.2764553725719452
node_279: feature_name=cg15617847	feature_id[5148].value <= threshold=0.05914711393415928
node_280: feature_name=cg00216961	feature_id[816].value <= threshold=0.42650654911994934
node_281: feature_name=cg23946462	feature_id[7146].value <= threshold=0.9469821751117706
node_282: feature_name=cg06143290	feature_id[2554].value <= threshold=0.9263531863689423
node_283: feature_name=cg21528710	feature_id[6588].value <= threshold=0.8718287348747253
node_284: feature_name=cg14178043	feature_id[4760].value <= threshold=0.6852438449859619
node_285: feature_name=cg23906687	feature_id[7133].value > threshold=0.5761600136756897
node_287: feature_name=cg13838713	feature_id[4641].value > threshold=0.6983235478401184

node_289: feature_name=cg10239163	feature_id[3694].value > threshold=0.49343886971473694
node_291: feature_name=cg01197763	feature_id[1134].value > threshold=0.5575756281614304
node_293: feature_name=cg13210470	feature_id[4437].value > threshold=0.08023958280682564
node_295: feature_name=cg03731348	feature_id[1909].value > threshold=0.8963189721107483
node_297: feature_name=cg26929355	feature_id[633].value <= threshold=0.7743391394615173
Class: ossifying fibromyxoid tumour (OFMT)	
Rules_115	passed counts:1
node_0: feature_name=cg11915444	feature_id[4116].value > threshold=0.3402601182460785
node_10: feature_name=cg12109728	feature_id[4182].value > threshold=0.7068270146846771
node_20: feature_name=cg10844382	feature_id[3856].value > threshold=0.5988894104957581
node_24: feature_name=cg00631702	feature_id[943].value <= threshold=0.37470583617687225
node_25: feature_name=cg07912144	feature_id[3075].value > threshold=0.5578519105911255
node_29: feature_name=cg24407065	feature_id[388].value > threshold=0.5364363789558411
node_33: feature_name=cg15275017	feature_id[5069].value > threshold=0.4538573920726776
node_51: feature_name=cg07281938	feature_id[351].value > threshold=0.7506992518901825
node_73: feature_name=cg17524821	feature_id[5626].value > threshold=0.4504699558019638
node_75: feature_name=cg02973735	feature_id[1677].value > threshold=0.29383400082588196
node_77: feature_name=cg23345038	feature_id[7002].value > threshold=0.6255057156085968
node_79: feature_name=cg17537493	feature_id[5630].value > threshold=0.5825372636318207
node_85: feature_name=cg12948116	feature_id[4375].value <= threshold=0.5658791363239288
node_86: feature_name=cg06038180	feature_id[2529].value > threshold=0.43877437710762024
node_88: feature_name=cg15720017	feature_id[5175].value > threshold=0.7009969055652618

node_90: feature_name=cg13070215	feature_id[314].value <= threshold=0.14645694941282272
node_91: feature_name=cg01014262	feature_id[1082].value <= threshold=0.5737917125225067
node_92: feature_name=cg16863382	feature_id[5471].value > threshold=0.7200668156147003
node_102: feature_name=cg06633739	feature_id[2686].value > threshold=0.47115950286388397
node_126: feature_name=cg22946562	feature_id[744].value > threshold=0.6194809079170227
node_170: feature_name=cg12120430	feature_id[4185].value > threshold=0.5775820016860962
node_172: feature_name=cg02137970	feature_id[1411].value > threshold=0.6705857813358307
node_196: feature_name=cg03393445	feature_id[1807].value > threshold=0.4260125905275345
node_218: feature_name=cg23525541	feature_id[7049].value > threshold=0.4485483318567276
node_222: feature_name=cg10543634	feature_id[3775].value > threshold=0.29505227506160736
node_224: feature_name=cg07344990	feature_id[2926].value > threshold=0.5750180780887604
node_226: feature_name=cg10558233	feature_id[3780].value <= threshold=0.7217690348625183
node_227: feature_name=cg08097359	feature_id[3116].value > threshold=0.3598180413246155
node_229: feature_name=cg22737001	feature_id[6843].value <= threshold=0.46281898021698
node_230: feature_name=cg24276624	feature_id[7242].value > threshold=0.6169979274272919
node_232: feature_name=cg04026354	feature_id[1982].value <= threshold=0.42332448065280914
node_233: feature_name=cg06038180	feature_id[2529].value > threshold=0.6430551409721375
node_235: feature_name=cg14104252	feature_id[4735].value > threshold=0.5213949382305145
node_239: feature_name=cg06711298	feature_id[2707].value > threshold=0.36770085990428925
node_241: feature_name=cg11703722	feature_id[4056].value > threshold=0.5125315636396408
node_243: feature_name=cg21230793	feature_id[6514].value > threshold=0.27807849645614624
node_245: feature_name=cg06225767	feature_id[2575].value > threshold=0.18034886568784714

node_247: feature_name=cg00941576	feature_id[1053].value > threshold=0.2476368322968483
node_249: feature_name=cg10149889	feature_id[3676].value > threshold=0.633601188659668
node_257: feature_name=cg07608094	feature_id[2999].value > threshold=0.7820693254470825
node_259: feature_name=cg03315432	feature_id[1783].value > threshold=0.6790387332439423
node_261: feature_name=cg07894983	feature_id[3070].value > threshold=0.3577418476343155
node_263: feature_name=cg15646741	feature_id[5160].value > threshold=0.2826688587665558
node_273: feature_name=cg09701233	feature_id[3546].value > threshold=0.5189756155014038
node_275: feature_name=cg09230938	feature_id[738].value > threshold=0.3108274042606354
node_277: feature_name=cg05334190	feature_id[2341].value > threshold=0.2764553725719452
node_279: feature_name=cg15617847	feature_id[5148].value <= threshold=0.05914711393415928
node_280: feature_name=cg00216961	feature_id[816].value <= threshold=0.42650654911994934
node_281: feature_name=cg23946462	feature_id[7146].value <= threshold=0.9469821751117706
node_282: feature_name=cg06143290	feature_id[2554].value <= threshold=0.9263531863689423
node_283: feature_name=cg21528710	feature_id[6588].value <= threshold=0.8718287348747253
node_284: feature_name=cg14178043	feature_id[4760].value <= threshold=0.6852438449859619
node_285: feature_name=cg23906687	feature_id[7133].value > threshold=0.5761600136756897
node_287: feature_name=cg13838713	feature_id[4641].value > threshold=0.6983235478401184
node_289: feature_name=cg10239163	feature_id[3694].value > threshold=0.49343886971473694
node_291: feature_name=cg01197763	feature_id[1134].value > threshold=0.5575756281614304
node_293: feature_name=cg13210470	feature_id[4437].value > threshold=0.08023958280682564
node_295: feature_name=cg03731348	feature_id[1909].value <= threshold=0.8963189721107483
Class: alveolar soft part sarcoma (ASPS)	

Rules_116	passed counts:1
node_0: feature_name=cg11915444	feature_id[4116].value > threshold=0.3402601182460785
node_10: feature_name=cg12109728	feature_id[4182].value > threshold=0.7068270146846771
node_20: feature_name=cg10844382	feature_id[3856].value > threshold=0.5988894104957581
node_24: feature_name=cg00631702	feature_id[943].value <= threshold=0.37470583617687225
node_25: feature_name=cg07912144	feature_id[3075].value > threshold=0.5578519105911255
node_29: feature_name=cg24407065	feature_id[388].value > threshold=0.5364363789558411
node_33: feature_name=cg15275017	feature_id[5069].value > threshold=0.4538573920726776
node_51: feature_name=cg07281938	feature_id[351].value > threshold=0.7506992518901825
node_73: feature_name=cg17524821	feature_id[5626].value > threshold=0.4504699558019638
node_75: feature_name=cg02973735	feature_id[1677].value > threshold=0.29383400082588196
node_77: feature_name=cg23345038	feature_id[7002].value > threshold=0.6255057156085968
node_79: feature_name=cg17537493	feature_id[5630].value > threshold=0.5825372636318207
node_85: feature_name=cg12948116	feature_id[4375].value <= threshold=0.5658791363239288
node_86: feature_name=cg06038180	feature_id[2529].value > threshold=0.43877437710762024
node_88: feature_name=cg15720017	feature_id[5175].value > threshold=0.7009969055652618
node_90: feature_name=cg13070215	feature_id[314].value <= threshold=0.14645694941282272
node_91: feature_name=cg01014262	feature_id[1082].value <= threshold=0.5737917125225067
node_92: feature_name=cg16863382	feature_id[5471].value > threshold=0.7200668156147003
node_102: feature_name=cg06633739	feature_id[2686].value > threshold=0.47115950286388397
node_126: feature_name=cg22946562	feature_id[744].value > threshold=0.6194809079170227

node_170: feature_name=cg12120430	feature_id[4185].value > threshold=0.5775820016860962
node_172: feature_name=cg02137970	feature_id[1411].value > threshold=0.6705857813358307
node_196: feature_name=cg03393445	feature_id[1807].value > threshold=0.4260125905275345
node_218: feature_name=cg23525541	feature_id[7049].value > threshold=0.4485483318567276
node_222: feature_name=cg10543634	feature_id[3775].value > threshold=0.29505227506160736
node_224: feature_name=cg07344990	feature_id[2926].value > threshold=0.5750180780887604
node_226: feature_name=cg10558233	feature_id[3780].value <= threshold=0.7217690348625183
node_227: feature_name=cg08097359	feature_id[3116].value > threshold=0.3598180413246155
node_229: feature_name=cg22737001	feature_id[6843].value <= threshold=0.46281898021698
node_230: feature_name=cg24276624	feature_id[7242].value > threshold=0.6169979274272919
node_232: feature_name=cg04026354	feature_id[1982].value <= threshold=0.42332448065280914
node_233: feature_name=cg06038180	feature_id[2529].value > threshold=0.6430551409721375
node_235: feature_name=cg14104252	feature_id[4735].value > threshold=0.5213949382305145
node_239: feature_name=cg06711298	feature_id[2707].value > threshold=0.36770085990428925
node_241: feature_name=cg11703722	feature_id[4056].value > threshold=0.5125315636396408
node_243: feature_name=cg21230793	feature_id[6514].value > threshold=0.27807849645614624
node_245: feature_name=cg06225767	feature_id[2575].value > threshold=0.18034886568784714
node_247: feature_name=cg00941576	feature_id[1053].value > threshold=0.2476368322968483
node_249: feature_name=cg10149889	feature_id[3676].value > threshold=0.633601188659668
node_257: feature_name=cg07608094	feature_id[2999].value > threshold=0.7820693254470825
node_259: feature_name=cg03315432	feature_id[1783].value > threshold=0.6790387332439423
node_261: feature_name=cg07894983	feature_id[3070].value > threshold=0.3577418476343155

node_263: feature_name=cg15646741	feature_id[5160].value > threshold=0.2826688587665558
node_273: feature_name=cg09701233	feature_id[3546].value > threshold=0.5189756155014038
node_275: feature_name=cg09230938	feature_id[738].value > threshold=0.3108274042606354
node_277: feature_name=cg05334190	feature_id[2341].value > threshold=0.2764553725719452
node_279: feature_name=cg15617847	feature_id[5148].value <= threshold=0.05914711393415928
node_280: feature_name=cg00216961	feature_id[816].value <= threshold=0.42650654911994934
node_281: feature_name=cg23946462	feature_id[7146].value <= threshold=0.9469821751117706
node_282: feature_name=cg06143290	feature_id[2554].value <= threshold=0.9263531863689423
node_283: feature_name=cg21528710	feature_id[6588].value <= threshold=0.8718287348747253
node_284: feature_name=cg14178043	feature_id[4760].value <= threshold=0.6852438449859619
node_285: feature_name=cg23906687	feature_id[7133].value > threshold=0.5761600136756897
node_287: feature_name=cg13838713	feature_id[4641].value > threshold=0.6983235478401184
node_289: feature_name=cg10239163	feature_id[3694].value > threshold=0.49343886971473694
node_291: feature_name=cg01197763	feature_id[1134].value > threshold=0.5575756281614304
node_293: feature_name=cg13210470	feature_id[4437].value <= threshold=0.08023958280682564
Class: malignant rhabdoid tumour (MRT)	
Rules_117	passed counts:1
node_0: feature_name=cg11915444	feature_id[4116].value > threshold=0.3402601182460785
node_10: feature_name=cg12109728	feature_id[4182].value > threshold=0.7068270146846771
node_20: feature_name=cg10844382	feature_id[3856].value > threshold=0.5988894104957581
node_24: feature_name=cg00631702	feature_id[943].value <= threshold=0.37470583617687225
node_25: feature_name=cg07912144	feature_id[3075].value > threshold=0.5578519105911255

node_29: feature_name=cg24407065	feature_id[388].value > threshold=0.5364363789558411
node_33: feature_name=cg15275017	feature_id[5069].value > threshold=0.4538573920726776
node_51: feature_name=cg07281938	feature_id[351].value > threshold=0.7506992518901825
node_73: feature_name=cg17524821	feature_id[5626].value > threshold=0.4504699558019638
node_75: feature_name=cg02973735	feature_id[1677].value > threshold=0.29383400082588196
node_77: feature_name=cg23345038	feature_id[7002].value > threshold=0.6255057156085968
node_79: feature_name=cg17537493	feature_id[5630].value > threshold=0.5825372636318207
node_85: feature_name=cg12948116	feature_id[4375].value <= threshold=0.5658791363239288
node_86: feature_name=cg06038180	feature_id[2529].value > threshold=0.43877437710762024
node_88: feature_name=cg15720017	feature_id[5175].value > threshold=0.7009969055652618
node_90: feature_name=cg13070215	feature_id[314].value <= threshold=0.14645694941282272
node_91: feature_name=cg01014262	feature_id[1082].value <= threshold=0.5737917125225067
node_92: feature_name=cg16863382	feature_id[5471].value > threshold=0.7200668156147003
node_102: feature_name=cg06633739	feature_id[2686].value > threshold=0.47115950286388397
node_126: feature_name=cg22946562	feature_id[744].value > threshold=0.6194809079170227
node_170: feature_name=cg12120430	feature_id[4185].value > threshold=0.5775820016860962
node_172: feature_name=cg02137970	feature_id[1411].value > threshold=0.6705857813358307
node_196: feature_name=cg03393445	feature_id[1807].value > threshold=0.4260125905275345
node_218: feature_name=cg23525541	feature_id[7049].value > threshold=0.4485483318567276
node_222: feature_name=cg10543634	feature_id[3775].value > threshold=0.29505227506160736
node_224: feature_name=cg07344990	feature_id[2926].value > threshold=0.5750180780887604
node_226: feature_name=cg10558233	feature_id[3780].value <= threshold=0.7217690348625183

node_227: feature_name=cg08097359	feature_id[3116].value > threshold=0.3598180413246155
node_229: feature_name=cg22737001	feature_id[6843].value <= threshold=0.46281898021698
node_230: feature_name=cg24276624	feature_id[7242].value > threshold=0.6169979274272919
node_232: feature_name=cg04026354	feature_id[1982].value <= threshold=0.42332448065280914
node_233: feature_name=cg06038180	feature_id[2529].value > threshold=0.6430551409721375
node_235: feature_name=cg14104252	feature_id[4735].value > threshold=0.5213949382305145
node_239: feature_name=cg06711298	feature_id[2707].value > threshold=0.36770085990428925
node_241: feature_name=cg11703722	feature_id[4056].value > threshold=0.5125315636396408
node_243: feature_name=cg21230793	feature_id[6514].value > threshold=0.27807849645614624
node_245: feature_name=cg06225767	feature_id[2575].value > threshold=0.18034886568784714
node_247: feature_name=cg00941576	feature_id[1053].value > threshold=0.2476368322968483
node_249: feature_name=cg10149889	feature_id[3676].value > threshold=0.633601188659668
node_257: feature_name=cg07608094	feature_id[2999].value > threshold=0.7820693254470825
node_259: feature_name=cg03315432	feature_id[1783].value > threshold=0.6790387332439423
node_261: feature_name=cg07894983	feature_id[3070].value > threshold=0.3577418476343155
node_263: feature_name=cg15646741	feature_id[5160].value > threshold=0.2826688587665558
node_273: feature_name=cg09701233	feature_id[3546].value > threshold=0.5189756155014038
node_275: feature_name=cg09230938	feature_id[738].value > threshold=0.3108274042606354
node_277: feature_name=cg05334190	feature_id[2341].value > threshold=0.2764553725719452
node_279: feature_name=cg15617847	feature_id[5148].value <= threshold=0.05914711393415928
node_280: feature_name=cg00216961	feature_id[816].value <= threshold=0.42650654911994934
node_281: feature_name=cg23946462	feature_id[7146].value <= threshold=0.9469821751117706

node_282: feature_name=cg06143290	feature_id[2554].value <= threshold=0.9263531863689423
node_283: feature_name=cg21528710	feature_id[6588].value <= threshold=0.8718287348747253
node_284: feature_name=cg14178043	feature_id[4760].value <= threshold=0.6852438449859619
node_285: feature_name=cg23906687	feature_id[7133].value > threshold=0.5761600136756897
node_287: feature_name=cg13838713	feature_id[4641].value > threshold=0.6983235478401184
node_289: feature_name=cg10239163	feature_id[3694].value > threshold=0.49343886971473694
node_291: feature_name=cg01197763	feature_id[1134].value <= threshold=0.5575756281614304
Class: synovial sarcoma (SYSA)	
Rules_118	passed counts:1
node_0: feature_name=cg11915444	feature_id[4116].value > threshold=0.3402601182460785
node_10: feature_name=cg12109728	feature_id[4182].value > threshold=0.7068270146846771
node_20: feature_name=cg10844382	feature_id[3856].value > threshold=0.5988894104957581
node_24: feature_name=cg00631702	feature_id[943].value <= threshold=0.37470583617687225
node_25: feature_name=cg07912144	feature_id[3075].value > threshold=0.5578519105911255
node_29: feature_name=cg24407065	feature_id[388].value > threshold=0.5364363789558411
node_33: feature_name=cg15275017	feature_id[5069].value > threshold=0.4538573920726776
node_51: feature_name=cg07281938	feature_id[351].value > threshold=0.7506992518901825
node_73: feature_name=cg17524821	feature_id[5626].value > threshold=0.4504699558019638
node_75: feature_name=cg02973735	feature_id[1677].value > threshold=0.29383400082588196
node_77: feature_name=cg23345038	feature_id[7002].value > threshold=0.6255057156085968
node_79: feature_name=cg17537493	feature_id[5630].value > threshold=0.5825372636318207
node_85: feature_name=cg12948116	feature_id[4375].value <= threshold=0.5658791363239288

node_86: feature_name=cg06038180	feature_id[2529].value > threshold=0.43877437710762024
node_88: feature_name=cg15720017	feature_id[5175].value > threshold=0.7009969055652618
node_90: feature_name=cg13070215	feature_id[314].value <= threshold=0.14645694941282272
node_91: feature_name=cg01014262	feature_id[1082].value <= threshold=0.5737917125225067
node_92: feature_name=cg16863382	feature_id[5471].value > threshold=0.7200668156147003
node_102: feature_name=cg06633739	feature_id[2686].value > threshold=0.47115950286388397
node_126: feature_name=cg22946562	feature_id[744].value > threshold=0.6194809079170227
node_170: feature_name=cg12120430	feature_id[4185].value > threshold=0.5775820016860962
node_172: feature_name=cg02137970	feature_id[1411].value > threshold=0.6705857813358307
node_196: feature_name=cg03393445	feature_id[1807].value > threshold=0.4260125905275345
node_218: feature_name=cg23525541	feature_id[7049].value > threshold=0.4485483318567276
node_222: feature_name=cg10543634	feature_id[3775].value > threshold=0.29505227506160736
node_224: feature_name=cg07344990	feature_id[2926].value > threshold=0.5750180780887604
node_226: feature_name=cg10558233	feature_id[3780].value <= threshold=0.7217690348625183
node_227: feature_name=cg08097359	feature_id[3116].value > threshold=0.3598180413246155
node_229: feature_name=cg22737001	feature_id[6843].value <= threshold=0.46281898021698
node_230: feature_name=cg24276624	feature_id[7242].value > threshold=0.6169979274272919
node_232: feature_name=cg04026354	feature_id[1982].value <= threshold=0.42332448065280914
node_233: feature_name=cg06038180	feature_id[2529].value > threshold=0.6430551409721375
node_235: feature_name=cg14104252	feature_id[4735].value > threshold=0.5213949382305145
node_239: feature_name=cg06711298	feature_id[2707].value > threshold=0.36770085990428925
node_241: feature_name=cg11703722	feature_id[4056].value > threshold=0.5125315636396408

node_243: feature_name=cg21230793	feature_id[6514].value > threshold=0.27807849645614624
node_245: feature_name=cg06225767	feature_id[2575].value > threshold=0.18034886568784714
node_247: feature_name=cg00941576	feature_id[1053].value > threshold=0.2476368322968483
node_249: feature_name=cg10149889	feature_id[3676].value > threshold=0.633601188659668
node_257: feature_name=cg07608094	feature_id[2999].value > threshold=0.7820693254470825
node_259: feature_name=cg03315432	feature_id[1783].value > threshold=0.6790387332439423
node_261: feature_name=cg07894983	feature_id[3070].value > threshold=0.3577418476343155
node_263: feature_name=cg15646741	feature_id[5160].value <= threshold=0.2826688587665558
node_264: feature_name=cg05400196	feature_id[2366].value <= threshold=0.666697084903717
node_265: feature_name=cg11936643	feature_id[4120].value > threshold=0.19320054352283478
node_267: feature_name=cg02436098	feature_id[1507].value > threshold=0.20541958510875702
node_269: feature_name=cg05041061	feature_id[2257].value > threshold=0.778236985206604
Class: Kaposi sarcoma (KS)	
Rules_119	passed counts:1
node_0: feature_name=cg11915444	feature_id[4116].value > threshold=0.3402601182460785
node_10: feature_name=cg12109728	feature_id[4182].value > threshold=0.7068270146846771
node_20: feature_name=cg10844382	feature_id[3856].value > threshold=0.5988894104957581
node_24: feature_name=cg00631702	feature_id[943].value <= threshold=0.37470583617687225
node_25: feature_name=cg07912144	feature_id[3075].value > threshold=0.5578519105911255
node_29: feature_name=cg24407065	feature_id[388].value > threshold=0.5364363789558411
node_33: feature_name=cg15275017	feature_id[5069].value > threshold=0.4538573920726776
node_51: feature_name=cg07281938	feature_id[351].value > threshold=0.7506992518901825

node_73: feature_name=cg17524821	feature_id[5626].value > threshold=0.4504699558019638
node_75: feature_name=cg02973735	feature_id[1677].value > threshold=0.29383400082588196
node_77: feature_name=cg23345038	feature_id[7002].value > threshold=0.6255057156085968
node_79: feature_name=cg17537493	feature_id[5630].value > threshold=0.5825372636318207
node_85: feature_name=cg12948116	feature_id[4375].value <= threshold=0.5658791363239288
node_86: feature_name=cg06038180	feature_id[2529].value > threshold=0.43877437710762024
node_88: feature_name=cg15720017	feature_id[5175].value > threshold=0.7009969055652618
node_90: feature_name=cg13070215	feature_id[314].value <= threshold=0.14645694941282272
node_91: feature_name=cg01014262	feature_id[1082].value <= threshold=0.5737917125225067
node_92: feature_name=cg16863382	feature_id[5471].value > threshold=0.7200668156147003
node_102: feature_name=cg06633739	feature_id[2686].value > threshold=0.47115950286388397
node_126: feature_name=cg22946562	feature_id[744].value > threshold=0.6194809079170227
node_170: feature_name=cg12120430	feature_id[4185].value > threshold=0.5775820016860962
node_172: feature_name=cg02137970	feature_id[1411].value > threshold=0.6705857813358307
node_196: feature_name=cg03393445	feature_id[1807].value > threshold=0.4260125905275345
node_218: feature_name=cg23525541	feature_id[7049].value > threshold=0.4485483318567276
node_222: feature_name=cg10543634	feature_id[3775].value > threshold=0.29505227506160736
node_224: feature_name=cg07344990	feature_id[2926].value > threshold=0.5750180780887604
node_226: feature_name=cg10558233	feature_id[3780].value <= threshold=0.7217690348625183
node_227: feature_name=cg08097359	feature_id[3116].value > threshold=0.3598180413246155
node_229: feature_name=cg22737001	feature_id[6843].value <= threshold=0.46281898021698
node_230: feature_name=cg24276624	feature_id[7242].value > threshold=0.6169979274272919

node_232: feature_name=cg04026354	feature_id[1982].value <= threshold=0.42332448065280914
node_233: feature_name=cg06038180	feature_id[2529].value > threshold=0.6430551409721375
node_235: feature_name=cg14104252	feature_id[4735].value > threshold=0.5213949382305145
node_239: feature_name=cg06711298	feature_id[2707].value > threshold=0.36770085990428925
node_241: feature_name=cg11703722	feature_id[4056].value > threshold=0.5125315636396408
node_243: feature_name=cg21230793	feature_id[6514].value > threshold=0.27807849645614624
node_245: feature_name=cg06225767	feature_id[2575].value > threshold=0.18034886568784714
node_247: feature_name=cg00941576	feature_id[1053].value > threshold=0.2476368322968483
node_249: feature_name=cg10149889	feature_id[3676].value > threshold=0.633601188659668
node_257: feature_name=cg07608094	feature_id[2999].value > threshold=0.7820693254470825
node_259: feature_name=cg03315432	feature_id[1783].value > threshold=0.6790387332439423
node_261: feature_name=cg07894983	feature_id[3070].value > threshold=0.3577418476343155
node_263: feature_name=cg15646741	feature_id[5160].value <= threshold=0.2826688587665558
node_264: feature_name=cg05400196	feature_id[2366].value <= threshold=0.666697084903717
node_265: feature_name=cg11936643	feature_id[4120].value > threshold=0.19320054352283478
node_267: feature_name=cg02436098	feature_id[1507].value > threshold=0.20541958510875702
node_269: feature_name=cg05041061	feature_id[2257].value <= threshold=0.778236985206604
Class: alveolar soft part sarcoma (ASPS)	
Rules_120	passed counts:1
node_0: feature_name=cg11915444	feature_id[4116].value > threshold=0.3402601182460785
node_10: feature_name=cg12109728	feature_id[4182].value > threshold=0.7068270146846771
node_20: feature_name=cg10844382	feature_id[3856].value > threshold=0.5988894104957581

node_24: feature_name=cg00631702	feature_id[943].value <= threshold=0.37470583617687225
node_25: feature_name=cg07912144	feature_id[3075].value > threshold=0.5578519105911255
node_29: feature_name=cg24407065	feature_id[388].value > threshold=0.5364363789558411
node_33: feature_name=cg15275017	feature_id[5069].value > threshold=0.4538573920726776
node_51: feature_name=cg07281938	feature_id[351].value > threshold=0.7506992518901825
node_73: feature_name=cg17524821	feature_id[5626].value > threshold=0.4504699558019638
node_75: feature_name=cg02973735	feature_id[1677].value > threshold=0.29383400082588196
node_77: feature_name=cg23345038	feature_id[7002].value > threshold=0.6255057156085968
node_79: feature_name=cg17537493	feature_id[5630].value > threshold=0.5825372636318207
node_85: feature_name=cg12948116	feature_id[4375].value <= threshold=0.5658791363239288
node_86: feature_name=cg06038180	feature_id[2529].value > threshold=0.43877437710762024
node_88: feature_name=cg15720017	feature_id[5175].value > threshold=0.7009969055652618
node_90: feature_name=cg13070215	feature_id[314].value <= threshold=0.14645694941282272
node_91: feature_name=cg01014262	feature_id[1082].value <= threshold=0.5737917125225067
node_92: feature_name=cg16863382	feature_id[5471].value > threshold=0.7200668156147003
node_102: feature_name=cg06633739	feature_id[2686].value > threshold=0.47115950286388397
node_126: feature_name=cg22946562	feature_id[744].value > threshold=0.6194809079170227
node_170: feature_name=cg12120430	feature_id[4185].value > threshold=0.5775820016860962
node_172: feature_name=cg02137970	feature_id[1411].value > threshold=0.6705857813358307
node_196: feature_name=cg03393445	feature_id[1807].value > threshold=0.4260125905275345
node_218: feature_name=cg23525541	feature_id[7049].value > threshold=0.4485483318567276
node_222: feature_name=cg10543634	feature_id[3775].value > threshold=0.29505227506160736

node_224: feature_name=cg07344990	feature_id[2926].value > threshold=0.5750180780887604
node_226: feature_name=cg10558233	feature_id[3780].value <= threshold=0.7217690348625183
node_227: feature_name=cg08097359	feature_id[3116].value > threshold=0.3598180413246155
node_229: feature_name=cg22737001	feature_id[6843].value <= threshold=0.46281898021698
node_230: feature_name=cg24276624	feature_id[7242].value > threshold=0.6169979274272919
node_232: feature_name=cg04026354	feature_id[1982].value <= threshold=0.42332448065280914
node_233: feature_name=cg06038180	feature_id[2529].value > threshold=0.6430551409721375
node_235: feature_name=cg14104252	feature_id[4735].value > threshold=0.5213949382305145
node_239: feature_name=cg06711298	feature_id[2707].value > threshold=0.36770085990428925
node_241: feature_name=cg11703722	feature_id[4056].value > threshold=0.5125315636396408
node_243: feature_name=cg21230793	feature_id[6514].value > threshold=0.27807849645614624
node_245: feature_name=cg06225767	feature_id[2575].value > threshold=0.18034886568784714
node_247: feature_name=cg00941576	feature_id[1053].value > threshold=0.2476368322968483
node_249: feature_name=cg10149889	feature_id[3676].value > threshold=0.633601188659668
node_257: feature_name=cg07608094	feature_id[2999].value > threshold=0.7820693254470825
node_259: feature_name=cg03315432	feature_id[1783].value > threshold=0.6790387332439423
node_261: feature_name=cg07894983	feature_id[3070].value > threshold=0.3577418476343155
node_263: feature_name=cg15646741	feature_id[5160].value <= threshold=0.2826688587665558
node_264: feature_name=cg05400196	feature_id[2366].value <= threshold=0.666697084903717
node_265: feature_name=cg11936643	feature_id[4120].value > threshold=0.19320054352283478
node_267: feature_name=cg02436098	feature_id[1507].value <= threshold=0.20541958510875702
Class: sarcoma (SARC)	

Rules_121	passed counts:1
node_0: feature_name=cg11915444	feature_id[4116].value > threshold=0.3402601182460785
node_10: feature_name=cg12109728	feature_id[4182].value > threshold=0.7068270146846771
node_20: feature_name=cg10844382	feature_id[3856].value > threshold=0.5988894104957581
node_24: feature_name=cg00631702	feature_id[943].value <= threshold=0.37470583617687225
node_25: feature_name=cg07912144	feature_id[3075].value > threshold=0.5578519105911255
node_29: feature_name=cg24407065	feature_id[388].value > threshold=0.5364363789558411
node_33: feature_name=cg15275017	feature_id[5069].value > threshold=0.4538573920726776
node_51: feature_name=cg07281938	feature_id[351].value > threshold=0.7506992518901825
node_73: feature_name=cg17524821	feature_id[5626].value > threshold=0.4504699558019638
node_75: feature_name=cg02973735	feature_id[1677].value > threshold=0.29383400082588196
node_77: feature_name=cg23345038	feature_id[7002].value > threshold=0.6255057156085968
node_79: feature_name=cg17537493	feature_id[5630].value > threshold=0.5825372636318207
node_85: feature_name=cg12948116	feature_id[4375].value <= threshold=0.5658791363239288
node_86: feature_name=cg06038180	feature_id[2529].value > threshold=0.43877437710762024
node_88: feature_name=cg15720017	feature_id[5175].value > threshold=0.7009969055652618
node_90: feature_name=cg13070215	feature_id[314].value <= threshold=0.14645694941282272
node_91: feature_name=cg01014262	feature_id[1082].value <= threshold=0.5737917125225067
node_92: feature_name=cg16863382	feature_id[5471].value > threshold=0.7200668156147003
node_102: feature_name=cg06633739	feature_id[2686].value > threshold=0.47115950286388397
node_126: feature_name=cg22946562	feature_id[744].value > threshold=0.6194809079170227

node_170: feature_name=cg12120430	feature_id[4185].value > threshold=0.5775820016860962
node_172: feature_name=cg02137970	feature_id[1411].value > threshold=0.6705857813358307
node_196: feature_name=cg03393445	feature_id[1807].value > threshold=0.4260125905275345
node_218: feature_name=cg23525541	feature_id[7049].value > threshold=0.4485483318567276
node_222: feature_name=cg10543634	feature_id[3775].value > threshold=0.29505227506160736
node_224: feature_name=cg07344990	feature_id[2926].value > threshold=0.5750180780887604
node_226: feature_name=cg10558233	feature_id[3780].value <= threshold=0.7217690348625183
node_227: feature_name=cg08097359	feature_id[3116].value > threshold=0.3598180413246155
node_229: feature_name=cg22737001	feature_id[6843].value <= threshold=0.46281898021698
node_230: feature_name=cg24276624	feature_id[7242].value > threshold=0.6169979274272919
node_232: feature_name=cg04026354	feature_id[1982].value <= threshold=0.42332448065280914
node_233: feature_name=cg06038180	feature_id[2529].value > threshold=0.6430551409721375
node_235: feature_name=cg14104252	feature_id[4735].value > threshold=0.5213949382305145
node_239: feature_name=cg06711298	feature_id[2707].value > threshold=0.36770085990428925
node_241: feature_name=cg11703722	feature_id[4056].value > threshold=0.5125315636396408
node_243: feature_name=cg21230793	feature_id[6514].value > threshold=0.27807849645614624
node_245: feature_name=cg06225767	feature_id[2575].value > threshold=0.18034886568784714
node_247: feature_name=cg00941576	feature_id[1053].value > threshold=0.2476368322968483
node_249: feature_name=cg10149889	feature_id[3676].value <= threshold=0.633601188659668
node_250: feature_name=cg03631837	feature_id[1881].value <= threshold=0.8389041423797607
node_251: feature_name=cg18201671	feature_id[5790].value > threshold=0.7097389698028564
node_253: feature_name=cg14406727	feature_id[4837].value > threshold=0.7478070855140686

Class: leiomyosarcoma (LMS)	
Rules_122	passed counts:1
node_0: feature_name=cg11915444	feature_id[4116].value > threshold=0.3402601182460785
node_10: feature_name=cg12109728	feature_id[4182].value > threshold=0.7068270146846771
node_20: feature_name=cg10844382	feature_id[3856].value > threshold=0.5988894104957581
node_24: feature_name=cg00631702	feature_id[943].value <= threshold=0.37470583617687225
node_25: feature_name=cg07912144	feature_id[3075].value > threshold=0.5578519105911255
node_29: feature_name=cg24407065	feature_id[388].value > threshold=0.5364363789558411
node_33: feature_name=cg15275017	feature_id[5069].value > threshold=0.4538573920726776
node_51: feature_name=cg07281938	feature_id[351].value > threshold=0.7506992518901825
node_73: feature_name=cg17524821	feature_id[5626].value > threshold=0.4504699558019638
node_75: feature_name=cg02973735	feature_id[1677].value > threshold=0.29383400082588196
node_77: feature_name=cg23345038	feature_id[7002].value > threshold=0.6255057156085968
node_79: feature_name=cg17537493	feature_id[5630].value > threshold=0.5825372636318207
node_85: feature_name=cg12948116	feature_id[4375].value <= threshold=0.5658791363239288
node_86: feature_name=cg06038180	feature_id[2529].value > threshold=0.43877437710762024
node_88: feature_name=cg15720017	feature_id[5175].value > threshold=0.7009969055652618
node_90: feature_name=cg13070215	feature_id[314].value <= threshold=0.14645694941282272
node_91: feature_name=cg01014262	feature_id[1082].value <= threshold=0.5737917125225067
node_92: feature_name=cg16863382	feature_id[5471].value > threshold=0.7200668156147003
node_102: feature_name=cg06633739	feature_id[2686].value > threshold=0.47115950286388397
node_126: feature_name=cg22946562	feature_id[744].value > threshold=0.6194809079170227

node_170: feature_name=cg12120430	feature_id[4185].value > threshold=0.5775820016860962
node_172: feature_name=cg02137970	feature_id[1411].value > threshold=0.6705857813358307
node_196: feature_name=cg03393445	feature_id[1807].value > threshold=0.4260125905275345
node_218: feature_name=cg23525541	feature_id[7049].value > threshold=0.4485483318567276
node_222: feature_name=cg10543634	feature_id[3775].value > threshold=0.29505227506160736
node_224: feature_name=cg07344990	feature_id[2926].value > threshold=0.5750180780887604
node_226: feature_name=cg10558233	feature_id[3780].value <= threshold=0.7217690348625183
node_227: feature_name=cg08097359	feature_id[3116].value > threshold=0.3598180413246155
node_229: feature_name=cg22737001	feature_id[6843].value <= threshold=0.46281898021698
node_230: feature_name=cg24276624	feature_id[7242].value > threshold=0.6169979274272919
node_232: feature_name=cg04026354	feature_id[1982].value <= threshold=0.42332448065280914
node_233: feature_name=cg06038180	feature_id[2529].value > threshold=0.6430551409721375
node_235: feature_name=cg14104252	feature_id[4735].value > threshold=0.5213949382305145
node_239: feature_name=cg06711298	feature_id[2707].value > threshold=0.36770085990428925
node_241: feature_name=cg11703722	feature_id[4056].value > threshold=0.5125315636396408
node_243: feature_name=cg21230793	feature_id[6514].value > threshold=0.27807849645614624
node_245: feature_name=cg06225767	feature_id[2575].value > threshold=0.18034886568784714
node_247: feature_name=cg00941576	feature_id[1053].value > threshold=0.2476368322968483
node_249: feature_name=cg10149889	feature_id[3676].value <= threshold=0.633601188659668
node_250: feature_name=cg03631837	feature_id[1881].value <= threshold=0.8389041423797607
node_251: feature_name=cg18201671	feature_id[5790].value > threshold=0.7097389698028564
node_253: feature_name=cg14406727	feature_id[4837].value <= threshold=0.7478070855140686

Class: atypical fibroxanthoma (AFX)/pleomorphic dermal sarcoma (PDS)	
Rules_123	passed counts:1
node_0: feature_name=cg11915444	feature_id[4116].value > threshold=0.3402601182460785
node_10: feature_name=cg12109728	feature_id[4182].value > threshold=0.7068270146846771
node_20: feature_name=cg10844382	feature_id[3856].value > threshold=0.5988894104957581
node_24: feature_name=cg00631702	feature_id[943].value <= threshold=0.37470583617687225
node_25: feature_name=cg07912144	feature_id[3075].value > threshold=0.5578519105911255
node_29: feature_name=cg24407065	feature_id[388].value > threshold=0.5364363789558411
node_33: feature_name=cg15275017	feature_id[5069].value > threshold=0.4538573920726776
node_51: feature_name=cg07281938	feature_id[351].value > threshold=0.7506992518901825
node_73: feature_name=cg17524821	feature_id[5626].value > threshold=0.4504699558019638
node_75: feature_name=cg02973735	feature_id[1677].value > threshold=0.29383400082588196
node_77: feature_name=cg23345038	feature_id[7002].value > threshold=0.6255057156085968
node_79: feature_name=cg17537493	feature_id[5630].value > threshold=0.5825372636318207
node_85: feature_name=cg12948116	feature_id[4375].value <= threshold=0.5658791363239288
node_86: feature_name=cg06038180	feature_id[2529].value > threshold=0.43877437710762024
node_88: feature_name=cg15720017	feature_id[5175].value > threshold=0.7009969055652618
node_90: feature_name=cg13070215	feature_id[314].value <= threshold=0.14645694941282272
node_91: feature_name=cg01014262	feature_id[1082].value <= threshold=0.5737917125225067
node_92: feature_name=cg16863382	feature_id[5471].value > threshold=0.7200668156147003
node_102: feature_name=cg06633739	feature_id[2686].value > threshold=0.47115950286388397
node_126: feature_name=cg22946562	feature_id[744].value > threshold=0.6194809079170227

node_170: feature_name=cg12120430	feature_id[4185].value > threshold=0.5775820016860962
node_172: feature_name=cg02137970	feature_id[1411].value > threshold=0.6705857813358307
node_196: feature_name=cg03393445	feature_id[1807].value > threshold=0.4260125905275345
node_218: feature_name=cg23525541	feature_id[7049].value > threshold=0.4485483318567276
node_222: feature_name=cg10543634	feature_id[3775].value > threshold=0.29505227506160736
node_224: feature_name=cg07344990	feature_id[2926].value > threshold=0.5750180780887604
node_226: feature_name=cg10558233	feature_id[3780].value <= threshold=0.7217690348625183
node_227: feature_name=cg08097359	feature_id[3116].value > threshold=0.3598180413246155
node_229: feature_name=cg22737001	feature_id[6843].value <= threshold=0.46281898021698
node_230: feature_name=cg24276624	feature_id[7242].value > threshold=0.6169979274272919
node_232: feature_name=cg04026354	feature_id[1982].value <= threshold=0.42332448065280914
node_233: feature_name=cg06038180	feature_id[2529].value > threshold=0.6430551409721375
node_235: feature_name=cg14104252	feature_id[4735].value <= threshold=0.5213949382305145
node_236: feature_name=cg12163781	feature_id[4197].value <= threshold=0.6278624832630157
Class: osteoblastoma (OB)	
Rules_124	passed counts:1
node_0: feature_name=cg11915444	feature_id[4116].value > threshold=0.3402601182460785
node_10: feature_name=cg12109728	feature_id[4182].value > threshold=0.7068270146846771
node_20: feature_name=cg10844382	feature_id[3856].value > threshold=0.5988894104957581
node_24: feature_name=cg00631702	feature_id[943].value <= threshold=0.37470583617687225
node_25: feature_name=cg07912144	feature_id[3075].value > threshold=0.5578519105911255
node_29: feature_name=cg24407065	feature_id[388].value > threshold=0.5364363789558411

node_33: feature_name=cg15275017	feature_id[5069].value > threshold=0.4538573920726776
node_51: feature_name=cg07281938	feature_id[351].value > threshold=0.7506992518901825
node_73: feature_name=cg17524821	feature_id[5626].value > threshold=0.4504699558019638
node_75: feature_name=cg02973735	feature_id[1677].value > threshold=0.29383400082588196
node_77: feature_name=cg23345038	feature_id[7002].value > threshold=0.6255057156085968
node_79: feature_name=cg17537493	feature_id[5630].value > threshold=0.5825372636318207
node_85: feature_name=cg12948116	feature_id[4375].value <= threshold=0.5658791363239288
node_86: feature_name=cg06038180	feature_id[2529].value > threshold=0.43877437710762024
node_88: feature_name=cg15720017	feature_id[5175].value > threshold=0.7009969055652618
node_90: feature_name=cg13070215	feature_id[314].value <= threshold=0.14645694941282272
node_91: feature_name=cg01014262	feature_id[1082].value <= threshold=0.5737917125225067
node_92: feature_name=cg16863382	feature_id[5471].value > threshold=0.7200668156147003
node_102: feature_name=cg06633739	feature_id[2686].value > threshold=0.47115950286388397
node_126: feature_name=cg22946562	feature_id[744].value > threshold=0.6194809079170227
node_170: feature_name=cg12120430	feature_id[4185].value > threshold=0.5775820016860962
node_172: feature_name=cg02137970	feature_id[1411].value > threshold=0.6705857813358307
node_196: feature_name=cg03393445	feature_id[1807].value > threshold=0.4260125905275345
node_218: feature_name=cg23525541	feature_id[7049].value <= threshold=0.4485483318567276
node_219: feature_name=cg24964215	feature_id[7404].value <= threshold=0.04320882819592953
Class: osteoblastoma (OB)	
Rules_125	passed counts:1
node_0: feature_name=cg11915444	feature_id[4116].value > threshold=0.3402601182460785

node_10: feature_name=cg12109728	feature_id[4182].value > threshold=0.7068270146846771
node_20: feature_name=cg10844382	feature_id[3856].value > threshold=0.5988894104957581
node_24: feature_name=cg00631702	feature_id[943].value <= threshold=0.37470583617687225
node_25: feature_name=cg07912144	feature_id[3075].value > threshold=0.5578519105911255
node_29: feature_name=cg24407065	feature_id[388].value > threshold=0.5364363789558411
node_33: feature_name=cg15275017	feature_id[5069].value > threshold=0.4538573920726776
node_51: feature_name=cg07281938	feature_id[351].value > threshold=0.7506992518901825
node_73: feature_name=cg17524821	feature_id[5626].value > threshold=0.4504699558019638
node_75: feature_name=cg02973735	feature_id[1677].value > threshold=0.29383400082588196
node_77: feature_name=cg23345038	feature_id[7002].value > threshold=0.6255057156085968
node_79: feature_name=cg17537493	feature_id[5630].value > threshold=0.5825372636318207
node_85: feature_name=cg12948116	feature_id[4375].value <= threshold=0.5658791363239288
node_86: feature_name=cg06038180	feature_id[2529].value > threshold=0.43877437710762024
node_88: feature_name=cg15720017	feature_id[5175].value > threshold=0.7009969055652618
node_90: feature_name=cg13070215	feature_id[314].value <= threshold=0.14645694941282272
node_91: feature_name=cg01014262	feature_id[1082].value <= threshold=0.5737917125225067
node_92: feature_name=cg16863382	feature_id[5471].value > threshold=0.7200668156147003
node_102: feature_name=cg06633739	feature_id[2686].value > threshold=0.47115950286388397
node_126: feature_name=cg22946562	feature_id[744].value > threshold=0.6194809079170227
node_170: feature_name=cg12120430	feature_id[4185].value > threshold=0.5775820016860962
node_172: feature_name=cg02137970	feature_id[1411].value > threshold=0.6705857813358307
node_196: feature_name=cg03393445	feature_id[1807].value <= threshold=0.4260125905275345

node_197: feature_name=cg01297744	feature_id[1157].value > threshold=0.8005762100219727
node_211: feature_name=cg14401746	feature_id[4834].value > threshold=0.3708716481924057
node_213: feature_name=cg22060153	feature_id[6700].value <= threshold=0.7927507758140564
node_214: feature_name=cg06862949	feature_id[2759].value > threshold=0.7899251878261566
Class: extraskeletal myxoid chondrosarcoma (EMCS)	
Rules_126	passed counts:1
node_0: feature_name=cg11915444	feature_id[4116].value > threshold=0.3402601182460785
node_10: feature_name=cg12109728	feature_id[4182].value > threshold=0.7068270146846771
node_20: feature_name=cg10844382	feature_id[3856].value > threshold=0.5988894104957581
node_24: feature_name=cg00631702	feature_id[943].value <= threshold=0.37470583617687225
node_25: feature_name=cg07912144	feature_id[3075].value > threshold=0.5578519105911255
node_29: feature_name=cg24407065	feature_id[388].value > threshold=0.5364363789558411
node_33: feature_name=cg15275017	feature_id[5069].value > threshold=0.4538573920726776
node_51: feature_name=cg07281938	feature_id[351].value > threshold=0.7506992518901825
node_73: feature_name=cg17524821	feature_id[5626].value > threshold=0.4504699558019638
node_75: feature_name=cg02973735	feature_id[1677].value > threshold=0.29383400082588196
node_77: feature_name=cg23345038	feature_id[7002].value > threshold=0.6255057156085968
node_79: feature_name=cg17537493	feature_id[5630].value > threshold=0.5825372636318207
node_85: feature_name=cg12948116	feature_id[4375].value <= threshold=0.5658791363239288
node_86: feature_name=cg06038180	feature_id[2529].value > threshold=0.43877437710762024
node_88: feature_name=cg15720017	feature_id[5175].value > threshold=0.7009969055652618
node_90: feature_name=cg13070215	feature_id[314].value <= threshold=0.14645694941282272

node_91:<br>feature_name=cg01014262	feature_id[1082].value <=<br>threshold=0.5737917125225067
node_92:<br>feature_name=cg16863382	feature_id[5471].value ><br>threshold=0.7200668156147003
node_102:<br>feature_name=cg06633739	feature_id[2686].value ><br>threshold=0.47115950286388397
node_126:<br>feature_name=cg22946562	feature_id[744].value ><br>threshold=0.6194809079170227
node_170:<br>feature_name=cg12120430	feature_id[4185].value ><br>threshold=0.5775820016860962
node_172:<br>feature_name=cg02137970	feature_id[1411].value ><br>threshold=0.6705857813358307
node_196:<br>feature_name=cg03393445	feature_id[1807].value <=<br>threshold=0.4260125905275345
node_197:<br>feature_name=cg01297744	feature_id[1157].value ><br>threshold=0.8005762100219727
node_211:<br>feature_name=cg14401746	feature_id[4834].value ><br>threshold=0.3708716481924057
node_213:<br>feature_name=cg22060153	feature_id[6700].value <=<br>threshold=0.7927507758140564
node_214:<br>feature_name=cg06862949	feature_id[2759].value <=<br>threshold=0.7899251878261566
Class: angiosarcoma (AS)	
Rules_127	passed counts:1
node_0:<br>feature_name=cg11915444	feature_id[4116].value ><br>threshold=0.3402601182460785
node_10:<br>feature_name=cg12109728	feature_id[4182].value ><br>threshold=0.7068270146846771
node_20:<br>feature_name=cg10844382	feature_id[3856].value ><br>threshold=0.5988894104957581
node_24:<br>feature_name=cg00631702	feature_id[943].value <=<br>threshold=0.37470583617687225
node_25:<br>feature_name=cg07912144	feature_id[3075].value ><br>threshold=0.5578519105911255
node_29:<br>feature_name=cg24407065	feature_id[388].value ><br>threshold=0.5364363789558411
node_33:<br>feature_name=cg15275017	feature_id[5069].value ><br>threshold=0.4538573920726776
node_51:<br>feature_name=cg07281938	feature_id[351].value ><br>threshold=0.7506992518901825
node_73:<br>feature_name=cg17524821	feature_id[5626].value ><br>threshold=0.4504699558019638

node_75: feature_name=cg02973735	feature_id[1677].value > threshold=0.29383400082588196
node_77: feature_name=cg23345038	feature_id[7002].value > threshold=0.6255057156085968
node_79: feature_name=cg17537493	feature_id[5630].value > threshold=0.5825372636318207
node_85: feature_name=cg12948116	feature_id[4375].value <= threshold=0.5658791363239288
node_86: feature_name=cg06038180	feature_id[2529].value > threshold=0.43877437710762024
node_88: feature_name=cg15720017	feature_id[5175].value > threshold=0.7009969055652618
node_90: feature_name=cg13070215	feature_id[314].value <= threshold=0.14645694941282272
node_91: feature_name=cg01014262	feature_id[1082].value <= threshold=0.5737917125225067
node_92: feature_name=cg16863382	feature_id[5471].value > threshold=0.7200668156147003
node_102: feature_name=cg06633739	feature_id[2686].value > threshold=0.47115950286388397
node_126: feature_name=cg22946562	feature_id[744].value > threshold=0.6194809079170227
node_170: feature_name=cg12120430	feature_id[4185].value > threshold=0.5775820016860962
node_172: feature_name=cg02137970	feature_id[1411].value > threshold=0.6705857813358307
node_196: feature_name=cg03393445	feature_id[1807].value <= threshold=0.4260125905275345
node_197: feature_name=cg01297744	feature_id[1157].value <= threshold=0.8005762100219727
node_198: feature_name=cg08831077	feature_id[3310].value <= threshold=0.4210805594921112
node_199: feature_name=cg11526020	feature_id[4010].value <= threshold=0.8567313253879547
node_200: feature_name=cg02665399	feature_id[1584].value > threshold=0.8073965907096863
node_202: feature_name=cg02364610	feature_id[1491].value > threshold=0.058304985985159874
node_204: feature_name=cg16292933	feature_id[5314].value > threshold=0.6614532470703125
node_206: feature_name=cg14210790	feature_id[4774].value > threshold=0.6849235892295837
Class: well differentiated liposarcoma (WDLS)/dedifferentiated liposarcoma (DDLS)	

Rules_128	passed counts:1
node_0: feature_name=cg11915444	feature_id[4116].value > threshold=0.3402601182460785
node_10: feature_name=cg12109728	feature_id[4182].value > threshold=0.7068270146846771
node_20: feature_name=cg10844382	feature_id[3856].value > threshold=0.5988894104957581
node_24: feature_name=cg00631702	feature_id[943].value <= threshold=0.37470583617687225
node_25: feature_name=cg07912144	feature_id[3075].value > threshold=0.5578519105911255
node_29: feature_name=cg24407065	feature_id[388].value > threshold=0.5364363789558411
node_33: feature_name=cg15275017	feature_id[5069].value > threshold=0.4538573920726776
node_51: feature_name=cg07281938	feature_id[351].value > threshold=0.7506992518901825
node_73: feature_name=cg17524821	feature_id[5626].value > threshold=0.4504699558019638
node_75: feature_name=cg02973735	feature_id[1677].value > threshold=0.29383400082588196
node_77: feature_name=cg23345038	feature_id[7002].value > threshold=0.6255057156085968
node_79: feature_name=cg17537493	feature_id[5630].value > threshold=0.5825372636318207
node_85: feature_name=cg12948116	feature_id[4375].value <= threshold=0.5658791363239288
node_86: feature_name=cg06038180	feature_id[2529].value > threshold=0.43877437710762024
node_88: feature_name=cg15720017	feature_id[5175].value > threshold=0.7009969055652618
node_90: feature_name=cg13070215	feature_id[314].value <= threshold=0.14645694941282272
node_91: feature_name=cg01014262	feature_id[1082].value <= threshold=0.5737917125225067
node_92: feature_name=cg16863382	feature_id[5471].value > threshold=0.7200668156147003
node_102: feature_name=cg06633739	feature_id[2686].value > threshold=0.47115950286388397
node_126: feature_name=cg22946562	feature_id[744].value > threshold=0.6194809079170227

node_170: feature_name=cg12120430	feature_id[4185].value > threshold=0.5775820016860962
node_172: feature_name=cg02137970	feature_id[1411].value > threshold=0.6705857813358307
node_196: feature_name=cg03393445	feature_id[1807].value <= threshold=0.4260125905275345
node_197: feature_name=cg01297744	feature_id[1157].value <= threshold=0.8005762100219727
node_198: feature_name=cg08831077	feature_id[3310].value <= threshold=0.4210805594921112
node_199: feature_name=cg11526020	feature_id[4010].value <= threshold=0.8567313253879547
node_200: feature_name=cg02665399	feature_id[1584].value > threshold=0.8073965907096863
node_202: feature_name=cg02364610	feature_id[1491].value > threshold=0.058304985985159874
node_204: feature_name=cg16292933	feature_id[5314].value > threshold=0.6614532470703125
node_206: feature_name=cg14210790	feature_id[4774].value <= threshold=0.6849235892295837
Class: undifferentiated sarcoma (USARC)	
Rules_129	passed counts:1
node_0: feature_name=cg11915444	feature_id[4116].value > threshold=0.3402601182460785
node_10: feature_name=cg12109728	feature_id[4182].value > threshold=0.7068270146846771
node_20: feature_name=cg10844382	feature_id[3856].value > threshold=0.5988894104957581
node_24: feature_name=cg00631702	feature_id[943].value <= threshold=0.37470583617687225
node_25: feature_name=cg07912144	feature_id[3075].value > threshold=0.5578519105911255
node_29: feature_name=cg24407065	feature_id[388].value > threshold=0.5364363789558411
node_33: feature_name=cg15275017	feature_id[5069].value > threshold=0.4538573920726776
node_51: feature_name=cg07281938	feature_id[351].value > threshold=0.7506992518901825
node_73: feature_name=cg17524821	feature_id[5626].value > threshold=0.4504699558019638
node_75: feature_name=cg02973735	feature_id[1677].value > threshold=0.29383400082588196

node_77: feature_name=cg23345038	feature_id[7002].value > threshold=0.6255057156085968
node_79: feature_name=cg17537493	feature_id[5630].value > threshold=0.5825372636318207
node_85: feature_name=cg12948116	feature_id[4375].value <= threshold=0.5658791363239288
node_86: feature_name=cg06038180	feature_id[2529].value > threshold=0.43877437710762024
node_88: feature_name=cg15720017	feature_id[5175].value > threshold=0.7009969055652618
node_90: feature_name=cg13070215	feature_id[314].value <= threshold=0.14645694941282272
node_91: feature_name=cg01014262	feature_id[1082].value <= threshold=0.5737917125225067
node_92: feature_name=cg16863382	feature_id[5471].value > threshold=0.7200668156147003
node_102: feature_name=cg06633739	feature_id[2686].value > threshold=0.47115950286388397
node_126: feature_name=cg22946562	feature_id[744].value > threshold=0.6194809079170227
node_170: feature_name=cg12120430	feature_id[4185].value > threshold=0.5775820016860962
node_172: feature_name=cg02137970	feature_id[1411].value > threshold=0.6705857813358307
node_196: feature_name=cg03393445	feature_id[1807].value <= threshold=0.4260125905275345
node_197: feature_name=cg01297744	feature_id[1157].value <= threshold=0.8005762100219727
node_198: feature_name=cg08831077	feature_id[3310].value <= threshold=0.4210805594921112
node_199: feature_name=cg11526020	feature_id[4010].value <= threshold=0.8567313253879547
node_200: feature_name=cg02665399	feature_id[1584].value > threshold=0.8073965907096863
node_202: feature_name=cg02364610	feature_id[1491].value > threshold=0.058304985985159874
node_204: feature_name=cg16292933	feature_id[5314].value <= threshold=0.6614532470703125
Class: small blue round cell tumour (SBRCT)	
Rules_130	passed counts:1
node_0: feature_name=cg11915444	feature_id[4116].value > threshold=0.3402601182460785

node_10: feature_name=cg12109728	feature_id[4182].value > threshold=0.7068270146846771
node_20: feature_name=cg10844382	feature_id[3856].value > threshold=0.5988894104957581
node_24: feature_name=cg00631702	feature_id[943].value <= threshold=0.37470583617687225
node_25: feature_name=cg07912144	feature_id[3075].value > threshold=0.5578519105911255
node_29: feature_name=cg24407065	feature_id[388].value > threshold=0.5364363789558411
node_33: feature_name=cg15275017	feature_id[5069].value > threshold=0.4538573920726776
node_51: feature_name=cg07281938	feature_id[351].value > threshold=0.7506992518901825
node_73: feature_name=cg17524821	feature_id[5626].value > threshold=0.4504699558019638
node_75: feature_name=cg02973735	feature_id[1677].value > threshold=0.29383400082588196
node_77: feature_name=cg23345038	feature_id[7002].value > threshold=0.6255057156085968
node_79: feature_name=cg17537493	feature_id[5630].value > threshold=0.5825372636318207
node_85: feature_name=cg12948116	feature_id[4375].value <= threshold=0.5658791363239288
node_86: feature_name=cg06038180	feature_id[2529].value > threshold=0.43877437710762024
node_88: feature_name=cg15720017	feature_id[5175].value > threshold=0.7009969055652618
node_90: feature_name=cg13070215	feature_id[314].value <= threshold=0.14645694941282272
node_91: feature_name=cg01014262	feature_id[1082].value <= threshold=0.5737917125225067
node_92: feature_name=cg16863382	feature_id[5471].value > threshold=0.7200668156147003
node_102: feature_name=cg06633739	feature_id[2686].value > threshold=0.47115950286388397
node_126: feature_name=cg22946562	feature_id[744].value > threshold=0.6194809079170227
node_170: feature_name=cg12120430	feature_id[4185].value > threshold=0.5775820016860962
node_172: feature_name=cg02137970	feature_id[1411].value > threshold=0.6705857813358307
node_196: feature_name=cg03393445	feature_id[1807].value <= threshold=0.4260125905275345

node_197: feature_name=cg01297744	feature_id[1157].value <= threshold=0.8005762100219727
node_198: feature_name=cg08831077	feature_id[3310].value <= threshold=0.4210805594921112
node_199: feature_name=cg11526020	feature_id[4010].value <= threshold=0.8567313253879547
node_200: feature_name=cg02665399	feature_id[1584].value > threshold=0.8073965907096863
node_202: feature_name=cg02364610	feature_id[1491].value <= threshold=0.058304985985159874
Class: mesenchymal chondrosarcoma (CSA (MES))	
Rules_131	passed counts:1
node_0: feature_name=cg11915444	feature_id[4116].value > threshold=0.3402601182460785
node_10: feature_name=cg12109728	feature_id[4182].value > threshold=0.7068270146846771
node_20: feature_name=cg10844382	feature_id[3856].value > threshold=0.5988894104957581
node_24: feature_name=cg00631702	feature_id[943].value <= threshold=0.37470583617687225
node_25: feature_name=cg07912144	feature_id[3075].value > threshold=0.5578519105911255
node_29: feature_name=cg24407065	feature_id[388].value > threshold=0.5364363789558411
node_33: feature_name=cg15275017	feature_id[5069].value > threshold=0.4538573920726776
node_51: feature_name=cg07281938	feature_id[351].value > threshold=0.7506992518901825
node_73: feature_name=cg17524821	feature_id[5626].value > threshold=0.4504699558019638
node_75: feature_name=cg02973735	feature_id[1677].value > threshold=0.29383400082588196
node_77: feature_name=cg23345038	feature_id[7002].value > threshold=0.6255057156085968
node_79: feature_name=cg17537493	feature_id[5630].value > threshold=0.5825372636318207
node_85: feature_name=cg12948116	feature_id[4375].value <= threshold=0.5658791363239288
node_86: feature_name=cg06038180	feature_id[2529].value > threshold=0.43877437710762024
node_88: feature_name=cg15720017	feature_id[5175].value > threshold=0.7009969055652618

node_90: feature_name=cg13070215	feature_id[314].value <= threshold=0.14645694941282272
node_91: feature_name=cg01014262	feature_id[1082].value <= threshold=0.5737917125225067
node_92: feature_name=cg16863382	feature_id[5471].value > threshold=0.7200668156147003
node_102: feature_name=cg06633739	feature_id[2686].value > threshold=0.47115950286388397
node_126: feature_name=cg22946562	feature_id[744].value > threshold=0.6194809079170227
node_170: feature_name=cg12120430	feature_id[4185].value > threshold=0.5775820016860962
node_172: feature_name=cg02137970	feature_id[1411].value > threshold=0.6705857813358307
node_196: feature_name=cg03393445	feature_id[1807].value <= threshold=0.4260125905275345
node_197: feature_name=cg01297744	feature_id[1157].value <= threshold=0.8005762100219727
node_198: feature_name=cg08831077	feature_id[3310].value <= threshold=0.4210805594921112
node_199: feature_name=cg11526020	feature_id[4010].value <= threshold=0.8567313253879547
node_200: feature_name=cg02665399	feature_id[1584].value <= threshold=0.8073965907096863
Class: Ewing sarcoma (EWING)	
Rules_132	passed counts:1
node_0: feature_name=cg11915444	feature_id[4116].value > threshold=0.3402601182460785
node_10: feature_name=cg12109728	feature_id[4182].value > threshold=0.7068270146846771
node_20: feature_name=cg10844382	feature_id[3856].value > threshold=0.5988894104957581
node_24: feature_name=cg00631702	feature_id[943].value <= threshold=0.37470583617687225
node_25: feature_name=cg07912144	feature_id[3075].value > threshold=0.5578519105911255
node_29: feature_name=cg24407065	feature_id[388].value > threshold=0.5364363789558411
node_33: feature_name=cg15275017	feature_id[5069].value > threshold=0.4538573920726776
node_51: feature_name=cg07281938	feature_id[351].value > threshold=0.7506992518901825

node_73: feature_name=cg17524821	feature_id[5626].value > threshold=0.4504699558019638
node_75: feature_name=cg02973735	feature_id[1677].value > threshold=0.29383400082588196
node_77: feature_name=cg23345038	feature_id[7002].value > threshold=0.6255057156085968
node_79: feature_name=cg17537493	feature_id[5630].value > threshold=0.5825372636318207
node_85: feature_name=cg12948116	feature_id[4375].value <= threshold=0.5658791363239288
node_86: feature_name=cg06038180	feature_id[2529].value > threshold=0.43877437710762024
node_88: feature_name=cg15720017	feature_id[5175].value > threshold=0.7009969055652618
node_90: feature_name=cg13070215	feature_id[314].value <= threshold=0.14645694941282272
node_91: feature_name=cg01014262	feature_id[1082].value <= threshold=0.5737917125225067
node_92: feature_name=cg16863382	feature_id[5471].value > threshold=0.7200668156147003
node_102: feature_name=cg06633739	feature_id[2686].value > threshold=0.47115950286388397
node_126: feature_name=cg22946562	feature_id[744].value > threshold=0.6194809079170227
node_170: feature_name=cg12120430	feature_id[4185].value > threshold=0.5775820016860962
node_172: feature_name=cg02137970	feature_id[1411].value <= threshold=0.6705857813358307
node_173: feature_name=cg16185457	feature_id[5282].value > threshold=0.5055856853723526
node_183: feature_name=cg04993279	feature_id[2243].value > threshold=0.46021124720573425
node_191: feature_name=cg09043524	feature_id[3372].value <= threshold=0.7900851368904114
node_192: feature_name=cg06422467	feature_id[2625].value > threshold=0.22639098018407822
Class: inflammatory myofibroblastic tumour (IMT)	
Rules_133	passed counts:1
node_0: feature_name=cg11915444	feature_id[4116].value > threshold=0.3402601182460785
node_10: feature_name=cg12109728	feature_id[4182].value > threshold=0.7068270146846771

node_20:<br>feature_name=cg10844382	feature_id[3856].value ><br>threshold=0.5988894104957581
node_24:<br>feature_name=cg00631702	feature_id[943].value <=<br>threshold=0.37470583617687225
node_25:<br>feature_name=cg07912144	feature_id[3075].value ><br>threshold=0.5578519105911255
node_29:<br>feature_name=cg24407065	feature_id[388].value ><br>threshold=0.5364363789558411
node_33:<br>feature_name=cg15275017	feature_id[5069].value ><br>threshold=0.4538573920726776
node_51:<br>feature_name=cg07281938	feature_id[351].value ><br>threshold=0.7506992518901825
node_73:<br>feature_name=cg17524821	feature_id[5626].value ><br>threshold=0.4504699558019638
node_75:<br>feature_name=cg02973735	feature_id[1677].value ><br>threshold=0.29383400082588196
node_77:<br>feature_name=cg23345038	feature_id[7002].value ><br>threshold=0.6255057156085968
node_79:<br>feature_name=cg17537493	feature_id[5630].value ><br>threshold=0.5825372636318207
node_85:<br>feature_name=cg12948116	feature_id[4375].value <=<br>threshold=0.5658791363239288
node_86:<br>feature_name=cg06038180	feature_id[2529].value ><br>threshold=0.43877437710762024
node_88:<br>feature_name=cg15720017	feature_id[5175].value ><br>threshold=0.7009969055652618
node_90:<br>feature_name=cg13070215	feature_id[314].value <=<br>threshold=0.14645694941282272
node_91:<br>feature_name=cg01014262	feature_id[1082].value <=<br>threshold=0.5737917125225067
node_92:<br>feature_name=cg16863382	feature_id[5471].value ><br>threshold=0.7200668156147003
node_102:<br>feature_name=cg06633739	feature_id[2686].value ><br>threshold=0.47115950286388397
node_126:<br>feature_name=cg22946562	feature_id[744].value ><br>threshold=0.6194809079170227
node_170:<br>feature_name=cg12120430	feature_id[4185].value ><br>threshold=0.5775820016860962
node_172:<br>feature_name=cg02137970	feature_id[1411].value <=<br>threshold=0.6705857813358307
node_173:<br>feature_name=cg16185457	feature_id[5282].value ><br>threshold=0.5055856853723526
node_183:<br>feature_name=cg04993279	feature_id[2243].value ><br>threshold=0.46021124720573425

node_191: feature_name=cg09043524	feature_id[3372].value <= threshold=0.7900851368904114
node_192: feature_name=cg06422467	feature_id[2625].value <= threshold=0.22639098018407822
Class: sarcoma (SARC)	
Rules_134	passed counts:1
node_0: feature_name=cg11915444	feature_id[4116].value > threshold=0.3402601182460785
node_10: feature_name=cg12109728	feature_id[4182].value > threshold=0.7068270146846771
node_20: feature_name=cg10844382	feature_id[3856].value > threshold=0.5988894104957581
node_24: feature_name=cg00631702	feature_id[943].value <= threshold=0.37470583617687225
node_25: feature_name=cg07912144	feature_id[3075].value > threshold=0.5578519105911255
node_29: feature_name=cg24407065	feature_id[388].value > threshold=0.5364363789558411
node_33: feature_name=cg15275017	feature_id[5069].value > threshold=0.4538573920726776
node_51: feature_name=cg07281938	feature_id[351].value > threshold=0.7506992518901825
node_73: feature_name=cg17524821	feature_id[5626].value > threshold=0.4504699558019638
node_75: feature_name=cg02973735	feature_id[1677].value > threshold=0.29383400082588196
node_77: feature_name=cg23345038	feature_id[7002].value > threshold=0.6255057156085968
node_79: feature_name=cg17537493	feature_id[5630].value > threshold=0.5825372636318207
node_85: feature_name=cg12948116	feature_id[4375].value <= threshold=0.5658791363239288
node_86: feature_name=cg06038180	feature_id[2529].value > threshold=0.43877437710762024
node_88: feature_name=cg15720017	feature_id[5175].value > threshold=0.7009969055652618
node_90: feature_name=cg13070215	feature_id[314].value <= threshold=0.14645694941282272
node_91: feature_name=cg01014262	feature_id[1082].value <= threshold=0.5737917125225067
node_92: feature_name=cg16863382	feature_id[5471].value > threshold=0.7200668156147003

node_102: feature_name=cg06633739	feature_id[2686].value > threshold=0.47115950286388397
node_126: feature_name=cg22946562	feature_id[744].value > threshold=0.6194809079170227
node_170: feature_name=cg12120430	feature_id[4185].value > threshold=0.5775820016860962
node_172: feature_name=cg02137970	feature_id[1411].value <= threshold=0.6705857813358307
node_173: feature_name=cg16185457	feature_id[5282].value > threshold=0.5055856853723526
node_183: feature_name=cg04993279	feature_id[2243].value <= threshold=0.46021124720573425
node_184: feature_name=cg04655520	feature_id[2141].value <= threshold=0.08191334456205368
node_185: feature_name=cg11103999	feature_id[3923].value > threshold=0.8651433885097504
node_187: feature_name=cg00044665	feature_id[769].value > threshold=0.4028041586279869
Class: leiomyosarcoma (LMS)	
Rules_135	passed counts:1
node_0: feature_name=cg11915444	feature_id[4116].value > threshold=0.3402601182460785
node_10: feature_name=cg12109728	feature_id[4182].value > threshold=0.7068270146846771
node_20: feature_name=cg10844382	feature_id[3856].value > threshold=0.5988894104957581
node_24: feature_name=cg00631702	feature_id[943].value <= threshold=0.37470583617687225
node_25: feature_name=cg07912144	feature_id[3075].value > threshold=0.5578519105911255
node_29: feature_name=cg24407065	feature_id[388].value > threshold=0.5364363789558411
node_33: feature_name=cg15275017	feature_id[5069].value > threshold=0.4538573920726776
node_51: feature_name=cg07281938	feature_id[351].value > threshold=0.7506992518901825
node_73: feature_name=cg17524821	feature_id[5626].value > threshold=0.4504699558019638
node_75: feature_name=cg02973735	feature_id[1677].value > threshold=0.29383400082588196
node_77: feature_name=cg23345038	feature_id[7002].value > threshold=0.6255057156085968

node_79: feature_name=cg17537493	feature_id[5630].value > threshold=0.5825372636318207
node_85: feature_name=cg12948116	feature_id[4375].value <= threshold=0.5658791363239288
node_86: feature_name=cg06038180	feature_id[2529].value > threshold=0.43877437710762024
node_88: feature_name=cg15720017	feature_id[5175].value > threshold=0.7009969055652618
node_90: feature_name=cg13070215	feature_id[314].value <= threshold=0.14645694941282272
node_91: feature_name=cg01014262	feature_id[1082].value <= threshold=0.5737917125225067
node_92: feature_name=cg16863382	feature_id[5471].value > threshold=0.7200668156147003
node_102: feature_name=cg06633739	feature_id[2686].value > threshold=0.47115950286388397
node_126: feature_name=cg22946562	feature_id[744].value > threshold=0.6194809079170227
node_170: feature_name=cg12120430	feature_id[4185].value > threshold=0.5775820016860962
node_172: feature_name=cg02137970	feature_id[1411].value <= threshold=0.6705857813358307
node_173: feature_name=cg16185457	feature_id[5282].value > threshold=0.5055856853723526
node_183: feature_name=cg04993279	feature_id[2243].value <= threshold=0.46021124720573425
node_184: feature_name=cg04655520	feature_id[2141].value <= threshold=0.08191334456205368
node_185: feature_name=cg11103999	feature_id[3923].value > threshold=0.8651433885097504
node_187: feature_name=cg00044665	feature_id[769].value <= threshold=0.4028041586279869
Class: high-grade endometrial stromal sarcoma (ESS (HG))	
Rules_136	passed counts:1
node_0: feature_name=cg11915444	feature_id[4116].value > threshold=0.3402601182460785
node_10: feature_name=cg12109728	feature_id[4182].value > threshold=0.7068270146846771
node_20: feature_name=cg10844382	feature_id[3856].value > threshold=0.5988894104957581
node_24: feature_name=cg00631702	feature_id[943].value <= threshold=0.37470583617687225

node_25: feature_name=cg07912144	feature_id[3075].value > threshold=0.5578519105911255
node_29: feature_name=cg24407065	feature_id[388].value > threshold=0.5364363789558411
node_33: feature_name=cg15275017	feature_id[5069].value > threshold=0.4538573920726776
node_51: feature_name=cg07281938	feature_id[351].value > threshold=0.7506992518901825
node_73: feature_name=cg17524821	feature_id[5626].value > threshold=0.4504699558019638
node_75: feature_name=cg02973735	feature_id[1677].value > threshold=0.29383400082588196
node_77: feature_name=cg23345038	feature_id[7002].value > threshold=0.6255057156085968
node_79: feature_name=cg17537493	feature_id[5630].value > threshold=0.5825372636318207
node_85: feature_name=cg12948116	feature_id[4375].value <= threshold=0.5658791363239288
node_86: feature_name=cg06038180	feature_id[2529].value > threshold=0.43877437710762024
node_88: feature_name=cg15720017	feature_id[5175].value > threshold=0.7009969055652618
node_90: feature_name=cg13070215	feature_id[314].value <= threshold=0.14645694941282272
node_91: feature_name=cg01014262	feature_id[1082].value <= threshold=0.5737917125225067
node_92: feature_name=cg16863382	feature_id[5471].value > threshold=0.7200668156147003
node_102: feature_name=cg06633739	feature_id[2686].value > threshold=0.47115950286388397
node_126: feature_name=cg22946562	feature_id[744].value > threshold=0.6194809079170227
node_170: feature_name=cg12120430	feature_id[4185].value > threshold=0.5775820016860962
node_172: feature_name=cg02137970	feature_id[1411].value <= threshold=0.6705857813358307
node_173: feature_name=cg16185457	feature_id[5282].value <= threshold=0.5055856853723526
node_174: feature_name=cg09040552	feature_id[3370].value > threshold=0.596051037311554
node_176: feature_name=cg12064373	feature_id[4162].value <= threshold=0.10608945414423943
node_177: feature_name=cg04874782	feature_id[2203].value <= threshold=0.24745557457208633

node_178: feature_name=cg10963218	feature_id[3883].value > threshold=0.6643033921718597
Class: malignant peripheral nerve sheath tumour (MPNST)	
Rules_137	passed counts:1
node_0: feature_name=cg11915444	feature_id[4116].value > threshold=0.3402601182460785
node_10: feature_name=cg12109728	feature_id[4182].value > threshold=0.7068270146846771
node_20: feature_name=cg10844382	feature_id[3856].value > threshold=0.5988894104957581
node_24: feature_name=cg00631702	feature_id[943].value <= threshold=0.37470583617687225
node_25: feature_name=cg07912144	feature_id[3075].value > threshold=0.5578519105911255
node_29: feature_name=cg24407065	feature_id[388].value > threshold=0.5364363789558411
node_33: feature_name=cg15275017	feature_id[5069].value > threshold=0.4538573920726776
node_51: feature_name=cg07281938	feature_id[351].value > threshold=0.7506992518901825
node_73: feature_name=cg17524821	feature_id[5626].value > threshold=0.4504699558019638
node_75: feature_name=cg02973735	feature_id[1677].value > threshold=0.29383400082588196
node_77: feature_name=cg23345038	feature_id[7002].value > threshold=0.6255057156085968
node_79: feature_name=cg17537493	feature_id[5630].value > threshold=0.5825372636318207
node_85: feature_name=cg12948116	feature_id[4375].value <= threshold=0.5658791363239288
node_86: feature_name=cg06038180	feature_id[2529].value > threshold=0.43877437710762024
node_88: feature_name=cg15720017	feature_id[5175].value > threshold=0.7009969055652618
node_90: feature_name=cg13070215	feature_id[314].value <= threshold=0.14645694941282272
node_91: feature_name=cg01014262	feature_id[1082].value <= threshold=0.5737917125225067
node_92: feature_name=cg16863382	feature_id[5471].value > threshold=0.7200668156147003
node_102: feature_name=cg06633739	feature_id[2686].value > threshold=0.47115950286388397

node_126: feature_name=cg22946562	feature_id[744].value <= threshold=0.6194809079170227
node_127: feature_name=cg23920016	feature_id[7139].value > threshold=0.7449243068695068
node_151: feature_name=cg21617218	feature_id[6608].value > threshold=0.8795748353004456
node_153: feature_name=cg01663970	feature_id[1273].value > threshold=0.8442256152629852
node_155: feature_name=cg00686915	feature_id[965].value > threshold=0.20000632852315903
node_157: feature_name=cg11628781	feature_id[4033].value <= threshold=0.9032271206378937
node_158: feature_name=cg20173014	feature_id[6271].value > threshold=0.8712336122989655
node_160: feature_name=cg04961225	feature_id[2231].value > threshold=0.6663023084402084
node_162: feature_name=cg14340089	feature_id[4825].value > threshold=0.6815699338912964
node_164: feature_name=cg04576568	feature_id[2126].value > threshold=0.5339387804269791
node_166: feature_name=cg25650076	feature_id[389].value > threshold=0.45621342677623034
Class: giant cell tumour of bone (GCTB)	
Rules_138	passed counts:1
node_0: feature_name=cg11915444	feature_id[4116].value > threshold=0.3402601182460785
node_10: feature_name=cg12109728	feature_id[4182].value > threshold=0.7068270146846771
node_20: feature_name=cg10844382	feature_id[3856].value > threshold=0.5988894104957581
node_24: feature_name=cg00631702	feature_id[943].value <= threshold=0.37470583617687225
node_25: feature_name=cg07912144	feature_id[3075].value > threshold=0.5578519105911255
node_29: feature_name=cg24407065	feature_id[388].value > threshold=0.5364363789558411
node_33: feature_name=cg15275017	feature_id[5069].value > threshold=0.4538573920726776
node_51: feature_name=cg07281938	feature_id[351].value > threshold=0.7506992518901825
node_73: feature_name=cg17524821	feature_id[5626].value > threshold=0.4504699558019638

node_75: feature_name=cg02973735	feature_id[1677].value > threshold=0.29383400082588196
node_77: feature_name=cg23345038	feature_id[7002].value > threshold=0.6255057156085968
node_79: feature_name=cg17537493	feature_id[5630].value > threshold=0.5825372636318207
node_85: feature_name=cg12948116	feature_id[4375].value <= threshold=0.5658791363239288
node_86: feature_name=cg06038180	feature_id[2529].value > threshold=0.43877437710762024
node_88: feature_name=cg15720017	feature_id[5175].value > threshold=0.7009969055652618
node_90: feature_name=cg13070215	feature_id[314].value <= threshold=0.14645694941282272
node_91: feature_name=cg01014262	feature_id[1082].value <= threshold=0.5737917125225067
node_92: feature_name=cg16863382	feature_id[5471].value > threshold=0.7200668156147003
node_102: feature_name=cg06633739	feature_id[2686].value > threshold=0.47115950286388397
node_126: feature_name=cg22946562	feature_id[744].value <= threshold=0.6194809079170227
node_127: feature_name=cg23920016	feature_id[7139].value > threshold=0.7449243068695068
node_151: feature_name=cg21617218	feature_id[6608].value > threshold=0.8795748353004456
node_153: feature_name=cg01663970	feature_id[1273].value > threshold=0.8442256152629852
node_155: feature_name=cg00686915	feature_id[965].value > threshold=0.20000632852315903
node_157: feature_name=cg11628781	feature_id[4033].value <= threshold=0.9032271206378937
node_158: feature_name=cg20173014	feature_id[6271].value > threshold=0.8712336122989655
node_160: feature_name=cg04961225	feature_id[2231].value > threshold=0.6663023084402084
node_162: feature_name=cg14340089	feature_id[4825].value > threshold=0.6815699338912964
node_164: feature_name=cg04576568	feature_id[2126].value > threshold=0.5339387804269791
node_166: feature_name=cg25650076	feature_id[389].value <= threshold=0.45621342677623034
Class: leiomyosarcoma (LMS)	

Rules_139	passed counts:1
node_0: feature_name=cg11915444	feature_id[4116].value > threshold=0.3402601182460785
node_10: feature_name=cg12109728	feature_id[4182].value > threshold=0.7068270146846771
node_20: feature_name=cg10844382	feature_id[3856].value > threshold=0.5988894104957581
node_24: feature_name=cg00631702	feature_id[943].value <= threshold=0.37470583617687225
node_25: feature_name=cg07912144	feature_id[3075].value > threshold=0.5578519105911255
node_29: feature_name=cg24407065	feature_id[388].value > threshold=0.5364363789558411
node_33: feature_name=cg15275017	feature_id[5069].value > threshold=0.4538573920726776
node_51: feature_name=cg07281938	feature_id[351].value > threshold=0.7506992518901825
node_73: feature_name=cg17524821	feature_id[5626].value > threshold=0.4504699558019638
node_75: feature_name=cg02973735	feature_id[1677].value > threshold=0.29383400082588196
node_77: feature_name=cg23345038	feature_id[7002].value > threshold=0.6255057156085968
node_79: feature_name=cg17537493	feature_id[5630].value > threshold=0.5825372636318207
node_85: feature_name=cg12948116	feature_id[4375].value <= threshold=0.5658791363239288
node_86: feature_name=cg06038180	feature_id[2529].value > threshold=0.43877437710762024
node_88: feature_name=cg15720017	feature_id[5175].value > threshold=0.7009969055652618
node_90: feature_name=cg13070215	feature_id[314].value <= threshold=0.14645694941282272
node_91: feature_name=cg01014262	feature_id[1082].value <= threshold=0.5737917125225067
node_92: feature_name=cg16863382	feature_id[5471].value > threshold=0.7200668156147003
node_102: feature_name=cg06633739	feature_id[2686].value > threshold=0.47115950286388397
node_126: feature_name=cg22946562	feature_id[744].value <= threshold=0.6194809079170227

node_127: feature_name=cg23920016	feature_id[7139].value > threshold=0.7449243068695068
node_151: feature_name=cg21617218	feature_id[6608].value > threshold=0.8795748353004456
node_153: feature_name=cg01663970	feature_id[1273].value > threshold=0.8442256152629852
node_155: feature_name=cg00686915	feature_id[965].value > threshold=0.20000632852315903
node_157: feature_name=cg11628781	feature_id[4033].value <= threshold=0.9032271206378937
node_158: feature_name=cg20173014	feature_id[6271].value > threshold=0.8712336122989655
node_160: feature_name=cg04961225	feature_id[2231].value > threshold=0.6663023084402084
node_162: feature_name=cg14340089	feature_id[4825].value > threshold=0.6815699338912964
node_164: feature_name=cg04576568	feature_id[2126].value <= threshold=0.5339387804269791
Class: well differentiated liposarcoma (WDLS)/dedifferentiated liposarcoma (DDLS)	
Rules_140	passed counts:1
node_0: feature_name=cg11915444	feature_id[4116].value > threshold=0.3402601182460785
node_10: feature_name=cg12109728	feature_id[4182].value > threshold=0.7068270146846771
node_20: feature_name=cg10844382	feature_id[3856].value > threshold=0.5988894104957581
node_24: feature_name=cg00631702	feature_id[943].value <= threshold=0.37470583617687225
node_25: feature_name=cg07912144	feature_id[3075].value > threshold=0.5578519105911255
node_29: feature_name=cg24407065	feature_id[388].value > threshold=0.5364363789558411
node_33: feature_name=cg15275017	feature_id[5069].value > threshold=0.4538573920726776
node_51: feature_name=cg07281938	feature_id[351].value > threshold=0.7506992518901825
node_73: feature_name=cg17524821	feature_id[5626].value > threshold=0.4504699558019638
node_75: feature_name=cg02973735	feature_id[1677].value > threshold=0.29383400082588196
node_77: feature_name=cg23345038	feature_id[7002].value > threshold=0.6255057156085968

node_79: feature_name=cg17537493	feature_id[5630].value > threshold=0.5825372636318207
node_85: feature_name=cg12948116	feature_id[4375].value <= threshold=0.5658791363239288
node_86: feature_name=cg06038180	feature_id[2529].value > threshold=0.43877437710762024
node_88: feature_name=cg15720017	feature_id[5175].value > threshold=0.7009969055652618
node_90: feature_name=cg13070215	feature_id[314].value <= threshold=0.14645694941282272
node_91: feature_name=cg01014262	feature_id[1082].value <= threshold=0.5737917125225067
node_92: feature_name=cg16863382	feature_id[5471].value > threshold=0.7200668156147003
node_102: feature_name=cg06633739	feature_id[2686].value > threshold=0.47115950286388397
node_126: feature_name=cg22946562	feature_id[744].value <= threshold=0.6194809079170227
node_127: feature_name=cg23920016	feature_id[7139].value > threshold=0.7449243068695068
node_151: feature_name=cg21617218	feature_id[6608].value > threshold=0.8795748353004456
node_153: feature_name=cg01663970	feature_id[1273].value > threshold=0.8442256152629852
node_155: feature_name=cg00686915	feature_id[965].value > threshold=0.20000632852315903
node_157: feature_name=cg11628781	feature_id[4033].value <= threshold=0.9032271206378937
node_158: feature_name=cg20173014	feature_id[6271].value > threshold=0.8712336122989655
node_160: feature_name=cg04961225	feature_id[2231].value > threshold=0.6663023084402084
node_162: feature_name=cg14340089	feature_id[4825].value <= threshold=0.6815699338912964
Class: Ewing sarcoma (EWING)	
Rules_141	passed counts:1
node_0: feature_name=cg11915444	feature_id[4116].value > threshold=0.3402601182460785
node_10: feature_name=cg12109728	feature_id[4182].value > threshold=0.7068270146846771
node_20: feature_name=cg10844382	feature_id[3856].value > threshold=0.5988894104957581

node_24: feature_name=cg00631702	feature_id[943].value <= threshold=0.37470583617687225
node_25: feature_name=cg07912144	feature_id[3075].value > threshold=0.5578519105911255
node_29: feature_name=cg24407065	feature_id[388].value > threshold=0.5364363789558411
node_33: feature_name=cg15275017	feature_id[5069].value > threshold=0.4538573920726776
node_51: feature_name=cg07281938	feature_id[351].value > threshold=0.7506992518901825
node_73: feature_name=cg17524821	feature_id[5626].value > threshold=0.4504699558019638
node_75: feature_name=cg02973735	feature_id[1677].value > threshold=0.29383400082588196
node_77: feature_name=cg23345038	feature_id[7002].value > threshold=0.6255057156085968
node_79: feature_name=cg17537493	feature_id[5630].value > threshold=0.5825372636318207
node_85: feature_name=cg12948116	feature_id[4375].value <= threshold=0.5658791363239288
node_86: feature_name=cg06038180	feature_id[2529].value > threshold=0.43877437710762024
node_88: feature_name=cg15720017	feature_id[5175].value > threshold=0.7009969055652618
node_90: feature_name=cg13070215	feature_id[314].value <= threshold=0.14645694941282272
node_91: feature_name=cg01014262	feature_id[1082].value <= threshold=0.5737917125225067
node_92: feature_name=cg16863382	feature_id[5471].value > threshold=0.7200668156147003
node_102: feature_name=cg06633739	feature_id[2686].value > threshold=0.47115950286388397
node_126: feature_name=cg22946562	feature_id[744].value <= threshold=0.6194809079170227
node_127: feature_name=cg23920016	feature_id[7139].value > threshold=0.7449243068695068
node_151: feature_name=cg21617218	feature_id[6608].value > threshold=0.8795748353004456
node_153: feature_name=cg01663970	feature_id[1273].value > threshold=0.8442256152629852
node_155: feature_name=cg00686915	feature_id[965].value > threshold=0.20000632852315903
node_157: feature_name=cg11628781	feature_id[4033].value <= threshold=0.9032271206378937

node_158: feature_name=cg20173014	feature_id[6271].value > threshold=0.8712336122989655
node_160: feature_name=cg04961225	feature_id[2231].value <= threshold=0.6663023084402084
Class: atypical fibroxanthoma (AFX)/pleomorphic dermal sarcoma (PDS)	
Rules_142	passed counts:1
node_0: feature_name=cg11915444	feature_id[4116].value > threshold=0.3402601182460785
node_10: feature_name=cg12109728	feature_id[4182].value > threshold=0.7068270146846771
node_20: feature_name=cg10844382	feature_id[3856].value > threshold=0.5988894104957581
node_24: feature_name=cg00631702	feature_id[943].value <= threshold=0.37470583617687225
node_25: feature_name=cg07912144	feature_id[3075].value > threshold=0.5578519105911255
node_29: feature_name=cg24407065	feature_id[388].value > threshold=0.5364363789558411
node_33: feature_name=cg15275017	feature_id[5069].value > threshold=0.4538573920726776
node_51: feature_name=cg07281938	feature_id[351].value > threshold=0.7506992518901825
node_73: feature_name=cg17524821	feature_id[5626].value > threshold=0.4504699558019638
node_75: feature_name=cg02973735	feature_id[1677].value > threshold=0.29383400082588196
node_77: feature_name=cg23345038	feature_id[7002].value > threshold=0.6255057156085968
node_79: feature_name=cg17537493	feature_id[5630].value > threshold=0.5825372636318207
node_85: feature_name=cg12948116	feature_id[4375].value <= threshold=0.5658791363239288
node_86: feature_name=cg06038180	feature_id[2529].value > threshold=0.43877437710762024
node_88: feature_name=cg15720017	feature_id[5175].value > threshold=0.7009969055652618
node_90: feature_name=cg13070215	feature_id[314].value <= threshold=0.14645694941282272
node_91: feature_name=cg01014262	feature_id[1082].value <= threshold=0.5737917125225067
node_92: feature_name=cg16863382	feature_id[5471].value > threshold=0.7200668156147003

node_102: feature_name=cg06633739	feature_id[2686].value > threshold=0.47115950286388397
node_126: feature_name=cg22946562	feature_id[744].value <= threshold=0.6194809079170227
node_127: feature_name=cg23920016	feature_id[7139].value > threshold=0.7449243068695068
node_151: feature_name=cg21617218	feature_id[6608].value > threshold=0.8795748353004456
node_153: feature_name=cg01663970	feature_id[1273].value > threshold=0.8442256152629852
node_155: feature_name=cg00686915	feature_id[965].value > threshold=0.20000632852315903
node_157: feature_name=cg11628781	feature_id[4033].value <= threshold=0.9032271206378937
node_158: feature_name=cg20173014	feature_id[6271].value <= threshold=0.8712336122989655
Class: gastrointestinal stromal tumour (GIST)	
Rules_143	passed counts:1
node_0: feature_name=cg11915444	feature_id[4116].value > threshold=0.3402601182460785
node_10: feature_name=cg12109728	feature_id[4182].value > threshold=0.7068270146846771
node_20: feature_name=cg10844382	feature_id[3856].value > threshold=0.5988894104957581
node_24: feature_name=cg00631702	feature_id[943].value <= threshold=0.37470583617687225
node_25: feature_name=cg07912144	feature_id[3075].value > threshold=0.5578519105911255
node_29: feature_name=cg24407065	feature_id[388].value > threshold=0.5364363789558411
node_33: feature_name=cg15275017	feature_id[5069].value > threshold=0.4538573920726776
node_51: feature_name=cg07281938	feature_id[351].value > threshold=0.7506992518901825
node_73: feature_name=cg17524821	feature_id[5626].value > threshold=0.4504699558019638
node_75: feature_name=cg02973735	feature_id[1677].value > threshold=0.29383400082588196
node_77: feature_name=cg23345038	feature_id[7002].value > threshold=0.6255057156085968
node_79: feature_name=cg17537493	feature_id[5630].value > threshold=0.5825372636318207

node_85: feature_name=cg12948116	feature_id[4375].value <= threshold=0.5658791363239288
node_86: feature_name=cg06038180	feature_id[2529].value > threshold=0.43877437710762024
node_88: feature_name=cg15720017	feature_id[5175].value > threshold=0.7009969055652618
node_90: feature_name=cg13070215	feature_id[314].value <= threshold=0.14645694941282272
node_91: feature_name=cg01014262	feature_id[1082].value <= threshold=0.5737917125225067
node_92: feature_name=cg16863382	feature_id[5471].value > threshold=0.7200668156147003
node_102: feature_name=cg06633739	feature_id[2686].value > threshold=0.47115950286388397
node_126: feature_name=cg22946562	feature_id[744].value <= threshold=0.6194809079170227
node_127: feature_name=cg23920016	feature_id[7139].value <= threshold=0.7449243068695068
node_128: feature_name=cg16521032	feature_id[5379].value > threshold=0.8948074877262115
node_136: feature_name=cg22345063	feature_id[6755].value > threshold=0.38597847521305084
node_144: feature_name=cg05650238	feature_id[2427].value <= threshold=0.7126822769641876
node_145: feature_name=cg20757748	feature_id[6410].value > threshold=0.3033527359366417
node_147: feature_name=cg06586578	feature_id[2671].value > threshold=0.83711838722229
Class: leiomyosarcoma (LMS)	
Rules_144	passed counts:1
node_0: feature_name=cg11915444	feature_id[4116].value > threshold=0.3402601182460785
node_10: feature_name=cg12109728	feature_id[4182].value > threshold=0.7068270146846771
node_20: feature_name=cg10844382	feature_id[3856].value > threshold=0.5988894104957581
node_24: feature_name=cg00631702	feature_id[943].value <= threshold=0.37470583617687225
node_25: feature_name=cg07912144	feature_id[3075].value > threshold=0.5578519105911255
node_29: feature_name=cg24407065	feature_id[388].value > threshold=0.5364363789558411

node_33: feature_name=cg15275017	feature_id[5069].value > threshold=0.4538573920726776
node_51: feature_name=cg07281938	feature_id[351].value > threshold=0.7506992518901825
node_73: feature_name=cg17524821	feature_id[5626].value > threshold=0.4504699558019638
node_75: feature_name=cg02973735	feature_id[1677].value > threshold=0.29383400082588196
node_77: feature_name=cg23345038	feature_id[7002].value > threshold=0.6255057156085968
node_79: feature_name=cg17537493	feature_id[5630].value > threshold=0.5825372636318207
node_85: feature_name=cg12948116	feature_id[4375].value <= threshold=0.5658791363239288
node_86: feature_name=cg06038180	feature_id[2529].value > threshold=0.43877437710762024
node_88: feature_name=cg15720017	feature_id[5175].value > threshold=0.7009969055652618
node_90: feature_name=cg13070215	feature_id[314].value <= threshold=0.14645694941282272
node_91: feature_name=cg01014262	feature_id[1082].value <= threshold=0.5737917125225067
node_92: feature_name=cg16863382	feature_id[5471].value > threshold=0.7200668156147003
node_102: feature_name=cg06633739	feature_id[2686].value > threshold=0.47115950286388397
node_126: feature_name=cg22946562	feature_id[744].value <= threshold=0.6194809079170227
node_127: feature_name=cg23920016	feature_id[7139].value <= threshold=0.7449243068695068
node_128: feature_name=cg16521032	feature_id[5379].value > threshold=0.8948074877262115
node_136: feature_name=cg22345063	feature_id[6755].value > threshold=0.38597847521305084
node_144: feature_name=cg05650238	feature_id[2427].value <= threshold=0.7126822769641876
node_145: feature_name=cg20757748	feature_id[6410].value > threshold=0.3033527359366417
node_147: feature_name=cg06586578	feature_id[2671].value <= threshold=0.83711838722229
Class: giant cell tumour of bone (GCTB)	
Rules_145	passed counts:1

node_0: feature_name=cg11915444	feature_id[4116].value > threshold=0.3402601182460785
node_10: feature_name=cg12109728	feature_id[4182].value > threshold=0.7068270146846771
node_20: feature_name=cg10844382	feature_id[3856].value > threshold=0.5988894104957581
node_24: feature_name=cg00631702	feature_id[943].value <= threshold=0.37470583617687225
node_25: feature_name=cg07912144	feature_id[3075].value > threshold=0.5578519105911255
node_29: feature_name=cg24407065	feature_id[388].value > threshold=0.5364363789558411
node_33: feature_name=cg15275017	feature_id[5069].value > threshold=0.4538573920726776
node_51: feature_name=cg07281938	feature_id[351].value > threshold=0.7506992518901825
node_73: feature_name=cg17524821	feature_id[5626].value > threshold=0.4504699558019638
node_75: feature_name=cg02973735	feature_id[1677].value > threshold=0.29383400082588196
node_77: feature_name=cg23345038	feature_id[7002].value > threshold=0.6255057156085968
node_79: feature_name=cg17537493	feature_id[5630].value > threshold=0.5825372636318207
node_85: feature_name=cg12948116	feature_id[4375].value <= threshold=0.5658791363239288
node_86: feature_name=cg06038180	feature_id[2529].value > threshold=0.43877437710762024
node_88: feature_name=cg15720017	feature_id[5175].value > threshold=0.7009969055652618
node_90: feature_name=cg13070215	feature_id[314].value <= threshold=0.14645694941282272
node_91: feature_name=cg01014262	feature_id[1082].value <= threshold=0.5737917125225067
node_92: feature_name=cg16863382	feature_id[5471].value > threshold=0.7200668156147003
node_102: feature_name=cg06633739	feature_id[2686].value > threshold=0.47115950286388397
node_126: feature_name=cg22946562	feature_id[744].value <= threshold=0.6194809079170227
node_127: feature_name=cg23920016	feature_id[7139].value <= threshold=0.7449243068695068
node_128: feature_name=cg16521032	feature_id[5379].value > threshold=0.8948074877262115

node_136: feature_name=cg22345063	feature_id[6755].value > threshold=0.38597847521305084
node_144: feature_name=cg05650238	feature_id[2427].value <= threshold=0.7126822769641876
node_145: feature_name=cg20757748	feature_id[6410].value <= threshold=0.3033527359366417
Class: chondroblastoma (CB)	
Rules_146	passed counts:1
node_0: feature_name=cg11915444	feature_id[4116].value > threshold=0.3402601182460785
node_10: feature_name=cg12109728	feature_id[4182].value > threshold=0.7068270146846771
node_20: feature_name=cg10844382	feature_id[3856].value > threshold=0.5988894104957581
node_24: feature_name=cg00631702	feature_id[943].value <= threshold=0.37470583617687225
node_25: feature_name=cg07912144	feature_id[3075].value > threshold=0.5578519105911255
node_29: feature_name=cg24407065	feature_id[388].value > threshold=0.5364363789558411
node_33: feature_name=cg15275017	feature_id[5069].value > threshold=0.4538573920726776
node_51: feature_name=cg07281938	feature_id[351].value > threshold=0.7506992518901825
node_73: feature_name=cg17524821	feature_id[5626].value > threshold=0.4504699558019638
node_75: feature_name=cg02973735	feature_id[1677].value > threshold=0.29383400082588196
node_77: feature_name=cg23345038	feature_id[7002].value > threshold=0.6255057156085968
node_79: feature_name=cg17537493	feature_id[5630].value > threshold=0.5825372636318207
node_85: feature_name=cg12948116	feature_id[4375].value <= threshold=0.5658791363239288
node_86: feature_name=cg06038180	feature_id[2529].value > threshold=0.43877437710762024
node_88: feature_name=cg15720017	feature_id[5175].value > threshold=0.7009969055652618
node_90: feature_name=cg13070215	feature_id[314].value <= threshold=0.14645694941282272
node_91: feature_name=cg01014262	feature_id[1082].value <= threshold=0.5737917125225067

node_92: feature_name=cg16863382	feature_id[5471].value > threshold=0.7200668156147003
node_102: feature_name=cg06633739	feature_id[2686].value > threshold=0.47115950286388397
node_126: feature_name=cg22946562	feature_id[744].value <= threshold=0.6194809079170227
node_127: feature_name=cg23920016	feature_id[7139].value <= threshold=0.7449243068695068
node_128: feature_name=cg16521032	feature_id[5379].value > threshold=0.8948074877262115
node_136: feature_name=cg22345063	feature_id[6755].value <= threshold=0.38597847521305084
node_137: feature_name=cg24033503	feature_id[7174].value <= threshold=0.9229673147201538
node_138: feature_name=cg24090911	feature_id[7186].value > threshold=0.16473388671875
node_140: feature_name=cg24881558	feature_id[7382].value > threshold=0.632175624370575
Class: alveolar soft part sarcoma (ASPS)	
Rules_147	passed counts:1
node_0: feature_name=cg11915444	feature_id[4116].value > threshold=0.3402601182460785
node_10: feature_name=cg12109728	feature_id[4182].value > threshold=0.7068270146846771
node_20: feature_name=cg10844382	feature_id[3856].value > threshold=0.5988894104957581
node_24: feature_name=cg00631702	feature_id[943].value <= threshold=0.37470583617687225
node_25: feature_name=cg07912144	feature_id[3075].value > threshold=0.5578519105911255
node_29: feature_name=cg24407065	feature_id[388].value > threshold=0.5364363789558411
node_33: feature_name=cg15275017	feature_id[5069].value > threshold=0.4538573920726776
node_51: feature_name=cg07281938	feature_id[351].value > threshold=0.7506992518901825
node_73: feature_name=cg17524821	feature_id[5626].value > threshold=0.4504699558019638
node_75: feature_name=cg02973735	feature_id[1677].value > threshold=0.29383400082588196
node_77: feature_name=cg23345038	feature_id[7002].value > threshold=0.6255057156085968

node_79: feature_name=cg17537493	feature_id[5630].value > threshold=0.5825372636318207
node_85: feature_name=cg12948116	feature_id[4375].value <= threshold=0.5658791363239288
node_86: feature_name=cg06038180	feature_id[2529].value > threshold=0.43877437710762024
node_88: feature_name=cg15720017	feature_id[5175].value > threshold=0.7009969055652618
node_90: feature_name=cg13070215	feature_id[314].value <= threshold=0.14645694941282272
node_91: feature_name=cg01014262	feature_id[1082].value <= threshold=0.5737917125225067
node_92: feature_name=cg16863382	feature_id[5471].value > threshold=0.7200668156147003
node_102: feature_name=cg06633739	feature_id[2686].value > threshold=0.47115950286388397
node_126: feature_name=cg22946562	feature_id[744].value <= threshold=0.6194809079170227
node_127: feature_name=cg23920016	feature_id[7139].value <= threshold=0.7449243068695068
node_128: feature_name=cg16521032	feature_id[5379].value > threshold=0.8948074877262115
node_136: feature_name=cg22345063	feature_id[6755].value <= threshold=0.38597847521305084
node_137: feature_name=cg24033503	feature_id[7174].value <= threshold=0.9229673147201538
node_138: feature_name=cg24090911	feature_id[7186].value > threshold=0.16473388671875
node_140: feature_name=cg24881558	feature_id[7382].value <= threshold=0.632175624370575
Class: high-grade conventional osteosarcoma (OS (HG))	
Rules_148	passed counts:1
node_0: feature_name=cg11915444	feature_id[4116].value > threshold=0.3402601182460785
node_10: feature_name=cg12109728	feature_id[4182].value > threshold=0.7068270146846771
node_20: feature_name=cg10844382	feature_id[3856].value > threshold=0.5988894104957581
node_24: feature_name=cg00631702	feature_id[943].value <= threshold=0.37470583617687225
node_25: feature_name=cg07912144	feature_id[3075].value > threshold=0.5578519105911255

node_29: feature_name=cg24407065	feature_id[388].value > threshold=0.5364363789558411
node_33: feature_name=cg15275017	feature_id[5069].value > threshold=0.4538573920726776
node_51: feature_name=cg07281938	feature_id[351].value > threshold=0.7506992518901825
node_73: feature_name=cg17524821	feature_id[5626].value > threshold=0.4504699558019638
node_75: feature_name=cg02973735	feature_id[1677].value > threshold=0.29383400082588196
node_77: feature_name=cg23345038	feature_id[7002].value > threshold=0.6255057156085968
node_79: feature_name=cg17537493	feature_id[5630].value > threshold=0.5825372636318207
node_85: feature_name=cg12948116	feature_id[4375].value <= threshold=0.5658791363239288
node_86: feature_name=cg06038180	feature_id[2529].value > threshold=0.43877437710762024
node_88: feature_name=cg15720017	feature_id[5175].value > threshold=0.7009969055652618
node_90: feature_name=cg13070215	feature_id[314].value <= threshold=0.14645694941282272
node_91: feature_name=cg01014262	feature_id[1082].value <= threshold=0.5737917125225067
node_92: feature_name=cg16863382	feature_id[5471].value > threshold=0.7200668156147003
node_102: feature_name=cg06633739	feature_id[2686].value > threshold=0.47115950286388397
node_126: feature_name=cg22946562	feature_id[744].value <= threshold=0.6194809079170227
node_127: feature_name=cg23920016	feature_id[7139].value <= threshold=0.7449243068695068
node_128: feature_name=cg16521032	feature_id[5379].value > threshold=0.8948074877262115
node_136: feature_name=cg22345063	feature_id[6755].value <= threshold=0.38597847521305084
node_137: feature_name=cg24033503	feature_id[7174].value <= threshold=0.9229673147201538
node_138: feature_name=cg24090911	feature_id[7186].value <= threshold=0.16473388671875
Class: malignant peripheral nerve sheath tumour (MPNST)	
Rules_149	passed counts:1

node_0: feature_name=cg11915444	feature_id[4116].value > threshold=0.3402601182460785
node_10: feature_name=cg12109728	feature_id[4182].value > threshold=0.7068270146846771
node_20: feature_name=cg10844382	feature_id[3856].value > threshold=0.5988894104957581
node_24: feature_name=cg00631702	feature_id[943].value <= threshold=0.37470583617687225
node_25: feature_name=cg07912144	feature_id[3075].value > threshold=0.5578519105911255
node_29: feature_name=cg24407065	feature_id[388].value > threshold=0.5364363789558411
node_33: feature_name=cg15275017	feature_id[5069].value > threshold=0.4538573920726776
node_51: feature_name=cg07281938	feature_id[351].value > threshold=0.7506992518901825
node_73: feature_name=cg17524821	feature_id[5626].value > threshold=0.4504699558019638
node_75: feature_name=cg02973735	feature_id[1677].value > threshold=0.29383400082588196
node_77: feature_name=cg23345038	feature_id[7002].value > threshold=0.6255057156085968
node_79: feature_name=cg17537493	feature_id[5630].value > threshold=0.5825372636318207
node_85: feature_name=cg12948116	feature_id[4375].value <= threshold=0.5658791363239288
node_86: feature_name=cg06038180	feature_id[2529].value > threshold=0.43877437710762024
node_88: feature_name=cg15720017	feature_id[5175].value > threshold=0.7009969055652618
node_90: feature_name=cg13070215	feature_id[314].value <= threshold=0.14645694941282272
node_91: feature_name=cg01014262	feature_id[1082].value <= threshold=0.5737917125225067
node_92: feature_name=cg16863382	feature_id[5471].value > threshold=0.7200668156147003
node_102: feature_name=cg06633739	feature_id[2686].value > threshold=0.47115950286388397
node_126: feature_name=cg22946562	feature_id[744].value <= threshold=0.6194809079170227
node_127: feature_name=cg23920016	feature_id[7139].value <= threshold=0.7449243068695068
node_128: feature_name=cg16521032	feature_id[5379].value <= threshold=0.8948074877262115

node_129: feature_name=cg09597070	feature_id[555].value <= threshold=0.67006054520607
node_130: feature_name=cg08411881	feature_id[3200].value <= threshold=0.7572305798530579
node_131: feature_name=cg16636756	feature_id[5414].value > threshold=0.15655579417943954
Class: cutaneous squamous cell carcinoma (SCC (CUT))	
Rules_150	passed counts:1
node_0: feature_name=cg11915444	feature_id[4116].value > threshold=0.3402601182460785
node_10: feature_name=cg12109728	feature_id[4182].value > threshold=0.7068270146846771
node_20: feature_name=cg10844382	feature_id[3856].value > threshold=0.5988894104957581
node_24: feature_name=cg00631702	feature_id[943].value <= threshold=0.37470583617687225
node_25: feature_name=cg07912144	feature_id[3075].value > threshold=0.5578519105911255
node_29: feature_name=cg24407065	feature_id[388].value > threshold=0.5364363789558411
node_33: feature_name=cg15275017	feature_id[5069].value > threshold=0.4538573920726776
node_51: feature_name=cg07281938	feature_id[351].value > threshold=0.7506992518901825
node_73: feature_name=cg17524821	feature_id[5626].value > threshold=0.4504699558019638
node_75: feature_name=cg02973735	feature_id[1677].value > threshold=0.29383400082588196
node_77: feature_name=cg23345038	feature_id[7002].value > threshold=0.6255057156085968
node_79: feature_name=cg17537493	feature_id[5630].value > threshold=0.5825372636318207
node_85: feature_name=cg12948116	feature_id[4375].value <= threshold=0.5658791363239288
node_86: feature_name=cg06038180	feature_id[2529].value > threshold=0.43877437710762024
node_88: feature_name=cg15720017	feature_id[5175].value > threshold=0.7009969055652618
node_90: feature_name=cg13070215	feature_id[314].value <= threshold=0.14645694941282272
node_91: feature_name=cg01014262	feature_id[1082].value <= threshold=0.5737917125225067

node_92: feature_name=cg16863382	feature_id[5471].value > threshold=0.7200668156147003
node_102: feature_name=cg06633739	feature_id[2686].value > threshold=0.47115950286388397
node_126: feature_name=cg22946562	feature_id[744].value <= threshold=0.6194809079170227
node_127: feature_name=cg23920016	feature_id[7139].value <= threshold=0.7449243068695068
node_128: feature_name=cg16521032	feature_id[5379].value <= threshold=0.8948074877262115
node_129: feature_name=cg09597070	feature_id[555].value <= threshold=0.67006054520607
node_130: feature_name=cg08411881	feature_id[3200].value <= threshold=0.7572305798530579
node_131: feature_name=cg16636756	feature_id[5414].value <= threshold=0.15655579417943954
Class: nodular fasciitis(NFA)	
Rules_151	passed counts:1
node_0: feature_name=cg11915444	feature_id[4116].value > threshold=0.3402601182460785
node_10: feature_name=cg12109728	feature_id[4182].value > threshold=0.7068270146846771
node_20: feature_name=cg10844382	feature_id[3856].value > threshold=0.5988894104957581
node_24: feature_name=cg00631702	feature_id[943].value <= threshold=0.37470583617687225
node_25: feature_name=cg07912144	feature_id[3075].value > threshold=0.5578519105911255
node_29: feature_name=cg24407065	feature_id[388].value > threshold=0.5364363789558411
node_33: feature_name=cg15275017	feature_id[5069].value > threshold=0.4538573920726776
node_51: feature_name=cg07281938	feature_id[351].value > threshold=0.7506992518901825
node_73: feature_name=cg17524821	feature_id[5626].value > threshold=0.4504699558019638
node_75: feature_name=cg02973735	feature_id[1677].value > threshold=0.29383400082588196
node_77: feature_name=cg23345038	feature_id[7002].value > threshold=0.6255057156085968
node_79: feature_name=cg17537493	feature_id[5630].value > threshold=0.5825372636318207

node_85: feature_name=cg12948116	feature_id[4375].value <= threshold=0.5658791363239288
node_86: feature_name=cg06038180	feature_id[2529].value > threshold=0.43877437710762024
node_88: feature_name=cg15720017	feature_id[5175].value > threshold=0.7009969055652618
node_90: feature_name=cg13070215	feature_id[314].value <= threshold=0.14645694941282272
node_91: feature_name=cg01014262	feature_id[1082].value <= threshold=0.5737917125225067
node_92: feature_name=cg16863382	feature_id[5471].value > threshold=0.7200668156147003
node_102: feature_name=cg06633739	feature_id[2686].value <= threshold=0.47115950286388397
node_103: feature_name=cg03861097	feature_id[1943].value > threshold=0.24196631461381912
node_123: feature_name=cg11468635	feature_id[181].value > threshold=0.9009453654289246
Class: melanoma (MEL)	
Rules_152	passed counts:1
node_0: feature_name=cg11915444	feature_id[4116].value > threshold=0.3402601182460785
node_10: feature_name=cg12109728	feature_id[4182].value > threshold=0.7068270146846771
node_20: feature_name=cg10844382	feature_id[3856].value > threshold=0.5988894104957581
node_24: feature_name=cg00631702	feature_id[943].value <= threshold=0.37470583617687225
node_25: feature_name=cg07912144	feature_id[3075].value > threshold=0.5578519105911255
node_29: feature_name=cg24407065	feature_id[388].value > threshold=0.5364363789558411
node_33: feature_name=cg15275017	feature_id[5069].value > threshold=0.4538573920726776
node_51: feature_name=cg07281938	feature_id[351].value > threshold=0.7506992518901825
node_73: feature_name=cg17524821	feature_id[5626].value > threshold=0.4504699558019638
node_75: feature_name=cg02973735	feature_id[1677].value > threshold=0.29383400082588196
node_77: feature_name=cg23345038	feature_id[7002].value > threshold=0.6255057156085968

node_79: feature_name=cg17537493	feature_id[5630].value > threshold=0.5825372636318207
node_85: feature_name=cg12948116	feature_id[4375].value <= threshold=0.5658791363239288
node_86: feature_name=cg06038180	feature_id[2529].value > threshold=0.43877437710762024
node_88: feature_name=cg15720017	feature_id[5175].value > threshold=0.7009969055652618
node_90: feature_name=cg13070215	feature_id[314].value <= threshold=0.14645694941282272
node_91: feature_name=cg01014262	feature_id[1082].value <= threshold=0.5737917125225067
node_92: feature_name=cg16863382	feature_id[5471].value > threshold=0.7200668156147003
node_102: feature_name=cg06633739	feature_id[2686].value <= threshold=0.47115950286388397
node_103: feature_name=cg03861097	feature_id[1943].value <= threshold=0.24196631461381912
node_104: feature_name=cg05145297	feature_id[2285].value > threshold=0.5227190256118774
node_106: feature_name=cg16797656	feature_id[5460].value > threshold=0.4279162883758545
node_108: feature_name=cg03606646	feature_id[1871].value > threshold=0.3077465817332268
node_110: feature_name=cg22046535	feature_id[6692].value > threshold=0.5624897629022598
node_112: feature_name=cg23723410	feature_id[7092].value > threshold=0.05077140033245087
node_114: feature_name=cg13728069	feature_id[4600].value > threshold=0.7036105096340179
node_116: feature_name=cg13086402	feature_id[4402].value > threshold=0.6883106529712677
node_118: feature_name=cg00825317	feature_id[1007].value > threshold=0.8662532269954681
node_120: feature_name=cg01066494	feature_id[1103].value > threshold=0.7783335149288177
Class: undifferentiated sarcoma (USARC)	
Rules_153	passed counts:1
node_0: feature_name=cg11915444	feature_id[4116].value > threshold=0.3402601182460785
node_10: feature_name=cg12109728	feature_id[4182].value > threshold=0.7068270146846771

node_20: feature_name=cg10844382	feature_id[3856].value > threshold=0.5988894104957581
node_24: feature_name=cg00631702	feature_id[943].value <= threshold=0.37470583617687225
node_25: feature_name=cg07912144	feature_id[3075].value > threshold=0.5578519105911255
node_29: feature_name=cg24407065	feature_id[388].value > threshold=0.5364363789558411
node_33: feature_name=cg15275017	feature_id[5069].value > threshold=0.4538573920726776
node_51: feature_name=cg07281938	feature_id[351].value > threshold=0.7506992518901825
node_73: feature_name=cg17524821	feature_id[5626].value > threshold=0.4504699558019638
node_75: feature_name=cg02973735	feature_id[1677].value > threshold=0.29383400082588196
node_77: feature_name=cg23345038	feature_id[7002].value > threshold=0.6255057156085968
node_79: feature_name=cg17537493	feature_id[5630].value > threshold=0.5825372636318207
node_85: feature_name=cg12948116	feature_id[4375].value <= threshold=0.5658791363239288
node_86: feature_name=cg06038180	feature_id[2529].value > threshold=0.43877437710762024
node_88: feature_name=cg15720017	feature_id[5175].value > threshold=0.7009969055652618
node_90: feature_name=cg13070215	feature_id[314].value <= threshold=0.14645694941282272
node_91: feature_name=cg01014262	feature_id[1082].value <= threshold=0.5737917125225067
node_92: feature_name=cg16863382	feature_id[5471].value > threshold=0.7200668156147003
node_102: feature_name=cg06633739	feature_id[2686].value <= threshold=0.47115950286388397
node_103: feature_name=cg03861097	feature_id[1943].value <= threshold=0.24196631461381912
node_104: feature_name=cg05145297	feature_id[2285].value > threshold=0.5227190256118774
node_106: feature_name=cg16797656	feature_id[5460].value > threshold=0.4279162883758545
node_108: feature_name=cg03606646	feature_id[1871].value > threshold=0.3077465817332268
node_110: feature_name=cg22046535	feature_id[6692].value > threshold=0.5624897629022598

node_112: feature_name=cg23723410	feature_id[7092].value > threshold=0.05077140033245087
node_114: feature_name=cg13728069	feature_id[4600].value > threshold=0.7036105096340179
node_116: feature_name=cg13086402	feature_id[4402].value > threshold=0.6883106529712677
node_118: feature_name=cg00825317	feature_id[1007].value > threshold=0.866253226995468l
node_120: feature_name=cg01066494	feature_id[1103].value <= threshold=0.7783335149288177
Class: low-grade endometrial stromal sarcoma (ESS (LG))	
Rules_154	passed counts:1
node_0: feature_name=cg11915444	feature_id[4116].value > threshold=0.3402601182460785
node_10: feature_name=cg12109728	feature_id[4182].value > threshold=0.7068270146846771
node_20: feature_name=cg10844382	feature_id[3856].value > threshold=0.5988894104957581
node_24: feature_name=cg00631702	feature_id[943].value <= threshold=0.37470583617687225
node_25: feature_name=cg07912144	feature_id[3075].value > threshold=0.5578519105911255
node_29: feature_name=cg24407065	feature_id[388].value > threshold=0.5364363789558411
node_33: feature_name=cg15275017	feature_id[5069].value > threshold=0.4538573920726776
node_51: feature_name=cg07281938	feature_id[351].value > threshold=0.7506992518901825
node_73: feature_name=cg17524821	feature_id[5626].value > threshold=0.4504699558019638
node_75: feature_name=cg02973735	feature_id[1677].value > threshold=0.29383400082588196
node_77: feature_name=cg23345038	feature_id[7002].value > threshold=0.6255057156085968
node_79: feature_name=cg17537493	feature_id[5630].value > threshold=0.5825372636318207
node_85: feature_name=cg12948116	feature_id[4375].value <= threshold=0.5658791363239288
node_86: feature_name=cg06038180	feature_id[2529].value > threshold=0.43877437710762024
node_88: feature_name=cg15720017	feature_id[5175].value > threshold=0.7009969055652618

node_90: feature_name=cg13070215	feature_id[314].value <= threshold=0.14645694941282272
node_91: feature_name=cg01014262	feature_id[1082].value <= threshold=0.5737917125225067
node_92: feature_name=cg16863382	feature_id[5471].value > threshold=0.7200668156147003
node_102: feature_name=cg06633739	feature_id[2686].value <= threshold=0.47115950286388397
node_103: feature_name=cg03861097	feature_id[1943].value <= threshold=0.24196631461381912
node_104: feature_name=cg05145297	feature_id[2285].value > threshold=0.5227190256118774
node_106: feature_name=cg16797656	feature_id[5460].value > threshold=0.4279162883758545
node_108: feature_name=cg03606646	feature_id[1871].value > threshold=0.3077465817332268
node_110: feature_name=cg22046535	feature_id[6692].value > threshold=0.5624897629022598
node_112: feature_name=cg23723410	feature_id[7092].value > threshold=0.05077140033245087
node_114: feature_name=cg13728069	feature_id[4600].value > threshold=0.7036105096340179
node_116: feature_name=cg13086402	feature_id[4402].value > threshold=0.6883106529712677
node_118: feature_name=cg00825317	feature_id[1007].value <= threshold=0.8662532269954681
Class: chondroblastoma (CB)	
Rules_155	passed counts:1
node_0: feature_name=cg11915444	feature_id[4116].value > threshold=0.3402601182460785
node_10: feature_name=cg12109728	feature_id[4182].value > threshold=0.7068270146846771
node_20: feature_name=cg10844382	feature_id[3856].value > threshold=0.5988894104957581
node_24: feature_name=cg00631702	feature_id[943].value <= threshold=0.37470583617687225
node_25: feature_name=cg07912144	feature_id[3075].value > threshold=0.5578519105911255
node_29: feature_name=cg24407065	feature_id[388].value > threshold=0.5364363789558411
node_33: feature_name=cg15275017	feature_id[5069].value > threshold=0.4538573920726776

node_51: feature_name=cg07281938	feature_id[351].value > threshold=0.7506992518901825
node_73: feature_name=cg17524821	feature_id[5626].value > threshold=0.4504699558019638
node_75: feature_name=cg02973735	feature_id[1677].value > threshold=0.29383400082588196
node_77: feature_name=cg23345038	feature_id[7002].value > threshold=0.6255057156085968
node_79: feature_name=cg17537493	feature_id[5630].value > threshold=0.5825372636318207
node_85: feature_name=cg12948116	feature_id[4375].value <= threshold=0.5658791363239288
node_86: feature_name=cg06038180	feature_id[2529].value > threshold=0.43877437710762024
node_88: feature_name=cg15720017	feature_id[5175].value > threshold=0.7009969055652618
node_90: feature_name=cg13070215	feature_id[314].value <= threshold=0.14645694941282272
node_91: feature_name=cg01014262	feature_id[1082].value <= threshold=0.5737917125225067
node_92: feature_name=cg16863382	feature_id[5471].value > threshold=0.7200668156147003
node_102: feature_name=cg06633739	feature_id[2686].value <= threshold=0.47115950286388397
node_103: feature_name=cg03861097	feature_id[1943].value <= threshold=0.24196631461381912
node_104: feature_name=cg05145297	feature_id[2285].value > threshold=0.5227190256118774
node_106: feature_name=cg16797656	feature_id[5460].value > threshold=0.4279162883758545
node_108: feature_name=cg03606646	feature_id[1871].value > threshold=0.3077465817332268
node_110: feature_name=cg22046535	feature_id[6692].value > threshold=0.5624897629022598
node_112: feature_name=cg23723410	feature_id[7092].value > threshold=0.05077140033245087
node_114: feature_name=cg13728069	feature_id[4600].value > threshold=0.7036105096340179
node_116: feature_name=cg13086402	feature_id[4402].value <= threshold=0.6883106529712677
Class: high-grade conventional osteosarcoma (OS (HG))	
Rules_156	passed counts:1

node_0: feature_name=cg11915444	feature_id[4116].value > threshold=0.3402601182460785
node_10: feature_name=cg12109728	feature_id[4182].value > threshold=0.7068270146846771
node_20: feature_name=cg10844382	feature_id[3856].value > threshold=0.5988894104957581
node_24: feature_name=cg00631702	feature_id[943].value <= threshold=0.37470583617687225
node_25: feature_name=cg07912144	feature_id[3075].value > threshold=0.5578519105911255
node_29: feature_name=cg24407065	feature_id[388].value > threshold=0.5364363789558411
node_33: feature_name=cg15275017	feature_id[5069].value > threshold=0.4538573920726776
node_51: feature_name=cg07281938	feature_id[351].value > threshold=0.7506992518901825
node_73: feature_name=cg17524821	feature_id[5626].value > threshold=0.4504699558019638
node_75: feature_name=cg02973735	feature_id[1677].value > threshold=0.29383400082588196
node_77: feature_name=cg23345038	feature_id[7002].value > threshold=0.6255057156085968
node_79: feature_name=cg17537493	feature_id[5630].value > threshold=0.5825372636318207
node_85: feature_name=cg12948116	feature_id[4375].value <= threshold=0.5658791363239288
node_86: feature_name=cg06038180	feature_id[2529].value > threshold=0.43877437710762024
node_88: feature_name=cg15720017	feature_id[5175].value > threshold=0.7009969055652618
node_90: feature_name=cg13070215	feature_id[314].value <= threshold=0.14645694941282272
node_91: feature_name=cg01014262	feature_id[1082].value <= threshold=0.5737917125225067
node_92: feature_name=cg16863382	feature_id[5471].value > threshold=0.7200668156147003
node_102: feature_name=cg06633739	feature_id[2686].value <= threshold=0.47115950286388397
node_103: feature_name=cg03861097	feature_id[1943].value <= threshold=0.24196631461381912
node_104: feature_name=cg05145297	feature_id[2285].value > threshold=0.5227190256118774
node_106: feature_name=cg16797656	feature_id[5460].value > threshold=0.4279162883758545

node_108: feature_name=cg03606646	feature_id[1871].value > threshold=0.3077465817332268
node_110: feature_name=cg22046535	feature_id[6692].value > threshold=0.5624897629022598
node_112: feature_name=cg23723410	feature_id[7092].value > threshold=0.05077140033245087
node_114: feature_name=cg13728069	feature_id[4600].value <= threshold=0.7036105096340179
Class: malignant peripheral nerve sheath tumour (MPNST)	
Rules_157	passed counts:1
node_0: feature_name=cg11915444	feature_id[4116].value > threshold=0.3402601182460785
node_10: feature_name=cg12109728	feature_id[4182].value > threshold=0.7068270146846771
node_20: feature_name=cg10844382	feature_id[3856].value > threshold=0.5988894104957581
node_24: feature_name=cg00631702	feature_id[943].value <= threshold=0.37470583617687225
node_25: feature_name=cg07912144	feature_id[3075].value > threshold=0.5578519105911255
node_29: feature_name=cg24407065	feature_id[388].value > threshold=0.5364363789558411
node_33: feature_name=cg15275017	feature_id[5069].value > threshold=0.4538573920726776
node_51: feature_name=cg07281938	feature_id[351].value > threshold=0.7506992518901825
node_73: feature_name=cg17524821	feature_id[5626].value > threshold=0.4504699558019638
node_75: feature_name=cg02973735	feature_id[1677].value > threshold=0.29383400082588196
node_77: feature_name=cg23345038	feature_id[7002].value > threshold=0.6255057156085968
node_79: feature_name=cg17537493	feature_id[5630].value > threshold=0.5825372636318207
node_85: feature_name=cg12948116	feature_id[4375].value <= threshold=0.5658791363239288
node_86: feature_name=cg06038180	feature_id[2529].value > threshold=0.43877437710762024
node_88: feature_name=cg15720017	feature_id[5175].value > threshold=0.7009969055652618
node_90: feature_name=cg13070215	feature_id[314].value <= threshold=0.14645694941282272

node_91: feature_name=cg01014262	feature_id[1082].value <= threshold=0.5737917125225067
node_92: feature_name=cg16863382	feature_id[5471].value > threshold=0.7200668156147003
node_102: feature_name=cg06633739	feature_id[2686].value <= threshold=0.47115950286388397
node_103: feature_name=cg03861097	feature_id[1943].value <= threshold=0.24196631461381912
node_104: feature_name=cg05145297	feature_id[2285].value > threshold=0.5227190256118774
node_106: feature_name=cg16797656	feature_id[5460].value > threshold=0.4279162883758545
node_108: feature_name=cg03606646	feature_id[1871].value > threshold=0.3077465817332268
node_110: feature_name=cg22046535	feature_id[6692].value > threshold=0.5624897629022598
node_112: feature_name=cg23723410	feature_id[7092].value <= threshold=0.05077140033245087
Class: extraskeletal myxoid chondrosarcoma (EMCS)	
Rules_158	passed counts:1
node_0: feature_name=cg11915444	feature_id[4116].value > threshold=0.3402601182460785
node_10: feature_name=cg12109728	feature_id[4182].value > threshold=0.7068270146846771
node_20: feature_name=cg10844382	feature_id[3856].value > threshold=0.5988894104957581
node_24: feature_name=cg00631702	feature_id[943].value <= threshold=0.37470583617687225
node_25: feature_name=cg07912144	feature_id[3075].value > threshold=0.5578519105911255
node_29: feature_name=cg24407065	feature_id[388].value > threshold=0.5364363789558411
node_33: feature_name=cg15275017	feature_id[5069].value > threshold=0.4538573920726776
node_51: feature_name=cg07281938	feature_id[351].value > threshold=0.7506992518901825
node_73: feature_name=cg17524821	feature_id[5626].value > threshold=0.4504699558019638
node_75: feature_name=cg02973735	feature_id[1677].value > threshold=0.29383400082588196
node_77: feature_name=cg23345038	feature_id[7002].value > threshold=0.6255057156085968

node_79:<br>feature_name=cg17537493	feature_id[5630].value ><br>threshold=0.5825372636318207
node_85:<br>feature_name=cg12948116	feature_id[4375].value <=<br>threshold=0.5658791363239288
node_86:<br>feature_name=cg06038180	feature_id[2529].value ><br>threshold=0.43877437710762024
node_88:<br>feature_name=cg15720017	feature_id[5175].value ><br>threshold=0.7009969055652618
node_90:<br>feature_name=cg13070215	feature_id[314].value <=<br>threshold=0.14645694941282272
node_91:<br>feature_name=cg01014262	feature_id[1082].value <=<br>threshold=0.5737917125225067
node_92:<br>feature_name=cg16863382	feature_id[5471].value ><br>threshold=0.7200668156147003
node_102:<br>feature_name=cg06633739	feature_id[2686].value <=<br>threshold=0.47115950286388397
node_103:<br>feature_name=cg03861097	feature_id[1943].value <=<br>threshold=0.24196631461381912
node_104:<br>feature_name=cg05145297	feature_id[2285].value ><br>threshold=0.5227190256118774
node_106:<br>feature_name=cg16797656	feature_id[5460].value ><br>threshold=0.4279162883758545
node_108:<br>feature_name=cg03606646	feature_id[1871].value ><br>threshold=0.3077465817332268
node_110:<br>feature_name=cg22046535	feature_id[6692].value <=<br>threshold=0.5624897629022598
Class: melanoma (MEL)	
Rules_159	passed counts:1
node_0:<br>feature_name=cg11915444	feature_id[4116].value ><br>threshold=0.3402601182460785
node_10:<br>feature_name=cg12109728	feature_id[4182].value ><br>threshold=0.7068270146846771
node_20:<br>feature_name=cg10844382	feature_id[3856].value ><br>threshold=0.5988894104957581
node_24:<br>feature_name=cg00631702	feature_id[943].value <=<br>threshold=0.37470583617687225
node_25:<br>feature_name=cg07912144	feature_id[3075].value ><br>threshold=0.5578519105911255
node_29:<br>feature_name=cg24407065	feature_id[388].value ><br>threshold=0.5364363789558411
node_33:<br>feature_name=cg15275017	feature_id[5069].value ><br>threshold=0.4538573920726776

node_51: feature_name=cg07281938	feature_id[351].value > threshold=0.7506992518901825
node_73: feature_name=cg17524821	feature_id[5626].value > threshold=0.4504699558019638
node_75: feature_name=cg02973735	feature_id[1677].value > threshold=0.29383400082588196
node_77: feature_name=cg23345038	feature_id[7002].value > threshold=0.6255057156085968
node_79: feature_name=cg17537493	feature_id[5630].value > threshold=0.5825372636318207
node_85: feature_name=cg12948116	feature_id[4375].value <= threshold=0.5658791363239288
node_86: feature_name=cg06038180	feature_id[2529].value > threshold=0.43877437710762024
node_88: feature_name=cg15720017	feature_id[5175].value > threshold=0.7009969055652618
node_90: feature_name=cg13070215	feature_id[314].value <= threshold=0.14645694941282272
node_91: feature_name=cg01014262	feature_id[1082].value <= threshold=0.5737917125225067
node_92: feature_name=cg16863382	feature_id[5471].value > threshold=0.7200668156147003
node_102: feature_name=cg06633739	feature_id[2686].value <= threshold=0.47115950286388397
node_103: feature_name=cg03861097	feature_id[1943].value <= threshold=0.24196631461381912
node_104: feature_name=cg05145297	feature_id[2285].value > threshold=0.5227190256118774
node_106: feature_name=cg16797656	feature_id[5460].value > threshold=0.4279162883758545
node_108: feature_name=cg03606646	feature_id[1871].value <= threshold=0.3077465817332268
Class: leiomyosarcoma (LMS)	
Rules_160	passed counts:1
node_0: feature_name=cg11915444	feature_id[4116].value > threshold=0.3402601182460785
node_10: feature_name=cg12109728	feature_id[4182].value > threshold=0.7068270146846771
node_20: feature_name=cg10844382	feature_id[3856].value > threshold=0.5988894104957581
node_24: feature_name=cg00631702	feature_id[943].value <= threshold=0.37470583617687225

node_25: feature_name=cg07912144	feature_id[3075].value > threshold=0.5578519105911255
node_29: feature_name=cg24407065	feature_id[388].value > threshold=0.5364363789558411
node_33: feature_name=cg15275017	feature_id[5069].value > threshold=0.4538573920726776
node_51: feature_name=cg07281938	feature_id[351].value > threshold=0.7506992518901825
node_73: feature_name=cg17524821	feature_id[5626].value > threshold=0.4504699558019638
node_75: feature_name=cg02973735	feature_id[1677].value > threshold=0.29383400082588196
node_77: feature_name=cg23345038	feature_id[7002].value > threshold=0.6255057156085968
node_79: feature_name=cg17537493	feature_id[5630].value > threshold=0.5825372636318207
node_85: feature_name=cg12948116	feature_id[4375].value <= threshold=0.5658791363239288
node_86: feature_name=cg06038180	feature_id[2529].value > threshold=0.43877437710762024
node_88: feature_name=cg15720017	feature_id[5175].value > threshold=0.7009969055652618
node_90: feature_name=cg13070215	feature_id[314].value <= threshold=0.14645694941282272
node_91: feature_name=cg01014262	feature_id[1082].value <= threshold=0.5737917125225067
node_92: feature_name=cg16863382	feature_id[5471].value <= threshold=0.7200668156147003
node_93: feature_name=cg18601229	feature_id[5883].value <= threshold=0.7762220203876495
node_94: feature_name=cg04819180	feature_id[2191].value <= threshold=0.8672285974025726
node_95: feature_name=cg09370702	feature_id[3456].value > threshold=0.8020930886268616
node_97: feature_name=cg08625990	feature_id[3258].value > threshold=0.8129918575286865
Class: rhabdomyosarcoma (RMS)	
Rules_161	passed counts:1
node_0: feature_name=cg11915444	feature_id[4116].value > threshold=0.3402601182460785
node_10: feature_name=cg12109728	feature_id[4182].value > threshold=0.7068270146846771

node_20: feature_name=cg10844382	feature_id[3856].value > threshold=0.5988894104957581
node_24: feature_name=cg00631702	feature_id[943].value <= threshold=0.37470583617687225
node_25: feature_name=cg07912144	feature_id[3075].value > threshold=0.5578519105911255
node_29: feature_name=cg24407065	feature_id[388].value > threshold=0.5364363789558411
node_33: feature_name=cg15275017	feature_id[5069].value > threshold=0.4538573920726776
node_51: feature_name=cg07281938	feature_id[351].value > threshold=0.7506992518901825
node_73: feature_name=cg17524821	feature_id[5626].value > threshold=0.4504699558019638
node_75: feature_name=cg02973735	feature_id[1677].value > threshold=0.29383400082588196
node_77: feature_name=cg23345038	feature_id[7002].value > threshold=0.6255057156085968
node_79: feature_name=cg17537493	feature_id[5630].value <= threshold=0.5825372636318207
node_80: feature_name=cg07990390	feature_id[515].value > threshold=0.7988054752349854
node_82: feature_name=cg10006956	feature_id[3627].value > threshold=0.9051482081413269
Class: epithelioid haemangioendothelioma (EHE)	
Rules_162	passed counts:1
node_0: feature_name=cg11915444	feature_id[4116].value > threshold=0.3402601182460785
node_10: feature_name=cg12109728	feature_id[4182].value > threshold=0.7068270146846771
node_20: feature_name=cg10844382	feature_id[3856].value > threshold=0.5988894104957581
node_24: feature_name=cg00631702	feature_id[943].value <= threshold=0.37470583617687225
node_25: feature_name=cg07912144	feature_id[3075].value > threshold=0.5578519105911255
node_29: feature_name=cg24407065	feature_id[388].value > threshold=0.5364363789558411
node_33: feature_name=cg15275017	feature_id[5069].value > threshold=0.4538573920726776
node_51: feature_name=cg07281938	feature_id[351].value <= threshold=0.7506992518901825

node_52: feature_name=cg21189849	feature_id[6504].value > threshold=0.7545044124126434
node_58: feature_name=cg02867857	feature_id[1644].value > threshold=0.5450765490531921
node_60: feature_name=cg00863893	feature_id[1017].value <= threshold=0.5313664376735687
node_61: feature_name=cg22397446	feature_id[6765].value <= threshold=0.02860488835722208
node_62: feature_name=cg19781117	feature_id[6171].value > threshold=0.6593038737773895
node_64: feature_name=cg03385114	feature_id[1805].value > threshold=0.566239520907402
node_66: feature_name=cg09043524	feature_id[3372].value > threshold=0.7512756288051605
node_68: feature_name=cg04627183	feature_id[2137].value > threshold=0.8674522340297699
Class: angiomatoid fibrous histiocytoma (AFH)	
Rules_163	passed counts:1
node_0: feature_name=cg11915444	feature_id[4116].value > threshold=0.3402601182460785
node_10: feature_name=cg12109728	feature_id[4182].value > threshold=0.7068270146846771
node_20: feature_name=cg10844382	feature_id[3856].value > threshold=0.5988894104957581
node_24: feature_name=cg00631702	feature_id[943].value <= threshold=0.37470583617687225
node_25: feature_name=cg07912144	feature_id[3075].value > threshold=0.5578519105911255
node_29: feature_name=cg24407065	feature_id[388].value > threshold=0.5364363789558411
node_33: feature_name=cg15275017	feature_id[5069].value > threshold=0.4538573920726776
node_51: feature_name=cg07281938	feature_id[351].value <= threshold=0.7506992518901825
node_52: feature_name=cg21189849	feature_id[6504].value > threshold=0.7545044124126434
node_58: feature_name=cg02867857	feature_id[1644].value > threshold=0.5450765490531921
node_60: feature_name=cg00863893	feature_id[1017].value <= threshold=0.5313664376735687
node_61: feature_name=cg22397446	feature_id[6765].value <= threshold=0.02860488835722208

node_62: feature_name=cg19781117	feature_id[6171].value > threshold=0.6593038737773895
node_64: feature_name=cg03385114	feature_id[1805].value > threshold=0.566239520907402
node_66: feature_name=cg09043524	feature_id[3372].value > threshold=0.7512756288051605
node_68: feature_name=cg04627183	feature_id[2137].value <= threshold=0.8674522340297699
Class: angioleiomyoma (ALMO)/myopericytoma (MPC)	
Rules_164	passed counts:1
node_0: feature_name=cg11915444	feature_id[4116].value > threshold=0.3402601182460785
node_10: feature_name=cg12109728	feature_id[4182].value > threshold=0.7068270146846771
node_20: feature_name=cg10844382	feature_id[3856].value > threshold=0.5988894104957581
node_24: feature_name=cg00631702	feature_id[943].value <= threshold=0.37470583617687225
node_25: feature_name=cg07912144	feature_id[3075].value > threshold=0.5578519105911255
node_29: feature_name=cg24407065	feature_id[388].value > threshold=0.5364363789558411
node_33: feature_name=cg15275017	feature_id[5069].value > threshold=0.4538573920726776
node_51: feature_name=cg07281938	feature_id[351].value <= threshold=0.7506992518901825
node_52: feature_name=cg21189849	feature_id[6504].value > threshold=0.7545044124126434
node_58: feature_name=cg02867857	feature_id[1644].value > threshold=0.5450765490531921
node_60: feature_name=cg00863893	feature_id[1017].value <= threshold=0.5313664376735687
node_61: feature_name=cg22397446	feature_id[6765].value <= threshold=0.028604888357222208
node_62: feature_name=cg19781117	feature_id[6171].value > threshold=0.6593038737773895
node_64: feature_name=cg03385114	feature_id[1805].value > threshold=0.566239520907402
node_66: feature_name=cg09043524	feature_id[3372].value <= threshold=0.7512756288051605
Class: chondroblastoma (CB)	

Rules_165	passed counts:1
node_0: feature_name=cg11915444	feature_id[4116].value > threshold=0.3402601182460785
node_10: feature_name=cg12109728	feature_id[4182].value > threshold=0.7068270146846771
node_20: feature_name=cg10844382	feature_id[3856].value > threshold=0.5988894104957581
node_24: feature_name=cg00631702	feature_id[943].value <= threshold=0.37470583617687225
node_25: feature_name=cg07912144	feature_id[3075].value > threshold=0.5578519105911255
node_29: feature_name=cg24407065	feature_id[388].value > threshold=0.5364363789558411
node_33: feature_name=cg15275017	feature_id[5069].value > threshold=0.4538573920726776
node_51: feature_name=cg07281938	feature_id[351].value <= threshold=0.7506992518901825
node_52: feature_name=cg21189849	feature_id[6504].value > threshold=0.7545044124126434
node_58: feature_name=cg02867857	feature_id[1644].value > threshold=0.5450765490531921
node_60: feature_name=cg00863893	feature_id[1017].value <= threshold=0.5313664376735687
node_61: feature_name=cg22397446	feature_id[6765].value <= threshold=0.028604888357222208
node_62: feature_name=cg19781117	feature_id[6171].value > threshold=0.6593038737773895
node_64: feature_name=cg03385114	feature_id[1805].value <= threshold=0.566239520907402
Class: osteoblastoma (OB)	
Rules_166	passed counts:1
node_0: feature_name=cg11915444	feature_id[4116].value > threshold=0.3402601182460785
node_10: feature_name=cg12109728	feature_id[4182].value > threshold=0.7068270146846771
node_20: feature_name=cg10844382	feature_id[3856].value > threshold=0.5988894104957581
node_24: feature_name=cg00631702	feature_id[943].value <= threshold=0.37470583617687225
node_25: feature_name=cg07912144	feature_id[3075].value > threshold=0.5578519105911255

node_29: feature_name=cg24407065	feature_id[388].value > threshold=0.5364363789558411
node_33: feature_name=cg15275017	feature_id[5069].value > threshold=0.4538573920726776
node_51: feature_name=cg07281938	feature_id[351].value <= threshold=0.7506992518901825
node_52: feature_name=cg21189849	feature_id[6504].value > threshold=0.7545044124126434
node_58: feature_name=cg02867857	feature_id[1644].value > threshold=0.5450765490531921
node_60: feature_name=cg00863893	feature_id[1017].value <= threshold=0.5313664376735687
node_61: feature_name=cg22397446	feature_id[6765].value <= threshold=0.02860488835722208
node_62: feature_name=cg19781117	feature_id[6171].value <= threshold=0.6593038737773895
Class: solitary fibrous tumour (SFT)	
Rules_167	passed counts:1
node_0: feature_name=cg11915444	feature_id[4116].value > threshold=0.3402601182460785
node_10: feature_name=cg12109728	feature_id[4182].value > threshold=0.7068270146846771
node_20: feature_name=cg10844382	feature_id[3856].value > threshold=0.5988894104957581
node_24: feature_name=cg00631702	feature_id[943].value <= threshold=0.37470583617687225
node_25: feature_name=cg07912144	feature_id[3075].value > threshold=0.5578519105911255
node_29: feature_name=cg24407065	feature_id[388].value > threshold=0.5364363789558411
node_33: feature_name=cg15275017	feature_id[5069].value > threshold=0.4538573920726776
node_51: feature_name=cg07281938	feature_id[351].value <= threshold=0.7506992518901825
node_52: feature_name=cg21189849	feature_id[6504].value <= threshold=0.7545044124126434
node_53: feature_name=cg11717552	feature_id[4060].value <= threshold=0.8407479226589203
node_54: feature_name=cg07043952	feature_id[2823].value <= threshold=0.8715284764766693
Class: fibrous dysplasia (FDY)	

Rules_168	passed counts:1
node_0: feature_name=cg11915444	feature_id[4116].value > threshold=0.3402601182460785
node_10: feature_name=cg12109728	feature_id[4182].value > threshold=0.7068270146846771
node_20: feature_name=cg10844382	feature_id[3856].value > threshold=0.5988894104957581
node_24: feature_name=cg00631702	feature_id[943].value <= threshold=0.37470583617687225
node_25: feature_name=cg07912144	feature_id[3075].value > threshold=0.5578519105911255
node_29: feature_name=cg24407065	feature_id[388].value > threshold=0.5364363789558411
node_33: feature_name=cg15275017	feature_id[5069].value <= threshold=0.4538573920726776
node_34: feature_name=cg08236537	feature_id[3160].value > threshold=0.4830351173877716
node_46: feature_name=cg13287523	feature_id[632].value <= threshold=0.6652203798294067
node_47: feature_name=cg11103999	feature_id[3923].value > threshold=0.8052912056446075
Class: angioleiomyoma (ALMO)/myopericytoma (MPC)	
Rules_169	passed counts:1
node_0: feature_name=cg11915444	feature_id[4116].value > threshold=0.3402601182460785
node_10: feature_name=cg12109728	feature_id[4182].value > threshold=0.7068270146846771
node_20: feature_name=cg10844382	feature_id[3856].value > threshold=0.5988894104957581
node_24: feature_name=cg00631702	feature_id[943].value <= threshold=0.37470583617687225
node_25: feature_name=cg07912144	feature_id[3075].value > threshold=0.5578519105911255
node_29: feature_name=cg24407065	feature_id[388].value > threshold=0.5364363789558411
node_33: feature_name=cg15275017	feature_id[5069].value <= threshold=0.4538573920726776
node_34: feature_name=cg08236537	feature_id[3160].value > threshold=0.4830351173877716
node_46: feature_name=cg13287523	feature_id[632].value <= threshold=0.6652203798294067

node_47: feature_name=cg11103999	feature_id[3923].value <= threshold=0.8052912056446075
Class: undifferentiated sarcoma (USARC)	
Rules_170	passed counts:1
node_0: feature_name=cg11915444	feature_id[4116].value > threshold=0.3402601182460785
node_10: feature_name=cg12109728	feature_id[4182].value > threshold=0.7068270146846771
node_20: feature_name=cg10844382	feature_id[3856].value > threshold=0.5988894104957581
node_24: feature_name=cg00631702	feature_id[943].value <= threshold=0.37470583617687225
node_25: feature_name=cg07912144	feature_id[3075].value > threshold=0.5578519105911255
node_29: feature_name=cg24407065	feature_id[388].value > threshold=0.5364363789558411
node_33: feature_name=cg15275017	feature_id[5069].value <= threshold=0.4538573920726776
node_34: feature_name=cg08236537	feature_id[3160].value <= threshold=0.4830351173877716
node_35: feature_name=cg21591452	feature_id[6603].value <= threshold=0.878025621175766
node_36: feature_name=cg07368061	feature_id[2933].value <= threshold=0.6850627958774567
node_37: feature_name=cg21562750	feature_id[6600].value > threshold=0.680396169424057
node_39: feature_name=cg09268338	feature_id[3430].value > threshold=0.851186990737915
node_41: feature_name=cg10507281	feature_id[3759].value > threshold=0.928876131772995
Class: sclerosing epithelioid fibrosarcoma(SEF)	
Rules_171	passed counts:1
node_0: feature_name=cg11915444	feature_id[4116].value > threshold=0.3402601182460785
node_10: feature_name=cg12109728	feature_id[4182].value > threshold=0.7068270146846771
node_20: feature_name=cg10844382	feature_id[3856].value > threshold=0.5988894104957581
node_24: feature_name=cg00631702	feature_id[943].value <= threshold=0.37470583617687225

node_25: feature_name=cg07912144	feature_id[3075].value > threshold=0.5578519105911255
node_29: feature_name=cg24407065	feature_id[388].value > threshold=0.5364363789558411
node_33: feature_name=cg15275017	feature_id[5069].value <= threshold=0.4538573920726776
node_34: feature_name=cg08236537	feature_id[3160].value <= threshold=0.4830351173877716
node_35: feature_name=cg21591452	feature_id[6603].value <= threshold=0.878025621175766
node_36: feature_name=cg07368061	feature_id[2933].value <= threshold=0.6850627958774567
node_37: feature_name=cg21562750	feature_id[6600].value > threshold=0.680396169424057
node_39: feature_name=cg09268338	feature_id[3430].value > threshold=0.851186990737915
node_41: feature_name=cg10507281	feature_id[3759].value <= threshold=0.928876131772995
Class: embryonal rhabdomyosarcoma (RMS (EMB))	
Rules_172	passed counts:1
node_0: feature_name=cg11915444	feature_id[4116].value > threshold=0.3402601182460785
node_10: feature_name=cg12109728	feature_id[4182].value > threshold=0.7068270146846771
node_20: feature_name=cg10844382	feature_id[3856].value > threshold=0.5988894104957581
node_24: feature_name=cg00631702	feature_id[943].value <= threshold=0.37470583617687225
node_25: feature_name=cg07912144	feature_id[3075].value > threshold=0.5578519105911255
node_29: feature_name=cg24407065	feature_id[388].value > threshold=0.5364363789558411
node_33: feature_name=cg15275017	feature_id[5069].value <= threshold=0.4538573920726776
node_34: feature_name=cg08236537	feature_id[3160].value <= threshold=0.4830351173877716
node_35: feature_name=cg21591452	feature_id[6603].value <= threshold=0.878025621175766
node_36: feature_name=cg07368061	feature_id[2933].value <= threshold=0.6850627958774567
node_37: feature_name=cg21562750	feature_id[6600].value > threshold=0.680396169424057

node_39: feature_name=cg09268338	feature_id[3430].value <= threshold=0.851186990737915
Class: high-grade conventional osteosarcoma (OS (HG))	
Rules_173	passed counts:1
node_0: feature_name=cg11915444	feature_id[4116].value > threshold=0.3402601182460785
node_10: feature_name=cg12109728	feature_id[4182].value > threshold=0.7068270146846771
node_20: feature_name=cg10844382	feature_id[3856].value > threshold=0.5988894104957581
node_24: feature_name=cg00631702	feature_id[943].value <= threshold=0.37470583617687225
node_25: feature_name=cg07912144	feature_id[3075].value > threshold=0.5578519105911255
node_29: feature_name=cg24407065	feature_id[388].value <= threshold=0.5364363789558411
node_30: feature_name=cg03300805	feature_id[1780].value <= threshold=0.03215229045599699
Class: embryonal rhabdomyosarcoma (RMS (EMB))	
Rules_174	passed counts:1
node_0: feature_name=cg11915444	feature_id[4116].value > threshold=0.3402601182460785
node_10: feature_name=cg12109728	feature_id[4182].value > threshold=0.7068270146846771
node_20: feature_name=cg10844382	feature_id[3856].value > threshold=0.5988894104957581
node_24: feature_name=cg00631702	feature_id[943].value <= threshold=0.37470583617687225
node_25: feature_name=cg07912144	feature_id[3075].value <= threshold=0.5578519105911255
node_26: feature_name=cg07478111	feature_id[2959].value <= threshold=0.7341253161430359
Class: mesenchymal chondrosarcoma (CSA (MES))	
Rules_175	passed counts:1
node_0: feature_name=cg11915444	feature_id[4116].value > threshold=0.3402601182460785
node_10: feature_name=cg12109728	feature_id[4182].value > threshold=0.7068270146846771
node_20: feature_name=cg10844382	feature_id[3856].value <= threshold=0.5988894104957581

node_21: feature_name=cg02563156	feature_id[1540].value <= threshold=0.6083262264728546
Class: sarcoma (SARC)	
Rules_176	passed counts:1
node_0: feature_name=cg11915444	feature_id[4116].value > threshold=0.3402601182460785
node_10: feature_name=cg12109728	feature_id[4182].value <= threshold=0.7068270146846771
node_11: feature_name=cg23902076	feature_id[7131].value > threshold=0.8691235482692719
node_13: feature_name=cg20099458	feature_id[6250].value <= threshold=0.7871655821800232
node_14: feature_name=cg03333116	feature_id[1790].value > threshold=0.8223622441291809
node_16: feature_name=cg15127656	feature_id[5038].value > threshold=0.9118743538856506
Class: embryonal rhabdomyosarcoma (RMS (EMB))	
Rules_177	passed counts:1
node_0: feature_name=cg11915444	feature_id[4116].value > threshold=0.3402601182460785
node_10: feature_name=cg12109728	feature_id[4182].value <= threshold=0.7068270146846771
node_11: feature_name=cg23902076	feature_id[7131].value > threshold=0.8691235482692719
node_13: feature_name=cg20099458	feature_id[6250].value <= threshold=0.7871655821800232
node_14: feature_name=cg03333116	feature_id[1790].value > threshold=0.8223622441291809
node_16: feature_name=cg15127656	feature_id[5038].value <= threshold=0.9118743538856506
Class: angiosarcoma (AS)	
Rules_178	passed counts:1
node_0: feature_name=cg11915444	feature_id[4116].value > threshold=0.3402601182460785
node_10: feature_name=cg12109728	feature_id[4182].value <= threshold=0.7068270146846771
node_11: feature_name=cg23902076	feature_id[7131].value > threshold=0.8691235482692719
node_13: feature_name=cg20099458	feature_id[6250].value <= threshold=0.7871655821800232

node_14: feature_name=cg03333116	feature_id[1790].value <= threshold=0.8223622441291809
Class: gastrointestinal stromal tumour (GIST)	
Rules_179	passed counts:1
node_0: feature_name=cg11915444	feature_id[4116].value <= threshold=0.3402601182460785
node_1: feature_name=cg00982952	feature_id[1071].value > threshold=0.7386334538459778
node_3: feature_name=cg22315164	feature_id[6749].value > threshold=0.35902709513902664
node_5: feature_name=cg09180239	feature_id[3404].value > threshold=0.5538817346096039
node_7: feature_name=cg00366037	feature_id[856].value > threshold=0.8601809740066528
Class: chordoma (CHORD)	
Rules_180	passed counts:1
node_0: feature_name=cg11915444	feature_id[4116].value <= threshold=0.3402601182460785
node_1: feature_name=cg00982952	feature_id[1071].value > threshold=0.7386334538459778
node_3: feature_name=cg22315164	feature_id[6749].value > threshold=0.35902709513902664
node_5: feature_name=cg09180239	feature_id[3404].value > threshold=0.5538817346096039
node_7: feature_name=cg00366037	feature_id[856].value <= threshold=0.8601809740066528
Class: infantile fibrosarcoma (IFS)	
Rules_181	passed counts:1
node_0: feature_name=cg11915444	feature_id[4116].value <= threshold=0.3402601182460785
node_1: feature_name=cg00982952	feature_id[1071].value > threshold=0.7386334538459778
node_3: feature_name=cg22315164	feature_id[6749].value > threshold=0.35902709513902664
node_5: feature_name=cg09180239	feature_id[3404].value <= threshold=0.5538817346096039
Class: angiosarcoma (AS)	

(2) Rules on the LightGBM feature list

Rules_0	passed counts:72

node_0: feature_name=cg11915444	feature_id[1171].value <= threshold=0.3402601182460785
node_1: feature_name=cg00204976	feature_id[3433].value <= threshold=0.7535275816917419
Class: Ewing sarcoma (EWING)	
Rules_1	passed counts:70
node_0: feature_name=cg11915444	feature_id[1171].value > threshold=0.3402601182460785
node_10: feature_name=cg12109728	feature_id[2810].value > threshold=0.7068270146846771
node_20: feature_name=cg16412000	feature_id[789].value > threshold=0.6741803884506226
node_24: feature_name=cg17115147	feature_id[1434].value > threshold=0.2961975038051605
node_368: feature_name=cg27543578	feature_id[878].value <= threshold=0.8884280920028687
node_369: feature_name=cg00133595	feature_id[2504].value <= threshold=0.6530520617961884
Class: high-grade conventional osteosarcoma (OS (HG))	
Rules_2	passed counts:67
node_0: feature_name=cg11915444	feature_id[1171].value > threshold=0.3402601182460785
node_10: feature_name=cg12109728	feature_id[2810].value <= threshold=0.7068270146846771
node_11: feature_name=cg23902076	feature_id[1902].value <= threshold=0.8691235482692719
Class: chordoma (CHORD)	
Rules_3	passed counts:63
node_0: feature_name=cg11915444	feature_id[1171].value > threshold=0.3402601182460785
node_10: feature_name=cg12109728	feature_id[2810].value > threshold=0.7068270146846771
node_20: feature_name=cg16412000	feature_id[789].value > threshold=0.6741803884506226
node_24: feature_name=cg17115147	feature_id[1434].value <= threshold=0.2961975038051605
node_25: feature_name=cg24474622	feature_id[2737].value > threshold=0.7211934924125671

node_29: feature_name=cg10472711	feature_id[612].value > threshold=0.45985929667949677
node_37: feature_name=cg01937669	feature_id[188].value <= threshold=0.4809463769197464
node_38: feature_name=cg02157052	feature_id[451].value > threshold=0.2869153320789337
node_344: feature_name=cg21189849	feature_id[189].value <= threshold=0.7746135592460632
node_345: feature_name=cg00680875	feature_id[833].value > threshold=0.352913498878479
Class: chondrosarcoma (CSA)	
Rules_4	passed counts:62
node_0: feature_name=cg11915444	feature_id[1171].value > threshold=0.3402601182460785
node_10: feature_name=cg12109728	feature_id[2810].value > threshold=0.7068270146846771
node_20: feature_name=cg16412000	feature_id[789].value <= threshold=0.6741803884506226
node_21: feature_name=cg08571020	feature_id[921].value <= threshold=0.7741039395332336
Class: alveolar rhabdomyosarcoma (RMS (ALV))	
Rules_5	passed counts:54
node_0: feature_name=cg11915444	feature_id[1171].value > threshold=0.3402601182460785
node_10: feature_name=cg12109728	feature_id[2810].value > threshold=0.7068270146846771
node_20: feature_name=cg16412000	feature_id[789].value > threshold=0.6741803884506226
node_24: feature_name=cg17115147	feature_id[1434].value <= threshold=0.2961975038051605
node_25: feature_name=cg24474622	feature_id[2737].value <= threshold=0.7211934924125671
node_26: feature_name=cg09828346	feature_id[2249].value <= threshold=0.73759526014328
Class: synovial sarcoma (SYSA)	
Rules_6	passed counts:49
node_0: feature_name=cg11915444	feature_id[1171].value > threshold=0.3402601182460785

node_10: feature_name=cg12109728	feature_id[2810].value > threshold=0.7068270146846771
node_20: feature_name=cg16412000	feature_id[789].value > threshold=0.6741803884506226
node_24: feature_name=cg17115147	feature_id[1434].value <= threshold=0.2961975038051605
node_25: feature_name=cg24474622	feature_id[2737].value > threshold=0.7211934924125671
node_29: feature_name=cg10472711	feature_id[612].value <= threshold=0.45985929667949677
node_30: feature_name=cg06132803	feature_id[3655].value > threshold=0.420499712228775
Class: gastrointestinal stromal tumour (GIST)	
Rules_7	passed counts:48
node_0: feature_name=cg11915444	feature_id[1171].value > threshold=0.3402601182460785
node_10: feature_name=cg12109728	feature_id[2810].value > threshold=0.7068270146846771
node_20: feature_name=cg16412000	feature_id[789].value > threshold=0.6741803884506226
node_24: feature_name=cg17115147	feature_id[1434].value <= threshold=0.2961975038051605
node_25: feature_name=cg24474622	feature_id[2737].value > threshold=0.7211934924125671
node_29: feature_name=cg10472711	feature_id[612].value > threshold=0.45985929667949677
node_37: feature_name=cg01937669	feature_id[188].value > threshold=0.4809463769197464
node_363: feature_name=cg14588828	feature_id[2222].value > threshold=0.9071650803089142
Class: leiomyosarcoma (LMS)	
Rules_8	passed counts:42
node_0: feature_name=cg11915444	feature_id[1171].value > threshold=0.3402601182460785
node_10: feature_name=cg12109728	feature_id[2810].value > threshold=0.7068270146846771
node_20: feature_name=cg16412000	feature_id[789].value > threshold=0.6741803884506226
node_24: feature_name=cg17115147	feature_id[1434].value <= threshold=0.2961975038051605

node_25: feature_name=cg24474622	feature_id[2737].value > threshold=0.7211934924125671
node_29: feature_name=cg10472711	feature_id[612].value > threshold=0.4598592967949677
node_37: feature_name=cg01937669	feature_id[188].value <= threshold=0.4809463769197464
node_38: feature_name=cg02157052	feature_id[451].value <= threshold=0.2869153320789337
node_39: feature_name=cg18751958	feature_id[1427].value <= threshold=0.4813787043094635
Class: desmoplastic small round cell tumour (DSRCT)	
Rules_9	passed counts:40
node_0: feature_name=cg11915444	feature_id[1171].value > threshold=0.3402601182460785
node_10: feature_name=cg12109728	feature_id[2810].value > threshold=0.7068270146846771
node_20: feature_name=cg16412000	feature_id[789].value > threshold=0.6741803884506226
node_24: feature_name=cg17115147	feature_id[1434].value <= threshold=0.2961975038051605
node_25: feature_name=cg24474622	feature_id[2737].value > threshold=0.7211934924125671
node_29: feature_name=cg10472711	feature_id[612].value > threshold=0.4598592967949677
node_37: feature_name=cg01937669	feature_id[188].value <= threshold=0.4809463769197464
node_38: feature_name=cg02157052	feature_id[451].value <= threshold=0.2869153320789337
node_39: feature_name=cg18751958	feature_id[1427].value > threshold=0.4813787043094635
node_41: feature_name=cg22512847	feature_id[1152].value > threshold=0.5129324495792389
node_43: feature_name=cg23345038	feature_id[1147].value > threshold=0.6318148374557495
node_45: feature_name=cg17439009	feature_id[1815].value > threshold=0.48514117300510406
node_337: feature_name=cg14005246	feature_id[69].value > threshold=0.4851873368024826
Class: embryonal rhabdomyosarcoma (RMS (EMB))	

Rules_10	passed counts:40
node_0: feature_name=cg11915444	feature_id[1171].value > threshold=0.3402601182460785
node_10: feature_name=cg12109728	feature_id[2810].value > threshold=0.7068270146846771
node_20: feature_name=cg16412000	feature_id[789].value > threshold=0.6741803884506226
node_24: feature_name=cg17115147	feature_id[1434].value <= threshold=0.2961975038051605
node_25: feature_name=cg24474622	feature_id[2737].value > threshold=0.7211934924125671
node_29: feature_name=cg10472711	feature_id[612].value > threshold=0.45985929667949677
node_37: feature_name=cg01937669	feature_id[188].value <= threshold=0.4809463769197464
node_38: feature_name=cg02157052	feature_id[451].value <= threshold=0.2869153320789337
node_39: feature_name=cg18751958	feature_id[1427].value > threshold=0.4813787043094635
node_41: feature_name=cg22512847	feature_id[1152].value > threshold=0.5129324495792389
node_43: feature_name=cg23345038	feature_id[1147].value > threshold=0.6318148374557495
node_45: feature_name=cg17439009	feature_id[1815].value <= threshold=0.48514117300510406
node_46: feature_name=cg17537493	feature_id[2772].value <= threshold=0.5825372636318207
node_47: feature_name=cg16572224	feature_id[2780].value > threshold=0.6722612977027893
Class: angiosarcoma (AS)	
Rules_11	passed counts:38
node_0: feature_name=cg11915444	feature_id[1171].value > threshold=0.3402601182460785
node_10: feature_name=cg12109728	feature_id[2810].value > threshold=0.7068270146846771
node_20: feature_name=cg16412000	feature_id[789].value > threshold=0.6741803884506226
node_24: feature_name=cg17115147	feature_id[1434].value <= threshold=0.2961975038051605
node_25: feature_name=cg24474622	feature_id[2737].value > threshold=0.7211934924125671

node_29: feature_name=cg10472711	feature_id[612].value > threshold=0.45985929667949677
node_37: feature_name=cg01937669	feature_id[188].value <= threshold=0.4809463769197464
node_38: feature_name=cg02157052	feature_id[451].value <= threshold=0.2869153320789337
node_39: feature_name=cg18751958	feature_id[1427].value > threshold=0.4813787043094635
node_41: feature_name=cg22512847	feature_id[1152].value > threshold=0.5129324495792389
node_43: feature_name=cg23345038	feature_id[1147].value <= threshold=0.6318148374557495
Class: myxoid liposarcoma (MLS)	
Rules_12	passed counts:38
node_0: feature_name=cg11915444	feature_id[1171].value > threshold=0.3402601182460785
node_10: feature_name=cg12109728	feature_id[2810].value > threshold=0.7068270146846771
node_20: feature_name=cg16412000	feature_id[789].value > threshold=0.6741803884506226
node_24: feature_name=cg17115147	feature_id[1434].value <= threshold=0.2961975038051605
node_25: feature_name=cg24474622	feature_id[2737].value > threshold=0.7211934924125671
node_29: feature_name=cg10472711	feature_id[612].value > threshold=0.45985929667949677
node_37: feature_name=cg01937669	feature_id[188].value <= threshold=0.4809463769197464
node_38: feature_name=cg02157052	feature_id[451].value <= threshold=0.2869153320789337
node_39: feature_name=cg18751958	feature_id[1427].value > threshold=0.4813787043094635
node_41: feature_name=cg22512847	feature_id[1152].value <= threshold=0.5129324495792389
Class: dermatofibrosarcoma protuberans (DFSP)	
Rules_13	passed counts:35
node_0: feature_name=cg11915444	feature_id[1171].value > threshold=0.3402601182460785
node_10: feature_name=cg12109728	feature_id[2810].value > threshold=0.7068270146846771

node_20: feature_name=cg16412000	feature_id[789].value > threshold=0.6741803884506226
node_24: feature_name=cg17115147	feature_id[1434].value <= threshold=0.2961975038051605
node_25: feature_name=cg24474622	feature_id[2737].value > threshold=0.7211934924125671
node_29: feature_name=cg10472711	feature_id[612].value > threshold=0.45985929667949677
node_37: feature_name=cg01937669	feature_id[188].value <= threshold=0.4809463769197464
node_38: feature_name=cg02157052	feature_id[451].value <= threshold=0.2869153320789337
node_39: feature_name=cg18751958	feature_id[1427].value > threshold=0.4813787043094635
node_41: feature_name=cg22512847	feature_id[1152].value > threshold=0.5129324495792389
node_43: feature_name=cg23345038	feature_id[1147].value > threshold=0.6318148374557495
node_45: feature_name=cg17439009	feature_id[1815].value <= threshold=0.48514117300510406
node_46: feature_name=cg17537493	feature_id[2772].value > threshold=0.5825372636318207
node_52: feature_name=cg01683570	feature_id[110].value > threshold=0.6040188074111938
node_54: feature_name=cg15720017	feature_id[168].value > threshold=0.7067741453647614
node_60: feature_name=cg23690444	feature_id[120].value <= threshold=0.16037724912166595
node_61: feature_name=cg00567872	feature_id[236].value > threshold=0.7005765736103058
node_85: feature_name=cg01014262	feature_id[764].value <= threshold=0.5737917125225067
node_86: feature_name=cg22946562	feature_id[1148].value <= threshold=0.6328203976154327
node_87: feature_name=cg18120975	feature_id[2767].value > threshold=0.7081544995307922
node_91: feature_name=cg17171539	feature_id[25].value <= threshold=0.501024603843689
node_92: feature_name=cg00007036	feature_id[1586].value > threshold=0.943472146987915
node_104: feature_name=cg14156441	feature_id[3273].value <= threshold=0.24960360676050186
node_105: feature_name=cg10885338	feature_id[3603].value <= threshold=0.7335180938243866

Class: undifferentiated sarcoma (USARC)	
Rules_14	passed counts:30
node_0: feature_name=cg11915444	feature_id[1171].value > threshold=0.3402601182460785
node_10: feature_name=cg12109728	feature_id[2810].value > threshold=0.7068270146846771
node_20: feature_name=cg16412000	feature_id[789].value > threshold=0.6741803884506226
node_24: feature_name=cg17115147	feature_id[1434].value <= threshold=0.2961975038051605
node_25: feature_name=cg24474622	feature_id[2737].value > threshold=0.7211934924125671
node_29: feature_name=cg10472711	feature_id[612].value > threshold=0.45985929667949677
node_37: feature_name=cg01937669	feature_id[188].value <= threshold=0.4809463769197464
node_38: feature_name=cg02157052	feature_id[451].value <= threshold=0.2869153320789337
node_39: feature_name=cg18751958	feature_id[1427].value > threshold=0.4813787043094635
node_41: feature_name=cg22512847	feature_id[1152].value > threshold=0.5129324495792389
node_43: feature_name=cg23345038	feature_id[1147].value > threshold=0.6318148374557495
node_45: feature_name=cg17439009	feature_id[1815].value <= threshold=0.48514117300510406
node_46: feature_name=cg17537493	feature_id[2772].value > threshold=0.5825372636318207
node_52: feature_name=cg01683570	feature_id[110].value <= threshold=0.6040188074111938
Class: schwannoma (SWN)	
Rules_15	passed counts:29
node_0: feature_name=cg11915444	feature_id[1171].value > threshold=0.3402601182460785
node_10: feature_name=cg12109728	feature_id[2810].value > threshold=0.7068270146846771
node_20: feature_name=cg16412000	feature_id[789].value > threshold=0.6741803884506226
node_24: feature_name=cg17115147	feature_id[1434].value <= threshold=0.2961975038051605

node_25: feature_name=cg24474622	feature_id[2737].value > threshold=0.7211934924125671
node_29: feature_name=cg10472711	feature_id[612].value > threshold=0.4598592966794967 7
node_37: feature_name=cg01937669	feature_id[188].value <= threshold=0.4809463769197464
node_38: feature_name=cg02157052	feature_id[451].value <= threshold=0.2869153320789337
node_39: feature_name=cg18751958	feature_id[1427].value > threshold=0.4813787043094635
node_41: feature_name=cg22512847	feature_id[1152].value > threshold=0.5129324495792389
node_43: feature_name=cg23345038	feature_id[1147].value > threshold=0.6318148374557495
node_45: feature_name=cg17439009	feature_id[1815].value <= threshold=0.48514117300510406
node_46: feature_name=cg17537493	feature_id[2772].value > threshold=0.5825372636318207
node_52: feature_name=cg01683570	feature_id[110].value > threshold=0.6040188074111938
node_54: feature_name=cg15720017	feature_id[168].value > threshold=0.7067741453647614
node_60: feature_name=cg23690444	feature_id[120].value <= threshold=0.16037724912166595
node_61: feature_name=cg00567872	feature_id[236].value > threshold=0.7005765736103058
node_85: feature_name=cg01014262	feature_id[764].value <= threshold=0.5737917125225067
node_86: feature_name=cg22946562	feature_id[1148].value > threshold=0.6328203976154327
node_134: feature_name=cg14514032	feature_id[202].value > threshold=0.4389604330062866
node_312: feature_name=cg21670987	feature_id[1509].value > threshold=0.32477423548698425
node_324: feature_name=cg10520924	feature_id[1943].value <= threshold=0.8430010974407196
Class: malignant peripheral nerve sheath tumour (MPNST)	
Rules_16	passed counts:27
node_0: feature_name=cg11915444	feature_id[1171].value > threshold=0.3402601182460785

node_10: feature_name=cg12109728	feature_id[2810].value > threshold=0.7068270146846771
node_20: feature_name=cg16412000	feature_id[789].value > threshold=0.6741803884506226
node_24: feature_name=cg17115147	feature_id[1434].value <= threshold=0.2961975038051605
node_25: feature_name=cg24474622	feature_id[2737].value > threshold=0.7211934924125671
node_29: feature_name=cg10472711	feature_id[612].value > threshold=0.45985929667949677
node_37: feature_name=cg01937669	feature_id[188].value <= threshold=0.4809463769197464
node_38: feature_name=cg02157052	feature_id[451].value <= threshold=0.2869153320789337
node_39: feature_name=cg18751958	feature_id[1427].value > threshold=0.4813787043094635
node_41: feature_name=cg22512847	feature_id[1152].value > threshold=0.5129324495792389
node_43: feature_name=cg23345038	feature_id[1147].value > threshold=0.6318148374557495
node_45: feature_name=cg17439009	feature_id[1815].value <= threshold=0.48514117300510406
node_46: feature_name=cg17537493	feature_id[2772].value > threshold=0.5825372636318207
node_52: feature_name=cg01683570	feature_id[110].value > threshold=0.6040188074111938
node_54: feature_name=cg15720017	feature_id[168].value > threshold=0.7067741453647614
node_60: feature_name=cg23690444	feature_id[120].value <= threshold=0.16037724912166595
node_61: feature_name=cg00567872	feature_id[236].value <= threshold=0.7005765736103058
node_62: feature_name=cg09293488	feature_id[2629].value <= threshold=0.839823454618454
node_63: feature_name=cg19201770	feature_id[2194].value > threshold=0.5603366196155548
Class: alveolar soft part sarcoma (ASPS)	
Rules_17	passed counts:27
node_0: feature_name=cg11915444	feature_id[1171].value > threshold=0.3402601182460785
node_10: feature_name=cg12109728	feature_id[2810].value > threshold=0.7068270146846771

node_20: feature_name=cg16412000	feature_id[789].value > threshold=0.6741803884506226
node_24: feature_name=cg17115147	feature_id[1434].value <= threshold=0.2961975038051605
node_25: feature_name=cg24474622	feature_id[2737].value > threshold=0.7211934924125671
node_29: feature_name=cg10472711	feature_id[612].value > threshold=0.4598592966794967 7
node_37: feature_name=cg01937669	feature_id[188].value <= threshold=0.4809463769197464
node_38: feature_name=cg02157052	feature_id[451].value <= threshold=0.2869153320789337
node_39: feature_name=cg18751958	feature_id[1427].value > threshold=0.4813787043094635
node_41: feature_name=cg22512847	feature_id[1152].value > threshold=0.5129324495792389
node_43: feature_name=cg23345038	feature_id[1147].value > threshold=0.6318148374557495
node_45: feature_name=cg17439009	feature_id[1815].value <= threshold=0.48514117300510406
node_46: feature_name=cg17537493	feature_id[2772].value > threshold=0.5825372636318207
node_52: feature_name=cg01683570	feature_id[110].value > threshold=0.6040188074111938
node_54: feature_name=cg15720017	feature_id[168].value <= threshold=0.7067741453647614
node_55: feature_name=cg17216243	feature_id[1006].value > threshold=0.48612095415592194
Class: solitary fibrous tumour (SFT)	
Rules_18	passed counts:25
node_0: feature_name=cg11915444	feature_id[1171].value > threshold=0.3402601182460785
node_10: feature_name=cg12109728	feature_id[2810].value > threshold=0.7068270146846771
node_20: feature_name=cg16412000	feature_id[789].value > threshold=0.6741803884506226
node_24: feature_name=cg17115147	feature_id[1434].value <= threshold=0.2961975038051605
node_25: feature_name=cg24474622	feature_id[2737].value > threshold=0.7211934924125671
node_29: feature_name=cg10472711	feature_id[612].value > threshold=0.45985929667949677

node_37: feature_name=cg01937669	feature_id[188].value <= threshold=0.4809463769197464
node_38: feature_name=cg02157052	feature_id[451].value <= threshold=0.2869153320789337
node_39: feature_name=cg18751958	feature_id[1427].value > threshold=0.4813787043094635
node_41: feature_name=cg22512847	feature_id[1152].value > threshold=0.5129324495792389
node_43: feature_name=cg23345038	feature_id[1147].value > threshold=0.6318148374557495
node_45: feature_name=cg17439009	feature_id[1815].value <= threshold=0.48514117300510406
node_46: feature_name=cg17537493	feature_id[2772].value > threshold=0.5825372636318207
node_52: feature_name=cg01683570	feature_id[110].value > threshold=0.6040188074111938
node_54: feature_name=cg15720017	feature_id[168].value > threshold=0.7067741453647614
node_60: feature_name=cg23690444	feature_id[120].value > threshold=0.16037724912166595
Class: epithelioid sarcoma (ES)	
Rules_19	passed counts:21
node_0: feature_name=cg11915444	feature_id[1171].value > threshold=0.3402601182460785
node_10: feature_name=cg12109728	feature_id[2810].value > threshold=0.7068270146846771
node_20: feature_name=cg16412000	feature_id[789].value > threshold=0.6741803884506226
node_24: feature_name=cg17115147	feature_id[1434].value <= threshold=0.2961975038051605
node_25: feature_name=cg24474622	feature_id[2737].value > threshold=0.7211934924125671
node_29: feature_name=cg10472711	feature_id[612].value > threshold=0.45985929667949677
node_37: feature_name=cg01937669	feature_id[188].value <= threshold=0.4809463769197464
node_38: feature_name=cg02157052	feature_id[451].value <= threshold=0.2869153320789337
node_39: feature_name=cg18751958	feature_id[1427].value > threshold=0.4813787043094635
node_41: feature_name=cg22512847	feature_id[1152].value > threshold=0.5129324495792389

node_43: feature_name=cg23345038	feature_id[1147].value > threshold=0.6318148374557495
node_45: feature_name=cg17439009	feature_id[1815].value <= threshold=0.48514117300510406
node_46: feature_name=cg17537493	feature_id[2772].value > threshold=0.5825372636318207
node_52: feature_name=cg01683570	feature_id[110].value > threshold=0.6040188074111938
node_54: feature_name=cg15720017	feature_id[168].value > threshold=0.7067741453647614
node_60: feature_name=cg23690444	feature_id[120].value <= threshold=0.16037724912166595
node_61: feature_name=cg00567872	feature_id[236].value > threshold=0.7005765736103058
node_85: feature_name=cg01014262	feature_id[764].value <= threshold=0.5737917125225067
node_86: feature_name=cg22946562	feature_id[1148].value > threshold=0.6328203976154327
node_134: feature_name=cg14514032	feature_id[202].value <= threshold=0.4389604330062866
node_135: feature_name=cg13488220	feature_id[241].value > threshold=0.7352153658866882
node_143: feature_name=cg03840920	feature_id[256].value > threshold=0.502630889415741
node_147: feature_name=cg13634090	feature_id[822].value > threshold=0.7207068204879761
node_151: feature_name=cg19584674	feature_id[2364].value <= threshold=0.41758519411087036
node_152: feature_name=cg07344990	feature_id[411].value > threshold=0.5484964847564697
node_158: feature_name=cg10298992	feature_id[3066].value <= threshold=0.5707973837852478
node_159: feature_name=cg24852135	feature_id[47].value > threshold=0.8445721566677094
node_171: feature_name=cg06474428	feature_id[3652].value <= threshold=0.9347167015075684
Class: well differentiated liposarcoma (WDLS)/dedifferentiated liposarcoma (DDLS)	
Rules_20	passed counts:18
node_0: feature_name=cg11915444	feature_id[1171].value > threshold=0.3402601182460785

node_10: feature_name=cg12109728	feature_id[2810].value > threshold=0.7068270146846771
node_20: feature_name=cg16412000	feature_id[789].value > threshold=0.6741803884506226
node_24: feature_name=cg17115147	feature_id[1434].value <= threshold=0.2961975038051605
node_25: feature_name=cg24474622	feature_id[2737].value > threshold=0.7211934924125671
node_29: feature_name=cg10472711	feature_id[612].value > threshold=0.45985929667949677
node_37: feature_name=cg01937669	feature_id[188].value <= threshold=0.4809463769197464
node_38: feature_name=cg02157052	feature_id[451].value <= threshold=0.2869153320789337
node_39: feature_name=cg18751958	feature_id[1427].value > threshold=0.4813787043094635
node_41: feature_name=cg22512847	feature_id[1152].value > threshold=0.5129324495792389
node_43: feature_name=cg23345038	feature_id[1147].value > threshold=0.6318148374557495
node_45: feature_name=cg17439009	feature_id[1815].value <= threshold=0.48514117300510406
node_46: feature_name=cg17537493	feature_id[2772].value > threshold=0.5825372636318207
node_52: feature_name=cg01683570	feature_id[110].value > threshold=0.6040188074111938
node_54: feature_name=cg15720017	feature_id[168].value > threshold=0.7067741453647614
node_60: feature_name=cg23690444	feature_id[120].value <= threshold=0.16037724912166595
node_61: feature_name=cg00567872	feature_id[236].value > threshold=0.7005765736103058
node_85: feature_name=cg01014262	feature_id[764].value > threshold=0.5737917125225067
node_331: feature_name=cg16897462	feature_id[3257].value > threshold=0.24157879501581192
Class: malignant rhabdoid tumour (MRT)	
Rules_21	passed counts:16
node_0: feature_name=cg11915444	feature_id[1171].value > threshold=0.3402601182460785
node_10: feature_name=cg12109728	feature_id[2810].value > threshold=0.7068270146846771

node_20: feature_name=cg16412000	feature_id[789].value > threshold=0.6741803884506226
node_24: feature_name=cg17115147	feature_id[1434].value <= threshold=0.2961975038051605
node_25: feature_name=cg24474622	feature_id[2737].value > threshold=0.7211934924125671
node_29: feature_name=cg10472711	feature_id[612].value > threshold=0.4598592966794967 7
node_37: feature_name=cg01937669	feature_id[188].value <= threshold=0.4809463769197464
node_38: feature_name=cg02157052	feature_id[451].value <= threshold=0.2869153320789337
node_39: feature_name=cg18751958	feature_id[1427].value > threshold=0.4813787043094635
node_41: feature_name=cg22512847	feature_id[1152].value > threshold=0.5129324495792389
node_43: feature_name=cg23345038	feature_id[1147].value > threshold=0.6318148374557495
node_45: feature_name=cg17439009	feature_id[1815].value <= threshold=0.48514117300510406
node_46: feature_name=cg17537493	feature_id[2772].value > threshold=0.5825372636318207
node_52: feature_name=cg01683570	feature_id[110].value > threshold=0.6040188074111938
node_54: feature_name=cg15720017	feature_id[168].value > threshold=0.7067741453647614
node_60: feature_name=cg23690444	feature_id[120].value <= threshold=0.16037724912166595
node_61: feature_name=cg00567872	feature_id[236].value > threshold=0.7005765736103058
node_85: feature_name=cg01014262	feature_id[764].value <= threshold=0.5737917125225067
node_86: feature_name=cg22946562	feature_id[1148].value > threshold=0.6328203976154327
node_134: feature_name=cg14514032	feature_id[202].value <= threshold=0.4389604330062866
node_135: feature_name=cg13488220	feature_id[241].value <= threshold=0.7352153658866882
node_136: feature_name=cg06132803	feature_id[3655].value > threshold=0.29061736911535263
Class: angioleiomyoma (ALMO)/myopericytoma (MPC)	

Rules_22	passed counts:13
node_0: feature_name=cg11915444	feature_id[1171].value > threshold=0.3402601182460785
node_10: feature_name=cg12109728	feature_id[2810].value > threshold=0.7068270146846771
node_20: feature_name=cg16412000	feature_id[789].value > threshold=0.6741803884506226
node_24: feature_name=cg17115147	feature_id[1434].value <= threshold=0.2961975038051605
node_25: feature_name=cg24474622	feature_id[2737].value > threshold=0.7211934924125671
node_29: feature_name=cg10472711	feature_id[612].value > threshold=0.45985929667949677
node_37: feature_name=cg01937669	feature_id[188].value <= threshold=0.4809463769197464
node_38: feature_name=cg02157052	feature_id[451].value <= threshold=0.2869153320789337
node_39: feature_name=cg18751958	feature_id[1427].value > threshold=0.4813787043094635
node_41: feature_name=cg22512847	feature_id[1152].value > threshold=0.5129324495792389
node_43: feature_name=cg23345038	feature_id[1147].value > threshold=0.6318148374557495
node_45: feature_name=cg17439009	feature_id[1815].value <= threshold=0.48514117300510406
node_46: feature_name=cg17537493	feature_id[2772].value > threshold=0.5825372636318207
node_52: feature_name=cg01683570	feature_id[110].value > threshold=0.6040188074111938
node_54: feature_name=cg15720017	feature_id[168].value > threshold=0.7067741453647614
node_60: feature_name=cg23690444	feature_id[120].value <= threshold=0.16037724912166595
node_61: feature_name=cg00567872	feature_id[236].value > threshold=0.7005765736103058
node_85: feature_name=cg01014262	feature_id[764].value <= threshold=0.5737917125225067
node_86: feature_name=cg22946562	feature_id[1148].value > threshold=0.6328203976154327
node_134: feature_name=cg14514032	feature_id[202].value <= threshold=0.4389604330062866
node_135: feature_name=cg13488220	feature_id[241].value > threshold=0.7352153658866882

node_143: feature_name=cg03840920	feature_id[256].value > threshold=0.502630889415741
node_147: feature_name=cg13634090	feature_id[822].value > threshold=0.7207068204879761
node_151: feature_name=cg19584674	feature_id[2364].value > threshold=0.41758519411087036
node_307: feature_name=cg07795968	feature_id[2451].value <= threshold=0.7859841883182526
Class: small blue round cell tumour (SBRCT)	
Rules_23	passed counts:13
node_0: feature_name=cg11915444	feature_id[1171].value > threshold=0.3402601182460785
node_10: feature_name=cg12109728	feature_id[2810].value > threshold=0.7068270146846771
node_20: feature_name=cg16412000	feature_id[789].value > threshold=0.6741803884506226
node_24: feature_name=cg17115147	feature_id[1434].value <= threshold=0.2961975038051605
node_25: feature_name=cg24474622	feature_id[2737].value > threshold=0.7211934924125671
node_29: feature_name=cg10472711	feature_id[612].value > threshold=0.45985929667949677
node_37: feature_name=cg01937669	feature_id[188].value <= threshold=0.4809463769197464
node_38: feature_name=cg02157052	feature_id[451].value <= threshold=0.2869153320789337
node_39: feature_name=cg18751958	feature_id[1427].value > threshold=0.4813787043094635
node_41: feature_name=cg22512847	feature_id[1152].value > threshold=0.5129324495792389
node_43: feature_name=cg23345038	feature_id[1147].value > threshold=0.6318148374557495
node_45: feature_name=cg17439009	feature_id[1815].value <= threshold=0.48514117300510406
node_46: feature_name=cg17537493	feature_id[2772].value > threshold=0.5825372636318207
node_52: feature_name=cg01683570	feature_id[110].value > threshold=0.6040188074111938
node_54: feature_name=cg15720017	feature_id[168].value > threshold=0.7067741453647614
node_60: feature_name=cg23690444	feature_id[120].value <= threshold=0.16037724912166595

node_61: feature_name=cg00567872	feature_id[236].value > threshold=0.7005765736103058
node_85: feature_name=cg01014262	feature_id[764].value <= threshold=0.5737917125225067
node_86: feature_name=cg22946562	feature_id[1148].value > threshold=0.6328203976154327
node_134: feature_name=cg14514032	feature_id[202].value <= threshold=0.4389604330062866
node_135: feature_name=cg13488220	feature_id[241].value > threshold=0.7352153658866882
node_143: feature_name=cg03840920	feature_id[256].value > threshold=0.502630889415741
node_147: feature_name=cg13634090	feature_id[822].value > threshold=0.7207068204879761
node_151: feature_name=cg19584674	feature_id[2364].value <= threshold=0.41758519411087036
node_152: feature_name=cg07344990	feature_id[411].value <= threshold=0.5484964847564697
node_153: feature_name=cg18369990	feature_id[2197].value > threshold=0.621816486120224
Class: low-grade endometrial stromal sarcoma (ESS (LG))	
Rules_24	passed counts:13
node_0: feature_name=cg11915444	feature_id[1171].value > threshold=0.3402601182460785
node_10: feature_name=cg12109728	feature_id[2810].value > threshold=0.7068270146846771
node_20: feature_name=cg16412000	feature_id[789].value > threshold=0.6741803884506226
node_24: feature_name=cg17115147	feature_id[1434].value <= threshold=0.2961975038051605
node_25: feature_name=cg24474622	feature_id[2737].value > threshold=0.7211934924125671
node_29: feature_name=cg10472711	feature_id[612].value > threshold=0.45985929667949677
node_37: feature_name=cg01937669	feature_id[188].value <= threshold=0.4809463769197464
node_38: feature_name=cg02157052	feature_id[451].value <= threshold=0.2869153320789337
node_39: feature_name=cg18751958	feature_id[1427].value > threshold=0.4813787043094635

node_41: feature_name=cg22512847	feature_id[1152].value > threshold=0.5129324495792389
node_43: feature_name=cg23345038	feature_id[1147].value > threshold=0.6318148374557495
node_45: feature_name=cg17439009	feature_id[1815].value <= threshold=0.48514117300510406
node_46: feature_name=cg17537493	feature_id[2772].value > threshold=0.5825372636318207
node_52: feature_name=cg01683570	feature_id[110].value > threshold=0.6040188074111938
node_54: feature_name=cg15720017	feature_id[168].value > threshold=0.7067741453647614
node_60: feature_name=cg23690444	feature_id[120].value <= threshold=0.16037724912166595
node_61: feature_name=cg00567872	feature_id[236].value > threshold=0.7005765736103058
node_85: feature_name=cg01014262	feature_id[764].value <= threshold=0.5737917125225067
node_86: feature_name=cg22946562	feature_id[1148].value > threshold=0.6328203976154327
node_134: feature_name=cg14514032	feature_id[202].value <= threshold=0.4389604330062866
node_135: feature_name=cg13488220	feature_id[241].value > threshold=0.7352153658866882
node_143: feature_name=cg03840920	feature_id[256].value <= threshold=0.502630889415741
node_144: feature_name=cg12669088	feature_id[444].value <= threshold=0.8584775626659393
Class: desmoid-type fibromatosis (DTFM)	
Rules_25	passed counts:13
node_0: feature_name=cg11915444	feature_id[1171].value > threshold=0.3402601182460785
node_10: feature_name=cg12109728	feature_id[2810].value > threshold=0.7068270146846771
node_20: feature_name=cg16412000	feature_id[789].value > threshold=0.6741803884506226
node_24: feature_name=cg17115147	feature_id[1434].value <= threshold=0.2961975038051605
node_25: feature_name=cg24474622	feature_id[2737].value > threshold=0.7211934924125671
node_29: feature_name=cg10472711	feature_id[612].value > threshold=0.45985929667949677

node_37: feature_name=cg01937669	feature_id[188].value <= threshold=0.4809463769197464
node_38: feature_name=cg02157052	feature_id[451].value <= threshold=0.2869153320789337
node_39: feature_name=cg18751958	feature_id[1427].value > threshold=0.4813787043094635
node_41: feature_name=cg22512847	feature_id[1152].value > threshold=0.5129324495792389
node_43: feature_name=cg23345038	feature_id[1147].value > threshold=0.6318148374557495
node_45: feature_name=cg17439009	feature_id[1815].value <= threshold=0.48514117300510406
node_46: feature_name=cg17537493	feature_id[2772].value > threshold=0.5825372636318207
node_52: feature_name=cg01683570	feature_id[110].value > threshold=0.6040188074111938
node_54: feature_name=cg15720017	feature_id[168].value > threshold=0.7067741453647614
node_60: feature_name=cg23690444	feature_id[120].value <= threshold=0.16037724912166595
node_61: feature_name=cg00567872	feature_id[236].value > threshold=0.7005765736103058
node_85: feature_name=cg01014262	feature_id[764].value <= threshold=0.5737917125225067
node_86: feature_name=cg22946562	feature_id[1148].value <= threshold=0.6328203976154327
node_87: feature_name=cg18120975	feature_id[2767].value <= threshold=0.7081544995307922
node_88: feature_name=cg15592324	feature_id[383].value <= threshold=0.9721506536006927
Class: high-grade conventional osteosarcoma (OS (HG))	
Rules_26	passed counts:12
node_0: feature_name=cg11915444	feature_id[1171].value > threshold=0.3402601182460785
node_10: feature_name=cg12109728	feature_id[2810].value > threshold=0.7068270146846771
node_20: feature_name=cg16412000	feature_id[789].value > threshold=0.6741803884506226
node_24: feature_name=cg17115147	feature_id[1434].value <= threshold=0.2961975038051605

node_25: feature_name=cg24474622	feature_id[2737].value > threshold=0.7211934924125671
node_29: feature_name=cg10472711	feature_id[612].value > threshold=0.45985929667949677
node_37: feature_name=cg01937669	feature_id[188].value <= threshold=0.4809463769197464
node_38: feature_name=cg02157052	feature_id[451].value <= threshold=0.2869153320789337
node_39: feature_name=cg18751958	feature_id[1427].value > threshold=0.4813787043094635
node_41: feature_name=cg22512847	feature_id[1152].value > threshold=0.5129324495792389
node_43: feature_name=cg23345038	feature_id[1147].value > threshold=0.6318148374557495
node_45: feature_name=cg17439009	feature_id[1815].value <= threshold=0.48514117300510406
node_46: feature_name=cg17537493	feature_id[2772].value > threshold=0.5825372636318207
node_52: feature_name=cg01683570	feature_id[110].value > threshold=0.6040188074111938
node_54: feature_name=cg15720017	feature_id[168].value > threshold=0.7067741453647614
node_60: feature_name=cg23690444	feature_id[120].value <= threshold=0.16037724912166595
node_61: feature_name=cg00567872	feature_id[236].value > threshold=0.7005765736103058
node_85: feature_name=cg01014262	feature_id[764].value <= threshold=0.5737917125225067
node_86: feature_name=cg22946562	feature_id[1148].value > threshold=0.6328203976154327
node_134: feature_name=cg14514032	feature_id[202].value <= threshold=0.4389604330062866
node_135: feature_name=cg13488220	feature_id[241].value > threshold=0.7352153658866882
node_143: feature_name=cg03840920	feature_id[256].value > threshold=0.502630889415741
node_147: feature_name=cg13634090	feature_id[822].value > threshold=0.7207068204879761
node_151: feature_name=cg19584674	feature_id[2364].value <= threshold=0.41758519411087036
node_152: feature_name=cg07344990	feature_id[411].value > threshold=0.5484964847564697
node_158: feature_name=cg10298992	feature_id[3066].value > threshold=0.5707973837852478

node_174: feature_name=cg11843516	feature_id[20].value <= threshold=0.5590928494930267
node_175: feature_name=cg05185926	feature_id[448].value > threshold=0.17318664491176605
node_177: feature_name=cg17009731	feature_id[1220].value > threshold=0.6666513085365295
node_179: feature_name=cg01380319	feature_id[515].value <= threshold=0.39251960813999176
node_180: feature_name=cg20700977	feature_id[303].value > threshold=0.8422040641307831
node_184: feature_name=cg24259291	feature_id[164].value > threshold=0.4872268736362457
node_188: feature_name=cg08097657	feature_id[67].value > threshold=0.3371318429708481
node_190: feature_name=cg07891483	feature_id[59].value > threshold=0.6211732923984528
node_194: feature_name=cg20122645	feature_id[275].value > threshold=0.6094755828380585
node_196: feature_name=cg04026354	feature_id[1763].value > threshold=0.4341808259487152
node_286: feature_name=cg25408950	feature_id[772].value <= threshold=0.680083692073822
node_287: feature_name=cg17216243	feature_id[1006].value <= threshold=0.6291826963424683
Class: inflammatory myofibroblastic tumour (IMT)	
Rules_27	passed counts:12
node_0: feature_name=cg11915444	feature_id[1171].value > threshold=0.3402601182460785
node_10: feature_name=cg12109728	feature_id[2810].value > threshold=0.7068270146846771
node_20: feature_name=cg16412000	feature_id[789].value > threshold=0.6741803884506226
node_24: feature_name=cg17115147	feature_id[1434].value <= threshold=0.2961975038051605
node_25: feature_name=cg24474622	feature_id[2737].value > threshold=0.7211934924125671
node_29: feature_name=cg10472711	feature_id[612].value > threshold=0.45985929667949677
node_37: feature_name=cg01937669	feature_id[188].value <= threshold=0.4809463769197464

node_38: feature_name=cg02157052	feature_id[451].value <= threshold=0.2869153320789337
node_39: feature_name=cg18751958	feature_id[1427].value > threshold=0.4813787043094635
node_41: feature_name=cg22512847	feature_id[1152].value > threshold=0.5129324495792389
node_43: feature_name=cg23345038	feature_id[1147].value > threshold=0.6318148374557495
node_45: feature_name=cg17439009	feature_id[1815].value <= threshold=0.48514117300510406
node_46: feature_name=cg17537493	feature_id[2772].value > threshold=0.5825372636318207
node_52: feature_name=cg01683570	feature_id[110].value > threshold=0.6040188074111938
node_54: feature_name=cg15720017	feature_id[168].value > threshold=0.7067741453647614
node_60: feature_name=cg23690444	feature_id[120].value <= threshold=0.16037724912166595
node_61: feature_name=cg00567872	feature_id[236].value > threshold=0.7005765736103058
node_85: feature_name=cg01014262	feature_id[764].value <= threshold=0.5737917125225067
node_86: feature_name=cg22946562	feature_id[1148].value > threshold=0.6328203976154327
node_134: feature_name=cg14514032	feature_id[202].value <= threshold=0.4389604330062866
node_135: feature_name=cg13488220	feature_id[241].value > threshold=0.7352153658866882
node_143: feature_name=cg03840920	feature_id[256].value > threshold=0.502630889415741
node_147: feature_name=cg13634090	feature_id[822].value > threshold=0.7207068204879761
node_151: feature_name=cg19584674	feature_id[2364].value <= threshold=0.41758519411087036
node_152: feature_name=cg07344990	feature_id[411].value > threshold=0.5484964847564697
node_158: feature_name=cg10298992	feature_id[3066].value > threshold=0.5707973837852478
node_174: feature_name=cg11843516	feature_id[20].value <= threshold=0.5590928494930267
node_175: feature_name=cg05185926	feature_id[448].value > threshold=0.17318664491176605
node_177: feature_name=cg17009731	feature_id[1220].value > threshold=0.6666513085365295

node_179: feature_name=cg01380319	feature_id[515].value <= threshold=0.39251960813999176
node_180: feature_name=cg20700977	feature_id[303].value > threshold=0.8422040641307831
node_184: feature_name=cg24259291	feature_id[164].value > threshold=0.4872268736362457
node_188: feature_name=cg08097657	feature_id[67].value > threshold=0.3371318429708481
node_190: feature_name=cg07891483	feature_id[59].value > threshold=0.6211732923984528
node_194: feature_name=cg20122645	feature_id[275].value > threshold=0.6094755828380585
node_196: feature_name=cg04026354	feature_id[1763].value <= threshold=0.4341808259487152
node_197: feature_name=cg10149889	feature_id[1017].value > threshold=0.6150195300579071
node_201: feature_name=cg14890730	feature_id[337].value <= threshold=0.4372602105140686
node_202: feature_name=cg08331427	feature_id[132].value > threshold=0.5443233847618103
node_208: feature_name=cg22730007	feature_id[2544].value > threshold=0.28619489073753357
node_210: feature_name=cg17193551	feature_id[2057].value > threshold=0.4393353909254074
node_212: feature_name=cg14671764	feature_id[584].value > threshold=0.9353199899196625
node_268: feature_name=cg21377260	feature_id[534].value > threshold=0.9840706288814545
node_276: feature_name=cg20934096	feature_id[251].value > threshold=0.7597180306911469
Class: undifferentiated sarcoma (USARC)	
Rules_28	passed counts:12
node_0: feature_name=cg11915444	feature_id[1171].value > threshold=0.3402601182460785
node_10: feature_name=cg12109728	feature_id[2810].value > threshold=0.7068270146846771
node_20: feature_name=cg16412000	feature_id[789].value > threshold=0.6741803884506226
node_24: feature_name=cg17115147	feature_id[1434].value <= threshold=0.2961975038051605
node_25: feature_name=cg24474622	feature_id[2737].value > threshold=0.7211934924125671

node_29: feature_name=cg10472711	feature_id[612].value > threshold=0.45985929667949677
node_37: feature_name=cg01937669	feature_id[188].value <= threshold=0.4809463769197464
node_38: feature_name=cg02157052	feature_id[451].value <= threshold=0.2869153320789337
node_39: feature_name=cg18751958	feature_id[1427].value > threshold=0.4813787043094635
node_41: feature_name=cg22512847	feature_id[1152].value > threshold=0.5129324495792389
node_43: feature_name=cg23345038	feature_id[1147].value > threshold=0.6318148374557495
node_45: feature_name=cg17439009	feature_id[1815].value <= threshold=0.48514117300510406
node_46: feature_name=cg17537493	feature_id[2772].value > threshold=0.5825372636318207
node_52: feature_name=cg01683570	feature_id[110].value > threshold=0.6040188074111938
node_54: feature_name=cg15720017	feature_id[168].value > threshold=0.7067741453647614
node_60: feature_name=cg23690444	feature_id[120].value <= threshold=0.16037724912166595
node_61: feature_name=cg00567872	feature_id[236].value > threshold=0.7005765736103058
node_85: feature_name=cg01014262	feature_id[764].value <= threshold=0.5737917125225067
node_86: feature_name=cg22946562	feature_id[1148].value > threshold=0.6328203976154327
node_134: feature_name=cg14514032	feature_id[202].value <= threshold=0.4389604330062866
node_135: feature_name=cg13488220	feature_id[241].value > threshold=0.7352153658866882
node_143: feature_name=cg03840920	feature_id[256].value > threshold=0.502630889415741
node_147: feature_name=cg13634090	feature_id[822].value <= threshold=0.7207068204879761
node_148: feature_name=cg21698310	feature_id[178].value > threshold=0.07961717247962952
Class: fibrous dysplasia (FDY)	
Rules_29	passed counts:11
node_0: feature_name=cg11915444	feature_id[1171].value > threshold=0.3402601182460785

node_10: feature_name=cg12109728	feature_id[2810].value > threshold=0.7068270146846771
node_20: feature_name=cg16412000	feature_id[789].value > threshold=0.6741803884506226
node_24: feature_name=cg17115147	feature_id[1434].value <= threshold=0.2961975038051605
node_25: feature_name=cg24474622	feature_id[2737].value > threshold=0.7211934924125671
node_29: feature_name=cg10472711	feature_id[612].value > threshold=0.45985929667949677
node_37: feature_name=cg01937669	feature_id[188].value <= threshold=0.4809463769197464
node_38: feature_name=cg02157052	feature_id[451].value <= threshold=0.2869153320789337
node_39: feature_name=cg18751958	feature_id[1427].value > threshold=0.4813787043094635
node_41: feature_name=cg22512847	feature_id[1152].value > threshold=0.5129324495792389
node_43: feature_name=cg23345038	feature_id[1147].value > threshold=0.6318148374557495
node_45: feature_name=cg17439009	feature_id[1815].value <= threshold=0.48514117300510406
node_46: feature_name=cg17537493	feature_id[2772].value > threshold=0.5825372636318207
node_52: feature_name=cg01683570	feature_id[110].value > threshold=0.6040188074111938
node_54: feature_name=cg15720017	feature_id[168].value > threshold=0.7067741453647614
node_60: feature_name=cg23690444	feature_id[120].value <= threshold=0.16037724912166595
node_61: feature_name=cg00567872	feature_id[236].value > threshold=0.7005765736103058
node_85: feature_name=cg01014262	feature_id[764].value <= threshold=0.5737917125225067
node_86: feature_name=cg22946562	feature_id[1148].value > threshold=0.6328203976154327
node_134: feature_name=cg14514032	feature_id[202].value > threshold=0.4389604330062866
node_312: feature_name=cg21670987	feature_id[1509].value <= threshold=0.32477423548698425
node_313: feature_name=cg12939390	feature_id[143].value <= threshold=0.23070813715457916
Class: clear cell sarcoma of the kidney (CCSK)	

Rules_30	passed counts:11
node_0: feature_name=cg11915444	feature_id[1171].value > threshold=0.3402601182460785
node_10: feature_name=cg12109728	feature_id[2810].value > threshold=0.7068270146846771
node_20: feature_name=cg16412000	feature_id[789].value > threshold=0.6741803884506226
node_24: feature_name=cg17115147	feature_id[1434].value <= threshold=0.2961975038051605
node_25: feature_name=cg24474622	feature_id[2737].value > threshold=0.7211934924125671
node_29: feature_name=cg10472711	feature_id[612].value > threshold=0.45985929667949677
node_37: feature_name=cg01937669	feature_id[188].value <= threshold=0.4809463769197464
node_38: feature_name=cg02157052	feature_id[451].value <= threshold=0.2869153320789337
node_39: feature_name=cg18751958	feature_id[1427].value > threshold=0.4813787043094635
node_41: feature_name=cg22512847	feature_id[1152].value > threshold=0.5129324495792389
node_43: feature_name=cg23345038	feature_id[1147].value > threshold=0.6318148374557495
node_45: feature_name=cg17439009	feature_id[1815].value <= threshold=0.48514117300510406
node_46: feature_name=cg17537493	feature_id[2772].value > threshold=0.5825372636318207
node_52: feature_name=cg01683570	feature_id[110].value > threshold=0.6040188074111938
node_54: feature_name=cg15720017	feature_id[168].value > threshold=0.7067741453647614
node_60: feature_name=cg23690444	feature_id[120].value <= threshold=0.16037724912166595
node_61: feature_name=cg00567872	feature_id[236].value > threshold=0.7005765736103058
node_85: feature_name=cg01014262	feature_id[764].value <= threshold=0.5737917125225067
node_86: feature_name=cg22946562	feature_id[1148].value > threshold=0.6328203976154327
node_134: feature_name=cg14514032	feature_id[202].value <= threshold=0.4389604330062866

node_135: feature_name=cg13488220	feature_id[241].value > threshold=0.7352153658866882
node_143: feature_name=cg03840920	feature_id[256].value > threshold=0.502630889415741
node_147: feature_name=cg13634090	feature_id[822].value > threshold=0.7207068204879761
node_151: feature_name=cg19584674	feature_id[2364].value <= threshold=0.41758519411087036
node_152: feature_name=cg07344990	feature_id[411].value > threshold=0.5484964847564697
node_158: feature_name=cg10298992	feature_id[3066].value > threshold=0.5707973837852478
node_174: feature_name=cg11843516	feature_id[20].value > threshold=0.5590928494930267
Class: ossifying fibromyxoid tumour (OFMT)	
Rules_31	passed counts:11
node_0: feature_name=cg11915444	feature_id[1171].value > threshold=0.3402601182460785
node_10: feature_name=cg12109728	feature_id[2810].value > threshold=0.7068270146846771
node_20: feature_name=cg16412000	feature_id[789].value > threshold=0.6741803884506226
node_24: feature_name=cg17115147	feature_id[1434].value <= threshold=0.2961975038051605
node_25: feature_name=cg24474622	feature_id[2737].value > threshold=0.7211934924125671
node_29: feature_name=cg10472711	feature_id[612].value > threshold=0.45985929667949677
node_37: feature_name=cg01937669	feature_id[188].value <= threshold=0.4809463769197464
node_38: feature_name=cg02157052	feature_id[451].value <= threshold=0.2869153320789337
node_39: feature_name=cg18751958	feature_id[1427].value > threshold=0.4813787043094635
node_41: feature_name=cg22512847	feature_id[1152].value > threshold=0.5129324495792389
node_43: feature_name=cg23345038	feature_id[1147].value > threshold=0.6318148374557495
node_45: feature_name=cg17439009	feature_id[1815].value <= threshold=0.48514117300510406
node_46: feature_name=cg17537493	feature_id[2772].value > threshold=0.5825372636318207

node_52: feature_name=cg01683570	feature_id[110].value > threshold=0.6040188074111938
node_54: feature_name=cg15720017	feature_id[168].value > threshold=0.7067741453647614
node_60: feature_name=cg23690444	feature_id[120].value <= threshold=0.16037724912166595
node_61: feature_name=cg00567872	feature_id[236].value > threshold=0.7005765736103058
node_85: feature_name=cg01014262	feature_id[764].value <= threshold=0.5737917125225067
node_86: feature_name=cg22946562	feature_id[1148].value > threshold=0.6328203976154327
node_134: feature_name=cg14514032	feature_id[202].value <= threshold=0.4389604330062866
node_135: feature_name=cg13488220	feature_id[241].value > threshold=0.7352153658866882
node_143: feature_name=cg03840920	feature_id[256].value > threshold=0.502630889415741
node_147: feature_name=cg13634090	feature_id[822].value > threshold=0.7207068204879761
node_151: feature_name=cg19584674	feature_id[2364].value <= threshold=0.41758519411087036
node_152: feature_name=cg07344990	feature_id[411].value > threshold=0.5484964847564697
node_158: feature_name=cg10298992	feature_id[3066].value > threshold=0.5707973837852478
node_174: feature_name=cg11843516	feature_id[20].value <= threshold=0.5590928494930267
node_175: feature_name=cg05185926	feature_id[448].value > threshold=0.17318664491176605
node_177: feature_name=cg17009731	feature_id[1220].value > threshold=0.6666513085365295
node_179: feature_name=cg01380319	feature_id[515].value <= threshold=0.39251960813999176
node_180: feature_name=cg20700977	feature_id[303].value > threshold=0.8422040641307831
node_184: feature_name=cg24259291	feature_id[164].value > threshold=0.4872268736362457
node_188: feature_name=cg08097657	feature_id[67].value > threshold=0.3371318429708481
node_190: feature_name=cg07891483	feature_id[59].value > threshold=0.6211732923984528
node_194: feature_name=cg20122645	feature_id[275].value > threshold=0.6094755828380585

node_196: feature_name=cg04026354	feature_id[1763].value > threshold=0.4341808259487152
node_286: feature_name=cg25408950	feature_id[772].value > threshold=0.680083692073822
node_290: feature_name=cg15188939	feature_id[1626].value <= threshold=0.6365727782249451
node_291: feature_name=cg23421128	feature_id[1104].value > threshold=0.14253754168748856
Class: angiomatoid fibrous histiocytoma (AFH)	
Rules_32	passed counts:10
node_0: feature_name=cg11915444	feature_id[1171].value > threshold=0.3402601182460785
node_10: feature_name=cg12109728	feature_id[2810].value > threshold=0.7068270146846771
node_20: feature_name=cg16412000	feature_id[789].value > threshold=0.6741803884506226
node_24: feature_name=cg17115147	feature_id[1434].value <= threshold=0.2961975038051605
node_25: feature_name=cg24474622	feature_id[2737].value > threshold=0.7211934924125671
node_29: feature_name=cg10472711	feature_id[612].value > threshold=0.45985929667949677
node_37: feature_name=cg01937669	feature_id[188].value <= threshold=0.4809463769197464
node_38: feature_name=cg02157052	feature_id[451].value <= threshold=0.2869153320789337
node_39: feature_name=cg18751958	feature_id[1427].value > threshold=0.4813787043094635
node_41: feature_name=cg22512847	feature_id[1152].value > threshold=0.5129324495792389
node_43: feature_name=cg23345038	feature_id[1147].value > threshold=0.6318148374557495
node_45: feature_name=cg17439009	feature_id[1815].value <= threshold=0.48514117300510406
node_46: feature_name=cg17537493	feature_id[2772].value > threshold=0.5825372636318207
node_52: feature_name=cg01683570	feature_id[110].value > threshold=0.6040188074111938
node_54: feature_name=cg15720017	feature_id[168].value > threshold=0.7067741453647614
node_60: feature_name=cg23690444	feature_id[120].value <= threshold=0.16037724912166595

node_61: feature_name=cg00567872	feature_id[236].value > threshold=0.7005765736103058
node_85: feature_name=cg01014262	feature_id[764].value <= threshold=0.5737917125225067
node_86: feature_name=cg22946562	feature_id[1148].value > threshold=0.6328203976154327
node_134: feature_name=cg14514032	feature_id[202].value <= threshold=0.4389604330062866
node_135: feature_name=cg13488220	feature_id[241].value > threshold=0.7352153658866882
node_143: feature_name=cg03840920	feature_id[256].value > threshold=0.502630889415741
node_147: feature_name=cg13634090	feature_id[822].value > threshold=0.7207068204879761
node_151: feature_name=cg19584674	feature_id[2364].value <= threshold=0.41758519411087036
node_152: feature_name=cg07344990	feature_id[411].value > threshold=0.5484964847564697
node_158: feature_name=cg10298992	feature_id[3066].value > threshold=0.5707973837852478
node_174: feature_name=cg11843516	feature_id[20].value <= threshold=0.5590928494930267
node_175: feature_name=cg05185926	feature_id[448].value > threshold=0.17318664491176605
node_177: feature_name=cg17009731	feature_id[1220].value > threshold=0.6666513085365295
node_179: feature_name=cg01380319	feature_id[515].value > threshold=0.39251960813999176
Class: giant cell tumour of bone (GCTB)	
Rules_33	passed counts:10
node_0: feature_name=cg11915444	feature_id[1171].value > threshold=0.3402601182460785
node_10: feature_name=cg12109728	feature_id[2810].value > threshold=0.7068270146846771
node_20: feature_name=cg16412000	feature_id[789].value > threshold=0.6741803884506226
node_24: feature_name=cg17115147	feature_id[1434].value <= threshold=0.2961975038051605
node_25: feature_name=cg24474622	feature_id[2737].value > threshold=0.7211934924125671
node_29: feature_name=cg10472711	feature_id[612].value > threshold=0.45985929667949677

node_37: feature_name=cg01937669	feature_id[188].value <= threshold=0.4809463769197464
node_38: feature_name=cg02157052	feature_id[451].value <= threshold=0.2869153320789337
node_39: feature_name=cg18751958	feature_id[1427].value > threshold=0.4813787043094635
node_41: feature_name=cg22512847	feature_id[1152].value > threshold=0.5129324495792389
node_43: feature_name=cg23345038	feature_id[1147].value > threshold=0.6318148374557495
node_45: feature_name=cg17439009	feature_id[1815].value <= threshold=0.48514117300510406
node_46: feature_name=cg17537493	feature_id[2772].value > threshold=0.5825372636318207
node_52: feature_name=cg01683570	feature_id[110].value > threshold=0.6040188074111938
node_54: feature_name=cg15720017	feature_id[168].value > threshold=0.7067741453647614
node_60: feature_name=cg23690444	feature_id[120].value <= threshold=0.16037724912166595
node_61: feature_name=cg00567872	feature_id[236].value > threshold=0.7005765736103058
node_85: feature_name=cg01014262	feature_id[764].value <= threshold=0.5737917125225067
node_86: feature_name=cg22946562	feature_id[1148].value > threshold=0.6328203976154327
node_134: feature_name=cg14514032	feature_id[202].value <= threshold=0.4389604330062866
node_135: feature_name=cg13488220	feature_id[241].value > threshold=0.7352153658866882
node_143: feature_name=cg03840920	feature_id[256].value > threshold=0.502630889415741
node_147: feature_name=cg13634090	feature_id[822].value > threshold=0.7207068204879761
node_151: feature_name=cg19584674	feature_id[2364].value <= threshold=0.41758519411087036
node_152: feature_name=cg07344990	feature_id[411].value > threshold=0.5484964847564697
node_158: feature_name=cg10298992	feature_id[3066].value > threshold=0.5707973837852478
node_174: feature_name=cg11843516	feature_id[20].value <= threshold=0.5590928494930267
node_175: feature_name=cg05185926	feature_id[448].value > threshold=0.17318664491176605

node_177: feature_name=cg17009731	feature_id[1220].value > threshold=0.6665130853652 95
node_179: feature_name=cg01380319	feature_id[515].value <= threshold=0.39251960813999176
node_180: feature_name=cg20700977	feature_id[303].value > threshold=0.8422040641307831
node_184: feature_name=cg24259291	feature_id[164].value > threshold=0.4872268736362457
node_188: feature_name=cg08097657	feature_id[67].value > threshold=0.3371318429708481
node_190: feature_name=cg07891483	feature_id[59].value > threshold=0.6211732923984528
node_194: feature_name=cg20122645	feature_id[275].value > threshold=0.6094755828380585
node_196: feature_name=cg04026354	feature_id[1763].value <= threshold=0.4341808259487152
node_197: feature_name=cg10149889	feature_id[1017].value > threshold=0.6150195300579071
node_201: feature_name=cg14890730	feature_id[337].value > threshold=0.4372602105140686
node_279: feature_name=cg27143703	feature_id[3174].value > threshold=0.42068085074424744
Class: reactive tissue (REA)	
Rules_34	passed counts:10
node_0: feature_name=cg11915444	feature_id[1171].value > threshold=0.3402601182460785
node_10: feature_name=cg12109728	feature_id[2810].value > threshold=0.7068270146846771
node_20: feature_name=cg16412000	feature_id[789].value > threshold=0.6741803884506226
node_24: feature_name=cg17115147	feature_id[1434].value <= threshold=0.2961975038051605
node_25: feature_name=cg24474622	feature_id[2737].value > threshold=0.7211934924125671
node_29: feature_name=cg10472711	feature_id[612].value > threshold=0.45985929667949677
node_37: feature_name=cg01937669	feature_id[188].value <= threshold=0.4809463769197464
node_38: feature_name=cg02157052	feature_id[451].value <= threshold=0.2869153320789337
node_39: feature_name=cg18751958	feature_id[1427].value > threshold=0.4813787043094635

node_41: feature_name=cg22512847	feature_id[1152].value > threshold=0.5129324495792389
node_43: feature_name=cg23345038	feature_id[1147].value > threshold=0.6318148374557495
node_45: feature_name=cg17439009	feature_id[1815].value <= threshold=0.48514117300510406
node_46: feature_name=cg17537493	feature_id[2772].value > threshold=0.5825372636318207
node_52: feature_name=cg01683570	feature_id[110].value > threshold=0.6040188074111938
node_54: feature_name=cg15720017	feature_id[168].value > threshold=0.7067741453647614
node_60: feature_name=cg23690444	feature_id[120].value <= threshold=0.16037724912166595
node_61: feature_name=cg00567872	feature_id[236].value > threshold=0.7005765736103058
node_85: feature_name=cg01014262	feature_id[764].value <= threshold=0.5737917125225067
node_86: feature_name=cg22946562	feature_id[1148].value > threshold=0.6328203976154327
node_134: feature_name=cg14514032	feature_id[202].value <= threshold=0.4389604330062866
node_135: feature_name=cg13488220	feature_id[241].value > threshold=0.7352153658866882
node_143: feature_name=cg03840920	feature_id[256].value > threshold=0.502630889415741
node_147: feature_name=cg13634090	feature_id[822].value > threshold=0.7207068204879761
node_151: feature_name=cg19584674	feature_id[2364].value <= threshold=0.41758519411087036
node_152: feature_name=cg07344990	feature_id[411].value > threshold=0.5484964847564697
node_158: feature_name=cg10298992	feature_id[3066].value > threshold=0.5707973837852478
node_174: feature_name=cg11843516	feature_id[20].value <= threshold=0.5590928494930267
node_175: feature_name=cg05185926	feature_id[448].value > threshold=0.17318664491176605
node_177: feature_name=cg17009731	feature_id[1220].value > threshold=0.6666513085365295
node_179: feature_name=cg01380319	feature_id[515].value <= threshold=0.39251960813999176
node_180: feature_name=cg20700977	feature_id[303].value <= threshold=0.8422040641307831

node_181: feature_name=cg06825317	feature_id[752].value > threshold=0.3880208432674408
Class: leiomyosarcoma (LMS)	
Rules_35	passed counts:10
node_0: feature_name=cg11915444	feature_id[1171].value > threshold=0.3402601182460785
node_10: feature_name=cg12109728	feature_id[2810].value > threshold=0.7068270146846771
node_20: feature_name=cg16412000	feature_id[789].value > threshold=0.6741803884506226
node_24: feature_name=cg17115147	feature_id[1434].value <= threshold=0.2961975038051605
node_25: feature_name=cg24474622	feature_id[2737].value > threshold=0.7211934924125671
node_29: feature_name=cg10472711	feature_id[612].value > threshold=0.45985929667949677
node_37: feature_name=cg01937669	feature_id[188].value <= threshold=0.4809463769197464
node_38: feature_name=cg02157052	feature_id[451].value <= threshold=0.2869153320789337
node_39: feature_name=cg18751958	feature_id[1427].value > threshold=0.4813787043094635
node_41: feature_name=cg22512847	feature_id[1152].value > threshold=0.5129324495792389
node_43: feature_name=cg23345038	feature_id[1147].value > threshold=0.6318148374557495
node_45: feature_name=cg17439009	feature_id[1815].value <= threshold=0.48514117300510406
node_46: feature_name=cg17537493	feature_id[2772].value > threshold=0.5825372636318207
node_52: feature_name=cg01683570	feature_id[110].value > threshold=0.6040188074111938
node_54: feature_name=cg15720017	feature_id[168].value > threshold=0.7067741453647614
node_60: feature_name=cg23690444	feature_id[120].value <= threshold=0.16037724912166595
node_61: feature_name=cg00567872	feature_id[236].value > threshold=0.7005765736103058
node_85: feature_name=cg01014262	feature_id[764].value <= threshold=0.5737917125225067
node_86: feature_name=cg22946562	feature_id[1148].value > threshold=0.6328203976154327

node_134: feature_name=cg14514032	feature_id[202].value <= threshold=0.4389604330062866
node_135: feature_name=cg13488220	feature_id[241].value > threshold=0.7352153658866882
node_143: feature_name=cg03840920	feature_id[256].value > threshold=0.502630889415741
node_147: feature_name=cg13634090	feature_id[822].value > threshold=0.7207068204879761
node_151: feature_name=cg19584674	feature_id[2364].value <= threshold=0.41758519411087036
node_152: feature_name=cg07344990	feature_id[411].value > threshold=0.5484964847564697
node_158: feature_name=cg10298992	feature_id[3066].value > threshold=0.5707973837852478
node_174: feature_name=cg11843516	feature_id[20].value <= threshold=0.5590928494930267
node_175: feature_name=cg05185926	feature_id[448].value > threshold=0.17318664491176605
node_177: feature_name=cg17009731	feature_id[1220].value <= threshold=0.6666513085365295
Class: extraskeletal myxoid chondrosarcoma (EMCS)	
Rules_36	passed counts:10
node_0: feature_name=cg11915444	feature_id[1171].value > threshold=0.3402601182460785
node_10: feature_name=cg12109728	feature_id[2810].value > threshold=0.7068270146846771
node_20: feature_name=cg16412000	feature_id[789].value > threshold=0.6741803884506226
node_24: feature_name=cg17115147	feature_id[1434].value <= threshold=0.2961975038051605
node_25: feature_name=cg24474622	feature_id[2737].value > threshold=0.7211934924125671
node_29: feature_name=cg10472711	feature_id[612].value > threshold=0.45985929667949677
node_37: feature_name=cg01937669	feature_id[188].value <= threshold=0.4809463769197464
node_38: feature_name=cg02157052	feature_id[451].value <= threshold=0.2869153320789337
node_39: feature_name=cg18751958	feature_id[1427].value > threshold=0.4813787043094635

node_41: feature_name=cg22512847	feature_id[1152].value > threshold=0.5129324495792389
node_43: feature_name=cg23345038	feature_id[1147].value > threshold=0.6318148374557495
node_45: feature_name=cg17439009	feature_id[1815].value <= threshold=0.48514117300510406
node_46: feature_name=cg17537493	feature_id[2772].value > threshold=0.5825372636318207
node_52: feature_name=cg01683570	feature_id[110].value > threshold=0.6040188074111938
node_54: feature_name=cg15720017	feature_id[168].value > threshold=0.7067741453647614
node_60: feature_name=cg23690444	feature_id[120].value <= threshold=0.16037724912166595
node_61: feature_name=cg00567872	feature_id[236].value > threshold=0.7005765736103058
node_85: feature_name=cg01014262	feature_id[764].value <= threshold=0.5737917125225067
node_86: feature_name=cg22946562	feature_id[1148].value > threshold=0.6328203976154327
node_134: feature_name=cg14514032	feature_id[202].value <= threshold=0.4389604330062866
node_135: feature_name=cg13488220	feature_id[241].value > threshold=0.7352153658866882
node_143: feature_name=cg03840920	feature_id[256].value > threshold=0.502630889415741
node_147: feature_name=cg13634090	feature_id[822].value > threshold=0.7207068204879761
node_151: feature_name=cg19584674	feature_id[2364].value <= threshold=0.41758519411087036
node_152: feature_name=cg07344990	feature_id[411].value > threshold=0.5484964847564697
node_158: feature_name=cg10298992	feature_id[3066].value > threshold=0.5707973837852478
node_174: feature_name=cg11843516	feature_id[20].value <= threshold=0.5590928494930267
node_175: feature_name=cg05185926	feature_id[448].value <= threshold=0.17318664491176605
Class: control (CTRL)	
Rules_37	passed counts:9
node_0: feature_name=cg11915444	feature_id[1171].value > threshold=0.3402601182460785

node_10: feature_name=cg12109728	feature_id[2810].value > threshold=0.7068270146846771
node_20: feature_name=cg16412000	feature_id[789].value > threshold=0.6741803884506226
node_24: feature_name=cg17115147	feature_id[1434].value <= threshold=0.2961975038051605
node_25: feature_name=cg24474622	feature_id[2737].value > threshold=0.7211934924125671
node_29: feature_name=cg10472711	feature_id[612].value > threshold=0.45985929667949677
node_37: feature_name=cg01937669	feature_id[188].value <= threshold=0.4809463769197464
node_38: feature_name=cg02157052	feature_id[451].value <= threshold=0.2869153320789337
node_39: feature_name=cg18751958	feature_id[1427].value > threshold=0.4813787043094635
node_41: feature_name=cg22512847	feature_id[1152].value > threshold=0.5129324495792389
node_43: feature_name=cg23345038	feature_id[1147].value > threshold=0.6318148374557495
node_45: feature_name=cg17439009	feature_id[1815].value <= threshold=0.48514117300510406
node_46: feature_name=cg17537493	feature_id[2772].value > threshold=0.5825372636318207
node_52: feature_name=cg01683570	feature_id[110].value > threshold=0.6040188074111938
node_54: feature_name=cg15720017	feature_id[168].value > threshold=0.7067741453647614
node_60: feature_name=cg23690444	feature_id[120].value <= threshold=0.16037724912166595
node_61: feature_name=cg00567872	feature_id[236].value > threshold=0.7005765736103058
node_85: feature_name=cg01014262	feature_id[764].value <= threshold=0.5737917125225067
node_86: feature_name=cg22946562	feature_id[1148].value > threshold=0.6328203976154327
node_134: feature_name=cg14514032	feature_id[202].value > threshold=0.4389604330062866
node_312: feature_name=cg21670987	feature_id[1509].value <= threshold=0.32477423548698425
node_313: feature_name=cg12939390	feature_id[143].value > threshold=0.23070813715457916
node_315: feature_name=cg10907727	feature_id[284].value <= threshold=0.6596133708953857

Class: high-grade endometrial stromal sarcoma (ESS (HG))	
Rules_38	passed counts:9
node_0: feature_name=cg11915444	feature_id[1171].value > threshold=0.3402601182460785
node_10: feature_name=cg12109728	feature_id[2810].value > threshold=0.7068270146846771
node_20: feature_name=cg16412000	feature_id[789].value > threshold=0.6741803884506226
node_24: feature_name=cg17115147	feature_id[1434].value <= threshold=0.2961975038051605
node_25: feature_name=cg24474622	feature_id[2737].value > threshold=0.7211934924125671
node_29: feature_name=cg10472711	feature_id[612].value > threshold=0.45985929667949677
node_37: feature_name=cg01937669	feature_id[188].value <= threshold=0.4809463769197464
node_38: feature_name=cg02157052	feature_id[451].value <= threshold=0.2869153320789337
node_39: feature_name=cg18751958	feature_id[1427].value > threshold=0.4813787043094635
node_41: feature_name=cg22512847	feature_id[1152].value > threshold=0.5129324495792389
node_43: feature_name=cg23345038	feature_id[1147].value > threshold=0.6318148374557495
node_45: feature_name=cg17439009	feature_id[1815].value <= threshold=0.48514117300510406
node_46: feature_name=cg17537493	feature_id[2772].value > threshold=0.5825372636318207
node_52: feature_name=cg01683570	feature_id[110].value > threshold=0.6040188074111938
node_54: feature_name=cg15720017	feature_id[168].value > threshold=0.7067741453647614
node_60: feature_name=cg23690444	feature_id[120].value <= threshold=0.16037724912166595
node_61: feature_name=cg00567872	feature_id[236].value > threshold=0.7005765736103058
node_85: feature_name=cg01014262	feature_id[764].value <= threshold=0.5737917125225067
node_86: feature_name=cg22946562	feature_id[1148].value > threshold=0.6328203976154327

node_134: feature_name=cg14514032	feature_id[202].value <= threshold=0.4389604330062866
node_135: feature_name=cg13488220	feature_id[241].value > threshold=0.7352153658866882
node_143: feature_name=cg03840920	feature_id[256].value > threshold=0.502630889415741
node_147: feature_name=cg13634090	feature_id[822].value > threshold=0.7207068204879761
node_151: feature_name=cg19584674	feature_id[2364].value <= threshold=0.41758519411087036
node_152: feature_name=cg07344990	feature_id[411].value > threshold=0.5484964847564697
node_158: feature_name=cg10298992	feature_id[3066].value > threshold=0.5707973837852478
node_174: feature_name=cg11843516	feature_id[20].value <= threshold=0.5590928494930267
node_175: feature_name=cg05185926	feature_id[448].value > threshold=0.17318664491176605
node_177: feature_name=cg17009731	feature_id[1220].value > threshold=0.6666513085365295
node_179: feature_name=cg01380319	feature_id[515].value <= threshold=0.39251960813999176
node_180: feature_name=cg20700977	feature_id[303].value > threshold=0.8422040641307831
node_184: feature_name=cg24259291	feature_id[164].value > threshold=0.4872268736362457
node_188: feature_name=cg08097657	feature_id[67].value > threshold=0.3371318429708481
node_190: feature_name=cg07891483	feature_id[59].value > threshold=0.6211732923984528
node_194: feature_name=cg20122645	feature_id[275].value > threshold=0.6094755828380585
node_196: feature_name=cg04026354	feature_id[1763].value <= threshold=0.4341808259487152
node_197: feature_name=cg10149889	feature_id[1017].value > threshold=0.6150195300579071
node_201: feature_name=cg14890730	feature_id[337].value <= threshold=0.4372602105140686
node_202: feature_name=cg08331427	feature_id[132].value > threshold=0.5443233847618103
node_208: feature_name=cg22730007	feature_id[2544].value > threshold=0.28619489073753357
node_210: feature_name=cg17193551	feature_id[2057].value > threshold=0.4393353909254074

node_212: feature_name=cg14671764	feature_id[584].value <= threshold=0.9353199899196625
node_213: feature_name=cg20140662	feature_id[2044].value > threshold=0.38030287623405457
node_215: feature_name=cg07891483	feature_id[59].value > threshold=0.829958975315094
node_219: feature_name=cg04884579	feature_id[1861].value <= threshold=0.6106154918670654
node_220: feature_name=cg25066857	feature_id[3759].value > threshold=0.8149203658103943
Class: lipoma (LIPO)	
Rules_39	passed counts:9
node_0: feature_name=cg11915444	feature_id[1171].value > threshold=0.3402601182460785
node_10: feature_name=cg12109728	feature_id[2810].value > threshold=0.7068270146846771
node_20: feature_name=cg16412000	feature_id[789].value > threshold=0.6741803884506226
node_24: feature_name=cg17115147	feature_id[1434].value <= threshold=0.2961975038051605
node_25: feature_name=cg24474622	feature_id[2737].value > threshold=0.7211934924125671
node_29: feature_name=cg10472711	feature_id[612].value > threshold=0.45985929667949677
node_37: feature_name=cg01937669	feature_id[188].value <= threshold=0.4809463769197464
node_38: feature_name=cg02157052	feature_id[451].value <= threshold=0.2869153320789337
node_39: feature_name=cg18751958	feature_id[1427].value > threshold=0.4813787043094635
node_41: feature_name=cg22512847	feature_id[1152].value > threshold=0.5129324495792389
node_43: feature_name=cg23345038	feature_id[1147].value > threshold=0.6318148374557495
node_45: feature_name=cg17439009	feature_id[1815].value <= threshold=0.48514117300510406
node_46: feature_name=cg17537493	feature_id[2772].value > threshold=0.5825372636318207
node_52: feature_name=cg01683570	feature_id[110].value > threshold=0.6040188074111938
node_54: feature_name=cg15720017	feature_id[168].value > threshold=0.7067741453647614

node_60: feature_name=cg23690444	feature_id[120].value <= threshold=0.16037724912166595
node_61: feature_name=cg00567872	feature_id[236].value > threshold=0.7005765736103058
node_85: feature_name=cg01014262	feature_id[764].value <= threshold=0.5737917125225067
node_86: feature_name=cg22946562	feature_id[1148].value > threshold=0.6328203976154327
node_134: feature_name=cg14514032	feature_id[202].value <= threshold=0.4389604330062866
node_135: feature_name=cg13488220	feature_id[241].value > threshold=0.7352153658866882
node_143: feature_name=cg03840920	feature_id[256].value > threshold=0.502630889415741
node_147: feature_name=cg13634090	feature_id[822].value > threshold=0.7207068204879761
node_151: feature_name=cg19584674	feature_id[2364].value <= threshold=0.41758519411087036
node_152: feature_name=cg07344990	feature_id[411].value > threshold=0.5484964847564697
node_158: feature_name=cg10298992	feature_id[3066].value > threshold=0.5707973837852478
node_174: feature_name=cg11843516	feature_id[20].value <= threshold=0.5590928494930267
node_175: feature_name=cg05185926	feature_id[448].value > threshold=0.17318664491176605
node_177: feature_name=cg17009731	feature_id[1220].value > threshold=0.6666513085365295
node_179: feature_name=cg01380319	feature_id[515].value <= threshold=0.39251960813999176
node_180: feature_name=cg20700977	feature_id[303].value > threshold=0.8422040641307831
node_184: feature_name=cg24259291	feature_id[164].value > threshold=0.4872268736362457
node_188: feature_name=cg08097657	feature_id[67].value > threshold=0.3371318429708481
node_190: feature_name=cg07891483	feature_id[59].value > threshold=0.6211732923984528
node_194: feature_name=cg20122645	feature_id[275].value > threshold=0.6094755828380585
node_196: feature_name=cg04026354	feature_id[1763].value <= threshold=0.4341808259487152
node_197: feature_name=cg10149889	feature_id[1017].value > threshold=0.6150195300579071

node_201: feature_name=cg14890730	feature_id[337].value <= threshold=0.4372602105140686
node_202: feature_name=cg08331427	feature_id[132].value <= threshold=0.5443233847618103
node_203: feature_name=cg04963697	feature_id[102].value > threshold=0.7484472692012787
Class: epithelioid haemangioendothelioma (EHE)	
Rules_40	passed counts:9
node_0: feature_name=cg11915444	feature_id[1171].value > threshold=0.3402601182460785
node_10: feature_name=cg12109728	feature_id[2810].value > threshold=0.7068270146846771
node_20: feature_name=cg16412000	feature_id[789].value > threshold=0.6741803884506226
node_24: feature_name=cg17115147	feature_id[1434].value <= threshold=0.2961975038051605
node_25: feature_name=cg24474622	feature_id[2737].value > threshold=0.7211934924125671
node_29: feature_name=cg10472711	feature_id[612].value > threshold=0.45985929667949677
node_37: feature_name=cg01937669	feature_id[188].value <= threshold=0.4809463769197464
node_38: feature_name=cg02157052	feature_id[451].value <= threshold=0.2869153320789337
node_39: feature_name=cg18751958	feature_id[1427].value > threshold=0.4813787043094635
node_41: feature_name=cg22512847	feature_id[1152].value > threshold=0.5129324495792389
node_43: feature_name=cg23345038	feature_id[1147].value > threshold=0.6318148374557495
node_45: feature_name=cg17439009	feature_id[1815].value <= threshold=0.48514117300510406
node_46: feature_name=cg17537493	feature_id[2772].value > threshold=0.5825372636318207
node_52: feature_name=cg01683570	feature_id[110].value > threshold=0.6040188074111938
node_54: feature_name=cg15720017	feature_id[168].value > threshold=0.7067741453647614
node_60: feature_name=cg23690444	feature_id[120].value <= threshold=0.16037724912166595
node_61: feature_name=cg00567872	feature_id[236].value > threshold=0.7005765736103058

node_85: feature_name=cg01014262	feature_id[764].value <= threshold=0.5737917125225067
node_86: feature_name=cg22946562	feature_id[1148].value > threshold=0.6328203976154327
node_134: feature_name=cg14514032	feature_id[202].value <= threshold=0.4389604330062866
node_135: feature_name=cg13488220	feature_id[241].value > threshold=0.7352153658866882
node_143: feature_name=cg03840920	feature_id[256].value > threshold=0.502630889415741
node_147: feature_name=cg13634090	feature_id[822].value > threshold=0.7207068204879761
node_151: feature_name=cg19584674	feature_id[2364].value <= threshold=0.41758519411087036
node_152: feature_name=cg07344990	feature_id[411].value > threshold=0.5484964847564697
node_158: feature_name=cg10298992	feature_id[3066].value > threshold=0.5707973837852478
node_174: feature_name=cg11843516	feature_id[20].value <= threshold=0.5590928494930267
node_175: feature_name=cg05185926	feature_id[448].value > threshold=0.17318664491176605
node_177: feature_name=cg17009731	feature_id[1220].value > threshold=0.6666513085365295
node_179: feature_name=cg01380319	feature_id[515].value <= threshold=0.39251960813999176
node_180: feature_name=cg20700977	feature_id[303].value > threshold=0.8422040641307831
node_184: feature_name=cg24259291	feature_id[164].value > threshold=0.4872268736362457
node_188: feature_name=cg08097657	feature_id[67].value > threshold=0.3371318429708481
node_190: feature_name=cg07891483	feature_id[59].value > threshold=0.6211732923984528
node_194: feature_name=cg20122645	feature_id[275].value > threshold=0.6094755828380585
node_196: feature_name=cg04026354	feature_id[1763].value <= threshold=0.4341808259487152
node_197: feature_name=cg10149889	feature_id[1017].value <= threshold=0.6150195300579071
node_198: feature_name=cg16489468	feature_id[196].value > threshold=0.6215258538722992
Class: nodular fasciitis(NFA)	

Rules_41	passed counts:9
node_0: feature_name=cg11915444	feature_id[1171].value > threshold=0.3402601182460785
node_10: feature_name=cg12109728	feature_id[2810].value > threshold=0.7068270146846771
node_20: feature_name=cg16412000	feature_id[789].value > threshold=0.6741803884506226
node_24: feature_name=cg17115147	feature_id[1434].value <= threshold=0.2961975038051605
node_25: feature_name=cg24474622	feature_id[2737].value > threshold=0.7211934924125671
node_29: feature_name=cg10472711	feature_id[612].value > threshold=0.45985929667949677
node_37: feature_name=cg01937669	feature_id[188].value <= threshold=0.4809463769197464
node_38: feature_name=cg02157052	feature_id[451].value <= threshold=0.2869153320789337
node_39: feature_name=cg18751958	feature_id[1427].value > threshold=0.4813787043094635
node_41: feature_name=cg22512847	feature_id[1152].value > threshold=0.5129324495792389
node_43: feature_name=cg23345038	feature_id[1147].value > threshold=0.6318148374557495
node_45: feature_name=cg17439009	feature_id[1815].value <= threshold=0.48514117300510406
node_46: feature_name=cg17537493	feature_id[2772].value > threshold=0.5825372636318207
node_52: feature_name=cg01683570	feature_id[110].value > threshold=0.6040188074111938
node_54: feature_name=cg15720017	feature_id[168].value > threshold=0.7067741453647614
node_60: feature_name=cg23690444	feature_id[120].value <= threshold=0.16037724912166595
node_61: feature_name=cg00567872	feature_id[236].value > threshold=0.7005765736103058
node_85: feature_name=cg01014262	feature_id[764].value <= threshold=0.5737917125225067
node_86: feature_name=cg22946562	feature_id[1148].value > threshold=0.6328203976154327
node_134: feature_name=cg14514032	feature_id[202].value <= threshold=0.4389604330062866

node_135: feature_name=cg13488220	feature_id[241].value > threshold=0.7352153658866882
node_143: feature_name=cg03840920	feature_id[256].value > threshold=0.502630889415741
node_147: feature_name=cg13634090	feature_id[822].value > threshold=0.7207068204879761
node_151: feature_name=cg19584674	feature_id[2364].value <= threshold=0.41758519411087036
node_152: feature_name=cg07344990	feature_id[411].value > threshold=0.5484964847564697
node_158: feature_name=cg10298992	feature_id[3066].value > threshold=0.5707973837852478
node_174: feature_name=cg11843516	feature_id[20].value <= threshold=0.5590928494930267
node_175: feature_name=cg05185926	feature_id[448].value > threshold=0.17318664491176605
node_177: feature_name=cg17009731	feature_id[1220].value > threshold=0.6666513085365295
node_179: feature_name=cg01380319	feature_id[515].value <= threshold=0.39251960813999176
node_180: feature_name=cg20700977	feature_id[303].value > threshold=0.8422040641307831
node_184: feature_name=cg24259291	feature_id[164].value > threshold=0.4872268736362457
node_188: feature_name=cg08097657	feature_id[67].value > threshold=0.3371318429708481
node_190: feature_name=cg07891483	feature_id[59].value <= threshold=0.6211732923984528
node_191: feature_name=cg23903708	feature_id[1504].value <= threshold=0.8707234263420105
Class: neurofibroma (NFB)	
Rules_42	passed counts:9
node_0: feature_name=cg11915444	feature_id[1171].value > threshold=0.3402601182460785
node_10: feature_name=cg12109728	feature_id[2810].value > threshold=0.7068270146846771
node_20: feature_name=cg16412000	feature_id[789].value > threshold=0.6741803884506226
node_24: feature_name=cg17115147	feature_id[1434].value <= threshold=0.2961975038051605
node_25: feature_name=cg24474622	feature_id[2737].value > threshold=0.7211934924125671

node_29: feature_name=cg10472711	feature_id[612].value > threshold=0.4598592966794967 7
node_37: feature_name=cg01937669	feature_id[188].value <= threshold=0.4809463769197464
node_38: feature_name=cg02157052	feature_id[451].value <= threshold=0.2869153320789337
node_39: feature_name=cg18751958	feature_id[1427].value > threshold=0.4813787043094635
node_41: feature_name=cg22512847	feature_id[1152].value > threshold=0.5129324495792389
node_43: feature_name=cg23345038	feature_id[1147].value > threshold=0.6318148374557495
node_45: feature_name=cg17439009	feature_id[1815].value <= threshold=0.48514117300510406
node_46: feature_name=cg17537493	feature_id[2772].value > threshold=0.5825372636318207
node_52: feature_name=cg01683570	feature_id[110].value > threshold=0.6040188074111938
node_54: feature_name=cg15720017	feature_id[168].value > threshold=0.7067741453647614
node_60: feature_name=cg23690444	feature_id[120].value <= threshold=0.16037724912166595
node_61: feature_name=cg00567872	feature_id[236].value > threshold=0.7005765736103058
node_85: feature_name=cg01014262	feature_id[764].value <= threshold=0.5737917125225067
node_86: feature_name=cg22946562	feature_id[1148].value > threshold=0.6328203976154327
node_134: feature_name=cg14514032	feature_id[202].value <= threshold=0.4389604330062866
node_135: feature_name=cg13488220	feature_id[241].value > threshold=0.7352153658866882
node_143: feature_name=cg03840920	feature_id[256].value > threshold=0.502630889415741
node_147: feature_name=cg13634090	feature_id[822].value > threshold=0.7207068204879761
node_151: feature_name=cg19584674	feature_id[2364].value <= threshold=0.41758519411087036
node_152: feature_name=cg07344990	feature_id[411].value > threshold=0.5484964847564697
node_158: feature_name=cg10298992	feature_id[3066].value > threshold=0.5707973837852478
node_174: feature_name=cg11843516	feature_id[20].value <= threshold=0.5590928494930267

node_175: feature_name=cg05185926	feature_id[448].value > threshold=0.17318664491176605
node_177: feature_name=cg17009731	feature_id[1220].value > threshold=0.6666513085365295
node_179: feature_name=cg01380319	feature_id[515].value <= threshold=0.39251960813999176
node_180: feature_name=cg20700977	feature_id[303].value > threshold=0.8422040641307831
node_184: feature_name=cg24259291	feature_id[164].value > threshold=0.4872268736362457
node_188: feature_name=cg08097657	feature_id[67].value <= threshold=0.3371318429708481
Class: infantile fibrosarcoma (IFS)	
Rules_43	passed counts:9
node_0: feature_name=cg11915444	feature_id[1171].value > threshold=0.3402601182460785
node_10: feature_name=cg12109728	feature_id[2810].value > threshold=0.7068270146846771
node_20: feature_name=cg16412000	feature_id[789].value > threshold=0.6741803884506226
node_24: feature_name=cg17115147	feature_id[1434].value <= threshold=0.2961975038051605
node_25: feature_name=cg24474622	feature_id[2737].value > threshold=0.7211934924125671
node_29: feature_name=cg10472711	feature_id[612].value > threshold=0.45985929667949677
node_37: feature_name=cg01937669	feature_id[188].value <= threshold=0.4809463769197464
node_38: feature_name=cg02157052	feature_id[451].value <= threshold=0.2869153320789337
node_39: feature_name=cg18751958	feature_id[1427].value > threshold=0.4813787043094635
node_41: feature_name=cg22512847	feature_id[1152].value > threshold=0.5129324495792389
node_43: feature_name=cg23345038	feature_id[1147].value > threshold=0.6318148374557495
node_45: feature_name=cg17439009	feature_id[1815].value <= threshold=0.48514117300510406
node_46: feature_name=cg17537493	feature_id[2772].value > threshold=0.5825372636318207
node_52: feature_name=cg01683570	feature_id[110].value > threshold=0.6040188074111938

node_54: feature_name=cg15720017	feature_id[168].value > threshold=0.7067741453647614
node_60: feature_name=cg23690444	feature_id[120].value <= threshold=0.16037724912166595
node_61: feature_name=cg00567872	feature_id[236].value > threshold=0.7005765736103058
node_85: feature_name=cg01014262	feature_id[764].value <= threshold=0.5737917125225067
node_86: feature_name=cg22946562	feature_id[1148].value > threshold=0.6328203976154327
node_134: feature_name=cg14514032	feature_id[202].value <= threshold=0.4389604330062866
node_135: feature_name=cg13488220	feature_id[241].value > threshold=0.7352153658866882
node_143: feature_name=cg03840920	feature_id[256].value > threshold=0.502630889415741
node_147: feature_name=cg13634090	feature_id[822].value > threshold=0.7207068204879761
node_151: feature_name=cg19584674	feature_id[2364].value <= threshold=0.41758519411087036
node_152: feature_name=cg07344990	feature_id[411].value > threshold=0.5484964847564697
node_158: feature_name=cg10298992	feature_id[3066].value > threshold=0.5707973837852478
node_174: feature_name=cg11843516	feature_id[20].value <= threshold=0.5590928494930267
node_175: feature_name=cg05185926	feature_id[448].value > threshold=0.17318664491176605
node_177: feature_name=cg17009731	feature_id[1220].value > threshold=0.6666513085365295
node_179: feature_name=cg01380319	feature_id[515].value <= threshold=0.39251960813999176
node_180: feature_name=cg20700977	feature_id[303].value > threshold=0.8422040641307831
node_184: feature_name=cg24259291	feature_id[164].value <= threshold=0.4872268736362457
node_185: feature_name=cg00512280	feature_id[1400].value > threshold=0.05498958192765713
Class: Langerhans cell histiocytosis (LCH)	
Rules_44	passed counts:9
node_0: feature_name=cg11915444	feature_id[1171].value > threshold=0.3402601182460785

node_10: feature_name=cg12109728	feature_id[2810].value > threshold=0.7068270146846771
node_20: feature_name=cg16412000	feature_id[789].value > threshold=0.6741803884506226
node_24: feature_name=cg17115147	feature_id[1434].value <= threshold=0.2961975038051605
node_25: feature_name=cg24474622	feature_id[2737].value > threshold=0.7211934924125671
node_29: feature_name=cg10472711	feature_id[612].value > threshold=0.45985929667949677
node_37: feature_name=cg01937669	feature_id[188].value <= threshold=0.4809463769197464
node_38: feature_name=cg02157052	feature_id[451].value <= threshold=0.2869153320789337
node_39: feature_name=cg18751958	feature_id[1427].value > threshold=0.4813787043094635
node_41: feature_name=cg22512847	feature_id[1152].value > threshold=0.5129324495792389
node_43: feature_name=cg23345038	feature_id[1147].value > threshold=0.6318148374557495
node_45: feature_name=cg17439009	feature_id[1815].value <= threshold=0.48514117300510406
node_46: feature_name=cg17537493	feature_id[2772].value > threshold=0.5825372636318207
node_52: feature_name=cg01683570	feature_id[110].value > threshold=0.6040188074111938
node_54: feature_name=cg15720017	feature_id[168].value > threshold=0.7067741453647614
node_60: feature_name=cg23690444	feature_id[120].value <= threshold=0.16037724912166595
node_61: feature_name=cg00567872	feature_id[236].value > threshold=0.7005765736103058
node_85: feature_name=cg01014262	feature_id[764].value <= threshold=0.5737917125225067
node_86: feature_name=cg22946562	feature_id[1148].value <= threshold=0.6328203976154327
node_87: feature_name=cg18120975	feature_id[2767].value > threshold=0.7081544995307922
node_91: feature_name=cg17171539	feature_id[25].value > threshold=0.501024603843689
node_111: feature_name=cg21241424	feature_id[1155].value > threshold=0.7665272355079651
Class: melanoma (MEL)	

Rules_45	passed counts:8
node_0: feature_name=cg11915444	feature_id[1171].value > threshold=0.3402601182460785
node_10: feature_name=cg12109728	feature_id[2810].value > threshold=0.7068270146846771
node_20: feature_name=cg16412000	feature_id[789].value > threshold=0.6741803884506226
node_24: feature_name=cg17115147	feature_id[1434].value <= threshold=0.2961975038051605
node_25: feature_name=cg24474622	feature_id[2737].value > threshold=0.7211934924125671
node_29: feature_name=cg10472711	feature_id[612].value > threshold=0.45985929667949677
node_37: feature_name=cg01937669	feature_id[188].value <= threshold=0.4809463769197464
node_38: feature_name=cg02157052	feature_id[451].value <= threshold=0.2869153320789337
node_39: feature_name=cg18751958	feature_id[1427].value > threshold=0.4813787043094635
node_41: feature_name=cg22512847	feature_id[1152].value > threshold=0.5129324495792389
node_43: feature_name=cg23345038	feature_id[1147].value > threshold=0.6318148374557495
node_45: feature_name=cg17439009	feature_id[1815].value <= threshold=0.48514117300510406
node_46: feature_name=cg17537493	feature_id[2772].value > threshold=0.5825372636318207
node_52: feature_name=cg01683570	feature_id[110].value > threshold=0.6040188074111938
node_54: feature_name=cg15720017	feature_id[168].value > threshold=0.7067741453647614
node_60: feature_name=cg23690444	feature_id[120].value <= threshold=0.16037724912166595
node_61: feature_name=cg00567872	feature_id[236].value > threshold=0.7005765736103058
node_85: feature_name=cg01014262	feature_id[764].value <= threshold=0.5737917125225067
node_86: feature_name=cg22946562	feature_id[1148].value > threshold=0.6328203976154327
node_134: feature_name=cg14514032	feature_id[202].value <= threshold=0.4389604330062866

node_135: feature_name=cg13488220	feature_id[241].value > threshold=0.7352153658866882
node_143: feature_name=cg03840920	feature_id[256].value > threshold=0.502630889415741
node_147: feature_name=cg13634090	feature_id[822].value > threshold=0.7207068204879761
node_151: feature_name=cg19584674	feature_id[2364].value <= threshold=0.41758519411087036
node_152: feature_name=cg07344990	feature_id[411].value > threshold=0.5484964847564697
node_158: feature_name=cg10298992	feature_id[3066].value > threshold=0.5707973837852478
node_174: feature_name=cg11843516	feature_id[20].value <= threshold=0.5590928494930267
node_175: feature_name=cg05185926	feature_id[448].value > threshold=0.17318664491176605
node_177: feature_name=cg17009731	feature_id[1220].value > threshold=0.6666513085365295
node_179: feature_name=cg01380319	feature_id[515].value <= threshold=0.39251960813999176
node_180: feature_name=cg20700977	feature_id[303].value > threshold=0.8422040641307831
node_184: feature_name=cg24259291	feature_id[164].value > threshold=0.4872268736362457
node_188: feature_name=cg08097657	feature_id[67].value > threshold=0.3371318429708481
node_190: feature_name=cg07891483	feature_id[59].value > threshold=0.6211732923984528
node_194: feature_name=cg20122645	feature_id[275].value > threshold=0.6094755828380585
node_196: feature_name=cg04026354	feature_id[1763].value <= threshold=0.4341808259487152
node_197: feature_name=cg10149889	feature_id[1017].value > threshold=0.6150195300579071
node_201: feature_name=cg14890730	feature_id[337].value <= threshold=0.4372602105140686
node_202: feature_name=cg08331427	feature_id[132].value > threshold=0.5443233847618103
node_208: feature_name=cg22730007	feature_id[2544].value > threshold=0.28619489073753357
node_210: feature_name=cg17193551	feature_id[2057].value > threshold=0.4393353909254074
node_212: feature_name=cg14671764	feature_id[584].value <= threshold=0.9353199899196625

node_213: feature_name=cg20140662	feature_id[2044].value > threshold=0.38030287623405457
node_215: feature_name=cg07891483	feature_id[59].value > threshold=0.829958975315094
node_219: feature_name=cg04884579	feature_id[1861].value <= threshold=0.6106154918670654
node_220: feature_name=cg25066857	feature_id[3759].value <= threshold=0.8149203658103943
node_221: feature_name=cg04813695	feature_id[1758].value <= threshold=0.8937293887138367
Class: myositis proliferans (MP)	
Rules_46	passed counts:8
node_0: feature_name=cg11915444	feature_id[1171].value > threshold=0.3402601182460785
node_10: feature_name=cg12109728	feature_id[2810].value > threshold=0.7068270146846771
node_20: feature_name=cg16412000	feature_id[789].value > threshold=0.6741803884506226
node_24: feature_name=cg17115147	feature_id[1434].value <= threshold=0.2961975038051605
node_25: feature_name=cg24474622	feature_id[2737].value > threshold=0.7211934924125671
node_29: feature_name=cg10472711	feature_id[612].value > threshold=0.45985929667949677
node_37: feature_name=cg01937669	feature_id[188].value <= threshold=0.4809463769197464
node_38: feature_name=cg02157052	feature_id[451].value <= threshold=0.2869153320789337
node_39: feature_name=cg18751958	feature_id[1427].value > threshold=0.4813787043094635
node_41: feature_name=cg22512847	feature_id[1152].value > threshold=0.5129324495792389
node_43: feature_name=cg23345038	feature_id[1147].value > threshold=0.6318148374557495
node_45: feature_name=cg17439009	feature_id[1815].value <= threshold=0.48514117300510406
node_46: feature_name=cg17537493	feature_id[2772].value > threshold=0.5825372636318207
node_52: feature_name=cg01683570	feature_id[110].value > threshold=0.6040188074111938
node_54: feature_name=cg15720017	feature_id[168].value > threshold=0.7067741453647614

node_60: feature_name=cg23690444	feature_id[120].value <= threshold=0.16037724912166595
node_61: feature_name=cg00567872	feature_id[236].value > threshold=0.7005765736103058
node_85: feature_name=cg01014262	feature_id[764].value <= threshold=0.5737917125225067
node_86: feature_name=cg22946562	feature_id[1148].value > threshold=0.6328203976154327
node_134: feature_name=cg14514032	feature_id[202].value <= threshold=0.4389604330062866
node_135: feature_name=cg13488220	feature_id[241].value > threshold=0.7352153658866882
node_143: feature_name=cg03840920	feature_id[256].value > threshold=0.502630889415741
node_147: feature_name=cg13634090	feature_id[822].value > threshold=0.7207068204879761
node_151: feature_name=cg19584674	feature_id[2364].value <= threshold=0.41758519411087036
node_152: feature_name=cg07344990	feature_id[411].value > threshold=0.5484964847564697
node_158: feature_name=cg10298992	feature_id[3066].value > threshold=0.5707973837852478
node_174: feature_name=cg11843516	feature_id[20].value <= threshold=0.5590928494930267
node_175: feature_name=cg05185926	feature_id[448].value > threshold=0.17318664491176605
node_177: feature_name=cg17009731	feature_id[1220].value > threshold=0.6666513085365295
node_179: feature_name=cg01380319	feature_id[515].value <= threshold=0.39251960813999176
node_180: feature_name=cg20700977	feature_id[303].value > threshold=0.8422040641307831
node_184: feature_name=cg24259291	feature_id[164].value > threshold=0.4872268736362457
node_188: feature_name=cg08097657	feature_id[67].value > threshold=0.3371318429708481
node_190: feature_name=cg07891483	feature_id[59].value > threshold=0.6211732923984528
node_194: feature_name=cg20122645	feature_id[275].value <= threshold=0.6094755828380585
Class: cutaneous squamous cell carcinoma (SCC (CUT))	

Rules_47	passed counts:7
node_0: feature_name=cg11915444	feature_id[1171].value > threshold=0.3402601182460785
node_10: feature_name=cg12109728	feature_id[2810].value > threshold=0.7068270146846771
node_20: feature_name=cg16412000	feature_id[789].value > threshold=0.6741803884506226
node_24: feature_name=cg17115147	feature_id[1434].value <= threshold=0.2961975038051605
node_25: feature_name=cg24474622	feature_id[2737].value > threshold=0.7211934924125671
node_29: feature_name=cg10472711	feature_id[612].value > threshold=0.45985929667949677
node_37: feature_name=cg01937669	feature_id[188].value <= threshold=0.4809463769197464
node_38: feature_name=cg02157052	feature_id[451].value <= threshold=0.2869153320789337
node_39: feature_name=cg18751958	feature_id[1427].value > threshold=0.4813787043094635
node_41: feature_name=cg22512847	feature_id[1152].value > threshold=0.5129324495792389
node_43: feature_name=cg23345038	feature_id[1147].value > threshold=0.6318148374557495
node_45: feature_name=cg17439009	feature_id[1815].value <= threshold=0.48514117300510406
node_46: feature_name=cg17537493	feature_id[2772].value > threshold=0.5825372636318207
node_52: feature_name=cg01683570	feature_id[110].value > threshold=0.6040188074111938
node_54: feature_name=cg15720017	feature_id[168].value > threshold=0.7067741453647614
node_60: feature_name=cg23690444	feature_id[120].value <= threshold=0.16037724912166595
node_61: feature_name=cg00567872	feature_id[236].value > threshold=0.7005765736103058
node_85: feature_name=cg01014262	feature_id[764].value <= threshold=0.5737917125225067
node_86: feature_name=cg22946562	feature_id[1148].value > threshold=0.6328203976154327
node_134: feature_name=cg14514032	feature_id[202].value <= threshold=0.4389604330062866
node_135: feature_name=cg13488220	feature_id[241].value > threshold=0.7352153658866882

node_143: feature_name=cg03840920	feature_id[256].value > threshold=0.502630889415741
node_147: feature_name=cg13634090	feature_id[822].value > threshold=0.7207068204879761
node_151: feature_name=cg19584674	feature_id[2364].value <= threshold=0.41758519411087036
node_152: feature_name=cg07344990	feature_id[411].value > threshold=0.5484964847564697
node_158: feature_name=cg10298992	feature_id[3066].value > threshold=0.5707973837852478
node_174: feature_name=cg11843516	feature_id[20].value <= threshold=0.5590928494930267
node_175: feature_name=cg05185926	feature_id[448].value > threshold=0.17318664491176605
node_177: feature_name=cg17009731	feature_id[1220].value > threshold=0.6666513085365295
node_179: feature_name=cg01380319	feature_id[515].value <= threshold=0.39251960813999176
node_180: feature_name=cg20700977	feature_id[303].value > threshold=0.8422040641307831
node_184: feature_name=cg24259291	feature_id[164].value > threshold=0.4872268736362457
node_188: feature_name=cg08097657	feature_id[67].value > threshold=0.3371318429708481
node_190: feature_name=cg07891483	feature_id[59].value > threshold=0.6211732923984528
node_194: feature_name=cg20122645	feature_id[275].value > threshold=0.6094755828380585
node_196: feature_name=cg04026354	feature_id[1763].value <= threshold=0.4341808259487152
node_197: feature_name=cg10149889	feature_id[1017].value > threshold=0.6150195300579071
node_201: feature_name=cg14890730	feature_id[337].value <= threshold=0.4372602105140686
node_202: feature_name=cg08331427	feature_id[132].value > threshold=0.5443233847618103
node_208: feature_name=cg22730007	feature_id[2544].value > threshold=0.28619489073753357
node_210: feature_name=cg17193551	feature_id[2057].value > threshold=0.4393353909254074
node_212: feature_name=cg14671764	feature_id[584].value <= threshold=0.9353199899196625
node_213: feature_name=cg20140662	feature_id[2044].value > threshold=0.38030287623405457

node_215: feature_name=cg07891483	feature_id[59].value > threshold=0.829958975315094
node_219: feature_name=cg04884579	feature_id[1861].value > threshold=0.6106154918670654
node_229: feature_name=cg19548524	feature_id[2985].value <= threshold=0.8623353838920593
node_230: feature_name=cg18763720	feature_id[523].value <= threshold=0.3843051791191101
Class: clear cell chondrosarcoma (CSA (CC))	
Rules_48	passed counts:7
node_0: feature_name=cg11915444	feature_id[1171].value > threshold=0.3402601182460785
node_10: feature_name=cg12109728	feature_id[2810].value > threshold=0.7068270146846771
node_20: feature_name=cg16412000	feature_id[789].value > threshold=0.6741803884506226
node_24: feature_name=cg17115147	feature_id[1434].value <= threshold=0.2961975038051605
node_25: feature_name=cg24474622	feature_id[2737].value > threshold=0.7211934924125671
node_29: feature_name=cg10472711	feature_id[612].value > threshold=0.45985929667949677
node_37: feature_name=cg01937669	feature_id[188].value <= threshold=0.4809463769197464
node_38: feature_name=cg02157052	feature_id[451].value <= threshold=0.2869153320789337
node_39: feature_name=cg18751958	feature_id[1427].value > threshold=0.4813787043094635
node_41: feature_name=cg22512847	feature_id[1152].value > threshold=0.5129324495792389
node_43: feature_name=cg23345038	feature_id[1147].value > threshold=0.6318148374557495
node_45: feature_name=cg17439009	feature_id[1815].value <= threshold=0.48514117300510406
node_46: feature_name=cg17537493	feature_id[2772].value > threshold=0.5825372636318207
node_52: feature_name=cg01683570	feature_id[110].value > threshold=0.6040188074111938
node_54: feature_name=cg15720017	feature_id[168].value > threshold=0.7067741453647614
node_60: feature_name=cg23690444	feature_id[120].value <= threshold=0.16037724912166595

node_61: feature_name=cg00567872	feature_id[236].value > threshold=0.7005765736103058
node_85: feature_name=cg01014262	feature_id[764].value <= threshold=0.5737917125225067
node_86: feature_name=cg22946562	feature_id[1148].value > threshold=0.6328203976154327
node_134: feature_name=cg14514032	feature_id[202].value <= threshold=0.4389604330062866
node_135: feature_name=cg13488220	feature_id[241].value > threshold=0.7352153658866882
node_143: feature_name=cg03840920	feature_id[256].value > threshold=0.502630889415741
node_147: feature_name=cg13634090	feature_id[822].value > threshold=0.7207068204879761
node_151: feature_name=cg19584674	feature_id[2364].value <= threshold=0.41758519411087036
node_152: feature_name=cg07344990	feature_id[411].value > threshold=0.5484964847564697
node_158: feature_name=cg10298992	feature_id[3066].value > threshold=0.5707973837852478
node_174: feature_name=cg11843516	feature_id[20].value <= threshold=0.5590928494930267
node_175: feature_name=cg05185926	feature_id[448].value > threshold=0.17318664491176605
node_177: feature_name=cg17009731	feature_id[1220].value > threshold=0.6666513085365295
node_179: feature_name=cg01380319	feature_id[515].value <= threshold=0.39251960813999176
node_180: feature_name=cg20700977	feature_id[303].value > threshold=0.8422040641307831
node_184: feature_name=cg24259291	feature_id[164].value > threshold=0.4872268736362457
node_188: feature_name=cg08097657	feature_id[67].value > threshold=0.3371318429708481
node_190: feature_name=cg07891483	feature_id[59].value > threshold=0.6211732923984528
node_194: feature_name=cg20122645	feature_id[275].value > threshold=0.6094755828380585
node_196: feature_name=cg04026354	feature_id[1763].value <= threshold=0.4341808259487152
node_197: feature_name=cg10149889	feature_id[1017].value > threshold=0.6150195300579071
node_201: feature_name=cg14890730	feature_id[337].value <= threshold=0.4372602105140686

node_202: feature_name=cg08331427	feature_id[132].value > threshold=0.5443233847618103
node_208: feature_name=cg22730007	feature_id[2544].value > threshold=0.28619489073753357
node_210: feature_name=cg17193551	feature_id[2057].value > threshold=0.4393353909254074
node_212: feature_name=cg14671764	feature_id[584].value <= threshold=0.9353199899196625
node_213: feature_name=cg20140662	feature_id[2044].value > threshold=0.38030287623405457
node_215: feature_name=cg07891483	feature_id[59].value <= threshold=0.829958975315094
node_216: feature_name=cg14487665	feature_id[3561].value > threshold=0.8762882351875305
Class: plexiform neurofibroma (NFB (PLEX))	
Rules_49	passed counts:7
node_0: feature_name=cg11915444	feature_id[1171].value > threshold=0.3402601182460785
node_10: feature_name=cg12109728	feature_id[2810].value > threshold=0.7068270146846771
node_20: feature_name=cg16412000	feature_id[789].value > threshold=0.6741803884506226
node_24: feature_name=cg17115147	feature_id[1434].value <= threshold=0.2961975038051605
node_25: feature_name=cg24474622	feature_id[2737].value > threshold=0.7211934924125671
node_29: feature_name=cg10472711	feature_id[612].value > threshold=0.45985929667949677
node_37: feature_name=cg01937669	feature_id[188].value <= threshold=0.4809463769197464
node_38: feature_name=cg02157052	feature_id[451].value <= threshold=0.2869153320789337
node_39: feature_name=cg18751958	feature_id[1427].value > threshold=0.4813787043094635
node_41: feature_name=cg22512847	feature_id[1152].value > threshold=0.5129324495792389
node_43: feature_name=cg23345038	feature_id[1147].value > threshold=0.6318148374557495
node_45: feature_name=cg17439009	feature_id[1815].value <= threshold=0.48514117300510406
node_46: feature_name=cg17537493	feature_id[2772].value > threshold=0.5825372636318207

node_52: feature_name=cg01683570	feature_id[110].value > threshold=0.6040188074111938
node_54: feature_name=cg15720017	feature_id[168].value > threshold=0.7067741453647614
node_60: feature_name=cg23690444	feature_id[120].value <= threshold=0.16037724912166595
node_61: feature_name=cg00567872	feature_id[236].value > threshold=0.7005765736103058
node_85: feature_name=cg01014262	feature_id[764].value <= threshold=0.5737917125225067
node_86: feature_name=cg22946562	feature_id[1148].value > threshold=0.6328203976154327
node_134: feature_name=cg14514032	feature_id[202].value <= threshold=0.4389604330062866
node_135: feature_name=cg13488220	feature_id[241].value > threshold=0.7352153658866882
node_143: feature_name=cg03840920	feature_id[256].value > threshold=0.502630889415741
node_147: feature_name=cg13634090	feature_id[822].value > threshold=0.7207068204879761
node_151: feature_name=cg19584674	feature_id[2364].value <= threshold=0.41758519411087036
node_152: feature_name=cg07344990	feature_id[411].value > threshold=0.5484964847564697
node_158: feature_name=cg10298992	feature_id[3066].value > threshold=0.5707973837852478
node_174: feature_name=cg11843516	feature_id[20].value <= threshold=0.5590928494930267
node_175: feature_name=cg05185926	feature_id[448].value > threshold=0.17318664491176605
node_177: feature_name=cg17009731	feature_id[1220].value > threshold=0.6666513085365295
node_179: feature_name=cg01380319	feature_id[515].value <= threshold=0.39251960813999176
node_180: feature_name=cg20700977	feature_id[303].value > threshold=0.8422040641307831
node_184: feature_name=cg24259291	feature_id[164].value > threshold=0.4872268736362457
node_188: feature_name=cg08097657	feature_id[67].value > threshold=0.3371318429708481
node_190: feature_name=cg07891483	feature_id[59].value > threshold=0.6211732923984528
node_194: feature_name=cg20122645	feature_id[275].value > threshold=0.6094755828380585

node_196: feature_name=cg04026354	feature_id[1763].value <= threshold=0.4341808259487152
node_197: feature_name=cg10149889	feature_id[1017].value > threshold=0.6150195300579071
node_201: feature_name=cg14890730	feature_id[337].value <= threshold=0.4372602105140686
node_202: feature_name=cg08331427	feature_id[132].value > threshold=0.5443233847618103
node_208: feature_name=cg22730007	feature_id[2544].value > threshold=0.28619489073753357
node_210: feature_name=cg17193551	feature_id[2057].value > threshold=0.4393353909254074
node_212: feature_name=cg14671764	feature_id[584].value <= threshold=0.9353199899196625
node_213: feature_name=cg20140662	feature_id[2044].value <= threshold=0.38030287623405457
Class: osteoblastoma (OB)	
Rules_50	passed counts:7
node_0: feature_name=cg11915444	feature_id[1171].value > threshold=0.3402601182460785
node_10: feature_name=cg12109728	feature_id[2810].value > threshold=0.7068270146846771
node_20: feature_name=cg16412000	feature_id[789].value > threshold=0.6741803884506226
node_24: feature_name=cg17115147	feature_id[1434].value <= threshold=0.2961975038051605
node_25: feature_name=cg24474622	feature_id[2737].value > threshold=0.7211934924125671
node_29: feature_name=cg10472711	feature_id[612].value > threshold=0.45985929667949677
node_37: feature_name=cg01937669	feature_id[188].value <= threshold=0.4809463769197464
node_38: feature_name=cg02157052	feature_id[451].value <= threshold=0.2869153320789337
node_39: feature_name=cg18751958	feature_id[1427].value > threshold=0.4813787043094635
node_41: feature_name=cg22512847	feature_id[1152].value > threshold=0.5129324495792389
node_43: feature_name=cg23345038	feature_id[1147].value > threshold=0.6318148374557495
node_45: feature_name=cg17439009	feature_id[1815].value <= threshold=0.48514117300510406

node_46: feature_name=cg17537493	feature_id[2772].value > threshold=0.5825372636318207
node_52: feature_name=cg01683570	feature_id[110].value > threshold=0.6040188074111938
node_54: feature_name=cg15720017	feature_id[168].value > threshold=0.7067741453647614
node_60: feature_name=cg23690444	feature_id[120].value <= threshold=0.16037724912166595
node_61: feature_name=cg00567872	feature_id[236].value > threshold=0.7005765736103058
node_85: feature_name=cg01014262	feature_id[764].value <= threshold=0.5737917125225067
node_86: feature_name=cg22946562	feature_id[1148].value > threshold=0.6328203976154327
node_134: feature_name=cg14514032	feature_id[202].value <= threshold=0.4389604330062866
node_135: feature_name=cg13488220	feature_id[241].value > threshold=0.7352153658866882
node_143: feature_name=cg03840920	feature_id[256].value > threshold=0.502630889415741
node_147: feature_name=cg13634090	feature_id[822].value > threshold=0.7207068204879761
node_151: feature_name=cg19584674	feature_id[2364].value <= threshold=0.41758519411087036
node_152: feature_name=cg07344990	feature_id[411].value > threshold=0.5484964847564697
node_158: feature_name=cg10298992	feature_id[3066].value > threshold=0.5707973837852478
node_174: feature_name=cg11843516	feature_id[20].value <= threshold=0.5590928494930267
node_175: feature_name=cg05185926	feature_id[448].value > threshold=0.17318664491176605
node_177: feature_name=cg17009731	feature_id[1220].value > threshold=0.6666513085365295
node_179: feature_name=cg01380319	feature_id[515].value <= threshold=0.39251960813999176
node_180: feature_name=cg20700977	feature_id[303].value > threshold=0.8422040641307831
node_184: feature_name=cg24259291	feature_id[164].value > threshold=0.4872268736362457
node_188: feature_name=cg08097657	feature_id[67].value > threshold=0.3371318429708481
node_190: feature_name=cg07891483	feature_id[59].value > threshold=0.6211732923984528

node_194: feature_name=cg20122645	feature_id[275].value > threshold=0.6094755828380585
node_196: feature_name=cg04026354	feature_id[1763].value <= threshold=0.4341808259487152
node_197: feature_name=cg10149889	feature_id[1017].value > threshold=0.6150195300579071
node_201: feature_name=cg14890730	feature_id[337].value <= threshold=0.4372602105140686
node_202: feature_name=cg08331427	feature_id[132].value > threshold=0.5443233847618103
node_208: feature_name=cg22730007	feature_id[2544].value > threshold=0.28619489073753357
node_210: feature_name=cg17193551	feature_id[2057].value <= threshold=0.4393353909254074
Class: low-grade fibromyxoid sarcoma (LGFMS)	
Rules_51	passed counts:7
node_0: feature_name=cg11915444	feature_id[1171].value > threshold=0.3402601182460785
node_10: feature_name=cg12109728	feature_id[2810].value > threshold=0.7068270146846771
node_20: feature_name=cg16412000	feature_id[789].value > threshold=0.6741803884506226
node_24: feature_name=cg17115147	feature_id[1434].value <= threshold=0.2961975038051605
node_25: feature_name=cg24474622	feature_id[2737].value > threshold=0.7211934924125671
node_29: feature_name=cg10472711	feature_id[612].value > threshold=0.45985929667949677
node_37: feature_name=cg01937669	feature_id[188].value <= threshold=0.4809463769197464
node_38: feature_name=cg02157052	feature_id[451].value <= threshold=0.2869153320789337
node_39: feature_name=cg18751958	feature_id[1427].value > threshold=0.4813787043094635
node_41: feature_name=cg22512847	feature_id[1152].value > threshold=0.5129324495792389
node_43: feature_name=cg23345038	feature_id[1147].value > threshold=0.6318148374557495
node_45: feature_name=cg17439009	feature_id[1815].value <= threshold=0.48514117300510406
node_46: feature_name=cg17537493	feature_id[2772].value > threshold=0.5825372636318207

node_52: feature_name=cg01683570	feature_id[110].value > threshold=0.6040188074111938
node_54: feature_name=cg15720017	feature_id[168].value > threshold=0.7067741453647614
node_60: feature_name=cg23690444	feature_id[120].value <= threshold=0.16037724912166595
node_61: feature_name=cg00567872	feature_id[236].value > threshold=0.7005765736103058
node_85: feature_name=cg01014262	feature_id[764].value <= threshold=0.5737917125225067
node_86: feature_name=cg22946562	feature_id[1148].value > threshold=0.6328203976154327
node_134: feature_name=cg14514032	feature_id[202].value <= threshold=0.4389604330062866
node_135: feature_name=cg13488220	feature_id[241].value > threshold=0.7352153658866882
node_143: feature_name=cg03840920	feature_id[256].value > threshold=0.502630889415741
node_147: feature_name=cg13634090	feature_id[822].value > threshold=0.7207068204879761
node_151: feature_name=cg19584674	feature_id[2364].value <= threshold=0.41758519411087036
node_152: feature_name=cg07344990	feature_id[411].value > threshold=0.5484964847564697
node_158: feature_name=cg10298992	feature_id[3066].value > threshold=0.5707973837852478
node_174: feature_name=cg11843516	feature_id[20].value <= threshold=0.5590928494930267
node_175: feature_name=cg05185926	feature_id[448].value > threshold=0.17318664491176605
node_177: feature_name=cg17009731	feature_id[1220].value > threshold=0.6666513085365295
node_179: feature_name=cg01380319	feature_id[515].value <= threshold=0.39251960813999176
node_180: feature_name=cg20700977	feature_id[303].value > threshold=0.8422040641307831
node_184: feature_name=cg24259291	feature_id[164].value > threshold=0.4872268736362457
node_188: feature_name=cg08097657	feature_id[67].value > threshold=0.3371318429708481
node_190: feature_name=cg07891483	feature_id[59].value > threshold=0.6211732923984528
node_194: feature_name=cg20122645	feature_id[275].value > threshold=0.6094755828380585

node_196: feature_name=cg04026354	feature_id[1763].value <= threshold=0.4341808259487152
node_197: feature_name=cg10149889	feature_id[1017].value > threshold=0.6150195300579071
node_201: feature_name=cg14890730	feature_id[337].value <= threshold=0.4372602105140686
node_202: feature_name=cg08331427	feature_id[132].value > threshold=0.5443233847618103
node_208: feature_name=cg22730007	feature_id[2544].value <= threshold=0.28619489073753357
Class: mesenchymal chondrosarcoma (CSA (MES))	
Rules_52	passed counts:7
node_0: feature_name=cg11915444	feature_id[1171].value > threshold=0.3402601182460785
node_10: feature_name=cg12109728	feature_id[2810].value > threshold=0.7068270146846771
node_20: feature_name=cg16412000	feature_id[789].value > threshold=0.6741803884506226
node_24: feature_name=cg17115147	feature_id[1434].value <= threshold=0.2961975038051605
node_25: feature_name=cg24474622	feature_id[2737].value > threshold=0.7211934924125671
node_29: feature_name=cg10472711	feature_id[612].value > threshold=0.45985929667949677
node_37: feature_name=cg01937669	feature_id[188].value <= threshold=0.4809463769197464
node_38: feature_name=cg02157052	feature_id[451].value <= threshold=0.2869153320789337
node_39: feature_name=cg18751958	feature_id[1427].value > threshold=0.4813787043094635
node_41: feature_name=cg22512847	feature_id[1152].value > threshold=0.5129324495792389
node_43: feature_name=cg23345038	feature_id[1147].value > threshold=0.6318148374557495
node_45: feature_name=cg17439009	feature_id[1815].value <= threshold=0.48514117300510406
node_46: feature_name=cg17537493	feature_id[2772].value > threshold=0.5825372636318207
node_52: feature_name=cg01683570	feature_id[110].value > threshold=0.6040188074111938

node_54: feature_name=cg15720017	feature_id[168].value > threshold=0.7067741453647614
node_60: feature_name=cg23690444	feature_id[120].value <= threshold=0.16037724912166595
node_61: feature_name=cg00567872	feature_id[236].value > threshold=0.7005765736103058
node_85: feature_name=cg01014262	feature_id[764].value <= threshold=0.5737917125225067
node_86: feature_name=cg22946562	feature_id[1148].value > threshold=0.6328203976154327
node_134: feature_name=cg14514032	feature_id[202].value <= threshold=0.4389604330062866
node_135: feature_name=cg13488220	feature_id[241].value > threshold=0.7352153658866882
node_143: feature_name=cg03840920	feature_id[256].value > threshold=0.502630889415741
node_147: feature_name=cg13634090	feature_id[822].value > threshold=0.7207068204879761
node_151: feature_name=cg19584674	feature_id[2364].value <= threshold=0.41758519411087036
node_152: feature_name=cg07344990	feature_id[411].value > threshold=0.5484964847564697
node_158: feature_name=cg10298992	feature_id[3066].value > threshold=0.5707973837852478
node_174: feature_name=cg11843516	feature_id[20].value <= threshold=0.5590928494930267
node_175: feature_name=cg05185926	feature_id[448].value > threshold=0.17318664491176605
node_177: feature_name=cg17009731	feature_id[1220].value > threshold=0.6665130853652950
node_179: feature_name=cg01380319	feature_id[515].value <= threshold=0.39251960813999176
node_180: feature_name=cg20700977	feature_id[303].value > threshold=0.8422040641307831
node_184: feature_name=cg24259291	feature_id[164].value > threshold=0.4872268736362457
node_188: feature_name=cg08097657	feature_id[67].value > threshold=0.3371318429708481
node_190: feature_name=cg07891483	feature_id[59].value > threshold=0.6211732923984528
node_194: feature_name=cg20122645	feature_id[275].value > threshold=0.6094755828380585
node_196: feature_name=cg04026354	feature_id[1763].value <= threshold=0.4341808259487152

node_197: feature_name=cg10149889	feature_id[1017].value <= threshold=0.6150195300579071
node_198: feature_name=cg16489468	feature_id[196].value <= threshold=0.6215258538722992
Class: myositis ossificans (MO)	
Rules_53	passed counts:7
node_0: feature_name=cg11915444	feature_id[1171].value > threshold=0.3402601182460785
node_10: feature_name=cg12109728	feature_id[2810].value > threshold=0.7068270146846771
node_20: feature_name=cg16412000	feature_id[789].value > threshold=0.6741803884506226
node_24: feature_name=cg17115147	feature_id[1434].value <= threshold=0.2961975038051605
node_25: feature_name=cg24474622	feature_id[2737].value > threshold=0.7211934924125671
node_29: feature_name=cg10472711	feature_id[612].value > threshold=0.45985929667949677
node_37: feature_name=cg01937669	feature_id[188].value <= threshold=0.4809463769197464
node_38: feature_name=cg02157052	feature_id[451].value <= threshold=0.2869153320789337
node_39: feature_name=cg18751958	feature_id[1427].value > threshold=0.4813787043094635
node_41: feature_name=cg22512847	feature_id[1152].value > threshold=0.5129324495792389
node_43: feature_name=cg23345038	feature_id[1147].value > threshold=0.6318148374557495
node_45: feature_name=cg17439009	feature_id[1815].value <= threshold=0.48514117300510406
node_46: feature_name=cg17537493	feature_id[2772].value > threshold=0.5825372636318207
node_52: feature_name=cg01683570	feature_id[110].value > threshold=0.6040188074111938
node_54: feature_name=cg15720017	feature_id[168].value > threshold=0.7067741453647614
node_60: feature_name=cg23690444	feature_id[120].value <= threshold=0.16037724912166595
node_61: feature_name=cg00567872	feature_id[236].value > threshold=0.7005765736103058
node_85: feature_name=cg01014262	feature_id[764].value <= threshold=0.5737917125225067

node_86: feature_name=cg22946562	feature_id[1148].value > threshold=0.6328203976154327
node_134: feature_name=cg14514032	feature_id[202].value <= threshold=0.4389604330062866
node_135: feature_name=cg13488220	feature_id[241].value > threshold=0.7352153658866882
node_143: feature_name=cg03840920	feature_id[256].value > threshold=0.502630889415741
node_147: feature_name=cg13634090	feature_id[822].value > threshold=0.7207068204879761
node_151: feature_name=cg19584674	feature_id[2364].value <= threshold=0.41758519411087036
node_152: feature_name=cg07344990	feature_id[411].value > threshold=0.5484964847564697
node_158: feature_name=cg10298992	feature_id[3066].value > threshold=0.5707973837852478
node_174: feature_name=cg11843516	feature_id[20].value <= threshold=0.5590928494930267
node_175: feature_name=cg05185926	feature_id[448].value > threshold=0.17318664491176605
node_177: feature_name=cg17009731	feature_id[1220].value > threshold=0.6666513085365295
node_179: feature_name=cg01380319	feature_id[515].value <= threshold=0.39251960813999176
node_180: feature_name=cg20700977	feature_id[303].value <= threshold=0.8422040641307831
node_181: feature_name=cg06825317	feature_id[752].value <= threshold=0.3880208432674408
Class: leiomyoma (LMO)	
Rules_54	passed counts:7
node_0: feature_name=cg11915444	feature_id[1171].value > threshold=0.3402601182460785
node_10: feature_name=cg12109728	feature_id[2810].value > threshold=0.7068270146846771
node_20: feature_name=cg16412000	feature_id[789].value > threshold=0.6741803884506226
node_24: feature_name=cg17115147	feature_id[1434].value <= threshold=0.2961975038051605
node_25: feature_name=cg24474622	feature_id[2737].value > threshold=0.7211934924125671
node_29: feature_name=cg10472711	feature_id[612].value > threshold=0.45985929667949677

node_37: feature_name=cg01937669	feature_id[188].value <= threshold=0.4809463769197464
node_38: feature_name=cg02157052	feature_id[451].value <= threshold=0.2869153320789337
node_39: feature_name=cg18751958	feature_id[1427].value > threshold=0.4813787043094635
node_41: feature_name=cg22512847	feature_id[1152].value > threshold=0.5129324495792389
node_43: feature_name=cg23345038	feature_id[1147].value > threshold=0.6318148374557495
node_45: feature_name=cg17439009	feature_id[1815].value <= threshold=0.48514117300510406
node_46: feature_name=cg17537493	feature_id[2772].value > threshold=0.5825372636318207
node_52: feature_name=cg01683570	feature_id[110].value > threshold=0.6040188074111938
node_54: feature_name=cg15720017	feature_id[168].value > threshold=0.7067741453647614
node_60: feature_name=cg23690444	feature_id[120].value <= threshold=0.16037724912166595
node_61: feature_name=cg00567872	feature_id[236].value > threshold=0.7005765736103058
node_85: feature_name=cg01014262	feature_id[764].value <= threshold=0.5737917125225067
node_86: feature_name=cg22946562	feature_id[1148].value <= threshold=0.6328203976154327
node_87: feature_name=cg18120975	feature_id[2767].value > threshold=0.7081544995307922
node_91: feature_name=cg17171539	feature_id[25].value > threshold=0.501024603843689
node_111: feature_name=cg21241424	feature_id[1155].value <= threshold=0.7665272355079651
node_112: feature_name=cg21897315	feature_id[3800].value <= threshold=0.48165470361709595
Class: leiomyosarcoma (LMS)	
Rules_55	passed counts:6
node_0: feature_name=cg11915444	feature_id[1171].value > threshold=0.3402601182460785
node_10: feature_name=cg12109728	feature_id[2810].value > threshold=0.7068270146846771
node_20: feature_name=cg16412000	feature_id[789].value > threshold=0.6741803884506226

node_24: feature_name=cg17115147	feature_id[1434].value <= threshold=0.2961975038051605
node_25: feature_name=cg24474622	feature_id[2737].value > threshold=0.7211934924125671
node_29: feature_name=cg10472711	feature_id[612].value > threshold=0.4598529667949677
node_37: feature_name=cg01937669	feature_id[188].value <= threshold=0.4809463769197464
node_38: feature_name=cg02157052	feature_id[451].value <= threshold=0.2869153320789337
node_39: feature_name=cg18751958	feature_id[1427].value > threshold=0.4813787043094635
node_41: feature_name=cg22512847	feature_id[1152].value > threshold=0.5129324495792389
node_43: feature_name=cg23345038	feature_id[1147].value > threshold=0.6318148374557495
node_45: feature_name=cg17439009	feature_id[1815].value <= threshold=0.48514117300510406
node_46: feature_name=cg17537493	feature_id[2772].value > threshold=0.5825372636318207
node_52: feature_name=cg01683570	feature_id[110].value > threshold=0.6040188074111938
node_54: feature_name=cg15720017	feature_id[168].value > threshold=0.7067741453647614
node_60: feature_name=cg23690444	feature_id[120].value <= threshold=0.16037724912166595
node_61: feature_name=cg00567872	feature_id[236].value > threshold=0.7005765736103058
node_85: feature_name=cg01014262	feature_id[764].value <= threshold=0.5737917125225067
node_86: feature_name=cg22946562	feature_id[1148].value > threshold=0.6328203976154327
node_134: feature_name=cg14514032	feature_id[202].value > threshold=0.4389604330062866
node_312: feature_name=cg21670987	feature_id[1509].value <= threshold=0.32477423548698425
node_313: feature_name=cg12939390	feature_id[143].value > threshold=0.23070813715457916
node_315: feature_name=cg10907727	feature_id[284].value > threshold=0.6596133708953857
node_317: feature_name=cg21241424	feature_id[1155].value > threshold=0.7687841951847076
Class: small blue round cell tumour (SBRCT)	

Rules_56	passed counts:6
node_0: feature_name=cg11915444	feature_id[1171].value > threshold=0.3402601182460785
node_10: feature_name=cg12109728	feature_id[2810].value > threshold=0.7068270146846771
node_20: feature_name=cg16412000	feature_id[789].value > threshold=0.6741803884506226
node_24: feature_name=cg17115147	feature_id[1434].value <= threshold=0.2961975038051605
node_25: feature_name=cg24474622	feature_id[2737].value > threshold=0.7211934924125671
node_29: feature_name=cg10472711	feature_id[612].value > threshold=0.45985929667949677
node_37: feature_name=cg01937669	feature_id[188].value <= threshold=0.4809463769197464
node_38: feature_name=cg02157052	feature_id[451].value <= threshold=0.2869153320789337
node_39: feature_name=cg18751958	feature_id[1427].value > threshold=0.4813787043094635
node_41: feature_name=cg22512847	feature_id[1152].value > threshold=0.5129324495792389
node_43: feature_name=cg23345038	feature_id[1147].value > threshold=0.6318148374557495
node_45: feature_name=cg17439009	feature_id[1815].value <= threshold=0.48514117300510406
node_46: feature_name=cg17537493	feature_id[2772].value > threshold=0.5825372636318207
node_52: feature_name=cg01683570	feature_id[110].value > threshold=0.6040188074111938
node_54: feature_name=cg15720017	feature_id[168].value > threshold=0.7067741453647614
node_60: feature_name=cg23690444	feature_id[120].value <= threshold=0.16037724912166595
node_61: feature_name=cg00567872	feature_id[236].value > threshold=0.7005765736103058
node_85: feature_name=cg01014262	feature_id[764].value <= threshold=0.5737917125225067
node_86: feature_name=cg22946562	feature_id[1148].value > threshold=0.6328203976154327
node_134: feature_name=cg14514032	feature_id[202].value <= threshold=0.4389604330062866

node_135: feature_name=cg13488220	feature_id[241].value > threshold=0.7352153658866882
node_143: feature_name=cg03840920	feature_id[256].value > threshold=0.502630889415741
node_147: feature_name=cg13634090	feature_id[822].value > threshold=0.7207068204879761
node_151: feature_name=cg19584674	feature_id[2364].value <= threshold=0.41758519411087036
node_152: feature_name=cg07344990	feature_id[411].value > threshold=0.5484964847564697
node_158: feature_name=cg10298992	feature_id[3066].value > threshold=0.5707973837852478
node_174: feature_name=cg11843516	feature_id[20].value <= threshold=0.5590928494930267
node_175: feature_name=cg05185926	feature_id[448].value > threshold=0.17318664491176605
node_177: feature_name=cg17009731	feature_id[1220].value > threshold=0.6666513085365295
node_179: feature_name=cg01380319	feature_id[515].value <= threshold=0.39251960813999176
node_180: feature_name=cg20700977	feature_id[303].value > threshold=0.8422040641307831
node_184: feature_name=cg24259291	feature_id[164].value > threshold=0.4872268736362457
node_188: feature_name=cg08097657	feature_id[67].value > threshold=0.3371318429708481
node_190: feature_name=cg07891483	feature_id[59].value > threshold=0.6211732923984528
node_194: feature_name=cg20122645	feature_id[275].value > threshold=0.6094755828380585
node_196: feature_name=cg04026354	feature_id[1763].value <= threshold=0.4341808259487152
node_197: feature_name=cg10149889	feature_id[1017].value > threshold=0.6150195300579071
node_201: feature_name=cg14890730	feature_id[337].value <= threshold=0.4372602105140686
node_202: feature_name=cg08331427	feature_id[132].value > threshold=0.5443233847618103
node_208: feature_name=cg22730007	feature_id[2544].value > threshold=0.28619489073753357
node_210: feature_name=cg17193551	feature_id[2057].value > threshold=0.4393353909254074
node_212: feature_name=cg14671764	feature_id[584].value <= threshold=0.9353199899196625

node_213: feature_name=cg20140662	feature_id[2044].value > threshold=0.38030287623405457
node_215: feature_name=cg07891483	feature_id[59].value > threshold=0.829958975315094
node_219: feature_name=cg04884579	feature_id[1861].value > threshold=0.6106154918670654
node_229: feature_name=cg19548524	feature_id[2985].value > threshold=0.8623353838920593
node_233: feature_name=cg14788686	feature_id[3556].value <= threshold=0.35090239346027374
Class: sarcoma (SARC)	
Rules_57	passed counts:6
node_0: feature_name=cg11915444	feature_id[1171].value > threshold=0.3402601182460785
node_10: feature_name=cg12109728	feature_id[2810].value > threshold=0.7068270146846771
node_20: feature_name=cg16412000	feature_id[789].value > threshold=0.6741803884506226
node_24: feature_name=cg17115147	feature_id[1434].value <= threshold=0.2961975038051605
node_25: feature_name=cg24474622	feature_id[2737].value > threshold=0.7211934924125671
node_29: feature_name=cg10472711	feature_id[612].value > threshold=0.45985929667949677
node_37: feature_name=cg01937669	feature_id[188].value <= threshold=0.4809463769197464
node_38: feature_name=cg02157052	feature_id[451].value <= threshold=0.2869153320789337
node_39: feature_name=cg18751958	feature_id[1427].value > threshold=0.4813787043094635
node_41: feature_name=cg22512847	feature_id[1152].value > threshold=0.5129324495792389
node_43: feature_name=cg23345038	feature_id[1147].value > threshold=0.6318148374557495
node_45: feature_name=cg17439009	feature_id[1815].value <= threshold=0.48514117300510406
node_46: feature_name=cg17537493	feature_id[2772].value > threshold=0.5825372636318207
node_52: feature_name=cg01683570	feature_id[110].value > threshold=0.6040188074111938
node_54: feature_name=cg15720017	feature_id[168].value > threshold=0.7067741453647614

node_60: feature_name=cg23690444	feature_id[120].value <= threshold=0.16037724912166595
node_61: feature_name=cg00567872	feature_id[236].value > threshold=0.7005765736103058
node_85: feature_name=cg01014262	feature_id[764].value <= threshold=0.5737917125225067
node_86: feature_name=cg22946562	feature_id[1148].value <= threshold=0.6328203976154327
node_87: feature_name=cg18120975	feature_id[2767].value > threshold=0.7081544995307922
node_91: feature_name=cg17171539	feature_id[25].value > threshold=0.501024603843689
node_111: feature_name=cg21241424	feature_id[1155].value <= threshold=0.7665272355079651
node_112: feature_name=cg21897315	feature_id[3800].value > threshold=0.48165470361709595
node_114: feature_name=cg04375492	feature_id[2874].value <= threshold=0.7812598049640656
Class: sarcoma (SARC)	
Rules_58	passed counts:6
node_0: feature_name=cg11915444	feature_id[1171].value > threshold=0.3402601182460785
node_10: feature_name=cg12109728	feature_id[2810].value > threshold=0.7068270146846771
node_20: feature_name=cg16412000	feature_id[789].value > threshold=0.6741803884506226
node_24: feature_name=cg17115147	feature_id[1434].value <= threshold=0.2961975038051605
node_25: feature_name=cg24474622	feature_id[2737].value > threshold=0.7211934924125671
node_29: feature_name=cg10472711	feature_id[612].value > threshold=0.45985929667949677
node_37: feature_name=cg01937669	feature_id[188].value <= threshold=0.4809463769197464
node_38: feature_name=cg02157052	feature_id[451].value <= threshold=0.2869153320789337
node_39: feature_name=cg18751958	feature_id[1427].value > threshold=0.4813787043094635
node_41: feature_name=cg22512847	feature_id[1152].value > threshold=0.5129324495792389
node_43: feature_name=cg23345038	feature_id[1147].value > threshold=0.6318148374557495

node_45: feature_name=cg17439009	feature_id[1815].value <= threshold=0.48514117300510406
node_46: feature_name=cg17537493	feature_id[2772].value > threshold=0.5825372636318207
node_52: feature_name=cg01683570	feature_id[110].value > threshold=0.6040188074111938
node_54: feature_name=cg15720017	feature_id[168].value > threshold=0.7067741453647614
node_60: feature_name=cg23690444	feature_id[120].value <= threshold=0.16037724912166595
node_61: feature_name=cg00567872	feature_id[236].value > threshold=0.7005765736103058
node_85: feature_name=cg01014262	feature_id[764].value <= threshold=0.5737917125225067
node_86: feature_name=cg22946562	feature_id[1148].value <= threshold=0.6328203976154327
node_87: feature_name=cg18120975	feature_id[2767].value > threshold=0.7081544995307922
node_91: feature_name=cg17171539	feature_id[25].value <= threshold=0.501024603843689
node_92: feature_name=cg00007036	feature_id[1586].value <= threshold=0.943472146987915
node_93: feature_name=cg16570314	feature_id[479].value > threshold=0.7333994507789612
Class: atypical fibroxanthoma (AFX)/pleomorphic dermal sarcoma (PDS)	
Rules_59	passed counts:5
node_0: feature_name=cg11915444	feature_id[1171].value > threshold=0.3402601182460785
node_10: feature_name=cg12109728	feature_id[2810].value > threshold=0.7068270146846771
node_20: feature_name=cg16412000	feature_id[789].value > threshold=0.6741803884506226
node_24: feature_name=cg17115147	feature_id[1434].value <= threshold=0.2961975038051605
node_25: feature_name=cg24474622	feature_id[2737].value > threshold=0.7211934924125671
node_29: feature_name=cg10472711	feature_id[612].value > threshold=0.45985929667949677
node_37: feature_name=cg01937669	feature_id[188].value <= threshold=0.4809463769197464

node_38: feature_name=cg02157052	feature_id[451].value > threshold=0.2869153320789337
node_344: feature_name=cg21189849	feature_id[189].value > threshold=0.7746135592460632
node_348: feature_name=cg06937205	feature_id[751].value <= threshold=0.4331420361995697
Class: rhabdomyosarcoma (RMS)	
Rules_60	passed counts:5
node_0: feature_name=cg11915444	feature_id[1171].value > threshold=0.3402601182460785
node_10: feature_name=cg12109728	feature_id[2810].value > threshold=0.7068270146846771
node_20: feature_name=cg16412000	feature_id[789].value > threshold=0.6741803884506226
node_24: feature_name=cg17115147	feature_id[1434].value <= threshold=0.2961975038051605
node_25: feature_name=cg24474622	feature_id[2737].value > threshold=0.7211934924125671
node_29: feature_name=cg10472711	feature_id[612].value > threshold=0.45985929667949677
node_37: feature_name=cg01937669	feature_id[188].value <= threshold=0.4809463769197464
node_38: feature_name=cg02157052	feature_id[451].value <= threshold=0.2869153320789337
node_39: feature_name=cg18751958	feature_id[1427].value > threshold=0.4813787043094635
node_41: feature_name=cg22512847	feature_id[1152].value > threshold=0.5129324495792389
node_43: feature_name=cg23345038	feature_id[1147].value > threshold=0.6318148374557495
node_45: feature_name=cg17439009	feature_id[1815].value <= threshold=0.48514117300510406
node_46: feature_name=cg17537493	feature_id[2772].value > threshold=0.5825372636318207
node_52: feature_name=cg01683570	feature_id[110].value > threshold=0.6040188074111938
node_54: feature_name=cg15720017	feature_id[168].value > threshold=0.7067741453647614
node_60: feature_name=cg23690444	feature_id[120].value <= threshold=0.16037724912166595
node_61: feature_name=cg00567872	feature_id[236].value > threshold=0.7005765736103058

node_85: feature_name=cg01014262	feature_id[764].value <= threshold=0.5737917125225067
node_86: feature_name=cg22946562	feature_id[1148].value > threshold=0.6328203976154327
node_134: feature_name=cg14514032	feature_id[202].value > threshold=0.4389604330062866
node_312: feature_name=cg21670987	feature_id[1509].value <= threshold=0.32477423548698425
node_313: feature_name=cg12939390	feature_id[143].value > threshold=0.23070813715457916
node_315: feature_name=cg10907727	feature_id[284].value > threshold=0.6596133708953857
node_317: feature_name=cg21241424	feature_id[1155].value <= threshold=0.7687841951847076
node_318: feature_name=cg19691659	feature_id[953].value > threshold=0.6708230972290039
Class: low-grade endometrial stromal sarcoma (ESS (LG))	
Rules_61	passed counts:5
node_0: feature_name=cg11915444	feature_id[1171].value > threshold=0.3402601182460785
node_10: feature_name=cg12109728	feature_id[2810].value > threshold=0.7068270146846771
node_20: feature_name=cg16412000	feature_id[789].value > threshold=0.6741803884506226
node_24: feature_name=cg17115147	feature_id[1434].value <= threshold=0.2961975038051605
node_25: feature_name=cg24474622	feature_id[2737].value > threshold=0.7211934924125671
node_29: feature_name=cg10472711	feature_id[612].value > threshold=0.45985929667949677
node_37: feature_name=cg01937669	feature_id[188].value <= threshold=0.4809463769197464
node_38: feature_name=cg02157052	feature_id[451].value <= threshold=0.2869153320789337
node_39: feature_name=cg18751958	feature_id[1427].value > threshold=0.4813787043094635
node_41: feature_name=cg22512847	feature_id[1152].value > threshold=0.5129324495792389
node_43: feature_name=cg23345038	feature_id[1147].value > threshold=0.6318148374557495

node_45: feature_name=cg17439009	feature_id[1815].value <= threshold=0.48514117300510406
node_46: feature_name=cg17537493	feature_id[2772].value > threshold=0.5825372636318207
node_52: feature_name=cg01683570	feature_id[110].value > threshold=0.6040188074111938
node_54: feature_name=cg15720017	feature_id[168].value > threshold=0.7067741453647614
node_60: feature_name=cg23690444	feature_id[120].value <= threshold=0.16037724912166595
node_61: feature_name=cg00567872	feature_id[236].value > threshold=0.7005765736103058
node_85: feature_name=cg01014262	feature_id[764].value <= threshold=0.5737917125225067
node_86: feature_name=cg22946562	feature_id[1148].value > threshold=0.6328203976154327
node_134: feature_name=cg14514032	feature_id[202].value <= threshold=0.4389604330062866
node_135: feature_name=cg13488220	feature_id[241].value > threshold=0.7352153658866882
node_143: feature_name=cg03840920	feature_id[256].value > threshold=0.502630889415741
node_147: feature_name=cg13634090	feature_id[822].value > threshold=0.7207068204879761
node_151: feature_name=cg19584674	feature_id[2364].value <= threshold=0.41758519411087036
node_152: feature_name=cg07344990	feature_id[411].value > threshold=0.5484964847564697
node_158: feature_name=cg10298992	feature_id[3066].value > threshold=0.5707973837852478
node_174: feature_name=cg11843516	feature_id[20].value <= threshold=0.5590928494930267
node_175: feature_name=cg05185926	feature_id[448].value > threshold=0.17318664491176605
node_177: feature_name=cg17009731	feature_id[1220].value > threshold=0.6666513085365295
node_179: feature_name=cg01380319	feature_id[515].value <= threshold=0.39251960813999176
node_180: feature_name=cg20700977	feature_id[303].value > threshold=0.8422040641307831
node_184: feature_name=cg24259291	feature_id[164].value > threshold=0.4872268736362457
node_188: feature_name=cg08097657	feature_id[67].value > threshold=0.3371318429708481

node_190: feature_name=cg07891483	feature_id[59].value > threshold=0.6211732923984528
node_194: feature_name=cg20122645	feature_id[275].value > threshold=0.6094755828380585
node_196: feature_name=cg04026354	feature_id[1763].value <= threshold=0.4341808259487152
node_197: feature_name=cg10149889	feature_id[1017].value > threshold=0.6150195300579071
node_201: feature_name=cg14890730	feature_id[337].value <= threshold=0.4372602105140686
node_202: feature_name=cg08331427	feature_id[132].value > threshold=0.5443233847618103
node_208: feature_name=cg22730007	feature_id[2544].value > threshold=0.28619489073753357
node_210: feature_name=cg17193551	feature_id[2057].value > threshold=0.4393353909254074
node_212: feature_name=cg14671764	feature_id[584].value <= threshold=0.9353199899196625
node_213: feature_name=cg20140662	feature_id[2044].value > threshold=0.38030287623405457
node_215: feature_name=cg07891483	feature_id[59].value > threshold=0.829958975315094
node_219: feature_name=cg04884579	feature_id[1861].value > threshold=0.6106154918670654
node_229: feature_name=cg19548524	feature_id[2985].value > threshold=0.8623353838920593
node_233: feature_name=cg14788686	feature_id[3556].value > threshold=0.35090239346027374
node_235: feature_name=cg05380734	feature_id[2470].value > threshold=0.7112160623073578
Class: clear cell sarcoma of soft parts (CCS)	
Rules_62	passed counts:5
node_0: feature_name=cg11915444	feature_id[1171].value > threshold=0.3402601182460785
node_10: feature_name=cg12109728	feature_id[2810].value > threshold=0.7068270146846771
node_20: feature_name=cg16412000	feature_id[789].value > threshold=0.6741803884506226
node_24: feature_name=cg17115147	feature_id[1434].value <= threshold=0.2961975038051605
node_25: feature_name=cg24474622	feature_id[2737].value > threshold=0.7211934924125671

node_29: feature_name=cg10472711	feature_id[612].value > threshold=0.45985929667949677
node_37: feature_name=cg01937669	feature_id[188].value <= threshold=0.4809463769197464
node_38: feature_name=cg02157052	feature_id[451].value <= threshold=0.2869153320789337
node_39: feature_name=cg18751958	feature_id[1427].value > threshold=0.4813787043094635
node_41: feature_name=cg22512847	feature_id[1152].value > threshold=0.5129324495792389
node_43: feature_name=cg23345038	feature_id[1147].value > threshold=0.6318148374557495
node_45: feature_name=cg17439009	feature_id[1815].value <= threshold=0.48514117300510406
node_46: feature_name=cg17537493	feature_id[2772].value > threshold=0.5825372636318207
node_52: feature_name=cg01683570	feature_id[110].value > threshold=0.6040188074111938
node_54: feature_name=cg15720017	feature_id[168].value > threshold=0.7067741453647614
node_60: feature_name=cg23690444	feature_id[120].value <= threshold=0.16037724912166595
node_61: feature_name=cg00567872	feature_id[236].value > threshold=0.7005765736103058
node_85: feature_name=cg01014262	feature_id[764].value <= threshold=0.5737917125225067
node_86: feature_name=cg22946562	feature_id[1148].value <= threshold=0.6328203976154327
node_87: feature_name=cg18120975	feature_id[2767].value > threshold=0.7081544995307922
node_91: feature_name=cg17171539	feature_id[25].value > threshold=0.501024603843689
node_111: feature_name=cg21241424	feature_id[1155].value <= threshold=0.7665272355079651
node_112: feature_name=cg21897315	feature_id[3800].value > threshold=0.48165470361709595
node_114: feature_name=cg04375492	feature_id[2874].value > threshold=0.7812598049640656
node_116: feature_name=cg09892984	feature_id[3615].value > threshold=0.6039115190505981
node_118: feature_name=cg05076082	feature_id[215].value <= threshold=0.7612724006175995
Class: atypical fibroxanthoma (AFX)/pleomorphic dermal sarcoma (PDS)	

Rules_63	passed counts:5
node_0: feature_name=cg11915444	feature_id[1171].value > threshold=0.3402601182460785
node_10: feature_name=cg12109728	feature_id[2810].value > threshold=0.7068270146846771
node_20: feature_name=cg16412000	feature_id[789].value > threshold=0.6741803884506226
node_24: feature_name=cg17115147	feature_id[1434].value <= threshold=0.2961975038051605
node_25: feature_name=cg24474622	feature_id[2737].value > threshold=0.7211934924125671
node_29: feature_name=cg10472711	feature_id[612].value > threshold=0.45985929667949677
node_37: feature_name=cg01937669	feature_id[188].value <= threshold=0.4809463769197464
node_38: feature_name=cg02157052	feature_id[451].value <= threshold=0.2869153320789337
node_39: feature_name=cg18751958	feature_id[1427].value > threshold=0.4813787043094635
node_41: feature_name=cg22512847	feature_id[1152].value > threshold=0.5129324495792389
node_43: feature_name=cg23345038	feature_id[1147].value > threshold=0.6318148374557495
node_45: feature_name=cg17439009	feature_id[1815].value <= threshold=0.48514117300510406
node_46: feature_name=cg17537493	feature_id[2772].value > threshold=0.5825372636318207
node_52: feature_name=cg01683570	feature_id[110].value > threshold=0.6040188074111938
node_54: feature_name=cg15720017	feature_id[168].value > threshold=0.7067741453647614
node_60: feature_name=cg23690444	feature_id[120].value <= threshold=0.16037724912166595
node_61: feature_name=cg00567872	feature_id[236].value > threshold=0.7005765736103058
node_85: feature_name=cg01014262	feature_id[764].value <= threshold=0.5737917125225067
node_86: feature_name=cg22946562	feature_id[1148].value <= threshold=0.6328203976154327
node_87: feature_name=cg18120975	feature_id[2767].value > threshold=0.7081544995307922

node_91: feature_name=cg17171539	feature_id[25].value > threshold=0.501024603843689
node_111: feature_name=cg21241424	feature_id[1155].value <= threshold=0.7665272355079651
node_112: feature_name=cg21897315	feature_id[3800].value > threshold=0.48165470361709595
node_114: feature_name=cg04375492	feature_id[2874].value > threshold=0.7812598049640656
node_116: feature_name=cg09892984	feature_id[3615].value <= threshold=0.6039115190505981
Class: clear cell sarcoma of soft parts (CCS)	
Rules_64	passed counts:4
node_0: feature_name=cg11915444	feature_id[1171].value > threshold=0.3402601182460785
node_10: feature_name=cg12109728	feature_id[2810].value > threshold=0.7068270146846771
node_20: feature_name=cg16412000	feature_id[789].value > threshold=0.6741803884506226
node_24: feature_name=cg17115147	feature_id[1434].value <= threshold=0.2961975038051605
node_25: feature_name=cg24474622	feature_id[2737].value > threshold=0.7211934924125671
node_29: feature_name=cg10472711	feature_id[612].value > threshold=0.45985929667949677
node_37: feature_name=cg01937669	feature_id[188].value <= threshold=0.4809463769197464
node_38: feature_name=cg02157052	feature_id[451].value > threshold=0.2869153320789337
node_344: feature_name=cg21189849	feature_id[189].value > threshold=0.7746135592460632
node_348: feature_name=cg06937205	feature_id[751].value > threshold=0.4331420361995697
node_350: feature_name=cg20165037	feature_id[2558].value > threshold=0.8168091177940369
node_352: feature_name=cg17454920	feature_id[3003].value <= threshold=0.5224908739328384
Class: malignant rhabdoid tumour (MRT)	
Rules_65	passed counts:4
node_0: feature_name=cg11915444	feature_id[1171].value > threshold=0.3402601182460785

node_10: feature_name=cg12109728	feature_id[2810].value > threshold=0.7068270146846771
node_20: feature_name=cg16412000	feature_id[789].value > threshold=0.6741803884506226
node_24: feature_name=cg17115147	feature_id[1434].value <= threshold=0.2961975038051605
node_25: feature_name=cg24474622	feature_id[2737].value > threshold=0.7211934924125671
node_29: feature_name=cg10472711	feature_id[612].value > threshold=0.45985929667949677
node_37: feature_name=cg01937669	feature_id[188].value <= threshold=0.4809463769197464
node_38: feature_name=cg02157052	feature_id[451].value > threshold=0.2869153320789337
node_344: feature_name=cg21189849	feature_id[189].value > threshold=0.7746135592460632
node_348: feature_name=cg06937205	feature_id[751].value > threshold=0.4331420361995697
node_350: feature_name=cg20165037	feature_id[2558].value <= threshold=0.8168091177940369
Class: muscle tissue (MUS)	
Rules_66	passed counts:4
node_0: feature_name=cg11915444	feature_id[1171].value > threshold=0.3402601182460785
node_10: feature_name=cg12109728	feature_id[2810].value > threshold=0.7068270146846771
node_20: feature_name=cg16412000	feature_id[789].value > threshold=0.6741803884506226
node_24: feature_name=cg17115147	feature_id[1434].value <= threshold=0.2961975038051605
node_25: feature_name=cg24474622	feature_id[2737].value > threshold=0.7211934924125671
node_29: feature_name=cg10472711	feature_id[612].value > threshold=0.45985929667949677
node_37: feature_name=cg01937669	feature_id[188].value <= threshold=0.4809463769197464
node_38: feature_name=cg02157052	feature_id[451].value <= threshold=0.2869153320789337
node_39: feature_name=cg18751958	feature_id[1427].value > threshold=0.4813787043094635
node_41: feature_name=cg22512847	feature_id[1152].value > threshold=0.5129324495792389

node_43: feature_name=cg23345038	feature_id[1147].value > threshold=0.6318148374557495
node_45: feature_name=cg17439009	feature_id[1815].value <= threshold=0.48514117300510406
node_46: feature_name=cg17537493	feature_id[2772].value > threshold=0.5825372636318207
node_52: feature_name=cg01683570	feature_id[110].value > threshold=0.6040188074111938
node_54: feature_name=cg15720017	feature_id[168].value > threshold=0.7067741453647614
node_60: feature_name=cg23690444	feature_id[120].value <= threshold=0.16037724912166595
node_61: feature_name=cg00567872	feature_id[236].value > threshold=0.7005765736103058
node_85: feature_name=cg01014262	feature_id[764].value <= threshold=0.5737917125225067
node_86: feature_name=cg22946562	feature_id[1148].value > threshold=0.6328203976154327
node_134: feature_name=cg14514032	feature_id[202].value <= threshold=0.4389604330062866
node_135: feature_name=cg13488220	feature_id[241].value > threshold=0.7352153658866882
node_143: feature_name=cg03840920	feature_id[256].value > threshold=0.502630889415741
node_147: feature_name=cg13634090	feature_id[822].value > threshold=0.7207068204879761
node_151: feature_name=cg19584674	feature_id[2364].value <= threshold=0.41758519411087036
node_152: feature_name=cg07344990	feature_id[411].value > threshold=0.5484964847564697
node_158: feature_name=cg10298992	feature_id[3066].value > threshold=0.5707973837852478
node_174: feature_name=cg11843516	feature_id[20].value <= threshold=0.5590928494930267
node_175: feature_name=cg05185926	feature_id[448].value > threshold=0.17318664491176605
node_177: feature_name=cg17009731	feature_id[1220].value > threshold=0.6666513085365295
node_179: feature_name=cg01380319	feature_id[515].value <= threshold=0.39251960813999176
node_180: feature_name=cg20700977	feature_id[303].value > threshold=0.8422040641307831
node_184: feature_name=cg24259291	feature_id[164].value > threshold=0.4872268736362457

node_188: feature_name=cg08097657	feature_id[67].value > threshold=0.3371318429708481
node_190: feature_name=cg07891483	feature_id[59].value > threshold=0.6211732923984528
node_194: feature_name=cg20122645	feature_id[275].value > threshold=0.6094755828380585
node_196: feature_name=cg04026354	feature_id[1763].value <= threshold=0.4341808259487152
node_197: feature_name=cg10149889	feature_id[1017].value > threshold=0.6150195300579071
node_201: feature_name=cg14890730	feature_id[337].value <= threshold=0.4372602105140686
node_202: feature_name=cg08331427	feature_id[132].value > threshold=0.5443233847618103
node_208: feature_name=cg22730007	feature_id[2544].value > threshold=0.28619489073753357
node_210: feature_name=cg17193551	feature_id[2057].value > threshold=0.4393353909254074
node_212: feature_name=cg14671764	feature_id[584].value <= threshold=0.9353199899196625
node_213: feature_name=cg20140662	feature_id[2044].value > threshold=0.38030287623405457
node_215: feature_name=cg07891483	feature_id[59].value > threshold=0.829958975315094
node_219: feature_name=cg04884579	feature_id[1861].value > threshold=0.6106154918670654
node_229: feature_name=cg19548524	feature_id[2985].value > threshold=0.8623353838920593
node_233: feature_name=cg14788686	feature_id[3556].value > threshold=0.35090239346027374
node_235: feature_name=cg05380734	feature_id[2470].value <= threshold=0.7112160623073578
node_236: feature_name=cg01166827	feature_id[3708].value > threshold=0.6359498500823975
node_238: feature_name=cg13251842	feature_id[714].value > threshold=0.8916542232036591
node_240: feature_name=cg02829279	feature_id[623].value > threshold=0.450528159737587
node_242: feature_name=cg24358599	feature_id[86].value <= threshold=0.8132582604885101
node_243: feature_name=cg01824933	feature_id[300].value > threshold=0.5986265540122986
Class: well differentiated liposarcoma (WDLS)/dedifferentiated liposarcoma (DDLS)	

Rules_67	passed counts:4
node_0: feature_name=cg11915444	feature_id[1171].value > threshold=0.3402601182460785
node_10: feature_name=cg12109728	feature_id[2810].value > threshold=0.7068270146846771
node_20: feature_name=cg16412000	feature_id[789].value > threshold=0.6741803884506226
node_24: feature_name=cg17115147	feature_id[1434].value <= threshold=0.2961975038051605
node_25: feature_name=cg24474622	feature_id[2737].value > threshold=0.7211934924125671
node_29: feature_name=cg10472711	feature_id[612].value > threshold=0.45985929667949677
node_37: feature_name=cg01937669	feature_id[188].value <= threshold=0.4809463769197464
node_38: feature_name=cg02157052	feature_id[451].value <= threshold=0.2869153320789337
node_39: feature_name=cg18751958	feature_id[1427].value > threshold=0.4813787043094635
node_41: feature_name=cg22512847	feature_id[1152].value > threshold=0.5129324495792389
node_43: feature_name=cg23345038	feature_id[1147].value > threshold=0.6318148374557495
node_45: feature_name=cg17439009	feature_id[1815].value <= threshold=0.48514117300510406
node_46: feature_name=cg17537493	feature_id[2772].value > threshold=0.5825372636318207
node_52: feature_name=cg01683570	feature_id[110].value > threshold=0.6040188074111938
node_54: feature_name=cg15720017	feature_id[168].value > threshold=0.7067741453647614
node_60: feature_name=cg23690444	feature_id[120].value <= threshold=0.16037724912166595
node_61: feature_name=cg00567872	feature_id[236].value > threshold=0.7005765736103058
node_85: feature_name=cg01014262	feature_id[764].value <= threshold=0.5737917125225067
node_86: feature_name=cg22946562	feature_id[1148].value > threshold=0.6328203976154327
node_134: feature_name=cg14514032	feature_id[202].value <= threshold=0.4389604330062866

node_135: feature_name=cg13488220	feature_id[241].value > threshold=0.7352153658866882
node_143: feature_name=cg03840920	feature_id[256].value > threshold=0.502630889415741
node_147: feature_name=cg13634090	feature_id[822].value > threshold=0.7207068204879761
node_151: feature_name=cg19584674	feature_id[2364].value <= threshold=0.41758519411087036
node_152: feature_name=cg07344990	feature_id[411].value > threshold=0.5484964847564697
node_158: feature_name=cg10298992	feature_id[3066].value > threshold=0.5707973837852478
node_174: feature_name=cg11843516	feature_id[20].value <= threshold=0.5590928494930267
node_175: feature_name=cg05185926	feature_id[448].value > threshold=0.17318664491176605
node_177: feature_name=cg17009731	feature_id[1220].value > threshold=0.6666513085365295
node_179: feature_name=cg01380319	feature_id[515].value <= threshold=0.39251960813999176
node_180: feature_name=cg20700977	feature_id[303].value > threshold=0.8422040641307831
node_184: feature_name=cg24259291	feature_id[164].value > threshold=0.4872268736362457
node_188: feature_name=cg08097657	feature_id[67].value > threshold=0.3371318429708481
node_190: feature_name=cg07891483	feature_id[59].value > threshold=0.6211732923984528
node_194: feature_name=cg20122645	feature_id[275].value > threshold=0.6094755828380585
node_196: feature_name=cg04026354	feature_id[1763].value <= threshold=0.4341808259487152
node_197: feature_name=cg10149889	feature_id[1017].value > threshold=0.6150195300579071
node_201: feature_name=cg14890730	feature_id[337].value <= threshold=0.4372602105140686
node_202: feature_name=cg08331427	feature_id[132].value > threshold=0.5443233847618103
node_208: feature_name=cg22730007	feature_id[2544].value > threshold=0.28619489073753357
node_210: feature_name=cg17193551	feature_id[2057].value > threshold=0.4393353909254074
node_212: feature_name=cg14671764	feature_id[584].value <= threshold=0.9353199899196625

node_213: feature_name=cg20140662	feature_id[2044].value > threshold=0.38030287623405457
node_215: feature_name=cg07891483	feature_id[59].value > threshold=0.829958975315094
node_219: feature_name=cg04884579	feature_id[1861].value > threshold=0.6106154918670654
node_229: feature_name=cg19548524	feature_id[2985].value > threshold=0.8623353838920593
node_233: feature_name=cg14788686	feature_id[3556].value > threshold=0.35090239346027374
node_235: feature_name=cg05380734	feature_id[2470].value <= threshold=0.7112160623073578
node_236: feature_name=cg01166827	feature_id[3708].value > threshold=0.6359498500823975
node_238: feature_name=cg13251842	feature_id[714].value <= threshold=0.8916542232036591
Class: chondrosarcoma (CSA)	
Rules_68	passed counts:4
node_0: feature_name=cg11915444	feature_id[1171].value > threshold=0.3402601182460785
node_10: feature_name=cg12109728	feature_id[2810].value > threshold=0.7068270146846771
node_20: feature_name=cg16412000	feature_id[789].value > threshold=0.6741803884506226
node_24: feature_name=cg17115147	feature_id[1434].value <= threshold=0.2961975038051605
node_25: feature_name=cg24474622	feature_id[2737].value > threshold=0.7211934924125671
node_29: feature_name=cg10472711	feature_id[612].value > threshold=0.45985929667949677
node_37: feature_name=cg01937669	feature_id[188].value <= threshold=0.4809463769197464
node_38: feature_name=cg02157052	feature_id[451].value <= threshold=0.2869153320789337
node_39: feature_name=cg18751958	feature_id[1427].value > threshold=0.4813787043094635
node_41: feature_name=cg22512847	feature_id[1152].value > threshold=0.5129324495792389
node_43: feature_name=cg23345038	feature_id[1147].value > threshold=0.6318148374557495
node_45: feature_name=cg17439009	feature_id[1815].value <= threshold=0.48514117300510406

node_46: feature_name=cg17537493	feature_id[2772].value > threshold=0.5825372636318207
node_52: feature_name=cg01683570	feature_id[110].value > threshold=0.6040188074111938
node_54: feature_name=cg15720017	feature_id[168].value > threshold=0.7067741453647614
node_60: feature_name=cg23690444	feature_id[120].value <= threshold=0.16037724912166595
node_61: feature_name=cg00567872	feature_id[236].value > threshold=0.7005765736103058
node_85: feature_name=cg01014262	feature_id[764].value <= threshold=0.5737917125225067
node_86: feature_name=cg22946562	feature_id[1148].value > threshold=0.6328203976154327
node_134: feature_name=cg14514032	feature_id[202].value <= threshold=0.4389604330062866
node_135: feature_name=cg13488220	feature_id[241].value > threshold=0.7352153658866882
node_143: feature_name=cg03840920	feature_id[256].value > threshold=0.502630889415741
node_147: feature_name=cg13634090	feature_id[822].value > threshold=0.7207068204879761
node_151: feature_name=cg19584674	feature_id[2364].value <= threshold=0.41758519411087036
node_152: feature_name=cg07344990	feature_id[411].value > threshold=0.5484964847564697
node_158: feature_name=cg10298992	feature_id[3066].value > threshold=0.5707973837852478
node_174: feature_name=cg11843516	feature_id[20].value <= threshold=0.5590928494930267
node_175: feature_name=cg05185926	feature_id[448].value > threshold=0.17318664491176605
node_177: feature_name=cg17009731	feature_id[1220].value > threshold=0.6666513085365295
node_179: feature_name=cg01380319	feature_id[515].value <= threshold=0.39251960813999176
node_180: feature_name=cg20700977	feature_id[303].value > threshold=0.8422040641307831
node_184: feature_name=cg24259291	feature_id[164].value > threshold=0.4872268736362457
node_188: feature_name=cg08097657	feature_id[67].value > threshold=0.3371318429708481
node_190: feature_name=cg07891483	feature_id[59].value > threshold=0.6211732923984528

node_194: feature_name=cg20122645	feature_id[275].value > threshold=0.6094755828380585
node_196: feature_name=cg04026354	feature_id[1763].value <= threshold=0.4341808259487152
node_197: feature_name=cg10149889	feature_id[1017].value > threshold=0.6150195300579071
node_201: feature_name=cg14890730	feature_id[337].value <= threshold=0.4372602105140686
node_202: feature_name=cg08331427	feature_id[132].value > threshold=0.5443233847618103
node_208: feature_name=cg22730007	feature_id[2544].value > threshold=0.28619489073753357
node_210: feature_name=cg17193551	feature_id[2057].value > threshold=0.4393353909254074
node_212: feature_name=cg14671764	feature_id[584].value <= threshold=0.9353199899196625
node_213: feature_name=cg20140662	feature_id[2044].value > threshold=0.38030287623405457
node_215: feature_name=cg07891483	feature_id[59].value > threshold=0.829958975315094
node_219: feature_name=cg04884579	feature_id[1861].value > threshold=0.6106154918670654
node_229: feature_name=cg19548524	feature_id[2985].value > threshold=0.8623353838920593
node_233: feature_name=cg14788686	feature_id[3556].value > threshold=0.35090239346027374
node_235: feature_name=cg05380734	feature_id[2470].value <= threshold=0.7112160623073578
node_236: feature_name=cg01166827	feature_id[3708].value <= threshold=0.6359498500823975
Class: chondroblastoma (CB)	
Rules_69	passed counts:4
node_0: feature_name=cg11915444	feature_id[1171].value > threshold=0.3402601182460785
node_10: feature_name=cg12109728	feature_id[2810].value > threshold=0.7068270146846771
node_20: feature_name=cg16412000	feature_id[789].value > threshold=0.6741803884506226
node_24: feature_name=cg17115147	feature_id[1434].value <= threshold=0.2961975038051605
node_25: feature_name=cg24474622	feature_id[2737].value > threshold=0.7211934924125671

node_29: feature_name=cg10472711	feature_id[612].value > threshold=0.4598592966794967
node_37: feature_name=cg01937669	feature_id[188].value <= threshold=0.4809463769197464
node_38: feature_name=cg02157052	feature_id[451].value <= threshold=0.2869153320789337
node_39: feature_name=cg18751958	feature_id[1427].value > threshold=0.4813787043094635
node_41: feature_name=cg22512847	feature_id[1152].value > threshold=0.5129324495792389
node_43: feature_name=cg23345038	feature_id[1147].value > threshold=0.6318148374557495
node_45: feature_name=cg17439009	feature_id[1815].value <= threshold=0.48514117300510406
node_46: feature_name=cg17537493	feature_id[2772].value > threshold=0.5825372636318207
node_52: feature_name=cg01683570	feature_id[110].value > threshold=0.6040188074111938
node_54: feature_name=cg15720017	feature_id[168].value > threshold=0.7067741453647614
node_60: feature_name=cg23690444	feature_id[120].value <= threshold=0.16037724912166595
node_61: feature_name=cg00567872	feature_id[236].value > threshold=0.7005765736103058
node_85: feature_name=cg01014262	feature_id[764].value <= threshold=0.5737917125225067
node_86: feature_name=cg22946562	feature_id[1148].value <= threshold=0.6328203976154327
node_87: feature_name=cg18120975	feature_id[2767].value > threshold=0.7081544995307922
node_91: feature_name=cg17171539	feature_id[25].value > threshold=0.501024603843689
node_111: feature_name=cg21241424	feature_id[1155].value <= threshold=0.7665272355079651
node_112: feature_name=cg21897315	feature_id[3800].value > threshold=0.48165470361709595
node_114: feature_name=cg04375492	feature_id[2874].value > threshold=0.7812598049640656
node_116: feature_name=cg09892984	feature_id[3615].value > threshold=0.6039115190505981
node_118: feature_name=cg05076082	feature_id[215].value > threshold=0.7612724006175995
node_120: feature_name=cg15776300	feature_id[3872].value > threshold=0.922246515750885

Class: undifferentiated sarcoma (USARC)	
Rules_70	passed counts:4
node_0: feature_name=cg11915444	feature_id[1171].value > threshold=0.3402601182460785
node_10: feature_name=cg12109728	feature_id[2810].value > threshold=0.7068270146846771
node_20: feature_name=cg16412000	feature_id[789].value > threshold=0.6741803884506226
node_24: feature_name=cg17115147	feature_id[1434].value <= threshold=0.2961975038051605
node_25: feature_name=cg24474622	feature_id[2737].value > threshold=0.7211934924125671
node_29: feature_name=cg10472711	feature_id[612].value > threshold=0.4598592967949677
node_37: feature_name=cg01937669	feature_id[188].value <= threshold=0.4809463769197464
node_38: feature_name=cg02157052	feature_id[451].value <= threshold=0.2869153320789337
node_39: feature_name=cg18751958	feature_id[1427].value > threshold=0.4813787043094635
node_41: feature_name=cg22512847	feature_id[1152].value > threshold=0.5129324495792389
node_43: feature_name=cg23345038	feature_id[1147].value > threshold=0.6318148374557495
node_45: feature_name=cg17439009	feature_id[1815].value <= threshold=0.48514117300510406
node_46: feature_name=cg17537493	feature_id[2772].value > threshold=0.5825372636318207
node_52: feature_name=cg01683570	feature_id[110].value > threshold=0.6040188074111938
node_54: feature_name=cg15720017	feature_id[168].value > threshold=0.7067741453647614
node_60: feature_name=cg23690444	feature_id[120].value <= threshold=0.16037724912166595
node_61: feature_name=cg00567872	feature_id[236].value <= threshold=0.7005765736103058
node_62: feature_name=cg09293488	feature_id[2629].value > threshold=0.839823454618454
node_68: feature_name=cg04838832	feature_id[1184].value > threshold=0.41919413208961487
Class: muscle tissue (MUS)	

Rules_71	passed counts:4
node_0: feature_name=cg11915444	feature_id[1171].value > threshold=0.3402601182460785
node_10: feature_name=cg12109728	feature_id[2810].value > threshold=0.7068270146846771
node_20: feature_name=cg16412000	feature_id[789].value > threshold=0.6741803884506226
node_24: feature_name=cg17115147	feature_id[1434].value <= threshold=0.2961975038051605
node_25: feature_name=cg24474622	feature_id[2737].value > threshold=0.7211934924125671
node_29: feature_name=cg10472711	feature_id[612].value > threshold=0.45985929667949677
node_37: feature_name=cg01937669	feature_id[188].value <= threshold=0.4809463769197464
node_38: feature_name=cg02157052	feature_id[451].value <= threshold=0.2869153320789337
node_39: feature_name=cg18751958	feature_id[1427].value > threshold=0.4813787043094635
node_41: feature_name=cg22512847	feature_id[1152].value > threshold=0.5129324495792389
node_43: feature_name=cg23345038	feature_id[1147].value > threshold=0.6318148374557495
node_45: feature_name=cg17439009	feature_id[1815].value <= threshold=0.48514117300510406
node_46: feature_name=cg17537493	feature_id[2772].value > threshold=0.5825372636318207
node_52: feature_name=cg01683570	feature_id[110].value > threshold=0.6040188074111938
node_54: feature_name=cg15720017	feature_id[168].value > threshold=0.7067741453647614
node_60: feature_name=cg23690444	feature_id[120].value <= threshold=0.16037724912166595
node_61: feature_name=cg00567872	feature_id[236].value <= threshold=0.7005765736103058
node_62: feature_name=cg09293488	feature_id[2629].value > threshold=0.839823454618454
node_68: feature_name=cg04838832	feature_id[1184].value <= threshold=0.41919413208961487
node_69: feature_name=cg18153137	feature_id[2198].value > threshold=0.08548648655414581
Class: infantile fibrosarcoma (IFS)	

Rules_72	passed counts:4
node_0: feature_name=cg11915444	feature_id[1171].value > threshold=0.3402601182460785
node_10: feature_name=cg12109728	feature_id[2810].value > threshold=0.7068270146846771
node_20: feature_name=cg16412000	feature_id[789].value > threshold=0.6741803884506226
node_24: feature_name=cg17115147	feature_id[1434].value <= threshold=0.2961975038051605
node_25: feature_name=cg24474622	feature_id[2737].value > threshold=0.7211934924125671
node_29: feature_name=cg10472711	feature_id[612].value > threshold=0.45985929667949677
node_37: feature_name=cg01937669	feature_id[188].value <= threshold=0.4809463769197464
node_38: feature_name=cg02157052	feature_id[451].value <= threshold=0.2869153320789337
node_39: feature_name=cg18751958	feature_id[1427].value > threshold=0.4813787043094635
node_41: feature_name=cg22512847	feature_id[1152].value > threshold=0.5129324495792389
node_43: feature_name=cg23345038	feature_id[1147].value > threshold=0.6318148374557495
node_45: feature_name=cg17439009	feature_id[1815].value <= threshold=0.48514117300510406
node_46: feature_name=cg17537493	feature_id[2772].value <= threshold=0.5825372636318207
node_47: feature_name=cg16572224	feature_id[2780].value <= threshold=0.6722612977027893
node_48: feature_name=cg00760872	feature_id[2909].value <= threshold=0.46596916019916534
Class: Kaposi sarcoma (KS)	
Rules_73	passed counts:3
node_0: feature_name=cg11915444	feature_id[1171].value > threshold=0.3402601182460785
node_10: feature_name=cg12109728	feature_id[2810].value > threshold=0.7068270146846771
node_20: feature_name=cg16412000	feature_id[789].value > threshold=0.6741803884506226
node_24: feature_name=cg17115147	feature_id[1434].value > threshold=0.2961975038051605

node_368: feature_name=cg27543578	feature_id[878].value > threshold=0.8884280920028687
node_372: feature_name=cg11400707	feature_id[1940].value > threshold=0.6575311124324799
Class: malignant peripheral nerve sheath tumour (MPNST)	
Rules_74	passed counts:3
node_0: feature_name=cg11915444	feature_id[1171].value > threshold=0.3402601182460785
node_10: feature_name=cg12109728	feature_id[2810].value > threshold=0.7068270146846771
node_20: feature_name=cg16412000	feature_id[789].value > threshold=0.6741803884506226
node_24: feature_name=cg17115147	feature_id[1434].value <= threshold=0.2961975038051605
node_25: feature_name=cg24474622	feature_id[2737].value > threshold=0.7211934924125671
node_29: feature_name=cg10472711	feature_id[612].value > threshold=0.45985929667949677
node_37: feature_name=cg01937669	feature_id[188].value <= threshold=0.4809463769197464
node_38: feature_name=cg02157052	feature_id[451].value > threshold=0.2869153320789337
node_344: feature_name=cg21189849	feature_id[189].value > threshold=0.7746135592460632
node_348: feature_name=cg06937205	feature_id[751].value > threshold=0.4331420361995697
node_350: feature_name=cg20165037	feature_id[2558].value > threshold=0.8168091177940369
node_352: feature_name=cg17454920	feature_id[3003].value > threshold=0.5224908739328384
node_354: feature_name=cg25878830	feature_id[1496].value > threshold=0.4498005360364914
node_356: feature_name=cg26070874	feature_id[1040].value > threshold=0.8498157262802124
Class: small blue round cell tumour (SBRCT)	
Rules_75	passed counts:3
node_0: feature_name=cg11915444	feature_id[1171].value > threshold=0.3402601182460785
node_10: feature_name=cg12109728	feature_id[2810].value > threshold=0.7068270146846771

node_20: feature_name=cg16412000	feature_id[789].value > threshold=0.6741803884506226
node_24: feature_name=cg17115147	feature_id[1434].value <= threshold=0.2961975038051605
node_25: feature_name=cg24474622	feature_id[2737].value > threshold=0.7211934924125671
node_29: feature_name=cg10472711	feature_id[612].value > threshold=0.45985929667949677
node_37: feature_name=cg01937669	feature_id[188].value <= threshold=0.4809463769197464
node_38: feature_name=cg02157052	feature_id[451].value > threshold=0.2869153320789337
node_344: feature_name=cg21189849	feature_id[189].value > threshold=0.7746135592460632
node_348: feature_name=cg06937205	feature_id[751].value > threshold=0.4331420361995697
node_350: feature_name=cg20165037	feature_id[2558].value > threshold=0.8168091177940369
node_352: feature_name=cg17454920	feature_id[3003].value > threshold=0.5224908739328384
node_354: feature_name=cg25878830	feature_id[1496].value > threshold=0.4498005360364914
node_356: feature_name=cg26070874	feature_id[1040].value <= threshold=0.8498157262802124
node_357: feature_name=cg19730379	feature_id[3228].value <= threshold=0.3024083226919174
Class: epithelioid sarcoma (ES)	
Rules_76	passed counts:3
node_0: feature_name=cg11915444	feature_id[1171].value > threshold=0.3402601182460785
node_10: feature_name=cg12109728	feature_id[2810].value > threshold=0.7068270146846771
node_20: feature_name=cg16412000	feature_id[789].value > threshold=0.6741803884506226
node_24: feature_name=cg17115147	feature_id[1434].value <= threshold=0.2961975038051605
node_25: feature_name=cg24474622	feature_id[2737].value > threshold=0.7211934924125671
node_29: feature_name=cg10472711	feature_id[612].value > threshold=0.45985929667949677
node_37: feature_name=cg01937669	feature_id[188].value <= threshold=0.4809463769197464

node_38: feature_name=cg02157052	feature_id[451].value > threshold=0.2869153320789337
node_344: feature_name=cg21189849	feature_id[189].value > threshold=0.7746135592460632
node_348: feature_name=cg06937205	feature_id[751].value > threshold=0.4331420361995697
node_350: feature_name=cg20165037	feature_id[2558].value > threshold=0.8168091177940369
node_352: feature_name=cg17454920	feature_id[3003].value > threshold=0.5224908739328384
node_354: feature_name=cg25878830	feature_id[1496].value <= threshold=0.4498005360364914
Class: solitary fibrous tumour (SFT)	
Rules_77	passed counts:3
node_0: feature_name=cg11915444	feature_id[1171].value > threshold=0.3402601182460785
node_10: feature_name=cg12109728	feature_id[2810].value > threshold=0.7068270146846771
node_20: feature_name=cg16412000	feature_id[789].value > threshold=0.6741803884506226
node_24: feature_name=cg17115147	feature_id[1434].value <= threshold=0.2961975038051605
node_25: feature_name=cg24474622	feature_id[2737].value > threshold=0.7211934924125671
node_29: feature_name=cg10472711	feature_id[612].value > threshold=0.45985929667949677
node_37: feature_name=cg01937669	feature_id[188].value <= threshold=0.4809463769197464
node_38: feature_name=cg02157052	feature_id[451].value <= threshold=0.2869153320789337
node_39: feature_name=cg18751958	feature_id[1427].value > threshold=0.4813787043094635
node_41: feature_name=cg22512847	feature_id[1152].value > threshold=0.5129324495792389
node_43: feature_name=cg23345038	feature_id[1147].value > threshold=0.6318148374557495
node_45: feature_name=cg17439009	feature_id[1815].value <= threshold=0.48514117300510406
node_46: feature_name=cg17537493	feature_id[2772].value > threshold=0.5825372636318207
node_52: feature_name=cg01683570	feature_id[110].value > threshold=0.6040188074111938

node_54: feature_name=cg15720017	feature_id[168].value > threshold=0.7067741453647614
node_60: feature_name=cg23690444	feature_id[120].value <= threshold=0.16037724912166595
node_61: feature_name=cg00567872	feature_id[236].value > threshold=0.7005765736103058
node_85: feature_name=cg01014262	feature_id[764].value <= threshold=0.5737917125225067
node_86: feature_name=cg22946562	feature_id[1148].value > threshold=0.6328203976154327
node_134: feature_name=cg14514032	feature_id[202].value <= threshold=0.4389604330062866
node_135: feature_name=cg13488220	feature_id[241].value > threshold=0.7352153658866882
node_143: feature_name=cg03840920	feature_id[256].value > threshold=0.502630889415741
node_147: feature_name=cg13634090	feature_id[822].value > threshold=0.7207068204879761
node_151: feature_name=cg19584674	feature_id[2364].value <= threshold=0.41758519411087036
node_152: feature_name=cg07344990	feature_id[411].value > threshold=0.5484964847564697
node_158: feature_name=cg10298992	feature_id[3066].value > threshold=0.5707973837852478
node_174: feature_name=cg11843516	feature_id[20].value <= threshold=0.5590928494930267
node_175: feature_name=cg05185926	feature_id[448].value > threshold=0.17318664491176605
node_177: feature_name=cg17009731	feature_id[1220].value > threshold=0.6666513085365295
node_179: feature_name=cg01380319	feature_id[515].value <= threshold=0.39251960813999176
node_180: feature_name=cg20700977	feature_id[303].value > threshold=0.8422040641307831
node_184: feature_name=cg24259291	feature_id[164].value > threshold=0.4872268736362457
node_188: feature_name=cg08097657	feature_id[67].value > threshold=0.3371318429708481
node_190: feature_name=cg07891483	feature_id[59].value > threshold=0.6211732923984528
node_194: feature_name=cg20122645	feature_id[275].value > threshold=0.6094755828380585
node_196: feature_name=cg04026354	feature_id[1763].value <= threshold=0.4341808259487152

node_197: feature_name=cg10149889	feature_id[1017].value > threshold=0.6150195300579071
node_201: feature_name=cg14890730	feature_id[337].value <= threshold=0.4372602105140686
node_202: feature_name=cg08331427	feature_id[132].value > threshold=0.5443233847618103
node_208: feature_name=cg22730007	feature_id[2544].value > threshold=0.28619489073753357
node_210: feature_name=cg17193551	feature_id[2057].value > threshold=0.4393353909254074
node_212: feature_name=cg14671764	feature_id[584].value > threshold=0.9353199899196625
node_268: feature_name=cg21377260	feature_id[534].value <= threshold=0.9840706288814545
node_269: feature_name=cg27143326	feature_id[1403].value > threshold=0.8949433267116547
Class: sclerosing epithelioid fibrosarcoma(SEF)	
Rules_78	passed counts:3
node_0: feature_name=cg11915444	feature_id[1171].value > threshold=0.3402601182460785
node_10: feature_name=cg12109728	feature_id[2810].value > threshold=0.7068270146846771
node_20: feature_name=cg16412000	feature_id[789].value > threshold=0.6741803884506226
node_24: feature_name=cg17115147	feature_id[1434].value <= threshold=0.2961975038051605
node_25: feature_name=cg24474622	feature_id[2737].value > threshold=0.7211934924125671
node_29: feature_name=cg10472711	feature_id[612].value > threshold=0.45985929667949677
node_37: feature_name=cg01937669	feature_id[188].value <= threshold=0.4809463769197464
node_38: feature_name=cg02157052	feature_id[451].value <= threshold=0.2869153320789337
node_39: feature_name=cg18751958	feature_id[1427].value > threshold=0.4813787043094635
node_41: feature_name=cg22512847	feature_id[1152].value > threshold=0.5129324495792389
node_43: feature_name=cg23345038	feature_id[1147].value > threshold=0.6318148374557495
node_45: feature_name=cg17439009	feature_id[1815].value <= threshold=0.48514117300510406

node_46: feature_name=cg17537493	feature_id[2772].value > threshold=0.5825372636318207
node_52: feature_name=cg01683570	feature_id[110].value > threshold=0.6040188074111938
node_54: feature_name=cg15720017	feature_id[168].value > threshold=0.7067741453647614
node_60: feature_name=cg23690444	feature_id[120].value <= threshold=0.16037724912166595
node_61: feature_name=cg00567872	feature_id[236].value > threshold=0.7005765736103058
node_85: feature_name=cg01014262	feature_id[764].value <= threshold=0.5737917125225067
node_86: feature_name=cg22946562	feature_id[1148].value > threshold=0.6328203976154327
node_134: feature_name=cg14514032	feature_id[202].value <= threshold=0.4389604330062866
node_135: feature_name=cg13488220	feature_id[241].value > threshold=0.7352153658866882
node_143: feature_name=cg03840920	feature_id[256].value > threshold=0.502630889415741
node_147: feature_name=cg13634090	feature_id[822].value > threshold=0.7207068204879761
node_151: feature_name=cg19584674	feature_id[2364].value <= threshold=0.41758519411087036
node_152: feature_name=cg07344990	feature_id[411].value > threshold=0.5484964847564697
node_158: feature_name=cg10298992	feature_id[3066].value > threshold=0.5707973837852478
node_174: feature_name=cg11843516	feature_id[20].value <= threshold=0.5590928494930267
node_175: feature_name=cg05185926	feature_id[448].value > threshold=0.17318664491176605
node_177: feature_name=cg17009731	feature_id[1220].value > threshold=0.6666513085365295
node_179: feature_name=cg01380319	feature_id[515].value <= threshold=0.39251960813999176
node_180: feature_name=cg20700977	feature_id[303].value > threshold=0.8422040641307831
node_184: feature_name=cg24259291	feature_id[164].value > threshold=0.4872268736362457
node_188: feature_name=cg08097657	feature_id[67].value > threshold=0.3371318429708481
node_190: feature_name=cg07891483	feature_id[59].value > threshold=0.6211732923984528

node_194: feature_name=cg20122645	feature_id[275].value > threshold=0.6094755828380585
node_196: feature_name=cg04026354	feature_id[1763].value <= threshold=0.4341808259487152
node_197: feature_name=cg10149889	feature_id[1017].value > threshold=0.6150195300579071
node_201: feature_name=cg14890730	feature_id[337].value <= threshold=0.4372602105140686
node_202: feature_name=cg08331427	feature_id[132].value > threshold=0.5443233847618103
node_208: feature_name=cg22730007	feature_id[2544].value > threshold=0.28619489073753357
node_210: feature_name=cg17193551	feature_id[2057].value > threshold=0.4393353909254074
node_212: feature_name=cg14671764	feature_id[584].value <= threshold=0.9353199899196625
node_213: feature_name=cg20140662	feature_id[2044].value > threshold=0.38030287623405457
node_215: feature_name=cg07891483	feature_id[59].value > threshold=0.829958975315094
node_219: feature_name=cg04884579	feature_id[1861].value > threshold=0.6106154918670654
node_229: feature_name=cg19548524	feature_id[2985].value > threshold=0.8623353838920593
node_233: feature_name=cg14788686	feature_id[3556].value > threshold=0.35090239346027374
node_235: feature_name=cg05380734	feature_id[2470].value <= threshold=0.7112160623073578
node_236: feature_name=cg01166827	feature_id[3708].value > threshold=0.6359498500823975
node_238: feature_name=cg13251842	feature_id[714].value > threshold=0.8916542232036591
node_240: feature_name=cg02829279	feature_id[623].value > threshold=0.450528159737587
node_242: feature_name=cg24358599	feature_id[86].value > threshold=0.8132582604885101
node_246: feature_name=cg05544807	feature_id[3661].value > threshold=0.6726464331150055
Class: atypical fibroxanthoma (AFX)/pleomorphic dermal sarcoma (PDS)	
Rules_79	passed counts:3

node_0: feature_name=cg11915444	feature_id[1171].value > threshold=0.3402601182460785
node_10: feature_name=cg12109728	feature_id[2810].value > threshold=0.7068270146846771
node_20: feature_name=cg16412000	feature_id[789].value > threshold=0.6741803884506226
node_24: feature_name=cg17115147	feature_id[1434].value <= threshold=0.2961975038051605
node_25: feature_name=cg24474622	feature_id[2737].value > threshold=0.7211934924125671
node_29: feature_name=cg10472711	feature_id[612].value > threshold=0.45985929667949677
node_37: feature_name=cg01937669	feature_id[188].value <= threshold=0.4809463769197464
node_38: feature_name=cg02157052	feature_id[451].value <= threshold=0.2869153320789337
node_39: feature_name=cg18751958	feature_id[1427].value > threshold=0.4813787043094635
node_41: feature_name=cg22512847	feature_id[1152].value > threshold=0.5129324495792389
node_43: feature_name=cg23345038	feature_id[1147].value > threshold=0.6318148374557495
node_45: feature_name=cg17439009	feature_id[1815].value <= threshold=0.48514117300510406
node_46: feature_name=cg17537493	feature_id[2772].value > threshold=0.5825372636318207
node_52: feature_name=cg01683570	feature_id[110].value > threshold=0.6040188074111938
node_54: feature_name=cg15720017	feature_id[168].value > threshold=0.7067741453647614
node_60: feature_name=cg23690444	feature_id[120].value <= threshold=0.16037724912166595
node_61: feature_name=cg00567872	feature_id[236].value > threshold=0.7005765736103058
node_85: feature_name=cg01014262	feature_id[764].value <= threshold=0.5737917125225067
node_86: feature_name=cg22946562	feature_id[1148].value > threshold=0.6328203976154327
node_134: feature_name=cg14514032	feature_id[202].value <= threshold=0.4389604330062866
node_135: feature_name=cg13488220	feature_id[241].value > threshold=0.7352153658866882
node_143: feature_name=cg03840920	feature_id[256].value > threshold=0.502630889415741

node_147: feature_name=cg13634090	feature_id[822].value > threshold=0.7207068204879761
node_151: feature_name=cg19584674	feature_id[2364].value <= threshold=0.41758519411087036
node_152: feature_name=cg07344990	feature_id[411].value > threshold=0.5484964847564697
node_158: feature_name=cg10298992	feature_id[3066].value > threshold=0.5707973837852478
node_174: feature_name=cg11843516	feature_id[20].value <= threshold=0.5590928494930267
node_175: feature_name=cg05185926	feature_id[448].value > threshold=0.17318664491176605
node_177: feature_name=cg17009731	feature_id[1220].value > threshold=0.6666513085365295
node_179: feature_name=cg01380319	feature_id[515].value <= threshold=0.39251960813999176
node_180: feature_name=cg20700977	feature_id[303].value > threshold=0.8422040641307831
node_184: feature_name=cg24259291	feature_id[164].value > threshold=0.4872268736362457
node_188: feature_name=cg08097657	feature_id[67].value > threshold=0.3371318429708481
node_190: feature_name=cg07891483	feature_id[59].value > threshold=0.6211732923984528
node_194: feature_name=cg20122645	feature_id[275].value > threshold=0.6094755828380585
node_196: feature_name=cg04026354	feature_id[1763].value <= threshold=0.4341808259487152
node_197: feature_name=cg10149889	feature_id[1017].value > threshold=0.6150195300579071
node_201: feature_name=cg14890730	feature_id[337].value <= threshold=0.4372602105140686
node_202: feature_name=cg08331427	feature_id[132].value > threshold=0.5443233847618103
node_208: feature_name=cg22730007	feature_id[2544].value > threshold=0.28619489073753357
node_210: feature_name=cg17193551	feature_id[2057].value > threshold=0.4393353909254074
node_212: feature_name=cg14671764	feature_id[584].value <= threshold=0.9353199899196625
node_213: feature_name=cg20140662	feature_id[2044].value > threshold=0.38030287623405457
node_215: feature_name=cg07891483	feature_id[59].value > threshold=0.829958975315094

node_219: feature_name=cg04884579	feature_id[1861].value > threshold=0.6106154918670654
node_229: feature_name=cg19548524	feature_id[2985].value > threshold=0.8623353838920593
node_233: feature_name=cg14788686	feature_id[3556].value > threshold=0.35090239346027374
node_235: feature_name=cg05380734	feature_id[2470].value <= threshold=0.7112160623073578
node_236: feature_name=cg01166827	feature_id[3708].value > threshold=0.6359498500823975
node_238: feature_name=cg13251842	feature_id[714].value > threshold=0.8916542232036591
node_240: feature_name=cg02829279	feature_id[623].value <= threshold=0.450528159737587
Class: Ewing sarcoma (EWING)	
Rules_80	passed counts:3
node_0: feature_name=cg11915444	feature_id[1171].value > threshold=0.3402601182460785
node_10: feature_name=cg12109728	feature_id[2810].value > threshold=0.7068270146846771
node_20: feature_name=cg16412000	feature_id[789].value > threshold=0.6741803884506226
node_24: feature_name=cg17115147	feature_id[1434].value <= threshold=0.2961975038051605
node_25: feature_name=cg24474622	feature_id[2737].value > threshold=0.7211934924125671
node_29: feature_name=cg10472711	feature_id[612].value > threshold=0.45985929667949677
node_37: feature_name=cg01937669	feature_id[188].value <= threshold=0.4809463769197464
node_38: feature_name=cg02157052	feature_id[451].value <= threshold=0.2869153320789337
node_39: feature_name=cg18751958	feature_id[1427].value > threshold=0.4813787043094635
node_41: feature_name=cg22512847	feature_id[1152].value > threshold=0.5129324495792389
node_43: feature_name=cg23345038	feature_id[1147].value > threshold=0.6318148374557495
node_45: feature_name=cg17439009	feature_id[1815].value <= threshold=0.48514117300510406
node_46: feature_name=cg17537493	feature_id[2772].value > threshold=0.5825372636318207

node_52: feature_name=cg01683570	feature_id[110].value > threshold=0.6040188074111938
node_54: feature_name=cg15720017	feature_id[168].value > threshold=0.7067741453647614
node_60: feature_name=cg23690444	feature_id[120].value <= threshold=0.16037724912166595
node_61: feature_name=cg00567872	feature_id[236].value > threshold=0.7005765736103058
node_85: feature_name=cg01014262	feature_id[764].value <= threshold=0.5737917125225067
node_86: feature_name=cg22946562	feature_id[1148].value > threshold=0.6328203976154327
node_134: feature_name=cg14514032	feature_id[202].value <= threshold=0.4389604330062866
node_135: feature_name=cg13488220	feature_id[241].value > threshold=0.7352153658866882
node_143: feature_name=cg03840920	feature_id[256].value > threshold=0.502630889415741
node_147: feature_name=cg13634090	feature_id[822].value > threshold=0.7207068204879761
node_151: feature_name=cg19584674	feature_id[2364].value <= threshold=0.41758519411087036
node_152: feature_name=cg07344990	feature_id[411].value > threshold=0.5484964847564697
node_158: feature_name=cg10298992	feature_id[3066].value <= threshold=0.5707973837852478
node_159: feature_name=cg24852135	feature_id[47].value <= threshold=0.8445721566677094
node_160: feature_name=cg25606046	feature_id[2007].value > threshold=0.8722946643829346
Class: embryonal rhabdomyosarcoma (RMS (EMB))	
Rules_81	passed counts:2
node_0: feature_name=cg11915444	feature_id[1171].value > threshold=0.3402601182460785
node_10: feature_name=cg12109728	feature_id[2810].value > threshold=0.7068270146846771
node_20: feature_name=cg16412000	feature_id[789].value > threshold=0.6741803884506226
node_24: feature_name=cg17115147	feature_id[1434].value > threshold=0.2961975038051605

node_368: feature_name=cg27543578	feature_id[878].value > threshold=0.8884280920028687
node_372: feature_name=cg11400707	feature_id[1940].value <= threshold=0.6575311124324799
node_373: feature_name=cg03042971	feature_id[1579].value <= threshold=0.6336731016635895
Class: giant cell tumour of bone (GCTB)	
Rules_82	passed counts:2
node_0: feature_name=cg11915444	feature_id[1171].value > threshold=0.3402601182460785
node_10: feature_name=cg12109728	feature_id[2810].value > threshold=0.7068270146846771
node_20: feature_name=cg16412000	feature_id[789].value > threshold=0.6741803884506226
node_24: feature_name=cg17115147	feature_id[1434].value <= threshold=0.2961975038051605
node_25: feature_name=cg24474622	feature_id[2737].value > threshold=0.7211934924125671
node_29: feature_name=cg10472711	feature_id[612].value > threshold=0.45985929667949677
node_37: feature_name=cg01937669	feature_id[188].value > threshold=0.4809463769197464
node_363: feature_name=cg14588828	feature_id[2222].value <= threshold=0.9071650803089142
node_364: feature_name=cg24881558	feature_id[3465].value <= threshold=0.7283148765563965
Class: ossifying fibromyxoid tumour (OFMT)	
Rules_83	passed counts:2
node_0: feature_name=cg11915444	feature_id[1171].value > threshold=0.3402601182460785
node_10: feature_name=cg12109728	feature_id[2810].value > threshold=0.7068270146846771
node_20: feature_name=cg16412000	feature_id[789].value > threshold=0.6741803884506226
node_24: feature_name=cg17115147	feature_id[1434].value <= threshold=0.2961975038051605
node_25: feature_name=cg24474622	feature_id[2737].value > threshold=0.7211934924125671
node_29: feature_name=cg10472711	feature_id[612].value > threshold=0.45985929667949677

node_37: feature_name=cg01937669	feature_id[188].value <= threshold=0.4809463769197464
node_38: feature_name=cg02157052	feature_id[451].value <= threshold=0.2869153320789337
node_39: feature_name=cg18751958	feature_id[1427].value > threshold=0.4813787043094635
node_41: feature_name=cg22512847	feature_id[1152].value > threshold=0.5129324495792389
node_43: feature_name=cg23345038	feature_id[1147].value > threshold=0.6318148374557495
node_45: feature_name=cg17439009	feature_id[1815].value <= threshold=0.48514117300510406
node_46: feature_name=cg17537493	feature_id[2772].value > threshold=0.5825372636318207
node_52: feature_name=cg01683570	feature_id[110].value > threshold=0.6040188074111938
node_54: feature_name=cg15720017	feature_id[168].value > threshold=0.7067741453647614
node_60: feature_name=cg23690444	feature_id[120].value <= threshold=0.16037724912166595
node_61: feature_name=cg00567872	feature_id[236].value > threshold=0.7005765736103058
node_85: feature_name=cg01014262	feature_id[764].value > threshold=0.5737917125225067
node_331: feature_name=cg16897462	feature_id[3257].value <= threshold=0.24157879501581192
node_332: feature_name=cg13800349	feature_id[3278].value > threshold=0.5597856789827347
Class: Langerhans cell histiocytosis (LCH)	
Rules_84	passed counts:2
node_0: feature_name=cg11915444	feature_id[1171].value > threshold=0.3402601182460785
node_10: feature_name=cg12109728	feature_id[2810].value > threshold=0.7068270146846771
node_20: feature_name=cg16412000	feature_id[789].value > threshold=0.6741803884506226
node_24: feature_name=cg17115147	feature_id[1434].value <= threshold=0.2961975038051605
node_25: feature_name=cg24474622	feature_id[2737].value > threshold=0.7211934924125671
node_29: feature_name=cg10472711	feature_id[612].value > threshold=0.45985929667949677

node_37: feature_name=cg01937669	feature_id[188].value <= threshold=0.4809463769197464
node_38: feature_name=cg02157052	feature_id[451].value <= threshold=0.2869153320789337
node_39: feature_name=cg18751958	feature_id[1427].value > threshold=0.4813787043094635
node_41: feature_name=cg22512847	feature_id[1152].value > threshold=0.5129324495792389
node_43: feature_name=cg23345038	feature_id[1147].value > threshold=0.6318148374557495
node_45: feature_name=cg17439009	feature_id[1815].value <= threshold=0.48514117300510406
node_46: feature_name=cg17537493	feature_id[2772].value > threshold=0.5825372636318207
node_52: feature_name=cg01683570	feature_id[110].value > threshold=0.6040188074111938
node_54: feature_name=cg15720017	feature_id[168].value > threshold=0.7067741453647614
node_60: feature_name=cg23690444	feature_id[120].value <= threshold=0.16037724912166595
node_61: feature_name=cg00567872	feature_id[236].value > threshold=0.7005765736103058
node_85: feature_name=cg01014262	feature_id[764].value > threshold=0.5737917125225067
node_331: feature_name=cg16897462	feature_id[3257].value <= threshold=0.24157879501581192
node_332: feature_name=cg13800349	feature_id[3278].value <= threshold=0.5597856789827347
Class: synovial sarcoma (SYSA)	
Rules_85	passed counts:2
node_0: feature_name=cg11915444	feature_id[1171].value > threshold=0.3402601182460785
node_10: feature_name=cg12109728	feature_id[2810].value > threshold=0.7068270146846771
node_20: feature_name=cg16412000	feature_id[789].value > threshold=0.6741803884506226
node_24: feature_name=cg17115147	feature_id[1434].value <= threshold=0.2961975038051605
node_25: feature_name=cg24474622	feature_id[2737].value > threshold=0.7211934924125671
node_29: feature_name=cg10472711	feature_id[612].value > threshold=0.45985929667949677

node_37: feature_name=cg01937669	feature_id[188].value <= threshold=0.4809463769197464
node_38: feature_name=cg02157052	feature_id[451].value <= threshold=0.2869153320789337
node_39: feature_name=cg18751958	feature_id[1427].value > threshold=0.4813787043094635
node_41: feature_name=cg22512847	feature_id[1152].value > threshold=0.5129324495792389
node_43: feature_name=cg23345038	feature_id[1147].value > threshold=0.6318148374557495
node_45: feature_name=cg17439009	feature_id[1815].value <= threshold=0.48514117300510406
node_46: feature_name=cg17537493	feature_id[2772].value > threshold=0.5825372636318207
node_52: feature_name=cg01683570	feature_id[110].value > threshold=0.6040188074111938
node_54: feature_name=cg15720017	feature_id[168].value > threshold=0.7067741453647614
node_60: feature_name=cg23690444	feature_id[120].value <= threshold=0.16037724912166595
node_61: feature_name=cg00567872	feature_id[236].value > threshold=0.7005765736103058
node_85: feature_name=cg01014262	feature_id[764].value <= threshold=0.5737917125225067
node_86: feature_name=cg22946562	feature_id[1148].value > threshold=0.6328203976154327
node_134: feature_name=cg14514032	feature_id[202].value > threshold=0.4389604330062866
node_312: feature_name=cg21670987	feature_id[1509].value <= threshold=0.32477423548698425
node_313: feature_name=cg12939390	feature_id[143].value > threshold=0.23070813715457916
node_315: feature_name=cg10907727	feature_id[284].value > threshold=0.6596133708953857
node_317: feature_name=cg21241424	feature_id[1155].value <= threshold=0.7687841951847076
node_318: feature_name=cg19691659	feature_id[953].value <= threshold=0.6708230972290039
node_319: feature_name=cg04002063	feature_id[2129].value <= threshold=0.8756260275840759
Class: well differentiated liposarcoma (WDLS)/dedifferentiated liposarcoma (DDLS)	

Rules_86	passed counts:2
node_0: feature_name=cg11915444	feature_id[1171].value > threshold=0.3402601182460785
node_10: feature_name=cg12109728	feature_id[2810].value > threshold=0.7068270146846771
node_20: feature_name=cg16412000	feature_id[789].value > threshold=0.6741803884506226
node_24: feature_name=cg17115147	feature_id[1434].value <= threshold=0.2961975038051605
node_25: feature_name=cg24474622	feature_id[2737].value > threshold=0.7211934924125671
node_29: feature_name=cg10472711	feature_id[612].value > threshold=0.45985929667949677
node_37: feature_name=cg01937669	feature_id[188].value <= threshold=0.4809463769197464
node_38: feature_name=cg02157052	feature_id[451].value <= threshold=0.2869153320789337
node_39: feature_name=cg18751958	feature_id[1427].value > threshold=0.4813787043094635
node_41: feature_name=cg22512847	feature_id[1152].value > threshold=0.5129324495792389
node_43: feature_name=cg23345038	feature_id[1147].value > threshold=0.6318148374557495
node_45: feature_name=cg17439009	feature_id[1815].value <= threshold=0.48514117300510406
node_46: feature_name=cg17537493	feature_id[2772].value > threshold=0.5825372636318207
node_52: feature_name=cg01683570	feature_id[110].value > threshold=0.6040188074111938
node_54: feature_name=cg15720017	feature_id[168].value > threshold=0.7067741453647614
node_60: feature_name=cg23690444	feature_id[120].value <= threshold=0.16037724912166595
node_61: feature_name=cg00567872	feature_id[236].value > threshold=0.7005765736103058
node_85: feature_name=cg01014262	feature_id[764].value <= threshold=0.5737917125225067
node_86: feature_name=cg22946562	feature_id[1148].value > threshold=0.6328203976154327
node_134: feature_name=cg14514032	feature_id[202].value <= threshold=0.4389604330062866
node_135: feature_name=cg13488220	feature_id[241].value > threshold=0.7352153658866882

node_143: feature_name=cg03840920	feature_id[256].value > threshold=0.502630889415741
node_147: feature_name=cg13634090	feature_id[822].value > threshold=0.7207068204879761
node_151: feature_name=cg19584674	feature_id[2364].value > threshold=0.41758519411087036
node_307: feature_name=cg07795968	feature_id[2451].value > threshold=0.7859841883182526
node_309: feature_name=cg12552626	feature_id[687].value > threshold=0.893171489238739
Class: sclerosing epithelioid fibrosarcoma(SEF)	
Rules_87	passed counts:2
node_0: feature_name=cg11915444	feature_id[1171].value > threshold=0.3402601182460785
node_10: feature_name=cg12109728	feature_id[2810].value > threshold=0.7068270146846771
node_20: feature_name=cg16412000	feature_id[789].value > threshold=0.6741803884506226
node_24: feature_name=cg17115147	feature_id[1434].value <= threshold=0.2961975038051605
node_25: feature_name=cg24474622	feature_id[2737].value > threshold=0.7211934924125671
node_29: feature_name=cg10472711	feature_id[612].value > threshold=0.45985929667949677
node_37: feature_name=cg01937669	feature_id[188].value <= threshold=0.4809463769197464
node_38: feature_name=cg02157052	feature_id[451].value <= threshold=0.2869153320789337
node_39: feature_name=cg18751958	feature_id[1427].value > threshold=0.4813787043094635
node_41: feature_name=cg22512847	feature_id[1152].value > threshold=0.5129324495792389
node_43: feature_name=cg23345038	feature_id[1147].value > threshold=0.6318148374557495
node_45: feature_name=cg17439009	feature_id[1815].value <= threshold=0.48514117300510406
node_46: feature_name=cg17537493	feature_id[2772].value > threshold=0.5825372636318207
node_52: feature_name=cg01683570	feature_id[110].value > threshold=0.6040188074111938
node_54: feature_name=cg15720017	feature_id[168].value > threshold=0.7067741453647614

node_60: feature_name=cg23690444	feature_id[120].value <= threshold=0.16037724912166595
node_61: feature_name=cg00567872	feature_id[236].value > threshold=0.7005765736103058
node_85: feature_name=cg01014262	feature_id[764].value <= threshold=0.5737917125225067
node_86: feature_name=cg22946562	feature_id[1148].value > threshold=0.6328203976154327
node_134: feature_name=cg14514032	feature_id[202].value <= threshold=0.4389604330062866
node_135: feature_name=cg13488220	feature_id[241].value > threshold=0.7352153658866882
node_143: feature_name=cg03840920	feature_id[256].value > threshold=0.502630889415741
node_147: feature_name=cg13634090	feature_id[822].value > threshold=0.7207068204879761
node_151: feature_name=cg19584674	feature_id[2364].value <= threshold=0.41758519411087036
node_152: feature_name=cg07344990	feature_id[411].value > threshold=0.5484964847564697
node_158: feature_name=cg10298992	feature_id[3066].value > threshold=0.5707973837852478
node_174: feature_name=cg11843516	feature_id[20].value <= threshold=0.5590928494930267
node_175: feature_name=cg05185926	feature_id[448].value > threshold=0.17318664491176605
node_177: feature_name=cg17009731	feature_id[1220].value > threshold=0.6666513085365295
node_179: feature_name=cg01380319	feature_id[515].value <= threshold=0.39251960813999176
node_180: feature_name=cg20700977	feature_id[303].value > threshold=0.8422040641307831
node_184: feature_name=cg24259291	feature_id[164].value > threshold=0.4872268736362457
node_188: feature_name=cg08097657	feature_id[67].value > threshold=0.3371318429708481
node_190: feature_name=cg07891483	feature_id[59].value > threshold=0.6211732923984528
node_194: feature_name=cg20122645	feature_id[275].value > threshold=0.6094755828380585
node_196: feature_name=cg04026354	feature_id[1763].value > threshold=0.4341808259487152
node_286: feature_name=cg25408950	feature_id[772].value > threshold=0.680083692073822

node_290: feature_name=cg15188939	feature_id[1626].value > threshold=0.6365727782249451
node_294: feature_name=cg00444740	feature_id[3167].value > threshold=0.5775478780269623
node_296: feature_name=cg03555203	feature_id[3682].value > threshold=0.8000460863113403
node_298: feature_name=cg26685539	feature_id[1788].value > threshold=0.8831613063812256
node_300: feature_name=cg02353448	feature_id[74].value <= threshold=0.9122499525547028
Class: melanoma (MEL)	
Rules_88	passed counts:2
node_0: feature_name=cg11915444	feature_id[1171].value > threshold=0.3402601182460785
node_10: feature_name=cg12109728	feature_id[2810].value > threshold=0.7068270146846771
node_20: feature_name=cg16412000	feature_id[789].value > threshold=0.6741803884506226
node_24: feature_name=cg17115147	feature_id[1434].value <= threshold=0.2961975038051605
node_25: feature_name=cg24474622	feature_id[2737].value > threshold=0.7211934924125671
node_29: feature_name=cg10472711	feature_id[612].value > threshold=0.45985929667949677
node_37: feature_name=cg01937669	feature_id[188].value <= threshold=0.4809463769197464
node_38: feature_name=cg02157052	feature_id[451].value <= threshold=0.2869153320789337
node_39: feature_name=cg18751958	feature_id[1427].value > threshold=0.4813787043094635
node_41: feature_name=cg22512847	feature_id[1152].value > threshold=0.5129324495792389
node_43: feature_name=cg23345038	feature_id[1147].value > threshold=0.6318148374557495
node_45: feature_name=cg17439009	feature_id[1815].value <= threshold=0.48514117300510406
node_46: feature_name=cg17537493	feature_id[2772].value > threshold=0.5825372636318207
node_52: feature_name=cg01683570	feature_id[110].value > threshold=0.6040188074111938
node_54: feature_name=cg15720017	feature_id[168].value > threshold=0.7067741453647614

node_60: feature_name=cg23690444	feature_id[120].value <= threshold=0.16037724912166595
node_61: feature_name=cg00567872	feature_id[236].value > threshold=0.7005765736103058
node_85: feature_name=cg01014262	feature_id[764].value <= threshold=0.5737917125225067
node_86: feature_name=cg22946562	feature_id[1148].value > threshold=0.6328203976154327
node_134: feature_name=cg14514032	feature_id[202].value <= threshold=0.4389604330062866
node_135: feature_name=cg13488220	feature_id[241].value > threshold=0.7352153658866882
node_143: feature_name=cg03840920	feature_id[256].value > threshold=0.502630889415741
node_147: feature_name=cg13634090	feature_id[822].value > threshold=0.7207068204879761
node_151: feature_name=cg19584674	feature_id[2364].value <= threshold=0.41758519411087036
node_152: feature_name=cg07344990	feature_id[411].value > threshold=0.5484964847564697
node_158: feature_name=cg10298992	feature_id[3066].value > threshold=0.5707973837852478
node_174: feature_name=cg11843516	feature_id[20].value <= threshold=0.5590928494930267
node_175: feature_name=cg05185926	feature_id[448].value > threshold=0.17318664491176605
node_177: feature_name=cg17009731	feature_id[1220].value > threshold=0.6666513085365295
node_179: feature_name=cg01380319	feature_id[515].value <= threshold=0.39251960813999176
node_180: feature_name=cg20700977	feature_id[303].value > threshold=0.8422040641307831
node_184: feature_name=cg24259291	feature_id[164].value > threshold=0.4872268736362457
node_188: feature_name=cg08097657	feature_id[67].value > threshold=0.3371318429708481
node_190: feature_name=cg07891483	feature_id[59].value > threshold=0.6211732923984528
node_194: feature_name=cg20122645	feature_id[275].value > threshold=0.6094755828380585
node_196: feature_name=cg04026354	feature_id[1763].value > threshold=0.4341808259487152
node_286: feature_name=cg25408950	feature_id[772].value > threshold=0.680083692073822

node_290: feature_name=cg15188939	feature_id[1626].value > threshold=0.6365727782249451
node_294: feature_name=cg00444740	feature_id[3167].value > threshold=0.5775478780269623
node_296: feature_name=cg03555203	feature_id[3682].value > threshold=0.8000460863113403
node_298: feature_name=cg26685539	feature_id[1788].value <= threshold=0.8831613063812256
Class: well differentiated liposarcoma (WDLS)/dedifferentiated liposarcoma (DDLS)	
Rules_89	passed counts:2
node_0: feature_name=cg11915444	feature_id[1171].value > threshold=0.3402601182460785
node_10: feature_name=cg12109728	feature_id[2810].value > threshold=0.7068270146846771
node_20: feature_name=cg16412000	feature_id[789].value > threshold=0.6741803884506226
node_24: feature_name=cg17115147	feature_id[1434].value <= threshold=0.2961975038051605
node_25: feature_name=cg24474622	feature_id[2737].value > threshold=0.7211934924125671
node_29: feature_name=cg10472711	feature_id[612].value > threshold=0.45985929667949677
node_37: feature_name=cg01937669	feature_id[188].value <= threshold=0.4809463769197464
node_38: feature_name=cg02157052	feature_id[451].value <= threshold=0.2869153320789337
node_39: feature_name=cg18751958	feature_id[1427].value > threshold=0.4813787043094635
node_41: feature_name=cg22512847	feature_id[1152].value > threshold=0.5129324495792389
node_43: feature_name=cg23345038	feature_id[1147].value > threshold=0.6318148374557495
node_45: feature_name=cg17439009	feature_id[1815].value <= threshold=0.48514117300510406
node_46: feature_name=cg17537493	feature_id[2772].value > threshold=0.5825372636318207
node_52: feature_name=cg01683570	feature_id[110].value > threshold=0.6040188074111938
node_54: feature_name=cg15720017	feature_id[168].value > threshold=0.7067741453647614

node_60: feature_name=cg23690444	feature_id[120].value <= threshold=0.16037724912166595
node_61: feature_name=cg00567872	feature_id[236].value > threshold=0.7005765736103058
node_85: feature_name=cg01014262	feature_id[764].value <= threshold=0.5737917125225067
node_86: feature_name=cg22946562	feature_id[1148].value > threshold=0.6328203976154327
node_134: feature_name=cg14514032	feature_id[202].value <= threshold=0.4389604330062866
node_135: feature_name=cg13488220	feature_id[241].value > threshold=0.7352153658866882
node_143: feature_name=cg03840920	feature_id[256].value > threshold=0.502630889415741
node_147: feature_name=cg13634090	feature_id[822].value > threshold=0.7207068204879761
node_151: feature_name=cg19584674	feature_id[2364].value <= threshold=0.41758519411087036
node_152: feature_name=cg07344990	feature_id[411].value > threshold=0.5484964847564697
node_158: feature_name=cg10298992	feature_id[3066].value > threshold=0.5707973837852478
node_174: feature_name=cg11843516	feature_id[20].value <= threshold=0.5590928494930267
node_175: feature_name=cg05185926	feature_id[448].value > threshold=0.17318664491176605
node_177: feature_name=cg17009731	feature_id[1220].value > threshold=0.6666513085365295
node_179: feature_name=cg01380319	feature_id[515].value <= threshold=0.39251960813999176
node_180: feature_name=cg20700977	feature_id[303].value > threshold=0.8422040641307831
node_184: feature_name=cg24259291	feature_id[164].value > threshold=0.4872268736362457
node_188: feature_name=cg08097657	feature_id[67].value > threshold=0.3371318429708481
node_190: feature_name=cg07891483	feature_id[59].value > threshold=0.6211732923984528
node_194: feature_name=cg20122645	feature_id[275].value > threshold=0.6094755828380585
node_196: feature_name=cg04026354	feature_id[1763].value > threshold=0.4341808259487152
node_286: feature_name=cg25408950	feature_id[772].value > threshold=0.680083692073822

node_290: feature_name=cg15188939	feature_id[1626].value > threshold=0.6365727782249451
node_294: feature_name=cg00444740	feature_id[3167].value > threshold=0.5775478780269623
node_296: feature_name=cg03555203	feature_id[3682].value <= threshold=0.8000460863113403
Class: Kaposi sarcoma (KS)	
Rules_90	passed counts:2
node_0: feature_name=cg11915444	feature_id[1171].value > threshold=0.3402601182460785
node_10: feature_name=cg12109728	feature_id[2810].value > threshold=0.7068270146846771
node_20: feature_name=cg16412000	feature_id[789].value > threshold=0.6741803884506226
node_24: feature_name=cg17115147	feature_id[1434].value <= threshold=0.2961975038051605
node_25: feature_name=cg24474622	feature_id[2737].value > threshold=0.7211934924125671
node_29: feature_name=cg10472711	feature_id[612].value > threshold=0.45985929667949677
node_37: feature_name=cg01937669	feature_id[188].value <= threshold=0.4809463769197464
node_38: feature_name=cg02157052	feature_id[451].value <= threshold=0.2869153320789337
node_39: feature_name=cg18751958	feature_id[1427].value > threshold=0.4813787043094635
node_41: feature_name=cg22512847	feature_id[1152].value > threshold=0.5129324495792389
node_43: feature_name=cg23345038	feature_id[1147].value > threshold=0.6318148374557495
node_45: feature_name=cg17439009	feature_id[1815].value <= threshold=0.48514117300510406
node_46: feature_name=cg17537493	feature_id[2772].value > threshold=0.5825372636318207
node_52: feature_name=cg01683570	feature_id[110].value > threshold=0.6040188074111938
node_54: feature_name=cg15720017	feature_id[168].value > threshold=0.7067741453647614
node_60: feature_name=cg23690444	feature_id[120].value <= threshold=0.16037724912166595
node_61: feature_name=cg00567872	feature_id[236].value > threshold=0.7005765736103058

node_85: feature_name=cg01014262	feature_id[764].value <= threshold=0.5737917125225067
node_86: feature_name=cg22946562	feature_id[1148].value > threshold=0.6328203976154327
node_134: feature_name=cg14514032	feature_id[202].value <= threshold=0.4389604330062866
node_135: feature_name=cg13488220	feature_id[241].value > threshold=0.7352153658866882
node_143: feature_name=cg03840920	feature_id[256].value > threshold=0.502630889415741
node_147: feature_name=cg13634090	feature_id[822].value > threshold=0.7207068204879761
node_151: feature_name=cg19584674	feature_id[2364].value <= threshold=0.41758519411087036
node_152: feature_name=cg07344990	feature_id[411].value > threshold=0.5484964847564697
node_158: feature_name=cg10298992	feature_id[3066].value > threshold=0.5707973837852478
node_174: feature_name=cg11843516	feature_id[20].value <= threshold=0.5590928494930267
node_175: feature_name=cg05185926	feature_id[448].value > threshold=0.17318664491176605
node_177: feature_name=cg17009731	feature_id[1220].value > threshold=0.6666513085365295
node_179: feature_name=cg01380319	feature_id[515].value <= threshold=0.39251960813999176
node_180: feature_name=cg20700977	feature_id[303].value > threshold=0.8422040641307831
node_184: feature_name=cg24259291	feature_id[164].value > threshold=0.4872268736362457
node_188: feature_name=cg08097657	feature_id[67].value > threshold=0.3371318429708481
node_190: feature_name=cg07891483	feature_id[59].value > threshold=0.6211732923984528
node_194: feature_name=cg20122645	feature_id[275].value > threshold=0.6094755828380585
node_196: feature_name=cg04026354	feature_id[1763].value > threshold=0.4341808259487152
node_286: feature_name=cg25408950	feature_id[772].value > threshold=0.680083692073822
node_290: feature_name=cg15188939	feature_id[1626].value > threshold=0.6365727782249451
node_294: feature_name=cg00444740	feature_id[3167].value <= threshold=0.5775478780269623

Class: chordoma (CHORD)	
Rules_91	passed counts:2
node_0: feature_name=cg11915444	feature_id[1171].value > threshold=0.3402601182460785
node_10: feature_name=cg12109728	feature_id[2810].value > threshold=0.7068270146846771
node_20: feature_name=cg16412000	feature_id[789].value > threshold=0.6741803884506226
node_24: feature_name=cg17115147	feature_id[1434].value <= threshold=0.2961975038051605
node_25: feature_name=cg24474622	feature_id[2737].value > threshold=0.7211934924125671
node_29: feature_name=cg10472711	feature_id[612].value > threshold=0.4598592966794967 7
node_37: feature_name=cg01937669	feature_id[188].value <= threshold=0.4809463769197464
node_38: feature_name=cg02157052	feature_id[451].value <= threshold=0.2869153320789337
node_39: feature_name=cg18751958	feature_id[1427].value > threshold=0.4813787043094635
node_41: feature_name=cg22512847	feature_id[1152].value > threshold=0.5129324495792389
node_43: feature_name=cg23345038	feature_id[1147].value > threshold=0.6318148374557495
node_45: feature_name=cg17439009	feature_id[1815].value <= threshold=0.48514117300510406
node_46: feature_name=cg17537493	feature_id[2772].value > threshold=0.5825372636318207
node_52: feature_name=cg01683570	feature_id[110].value > threshold=0.6040188074111938
node_54: feature_name=cg15720017	feature_id[168].value > threshold=0.7067741453647614
node_60: feature_name=cg23690444	feature_id[120].value <= threshold=0.16037724912166595
node_61: feature_name=cg00567872	feature_id[236].value > threshold=0.7005765736103058
node_85: feature_name=cg01014262	feature_id[764].value <= threshold=0.5737917125225067
node_86: feature_name=cg22946562	feature_id[1148].value > threshold=0.6328203976154327
node_134: feature_name=cg14514032	feature_id[202].value <= threshold=0.4389604330062866

node_135: feature_name=cg13488220	feature_id[241].value > threshold=0.7352153658866882
node_143: feature_name=cg03840920	feature_id[256].value > threshold=0.502630889415741
node_147: feature_name=cg13634090	feature_id[822].value > threshold=0.7207068204879761
node_151: feature_name=cg19584674	feature_id[2364].value <= threshold=0.41758519411087036
node_152: feature_name=cg07344990	feature_id[411].value > threshold=0.5484964847564697
node_158: feature_name=cg10298992	feature_id[3066].value > threshold=0.5707973837852478
node_174: feature_name=cg11843516	feature_id[20].value <= threshold=0.5590928494930267
node_175: feature_name=cg05185926	feature_id[448].value > threshold=0.17318664491176605
node_177: feature_name=cg17009731	feature_id[1220].value > threshold=0.6666513085365295
node_179: feature_name=cg01380319	feature_id[515].value <= threshold=0.39251960813999176
node_180: feature_name=cg20700977	feature_id[303].value > threshold=0.8422040641307831
node_184: feature_name=cg24259291	feature_id[164].value > threshold=0.4872268736362457
node_188: feature_name=cg08097657	feature_id[67].value > threshold=0.3371318429708481
node_190: feature_name=cg07891483	feature_id[59].value > threshold=0.6211732923984528
node_194: feature_name=cg20122645	feature_id[275].value > threshold=0.6094755828380585
node_196: feature_name=cg04026354	feature_id[1763].value > threshold=0.4341808259487152
node_286: feature_name=cg25408950	feature_id[772].value <= threshold=0.680083692073822
node_287: feature_name=cg17216243	feature_id[1006].value > threshold=0.6291826963424683
Class: chordoma (CHORD)	
Rules_92	passed counts:2
node_0: feature_name=cg11915444	feature_id[1171].value > threshold=0.3402601182460785
node_10: feature_name=cg12109728	feature_id[2810].value > threshold=0.7068270146846771

node_20: feature_name=cg16412000	feature_id[789].value > threshold=0.6741803884506226
node_24: feature_name=cg17115147	feature_id[1434].value <= threshold=0.2961975038051605
node_25: feature_name=cg24474622	feature_id[2737].value > threshold=0.7211934924125671
node_29: feature_name=cg10472711	feature_id[612].value > threshold=0.45985929667949677
node_37: feature_name=cg01937669	feature_id[188].value <= threshold=0.4809463769197464
node_38: feature_name=cg02157052	feature_id[451].value <= threshold=0.2869153320789337
node_39: feature_name=cg18751958	feature_id[1427].value > threshold=0.4813787043094635
node_41: feature_name=cg22512847	feature_id[1152].value > threshold=0.5129324495792389
node_43: feature_name=cg23345038	feature_id[1147].value > threshold=0.6318148374557495
node_45: feature_name=cg17439009	feature_id[1815].value <= threshold=0.48514117300510406
node_46: feature_name=cg17537493	feature_id[2772].value > threshold=0.5825372636318207
node_52: feature_name=cg01683570	feature_id[110].value > threshold=0.6040188074111938
node_54: feature_name=cg15720017	feature_id[168].value > threshold=0.7067741453647614
node_60: feature_name=cg23690444	feature_id[120].value <= threshold=0.16037724912166595
node_61: feature_name=cg00567872	feature_id[236].value > threshold=0.7005765736103058
node_85: feature_name=cg01014262	feature_id[764].value <= threshold=0.5737917125225067
node_86: feature_name=cg22946562	feature_id[1148].value > threshold=0.6328203976154327
node_134: feature_name=cg14514032	feature_id[202].value <= threshold=0.4389604330062866
node_135: feature_name=cg13488220	feature_id[241].value > threshold=0.7352153658866882
node_143: feature_name=cg03840920	feature_id[256].value > threshold=0.502630889415741
node_147: feature_name=cg13634090	feature_id[822].value > threshold=0.7207068204879761
node_151: feature_name=cg19584674	feature_id[2364].value <= threshold=0.41758519411087036

node_152: feature_name=cg07344990	feature_id[411].value > threshold=0.5484964847564697
node_158: feature_name=cg10298992	feature_id[3066].value > threshold=0.5707973837852478
node_174: feature_name=cg11843516	feature_id[20].value <= threshold=0.5590928494930267
node_175: feature_name=cg05185926	feature_id[448].value > threshold=0.17318664491176605
node_177: feature_name=cg17009731	feature_id[1220].value > threshold=0.6665130853652954
node_179: feature_name=cg01380319	feature_id[515].value <= threshold=0.39251960813999176
node_180: feature_name=cg20700977	feature_id[303].value > threshold=0.8422040641307831
node_184: feature_name=cg24259291	feature_id[164].value > threshold=0.4872268736362457
node_188: feature_name=cg08097657	feature_id[67].value > threshold=0.3371318429708481
node_190: feature_name=cg07891483	feature_id[59].value > threshold=0.6211732923984528
node_194: feature_name=cg20122645	feature_id[275].value > threshold=0.6094755828380585
node_196: feature_name=cg04026354	feature_id[1763].value <= threshold=0.4341808259487152
node_197: feature_name=cg10149889	feature_id[1017].value > threshold=0.6150195300579071
node_201: feature_name=cg14890730	feature_id[337].value <= threshold=0.4372602105140686
node_202: feature_name=cg08331427	feature_id[132].value > threshold=0.5443233847618103
node_208: feature_name=cg22730007	feature_id[2544].value > threshold=0.28619489073753357
node_210: feature_name=cg17193551	feature_id[2057].value > threshold=0.4393353909254074
node_212: feature_name=cg14671764	feature_id[584].value > threshold=0.9353199899196625
node_268: feature_name=cg21377260	feature_id[534].value > threshold=0.9840706288814545
node_276: feature_name=cg20934096	feature_id[251].value <= threshold=0.7597180306911469
Class: well differentiated liposarcoma (WDLS)/dedifferentiated liposarcoma (DDLS)	

Rules_93	passed counts:2
node_0: feature_name=cg11915444	feature_id[1171].value > threshold=0.3402601182460785
node_10: feature_name=cg12109728	feature_id[2810].value > threshold=0.7068270146846771
node_20: feature_name=cg16412000	feature_id[789].value > threshold=0.6741803884506226
node_24: feature_name=cg17115147	feature_id[1434].value <= threshold=0.2961975038051605
node_25: feature_name=cg24474622	feature_id[2737].value > threshold=0.7211934924125671
node_29: feature_name=cg10472711	feature_id[612].value > threshold=0.45985929667949677
node_37: feature_name=cg01937669	feature_id[188].value <= threshold=0.4809463769197464
node_38: feature_name=cg02157052	feature_id[451].value <= threshold=0.2869153320789337
node_39: feature_name=cg18751958	feature_id[1427].value > threshold=0.4813787043094635
node_41: feature_name=cg22512847	feature_id[1152].value > threshold=0.5129324495792389
node_43: feature_name=cg23345038	feature_id[1147].value > threshold=0.6318148374557495
node_45: feature_name=cg17439009	feature_id[1815].value <= threshold=0.48514117300510406
node_46: feature_name=cg17537493	feature_id[2772].value > threshold=0.5825372636318207
node_52: feature_name=cg01683570	feature_id[110].value > threshold=0.6040188074111938
node_54: feature_name=cg15720017	feature_id[168].value > threshold=0.7067741453647614
node_60: feature_name=cg23690444	feature_id[120].value <= threshold=0.16037724912166595
node_61: feature_name=cg00567872	feature_id[236].value > threshold=0.7005765736103058
node_85: feature_name=cg01014262	feature_id[764].value <= threshold=0.5737917125225067
node_86: feature_name=cg22946562	feature_id[1148].value > threshold=0.6328203976154327
node_134: feature_name=cg14514032	feature_id[202].value <= threshold=0.4389604330062866
node_135: feature_name=cg13488220	feature_id[241].value > threshold=0.7352153658866882

node_143: feature_name=cg03840920	feature_id[256].value > threshold=0.502630889415741
node_147: feature_name=cg13634090	feature_id[822].value > threshold=0.7207068204879761
node_151: feature_name=cg19584674	feature_id[2364].value <= threshold=0.41758519411087036
node_152: feature_name=cg07344990	feature_id[411].value > threshold=0.5484964847564697
node_158: feature_name=cg10298992	feature_id[3066].value > threshold=0.5707973837852478
node_174: feature_name=cg11843516	feature_id[20].value <= threshold=0.5590928494930267
node_175: feature_name=cg05185926	feature_id[448].value > threshold=0.17318664491176605
node_177: feature_name=cg17009731	feature_id[1220].value > threshold=0.6666513085365295
node_179: feature_name=cg01380319	feature_id[515].value <= threshold=0.39251960813999176
node_180: feature_name=cg20700977	feature_id[303].value > threshold=0.8422040641307831
node_184: feature_name=cg24259291	feature_id[164].value > threshold=0.4872268736362457
node_188: feature_name=cg08097657	feature_id[67].value > threshold=0.3371318429708481
node_190: feature_name=cg07891483	feature_id[59].value > threshold=0.6211732923984528
node_194: feature_name=cg20122645	feature_id[275].value > threshold=0.6094755828380585
node_196: feature_name=cg04026354	feature_id[1763].value <= threshold=0.4341808259487152
node_197: feature_name=cg10149889	feature_id[1017].value > threshold=0.6150195300579071
node_201: feature_name=cg14890730	feature_id[337].value <= threshold=0.4372602105140686
node_202: feature_name=cg08331427	feature_id[132].value > threshold=0.5443233847618103
node_208: feature_name=cg22730007	feature_id[2544].value > threshold=0.28619489073753357
node_210: feature_name=cg17193551	feature_id[2057].value > threshold=0.4393353909254074
node_212: feature_name=cg14671764	feature_id[584].value > threshold=0.9353199899196625
node_268: feature_name=cg21377260	feature_id[534].value <= threshold=0.9840706288814545

node_269: feature_name=cg27143326	feature_id[1403].value <= threshold=0.8949433267116547
node_270: feature_name=cg01339351	feature_id[2694].value > threshold=0.8992902934551239
Class: chondroblastoma (CB)	
Rules_94	passed counts:2
node_0: feature_name=cg11915444	feature_id[1171].value > threshold=0.3402601182460785
node_10: feature_name=cg12109728	feature_id[2810].value > threshold=0.7068270146846771
node_20: feature_name=cg16412000	feature_id[789].value > threshold=0.6741803884506226
node_24: feature_name=cg17115147	feature_id[1434].value <= threshold=0.2961975038051605
node_25: feature_name=cg24474622	feature_id[2737].value > threshold=0.7211934924125671
node_29: feature_name=cg10472711	feature_id[612].value > threshold=0.45985929667949677
node_37: feature_name=cg01937669	feature_id[188].value <= threshold=0.4809463769197464
node_38: feature_name=cg02157052	feature_id[451].value <= threshold=0.2869153320789337
node_39: feature_name=cg18751958	feature_id[1427].value > threshold=0.4813787043094635
node_41: feature_name=cg22512847	feature_id[1152].value > threshold=0.5129324495792389
node_43: feature_name=cg23345038	feature_id[1147].value > threshold=0.6318148374557495
node_45: feature_name=cg17439009	feature_id[1815].value <= threshold=0.48514117300510406
node_46: feature_name=cg17537493	feature_id[2772].value > threshold=0.5825372636318207
node_52: feature_name=cg01683570	feature_id[110].value > threshold=0.6040188074111938
node_54: feature_name=cg15720017	feature_id[168].value > threshold=0.7067741453647614
node_60: feature_name=cg23690444	feature_id[120].value <= threshold=0.16037724912166595
node_61: feature_name=cg00567872	feature_id[236].value > threshold=0.7005765736103058
node_85: feature_name=cg01014262	feature_id[764].value <= threshold=0.5737917125225067

node_86: feature_name=cg22946562	feature_id[1148].value > threshold=0.6328203976154327
node_134: feature_name=cg14514032	feature_id[202].value <= threshold=0.4389604330062866
node_135: feature_name=cg13488220	feature_id[241].value > threshold=0.7352153658866882
node_143: feature_name=cg03840920	feature_id[256].value > threshold=0.502630889415741
node_147: feature_name=cg13634090	feature_id[822].value > threshold=0.7207068204879761
node_151: feature_name=cg19584674	feature_id[2364].value <= threshold=0.41758519411087036
node_152: feature_name=cg07344990	feature_id[411].value > threshold=0.5484964847564697
node_158: feature_name=cg10298992	feature_id[3066].value > threshold=0.5707973837852478
node_174: feature_name=cg11843516	feature_id[20].value <= threshold=0.5590928494930267
node_175: feature_name=cg05185926	feature_id[448].value > threshold=0.17318664491176605
node_177: feature_name=cg17009731	feature_id[1220].value > threshold=0.6666513085365295
node_179: feature_name=cg01380319	feature_id[515].value <= threshold=0.39251960813999176
node_180: feature_name=cg20700977	feature_id[303].value > threshold=0.8422040641307831
node_184: feature_name=cg24259291	feature_id[164].value > threshold=0.4872268736362457
node_188: feature_name=cg08097657	feature_id[67].value > threshold=0.3371318429708481
node_190: feature_name=cg07891483	feature_id[59].value > threshold=0.6211732923984528
node_194: feature_name=cg20122645	feature_id[275].value > threshold=0.6094755828380585
node_196: feature_name=cg04026354	feature_id[1763].value <= threshold=0.4341808259487152
node_197: feature_name=cg10149889	feature_id[1017].value > threshold=0.6150195300579071
node_201: feature_name=cg14890730	feature_id[337].value <= threshold=0.4372602105140686
node_202: feature_name=cg08331427	feature_id[132].value > threshold=0.5443233847618103
node_208: feature_name=cg22730007	feature_id[2544].value > threshold=0.28619489073753357

node_210: feature_name=cg17193551	feature_id[2057].value > threshold=0.4393353909254074
node_212: feature_name=cg14671764	feature_id[584].value <= threshold=0.9353199899196625
node_213: feature_name=cg20140662	feature_id[2044].value > threshold=0.38030287623405457
node_215: feature_name=cg07891483	feature_id[59].value > threshold=0.829958975315094
node_219: feature_name=cg04884579	feature_id[1861].value > threshold=0.6106154918670654
node_229: feature_name=cg19548524	feature_id[2985].value > threshold=0.8623353838920593
node_233: feature_name=cg14788686	feature_id[3556].value > threshold=0.35090239346027374
node_235: feature_name=cg05380734	feature_id[2470].value <= threshold=0.7112160623073578
node_236: feature_name=cg01166827	feature_id[3708].value > threshold=0.6359498500823975
node_238: feature_name=cg13251842	feature_id[714].value > threshold=0.8916542232036591
node_240: feature_name=cg02829279	feature_id[623].value > threshold=0.450528159737587
node_242: feature_name=cg24358599	feature_id[86].value > threshold=0.8132582604885101
node_246: feature_name=cg05544807	feature_id[3661].value <= threshold=0.6726464331150055
node_247: feature_name=cg05056653	feature_id[3122].value > threshold=0.35445791482925415
node_249: feature_name=cg26308909	feature_id[770].value > threshold=0.3754204213619232
node_251: feature_name=cg11197908	feature_id[3058].value > threshold=0.36795753240585327
node_253: feature_name=cg00039385	feature_id[646].value > threshold=0.18521490693092346
Class: embryonal rhabdomyosarcoma (RMS (EMB))	
Rules_95	passed counts:2
node_0: feature_name=cg11915444	feature_id[1171].value > threshold=0.3402601182460785
node_10: feature_name=cg12109728	feature_id[2810].value > threshold=0.7068270146846771

node_20: feature_name=cg16412000	feature_id[789].value > threshold=0.6741803884506226
node_24: feature_name=cg17115147	feature_id[1434].value <= threshold=0.2961975038051605
node_25: feature_name=cg24474622	feature_id[2737].value > threshold=0.7211934924125671
node_29: feature_name=cg10472711	feature_id[612].value > threshold=0.45985929667949677
node_37: feature_name=cg01937669	feature_id[188].value <= threshold=0.4809463769197464
node_38: feature_name=cg02157052	feature_id[451].value <= threshold=0.2869153320789337
node_39: feature_name=cg18751958	feature_id[1427].value > threshold=0.4813787043094635
node_41: feature_name=cg22512847	feature_id[1152].value > threshold=0.5129324495792389
node_43: feature_name=cg23345038	feature_id[1147].value > threshold=0.6318148374557495
node_45: feature_name=cg17439009	feature_id[1815].value <= threshold=0.48514117300510406
node_46: feature_name=cg17537493	feature_id[2772].value > threshold=0.5825372636318207
node_52: feature_name=cg01683570	feature_id[110].value > threshold=0.6040188074111938
node_54: feature_name=cg15720017	feature_id[168].value > threshold=0.7067741453647614
node_60: feature_name=cg23690444	feature_id[120].value <= threshold=0.16037724912166595
node_61: feature_name=cg00567872	feature_id[236].value > threshold=0.7005765736103058
node_85: feature_name=cg01014262	feature_id[764].value <= threshold=0.5737917125225067
node_86: feature_name=cg22946562	feature_id[1148].value > threshold=0.6328203976154327
node_134: feature_name=cg14514032	feature_id[202].value <= threshold=0.4389604330062866
node_135: feature_name=cg13488220	feature_id[241].value > threshold=0.7352153658866882
node_143: feature_name=cg03840920	feature_id[256].value > threshold=0.502630889415741
node_147: feature_name=cg13634090	feature_id[822].value > threshold=0.7207068204879761
node_151: feature_name=cg19584674	feature_id[2364].value <= threshold=0.41758519411087036

node_152: feature_name=cg07344990	feature_id[411].value > threshold=0.5484964847564697
node_158: feature_name=cg10298992	feature_id[3066].value > threshold=0.5707973837852478
node_174: feature_name=cg11843516	feature_id[20].value <= threshold=0.5590928494930267
node_175: feature_name=cg05185926	feature_id[448].value > threshold=0.17318664491176605
node_177: feature_name=cg17009731	feature_id[1220].value > threshold=0.6666513085365295
node_179: feature_name=cg01380319	feature_id[515].value <= threshold=0.39251960813999176
node_180: feature_name=cg20700977	feature_id[303].value > threshold=0.8422040641307831
node_184: feature_name=cg24259291	feature_id[164].value > threshold=0.4872268736362457
node_188: feature_name=cg08097657	feature_id[67].value > threshold=0.3371318429708481
node_190: feature_name=cg07891483	feature_id[59].value > threshold=0.6211732923984528
node_194: feature_name=cg20122645	feature_id[275].value > threshold=0.6094755828380585
node_196: feature_name=cg04026354	feature_id[1763].value <= threshold=0.4341808259487152
node_197: feature_name=cg10149889	feature_id[1017].value > threshold=0.6150195300579071
node_201: feature_name=cg14890730	feature_id[337].value <= threshold=0.4372602105140686
node_202: feature_name=cg08331427	feature_id[132].value > threshold=0.5443233847618103
node_208: feature_name=cg22730007	feature_id[2544].value > threshold=0.28619489073753357
node_210: feature_name=cg17193551	feature_id[2057].value > threshold=0.4393353909254074
node_212: feature_name=cg14671764	feature_id[584].value <= threshold=0.9353199899196625
node_213: feature_name=cg20140662	feature_id[2044].value > threshold=0.38030287623405457
node_215: feature_name=cg07891483	feature_id[59].value > threshold=0.829958975315094
node_219: feature_name=cg04884579	feature_id[1861].value > threshold=0.6106154918670654
node_229: feature_name=cg19548524	feature_id[2985].value > threshold=0.8623353838920593

node_233: feature_name=cg14788686	feature_id[3556].value > threshold=0.35090239346027374
node_235: feature_name=cg05380734	feature_id[2470].value <= threshold=0.7112160623073578
node_236: feature_name=cg01166827	feature_id[3708].value > threshold=0.6359498500823975
node_238: feature_name=cg13251842	feature_id[714].value > threshold=0.8916542232036591
node_240: feature_name=cg02829279	feature_id[623].value > threshold=0.450528159737587
node_242: feature_name=cg24358599	feature_id[86].value > threshold=0.8132582604885101
node_246: feature_name=cg05544807	feature_id[3661].value <= threshold=0.6726464331150055
node_247: feature_name=cg05056653	feature_id[3122].value > threshold=0.35445791482925415
node_249: feature_name=cg26308909	feature_id[770].value > threshold=0.3754204213619232
node_251: feature_name=cg11197908	feature_id[3058].value > threshold=0.36795753240585327
node_253: feature_name=cg00039385	feature_id[646].value <= threshold=0.18521490693092346
node_254: feature_name=cg04787317	feature_id[3379].value <= threshold=0.8155552744865417
Class: Kaposi sarcoma (KS)	
Rules_96	passed counts:2
node_0: feature_name=cg11915444	feature_id[1171].value > threshold=0.3402601182460785
node_10: feature_name=cg12109728	feature_id[2810].value > threshold=0.7068270146846771
node_20: feature_name=cg16412000	feature_id[789].value > threshold=0.6741803884506226
node_24: feature_name=cg17115147	feature_id[1434].value <= threshold=0.2961975038051605
node_25: feature_name=cg24474622	feature_id[2737].value > threshold=0.7211934924125671
node_29: feature_name=cg10472711	feature_id[612].value > threshold=0.45985929667949677
node_37: feature_name=cg01937669	feature_id[188].value <= threshold=0.4809463769197464
node_38: feature_name=cg02157052	feature_id[451].value <= threshold=0.2869153320789337

node_39: feature_name=cg18751958	feature_id[1427].value > threshold=0.4813787043094635
node_41: feature_name=cg22512847	feature_id[1152].value > threshold=0.5129324495792389
node_43: feature_name=cg23345038	feature_id[1147].value > threshold=0.6318148374557495
node_45: feature_name=cg17439009	feature_id[1815].value <= threshold=0.48514117300510406
node_46: feature_name=cg17537493	feature_id[2772].value > threshold=0.5825372636318207
node_52: feature_name=cg01683570	feature_id[110].value > threshold=0.6040188074111938
node_54: feature_name=cg15720017	feature_id[168].value > threshold=0.7067741453647614
node_60: feature_name=cg23690444	feature_id[120].value <= threshold=0.16037724912166595
node_61: feature_name=cg00567872	feature_id[236].value > threshold=0.7005765736103058
node_85: feature_name=cg01014262	feature_id[764].value <= threshold=0.5737917125225067
node_86: feature_name=cg22946562	feature_id[1148].value > threshold=0.6328203976154327
node_134: feature_name=cg14514032	feature_id[202].value <= threshold=0.4389604330062866
node_135: feature_name=cg13488220	feature_id[241].value > threshold=0.7352153658866882
node_143: feature_name=cg03840920	feature_id[256].value > threshold=0.502630889415741
node_147: feature_name=cg13634090	feature_id[822].value > threshold=0.7207068204879761
node_151: feature_name=cg19584674	feature_id[2364].value <= threshold=0.41758519411087036
node_152: feature_name=cg07344990	feature_id[411].value > threshold=0.5484964847564697
node_158: feature_name=cg10298992	feature_id[3066].value > threshold=0.5707973837852478
node_174: feature_name=cg11843516	feature_id[20].value <= threshold=0.5590928494930267
node_175: feature_name=cg05185926	feature_id[448].value > threshold=0.17318664491176605
node_177: feature_name=cg17009731	feature_id[1220].value > threshold=0.6666513085365295
node_179: feature_name=cg01380319	feature_id[515].value <= threshold=0.39251960813999176

node_180: feature_name=cg20700977	feature_id[303].value > threshold=0.8422040641307831
node_184: feature_name=cg24259291	feature_id[164].value > threshold=0.4872268736362457
node_188: feature_name=cg08097657	feature_id[67].value > threshold=0.3371318429708481
node_190: feature_name=cg07891483	feature_id[59].value > threshold=0.6211732923984528
node_194: feature_name=cg20122645	feature_id[275].value > threshold=0.6094755828380585
node_196: feature_name=cg04026354	feature_id[1763].value <= threshold=0.4341808259487152
node_197: feature_name=cg10149889	feature_id[1017].value > threshold=0.6150195300579071
node_201: feature_name=cg14890730	feature_id[337].value <= threshold=0.4372602105140686
node_202: feature_name=cg08331427	feature_id[132].value > threshold=0.5443233847618103
node_208: feature_name=cg22730007	feature_id[2544].value > threshold=0.28619489073753357
node_210: feature_name=cg17193551	feature_id[2057].value > threshold=0.4393353909254074
node_212: feature_name=cg14671764	feature_id[584].value <= threshold=0.9353199899196625
node_213: feature_name=cg20140662	feature_id[2044].value > threshold=0.38030287623405457
node_215: feature_name=cg07891483	feature_id[59].value > threshold=0.829958975315094
node_219: feature_name=cg04884579	feature_id[1861].value > threshold=0.6106154918670654
node_229: feature_name=cg19548524	feature_id[2985].value > threshold=0.8623353838920593
node_233: feature_name=cg14788686	feature_id[3556].value > threshold=0.35090239346027374
node_235: feature_name=cg05380734	feature_id[2470].value <= threshold=0.7112160623073578
node_236: feature_name=cg01166827	feature_id[3708].value > threshold=0.6359498500823975
node_238: feature_name=cg13251842	feature_id[714].value > threshold=0.8916542232036591
node_240: feature_name=cg02829279	feature_id[623].value > threshold=0.450528159737587
node_242: feature_name=cg24358599	feature_id[86].value > threshold=0.8132582604885101

node_246: feature_name=cg05544807	feature_id[3661].value <= threshold=0.6726464331150055
node_247: feature_name=cg05056653	feature_id[3122].value > threshold=0.35445791482925415
node_249: feature_name=cg26308909	feature_id[770].value > threshold=0.3754204213619232
node_251: feature_name=cg11197908	feature_id[3058].value <= threshold=0.36795753240585327
Class: rhabdomyosarcoma (RMS)	
Rules_97	passed counts:2
node_0: feature_name=cg11915444	feature_id[1171].value > threshold=0.3402601182460785
node_10: feature_name=cg12109728	feature_id[2810].value > threshold=0.7068270146846771
node_20: feature_name=cg16412000	feature_id[789].value > threshold=0.6741803884506226
node_24: feature_name=cg17115147	feature_id[1434].value <= threshold=0.2961975038051605
node_25: feature_name=cg24474622	feature_id[2737].value > threshold=0.7211934924125671
node_29: feature_name=cg10472711	feature_id[612].value > threshold=0.45985929667949677
node_37: feature_name=cg01937669	feature_id[188].value <= threshold=0.4809463769197464
node_38: feature_name=cg02157052	feature_id[451].value <= threshold=0.2869153320789337
node_39: feature_name=cg18751958	feature_id[1427].value > threshold=0.4813787043094635
node_41: feature_name=cg22512847	feature_id[1152].value > threshold=0.5129324495792389
node_43: feature_name=cg23345038	feature_id[1147].value > threshold=0.6318148374557495
node_45: feature_name=cg17439009	feature_id[1815].value <= threshold=0.48514117300510406
node_46: feature_name=cg17537493	feature_id[2772].value > threshold=0.5825372636318207
node_52: feature_name=cg01683570	feature_id[110].value > threshold=0.6040188074111938
node_54: feature_name=cg15720017	feature_id[168].value > threshold=0.7067741453647614
node_60: feature_name=cg23690444	feature_id[120].value <= threshold=0.16037724912166595

node_61: feature_name=cg00567872	feature_id[236].value > threshold=0.7005765736103058
node_85: feature_name=cg01014262	feature_id[764].value <= threshold=0.5737917125225067
node_86: feature_name=cg22946562	feature_id[1148].value > threshold=0.6328203976154327
node_134: feature_name=cg14514032	feature_id[202].value <= threshold=0.4389604330062866
node_135: feature_name=cg13488220	feature_id[241].value > threshold=0.7352153658866882
node_143: feature_name=cg03840920	feature_id[256].value > threshold=0.502630889415741
node_147: feature_name=cg13634090	feature_id[822].value > threshold=0.7207068204879761
node_151: feature_name=cg19584674	feature_id[2364].value <= threshold=0.41758519411087036
node_152: feature_name=cg07344990	feature_id[411].value > threshold=0.5484964847564697
node_158: feature_name=cg10298992	feature_id[3066].value > threshold=0.5707973837852478
node_174: feature_name=cg11843516	feature_id[20].value <= threshold=0.5590928494930267
node_175: feature_name=cg05185926	feature_id[448].value > threshold=0.17318664491176605
node_177: feature_name=cg17009731	feature_id[1220].value > threshold=0.6666513085365295
node_179: feature_name=cg01380319	feature_id[515].value <= threshold=0.39251960813999176
node_180: feature_name=cg20700977	feature_id[303].value > threshold=0.8422040641307831
node_184: feature_name=cg24259291	feature_id[164].value > threshold=0.4872268736362457
node_188: feature_name=cg08097657	feature_id[67].value > threshold=0.3371318429708481
node_190: feature_name=cg07891483	feature_id[59].value > threshold=0.6211732923984528
node_194: feature_name=cg20122645	feature_id[275].value > threshold=0.6094755828380585
node_196: feature_name=cg04026354	feature_id[1763].value <= threshold=0.4341808259487152
node_197: feature_name=cg10149889	feature_id[1017].value > threshold=0.6150195300579071
node_201: feature_name=cg14890730	feature_id[337].value <= threshold=0.4372602105140686

node_202: feature_name=cg08331427	feature_id[132].value > threshold=0.5443233847618103
node_208: feature_name=cg22730007	feature_id[2544].value > threshold=0.28619489073753357
node_210: feature_name=cg17193551	feature_id[2057].value > threshold=0.4393353909254074
node_212: feature_name=cg14671764	feature_id[584].value <= threshold=0.9353199899196625
node_213: feature_name=cg20140662	feature_id[2044].value > threshold=0.38030287623405457
node_215: feature_name=cg07891483	feature_id[59].value > threshold=0.829958975315094
node_219: feature_name=cg04884579	feature_id[1861].value > threshold=0.6106154918670654
node_229: feature_name=cg19548524	feature_id[2985].value > threshold=0.8623353838920593
node_233: feature_name=cg14788686	feature_id[3556].value > threshold=0.35090239346027374
node_235: feature_name=cg05380734	feature_id[2470].value <= threshold=0.7112160623073578
node_236: feature_name=cg01166827	feature_id[3708].value > threshold=0.6359498500823975
node_238: feature_name=cg13251842	feature_id[714].value > threshold=0.8916542232036591
node_240: feature_name=cg02829279	feature_id[623].value > threshold=0.450528159737587
node_242: feature_name=cg24358599	feature_id[86].value > threshold=0.8132582604885101
node_246: feature_name=cg05544807	feature_id[3661].value <= threshold=0.6726464331150055
node_247: feature_name=cg05056653	feature_id[3122].value > threshold=0.35445791482925415
node_249: feature_name=cg26308909	feature_id[770].value <= threshold=0.3754204213619232
Class: sclerosing epithelioid fibrosarcoma(SEF)	
Rules_98	passed counts:2
node_0: feature_name=cg11915444	feature_id[1171].value > threshold=0.3402601182460785
node_10: feature_name=cg12109728	feature_id[2810].value > threshold=0.7068270146846771
node_20: feature_name=cg16412000	feature_id[789].value > threshold=0.6741803884506226

node_24: feature_name=cg17115147	feature_id[1434].value <= threshold=0.2961975038051605
node_25: feature_name=cg24474622	feature_id[2737].value > threshold=0.7211934924125671
node_29: feature_name=cg10472711	feature_id[612].value > threshold=0.4598592967949677
node_37: feature_name=cg01937669	feature_id[188].value <= threshold=0.4809463769197464
node_38: feature_name=cg02157052	feature_id[451].value <= threshold=0.2869153320789337
node_39: feature_name=cg18751958	feature_id[1427].value > threshold=0.4813787043094635
node_41: feature_name=cg22512847	feature_id[1152].value > threshold=0.5129324495792389
node_43: feature_name=cg23345038	feature_id[1147].value > threshold=0.6318148374557495
node_45: feature_name=cg17439009	feature_id[1815].value <= threshold=0.48514117300510406
node_46: feature_name=cg17537493	feature_id[2772].value > threshold=0.5825372636318207
node_52: feature_name=cg01683570	feature_id[110].value > threshold=0.6040188074111938
node_54: feature_name=cg15720017	feature_id[168].value > threshold=0.7067741453647614
node_60: feature_name=cg23690444	feature_id[120].value <= threshold=0.16037724912166595
node_61: feature_name=cg00567872	feature_id[236].value > threshold=0.7005765736103058
node_85: feature_name=cg01014262	feature_id[764].value <= threshold=0.5737917125225067
node_86: feature_name=cg22946562	feature_id[1148].value > threshold=0.6328203976154327
node_134: feature_name=cg14514032	feature_id[202].value <= threshold=0.4389604330062866
node_135: feature_name=cg13488220	feature_id[241].value > threshold=0.7352153658866882
node_143: feature_name=cg03840920	feature_id[256].value > threshold=0.502630889415741
node_147: feature_name=cg13634090	feature_id[822].value > threshold=0.7207068204879761
node_151: feature_name=cg19584674	feature_id[2364].value <= threshold=0.41758519411087036
node_152: feature_name=cg07344990	feature_id[411].value > threshold=0.5484964847564697

node_158: feature_name=cg10298992	feature_id[3066].value > threshold=0.5707973837852478
node_174: feature_name=cg11843516	feature_id[20].value <= threshold=0.5590928494930267
node_175: feature_name=cg05185926	feature_id[448].value > threshold=0.17318664491176605
node_177: feature_name=cg17009731	feature_id[1220].value > threshold=0.6666513085365295
node_179: feature_name=cg01380319	feature_id[515].value <= threshold=0.39251960813999176
node_180: feature_name=cg20700977	feature_id[303].value > threshold=0.8422040641307831
node_184: feature_name=cg24259291	feature_id[164].value > threshold=0.4872268736362457
node_188: feature_name=cg08097657	feature_id[67].value > threshold=0.3371318429708481
node_190: feature_name=cg07891483	feature_id[59].value > threshold=0.6211732923984528
node_194: feature_name=cg20122645	feature_id[275].value > threshold=0.6094755828380585
node_196: feature_name=cg04026354	feature_id[1763].value <= threshold=0.4341808259487152
node_197: feature_name=cg10149889	feature_id[1017].value > threshold=0.6150195300579071
node_201: feature_name=cg14890730	feature_id[337].value <= threshold=0.4372602105140686
node_202: feature_name=cg08331427	feature_id[132].value > threshold=0.5443233847618103
node_208: feature_name=cg22730007	feature_id[2544].value > threshold=0.28619489073753357
node_210: feature_name=cg17193551	feature_id[2057].value > threshold=0.4393353909254074
node_212: feature_name=cg14671764	feature_id[584].value <= threshold=0.9353199899196625
node_213: feature_name=cg20140662	feature_id[2044].value > threshold=0.38030287623405457
node_215: feature_name=cg07891483	feature_id[59].value > threshold=0.829958975315094
node_219: feature_name=cg04884579	feature_id[1861].value > threshold=0.6106154918670654
node_229: feature_name=cg19548524	feature_id[2985].value > threshold=0.8623353838920593
node_233: feature_name=cg14788686	feature_id[3556].value > threshold=0.35090239346027374

node_235: feature_name=cg05380734	feature_id[2470].value <= threshold=0.7112160623073578
node_236: feature_name=cg01166827	feature_id[3708].value > threshold=0.6359498500823975
node_238: feature_name=cg13251842	feature_id[714].value > threshold=0.8916542232036591
node_240: feature_name=cg02829279	feature_id[623].value > threshold=0.450528159737587
node_242: feature_name=cg24358599	feature_id[86].value > threshold=0.8132582604885101
node_246: feature_name=cg05544807	feature_id[3661].value <= threshold=0.6726464331150055
node_247: feature_name=cg05056653	feature_id[3122].value <= threshold=0.35445791482925415
Class: malignant peripheral nerve sheath tumour (MPNST)	
Rules_99	passed counts:2
node_0: feature_name=cg11915444	feature_id[1171].value > threshold=0.3402601182460785
node_10: feature_name=cg12109728	feature_id[2810].value > threshold=0.7068270146846771
node_20: feature_name=cg16412000	feature_id[789].value > threshold=0.6741803884506226
node_24: feature_name=cg17115147	feature_id[1434].value <= threshold=0.2961975038051605
node_25: feature_name=cg24474622	feature_id[2737].value > threshold=0.7211934924125671
node_29: feature_name=cg10472711	feature_id[612].value > threshold=0.45985929667949677
node_37: feature_name=cg01937669	feature_id[188].value <= threshold=0.4809463769197464
node_38: feature_name=cg02157052	feature_id[451].value <= threshold=0.2869153320789337
node_39: feature_name=cg18751958	feature_id[1427].value > threshold=0.4813787043094635
node_41: feature_name=cg22512847	feature_id[1152].value > threshold=0.5129324495792389
node_43: feature_name=cg23345038	feature_id[1147].value > threshold=0.6318148374557495
node_45: feature_name=cg17439009	feature_id[1815].value <= threshold=0.48514117300510406

node_46: feature_name=cg17537493	feature_id[2772].value > threshold=0.5825372636318207
node_52: feature_name=cg01683570	feature_id[110].value > threshold=0.6040188074111938
node_54: feature_name=cg15720017	feature_id[168].value > threshold=0.7067741453647614
node_60: feature_name=cg23690444	feature_id[120].value <= threshold=0.16037724912166595
node_61: feature_name=cg00567872	feature_id[236].value > threshold=0.7005765736103058
node_85: feature_name=cg01014262	feature_id[764].value <= threshold=0.5737917125225067
node_86: feature_name=cg22946562	feature_id[1148].value > threshold=0.6328203976154327
node_134: feature_name=cg14514032	feature_id[202].value <= threshold=0.4389604330062866
node_135: feature_name=cg13488220	feature_id[241].value > threshold=0.7352153658866882
node_143: feature_name=cg03840920	feature_id[256].value > threshold=0.502630889415741
node_147: feature_name=cg13634090	feature_id[822].value > threshold=0.7207068204879761
node_151: feature_name=cg19584674	feature_id[2364].value <= threshold=0.41758519411087036
node_152: feature_name=cg07344990	feature_id[411].value > threshold=0.5484964847564697
node_158: feature_name=cg10298992	feature_id[3066].value > threshold=0.5707973837852478
node_174: feature_name=cg11843516	feature_id[20].value <= threshold=0.5590928494930267
node_175: feature_name=cg05185926	feature_id[448].value > threshold=0.17318664491176605
node_177: feature_name=cg17009731	feature_id[1220].value > threshold=0.6666513085365295
node_179: feature_name=cg01380319	feature_id[515].value <= threshold=0.39251960813999176
node_180: feature_name=cg20700977	feature_id[303].value > threshold=0.8422040641307831
node_184: feature_name=cg24259291	feature_id[164].value > threshold=0.4872268736362457
node_188: feature_name=cg08097657	feature_id[67].value > threshold=0.3371318429708481
node_190: feature_name=cg07891483	feature_id[59].value > threshold=0.6211732923984528

node_194: feature_name=cg20122645	feature_id[275].value > threshold=0.6094755828380585
node_196: feature_name=cg04026354	feature_id[1763].value <= threshold=0.4341808259487152
node_197: feature_name=cg10149889	feature_id[1017].value > threshold=0.6150195300579071
node_201: feature_name=cg14890730	feature_id[337].value <= threshold=0.4372602105140686
node_202: feature_name=cg08331427	feature_id[132].value > threshold=0.5443233847618103
node_208: feature_name=cg22730007	feature_id[2544].value > threshold=0.28619489073753357
node_210: feature_name=cg17193551	feature_id[2057].value > threshold=0.4393353909254074
node_212: feature_name=cg14671764	feature_id[584].value <= threshold=0.9353199899196625
node_213: feature_name=cg20140662	feature_id[2044].value > threshold=0.38030287623405457
node_215: feature_name=cg07891483	feature_id[59].value > threshold=0.829958975315094
node_219: feature_name=cg04884579	feature_id[1861].value <= threshold=0.6106154918670654
node_220: feature_name=cg25066857	feature_id[3759].value <= threshold=0.8149203658103943
node_221: feature_name=cg04813695	feature_id[1758].value > threshold=0.8937293887138367
node_223: feature_name=cg12895304	feature_id[5].value > threshold=0.8254048228263855
Class: well differentiated liposarcoma (WDLS)/dedifferentiated liposarcoma (DDLS)	
Rules_100	passed counts:2
node_0: feature_name=cg11915444	feature_id[1171].value > threshold=0.3402601182460785
node_10: feature_name=cg12109728	feature_id[2810].value > threshold=0.7068270146846771
node_20: feature_name=cg16412000	feature_id[789].value > threshold=0.6741803884506226
node_24: feature_name=cg17115147	feature_id[1434].value <= threshold=0.2961975038051605
node_25: feature_name=cg24474622	feature_id[2737].value > threshold=0.7211934924125671

node_29: feature_name=cg10472711	feature_id[612].value > threshold=0.4598592966794967 7
node_37: feature_name=cg01937669	feature_id[188].value <= threshold=0.4809463769197464
node_38: feature_name=cg02157052	feature_id[451].value <= threshold=0.2869153320789337
node_39: feature_name=cg18751958	feature_id[1427].value > threshold=0.4813787043094635
node_41: feature_name=cg22512847	feature_id[1152].value > threshold=0.5129324495792389
node_43: feature_name=cg23345038	feature_id[1147].value > threshold=0.6318148374557495
node_45: feature_name=cg17439009	feature_id[1815].value <= threshold=0.48514117300510406
node_46: feature_name=cg17537493	feature_id[2772].value > threshold=0.5825372636318207
node_52: feature_name=cg01683570	feature_id[110].value > threshold=0.6040188074111938
node_54: feature_name=cg15720017	feature_id[168].value > threshold=0.7067741453647614
node_60: feature_name=cg23690444	feature_id[120].value <= threshold=0.16037724912166595
node_61: feature_name=cg00567872	feature_id[236].value > threshold=0.7005765736103058
node_85: feature_name=cg01014262	feature_id[764].value <= threshold=0.5737917125225067
node_86: feature_name=cg22946562	feature_id[1148].value > threshold=0.6328203976154327
node_134: feature_name=cg14514032	feature_id[202].value <= threshold=0.4389604330062866
node_135: feature_name=cg13488220	feature_id[241].value > threshold=0.7352153658866882
node_143: feature_name=cg03840920	feature_id[256].value > threshold=0.502630889415741
node_147: feature_name=cg13634090	feature_id[822].value > threshold=0.7207068204879761
node_151: feature_name=cg19584674	feature_id[2364].value <= threshold=0.41758519411087036
node_152: feature_name=cg07344990	feature_id[411].value > threshold=0.5484964847564697
node_158: feature_name=cg10298992	feature_id[3066].value <= threshold=0.5707973837852478
node_159: feature_name=cg24852135	feature_id[47].value <= threshold=0.8445721566677094

node_160: feature_name=cg25606046	feature_id[2007].value <= threshold=0.8722946643829346
node_161: feature_name=cg03212620	feature_id[1188].value > threshold=0.4703744798898697
node_163: feature_name=cg27096087	feature_id[2512].value <= threshold=0.8232799470424652
Class: chondroblastoma (CB)	
Rules_101	passed counts:2
node_0: feature_name=cg11915444	feature_id[1171].value > threshold=0.3402601182460785
node_10: feature_name=cg12109728	feature_id[2810].value > threshold=0.7068270146846771
node_20: feature_name=cg16412000	feature_id[789].value > threshold=0.6741803884506226
node_24: feature_name=cg17115147	feature_id[1434].value <= threshold=0.2961975038051605
node_25: feature_name=cg24474622	feature_id[2737].value > threshold=0.7211934924125671
node_29: feature_name=cg10472711	feature_id[612].value > threshold=0.45985929667949677
node_37: feature_name=cg01937669	feature_id[188].value <= threshold=0.4809463769197464
node_38: feature_name=cg02157052	feature_id[451].value <= threshold=0.2869153320789337
node_39: feature_name=cg18751958	feature_id[1427].value > threshold=0.4813787043094635
node_41: feature_name=cg22512847	feature_id[1152].value > threshold=0.5129324495792389
node_43: feature_name=cg23345038	feature_id[1147].value > threshold=0.6318148374557495
node_45: feature_name=cg17439009	feature_id[1815].value <= threshold=0.48514117300510406
node_46: feature_name=cg17537493	feature_id[2772].value > threshold=0.5825372636318207
node_52: feature_name=cg01683570	feature_id[110].value > threshold=0.6040188074111938
node_54: feature_name=cg15720017	feature_id[168].value > threshold=0.7067741453647614
node_60: feature_name=cg23690444	feature_id[120].value <= threshold=0.16037724912166595
node_61: feature_name=cg00567872	feature_id[236].value > threshold=0.7005765736103058

node_85: feature_name=cg01014262	feature_id[764].value <= threshold=0.5737917125225067
node_86: feature_name=cg22946562	feature_id[1148].value > threshold=0.6328203976154327
node_134: feature_name=cg14514032	feature_id[202].value <= threshold=0.4389604330062866
node_135: feature_name=cg13488220	feature_id[241].value > threshold=0.7352153658866882
node_143: feature_name=cg03840920	feature_id[256].value > threshold=0.502630889415741
node_147: feature_name=cg13634090	feature_id[822].value > threshold=0.7207068204879761
node_151: feature_name=cg19584674	feature_id[2364].value <= threshold=0.41758519411087036
node_152: feature_name=cg07344990	feature_id[411].value > threshold=0.5484964847564697
node_158: feature_name=cg10298992	feature_id[3066].value <= threshold=0.5707973837852478
node_159: feature_name=cg24852135	feature_id[47].value <= threshold=0.8445721566677094
node_160: feature_name=cg25606046	feature_id[2007].value <= threshold=0.8722946643829346
node_161: feature_name=cg03212620	feature_id[1188].value <= threshold=0.4703744798898697
Class: sarcoma (SARC)	
Rules_102	passed counts:2
node_0: feature_name=cg11915444	feature_id[1171].value > threshold=0.3402601182460785
node_10: feature_name=cg12109728	feature_id[2810].value > threshold=0.7068270146846771
node_20: feature_name=cg16412000	feature_id[789].value > threshold=0.6741803884506226
node_24: feature_name=cg17115147	feature_id[1434].value <= threshold=0.2961975038051605
node_25: feature_name=cg24474622	feature_id[2737].value > threshold=0.7211934924125671
node_29: feature_name=cg10472711	feature_id[612].value > threshold=0.45985929667949677
node_37: feature_name=cg01937669	feature_id[188].value <= threshold=0.4809463769197464
node_38: feature_name=cg02157052	feature_id[451].value <= threshold=0.2869153320789337

node_39: feature_name=cg18751958	feature_id[1427].value > threshold=0.4813787043094635
node_41: feature_name=cg22512847	feature_id[1152].value > threshold=0.5129324495792389
node_43: feature_name=cg23345038	feature_id[1147].value > threshold=0.6318148374557495
node_45: feature_name=cg17439009	feature_id[1815].value <= threshold=0.48514117300510406
node_46: feature_name=cg17537493	feature_id[2772].value > threshold=0.5825372636318207
node_52: feature_name=cg01683570	feature_id[110].value > threshold=0.6040188074111938
node_54: feature_name=cg15720017	feature_id[168].value > threshold=0.7067741453647614
node_60: feature_name=cg23690444	feature_id[120].value <= threshold=0.16037724912166595
node_61: feature_name=cg00567872	feature_id[236].value > threshold=0.7005765736103058
node_85: feature_name=cg01014262	feature_id[764].value <= threshold=0.5737917125225067
node_86: feature_name=cg22946562	feature_id[1148].value > threshold=0.6328203976154327
node_134: feature_name=cg14514032	feature_id[202].value <= threshold=0.4389604330062866
node_135: feature_name=cg13488220	feature_id[241].value > threshold=0.7352153658866882
node_143: feature_name=cg03840920	feature_id[256].value > threshold=0.502630889415741
node_147: feature_name=cg13634090	feature_id[822].value > threshold=0.7207068204879761
node_151: feature_name=cg19584674	feature_id[2364].value <= threshold=0.41758519411087036
node_152: feature_name=cg07344990	feature_id[411].value <= threshold=0.5484964847564697
node_153: feature_name=cg18369990	feature_id[2197].value <= threshold=0.621816486120224
node_154: feature_name=cg14560240	feature_id[2397].value > threshold=0.538348525762558
Class: mesenchymal chondrosarcoma (CSA (MES))	
Rules_103	passed counts:2

node_0: feature_name=cg11915444	feature_id[1171].value > threshold=0.3402601182460785
node_10: feature_name=cg12109728	feature_id[2810].value > threshold=0.7068270146846771
node_20: feature_name=cg16412000	feature_id[789].value > threshold=0.6741803884506226
node_24: feature_name=cg17115147	feature_id[1434].value <= threshold=0.2961975038051605
node_25: feature_name=cg24474622	feature_id[2737].value > threshold=0.7211934924125671
node_29: feature_name=cg10472711	feature_id[612].value > threshold=0.45985929667949677
node_37: feature_name=cg01937669	feature_id[188].value <= threshold=0.4809463769197464
node_38: feature_name=cg02157052	feature_id[451].value <= threshold=0.2869153320789337
node_39: feature_name=cg18751958	feature_id[1427].value > threshold=0.4813787043094635
node_41: feature_name=cg22512847	feature_id[1152].value > threshold=0.5129324495792389
node_43: feature_name=cg23345038	feature_id[1147].value > threshold=0.6318148374557495
node_45: feature_name=cg17439009	feature_id[1815].value <= threshold=0.48514117300510406
node_46: feature_name=cg17537493	feature_id[2772].value > threshold=0.5825372636318207
node_52: feature_name=cg01683570	feature_id[110].value > threshold=0.6040188074111938
node_54: feature_name=cg15720017	feature_id[168].value > threshold=0.7067741453647614
node_60: feature_name=cg23690444	feature_id[120].value <= threshold=0.16037724912166595
node_61: feature_name=cg00567872	feature_id[236].value > threshold=0.7005765736103058
node_85: feature_name=cg01014262	feature_id[764].value <= threshold=0.5737917125225067
node_86: feature_name=cg22946562	feature_id[1148].value <= threshold=0.6328203976154327
node_87: feature_name=cg18120975	feature_id[2767].value > threshold=0.7081544995307922
node_91: feature_name=cg17171539	feature_id[25].value > threshold=0.501024603843689
node_111: feature_name=cg21241424	feature_id[1155].value <= threshold=0.7665272355079651

node_112: feature_name=cg21897315	feature_id[3800].value > threshold=0.48165470361709595
node_114: feature_name=cg04375492	feature_id[2874].value > threshold=0.7812598049640656
node_116: feature_name=cg09892984	feature_id[3615].value > threshold=0.6039115190505981
node_118: feature_name=cg05076082	feature_id[215].value > threshold=0.7612724006175995
node_120: feature_name=cg15776300	feature_id[3872].value <= threshold=0.922246515750885
node_121: feature_name=cg12109728	feature_id[2810].value <= threshold=0.8809287846088409
Class: malignant peripheral nerve sheath tumour (MPNST)	
Rules_104	passed counts:2
node_0: feature_name=cg11915444	feature_id[1171].value > threshold=0.3402601182460785
node_10: feature_name=cg12109728	feature_id[2810].value > threshold=0.7068270146846771
node_20: feature_name=cg16412000	feature_id[789].value > threshold=0.6741803884506226
node_24: feature_name=cg17115147	feature_id[1434].value <= threshold=0.2961975038051605
node_25: feature_name=cg24474622	feature_id[2737].value > threshold=0.7211934924125671
node_29: feature_name=cg10472711	feature_id[612].value > threshold=0.45985929667949677
node_37: feature_name=cg01937669	feature_id[188].value <= threshold=0.4809463769197464
node_38: feature_name=cg02157052	feature_id[451].value <= threshold=0.2869153320789337
node_39: feature_name=cg18751958	feature_id[1427].value > threshold=0.4813787043094635
node_41: feature_name=cg22512847	feature_id[1152].value > threshold=0.5129324495792389
node_43: feature_name=cg23345038	feature_id[1147].value > threshold=0.6318148374557495
node_45: feature_name=cg17439009	feature_id[1815].value <= threshold=0.48514117300510406
node_46: feature_name=cg17537493	feature_id[2772].value > threshold=0.5825372636318207

node_52: feature_name=cg01683570	feature_id[110].value > threshold=0.6040188074111938
node_54: feature_name=cg15720017	feature_id[168].value > threshold=0.7067741453647614
node_60: feature_name=cg23690444	feature_id[120].value <= threshold=0.16037724912166595
node_61: feature_name=cg00567872	feature_id[236].value > threshold=0.7005765736103058
node_85: feature_name=cg01014262	feature_id[764].value <= threshold=0.5737917125225067
node_86: feature_name=cg22946562	feature_id[1148].value <= threshold=0.6328203976154327
node_87: feature_name=cg18120975	feature_id[2767].value > threshold=0.7081544995307922
node_91: feature_name=cg17171539	feature_id[25].value <= threshold=0.501024603843689
node_92: feature_name=cg00007036	feature_id[1586].value > threshold=0.943472146987915
node_104: feature_name=cg14156441	feature_id[3273].value > threshold=0.24960360676050186
Class: epithelioid sarcoma (ES)	
Rules_105	passed counts:2
node_0: feature_name=cg11915444	feature_id[1171].value > threshold=0.3402601182460785
node_10: feature_name=cg12109728	feature_id[2810].value > threshold=0.7068270146846771
node_20: feature_name=cg16412000	feature_id[789].value > threshold=0.6741803884506226
node_24: feature_name=cg17115147	feature_id[1434].value <= threshold=0.2961975038051605
node_25: feature_name=cg24474622	feature_id[2737].value > threshold=0.7211934924125671
node_29: feature_name=cg10472711	feature_id[612].value > threshold=0.45985929667949677
node_37: feature_name=cg01937669	feature_id[188].value <= threshold=0.4809463769197464
node_38: feature_name=cg02157052	feature_id[451].value <= threshold=0.2869153320789337
node_39: feature_name=cg18751958	feature_id[1427].value > threshold=0.4813787043094635
node_41: feature_name=cg22512847	feature_id[1152].value > threshold=0.5129324495792389

node_43: feature_name=cg23345038	feature_id[1147].value > threshold=0.6318148374557495
node_45: feature_name=cg17439009	feature_id[1815].value <= threshold=0.48514117300510406
node_46: feature_name=cg17537493	feature_id[2772].value > threshold=0.5825372636318207
node_52: feature_name=cg01683570	feature_id[110].value > threshold=0.6040188074111938
node_54: feature_name=cg15720017	feature_id[168].value > threshold=0.7067741453647614
node_60: feature_name=cg23690444	feature_id[120].value <= threshold=0.16037724912166595
node_61: feature_name=cg00567872	feature_id[236].value > threshold=0.7005765736103058
node_85: feature_name=cg01014262	feature_id[764].value <= threshold=0.5737917125225067
node_86: feature_name=cg22946562	feature_id[1148].value <= threshold=0.6328203976154327
node_87: feature_name=cg18120975	feature_id[2767].value > threshold=0.7081544995307922
node_91: feature_name=cg17171539	feature_id[25].value <= threshold=0.501024603843689
node_92: feature_name=cg00007036	feature_id[1586].value <= threshold=0.943472146987915
node_93: feature_name=cg16570314	feature_id[479].value <= threshold=0.7333994507789612
node_94: feature_name=cg15117891	feature_id[2220].value > threshold=0.9664478302001953
Class: leiomyosarcoma (LMS)	
Rules_106	passed counts:2
node_0: feature_name=cg11915444	feature_id[1171].value > threshold=0.3402601182460785
node_10: feature_name=cg12109728	feature_id[2810].value > threshold=0.7068270146846771
node_20: feature_name=cg16412000	feature_id[789].value > threshold=0.6741803884506226
node_24: feature_name=cg17115147	feature_id[1434].value <= threshold=0.2961975038051605
node_25: feature_name=cg24474622	feature_id[2737].value > threshold=0.7211934924125671
node_29: feature_name=cg10472711	feature_id[612].value > threshold=0.45985929667949677

node_37: feature_name=cg01937669	feature_id[188].value <= threshold=0.4809463769197464
node_38: feature_name=cg02157052	feature_id[451].value <= threshold=0.2869153320789337
node_39: feature_name=cg18751958	feature_id[1427].value > threshold=0.4813787043094635
node_41: feature_name=cg22512847	feature_id[1152].value > threshold=0.5129324495792389
node_43: feature_name=cg23345038	feature_id[1147].value > threshold=0.6318148374557495
node_45: feature_name=cg17439009	feature_id[1815].value <= threshold=0.48514117300510406
node_46: feature_name=cg17537493	feature_id[2772].value > threshold=0.5825372636318207
node_52: feature_name=cg01683570	feature_id[110].value > threshold=0.6040188074111938
node_54: feature_name=cg15720017	feature_id[168].value > threshold=0.7067741453647614
node_60: feature_name=cg23690444	feature_id[120].value <= threshold=0.16037724912166595
node_61: feature_name=cg00567872	feature_id[236].value > threshold=0.7005765736103058
node_85: feature_name=cg01014262	feature_id[764].value <= threshold=0.5737917125225067
node_86: feature_name=cg22946562	feature_id[1148].value <= threshold=0.6328203976154327
node_87: feature_name=cg18120975	feature_id[2767].value > threshold=0.7081544995307922
node_91: feature_name=cg17171539	feature_id[25].value <= threshold=0.501024603843689
node_92: feature_name=cg00007036	feature_id[1586].value <= threshold=0.943472146987915
node_93: feature_name=cg16570314	feature_id[479].value <= threshold=0.7333994507789612
node_94: feature_name=cg15117891	feature_id[2220].value <= threshold=0.9664478302001953
node_95: feature_name=cg00967711	feature_id[414].value <= threshold=0.22273091971874237
Class: cutaneous squamous cell carcinoma (SCC (CUT))	
Rules_107	passed counts:2

node_0: feature_name=cg11915444	feature_id[1171].value > threshold=0.3402601182460785
node_10: feature_name=cg12109728	feature_id[2810].value > threshold=0.7068270146846771
node_20: feature_name=cg16412000	feature_id[789].value > threshold=0.6741803884506226
node_24: feature_name=cg17115147	feature_id[1434].value <= threshold=0.2961975038051605
node_25: feature_name=cg24474622	feature_id[2737].value > threshold=0.7211934924125671
node_29: feature_name=cg10472711	feature_id[612].value <= threshold=0.45985929667949677
node_30: feature_name=cg06132803	feature_id[3655].value <= threshold=0.420499712228775
node_31: feature_name=cg04248332	feature_id[3387].value > threshold=0.6364206075668335
Class: leiomyosarcoma (LMS)	
Rules_108	passed counts:2
node_0: feature_name=cg11915444	feature_id[1171].value > threshold=0.3402601182460785
node_10: feature_name=cg12109728	feature_id[2810].value <= threshold=0.7068270146846771
node_11: feature_name=cg23902076	feature_id[1902].value > threshold=0.8691235482692719
node_13: feature_name=cg17174566	feature_id[2578].value > threshold=0.8555663526058197
Class: synovial sarcoma (SYSA)	
Rules_109	passed counts:2
node_0: feature_name=cg11915444	feature_id[1171].value <= threshold=0.3402601182460785
node_1: feature_name=cg00204976	feature_id[3433].value > threshold=0.7535275816917419
node_3: feature_name=cg22663660	feature_id[38].value <= threshold=0.5185286104679108
Class: alveolar rhabdomyosarcoma (RMS (ALV))	
Rules_110	passed counts:1
node_0: feature_name=cg11915444	feature_id[1171].value > threshold=0.3402601182460785

node_10: feature_name=cg12109728	feature_id[2810].value > threshold=0.7068270146846771
node_20: feature_name=cg16412000	feature_id[789].value > threshold=0.6741803884506226
node_24: feature_name=cg17115147	feature_id[1434].value > threshold=0.2961975038051605
node_368: feature_name=cg27543578	feature_id[878].value > threshold=0.8884280920028687
node_372: feature_name=cg11400707	feature_id[1940].value <= threshold=0.6575311124324799
node_373: feature_name=cg03042971	feature_id[1579].value > threshold=0.6336731016635895
node_375: feature_name=cg26207909	feature_id[3746].value > threshold=0.9737023711204529
Class: undifferentiated sarcoma (USARC)	
Rules_111	passed counts:1
node_0: feature_name=cg11915444	feature_id[1171].value > threshold=0.3402601182460785
node_10: feature_name=cg12109728	feature_id[2810].value > threshold=0.7068270146846771
node_20: feature_name=cg16412000	feature_id[789].value > threshold=0.6741803884506226
node_24: feature_name=cg17115147	feature_id[1434].value > threshold=0.2961975038051605
node_368: feature_name=cg27543578	feature_id[878].value > threshold=0.8884280920028687
node_372: feature_name=cg11400707	feature_id[1940].value <= threshold=0.6575311124324799
node_373: feature_name=cg03042971	feature_id[1579].value > threshold=0.6336731016635895
node_375: feature_name=cg26207909	feature_id[3746].value <= threshold=0.9737023711204529
Class: chondrosarcoma (CSA)	
Rules_112	passed counts:1
node_0: feature_name=cg11915444	feature_id[1171].value > threshold=0.3402601182460785
node_10: feature_name=cg12109728	feature_id[2810].value > threshold=0.7068270146846771
node_20: feature_name=cg16412000	feature_id[789].value > threshold=0.6741803884506226

node_24: feature_name=cg17115147	feature_id[1434].value > threshold=0.2961975038051605
node_368: feature_name=cg27543578	feature_id[878].value <= threshold=0.8884280920028687
node_369: feature_name=cg00133595	feature_id[2504].value > threshold=0.6530520617961884
Class: epithelioid sarcoma (ES)	
Rules_113	passed counts:1
node_0: feature_name=cg11915444	feature_id[1171].value > threshold=0.3402601182460785
node_10: feature_name=cg12109728	feature_id[2810].value > threshold=0.7068270146846771
node_20: feature_name=cg16412000	feature_id[789].value > threshold=0.6741803884506226
node_24: feature_name=cg17115147	feature_id[1434].value <= threshold=0.2961975038051605
node_25: feature_name=cg24474622	feature_id[2737].value > threshold=0.7211934924125671
node_29: feature_name=cg10472711	feature_id[612].value > threshold=0.45985929667949677
node_37: feature_name=cg01937669	feature_id[188].value > threshold=0.4809463769197464
node_363: feature_name=cg14588828	feature_id[2222].value <= threshold=0.9071650803089142
node_364: feature_name=cg24881558	feature_id[3465].value > threshold=0.7283148765563965
Class: giant cell tumour of bone (GCTB)	
Rules_114	passed counts:1
node_0: feature_name=cg11915444	feature_id[1171].value > threshold=0.3402601182460785
node_10: feature_name=cg12109728	feature_id[2810].value > threshold=0.7068270146846771
node_20: feature_name=cg16412000	feature_id[789].value > threshold=0.6741803884506226
node_24: feature_name=cg17115147	feature_id[1434].value <= threshold=0.2961975038051605
node_25: feature_name=cg24474622	feature_id[2737].value > threshold=0.7211934924125671
node_29: feature_name=cg10472711	feature_id[612].value > threshold=0.45985929667949677

node_37: feature_name=cg01937669	feature_id[188].value <= threshold=0.4809463769197464
node_38: feature_name=cg02157052	feature_id[451].value > threshold=0.2869153320789337
node_344: feature_name=cg21189849	feature_id[189].value > threshold=0.7746135592460632
node_348: feature_name=cg06937205	feature_id[751].value > threshold=0.4331420361995697
node_350: feature_name=cg20165037	feature_id[2558].value > threshold=0.8168091177940369
node_352: feature_name=cg17454920	feature_id[3003].value > threshold=0.5224908739328384
node_354: feature_name=cg25878830	feature_id[1496].value > threshold=0.4498005360364914
node_356: feature_name=cg26070874	feature_id[1040].value <= threshold=0.8498157262802124
node_357: feature_name=cg19730379	feature_id[3228].value > threshold=0.3024083226919174
node_359: feature_name=cg09647352	feature_id[2824].value > threshold=0.8696425259113312
Class: melanoma (MEL)	
Rules_115	passed counts:1
node_0: feature_name=cg11915444	feature_id[1171].value > threshold=0.3402601182460785
node_10: feature_name=cg12109728	feature_id[2810].value > threshold=0.7068270146846771
node_20: feature_name=cg16412000	feature_id[789].value > threshold=0.6741803884506226
node_24: feature_name=cg17115147	feature_id[1434].value <= threshold=0.2961975038051605
node_25: feature_name=cg24474622	feature_id[2737].value > threshold=0.7211934924125671
node_29: feature_name=cg10472711	feature_id[612].value > threshold=0.45985929667949677
node_37: feature_name=cg01937669	feature_id[188].value <= threshold=0.4809463769197464
node_38: feature_name=cg02157052	feature_id[451].value > threshold=0.2869153320789337
node_344: feature_name=cg21189849	feature_id[189].value > threshold=0.7746135592460632
node_348: feature_name=cg06937205	feature_id[751].value > threshold=0.4331420361995697

node_350: feature_name=cg20165037	feature_id[2558].value > threshold=0.8168091177940369
node_352: feature_name=cg17454920	feature_id[3003].value > threshold=0.5224908739328384
node_354: feature_name=cg25878830	feature_id[1496].value > threshold=0.4498005360364914
node_356: feature_name=cg26070874	feature_id[1040].value <= threshold=0.8498157262802124
node_357: feature_name=cg19730379	feature_id[3228].value > threshold=0.3024083226919174
node_359: feature_name=cg09647352	feature_id[2824].value <= threshold=0.8696425259113312
Class: malignant peripheral nerve sheath tumour (MPNST)	
Rules_116	passed counts:1
node_0: feature_name=cg11915444	feature_id[1171].value > threshold=0.3402601182460785
node_10: feature_name=cg12109728	feature_id[2810].value > threshold=0.7068270146846771
node_20: feature_name=cg16412000	feature_id[789].value > threshold=0.6741803884506226
node_24: feature_name=cg17115147	feature_id[1434].value <= threshold=0.2961975038051605
node_25: feature_name=cg24474622	feature_id[2737].value > threshold=0.7211934924125671
node_29: feature_name=cg10472711	feature_id[612].value > threshold=0.45985929667949677
node_37: feature_name=cg01937669	feature_id[188].value <= threshold=0.4809463769197464
node_38: feature_name=cg02157052	feature_id[451].value > threshold=0.2869153320789337
node_344: feature_name=cg21189849	feature_id[189].value <= threshold=0.7746135592460632
node_345: feature_name=cg00680875	feature_id[833].value <= threshold=0.352913498878479
Class: clear cell chondrosarcoma (CSA (CC))	
Rules_117	passed counts:1
node_0: feature_name=cg11915444	feature_id[1171].value > threshold=0.3402601182460785
node_10: feature_name=cg12109728	feature_id[2810].value > threshold=0.7068270146846771

node_20: feature_name=cg16412000	feature_id[789].value > threshold=0.6741803884506226
node_24: feature_name=cg17115147	feature_id[1434].value <= threshold=0.2961975038051605
node_25: feature_name=cg24474622	feature_id[2737].value > threshold=0.7211934924125671
node_29: feature_name=cg10472711	feature_id[612].value > threshold=0.45985929667949677
node_37: feature_name=cg01937669	feature_id[188].value <= threshold=0.4809463769197464
node_38: feature_name=cg02157052	feature_id[451].value <= threshold=0.2869153320789337
node_39: feature_name=cg18751958	feature_id[1427].value > threshold=0.4813787043094635
node_41: feature_name=cg22512847	feature_id[1152].value > threshold=0.5129324495792389
node_43: feature_name=cg23345038	feature_id[1147].value > threshold=0.6318148374557495
node_45: feature_name=cg17439009	feature_id[1815].value > threshold=0.48514117300510406
node_337: feature_name=cg14005246	feature_id[69].value <= threshold=0.4851873368024826
node_338: feature_name=cg23181573	feature_id[3479].value > threshold=0.5930750370025635
node_340: feature_name=cg08775793	feature_id[265].value > threshold=0.8249524533748627
Class: malignant peripheral nerve sheath tumour (MPNST)	
Rules_118	passed counts:1
node_0: feature_name=cg11915444	feature_id[1171].value > threshold=0.3402601182460785
node_10: feature_name=cg12109728	feature_id[2810].value > threshold=0.7068270146846771
node_20: feature_name=cg16412000	feature_id[789].value > threshold=0.6741803884506226
node_24: feature_name=cg17115147	feature_id[1434].value <= threshold=0.2961975038051605
node_25: feature_name=cg24474622	feature_id[2737].value > threshold=0.7211934924125671
node_29: feature_name=cg10472711	feature_id[612].value > threshold=0.45985929667949677

node_37: feature_name=cg01937669	feature_id[188].value <= threshold=0.4809463769197464
node_38: feature_name=cg02157052	feature_id[451].value <= threshold=0.2869153320789337
node_39: feature_name=cg18751958	feature_id[1427].value > threshold=0.4813787043094635
node_41: feature_name=cg22512847	feature_id[1152].value > threshold=0.5129324495792389
node_43: feature_name=cg23345038	feature_id[1147].value > threshold=0.6318148374557495
node_45: feature_name=cg17439009	feature_id[1815].value > threshold=0.48514117300510406
node_337: feature_name=cg14005246	feature_id[69].value <= threshold=0.4851873368024826
node_338: feature_name=cg23181573	feature_id[3479].value > threshold=0.5930750370025635
node_340: feature_name=cg08775793	feature_id[265].value <= threshold=0.8249524533748627
Class: malignant rhabdoid tumour (MRT)	
Rules_119	passed counts:1
node_0: feature_name=cg11915444	feature_id[1171].value > threshold=0.3402601182460785
node_10: feature_name=cg12109728	feature_id[2810].value > threshold=0.7068270146846771
node_20: feature_name=cg16412000	feature_id[789].value > threshold=0.6741803884506226
node_24: feature_name=cg17115147	feature_id[1434].value <= threshold=0.2961975038051605
node_25: feature_name=cg24474622	feature_id[2737].value > threshold=0.7211934924125671
node_29: feature_name=cg10472711	feature_id[612].value > threshold=0.45985929667949677
node_37: feature_name=cg01937669	feature_id[188].value <= threshold=0.4809463769197464
node_38: feature_name=cg02157052	feature_id[451].value <= threshold=0.2869153320789337
node_39: feature_name=cg18751958	feature_id[1427].value > threshold=0.4813787043094635
node_41: feature_name=cg22512847	feature_id[1152].value > threshold=0.5129324495792389
node_43: feature_name=cg23345038	feature_id[1147].value > threshold=0.6318148374557495

node_45: feature_name=cg17439009	feature_id[1815].value > threshold=0.48514117300510406
node_337: feature_name=cg14005246	feature_id[69].value <= threshold=0.4851873368024826
node_338: feature_name=cg23181573	feature_id[3479].value <= threshold=0.5930750370025635
Class: mesenchymal chondrosarcoma (CSA (MES))	
Rules_120	passed counts:1
node_0: feature_name=cg11915444	feature_id[1171].value > threshold=0.3402601182460785
node_10: feature_name=cg12109728	feature_id[2810].value > threshold=0.7068270146846771
node_20: feature_name=cg16412000	feature_id[789].value > threshold=0.6741803884506226
node_24: feature_name=cg17115147	feature_id[1434].value <= threshold=0.2961975038051605
node_25: feature_name=cg24474622	feature_id[2737].value > threshold=0.7211934924125671
node_29: feature_name=cg10472711	feature_id[612].value > threshold=0.45985929667949677
node_37: feature_name=cg01937669	feature_id[188].value <= threshold=0.4809463769197464
node_38: feature_name=cg02157052	feature_id[451].value <= threshold=0.2869153320789337
node_39: feature_name=cg18751958	feature_id[1427].value > threshold=0.4813787043094635
node_41: feature_name=cg22512847	feature_id[1152].value > threshold=0.5129324495792389
node_43: feature_name=cg23345038	feature_id[1147].value > threshold=0.6318148374557495
node_45: feature_name=cg17439009	feature_id[1815].value <= threshold=0.48514117300510406
node_46: feature_name=cg17537493	feature_id[2772].value > threshold=0.5825372636318207
node_52: feature_name=cg01683570	feature_id[110].value > threshold=0.6040188074111938
node_54: feature_name=cg15720017	feature_id[168].value > threshold=0.7067741453647614
node_60: feature_name=cg23690444	feature_id[120].value <= threshold=0.16037724912166595

node_61: feature_name=cg00567872	feature_id[236].value > threshold=0.7005765736103058
node_85: feature_name=cg01014262	feature_id[764].value <= threshold=0.5737917125225067
node_86: feature_name=cg22946562	feature_id[1148].value > threshold=0.6328203976154327
node_134: feature_name=cg14514032	feature_id[202].value > threshold=0.4389604330062866
node_312: feature_name=cg21670987	feature_id[1509].value > threshold=0.32477423548698425
node_324: feature_name=cg10520924	feature_id[1943].value > threshold=0.8430010974407196
node_326: feature_name=cg15354065	feature_id[2790].value > threshold=0.7598327696323395
node_328: feature_name=cg22964758	feature_id[424].value > threshold=0.68940070271492
Class: high-grade endometrial stromal sarcoma (ESS (HG))	
Rules_121	passed counts:1
node_0: feature_name=cg11915444	feature_id[1171].value > threshold=0.3402601182460785
node_10: feature_name=cg12109728	feature_id[2810].value > threshold=0.7068270146846771
node_20: feature_name=cg16412000	feature_id[789].value > threshold=0.6741803884506226
node_24: feature_name=cg17115147	feature_id[1434].value <= threshold=0.2961975038051605
node_25: feature_name=cg24474622	feature_id[2737].value > threshold=0.7211934924125671
node_29: feature_name=cg10472711	feature_id[612].value > threshold=0.45985929667949677
node_37: feature_name=cg01937669	feature_id[188].value <= threshold=0.4809463769197464
node_38: feature_name=cg02157052	feature_id[451].value <= threshold=0.2869153320789337
node_39: feature_name=cg18751958	feature_id[1427].value > threshold=0.4813787043094635
node_41: feature_name=cg22512847	feature_id[1152].value > threshold=0.5129324495792389
node_43: feature_name=cg23345038	feature_id[1147].value > threshold=0.6318148374557495

node_45: feature_name=cg17439009	feature_id[1815].value <= threshold=0.48514117300510406
node_46: feature_name=cg17537493	feature_id[2772].value > threshold=0.5825372636318207
node_52: feature_name=cg01683570	feature_id[110].value > threshold=0.6040188074111938
node_54: feature_name=cg15720017	feature_id[168].value > threshold=0.7067741453647614
node_60: feature_name=cg23690444	feature_id[120].value <= threshold=0.16037724912166595
node_61: feature_name=cg00567872	feature_id[236].value > threshold=0.7005765736103058
node_85: feature_name=cg01014262	feature_id[764].value <= threshold=0.5737917125225067
node_86: feature_name=cg22946562	feature_id[1148].value > threshold=0.6328203976154327
node_134: feature_name=cg14514032	feature_id[202].value > threshold=0.4389604330062866
node_312: feature_name=cg21670987	feature_id[1509].value > threshold=0.32477423548698425
node_324: feature_name=cg10520924	feature_id[1943].value > threshold=0.8430010974407196
node_326: feature_name=cg15354065	feature_id[2790].value > threshold=0.7598327696323395
node_328: feature_name=cg22964758	feature_id[424].value <= threshold=0.68940070271492
Class: sarcoma (SARC)	
Rules_122	passed counts:1
node_0: feature_name=cg11915444	feature_id[1171].value > threshold=0.3402601182460785
node_10: feature_name=cg12109728	feature_id[2810].value > threshold=0.7068270146846771
node_20: feature_name=cg16412000	feature_id[789].value > threshold=0.6741803884506226
node_24: feature_name=cg17115147	feature_id[1434].value <= threshold=0.2961975038051605
node_25: feature_name=cg24474622	feature_id[2737].value > threshold=0.7211934924125671
node_29: feature_name=cg10472711	feature_id[612].value > threshold=0.45985929667949677
node_37: feature_name=cg01937669	feature_id[188].value <= threshold=0.4809463769197464

node_38: feature_name=cg02157052	feature_id[451].value <= threshold=0.2869153320789337
node_39: feature_name=cg18751958	feature_id[1427].value > threshold=0.4813787043094635
node_41: feature_name=cg22512847	feature_id[1152].value > threshold=0.5129324495792389
node_43: feature_name=cg23345038	feature_id[1147].value > threshold=0.6318148374557495
node_45: feature_name=cg17439009	feature_id[1815].value <= threshold=0.48514117300510406
node_46: feature_name=cg17537493	feature_id[2772].value > threshold=0.5825372636318207
node_52: feature_name=cg01683570	feature_id[110].value > threshold=0.6040188074111938
node_54: feature_name=cg15720017	feature_id[168].value > threshold=0.7067741453647614
node_60: feature_name=cg23690444	feature_id[120].value <= threshold=0.16037724912166595
node_61: feature_name=cg00567872	feature_id[236].value > threshold=0.7005765736103058
node_85: feature_name=cg01014262	feature_id[764].value <= threshold=0.5737917125225067
node_86: feature_name=cg22946562	feature_id[1148].value > threshold=0.6328203976154327
node_134: feature_name=cg14514032	feature_id[202].value > threshold=0.4389604330062866
node_312: feature_name=cg21670987	feature_id[1509].value > threshold=0.32477423548698425
node_324: feature_name=cg10520924	feature_id[1943].value > threshold=0.8430010974407196
node_326: feature_name=cg15354065	feature_id[2790].value <= threshold=0.7598327696323395
Class: embryonal rhabdomyosarcoma (RMS (EMB))	
Rules_123	passed counts:1
node_0: feature_name=cg11915444	feature_id[1171].value > threshold=0.3402601182460785
node_10: feature_name=cg12109728	feature_id[2810].value > threshold=0.7068270146846771
node_20: feature_name=cg16412000	feature_id[789].value > threshold=0.6741803884506226

node_24: feature_name=cg17115147	feature_id[1434].value <= threshold=0.2961975038051605
node_25: feature_name=cg24474622	feature_id[2737].value > threshold=0.7211934924125671
node_29: feature_name=cg10472711	feature_id[612].value > threshold=0.4598592967949677
node_37: feature_name=cg01937669	feature_id[188].value <= threshold=0.4809463769197464
node_38: feature_name=cg02157052	feature_id[451].value <= threshold=0.2869153320789337
node_39: feature_name=cg18751958	feature_id[1427].value > threshold=0.4813787043094635
node_41: feature_name=cg22512847	feature_id[1152].value > threshold=0.5129324495792389
node_43: feature_name=cg23345038	feature_id[1147].value > threshold=0.6318148374557495
node_45: feature_name=cg17439009	feature_id[1815].value <= threshold=0.48514117300510406
node_46: feature_name=cg17537493	feature_id[2772].value > threshold=0.5825372636318207
node_52: feature_name=cg01683570	feature_id[110].value > threshold=0.6040188074111938
node_54: feature_name=cg15720017	feature_id[168].value > threshold=0.7067741453647614
node_60: feature_name=cg23690444	feature_id[120].value <= threshold=0.16037724912166595
node_61: feature_name=cg00567872	feature_id[236].value > threshold=0.7005765736103058
node_85: feature_name=cg01014262	feature_id[764].value <= threshold=0.5737917125225067
node_86: feature_name=cg22946562	feature_id[1148].value > threshold=0.6328203976154327
node_134: feature_name=cg14514032	feature_id[202].value > threshold=0.4389604330062866
node_312: feature_name=cg21670987	feature_id[1509].value <= threshold=0.32477423548698425
node_313: feature_name=cg12939390	feature_id[143].value > threshold=0.23070813715457916
node_315: feature_name=cg10907727	feature_id[284].value > threshold=0.6596133708953857
node_317: feature_name=cg21241424	feature_id[1155].value <= threshold=0.7687841951847076
node_318: feature_name=cg19691659	feature_id[953].value <= threshold=0.6708230972290039

node_319: feature_name=cg04002063	feature_id[2129].value > threshold=0.8756260275840759
Class: sclerosing epithelioid fibrosarcoma(SEF)	
Rules_124	passed counts:1
node_0: feature_name=cg11915444	feature_id[1171].value > threshold=0.3402601182460785
node_10: feature_name=cg12109728	feature_id[2810].value > threshold=0.7068270146846771
node_20: feature_name=cg16412000	feature_id[789].value > threshold=0.6741803884506226
node_24: feature_name=cg17115147	feature_id[1434].value <= threshold=0.2961975038051605
node_25: feature_name=cg24474622	feature_id[2737].value > threshold=0.7211934924125671
node_29: feature_name=cg10472711	feature_id[612].value > threshold=0.4598529667949677
node_37: feature_name=cg01937669	feature_id[188].value <= threshold=0.4809463769197464
node_38: feature_name=cg02157052	feature_id[451].value <= threshold=0.2869153320789337
node_39: feature_name=cg18751958	feature_id[1427].value > threshold=0.4813787043094635
node_41: feature_name=cg22512847	feature_id[1152].value > threshold=0.5129324495792389
node_43: feature_name=cg23345038	feature_id[1147].value > threshold=0.6318148374557495
node_45: feature_name=cg17439009	feature_id[1815].value <= threshold=0.48514117300510406
node_46: feature_name=cg17537493	feature_id[2772].value > threshold=0.5825372636318207
node_52: feature_name=cg01683570	feature_id[110].value > threshold=0.6040188074111938
node_54: feature_name=cg15720017	feature_id[168].value > threshold=0.7067741453647614
node_60: feature_name=cg23690444	feature_id[120].value <= threshold=0.16037724912166595
node_61: feature_name=cg00567872	feature_id[236].value > threshold=0.7005765736103058
node_85: feature_name=cg01014262	feature_id[764].value <= threshold=0.5737917125225067
node_86: feature_name=cg22946562	feature_id[1148].value > threshold=0.6328203976154327

node_134: feature_name=cg14514032	feature_id[202].value <= threshold=0.4389604330062866
node_135: feature_name=cg13488220	feature_id[241].value > threshold=0.7352153658866882
node_143: feature_name=cg03840920	feature_id[256].value > threshold=0.502630889415741
node_147: feature_name=cg13634090	feature_id[822].value > threshold=0.7207068204879761
node_151: feature_name=cg19584674	feature_id[2364].value > threshold=0.41758519411087036
node_307: feature_name=cg07795968	feature_id[2451].value > threshold=0.7859841883182526
node_309: feature_name=cg12552626	feature_id[687].value <= threshold=0.893171489238739
Class: clear cell sarcoma of the kidney (CCSK)	
Rules_125	passed counts:1
node_0: feature_name=cg11915444	feature_id[1171].value > threshold=0.3402601182460785
node_10: feature_name=cg12109728	feature_id[2810].value > threshold=0.7068270146846771
node_20: feature_name=cg16412000	feature_id[789].value > threshold=0.6741803884506226
node_24: feature_name=cg17115147	feature_id[1434].value <= threshold=0.2961975038051605
node_25: feature_name=cg24474622	feature_id[2737].value > threshold=0.7211934924125671
node_29: feature_name=cg10472711	feature_id[612].value > threshold=0.45985929667949677
node_37: feature_name=cg01937669	feature_id[188].value <= threshold=0.4809463769197464
node_38: feature_name=cg02157052	feature_id[451].value <= threshold=0.2869153320789337
node_39: feature_name=cg18751958	feature_id[1427].value > threshold=0.4813787043094635
node_41: feature_name=cg22512847	feature_id[1152].value > threshold=0.5129324495792389
node_43: feature_name=cg23345038	feature_id[1147].value > threshold=0.6318148374557495
node_45: feature_name=cg17439009	feature_id[1815].value <= threshold=0.48514117300510406
node_46: feature_name=cg17537493	feature_id[2772].value > threshold=0.5825372636318207

node_52: feature_name=cg01683570	feature_id[110].value > threshold=0.6040188074111938
node_54: feature_name=cg15720017	feature_id[168].value > threshold=0.7067741453647614
node_60: feature_name=cg23690444	feature_id[120].value <= threshold=0.16037724912166595
node_61: feature_name=cg00567872	feature_id[236].value > threshold=0.7005765736103058
node_85: feature_name=cg01014262	feature_id[764].value <= threshold=0.5737917125225067
node_86: feature_name=cg22946562	feature_id[1148].value > threshold=0.6328203976154327
node_134: feature_name=cg14514032	feature_id[202].value <= threshold=0.4389604330062866
node_135: feature_name=cg13488220	feature_id[241].value > threshold=0.7352153658866882
node_143: feature_name=cg03840920	feature_id[256].value > threshold=0.502630889415741
node_147: feature_name=cg13634090	feature_id[822].value > threshold=0.7207068204879761
node_151: feature_name=cg19584674	feature_id[2364].value <= threshold=0.41758519411087036
node_152: feature_name=cg07344990	feature_id[411].value > threshold=0.5484964847564697
node_158: feature_name=cg10298992	feature_id[3066].value > threshold=0.5707973837852478
node_174: feature_name=cg11843516	feature_id[20].value <= threshold=0.5590928494930267
node_175: feature_name=cg05185926	feature_id[448].value > threshold=0.17318664491176605
node_177: feature_name=cg17009731	feature_id[1220].value > threshold=0.6666513085365295
node_179: feature_name=cg01380319	feature_id[515].value <= threshold=0.39251960813999176
node_180: feature_name=cg20700977	feature_id[303].value > threshold=0.8422040641307831
node_184: feature_name=cg24259291	feature_id[164].value > threshold=0.4872268736362457
node_188: feature_name=cg08097657	feature_id[67].value > threshold=0.3371318429708481
node_190: feature_name=cg07891483	feature_id[59].value > threshold=0.6211732923984528
node_194: feature_name=cg20122645	feature_id[275].value > threshold=0.6094755828380585

node_196: feature_name=cg04026354	feature_id[1763].value > threshold=0.4341808259487152
node_286: feature_name=cg25408950	feature_id[772].value > threshold=0.680083692073822
node_290: feature_name=cg15188939	feature_id[1626].value > threshold=0.6365727782249451
node_294: feature_name=cg00444740	feature_id[3167].value > threshold=0.5775478780269623
node_296: feature_name=cg03555203	feature_id[3682].value > threshold=0.8000460863113403
node_298: feature_name=cg26685539	feature_id[1788].value > threshold=0.8831613063812256
node_300: feature_name=cg02353448	feature_id[74].value > threshold=0.9122499525547028
node_302: feature_name=cg02707799	feature_id[3403].value > threshold=0.7753476798534393
Class: malignant rhabdoid tumour (MRT)	
Rules_126	passed counts:1
node_0: feature_name=cg11915444	feature_id[1171].value > threshold=0.3402601182460785
node_10: feature_name=cg12109728	feature_id[2810].value > threshold=0.7068270146846771
node_20: feature_name=cg16412000	feature_id[789].value > threshold=0.6741803884506226
node_24: feature_name=cg17115147	feature_id[1434].value <= threshold=0.2961975038051605
node_25: feature_name=cg24474622	feature_id[2737].value > threshold=0.7211934924125671
node_29: feature_name=cg10472711	feature_id[612].value > threshold=0.45985929667949677
node_37: feature_name=cg01937669	feature_id[188].value <= threshold=0.4809463769197464
node_38: feature_name=cg02157052	feature_id[451].value <= threshold=0.2869153320789337
node_39: feature_name=cg18751958	feature_id[1427].value > threshold=0.4813787043094635
node_41: feature_name=cg22512847	feature_id[1152].value > threshold=0.5129324495792389
node_43: feature_name=cg23345038	feature_id[1147].value > threshold=0.6318148374557495
node_45: feature_name=cg17439009	feature_id[1815].value <= threshold=0.48514117300510406

node_46: feature_name=cg17537493	feature_id[2772].value > threshold=0.5825372636318207
node_52: feature_name=cg01683570	feature_id[110].value > threshold=0.6040188074111938
node_54: feature_name=cg15720017	feature_id[168].value > threshold=0.7067741453647614
node_60: feature_name=cg23690444	feature_id[120].value <= threshold=0.16037724912166595
node_61: feature_name=cg00567872	feature_id[236].value > threshold=0.7005765736103058
node_85: feature_name=cg01014262	feature_id[764].value <= threshold=0.5737917125225067
node_86: feature_name=cg22946562	feature_id[1148].value > threshold=0.6328203976154327
node_134: feature_name=cg14514032	feature_id[202].value <= threshold=0.4389604330062866
node_135: feature_name=cg13488220	feature_id[241].value > threshold=0.7352153658866882
node_143: feature_name=cg03840920	feature_id[256].value > threshold=0.502630889415741
node_147: feature_name=cg13634090	feature_id[822].value > threshold=0.7207068204879761
node_151: feature_name=cg19584674	feature_id[2364].value <= threshold=0.41758519411087036
node_152: feature_name=cg07344990	feature_id[411].value > threshold=0.5484964847564697
node_158: feature_name=cg10298992	feature_id[3066].value > threshold=0.5707973837852478
node_174: feature_name=cg11843516	feature_id[20].value <= threshold=0.5590928494930267
node_175: feature_name=cg05185926	feature_id[448].value > threshold=0.17318664491176605
node_177: feature_name=cg17009731	feature_id[1220].value > threshold=0.6665130853652954
node_179: feature_name=cg01380319	feature_id[515].value <= threshold=0.39251960813999176
node_180: feature_name=cg20700977	feature_id[303].value > threshold=0.8422040641307831
node_184: feature_name=cg24259291	feature_id[164].value > threshold=0.4872268736362457
node_188: feature_name=cg08097657	feature_id[67].value > threshold=0.3371318429708481
node_190: feature_name=cg07891483	feature_id[59].value > threshold=0.6211732923984528

node_194: feature_name=cg20122645	feature_id[275].value > threshold=0.6094755828380585
node_196: feature_name=cg04026354	feature_id[1763].value > threshold=0.4341808259487152
node_286: feature_name=cg25408950	feature_id[772].value > threshold=0.680083692073822
node_290: feature_name=cg15188939	feature_id[1626].value > threshold=0.6365727782249451
node_294: feature_name=cg00444740	feature_id[3167].value > threshold=0.5775478780269623
node_296: feature_name=cg03555203	feature_id[3682].value > threshold=0.8000460863113403
node_298: feature_name=cg26685539	feature_id[1788].value > threshold=0.8831613063812256
node_300: feature_name=cg02353448	feature_id[74].value > threshold=0.9122499525547028
node_302: feature_name=cg02707799	feature_id[3403].value <= threshold=0.7753476798534393
Class: angiosarcoma (AS)	
Rules_127	passed counts:1
node_0: feature_name=cg11915444	feature_id[1171].value > threshold=0.3402601182460785
node_10: feature_name=cg12109728	feature_id[2810].value > threshold=0.7068270146846771
node_20: feature_name=cg16412000	feature_id[789].value > threshold=0.6741803884506226
node_24: feature_name=cg17115147	feature_id[1434].value <= threshold=0.2961975038051605
node_25: feature_name=cg24474622	feature_id[2737].value > threshold=0.7211934924125671
node_29: feature_name=cg10472711	feature_id[612].value > threshold=0.45985929667949677
node_37: feature_name=cg01937669	feature_id[188].value <= threshold=0.4809463769197464
node_38: feature_name=cg02157052	feature_id[451].value <= threshold=0.2869153320789337
node_39: feature_name=cg18751958	feature_id[1427].value > threshold=0.4813787043094635
node_41: feature_name=cg22512847	feature_id[1152].value > threshold=0.5129324495792389
node_43: feature_name=cg23345038	feature_id[1147].value > threshold=0.6318148374557495

node_45: feature_name=cg17439009	feature_id[1815].value <= threshold=0.48514117300510406
node_46: feature_name=cg17537493	feature_id[2772].value > threshold=0.5825372636318207
node_52: feature_name=cg01683570	feature_id[110].value > threshold=0.6040188074111938
node_54: feature_name=cg15720017	feature_id[168].value > threshold=0.7067741453647614
node_60: feature_name=cg23690444	feature_id[120].value <= threshold=0.16037724912166595
node_61: feature_name=cg00567872	feature_id[236].value > threshold=0.7005765736103058
node_85: feature_name=cg01014262	feature_id[764].value <= threshold=0.5737917125225067
node_86: feature_name=cg22946562	feature_id[1148].value > threshold=0.6328203976154327
node_134: feature_name=cg14514032	feature_id[202].value <= threshold=0.4389604330062866
node_135: feature_name=cg13488220	feature_id[241].value > threshold=0.7352153658866882
node_143: feature_name=cg03840920	feature_id[256].value > threshold=0.502630889415741
node_147: feature_name=cg13634090	feature_id[822].value > threshold=0.7207068204879761
node_151: feature_name=cg19584674	feature_id[2364].value <= threshold=0.41758519411087036
node_152: feature_name=cg07344990	feature_id[411].value > threshold=0.5484964847564697
node_158: feature_name=cg10298992	feature_id[3066].value > threshold=0.5707973837852478
node_174: feature_name=cg11843516	feature_id[20].value <= threshold=0.5590928494930267
node_175: feature_name=cg05185926	feature_id[448].value > threshold=0.17318664491176605
node_177: feature_name=cg17009731	feature_id[1220].value > threshold=0.6666513085365295
node_179: feature_name=cg01380319	feature_id[515].value <= threshold=0.39251960813999176
node_180: feature_name=cg20700977	feature_id[303].value > threshold=0.8422040641307831
node_184: feature_name=cg24259291	feature_id[164].value > threshold=0.4872268736362457
node_188: feature_name=cg08097657	feature_id[67].value > threshold=0.3371318429708481

node_190: feature_name=cg07891483	feature_id[59].value > threshold=0.6211732923984528
node_194: feature_name=cg20122645	feature_id[275].value > threshold=0.6094755828380585
node_196: feature_name=cg04026354	feature_id[1763].value > threshold=0.4341808259487152
node_286: feature_name=cg25408950	feature_id[772].value > threshold=0.680083692073822
node_290: feature_name=cg15188939	feature_id[1626].value <= threshold=0.6365727782249451
node_291: feature_name=cg23421128	feature_id[1104].value <= threshold=0.14253754168748856
Class: sarcoma (SARC)	
Rules_128	passed counts:1
node_0: feature_name=cg11915444	feature_id[1171].value > threshold=0.3402601182460785
node_10: feature_name=cg12109728	feature_id[2810].value > threshold=0.7068270146846771
node_20: feature_name=cg16412000	feature_id[789].value > threshold=0.6741803884506226
node_24: feature_name=cg17115147	feature_id[1434].value <= threshold=0.2961975038051605
node_25: feature_name=cg24474622	feature_id[2737].value > threshold=0.7211934924125671
node_29: feature_name=cg10472711	feature_id[612].value > threshold=0.45985929667949677
node_37: feature_name=cg01937669	feature_id[188].value <= threshold=0.4809463769197464
node_38: feature_name=cg02157052	feature_id[451].value <= threshold=0.2869153320789337
node_39: feature_name=cg18751958	feature_id[1427].value > threshold=0.4813787043094635
node_41: feature_name=cg22512847	feature_id[1152].value > threshold=0.5129324495792389
node_43: feature_name=cg23345038	feature_id[1147].value > threshold=0.6318148374557495
node_45: feature_name=cg17439009	feature_id[1815].value <= threshold=0.48514117300510406
node_46: feature_name=cg17537493	feature_id[2772].value > threshold=0.5825372636318207
node_52: feature_name=cg01683570	feature_id[110].value > threshold=0.6040188074111938

node_54: feature_name=cg15720017	feature_id[168].value > threshold=0.7067741453647614
node_60: feature_name=cg23690444	feature_id[120].value <= threshold=0.16037724912166595
node_61: feature_name=cg00567872	feature_id[236].value > threshold=0.7005765736103058
node_85: feature_name=cg01014262	feature_id[764].value <= threshold=0.5737917125225067
node_86: feature_name=cg22946562	feature_id[1148].value > threshold=0.6328203976154327
node_134: feature_name=cg14514032	feature_id[202].value <= threshold=0.4389604330062866
node_135: feature_name=cg13488220	feature_id[241].value > threshold=0.7352153658866882
node_143: feature_name=cg03840920	feature_id[256].value > threshold=0.502630889415741
node_147: feature_name=cg13634090	feature_id[822].value > threshold=0.7207068204879761
node_151: feature_name=cg19584674	feature_id[2364].value <= threshold=0.41758519411087036
node_152: feature_name=cg07344990	feature_id[411].value > threshold=0.5484964847564697
node_158: feature_name=cg10298992	feature_id[3066].value > threshold=0.5707973837852478
node_174: feature_name=cg11843516	feature_id[20].value <= threshold=0.5590928494930267
node_175: feature_name=cg05185926	feature_id[448].value > threshold=0.17318664491176605
node_177: feature_name=cg17009731	feature_id[1220].value > threshold=0.6665130853652954
node_179: feature_name=cg01380319	feature_id[515].value <= threshold=0.39251960813999176
node_180: feature_name=cg20700977	feature_id[303].value > threshold=0.8422040641307831
node_184: feature_name=cg24259291	feature_id[164].value > threshold=0.4872268736362457
node_188: feature_name=cg08097657	feature_id[67].value > threshold=0.3371318429708481
node_190: feature_name=cg07891483	feature_id[59].value > threshold=0.6211732923984528
node_194: feature_name=cg20122645	feature_id[275].value > threshold=0.6094755828380585
node_196: feature_name=cg04026354	feature_id[1763].value <= threshold=0.4341808259487152

node_197: feature_name=cg10149889	feature_id[1017].value > threshold=0.6150195300579071
node_201: feature_name=cg14890730	feature_id[337].value > threshold=0.4372602105140686
node_279: feature_name=cg27143703	feature_id[3174].value <= threshold=0.42068085074424744
node_280: feature_name=cg01856887	feature_id[1987].value > threshold=0.6295701563358307
node_282: feature_name=cg00503920	feature_id[3717].value > threshold=0.8432826399803162
Class: well differentiated liposarcoma (WDLS)/dedifferentiated liposarcoma (DDLS)	
Rules_129	passed counts:1
node_0: feature_name=cg11915444	feature_id[1171].value > threshold=0.3402601182460785
node_10: feature_name=cg12109728	feature_id[2810].value > threshold=0.7068270146846771
node_20: feature_name=cg16412000	feature_id[789].value > threshold=0.6741803884506226
node_24: feature_name=cg17115147	feature_id[1434].value <= threshold=0.2961975038051605
node_25: feature_name=cg24474622	feature_id[2737].value > threshold=0.7211934924125671
node_29: feature_name=cg10472711	feature_id[612].value > threshold=0.45985929667949677
node_37: feature_name=cg01937669	feature_id[188].value <= threshold=0.4809463769197464
node_38: feature_name=cg02157052	feature_id[451].value <= threshold=0.2869153320789337
node_39: feature_name=cg18751958	feature_id[1427].value > threshold=0.4813787043094635
node_41: feature_name=cg22512847	feature_id[1152].value > threshold=0.5129324495792389
node_43: feature_name=cg23345038	feature_id[1147].value > threshold=0.6318148374557495
node_45: feature_name=cg17439009	feature_id[1815].value <= threshold=0.48514117300510406
node_46: feature_name=cg17537493	feature_id[2772].value > threshold=0.5825372636318207
node_52: feature_name=cg01683570	feature_id[110].value > threshold=0.6040188074111938

node_54: feature_name=cg15720017	feature_id[168].value > threshold=0.7067741453647614
node_60: feature_name=cg23690444	feature_id[120].value <= threshold=0.16037724912166595
node_61: feature_name=cg00567872	feature_id[236].value > threshold=0.7005765736103058
node_85: feature_name=cg01014262	feature_id[764].value <= threshold=0.5737917125225067
node_86: feature_name=cg22946562	feature_id[1148].value > threshold=0.6328203976154327
node_134: feature_name=cg14514032	feature_id[202].value <= threshold=0.4389604330062866
node_135: feature_name=cg13488220	feature_id[241].value > threshold=0.7352153658866882
node_143: feature_name=cg03840920	feature_id[256].value > threshold=0.502630889415741
node_147: feature_name=cg13634090	feature_id[822].value > threshold=0.7207068204879761
node_151: feature_name=cg19584674	feature_id[2364].value <= threshold=0.41758519411087036
node_152: feature_name=cg07344990	feature_id[411].value > threshold=0.5484964847564697
node_158: feature_name=cg10298992	feature_id[3066].value > threshold=0.5707973837852478
node_174: feature_name=cg11843516	feature_id[20].value <= threshold=0.5590928494930267
node_175: feature_name=cg05185926	feature_id[448].value > threshold=0.17318664491176605
node_177: feature_name=cg17009731	feature_id[1220].value > threshold=0.6665130085365295
node_179: feature_name=cg01380319	feature_id[515].value <= threshold=0.39251960813999176
node_180: feature_name=cg20700977	feature_id[303].value > threshold=0.8422040641307831
node_184: feature_name=cg24259291	feature_id[164].value > threshold=0.4872268736362457
node_188: feature_name=cg08097657	feature_id[67].value > threshold=0.3371318429708481
node_190: feature_name=cg07891483	feature_id[59].value > threshold=0.6211732923984528
node_194: feature_name=cg20122645	feature_id[275].value > threshold=0.6094755828380585
node_196: feature_name=cg04026354	feature_id[1763].value <= threshold=0.4341808259487152

node_197: feature_name=cg10149889	feature_id[1017].value > threshold=0.6150195300579071
node_201: feature_name=cg14890730	feature_id[337].value > threshold=0.4372602105140686
node_279: feature_name=cg27143703	feature_id[3174].value <= threshold=0.42068085074424744
node_280: feature_name=cg01856887	feature_id[1987].value > threshold=0.6295701563358307
node_282: feature_name=cg00503920	feature_id[3717].value <= threshold=0.8432826399803162
Class: atypical fibroxanthoma (AFX)/pleomorphic dermal sarcoma (PDS)	
Rules_130	passed counts:1
node_0: feature_name=cg11915444	feature_id[1171].value > threshold=0.3402601182460785
node_10: feature_name=cg12109728	feature_id[2810].value > threshold=0.7068270146846771
node_20: feature_name=cg16412000	feature_id[789].value > threshold=0.6741803884506226
node_24: feature_name=cg17115147	feature_id[1434].value <= threshold=0.2961975038051605
node_25: feature_name=cg24474622	feature_id[2737].value > threshold=0.7211934924125671
node_29: feature_name=cg10472711	feature_id[612].value > threshold=0.45985929667949677
node_37: feature_name=cg01937669	feature_id[188].value <= threshold=0.4809463769197464
node_38: feature_name=cg02157052	feature_id[451].value <= threshold=0.2869153320789337
node_39: feature_name=cg18751958	feature_id[1427].value > threshold=0.4813787043094635
node_41: feature_name=cg22512847	feature_id[1152].value > threshold=0.5129324495792389
node_43: feature_name=cg23345038	feature_id[1147].value > threshold=0.6318148374557495
node_45: feature_name=cg17439009	feature_id[1815].value <= threshold=0.48514117300510406
node_46: feature_name=cg17537493	feature_id[2772].value > threshold=0.5825372636318207
node_52: feature_name=cg01683570	feature_id[110].value > threshold=0.6040188074111938

node_54: feature_name=cg15720017	feature_id[168].value > threshold=0.7067741453647614
node_60: feature_name=cg23690444	feature_id[120].value <= threshold=0.16037724912166595
node_61: feature_name=cg00567872	feature_id[236].value > threshold=0.7005765736103058
node_85: feature_name=cg01014262	feature_id[764].value <= threshold=0.5737917125225067
node_86: feature_name=cg22946562	feature_id[1148].value > threshold=0.6328203976154327
node_134: feature_name=cg14514032	feature_id[202].value <= threshold=0.4389604330062866
node_135: feature_name=cg13488220	feature_id[241].value > threshold=0.7352153658866882
node_143: feature_name=cg03840920	feature_id[256].value > threshold=0.502630889415741
node_147: feature_name=cg13634090	feature_id[822].value > threshold=0.7207068204879761
node_151: feature_name=cg19584674	feature_id[2364].value <= threshold=0.41758519411087036
node_152: feature_name=cg07344990	feature_id[411].value > threshold=0.5484964847564697
node_158: feature_name=cg10298992	feature_id[3066].value > threshold=0.5707973837852478
node_174: feature_name=cg11843516	feature_id[20].value <= threshold=0.5590928494930267
node_175: feature_name=cg05185926	feature_id[448].value > threshold=0.17318664491176605
node_177: feature_name=cg17009731	feature_id[1220].value > threshold=0.6665130085365295
node_179: feature_name=cg01380319	feature_id[515].value <= threshold=0.39251960813999176
node_180: feature_name=cg20700977	feature_id[303].value > threshold=0.8422040641307831
node_184: feature_name=cg24259291	feature_id[164].value > threshold=0.4872268736362457
node_188: feature_name=cg08097657	feature_id[67].value > threshold=0.3371318429708481
node_190: feature_name=cg07891483	feature_id[59].value > threshold=0.6211732923984528
node_194: feature_name=cg20122645	feature_id[275].value > threshold=0.6094755828380585
node_196: feature_name=cg04026354	feature_id[1763].value <= threshold=0.4341808259487152

node_197: feature_name=cg10149889	feature_id[1017].value > threshold=0.6150195300579071
node_201: feature_name=cg14890730	feature_id[337].value > threshold=0.4372602105140686
node_279: feature_name=cg27143703	feature_id[3174].value <= threshold=0.42068085074424744
node_280: feature_name=cg01856887	feature_id[1987].value <= threshold=0.6295701563358307
Class: fibrous dysplasia (FDY)	
Rules_131	passed counts:1
node_0: feature_name=cg11915444	feature_id[1171].value > threshold=0.3402601182460785
node_10: feature_name=cg12109728	feature_id[2810].value > threshold=0.7068270146846771
node_20: feature_name=cg16412000	feature_id[789].value > threshold=0.6741803884506226
node_24: feature_name=cg17115147	feature_id[1434].value <= threshold=0.2961975038051605
node_25: feature_name=cg24474622	feature_id[2737].value > threshold=0.7211934924125671
node_29: feature_name=cg10472711	feature_id[612].value > threshold=0.45985929667949677
node_37: feature_name=cg01937669	feature_id[188].value <= threshold=0.4809463769197464
node_38: feature_name=cg02157052	feature_id[451].value <= threshold=0.2869153320789337
node_39: feature_name=cg18751958	feature_id[1427].value > threshold=0.4813787043094635
node_41: feature_name=cg22512847	feature_id[1152].value > threshold=0.5129324495792389
node_43: feature_name=cg23345038	feature_id[1147].value > threshold=0.6318148374557495
node_45: feature_name=cg17439009	feature_id[1815].value <= threshold=0.48514117300510406
node_46: feature_name=cg17537493	feature_id[2772].value > threshold=0.5825372636318207
node_52: feature_name=cg01683570	feature_id[110].value > threshold=0.6040188074111938
node_54: feature_name=cg15720017	feature_id[168].value > threshold=0.7067741453647614
node_60: feature_name=cg23690444	feature_id[120].value <= threshold=0.16037724912166595

node_61: feature_name=cg00567872	feature_id[236].value > threshold=0.7005765736103058
node_85: feature_name=cg01014262	feature_id[764].value <= threshold=0.5737917125225067
node_86: feature_name=cg22946562	feature_id[1148].value > threshold=0.6328203976154327
node_134: feature_name=cg14514032	feature_id[202].value <= threshold=0.4389604330062866
node_135: feature_name=cg13488220	feature_id[241].value > threshold=0.7352153658866882
node_143: feature_name=cg03840920	feature_id[256].value > threshold=0.502630889415741
node_147: feature_name=cg13634090	feature_id[822].value > threshold=0.7207068204879761
node_151: feature_name=cg19584674	feature_id[2364].value <= threshold=0.41758519411087036
node_152: feature_name=cg07344990	feature_id[411].value > threshold=0.5484964847564697
node_158: feature_name=cg10298992	feature_id[3066].value > threshold=0.5707973837852478
node_174: feature_name=cg11843516	feature_id[20].value <= threshold=0.5590928494930267
node_175: feature_name=cg05185926	feature_id[448].value > threshold=0.17318664491176605
node_177: feature_name=cg17009731	feature_id[1220].value > threshold=0.6666513085365295
node_179: feature_name=cg01380319	feature_id[515].value <= threshold=0.39251960813999176
node_180: feature_name=cg20700977	feature_id[303].value > threshold=0.8422040641307831
node_184: feature_name=cg24259291	feature_id[164].value > threshold=0.4872268736362457
node_188: feature_name=cg08097657	feature_id[67].value > threshold=0.3371318429708481
node_190: feature_name=cg07891483	feature_id[59].value > threshold=0.6211732923984528
node_194: feature_name=cg20122645	feature_id[275].value > threshold=0.6094755828380585
node_196: feature_name=cg04026354	feature_id[1763].value <= threshold=0.4341808259487152
node_197: feature_name=cg10149889	feature_id[1017].value > threshold=0.6150195300579071
node_201: feature_name=cg14890730	feature_id[337].value <= threshold=0.4372602105140686

node_202: feature_name=cg08331427	feature_id[132].value > threshold=0.5443233847618103
node_208: feature_name=cg22730007	feature_id[2544].value > threshold=0.28619489073753357
node_210: feature_name=cg17193551	feature_id[2057].value > threshold=0.4393353909254074
node_212: feature_name=cg14671764	feature_id[584].value > threshold=0.9353199899196625
node_268: feature_name=cg21377260	feature_id[534].value <= threshold=0.9840706288814545
node_269: feature_name=cg27143326	feature_id[1403].value <= threshold=0.8949433267116547
node_270: feature_name=cg01339351	feature_id[2694].value <= threshold=0.8992902934551239
node_271: feature_name=cg03407184	feature_id[3141].value > threshold=0.7490815222263336
Class: Ewing sarcoma (EWING)	
Rules_132	passed counts:1
node_0: feature_name=cg11915444	feature_id[1171].value > threshold=0.3402601182460785
node_10: feature_name=cg12109728	feature_id[2810].value > threshold=0.7068270146846771
node_20: feature_name=cg16412000	feature_id[789].value > threshold=0.6741803884506226
node_24: feature_name=cg17115147	feature_id[1434].value <= threshold=0.2961975038051605
node_25: feature_name=cg24474622	feature_id[2737].value > threshold=0.7211934924125671
node_29: feature_name=cg10472711	feature_id[612].value > threshold=0.45985929667949677
node_37: feature_name=cg01937669	feature_id[188].value <= threshold=0.4809463769197464
node_38: feature_name=cg02157052	feature_id[451].value <= threshold=0.2869153320789337
node_39: feature_name=cg18751958	feature_id[1427].value > threshold=0.4813787043094635
node_41: feature_name=cg22512847	feature_id[1152].value > threshold=0.5129324495792389
node_43: feature_name=cg23345038	feature_id[1147].value > threshold=0.6318148374557495
node_45: feature_name=cg17439009	feature_id[1815].value <= threshold=0.48514117300510406

node_46: feature_name=cg17537493	feature_id[2772].value > threshold=0.5825372636318207
node_52: feature_name=cg01683570	feature_id[110].value > threshold=0.6040188074111938
node_54: feature_name=cg15720017	feature_id[168].value > threshold=0.7067741453647614
node_60: feature_name=cg23690444	feature_id[120].value <= threshold=0.16037724912166595
node_61: feature_name=cg00567872	feature_id[236].value > threshold=0.7005765736103058
node_85: feature_name=cg01014262	feature_id[764].value <= threshold=0.5737917125225067
node_86: feature_name=cg22946562	feature_id[1148].value > threshold=0.6328203976154327
node_134: feature_name=cg14514032	feature_id[202].value <= threshold=0.4389604330062866
node_135: feature_name=cg13488220	feature_id[241].value > threshold=0.7352153658866882
node_143: feature_name=cg03840920	feature_id[256].value > threshold=0.502630889415741
node_147: feature_name=cg13634090	feature_id[822].value > threshold=0.7207068204879761
node_151: feature_name=cg19584674	feature_id[2364].value <= threshold=0.41758519411087036
node_152: feature_name=cg07344990	feature_id[411].value > threshold=0.5484964847564697
node_158: feature_name=cg10298992	feature_id[3066].value > threshold=0.5707973837852478
node_174: feature_name=cg11843516	feature_id[20].value <= threshold=0.5590928494930267
node_175: feature_name=cg05185926	feature_id[448].value > threshold=0.17318664491176605
node_177: feature_name=cg17009731	feature_id[1220].value > threshold=0.6666513085365295
node_179: feature_name=cg01380319	feature_id[515].value <= threshold=0.39251960813999176
node_180: feature_name=cg20700977	feature_id[303].value > threshold=0.8422040641307831
node_184: feature_name=cg24259291	feature_id[164].value > threshold=0.4872268736362457
node_188: feature_name=cg08097657	feature_id[67].value > threshold=0.3371318429708481
node_190: feature_name=cg07891483	feature_id[59].value > threshold=0.6211732923984528

node_194: feature_name=cg20122645	feature_id[275].value > threshold=0.6094755828380585
node_196: feature_name=cg04026354	feature_id[1763].value <= threshold=0.4341808259487152
node_197: feature_name=cg10149889	feature_id[1017].value > threshold=0.6150195300579071
node_201: feature_name=cg14890730	feature_id[337].value <= threshold=0.4372602105140686
node_202: feature_name=cg08331427	feature_id[132].value > threshold=0.5443233847618103
node_208: feature_name=cg22730007	feature_id[2544].value > threshold=0.28619489073753357
node_210: feature_name=cg17193551	feature_id[2057].value > threshold=0.4393353909254074
node_212: feature_name=cg14671764	feature_id[584].value > threshold=0.9353199899196625
node_268: feature_name=cg21377260	feature_id[534].value <= threshold=0.9840706288814545
node_269: feature_name=cg27143326	feature_id[1403].value <= threshold=0.8949433267116547
node_270: feature_name=cg01339351	feature_id[2694].value <= threshold=0.8992902934551239
node_271: feature_name=cg03407184	feature_id[3141].value <= threshold=0.7490815222263336
Class: chondrosarcoma (CSA)	
Rules_133	passed counts:1
node_0: feature_name=cg11915444	feature_id[1171].value > threshold=0.3402601182460785
node_10: feature_name=cg12109728	feature_id[2810].value > threshold=0.7068270146846771
node_20: feature_name=cg16412000	feature_id[789].value > threshold=0.6741803884506226
node_24: feature_name=cg17115147	feature_id[1434].value <= threshold=0.2961975038051605
node_25: feature_name=cg24474622	feature_id[2737].value > threshold=0.7211934924125671
node_29: feature_name=cg10472711	feature_id[612].value > threshold=0.45985929667949677
node_37: feature_name=cg01937669	feature_id[188].value <= threshold=0.4809463769197464
node_38: feature_name=cg02157052	feature_id[451].value <= threshold=0.2869153320789337

node_39: feature_name=cg18751958	feature_id[1427].value > threshold=0.4813787043094635
node_41: feature_name=cg22512847	feature_id[1152].value > threshold=0.5129324495792389
node_43: feature_name=cg23345038	feature_id[1147].value > threshold=0.6318148374557495
node_45: feature_name=cg17439009	feature_id[1815].value <= threshold=0.48514117300510406
node_46: feature_name=cg17537493	feature_id[2772].value > threshold=0.5825372636318207
node_52: feature_name=cg01683570	feature_id[110].value > threshold=0.6040188074111938
node_54: feature_name=cg15720017	feature_id[168].value > threshold=0.7067741453647614
node_60: feature_name=cg23690444	feature_id[120].value <= threshold=0.16037724912166595
node_61: feature_name=cg00567872	feature_id[236].value > threshold=0.7005765736103058
node_85: feature_name=cg01014262	feature_id[764].value <= threshold=0.5737917125225067
node_86: feature_name=cg22946562	feature_id[1148].value > threshold=0.6328203976154327
node_134: feature_name=cg14514032	feature_id[202].value <= threshold=0.4389604330062866
node_135: feature_name=cg13488220	feature_id[241].value > threshold=0.7352153658866882
node_143: feature_name=cg03840920	feature_id[256].value > threshold=0.502630889415741
node_147: feature_name=cg13634090	feature_id[822].value > threshold=0.7207068204879761
node_151: feature_name=cg19584674	feature_id[2364].value <= threshold=0.41758519411087036
node_152: feature_name=cg07344990	feature_id[411].value > threshold=0.5484964847564697
node_158: feature_name=cg10298992	feature_id[3066].value > threshold=0.5707973837852478
node_174: feature_name=cg11843516	feature_id[20].value <= threshold=0.5590928494930267
node_175: feature_name=cg05185926	feature_id[448].value > threshold=0.17318664491176605
node_177: feature_name=cg17009731	feature_id[1220].value > threshold=0.6666513085365295
node_179: feature_name=cg01380319	feature_id[515].value <= threshold=0.39251960813999176

node_180: feature_name=cg20700977	feature_id[303].value > threshold=0.8422040641307831
node_184: feature_name=cg24259291	feature_id[164].value > threshold=0.4872268736362457
node_188: feature_name=cg08097657	feature_id[67].value > threshold=0.3371318429708481
node_190: feature_name=cg07891483	feature_id[59].value > threshold=0.6211732923984528
node_194: feature_name=cg20122645	feature_id[275].value > threshold=0.6094755828380585
node_196: feature_name=cg04026354	feature_id[1763].value <= threshold=0.4341808259487152
node_197: feature_name=cg10149889	feature_id[1017].value > threshold=0.6150195300579071
node_201: feature_name=cg14890730	feature_id[337].value <= threshold=0.4372602105140686
node_202: feature_name=cg08331427	feature_id[132].value > threshold=0.5443233847618103
node_208: feature_name=cg22730007	feature_id[2544].value > threshold=0.28619489073753357
node_210: feature_name=cg17193551	feature_id[2057].value > threshold=0.4393353909254074
node_212: feature_name=cg14671764	feature_id[584].value <= threshold=0.9353199899196625
node_213: feature_name=cg20140662	feature_id[2044].value > threshold=0.38030287623405457
node_215: feature_name=cg07891483	feature_id[59].value > threshold=0.829958975315094
node_219: feature_name=cg04884579	feature_id[1861].value > threshold=0.6106154918670654
node_229: feature_name=cg19548524	feature_id[2985].value > threshold=0.8623353838920593
node_233: feature_name=cg14788686	feature_id[3556].value > threshold=0.35090239346027374
node_235: feature_name=cg05380734	feature_id[2470].value <= threshold=0.7112160623073578
node_236: feature_name=cg01166827	feature_id[3708].value > threshold=0.6359498500823975
node_238: feature_name=cg13251842	feature_id[714].value > threshold=0.8916542232036591
node_240: feature_name=cg02829279	feature_id[623].value > threshold=0.450528159737587
node_242: feature_name=cg24358599	feature_id[86].value > threshold=0.8132582604885101

node_246: feature_name=cg05544807	feature_id[3661].value <= threshold=0.6726464331150055
node_247: feature_name=cg05056653	feature_id[3122].value > threshold=0.35445791482925415
node_249: feature_name=cg26308909	feature_id[770].value > threshold=0.3754204213619232
node_251: feature_name=cg11197908	feature_id[3058].value > threshold=0.36795753240585327
node_253: feature_name=cg00039385	feature_id[646].value <= threshold=0.18521490693092346
node_254: feature_name=cg04787317	feature_id[3379].value > threshold=0.8155552744865417
node_256: feature_name=cg00169184	feature_id[2503].value > threshold=0.9175277948379517
node_258: feature_name=cg11546709	feature_id[966].value > threshold=0.3400330990552902
node_260: feature_name=cg17276624	feature_id[1163].value > threshold=0.8622665405273438
node_262: feature_name=cg18628371	feature_id[3240].value > threshold=0.598172664642334
Class: high-grade endometrial stromal sarcoma (ESS (HG))	
Rules_134	passed counts:1
node_0: feature_name=cg11915444	feature_id[1171].value > threshold=0.3402601182460785
node_10: feature_name=cg12109728	feature_id[2810].value > threshold=0.7068270146846771
node_20: feature_name=cg16412000	feature_id[789].value > threshold=0.6741803884506226
node_24: feature_name=cg17115147	feature_id[1434].value <= threshold=0.2961975038051605
node_25: feature_name=cg24474622	feature_id[2737].value > threshold=0.7211934924125671
node_29: feature_name=cg10472711	feature_id[612].value > threshold=0.45985929667949677
node_37: feature_name=cg01937669	feature_id[188].value <= threshold=0.4809463769197464
node_38: feature_name=cg02157052	feature_id[451].value <= threshold=0.2869153320789337
node_39: feature_name=cg18751958	feature_id[1427].value > threshold=0.4813787043094635

node_41: feature_name=cg22512847	feature_id[1152].value > threshold=0.5129324495792389
node_43: feature_name=cg23345038	feature_id[1147].value > threshold=0.6318148374557495
node_45: feature_name=cg17439009	feature_id[1815].value <= threshold=0.48514117300510406
node_46: feature_name=cg17537493	feature_id[2772].value > threshold=0.5825372636318207
node_52: feature_name=cg01683570	feature_id[110].value > threshold=0.6040188074111938
node_54: feature_name=cg15720017	feature_id[168].value > threshold=0.7067741453647614
node_60: feature_name=cg23690444	feature_id[120].value <= threshold=0.16037724912166595
node_61: feature_name=cg00567872	feature_id[236].value > threshold=0.7005765736103058
node_85: feature_name=cg01014262	feature_id[764].value <= threshold=0.5737917125225067
node_86: feature_name=cg22946562	feature_id[1148].value > threshold=0.6328203976154327
node_134: feature_name=cg14514032	feature_id[202].value <= threshold=0.4389604330062866
node_135: feature_name=cg13488220	feature_id[241].value > threshold=0.7352153658866882
node_143: feature_name=cg03840920	feature_id[256].value > threshold=0.502630889415741
node_147: feature_name=cg13634090	feature_id[822].value > threshold=0.7207068204879761
node_151: feature_name=cg19584674	feature_id[2364].value <= threshold=0.41758519411087036
node_152: feature_name=cg07344990	feature_id[411].value > threshold=0.5484964847564697
node_158: feature_name=cg10298992	feature_id[3066].value > threshold=0.5707973837852478
node_174: feature_name=cg11843516	feature_id[20].value <= threshold=0.5590928494930267
node_175: feature_name=cg05185926	feature_id[448].value > threshold=0.17318664491176605
node_177: feature_name=cg17009731	feature_id[1220].value > threshold=0.6666513085365295
node_179: feature_name=cg01380319	feature_id[515].value <= threshold=0.39251960813999176
node_180: feature_name=cg20700977	feature_id[303].value > threshold=0.8422040641307831

node_184: feature_name=cg24259291	feature_id[164].value > threshold=0.4872268736362457
node_188: feature_name=cg08097657	feature_id[67].value > threshold=0.3371318429708481
node_190: feature_name=cg07891483	feature_id[59].value > threshold=0.6211732923984528
node_194: feature_name=cg20122645	feature_id[275].value > threshold=0.6094755828380585
node_196: feature_name=cg04026354	feature_id[1763].value <= threshold=0.4341808259487152
node_197: feature_name=cg10149889	feature_id[1017].value > threshold=0.6150195300579071
node_201: feature_name=cg14890730	feature_id[337].value <= threshold=0.4372602105140686
node_202: feature_name=cg08331427	feature_id[132].value > threshold=0.5443233847618103
node_208: feature_name=cg22730007	feature_id[2544].value > threshold=0.28619489073753357
node_210: feature_name=cg17193551	feature_id[2057].value > threshold=0.4393353909254074
node_212: feature_name=cg14671764	feature_id[584].value <= threshold=0.9353199899196625
node_213: feature_name=cg20140662	feature_id[2044].value > threshold=0.38030287623405457
node_215: feature_name=cg07891483	feature_id[59].value > threshold=0.829958975315094
node_219: feature_name=cg04884579	feature_id[1861].value > threshold=0.6106154918670654
node_229: feature_name=cg19548524	feature_id[2985].value > threshold=0.8623353838920593
node_233: feature_name=cg14788686	feature_id[3556].value > threshold=0.35090239346027374
node_235: feature_name=cg05380734	feature_id[2470].value <= threshold=0.7112160623073578
node_236: feature_name=cg01166827	feature_id[3708].value > threshold=0.6359498500823975
node_238: feature_name=cg13251842	feature_id[714].value > threshold=0.8916542232036591
node_240: feature_name=cg02829279	feature_id[623].value > threshold=0.450528159737587
node_242: feature_name=cg24358599	feature_id[86].value > threshold=0.8132582604885101
node_246: feature_name=cg05544807	feature_id[3661].value <= threshold=0.6726464331150055

node_247: feature_name=cg05056653	feature_id[3122].value > threshold=0.35445791482925415
node_249: feature_name=cg26308909	feature_id[770].value > threshold=0.3754204213619232
node_251: feature_name=cg11197908	feature_id[3058].value > threshold=0.36795753240585327
node_253: feature_name=cg00039385	feature_id[646].value <= threshold=0.18521490693092346
node_254: feature_name=cg04787317	feature_id[3379].value > threshold=0.8155552744865417
node_256: feature_name=cg00169184	feature_id[2503].value > threshold=0.9175277948379517
node_258: feature_name=cg11546709	feature_id[966].value > threshold=0.3400330990552902
node_260: feature_name=cg17276624	feature_id[1163].value > threshold=0.8622665405273438
node_262: feature_name=cg18628371	feature_id[3240].value <= threshold=0.598172664642334
Class: sarcoma (SARC)	
Rules_135	passed counts:1
node_0: feature_name=cg11915444	feature_id[1171].value > threshold=0.3402601182460785
node_10: feature_name=cg12109728	feature_id[2810].value > threshold=0.7068270146846771
node_20: feature_name=cg16412000	feature_id[789].value > threshold=0.6741803884506226
node_24: feature_name=cg17115147	feature_id[1434].value <= threshold=0.2961975038051605
node_25: feature_name=cg24474622	feature_id[2737].value > threshold=0.7211934924125671
node_29: feature_name=cg10472711	feature_id[612].value > threshold=0.45985929667949677
node_37: feature_name=cg01937669	feature_id[188].value <= threshold=0.4809463769197464
node_38: feature_name=cg02157052	feature_id[451].value <= threshold=0.2869153320789337
node_39: feature_name=cg18751958	feature_id[1427].value > threshold=0.4813787043094635
node_41: feature_name=cg22512847	feature_id[1152].value > threshold=0.5129324495792389
node_43: feature_name=cg23345038	feature_id[1147].value > threshold=0.6318148374557495

node_45: feature_name=cg17439009	feature_id[1815].value <= threshold=0.48514117300510406
node_46: feature_name=cg17537493	feature_id[2772].value > threshold=0.5825372636318207
node_52: feature_name=cg01683570	feature_id[110].value > threshold=0.6040188074111938
node_54: feature_name=cg15720017	feature_id[168].value > threshold=0.7067741453647614
node_60: feature_name=cg23690444	feature_id[120].value <= threshold=0.16037724912166595
node_61: feature_name=cg00567872	feature_id[236].value > threshold=0.7005765736103058
node_85: feature_name=cg01014262	feature_id[764].value <= threshold=0.5737917125225067
node_86: feature_name=cg22946562	feature_id[1148].value > threshold=0.6328203976154327
node_134: feature_name=cg14514032	feature_id[202].value <= threshold=0.4389604330062866
node_135: feature_name=cg13488220	feature_id[241].value > threshold=0.7352153658866882
node_143: feature_name=cg03840920	feature_id[256].value > threshold=0.502630889415741
node_147: feature_name=cg13634090	feature_id[822].value > threshold=0.7207068204879761
node_151: feature_name=cg19584674	feature_id[2364].value <= threshold=0.41758519411087036
node_152: feature_name=cg07344990	feature_id[411].value > threshold=0.5484964847564697
node_158: feature_name=cg10298992	feature_id[3066].value > threshold=0.5707973837852478
node_174: feature_name=cg11843516	feature_id[20].value <= threshold=0.5590928494930267
node_175: feature_name=cg05185926	feature_id[448].value > threshold=0.17318664491176605
node_177: feature_name=cg17009731	feature_id[1220].value > threshold=0.6666513085365295
node_179: feature_name=cg01380319	feature_id[515].value <= threshold=0.39251960813999176
node_180: feature_name=cg20700977	feature_id[303].value > threshold=0.8422040641307831
node_184: feature_name=cg24259291	feature_id[164].value > threshold=0.4872268736362457
node_188: feature_name=cg08097657	feature_id[67].value > threshold=0.3371318429708481

node_190: feature_name=cg07891483	feature_id[59].value > threshold=0.6211732923984528
node_194: feature_name=cg20122645	feature_id[275].value > threshold=0.6094755828380585
node_196: feature_name=cg04026354	feature_id[1763].value <= threshold=0.4341808259487152
node_197: feature_name=cg10149889	feature_id[1017].value > threshold=0.6150195300579071
node_201: feature_name=cg14890730	feature_id[337].value <= threshold=0.4372602105140686
node_202: feature_name=cg08331427	feature_id[132].value > threshold=0.5443233847618103
node_208: feature_name=cg22730007	feature_id[2544].value > threshold=0.28619489073753357
node_210: feature_name=cg17193551	feature_id[2057].value > threshold=0.4393353909254074
node_212: feature_name=cg14671764	feature_id[584].value <= threshold=0.9353199899196625
node_213: feature_name=cg20140662	feature_id[2044].value > threshold=0.38030287623405457
node_215: feature_name=cg07891483	feature_id[59].value > threshold=0.829958975315094
node_219: feature_name=cg04884579	feature_id[1861].value > threshold=0.6106154918670654
node_229: feature_name=cg19548524	feature_id[2985].value > threshold=0.8623353838920593
node_233: feature_name=cg14788686	feature_id[3556].value > threshold=0.35090239346027374
node_235: feature_name=cg05380734	feature_id[2470].value <= threshold=0.7112160623073578
node_236: feature_name=cg01166827	feature_id[3708].value > threshold=0.6359498500823975
node_238: feature_name=cg13251842	feature_id[714].value > threshold=0.8916542232036591
node_240: feature_name=cg02829279	feature_id[623].value > threshold=0.450528159737587
node_242: feature_name=cg24358599	feature_id[86].value > threshold=0.8132582604885101
node_246: feature_name=cg05544807	feature_id[3661].value <= threshold=0.6726464331150055
node_247: feature_name=cg05056653	feature_id[3122].value > threshold=0.35445791482925415
node_249: feature_name=cg26308909	feature_id[770].value > threshold=0.3754204213619232

node_251: feature_name=cg11197908	feature_id[3058].value > threshold=0.36795753240585327
node_253: feature_name=cg00039385	feature_id[646].value <= threshold=0.18521490693092346
node_254: feature_name=cg04787317	feature_id[3379].value > threshold=0.815552744865417
node_256: feature_name=cg00169184	feature_id[2503].value > threshold=0.9175277948379517
node_258: feature_name=cg11546709	feature_id[966].value > threshold=0.3400330990552902
node_260: feature_name=cg17276624	feature_id[1163].value <= threshold=0.8622665405273438
Class: epithelioid sarcoma (ES)	
Rules_136	passed counts:1
node_0: feature_name=cg11915444	feature_id[1171].value > threshold=0.3402601182460785
node_10: feature_name=cg12109728	feature_id[2810].value > threshold=0.7068270146846771
node_20: feature_name=cg16412000	feature_id[789].value > threshold=0.6741803884506226
node_24: feature_name=cg17115147	feature_id[1434].value <= threshold=0.2961975038051605
node_25: feature_name=cg24474622	feature_id[2737].value > threshold=0.7211934924125671
node_29: feature_name=cg10472711	feature_id[612].value > threshold=0.45985929667949677
node_37: feature_name=cg01937669	feature_id[188].value <= threshold=0.4809463769197464
node_38: feature_name=cg02157052	feature_id[451].value <= threshold=0.2869153320789337
node_39: feature_name=cg18751958	feature_id[1427].value > threshold=0.4813787043094635
node_41: feature_name=cg22512847	feature_id[1152].value > threshold=0.5129324495792389
node_43: feature_name=cg23345038	feature_id[1147].value > threshold=0.6318148374557495
node_45: feature_name=cg17439009	feature_id[1815].value <= threshold=0.48514117300510406
node_46: feature_name=cg17537493	feature_id[2772].value > threshold=0.5825372636318207
node_52: feature_name=cg01683570	feature_id[110].value > threshold=0.6040188074111938

node_54: feature_name=cg15720017	feature_id[168].value > threshold=0.7067741453647614
node_60: feature_name=cg23690444	feature_id[120].value <= threshold=0.16037724912166595
node_61: feature_name=cg00567872	feature_id[236].value > threshold=0.7005765736103058
node_85: feature_name=cg01014262	feature_id[764].value <= threshold=0.5737917125225067
node_86: feature_name=cg22946562	feature_id[1148].value > threshold=0.6328203976154327
node_134: feature_name=cg14514032	feature_id[202].value <= threshold=0.4389604330062866
node_135: feature_name=cg13488220	feature_id[241].value > threshold=0.7352153658866882
node_143: feature_name=cg03840920	feature_id[256].value > threshold=0.502630889415741
node_147: feature_name=cg13634090	feature_id[822].value > threshold=0.7207068204879761
node_151: feature_name=cg19584674	feature_id[2364].value <= threshold=0.41758519411087036
node_152: feature_name=cg07344990	feature_id[411].value > threshold=0.5484964847564697
node_158: feature_name=cg10298992	feature_id[3066].value > threshold=0.5707973837852478
node_174: feature_name=cg11843516	feature_id[20].value <= threshold=0.5590928494930267
node_175: feature_name=cg05185926	feature_id[448].value > threshold=0.17318664491176605
node_177: feature_name=cg17009731	feature_id[1220].value > threshold=0.6665130853652954
node_179: feature_name=cg01380319	feature_id[515].value <= threshold=0.39251960813999176
node_180: feature_name=cg20700977	feature_id[303].value > threshold=0.8422040641307831
node_184: feature_name=cg24259291	feature_id[164].value > threshold=0.4872268736362457
node_188: feature_name=cg08097657	feature_id[67].value > threshold=0.3371318429708481
node_190: feature_name=cg07891483	feature_id[59].value > threshold=0.6211732923984528
node_194: feature_name=cg20122645	feature_id[275].value > threshold=0.6094755828380585
node_196: feature_name=cg04026354	feature_id[1763].value <= threshold=0.4341808259487152

node_197: feature_name=cg10149889	feature_id[1017].value > threshold=0.6150195300579071
node_201: feature_name=cg14890730	feature_id[337].value <= threshold=0.4372602105140686
node_202: feature_name=cg08331427	feature_id[132].value > threshold=0.5443233847618103
node_208: feature_name=cg22730007	feature_id[2544].value > threshold=0.28619489073753357
node_210: feature_name=cg17193551	feature_id[2057].value > threshold=0.4393353909254074
node_212: feature_name=cg14671764	feature_id[584].value <= threshold=0.9353199899196625
node_213: feature_name=cg20140662	feature_id[2044].value > threshold=0.38030287623405457
node_215: feature_name=cg07891483	feature_id[59].value > threshold=0.829958975315094
node_219: feature_name=cg04884579	feature_id[1861].value > threshold=0.6106154918670654
node_229: feature_name=cg19548524	feature_id[2985].value > threshold=0.8623353838920593
node_233: feature_name=cg14788686	feature_id[3556].value > threshold=0.35090239346027374
node_235: feature_name=cg05380734	feature_id[2470].value <= threshold=0.7112160623073578
node_236: feature_name=cg01166827	feature_id[3708].value > threshold=0.6359498500823975
node_238: feature_name=cg13251842	feature_id[714].value > threshold=0.8916542232036591
node_240: feature_name=cg02829279	feature_id[623].value > threshold=0.450528159737587
node_242: feature_name=cg24358599	feature_id[86].value > threshold=0.8132582604885101
node_246: feature_name=cg05544807	feature_id[3661].value <= threshold=0.6726464331150055
node_247: feature_name=cg05056653	feature_id[3122].value > threshold=0.35445791482925415
node_249: feature_name=cg26308909	feature_id[770].value > threshold=0.3754204213619232
node_251: feature_name=cg11197908	feature_id[3058].value > threshold=0.36795753240585327
node_253: feature_name=cg00039385	feature_id[646].value <= threshold=0.18521490693092346
node_254: feature_name=cg04787317	feature_id[3379].value > threshold=0.8155552744865417

node_256: feature_name=cg00169184	feature_id[2503].value > threshold=0.9175277948379517
node_258: feature_name=cg11546709	feature_id[966].value <= threshold=0.3400330990552902
Class: small blue round cell tumour (SBRCT)	
Rules_137	passed counts:1
node_0: feature_name=cg11915444	feature_id[1171].value > threshold=0.3402601182460785
node_10: feature_name=cg12109728	feature_id[2810].value > threshold=0.7068270146846771
node_20: feature_name=cg16412000	feature_id[789].value > threshold=0.6741803884506226
node_24: feature_name=cg17115147	feature_id[1434].value <= threshold=0.2961975038051605
node_25: feature_name=cg24474622	feature_id[2737].value > threshold=0.7211934924125671
node_29: feature_name=cg10472711	feature_id[612].value > threshold=0.45985929667949677
node_37: feature_name=cg01937669	feature_id[188].value <= threshold=0.4809463769197464
node_38: feature_name=cg02157052	feature_id[451].value <= threshold=0.2869153320789337
node_39: feature_name=cg18751958	feature_id[1427].value > threshold=0.4813787043094635
node_41: feature_name=cg22512847	feature_id[1152].value > threshold=0.5129324495792389
node_43: feature_name=cg23345038	feature_id[1147].value > threshold=0.6318148374557495
node_45: feature_name=cg17439009	feature_id[1815].value <= threshold=0.48514117300510406
node_46: feature_name=cg17537493	feature_id[2772].value > threshold=0.5825372636318207
node_52: feature_name=cg01683570	feature_id[110].value > threshold=0.6040188074111938
node_54: feature_name=cg15720017	feature_id[168].value > threshold=0.7067741453647614
node_60: feature_name=cg23690444	feature_id[120].value <= threshold=0.16037724912166595
node_61: feature_name=cg00567872	feature_id[236].value > threshold=0.7005765736103058
node_85: feature_name=cg01014262	feature_id[764].value <= threshold=0.5737917125225067

node_86: feature_name=cg22946562	feature_id[1148].value > threshold=0.6328203976154327
node_134: feature_name=cg14514032	feature_id[202].value <= threshold=0.4389604330062866
node_135: feature_name=cg13488220	feature_id[241].value > threshold=0.7352153658866882
node_143: feature_name=cg03840920	feature_id[256].value > threshold=0.502630889415741
node_147: feature_name=cg13634090	feature_id[822].value > threshold=0.7207068204879761
node_151: feature_name=cg19584674	feature_id[2364].value <= threshold=0.41758519411087036
node_152: feature_name=cg07344990	feature_id[411].value > threshold=0.5484964847564697
node_158: feature_name=cg10298992	feature_id[3066].value > threshold=0.5707973837852478
node_174: feature_name=cg11843516	feature_id[20].value <= threshold=0.5590928494930267
node_175: feature_name=cg05185926	feature_id[448].value > threshold=0.17318664491176605
node_177: feature_name=cg17009731	feature_id[1220].value > threshold=0.6666513085365295
node_179: feature_name=cg01380319	feature_id[515].value <= threshold=0.39251960813999176
node_180: feature_name=cg20700977	feature_id[303].value > threshold=0.8422040641307831
node_184: feature_name=cg24259291	feature_id[164].value > threshold=0.4872268736362457
node_188: feature_name=cg08097657	feature_id[67].value > threshold=0.3371318429708481
node_190: feature_name=cg07891483	feature_id[59].value > threshold=0.6211732923984528
node_194: feature_name=cg20122645	feature_id[275].value > threshold=0.6094755828380585
node_196: feature_name=cg04026354	feature_id[1763].value <= threshold=0.4341808259487152
node_197: feature_name=cg10149889	feature_id[1017].value > threshold=0.6150195300579071
node_201: feature_name=cg14890730	feature_id[337].value <= threshold=0.4372602105140686
node_202: feature_name=cg08331427	feature_id[132].value > threshold=0.5443233847618103
node_208: feature_name=cg22730007	feature_id[2544].value > threshold=0.28619489073753357

node_210: feature_name=cg17193551	feature_id[2057].value > threshold=0.4393353909254074
node_212: feature_name=cg14671764	feature_id[584].value <= threshold=0.9353199899196625
node_213: feature_name=cg20140662	feature_id[2044].value > threshold=0.38030287623405457
node_215: feature_name=cg07891483	feature_id[59].value > threshold=0.829958975315094
node_219: feature_name=cg04884579	feature_id[1861].value > threshold=0.6106154918670654
node_229: feature_name=cg19548524	feature_id[2985].value > threshold=0.8623353838920593
node_233: feature_name=cg14788686	feature_id[3556].value > threshold=0.35090239346027374
node_235: feature_name=cg05380734	feature_id[2470].value <= threshold=0.7112160623073578
node_236: feature_name=cg01166827	feature_id[3708].value > threshold=0.6359498500823975
node_238: feature_name=cg13251842	feature_id[714].value > threshold=0.8916542232036591
node_240: feature_name=cg02829279	feature_id[623].value > threshold=0.450528159737587
node_242: feature_name=cg24358599	feature_id[86].value > threshold=0.8132582604885101
node_246: feature_name=cg05544807	feature_id[3661].value <= threshold=0.6726464331150055
node_247: feature_name=cg05056653	feature_id[3122].value > threshold=0.35445791482925415
node_249: feature_name=cg26308909	feature_id[770].value > threshold=0.3754204213619232
node_251: feature_name=cg11197908	feature_id[3058].value > threshold=0.36795753240585327
node_253: feature_name=cg00039385	feature_id[646].value <= threshold=0.18521490693092346
node_254: feature_name=cg04787317	feature_id[3379].value > threshold=0.8155552744865417
node_256: feature_name=cg00169184	feature_id[2503].value <= threshold=0.9175277948379517
Class: chordoma (CHORD)	
Rules_138	passed counts:1
node_0: feature_name=cg11915444	feature_id[1171].value > threshold=0.3402601182460785

node_10: feature_name=cg12109728	feature_id[2810].value > threshold=0.7068270146846771
node_20: feature_name=cg16412000	feature_id[789].value > threshold=0.6741803884506226
node_24: feature_name=cg17115147	feature_id[1434].value <= threshold=0.2961975038051605
node_25: feature_name=cg24474622	feature_id[2737].value > threshold=0.7211934924125671
node_29: feature_name=cg10472711	feature_id[612].value > threshold=0.45985929667949677
node_37: feature_name=cg01937669	feature_id[188].value <= threshold=0.4809463769197464
node_38: feature_name=cg02157052	feature_id[451].value <= threshold=0.2869153320789337
node_39: feature_name=cg18751958	feature_id[1427].value > threshold=0.4813787043094635
node_41: feature_name=cg22512847	feature_id[1152].value > threshold=0.5129324495792389
node_43: feature_name=cg23345038	feature_id[1147].value > threshold=0.6318148374557495
node_45: feature_name=cg17439009	feature_id[1815].value <= threshold=0.48514117300510406
node_46: feature_name=cg17537493	feature_id[2772].value > threshold=0.5825372636318207
node_52: feature_name=cg01683570	feature_id[110].value > threshold=0.6040188074111938
node_54: feature_name=cg15720017	feature_id[168].value > threshold=0.7067741453647614
node_60: feature_name=cg23690444	feature_id[120].value <= threshold=0.16037724912166595
node_61: feature_name=cg00567872	feature_id[236].value > threshold=0.7005765736103058
node_85: feature_name=cg01014262	feature_id[764].value <= threshold=0.5737917125225067
node_86: feature_name=cg22946562	feature_id[1148].value > threshold=0.6328203976154327
node_134: feature_name=cg14514032	feature_id[202].value <= threshold=0.4389604330062866
node_135: feature_name=cg13488220	feature_id[241].value > threshold=0.7352153658866882
node_143: feature_name=cg03840920	feature_id[256].value > threshold=0.502630889415741
node_147: feature_name=cg13634090	feature_id[822].value > threshold=0.7207068204879761

node_151: feature_name=cg19584674	feature_id[2364].value <= threshold=0.41758519411087036
node_152: feature_name=cg07344990	feature_id[411].value > threshold=0.5484964847564697
node_158: feature_name=cg10298992	feature_id[3066].value > threshold=0.5707973837852478
node_174: feature_name=cg11843516	feature_id[20].value <= threshold=0.5590928494930267
node_175: feature_name=cg05185926	feature_id[448].value > threshold=0.17318664491176605
node_177: feature_name=cg17009731	feature_id[1220].value > threshold=0.6666513085365295
node_179: feature_name=cg01380319	feature_id[515].value <= threshold=0.39251960813999176
node_180: feature_name=cg20700977	feature_id[303].value > threshold=0.8422040641307831
node_184: feature_name=cg24259291	feature_id[164].value > threshold=0.4872268736362457
node_188: feature_name=cg08097657	feature_id[67].value > threshold=0.3371318429708481
node_190: feature_name=cg07891483	feature_id[59].value > threshold=0.6211732923984528
node_194: feature_name=cg20122645	feature_id[275].value > threshold=0.6094755828380585
node_196: feature_name=cg04026354	feature_id[1763].value <= threshold=0.4341808259487152
node_197: feature_name=cg10149889	feature_id[1017].value > threshold=0.6150195300579071
node_201: feature_name=cg14890730	feature_id[337].value <= threshold=0.4372602105140686
node_202: feature_name=cg08331427	feature_id[132].value > threshold=0.5443233847618103
node_208: feature_name=cg22730007	feature_id[2544].value > threshold=0.28619489073753357
node_210: feature_name=cg17193551	feature_id[2057].value > threshold=0.4393353909254074
node_212: feature_name=cg14671764	feature_id[584].value <= threshold=0.9353199899196625
node_213: feature_name=cg20140662	feature_id[2044].value > threshold=0.38030287623405457
node_215: feature_name=cg07891483	feature_id[59].value > threshold=0.829958975315094
node_219: feature_name=cg04884579	feature_id[1861].value > threshold=0.6106154918670654

node_229: feature_name=cg19548524	feature_id[2985].value > threshold=0.8623353838920593
node_233: feature_name=cg14788686	feature_id[3556].value > threshold=0.35090239346027374
node_235: feature_name=cg05380734	feature_id[2470].value <= threshold=0.7112160623073578
node_236: feature_name=cg01166827	feature_id[3708].value > threshold=0.6359498500823975
node_238: feature_name=cg13251842	feature_id[714].value > threshold=0.8916542232036591
node_240: feature_name=cg02829279	feature_id[623].value > threshold=0.450528159737587
node_242: feature_name=cg24358599	feature_id[86].value <= threshold=0.8132582604885101
node_243: feature_name=cg01824933	feature_id[300].value <= threshold=0.5986265540122986
Class: angiosarcoma (AS)	
Rules_139	passed counts:1
node_0: feature_name=cg11915444	feature_id[1171].value > threshold=0.3402601182460785
node_10: feature_name=cg12109728	feature_id[2810].value > threshold=0.7068270146846771
node_20: feature_name=cg16412000	feature_id[789].value > threshold=0.6741803884506226
node_24: feature_name=cg17115147	feature_id[1434].value <= threshold=0.2961975038051605
node_25: feature_name=cg24474622	feature_id[2737].value > threshold=0.7211934924125671
node_29: feature_name=cg10472711	feature_id[612].value > threshold=0.45985929667949677
node_37: feature_name=cg01937669	feature_id[188].value <= threshold=0.4809463769197464
node_38: feature_name=cg02157052	feature_id[451].value <= threshold=0.2869153320789337
node_39: feature_name=cg18751958	feature_id[1427].value > threshold=0.4813787043094635
node_41: feature_name=cg22512847	feature_id[1152].value > threshold=0.5129324495792389
node_43: feature_name=cg23345038	feature_id[1147].value > threshold=0.6318148374557495
node_45: feature_name=cg17439009	feature_id[1815].value <= threshold=0.48514117300510406

node_46: feature_name=cg17537493	feature_id[2772].value > threshold=0.5825372636318207
node_52: feature_name=cg01683570	feature_id[110].value > threshold=0.6040188074111938
node_54: feature_name=cg15720017	feature_id[168].value > threshold=0.7067741453647614
node_60: feature_name=cg23690444	feature_id[120].value <= threshold=0.16037724912166595
node_61: feature_name=cg00567872	feature_id[236].value > threshold=0.7005765736103058
node_85: feature_name=cg01014262	feature_id[764].value <= threshold=0.5737917125225067
node_86: feature_name=cg22946562	feature_id[1148].value > threshold=0.6328203976154327
node_134: feature_name=cg14514032	feature_id[202].value <= threshold=0.4389604330062866
node_135: feature_name=cg13488220	feature_id[241].value > threshold=0.7352153658866882
node_143: feature_name=cg03840920	feature_id[256].value > threshold=0.502630889415741
node_147: feature_name=cg13634090	feature_id[822].value > threshold=0.7207068204879761
node_151: feature_name=cg19584674	feature_id[2364].value <= threshold=0.41758519411087036
node_152: feature_name=cg07344990	feature_id[411].value > threshold=0.5484964847564697
node_158: feature_name=cg10298992	feature_id[3066].value > threshold=0.5707973837852478
node_174: feature_name=cg11843516	feature_id[20].value <= threshold=0.5590928494930267
node_175: feature_name=cg05185926	feature_id[448].value > threshold=0.17318664491176605
node_177: feature_name=cg17009731	feature_id[1220].value > threshold=0.6666513085365295
node_179: feature_name=cg01380319	feature_id[515].value <= threshold=0.39251960813999176
node_180: feature_name=cg20700977	feature_id[303].value > threshold=0.8422040641307831
node_184: feature_name=cg24259291	feature_id[164].value > threshold=0.4872268736362457
node_188: feature_name=cg08097657	feature_id[67].value > threshold=0.3371318429708481
node_190: feature_name=cg07891483	feature_id[59].value > threshold=0.6211732923984528

node_194: feature_name=cg20122645	feature_id[275].value > threshold=0.6094755828380585
node_196: feature_name=cg04026354	feature_id[1763].value <= threshold=0.4341808259487152
node_197: feature_name=cg10149889	feature_id[1017].value > threshold=0.6150195300579071
node_201: feature_name=cg14890730	feature_id[337].value <= threshold=0.4372602105140686
node_202: feature_name=cg08331427	feature_id[132].value > threshold=0.5443233847618103
node_208: feature_name=cg22730007	feature_id[2544].value > threshold=0.28619489073753357
node_210: feature_name=cg17193551	feature_id[2057].value > threshold=0.4393353909254074
node_212: feature_name=cg14671764	feature_id[584].value <= threshold=0.9353199899196625
node_213: feature_name=cg20140662	feature_id[2044].value > threshold=0.38030287623405457
node_215: feature_name=cg07891483	feature_id[59].value > threshold=0.829958975315094
node_219: feature_name=cg04884579	feature_id[1861].value > threshold=0.6106154918670654
node_229: feature_name=cg19548524	feature_id[2985].value <= threshold=0.8623353838920593
node_230: feature_name=cg18763720	feature_id[523].value > threshold=0.3843051791191101
Class: high-grade conventional osteosarcoma (OS (HG))	
Rules_140	passed counts:1
node_0: feature_name=cg11915444	feature_id[1171].value > threshold=0.3402601182460785
node_10: feature_name=cg12109728	feature_id[2810].value > threshold=0.7068270146846771
node_20: feature_name=cg16412000	feature_id[789].value > threshold=0.6741803884506226
node_24: feature_name=cg17115147	feature_id[1434].value <= threshold=0.2961975038051605
node_25: feature_name=cg24474622	feature_id[2737].value > threshold=0.7211934924125671
node_29: feature_name=cg10472711	feature_id[612].value > threshold=0.45985929667949677

node_37: feature_name=cg01937669	feature_id[188].value <= threshold=0.4809463769197464
node_38: feature_name=cg02157052	feature_id[451].value <= threshold=0.2869153320789337
node_39: feature_name=cg18751958	feature_id[1427].value > threshold=0.4813787043094635
node_41: feature_name=cg22512847	feature_id[1152].value > threshold=0.5129324495792389
node_43: feature_name=cg23345038	feature_id[1147].value > threshold=0.6318148374557495
node_45: feature_name=cg17439009	feature_id[1815].value <= threshold=0.48514117300510406
node_46: feature_name=cg17537493	feature_id[2772].value > threshold=0.5825372636318207
node_52: feature_name=cg01683570	feature_id[110].value > threshold=0.6040188074111938
node_54: feature_name=cg15720017	feature_id[168].value > threshold=0.7067741453647614
node_60: feature_name=cg23690444	feature_id[120].value <= threshold=0.16037724912166595
node_61: feature_name=cg00567872	feature_id[236].value > threshold=0.7005765736103058
node_85: feature_name=cg01014262	feature_id[764].value <= threshold=0.5737917125225067
node_86: feature_name=cg22946562	feature_id[1148].value > threshold=0.6328203976154327
node_134: feature_name=cg14514032	feature_id[202].value <= threshold=0.4389604330062866
node_135: feature_name=cg13488220	feature_id[241].value > threshold=0.7352153658866882
node_143: feature_name=cg03840920	feature_id[256].value > threshold=0.502630889415741
node_147: feature_name=cg13634090	feature_id[822].value > threshold=0.7207068204879761
node_151: feature_name=cg19584674	feature_id[2364].value <= threshold=0.41758519411087036
node_152: feature_name=cg07344990	feature_id[411].value > threshold=0.5484964847564697
node_158: feature_name=cg10298992	feature_id[3066].value > threshold=0.5707973837852478
node_174: feature_name=cg11843516	feature_id[20].value <= threshold=0.5590928494930267
node_175: feature_name=cg05185926	feature_id[448].value > threshold=0.17318664491176605

node_177: feature_name=cg17009731	feature_id[1220].value > threshold=0.6666513085365295
node_179: feature_name=cg01380319	feature_id[515].value <= threshold=0.39251960813999176
node_180: feature_name=cg20700977	feature_id[303].value > threshold=0.8422040641307831
node_184: feature_name=cg24259291	feature_id[164].value > threshold=0.4872268736362457
node_188: feature_name=cg08097657	feature_id[67].value > threshold=0.3371318429708481
node_190: feature_name=cg07891483	feature_id[59].value > threshold=0.6211732923984528
node_194: feature_name=cg20122645	feature_id[275].value > threshold=0.6094755828380585
node_196: feature_name=cg04026354	feature_id[1763].value <= threshold=0.4341808259487152
node_197: feature_name=cg10149889	feature_id[1017].value > threshold=0.6150195300579071
node_201: feature_name=cg14890730	feature_id[337].value <= threshold=0.4372602105140686
node_202: feature_name=cg08331427	feature_id[132].value > threshold=0.5443233847618103
node_208: feature_name=cg22730007	feature_id[2544].value > threshold=0.28619489073753357
node_210: feature_name=cg17193551	feature_id[2057].value > threshold=0.4393353909254074
node_212: feature_name=cg14671764	feature_id[584].value <= threshold=0.9353199899196625
node_213: feature_name=cg20140662	feature_id[2044].value > threshold=0.38030287623405457
node_215: feature_name=cg07891483	feature_id[59].value > threshold=0.829958975315094
node_219: feature_name=cg04884579	feature_id[1861].value <= threshold=0.6106154918670654
node_220: feature_name=cg25066857	feature_id[3759].value <= threshold=0.8149203658103943
node_221: feature_name=cg04813695	feature_id[1758].value > threshold=0.8937293887138367
node_223: feature_name=cg12895304	feature_id[5].value <= threshold=0.8254048228263855
node_224: feature_name=cg21863499	feature_id[2551].value > threshold=0.07621219754219055
Class: myositis ossificans (MO)	

Rules_141	passed counts:1
node_0: feature_name=cg11915444	feature_id[1171].value > threshold=0.3402601182460785
node_10: feature_name=cg12109728	feature_id[2810].value > threshold=0.7068270146846771
node_20: feature_name=cg16412000	feature_id[789].value > threshold=0.6741803884506226
node_24: feature_name=cg17115147	feature_id[1434].value <= threshold=0.2961975038051605
node_25: feature_name=cg24474622	feature_id[2737].value > threshold=0.7211934924125671
node_29: feature_name=cg10472711	feature_id[612].value > threshold=0.45985929667949677
node_37: feature_name=cg01937669	feature_id[188].value <= threshold=0.4809463769197464
node_38: feature_name=cg02157052	feature_id[451].value <= threshold=0.2869153320789337
node_39: feature_name=cg18751958	feature_id[1427].value > threshold=0.4813787043094635
node_41: feature_name=cg22512847	feature_id[1152].value > threshold=0.5129324495792389
node_43: feature_name=cg23345038	feature_id[1147].value > threshold=0.6318148374557495
node_45: feature_name=cg17439009	feature_id[1815].value <= threshold=0.48514117300510406
node_46: feature_name=cg17537493	feature_id[2772].value > threshold=0.5825372636318207
node_52: feature_name=cg01683570	feature_id[110].value > threshold=0.6040188074111938
node_54: feature_name=cg15720017	feature_id[168].value > threshold=0.7067741453647614
node_60: feature_name=cg23690444	feature_id[120].value <= threshold=0.16037724912166595
node_61: feature_name=cg00567872	feature_id[236].value > threshold=0.7005765736103058
node_85: feature_name=cg01014262	feature_id[764].value <= threshold=0.5737917125225067
node_86: feature_name=cg22946562	feature_id[1148].value > threshold=0.6328203976154327
node_134: feature_name=cg14514032	feature_id[202].value <= threshold=0.4389604330062866

node_135: feature_name=cg13488220	feature_id[241].value > threshold=0.7352153658866882
node_143: feature_name=cg03840920	feature_id[256].value > threshold=0.502630889415741
node_147: feature_name=cg13634090	feature_id[822].value > threshold=0.7207068204879761
node_151: feature_name=cg19584674	feature_id[2364].value <= threshold=0.41758519411087036
node_152: feature_name=cg07344990	feature_id[411].value > threshold=0.5484964847564697
node_158: feature_name=cg10298992	feature_id[3066].value > threshold=0.5707973837852478
node_174: feature_name=cg11843516	feature_id[20].value <= threshold=0.5590928494930267
node_175: feature_name=cg05185926	feature_id[448].value > threshold=0.17318664491176605
node_177: feature_name=cg17009731	feature_id[1220].value > threshold=0.6666513085365295
node_179: feature_name=cg01380319	feature_id[515].value <= threshold=0.39251960813999176
node_180: feature_name=cg20700977	feature_id[303].value > threshold=0.8422040641307831
node_184: feature_name=cg24259291	feature_id[164].value > threshold=0.4872268736362457
node_188: feature_name=cg08097657	feature_id[67].value > threshold=0.3371318429708481
node_190: feature_name=cg07891483	feature_id[59].value > threshold=0.6211732923984528
node_194: feature_name=cg20122645	feature_id[275].value > threshold=0.6094755828380585
node_196: feature_name=cg04026354	feature_id[1763].value <= threshold=0.4341808259487152
node_197: feature_name=cg10149889	feature_id[1017].value > threshold=0.6150195300579071
node_201: feature_name=cg14890730	feature_id[337].value <= threshold=0.4372602105140686
node_202: feature_name=cg08331427	feature_id[132].value > threshold=0.5443233847618103
node_208: feature_name=cg22730007	feature_id[2544].value > threshold=0.28619489073753357
node_210: feature_name=cg17193551	feature_id[2057].value > threshold=0.4393353909254074
node_212: feature_name=cg14671764	feature_id[584].value <= threshold=0.9353199899196625

node_213: feature_name=cg20140662	feature_id[2044].value > threshold=0.38030287623405457
node_215: feature_name=cg07891483	feature_id[59].value > threshold=0.829958975315094
node_219: feature_name=cg04884579	feature_id[1861].value <= threshold=0.6106154918670654
node_220: feature_name=cg25066857	feature_id[3759].value <= threshold=0.8149203658103943
node_221: feature_name=cg04813695	feature_id[1758].value > threshold=0.8937293887138367
node_223: feature_name=cg12895304	feature_id[5].value <= threshold=0.8254048228263855
node_224: feature_name=cg21863499	feature_id[2551].value <= threshold=0.07621219754219055
Class: embryonal rhabdomyosarcoma (RMS (EMB))	
Rules_142	passed counts:1
node_0: feature_name=cg11915444	feature_id[1171].value > threshold=0.3402601182460785
node_10: feature_name=cg12109728	feature_id[2810].value > threshold=0.7068270146846771
node_20: feature_name=cg16412000	feature_id[789].value > threshold=0.6741803884506226
node_24: feature_name=cg17115147	feature_id[1434].value <= threshold=0.2961975038051605
node_25: feature_name=cg24474622	feature_id[2737].value > threshold=0.7211934924125671
node_29: feature_name=cg10472711	feature_id[612].value > threshold=0.45985929667949677
node_37: feature_name=cg01937669	feature_id[188].value <= threshold=0.4809463769197464
node_38: feature_name=cg02157052	feature_id[451].value <= threshold=0.2869153320789337
node_39: feature_name=cg18751958	feature_id[1427].value > threshold=0.4813787043094635
node_41: feature_name=cg22512847	feature_id[1152].value > threshold=0.5129324495792389
node_43: feature_name=cg23345038	feature_id[1147].value > threshold=0.6318148374557495
node_45: feature_name=cg17439009	feature_id[1815].value <= threshold=0.48514117300510406

node_46: feature_name=cg17537493	feature_id[2772].value > threshold=0.5825372636318207
node_52: feature_name=cg01683570	feature_id[110].value > threshold=0.6040188074111938
node_54: feature_name=cg15720017	feature_id[168].value > threshold=0.7067741453647614
node_60: feature_name=cg23690444	feature_id[120].value <= threshold=0.16037724912166595
node_61: feature_name=cg00567872	feature_id[236].value > threshold=0.7005765736103058
node_85: feature_name=cg01014262	feature_id[764].value <= threshold=0.5737917125225067
node_86: feature_name=cg22946562	feature_id[1148].value > threshold=0.6328203976154327
node_134: feature_name=cg14514032	feature_id[202].value <= threshold=0.4389604330062866
node_135: feature_name=cg13488220	feature_id[241].value > threshold=0.7352153658866882
node_143: feature_name=cg03840920	feature_id[256].value > threshold=0.502630889415741
node_147: feature_name=cg13634090	feature_id[822].value > threshold=0.7207068204879761
node_151: feature_name=cg19584674	feature_id[2364].value <= threshold=0.41758519411087036
node_152: feature_name=cg07344990	feature_id[411].value > threshold=0.5484964847564697
node_158: feature_name=cg10298992	feature_id[3066].value > threshold=0.5707973837852478
node_174: feature_name=cg11843516	feature_id[20].value <= threshold=0.5590928494930267
node_175: feature_name=cg05185926	feature_id[448].value > threshold=0.17318664491176605
node_177: feature_name=cg17009731	feature_id[1220].value > threshold=0.6666513085365295
node_179: feature_name=cg01380319	feature_id[515].value <= threshold=0.39251960813999176
node_180: feature_name=cg20700977	feature_id[303].value > threshold=0.8422040641307831
node_184: feature_name=cg24259291	feature_id[164].value > threshold=0.4872268736362457
node_188: feature_name=cg08097657	feature_id[67].value > threshold=0.3371318429708481
node_190: feature_name=cg07891483	feature_id[59].value > threshold=0.6211732923984528

node_194: feature_name=cg20122645	feature_id[275].value > threshold=0.6094755828380585
node_196: feature_name=cg04026354	feature_id[1763].value <= threshold=0.4341808259487152
node_197: feature_name=cg10149889	feature_id[1017].value > threshold=0.6150195300579071
node_201: feature_name=cg14890730	feature_id[337].value <= threshold=0.4372602105140686
node_202: feature_name=cg08331427	feature_id[132].value > threshold=0.5443233847618103
node_208: feature_name=cg22730007	feature_id[2544].value > threshold=0.28619489073753357
node_210: feature_name=cg17193551	feature_id[2057].value > threshold=0.4393353909254074
node_212: feature_name=cg14671764	feature_id[584].value <= threshold=0.9353199899196625
node_213: feature_name=cg20140662	feature_id[2044].value > threshold=0.38030287623405457
node_215: feature_name=cg07891483	feature_id[59].value <= threshold=0.829958975315094
node_216: feature_name=cg14487665	feature_id[3561].value <= threshold=0.8762882351875305
Class: clear cell sarcoma of soft parts (CCS)	
Rules_143	passed counts:1
node_0: feature_name=cg11915444	feature_id[1171].value > threshold=0.3402601182460785
node_10: feature_name=cg12109728	feature_id[2810].value > threshold=0.7068270146846771
node_20: feature_name=cg16412000	feature_id[789].value > threshold=0.6741803884506226
node_24: feature_name=cg17115147	feature_id[1434].value <= threshold=0.2961975038051605
node_25: feature_name=cg24474622	feature_id[2737].value > threshold=0.7211934924125671
node_29: feature_name=cg10472711	feature_id[612].value > threshold=0.45985929667949677
node_37: feature_name=cg01937669	feature_id[188].value <= threshold=0.4809463769197464
node_38: feature_name=cg02157052	feature_id[451].value <= threshold=0.2869153320789337
node_39: feature_name=cg18751958	feature_id[1427].value > threshold=0.4813787043094635

node_41: feature_name=cg22512847	feature_id[1152].value > threshold=0.5129324495792389
node_43: feature_name=cg23345038	feature_id[1147].value > threshold=0.6318148374557495
node_45: feature_name=cg17439009	feature_id[1815].value <= threshold=0.48514117300510406
node_46: feature_name=cg17537493	feature_id[2772].value > threshold=0.5825372636318207
node_52: feature_name=cg01683570	feature_id[110].value > threshold=0.6040188074111938
node_54: feature_name=cg15720017	feature_id[168].value > threshold=0.7067741453647614
node_60: feature_name=cg23690444	feature_id[120].value <= threshold=0.16037724912166595
node_61: feature_name=cg00567872	feature_id[236].value > threshold=0.7005765736103058
node_85: feature_name=cg01014262	feature_id[764].value <= threshold=0.5737917125225067
node_86: feature_name=cg22946562	feature_id[1148].value > threshold=0.6328203976154327
node_134: feature_name=cg14514032	feature_id[202].value <= threshold=0.4389604330062866
node_135: feature_name=cg13488220	feature_id[241].value > threshold=0.7352153658866882
node_143: feature_name=cg03840920	feature_id[256].value > threshold=0.502630889415741
node_147: feature_name=cg13634090	feature_id[822].value > threshold=0.7207068204879761
node_151: feature_name=cg19584674	feature_id[2364].value <= threshold=0.41758519411087036
node_152: feature_name=cg07344990	feature_id[411].value > threshold=0.5484964847564697
node_158: feature_name=cg10298992	feature_id[3066].value > threshold=0.5707973837852478
node_174: feature_name=cg11843516	feature_id[20].value <= threshold=0.5590928494930267
node_175: feature_name=cg05185926	feature_id[448].value > threshold=0.17318664491176605
node_177: feature_name=cg17009731	feature_id[1220].value > threshold=0.6666513085365295
node_179: feature_name=cg01380319	feature_id[515].value <= threshold=0.39251960813999176
node_180: feature_name=cg20700977	feature_id[303].value > threshold=0.8422040641307831

node_184: feature_name=cg24259291	feature_id[164].value > threshold=0.4872268736362457
node_188: feature_name=cg08097657	feature_id[67].value > threshold=0.3371318429708481
node_190: feature_name=cg07891483	feature_id[59].value > threshold=0.6211732923984528
node_194: feature_name=cg20122645	feature_id[275].value > threshold=0.6094755828380585
node_196: feature_name=cg04026354	feature_id[1763].value <= threshold=0.4341808259487152
node_197: feature_name=cg10149889	feature_id[1017].value > threshold=0.6150195300579071
node_201: feature_name=cg14890730	feature_id[337].value <= threshold=0.4372602105140686
node_202: feature_name=cg08331427	feature_id[132].value <= threshold=0.5443233847618103
node_203: feature_name=cg04963697	feature_id[102].value <= threshold=0.7484472692012787
node_204: feature_name=cg23722792	feature_id[1201].value > threshold=0.8487101495265961
Class: well differentiated liposarcoma (WDLS)/dedifferentiated liposarcoma (DDLS)	
Rules_144	passed counts:1
node_0: feature_name=cg11915444	feature_id[1171].value > threshold=0.3402601182460785
node_10: feature_name=cg12109728	feature_id[2810].value > threshold=0.7068270146846771
node_20: feature_name=cg16412000	feature_id[789].value > threshold=0.6741803884506226
node_24: feature_name=cg17115147	feature_id[1434].value <= threshold=0.2961975038051605
node_25: feature_name=cg24474622	feature_id[2737].value > threshold=0.7211934924125671
node_29: feature_name=cg10472711	feature_id[612].value > threshold=0.45985929667949677
node_37: feature_name=cg01937669	feature_id[188].value <= threshold=0.4809463769197464
node_38: feature_name=cg02157052	feature_id[451].value <= threshold=0.2869153320789337
node_39: feature_name=cg18751958	feature_id[1427].value > threshold=0.4813787043094635

node_41: feature_name=cg22512847	feature_id[1152].value > threshold=0.5129324495792389
node_43: feature_name=cg23345038	feature_id[1147].value > threshold=0.6318148374557495
node_45: feature_name=cg17439009	feature_id[1815].value <= threshold=0.48514117300510406
node_46: feature_name=cg17537493	feature_id[2772].value > threshold=0.5825372636318207
node_52: feature_name=cg01683570	feature_id[110].value > threshold=0.6040188074111938
node_54: feature_name=cg15720017	feature_id[168].value > threshold=0.7067741453647614
node_60: feature_name=cg23690444	feature_id[120].value <= threshold=0.16037724912166595
node_61: feature_name=cg00567872	feature_id[236].value > threshold=0.7005765736103058
node_85: feature_name=cg01014262	feature_id[764].value <= threshold=0.5737917125225067
node_86: feature_name=cg22946562	feature_id[1148].value > threshold=0.6328203976154327
node_134: feature_name=cg14514032	feature_id[202].value <= threshold=0.4389604330062866
node_135: feature_name=cg13488220	feature_id[241].value > threshold=0.7352153658866882
node_143: feature_name=cg03840920	feature_id[256].value > threshold=0.502630889415741
node_147: feature_name=cg13634090	feature_id[822].value > threshold=0.7207068204879761
node_151: feature_name=cg19584674	feature_id[2364].value <= threshold=0.41758519411087036
node_152: feature_name=cg07344990	feature_id[411].value > threshold=0.5484964847564697
node_158: feature_name=cg10298992	feature_id[3066].value > threshold=0.5707973837852478
node_174: feature_name=cg11843516	feature_id[20].value <= threshold=0.5590928494930267
node_175: feature_name=cg05185926	feature_id[448].value > threshold=0.17318664491176605
node_177: feature_name=cg17009731	feature_id[1220].value > threshold=0.6666513085365295
node_179: feature_name=cg01380319	feature_id[515].value <= threshold=0.39251960813999176
node_180: feature_name=cg20700977	feature_id[303].value > threshold=0.8422040641307831

node_184: feature_name=cg24259291	feature_id[164].value > threshold=0.4872268736362457
node_188: feature_name=cg08097657	feature_id[67].value > threshold=0.3371318429708481
node_190: feature_name=cg07891483	feature_id[59].value > threshold=0.6211732923984528
node_194: feature_name=cg20122645	feature_id[275].value > threshold=0.6094755828380585
node_196: feature_name=cg04026354	feature_id[1763].value <= threshold=0.4341808259487152
node_197: feature_name=cg10149889	feature_id[1017].value > threshold=0.6150195300579071
node_201: feature_name=cg14890730	feature_id[337].value <= threshold=0.4372602105140686
node_202: feature_name=cg08331427	feature_id[132].value <= threshold=0.5443233847618103
node_203: feature_name=cg04963697	feature_id[102].value <= threshold=0.7484472692012787
node_204: feature_name=cg23722792	feature_id[1201].value <= threshold=0.8487101495265961
Class: chondroblastoma (CB)	
Rules_145	passed counts:1
node_0: feature_name=cg11915444	feature_id[1171].value > threshold=0.3402601182460785
node_10: feature_name=cg12109728	feature_id[2810].value > threshold=0.7068270146846771
node_20: feature_name=cg16412000	feature_id[789].value > threshold=0.6741803884506226
node_24: feature_name=cg17115147	feature_id[1434].value <= threshold=0.2961975038051605
node_25: feature_name=cg24474622	feature_id[2737].value > threshold=0.7211934924125671
node_29: feature_name=cg10472711	feature_id[612].value > threshold=0.45985929667949677
node_37: feature_name=cg01937669	feature_id[188].value <= threshold=0.4809463769197464
node_38: feature_name=cg02157052	feature_id[451].value <= threshold=0.2869153320789337
node_39: feature_name=cg18751958	feature_id[1427].value > threshold=0.4813787043094635
node_41: feature_name=cg22512847	feature_id[1152].value > threshold=0.5129324495792389

node_43: feature_name=cg23345038	feature_id[1147].value > threshold=0.6318148374557495
node_45: feature_name=cg17439009	feature_id[1815].value <= threshold=0.48514117300510406
node_46: feature_name=cg17537493	feature_id[2772].value > threshold=0.5825372636318207
node_52: feature_name=cg01683570	feature_id[110].value > threshold=0.6040188074111938
node_54: feature_name=cg15720017	feature_id[168].value > threshold=0.7067741453647614
node_60: feature_name=cg23690444	feature_id[120].value <= threshold=0.16037724912166595
node_61: feature_name=cg00567872	feature_id[236].value > threshold=0.7005765736103058
node_85: feature_name=cg01014262	feature_id[764].value <= threshold=0.5737917125225067
node_86: feature_name=cg22946562	feature_id[1148].value > threshold=0.6328203976154327
node_134: feature_name=cg14514032	feature_id[202].value <= threshold=0.4389604330062866
node_135: feature_name=cg13488220	feature_id[241].value > threshold=0.7352153658866882
node_143: feature_name=cg03840920	feature_id[256].value > threshold=0.502630889415741
node_147: feature_name=cg13634090	feature_id[822].value > threshold=0.7207068204879761
node_151: feature_name=cg19584674	feature_id[2364].value <= threshold=0.41758519411087036
node_152: feature_name=cg07344990	feature_id[411].value > threshold=0.5484964847564697
node_158: feature_name=cg10298992	feature_id[3066].value > threshold=0.5707973837852478
node_174: feature_name=cg11843516	feature_id[20].value <= threshold=0.5590928494930267
node_175: feature_name=cg05185926	feature_id[448].value > threshold=0.17318664491176605
node_177: feature_name=cg17009731	feature_id[1220].value > threshold=0.6666513085365295
node_179: feature_name=cg01380319	feature_id[515].value <= threshold=0.39251960813999176
node_180: feature_name=cg20700977	feature_id[303].value > threshold=0.8422040641307831
node_184: feature_name=cg24259291	feature_id[164].value > threshold=0.4872268736362457

node_188: feature_name=cg08097657	feature_id[67].value > threshold=0.3371318429708481
node_190: feature_name=cg07891483	feature_id[59].value <= threshold=0.6211732923984528
node_191: feature_name=cg23903708	feature_id[1504].value > threshold=0.8707234263420105
Class: clear cell sarcoma of soft parts (CCS)	
Rules_146	passed counts:1
node_0: feature_name=cg11915444	feature_id[1171].value > threshold=0.3402601182460785
node_10: feature_name=cg12109728	feature_id[2810].value > threshold=0.7068270146846771
node_20: feature_name=cg16412000	feature_id[789].value > threshold=0.6741803884506226
node_24: feature_name=cg17115147	feature_id[1434].value <= threshold=0.2961975038051605
node_25: feature_name=cg24474622	feature_id[2737].value > threshold=0.7211934924125671
node_29: feature_name=cg10472711	feature_id[612].value > threshold=0.45985929667949677
node_37: feature_name=cg01937669	feature_id[188].value <= threshold=0.4809463769197464
node_38: feature_name=cg02157052	feature_id[451].value <= threshold=0.2869153320789337
node_39: feature_name=cg18751958	feature_id[1427].value > threshold=0.4813787043094635
node_41: feature_name=cg22512847	feature_id[1152].value > threshold=0.5129324495792389
node_43: feature_name=cg23345038	feature_id[1147].value > threshold=0.6318148374557495
node_45: feature_name=cg17439009	feature_id[1815].value <= threshold=0.48514117300510406
node_46: feature_name=cg17537493	feature_id[2772].value > threshold=0.5825372636318207
node_52: feature_name=cg01683570	feature_id[110].value > threshold=0.6040188074111938
node_54: feature_name=cg15720017	feature_id[168].value > threshold=0.7067741453647614
node_60: feature_name=cg23690444	feature_id[120].value <= threshold=0.16037724912166595
node_61: feature_name=cg00567872	feature_id[236].value > threshold=0.7005765736103058

node_85: feature_name=cg01014262	feature_id[764].value <= threshold=0.5737917125225067
node_86: feature_name=cg22946562	feature_id[1148].value > threshold=0.6328203976154327
node_134: feature_name=cg14514032	feature_id[202].value <= threshold=0.4389604330062866
node_135: feature_name=cg13488220	feature_id[241].value > threshold=0.7352153658866882
node_143: feature_name=cg03840920	feature_id[256].value > threshold=0.502630889415741
node_147: feature_name=cg13634090	feature_id[822].value > threshold=0.7207068204879761
node_151: feature_name=cg19584674	feature_id[2364].value <= threshold=0.41758519411087036
node_152: feature_name=cg07344990	feature_id[411].value > threshold=0.5484964847564697
node_158: feature_name=cg10298992	feature_id[3066].value > threshold=0.5707973837852478
node_174: feature_name=cg11843516	feature_id[20].value <= threshold=0.5590928494930267
node_175: feature_name=cg05185926	feature_id[448].value > threshold=0.17318664491176605
node_177: feature_name=cg17009731	feature_id[1220].value > threshold=0.6666513085365295
node_179: feature_name=cg01380319	feature_id[515].value <= threshold=0.39251960813999176
node_180: feature_name=cg20700977	feature_id[303].value > threshold=0.8422040641307831
node_184: feature_name=cg24259291	feature_id[164].value <= threshold=0.4872268736362457
node_185: feature_name=cg00512280	feature_id[1400].value <= threshold=0.05498958192765713
Class: chordoma (CHORD)	
Rules_147	passed counts:1
node_0: feature_name=cg11915444	feature_id[1171].value > threshold=0.3402601182460785
node_10: feature_name=cg12109728	feature_id[2810].value > threshold=0.7068270146846771
node_20: feature_name=cg16412000	feature_id[789].value > threshold=0.6741803884506226
node_24: feature_name=cg17115147	feature_id[1434].value <= threshold=0.2961975038051605

node_25: feature_name=cg24474622	feature_id[2737].value > threshold=0.7211934924125671
node_29: feature_name=cg10472711	feature_id[612].value > threshold=0.45985929667949677
node_37: feature_name=cg01937669	feature_id[188].value <= threshold=0.4809463769197464
node_38: feature_name=cg02157052	feature_id[451].value <= threshold=0.2869153320789337
node_39: feature_name=cg18751958	feature_id[1427].value > threshold=0.4813787043094635
node_41: feature_name=cg22512847	feature_id[1152].value > threshold=0.5129324495792389
node_43: feature_name=cg23345038	feature_id[1147].value > threshold=0.6318148374557495
node_45: feature_name=cg17439009	feature_id[1815].value <= threshold=0.48514117300510406
node_46: feature_name=cg17537493	feature_id[2772].value > threshold=0.5825372636318207
node_52: feature_name=cg01683570	feature_id[110].value > threshold=0.6040188074111938
node_54: feature_name=cg15720017	feature_id[168].value > threshold=0.7067741453647614
node_60: feature_name=cg23690444	feature_id[120].value <= threshold=0.16037724912166595
node_61: feature_name=cg00567872	feature_id[236].value > threshold=0.7005765736103058
node_85: feature_name=cg01014262	feature_id[764].value <= threshold=0.5737917125225067
node_86: feature_name=cg22946562	feature_id[1148].value > threshold=0.6328203976154327
node_134: feature_name=cg14514032	feature_id[202].value <= threshold=0.4389604330062866
node_135: feature_name=cg13488220	feature_id[241].value > threshold=0.7352153658866882
node_143: feature_name=cg03840920	feature_id[256].value > threshold=0.502630889415741
node_147: feature_name=cg13634090	feature_id[822].value > threshold=0.7207068204879761
node_151: feature_name=cg19584674	feature_id[2364].value <= threshold=0.41758519411087036
node_152: feature_name=cg07344990	feature_id[411].value > threshold=0.5484964847564697
node_158: feature_name=cg10298992	feature_id[3066].value <= threshold=0.5707973837852478

node_159: feature_name=cg24852135	feature_id[47].value > threshold=0.8445721566677094
node_171: feature_name=cg06474428	feature_id[3652].value > threshold=0.9347167015075684
Class: lipoma (LIPO)	
Rules_148	passed counts:1
node_0: feature_name=cg11915444	feature_id[1171].value > threshold=0.3402601182460785
node_10: feature_name=cg12109728	feature_id[2810].value > threshold=0.7068270146846771
node_20: feature_name=cg16412000	feature_id[789].value > threshold=0.6741803884506226
node_24: feature_name=cg17115147	feature_id[1434].value <= threshold=0.2961975038051605
node_25: feature_name=cg24474622	feature_id[2737].value > threshold=0.7211934924125671
node_29: feature_name=cg10472711	feature_id[612].value > threshold=0.45985929667949677
node_37: feature_name=cg01937669	feature_id[188].value <= threshold=0.4809463769197464
node_38: feature_name=cg02157052	feature_id[451].value <= threshold=0.2869153320789337
node_39: feature_name=cg18751958	feature_id[1427].value > threshold=0.4813787043094635
node_41: feature_name=cg22512847	feature_id[1152].value > threshold=0.5129324495792389
node_43: feature_name=cg23345038	feature_id[1147].value > threshold=0.6318148374557495
node_45: feature_name=cg17439009	feature_id[1815].value <= threshold=0.48514117300510406
node_46: feature_name=cg17537493	feature_id[2772].value > threshold=0.5825372636318207
node_52: feature_name=cg01683570	feature_id[110].value > threshold=0.6040188074111938
node_54: feature_name=cg15720017	feature_id[168].value > threshold=0.7067741453647614
node_60: feature_name=cg23690444	feature_id[120].value <= threshold=0.16037724912166595
node_61: feature_name=cg00567872	feature_id[236].value > threshold=0.7005765736103058
node_85: feature_name=cg01014262	feature_id[764].value <= threshold=0.5737917125225067

node_86: feature_name=cg22946562	feature_id[1148].value > threshold=0.6328203976154327
node_134: feature_name=cg14514032	feature_id[202].value <= threshold=0.4389604330062866
node_135: feature_name=cg13488220	feature_id[241].value > threshold=0.7352153658866882
node_143: feature_name=cg03840920	feature_id[256].value > threshold=0.502630889415741
node_147: feature_name=cg13634090	feature_id[822].value > threshold=0.7207068204879761
node_151: feature_name=cg19584674	feature_id[2364].value <= threshold=0.41758519411087036
node_152: feature_name=cg07344990	feature_id[411].value > threshold=0.5484964847564697
node_158: feature_name=cg10298992	feature_id[3066].value <= threshold=0.5707973837852478
node_159: feature_name=cg24852135	feature_id[47].value <= threshold=0.8445721566677094
node_160: feature_name=cg25606046	feature_id[2007].value <= threshold=0.8722946643829346
node_161: feature_name=cg03212620	feature_id[1188].value > threshold=0.4703744798898697
node_163: feature_name=cg27096087	feature_id[2512].value > threshold=0.8232799470424652
node_165: feature_name=cg26963844	feature_id[175].value > threshold=0.05642275512218475
node_167: feature_name=cg01000937	feature_id[2904].value > threshold=0.5332071930170059
Class: rhabdomyosarcoma (RMS)	
Rules_149	passed counts:1
node_0: feature_name=cg11915444	feature_id[1171].value > threshold=0.3402601182460785
node_10: feature_name=cg12109728	feature_id[2810].value > threshold=0.7068270146846771
node_20: feature_name=cg16412000	feature_id[789].value > threshold=0.6741803884506226
node_24: feature_name=cg17115147	feature_id[1434].value <= threshold=0.2961975038051605
node_25: feature_name=cg24474622	feature_id[2737].value > threshold=0.7211934924125671
node_29: feature_name=cg10472711	feature_id[612].value > threshold=0.45985929667949677

node_37: feature_name=cg01937669	feature_id[188].value <= threshold=0.4809463769197464
node_38: feature_name=cg02157052	feature_id[451].value <= threshold=0.2869153320789337
node_39: feature_name=cg18751958	feature_id[1427].value > threshold=0.4813787043094635
node_41: feature_name=cg22512847	feature_id[1152].value > threshold=0.5129324495792389
node_43: feature_name=cg23345038	feature_id[1147].value > threshold=0.6318148374557495
node_45: feature_name=cg17439009	feature_id[1815].value <= threshold=0.48514117300510406
node_46: feature_name=cg17537493	feature_id[2772].value > threshold=0.5825372636318207
node_52: feature_name=cg01683570	feature_id[110].value > threshold=0.6040188074111938
node_54: feature_name=cg15720017	feature_id[168].value > threshold=0.7067741453647614
node_60: feature_name=cg23690444	feature_id[120].value <= threshold=0.16037724912166595
node_61: feature_name=cg00567872	feature_id[236].value > threshold=0.7005765736103058
node_85: feature_name=cg01014262	feature_id[764].value <= threshold=0.5737917125225067
node_86: feature_name=cg22946562	feature_id[1148].value > threshold=0.6328203976154327
node_134: feature_name=cg14514032	feature_id[202].value <= threshold=0.4389604330062866
node_135: feature_name=cg13488220	feature_id[241].value > threshold=0.7352153658866882
node_143: feature_name=cg03840920	feature_id[256].value > threshold=0.502630889415741
node_147: feature_name=cg13634090	feature_id[822].value > threshold=0.7207068204879761
node_151: feature_name=cg19584674	feature_id[2364].value <= threshold=0.41758519411087036
node_152: feature_name=cg07344990	feature_id[411].value > threshold=0.5484964847564697
node_158: feature_name=cg10298992	feature_id[3066].value <= threshold=0.5707973837852478
node_159: feature_name=cg24852135	feature_id[47].value <= threshold=0.8445721566677094
node_160: feature_name=cg25606046	feature_id[2007].value <= threshold=0.8722946643829346

node_161: feature_name=cg03212620	feature_id[1188].value > threshold=0.4703744798898697
node_163: feature_name=cg27096087	feature_id[2512].value > threshold=0.8232799470424652
node_165: feature_name=cg26963844	feature_id[175].value > threshold=0.05642275512218475
node_167: feature_name=cg01000937	feature_id[2904].value <= threshold=0.5332071930170059
Class: Langerhans cell histiocytosis (LCH)	
Rules_150	passed counts:1
node_0: feature_name=cg11915444	feature_id[1171].value > threshold=0.3402601182460785
node_10: feature_name=cg12109728	feature_id[2810].value > threshold=0.7068270146846771
node_20: feature_name=cg16412000	feature_id[789].value > threshold=0.6741803884506226
node_24: feature_name=cg17115147	feature_id[1434].value <= threshold=0.2961975038051605
node_25: feature_name=cg24474622	feature_id[2737].value > threshold=0.7211934924125671
node_29: feature_name=cg10472711	feature_id[612].value > threshold=0.45985929667949677
node_37: feature_name=cg01937669	feature_id[188].value <= threshold=0.4809463769197464
node_38: feature_name=cg02157052	feature_id[451].value <= threshold=0.2869153320789337
node_39: feature_name=cg18751958	feature_id[1427].value > threshold=0.4813787043094635
node_41: feature_name=cg22512847	feature_id[1152].value > threshold=0.5129324495792389
node_43: feature_name=cg23345038	feature_id[1147].value > threshold=0.6318148374557495
node_45: feature_name=cg17439009	feature_id[1815].value <= threshold=0.48514117300510406
node_46: feature_name=cg17537493	feature_id[2772].value > threshold=0.5825372636318207
node_52: feature_name=cg01683570	feature_id[110].value > threshold=0.6040188074111938
node_54: feature_name=cg15720017	feature_id[168].value > threshold=0.7067741453647614
node_60: feature_name=cg23690444	feature_id[120].value <= threshold=0.16037724912166595

node_61: feature_name=cg00567872	feature_id[236].value > threshold=0.7005765736103058
node_85: feature_name=cg01014262	feature_id[764].value <= threshold=0.5737917125225067
node_86: feature_name=cg22946562	feature_id[1148].value > threshold=0.6328203976154327
node_134: feature_name=cg14514032	feature_id[202].value <= threshold=0.4389604330062866
node_135: feature_name=cg13488220	feature_id[241].value > threshold=0.7352153658866882
node_143: feature_name=cg03840920	feature_id[256].value > threshold=0.502630889415741
node_147: feature_name=cg13634090	feature_id[822].value > threshold=0.7207068204879761
node_151: feature_name=cg19584674	feature_id[2364].value <= threshold=0.41758519411087036
node_152: feature_name=cg07344990	feature_id[411].value > threshold=0.5484964847564697
node_158: feature_name=cg10298992	feature_id[3066].value <= threshold=0.5707973837852478
node_159: feature_name=cg24852135	feature_id[47].value <= threshold=0.8445721566677094
node_160: feature_name=cg25606046	feature_id[2007].value <= threshold=0.8722946643829346
node_161: feature_name=cg03212620	feature_id[1188].value > threshold=0.4703744798898697
node_163: feature_name=cg27096087	feature_id[2512].value > threshold=0.8232799470424652
node_165: feature_name=cg26963844	feature_id[175].value <= threshold=0.05642275512218475
Class: high-grade conventional osteosarcoma (OS (HG))	
Rules_151	passed counts:1
node_0: feature_name=cg11915444	feature_id[1171].value > threshold=0.3402601182460785
node_10: feature_name=cg12109728	feature_id[2810].value > threshold=0.7068270146846771
node_20: feature_name=cg16412000	feature_id[789].value > threshold=0.6741803884506226
node_24: feature_name=cg17115147	feature_id[1434].value <= threshold=0.2961975038051605

node_25: feature_name=cg24474622	feature_id[2737].value > threshold=0.7211934924125671
node_29: feature_name=cg10472711	feature_id[612].value > threshold=0.4598592966794967 7
node_37: feature_name=cg01937669	feature_id[188].value <= threshold=0.4809463769197464
node_38: feature_name=cg02157052	feature_id[451].value <= threshold=0.2869153320789337
node_39: feature_name=cg18751958	feature_id[1427].value > threshold=0.4813787043094635
node_41: feature_name=cg22512847	feature_id[1152].value > threshold=0.5129324495792389
node_43: feature_name=cg23345038	feature_id[1147].value > threshold=0.6318148374557495
node_45: feature_name=cg17439009	feature_id[1815].value <= threshold=0.48514117300510406
node_46: feature_name=cg17537493	feature_id[2772].value > threshold=0.5825372636318207
node_52: feature_name=cg01683570	feature_id[110].value > threshold=0.6040188074111938
node_54: feature_name=cg15720017	feature_id[168].value > threshold=0.7067741453647614
node_60: feature_name=cg23690444	feature_id[120].value <= threshold=0.16037724912166595
node_61: feature_name=cg00567872	feature_id[236].value > threshold=0.7005765736103058
node_85: feature_name=cg01014262	feature_id[764].value <= threshold=0.5737917125225067
node_86: feature_name=cg22946562	feature_id[1148].value > threshold=0.6328203976154327
node_134: feature_name=cg14514032	feature_id[202].value <= threshold=0.4389604330062866
node_135: feature_name=cg13488220	feature_id[241].value > threshold=0.7352153658866882
node_143: feature_name=cg03840920	feature_id[256].value > threshold=0.502630889415741
node_147: feature_name=cg13634090	feature_id[822].value > threshold=0.7207068204879761
node_151: feature_name=cg19584674	feature_id[2364].value <= threshold=0.41758519411087036
node_152: feature_name=cg07344990	feature_id[411].value <= threshold=0.5484964847564697
node_153: feature_name=cg18369990	feature_id[2197].value <= threshold=0.621816486120224

node_154: feature_name=cg14560240	feature_id[2397].value <= threshold=0.538348525762558
Class: sarcoma (SARC)	
Rules_152	passed counts:1
node_0: feature_name=cg11915444	feature_id[1171].value > threshold=0.3402601182460785
node_10: feature_name=cg12109728	feature_id[2810].value > threshold=0.7068270146846771
node_20: feature_name=cg16412000	feature_id[789].value > threshold=0.6741803884506226
node_24: feature_name=cg17115147	feature_id[1434].value <= threshold=0.2961975038051605
node_25: feature_name=cg24474622	feature_id[2737].value > threshold=0.7211934924125671
node_29: feature_name=cg10472711	feature_id[612].value > threshold=0.4598592967949677
node_37: feature_name=cg01937669	feature_id[188].value <= threshold=0.4809463769197464
node_38: feature_name=cg02157052	feature_id[451].value <= threshold=0.2869153320789337
node_39: feature_name=cg18751958	feature_id[1427].value > threshold=0.4813787043094635
node_41: feature_name=cg22512847	feature_id[1152].value > threshold=0.5129324495792389
node_43: feature_name=cg23345038	feature_id[1147].value > threshold=0.6318148374557495
node_45: feature_name=cg17439009	feature_id[1815].value <= threshold=0.48514117300510406
node_46: feature_name=cg17537493	feature_id[2772].value > threshold=0.5825372636318207
node_52: feature_name=cg01683570	feature_id[110].value > threshold=0.6040188074111938
node_54: feature_name=cg15720017	feature_id[168].value > threshold=0.7067741453647614
node_60: feature_name=cg23690444	feature_id[120].value <= threshold=0.16037724912166595
node_61: feature_name=cg00567872	feature_id[236].value > threshold=0.7005765736103058
node_85: feature_name=cg01014262	feature_id[764].value <= threshold=0.5737917125225067
node_86: feature_name=cg22946562	feature_id[1148].value > threshold=0.6328203976154327

node_134: feature_name=cg14514032	feature_id[202].value <= threshold=0.4389604330062866
node_135: feature_name=cg13488220	feature_id[241].value > threshold=0.7352153658866882
node_143: feature_name=cg03840920	feature_id[256].value > threshold=0.502630889415741
node_147: feature_name=cg13634090	feature_id[822].value <= threshold=0.7207068204879761
node_148: feature_name=cg21698310	feature_id[178].value <= threshold=0.07961717247962952
Class: osteoblastoma (OB)	
Rules_153	passed counts:1
node_0: feature_name=cg11915444	feature_id[1171].value > threshold=0.3402601182460785
node_10: feature_name=cg12109728	feature_id[2810].value > threshold=0.7068270146846771
node_20: feature_name=cg16412000	feature_id[789].value > threshold=0.6741803884506226
node_24: feature_name=cg17115147	feature_id[1434].value <= threshold=0.2961975038051605
node_25: feature_name=cg24474622	feature_id[2737].value > threshold=0.7211934924125671
node_29: feature_name=cg10472711	feature_id[612].value > threshold=0.45985929667949677
node_37: feature_name=cg01937669	feature_id[188].value <= threshold=0.4809463769197464
node_38: feature_name=cg02157052	feature_id[451].value <= threshold=0.2869153320789337
node_39: feature_name=cg18751958	feature_id[1427].value > threshold=0.4813787043094635
node_41: feature_name=cg22512847	feature_id[1152].value > threshold=0.5129324495792389
node_43: feature_name=cg23345038	feature_id[1147].value > threshold=0.6318148374557495
node_45: feature_name=cg17439009	feature_id[1815].value <= threshold=0.48514117300510406
node_46: feature_name=cg17537493	feature_id[2772].value > threshold=0.5825372636318207
node_52: feature_name=cg01683570	feature_id[110].value > threshold=0.6040188074111938
node_54: feature_name=cg15720017	feature_id[168].value > threshold=0.7067741453647614

node_60: feature_name=cg23690444	feature_id[120].value <= threshold=0.16037724912166595
node_61: feature_name=cg00567872	feature_id[236].value > threshold=0.7005765736103058
node_85: feature_name=cg01014262	feature_id[764].value <= threshold=0.5737917125225067
node_86: feature_name=cg22946562	feature_id[1148].value > threshold=0.6328203976154327
node_134: feature_name=cg14514032	feature_id[202].value <= threshold=0.4389604330062866
node_135: feature_name=cg13488220	feature_id[241].value > threshold=0.7352153658866882
node_143: feature_name=cg03840920	feature_id[256].value <= threshold=0.502630889415741
node_144: feature_name=cg12669088	feature_id[444].value > threshold=0.8584775626659393
Class: low-grade fibromyxoid sarcoma (LGFMS)	
Rules_154	passed counts:1
node_0: feature_name=cg11915444	feature_id[1171].value > threshold=0.3402601182460785
node_10: feature_name=cg12109728	feature_id[2810].value > threshold=0.7068270146846771
node_20: feature_name=cg16412000	feature_id[789].value > threshold=0.6741803884506226
node_24: feature_name=cg17115147	feature_id[1434].value <= threshold=0.2961975038051605
node_25: feature_name=cg24474622	feature_id[2737].value > threshold=0.7211934924125671
node_29: feature_name=cg10472711	feature_id[612].value > threshold=0.45985929667949677
node_37: feature_name=cg01937669	feature_id[188].value <= threshold=0.4809463769197464
node_38: feature_name=cg02157052	feature_id[451].value <= threshold=0.2869153320789337
node_39: feature_name=cg18751958	feature_id[1427].value > threshold=0.4813787043094635
node_41: feature_name=cg22512847	feature_id[1152].value > threshold=0.5129324495792389
node_43: feature_name=cg23345038	feature_id[1147].value > threshold=0.6318148374557495
node_45: feature_name=cg17439009	feature_id[1815].value <= threshold=0.48514117300510406

node_46: feature_name=cg17537493	feature_id[2772].value > threshold=0.5825372636318207
node_52: feature_name=cg01683570	feature_id[110].value > threshold=0.6040188074111938
node_54: feature_name=cg15720017	feature_id[168].value > threshold=0.7067741453647614
node_60: feature_name=cg23690444	feature_id[120].value <= threshold=0.16037724912166595
node_61: feature_name=cg00567872	feature_id[236].value > threshold=0.7005765736103058
node_85: feature_name=cg01014262	feature_id[764].value <= threshold=0.5737917125225067
node_86: feature_name=cg22946562	feature_id[1148].value > threshold=0.6328203976154327
node_134: feature_name=cg14514032	feature_id[202].value <= threshold=0.4389604330062866
node_135: feature_name=cg13488220	feature_id[241].value <= threshold=0.7352153658866882
node_136: feature_name=cg06132803	feature_id[3655].value <= threshold=0.29061736911535263
node_137: feature_name=cg08692006	feature_id[2437].value > threshold=0.25290777534246445
node_139: feature_name=cg04384810	feature_id[756].value > threshold=0.37209058180451393
Class: synovial sarcoma (SYSA)	
Rules_155	passed counts:1
node_0: feature_name=cg11915444	feature_id[1171].value > threshold=0.3402601182460785
node_10: feature_name=cg12109728	feature_id[2810].value > threshold=0.7068270146846771
node_20: feature_name=cg16412000	feature_id[789].value > threshold=0.6741803884506226
node_24: feature_name=cg17115147	feature_id[1434].value <= threshold=0.2961975038051605
node_25: feature_name=cg24474622	feature_id[2737].value > threshold=0.7211934924125671
node_29: feature_name=cg10472711	feature_id[612].value > threshold=0.45985929667949677
node_37: feature_name=cg01937669	feature_id[188].value <= threshold=0.4809463769197464
node_38: feature_name=cg02157052	feature_id[451].value <= threshold=0.2869153320789337

node_39: feature_name=cg18751958	feature_id[1427].value > threshold=0.4813787043094635
node_41: feature_name=cg22512847	feature_id[1152].value > threshold=0.5129324495792389
node_43: feature_name=cg23345038	feature_id[1147].value > threshold=0.6318148374557495
node_45: feature_name=cg17439009	feature_id[1815].value <= threshold=0.48514117300510406
node_46: feature_name=cg17537493	feature_id[2772].value > threshold=0.5825372636318207
node_52: feature_name=cg01683570	feature_id[110].value > threshold=0.6040188074111938
node_54: feature_name=cg15720017	feature_id[168].value > threshold=0.7067741453647614
node_60: feature_name=cg23690444	feature_id[120].value <= threshold=0.16037724912166595
node_61: feature_name=cg00567872	feature_id[236].value > threshold=0.7005765736103058
node_85: feature_name=cg01014262	feature_id[764].value <= threshold=0.5737917125225067
node_86: feature_name=cg22946562	feature_id[1148].value > threshold=0.6328203976154327
node_134: feature_name=cg14514032	feature_id[202].value <= threshold=0.4389604330062866
node_135: feature_name=cg13488220	feature_id[241].value <= threshold=0.7352153658866882
node_136: feature_name=cg06132803	feature_id[3655].value <= threshold=0.29061736911535263
node_137: feature_name=cg08692006	feature_id[2437].value > threshold=0.25290777534246445
node_139: feature_name=cg04384810	feature_id[756].value <= threshold=0.37209058180451393
Class: malignant peripheral nerve sheath tumour (MPNST)	
Rules_156	passed counts:1
node_0: feature_name=cg11915444	feature_id[1171].value > threshold=0.3402601182460785
node_10: feature_name=cg12109728	feature_id[2810].value > threshold=0.7068270146846771
node_20: feature_name=cg16412000	feature_id[789].value > threshold=0.6741803884506226

node_24: feature_name=cg17115147	feature_id[1434].value <= threshold=0.2961975038051605
node_25: feature_name=cg24474622	feature_id[2737].value > threshold=0.7211934924125671
node_29: feature_name=cg10472711	feature_id[612].value > threshold=0.4598592967949677
node_37: feature_name=cg01937669	feature_id[188].value <= threshold=0.4809463769197464
node_38: feature_name=cg02157052	feature_id[451].value <= threshold=0.2869153320789337
node_39: feature_name=cg18751958	feature_id[1427].value > threshold=0.4813787043094635
node_41: feature_name=cg22512847	feature_id[1152].value > threshold=0.5129324495792389
node_43: feature_name=cg23345038	feature_id[1147].value > threshold=0.6318148374557495
node_45: feature_name=cg17439009	feature_id[1815].value <= threshold=0.48514117300510406
node_46: feature_name=cg17537493	feature_id[2772].value > threshold=0.5825372636318207
node_52: feature_name=cg01683570	feature_id[110].value > threshold=0.6040188074111938
node_54: feature_name=cg15720017	feature_id[168].value > threshold=0.7067741453647614
node_60: feature_name=cg23690444	feature_id[120].value <= threshold=0.16037724912166595
node_61: feature_name=cg00567872	feature_id[236].value > threshold=0.7005765736103058
node_85: feature_name=cg01014262	feature_id[764].value <= threshold=0.5737917125225067
node_86: feature_name=cg22946562	feature_id[1148].value > threshold=0.6328203976154327
node_134: feature_name=cg14514032	feature_id[202].value <= threshold=0.4389604330062866
node_135: feature_name=cg13488220	feature_id[241].value <= threshold=0.7352153658866882
node_136: feature_name=cg06132803	feature_id[3655].value <= threshold=0.29061736911535263
node_137: feature_name=cg08692006	feature_id[2437].value <= threshold=0.25290777534246445
Class: fibrous dysplasia (FDY)	
Rules_157	passed counts:1

node_0: feature_name=cg11915444	feature_id[1171].value > threshold=0.3402601182460785
node_10: feature_name=cg12109728	feature_id[2810].value > threshold=0.7068270146846771
node_20: feature_name=cg16412000	feature_id[789].value > threshold=0.6741803884506226
node_24: feature_name=cg17115147	feature_id[1434].value <= threshold=0.2961975038051605
node_25: feature_name=cg24474622	feature_id[2737].value > threshold=0.7211934924125671
node_29: feature_name=cg10472711	feature_id[612].value > threshold=0.45985929667949677
node_37: feature_name=cg01937669	feature_id[188].value <= threshold=0.4809463769197464
node_38: feature_name=cg02157052	feature_id[451].value <= threshold=0.2869153320789337
node_39: feature_name=cg18751958	feature_id[1427].value > threshold=0.4813787043094635
node_41: feature_name=cg22512847	feature_id[1152].value > threshold=0.5129324495792389
node_43: feature_name=cg23345038	feature_id[1147].value > threshold=0.6318148374557495
node_45: feature_name=cg17439009	feature_id[1815].value <= threshold=0.48514117300510406
node_46: feature_name=cg17537493	feature_id[2772].value > threshold=0.5825372636318207
node_52: feature_name=cg01683570	feature_id[110].value > threshold=0.6040188074111938
node_54: feature_name=cg15720017	feature_id[168].value > threshold=0.7067741453647614
node_60: feature_name=cg23690444	feature_id[120].value <= threshold=0.16037724912166595
node_61: feature_name=cg00567872	feature_id[236].value > threshold=0.7005765736103058
node_85: feature_name=cg01014262	feature_id[764].value <= threshold=0.5737917125225067
node_86: feature_name=cg22946562	feature_id[1148].value <= threshold=0.6328203976154327
node_87: feature_name=cg18120975	feature_id[2767].value > threshold=0.7081544995307922
node_91: feature_name=cg17171539	feature_id[25].value > threshold=0.501024603843689
node_111: feature_name=cg21241424	feature_id[1155].value <= threshold=0.7665272355079651

node_112: feature_name=cg21897315	feature_id[3800].value > threshold=0.48165470361709595
node_114: feature_name=cg04375492	feature_id[2874].value > threshold=0.7812598049640656
node_116: feature_name=cg09892984	feature_id[3615].value > threshold=0.6039115190505981
node_118: feature_name=cg05076082	feature_id[215].value > threshold=0.7612724006175995
node_120: feature_name=cg15776300	feature_id[3872].value <= threshold=0.922246515750885
node_121: feature_name=cg12109728	feature_id[2810].value > threshold=0.8809287846088409
node_123: feature_name=cg24482246	feature_id[2019].value > threshold=0.6093599200248718
node_125: feature_name=cg22221847	feature_id[3484].value > threshold=0.5291177108883858
node_127: feature_name=cg25950520	feature_id[771].value > threshold=0.7284954488277435
node_129: feature_name=cg26937267	feature_id[2919].value > threshold=0.8173473179340363
Class: inflammatory myofibroblastic tumour (IMT)	
Rules_158	passed counts:1
node_0: feature_name=cg11915444	feature_id[1171].value > threshold=0.3402601182460785
node_10: feature_name=cg12109728	feature_id[2810].value > threshold=0.7068270146846771
node_20: feature_name=cg16412000	feature_id[789].value > threshold=0.6741803884506226
node_24: feature_name=cg17115147	feature_id[1434].value <= threshold=0.2961975038051605
node_25: feature_name=cg24474622	feature_id[2737].value > threshold=0.7211934924125671
node_29: feature_name=cg10472711	feature_id[612].value > threshold=0.45985929667949677
node_37: feature_name=cg01937669	feature_id[188].value <= threshold=0.4809463769197464
node_38: feature_name=cg02157052	feature_id[451].value <= threshold=0.2869153320789337
node_39: feature_name=cg18751958	feature_id[1427].value > threshold=0.4813787043094635

node_41: feature_name=cg22512847	feature_id[1152].value > threshold=0.5129324495792389
node_43: feature_name=cg23345038	feature_id[1147].value > threshold=0.6318148374557495
node_45: feature_name=cg17439009	feature_id[1815].value <= threshold=0.48514117300510406
node_46: feature_name=cg17537493	feature_id[2772].value > threshold=0.5825372636318207
node_52: feature_name=cg01683570	feature_id[110].value > threshold=0.6040188074111938
node_54: feature_name=cg15720017	feature_id[168].value > threshold=0.7067741453647614
node_60: feature_name=cg23690444	feature_id[120].value <= threshold=0.16037724912166595
node_61: feature_name=cg00567872	feature_id[236].value > threshold=0.7005765736103058
node_85: feature_name=cg01014262	feature_id[764].value <= threshold=0.5737917125225067
node_86: feature_name=cg22946562	feature_id[1148].value <= threshold=0.6328203976154327
node_87: feature_name=cg18120975	feature_id[2767].value > threshold=0.7081544995307922
node_91: feature_name=cg17171539	feature_id[25].value > threshold=0.501024603843689
node_111: feature_name=cg21241424	feature_id[1155].value <= threshold=0.7665272355079651
node_112: feature_name=cg21897315	feature_id[3800].value > threshold=0.48165470361709595
node_114: feature_name=cg04375492	feature_id[2874].value > threshold=0.7812598049640656
node_116: feature_name=cg09892984	feature_id[3615].value > threshold=0.6039115190505981
node_118: feature_name=cg05076082	feature_id[215].value > threshold=0.7612724006175995
node_120: feature_name=cg15776300	feature_id[3872].value <= threshold=0.922246515750885
node_121: feature_name=cg12109728	feature_id[2810].value > threshold=0.8809287846088409
node_123: feature_name=cg24482246	feature_id[2019].value > threshold=0.6093599200248718
node_125: feature_name=cg22221847	feature_id[3484].value > threshold=0.5291177108883858
node_127: feature_name=cg25950520	feature_id[771].value > threshold=0.7284954488277435

node_129: feature_name=cg26937267	feature_id[2919].value <= threshold=0.8173473179340363
Class: giant cell tumour of bone (GCTB)	
Rules_159	passed counts:1
node_0: feature_name=cg11915444	feature_id[1171].value > threshold=0.3402601182460785
node_10: feature_name=cg12109728	feature_id[2810].value > threshold=0.7068270146846771
node_20: feature_name=cg16412000	feature_id[789].value > threshold=0.6741803884506226
node_24: feature_name=cg17115147	feature_id[1434].value <= threshold=0.2961975038051605
node_25: feature_name=cg24474622	feature_id[2737].value > threshold=0.7211934924125671
node_29: feature_name=cg10472711	feature_id[612].value > threshold=0.4598592967949677
node_37: feature_name=cg01937669	feature_id[188].value <= threshold=0.4809463769197464
node_38: feature_name=cg02157052	feature_id[451].value <= threshold=0.2869153320789337
node_39: feature_name=cg18751958	feature_id[1427].value > threshold=0.4813787043094635
node_41: feature_name=cg22512847	feature_id[1152].value > threshold=0.5129324495792389
node_43: feature_name=cg23345038	feature_id[1147].value > threshold=0.6318148374557495
node_45: feature_name=cg17439009	feature_id[1815].value <= threshold=0.48514117300510406
node_46: feature_name=cg17537493	feature_id[2772].value > threshold=0.5825372636318207
node_52: feature_name=cg01683570	feature_id[110].value > threshold=0.6040188074111938
node_54: feature_name=cg15720017	feature_id[168].value > threshold=0.7067741453647614
node_60: feature_name=cg23690444	feature_id[120].value <= threshold=0.16037724912166595
node_61: feature_name=cg00567872	feature_id[236].value > threshold=0.7005765736103058
node_85: feature_name=cg01014262	feature_id[764].value <= threshold=0.5737917125225067
node_86: feature_name=cg22946562	feature_id[1148].value <= threshold=0.6328203976154327

node_87: feature_name=cg18120975	feature_id[2767].value > threshold=0.7081544995307922
node_91: feature_name=cg17171539	feature_id[25].value > threshold=0.501024603843689
node_111: feature_name=cg21241424	feature_id[1155].value <= threshold=0.7665272355079651
node_112: feature_name=cg21897315	feature_id[3800].value > threshold=0.48165470361709595
node_114: feature_name=cg04375492	feature_id[2874].value > threshold=0.7812598049640656
node_116: feature_name=cg09892984	feature_id[3615].value > threshold=0.6039115190505981
node_118: feature_name=cg05076082	feature_id[215].value > threshold=0.7612724006175995
node_120: feature_name=cg15776300	feature_id[3872].value <= threshold=0.922246515750885
node_121: feature_name=cg12109728	feature_id[2810].value > threshold=0.8809287846088409
node_123: feature_name=cg24482246	feature_id[2019].value > threshold=0.6093599200248718
node_125: feature_name=cg22221847	feature_id[3484].value > threshold=0.5291177108883858
node_127: feature_name=cg25950520	feature_id[771].value <= threshold=0.7284954488277435
Class: sclerosing epithelioid fibrosarcoma(SEF)	
Rules_160	passed counts:1
node_0: feature_name=cg11915444	feature_id[1171].value > threshold=0.3402601182460785
node_10: feature_name=cg12109728	feature_id[2810].value > threshold=0.7068270146846771
node_20: feature_name=cg16412000	feature_id[789].value > threshold=0.6741803884506226
node_24: feature_name=cg17115147	feature_id[1434].value <= threshold=0.2961975038051605
node_25: feature_name=cg24474622	feature_id[2737].value > threshold=0.7211934924125671
node_29: feature_name=cg10472711	feature_id[612].value > threshold=0.45985929667949677
node_37: feature_name=cg01937669	feature_id[188].value <= threshold=0.4809463769197464
node_38: feature_name=cg02157052	feature_id[451].value <= threshold=0.2869153320789337

node_39: feature_name=cg18751958	feature_id[1427].value > threshold=0.4813787043094635
node_41: feature_name=cg22512847	feature_id[1152].value > threshold=0.5129324495792389
node_43: feature_name=cg23345038	feature_id[1147].value > threshold=0.6318148374557495
node_45: feature_name=cg17439009	feature_id[1815].value <= threshold=0.48514117300510406
node_46: feature_name=cg17537493	feature_id[2772].value > threshold=0.5825372636318207
node_52: feature_name=cg01683570	feature_id[110].value > threshold=0.6040188074111938
node_54: feature_name=cg15720017	feature_id[168].value > threshold=0.7067741453647614
node_60: feature_name=cg23690444	feature_id[120].value <= threshold=0.16037724912166595
node_61: feature_name=cg00567872	feature_id[236].value > threshold=0.7005765736103058
node_85: feature_name=cg01014262	feature_id[764].value <= threshold=0.5737917125225067
node_86: feature_name=cg22946562	feature_id[1148].value <= threshold=0.6328203976154327
node_87: feature_name=cg18120975	feature_id[2767].value > threshold=0.7081544995307922
node_91: feature_name=cg17171539	feature_id[25].value > threshold=0.501024603843689
node_111: feature_name=cg21241424	feature_id[1155].value <= threshold=0.7665272355079651
node_112: feature_name=cg21897315	feature_id[3800].value > threshold=0.48165470361709595
node_114: feature_name=cg04375492	feature_id[2874].value > threshold=0.7812598049640656
node_116: feature_name=cg09892984	feature_id[3615].value > threshold=0.6039115190505981
node_118: feature_name=cg05076082	feature_id[215].value > threshold=0.7612724006175995
node_120: feature_name=cg15776300	feature_id[3872].value <= threshold=0.922246515750885
node_121: feature_name=cg12109728	feature_id[2810].value > threshold=0.8809287846088409
node_123: feature_name=cg24482246	feature_id[2019].value > threshold=0.6093599200248718
node_125: feature_name=cg22221847	feature_id[3484].value <= threshold=0.5291177108883858

Class: Ewing sarcoma (EWING)	
Rules_161	passed counts:1
node_0: feature_name=cg11915444	feature_id[1171].value > threshold=0.3402601182460785
node_10: feature_name=cg12109728	feature_id[2810].value > threshold=0.7068270146846771
node_20: feature_name=cg16412000	feature_id[789].value > threshold=0.6741803884506226
node_24: feature_name=cg17115147	feature_id[1434].value <= threshold=0.2961975038051605
node_25: feature_name=cg24474622	feature_id[2737].value > threshold=0.7211934924125671
node_29: feature_name=cg10472711	feature_id[612].value > threshold=0.45985929667949677
node_37: feature_name=cg01937669	feature_id[188].value <= threshold=0.4809463769197464
node_38: feature_name=cg02157052	feature_id[451].value <= threshold=0.2869153320789337
node_39: feature_name=cg18751958	feature_id[1427].value > threshold=0.4813787043094635
node_41: feature_name=cg22512847	feature_id[1152].value > threshold=0.5129324495792389
node_43: feature_name=cg23345038	feature_id[1147].value > threshold=0.6318148374557495
node_45: feature_name=cg17439009	feature_id[1815].value <= threshold=0.48514117300510406
node_46: feature_name=cg17537493	feature_id[2772].value > threshold=0.5825372636318207
node_52: feature_name=cg01683570	feature_id[110].value > threshold=0.6040188074111938
node_54: feature_name=cg15720017	feature_id[168].value > threshold=0.7067741453647614
node_60: feature_name=cg23690444	feature_id[120].value <= threshold=0.16037724912166595
node_61: feature_name=cg00567872	feature_id[236].value > threshold=0.7005765736103058
node_85: feature_name=cg01014262	feature_id[764].value <= threshold=0.5737917125225067
node_86: feature_name=cg22946562	feature_id[1148].value <= threshold=0.6328203976154327
node_87: feature_name=cg18120975	feature_id[2767].value > threshold=0.7081544995307922

node_91: feature_name=cg17171539	feature_id[25].value > threshold=0.501024603843689
node_111: feature_name=cg21241424	feature_id[1155].value <= threshold=0.7665272355079651
node_112: feature_name=cg21897315	feature_id[3800].value > threshold=0.48165470361709595
node_114: feature_name=cg04375492	feature_id[2874].value > threshold=0.7812598049640656
node_116: feature_name=cg09892984	feature_id[3615].value > threshold=0.6039115190505981
node_118: feature_name=cg05076082	feature_id[215].value > threshold=0.7612724006175995
node_120: feature_name=cg15776300	feature_id[3872].value <= threshold=0.922246515750885
node_121: feature_name=cg12109728	feature_id[2810].value > threshold=0.8809287846088409
node_123: feature_name=cg24482246	feature_id[2019].value <= threshold=0.6093599200248718
Class: well differentiated liposarcoma (WDLS)/dedifferentiated liposarcoma (DDLS)	
Rules_162	passed counts:1
node_0: feature_name=cg11915444	feature_id[1171].value > threshold=0.3402601182460785
node_10: feature_name=cg12109728	feature_id[2810].value > threshold=0.7068270146846771
node_20: feature_name=cg16412000	feature_id[789].value > threshold=0.6741803884506226
node_24: feature_name=cg17115147	feature_id[1434].value <= threshold=0.2961975038051605
node_25: feature_name=cg24474622	feature_id[2737].value > threshold=0.7211934924125671
node_29: feature_name=cg10472711	feature_id[612].value > threshold=0.45985929667949677
node_37: feature_name=cg01937669	feature_id[188].value <= threshold=0.4809463769197464
node_38: feature_name=cg02157052	feature_id[451].value <= threshold=0.2869153320789337
node_39: feature_name=cg18751958	feature_id[1427].value > threshold=0.4813787043094635
node_41: feature_name=cg22512847	feature_id[1152].value > threshold=0.5129324495792389

node_43: feature_name=cg23345038	feature_id[1147].value > threshold=0.6318148374557495
node_45: feature_name=cg17439009	feature_id[1815].value <= threshold=0.48514117300510406
node_46: feature_name=cg17537493	feature_id[2772].value > threshold=0.5825372636318207
node_52: feature_name=cg01683570	feature_id[110].value > threshold=0.6040188074111938
node_54: feature_name=cg15720017	feature_id[168].value > threshold=0.7067741453647614
node_60: feature_name=cg23690444	feature_id[120].value <= threshold=0.16037724912166595
node_61: feature_name=cg00567872	feature_id[236].value > threshold=0.7005765736103058
node_85: feature_name=cg01014262	feature_id[764].value <= threshold=0.5737917125225067
node_86: feature_name=cg22946562	feature_id[1148].value <= threshold=0.6328203976154327
node_87: feature_name=cg18120975	feature_id[2767].value > threshold=0.7081544995307922
node_91: feature_name=cg17171539	feature_id[25].value <= threshold=0.501024603843689
node_92: feature_name=cg00007036	feature_id[1586].value > threshold=0.943472146987915
node_104: feature_name=cg14156441	feature_id[3273].value <= threshold=0.24960360676050186
node_105: feature_name=cg10885338	feature_id[3603].value > threshold=0.7335180938243866
node_107: feature_name=cg02197542	feature_id[220].value > threshold=0.700266033411026
Class: rhabdomyosarcoma (RMS)	
Rules_163	passed counts:1
node_0: feature_name=cg11915444	feature_id[1171].value > threshold=0.3402601182460785
node_10: feature_name=cg12109728	feature_id[2810].value > threshold=0.7068270146846771
node_20: feature_name=cg16412000	feature_id[789].value > threshold=0.6741803884506226
node_24: feature_name=cg17115147	feature_id[1434].value <= threshold=0.2961975038051605
node_25: feature_name=cg24474622	feature_id[2737].value > threshold=0.7211934924125671

node_29: feature_name=cg10472711	feature_id[612].value > threshold=0.45985929667949677
node_37: feature_name=cg01937669	feature_id[188].value <= threshold=0.4809463769197464
node_38: feature_name=cg02157052	feature_id[451].value <= threshold=0.2869153320789337
node_39: feature_name=cg18751958	feature_id[1427].value > threshold=0.4813787043094635
node_41: feature_name=cg22512847	feature_id[1152].value > threshold=0.5129324495792389
node_43: feature_name=cg23345038	feature_id[1147].value > threshold=0.6318148374557495
node_45: feature_name=cg17439009	feature_id[1815].value <= threshold=0.48514117300510406
node_46: feature_name=cg17537493	feature_id[2772].value > threshold=0.5825372636318207
node_52: feature_name=cg01683570	feature_id[110].value > threshold=0.6040188074111938
node_54: feature_name=cg15720017	feature_id[168].value > threshold=0.7067741453647614
node_60: feature_name=cg23690444	feature_id[120].value <= threshold=0.16037724912166595
node_61: feature_name=cg00567872	feature_id[236].value > threshold=0.7005765736103058
node_85: feature_name=cg01014262	feature_id[764].value <= threshold=0.5737917125225067
node_86: feature_name=cg22946562	feature_id[1148].value <= threshold=0.6328203976154327
node_87: feature_name=cg18120975	feature_id[2767].value > threshold=0.7081544995307922
node_91: feature_name=cg17171539	feature_id[25].value <= threshold=0.501024603843689
node_92: feature_name=cg00007036	feature_id[1586].value > threshold=0.943472146987915
node_104: feature_name=cg14156441	feature_id[3273].value <= threshold=0.24960360676050186
node_105: feature_name=cg10885338	feature_id[3603].value > threshold=0.7335180938243866
node_107: feature_name=cg02197542	feature_id[220].value <= threshold=0.700266033411026
Class: nodular fasciitis(NFA)	
Rules_164	passed counts:1

node_0: feature_name=cg11915444	feature_id[1171].value > threshold=0.3402601182460785
node_10: feature_name=cg12109728	feature_id[2810].value > threshold=0.7068270146846771
node_20: feature_name=cg16412000	feature_id[789].value > threshold=0.6741803884506226
node_24: feature_name=cg17115147	feature_id[1434].value <= threshold=0.2961975038051605
node_25: feature_name=cg24474622	feature_id[2737].value > threshold=0.7211934924125671
node_29: feature_name=cg10472711	feature_id[612].value > threshold=0.45985929667949677
node_37: feature_name=cg01937669	feature_id[188].value <= threshold=0.4809463769197464
node_38: feature_name=cg02157052	feature_id[451].value <= threshold=0.2869153320789337
node_39: feature_name=cg18751958	feature_id[1427].value > threshold=0.4813787043094635
node_41: feature_name=cg22512847	feature_id[1152].value > threshold=0.5129324495792389
node_43: feature_name=cg23345038	feature_id[1147].value > threshold=0.6318148374557495
node_45: feature_name=cg17439009	feature_id[1815].value <= threshold=0.48514117300510406
node_46: feature_name=cg17537493	feature_id[2772].value > threshold=0.5825372636318207
node_52: feature_name=cg01683570	feature_id[110].value > threshold=0.6040188074111938
node_54: feature_name=cg15720017	feature_id[168].value > threshold=0.7067741453647614
node_60: feature_name=cg23690444	feature_id[120].value <= threshold=0.16037724912166595
node_61: feature_name=cg00567872	feature_id[236].value > threshold=0.7005765736103058
node_85: feature_name=cg01014262	feature_id[764].value <= threshold=0.5737917125225067
node_86: feature_name=cg22946562	feature_id[1148].value <= threshold=0.6328203976154327
node_87: feature_name=cg18120975	feature_id[2767].value > threshold=0.7081544995307922
node_91: feature_name=cg17171539	feature_id[25].value <= threshold=0.501024603843689
node_92: feature_name=cg00007036	feature_id[1586].value <= threshold=0.943472146987915

node_93: feature_name=cg16570314	feature_id[479].value <= threshold=0.7333994507789612
node_94: feature_name=cg15117891	feature_id[2220].value <= threshold=0.9664478302001953
node_95: feature_name=cg00967711	feature_id[414].value > threshold=0.22273091971874237
node_97: feature_name=cg08642081	feature_id[2259].value > threshold=0.8612778782844543
node_99: feature_name=cg18749563	feature_id[3838].value > threshold=0.9820527732372284
Class: undifferentiated sarcoma (USARC)	
Rules_165	passed counts:1
node_0: feature_name=cg11915444	feature_id[1171].value > threshold=0.3402601182460785
node_10: feature_name=cg12109728	feature_id[2810].value > threshold=0.7068270146846771
node_20: feature_name=cg16412000	feature_id[789].value > threshold=0.6741803884506226
node_24: feature_name=cg17115147	feature_id[1434].value <= threshold=0.2961975038051605
node_25: feature_name=cg24474622	feature_id[2737].value > threshold=0.7211934924125671
node_29: feature_name=cg10472711	feature_id[612].value > threshold=0.45985929667949677
node_37: feature_name=cg01937669	feature_id[188].value <= threshold=0.4809463769197464
node_38: feature_name=cg02157052	feature_id[451].value <= threshold=0.2869153320789337
node_39: feature_name=cg18751958	feature_id[1427].value > threshold=0.4813787043094635
node_41: feature_name=cg22512847	feature_id[1152].value > threshold=0.5129324495792389
node_43: feature_name=cg23345038	feature_id[1147].value > threshold=0.6318148374557495
node_45: feature_name=cg17439009	feature_id[1815].value <= threshold=0.48514117300510406
node_46: feature_name=cg17537493	feature_id[2772].value > threshold=0.5825372636318207
node_52: feature_name=cg01683570	feature_id[110].value > threshold=0.6040188074111938
node_54: feature_name=cg15720017	feature_id[168].value > threshold=0.7067741453647614

node_60: feature_name=cg23690444	feature_id[120].value <= threshold=0.16037724912166595
node_61: feature_name=cg00567872	feature_id[236].value > threshold=0.7005765736103058
node_85: feature_name=cg01014262	feature_id[764].value <= threshold=0.5737917125225067
node_86: feature_name=cg22946562	feature_id[1148].value <= threshold=0.6328203976154327
node_87: feature_name=cg18120975	feature_id[2767].value > threshold=0.7081544995307922
node_91: feature_name=cg17171539	feature_id[25].value <= threshold=0.501024603843689
node_92: feature_name=cg00007036	feature_id[1586].value <= threshold=0.943472146987915
node_93: feature_name=cg16570314	feature_id[479].value <= threshold=0.7333994507789612
node_94: feature_name=cg15117891	feature_id[2220].value <= threshold=0.9664478302001953
node_95: feature_name=cg00967711	feature_id[414].value > threshold=0.22273091971874237
node_97: feature_name=cg08642081	feature_id[2259].value > threshold=0.8612778782844543
node_99: feature_name=cg18749563	feature_id[3838].value <= threshold=0.9820527732372284
Class: alveolar rhabdomyosarcoma (RMS (ALV))	
Rules_166	passed counts:1
node_0: feature_name=cg11915444	feature_id[1171].value > threshold=0.3402601182460785
node_10: feature_name=cg12109728	feature_id[2810].value > threshold=0.7068270146846771
node_20: feature_name=cg16412000	feature_id[789].value > threshold=0.6741803884506226
node_24: feature_name=cg17115147	feature_id[1434].value <= threshold=0.2961975038051605
node_25: feature_name=cg24474622	feature_id[2737].value > threshold=0.7211934924125671
node_29: feature_name=cg10472711	feature_id[612].value > threshold=0.45985929667949677
node_37: feature_name=cg01937669	feature_id[188].value <= threshold=0.4809463769197464

node_38: feature_name=cg02157052	feature_id[451].value <= threshold=0.2869153320789337
node_39: feature_name=cg18751958	feature_id[1427].value > threshold=0.4813787043094635
node_41: feature_name=cg22512847	feature_id[1152].value > threshold=0.5129324495792389
node_43: feature_name=cg23345038	feature_id[1147].value > threshold=0.6318148374557495
node_45: feature_name=cg17439009	feature_id[1815].value <= threshold=0.48514117300510406
node_46: feature_name=cg17537493	feature_id[2772].value > threshold=0.5825372636318207
node_52: feature_name=cg01683570	feature_id[110].value > threshold=0.6040188074111938
node_54: feature_name=cg15720017	feature_id[168].value > threshold=0.7067741453647614
node_60: feature_name=cg23690444	feature_id[120].value <= threshold=0.16037724912166595
node_61: feature_name=cg00567872	feature_id[236].value > threshold=0.7005765736103058
node_85: feature_name=cg01014262	feature_id[764].value <= threshold=0.5737917125225067
node_86: feature_name=cg22946562	feature_id[1148].value <= threshold=0.6328203976154327
node_87: feature_name=cg18120975	feature_id[2767].value > threshold=0.7081544995307922
node_91: feature_name=cg17171539	feature_id[25].value <= threshold=0.501024603843689
node_92: feature_name=cg00007036	feature_id[1586].value <= threshold=0.943472146987915
node_93: feature_name=cg16570314	feature_id[479].value <= threshold=0.7333994507789612
node_94: feature_name=cg15117891	feature_id[2220].value <= threshold=0.9664478302001953
node_95: feature_name=cg00967711	feature_id[414].value > threshold=0.22273091971874237
node_97: feature_name=cg08642081	feature_id[2259].value <= threshold=0.8612778782844543
Class: high-grade conventional osteosarcoma (OS (HG))	
Rules_167	passed counts:1

node_0: feature_name=cg11915444	feature_id[1171].value > threshold=0.3402601182460785
node_10: feature_name=cg12109728	feature_id[2810].value > threshold=0.7068270146846771
node_20: feature_name=cg16412000	feature_id[789].value > threshold=0.6741803884506226
node_24: feature_name=cg17115147	feature_id[1434].value <= threshold=0.2961975038051605
node_25: feature_name=cg24474622	feature_id[2737].value > threshold=0.7211934924125671
node_29: feature_name=cg10472711	feature_id[612].value > threshold=0.45985929667949677
node_37: feature_name=cg01937669	feature_id[188].value <= threshold=0.4809463769197464
node_38: feature_name=cg02157052	feature_id[451].value <= threshold=0.2869153320789337
node_39: feature_name=cg18751958	feature_id[1427].value > threshold=0.4813787043094635
node_41: feature_name=cg22512847	feature_id[1152].value > threshold=0.5129324495792389
node_43: feature_name=cg23345038	feature_id[1147].value > threshold=0.6318148374557495
node_45: feature_name=cg17439009	feature_id[1815].value <= threshold=0.48514117300510406
node_46: feature_name=cg17537493	feature_id[2772].value > threshold=0.5825372636318207
node_52: feature_name=cg01683570	feature_id[110].value > threshold=0.6040188074111938
node_54: feature_name=cg15720017	feature_id[168].value > threshold=0.7067741453647614
node_60: feature_name=cg23690444	feature_id[120].value <= threshold=0.16037724912166595
node_61: feature_name=cg00567872	feature_id[236].value > threshold=0.7005765736103058
node_85: feature_name=cg01014262	feature_id[764].value <= threshold=0.5737917125225067
node_86: feature_name=cg22946562	feature_id[1148].value <= threshold=0.6328203976154327
node_87: feature_name=cg18120975	feature_id[2767].value <= threshold=0.7081544995307922
node_88: feature_name=cg15592324	feature_id[383].value > threshold=0.9721506536006927
Class: chondroblastoma (CB)	

Rules_168	passed counts:1
node_0: feature_name=cg11915444	feature_id[1171].value > threshold=0.3402601182460785
node_10: feature_name=cg12109728	feature_id[2810].value > threshold=0.7068270146846771
node_20: feature_name=cg16412000	feature_id[789].value > threshold=0.6741803884506226
node_24: feature_name=cg17115147	feature_id[1434].value <= threshold=0.2961975038051605
node_25: feature_name=cg24474622	feature_id[2737].value > threshold=0.7211934924125671
node_29: feature_name=cg10472711	feature_id[612].value > threshold=0.45985929667949677
node_37: feature_name=cg01937669	feature_id[188].value <= threshold=0.4809463769197464
node_38: feature_name=cg02157052	feature_id[451].value <= threshold=0.2869153320789337
node_39: feature_name=cg18751958	feature_id[1427].value > threshold=0.4813787043094635
node_41: feature_name=cg22512847	feature_id[1152].value > threshold=0.5129324495792389
node_43: feature_name=cg23345038	feature_id[1147].value > threshold=0.6318148374557495
node_45: feature_name=cg17439009	feature_id[1815].value <= threshold=0.48514117300510406
node_46: feature_name=cg17537493	feature_id[2772].value > threshold=0.5825372636318207
node_52: feature_name=cg01683570	feature_id[110].value > threshold=0.6040188074111938
node_54: feature_name=cg15720017	feature_id[168].value > threshold=0.7067741453647614
node_60: feature_name=cg23690444	feature_id[120].value <= threshold=0.16037724912166595
node_61: feature_name=cg00567872	feature_id[236].value <= threshold=0.7005765736103058
node_62: feature_name=cg09293488	feature_id[2629].value > threshold=0.839823454618454
node_68: feature_name=cg04838832	feature_id[1184].value <= threshold=0.41919413208961487
node_69: feature_name=cg18153137	feature_id[2198].value <= threshold=0.08548648655414581

node_70: feature_name=cg18273464	feature_id[2051].value > threshold=0.8000432550907135
node_72: feature_name=cg18241094	feature_id[2373].value > threshold=0.3435447886586189
node_74: feature_name=cg23251296	feature_id[2342].value > threshold=0.36987772583961487
node_76: feature_name=cg05477920	feature_id[483].value > threshold=0.8196498453617096
node_78: feature_name=cg12092201	feature_id[1067].value > threshold=0.05550734885036945
node_80: feature_name=cg21450547	feature_id[439].value > threshold=0.1863430291414261
Class: embryonal rhabdomyosarcoma (RMS (EMB))	
Rules_169	passed counts:1
node_0: feature_name=cg11915444	feature_id[1171].value > threshold=0.3402601182460785
node_10: feature_name=cg12109728	feature_id[2810].value > threshold=0.7068270146846771
node_20: feature_name=cg16412000	feature_id[789].value > threshold=0.6741803884506226
node_24: feature_name=cg17115147	feature_id[1434].value <= threshold=0.2961975038051605
node_25: feature_name=cg24474622	feature_id[2737].value > threshold=0.7211934924125671
node_29: feature_name=cg10472711	feature_id[612].value > threshold=0.45985929667949677
node_37: feature_name=cg01937669	feature_id[188].value <= threshold=0.4809463769197464
node_38: feature_name=cg02157052	feature_id[451].value <= threshold=0.2869153320789337
node_39: feature_name=cg18751958	feature_id[1427].value > threshold=0.4813787043094635
node_41: feature_name=cg22512847	feature_id[1152].value > threshold=0.5129324495792389
node_43: feature_name=cg23345038	feature_id[1147].value > threshold=0.6318148374557495
node_45: feature_name=cg17439009	feature_id[1815].value <= threshold=0.48514117300510406
node_46: feature_name=cg17537493	feature_id[2772].value > threshold=0.5825372636318207

node_52: feature_name=cg01683570	feature_id[110].value > threshold=0.6040188074111938
node_54: feature_name=cg15720017	feature_id[168].value > threshold=0.7067741453647614
node_60: feature_name=cg23690444	feature_id[120].value <= threshold=0.16037724912166595
node_61: feature_name=cg00567872	feature_id[236].value <= threshold=0.7005765736103058
node_62: feature_name=cg09293488	feature_id[2629].value > threshold=0.839823454618454
node_68: feature_name=cg04838832	feature_id[1184].value <= threshold=0.41919413208961487
node_69: feature_name=cg18153137	feature_id[2198].value <= threshold=0.08548648655414581
node_70: feature_name=cg18273464	feature_id[2051].value > threshold=0.8000432550907135
node_72: feature_name=cg18241094	feature_id[2373].value > threshold=0.3435447886586189
node_74: feature_name=cg23251296	feature_id[2342].value > threshold=0.36987772583961487
node_76: feature_name=cg05477920	feature_id[483].value > threshold=0.8196498453617096
node_78: feature_name=cg12092201	feature_id[1067].value > threshold=0.05550734885036945
node_80: feature_name=cg21450547	feature_id[439].value <= threshold=0.1863430291414261
Class: angiomatoid fibrous histiocytoma (AFH)	
Rules_170	passed counts:1
node_0: feature_name=cg11915444	feature_id[1171].value > threshold=0.3402601182460785
node_10: feature_name=cg12109728	feature_id[2810].value > threshold=0.7068270146846771
node_20: feature_name=cg16412000	feature_id[789].value > threshold=0.6741803884506226
node_24: feature_name=cg17115147	feature_id[1434].value <= threshold=0.2961975038051605
node_25: feature_name=cg24474622	feature_id[2737].value > threshold=0.7211934924125671
node_29: feature_name=cg10472711	feature_id[612].value > threshold=0.45985929667949677
node_37: feature_name=cg01937669	feature_id[188].value <= threshold=0.4809463769197464

node_38: feature_name=cg02157052	feature_id[451].value <= threshold=0.2869153320789337
node_39: feature_name=cg18751958	feature_id[1427].value > threshold=0.4813787043094635
node_41: feature_name=cg22512847	feature_id[1152].value > threshold=0.5129324495792389
node_43: feature_name=cg23345038	feature_id[1147].value > threshold=0.6318148374557495
node_45: feature_name=cg17439009	feature_id[1815].value <= threshold=0.48514117300510406
node_46: feature_name=cg17537493	feature_id[2772].value > threshold=0.5825372636318207
node_52: feature_name=cg01683570	feature_id[110].value > threshold=0.6040188074111938
node_54: feature_name=cg15720017	feature_id[168].value > threshold=0.7067741453647614
node_60: feature_name=cg23690444	feature_id[120].value <= threshold=0.16037724912166595
node_61: feature_name=cg00567872	feature_id[236].value <= threshold=0.7005765736103058
node_62: feature_name=cg09293488	feature_id[2629].value > threshold=0.839823454618454
node_68: feature_name=cg04838832	feature_id[1184].value <= threshold=0.41919413208961487
node_69: feature_name=cg18153137	feature_id[2198].value <= threshold=0.08548648655414581
node_70: feature_name=cg18273464	feature_id[2051].value > threshold=0.8000432550907135
node_72: feature_name=cg18241094	feature_id[2373].value > threshold=0.3435447886586189
node_74: feature_name=cg23251296	feature_id[2342].value > threshold=0.36987772583961487
node_76: feature_name=cg05477920	feature_id[483].value > threshold=0.8196498453617096
node_78: feature_name=cg12092201	feature_id[1067].value <= threshold=0.05550734885036945
Class: rhabdomyosarcoma (RMS)	
Rules_171	passed counts:1
node_0: feature_name=cg11915444	feature_id[1171].value > threshold=0.3402601182460785
node_10: feature_name=cg12109728	feature_id[2810].value > threshold=0.7068270146846771

node_20: feature_name=cg16412000	feature_id[789].value > threshold=0.6741803884506226
node_24: feature_name=cg17115147	feature_id[1434].value <= threshold=0.2961975038051605
node_25: feature_name=cg24474622	feature_id[2737].value > threshold=0.7211934924125671
node_29: feature_name=cg10472711	feature_id[612].value > threshold=0.45985929667949677
node_37: feature_name=cg01937669	feature_id[188].value <= threshold=0.4809463769197464
node_38: feature_name=cg02157052	feature_id[451].value <= threshold=0.2869153320789337
node_39: feature_name=cg18751958	feature_id[1427].value > threshold=0.4813787043094635
node_41: feature_name=cg22512847	feature_id[1152].value > threshold=0.5129324495792389
node_43: feature_name=cg23345038	feature_id[1147].value > threshold=0.6318148374557495
node_45: feature_name=cg17439009	feature_id[1815].value <= threshold=0.48514117300510406
node_46: feature_name=cg17537493	feature_id[2772].value > threshold=0.5825372636318207
node_52: feature_name=cg01683570	feature_id[110].value > threshold=0.6040188074111938
node_54: feature_name=cg15720017	feature_id[168].value > threshold=0.7067741453647614
node_60: feature_name=cg23690444	feature_id[120].value <= threshold=0.16037724912166595
node_61: feature_name=cg00567872	feature_id[236].value <= threshold=0.7005765736103058
node_62: feature_name=cg09293488	feature_id[2629].value > threshold=0.839823454618454
node_68: feature_name=cg04838832	feature_id[1184].value <= threshold=0.41919413208961487
node_69: feature_name=cg18153137	feature_id[2198].value <= threshold=0.08548648655414581
node_70: feature_name=cg18273464	feature_id[2051].value > threshold=0.8000432550907135
node_72: feature_name=cg18241094	feature_id[2373].value > threshold=0.3435447886586189
node_74: feature_name=cg23251296	feature_id[2342].value > threshold=0.36987772583961487
node_76: feature_name=cg05477920	feature_id[483].value <= threshold=0.8196498453617096

Class: alveolar rhabdomyosarcoma (RMS (ALV))	
Rules_172	passed counts:1
node_0: feature_name=cg11915444	feature_id[1171].value > threshold=0.3402601182460785
node_10: feature_name=cg12109728	feature_id[2810].value > threshold=0.7068270146846771
node_20: feature_name=cg16412000	feature_id[789].value > threshold=0.6741803884506226
node_24: feature_name=cg17115147	feature_id[1434].value <= threshold=0.2961975038051605
node_25: feature_name=cg24474622	feature_id[2737].value > threshold=0.7211934924125671
node_29: feature_name=cg10472711	feature_id[612].value > threshold=0.45985929667949677
node_37: feature_name=cg01937669	feature_id[188].value <= threshold=0.4809463769197464
node_38: feature_name=cg02157052	feature_id[451].value <= threshold=0.2869153320789337
node_39: feature_name=cg18751958	feature_id[1427].value > threshold=0.4813787043094635
node_41: feature_name=cg22512847	feature_id[1152].value > threshold=0.5129324495792389
node_43: feature_name=cg23345038	feature_id[1147].value > threshold=0.6318148374557495
node_45: feature_name=cg17439009	feature_id[1815].value <= threshold=0.48514117300510406
node_46: feature_name=cg17537493	feature_id[2772].value > threshold=0.5825372636318207
node_52: feature_name=cg01683570	feature_id[110].value > threshold=0.6040188074111938
node_54: feature_name=cg15720017	feature_id[168].value > threshold=0.7067741453647614
node_60: feature_name=cg23690444	feature_id[120].value <= threshold=0.16037724912166595
node_61: feature_name=cg00567872	feature_id[236].value <= threshold=0.7005765736103058
node_62: feature_name=cg09293488	feature_id[2629].value > threshold=0.839823454618454
node_68: feature_name=cg04838832	feature_id[1184].value <= threshold=0.41919413208961487

node_69: feature_name=cg18153137	feature_id[2198].value <= threshold=0.08548648655414581
node_70: feature_name=cg18273464	feature_id[2051].value > threshold=0.8000432550907135
node_72: feature_name=cg18241094	feature_id[2373].value > threshold=0.3435447886586189
node_74: feature_name=cg23251296	feature_id[2342].value <= threshold=0.36987772583961487
Class: sarcoma (SARC)	
Rules_173	passed counts:1
node_0: feature_name=cg11915444	feature_id[1171].value > threshold=0.3402601182460785
node_10: feature_name=cg12109728	feature_id[2810].value > threshold=0.7068270146846771
node_20: feature_name=cg16412000	feature_id[789].value > threshold=0.6741803884506226
node_24: feature_name=cg17115147	feature_id[1434].value <= threshold=0.2961975038051605
node_25: feature_name=cg24474622	feature_id[2737].value > threshold=0.7211934924125671
node_29: feature_name=cg10472711	feature_id[612].value > threshold=0.45985929667949677
node_37: feature_name=cg01937669	feature_id[188].value <= threshold=0.4809463769197464
node_38: feature_name=cg02157052	feature_id[451].value <= threshold=0.2869153320789337
node_39: feature_name=cg18751958	feature_id[1427].value > threshold=0.4813787043094635
node_41: feature_name=cg22512847	feature_id[1152].value > threshold=0.5129324495792389
node_43: feature_name=cg23345038	feature_id[1147].value > threshold=0.6318148374557495
node_45: feature_name=cg17439009	feature_id[1815].value <= threshold=0.48514117300510406
node_46: feature_name=cg17537493	feature_id[2772].value > threshold=0.5825372636318207
node_52: feature_name=cg01683570	feature_id[110].value > threshold=0.6040188074111938
node_54: feature_name=cg15720017	feature_id[168].value > threshold=0.7067741453647614
node_60: feature_name=cg23690444	feature_id[120].value <= threshold=0.16037724912166595

node_61: feature_name=cg00567872	feature_id[236].value <= threshold=0.7005765736103058
node_62: feature_name=cg09293488	feature_id[2629].value > threshold=0.839823454618454
node_68: feature_name=cg04838832	feature_id[1184].value <= threshold=0.41919413208961487
node_69: feature_name=cg18153137	feature_id[2198].value <= threshold=0.08548648655414581
node_70: feature_name=cg18273464	feature_id[2051].value > threshold=0.8000432550907135
node_72: feature_name=cg18241094	feature_id[2373].value <= threshold=0.3435447886586189
Class: undifferentiated sarcoma (USARC)	
Rules_174	passed counts:1
node_0: feature_name=cg11915444	feature_id[1171].value > threshold=0.3402601182460785
node_10: feature_name=cg12109728	feature_id[2810].value > threshold=0.7068270146846771
node_20: feature_name=cg16412000	feature_id[789].value > threshold=0.6741803884506226
node_24: feature_name=cg17115147	feature_id[1434].value <= threshold=0.2961975038051605
node_25: feature_name=cg24474622	feature_id[2737].value > threshold=0.7211934924125671
node_29: feature_name=cg10472711	feature_id[612].value > threshold=0.45985929667949677
node_37: feature_name=cg01937669	feature_id[188].value <= threshold=0.4809463769197464
node_38: feature_name=cg02157052	feature_id[451].value <= threshold=0.2869153320789337
node_39: feature_name=cg18751958	feature_id[1427].value > threshold=0.4813787043094635
node_41: feature_name=cg22512847	feature_id[1152].value > threshold=0.5129324495792389
node_43: feature_name=cg23345038	feature_id[1147].value > threshold=0.6318148374557495
node_45: feature_name=cg17439009	feature_id[1815].value <= threshold=0.48514117300510406
node_46: feature_name=cg17537493	feature_id[2772].value > threshold=0.5825372636318207
node_52: feature_name=cg01683570	feature_id[110].value > threshold=0.6040188074111938

node_54: feature_name=cg15720017	feature_id[168].value > threshold=0.7067741453647614
node_60: feature_name=cg23690444	feature_id[120].value <= threshold=0.16037724912166595
node_61: feature_name=cg00567872	feature_id[236].value <= threshold=0.7005765736103058
node_62: feature_name=cg09293488	feature_id[2629].value > threshold=0.839823454618454
node_68: feature_name=cg04838832	feature_id[1184].value <= threshold=0.41919413208961487
node_69: feature_name=cg18153137	feature_id[2198].value <= threshold=0.08548648655414581
node_70: feature_name=cg18273464	feature_id[2051].value <= threshold=0.8000432550907135
Class: well differentiated liposarcoma (WDLS)/dedifferentiated liposarcoma (DDLS)	
Rules_175	passed counts:1
node_0: feature_name=cg11915444	feature_id[1171].value > threshold=0.3402601182460785
node_10: feature_name=cg12109728	feature_id[2810].value > threshold=0.7068270146846771
node_20: feature_name=cg16412000	feature_id[789].value > threshold=0.6741803884506226
node_24: feature_name=cg17115147	feature_id[1434].value <= threshold=0.2961975038051605
node_25: feature_name=cg24474622	feature_id[2737].value > threshold=0.7211934924125671
node_29: feature_name=cg10472711	feature_id[612].value > threshold=0.45985929667949677
node_37: feature_name=cg01937669	feature_id[188].value <= threshold=0.4809463769197464
node_38: feature_name=cg02157052	feature_id[451].value <= threshold=0.2869153320789337
node_39: feature_name=cg18751958	feature_id[1427].value > threshold=0.4813787043094635
node_41: feature_name=cg22512847	feature_id[1152].value > threshold=0.5129324495792389
node_43: feature_name=cg23345038	feature_id[1147].value > threshold=0.6318148374557495
node_45: feature_name=cg17439009	feature_id[1815].value <= threshold=0.48514117300510406

node_46: feature_name=cg17537493	feature_id[2772].value > threshold=0.5825372636318207
node_52: feature_name=cg01683570	feature_id[110].value > threshold=0.6040188074111938
node_54: feature_name=cg15720017	feature_id[168].value > threshold=0.7067741453647614
node_60: feature_name=cg23690444	feature_id[120].value <= threshold=0.16037724912166595
node_61: feature_name=cg00567872	feature_id[236].value <= threshold=0.7005765736103058
node_62: feature_name=cg09293488	feature_id[2629].value <= threshold=0.839823454618454
node_63: feature_name=cg19201770	feature_id[2194].value <= threshold=0.5603366196155548
node_64: feature_name=cg16172837	feature_id[1925].value > threshold=0.06044276803731918
Class: alveolar rhabdomyosarcoma (RMS (ALV))	
Rules_176	passed counts:1
node_0: feature_name=cg11915444	feature_id[1171].value > threshold=0.3402601182460785
node_10: feature_name=cg12109728	feature_id[2810].value > threshold=0.7068270146846771
node_20: feature_name=cg16412000	feature_id[789].value > threshold=0.6741803884506226
node_24: feature_name=cg17115147	feature_id[1434].value <= threshold=0.2961975038051605
node_25: feature_name=cg24474622	feature_id[2737].value > threshold=0.7211934924125671
node_29: feature_name=cg10472711	feature_id[612].value > threshold=0.45985929667949677
node_37: feature_name=cg01937669	feature_id[188].value <= threshold=0.4809463769197464
node_38: feature_name=cg02157052	feature_id[451].value <= threshold=0.2869153320789337
node_39: feature_name=cg18751958	feature_id[1427].value > threshold=0.4813787043094635
node_41: feature_name=cg22512847	feature_id[1152].value > threshold=0.5129324495792389
node_43: feature_name=cg23345038	feature_id[1147].value > threshold=0.6318148374557495

node_45: feature_name=cg17439009	feature_id[1815].value <= threshold=0.48514117300510406
node_46: feature_name=cg17537493	feature_id[2772].value > threshold=0.5825372636318207
node_52: feature_name=cg01683570	feature_id[110].value > threshold=0.6040188074111938
node_54: feature_name=cg15720017	feature_id[168].value > threshold=0.7067741453647614
node_60: feature_name=cg23690444	feature_id[120].value <= threshold=0.16037724912166595
node_61: feature_name=cg00567872	feature_id[236].value <= threshold=0.7005765736103058
node_62: feature_name=cg09293488	feature_id[2629].value <= threshold=0.839823454618454
node_63: feature_name=cg19201770	feature_id[2194].value <= threshold=0.5603366196155548
node_64: feature_name=cg16172837	feature_id[1925].value <= threshold=0.06044276803731918
Class: undifferentiated sarcoma (USARC)	
Rules_177	passed counts:1
node_0: feature_name=cg11915444	feature_id[1171].value > threshold=0.3402601182460785
node_10: feature_name=cg12109728	feature_id[2810].value > threshold=0.7068270146846771
node_20: feature_name=cg16412000	feature_id[789].value > threshold=0.6741803884506226
node_24: feature_name=cg17115147	feature_id[1434].value <= threshold=0.2961975038051605
node_25: feature_name=cg24474622	feature_id[2737].value > threshold=0.7211934924125671
node_29: feature_name=cg10472711	feature_id[612].value > threshold=0.45985929667949677
node_37: feature_name=cg01937669	feature_id[188].value <= threshold=0.4809463769197464
node_38: feature_name=cg02157052	feature_id[451].value <= threshold=0.2869153320789337
node_39: feature_name=cg18751958	feature_id[1427].value > threshold=0.4813787043094635
node_41: feature_name=cg22512847	feature_id[1152].value > threshold=0.5129324495792389
node_43: feature_name=cg23345038	feature_id[1147].value > threshold=0.6318148374557495

node_45: feature_name=cg17439009	feature_id[1815].value <= threshold=0.48514117300510406
node_46: feature_name=cg17537493	feature_id[2772].value > threshold=0.5825372636318207
node_52: feature_name=cg01683570	feature_id[110].value > threshold=0.6040188074111938
node_54: feature_name=cg15720017	feature_id[168].value <= threshold=0.7067741453647614
node_55: feature_name=cg17216243	feature_id[1006].value <= threshold=0.48612095415592194
node_56: feature_name=cg21863499	feature_id[2551].value > threshold=0.11701266095042229
Class: mesenchymal chondrosarcoma (CSA (MES))	
Rules_178	passed counts:1
node_0: feature_name=cg11915444	feature_id[1171].value > threshold=0.3402601182460785
node_10: feature_name=cg12109728	feature_id[2810].value > threshold=0.7068270146846771
node_20: feature_name=cg16412000	feature_id[789].value > threshold=0.6741803884506226
node_24: feature_name=cg17115147	feature_id[1434].value <= threshold=0.2961975038051605
node_25: feature_name=cg24474622	feature_id[2737].value > threshold=0.7211934924125671
node_29: feature_name=cg10472711	feature_id[612].value > threshold=0.45985929667949677
node_37: feature_name=cg01937669	feature_id[188].value <= threshold=0.4809463769197464
node_38: feature_name=cg02157052	feature_id[451].value <= threshold=0.2869153320789337
node_39: feature_name=cg18751958	feature_id[1427].value > threshold=0.4813787043094635
node_41: feature_name=cg22512847	feature_id[1152].value > threshold=0.5129324495792389
node_43: feature_name=cg23345038	feature_id[1147].value > threshold=0.6318148374557495
node_45: feature_name=cg17439009	feature_id[1815].value <= threshold=0.48514117300510406
node_46: feature_name=cg17537493	feature_id[2772].value > threshold=0.5825372636318207

node_52: feature_name=cg01683570	feature_id[110].value > threshold=0.6040188074111938
node_54: feature_name=cg15720017	feature_id[168].value <= threshold=0.7067741453647614
node_55: feature_name=cg17216243	feature_id[1006].value <= threshold=0.48612095415592194
node_56: feature_name=cg21863499	feature_id[2551].value <= threshold=0.11701266095042229
Class: gastrointestinal stromal tumour (GIST)	
Rules_179	passed counts:1
node_0: feature_name=cg11915444	feature_id[1171].value > threshold=0.3402601182460785
node_10: feature_name=cg12109728	feature_id[2810].value > threshold=0.7068270146846771
node_20: feature_name=cg16412000	feature_id[789].value > threshold=0.6741803884506226
node_24: feature_name=cg17115147	feature_id[1434].value <= threshold=0.2961975038051605
node_25: feature_name=cg24474622	feature_id[2737].value > threshold=0.7211934924125671
node_29: feature_name=cg10472711	feature_id[612].value > threshold=0.45985929667949677
node_37: feature_name=cg01937669	feature_id[188].value <= threshold=0.4809463769197464
node_38: feature_name=cg02157052	feature_id[451].value <= threshold=0.2869153320789337
node_39: feature_name=cg18751958	feature_id[1427].value > threshold=0.4813787043094635
node_41: feature_name=cg22512847	feature_id[1152].value > threshold=0.5129324495792389
node_43: feature_name=cg23345038	feature_id[1147].value > threshold=0.6318148374557495
node_45: feature_name=cg17439009	feature_id[1815].value <= threshold=0.48514117300510406
node_46: feature_name=cg17537493	feature_id[2772].value <= threshold=0.5825372636318207
node_47: feature_name=cg16572224	feature_id[2780].value <= threshold=0.6722612977027893
node_48: feature_name=cg00760872	feature_id[2909].value > threshold=0.46596916019916534
Class: epithelioid haemangioendothelioma (EHE)	

Rules_180	passed counts:1
node_0: feature_name=cg11915444	feature_id[1171].value > threshold=0.3402601182460785
node_10: feature_name=cg12109728	feature_id[2810].value > threshold=0.7068270146846771
node_20: feature_name=cg16412000	feature_id[789].value > threshold=0.6741803884506226
node_24: feature_name=cg17115147	feature_id[1434].value <= threshold=0.2961975038051605
node_25: feature_name=cg24474622	feature_id[2737].value > threshold=0.7211934924125671
node_29: feature_name=cg10472711	feature_id[612].value <= threshold=0.45985929667949677
node_30: feature_name=cg06132803	feature_id[3655].value <= threshold=0.420499712228775
node_31: feature_name=cg04248332	feature_id[3387].value <= threshold=0.6364206075668335
node_32: feature_name=cg05951828	feature_id[350].value > threshold=0.8983565866947174
Class: sclerosing epithelioid fibrosarcoma(SEF)	
Rules_181	passed counts:1
node_0: feature_name=cg11915444	feature_id[1171].value > threshold=0.3402601182460785
node_10: feature_name=cg12109728	feature_id[2810].value > threshold=0.7068270146846771
node_20: feature_name=cg16412000	feature_id[789].value > threshold=0.6741803884506226
node_24: feature_name=cg17115147	feature_id[1434].value <= threshold=0.2961975038051605
node_25: feature_name=cg24474622	feature_id[2737].value > threshold=0.7211934924125671
node_29: feature_name=cg10472711	feature_id[612].value <= threshold=0.45985929667949677
node_30: feature_name=cg06132803	feature_id[3655].value <= threshold=0.420499712228775
node_31: feature_name=cg04248332	feature_id[3387].value <= threshold=0.6364206075668335
node_32: feature_name=cg05951828	feature_id[350].value <= threshold=0.8983565866947174
Class: solitary fibrous tumour (SFT)	
Rules_182	passed counts:1

node_0: feature_name=cg11915444	feature_id[1171].value > threshold=0.3402601182460785
node_10: feature_name=cg12109728	feature_id[2810].value > threshold=0.7068270146846771
node_20: feature_name=cg16412000	feature_id[789].value > threshold=0.6741803884506226
node_24: feature_name=cg17115147	feature_id[1434].value <= threshold=0.2961975038051605
node_25: feature_name=cg24474622	feature_id[2737].value <= threshold=0.7211934924125671
node_26: feature_name=cg09828346	feature_id[2249].value > threshold=0.73759526014328
Class: gastrointestinal stromal tumour (GIST)	
Rules_183	passed counts:1
node_0: feature_name=cg11915444	feature_id[1171].value > threshold=0.3402601182460785
node_10: feature_name=cg12109728	feature_id[2810].value > threshold=0.7068270146846771
node_20: feature_name=cg16412000	feature_id[789].value <= threshold=0.6741803884506226
node_21: feature_name=cg08571020	feature_id[921].value > threshold=0.7741039395332336
Class: dermatofibrosarcoma protuberans (DFSP)	
Rules_184	passed counts:1
node_0: feature_name=cg11915444	feature_id[1171].value > threshold=0.3402601182460785
node_10: feature_name=cg12109728	feature_id[2810].value <= threshold=0.7068270146846771
node_11: feature_name=cg23902076	feature_id[1902].value > threshold=0.8691235482692719
node_13: feature_name=cg17174566	feature_id[2578].value <= threshold=0.8555663526058197
node_14: feature_name=cg22798362	feature_id[520].value > threshold=0.8689102232456207
node_16: feature_name=cg04884579	feature_id[1861].value > threshold=0.8975329697132111
Class: gastrointestinal stromal tumour (GIST)	
Rules_185	passed counts:1
node_0: feature_name=cg11915444	feature_id[1171].value > threshold=0.3402601182460785

node_10: feature_name=cg12109728	feature_id[2810].value <= threshold=0.7068270146846771
node_11: feature_name=cg23902076	feature_id[1902].value > threshold=0.8691235482692719
node_13: feature_name=cg17174566	feature_id[2578].value <= threshold=0.8555663526058197
node_14: feature_name=cg22798362	feature_id[520].value > threshold=0.8689102232456207
node_16: feature_name=cg04884579	feature_id[1861].value <= threshold=0.8975329697132111
Class: embryonal rhabdomyosarcoma (RMS (EMB))	
Rules_186	passed counts:1
node_0: feature_name=cg11915444	feature_id[1171].value > threshold=0.3402601182460785
node_10: feature_name=cg12109728	feature_id[2810].value <= threshold=0.7068270146846771
node_11: feature_name=cg23902076	feature_id[1902].value > threshold=0.8691235482692719
node_13: feature_name=cg17174566	feature_id[2578].value <= threshold=0.8555663526058197
node_14: feature_name=cg22798362	feature_id[520].value <= threshold=0.8689102232456207
Class: angiosarcoma (AS)	
Rules_187	passed counts:1
node_0: feature_name=cg11915444	feature_id[1171].value <= threshold=0.3402601182460785
node_1: feature_name=cg00204976	feature_id[3433].value > threshold=0.7535275816917419
node_3: feature_name=cg22663660	feature_id[38].value > threshold=0.5185286104679108
node_5: feature_name=cg26184501	feature_id[3451].value > threshold=0.5434053987264633
node_7: feature_name=cg14482093	feature_id[1629].value > threshold=0.5104826465249062
Class: angiosarcoma (AS)	
Rules_188	passed counts:1
node_0: feature_name=cg11915444	feature_id[1171].value <= threshold=0.3402601182460785

node_1: feature_name=cg00204976	feature_id[3433].value > threshold=0.7535275816917419
node_3: feature_name=cg22663660	feature_id[38].value > threshold=0.5185286104679108
node_5: feature_name=cg26184501	feature_id[3451].value > threshold=0.5434053987264633
node_7: feature_name=cg14482093	feature_id[1629].value <= threshold=0.5104826465249062
Class: infantile fibrosarcoma (IFS)	
Rules_189	passed counts:1
node_0: feature_name=cg11915444	feature_id[1171].value <= threshold=0.3402601182460785
node_1: feature_name=cg00204976	feature_id[3433].value > threshold=0.7535275816917419
node_3: feature_name=cg22663660	feature_id[38].value > threshold=0.5185286104679108
node_5: feature_name=cg26184501	feature_id[3451].value <= threshold=0.5434053987264633
Class: chordoma (CHORD)	

(3) Rules on the MCFS feature list

Rules_0	passed counts:77
node_0: feature_name=cg11915444	feature_id[2002].value > threshold=0.3402601182460785
node_10: feature_name=cg26016985	feature_id[4407].value > threshold=0.6493876278400421
node_16: feature_name=cg23157618	feature_id[5511].value > threshold=0.5759606957435608
node_20: feature_name=cg10480329	feature_id[1439].value <= threshold=0.5518321692943573
node_21: feature_name=cg25510609	feature_id[149].value > threshold=0.08151235431432724
node_39: feature_name=cg08726900	feature_id[250].value <= threshold=0.5245974063873291
Class: high-grade conventional osteosarcoma (OS (HG))	
Rules_1	passed counts:72
node_0: feature_name=cg11915444	feature_id[2002].value <= threshold=0.3402601182460785
node_1: feature_name=cg17156862	feature_id[2598].value > threshold=0.6409126222133636

Class: Ewing sarcoma (EWING)	
Rules_2	passed counts:65
node_0: feature_name=cg11915444	feature_id[2002].value > threshold=0.3402601182460785
node_10: feature_name=cg26016985	feature_id[4407].value <= threshold=0.6493876278400421
node_11: feature_name=cg14769121	feature_id[1178].value <= threshold=0.8216802477836609
Class: chordoma (CHORD)	
Rules_3	passed counts:64
node_0: feature_name=cg11915444	feature_id[2002].value > threshold=0.3402601182460785
node_10: feature_name=cg26016985	feature_id[4407].value > threshold=0.6493876278400421
node_16: feature_name=cg23157618	feature_id[5511].value > threshold=0.5759606957435608
node_20: feature_name=cg10480329	feature_id[1439].value > threshold=0.5518321692943573
node_48: feature_name=cg17843418	feature_id[4868].value > threshold=0.34969255328178406
node_52: feature_name=cg24407065	feature_id[4522].value > threshold=0.5364363789558411
node_56: feature_name=cg07281938	feature_id[249].value <= threshold=0.7506992518901825
node_57: feature_name=cg21189849	feature_id[1261].value <= threshold=0.7545044124126434
node_58: feature_name=cg11960033	feature_id[260].value > threshold=0.7065970599651337
Class: chondrosarcoma (CSA)	
Rules_4	passed counts:61
node_0: feature_name=cg11915444	feature_id[2002].value > threshold=0.3402601182460785
node_10: feature_name=cg26016985	feature_id[4407].value > threshold=0.6493876278400421
node_16: feature_name=cg23157618	feature_id[5511].value > threshold=0.5759606957435608
node_20: feature_name=cg10480329	feature_id[1439].value > threshold=0.5518321692943573
node_48: feature_name=cg17843418	feature_id[4868].value > threshold=0.34969255328178406

node_52: feature_name=cg24407065	feature_id[4522].value > threshold=0.5364363789558411
node_56: feature_name=cg07281938	feature_id[249].value > threshold=0.7506992518901825
node_78: feature_name=cg21759907	feature_id[1650].value <= threshold=0.46823520958423615
node_79: feature_name=cg25476129	feature_id[2302].value > threshold=0.1687404215335846
node_93: feature_name=cg16318412	feature_id[1590].value <= threshold=0.9159156084060669
Class: leiomyosarcoma (LMS)	
Rules_5	passed counts:61
node_0: feature_name=cg11915444	feature_id[2002].value > threshold=0.3402601182460785
node_10: feature_name=cg26016985	feature_id[4407].value > threshold=0.6493876278400421
node_16: feature_name=cg23157618	feature_id[5511].value <= threshold=0.5759606957435608
node_17: feature_name=cg23477348	feature_id[3774].value > threshold=0.19775588810443878
Class: alveolar rhabdomyosarcoma (RMS (ALV))	
Rules_6	passed counts:54
node_0: feature_name=cg11915444	feature_id[2002].value > threshold=0.3402601182460785
node_10: feature_name=cg26016985	feature_id[4407].value > threshold=0.6493876278400421
node_16: feature_name=cg23157618	feature_id[5511].value > threshold=0.5759606957435608
node_20: feature_name=cg10480329	feature_id[1439].value > threshold=0.5518321692943573
node_48: feature_name=cg17843418	feature_id[4868].value <= threshold=0.34969255328178406
node_49: feature_name=cg06551007	feature_id[1675].value > threshold=0.44745437800884247
Class: synovial sarcoma (SYSA)	
Rules_7	passed counts:50
node_0: feature_name=cg11915444	feature_id[2002].value > threshold=0.3402601182460785

node_10: feature_name=cg26016985	feature_id[4407].value > threshold=0.6493876278400421
node_16: feature_name=cg23157618	feature_id[5511].value > threshold=0.5759606957435608
node_20: feature_name=cg10480329	feature_id[1439].value > threshold=0.5518321692943573
node_48: feature_name=cg17843418	feature_id[4868].value > threshold=0.34969255328178406
node_52: feature_name=cg24407065	feature_id[4522].value <= threshold=0.5364363789558411
node_53: feature_name=cg27049344	feature_id[5181].value <= threshold=0.9606902599334717
Class: gastrointestinal stromal tumour (GIST)	
Rules_8	passed counts:41
node_0: feature_name=cg11915444	feature_id[2002].value > threshold=0.3402601182460785
node_10: feature_name=cg26016985	feature_id[4407].value > threshold=0.6493876278400421
node_16: feature_name=cg23157618	feature_id[5511].value > threshold=0.5759606957435608
node_20: feature_name=cg10480329	feature_id[1439].value > threshold=0.5518321692943573
node_48: feature_name=cg17843418	feature_id[4868].value > threshold=0.34969255328178406
node_52: feature_name=cg24407065	feature_id[4522].value > threshold=0.5364363789558411
node_56: feature_name=cg07281938	feature_id[249].value > threshold=0.7506992518901825
node_78: feature_name=cg21759907	feature_id[1650].value > threshold=0.46823520958423615
node_98: feature_name=cg10886334	feature_id[3975].value <= threshold=0.43698127567768097
node_99: feature_name=cg08091706	feature_id[4506].value > threshold=0.7506741881370544
Class: desmoplastic small round cell tumour (DSRCT)	
Rules_9	passed counts:40
node_0: feature_name=cg11915444	feature_id[2002].value > threshold=0.3402601182460785
node_10: feature_name=cg26016985	feature_id[4407].value > threshold=0.6493876278400421

node_16: feature_name=cg23157618	feature_id[5511].value > threshold=0.5759606957435608
node_20: feature_name=cg10480329	feature_id[1439].value > threshold=0.5518321692943573
node_48: feature_name=cg17843418	feature_id[4868].value > threshold=0.34969255328178406
node_52: feature_name=cg24407065	feature_id[4522].value > threshold=0.5364363789558411
node_56: feature_name=cg07281938	feature_id[249].value > threshold=0.7506992518901825
node_78: feature_name=cg21759907	feature_id[1650].value > threshold=0.46823520958423615
node_98: feature_name=cg10886334	feature_id[3975].value > threshold=0.43698127567768097
node_102: feature_name=cg09373983	feature_id[5025].value > threshold=0.5575815737247467
node_106: feature_name=cg12948116	feature_id[5360].value > threshold=0.5658791363239288
node_388: feature_name=cg02855309	feature_id[4351].value > threshold=0.2790501043200493
Class: embryonal rhabdomyosarcoma (RMS (EMB))	
Rules_10	passed counts:39
node_0: feature_name=cg11915444	feature_id[2002].value > threshold=0.3402601182460785
node_10: feature_name=cg26016985	feature_id[4407].value > threshold=0.6493876278400421
node_16: feature_name=cg23157618	feature_id[5511].value > threshold=0.5759606957435608
node_20: feature_name=cg10480329	feature_id[1439].value > threshold=0.5518321692943573
node_48: feature_name=cg17843418	feature_id[4868].value > threshold=0.34969255328178406
node_52: feature_name=cg24407065	feature_id[4522].value > threshold=0.5364363789558411
node_56: feature_name=cg07281938	feature_id[249].value > threshold=0.7506992518901825
node_78: feature_name=cg21759907	feature_id[1650].value > threshold=0.46823520958423615
node_98: feature_name=cg10886334	feature_id[3975].value > threshold=0.43698127567768097

node_102: feature_name=cg09373983	feature_id[5025].value > threshold=0.5575815737247467
node_106: feature_name=cg12948116	feature_id[5360].value <= threshold=0.5658791363239288
node_107: feature_name=cg21906519	feature_id[5251].value > threshold=0.6089984774589539
node_111: feature_name=cg17537493	feature_id[4217].value <= threshold=0.5825372636318207
node_112: feature_name=cg07260789	feature_id[1074].value <= threshold=0.8945248425006866
Class: angiosarcoma (AS)	
Rules_11	passed counts:38
node_0: feature_name=cg11915444	feature_id[2002].value > threshold=0.3402601182460785
node_10: feature_name=cg26016985	feature_id[4407].value > threshold=0.6493876278400421
node_16: feature_name=cg23157618	feature_id[5511].value > threshold=0.5759606957435608
node_20: feature_name=cg10480329	feature_id[1439].value > threshold=0.5518321692943573
node_48: feature_name=cg17843418	feature_id[4868].value > threshold=0.34969255328178406
node_52: feature_name=cg24407065	feature_id[4522].value > threshold=0.5364363789558411
node_56: feature_name=cg07281938	feature_id[249].value > threshold=0.7506992518901825
node_78: feature_name=cg21759907	feature_id[1650].value > threshold=0.46823520958423615
node_98: feature_name=cg10886334	feature_id[3975].value > threshold=0.43698127567768097
node_102: feature_name=cg09373983	feature_id[5025].value <= threshold=0.5575815737247467
node_103: feature_name=cg07642499	feature_id[3026].value > threshold=0.7077974379062653
Class: dermatofibrosarcoma protuberans (DFSP)	
Rules_12	passed counts:37
node_0: feature_name=cg11915444	feature_id[2002].value > threshold=0.3402601182460785
node_10: feature_name=cg26016985	feature_id[4407].value > threshold=0.6493876278400421

node_16: feature_name=cg23157618	feature_id[5511].value > threshold=0.5759606957435608
node_20: feature_name=cg10480329	feature_id[1439].value > threshold=0.5518321692943573
node_48: feature_name=cg17843418	feature_id[4868].value > threshold=0.34969255328178406
node_52: feature_name=cg24407065	feature_id[4522].value > threshold=0.5364363789558411
node_56: feature_name=cg07281938	feature_id[249].value > threshold=0.7506992518901825
node_78: feature_name=cg21759907	feature_id[1650].value > threshold=0.46823520958423615
node_98: feature_name=cg10886334	feature_id[3975].value > threshold=0.43698127567768097
node_102: feature_name=cg09373983	feature_id[5025].value > threshold=0.5575815737247467
node_106: feature_name=cg12948116	feature_id[5360].value <= threshold=0.5658791363239288
node_107: feature_name=cg21906519	feature_id[5251].value > threshold=0.6089984774589539
node_111: feature_name=cg17537493	feature_id[4217].value > threshold=0.5825372636318207
node_117: feature_name=cg15720017	feature_id[4443].value > threshold=0.7009969055652618
node_119: feature_name=cg06038180	feature_id[3480].value > threshold=0.4583848565816879
node_121: feature_name=cg08619651	feature_id[4882].value <= threshold=0.2105495035648346
node_122: feature_name=cg01014262	feature_id[1911].value <= threshold=0.5327437818050385
node_123: feature_name=cg16863382	feature_id[2922].value > threshold=0.7200668156147003
node_133: feature_name=cg06989443	feature_id[2327].value <= threshold=0.6579365730285645
node_134: feature_name=cg07344990	feature_id[2398].value > threshold=0.5562876164913177
node_138: feature_name=cg02966841	feature_id[2199].value <= threshold=0.7744501233100891
node_139: feature_name=cg06989253	feature_id[194].value > threshold=0.7899391353130341
node_171: feature_name=cg07041720	feature_id[932].value <= threshold=0.5004830807447433
node_172: feature_name=cg04716580	feature_id[1390].value > threshold=0.8913259208202362

node_182: feature_name=cg24426483	feature_id[3487].value > threshold=0.7537154853343964
node_186: feature_name=cg11847597	feature_id[2502].value > threshold=0.49640630185604095
Class: undifferentiated sarcoma (USARC)	
Rules_13	passed counts:37
node_0: feature_name=cg11915444	feature_id[2002].value > threshold=0.3402601182460785
node_10: feature_name=cg26016985	feature_id[4407].value > threshold=0.6493876278400421
node_16: feature_name=cg23157618	feature_id[5511].value > threshold=0.5759606957435608
node_20: feature_name=cg10480329	feature_id[1439].value > threshold=0.5518321692943573
node_48: feature_name=cg17843418	feature_id[4868].value > threshold=0.34969255328178406
node_52: feature_name=cg24407065	feature_id[4522].value > threshold=0.5364363789558411
node_56: feature_name=cg07281938	feature_id[249].value > threshold=0.7506992518901825
node_78: feature_name=cg21759907	feature_id[1650].value > threshold=0.46823520958423615
node_98: feature_name=cg10886334	feature_id[3975].value > threshold=0.43698127567768097
node_102: feature_name=cg09373983	feature_id[5025].value > threshold=0.5575815737247467
node_106: feature_name=cg12948116	feature_id[5360].value <= threshold=0.5658791363239288
node_107: feature_name=cg21906519	feature_id[5251].value <= threshold=0.6089984774589539
node_108: feature_name=cg05288253	feature_id[559].value > threshold=0.28334617614746094
Class: myxoid liposarcoma (MLS)	
Rules_14	passed counts:30
node_0: feature_name=cg11915444	feature_id[2002].value > threshold=0.3402601182460785
node_10: feature_name=cg26016985	feature_id[4407].value > threshold=0.6493876278400421
node_16: feature_name=cg23157618	feature_id[5511].value > threshold=0.5759606957435608

node_20: feature_name=cg10480329	feature_id[1439].value > threshold=0.5518321692943573
node_48: feature_name=cg17843418	feature_id[4868].value > threshold=0.34969255328178406
node_52: feature_name=cg24407065	feature_id[4522].value > threshold=0.5364363789558411
node_56: feature_name=cg07281938	feature_id[249].value > threshold=0.7506992518901825
node_78: feature_name=cg21759907	feature_id[1650].value > threshold=0.46823520958423615
node_98: feature_name=cg10886334	feature_id[3975].value > threshold=0.43698127567768097
node_102: feature_name=cg09373983	feature_id[5025].value > threshold=0.5575815737247467
node_106: feature_name=cg12948116	feature_id[5360].value <= threshold=0.5658791363239288
node_107: feature_name=cg21906519	feature_id[5251].value > threshold=0.6089984774589539
node_111: feature_name=cg17537493	feature_id[4217].value > threshold=0.5825372636318207
node_117: feature_name=cg15720017	feature_id[4443].value > threshold=0.7009969055652618
node_119: feature_name=cg06038180	feature_id[3480].value > threshold=0.4583848565816879
node_121: feature_name=cg08619651	feature_id[4882].value <= threshold=0.2105495035648346
node_122: feature_name=cg01014262	feature_id[1911].value <= threshold=0.5327437818050385
node_123: feature_name=cg16863382	feature_id[2922].value > threshold=0.7200668156147003
node_133: feature_name=cg06989443	feature_id[2327].value > threshold=0.6579365730285645
node_355: feature_name=cg27470978	feature_id[4804].value <= threshold=0.6264137625694275
node_356: feature_name=cg26964426	feature_id[2027].value > threshold=0.665222555398941
Class: malignant peripheral nerve sheath tumour (MPNST)	
Rules_15	passed counts:29
node_0: feature_name=cg11915444	feature_id[2002].value > threshold=0.3402601182460785

node_10: feature_name=cg26016985	feature_id[4407].value > threshold=0.6493876278400421
node_16: feature_name=cg23157618	feature_id[5511].value > threshold=0.5759606957435608
node_20: feature_name=cg10480329	feature_id[1439].value > threshold=0.5518321692943573
node_48: feature_name=cg17843418	feature_id[4868].value > threshold=0.34969255328178406
node_52: feature_name=cg24407065	feature_id[4522].value > threshold=0.5364363789558411
node_56: feature_name=cg07281938	feature_id[249].value > threshold=0.7506992518901825
node_78: feature_name=cg21759907	feature_id[1650].value > threshold=0.46823520958423615
node_98: feature_name=cg10886334	feature_id[3975].value > threshold=0.43698127567768097
node_102: feature_name=cg09373983	feature_id[5025].value > threshold=0.5575815737247467
node_106: feature_name=cg12948116	feature_id[5360].value <= threshold=0.5658791363239288
node_107: feature_name=cg21906519	feature_id[5251].value > threshold=0.6089984774589539
node_111: feature_name=cg17537493	feature_id[4217].value > threshold=0.5825372636318207
node_117: feature_name=cg15720017	feature_id[4443].value > threshold=0.7009969055652618
node_119: feature_name=cg06038180	feature_id[3480].value <= threshold=0.4583848565816879
Class: schwannoma (SWN)	
Rules_16	passed counts:29
node_0: feature_name=cg11915444	feature_id[2002].value > threshold=0.3402601182460785
node_10: feature_name=cg26016985	feature_id[4407].value > threshold=0.6493876278400421
node_16: feature_name=cg23157618	feature_id[5511].value > threshold=0.5759606957435608
node_20: feature_name=cg10480329	feature_id[1439].value > threshold=0.5518321692943573
node_48: feature_name=cg17843418	feature_id[4868].value > threshold=0.34969255328178406
node_52: feature_name=cg24407065	feature_id[4522].value > threshold=0.5364363789558411

node_56: feature_name=cg07281938	feature_id[249].value > threshold=0.7506992518901825
node_78: feature_name=cg21759907	feature_id[1650].value > threshold=0.46823520958423615
node_98: feature_name=cg10886334	feature_id[3975].value > threshold=0.43698127567768097
node_102: feature_name=cg09373983	feature_id[5025].value > threshold=0.5575815737247467
node_106: feature_name=cg12948116	feature_id[5360].value <= threshold=0.5658791363239288
node_107: feature_name=cg21906519	feature_id[5251].value > threshold=0.6089984774589539
node_111: feature_name=cg17537493	feature_id[4217].value > threshold=0.5825372636318207
node_117: feature_name=cg15720017	feature_id[4443].value <= threshold=0.7009969055652618
Class: solitary fibrous tumour (SFT)	
Rules_17	passed counts:27
node_0: feature_name=cg11915444	feature_id[2002].value > threshold=0.3402601182460785
node_10: feature_name=cg26016985	feature_id[4407].value > threshold=0.6493876278400421
node_16: feature_name=cg23157618	feature_id[5511].value > threshold=0.5759606957435608
node_20: feature_name=cg10480329	feature_id[1439].value > threshold=0.5518321692943573
node_48: feature_name=cg17843418	feature_id[4868].value > threshold=0.34969255328178406
node_52: feature_name=cg24407065	feature_id[4522].value > threshold=0.5364363789558411
node_56: feature_name=cg07281938	feature_id[249].value > threshold=0.7506992518901825
node_78: feature_name=cg21759907	feature_id[1650].value > threshold=0.46823520958423615
node_98: feature_name=cg10886334	feature_id[3975].value > threshold=0.43698127567768097
node_102: feature_name=cg09373983	feature_id[5025].value > threshold=0.5575815737247467
node_106: feature_name=cg12948116	feature_id[5360].value <= threshold=0.5658791363239288
node_107: feature_name=cg21906519	feature_id[5251].value > threshold=0.6089984774589539

node_111: feature_name=cg17537493	feature_id[4217].value > threshold=0.5825372636318207
node_117: feature_name=cg15720017	feature_id[4443].value > threshold=0.7009969055652618
node_119: feature_name=cg06038180	feature_id[3480].value > threshold=0.4583848565816879
node_121: feature_name=cg08619651	feature_id[4882].value > threshold=0.2105495035648346
Class: epithelioid sarcoma (ES)	
Rules_18	passed counts:23
node_0: feature_name=cg11915444	feature_id[2002].value > threshold=0.3402601182460785
node_10: feature_name=cg26016985	feature_id[4407].value > threshold=0.6493876278400421
node_16: feature_name=cg23157618	feature_id[5511].value > threshold=0.5759606957435608
node_20: feature_name=cg10480329	feature_id[1439].value > threshold=0.5518321692943573
node_48: feature_name=cg17843418	feature_id[4868].value > threshold=0.34969255328178406
node_52: feature_name=cg24407065	feature_id[4522].value > threshold=0.5364363789558411
node_56: feature_name=cg07281938	feature_id[249].value > threshold=0.7506992518901825
node_78: feature_name=cg21759907	feature_id[1650].value > threshold=0.46823520958423615
node_98: feature_name=cg10886334	feature_id[3975].value > threshold=0.43698127567768097
node_102: feature_name=cg09373983	feature_id[5025].value > threshold=0.5575815737247467
node_106: feature_name=cg12948116	feature_id[5360].value <= threshold=0.5658791363239288
node_107: feature_name=cg21906519	feature_id[5251].value > threshold=0.6089984774589539
node_111: feature_name=cg17537493	feature_id[4217].value > threshold=0.5825372636318207
node_117: feature_name=cg15720017	feature_id[4443].value > threshold=0.7009969055652618
node_119: feature_name=cg06038180	feature_id[3480].value > threshold=0.4583848565816879
node_121: feature_name=cg08619651	feature_id[4882].value <= threshold=0.2105495035648346

node_122: feature_name=cg01014262	feature_id[1911].value <= threshold=0.5327437818050385
node_123: feature_name=cg16863382	feature_id[2922].value <= threshold=0.7200668156147003
node_124: feature_name=cg11225330	feature_id[3185].value <= threshold=0.7814575433731079
Class: alveolar soft part sarcoma (ASPS)	
Rules_19	passed counts:22
node_0: feature_name=cg11915444	feature_id[2002].value > threshold=0.3402601182460785
node_10: feature_name=cg26016985	feature_id[4407].value > threshold=0.6493876278400421
node_16: feature_name=cg23157618	feature_id[5511].value > threshold=0.5759606957435608
node_20: feature_name=cg10480329	feature_id[1439].value > threshold=0.5518321692943573
node_48: feature_name=cg17843418	feature_id[4868].value > threshold=0.34969255328178406
node_52: feature_name=cg24407065	feature_id[4522].value > threshold=0.5364363789558411
node_56: feature_name=cg07281938	feature_id[249].value > threshold=0.7506992518901825
node_78: feature_name=cg21759907	feature_id[1650].value > threshold=0.46823520958423615
node_98: feature_name=cg10886334	feature_id[3975].value > threshold=0.43698127567768097
node_102: feature_name=cg09373983	feature_id[5025].value > threshold=0.5575815737247467
node_106: feature_name=cg12948116	feature_id[5360].value <= threshold=0.5658791363239288
node_107: feature_name=cg21906519	feature_id[5251].value > threshold=0.6089984774589539
node_111: feature_name=cg17537493	feature_id[4217].value > threshold=0.5825372636318207
node_117: feature_name=cg15720017	feature_id[4443].value > threshold=0.7009969055652618
node_119: feature_name=cg06038180	feature_id[3480].value > threshold=0.4583848565816879
node_121: feature_name=cg08619651	feature_id[4882].value <= threshold=0.2105495035648346
node_122: feature_name=cg01014262	feature_id[1911].value <= threshold=0.5327437818050385

node_123: feature_name=cg16863382	feature_id[2922].value > threshold=0.7200668156147003
node_133: feature_name=cg06989443	feature_id[2327].value <= threshold=0.6579365730285645
node_134: feature_name=cg07344990	feature_id[2398].value > threshold=0.5562876164913177
node_138: feature_name=cg02966841	feature_id[2199].value > threshold=0.7744501233100891
node_204: feature_name=cg12120430	feature_id[3727].value > threshold=0.5775820016860962
node_206: feature_name=cg18386876	feature_id[4066].value > threshold=0.6847257018089294
node_208: feature_name=cg02053092	feature_id[3610].value > threshold=0.39507485926151276
node_210: feature_name=cg10298992	feature_id[3603].value <= threshold=0.5707973837852478
node_211: feature_name=cg02436098	feature_id[1712].value > threshold=0.27362556010484695
node_225: feature_name=cg01794853	feature_id[1531].value > threshold=0.46636733412742615
Class: well differentiated liposarcoma (WDLS)/dedifferentiated liposarcoma (DDLS)	
Rules_20	passed counts:21
node_0: feature_name=cg11915444	feature_id[2002].value > threshold=0.3402601182460785
node_10: feature_name=cg26016985	feature_id[4407].value > threshold=0.6493876278400421
node_16: feature_name=cg23157618	feature_id[5511].value > threshold=0.5759606957435608
node_20: feature_name=cg10480329	feature_id[1439].value > threshold=0.5518321692943573
node_48: feature_name=cg17843418	feature_id[4868].value > threshold=0.34969255328178406
node_52: feature_name=cg24407065	feature_id[4522].value > threshold=0.5364363789558411
node_56: feature_name=cg07281938	feature_id[249].value > threshold=0.7506992518901825
node_78: feature_name=cg21759907	feature_id[1650].value > threshold=0.46823520958423615
node_98: feature_name=cg10886334	feature_id[3975].value > threshold=0.43698127567768097

node_102: feature_name=cg09373983	feature_id[5025].value > threshold=0.5575815737247467
node_106: feature_name=cg12948116	feature_id[5360].value <= threshold=0.5658791363239288
node_107: feature_name=cg21906519	feature_id[5251].value > threshold=0.6089984774589539
node_111: feature_name=cg17537493	feature_id[4217].value > threshold=0.5825372636318207
node_117: feature_name=cg15720017	feature_id[4443].value > threshold=0.7009969055652618
node_119: feature_name=cg06038180	feature_id[3480].value > threshold=0.4583848565816879
node_121: feature_name=cg08619651	feature_id[4882].value <= threshold=0.2105495035648346
node_122: feature_name=cg01014262	feature_id[1911].value > threshold=0.5327437818050385
node_382: feature_name=cg26240231	feature_id[4832].value > threshold=0.9200497567653656
Class: malignant rhabdoid tumour (MRT)	
Rules_21	passed counts:16
node_0: feature_name=cg11915444	feature_id[2002].value > threshold=0.3402601182460785
node_10: feature_name=cg26016985	feature_id[4407].value > threshold=0.6493876278400421
node_16: feature_name=cg23157618	feature_id[5511].value > threshold=0.5759606957435608
node_20: feature_name=cg10480329	feature_id[1439].value > threshold=0.5518321692943573
node_48: feature_name=cg17843418	feature_id[4868].value > threshold=0.34969255328178406
node_52: feature_name=cg24407065	feature_id[4522].value > threshold=0.5364363789558411
node_56: feature_name=cg07281938	feature_id[249].value > threshold=0.7506992518901825
node_78: feature_name=cg21759907	feature_id[1650].value > threshold=0.46823520958423615
node_98: feature_name=cg10886334	feature_id[3975].value > threshold=0.43698127567768097
node_102: feature_name=cg09373983	feature_id[5025].value > threshold=0.5575815737247467
node_106: feature_name=cg12948116	feature_id[5360].value <= threshold=0.5658791363239288

node_107: feature_name=cg21906519	feature_id[5251].value > threshold=0.6089984774589539
node_111: feature_name=cg17537493	feature_id[4217].value > threshold=0.5825372636318207
node_117: feature_name=cg15720017	feature_id[4443].value > threshold=0.7009969055652618
node_119: feature_name=cg06038180	feature_id[3480].value > threshold=0.4583848565816879
node_121: feature_name=cg08619651	feature_id[4882].value <= threshold=0.2105495035648346
node_122: feature_name=cg01014262	feature_id[1911].value <= threshold=0.5327437818050385
node_123: feature_name=cg16863382	feature_id[2922].value > threshold=0.7200668156147003
node_133: feature_name=cg06989443	feature_id[2327].value <= threshold=0.6579365730285645
node_134: feature_name=cg07344990	feature_id[2398].value <= threshold=0.5562876164913177
node_135: feature_name=cg01329151	feature_id[1681].value > threshold=0.20543088763952255
Class: low-grade endometrial stromal sarcoma (ESS (LG))	
Rules_22	passed counts:13
node_0: feature_name=cg11915444	feature_id[2002].value > threshold=0.3402601182460785
node_10: feature_name=cg26016985	feature_id[4407].value > threshold=0.6493876278400421
node_16: feature_name=cg23157618	feature_id[5511].value > threshold=0.5759606957435608
node_20: feature_name=cg10480329	feature_id[1439].value > threshold=0.5518321692943573
node_48: feature_name=cg17843418	feature_id[4868].value > threshold=0.34969255328178406
node_52: feature_name=cg24407065	feature_id[4522].value > threshold=0.5364363789558411
node_56: feature_name=cg07281938	feature_id[249].value > threshold=0.7506992518901825
node_78: feature_name=cg21759907	feature_id[1650].value > threshold=0.46823520958423615
node_98: feature_name=cg10886334	feature_id[3975].value > threshold=0.43698127567768097

node_102: feature_name=cg09373983	feature_id[5025].value > threshold=0.5575815737247467
node_106: feature_name=cg12948116	feature_id[5360].value <= threshold=0.5658791363239288
node_107: feature_name=cg21906519	feature_id[5251].value > threshold=0.6089984774589539
node_111: feature_name=cg17537493	feature_id[4217].value > threshold=0.5825372636318207
node_117: feature_name=cg15720017	feature_id[4443].value > threshold=0.7009969055652618
node_119: feature_name=cg06038180	feature_id[3480].value > threshold=0.4583848565816879
node_121: feature_name=cg08619651	feature_id[4882].value <= threshold=0.2105495035648346
node_122: feature_name=cg01014262	feature_id[1911].value <= threshold=0.5327437818050385
node_123: feature_name=cg16863382	feature_id[2922].value > threshold=0.7200668156147003
node_133: feature_name=cg06989443	feature_id[2327].value <= threshold=0.6579365730285645
node_134: feature_name=cg07344990	feature_id[2398].value > threshold=0.5562876164913177
node_138: feature_name=cg02966841	feature_id[2199].value > threshold=0.7744501233100891
node_204: feature_name=cg12120430	feature_id[3727].value > threshold=0.5775820016860962
node_206: feature_name=cg18386876	feature_id[4066].value > threshold=0.6847257018089294
node_208: feature_name=cg02053092	feature_id[3610].value > threshold=0.39507485926151276
node_210: feature_name=cg10298992	feature_id[3603].value > threshold=0.5707973837852478
node_228: feature_name=cg03840920	feature_id[4524].value <= threshold=0.5365143716335297
node_229: feature_name=cg21647035	feature_id[2479].value <= threshold=0.3486105799674988
Class: desmoid-type fibromatosis (DTFM)	
Rules_23	passed counts:13
node_0: feature_name=cg11915444	feature_id[2002].value > threshold=0.3402601182460785
node_10: feature_name=cg26016985	feature_id[4407].value > threshold=0.6493876278400421

node_16: feature_name=cg23157618	feature_id[5511].value > threshold=0.5759606957435608
node_20: feature_name=cg10480329	feature_id[1439].value > threshold=0.5518321692943573
node_48: feature_name=cg17843418	feature_id[4868].value > threshold=0.34969255328178406
node_52: feature_name=cg24407065	feature_id[4522].value > threshold=0.5364363789558411
node_56: feature_name=cg07281938	feature_id[249].value > threshold=0.7506992518901825
node_78: feature_name=cg21759907	feature_id[1650].value > threshold=0.46823520958423615
node_98: feature_name=cg10886334	feature_id[3975].value > threshold=0.43698127567768097
node_102: feature_name=cg09373983	feature_id[5025].value > threshold=0.5575815737247467
node_106: feature_name=cg12948116	feature_id[5360].value <= threshold=0.5658791363239288
node_107: feature_name=cg21906519	feature_id[5251].value > threshold=0.6089984774589539
node_111: feature_name=cg17537493	feature_id[4217].value > threshold=0.5825372636318207
node_117: feature_name=cg15720017	feature_id[4443].value > threshold=0.7009969055652618
node_119: feature_name=cg06038180	feature_id[3480].value > threshold=0.4583848565816879
node_121: feature_name=cg08619651	feature_id[4882].value <= threshold=0.2105495035648346
node_122: feature_name=cg01014262	feature_id[1911].value <= threshold=0.5327437818050385
node_123: feature_name=cg16863382	feature_id[2922].value > threshold=0.7200668156147003
node_133: feature_name=cg06989443	feature_id[2327].value <= threshold=0.6579365730285645
node_134: feature_name=cg07344990	feature_id[2398].value > threshold=0.5562876164913177
node_138: feature_name=cg02966841	feature_id[2199].value > threshold=0.7744501233100891
node_204: feature_name=cg12120430	feature_id[3727].value <= threshold=0.5775820016860962
Class: angioleiomyoma (ALMO)/myopericytoma (MPC)	

Rules_24	passed counts:12
node_0: feature_name=cg11915444	feature_id[2002].value > threshold=0.3402601182460785
node_10: feature_name=cg26016985	feature_id[4407].value > threshold=0.6493876278400421
node_16: feature_name=cg23157618	feature_id[5511].value > threshold=0.5759606957435608
node_20: feature_name=cg10480329	feature_id[1439].value > threshold=0.5518321692943573
node_48: feature_name=cg17843418	feature_id[4868].value > threshold=0.34969255328178406
node_52: feature_name=cg24407065	feature_id[4522].value > threshold=0.5364363789558411
node_56: feature_name=cg07281938	feature_id[249].value > threshold=0.7506992518901825
node_78: feature_name=cg21759907	feature_id[1650].value > threshold=0.46823520958423615
node_98: feature_name=cg10886334	feature_id[3975].value > threshold=0.43698127567768097
node_102: feature_name=cg09373983	feature_id[5025].value > threshold=0.5575815737247467
node_106: feature_name=cg12948116	feature_id[5360].value <= threshold=0.5658791363239288
node_107: feature_name=cg21906519	feature_id[5251].value > threshold=0.6089984774589539
node_111: feature_name=cg17537493	feature_id[4217].value > threshold=0.5825372636318207
node_117: feature_name=cg15720017	feature_id[4443].value > threshold=0.7009969055652618
node_119: feature_name=cg06038180	feature_id[3480].value > threshold=0.4583848565816879
node_121: feature_name=cg08619651	feature_id[4882].value <= threshold=0.2105495035648346
node_122: feature_name=cg01014262	feature_id[1911].value <= threshold=0.5327437818050385
node_123: feature_name=cg16863382	feature_id[2922].value > threshold=0.7200668156147003
node_133: feature_name=cg06989443	feature_id[2327].value > threshold=0.6579365730285645
node_355: feature_name=cg27470978	feature_id[4804].value > threshold=0.6264137625694275
node_361: feature_name=cg20252837	feature_id[420].value <= threshold=0.53105828166008

Class: small blue round cell tumour (SBRCT)	
Rules_25	passed counts:12
node_0: feature_name=cg11915444	feature_id[2002].value > threshold=0.3402601182460785
node_10: feature_name=cg26016985	feature_id[4407].value > threshold=0.6493876278400421
node_16: feature_name=cg23157618	feature_id[5511].value > threshold=0.5759606957435608
node_20: feature_name=cg10480329	feature_id[1439].value > threshold=0.5518321692943573
node_48: feature_name=cg17843418	feature_id[4868].value > threshold=0.34969255328178406
node_52: feature_name=cg24407065	feature_id[4522].value > threshold=0.5364363789558411
node_56: feature_name=cg07281938	feature_id[249].value > threshold=0.7506992518901825
node_78: feature_name=cg21759907	feature_id[1650].value > threshold=0.46823520958423615
node_98: feature_name=cg10886334	feature_id[3975].value > threshold=0.43698127567768097
node_102: feature_name=cg09373983	feature_id[5025].value > threshold=0.5575815737247467
node_106: feature_name=cg12948116	feature_id[5360].value <= threshold=0.5658791363239288
node_107: feature_name=cg21906519	feature_id[5251].value > threshold=0.6089984774589539
node_111: feature_name=cg17537493	feature_id[4217].value > threshold=0.5825372636318207
node_117: feature_name=cg15720017	feature_id[4443].value > threshold=0.7009969055652618
node_119: feature_name=cg06038180	feature_id[3480].value > threshold=0.4583848565816879
node_121: feature_name=cg08619651	feature_id[4882].value <= threshold=0.2105495035648346
node_122: feature_name=cg01014262	feature_id[1911].value <= threshold=0.5327437818050385
node_123: feature_name=cg16863382	feature_id[2922].value > threshold=0.7200668156147003
node_133: feature_name=cg06989443	feature_id[2327].value <= threshold=0.6579365730285645
node_134: feature_name=cg07344990	feature_id[2398].value > threshold=0.5562876164913177

node_138: feature_name=cg02966841	feature_id[2199].value > threshold=0.7744501233100891
node_204: feature_name=cg12120430	feature_id[3727].value > threshold=0.5775820016860962
node_206: feature_name=cg18386876	feature_id[4066].value > threshold=0.6847257018089294
node_208: feature_name=cg02053092	feature_id[3610].value > threshold=0.39507485926151276
node_210: feature_name=cg10298992	feature_id[3603].value > threshold=0.5707973837852478
node_228: feature_name=cg03840920	feature_id[4524].value > threshold=0.5365143716335297
node_234: feature_name=cg25364972	feature_id[1792].value > threshold=0.2418053299188614
node_238: feature_name=cg23485307	feature_id[221].value <= threshold=0.14884746074676514
node_239: feature_name=cg13972711	feature_id[4461].value <= threshold=0.8822346031665802
Class: infantile fibrosarcoma (IFS)	
Rules_26	passed counts:12
node_0: feature_name=cg11915444	feature_id[2002].value > threshold=0.3402601182460785
node_10: feature_name=cg26016985	feature_id[4407].value > threshold=0.6493876278400421
node_16: feature_name=cg23157618	feature_id[5511].value > threshold=0.5759606957435608
node_20: feature_name=cg10480329	feature_id[1439].value > threshold=0.5518321692943573
node_48: feature_name=cg17843418	feature_id[4868].value > threshold=0.34969255328178406
node_52: feature_name=cg24407065	feature_id[4522].value > threshold=0.5364363789558411
node_56: feature_name=cg07281938	feature_id[249].value > threshold=0.7506992518901825
node_78: feature_name=cg21759907	feature_id[1650].value > threshold=0.46823520958423615
node_98: feature_name=cg10886334	feature_id[3975].value > threshold=0.43698127567768097
node_102: feature_name=cg09373983	feature_id[5025].value > threshold=0.5575815737247467
node_106: feature_name=cg12948116	feature_id[5360].value <= threshold=0.5658791363239288

node_107: feature_name=cg21906519	feature_id[5251].value > threshold=0.6089984774589539
node_111: feature_name=cg17537493	feature_id[4217].value > threshold=0.5825372636318207
node_117: feature_name=cg15720017	feature_id[4443].value > threshold=0.7009969055652618
node_119: feature_name=cg06038180	feature_id[3480].value > threshold=0.4583848565816879
node_121: feature_name=cg08619651	feature_id[4882].value <= threshold=0.2105495035648346
node_122: feature_name=cg01014262	feature_id[1911].value <= threshold=0.5327437818050385
node_123: feature_name=cg16863382	feature_id[2922].value > threshold=0.7200668156147003
node_133: feature_name=cg06989443	feature_id[2327].value <= threshold=0.6579365730285645
node_134: feature_name=cg07344990	feature_id[2398].value > threshold=0.5562876164913177
node_138: feature_name=cg02966841	feature_id[2199].value > threshold=0.7744501233100891
node_204: feature_name=cg12120430	feature_id[3727].value > threshold=0.5775820016860962
node_206: feature_name=cg18386876	feature_id[4066].value > threshold=0.6847257018089294
node_208: feature_name=cg02053092	feature_id[3610].value > threshold=0.39507485926151276
node_210: feature_name=cg10298992	feature_id[3603].value > threshold=0.5707973837852478
node_228: feature_name=cg03840920	feature_id[4524].value > threshold=0.5365143716335297
node_234: feature_name=cg25364972	feature_id[1792].value <= threshold=0.2418053299188614
node_235: feature_name=cg07612468	feature_id[803].value <= threshold=0.8302715420722961
Class: ossifying fibromyxoid tumour (OFMT)	
Rules_27	passed counts:12
node_0: feature_name=cg11915444	feature_id[2002].value > threshold=0.3402601182460785
node_10: feature_name=cg26016985	feature_id[4407].value > threshold=0.6493876278400421
node_16: feature_name=cg23157618	feature_id[5511].value > threshold=0.5759606957435608

node_20: feature_name=cg10480329	feature_id[1439].value > threshold=0.5518321692943573
node_48: feature_name=cg17843418	feature_id[4868].value > threshold=0.34969255328178406
node_52: feature_name=cg24407065	feature_id[4522].value > threshold=0.5364363789558411
node_56: feature_name=cg07281938	feature_id[249].value > threshold=0.7506992518901825
node_78: feature_name=cg21759907	feature_id[1650].value > threshold=0.46823520958423615
node_98: feature_name=cg10886334	feature_id[3975].value > threshold=0.43698127567768097
node_102: feature_name=cg09373983	feature_id[5025].value > threshold=0.5575815737247467
node_106: feature_name=cg12948116	feature_id[5360].value <= threshold=0.5658791363239288
node_107: feature_name=cg21906519	feature_id[5251].value > threshold=0.6089984774589539
node_111: feature_name=cg17537493	feature_id[4217].value > threshold=0.5825372636318207
node_117: feature_name=cg15720017	feature_id[4443].value > threshold=0.7009969055652618
node_119: feature_name=cg06038180	feature_id[3480].value > threshold=0.4583848565816879
node_121: feature_name=cg08619651	feature_id[4882].value <= threshold=0.2105495035648346
node_122: feature_name=cg01014262	feature_id[1911].value <= threshold=0.5327437818050385
node_123: feature_name=cg16863382	feature_id[2922].value > threshold=0.7200668156147003
node_133: feature_name=cg06989443	feature_id[2327].value <= threshold=0.6579365730285645
node_134: feature_name=cg07344990	feature_id[2398].value > threshold=0.5562876164913177
node_138: feature_name=cg02966841	feature_id[2199].value > threshold=0.7744501233100891
node_204: feature_name=cg12120430	feature_id[3727].value > threshold=0.5775820016860962
node_206: feature_name=cg18386876	feature_id[4066].value > threshold=0.6847257018089294
node_208: feature_name=cg02053092	feature_id[3610].value <= threshold=0.39507485926151276
Class: fibrous dysplasia (FDY)	

Rules_28	passed counts:12
node_0: feature_name=cg11915444	feature_id[2002].value > threshold=0.3402601182460785
node_10: feature_name=cg26016985	feature_id[4407].value > threshold=0.6493876278400421
node_16: feature_name=cg23157618	feature_id[5511].value > threshold=0.5759606957435608
node_20: feature_name=cg10480329	feature_id[1439].value > threshold=0.5518321692943573
node_48: feature_name=cg17843418	feature_id[4868].value > threshold=0.34969255328178406
node_52: feature_name=cg24407065	feature_id[4522].value > threshold=0.5364363789558411
node_56: feature_name=cg07281938	feature_id[249].value > threshold=0.7506992518901825
node_78: feature_name=cg21759907	feature_id[1650].value > threshold=0.46823520958423615
node_98: feature_name=cg10886334	feature_id[3975].value > threshold=0.43698127567768097
node_102: feature_name=cg09373983	feature_id[5025].value > threshold=0.5575815737247467
node_106: feature_name=cg12948116	feature_id[5360].value <= threshold=0.5658791363239288
node_107: feature_name=cg21906519	feature_id[5251].value > threshold=0.6089984774589539
node_111: feature_name=cg17537493	feature_id[4217].value > threshold=0.5825372636318207
node_117: feature_name=cg15720017	feature_id[4443].value > threshold=0.7009969055652618
node_119: feature_name=cg06038180	feature_id[3480].value > threshold=0.4583848565816879
node_121: feature_name=cg08619651	feature_id[4882].value <= threshold=0.2105495035648346
node_122: feature_name=cg01014262	feature_id[1911].value <= threshold=0.5327437818050385
node_123: feature_name=cg16863382	feature_id[2922].value > threshold=0.7200668156147003
node_133: feature_name=cg06989443	feature_id[2327].value <= threshold=0.6579365730285645
node_134: feature_name=cg07344990	feature_id[2398].value > threshold=0.5562876164913177

node_138: feature_name=cg02966841	feature_id[2199].value > threshold=0.7744501233100891
node_204: feature_name=cg12120430	feature_id[3727].value > threshold=0.5775820016860962
node_206: feature_name=cg18386876	feature_id[4066].value <= threshold=0.6847257018089294
Class: clear cell sarcoma of the kidney (CCSK)	
Rules_29	passed counts:11
node_0: feature_name=cg11915444	feature_id[2002].value > threshold=0.3402601182460785
node_10: feature_name=cg26016985	feature_id[4407].value > threshold=0.6493876278400421
node_16: feature_name=cg23157618	feature_id[5511].value > threshold=0.5759606957435608
node_20: feature_name=cg10480329	feature_id[1439].value > threshold=0.5518321692943573
node_48: feature_name=cg17843418	feature_id[4868].value > threshold=0.34969255328178406
node_52: feature_name=cg24407065	feature_id[4522].value > threshold=0.5364363789558411
node_56: feature_name=cg07281938	feature_id[249].value > threshold=0.7506992518901825
node_78: feature_name=cg21759907	feature_id[1650].value > threshold=0.46823520958423615
node_98: feature_name=cg10886334	feature_id[3975].value > threshold=0.43698127567768097
node_102: feature_name=cg09373983	feature_id[5025].value > threshold=0.5575815737247467
node_106: feature_name=cg12948116	feature_id[5360].value <= threshold=0.5658791363239288
node_107: feature_name=cg21906519	feature_id[5251].value > threshold=0.6089984774589539
node_111: feature_name=cg17537493	feature_id[4217].value > threshold=0.5825372636318207
node_117: feature_name=cg15720017	feature_id[4443].value > threshold=0.7009969055652618
node_119: feature_name=cg06038180	feature_id[3480].value > threshold=0.4583848565816879
node_121: feature_name=cg08619651	feature_id[4882].value <= threshold=0.2105495035648346
node_122: feature_name=cg01014262	feature_id[1911].value <= threshold=0.5327437818050385

node_123: feature_name=cg16863382	feature_id[2922].value > threshold=0.7200668156147003
node_133: feature_name=cg06989443	feature_id[2327].value <= threshold=0.6579365730285645
node_134: feature_name=cg07344990	feature_id[2398].value > threshold=0.5562876164913177
node_138: feature_name=cg02966841	feature_id[2199].value > threshold=0.7744501233100891
node_204: feature_name=cg12120430	feature_id[3727].value > threshold=0.5775820016860962
node_206: feature_name=cg18386876	feature_id[4066].value > threshold=0.6847257018089294
node_208: feature_name=cg02053092	feature_id[3610].value > threshold=0.39507485926151276
node_210: feature_name=cg10298992	feature_id[3603].value > threshold=0.5707973837852478
node_228: feature_name=cg03840920	feature_id[4524].value > threshold=0.5365143716335297
node_234: feature_name=cg25364972	feature_id[1792].value > threshold=0.2418053299188614
node_238: feature_name=cg23485307	feature_id[221].value > threshold=0.14884746074676514
node_242: feature_name=cg04873169	feature_id[4696].value > threshold=0.6058756709098816
node_244: feature_name=cg17009731	feature_id[3218].value > threshold=0.6602252125740051
node_246: feature_name=cg18253910	feature_id[4458].value > threshold=0.6904322504997253
node_250: feature_name=cg04774476	feature_id[1257].value > threshold=0.5675267577171326
node_256: feature_name=cg19259111	feature_id[63].value > threshold=0.40771469473838806
node_264: feature_name=cg21483216	feature_id[5253].value > threshold=0.6332719326019287
node_266: feature_name=cg04026354	feature_id[3502].value > threshold=0.42332448065280914
node_336: feature_name=cg05824594	feature_id[1431].value <= threshold=0.629401296377182
node_337: feature_name=cg18475969	feature_id[2117].value > threshold=0.3116971254348755
node_351: feature_name=cg22286382	feature_id[2821].value <= threshold=0.6607459187507629
Class: inflammatory myofibroblastic tumour (IMT)	

Rules_30	passed counts:11
node_0: feature_name=cg11915444	feature_id[2002].value > threshold=0.3402601182460785
node_10: feature_name=cg26016985	feature_id[4407].value > threshold=0.6493876278400421
node_16: feature_name=cg23157618	feature_id[5511].value > threshold=0.5759606957435608
node_20: feature_name=cg10480329	feature_id[1439].value > threshold=0.5518321692943573
node_48: feature_name=cg17843418	feature_id[4868].value > threshold=0.34969255328178406
node_52: feature_name=cg24407065	feature_id[4522].value > threshold=0.5364363789558411
node_56: feature_name=cg07281938	feature_id[249].value > threshold=0.7506992518901825
node_78: feature_name=cg21759907	feature_id[1650].value > threshold=0.46823520958423615
node_98: feature_name=cg10886334	feature_id[3975].value > threshold=0.43698127567768097
node_102: feature_name=cg09373983	feature_id[5025].value > threshold=0.5575815737247467
node_106: feature_name=cg12948116	feature_id[5360].value <= threshold=0.5658791363239288
node_107: feature_name=cg21906519	feature_id[5251].value > threshold=0.6089984774589539
node_111: feature_name=cg17537493	feature_id[4217].value > threshold=0.5825372636318207
node_117: feature_name=cg15720017	feature_id[4443].value > threshold=0.7009969055652618
node_119: feature_name=cg06038180	feature_id[3480].value > threshold=0.4583848565816879
node_121: feature_name=cg08619651	feature_id[4882].value <= threshold=0.2105495035648346
node_122: feature_name=cg01014262	feature_id[1911].value <= threshold=0.5327437818050385
node_123: feature_name=cg16863382	feature_id[2922].value > threshold=0.7200668156147003
node_133: feature_name=cg06989443	feature_id[2327].value <= threshold=0.6579365730285645
node_134: feature_name=cg07344990	feature_id[2398].value > threshold=0.5562876164913177

node_138: feature_name=cg02966841	feature_id[2199].value > threshold=0.7744501233100891
node_204: feature_name=cg12120430	feature_id[3727].value > threshold=0.5775820016860962
node_206: feature_name=cg18386876	feature_id[4066].value > threshold=0.6847257018089294
node_208: feature_name=cg02053092	feature_id[3610].value > threshold=0.39507485926151276
node_210: feature_name=cg10298992	feature_id[3603].value > threshold=0.5707973837852478
node_228: feature_name=cg03840920	feature_id[4524].value > threshold=0.5365143716335297
node_234: feature_name=cg25364972	feature_id[1792].value > threshold=0.2418053299188614
node_238: feature_name=cg23485307	feature_id[221].value > threshold=0.14884746074676514
node_242: feature_name=cg04873169	feature_id[4696].value > threshold=0.6058756709098816
node_244: feature_name=cg17009731	feature_id[3218].value > threshold=0.6602252125740051
node_246: feature_name=cg18253910	feature_id[4458].value > threshold=0.6904322504997253
node_250: feature_name=cg04774476	feature_id[1257].value > threshold=0.5675267577171326
node_256: feature_name=cg19259111	feature_id[63].value <= threshold=0.40771469473838806
node_257: feature_name=cg10803714	feature_id[856].value <= threshold=0.4142659604549408
Class: small blue round cell tumour (SBRCT)	
Rules_31	passed counts:11
node_0: feature_name=cg11915444	feature_id[2002].value > threshold=0.3402601182460785
node_10: feature_name=cg26016985	feature_id[4407].value > threshold=0.6493876278400421
node_16: feature_name=cg23157618	feature_id[5511].value > threshold=0.5759606957435608
node_20: feature_name=cg10480329	feature_id[1439].value > threshold=0.5518321692943573
node_48: feature_name=cg17843418	feature_id[4868].value > threshold=0.34969255328178406
node_52: feature_name=cg24407065	feature_id[4522].value > threshold=0.5364363789558411

node_56: feature_name=cg07281938	feature_id[249].value > threshold=0.7506992518901825
node_78: feature_name=cg21759907	feature_id[1650].value > threshold=0.46823520958423615
node_98: feature_name=cg10886334	feature_id[3975].value > threshold=0.43698127567768097
node_102: feature_name=cg09373983	feature_id[5025].value > threshold=0.5575815737247467
node_106: feature_name=cg12948116	feature_id[5360].value <= threshold=0.5658791363239288
node_107: feature_name=cg21906519	feature_id[5251].value > threshold=0.6089984774589539
node_111: feature_name=cg17537493	feature_id[4217].value > threshold=0.5825372636318207
node_117: feature_name=cg15720017	feature_id[4443].value > threshold=0.7009969055652618
node_119: feature_name=cg06038180	feature_id[3480].value > threshold=0.4583848565816879
node_121: feature_name=cg08619651	feature_id[4882].value <= threshold=0.2105495035648346
node_122: feature_name=cg01014262	feature_id[1911].value <= threshold=0.5327437818050385
node_123: feature_name=cg16863382	feature_id[2922].value > threshold=0.7200668156147003
node_133: feature_name=cg06989443	feature_id[2327].value <= threshold=0.6579365730285645
node_134: feature_name=cg07344990	feature_id[2398].value > threshold=0.5562876164913177
node_138: feature_name=cg02966841	feature_id[2199].value <= threshold=0.7744501233100891
node_139: feature_name=cg06989253	feature_id[194].value <= threshold=0.7899391353130341
node_140: feature_name=cg24617568	feature_id[3282].value > threshold=0.26932457089424133
Class: atypical fibroxanthoma (AFX)/pleomorphic dermal sarcoma (PDS)	
Rules_32	passed counts:10
node_0: feature_name=cg11915444	feature_id[2002].value > threshold=0.3402601182460785
node_10: feature_name=cg26016985	feature_id[4407].value > threshold=0.6493876278400421

node_16: feature_name=cg23157618	feature_id[5511].value > threshold=0.5759606957435608
node_20: feature_name=cg10480329	feature_id[1439].value > threshold=0.5518321692943573
node_48: feature_name=cg17843418	feature_id[4868].value > threshold=0.34969255328178406
node_52: feature_name=cg24407065	feature_id[4522].value > threshold=0.5364363789558411
node_56: feature_name=cg07281938	feature_id[249].value > threshold=0.7506992518901825
node_78: feature_name=cg21759907	feature_id[1650].value > threshold=0.46823520958423615
node_98: feature_name=cg10886334	feature_id[3975].value > threshold=0.43698127567768097
node_102: feature_name=cg09373983	feature_id[5025].value > threshold=0.5575815737247467
node_106: feature_name=cg12948116	feature_id[5360].value <= threshold=0.5658791363239288
node_107: feature_name=cg21906519	feature_id[5251].value > threshold=0.6089984774589539
node_111: feature_name=cg17537493	feature_id[4217].value > threshold=0.5825372636318207
node_117: feature_name=cg15720017	feature_id[4443].value > threshold=0.7009969055652618
node_119: feature_name=cg06038180	feature_id[3480].value > threshold=0.4583848565816879
node_121: feature_name=cg08619651	feature_id[4882].value <= threshold=0.2105495035648346
node_122: feature_name=cg01014262	feature_id[1911].value <= threshold=0.5327437818050385
node_123: feature_name=cg16863382	feature_id[2922].value > threshold=0.7200668156147003
node_133: feature_name=cg06989443	feature_id[2327].value <= threshold=0.6579365730285645
node_134: feature_name=cg07344990	feature_id[2398].value > threshold=0.5562876164913177
node_138: feature_name=cg02966841	feature_id[2199].value > threshold=0.7744501233100891
node_204: feature_name=cg12120430	feature_id[3727].value > threshold=0.5775820016860962
node_206: feature_name=cg18386876	feature_id[4066].value > threshold=0.6847257018089294
node_208: feature_name=cg02053092	feature_id[3610].value > threshold=0.39507485926151276

node_210: feature_name=cg10298992	feature_id[3603].value > threshold=0.5707973837852478
node_228: feature_name=cg03840920	feature_id[4524].value > threshold=0.5365143716335297
node_234: feature_name=cg25364972	feature_id[1792].value > threshold=0.2418053299188614
node_238: feature_name=cg23485307	feature_id[221].value > threshold=0.14884746074676514
node_242: feature_name=cg04873169	feature_id[4696].value > threshold=0.6058756709098816
node_244: feature_name=cg17009731	feature_id[3218].value > threshold=0.6602252125740051
node_246: feature_name=cg18253910	feature_id[4458].value > threshold=0.6904322504997253
node_250: feature_name=cg04774476	feature_id[1257].value > threshold=0.5675267577171326
node_256: feature_name=cg19259111	feature_id[63].value > threshold=0.40771469473838806
node_264: feature_name=cg21483216	feature_id[5253].value > threshold=0.6332719326019287
node_266: feature_name=cg04026354	feature_id[3502].value > threshold=0.42332448065280914
node_336: feature_name=cg05824594	feature_id[1431].value > threshold=0.629401296377182
Class: angiomatoid fibrous histiocytoma (AFH)	
Rules_33	passed counts:10
node_0: feature_name=cg11915444	feature_id[2002].value > threshold=0.3402601182460785
node_10: feature_name=cg26016985	feature_id[4407].value > threshold=0.6493876278400421
node_16: feature_name=cg23157618	feature_id[5511].value > threshold=0.5759606957435608
node_20: feature_name=cg10480329	feature_id[1439].value > threshold=0.5518321692943573
node_48: feature_name=cg17843418	feature_id[4868].value > threshold=0.34969255328178406
node_52: feature_name=cg24407065	feature_id[4522].value > threshold=0.5364363789558411
node_56: feature_name=cg07281938	feature_id[249].value > threshold=0.7506992518901825
node_78: feature_name=cg21759907	feature_id[1650].value > threshold=0.46823520958423615

node_98: feature_name=cg10886334	feature_id[3975].value > threshold=0.43698127567768097
node_102: feature_name=cg09373983	feature_id[5025].value > threshold=0.5575815737247467
node_106: feature_name=cg12948116	feature_id[5360].value <= threshold=0.5658791363239288
node_107: feature_name=cg21906519	feature_id[5251].value > threshold=0.6089984774589539
node_111: feature_name=cg17537493	feature_id[4217].value > threshold=0.5825372636318207
node_117: feature_name=cg15720017	feature_id[4443].value > threshold=0.7009969055652618
node_119: feature_name=cg06038180	feature_id[3480].value > threshold=0.4583848565816879
node_121: feature_name=cg08619651	feature_id[4882].value <= threshold=0.2105495035648346
node_122: feature_name=cg01014262	feature_id[1911].value <= threshold=0.5327437818050385
node_123: feature_name=cg16863382	feature_id[2922].value > threshold=0.7200668156147003
node_133: feature_name=cg06989443	feature_id[2327].value <= threshold=0.6579365730285645
node_134: feature_name=cg07344990	feature_id[2398].value > threshold=0.5562876164913177
node_138: feature_name=cg02966841	feature_id[2199].value > threshold=0.7744501233100891
node_204: feature_name=cg12120430	feature_id[3727].value > threshold=0.5775820016860962
node_206: feature_name=cg18386876	feature_id[4066].value > threshold=0.6847257018089294
node_208: feature_name=cg02053092	feature_id[3610].value > threshold=0.39507485926151276
node_210: feature_name=cg10298992	feature_id[3603].value > threshold=0.5707973837852478
node_228: feature_name=cg03840920	feature_id[4524].value > threshold=0.5365143716335297
node_234: feature_name=cg25364972	feature_id[1792].value > threshold=0.2418053299188614
node_238: feature_name=cg23485307	feature_id[221].value > threshold=0.14884746074676514
node_242: feature_name=cg04873169	feature_id[4696].value > threshold=0.6058756709098816
node_244: feature_name=cg17009731	feature_id[3218].value > threshold=0.6602252125740051

node_246: feature_name=cg18253910	feature_id[4458].value > threshold=0.6904322504997253
node_250: feature_name=cg04774476	feature_id[1257].value > threshold=0.5675267577171326
node_256: feature_name=cg19259111	feature_id[63].value > threshold=0.40771469473838806
node_264: feature_name=cg21483216	feature_id[5253].value > threshold=0.6332719326019287
node_266: feature_name=cg04026354	feature_id[3502].value <= threshold=0.42332448065280914
node_267: feature_name=cg11444072	feature_id[116].value <= threshold=0.4940422773361206
node_268: feature_name=cg25424742	feature_id[2698].value <= threshold=0.762436032295227
Class: reactive tissue (REA)	
Rules_34	passed counts:10
node_0: feature_name=cg11915444	feature_id[2002].value > threshold=0.3402601182460785
node_10: feature_name=cg26016985	feature_id[4407].value > threshold=0.6493876278400421
node_16: feature_name=cg23157618	feature_id[5511].value > threshold=0.5759606957435608
node_20: feature_name=cg10480329	feature_id[1439].value > threshold=0.5518321692943573
node_48: feature_name=cg17843418	feature_id[4868].value > threshold=0.34969255328178406
node_52: feature_name=cg24407065	feature_id[4522].value > threshold=0.5364363789558411
node_56: feature_name=cg07281938	feature_id[249].value > threshold=0.7506992518901825
node_78: feature_name=cg21759907	feature_id[1650].value > threshold=0.46823520958423615
node_98: feature_name=cg10886334	feature_id[3975].value > threshold=0.43698127567768097
node_102: feature_name=cg09373983	feature_id[5025].value > threshold=0.5575815737247467
node_106: feature_name=cg12948116	feature_id[5360].value <= threshold=0.5658791363239288
node_107: feature_name=cg21906519	feature_id[5251].value > threshold=0.6089984774589539
node_111: feature_name=cg17537493	feature_id[4217].value > threshold=0.5825372636318207

node_117: feature_name=cg15720017	feature_id[4443].value > threshold=0.7009969055652618
node_119: feature_name=cg06038180	feature_id[3480].value > threshold=0.4583848565816879
node_121: feature_name=cg08619651	feature_id[4882].value <= threshold=0.2105495035648346
node_122: feature_name=cg01014262	feature_id[1911].value <= threshold=0.5327437818050385
node_123: feature_name=cg16863382	feature_id[2922].value > threshold=0.7200668156147003
node_133: feature_name=cg06989443	feature_id[2327].value <= threshold=0.6579365730285645
node_134: feature_name=cg07344990	feature_id[2398].value > threshold=0.5562876164913177
node_138: feature_name=cg02966841	feature_id[2199].value > threshold=0.7744501233100891
node_204: feature_name=cg12120430	feature_id[3727].value > threshold=0.5775820016860962
node_206: feature_name=cg18386876	feature_id[4066].value > threshold=0.6847257018089294
node_208: feature_name=cg02053092	feature_id[3610].value > threshold=0.39507485926151276
node_210: feature_name=cg10298992	feature_id[3603].value > threshold=0.5707973837852478
node_228: feature_name=cg03840920	feature_id[4524].value > threshold=0.5365143716335297
node_234: feature_name=cg25364972	feature_id[1792].value > threshold=0.2418053299188614
node_238: feature_name=cg23485307	feature_id[221].value > threshold=0.14884746074676514
node_242: feature_name=cg04873169	feature_id[4696].value > threshold=0.6058756709098816
node_244: feature_name=cg17009731	feature_id[3218].value > threshold=0.6602252125740051
node_246: feature_name=cg18253910	feature_id[4458].value > threshold=0.6904322504997253
node_250: feature_name=cg04774476	feature_id[1257].value <= threshold=0.5675267577171326
node_251: feature_name=cg08276755	feature_id[769].value > threshold=0.5270942002534866
Class: nodular fasciitis(NFA)	
Rules_35	passed counts:10

node_0: feature_name=cg11915444	feature_id[2002].value > threshold=0.3402601182460785
node_10: feature_name=cg26016985	feature_id[4407].value > threshold=0.6493876278400421
node_16: feature_name=cg23157618	feature_id[5511].value > threshold=0.5759606957435608
node_20: feature_name=cg10480329	feature_id[1439].value > threshold=0.5518321692943573
node_48: feature_name=cg17843418	feature_id[4868].value > threshold=0.34969255328178406
node_52: feature_name=cg24407065	feature_id[4522].value > threshold=0.5364363789558411
node_56: feature_name=cg07281938	feature_id[249].value > threshold=0.7506992518901825
node_78: feature_name=cg21759907	feature_id[1650].value > threshold=0.46823520958423615
node_98: feature_name=cg10886334	feature_id[3975].value > threshold=0.43698127567768097
node_102: feature_name=cg09373983	feature_id[5025].value > threshold=0.5575815737247467
node_106: feature_name=cg12948116	feature_id[5360].value <= threshold=0.5658791363239288
node_107: feature_name=cg21906519	feature_id[5251].value > threshold=0.6089984774589539
node_111: feature_name=cg17537493	feature_id[4217].value > threshold=0.5825372636318207
node_117: feature_name=cg15720017	feature_id[4443].value > threshold=0.7009969055652618
node_119: feature_name=cg06038180	feature_id[3480].value > threshold=0.4583848565816879
node_121: feature_name=cg08619651	feature_id[4882].value <= threshold=0.2105495035648346
node_122: feature_name=cg01014262	feature_id[1911].value <= threshold=0.5327437818050385
node_123: feature_name=cg16863382	feature_id[2922].value > threshold=0.7200668156147003
node_133: feature_name=cg06989443	feature_id[2327].value <= threshold=0.6579365730285645
node_134: feature_name=cg07344990	feature_id[2398].value > threshold=0.5562876164913177
node_138: feature_name=cg02966841	feature_id[2199].value > threshold=0.7744501233100891
node_204: feature_name=cg12120430	feature_id[3727].value > threshold=0.5775820016860962

node_206: feature_name=cg18386876	feature_id[4066].value > threshold=0.6847257018089294
node_208: feature_name=cg02053092	feature_id[3610].value > threshold=0.3950748592615l276
node_210: feature_name=cg10298992	feature_id[3603].value > threshold=0.5707973837852478
node_228: feature_name=cg03840920	feature_id[4524].value > threshold=0.5365143716335297
node_234: feature_name=cg25364972	feature_id[1792].value > threshold=0.2418053299188614
node_238: feature_name=cg23485307	feature_id[221].value > threshold=0.14884746074676514
node_242: feature_name=cg04873169	feature_id[4696].value <= threshold=0.6058756709098816
Class: cutaneous squamous cell carcinoma (SCC (CUT))	
Rules_36	passed counts:10
node_0: feature_name=cg11915444	feature_id[2002].value > threshold=0.3402601182460785
node_10: feature_name=cg26016985	feature_id[4407].value > threshold=0.6493876278400421
node_16: feature_name=cg23157618	feature_id[5511].value > threshold=0.5759606957435608
node_20: feature_name=cg10480329	feature_id[1439].value > threshold=0.5518321692943573
node_48: feature_name=cg17843418	feature_id[4868].value > threshold=0.34969255328178406
node_52: feature_name=cg24407065	feature_id[4522].value > threshold=0.5364363789558411
node_56: feature_name=cg07281938	feature_id[249].value > threshold=0.7506992518901825
node_78: feature_name=cg21759907	feature_id[1650].value > threshold=0.46823520958423615
node_98: feature_name=cg10886334	feature_id[3975].value > threshold=0.43698127567768097
node_102: feature_name=cg09373983	feature_id[5025].value > threshold=0.5575815737247467
node_106: feature_name=cg12948116	feature_id[5360].value <= threshold=0.5658791363239288
node_107: feature_name=cg21906519	feature_id[5251].value > threshold=0.6089984774589539

node_111: feature_name=cg17537493	feature_id[4217].value > threshold=0.5825372636318207
node_117: feature_name=cg15720017	feature_id[4443].value > threshold=0.7009969055652618
node_119: feature_name=cg06038180	feature_id[3480].value > threshold=0.4583848565816879
node_121: feature_name=cg08619651	feature_id[4882].value <= threshold=0.2105495035648346
node_122: feature_name=cg01014262	feature_id[1911].value <= threshold=0.5327437818050385
node_123: feature_name=cg16863382	feature_id[2922].value > threshold=0.7200668156147003
node_133: feature_name=cg06989443	feature_id[2327].value <= threshold=0.6579365730285645
node_134: feature_name=cg07344990	feature_id[2398].value > threshold=0.5562876164913177
node_138: feature_name=cg02966841	feature_id[2199].value > threshold=0.7744501233100891
node_204: feature_name=cg12120430	feature_id[3727].value > threshold=0.5775820016860962
node_206: feature_name=cg18386876	feature_id[4066].value > threshold=0.6847257018089294
node_208: feature_name=cg02053092	feature_id[3610].value > threshold=0.39507485926151276
node_210: feature_name=cg10298992	feature_id[3603].value > threshold=0.5707973837852478
node_228: feature_name=cg03840920	feature_id[4524].value > threshold=0.5365143716335297
node_234: feature_name=cg25364972	feature_id[1792].value > threshold=0.2418053299188614
node_238: feature_name=cg23485307	feature_id[221].value <= threshold=0.14884746074676514
node_239: feature_name=cg13972711	feature_id[4461].value > threshold=0.8822346031665802
Class: control (CTRL)	
Rules_37	passed counts:9
node_0: feature_name=cg11915444	feature_id[2002].value > threshold=0.3402601182460785
node_10: feature_name=cg26016985	feature_id[4407].value > threshold=0.6493876278400421
node_16: feature_name=cg23157618	feature_id[5511].value > threshold=0.5759606957435608

node_20: feature_name=cg10480329	feature_id[1439].value > threshold=0.5518321692943573
node_48: feature_name=cg17843418	feature_id[4868].value > threshold=0.34969255328178406
node_52: feature_name=cg24407065	feature_id[4522].value > threshold=0.5364363789558411
node_56: feature_name=cg07281938	feature_id[249].value > threshold=0.7506992518901825
node_78: feature_name=cg21759907	feature_id[1650].value > threshold=0.46823520958423615
node_98: feature_name=cg10886334	feature_id[3975].value > threshold=0.43698127567768097
node_102: feature_name=cg09373983	feature_id[5025].value > threshold=0.5575815737247467
node_106: feature_name=cg12948116	feature_id[5360].value <= threshold=0.5658791363239288
node_107: feature_name=cg21906519	feature_id[5251].value > threshold=0.6089984774589539
node_111: feature_name=cg17537493	feature_id[4217].value > threshold=0.5825372636318207
node_117: feature_name=cg15720017	feature_id[4443].value > threshold=0.7009969055652618
node_119: feature_name=cg06038180	feature_id[3480].value > threshold=0.4583848565816879
node_121: feature_name=cg08619651	feature_id[4882].value <= threshold=0.2105495035648346
node_122: feature_name=cg01014262	feature_id[1911].value <= threshold=0.5327437818050385
node_123: feature_name=cg16863382	feature_id[2922].value > threshold=0.7200668156147003
node_133: feature_name=cg06989443	feature_id[2327].value <= threshold=0.6579365730285645
node_134: feature_name=cg07344990	feature_id[2398].value > threshold=0.5562876164913177
node_138: feature_name=cg02966841	feature_id[2199].value > threshold=0.7744501233100891
node_204: feature_name=cg12120430	feature_id[3727].value > threshold=0.5775820016860962
node_206: feature_name=cg18386876	feature_id[4066].value > threshold=0.6847257018089294
node_208: feature_name=cg02053092	feature_id[3610].value > threshold=0.39507485926151276
node_210: feature_name=cg10298992	feature_id[3603].value > threshold=0.5707973837852478

node_228: feature_name=cg03840920	feature_id[4524].value > threshold=0.5365143716335297
node_234: feature_name=cg25364972	feature_id[1792].value > threshold=0.2418053299188614
node_238: feature_name=cg23485307	feature_id[221].value > threshold=0.14884746074676514
node_242: feature_name=cg04873169	feature_id[4696].value > threshold=0.6058756709098816
node_244: feature_name=cg17009731	feature_id[3218].value > threshold=0.6602252125740051
node_246: feature_name=cg18253910	feature_id[4458].value > threshold=0.6904322504997253
node_250: feature_name=cg04774476	feature_id[1257].value > threshold=0.5675267577171326
node_256: feature_name=cg19259111	feature_id[63].value > threshold=0.40771469473838806
node_264: feature_name=cg21483216	feature_id[5253].value > threshold=0.6332719326019287
node_266: feature_name=cg04026354	feature_id[3502].value <= threshold=0.42332448065280914
node_267: feature_name=cg11444072	feature_id[116].value > threshold=0.4940422773361206
node_273: feature_name=cg08571020	feature_id[381].value > threshold=0.4616774618625641
node_275: feature_name=cg08331427	feature_id[2081].value > threshold=0.5443233847618103
node_281: feature_name=cg00216961	feature_id[945].value > threshold=0.4166988283395767
node_333: feature_name=cg23366832	feature_id[2439].value > threshold=0.8551825881004333
Class: lipoma (LIPO)	
Rules_38	passed counts:9
node_0: feature_name=cg11915444	feature_id[2002].value > threshold=0.3402601182460785
node_10: feature_name=cg26016985	feature_id[4407].value > threshold=0.6493876278400421
node_16: feature_name=cg23157618	feature_id[5511].value > threshold=0.5759606957435608
node_20: feature_name=cg10480329	feature_id[1439].value > threshold=0.5518321692943573
node_48: feature_name=cg17843418	feature_id[4868].value > threshold=0.34969255328178406

node_52: feature_name=cg24407065	feature_id[4522].value > threshold=0.5364363789558411
node_56: feature_name=cg07281938	feature_id[249].value > threshold=0.7506992518901825
node_78: feature_name=cg21759907	feature_id[1650].value > threshold=0.46823520958423615
node_98: feature_name=cg10886334	feature_id[3975].value > threshold=0.43698127567768097
node_102: feature_name=cg09373983	feature_id[5025].value > threshold=0.5575815737247467
node_106: feature_name=cg12948116	feature_id[5360].value <= threshold=0.5658791363239288
node_107: feature_name=cg21906519	feature_id[5251].value > threshold=0.6089984774589539
node_111: feature_name=cg17537493	feature_id[4217].value > threshold=0.5825372636318207
node_117: feature_name=cg15720017	feature_id[4443].value > threshold=0.7009969055652618
node_119: feature_name=cg06038180	feature_id[3480].value > threshold=0.4583848565816879
node_121: feature_name=cg08619651	feature_id[4882].value <= threshold=0.2105495035648346
node_122: feature_name=cg01014262	feature_id[1911].value <= threshold=0.5327437818050385
node_123: feature_name=cg16863382	feature_id[2922].value > threshold=0.7200668156147003
node_133: feature_name=cg06989443	feature_id[2327].value <= threshold=0.6579365730285645
node_134: feature_name=cg07344990	feature_id[2398].value > threshold=0.5562876164913177
node_138: feature_name=cg02966841	feature_id[2199].value > threshold=0.7744501233100891
node_204: feature_name=cg12120430	feature_id[3727].value > threshold=0.5775820016860962
node_206: feature_name=cg18386876	feature_id[4066].value > threshold=0.6847257018089294
node_208: feature_name=cg02053092	feature_id[3610].value > threshold=0.39507485926151276
node_210: feature_name=cg10298992	feature_id[3603].value > threshold=0.5707973837852478
node_228: feature_name=cg03840920	feature_id[4524].value > threshold=0.5365143716335297
node_234: feature_name=cg25364972	feature_id[1792].value > threshold=0.2418053299188614

node_238: feature_name=cg23485307	feature_id[221].value > threshold=0.14884746074676514
node_242: feature_name=cg04873169	feature_id[4696].value > threshold=0.6058756709098816
node_244: feature_name=cg17009731	feature_id[3218].value > threshold=0.6602252125740051
node_246: feature_name=cg18253910	feature_id[4458].value > threshold=0.6904322504997253
node_250: feature_name=cg04774476	feature_id[1257].value > threshold=0.5675267577171326
node_256: feature_name=cg19259111	feature_id[63].value > threshold=0.40771469473838806
node_264: feature_name=cg21483216	feature_id[5253].value > threshold=0.6332719326019287
node_266: feature_name=cg04026354	feature_id[3502].value <= threshold=0.42332448065280914
node_267: feature_name=cg11444072	feature_id[116].value > threshold=0.4940422773361206
node_273: feature_name=cg08571020	feature_id[381].value > threshold=0.4616774618625641
node_275: feature_name=cg08331427	feature_id[2081].value > threshold=0.5443233847618103
node_281: feature_name=cg00216961	feature_id[945].value <= threshold=0.4166988283395767
node_282: feature_name=cg04716580	feature_id[1390].value > threshold=0.4204266667366028
node_286: feature_name=cg07891483	feature_id[1877].value > threshold=0.829958975315094
node_288: feature_name=cg01475325	feature_id[3969].value > threshold=0.45323844254016876
node_292: feature_name=cg19702397	feature_id[2066].value > threshold=0.3536848723888397
node_294: feature_name=cg21936959	feature_id[595].value <= threshold=0.5567200481891632
node_295: feature_name=cg05251593	feature_id[515].value > threshold=0.5597732663154602
Class: well differentiated liposarcoma (WDLS)/dedifferentiated liposarcoma (DDLS)	
Rules_39	passed counts:9
node_0: feature_name=cg11915444	feature_id[2002].value > threshold=0.3402601182460785

node_10: feature_name=cg26016985	feature_id[4407].value > threshold=0.6493876278400421
node_16: feature_name=cg23157618	feature_id[5511].value > threshold=0.5759606957435608
node_20: feature_name=cg10480329	feature_id[1439].value > threshold=0.5518321692943573
node_48: feature_name=cg17843418	feature_id[4868].value > threshold=0.34969255328178406
node_52: feature_name=cg24407065	feature_id[4522].value > threshold=0.5364363789558411
node_56: feature_name=cg07281938	feature_id[249].value > threshold=0.7506992518901825
node_78: feature_name=cg21759907	feature_id[1650].value > threshold=0.46823520958423615
node_98: feature_name=cg10886334	feature_id[3975].value > threshold=0.43698127567768097
node_102: feature_name=cg09373983	feature_id[5025].value > threshold=0.5575815737247467
node_106: feature_name=cg12948116	feature_id[5360].value <= threshold=0.5658791363239288
node_107: feature_name=cg21906519	feature_id[5251].value > threshold=0.6089984774589539
node_111: feature_name=cg17537493	feature_id[4217].value > threshold=0.5825372636318207
node_117: feature_name=cg15720017	feature_id[4443].value > threshold=0.7009969055652618
node_119: feature_name=cg06038180	feature_id[3480].value > threshold=0.4583848565816879
node_121: feature_name=cg08619651	feature_id[4882].value <= threshold=0.2105495035648346
node_122: feature_name=cg01014262	feature_id[1911].value <= threshold=0.5327437818050385
node_123: feature_name=cg16863382	feature_id[2922].value > threshold=0.7200668156147003
node_133: feature_name=cg06989443	feature_id[2327].value <= threshold=0.6579365730285645
node_134: feature_name=cg07344990	feature_id[2398].value > threshold=0.5562876164913177
node_138: feature_name=cg02966841	feature_id[2199].value > threshold=0.7744501233100891
node_204: feature_name=cg12120430	feature_id[3727].value > threshold=0.5775820016860962
node_206: feature_name=cg18386876	feature_id[4066].value > threshold=0.6847257018089294

node_208: feature_name=cg02053092	feature_id[3610].value > threshold=0.39507485926151276
node_210: feature_name=cg10298992	feature_id[3603].value > threshold=0.5707973837852478
node_228: feature_name=cg03840920	feature_id[4524].value > threshold=0.5365143716335297
node_234: feature_name=cg25364972	feature_id[1792].value > threshold=0.2418053299188614
node_238: feature_name=cg23485307	feature_id[221].value > threshold=0.14884746074676514
node_242: feature_name=cg04873169	feature_id[4696].value > threshold=0.6058756709098816
node_244: feature_name=cg17009731	feature_id[3218].value > threshold=0.6602252125740051
node_246: feature_name=cg18253910	feature_id[4458].value <= threshold=0.6904322504997253
node_247: feature_name=cg01559356	feature_id[3236].value > threshold=0.089854396879673
Class: neurofibroma (NFB)	
Rules_40	passed counts:9
node_0: feature_name=cg11915444	feature_id[2002].value > threshold=0.3402601182460785
node_10: feature_name=cg26016985	feature_id[4407].value > threshold=0.6493876278400421
node_16: feature_name=cg23157618	feature_id[5511].value > threshold=0.5759606957435608
node_20: feature_name=cg10480329	feature_id[1439].value > threshold=0.5518321692943573
node_48: feature_name=cg17843418	feature_id[4868].value > threshold=0.34969255328178406
node_52: feature_name=cg24407065	feature_id[4522].value > threshold=0.5364363789558411
node_56: feature_name=cg07281938	feature_id[249].value > threshold=0.7506992518901825
node_78: feature_name=cg21759907	feature_id[1650].value > threshold=0.46823520958423615
node_98: feature_name=cg10886334	feature_id[3975].value > threshold=0.43698127567768097
node_102: feature_name=cg09373983	feature_id[5025].value > threshold=0.5575815737247467
node_106: feature_name=cg12948116	feature_id[5360].value <= threshold=0.5658791363239288

node_107: feature_name=cg21906519	feature_id[5251].value > threshold=0.6089984774589539
node_111: feature_name=cg17537493	feature_id[4217].value > threshold=0.5825372636318207
node_117: feature_name=cg15720017	feature_id[4443].value > threshold=0.7009969055652618
node_119: feature_name=cg06038180	feature_id[3480].value > threshold=0.4583848565816879
node_121: feature_name=cg08619651	feature_id[4882].value <= threshold=0.2105495035648346
node_122: feature_name=cg01014262	feature_id[1911].value <= threshold=0.5327437818050385
node_123: feature_name=cg16863382	feature_id[2922].value > threshold=0.7200668156147003
node_133: feature_name=cg06989443	feature_id[2327].value <= threshold=0.6579365730285645
node_134: feature_name=cg07344990	feature_id[2398].value > threshold=0.5562876164913177
node_138: feature_name=cg02966841	feature_id[2199].value > threshold=0.7744501233100891
node_204: feature_name=cg12120430	feature_id[3727].value > threshold=0.5775820016860962
node_206: feature_name=cg18386876	feature_id[4066].value > threshold=0.6847257018089294
node_208: feature_name=cg02053092	feature_id[3610].value > threshold=0.39507485926151276
node_210: feature_name=cg10298992	feature_id[3603].value > threshold=0.5707973837852478
node_228: feature_name=cg03840920	feature_id[4524].value > threshold=0.5365143716335297
node_234: feature_name=cg25364972	feature_id[1792].value > threshold=0.2418053299188614
node_238: feature_name=cg23485307	feature_id[221].value > threshold=0.14884746074676514
node_242: feature_name=cg04873169	feature_id[4696].value > threshold=0.6058756709098816
node_244: feature_name=cg17009731	feature_id[3218].value <= threshold=0.6602252125740051
Class: extraskeletal myxoid chondrosarcoma (EMCS)	
Rules_41	passed counts:9

node_0: feature_name=cg11915444	feature_id[2002].value > threshold=0.3402601182460785
node_10: feature_name=cg26016985	feature_id[4407].value > threshold=0.6493876278400421
node_16: feature_name=cg23157618	feature_id[5511].value > threshold=0.5759606957435608
node_20: feature_name=cg10480329	feature_id[1439].value <= threshold=0.5518321692943573
node_21: feature_name=cg25510609	feature_id[149].value <= threshold=0.08151235431432724
node_22: feature_name=cg23850277	feature_id[2159].value > threshold=0.5682950615882874
Class: giant cell tumour of bone (GCTB)	
Rules_42	passed counts:8
node_0: feature_name=cg11915444	feature_id[2002].value > threshold=0.3402601182460785
node_10: feature_name=cg26016985	feature_id[4407].value > threshold=0.6493876278400421
node_16: feature_name=cg23157618	feature_id[5511].value > threshold=0.5759606957435608
node_20: feature_name=cg10480329	feature_id[1439].value > threshold=0.5518321692943573
node_48: feature_name=cg17843418	feature_id[4868].value > threshold=0.34969255328178406
node_52: feature_name=cg24407065	feature_id[4522].value > threshold=0.5364363789558411
node_56: feature_name=cg07281938	feature_id[249].value > threshold=0.7506992518901825
node_78: feature_name=cg21759907	feature_id[1650].value > threshold=0.46823520958423615
node_98: feature_name=cg10886334	feature_id[3975].value > threshold=0.43698127567768097
node_102: feature_name=cg09373983	feature_id[5025].value > threshold=0.5575815737247467
node_106: feature_name=cg12948116	feature_id[5360].value <= threshold=0.5658791363239288
node_107: feature_name=cg21906519	feature_id[5251].value > threshold=0.6089984774589539
node_111: feature_name=cg17537493	feature_id[4217].value > threshold=0.5825372636318207
node_117: feature_name=cg15720017	feature_id[4443].value > threshold=0.7009969055652618

node_119: feature_name=cg06038180	feature_id[3480].value > threshold=0.4583848565816879
node_121: feature_name=cg08619651	feature_id[4882].value <= threshold=0.2105495035648346
node_122: feature_name=cg01014262	feature_id[1911].value <= threshold=0.5327437818050385
node_123: feature_name=cg16863382	feature_id[2922].value > threshold=0.7200668156147003
node_133: feature_name=cg06989443	feature_id[2327].value <= threshold=0.6579365730285645
node_134: feature_name=cg07344990	feature_id[2398].value > threshold=0.5562876164913177
node_138: feature_name=cg02966841	feature_id[2199].value > threshold=0.7744501233100891
node_204: feature_name=cg12120430	feature_id[3727].value > threshold=0.5775820016860962
node_206: feature_name=cg18386876	feature_id[4066].value > threshold=0.6847257018089294
node_208: feature_name=cg02053092	feature_id[3610].value > threshold=0.39507485926151276
node_210: feature_name=cg10298992	feature_id[3603].value > threshold=0.5707973837852478
node_228: feature_name=cg03840920	feature_id[4524].value > threshold=0.5365143716335297
node_234: feature_name=cg25364972	feature_id[1792].value > threshold=0.2418053299188614
node_238: feature_name=cg23485307	feature_id[221].value > threshold=0.14884746074676514
node_242: feature_name=cg04873169	feature_id[4696].value > threshold=0.6058756709098816
node_244: feature_name=cg17009731	feature_id[3218].value > threshold=0.6602252125740051
node_246: feature_name=cg18253910	feature_id[4458].value > threshold=0.6904322504997253
node_250: feature_name=cg04774476	feature_id[1257].value > threshold=0.5675267577171326
node_256: feature_name=cg19259111	feature_id[63].value > threshold=0.40771469473838806
node_264: feature_name=cg21483216	feature_id[5253].value > threshold=0.6332719326019287
node_266: feature_name=cg04026354	feature_id[3502].value <= threshold=0.42332448065280914
node_267: feature_name=cg11444072	feature_id[116].value > threshold=0.4940422773361206

node_273: feature_name=cg08571020	feature_id[381].value > threshold=0.4616774618625641
node_275: feature_name=cg08331427	feature_id[2081].value <= threshold=0.5443233847618103
node_276: feature_name=cg23936766	feature_id[683].value <= threshold=0.3338508605957031
Class: epithelioid haemangioendothelioma (EHE)	
Rules_43	passed counts:8
node_0: feature_name=cg11915444	feature_id[2002].value > threshold=0.3402601182460785
node_10: feature_name=cg26016985	feature_id[4407].value > threshold=0.6493876278400421
node_16: feature_name=cg23157618	feature_id[5511].value > threshold=0.5759606957435608
node_20: feature_name=cg10480329	feature_id[1439].value > threshold=0.5518321692943573
node_48: feature_name=cg17843418	feature_id[4868].value > threshold=0.34969255328178406
node_52: feature_name=cg24407065	feature_id[4522].value > threshold=0.5364363789558411
node_56: feature_name=cg07281938	feature_id[249].value > threshold=0.7506992518901825
node_78: feature_name=cg21759907	feature_id[1650].value > threshold=0.46823520958423615
node_98: feature_name=cg10886334	feature_id[3975].value > threshold=0.43698127567768097
node_102: feature_name=cg09373983	feature_id[5025].value > threshold=0.5575815737247467
node_106: feature_name=cg12948116	feature_id[5360].value <= threshold=0.5658791363239288
node_107: feature_name=cg21906519	feature_id[5251].value > threshold=0.6089984774589539
node_111: feature_name=cg17537493	feature_id[4217].value > threshold=0.5825372636318207
node_117: feature_name=cg15720017	feature_id[4443].value > threshold=0.7009969055652618
node_119: feature_name=cg06038180	feature_id[3480].value > threshold=0.4583848565816879
node_121: feature_name=cg08619651	feature_id[4882].value <= threshold=0.2105495035648346
node_122: feature_name=cg01014262	feature_id[1911].value <= threshold=0.5327437818050385

node_123: feature_name=cg16863382	feature_id[2922].value > threshold=0.7200668156147003
node_133: feature_name=cg06989443	feature_id[2327].value <= threshold=0.6579365730285645
node_134: feature_name=cg07344990	feature_id[2398].value > threshold=0.5562876164913177
node_138: feature_name=cg02966841	feature_id[2199].value > threshold=0.7744501233100891
node_204: feature_name=cg12120430	feature_id[3727].value > threshold=0.5775820016860962
node_206: feature_name=cg18386876	feature_id[4066].value > threshold=0.6847257018089294
node_208: feature_name=cg02053092	feature_id[3610].value > threshold=0.39507485926151276
node_210: feature_name=cg10298992	feature_id[3603].value > threshold=0.5707973837852478
node_228: feature_name=cg03840920	feature_id[4524].value > threshold=0.5365143716335297
node_234: feature_name=cg25364972	feature_id[1792].value > threshold=0.2418053299188614
node_238: feature_name=cg23485307	feature_id[221].value > threshold=0.14884746074676514
node_242: feature_name=cg04873169	feature_id[4696].value > threshold=0.6058756709098816
node_244: feature_name=cg17009731	feature_id[3218].value > threshold=0.6602252125740051
node_246: feature_name=cg18253910	feature_id[4458].value > threshold=0.6904322504997253
node_250: feature_name=cg04774476	feature_id[1257].value > threshold=0.5675267577171326
node_256: feature_name=cg19259111	feature_id[63].value > threshold=0.40771469473838806
node_264: feature_name=cg21483216	feature_id[5253].value <= threshold=0.6332719326019287
Class: muscle tissue (MUS)	
Rules_44	passed counts:8
node_0: feature_name=cg11915444	feature_id[2002].value > threshold=0.3402601182460785
node_10: feature_name=cg26016985	feature_id[4407].value > threshold=0.6493876278400421
node_16: feature_name=cg23157618	feature_id[5511].value > threshold=0.5759606957435608

node_20: feature_name=cg10480329	feature_id[1439].value > threshold=0.5518321692943573
node_48: feature_name=cg17843418	feature_id[4868].value > threshold=0.34969255328178406
node_52: feature_name=cg24407065	feature_id[4522].value > threshold=0.5364363789558411
node_56: feature_name=cg07281938	feature_id[249].value > threshold=0.7506992518901825
node_78: feature_name=cg21759907	feature_id[1650].value > threshold=0.46823520958423615
node_98: feature_name=cg10886334	feature_id[3975].value > threshold=0.43698127567768097
node_102: feature_name=cg09373983	feature_id[5025].value > threshold=0.5575815737247467
node_106: feature_name=cg12948116	feature_id[5360].value <= threshold=0.5658791363239288
node_107: feature_name=cg21906519	feature_id[5251].value > threshold=0.6089984774589539
node_111: feature_name=cg17537493	feature_id[4217].value > threshold=0.5825372636318207
node_117: feature_name=cg15720017	feature_id[4443].value > threshold=0.7009969055652618
node_119: feature_name=cg06038180	feature_id[3480].value > threshold=0.4583848565816879
node_121: feature_name=cg08619651	feature_id[4882].value <= threshold=0.2105495035648346
node_122: feature_name=cg01014262	feature_id[1911].value <= threshold=0.5327437818050385
node_123: feature_name=cg16863382	feature_id[2922].value > threshold=0.7200668156147003
node_133: feature_name=cg06989443	feature_id[2327].value <= threshold=0.6579365730285645
node_134: feature_name=cg07344990	feature_id[2398].value > threshold=0.5562876164913177
node_138: feature_name=cg02966841	feature_id[2199].value > threshold=0.7744501233100891
node_204: feature_name=cg12120430	feature_id[3727].value > threshold=0.5775820016860962
node_206: feature_name=cg18386876	feature_id[4066].value > threshold=0.6847257018089294
node_208: feature_name=cg02053092	feature_id[3610].value > threshold=0.39507485926151276
node_210: feature_name=cg10298992	feature_id[3603].value > threshold=0.5707973837852478

node_228: feature_name=cg03840920	feature_id[4524].value > threshold=0.5365143716335297
node_234: feature_name=cg25364972	feature_id[1792].value > threshold=0.2418053299188614
node_238: feature_name=cg23485307	feature_id[221].value > threshold=0.14884746074676514
node_242: feature_name=cg04873169	feature_id[4696].value > threshold=0.6058756709098816
node_244: feature_name=cg17009731	feature_id[3218].value > threshold=0.6602252125740051
node_246: feature_name=cg18253910	feature_id[4458].value > threshold=0.6904322504997253
node_250: feature_name=cg04774476	feature_id[1257].value <= threshold=0.5675267577171326
node_251: feature_name=cg08276755	feature_id[769].value <= threshold=0.5270942002534866
node_252: feature_name=cg09230938	feature_id[163].value <= threshold=0.465945765376091
Class: myositis ossificans (MO)	
Rules_45	passed counts:7
node_0: feature_name=cg11915444	feature_id[2002].value > threshold=0.3402601182460785
node_10: feature_name=cg26016985	feature_id[4407].value > threshold=0.6493876278400421
node_16: feature_name=cg23157618	feature_id[5511].value > threshold=0.5759606957435608
node_20: feature_name=cg10480329	feature_id[1439].value > threshold=0.5518321692943573
node_48: feature_name=cg17843418	feature_id[4868].value > threshold=0.34969255328178406
node_52: feature_name=cg24407065	feature_id[4522].value > threshold=0.5364363789558411
node_56: feature_name=cg07281938	feature_id[249].value > threshold=0.7506992518901825
node_78: feature_name=cg21759907	feature_id[1650].value > threshold=0.46823520958423615
node_98: feature_name=cg10886334	feature_id[3975].value > threshold=0.43698127567768097
node_102: feature_name=cg09373983	feature_id[5025].value > threshold=0.5575815737247467
node_106: feature_name=cg12948116	feature_id[5360].value <= threshold=0.5658791363239288

node_107: feature_name=cg21906519	feature_id[5251].value > threshold=0.6089984774589539
node_111: feature_name=cg17537493	feature_id[4217].value > threshold=0.5825372636318207
node_117: feature_name=cg15720017	feature_id[4443].value > threshold=0.7009969055652618
node_119: feature_name=cg06038180	feature_id[3480].value > threshold=0.4583848565816879
node_121: feature_name=cg08619651	feature_id[4882].value <= threshold=0.2105495035648346
node_122: feature_name=cg01014262	feature_id[1911].value <= threshold=0.5327437818050385
node_123: feature_name=cg16863382	feature_id[2922].value > threshold=0.7200668156147003
node_133: feature_name=cg06989443	feature_id[2327].value <= threshold=0.6579365730285645
node_134: feature_name=cg07344990	feature_id[2398].value > threshold=0.5562876164913177
node_138: feature_name=cg02966841	feature_id[2199].value > threshold=0.7744501233100891
node_204: feature_name=cg12120430	feature_id[3727].value > threshold=0.5775820016860962
node_206: feature_name=cg18386876	feature_id[4066].value > threshold=0.6847257018089294
node_208: feature_name=cg02053092	feature_id[3610].value > threshold=0.39507485926151276
node_210: feature_name=cg10298992	feature_id[3603].value > threshold=0.5707973837852478
node_228: feature_name=cg03840920	feature_id[4524].value > threshold=0.5365143716335297
node_234: feature_name=cg25364972	feature_id[1792].value > threshold=0.2418053299188614
node_238: feature_name=cg23485307	feature_id[221].value > threshold=0.14884746074676514
node_242: feature_name=cg04873169	feature_id[4696].value > threshold=0.6058756709098816
node_244: feature_name=cg17009731	feature_id[3218].value > threshold=0.6602252125740051
node_246: feature_name=cg18253910	feature_id[4458].value > threshold=0.6904322504997253
node_250: feature_name=cg04774476	feature_id[1257].value > threshold=0.5675267577171326
node_256: feature_name=cg19259111	feature_id[63].value > threshold=0.40771469473838806

node_264: feature_name=cg21483216	feature_id[5253].value > threshold=0.6332719326019287
node_266: feature_name=cg04026354	feature_id[3502].value <= threshold=0.42332448065280914
node_267: feature_name=cg11444072	feature_id[116].value > threshold=0.4940422773361206
node_273: feature_name=cg08571020	feature_id[381].value > threshold=0.4616774618625641
node_275: feature_name=cg08331427	feature_id[2081].value > threshold=0.5443233847618103
node_281: feature_name=cg00216961	feature_id[945].value <= threshold=0.4166988283395767
node_282: feature_name=cg04716580	feature_id[1390].value > threshold=0.4204266667366028
node_286: feature_name=cg07891483	feature_id[1877].value > threshold=0.829958975315094
node_288: feature_name=cg01475325	feature_id[3969].value <= threshold=0.45323844254016876
node_289: feature_name=cg21925748	feature_id[5244].value <= threshold=0.7208462655544281
Class: sarcoma (SARC)	
Rules_46	passed counts:7
node_0: feature_name=cg11915444	feature_id[2002].value > threshold=0.3402601182460785
node_10: feature_name=cg26016985	feature_id[4407].value > threshold=0.6493876278400421
node_16: feature_name=cg23157618	feature_id[5511].value > threshold=0.5759606957435608
node_20: feature_name=cg10480329	feature_id[1439].value > threshold=0.5518321692943573
node_48: feature_name=cg17843418	feature_id[4868].value > threshold=0.34969255328178406
node_52: feature_name=cg24407065	feature_id[4522].value > threshold=0.5364363789558411
node_56: feature_name=cg07281938	feature_id[249].value > threshold=0.7506992518901825
node_78: feature_name=cg21759907	feature_id[1650].value > threshold=0.46823520958423615
node_98: feature_name=cg10886334	feature_id[3975].value > threshold=0.43698127567768097
node_102: feature_name=cg09373983	feature_id[5025].value > threshold=0.5575815737247467

node_106: feature_name=cg12948116	feature_id[5360].value <= threshold=0.5658791363239288
node_107: feature_name=cg21906519	feature_id[5251].value > threshold=0.6089984774589539
node_111: feature_name=cg17537493	feature_id[4217].value > threshold=0.5825372636318207
node_117: feature_name=cg15720017	feature_id[4443].value > threshold=0.7009969055652618
node_119: feature_name=cg06038180	feature_id[3480].value > threshold=0.4583848565816879
node_121: feature_name=cg08619651	feature_id[4882].value <= threshold=0.2105495035648346
node_122: feature_name=cg01014262	feature_id[1911].value <= threshold=0.5327437818050385
node_123: feature_name=cg16863382	feature_id[2922].value > threshold=0.7200668156147003
node_133: feature_name=cg06989443	feature_id[2327].value <= threshold=0.6579365730285645
node_134: feature_name=cg07344990	feature_id[2398].value > threshold=0.5562876164913177
node_138: feature_name=cg02966841	feature_id[2199].value > threshold=0.7744501233100891
node_204: feature_name=cg12120430	feature_id[3727].value > threshold=0.5775820016860962
node_206: feature_name=cg18386876	feature_id[4066].value > threshold=0.6847257018089294
node_208: feature_name=cg02053092	feature_id[3610].value > threshold=0.39507485926151276
node_210: feature_name=cg10298992	feature_id[3603].value > threshold=0.5707973837852478
node_228: feature_name=cg03840920	feature_id[4524].value > threshold=0.5365143716335297
node_234: feature_name=cg25364972	feature_id[1792].value > threshold=0.2418053299188614
node_238: feature_name=cg23485307	feature_id[221].value > threshold=0.14884746074676514
node_242: feature_name=cg04873169	feature_id[4696].value > threshold=0.6058756709098816
node_244: feature_name=cg17009731	feature_id[3218].value > threshold=0.6602252125740051
node_246: feature_name=cg18253910	feature_id[4458].value > threshold=0.6904322504997253
node_250: feature_name=cg04774476	feature_id[1257].value > threshold=0.5675267577171326

node_256: feature_name=cg19259111	feature_id[63].value > threshold=0.40771469473838806
node_264: feature_name=cg21483216	feature_id[5253].value > threshold=0.6332719326019287
node_266: feature_name=cg04026354	feature_id[3502].value <= threshold=0.42332448065280914
node_267: feature_name=cg11444072	feature_id[116].value > threshold=0.4940422773361206
node_273: feature_name=cg08571020	feature_id[381].value > threshold=0.4616774618625641
node_275: feature_name=cg08331427	feature_id[2081].value > threshold=0.5443233847618103
node_281: feature_name=cg00216961	feature_id[945].value <= threshold=0.4166988283395767
node_282: feature_name=cg04716580	feature_id[1390].value > threshold=0.4204266667366028
node_286: feature_name=cg07891483	feature_id[1877].value <= threshold=0.829958975315094
Class: plexiform neurofibroma (NFB (PLEX))	
Rules_47	passed counts:7
node_0: feature_name=cg11915444	feature_id[2002].value > threshold=0.3402601182460785
node_10: feature_name=cg26016985	feature_id[4407].value > threshold=0.6493876278400421
node_16: feature_name=cg23157618	feature_id[5511].value > threshold=0.5759606957435608
node_20: feature_name=cg10480329	feature_id[1439].value > threshold=0.5518321692943573
node_48: feature_name=cg17843418	feature_id[4868].value > threshold=0.34969255328178406
node_52: feature_name=cg24407065	feature_id[4522].value > threshold=0.5364363789558411
node_56: feature_name=cg07281938	feature_id[249].value > threshold=0.7506992518901825
node_78: feature_name=cg21759907	feature_id[1650].value > threshold=0.46823520958423615
node_98: feature_name=cg10886334	feature_id[3975].value > threshold=0.43698127567768097
node_102: feature_name=cg09373983	feature_id[5025].value > threshold=0.5575815737247467
node_106: feature_name=cg12948116	feature_id[5360].value <= threshold=0.5658791363239288

node_107: feature_name=cg21906519	feature_id[5251].value > threshold=0.6089984774589539
node_111: feature_name=cg17537493	feature_id[4217].value > threshold=0.5825372636318207
node_117: feature_name=cg15720017	feature_id[4443].value > threshold=0.7009969055652618
node_119: feature_name=cg06038180	feature_id[3480].value > threshold=0.4583848565816879
node_121: feature_name=cg08619651	feature_id[4882].value <= threshold=0.2105495035648346
node_122: feature_name=cg01014262	feature_id[1911].value <= threshold=0.5327437818050385
node_123: feature_name=cg16863382	feature_id[2922].value > threshold=0.7200668156147003
node_133: feature_name=cg06989443	feature_id[2327].value <= threshold=0.6579365730285645
node_134: feature_name=cg07344990	feature_id[2398].value > threshold=0.5562876164913177
node_138: feature_name=cg02966841	feature_id[2199].value > threshold=0.7744501233100891
node_204: feature_name=cg12120430	feature_id[3727].value > threshold=0.5775820016860962
node_206: feature_name=cg18386876	feature_id[4066].value > threshold=0.6847257018089294
node_208: feature_name=cg02053092	feature_id[3610].value > threshold=0.39507485926151276
node_210: feature_name=cg10298992	feature_id[3603].value > threshold=0.5707973837852478
node_228: feature_name=cg03840920	feature_id[4524].value > threshold=0.5365143716335297
node_234: feature_name=cg25364972	feature_id[1792].value > threshold=0.2418053299188614
node_238: feature_name=cg23485307	feature_id[221].value > threshold=0.14884746074676514
node_242: feature_name=cg04873169	feature_id[4696].value > threshold=0.6058756709098816
node_244: feature_name=cg17009731	feature_id[3218].value > threshold=0.6602252125740051
node_246: feature_name=cg18253910	feature_id[4458].value > threshold=0.6904322504997253
node_250: feature_name=cg04774476	feature_id[1257].value > threshold=0.5675267577171326
node_256: feature_name=cg19259111	feature_id[63].value > threshold=0.40771469473838806

node_264: feature_name=cg21483216	feature_id[5253].value > threshold=0.6332719326019287
node_266: feature_name=cg04026354	feature_id[3502].value <= threshold=0.42332448065280914
node_267: feature_name=cg11444072	feature_id[116].value > threshold=0.4940422773361206
node_273: feature_name=cg08571020	feature_id[381].value > threshold=0.4616774618625641
node_275: feature_name=cg08331427	feature_id[2081].value > threshold=0.5443233847618103
node_281: feature_name=cg00216961	feature_id[945].value <= threshold=0.4166988283395767
node_282: feature_name=cg04716580	feature_id[1390].value <= threshold=0.4204266667366028
node_283: feature_name=cg09180239	feature_id[2620].value > threshold=0.7707420885562897
Class: sclerosing epithelioid fibrosarcoma(SEF)	
Rules_48	passed counts:7
node_0: feature_name=cg11915444	feature_id[2002].value > threshold=0.3402601182460785
node_10: feature_name=cg26016985	feature_id[4407].value > threshold=0.6493876278400421
node_16: feature_name=cg23157618	feature_id[5511].value > threshold=0.5759606957435608
node_20: feature_name=cg10480329	feature_id[1439].value > threshold=0.5518321692943573
node_48: feature_name=cg17843418	feature_id[4868].value > threshold=0.34969255328178406
node_52: feature_name=cg24407065	feature_id[4522].value > threshold=0.5364363789558411
node_56: feature_name=cg07281938	feature_id[249].value > threshold=0.7506992518901825
node_78: feature_name=cg21759907	feature_id[1650].value > threshold=0.46823520958423615
node_98: feature_name=cg10886334	feature_id[3975].value > threshold=0.43698127567768097
node_102: feature_name=cg09373983	feature_id[5025].value > threshold=0.5575815737247467
node_106: feature_name=cg12948116	feature_id[5360].value <= threshold=0.5658791363239288
node_107: feature_name=cg21906519	feature_id[5251].value > threshold=0.6089984774589539

node_111: feature_name=cg17537493	feature_id[4217].value > threshold=0.5825372636318207
node_117: feature_name=cg15720017	feature_id[4443].value > threshold=0.7009969055652618
node_119: feature_name=cg06038180	feature_id[3480].value > threshold=0.4583848565816879
node_121: feature_name=cg08619651	feature_id[4882].value <= threshold=0.2105495035648346
node_122: feature_name=cg01014262	feature_id[1911].value <= threshold=0.5327437818050385
node_123: feature_name=cg16863382	feature_id[2922].value > threshold=0.7200668156147003
node_133: feature_name=cg06989443	feature_id[2327].value <= threshold=0.6579365730285645
node_134: feature_name=cg07344990	feature_id[2398].value > threshold=0.5562876164913177
node_138: feature_name=cg02966841	feature_id[2199].value > threshold=0.7744501233100891
node_204: feature_name=cg12120430	feature_id[3727].value > threshold=0.5775820016860962
node_206: feature_name=cg18386876	feature_id[4066].value > threshold=0.6847257018089294
node_208: feature_name=cg02053092	feature_id[3610].value > threshold=0.39507485926151276
node_210: feature_name=cg10298992	feature_id[3603].value > threshold=0.5707973837852478
node_228: feature_name=cg03840920	feature_id[4524].value > threshold=0.5365143716335297
node_234: feature_name=cg25364972	feature_id[1792].value > threshold=0.2418053299188614
node_238: feature_name=cg23485307	feature_id[221].value > threshold=0.14884746074676514
node_242: feature_name=cg04873169	feature_id[4696].value > threshold=0.6058756709098816
node_244: feature_name=cg17009731	feature_id[3218].value > threshold=0.6602252125740051
node_246: feature_name=cg18253910	feature_id[4458].value > threshold=0.6904322504997253
node_250: feature_name=cg04774476	feature_id[1257].value > threshold=0.5675267577171326
node_256: feature_name=cg19259111	feature_id[63].value > threshold=0.40771469473838806
node_264: feature_name=cg21483216	feature_id[5253].value > threshold=0.6332719326019287

node_266: feature_name=cg04026354	feature_id[3502].value <= threshold=0.42332448065280914
node_267: feature_name=cg11444072	feature_id[116].value > threshold=0.4940422773361206
node_273: feature_name=cg08571020	feature_id[381].value <= threshold=0.4616774618625641
Class: clear cell sarcoma of soft parts (CCS)	
Rules_49	passed counts:7
node_0: feature_name=cg11915444	feature_id[2002].value > threshold=0.3402601182460785
node_10: feature_name=cg26016985	feature_id[4407].value > threshold=0.6493876278400421
node_16: feature_name=cg23157618	feature_id[5511].value > threshold=0.5759606957435608
node_20: feature_name=cg10480329	feature_id[1439].value > threshold=0.5518321692943573
node_48: feature_name=cg17843418	feature_id[4868].value > threshold=0.34969255328178406
node_52: feature_name=cg24407065	feature_id[4522].value > threshold=0.5364363789558411
node_56: feature_name=cg07281938	feature_id[249].value > threshold=0.7506992518901825
node_78: feature_name=cg21759907	feature_id[1650].value > threshold=0.46823520958423615
node_98: feature_name=cg10886334	feature_id[3975].value > threshold=0.43698127567768097
node_102: feature_name=cg09373983	feature_id[5025].value > threshold=0.5575815737247467
node_106: feature_name=cg12948116	feature_id[5360].value <= threshold=0.5658791363239288
node_107: feature_name=cg21906519	feature_id[5251].value > threshold=0.6089984774589539
node_111: feature_name=cg17537493	feature_id[4217].value > threshold=0.5825372636318207
node_117: feature_name=cg15720017	feature_id[4443].value > threshold=0.7009969055652618
node_119: feature_name=cg06038180	feature_id[3480].value > threshold=0.4583848565816879
node_121: feature_name=cg08619651	feature_id[4882].value <= threshold=0.2105495035648346
node_122: feature_name=cg01014262	feature_id[1911].value <= threshold=0.5327437818050385

node_123: feature_name=cg16863382	feature_id[2922].value > threshold=0.7200668156147003
node_133: feature_name=cg06989443	feature_id[2327].value <= threshold=0.6579365730285645
node_134: feature_name=cg07344990	feature_id[2398].value > threshold=0.5562876164913177
node_138: feature_name=cg02966841	feature_id[2199].value <= threshold=0.7744501233100891
node_139: feature_name=cg06989253	feature_id[194].value <= threshold=0.7899391353130341
node_140: feature_name=cg24617568	feature_id[3282].value <= threshold=0.26932457089424133
node_141: feature_name=cg12614090	feature_id[2765].value > threshold=0.1813211366534233
Class: melanoma (MEL)	
Rules_50	passed counts:7
node_0: feature_name=cg11915444	feature_id[2002].value > threshold=0.3402601182460785
node_10: feature_name=cg26016985	feature_id[4407].value > threshold=0.6493876278400421
node_16: feature_name=cg23157618	feature_id[5511].value > threshold=0.5759606957435608
node_20: feature_name=cg10480329	feature_id[1439].value > threshold=0.5518321692943573
node_48: feature_name=cg17843418	feature_id[4868].value > threshold=0.34969255328178406
node_52: feature_name=cg24407065	feature_id[4522].value > threshold=0.5364363789558411
node_56: feature_name=cg07281938	feature_id[249].value > threshold=0.7506992518901825
node_78: feature_name=cg21759907	feature_id[1650].value > threshold=0.46823520958423615
node_98: feature_name=cg10886334	feature_id[3975].value > threshold=0.43698127567768097
node_102: feature_name=cg09373983	feature_id[5025].value > threshold=0.5575815737247467
node_106: feature_name=cg12948116	feature_id[5360].value <= threshold=0.5658791363239288
node_107: feature_name=cg21906519	feature_id[5251].value > threshold=0.6089984774589539
node_111: feature_name=cg17537493	feature_id[4217].value > threshold=0.5825372636318207

node_117: feature_name=cg15720017	feature_id[4443].value > threshold=0.7009969055652618
node_119: feature_name=cg06038180	feature_id[3480].value > threshold=0.4583848565816879
node_121: feature_name=cg08619651	feature_id[4882].value <= threshold=0.2105495035648346
node_122: feature_name=cg01014262	feature_id[1911].value <= threshold=0.5327437818050385
node_123: feature_name=cg16863382	feature_id[2922].value > threshold=0.7200668156147003
node_133: feature_name=cg06989443	feature_id[2327].value <= threshold=0.6579365730285645
node_134: feature_name=cg07344990	feature_id[2398].value > threshold=0.5562876164913177
node_138: feature_name=cg02966841	feature_id[2199].value <= threshold=0.7744501233100891
node_139: feature_name=cg06989253	feature_id[194].value <= threshold=0.7899391353130341
node_140: feature_name=cg24617568	feature_id[3282].value <= threshold=0.26932457089424133
node_141: feature_name=cg12614090	feature_id[2765].value <= threshold=0.1813211366534233
node_142: feature_name=cg10917602	feature_id[4685].value > threshold=0.5744736790657043
Class: high-grade conventional osteosarcoma (OS (HG))	
Rules_51	passed counts:7
node_0: feature_name=cg11915444	feature_id[2002].value > threshold=0.3402601182460785
node_10: feature_name=cg26016985	feature_id[4407].value > threshold=0.6493876278400421
node_16: feature_name=cg23157618	feature_id[5511].value > threshold=0.5759606957435608
node_20: feature_name=cg10480329	feature_id[1439].value > threshold=0.5518321692943573
node_48: feature_name=cg17843418	feature_id[4868].value > threshold=0.34969255328178406
node_52: feature_name=cg24407065	feature_id[4522].value > threshold=0.5364363789558411
node_56: feature_name=cg07281938	feature_id[249].value > threshold=0.7506992518901825

node_78: feature_name=cg21759907	feature_id[1650].value <= threshold=0.46823520958423615
node_79: feature_name=cg25476129	feature_id[2302].value <= threshold=0.1687404215335846
node_80: feature_name=cg26362368	feature_id[414].value > threshold=0.834072083234787
Class: leiomyoma (LMO)	
Rules_52	passed counts:6
node_0: feature_name=cg11915444	feature_id[2002].value > threshold=0.3402601182460785
node_10: feature_name=cg26016985	feature_id[4407].value > threshold=0.6493876278400421
node_16: feature_name=cg23157618	feature_id[5511].value > threshold=0.5759606957435608
node_20: feature_name=cg10480329	feature_id[1439].value > threshold=0.5518321692943573
node_48: feature_name=cg17843418	feature_id[4868].value > threshold=0.34969255328178406
node_52: feature_name=cg24407065	feature_id[4522].value > threshold=0.5364363789558411
node_56: feature_name=cg07281938	feature_id[249].value > threshold=0.7506992518901825
node_78: feature_name=cg21759907	feature_id[1650].value > threshold=0.46823520958423615
node_98: feature_name=cg10886334	feature_id[3975].value > threshold=0.43698127567768097
node_102: feature_name=cg09373983	feature_id[5025].value > threshold=0.5575815737247467
node_106: feature_name=cg12948116	feature_id[5360].value <= threshold=0.5658791363239288
node_107: feature_name=cg21906519	feature_id[5251].value > threshold=0.6089984774589539
node_111: feature_name=cg17537493	feature_id[4217].value > threshold=0.5825372636318207
node_117: feature_name=cg15720017	feature_id[4443].value > threshold=0.7009969055652618
node_119: feature_name=cg06038180	feature_id[3480].value > threshold=0.4583848565816879
node_121: feature_name=cg08619651	feature_id[4882].value <= threshold=0.2105495035648346
node_122: feature_name=cg01014262	feature_id[1911].value <= threshold=0.5327437818050385

node_123: feature_name=cg16863382	feature_id[2922].value > threshold=0.7200668156147003
node_133: feature_name=cg06989443	feature_id[2327].value <= threshold=0.6579365730285645
node_134: feature_name=cg07344990	feature_id[2398].value > threshold=0.5562876164913177
node_138: feature_name=cg02966841	feature_id[2199].value > threshold=0.7744501233100891
node_204: feature_name=cg12120430	feature_id[3727].value > threshold=0.5775820016860962
node_206: feature_name=cg18386876	feature_id[4066].value > threshold=0.6847257018089294
node_208: feature_name=cg02053092	feature_id[3610].value > threshold=0.39507485926151276
node_210: feature_name=cg10298992	feature_id[3603].value > threshold=0.5707973837852478
node_228: feature_name=cg03840920	feature_id[4524].value > threshold=0.5365143716335297
node_234: feature_name=cg25364972	feature_id[1792].value > threshold=0.2418053299188614
node_238: feature_name=cg23485307	feature_id[221].value > threshold=0.14884746074676514
node_242: feature_name=cg04873169	feature_id[4696].value > threshold=0.6058756709098816
node_244: feature_name=cg17009731	feature_id[3218].value > threshold=0.6602252125740051
node_246: feature_name=cg18253910	feature_id[4458].value > threshold=0.6904322504997253
node_250: feature_name=cg04774476	feature_id[1257].value > threshold=0.5675267577171326
node_256: feature_name=cg19259111	feature_id[63].value > threshold=0.40771469473838806
node_264: feature_name=cg21483216	feature_id[5253].value > threshold=0.6332719326019287
node_266: feature_name=cg04026354	feature_id[3502].value <= threshold=0.42332448065280914
node_267: feature_name=cg11444072	feature_id[116].value > threshold=0.4940422773361206
node_273: feature_name=cg08571020	feature_id[381].value > threshold=0.4616774618625641
node_275: feature_name=cg08331427	feature_id[2081].value > threshold=0.5443233847618103
node_281: feature_name=cg00216961	feature_id[945].value <= threshold=0.4166988283395767

node_282: feature_name=cg04716580	feature_id[1390].value > threshold=0.4204266667366028
node_286: feature_name=cg07891483	feature_id[1877].value > threshold=0.829958975315094
node_288: feature_name=cg01475325	feature_id[3969].value > threshold=0.45323844254016876
node_292: feature_name=cg19702397	feature_id[2066].value > threshold=0.3536848723888397
node_294: feature_name=cg21936959	feature_id[595].value > threshold=0.5567200481891632
node_308: feature_name=cg18645642	feature_id[262].value > threshold=0.42608243227005005
node_310: feature_name=cg27351239	feature_id[1874].value > threshold=0.6790103316307068
node_330: feature_name=cg18252102	feature_id[1205].value <= threshold=0.5534502267837524
Class: undifferentiated sarcoma (USARC)	
Rules_53	passed counts:6
node_0: feature_name=cg11915444	feature_id[2002].value > threshold=0.3402601182460785
node_10: feature_name=cg26016985	feature_id[4407].value > threshold=0.6493876278400421
node_16: feature_name=cg23157618	feature_id[5511].value > threshold=0.5759606957435608
node_20: feature_name=cg10480329	feature_id[1439].value > threshold=0.5518321692943573
node_48: feature_name=cg17843418	feature_id[4868].value > threshold=0.34969255328178406
node_52: feature_name=cg24407065	feature_id[4522].value > threshold=0.5364363789558411
node_56: feature_name=cg07281938	feature_id[249].value <= threshold=0.7506992518901825
node_57: feature_name=cg21189849	feature_id[1261].value > threshold=0.7545044124126434
node_63: feature_name=cg18048309	feature_id[2794].value <= threshold=0.36701037734746933
Class: mesenchymal chondrosarcoma (CSA (MES))	
Rules_54	passed counts:6
node_0: feature_name=cg11915444	feature_id[2002].value > threshold=0.3402601182460785

node_10: feature_name=cg26016985	feature_id[4407].value > threshold=0.6493876278400421
node_16: feature_name=cg23157618	feature_id[5511].value > threshold=0.5759606957435608
node_20: feature_name=cg10480329	feature_id[1439].value <= threshold=0.5518321692943573
node_21: feature_name=cg25510609	feature_id[149].value <= threshold=0.08151235431432724
node_22: feature_name=cg23850277	feature_id[2159].value <= threshold=0.5682950615882874
node_23: feature_name=cg01259126	feature_id[3146].value <= threshold=0.34355829656124115
Class: osteoblastoma (OB)	
Rules_55	passed counts:5
node_0: feature_name=cg11915444	feature_id[2002].value > threshold=0.3402601182460785
node_10: feature_name=cg26016985	feature_id[4407].value > threshold=0.6493876278400421
node_16: feature_name=cg23157618	feature_id[5511].value > threshold=0.5759606957435608
node_20: feature_name=cg10480329	feature_id[1439].value > threshold=0.5518321692943573
node_48: feature_name=cg17843418	feature_id[4868].value > threshold=0.34969255328178406
node_52: feature_name=cg24407065	feature_id[4522].value > threshold=0.5364363789558411
node_56: feature_name=cg07281938	feature_id[249].value > threshold=0.7506992518901825
node_78: feature_name=cg21759907	feature_id[1650].value > threshold=0.46823520958423615
node_98: feature_name=cg10886334	feature_id[3975].value > threshold=0.43698127567768097
node_102: feature_name=cg09373983	feature_id[5025].value > threshold=0.5575815737247467
node_106: feature_name=cg12948116	feature_id[5360].value <= threshold=0.5658791363239288
node_107: feature_name=cg21906519	feature_id[5251].value > threshold=0.6089984774589539
node_111: feature_name=cg17537493	feature_id[4217].value > threshold=0.5825372636318207
node_117: feature_name=cg15720017	feature_id[4443].value > threshold=0.7009969055652618

node_119: feature_name=cg06038180	feature_id[3480].value > threshold=0.4583848565816879
node_121: feature_name=cg08619651	feature_id[4882].value <= threshold=0.2105495035648346
node_122: feature_name=cg01014262	feature_id[1911].value <= threshold=0.5327437818050385
node_123: feature_name=cg16863382	feature_id[2922].value > threshold=0.7200668156147003
node_133: feature_name=cg06989443	feature_id[2327].value <= threshold=0.6579365730285645
node_134: feature_name=cg07344990	feature_id[2398].value > threshold=0.5562876164913177
node_138: feature_name=cg02966841	feature_id[2199].value > threshold=0.7744501233100891
node_204: feature_name=cg12120430	feature_id[3727].value > threshold=0.5775820016860962
node_206: feature_name=cg18386876	feature_id[4066].value > threshold=0.6847257018089294
node_208: feature_name=cg02053092	feature_id[3610].value > threshold=0.39507485926151276
node_210: feature_name=cg10298992	feature_id[3603].value > threshold=0.5707973837852478
node_228: feature_name=cg03840920	feature_id[4524].value > threshold=0.5365143716335297
node_234: feature_name=cg25364972	feature_id[1792].value > threshold=0.2418053299188614
node_238: feature_name=cg23485307	feature_id[221].value > threshold=0.14884746074676514
node_242: feature_name=cg04873169	feature_id[4696].value > threshold=0.6058756709098816
node_244: feature_name=cg17009731	feature_id[3218].value > threshold=0.6602252125740051
node_246: feature_name=cg18253910	feature_id[4458].value > threshold=0.6904322504997253
node_250: feature_name=cg04774476	feature_id[1257].value > threshold=0.5675267577171326
node_256: feature_name=cg19259111	feature_id[63].value > threshold=0.40771469473838806
node_264: feature_name=cg21483216	feature_id[5253].value > threshold=0.6332719326019287
node_266: feature_name=cg04026354	feature_id[3502].value > threshold=0.42332448065280914
node_336: feature_name=cg05824594	feature_id[1431].value <= threshold=0.629401296377182

node_337: feature_name=cg18475969	feature_id[2117].value <= threshold=0.3116971254348755
node_338: feature_name=cg19494591	feature_id[2196].value <= threshold=0.7268106639385223
Class: Langerhans cell histiocytosis (LCH)	
Rules_56	passed counts:5
node_0: feature_name=cg11915444	feature_id[2002].value > threshold=0.3402601182460785
node_10: feature_name=cg26016985	feature_id[4407].value > threshold=0.6493876278400421
node_16: feature_name=cg23157618	feature_id[5511].value > threshold=0.5759606957435608
node_20: feature_name=cg10480329	feature_id[1439].value > threshold=0.5518321692943573
node_48: feature_name=cg17843418	feature_id[4868].value > threshold=0.34969255328178406
node_52: feature_name=cg24407065	feature_id[4522].value > threshold=0.5364363789558411
node_56: feature_name=cg07281938	feature_id[249].value > threshold=0.7506992518901825
node_78: feature_name=cg21759907	feature_id[1650].value > threshold=0.46823520958423615
node_98: feature_name=cg10886334	feature_id[3975].value > threshold=0.43698127567768097
node_102: feature_name=cg09373983	feature_id[5025].value > threshold=0.5575815737247467
node_106: feature_name=cg12948116	feature_id[5360].value <= threshold=0.5658791363239288
node_107: feature_name=cg21906519	feature_id[5251].value > threshold=0.6089984774589539
node_111: feature_name=cg17537493	feature_id[4217].value > threshold=0.5825372636318207
node_117: feature_name=cg15720017	feature_id[4443].value > threshold=0.7009969055652618
node_119: feature_name=cg06038180	feature_id[3480].value > threshold=0.4583848565816879
node_121: feature_name=cg08619651	feature_id[4882].value <= threshold=0.2105495035648346
node_122: feature_name=cg01014262	feature_id[1911].value <= threshold=0.5327437818050385
node_123: feature_name=cg16863382	feature_id[2922].value > threshold=0.7200668156147003

node_133: feature_name=cg06989443	feature_id[2327].value <= threshold=0.6579365730285645
node_134: feature_name=cg07344990	feature_id[2398].value > threshold=0.5562876164913177
node_138: feature_name=cg02966841	feature_id[2199].value > threshold=0.7744501233100891
node_204: feature_name=cg12120430	feature_id[3727].value > threshold=0.5775820016860962
node_206: feature_name=cg18386876	feature_id[4066].value > threshold=0.6847257018089294
node_208: feature_name=cg02053092	feature_id[3610].value > threshold=0.39507485926151276
node_210: feature_name=cg10298992	feature_id[3603].value > threshold=0.5707973837852478
node_228: feature_name=cg03840920	feature_id[4524].value > threshold=0.5365143716335297
node_234: feature_name=cg25364972	feature_id[1792].value > threshold=0.2418053299188614
node_238: feature_name=cg23485307	feature_id[221].value > threshold=0.14884746074676514
node_242: feature_name=cg04873169	feature_id[4696].value > threshold=0.6058756709098816
node_244: feature_name=cg17009731	feature_id[3218].value > threshold=0.6602252125740051
node_246: feature_name=cg18253910	feature_id[4458].value > threshold=0.6904322504997253
node_250: feature_name=cg04774476	feature_id[1257].value > threshold=0.5675267577171326
node_256: feature_name=cg19259111	feature_id[63].value > threshold=0.40771469473838806
node_264: feature_name=cg21483216	feature_id[5253].value > threshold=0.6332719326019287
node_266: feature_name=cg04026354	feature_id[3502].value <= threshold=0.42332448065280914
node_267: feature_name=cg11444072	feature_id[116].value > threshold=0.4940422773361206
node_273: feature_name=cg08571020	feature_id[381].value > threshold=0.4616774618625641
node_275: feature_name=cg08331427	feature_id[2081].value > threshold=0.5443233847618103
node_281: feature_name=cg00216961	feature_id[945].value <= threshold=0.4166988283395767
node_282: feature_name=cg04716580	feature_id[1390].value > threshold=0.4204266667366028

node_286: feature_name=cg07891483	feature_id[1877].value > threshold=0.829958975315094
node_288: feature_name=cg01475325	feature_id[3969].value > threshold=0.45323844254016876
node_292: feature_name=cg19702397	feature_id[2066].value > threshold=0.3536848723888397
node_294: feature_name=cg21936959	feature_id[595].value > threshold=0.5567200481891632
node_308: feature_name=cg18645642	feature_id[262].value <= threshold=0.42608243227005005
Class: myositis proliferans (MP)	
Rules_57	passed counts:5
node_0: feature_name=cg11915444	feature_id[2002].value > threshold=0.3402601182460785
node_10: feature_name=cg26016985	feature_id[4407].value > threshold=0.6493876278400421
node_16: feature_name=cg23157618	feature_id[5511].value > threshold=0.5759606957435608
node_20: feature_name=cg10480329	feature_id[1439].value > threshold=0.5518321692943573
node_48: feature_name=cg17843418	feature_id[4868].value > threshold=0.34969255328178406
node_52: feature_name=cg24407065	feature_id[4522].value > threshold=0.5364363789558411
node_56: feature_name=cg07281938	feature_id[249].value > threshold=0.7506992518901825
node_78: feature_name=cg21759907	feature_id[1650].value > threshold=0.46823520958423615
node_98: feature_name=cg10886334	feature_id[3975].value > threshold=0.43698127567768097
node_102: feature_name=cg09373983	feature_id[5025].value > threshold=0.5575815737247467
node_106: feature_name=cg12948116	feature_id[5360].value <= threshold=0.5658791363239288
node_107: feature_name=cg21906519	feature_id[5251].value > threshold=0.6089984774589539
node_111: feature_name=cg17537493	feature_id[4217].value > threshold=0.5825372636318207
node_117: feature_name=cg15720017	feature_id[4443].value > threshold=0.7009969055652618
node_119: feature_name=cg06038180	feature_id[3480].value > threshold=0.4583848565816879

node_121: feature_name=cg08619651	feature_id[4882].value <= threshold=0.2105495035648346
node_122: feature_name=cg01014262	feature_id[1911].value <= threshold=0.5327437818050385
node_123: feature_name=cg16863382	feature_id[2922].value > threshold=0.7200668156147003
node_133: feature_name=cg06989443	feature_id[2327].value <= threshold=0.6579365730285645
node_134: feature_name=cg07344990	feature_id[2398].value > threshold=0.5562876164913177
node_138: feature_name=cg02966841	feature_id[2199].value > threshold=0.7744501233100891
node_204: feature_name=cg12120430	feature_id[3727].value > threshold=0.5775820016860962
node_206: feature_name=cg18386876	feature_id[4066].value > threshold=0.6847257018089294
node_208: feature_name=cg02053092	feature_id[3610].value > threshold=0.39507485926151276
node_210: feature_name=cg10298992	feature_id[3603].value > threshold=0.5707973837852478
node_228: feature_name=cg03840920	feature_id[4524].value > threshold=0.5365143716335297
node_234: feature_name=cg25364972	feature_id[1792].value > threshold=0.2418053299188614
node_238: feature_name=cg23485307	feature_id[221].value > threshold=0.14884746074676514
node_242: feature_name=cg04873169	feature_id[4696].value > threshold=0.6058756709098816
node_244: feature_name=cg17009731	feature_id[3218].value > threshold=0.6602252125740051
node_246: feature_name=cg18253910	feature_id[4458].value > threshold=0.6904322504997253
node_250: feature_name=cg04774476	feature_id[1257].value > threshold=0.5675267577171326
node_256: feature_name=cg19259111	feature_id[63].value > threshold=0.40771469473838806
node_264: feature_name=cg21483216	feature_id[5253].value > threshold=0.6332719326019287
node_266: feature_name=cg04026354	feature_id[3502].value <= threshold=0.42332448065280914
node_267: feature_name=cg11444072	feature_id[116].value > threshold=0.4940422773361206
node_273: feature_name=cg08571020	feature_id[381].value > threshold=0.4616774618625641

node_275: feature_name=cg08331427	feature_id[2081].value > threshold=0.5443233847618103
node_281: feature_name=cg00216961	feature_id[945].value <= threshold=0.4166988283395767
node_282: feature_name=cg04716580	feature_id[1390].value > threshold=0.4204266667366028
node_286: feature_name=cg07891483	feature_id[1877].value > threshold=0.829958975315094
node_288: feature_name=cg01475325	feature_id[3969].value > threshold=0.45323844254016876
node_292: feature_name=cg19702397	feature_id[2066].value <= threshold=0.3536848723888397
Class: low-grade fibromyxoid sarcoma (LGFMS)	
Rules_58	passed counts:5
node_0: feature_name=cg11915444	feature_id[2002].value > threshold=0.3402601182460785
node_10: feature_name=cg26016985	feature_id[4407].value > threshold=0.6493876278400421
node_16: feature_name=cg23157618	feature_id[5511].value > threshold=0.5759606957435608
node_20: feature_name=cg10480329	feature_id[1439].value > threshold=0.5518321692943573
node_48: feature_name=cg17843418	feature_id[4868].value > threshold=0.34969255328178406
node_52: feature_name=cg24407065	feature_id[4522].value > threshold=0.5364363789558411
node_56: feature_name=cg07281938	feature_id[249].value > threshold=0.7506992518901825
node_78: feature_name=cg21759907	feature_id[1650].value > threshold=0.46823520958423615
node_98: feature_name=cg10886334	feature_id[3975].value > threshold=0.43698127567768097
node_102: feature_name=cg09373983	feature_id[5025].value > threshold=0.5575815737247467
node_106: feature_name=cg12948116	feature_id[5360].value <= threshold=0.5658791363239288
node_107: feature_name=cg21906519	feature_id[5251].value > threshold=0.6089984774589539
node_111: feature_name=cg17537493	feature_id[4217].value > threshold=0.5825372636318207
node_117: feature_name=cg15720017	feature_id[4443].value > threshold=0.7009969055652618

node_119: feature_name=cg06038180	feature_id[3480].value > threshold=0.4583848565816879
node_121: feature_name=cg08619651	feature_id[4882].value <= threshold=0.2105495035648346
node_122: feature_name=cg01014262	feature_id[1911].value <= threshold=0.5327437818050385
node_123: feature_name=cg16863382	feature_id[2922].value > threshold=0.7200668156147003
node_133: feature_name=cg06989443	feature_id[2327].value <= threshold=0.6579365730285645
node_134: feature_name=cg07344990	feature_id[2398].value > threshold=0.5562876164913177
node_138: feature_name=cg02966841	feature_id[2199].value > threshold=0.7744501233100891
node_204: feature_name=cg12120430	feature_id[3727].value > threshold=0.5775820016860962
node_206: feature_name=cg18386876	feature_id[4066].value > threshold=0.6847257018089294
node_208: feature_name=cg02053092	feature_id[3610].value > threshold=0.39507485926151276
node_210: feature_name=cg10298992	feature_id[3603].value > threshold=0.5707973837852478
node_228: feature_name=cg03840920	feature_id[4524].value > threshold=0.5365143716335297
node_234: feature_name=cg25364972	feature_id[1792].value > threshold=0.2418053299188614
node_238: feature_name=cg23485307	feature_id[221].value > threshold=0.14884746074676514
node_242: feature_name=cg04873169	feature_id[4696].value > threshold=0.6058756709098816
node_244: feature_name=cg17009731	feature_id[3218].value > threshold=0.6602252125740051
node_246: feature_name=cg18253910	feature_id[4458].value > threshold=0.6904322504997253
node_250: feature_name=cg04774476	feature_id[1257].value > threshold=0.5675267577171326
node_256: feature_name=cg19259111	feature_id[63].value > threshold=0.40771469473838806
node_264: feature_name=cg21483216	feature_id[5253].value > threshold=0.6332719326019287
node_266: feature_name=cg04026354	feature_id[3502].value <= threshold=0.42332448065280914
node_267: feature_name=cg11444072	feature_id[116].value <= threshold=0.4940422773361206

node_268: feature_name=cg25424742	feature_id[2698].value > threshold=0.762436032295227
node_270: feature_name=cg23632333	feature_id[4343].value > threshold=0.88172447681427
Class: chondroblastoma (CB)	
Rules_59	passed counts:5
node_0: feature_name=cg11915444	feature_id[2002].value > threshold=0.3402601182460785
node_10: feature_name=cg26016985	feature_id[4407].value > threshold=0.6493876278400421
node_16: feature_name=cg23157618	feature_id[5511].value > threshold=0.5759606957435608
node_20: feature_name=cg10480329	feature_id[1439].value <= threshold=0.5518321692943573
node_21: feature_name=cg25510609	feature_id[149].value <= threshold=0.08151235431432724
node_22: feature_name=cg23850277	feature_id[2159].value <= threshold=0.5682950615882874
node_23: feature_name=cg01259126	feature_id[3146].value > threshold=0.34355829656124115
node_25: feature_name=cg07034004	feature_id[3345].value > threshold=0.20594919472932816
Class: Langerhans cell histiocytosis (LCH)	
Rules_60	passed counts:4
node_0: feature_name=cg11915444	feature_id[2002].value > threshold=0.3402601182460785
node_10: feature_name=cg26016985	feature_id[4407].value > threshold=0.6493876278400421
node_16: feature_name=cg23157618	feature_id[5511].value > threshold=0.5759606957435608
node_20: feature_name=cg10480329	feature_id[1439].value > threshold=0.5518321692943573
node_48: feature_name=cg17843418	feature_id[4868].value > threshold=0.34969255328178406
node_52: feature_name=cg24407065	feature_id[4522].value > threshold=0.5364363789558411
node_56: feature_name=cg07281938	feature_id[249].value > threshold=0.7506992518901825
node_78: feature_name=cg21759907	feature_id[1650].value > threshold=0.46823520958423615

node_98: feature_name=cg10886334	feature_id[3975].value > threshold=0.43698127567768097
node_102: feature_name=cg09373983	feature_id[5025].value > threshold=0.5575815737247467
node_106: feature_name=cg12948116	feature_id[5360].value <= threshold=0.5658791363239288
node_107: feature_name=cg21906519	feature_id[5251].value > threshold=0.6089984774589539
node_111: feature_name=cg17537493	feature_id[4217].value > threshold=0.5825372636318207
node_117: feature_name=cg15720017	feature_id[4443].value > threshold=0.7009969055652618
node_119: feature_name=cg06038180	feature_id[3480].value > threshold=0.4583848565816879
node_121: feature_name=cg08619651	feature_id[4882].value <= threshold=0.2105495035648346
node_122: feature_name=cg01014262	feature_id[1911].value <= threshold=0.5327437818050385
node_123: feature_name=cg16863382	feature_id[2922].value > threshold=0.7200668156147003
node_133: feature_name=cg06989443	feature_id[2327].value <= threshold=0.6579365730285645
node_134: feature_name=cg07344990	feature_id[2398].value > threshold=0.5562876164913177
node_138: feature_name=cg02966841	feature_id[2199].value > threshold=0.7744501233100891
node_204: feature_name=cg12120430	feature_id[3727].value > threshold=0.5775820016860962
node_206: feature_name=cg18386876	feature_id[4066].value > threshold=0.6847257018089294
node_208: feature_name=cg02053092	feature_id[3610].value > threshold=0.39507485926151276
node_210: feature_name=cg10298992	feature_id[3603].value > threshold=0.5707973837852478
node_228: feature_name=cg03840920	feature_id[4524].value > threshold=0.5365143716335297
node_234: feature_name=cg25364972	feature_id[1792].value > threshold=0.2418053299188614
node_238: feature_name=cg23485307	feature_id[221].value > threshold=0.14884746074676514
node_242: feature_name=cg04873169	feature_id[4696].value > threshold=0.6058756709098816
node_244: feature_name=cg17009731	feature_id[3218].value > threshold=0.6602252125740051

node_246: feature_name=cg18253910	feature_id[4458].value > threshold=0.6904322504997253
node_250: feature_name=cg04774476	feature_id[1257].value > threshold=0.5675267577171326
node_256: feature_name=cg19259111	feature_id[63].value > threshold=0.40771469473838806
node_264: feature_name=cg21483216	feature_id[5253].value > threshold=0.6332719326019287
node_266: feature_name=cg04026354	feature_id[3502].value > threshold=0.42332448065280914
node_336: feature_name=cg05824594	feature_id[1431].value <= threshold=0.629401296377182
node_337: feature_name=cg18475969	feature_id[2117].value <= threshold=0.3116971254348755
node_338: feature_name=cg19494591	feature_id[2196].value > threshold=0.7268106639385223
node_340: feature_name=cg09075515	feature_id[1529].value <= threshold=0.5144184827804565
Class: chordoma (CHORD)	
Rules_61	passed counts:4
node_0: feature_name=cg11915444	feature_id[2002].value > threshold=0.3402601182460785
node_10: feature_name=cg26016985	feature_id[4407].value > threshold=0.6493876278400421
node_16: feature_name=cg23157618	feature_id[5511].value > threshold=0.5759606957435608
node_20: feature_name=cg10480329	feature_id[1439].value > threshold=0.5518321692943573
node_48: feature_name=cg17843418	feature_id[4868].value > threshold=0.34969255328178406
node_52: feature_name=cg24407065	feature_id[4522].value > threshold=0.5364363789558411
node_56: feature_name=cg07281938	feature_id[249].value > threshold=0.7506992518901825
node_78: feature_name=cg21759907	feature_id[1650].value > threshold=0.46823520958423615
node_98: feature_name=cg10886334	feature_id[3975].value > threshold=0.43698127567768097
node_102: feature_name=cg09373983	feature_id[5025].value > threshold=0.5575815737247467
node_106: feature_name=cg12948116	feature_id[5360].value <= threshold=0.5658791363239288

node_107: feature_name=cg21906519	feature_id[5251].value > threshold=0.6089984774589539
node_111: feature_name=cg17537493	feature_id[4217].value > threshold=0.5825372636318207
node_117: feature_name=cg15720017	feature_id[4443].value > threshold=0.7009969055652618
node_119: feature_name=cg06038180	feature_id[3480].value > threshold=0.4583848565816879
node_121: feature_name=cg08619651	feature_id[4882].value <= threshold=0.2105495035648346
node_122: feature_name=cg01014262	feature_id[1911].value <= threshold=0.5327437818050385
node_123: feature_name=cg16863382	feature_id[2922].value > threshold=0.7200668156147003
node_133: feature_name=cg06989443	feature_id[2327].value <= threshold=0.6579365730285645
node_134: feature_name=cg07344990	feature_id[2398].value > threshold=0.5562876164913177
node_138: feature_name=cg02966841	feature_id[2199].value > threshold=0.7744501233100891
node_204: feature_name=cg12120430	feature_id[3727].value > threshold=0.5775820016860962
node_206: feature_name=cg18386876	feature_id[4066].value > threshold=0.6847257018089294
node_208: feature_name=cg02053092	feature_id[3610].value > threshold=0.39507485926151276
node_210: feature_name=cg10298992	feature_id[3603].value > threshold=0.5707973837852478
node_228: feature_name=cg03840920	feature_id[4524].value > threshold=0.5365143716335297
node_234: feature_name=cg25364972	feature_id[1792].value > threshold=0.2418053299188614
node_238: feature_name=cg23485307	feature_id[221].value > threshold=0.14884746074676514
node_242: feature_name=cg04873169	feature_id[4696].value > threshold=0.6058756709098816
node_244: feature_name=cg17009731	feature_id[3218].value > threshold=0.6602252125740051
node_246: feature_name=cg18253910	feature_id[4458].value > threshold=0.6904322504997253
node_250: feature_name=cg04774476	feature_id[1257].value > threshold=0.5675267577171326
node_256: feature_name=cg19259111	feature_id[63].value > threshold=0.40771469473838806

node_264: feature_name=cg21483216	feature_id[5253].value > threshold=0.6332719326019287
node_266: feature_name=cg04026354	feature_id[3502].value <= threshold=0.42332448065280914
node_267: feature_name=cg11444072	feature_id[116].value > threshold=0.4940422773361206
node_273: feature_name=cg08571020	feature_id[381].value > threshold=0.4616774618625641
node_275: feature_name=cg08331427	feature_id[2081].value > threshold=0.5443233847618103
node_281: feature_name=cg00216961	feature_id[945].value <= threshold=0.4166988283395767
node_282: feature_name=cg04716580	feature_id[1390].value > threshold=0.4204266667366028
node_286: feature_name=cg07891483	feature_id[1877].value > threshold=0.829958975315094
node_288: feature_name=cg01475325	feature_id[3969].value > threshold=0.45323844254016876
node_292: feature_name=cg19702397	feature_id[2066].value > threshold=0.3536848723888397
node_294: feature_name=cg21936959	feature_id[595].value > threshold=0.5567200481891632
node_308: feature_name=cg18645642	feature_id[262].value > threshold=0.42608243227005005
node_310: feature_name=cg27351239	feature_id[1874].value <= threshold=0.6790103316307068
node_311: feature_name=cg13107973	feature_id[1326].value > threshold=0.6562457382678986
Class: chondrosarcoma (CSA)	
Rules_62	passed counts:4
node_0: feature_name=cg11915444	feature_id[2002].value > threshold=0.3402601182460785
node_10: feature_name=cg26016985	feature_id[4407].value > threshold=0.6493876278400421
node_16: feature_name=cg23157618	feature_id[5511].value > threshold=0.5759606957435608
node_20: feature_name=cg10480329	feature_id[1439].value > threshold=0.5518321692943573
node_48: feature_name=cg17843418	feature_id[4868].value > threshold=0.34969255328178406
node_52: feature_name=cg24407065	feature_id[4522].value > threshold=0.5364363789558411

node_56: feature_name=cg07281938	feature_id[249].value > threshold=0.7506992518901825
node_78: feature_name=cg21759907	feature_id[1650].value > threshold=0.46823520958423615
node_98: feature_name=cg10886334	feature_id[3975].value > threshold=0.43698127567768097
node_102: feature_name=cg09373983	feature_id[5025].value > threshold=0.5575815737247467
node_106: feature_name=cg12948116	feature_id[5360].value <= threshold=0.5658791363239288
node_107: feature_name=cg21906519	feature_id[5251].value > threshold=0.6089984774589539
node_111: feature_name=cg17537493	feature_id[4217].value > threshold=0.5825372636318207
node_117: feature_name=cg15720017	feature_id[4443].value > threshold=0.7009969055652618
node_119: feature_name=cg06038180	feature_id[3480].value > threshold=0.4583848565816879
node_121: feature_name=cg08619651	feature_id[4882].value <= threshold=0.2105495035648346
node_122: feature_name=cg01014262	feature_id[1911].value <= threshold=0.5327437818050385
node_123: feature_name=cg16863382	feature_id[2922].value > threshold=0.7200668156147003
node_133: feature_name=cg06989443	feature_id[2327].value <= threshold=0.6579365730285645
node_134: feature_name=cg07344990	feature_id[2398].value > threshold=0.5562876164913177
node_138: feature_name=cg02966841	feature_id[2199].value > threshold=0.7744501233100891
node_204: feature_name=cg12120430	feature_id[3727].value > threshold=0.5775820016860962
node_206: feature_name=cg18386876	feature_id[4066].value > threshold=0.6847257018089294
node_208: feature_name=cg02053092	feature_id[3610].value > threshold=0.39507485926151276
node_210: feature_name=cg10298992	feature_id[3603].value <= threshold=0.5707973837852478
node_211: feature_name=cg02436098	feature_id[1712].value <= threshold=0.27362556010484695
node_212: feature_name=cg15464763	feature_id[2786].value <= threshold=0.6618955731391907
Class: rhabdomyosarcoma (RMS)	

Rules_63	passed counts:4
node_0: feature_name=cg11915444	feature_id[2002].value > threshold=0.3402601182460785
node_10: feature_name=cg26016985	feature_id[4407].value > threshold=0.6493876278400421
node_16: feature_name=cg23157618	feature_id[5511].value > threshold=0.5759606957435608
node_20: feature_name=cg10480329	feature_id[1439].value > threshold=0.5518321692943573
node_48: feature_name=cg17843418	feature_id[4868].value > threshold=0.34969255328178406
node_52: feature_name=cg24407065	feature_id[4522].value > threshold=0.5364363789558411
node_56: feature_name=cg07281938	feature_id[249].value > threshold=0.7506992518901825
node_78: feature_name=cg21759907	feature_id[1650].value > threshold=0.46823520958423615
node_98: feature_name=cg10886334	feature_id[3975].value > threshold=0.43698127567768097
node_102: feature_name=cg09373983	feature_id[5025].value > threshold=0.5575815737247467
node_106: feature_name=cg12948116	feature_id[5360].value <= threshold=0.5658791363239288
node_107: feature_name=cg21906519	feature_id[5251].value > threshold=0.6089984774589539
node_111: feature_name=cg17537493	feature_id[4217].value > threshold=0.5825372636318207
node_117: feature_name=cg15720017	feature_id[4443].value > threshold=0.7009969055652618
node_119: feature_name=cg06038180	feature_id[3480].value > threshold=0.4583848565816879
node_121: feature_name=cg08619651	feature_id[4882].value <= threshold=0.2105495035648346
node_122: feature_name=cg01014262	feature_id[1911].value <= threshold=0.5327437818050385
node_123: feature_name=cg16863382	feature_id[2922].value > threshold=0.7200668156147003
node_133: feature_name=cg06989443	feature_id[2327].value <= threshold=0.6579365730285645
node_134: feature_name=cg07344990	feature_id[2398].value > threshold=0.5562876164913177

node_138: feature_name=cg02966841	feature_id[2199].value <= threshold=0.7744501233100891
node_139: feature_name=cg06989253	feature_id[194].value <= threshold=0.7899391353130341
node_140: feature_name=cg24617568	feature_id[3282].value <= threshold=0.26932457089424133
node_141: feature_name=cg12614090	feature_id[2765].value <= threshold=0.1813211366534233
node_142: feature_name=cg10917602	feature_id[4685].value <= threshold=0.5744736790657043
node_143: feature_name=cg23723410	feature_id[34].value > threshold=0.3201078921556473
Class: sarcoma (SARC)	
Rules_64	passed counts:4
node_0: feature_name=cg11915444	feature_id[2002].value > threshold=0.3402601182460785
node_10: feature_name=cg26016985	feature_id[4407].value > threshold=0.6493876278400421
node_16: feature_name=cg23157618	feature_id[5511].value > threshold=0.5759606957435608
node_20: feature_name=cg10480329	feature_id[1439].value > threshold=0.5518321692943573
node_48: feature_name=cg17843418	feature_id[4868].value > threshold=0.34969255328178406
node_52: feature_name=cg24407065	feature_id[4522].value > threshold=0.5364363789558411
node_56: feature_name=cg07281938	feature_id[249].value > threshold=0.7506992518901825
node_78: feature_name=cg21759907	feature_id[1650].value > threshold=0.46823520958423615
node_98: feature_name=cg10886334	feature_id[3975].value > threshold=0.43698127567768097
node_102: feature_name=cg09373983	feature_id[5025].value > threshold=0.5575815737247467
node_106: feature_name=cg12948116	feature_id[5360].value <= threshold=0.5658791363239288
node_107: feature_name=cg21906519	feature_id[5251].value > threshold=0.6089984774589539
node_111: feature_name=cg17537493	feature_id[4217].value > threshold=0.5825372636318207
node_117: feature_name=cg15720017	feature_id[4443].value > threshold=0.7009969055652618

node_119: feature_name=cg06038180	feature_id[3480].value > threshold=0.4583848565816879
node_121: feature_name=cg08619651	feature_id[4882].value <= threshold=0.2105495035648346
node_122: feature_name=cg01014262	feature_id[1911].value <= threshold=0.5327437818050385
node_123: feature_name=cg16863382	feature_id[2922].value > threshold=0.7200668156147003
node_133: feature_name=cg06989443	feature_id[2327].value <= threshold=0.6579365730285645
node_134: feature_name=cg07344990	feature_id[2398].value > threshold=0.5562876164913177
node_138: feature_name=cg02966841	feature_id[2199].value <= threshold=0.7744501233100891
node_139: feature_name=cg06989253	feature_id[194].value <= threshold=0.7899391353130341
node_140: feature_name=cg24617568	feature_id[3282].value <= threshold=0.26932457089424133
node_141: feature_name=cg12614090	feature_id[2765].value <= threshold=0.1813211366534233
node_142: feature_name=cg10917602	feature_id[4685].value <= threshold=0.5744736790657043
node_143: feature_name=cg23723410	feature_id[34].value <= threshold=0.3201078921556473
node_144: feature_name=cg26804423	feature_id[1725].value <= threshold=0.39587317407131195
Class: clear cell sarcoma of soft parts (CCS)	
Rules_65	passed counts:4
node_0: feature_name=cg11915444	feature_id[2002].value > threshold=0.3402601182460785
node_10: feature_name=cg26016985	feature_id[4407].value > threshold=0.6493876278400421
node_16: feature_name=cg23157618	feature_id[5511].value > threshold=0.5759606957435608
node_20: feature_name=cg10480329	feature_id[1439].value > threshold=0.5518321692943573
node_48: feature_name=cg17843418	feature_id[4868].value > threshold=0.34969255328178406
node_52: feature_name=cg24407065	feature_id[4522].value > threshold=0.5364363789558411
node_56: feature_name=cg07281938	feature_id[249].value > threshold=0.7506992518901825

node_78: feature_name=cg21759907	feature_id[1650].value > threshold=0.46823520958423615
node_98: feature_name=cg10886334	feature_id[3975].value > threshold=0.43698127567768097
node_102: feature_name=cg09373983	feature_id[5025].value > threshold=0.5575815737247467
node_106: feature_name=cg12948116	feature_id[5360].value <= threshold=0.5658791363239288
node_107: feature_name=cg21906519	feature_id[5251].value > threshold=0.6089984774589539
node_111: feature_name=cg17537493	feature_id[4217].value > threshold=0.5825372636318207
node_117: feature_name=cg15720017	feature_id[4443].value > threshold=0.7009969055652618
node_119: feature_name=cg06038180	feature_id[3480].value > threshold=0.4583848565816879
node_121: feature_name=cg08619651	feature_id[4882].value <= threshold=0.2105495035648346
node_122: feature_name=cg01014262	feature_id[1911].value <= threshold=0.5327437818050385
node_123: feature_name=cg16863382	feature_id[2922].value <= threshold=0.7200668156147003
node_124: feature_name=cg11225330	feature_id[3185].value > threshold=0.7814575433731079
node_126: feature_name=cg15543534	feature_id[423].value > threshold=0.86179119348526
Class: alveolar rhabdomyosarcoma (RMS (ALV))	
Rules_66	passed counts:4
node_0: feature_name=cg11915444	feature_id[2002].value > threshold=0.3402601182460785
node_10: feature_name=cg26016985	feature_id[4407].value > threshold=0.6493876278400421
node_16: feature_name=cg23157618	feature_id[5511].value > threshold=0.5759606957435608
node_20: feature_name=cg10480329	feature_id[1439].value > threshold=0.5518321692943573
node_48: feature_name=cg17843418	feature_id[4868].value > threshold=0.34969255328178406
node_52: feature_name=cg24407065	feature_id[4522].value > threshold=0.5364363789558411

node_56: feature_name=cg07281938	feature_id[249].value <= threshold=0.7506992518901825
node_57: feature_name=cg21189849	feature_id[1261].value > threshold=0.7545044124126434
node_63: feature_name=cg18048309	feature_id[2794].value > threshold=0.36701037734746933
node_65: feature_name=cg05851887	feature_id[4211].value > threshold=0.7704950571060181
Class: clear cell chondrosarcoma (CSA (CC))	
Rules_67	passed counts:4
node_0: feature_name=cg11915444	feature_id[2002].value > threshold=0.3402601182460785
node_10: feature_name=cg26016985	feature_id[4407].value > threshold=0.6493876278400421
node_16: feature_name=cg23157618	feature_id[5511].value > threshold=0.5759606957435608
node_20: feature_name=cg10480329	feature_id[1439].value > threshold=0.5518321692943573
node_48: feature_name=cg17843418	feature_id[4868].value > threshold=0.34969255328178406
node_52: feature_name=cg24407065	feature_id[4522].value > threshold=0.5364363789558411
node_56: feature_name=cg07281938	feature_id[249].value <= threshold=0.7506992518901825
node_57: feature_name=cg21189849	feature_id[1261].value > threshold=0.7545044124126434
node_63: feature_name=cg18048309	feature_id[2794].value > threshold=0.36701037734746933
node_65: feature_name=cg05851887	feature_id[4211].value <= threshold=0.7704950571060181
node_66: feature_name=cg22575379	feature_id[2711].value <= threshold=0.4320530295372009
Class: chordoma (CHORD)	
Rules_68	passed counts:3
node_0: feature_name=cg11915444	feature_id[2002].value > threshold=0.3402601182460785
node_10: feature_name=cg26016985	feature_id[4407].value > threshold=0.6493876278400421
node_16: feature_name=cg23157618	feature_id[5511].value > threshold=0.5759606957435608

node_20: feature_name=cg10480329	feature_id[1439].value > threshold=0.5518321692943573
node_48: feature_name=cg17843418	feature_id[4868].value > threshold=0.34969255328178406
node_52: feature_name=cg24407065	feature_id[4522].value > threshold=0.5364363789558411
node_56: feature_name=cg07281938	feature_id[249].value > threshold=0.7506992518901825
node_78: feature_name=cg21759907	feature_id[1650].value > threshold=0.46823520958423615
node_98: feature_name=cg10886334	feature_id[3975].value > threshold=0.43698127567768097
node_102: feature_name=cg09373983	feature_id[5025].value > threshold=0.5575815737247467
node_106: feature_name=cg12948116	feature_id[5360].value <= threshold=0.5658791363239288
node_107: feature_name=cg21906519	feature_id[5251].value > threshold=0.6089984774589539
node_111: feature_name=cg17537493	feature_id[4217].value > threshold=0.5825372636318207
node_117: feature_name=cg15720017	feature_id[4443].value > threshold=0.7009969055652618
node_119: feature_name=cg06038180	feature_id[3480].value > threshold=0.4583848565816879
node_121: feature_name=cg08619651	feature_id[4882].value <= threshold=0.2105495035648346
node_122: feature_name=cg01014262	feature_id[1911].value <= threshold=0.5327437818050385
node_123: feature_name=cg16863382	feature_id[2922].value > threshold=0.7200668156147003
node_133: feature_name=cg06989443	feature_id[2327].value > threshold=0.6579365730285645
node_355: feature_name=cg27470978	feature_id[4804].value > threshold=0.6264137625694275
node_361: feature_name=cg20252837	feature_id[420].value > threshold=0.53105828166008
node_363: feature_name=cg27437304	feature_id[1051].value > threshold=0.7641371190547943
node_365: feature_name=cg08215532	feature_id[3203].value > threshold=0.45234426856040955
Class: embryonal rhabdomyosarcoma (RMS (EMB))	

Rules_69	passed counts:3
node_0: feature_name=cg11915444	feature_id[2002].value > threshold=0.3402601182460785
node_10: feature_name=cg26016985	feature_id[4407].value > threshold=0.6493876278400421
node_16: feature_name=cg23157618	feature_id[5511].value > threshold=0.5759606957435608
node_20: feature_name=cg10480329	feature_id[1439].value > threshold=0.5518321692943573
node_48: feature_name=cg17843418	feature_id[4868].value > threshold=0.34969255328178406
node_52: feature_name=cg24407065	feature_id[4522].value > threshold=0.5364363789558411
node_56: feature_name=cg07281938	feature_id[249].value > threshold=0.7506992518901825
node_78: feature_name=cg21759907	feature_id[1650].value > threshold=0.46823520958423615
node_98: feature_name=cg10886334	feature_id[3975].value > threshold=0.43698127567768097
node_102: feature_name=cg09373983	feature_id[5025].value > threshold=0.5575815737247467
node_106: feature_name=cg12948116	feature_id[5360].value <= threshold=0.5658791363239288
node_107: feature_name=cg21906519	feature_id[5251].value > threshold=0.6089984774589539
node_111: feature_name=cg17537493	feature_id[4217].value > threshold=0.5825372636318207
node_117: feature_name=cg15720017	feature_id[4443].value > threshold=0.7009969055652618
node_119: feature_name=cg06038180	feature_id[3480].value > threshold=0.4583848565816879
node_121: feature_name=cg08619651	feature_id[4882].value <= threshold=0.2105495035648346
node_122: feature_name=cg01014262	feature_id[1911].value <= threshold=0.5327437818050385
node_123: feature_name=cg16863382	feature_id[2922].value > threshold=0.7200668156147003
node_133: feature_name=cg06989443	feature_id[2327].value > threshold=0.6579365730285645
node_355: feature_name=cg27470978	feature_id[4804].value > threshold=0.6264137625694275
node_361: feature_name=cg20252837	feature_id[420].value > threshold=0.53105828166008

node_363: feature_name=cg27437304	feature_id[1051].value <= threshold=0.7641371190547943
Class: high-grade endometrial stromal sarcoma (ESS (HG))	
Rules_70	passed counts:3
node_0: feature_name=cg11915444	feature_id[2002].value > threshold=0.3402601182460785
node_10: feature_name=cg26016985	feature_id[4407].value > threshold=0.6493876278400421
node_16: feature_name=cg23157618	feature_id[5511].value > threshold=0.5759606957435608
node_20: feature_name=cg10480329	feature_id[1439].value > threshold=0.5518321692943573
node_48: feature_name=cg17843418	feature_id[4868].value > threshold=0.34969255328178406
node_52: feature_name=cg24407065	feature_id[4522].value > threshold=0.5364363789558411
node_56: feature_name=cg07281938	feature_id[249].value > threshold=0.7506992518901825
node_78: feature_name=cg21759907	feature_id[1650].value > threshold=0.46823520958423615
node_98: feature_name=cg10886334	feature_id[3975].value > threshold=0.43698127567768097
node_102: feature_name=cg09373983	feature_id[5025].value > threshold=0.5575815737247467
node_106: feature_name=cg12948116	feature_id[5360].value <= threshold=0.5658791363239288
node_107: feature_name=cg21906519	feature_id[5251].value > threshold=0.6089984774589539
node_111: feature_name=cg17537493	feature_id[4217].value > threshold=0.5825372636318207
node_117: feature_name=cg15720017	feature_id[4443].value > threshold=0.7009969055652618
node_119: feature_name=cg06038180	feature_id[3480].value > threshold=0.4583848565816879
node_121: feature_name=cg08619651	feature_id[4882].value <= threshold=0.2105495035648346
node_122: feature_name=cg01014262	feature_id[1911].value <= threshold=0.5327437818050385
node_123: feature_name=cg16863382	feature_id[2922].value > threshold=0.7200668156147003

node_133: feature_name=cg06989443	feature_id[2327].value <= threshold=0.6579365730285645
node_134: feature_name=cg07344990	feature_id[2398].value > threshold=0.5562876164913177
node_138: feature_name=cg02966841	feature_id[2199].value > threshold=0.7744501233100891
node_204: feature_name=cg12120430	feature_id[3727].value > threshold=0.5775820016860962
node_206: feature_name=cg18386876	feature_id[4066].value > threshold=0.6847257018089294
node_208: feature_name=cg02053092	feature_id[3610].value > threshold=0.39507485926151276
node_210: feature_name=cg10298992	feature_id[3603].value > threshold=0.5707973837852478
node_228: feature_name=cg03840920	feature_id[4524].value > threshold=0.5365143716335297
node_234: feature_name=cg25364972	feature_id[1792].value > threshold=0.2418053299188614
node_238: feature_name=cg23485307	feature_id[221].value > threshold=0.14884746074676514
node_242: feature_name=cg04873169	feature_id[4696].value > threshold=0.6058756709098816
node_244: feature_name=cg17009731	feature_id[3218].value > threshold=0.6602252125740051
node_246: feature_name=cg18253910	feature_id[4458].value > threshold=0.6904322504997253
node_250: feature_name=cg04774476	feature_id[1257].value > threshold=0.5675267577171326
node_256: feature_name=cg19259111	feature_id[63].value > threshold=0.40771469473838806
node_264: feature_name=cg21483216	feature_id[5253].value > threshold=0.6332719326019287
node_266: feature_name=cg04026354	feature_id[3502].value <= threshold=0.42332448065280914
node_267: feature_name=cg11444072	feature_id[116].value > threshold=0.4940422773361206
node_273: feature_name=cg08571020	feature_id[381].value > threshold=0.4616774618625641
node_275: feature_name=cg08331427	feature_id[2081].value > threshold=0.5443233847618103
node_281: feature_name=cg00216961	feature_id[945].value <= threshold=0.4166988283395767
node_282: feature_name=cg04716580	feature_id[1390].value > threshold=0.4204266667366028

node_286: feature_name=cg07891483	feature_id[1877].value > threshold=0.829958975315094
node_288: feature_name=cg01475325	feature_id[3969].value > threshold=0.45323844254016876
node_292: feature_name=cg19702397	feature_id[2066].value > threshold=0.3536848723888397
node_294: feature_name=cg21936959	feature_id[595].value > threshold=0.5567200481891632
node_308: feature_name=cg18645642	feature_id[262].value > threshold=0.42608243227005005
node_310: feature_name=cg27351239	feature_id[1874].value <= threshold=0.6790103316307068
node_311: feature_name=cg13107973	feature_id[1326].value <= threshold=0.6562457382678986
node_312: feature_name=cg10024583	feature_id[1175].value > threshold=0.518094539642334
Class: rhabdomyosarcoma (RMS)	
Rules_71	passed counts:3
node_0: feature_name=cg11915444	feature_id[2002].value > threshold=0.3402601182460785
node_10: feature_name=cg26016985	feature_id[4407].value > threshold=0.6493876278400421
node_16: feature_name=cg23157618	feature_id[5511].value > threshold=0.5759606957435608
node_20: feature_name=cg10480329	feature_id[1439].value > threshold=0.5518321692943573
node_48: feature_name=cg17843418	feature_id[4868].value > threshold=0.34969255328178406
node_52: feature_name=cg24407065	feature_id[4522].value > threshold=0.5364363789558411
node_56: feature_name=cg07281938	feature_id[249].value > threshold=0.7506992518901825
node_78: feature_name=cg21759907	feature_id[1650].value > threshold=0.46823520958423615
node_98: feature_name=cg10886334	feature_id[3975].value > threshold=0.43698127567768097
node_102: feature_name=cg09373983	feature_id[5025].value > threshold=0.5575815737247467
node_106: feature_name=cg12948116	feature_id[5360].value <= threshold=0.5658791363239288
node_107: feature_name=cg21906519	feature_id[5251].value > threshold=0.6089984774589539

node_111: feature_name=cg17537493	feature_id[4217].value > threshold=0.5825372636318207
node_117: feature_name=cg15720017	feature_id[4443].value > threshold=0.7009969055652618
node_119: feature_name=cg06038180	feature_id[3480].value > threshold=0.4583848565816879
node_121: feature_name=cg08619651	feature_id[4882].value <= threshold=0.2105495035648346
node_122: feature_name=cg01014262	feature_id[1911].value <= threshold=0.5327437818050385
node_123: feature_name=cg16863382	feature_id[2922].value > threshold=0.7200668156147003
node_133: feature_name=cg06989443	feature_id[2327].value <= threshold=0.6579365730285645
node_134: feature_name=cg07344990	feature_id[2398].value > threshold=0.5562876164913177
node_138: feature_name=cg02966841	feature_id[2199].value > threshold=0.7744501233100891
node_204: feature_name=cg12120430	feature_id[3727].value > threshold=0.5775820016860962
node_206: feature_name=cg18386876	feature_id[4066].value > threshold=0.6847257018089294
node_208: feature_name=cg02053092	feature_id[3610].value > threshold=0.39507485926151276
node_210: feature_name=cg10298992	feature_id[3603].value > threshold=0.5707973837852478
node_228: feature_name=cg03840920	feature_id[4524].value > threshold=0.5365143716335297
node_234: feature_name=cg25364972	feature_id[1792].value > threshold=0.2418053299188614
node_238: feature_name=cg23485307	feature_id[221].value > threshold=0.14884746074676514
node_242: feature_name=cg04873169	feature_id[4696].value > threshold=0.6058756709098816
node_244: feature_name=cg17009731	feature_id[3218].value > threshold=0.6602252125740051
node_246: feature_name=cg18253910	feature_id[4458].value > threshold=0.6904322504997253
node_250: feature_name=cg04774476	feature_id[1257].value > threshold=0.5675267577171326
node_256: feature_name=cg19259111	feature_id[63].value <= threshold=0.40771469473838806
node_257: feature_name=cg10803714	feature_id[856].value > threshold=0.4142659604549408

node_259: feature_name=cg23350812	feature_id[1133].value <= threshold=0.8047192692756653
Class: high-grade endometrial stromal sarcoma (ESS (HG))	
Rules_72	passed counts:3
node_0: feature_name=cg11915444	feature_id[2002].value > threshold=0.3402601182460785
node_10: feature_name=cg26016985	feature_id[4407].value > threshold=0.6493876278400421
node_16: feature_name=cg23157618	feature_id[5511].value > threshold=0.5759606957435608
node_20: feature_name=cg10480329	feature_id[1439].value > threshold=0.5518321692943573
node_48: feature_name=cg17843418	feature_id[4868].value > threshold=0.34969255328178406
node_52: feature_name=cg24407065	feature_id[4522].value > threshold=0.5364363789558411
node_56: feature_name=cg07281938	feature_id[249].value > threshold=0.7506992518901825
node_78: feature_name=cg21759907	feature_id[1650].value > threshold=0.46823520958423615
node_98: feature_name=cg10886334	feature_id[3975].value > threshold=0.43698127567768097
node_102: feature_name=cg09373983	feature_id[5025].value > threshold=0.5575815737247467
node_106: feature_name=cg12948116	feature_id[5360].value <= threshold=0.5658791363239288
node_107: feature_name=cg21906519	feature_id[5251].value > threshold=0.6089984774589539
node_111: feature_name=cg17537493	feature_id[4217].value > threshold=0.5825372636318207
node_117: feature_name=cg15720017	feature_id[4443].value > threshold=0.7009969055652618
node_119: feature_name=cg06038180	feature_id[3480].value > threshold=0.4583848565816879
node_121: feature_name=cg08619651	feature_id[4882].value <= threshold=0.2105495035648346
node_122: feature_name=cg01014262	feature_id[1911].value <= threshold=0.5327437818050385
node_123: feature_name=cg16863382	feature_id[2922].value > threshold=0.7200668156147003

node_133: feature_name=cg06989443	feature_id[2327].value <= threshold=0.6579365730285645
node_134: feature_name=cg07344990	feature_id[2398].value > threshold=0.5562876164913177
node_138: feature_name=cg02966841	feature_id[2199].value > threshold=0.7744501233100891
node_204: feature_name=cg12120430	feature_id[3727].value > threshold=0.5775820016860962
node_206: feature_name=cg18386876	feature_id[4066].value > threshold=0.6847257018089294
node_208: feature_name=cg02053092	feature_id[3610].value > threshold=0.39507485926151276
node_210: feature_name=cg10298992	feature_id[3603].value <= threshold=0.5707973837852478
node_211: feature_name=cg02436098	feature_id[1712].value <= threshold=0.27362556010484695
node_212: feature_name=cg15464763	feature_id[2786].value > threshold=0.6618955731391907
node_214: feature_name=cg15264991	feature_id[2805].value > threshold=0.701563149690628
Class: high-grade endometrial stromal sarcoma (ESS (HG))	
Rules_73	passed counts:3
node_0: feature_name=cg11915444	feature_id[2002].value > threshold=0.3402601182460785
node_10: feature_name=cg26016985	feature_id[4407].value > threshold=0.6493876278400421
node_16: feature_name=cg23157618	feature_id[5511].value > threshold=0.5759606957435608
node_20: feature_name=cg10480329	feature_id[1439].value > threshold=0.5518321692943573
node_48: feature_name=cg17843418	feature_id[4868].value > threshold=0.34969255328178406
node_52: feature_name=cg24407065	feature_id[4522].value > threshold=0.5364363789558411
node_56: feature_name=cg07281938	feature_id[249].value > threshold=0.7506992518901825
node_78: feature_name=cg21759907	feature_id[1650].value > threshold=0.46823520958423615
node_98: feature_name=cg10886334	feature_id[3975].value > threshold=0.43698127567768097

node_102: feature_name=cg09373983	feature_id[5025].value > threshold=0.5575815737247467
node_106: feature_name=cg12948116	feature_id[5360].value <= threshold=0.5658791363239288
node_107: feature_name=cg21906519	feature_id[5251].value > threshold=0.6089984774589539
node_111: feature_name=cg17537493	feature_id[4217].value > threshold=0.5825372636318207
node_117: feature_name=cg15720017	feature_id[4443].value > threshold=0.7009969055652618
node_119: feature_name=cg06038180	feature_id[3480].value > threshold=0.4583848565816879
node_121: feature_name=cg08619651	feature_id[4882].value <= threshold=0.2105495035648346
node_122: feature_name=cg01014262	feature_id[1911].value <= threshold=0.5327437818050385
node_123: feature_name=cg16863382	feature_id[2922].value > threshold=0.7200668156147003
node_133: feature_name=cg06989443	feature_id[2327].value <= threshold=0.6579365730285645
node_134: feature_name=cg07344990	feature_id[2398].value > threshold=0.5562876164913177
node_138: feature_name=cg02966841	feature_id[2199].value <= threshold=0.7744501233100891
node_139: feature_name=cg06989253	feature_id[194].value > threshold=0.7899391353130341
node_171: feature_name=cg07041720	feature_id[932].value > threshold=0.5004830807447433
node_189: feature_name=cg19374752	feature_id[196].value > threshold=0.6096120476722717
Class: leiomyosarcoma (LMS)	
Rules_74	passed counts:3
node_0: feature_name=cg11915444	feature_id[2002].value > threshold=0.3402601182460785
node_10: feature_name=cg26016985	feature_id[4407].value > threshold=0.6493876278400421
node_16: feature_name=cg23157618	feature_id[5511].value > threshold=0.5759606957435608
node_20: feature_name=cg10480329	feature_id[1439].value > threshold=0.5518321692943573
node_48: feature_name=cg17843418	feature_id[4868].value > threshold=0.34969255328178406

node_52: feature_name=cg24407065	feature_id[4522].value > threshold=0.5364363789558411
node_56: feature_name=cg07281938	feature_id[249].value > threshold=0.7506992518901825
node_78: feature_name=cg21759907	feature_id[1650].value > threshold=0.46823520958423615
node_98: feature_name=cg10886334	feature_id[3975].value > threshold=0.43698127567768097
node_102: feature_name=cg09373983	feature_id[5025].value > threshold=0.5575815737247467
node_106: feature_name=cg12948116	feature_id[5360].value <= threshold=0.5658791363239288
node_107: feature_name=cg21906519	feature_id[5251].value > threshold=0.6089984774589539
node_111: feature_name=cg17537493	feature_id[4217].value > threshold=0.5825372636318207
node_117: feature_name=cg15720017	feature_id[4443].value > threshold=0.7009969055652618
node_119: feature_name=cg06038180	feature_id[3480].value > threshold=0.4583848565816879
node_121: feature_name=cg08619651	feature_id[4882].value <= threshold=0.2105495035648346
node_122: feature_name=cg01014262	feature_id[1911].value <= threshold=0.5327437818050385
node_123: feature_name=cg16863382	feature_id[2922].value > threshold=0.7200668156147003
node_133: feature_name=cg06989443	feature_id[2327].value <= threshold=0.6579365730285645
node_134: feature_name=cg07344990	feature_id[2398].value > threshold=0.5562876164913177
node_138: feature_name=cg02966841	feature_id[2199].value <= threshold=0.7744501233100891
node_139: feature_name=cg06989253	feature_id[194].value > threshold=0.7899391353130341
node_171: feature_name=cg07041720	feature_id[932].value > threshold=0.5004830807447433
node_189: feature_name=cg19374752	feature_id[196].value <= threshold=0.6096120476722717
node_190: feature_name=cg23085846	feature_id[3499].value > threshold=0.8292354643344879
Class: well differentiated liposarcoma (WDLS)/dedifferentiated liposarcoma (DDLS)	

Rules_75	passed counts:3
node_0: feature_name=cg11915444	feature_id[2002].value > threshold=0.3402601182460785
node_10: feature_name=cg26016985	feature_id[4407].value > threshold=0.6493876278400421
node_16: feature_name=cg23157618	feature_id[5511].value > threshold=0.5759606957435608
node_20: feature_name=cg10480329	feature_id[1439].value > threshold=0.5518321692943573
node_48: feature_name=cg17843418	feature_id[4868].value > threshold=0.34969255328178406
node_52: feature_name=cg24407065	feature_id[4522].value > threshold=0.5364363789558411
node_56: feature_name=cg07281938	feature_id[249].value > threshold=0.7506992518901825
node_78: feature_name=cg21759907	feature_id[1650].value > threshold=0.46823520958423615
node_98: feature_name=cg10886334	feature_id[3975].value > threshold=0.43698127567768097
node_102: feature_name=cg09373983	feature_id[5025].value > threshold=0.5575815737247467
node_106: feature_name=cg12948116	feature_id[5360].value <= threshold=0.5658791363239288
node_107: feature_name=cg21906519	feature_id[5251].value > threshold=0.6089984774589539
node_111: feature_name=cg17537493	feature_id[4217].value > threshold=0.5825372636318207
node_117: feature_name=cg15720017	feature_id[4443].value > threshold=0.7009969055652618
node_119: feature_name=cg06038180	feature_id[3480].value > threshold=0.4583848565816879
node_121: feature_name=cg08619651	feature_id[4882].value <= threshold=0.2105495035648346
node_122: feature_name=cg01014262	feature_id[1911].value <= threshold=0.5327437818050385
node_123: feature_name=cg16863382	feature_id[2922].value > threshold=0.7200668156147003
node_133: feature_name=cg06989443	feature_id[2327].value <= threshold=0.6579365730285645
node_134: feature_name=cg07344990	feature_id[2398].value > threshold=0.5562876164913177
node_138: feature_name=cg02966841	feature_id[2199].value <= threshold=0.7744501233100891

node_139: feature_name=cg06989253	feature_id[194].value <= threshold=0.7899391353130341
node_140: feature_name=cg24617568	feature_id[3282].value <= threshold=0.26932457089424133
node_141: feature_name=cg12614090	feature_id[2765].value <= threshold=0.1813211366534233
node_142: feature_name=cg10917602	feature_id[4685].value <= threshold=0.5744736790657043
node_143: feature_name=cg23723410	feature_id[34].value <= threshold=0.3201078921556473
node_144: feature_name=cg26804423	feature_id[1725].value > threshold=0.39587317407131195
node_146: feature_name=cg20080282	feature_id[1150].value > threshold=0.856019914150238
Class: undifferentiated sarcoma (USARC)	
Rules_76	passed counts:3
node_0: feature_name=cg11915444	feature_id[2002].value > threshold=0.3402601182460785
node_10: feature_name=cg26016985	feature_id[4407].value > threshold=0.6493876278400421
node_16: feature_name=cg23157618	feature_id[5511].value > threshold=0.5759606957435608
node_20: feature_name=cg10480329	feature_id[1439].value > threshold=0.5518321692943573
node_48: feature_name=cg17843418	feature_id[4868].value > threshold=0.34969255328178406
node_52: feature_name=cg24407065	feature_id[4522].value > threshold=0.5364363789558411
node_56: feature_name=cg07281938	feature_id[249].value > threshold=0.7506992518901825
node_78: feature_name=cg21759907	feature_id[1650].value > threshold=0.46823520958423615
node_98: feature_name=cg10886334	feature_id[3975].value > threshold=0.43698127567768097
node_102: feature_name=cg09373983	feature_id[5025].value > threshold=0.5575815737247467
node_106: feature_name=cg12948116	feature_id[5360].value <= threshold=0.5658791363239288
node_107: feature_name=cg21906519	feature_id[5251].value > threshold=0.6089984774589539
node_111: feature_name=cg17537493	feature_id[4217].value <= threshold=0.5825372636318207

node_112: feature_name=cg07260789	feature_id[1074].value > threshold=0.8945248425006866
node_114: feature_name=cg11251006	feature_id[4182].value > threshold=0.7591733336448669
Class: Kaposi sarcoma (KS)	
Rules_77	passed counts:3
node_0: feature_name=cg11915444	feature_id[2002].value > threshold=0.3402601182460785
node_10: feature_name=cg26016985	feature_id[4407].value > threshold=0.6493876278400421
node_16: feature_name=cg23157618	feature_id[5511].value > threshold=0.5759606957435608
node_20: feature_name=cg10480329	feature_id[1439].value > threshold=0.5518321692943573
node_48: feature_name=cg17843418	feature_id[4868].value > threshold=0.34969255328178406
node_52: feature_name=cg24407065	feature_id[4522].value > threshold=0.5364363789558411
node_56: feature_name=cg07281938	feature_id[249].value > threshold=0.7506992518901825
node_78: feature_name=cg21759907	feature_id[1650].value <= threshold=0.46823520958423615
node_79: feature_name=cg25476129	feature_id[2302].value <= threshold=0.1687404215335846
node_80: feature_name=cg26362368	feature_id[414].value <= threshold=0.834072083234787
node_81: feature_name=cg15881332	feature_id[154].value > threshold=0.7577665150165558
Class: melanoma (MEL)	
Rules_78	passed counts:2
node_0: feature_name=cg11915444	feature_id[2002].value > threshold=0.3402601182460785
node_10: feature_name=cg26016985	feature_id[4407].value > threshold=0.6493876278400421
node_16: feature_name=cg23157618	feature_id[5511].value > threshold=0.5759606957435608
node_20: feature_name=cg10480329	feature_id[1439].value > threshold=0.5518321692943573
node_48: feature_name=cg17843418	feature_id[4868].value > threshold=0.34969255328178406

node_52: feature_name=cg24407065	feature_id[4522].value > threshold=0.5364363789558411
node_56: feature_name=cg07281938	feature_id[249].value > threshold=0.7506992518901825
node_78: feature_name=cg21759907	feature_id[1650].value > threshold=0.46823520958423615
node_98: feature_name=cg10886334	feature_id[3975].value > threshold=0.43698127567768097
node_102: feature_name=cg09373983	feature_id[5025].value > threshold=0.5575815737247467
node_106: feature_name=cg12948116	feature_id[5360].value <= threshold=0.5658791363239288
node_107: feature_name=cg21906519	feature_id[5251].value > threshold=0.6089984774589539
node_111: feature_name=cg17537493	feature_id[4217].value > threshold=0.5825372636318207
node_117: feature_name=cg15720017	feature_id[4443].value > threshold=0.7009969055652618
node_119: feature_name=cg06038180	feature_id[3480].value > threshold=0.4583848565816879
node_121: feature_name=cg08619651	feature_id[4882].value <= threshold=0.2105495035648346
node_122: feature_name=cg01014262	feature_id[1911].value > threshold=0.5327437818050385
node_382: feature_name=cg26240231	feature_id[4832].value <= threshold=0.9200497567653656
node_383: feature_name=cg09524639	feature_id[3704].value <= threshold=0.7802113592624664
Class: synovial sarcoma (SYSA)	
Rules_79	passed counts:2
node_0: feature_name=cg11915444	feature_id[2002].value > threshold=0.3402601182460785
node_10: feature_name=cg26016985	feature_id[4407].value > threshold=0.6493876278400421
node_16: feature_name=cg23157618	feature_id[5511].value > threshold=0.5759606957435608
node_20: feature_name=cg10480329	feature_id[1439].value > threshold=0.5518321692943573
node_48: feature_name=cg17843418	feature_id[4868].value > threshold=0.34969255328178406
node_52: feature_name=cg24407065	feature_id[4522].value > threshold=0.5364363789558411

node_56: feature_name=cg07281938	feature_id[249].value > threshold=0.7506992518901825
node_78: feature_name=cg21759907	feature_id[1650].value > threshold=0.46823520958423615
node_98: feature_name=cg10886334	feature_id[3975].value > threshold=0.43698127567768097
node_102: feature_name=cg09373983	feature_id[5025].value > threshold=0.5575815737247467
node_106: feature_name=cg12948116	feature_id[5360].value <= threshold=0.5658791363239288
node_107: feature_name=cg21906519	feature_id[5251].value > threshold=0.6089984774589539
node_111: feature_name=cg17537493	feature_id[4217].value > threshold=0.5825372636318207
node_117: feature_name=cg15720017	feature_id[4443].value > threshold=0.7009969055652618
node_119: feature_name=cg06038180	feature_id[3480].value > threshold=0.4583848565816879
node_121: feature_name=cg08619651	feature_id[4882].value <= threshold=0.2105495035648346
node_122: feature_name=cg01014262	feature_id[1911].value <= threshold=0.5327437818050385
node_123: feature_name=cg16863382	feature_id[2922].value > threshold=0.7200668156147003
node_133: feature_name=cg06989443	feature_id[2327].value > threshold=0.6579365730285645
node_355: feature_name=cg27470978	feature_id[4804].value > threshold=0.6264137625694275
node_361: feature_name=cg20252837	feature_id[420].value > threshold=0.53105828166008
node_363: feature_name=cg27437304	feature_id[1051].value > threshold=0.7641371190547943
node_365: feature_name=cg08215532	feature_id[3203].value <= threshold=0.45234426856040955
node_366: feature_name=cg12486498	feature_id[1977].value <= threshold=0.237603098154068
Class: rhabdomyosarcoma (RMS)	
Rules_80	passed counts:2
node_0: feature_name=cg11915444	feature_id[2002].value > threshold=0.3402601182460785
node_10: feature_name=cg26016985	feature_id[4407].value > threshold=0.6493876278400421

node_16: feature_name=cg23157618	feature_id[5511].value > threshold=0.5759606957435608
node_20: feature_name=cg10480329	feature_id[1439].value > threshold=0.5518321692943573
node_48: feature_name=cg17843418	feature_id[4868].value > threshold=0.34969255328178406
node_52: feature_name=cg24407065	feature_id[4522].value > threshold=0.5364363789558411
node_56: feature_name=cg07281938	feature_id[249].value > threshold=0.7506992518901825
node_78: feature_name=cg21759907	feature_id[1650].value > threshold=0.46823520958423615
node_98: feature_name=cg10886334	feature_id[3975].value > threshold=0.43698127567768097
node_102: feature_name=cg09373983	feature_id[5025].value > threshold=0.5575815737247467
node_106: feature_name=cg12948116	feature_id[5360].value <= threshold=0.5658791363239288
node_107: feature_name=cg21906519	feature_id[5251].value > threshold=0.6089984774589539
node_111: feature_name=cg17537493	feature_id[4217].value > threshold=0.5825372636318207
node_117: feature_name=cg15720017	feature_id[4443].value > threshold=0.7009969055652618
node_119: feature_name=cg06038180	feature_id[3480].value > threshold=0.4583848565816879
node_121: feature_name=cg08619651	feature_id[4882].value <= threshold=0.2105495035648346
node_122: feature_name=cg01014262	feature_id[1911].value <= threshold=0.5327437818050385
node_123: feature_name=cg16863382	feature_id[2922].value > threshold=0.7200668156147003
node_133: feature_name=cg06989443	feature_id[2327].value > threshold=0.6579365730285645
node_355: feature_name=cg27470978	feature_id[4804].value <= threshold=0.6264137625694275
node_356: feature_name=cg26964426	feature_id[2027].value <= threshold=0.665222555398941
node_357: feature_name=cg22427797	feature_id[1292].value > threshold=0.734585702419281
Class: high-grade endometrial stromal sarcoma (ESS (HG))	

Rules_81	passed counts:2
node_0: feature_name=cg11915444	feature_id[2002].value > threshold=0.3402601182460785
node_10: feature_name=cg26016985	feature_id[4407].value > threshold=0.6493876278400421
node_16: feature_name=cg23157618	feature_id[5511].value > threshold=0.5759606957435608
node_20: feature_name=cg10480329	feature_id[1439].value > threshold=0.5518321692943573
node_48: feature_name=cg17843418	feature_id[4868].value > threshold=0.34969255328178406
node_52: feature_name=cg24407065	feature_id[4522].value > threshold=0.5364363789558411
node_56: feature_name=cg07281938	feature_id[249].value > threshold=0.7506992518901825
node_78: feature_name=cg21759907	feature_id[1650].value > threshold=0.46823520958423615
node_98: feature_name=cg10886334	feature_id[3975].value > threshold=0.43698127567768097
node_102: feature_name=cg09373983	feature_id[5025].value > threshold=0.5575815737247467
node_106: feature_name=cg12948116	feature_id[5360].value <= threshold=0.5658791363239288
node_107: feature_name=cg21906519	feature_id[5251].value > threshold=0.6089984774589539
node_111: feature_name=cg17537493	feature_id[4217].value > threshold=0.5825372636318207
node_117: feature_name=cg15720017	feature_id[4443].value > threshold=0.7009969055652618
node_119: feature_name=cg06038180	feature_id[3480].value > threshold=0.4583848565816879
node_121: feature_name=cg08619651	feature_id[4882].value <= threshold=0.2105495035648346
node_122: feature_name=cg01014262	feature_id[1911].value <= threshold=0.5327437818050385
node_123: feature_name=cg16863382	feature_id[2922].value > threshold=0.7200668156147003
node_133: feature_name=cg06989443	feature_id[2327].value > threshold=0.6579365730285645
node_355: feature_name=cg27470978	feature_id[4804].value <= threshold=0.6264137625694275
node_356: feature_name=cg26964426	feature_id[2027].value <= threshold=0.665222555398941

node_357: feature_name=cg22427797	feature_id[1292].value <= threshold=0.734585702419281
Class: sarcoma (SARC)	
Rules_82	passed counts:2
node_0: feature_name=cg11915444	feature_id[2002].value > threshold=0.3402601182460785
node_10: feature_name=cg26016985	feature_id[4407].value > threshold=0.6493876278400421
node_16: feature_name=cg23157618	feature_id[5511].value > threshold=0.5759606957435608
node_20: feature_name=cg10480329	feature_id[1439].value > threshold=0.5518321692943573
node_48: feature_name=cg17843418	feature_id[4868].value > threshold=0.34969255328178406
node_52: feature_name=cg24407065	feature_id[4522].value > threshold=0.5364363789558411
node_56: feature_name=cg07281938	feature_id[249].value > threshold=0.7506992518901825
node_78: feature_name=cg21759907	feature_id[1650].value > threshold=0.46823520958423615
node_98: feature_name=cg10886334	feature_id[3975].value > threshold=0.43698127567768097
node_102: feature_name=cg09373983	feature_id[5025].value > threshold=0.5575815737247467
node_106: feature_name=cg12948116	feature_id[5360].value <= threshold=0.5658791363239288
node_107: feature_name=cg21906519	feature_id[5251].value > threshold=0.6089984774589539
node_111: feature_name=cg17537493	feature_id[4217].value > threshold=0.5825372636318207
node_117: feature_name=cg15720017	feature_id[4443].value > threshold=0.7009969055652618
node_119: feature_name=cg06038180	feature_id[3480].value > threshold=0.4583848565816879
node_121: feature_name=cg08619651	feature_id[4882].value <= threshold=0.2105495035648346
node_122: feature_name=cg01014262	feature_id[1911].value <= threshold=0.5327437818050385
node_123: feature_name=cg16863382	feature_id[2922].value > threshold=0.7200668156147003
node_133: feature_name=cg06989443	feature_id[2327].value <= threshold=0.6579365730285645

node_134: feature_name=cg07344990	feature_id[2398].value > threshold=0.5562876164913177
node_138: feature_name=cg02966841	feature_id[2199].value > threshold=0.7744501233100891
node_204: feature_name=cg12120430	feature_id[3727].value > threshold=0.5775820016860962
node_206: feature_name=cg18386876	feature_id[4066].value > threshold=0.6847257018089294
node_208: feature_name=cg02053092	feature_id[3610].value > threshold=0.39507485926151276
node_210: feature_name=cg10298992	feature_id[3603].value > threshold=0.5707973837852478
node_228: feature_name=cg03840920	feature_id[4524].value > threshold=0.5365143716335297
node_234: feature_name=cg25364972	feature_id[1792].value > threshold=0.2418053299188614
node_238: feature_name=cg23485307	feature_id[221].value > threshold=0.14884746074676514
node_242: feature_name=cg04873169	feature_id[4696].value > threshold=0.6058756709098816
node_244: feature_name=cg17009731	feature_id[3218].value > threshold=0.6602252125740051
node_246: feature_name=cg18253910	feature_id[4458].value > threshold=0.6904322504997253
node_250: feature_name=cg04774476	feature_id[1257].value > threshold=0.5675267577171326
node_256: feature_name=cg19259111	feature_id[63].value > threshold=0.40771469473838806
node_264: feature_name=cg21483216	feature_id[5253].value > threshold=0.6332719326019287
node_266: feature_name=cg04026354	feature_id[3502].value > threshold=0.42332448065280914
node_336: feature_name=cg05824594	feature_id[1431].value <= threshold=0.629401296377182
node_337: feature_name=cg18475969	feature_id[2117].value <= threshold=0.3116971254348755
node_338: feature_name=cg19494591	feature_id[2196].value > threshold=0.7268106639385223
node_340: feature_name=cg09075515	feature_id[1529].value > threshold=0.5144184827804565
node_342: feature_name=cg09322899	feature_id[2281].value > threshold=0.9401540160179138
Class: Kaposi sarcoma (KS)	

Rules_83	passed counts:2
node_0: feature_name=cg11915444	feature_id[2002].value > threshold=0.3402601182460785
node_10: feature_name=cg26016985	feature_id[4407].value > threshold=0.6493876278400421
node_16: feature_name=cg23157618	feature_id[5511].value > threshold=0.5759606957435608
node_20: feature_name=cg10480329	feature_id[1439].value > threshold=0.5518321692943573
node_48: feature_name=cg17843418	feature_id[4868].value > threshold=0.34969255328178406
node_52: feature_name=cg24407065	feature_id[4522].value > threshold=0.5364363789558411
node_56: feature_name=cg07281938	feature_id[249].value > threshold=0.7506992518901825
node_78: feature_name=cg21759907	feature_id[1650].value > threshold=0.46823520958423615
node_98: feature_name=cg10886334	feature_id[3975].value > threshold=0.43698127567768097
node_102: feature_name=cg09373983	feature_id[5025].value > threshold=0.5575815737247467
node_106: feature_name=cg12948116	feature_id[5360].value <= threshold=0.5658791363239288
node_107: feature_name=cg21906519	feature_id[5251].value > threshold=0.6089984774589539
node_111: feature_name=cg17537493	feature_id[4217].value > threshold=0.5825372636318207
node_117: feature_name=cg15720017	feature_id[4443].value > threshold=0.7009969055652618
node_119: feature_name=cg06038180	feature_id[3480].value > threshold=0.4583848565816879
node_121: feature_name=cg08619651	feature_id[4882].value <= threshold=0.2105495035648346
node_122: feature_name=cg01014262	feature_id[1911].value <= threshold=0.5327437818050385
node_123: feature_name=cg16863382	feature_id[2922].value > threshold=0.7200668156147003
node_133: feature_name=cg06989443	feature_id[2327].value <= threshold=0.6579365730285645
node_134: feature_name=cg07344990	feature_id[2398].value > threshold=0.5562876164913177

node_138: feature_name=cg02966841	feature_id[2199].value > threshold=0.7744501233100891
node_204: feature_name=cg12120430	feature_id[3727].value > threshold=0.5775820016860962
node_206: feature_name=cg18386876	feature_id[4066].value > threshold=0.6847257018089294
node_208: feature_name=cg02053092	feature_id[3610].value > threshold=0.39507485926151276
node_210: feature_name=cg10298992	feature_id[3603].value > threshold=0.5707973837852478
node_228: feature_name=cg03840920	feature_id[4524].value > threshold=0.5365143716335297
node_234: feature_name=cg25364972	feature_id[1792].value > threshold=0.2418053299188614
node_238: feature_name=cg23485307	feature_id[221].value > threshold=0.14884746074676514
node_242: feature_name=cg04873169	feature_id[4696].value > threshold=0.6058756709098816
node_244: feature_name=cg17009731	feature_id[3218].value > threshold=0.6602252125740051
node_246: feature_name=cg18253910	feature_id[4458].value > threshold=0.6904322504997253
node_250: feature_name=cg04774476	feature_id[1257].value > threshold=0.5675267577171326
node_256: feature_name=cg19259111	feature_id[63].value > threshold=0.40771469473838806
node_264: feature_name=cg21483216	feature_id[5253].value > threshold=0.6332719326019287
node_266: feature_name=cg04026354	feature_id[3502].value <= threshold=0.42332448065280914
node_267: feature_name=cg11444072	feature_id[116].value > threshold=0.4940422773361206
node_273: feature_name=cg08571020	feature_id[381].value > threshold=0.4616774618625641
node_275: feature_name=cg08331427	feature_id[2081].value > threshold=0.5443233847618103
node_281: feature_name=cg00216961	feature_id[945].value > threshold=0.4166988283395767
node_333: feature_name=cg23366832	feature_id[2439].value <= threshold=0.8551825881004333
Class: myositis proliferans (MP)	
Rules_84	passed counts:2

node_0: feature_name=cg11915444	feature_id[2002].value > threshold=0.3402601182460785
node_10: feature_name=cg26016985	feature_id[4407].value > threshold=0.6493876278400421
node_16: feature_name=cg23157618	feature_id[5511].value > threshold=0.5759606957435608
node_20: feature_name=cg10480329	feature_id[1439].value > threshold=0.5518321692943573
node_48: feature_name=cg17843418	feature_id[4868].value > threshold=0.34969255328178406
node_52: feature_name=cg24407065	feature_id[4522].value > threshold=0.5364363789558411
node_56: feature_name=cg07281938	feature_id[249].value > threshold=0.7506992518901825
node_78: feature_name=cg21759907	feature_id[1650].value > threshold=0.46823520958423615
node_98: feature_name=cg10886334	feature_id[3975].value > threshold=0.43698127567768097
node_102: feature_name=cg09373983	feature_id[5025].value > threshold=0.5575815737247467
node_106: feature_name=cg12948116	feature_id[5360].value <= threshold=0.5658791363239288
node_107: feature_name=cg21906519	feature_id[5251].value > threshold=0.6089984774589539
node_111: feature_name=cg17537493	feature_id[4217].value > threshold=0.5825372636318207
node_117: feature_name=cg15720017	feature_id[4443].value > threshold=0.7009969055652618
node_119: feature_name=cg06038180	feature_id[3480].value > threshold=0.4583848565816879
node_121: feature_name=cg08619651	feature_id[4882].value <= threshold=0.2105495035648346
node_122: feature_name=cg01014262	feature_id[1911].value <= threshold=0.5327437818050385
node_123: feature_name=cg16863382	feature_id[2922].value > threshold=0.7200668156147003
node_133: feature_name=cg06989443	feature_id[2327].value <= threshold=0.6579365730285645
node_134: feature_name=cg07344990	feature_id[2398].value > threshold=0.5562876164913177
node_138: feature_name=cg02966841	feature_id[2199].value > threshold=0.7744501233100891
node_204: feature_name=cg12120430	feature_id[3727].value > threshold=0.5775820016860962

node_206: feature_name=cg18386876	feature_id[4066].value > threshold=0.6847257018089294
node_208: feature_name=cg02053092	feature_id[3610].value > threshold=0.39507485926151276
node_210: feature_name=cg10298992	feature_id[3603].value > threshold=0.5707973837852478
node_228: feature_name=cg03840920	feature_id[4524].value > threshold=0.5365143716335297
node_234: feature_name=cg25364972	feature_id[1792].value > threshold=0.2418053299188614
node_238: feature_name=cg23485307	feature_id[221].value > threshold=0.14884746074676514
node_242: feature_name=cg04873169	feature_id[4696].value > threshold=0.6058756709098816
node_244: feature_name=cg17009731	feature_id[3218].value > threshold=0.6602252125740051
node_246: feature_name=cg18253910	feature_id[4458].value > threshold=0.6904322504997253
node_250: feature_name=cg04774476	feature_id[1257].value > threshold=0.5675267577171326
node_256: feature_name=cg19259111	feature_id[63].value > threshold=0.40771469473838806
node_264: feature_name=cg21483216	feature_id[5253].value > threshold=0.6332719326019287
node_266: feature_name=cg04026354	feature_id[3502].value <= threshold=0.42332448065280914
node_267: feature_name=cg11444072	feature_id[116].value > threshold=0.4940422773361206
node_273: feature_name=cg08571020	feature_id[381].value > threshold=0.4616774618625641
node_275: feature_name=cg08331427	feature_id[2081].value > threshold=0.5443233847618103
node_281: feature_name=cg00216961	feature_id[945].value <= threshold=0.4166988283395767
node_282: feature_name=cg04716580	feature_id[1390].value > threshold=0.4204266667366028
node_286: feature_name=cg07891483	feature_id[1877].value > threshold=0.829958975315094
node_288: feature_name=cg01475325	feature_id[3969].value > threshold=0.45323844254016876
node_292: feature_name=cg19702397	feature_id[2066].value > threshold=0.3536848723888397
node_294: feature_name=cg21936959	feature_id[595].value > threshold=0.5567200481891632

node_308: feature_name=cg18645642	feature_id[262].value > threshold=0.42608243227005005
node_310: feature_name=cg27351239	feature_id[1874].value <= threshold=0.6790103316307068
node_311: feature_name=cg13107973	feature_id[1326].value <= threshold=0.6562457382678986
node_312: feature_name=cg10024583	feature_id[1175].value <= threshold=0.518094539642334
node_313: feature_name=cg18259342	feature_id[4480].value > threshold=0.19059929996728897
node_315: feature_name=cg10979364	feature_id[992].value > threshold=0.44472505152225494
node_317: feature_name=cg14406727	feature_id[815].value > threshold=0.36684730648994446
node_319: feature_name=cg24926689	feature_id[2976].value > threshold=0.822247177362442
node_321: feature_name=cg11535638	feature_id[2812].value > threshold=0.7990793883800507
Class: Kaposi sarcoma (KS)	
Rules_85	passed counts:2
node_0: feature_name=cg11915444	feature_id[2002].value > threshold=0.3402601182460785
node_10: feature_name=cg26016985	feature_id[4407].value > threshold=0.6493876278400421
node_16: feature_name=cg23157618	feature_id[5511].value > threshold=0.5759606957435608
node_20: feature_name=cg10480329	feature_id[1439].value > threshold=0.5518321692943573
node_48: feature_name=cg17843418	feature_id[4868].value > threshold=0.34969255328178406
node_52: feature_name=cg24407065	feature_id[4522].value > threshold=0.5364363789558411
node_56: feature_name=cg07281938	feature_id[249].value > threshold=0.7506992518901825
node_78: feature_name=cg21759907	feature_id[1650].value > threshold=0.46823520958423615
node_98: feature_name=cg10886334	feature_id[3975].value > threshold=0.43698127567768097
node_102: feature_name=cg09373983	feature_id[5025].value > threshold=0.5575815737247467
node_106: feature_name=cg12948116	feature_id[5360].value <= threshold=0.5658791363239288

node_107: feature_name=cg21906519	feature_id[5251].value > threshold=0.6089984774589539
node_111: feature_name=cg17537493	feature_id[4217].value > threshold=0.5825372636318207
node_117: feature_name=cg15720017	feature_id[4443].value > threshold=0.7009969055652618
node_119: feature_name=cg06038180	feature_id[3480].value > threshold=0.4583848565816879
node_121: feature_name=cg08619651	feature_id[4882].value <= threshold=0.2105495035648346
node_122: feature_name=cg01014262	feature_id[1911].value <= threshold=0.5327437818050385
node_123: feature_name=cg16863382	feature_id[2922].value > threshold=0.7200668156147003
node_133: feature_name=cg06989443	feature_id[2327].value <= threshold=0.6579365730285645
node_134: feature_name=cg07344990	feature_id[2398].value > threshold=0.5562876164913177
node_138: feature_name=cg02966841	feature_id[2199].value > threshold=0.7744501233100891
node_204: feature_name=cg12120430	feature_id[3727].value > threshold=0.5775820016860962
node_206: feature_name=cg18386876	feature_id[4066].value > threshold=0.6847257018089294
node_208: feature_name=cg02053092	feature_id[3610].value > threshold=0.39507485926151276
node_210: feature_name=cg10298992	feature_id[3603].value > threshold=0.5707973837852478
node_228: feature_name=cg03840920	feature_id[4524].value > threshold=0.5365143716335297
node_234: feature_name=cg25364972	feature_id[1792].value > threshold=0.2418053299188614
node_238: feature_name=cg23485307	feature_id[221].value > threshold=0.14884746074676514
node_242: feature_name=cg04873169	feature_id[4696].value > threshold=0.6058756709098816
node_244: feature_name=cg17009731	feature_id[3218].value > threshold=0.6602252125740051
node_246: feature_name=cg18253910	feature_id[4458].value > threshold=0.6904322504997253
node_250: feature_name=cg04774476	feature_id[1257].value > threshold=0.5675267577171326
node_256: feature_name=cg19259111	feature_id[63].value > threshold=0.40771469473838806

node_264: feature_name=cg21483216	feature_id[5253].value > threshold=0.6332719326019287
node_266: feature_name=cg04026354	feature_id[3502].value <= threshold=0.42332448065280914
node_267: feature_name=cg11444072	feature_id[116].value > threshold=0.4940422773361206
node_273: feature_name=cg08571020	feature_id[381].value > threshold=0.4616774618625641
node_275: feature_name=cg08331427	feature_id[2081].value > threshold=0.5443233847618103
node_281: feature_name=cg00216961	feature_id[945].value <= threshold=0.4166988283395767
node_282: feature_name=cg04716580	feature_id[1390].value > threshold=0.4204266667366028
node_286: feature_name=cg07891483	feature_id[1877].value > threshold=0.829958975315094
node_288: feature_name=cg01475325	feature_id[3969].value > threshold=0.45323844254016876
node_292: feature_name=cg19702397	feature_id[2066].value > threshold=0.3536848723888397
node_294: feature_name=cg21936959	feature_id[595].value > threshold=0.5567200481891632
node_308: feature_name=cg18645642	feature_id[262].value > threshold=0.42608243227005005
node_310: feature_name=cg27351239	feature_id[1874].value <= threshold=0.6790103316307068
node_311: feature_name=cg13107973	feature_id[1326].value <= threshold=0.6562457382678986
node_312: feature_name=cg10024583	feature_id[1175].value <= threshold=0.518094539642334
node_313: feature_name=cg18259342	feature_id[4480].value > threshold=0.19059929996728897
node_315: feature_name=cg10979364	feature_id[992].value > threshold=0.44472505152225494
node_317: feature_name=cg14406727	feature_id[815].value > threshold=0.36684730648994446
node_319: feature_name=cg24926689	feature_id[2976].value <= threshold=0.822247177362442
Class: clear cell chondrosarcoma (CSA (CC))	
Rules_86	passed counts:2
node_0: feature_name=cg11915444	feature_id[2002].value > threshold=0.3402601182460785

node_10: feature_name=cg26016985	feature_id[4407].value > threshold=0.6493876278400421
node_16: feature_name=cg23157618	feature_id[5511].value > threshold=0.5759606957435608
node_20: feature_name=cg10480329	feature_id[1439].value > threshold=0.5518321692943573
node_48: feature_name=cg17843418	feature_id[4868].value > threshold=0.34969255328178406
node_52: feature_name=cg24407065	feature_id[4522].value > threshold=0.5364363789558411
node_56: feature_name=cg07281938	feature_id[249].value > threshold=0.7506992518901825
node_78: feature_name=cg21759907	feature_id[1650].value > threshold=0.46823520958423615
node_98: feature_name=cg10886334	feature_id[3975].value > threshold=0.43698127567768097
node_102: feature_name=cg09373983	feature_id[5025].value > threshold=0.5575815737247467
node_106: feature_name=cg12948116	feature_id[5360].value <= threshold=0.5658791363239288
node_107: feature_name=cg21906519	feature_id[5251].value > threshold=0.6089984774589539
node_111: feature_name=cg17537493	feature_id[4217].value > threshold=0.5825372636318207
node_117: feature_name=cg15720017	feature_id[4443].value > threshold=0.7009969055652618
node_119: feature_name=cg06038180	feature_id[3480].value > threshold=0.4583848565816879
node_121: feature_name=cg08619651	feature_id[4882].value <= threshold=0.2105495035648346
node_122: feature_name=cg01014262	feature_id[1911].value <= threshold=0.5327437818050385
node_123: feature_name=cg16863382	feature_id[2922].value > threshold=0.7200668156147003
node_133: feature_name=cg06989443	feature_id[2327].value <= threshold=0.6579365730285645
node_134: feature_name=cg07344990	feature_id[2398].value > threshold=0.5562876164913177
node_138: feature_name=cg02966841	feature_id[2199].value > threshold=0.7744501233100891
node_204: feature_name=cg12120430	feature_id[3727].value > threshold=0.5775820016860962
node_206: feature_name=cg18386876	feature_id[4066].value > threshold=0.6847257018089294

node_208: feature_name=cg02053092	feature_id[3610].value > threshold=0.39507485926151276
node_210: feature_name=cg10298992	feature_id[3603].value > threshold=0.5707973837852478
node_228: feature_name=cg03840920	feature_id[4524].value > threshold=0.5365143716335297
node_234: feature_name=cg25364972	feature_id[1792].value > threshold=0.2418053299188614
node_238: feature_name=cg23485307	feature_id[221].value > threshold=0.14884746074676514
node_242: feature_name=cg04873169	feature_id[4696].value > threshold=0.6058756709098816
node_244: feature_name=cg17009731	feature_id[3218].value > threshold=0.6602252125740051
node_246: feature_name=cg18253910	feature_id[4458].value > threshold=0.6904322504997253
node_250: feature_name=cg04774476	feature_id[1257].value > threshold=0.5675267577171326
node_256: feature_name=cg19259111	feature_id[63].value > threshold=0.40771469473838806
node_264: feature_name=cg21483216	feature_id[5253].value > threshold=0.6332719326019287
node_266: feature_name=cg04026354	feature_id[3502].value <= threshold=0.42332448065280914
node_267: feature_name=cg11444072	feature_id[116].value > threshold=0.4940422773361206
node_273: feature_name=cg08571020	feature_id[381].value > threshold=0.4616774618625641
node_275: feature_name=cg08331427	feature_id[2081].value > threshold=0.5443233847618103
node_281: feature_name=cg00216961	feature_id[945].value <= threshold=0.4166988283395767
node_282: feature_name=cg04716580	feature_id[1390].value > threshold=0.4204266667366028
node_286: feature_name=cg07891483	feature_id[1877].value > threshold=0.829958975315094
node_288: feature_name=cg01475325	feature_id[3969].value > threshold=0.45323844254016876
node_292: feature_name=cg19702397	feature_id[2066].value > threshold=0.3536848723888397
node_294: feature_name=cg21936959	feature_id[595].value > threshold=0.5567200481891632
node_308: feature_name=cg18645642	feature_id[262].value > threshold=0.42608243227005005

node_310: feature_name=cg27351239	feature_id[1874].value <= threshold=0.6790103316307068
node_311: feature_name=cg13107973	feature_id[1326].value <= threshold=0.6562457382678986
node_312: feature_name=cg10024583	feature_id[1175].value <= threshold=0.518094539642334
node_313: feature_name=cg18259342	feature_id[4480].value > threshold=0.19059929996728897
node_315: feature_name=cg10979364	feature_id[992].value > threshold=0.44472505152225494
node_317: feature_name=cg14406727	feature_id[815].value <= threshold=0.36684730648994446
Class: embryonal rhabdomyosarcoma (RMS (EMB))	
Rules_87	passed counts:2
node_0: feature_name=cg11915444	feature_id[2002].value > threshold=0.3402601182460785
node_10: feature_name=cg26016985	feature_id[4407].value > threshold=0.6493876278400421
node_16: feature_name=cg23157618	feature_id[5511].value > threshold=0.5759606957435608
node_20: feature_name=cg10480329	feature_id[1439].value > threshold=0.5518321692943573
node_48: feature_name=cg17843418	feature_id[4868].value > threshold=0.34969255328178406
node_52: feature_name=cg24407065	feature_id[4522].value > threshold=0.5364363789558411
node_56: feature_name=cg07281938	feature_id[249].value > threshold=0.7506992518901825
node_78: feature_name=cg21759907	feature_id[1650].value > threshold=0.46823520958423615
node_98: feature_name=cg10886334	feature_id[3975].value > threshold=0.43698127567768097
node_102: feature_name=cg09373983	feature_id[5025].value > threshold=0.5575815737247467
node_106: feature_name=cg12948116	feature_id[5360].value <= threshold=0.5658791363239288
node_107: feature_name=cg21906519	feature_id[5251].value > threshold=0.6089984774589539
node_111: feature_name=cg17537493	feature_id[4217].value > threshold=0.5825372636318207

node_117: feature_name=cg15720017	feature_id[4443].value > threshold=0.7009969055652618
node_119: feature_name=cg06038180	feature_id[3480].value > threshold=0.4583848565816879
node_121: feature_name=cg08619651	feature_id[4882].value <= threshold=0.2105495035648346
node_122: feature_name=cg01014262	feature_id[1911].value <= threshold=0.5327437818050385
node_123: feature_name=cg16863382	feature_id[2922].value > threshold=0.7200668156147003
node_133: feature_name=cg06989443	feature_id[2327].value <= threshold=0.6579365730285645
node_134: feature_name=cg07344990	feature_id[2398].value > threshold=0.5562876164913177
node_138: feature_name=cg02966841	feature_id[2199].value > threshold=0.7744501233100891
node_204: feature_name=cg12120430	feature_id[3727].value > threshold=0.5775820016860962
node_206: feature_name=cg18386876	feature_id[4066].value > threshold=0.6847257018089294
node_208: feature_name=cg02053092	feature_id[3610].value > threshold=0.39507485926151276
node_210: feature_name=cg10298992	feature_id[3603].value > threshold=0.5707973837852478
node_228: feature_name=cg03840920	feature_id[4524].value > threshold=0.5365143716335297
node_234: feature_name=cg25364972	feature_id[1792].value > threshold=0.2418053299188614
node_238: feature_name=cg23485307	feature_id[221].value > threshold=0.14884746074676514
node_242: feature_name=cg04873169	feature_id[4696].value > threshold=0.6058756709098816
node_244: feature_name=cg17009731	feature_id[3218].value > threshold=0.6602252125740051
node_246: feature_name=cg18253910	feature_id[4458].value > threshold=0.6904322504997253
node_250: feature_name=cg04774476	feature_id[1257].value > threshold=0.5675267577171326
node_256: feature_name=cg19259111	feature_id[63].value > threshold=0.40771469473838806
node_264: feature_name=cg21483216	feature_id[5253].value > threshold=0.6332719326019287
node_266: feature_name=cg04026354	feature_id[3502].value <= threshold=0.42332448065280914

node_267: feature_name=cg11444072	feature_id[116].value > threshold=0.4940422773361206
node_273: feature_name=cg08571020	feature_id[381].value > threshold=0.4616774618625641
node_275: feature_name=cg08331427	feature_id[2081].value > threshold=0.5443233847618103
node_281: feature_name=cg00216961	feature_id[945].value <= threshold=0.4166988283395767
node_282: feature_name=cg04716580	feature_id[1390].value > threshold=0.4204266667366028
node_286: feature_name=cg07891483	feature_id[1877].value > threshold=0.829958975315094
node_288: feature_name=cg01475325	feature_id[3969].value > threshold=0.45323844254016876
node_292: feature_name=cg19702397	feature_id[2066].value > threshold=0.3536848723888397
node_294: feature_name=cg21936959	feature_id[595].value > threshold=0.5567200481891632
node_308: feature_name=cg18645642	feature_id[262].value > threshold=0.42608243227005005
node_310: feature_name=cg27351239	feature_id[1874].value <= threshold=0.6790103316307068
node_311: feature_name=cg13107973	feature_id[1326].value <= threshold=0.6562457382678986
node_312: feature_name=cg10024583	feature_id[1175].value <= threshold=0.518094539642334
node_313: feature_name=cg18259342	feature_id[4480].value > threshold=0.19059929996728897
node_315: feature_name=cg10979364	feature_id[992].value <= threshold=0.44472505152225494
Class: malignant peripheral nerve sheath tumour (MPNST)	
Rules_88	passed counts:2
node_0: feature_name=cg11915444	feature_id[2002].value > threshold=0.3402601182460785
node_10: feature_name=cg26016985	feature_id[4407].value > threshold=0.6493876278400421
node_16: feature_name=cg23157618	feature_id[5511].value > threshold=0.5759606957435608
node_20: feature_name=cg10480329	feature_id[1439].value > threshold=0.5518321692943573

node_48: feature_name=cg17843418	feature_id[4868].value > threshold=0.34969255328178406
node_52: feature_name=cg24407065	feature_id[4522].value > threshold=0.5364363789558411
node_56: feature_name=cg07281938	feature_id[249].value > threshold=0.7506992518901825
node_78: feature_name=cg21759907	feature_id[1650].value > threshold=0.46823520958423615
node_98: feature_name=cg10886334	feature_id[3975].value > threshold=0.43698127567768097
node_102: feature_name=cg09373983	feature_id[5025].value > threshold=0.5575815737247467
node_106: feature_name=cg12948116	feature_id[5360].value <= threshold=0.5658791363239288
node_107: feature_name=cg21906519	feature_id[5251].value > threshold=0.6089984774589539
node_111: feature_name=cg17537493	feature_id[4217].value > threshold=0.5825372636318207
node_117: feature_name=cg15720017	feature_id[4443].value > threshold=0.7009969055652618
node_119: feature_name=cg06038180	feature_id[3480].value > threshold=0.4583848565816879
node_121: feature_name=cg08619651	feature_id[4882].value <= threshold=0.2105495035648346
node_122: feature_name=cg01014262	feature_id[1911].value <= threshold=0.5327437818050385
node_123: feature_name=cg16863382	feature_id[2922].value > threshold=0.7200668156147003
node_133: feature_name=cg06989443	feature_id[2327].value <= threshold=0.6579365730285645
node_134: feature_name=cg07344990	feature_id[2398].value > threshold=0.5562876164913177
node_138: feature_name=cg02966841	feature_id[2199].value > threshold=0.7744501233100891
node_204: feature_name=cg12120430	feature_id[3727].value > threshold=0.5775820016860962
node_206: feature_name=cg18386876	feature_id[4066].value > threshold=0.6847257018089294
node_208: feature_name=cg02053092	feature_id[3610].value > threshold=0.39507485926151276
node_210: feature_name=cg10298992	feature_id[3603].value > threshold=0.5707973837852478
node_228: feature_name=cg03840920	feature_id[4524].value > threshold=0.5365143716335297

node_234: feature_name=cg25364972	feature_id[1792].value > threshold=0.2418053299188614
node_238: feature_name=cg23485307	feature_id[221].value > threshold=0.14884746074676514
node_242: feature_name=cg04873169	feature_id[4696].value > threshold=0.6058756709098816
node_244: feature_name=cg17009731	feature_id[3218].value > threshold=0.6602252125740051
node_246: feature_name=cg18253910	feature_id[4458].value > threshold=0.6904322504997253
node_250: feature_name=cg04774476	feature_id[1257].value > threshold=0.5675267577171326
node_256: feature_name=cg19259111	feature_id[63].value > threshold=0.40771469473838806
node_264: feature_name=cg21483216	feature_id[5253].value > threshold=0.6332719326019287
node_266: feature_name=cg04026354	feature_id[3502].value <= threshold=0.42332448065280914
node_267: feature_name=cg11444072	feature_id[116].value > threshold=0.4940422773361206
node_273: feature_name=cg08571020	feature_id[381].value > threshold=0.4616774618625641
node_275: feature_name=cg08331427	feature_id[2081].value > threshold=0.5443233847618103
node_281: feature_name=cg00216961	feature_id[945].value <= threshold=0.4166988283395767
node_282: feature_name=cg04716580	feature_id[1390].value > threshold=0.4204266667366028
node_286: feature_name=cg07891483	feature_id[1877].value > threshold=0.829958975315094
node_288: feature_name=cg01475325	feature_id[3969].value > threshold=0.45323844254016876
node_292: feature_name=cg19702397	feature_id[2066].value > threshold=0.3536848723888397
node_294: feature_name=cg21936959	feature_id[595].value > threshold=0.5567200481891632
node_308: feature_name=cg18645642	feature_id[262].value > threshold=0.42608243227005005
node_310: feature_name=cg27351239	feature_id[1874].value <= threshold=0.6790103316307068
node_311: feature_name=cg13107973	feature_id[1326].value <= threshold=0.6562457382678986
node_312: feature_name=cg10024583	feature_id[1175].value <= threshold=0.518094539642334

node_313: feature_name=cg18259342	feature_id[4480].value <= threshold=0.19059929996728897
Class: Ewing sarcoma (EWING)	
Rules_89	passed counts:2
node_0: feature_name=cg11915444	feature_id[2002].value > threshold=0.3402601182460785
node_10: feature_name=cg26016985	feature_id[4407].value > threshold=0.6493876278400421
node_16: feature_name=cg23157618	feature_id[5511].value > threshold=0.5759606957435608
node_20: feature_name=cg10480329	feature_id[1439].value > threshold=0.5518321692943573
node_48: feature_name=cg17843418	feature_id[4868].value > threshold=0.34969255328178406
node_52: feature_name=cg24407065	feature_id[4522].value > threshold=0.5364363789558411
node_56: feature_name=cg07281938	feature_id[249].value > threshold=0.7506992518901825
node_78: feature_name=cg21759907	feature_id[1650].value > threshold=0.46823520958423615
node_98: feature_name=cg10886334	feature_id[3975].value > threshold=0.43698127567768097
node_102: feature_name=cg09373983	feature_id[5025].value > threshold=0.5575815737247467
node_106: feature_name=cg12948116	feature_id[5360].value <= threshold=0.5658791363239288
node_107: feature_name=cg21906519	feature_id[5251].value > threshold=0.6089984774589539
node_111: feature_name=cg17537493	feature_id[4217].value > threshold=0.5825372636318207
node_117: feature_name=cg15720017	feature_id[4443].value > threshold=0.7009969055652618
node_119: feature_name=cg06038180	feature_id[3480].value > threshold=0.4583848565816879
node_121: feature_name=cg08619651	feature_id[4882].value <= threshold=0.2105495035648346
node_122: feature_name=cg01014262	feature_id[1911].value <= threshold=0.5327437818050385
node_123: feature_name=cg16863382	feature_id[2922].value > threshold=0.7200668156147003
node_133: feature_name=cg06989443	feature_id[2327].value <= threshold=0.6579365730285645

node_134: feature_name=cg07344990	feature_id[2398].value > threshold=0.5562876164913177
node_138: feature_name=cg02966841	feature_id[2199].value > threshold=0.7744501233100891
node_204: feature_name=cg12120430	feature_id[3727].value > threshold=0.5775820016860962
node_206: feature_name=cg18386876	feature_id[4066].value > threshold=0.6847257018089294
node_208: feature_name=cg02053092	feature_id[3610].value > threshold=0.39507485926151276
node_210: feature_name=cg10298992	feature_id[3603].value > threshold=0.5707973837852478
node_228: feature_name=cg03840920	feature_id[4524].value > threshold=0.5365143716335297
node_234: feature_name=cg25364972	feature_id[1792].value > threshold=0.2418053299188614
node_238: feature_name=cg23485307	feature_id[221].value > threshold=0.14884746074676514
node_242: feature_name=cg04873169	feature_id[4696].value > threshold=0.6058756709098816
node_244: feature_name=cg17009731	feature_id[3218].value > threshold=0.6602252125740051
node_246: feature_name=cg18253910	feature_id[4458].value > threshold=0.6904322504997253
node_250: feature_name=cg04774476	feature_id[1257].value > threshold=0.5675267577171326
node_256: feature_name=cg19259111	feature_id[63].value > threshold=0.40771469473838806
node_264: feature_name=cg21483216	feature_id[5253].value > threshold=0.6332719326019287
node_266: feature_name=cg04026354	feature_id[3502].value <= threshold=0.42332448065280914
node_267: feature_name=cg11444072	feature_id[116].value > threshold=0.4940422773361206
node_273: feature_name=cg08571020	feature_id[381].value > threshold=0.4616774618625641
node_275: feature_name=cg08331427	feature_id[2081].value > threshold=0.5443233847618103
node_281: feature_name=cg00216961	feature_id[945].value <= threshold=0.4166988283395767
node_282: feature_name=cg04716580	feature_id[1390].value > threshold=0.4204266667366028
node_286: feature_name=cg07891483	feature_id[1877].value > threshold=0.829958975315094

node_288: feature_name=cg01475325	feature_id[3969].value > threshold=0.45323844254016876
node_292: feature_name=cg19702397	feature_id[2066].value > threshold=0.3536848723888397
node_294: feature_name=cg21936959	feature_id[595].value <= threshold=0.5567200481891632
node_295: feature_name=cg05251593	feature_id[515].value <= threshold=0.5597732663154602
node_296: feature_name=cg01343045	feature_id[1653].value > threshold=0.9222070276737213
Class: gastrointestinal stromal tumour (GIST)	
Rules_90	passed counts:2
node_0: feature_name=cg11915444	feature_id[2002].value > threshold=0.3402601182460785
node_10: feature_name=cg26016985	feature_id[4407].value > threshold=0.6493876278400421
node_16: feature_name=cg23157618	feature_id[5511].value > threshold=0.5759606957435608
node_20: feature_name=cg10480329	feature_id[1439].value > threshold=0.5518321692943573
node_48: feature_name=cg17843418	feature_id[4868].value > threshold=0.34969255328178406
node_52: feature_name=cg24407065	feature_id[4522].value > threshold=0.5364363789558411
node_56: feature_name=cg07281938	feature_id[249].value > threshold=0.7506992518901825
node_78: feature_name=cg21759907	feature_id[1650].value > threshold=0.46823520958423615
node_98: feature_name=cg10886334	feature_id[3975].value > threshold=0.43698127567768097
node_102: feature_name=cg09373983	feature_id[5025].value > threshold=0.5575815737247467
node_106: feature_name=cg12948116	feature_id[5360].value <= threshold=0.5658791363239288
node_107: feature_name=cg21906519	feature_id[5251].value > threshold=0.6089984774589539
node_111: feature_name=cg17537493	feature_id[4217].value > threshold=0.5825372636318207
node_117: feature_name=cg15720017	feature_id[4443].value > threshold=0.7009969055652618
node_119: feature_name=cg06038180	feature_id[3480].value > threshold=0.4583848565816879

node_121: feature_name=cg08619651	feature_id[4882].value <= threshold=0.2105495035648346
node_122: feature_name=cg01014262	feature_id[1911].value <= threshold=0.5327437818050385
node_123: feature_name=cg16863382	feature_id[2922].value > threshold=0.7200668156147003
node_133: feature_name=cg06989443	feature_id[2327].value <= threshold=0.6579365730285645
node_134: feature_name=cg07344990	feature_id[2398].value > threshold=0.5562876164913177
node_138: feature_name=cg02966841	feature_id[2199].value > threshold=0.7744501233100891
node_204: feature_name=cg12120430	feature_id[3727].value > threshold=0.5775820016860962
node_206: feature_name=cg18386876	feature_id[4066].value > threshold=0.6847257018089294
node_208: feature_name=cg02053092	feature_id[3610].value > threshold=0.39507485926151276
node_210: feature_name=cg10298992	feature_id[3603].value > threshold=0.5707973837852478
node_228: feature_name=cg03840920	feature_id[4524].value > threshold=0.5365143716335297
node_234: feature_name=cg25364972	feature_id[1792].value > threshold=0.2418053299188614
node_238: feature_name=cg23485307	feature_id[221].value > threshold=0.14884746074676514
node_242: feature_name=cg04873169	feature_id[4696].value > threshold=0.6058756709098816
node_244: feature_name=cg17009731	feature_id[3218].value > threshold=0.6602252125740051
node_246: feature_name=cg18253910	feature_id[4458].value > threshold=0.6904322504997253
node_250: feature_name=cg04774476	feature_id[1257].value > threshold=0.5675267577171326
node_256: feature_name=cg19259111	feature_id[63].value > threshold=0.40771469473838806
node_264: feature_name=cg21483216	feature_id[5253].value > threshold=0.6332719326019287
node_266: feature_name=cg04026354	feature_id[3502].value <= threshold=0.42332448065280914
node_267: feature_name=cg11444072	feature_id[116].value > threshold=0.4940422773361206
node_273: feature_name=cg08571020	feature_id[381].value > threshold=0.4616774618625641

node_275: feature_name=cg08331427	feature_id[2081].value > threshold=0.5443233847618103
node_281: feature_name=cg00216961	feature_id[945].value <= threshold=0.4166988283395767
node_282: feature_name=cg04716580	feature_id[1390].value > threshold=0.4204266667366028
node_286: feature_name=cg07891483	feature_id[1877].value > threshold=0.829958975315094
node_288: feature_name=cg01475325	feature_id[3969].value > threshold=0.45323844254016876
node_292: feature_name=cg19702397	feature_id[2066].value > threshold=0.3536848723888397
node_294: feature_name=cg21936959	feature_id[595].value <= threshold=0.5567200481891632
node_295: feature_name=cg05251593	feature_id[515].value <= threshold=0.5597732663154602
node_296: feature_name=cg01343045	feature_id[1653].value <= threshold=0.9222070276737213
node_297: feature_name=cg03701759	feature_id[3914].value > threshold=0.8587441444396973
Class: leiomyosarcoma (LMS)	
Rules_91	passed counts:2
node_0: feature_name=cg11915444	feature_id[2002].value > threshold=0.3402601182460785
node_10: feature_name=cg26016985	feature_id[4407].value > threshold=0.6493876278400421
node_16: feature_name=cg23157618	feature_id[5511].value > threshold=0.5759606957435608
node_20: feature_name=cg10480329	feature_id[1439].value > threshold=0.5518321692943573
node_48: feature_name=cg17843418	feature_id[4868].value > threshold=0.34969255328178406
node_52: feature_name=cg24407065	feature_id[4522].value > threshold=0.5364363789558411
node_56: feature_name=cg07281938	feature_id[249].value > threshold=0.7506992518901825
node_78: feature_name=cg21759907	feature_id[1650].value > threshold=0.46823520958423615
node_98: feature_name=cg10886334	feature_id[3975].value > threshold=0.43698127567768097
node_102: feature_name=cg09373983	feature_id[5025].value > threshold=0.5575815737247467

node_106: feature_name=cg12948116	feature_id[5360].value <= threshold=0.5658791363239288
node_107: feature_name=cg21906519	feature_id[5251].value > threshold=0.6089984774589539
node_111: feature_name=cg17537493	feature_id[4217].value > threshold=0.5825372636318207
node_117: feature_name=cg15720017	feature_id[4443].value > threshold=0.7009969055652618
node_119: feature_name=cg06038180	feature_id[3480].value > threshold=0.4583848565816879
node_121: feature_name=cg08619651	feature_id[4882].value <= threshold=0.2105495035648346
node_122: feature_name=cg01014262	feature_id[1911].value <= threshold=0.5327437818050385
node_123: feature_name=cg16863382	feature_id[2922].value > threshold=0.7200668156147003
node_133: feature_name=cg06989443	feature_id[2327].value <= threshold=0.6579365730285645
node_134: feature_name=cg07344990	feature_id[2398].value > threshold=0.5562876164913177
node_138: feature_name=cg02966841	feature_id[2199].value > threshold=0.7744501233100891
node_204: feature_name=cg12120430	feature_id[3727].value > threshold=0.5775820016860962
node_206: feature_name=cg18386876	feature_id[4066].value > threshold=0.6847257018089294
node_208: feature_name=cg02053092	feature_id[3610].value > threshold=0.39507485926151276
node_210: feature_name=cg10298992	feature_id[3603].value > threshold=0.5707973837852478
node_228: feature_name=cg03840920	feature_id[4524].value > threshold=0.5365143716335297
node_234: feature_name=cg25364972	feature_id[1792].value > threshold=0.2418053299188614
node_238: feature_name=cg23485307	feature_id[221].value > threshold=0.14884746074676514
node_242: feature_name=cg04873169	feature_id[4696].value > threshold=0.6058756709098816
node_244: feature_name=cg17009731	feature_id[3218].value > threshold=0.6602252125740051
node_246: feature_name=cg18253910	feature_id[4458].value > threshold=0.6904322504997253
node_250: feature_name=cg04774476	feature_id[1257].value > threshold=0.5675267577171326

node_256: feature_name=cg19259111	feature_id[63].value > threshold=0.40771469473838806
node_264: feature_name=cg21483216	feature_id[5253].value > threshold=0.6332719326019287
node_266: feature_name=cg04026354	feature_id[3502].value <= threshold=0.42332448065280914
node_267: feature_name=cg11444072	feature_id[116].value > threshold=0.4940422773361206
node_273: feature_name=cg08571020	feature_id[381].value > threshold=0.4616774618625641
node_275: feature_name=cg08331427	feature_id[2081].value > threshold=0.5443233847618103
node_281: feature_name=cg00216961	feature_id[945].value <= threshold=0.4166988283395767
node_282: feature_name=cg04716580	feature_id[1390].value > threshold=0.4204266667366028
node_286: feature_name=cg07891483	feature_id[1877].value > threshold=0.829958975315094
node_288: feature_name=cg01475325	feature_id[3969].value > threshold=0.45323844254016876
node_292: feature_name=cg19702397	feature_id[2066].value > threshold=0.3536848723888397
node_294: feature_name=cg21936959	feature_id[595].value <= threshold=0.5567200481891632
node_295: feature_name=cg05251593	feature_id[515].value <= threshold=0.5597732663154602
node_296: feature_name=cg01343045	feature_id[1653].value <= threshold=0.9222070276737213
node_297: feature_name=cg03701759	feature_id[3914].value <= threshold=0.8587441444396973
node_298: feature_name=cg12497581	feature_id[1864].value <= threshold=0.37188364565372467
Class: malignant peripheral nerve sheath tumour (MPNST)	
Rules_92	passed counts:2
node_0: feature_name=cg11915444	feature_id[2002].value > threshold=0.3402601182460785
node_10: feature_name=cg26016985	feature_id[4407].value > threshold=0.6493876278400421
node_16: feature_name=cg23157618	feature_id[5511].value > threshold=0.5759606957435608

node_20: feature_name=cg10480329	feature_id[1439].value > threshold=0.5518321692943573
node_48: feature_name=cg17843418	feature_id[4868].value > threshold=0.34969255328178406
node_52: feature_name=cg24407065	feature_id[4522].value > threshold=0.5364363789558411
node_56: feature_name=cg07281938	feature_id[249].value > threshold=0.7506992518901825
node_78: feature_name=cg21759907	feature_id[1650].value > threshold=0.46823520958423615
node_98: feature_name=cg10886334	feature_id[3975].value > threshold=0.43698127567768097
node_102: feature_name=cg09373983	feature_id[5025].value > threshold=0.5575815737247467
node_106: feature_name=cg12948116	feature_id[5360].value <= threshold=0.5658791363239288
node_107: feature_name=cg21906519	feature_id[5251].value > threshold=0.6089984774589539
node_111: feature_name=cg17537493	feature_id[4217].value > threshold=0.5825372636318207
node_117: feature_name=cg15720017	feature_id[4443].value > threshold=0.7009969055652618
node_119: feature_name=cg06038180	feature_id[3480].value > threshold=0.4583848565816879
node_121: feature_name=cg08619651	feature_id[4882].value <= threshold=0.2105495035648346
node_122: feature_name=cg01014262	feature_id[1911].value <= threshold=0.5327437818050385
node_123: feature_name=cg16863382	feature_id[2922].value > threshold=0.7200668156147003
node_133: feature_name=cg06989443	feature_id[2327].value <= threshold=0.6579365730285645
node_134: feature_name=cg07344990	feature_id[2398].value > threshold=0.5562876164913177
node_138: feature_name=cg02966841	feature_id[2199].value > threshold=0.7744501233100891
node_204: feature_name=cg12120430	feature_id[3727].value > threshold=0.5775820016860962
node_206: feature_name=cg18386876	feature_id[4066].value > threshold=0.6847257018089294
node_208: feature_name=cg02053092	feature_id[3610].value > threshold=0.39507485926151276
node_210: feature_name=cg10298992	feature_id[3603].value > threshold=0.5707973837852478

node_228: feature_name=cg03840920	feature_id[4524].value > threshold=0.5365143716335297
node_234: feature_name=cg25364972	feature_id[1792].value > threshold=0.2418053299188614
node_238: feature_name=cg23485307	feature_id[221].value > threshold=0.14884746074676514
node_242: feature_name=cg04873169	feature_id[4696].value > threshold=0.6058756709098816
node_244: feature_name=cg17009731	feature_id[3218].value > threshold=0.6602252125740051
node_246: feature_name=cg18253910	feature_id[4458].value > threshold=0.6904322504997253
node_250: feature_name=cg04774476	feature_id[1257].value <= threshold=0.5675267577171326
node_251: feature_name=cg08276755	feature_id[769].value <= threshold=0.5270942002534866
node_252: feature_name=cg09230938	feature_id[163].value > threshold=0.465945765376091
Class: chondroblastoma (CB)	
Rules_93	passed counts:2
node_0: feature_name=cg11915444	feature_id[2002].value > threshold=0.3402601182460785
node_10: feature_name=cg26016985	feature_id[4407].value > threshold=0.6493876278400421
node_16: feature_name=cg23157618	feature_id[5511].value > threshold=0.5759606957435608
node_20: feature_name=cg10480329	feature_id[1439].value > threshold=0.5518321692943573
node_48: feature_name=cg17843418	feature_id[4868].value > threshold=0.34969255328178406
node_52: feature_name=cg24407065	feature_id[4522].value > threshold=0.5364363789558411
node_56: feature_name=cg07281938	feature_id[249].value > threshold=0.7506992518901825
node_78: feature_name=cg21759907	feature_id[1650].value > threshold=0.46823520958423615
node_98: feature_name=cg10886334	feature_id[3975].value > threshold=0.43698127567768097
node_102: feature_name=cg09373983	feature_id[5025].value > threshold=0.5575815737247467
node_106: feature_name=cg12948116	feature_id[5360].value <= threshold=0.5658791363239288

node_107: feature_name=cg21906519	feature_id[5251].value > threshold=0.6089984774589539
node_111: feature_name=cg17537493	feature_id[4217].value > threshold=0.5825372636318207
node_117: feature_name=cg15720017	feature_id[4443].value > threshold=0.7009969055652618
node_119: feature_name=cg06038180	feature_id[3480].value > threshold=0.4583848565816879
node_121: feature_name=cg08619651	feature_id[4882].value <= threshold=0.2105495035648346
node_122: feature_name=cg01014262	feature_id[1911].value <= threshold=0.5327437818050385
node_123: feature_name=cg16863382	feature_id[2922].value > threshold=0.7200668156147003
node_133: feature_name=cg06989443	feature_id[2327].value <= threshold=0.6579365730285645
node_134: feature_name=cg07344990	feature_id[2398].value > threshold=0.5562876164913177
node_138: feature_name=cg02966841	feature_id[2199].value > threshold=0.7744501233100891
node_204: feature_name=cg12120430	feature_id[3727].value > threshold=0.5775820016860962
node_206: feature_name=cg18386876	feature_id[4066].value > threshold=0.6847257018089294
node_208: feature_name=cg02053092	feature_id[3610].value > threshold=0.39507485926151276
node_210: feature_name=cg10298992	feature_id[3603].value <= threshold=0.5707973837852478
node_211: feature_name=cg02436098	feature_id[1712].value <= threshold=0.27362556010484695
node_212: feature_name=cg15464763	feature_id[2786].value > threshold=0.6618955731391907
node_214: feature_name=cg15264991	feature_id[2805].value <= threshold=0.701563149690628
node_215: feature_name=cg04585669	feature_id[4112].value > threshold=0.2930298447608948
node_217: feature_name=cg11482422	feature_id[2778].value > threshold=0.12514593824744225
Class: chondroblastoma (CB)	
Rules_94	passed counts:2
node_0: feature_name=cg11915444	feature_id[2002].value > threshold=0.3402601182460785

node_10: feature_name=cg26016985	feature_id[4407].value > threshold=0.6493876278400421
node_16: feature_name=cg23157618	feature_id[5511].value > threshold=0.5759606957435608
node_20: feature_name=cg10480329	feature_id[1439].value > threshold=0.5518321692943573
node_48: feature_name=cg17843418	feature_id[4868].value > threshold=0.34969255328178406
node_52: feature_name=cg24407065	feature_id[4522].value > threshold=0.5364363789558411
node_56: feature_name=cg07281938	feature_id[249].value > threshold=0.7506992518901825
node_78: feature_name=cg21759907	feature_id[1650].value > threshold=0.46823520958423615
node_98: feature_name=cg10886334	feature_id[3975].value > threshold=0.43698127567768097
node_102: feature_name=cg09373983	feature_id[5025].value > threshold=0.5575815737247467
node_106: feature_name=cg12948116	feature_id[5360].value <= threshold=0.5658791363239288
node_107: feature_name=cg21906519	feature_id[5251].value > threshold=0.6089984774589539
node_111: feature_name=cg17537493	feature_id[4217].value > threshold=0.5825372636318207
node_117: feature_name=cg15720017	feature_id[4443].value > threshold=0.7009969055652618
node_119: feature_name=cg06038180	feature_id[3480].value > threshold=0.4583848565816879
node_121: feature_name=cg08619651	feature_id[4882].value <= threshold=0.2105495035648346
node_122: feature_name=cg01014262	feature_id[1911].value <= threshold=0.5327437818050385
node_123: feature_name=cg16863382	feature_id[2922].value > threshold=0.7200668156147003
node_133: feature_name=cg06989443	feature_id[2327].value <= threshold=0.6579365730285645
node_134: feature_name=cg07344990	feature_id[2398].value > threshold=0.5562876164913177
node_138: feature_name=cg02966841	feature_id[2199].value > threshold=0.7744501233100891
node_204: feature_name=cg12120430	feature_id[3727].value > threshold=0.5775820016860962
node_206: feature_name=cg18386876	feature_id[4066].value > threshold=0.6847257018089294

node_208: feature_name=cg02053092	feature_id[3610].value > threshold=0.39507485926151276
node_210: feature_name=cg10298992	feature_id[3603].value <= threshold=0.5707973837852478
node_211: feature_name=cg02436098	feature_id[1712].value <= threshold=0.27362556010484695
node_212: feature_name=cg15464763	feature_id[2786].value > threshold=0.6618955731391907
node_214: feature_name=cg15264991	feature_id[2805].value <= threshold=0.701563149690628
node_215: feature_name=cg04585669	feature_id[4112].value <= threshold=0.2930298447608948
Class: sarcoma (SARC)	
Rules_95	passed counts:2
node_0: feature_name=cg11915444	feature_id[2002].value > threshold=0.3402601182460785
node_10: feature_name=cg26016985	feature_id[4407].value > threshold=0.6493876278400421
node_16: feature_name=cg23157618	feature_id[5511].value > threshold=0.5759606957435608
node_20: feature_name=cg10480329	feature_id[1439].value > threshold=0.5518321692943573
node_48: feature_name=cg17843418	feature_id[4868].value > threshold=0.34969255328178406
node_52: feature_name=cg24407065	feature_id[4522].value > threshold=0.5364363789558411
node_56: feature_name=cg07281938	feature_id[249].value > threshold=0.7506992518901825
node_78: feature_name=cg21759907	feature_id[1650].value > threshold=0.46823520958423615
node_98: feature_name=cg10886334	feature_id[3975].value > threshold=0.43698127567768097
node_102: feature_name=cg09373983	feature_id[5025].value > threshold=0.5575815737247467
node_106: feature_name=cg12948116	feature_id[5360].value <= threshold=0.5658791363239288
node_107: feature_name=cg21906519	feature_id[5251].value > threshold=0.6089984774589539
node_111: feature_name=cg17537493	feature_id[4217].value > threshold=0.5825372636318207
node_117: feature_name=cg15720017	feature_id[4443].value > threshold=0.7009969055652618

node_119: feature_name=cg06038180	feature_id[3480].value > threshold=0.4583848565816879
node_121: feature_name=cg08619651	feature_id[4882].value <= threshold=0.2105495035648346
node_122: feature_name=cg01014262	feature_id[1911].value <= threshold=0.5327437818050385
node_123: feature_name=cg16863382	feature_id[2922].value > threshold=0.7200668156147003
node_133: feature_name=cg06989443	feature_id[2327].value <= threshold=0.6579365730285645
node_134: feature_name=cg07344990	feature_id[2398].value > threshold=0.5562876164913177
node_138: feature_name=cg02966841	feature_id[2199].value <= threshold=0.7744501233100891
node_139: feature_name=cg06989253	feature_id[194].value > threshold=0.7899391353130341
node_171: feature_name=cg07041720	feature_id[932].value > threshold=0.5004830807447433
node_189: feature_name=cg19374752	feature_id[196].value <= threshold=0.6096120476722717
node_190: feature_name=cg23085846	feature_id[3499].value <= threshold=0.8292354643344879
node_191: feature_name=cg04508340	feature_id[3427].value > threshold=0.8703204989433289
Class: alveolar soft part sarcoma (ASPS)	
Rules_96	passed counts:2
node_0: feature_name=cg11915444	feature_id[2002].value > threshold=0.3402601182460785
node_10: feature_name=cg26016985	feature_id[4407].value > threshold=0.6493876278400421
node_16: feature_name=cg23157618	feature_id[5511].value > threshold=0.5759606957435608
node_20: feature_name=cg10480329	feature_id[1439].value > threshold=0.5518321692943573
node_48: feature_name=cg17843418	feature_id[4868].value > threshold=0.34969255328178406
node_52: feature_name=cg24407065	feature_id[4522].value > threshold=0.5364363789558411
node_56: feature_name=cg07281938	feature_id[249].value > threshold=0.7506992518901825
node_78: feature_name=cg21759907	feature_id[1650].value > threshold=0.46823520958423615

node_98: feature_name=cg10886334	feature_id[3975].value > threshold=0.43698127567768097
node_102: feature_name=cg09373983	feature_id[5025].value > threshold=0.5575815737247467
node_106: feature_name=cg12948116	feature_id[5360].value <= threshold=0.5658791363239288
node_107: feature_name=cg21906519	feature_id[5251].value > threshold=0.6089984774589539
node_111: feature_name=cg17537493	feature_id[4217].value > threshold=0.5825372636318207
node_117: feature_name=cg15720017	feature_id[4443].value > threshold=0.7009969055652618
node_119: feature_name=cg06038180	feature_id[3480].value > threshold=0.4583848565816879
node_121: feature_name=cg08619651	feature_id[4882].value <= threshold=0.2105495035648346
node_122: feature_name=cg01014262	feature_id[1911].value <= threshold=0.5327437818050385
node_123: feature_name=cg16863382	feature_id[2922].value > threshold=0.7200668156147003
node_133: feature_name=cg06989443	feature_id[2327].value <= threshold=0.6579365730285645
node_134: feature_name=cg07344990	feature_id[2398].value > threshold=0.5562876164913177
node_138: feature_name=cg02966841	feature_id[2199].value <= threshold=0.7744501233100891
node_139: feature_name=cg06989253	feature_id[194].value > threshold=0.7899391353130341
node_171: feature_name=cg07041720	feature_id[932].value > threshold=0.5004830807447433
node_189: feature_name=cg19374752	feature_id[196].value <= threshold=0.6096120476722717
node_190: feature_name=cg23085846	feature_id[3499].value <= threshold=0.8292354643344879
node_191: feature_name=cg04508340	feature_id[3427].value <= threshold=0.8703204989433289
node_192: feature_name=cg05951828	feature_id[21].value <= threshold=0.8927997946739197
Class: giant cell tumour of bone (GCTB)	
Rules_97	passed counts:2
node_0: feature_name=cg11915444	feature_id[2002].value > threshold=0.3402601182460785

node_10: feature_name=cg26016985	feature_id[4407].value > threshold=0.6493876278400421
node_16: feature_name=cg23157618	feature_id[5511].value > threshold=0.5759606957435608
node_20: feature_name=cg10480329	feature_id[1439].value > threshold=0.5518321692943573
node_48: feature_name=cg17843418	feature_id[4868].value > threshold=0.34969255328178406
node_52: feature_name=cg24407065	feature_id[4522].value > threshold=0.5364363789558411
node_56: feature_name=cg07281938	feature_id[249].value > threshold=0.7506992518901825
node_78: feature_name=cg21759907	feature_id[1650].value > threshold=0.46823520958423615
node_98: feature_name=cg10886334	feature_id[3975].value > threshold=0.43698127567768097
node_102: feature_name=cg09373983	feature_id[5025].value > threshold=0.5575815737247467
node_106: feature_name=cg12948116	feature_id[5360].value <= threshold=0.5658791363239288
node_107: feature_name=cg21906519	feature_id[5251].value > threshold=0.6089984774589539
node_111: feature_name=cg17537493	feature_id[4217].value > threshold=0.5825372636318207
node_117: feature_name=cg15720017	feature_id[4443].value > threshold=0.7009969055652618
node_119: feature_name=cg06038180	feature_id[3480].value > threshold=0.4583848565816879
node_121: feature_name=cg08619651	feature_id[4882].value <= threshold=0.2105495035648346
node_122: feature_name=cg01014262	feature_id[1911].value <= threshold=0.5327437818050385
node_123: feature_name=cg16863382	feature_id[2922].value > threshold=0.7200668156147003
node_133: feature_name=cg06989443	feature_id[2327].value <= threshold=0.6579365730285645
node_134: feature_name=cg07344990	feature_id[2398].value > threshold=0.5562876164913177
node_138: feature_name=cg02966841	feature_id[2199].value <= threshold=0.7744501233100891
node_139: feature_name=cg06989253	feature_id[194].value > threshold=0.7899391353130341
node_171: feature_name=cg07041720	feature_id[932].value <= threshold=0.5004830807447433

node_172: feature_name=cg04716580	feature_id[1390].value > threshold=0.8913259208202362
node_182: feature_name=cg24426483	feature_id[3487].value <= threshold=0.7537154853343964
node_183: feature_name=cg14753356	feature_id[385].value <= threshold=0.3692520558834076
Class: epithelioid sarcoma (ES)	
Rules_98	passed counts:2
node_0: feature_name=cg11915444	feature_id[2002].value > threshold=0.3402601182460785
node_10: feature_name=cg26016985	feature_id[4407].value > threshold=0.6493876278400421
node_16: feature_name=cg23157618	feature_id[5511].value > threshold=0.5759606957435608
node_20: feature_name=cg10480329	feature_id[1439].value > threshold=0.5518321692943573
node_48: feature_name=cg17843418	feature_id[4868].value > threshold=0.34969255328178406
node_52: feature_name=cg24407065	feature_id[4522].value > threshold=0.5364363789558411
node_56: feature_name=cg07281938	feature_id[249].value > threshold=0.7506992518901825
node_78: feature_name=cg21759907	feature_id[1650].value > threshold=0.46823520958423615
node_98: feature_name=cg10886334	feature_id[3975].value > threshold=0.43698127567768097
node_102: feature_name=cg09373983	feature_id[5025].value > threshold=0.5575815737247467
node_106: feature_name=cg12948116	feature_id[5360].value <= threshold=0.5658791363239288
node_107: feature_name=cg21906519	feature_id[5251].value > threshold=0.6089984774589539
node_111: feature_name=cg17537493	feature_id[4217].value > threshold=0.5825372636318207
node_117: feature_name=cg15720017	feature_id[4443].value > threshold=0.7009969055652618
node_119: feature_name=cg06038180	feature_id[3480].value > threshold=0.4583848565816879
node_121: feature_name=cg08619651	feature_id[4882].value <= threshold=0.2105495035648346
node_122: feature_name=cg01014262	feature_id[1911].value <= threshold=0.5327437818050385

node_123: feature_name=cg16863382	feature_id[2922].value > threshold=0.7200668156147003
node_133: feature_name=cg06989443	feature_id[2327].value <= threshold=0.6579365730285645
node_134: feature_name=cg07344990	feature_id[2398].value > threshold=0.5562876164913177
node_138: feature_name=cg02966841	feature_id[2199].value <= threshold=0.7744501233100891
node_139: feature_name=cg06989253	feature_id[194].value <= threshold=0.7899391353130341
node_140: feature_name=cg24617568	feature_id[3282].value <= threshold=0.26932457089424133
node_141: feature_name=cg12614090	feature_id[2765].value <= threshold=0.1813211366534233
node_142: feature_name=cg10917602	feature_id[4685].value <= threshold=0.5744736790657043
node_143: feature_name=cg23723410	feature_id[34].value <= threshold=0.3201078921556473
node_144: feature_name=cg26804423	feature_id[1725].value > threshold=0.39587317407131195
node_146: feature_name=cg20080282	feature_id[1150].value <= threshold=0.856019914150238
node_147: feature_name=cg08924374	feature_id[4125].value <= threshold=0.07863279432058334
Class: low-grade fibromyxoid sarcoma (LGFMS)	
Rules_99	passed counts:2
node_0: feature_name=cg11915444	feature_id[2002].value > threshold=0.3402601182460785
node_10: feature_name=cg26016985	feature_id[4407].value > threshold=0.6493876278400421
node_16: feature_name=cg23157618	feature_id[5511].value > threshold=0.5759606957435608
node_20: feature_name=cg10480329	feature_id[1439].value > threshold=0.5518321692943573
node_48: feature_name=cg17843418	feature_id[4868].value > threshold=0.34969255328178406
node_52: feature_name=cg24407065	feature_id[4522].value > threshold=0.5364363789558411
node_56: feature_name=cg07281938	feature_id[249].value > threshold=0.7506992518901825
node_78: feature_name=cg21759907	feature_id[1650].value > threshold=0.46823520958423615

node_98: feature_name=cg10886334	feature_id[3975].value > threshold=0.43698127567768097
node_102: feature_name=cg09373983	feature_id[5025].value > threshold=0.5575815737247467
node_106: feature_name=cg12948116	feature_id[5360].value <= threshold=0.5658791363239288
node_107: feature_name=cg21906519	feature_id[5251].value > threshold=0.6089984774589539
node_111: feature_name=cg17537493	feature_id[4217].value > threshold=0.5825372636318207
node_117: feature_name=cg15720017	feature_id[4443].value > threshold=0.7009969055652618
node_119: feature_name=cg06038180	feature_id[3480].value > threshold=0.4583848565816879
node_121: feature_name=cg08619651	feature_id[4882].value <= threshold=0.2105495035648346
node_122: feature_name=cg01014262	feature_id[1911].value <= threshold=0.5327437818050385
node_123: feature_name=cg16863382	feature_id[2922].value <= threshold=0.7200668156147003
node_124: feature_name=cg11225330	feature_id[3185].value > threshold=0.7814575433731079
node_126: feature_name=cg15543534	feature_id[423].value <= threshold=0.86179119348526
node_127: feature_name=cg18809729	feature_id[5184].value > threshold=0.8457489907741547
Class: embryonal rhabdomyosarcoma (RMS (EMB))	
Rules_100	passed counts:2
node_0: feature_name=cg11915444	feature_id[2002].value > threshold=0.3402601182460785
node_10: feature_name=cg26016985	feature_id[4407].value > threshold=0.6493876278400421
node_16: feature_name=cg23157618	feature_id[5511].value > threshold=0.5759606957435608
node_20: feature_name=cg10480329	feature_id[1439].value > threshold=0.5518321692943573
node_48: feature_name=cg17843418	feature_id[4868].value > threshold=0.34969255328178406
node_52: feature_name=cg24407065	feature_id[4522].value > threshold=0.5364363789558411

node_56: feature_name=cg07281938	feature_id[249].value > threshold=0.7506992518901825
node_78: feature_name=cg21759907	feature_id[1650].value > threshold=0.46823520958423615
node_98: feature_name=cg10886334	feature_id[3975].value > threshold=0.43698127567768097
node_102: feature_name=cg09373983	feature_id[5025].value > threshold=0.5575815737247467
node_106: feature_name=cg12948116	feature_id[5360].value <= threshold=0.5658791363239288
node_107: feature_name=cg21906519	feature_id[5251].value > threshold=0.6089984774589539
node_111: feature_name=cg17537493	feature_id[4217].value > threshold=0.5825372636318207
node_117: feature_name=cg15720017	feature_id[4443].value > threshold=0.7009969055652618
node_119: feature_name=cg06038180	feature_id[3480].value > threshold=0.4583848565816879
node_121: feature_name=cg08619651	feature_id[4882].value <= threshold=0.2105495035648346
node_122: feature_name=cg01014262	feature_id[1911].value <= threshold=0.5327437818050385
node_123: feature_name=cg16863382	feature_id[2922].value <= threshold=0.7200668156147003
node_124: feature_name=cg11225330	feature_id[3185].value > threshold=0.7814575433731079
node_126: feature_name=cg15543534	feature_id[423].value <= threshold=0.86179119348526
node_127: feature_name=cg18809729	feature_id[5184].value <= threshold=0.8457489907741547
node_128: feature_name=cg20000539	feature_id[5329].value > threshold=0.7002188265323639
Class: undifferentiated sarcoma (USARC)	
Rules_101	passed counts:2
node_0: feature_name=cg11915444	feature_id[2002].value > threshold=0.3402601182460785
node_10: feature_name=cg26016985	feature_id[4407].value > threshold=0.6493876278400421
node_16: feature_name=cg23157618	feature_id[5511].value > threshold=0.5759606957435608
node_20: feature_name=cg10480329	feature_id[1439].value > threshold=0.5518321692943573

node_48: feature_name=cg17843418	feature_id[4868].value > threshold=0.34969255328178406
node_52: feature_name=cg24407065	feature_id[4522].value > threshold=0.5364363789558411
node_56: feature_name=cg07281938	feature_id[249].value > threshold=0.7506992518901825
node_78: feature_name=cg21759907	feature_id[1650].value <= threshold=0.46823520958423615
node_79: feature_name=cg25476129	feature_id[2302].value <= threshold=0.1687404215335846
node_80: feature_name=cg26362368	feature_id[414].value <= threshold=0.834072083234787
node_81: feature_name=cg15881332	feature_id[154].value <= threshold=0.7577665150165558
node_82: feature_name=cg14651518	feature_id[1456].value > threshold=0.7856765985488892
Class: undifferentiated sarcoma (USARC)	
Rules_102	passed counts:2
node_0: feature_name=cg11915444	feature_id[2002].value > threshold=0.3402601182460785
node_10: feature_name=cg26016985	feature_id[4407].value > threshold=0.6493876278400421
node_16: feature_name=cg23157618	feature_id[5511].value > threshold=0.5759606957435608
node_20: feature_name=cg10480329	feature_id[1439].value > threshold=0.5518321692943573
node_48: feature_name=cg17843418	feature_id[4868].value > threshold=0.34969255328178406
node_52: feature_name=cg24407065	feature_id[4522].value > threshold=0.5364363789558411
node_56: feature_name=cg07281938	feature_id[249].value <= threshold=0.7506992518901825
node_57: feature_name=cg21189849	feature_id[1261].value <= threshold=0.7545044124126434
node_58: feature_name=cg11960033	feature_id[260].value <= threshold=0.7065970599651337
node_59: feature_name=cg18148375	feature_id[1524].value <= threshold=0.6905054450035095
Class: clear cell chondrosarcoma (CSA (CC))	
Rules_103	passed counts:2

node_0: feature_name=cg11915444	feature_id[2002].value > threshold=0.3402601182460785
node_10: feature_name=cg26016985	feature_id[4407].value > threshold=0.6493876278400421
node_16: feature_name=cg23157618	feature_id[5511].value > threshold=0.5759606957435608
node_20: feature_name=cg10480329	feature_id[1439].value <= threshold=0.5518321692943573
node_21: feature_name=cg25510609	feature_id[149].value <= threshold=0.08151235431432724
node_22: feature_name=cg23850277	feature_id[2159].value <= threshold=0.5682950615882874
node_23: feature_name=cg01259126	feature_id[3146].value > threshold=0.34355829656124115
node_25: feature_name=cg07034004	feature_id[3345].value <= threshold=0.20594919472932816
node_26: feature_name=cg12895304	feature_id[651].value > threshold=0.9125175774097443
node_28: feature_name=cg02235663	feature_id[785].value <= threshold=0.4301222711801529
Class: high-grade conventional osteosarcoma (OS (HG))	
Rules_104	passed counts:2
node_0: feature_name=cg11915444	feature_id[2002].value > threshold=0.3402601182460785
node_10: feature_name=cg26016985	feature_id[4407].value > threshold=0.6493876278400421
node_16: feature_name=cg23157618	feature_id[5511].value > threshold=0.5759606957435608
node_20: feature_name=cg10480329	feature_id[1439].value <= threshold=0.5518321692943573
node_21: feature_name=cg25510609	feature_id[149].value <= threshold=0.08151235431432724
node_22: feature_name=cg23850277	feature_id[2159].value <= threshold=0.5682950615882874
node_23: feature_name=cg01259126	feature_id[3146].value > threshold=0.34355829656124115
node_25: feature_name=cg07034004	feature_id[3345].value <= threshold=0.20594919472932816
node_26: feature_name=cg12895304	feature_id[651].value <= threshold=0.9125175774097443

Class: mesenchymal chondrosarcoma (CSA (MES))	
Rules_105	passed counts:2
node_0: feature_name=cg11915444	feature_id[2002].value <= threshold=0.3402601182460785
node_1: feature_name=cg17156862	feature_id[2598].value <= threshold=0.6409126222133636
node_2: feature_name=cg08947774	feature_id[1302].value > threshold=0.6231959313154221
Class: alveolar rhabdomyosarcoma (RMS (ALV))	
Rules_106	passed counts:1
node_0: feature_name=cg11915444	feature_id[2002].value > threshold=0.3402601182460785
node_10: feature_name=cg26016985	feature_id[4407].value > threshold=0.6493876278400421
node_16: feature_name=cg23157618	feature_id[5511].value > threshold=0.5759606957435608
node_20: feature_name=cg10480329	feature_id[1439].value > threshold=0.5518321692943573
node_48: feature_name=cg17843418	feature_id[4868].value > threshold=0.34969255328178406
node_52: feature_name=cg24407065	feature_id[4522].value > threshold=0.5364363789558411
node_56: feature_name=cg07281938	feature_id[249].value > threshold=0.7506992518901825
node_78: feature_name=cg21759907	feature_id[1650].value > threshold=0.46823520958423615
node_98: feature_name=cg10886334	feature_id[3975].value > threshold=0.43698127567768097
node_102: feature_name=cg09373983	feature_id[5025].value > threshold=0.5575815737247467
node_106: feature_name=cg12948116	feature_id[5360].value > threshold=0.5658791363239288
node_388: feature_name=cg02855309	feature_id[4351].value <= threshold=0.2790501043200493
node_389: feature_name=cg00686915	feature_id[1417].value > threshold=0.39021433889865875
node_391: feature_name=cg02363010	feature_id[2123].value > threshold=0.6251022964715958

Class: malignant peripheral nerve sheath tumour (MPNST)	
Rules_107	passed counts:1
node_0: feature_name=cg11915444	feature_id[2002].value > threshold=0.3402601182460785
node_10: feature_name=cg26016985	feature_id[4407].value > threshold=0.6493876278400421
node_16: feature_name=cg23157618	feature_id[5511].value > threshold=0.5759606957435608
node_20: feature_name=cg10480329	feature_id[1439].value > threshold=0.5518321692943573
node_48: feature_name=cg17843418	feature_id[4868].value > threshold=0.34969255328178406
node_52: feature_name=cg24407065	feature_id[4522].value > threshold=0.5364363789558411
node_56: feature_name=cg07281938	feature_id[249].value > threshold=0.7506992518901825
node_78: feature_name=cg21759907	feature_id[1650].value > threshold=0.46823520958423615
node_98: feature_name=cg10886334	feature_id[3975].value > threshold=0.43698127567768097
node_102: feature_name=cg09373983	feature_id[5025].value > threshold=0.5575815737247467
node_106: feature_name=cg12948116	feature_id[5360].value > threshold=0.5658791363239288
node_388: feature_name=cg02855309	feature_id[4351].value <= threshold=0.2790501043200493
node_389: feature_name=cg00686915	feature_id[1417].value > threshold=0.39021433889865875
node_391: feature_name=cg02363010	feature_id[2123].value <= threshold=0.6251022964715958
Class: malignant rhabdoid tumour (MRT)	
Rules_108	passed counts:1
node_0: feature_name=cg11915444	feature_id[2002].value > threshold=0.3402601182460785
node_10: feature_name=cg26016985	feature_id[4407].value > threshold=0.6493876278400421
node_16: feature_name=cg23157618	feature_id[5511].value > threshold=0.5759606957435608
node_20: feature_name=cg10480329	feature_id[1439].value > threshold=0.5518321692943573

node_48: feature_name=cg17843418	feature_id[4868].value > threshold=0.34969255328178406
node_52: feature_name=cg24407065	feature_id[4522].value > threshold=0.5364363789558411
node_56: feature_name=cg07281938	feature_id[249].value > threshold=0.7506992518901825
node_78: feature_name=cg21759907	feature_id[1650].value > threshold=0.46823520958423615
node_98: feature_name=cg10886334	feature_id[3975].value > threshold=0.43698127567768097
node_102: feature_name=cg09373983	feature_id[5025].value > threshold=0.5575815737247467
node_106: feature_name=cg12948116	feature_id[5360].value > threshold=0.5658791363239288
node_388: feature_name=cg02855309	feature_id[4351].value <= threshold=0.2790501043200493
node_389: feature_name=cg00686915	feature_id[1417].value <= threshold=0.39021433889865875
Class: mesenchymal chondrosarcoma (CSA (MES))	
Rules_109	passed counts:1
node_0: feature_name=cg11915444	feature_id[2002].value > threshold=0.3402601182460785
node_10: feature_name=cg26016985	feature_id[4407].value > threshold=0.6493876278400421
node_16: feature_name=cg23157618	feature_id[5511].value > threshold=0.5759606957435608
node_20: feature_name=cg10480329	feature_id[1439].value > threshold=0.5518321692943573
node_48: feature_name=cg17843418	feature_id[4868].value > threshold=0.34969255328178406
node_52: feature_name=cg24407065	feature_id[4522].value > threshold=0.5364363789558411
node_56: feature_name=cg07281938	feature_id[249].value > threshold=0.7506992518901825
node_78: feature_name=cg21759907	feature_id[1650].value > threshold=0.46823520958423615
node_98: feature_name=cg10886334	feature_id[3975].value > threshold=0.43698127567768097
node_102: feature_name=cg09373983	feature_id[5025].value > threshold=0.5575815737247467

node_106: feature_name=cg12948116	feature_id[5360].value <= threshold=0.5658791363239288
node_107: feature_name=cg21906519	feature_id[5251].value > threshold=0.6089984774589539
node_111: feature_name=cg17537493	feature_id[4217].value > threshold=0.5825372636318207
node_117: feature_name=cg15720017	feature_id[4443].value > threshold=0.7009969055652618
node_119: feature_name=cg06038180	feature_id[3480].value > threshold=0.4583848565816879
node_121: feature_name=cg08619651	feature_id[4882].value <= threshold=0.2105495035648346
node_122: feature_name=cg01014262	feature_id[1911].value > threshold=0.5327437818050385
node_382: feature_name=cg26240231	feature_id[4832].value <= threshold=0.9200497567653656
node_383: feature_name=cg09524639	feature_id[3704].value > threshold=0.7802113592624664
Class: Langerhans cell histiocytosis (LCH)	
Rules_110	passed counts:1
node_0: feature_name=cg11915444	feature_id[2002].value > threshold=0.3402601182460785
node_10: feature_name=cg26016985	feature_id[4407].value > threshold=0.6493876278400421
node_16: feature_name=cg23157618	feature_id[5511].value > threshold=0.5759606957435608
node_20: feature_name=cg10480329	feature_id[1439].value > threshold=0.5518321692943573
node_48: feature_name=cg17843418	feature_id[4868].value > threshold=0.34969255328178406
node_52: feature_name=cg24407065	feature_id[4522].value > threshold=0.5364363789558411
node_56: feature_name=cg07281938	feature_id[249].value > threshold=0.7506992518901825
node_78: feature_name=cg21759907	feature_id[1650].value > threshold=0.46823520958423615
node_98: feature_name=cg10886334	feature_id[3975].value > threshold=0.43698127567768097
node_102: feature_name=cg09373983	feature_id[5025].value > threshold=0.5575815737247467
node_106: feature_name=cg12948116	feature_id[5360].value <= threshold=0.5658791363239288

node_107: feature_name=cg21906519	feature_id[5251].value > threshold=0.6089984774589539
node_111: feature_name=cg17537493	feature_id[4217].value > threshold=0.5825372636318207
node_117: feature_name=cg15720017	feature_id[4443].value > threshold=0.7009969055652618
node_119: feature_name=cg06038180	feature_id[3480].value > threshold=0.4583848565816879
node_121: feature_name=cg08619651	feature_id[4882].value <= threshold=0.2105495035648346
node_122: feature_name=cg01014262	feature_id[1911].value <= threshold=0.5327437818050385
node_123: feature_name=cg16863382	feature_id[2922].value > threshold=0.7200668156147003
node_133: feature_name=cg06989443	feature_id[2327].value > threshold=0.6579365730285645
node_355: feature_name=cg27470978	feature_id[4804].value > threshold=0.6264137625694275
node_361: feature_name=cg20252837	feature_id[420].value > threshold=0.53105828166008
node_363: feature_name=cg27437304	feature_id[1051].value > threshold=0.7641371190547943
node_365: feature_name=cg08215532	feature_id[3203].value <= threshold=0.45234426856040955
node_366: feature_name=cg12486498	feature_id[1977].value > threshold=0.237603098154068
node_368: feature_name=cg16892393	feature_id[3098].value > threshold=0.13003550469875336
node_370: feature_name=cg10982692	feature_id[4136].value > threshold=0.8049887418746948
node_372: feature_name=cg09996971	feature_id[4345].value > threshold=0.5518785715103149
node_374: feature_name=cg07083272	feature_id[4197].value > threshold=0.7356340289115906
node_376: feature_name=cg26607528	feature_id[4315].value > threshold=0.8817691504955292
node_378: feature_name=cg27492839	feature_id[3954].value > threshold=0.8696597218513489
Class: well differentiated liposarcoma (WDLS)/dedifferentiated liposarcoma (DDLS)	
Rules_111	passed counts:1

node_0: feature_name=cg11915444	feature_id[2002].value > threshold=0.3402601182460785
node_10: feature_name=cg26016985	feature_id[4407].value > threshold=0.6493876278400421
node_16: feature_name=cg23157618	feature_id[5511].value > threshold=0.5759606957435608
node_20: feature_name=cg10480329	feature_id[1439].value > threshold=0.5518321692943573
node_48: feature_name=cg17843418	feature_id[4868].value > threshold=0.34969255328178406
node_52: feature_name=cg24407065	feature_id[4522].value > threshold=0.5364363789558411
node_56: feature_name=cg07281938	feature_id[249].value > threshold=0.7506992518901825
node_78: feature_name=cg21759907	feature_id[1650].value > threshold=0.46823520958423615
node_98: feature_name=cg10886334	feature_id[3975].value > threshold=0.43698127567768097
node_102: feature_name=cg09373983	feature_id[5025].value > threshold=0.5575815737247467
node_106: feature_name=cg12948116	feature_id[5360].value <= threshold=0.5658791363239288
node_107: feature_name=cg21906519	feature_id[5251].value > threshold=0.6089984774589539
node_111: feature_name=cg17537493	feature_id[4217].value > threshold=0.5825372636318207
node_117: feature_name=cg15720017	feature_id[4443].value > threshold=0.7009969055652618
node_119: feature_name=cg06038180	feature_id[3480].value > threshold=0.4583848565816879
node_121: feature_name=cg08619651	feature_id[4882].value <= threshold=0.2105495035648346
node_122: feature_name=cg01014262	feature_id[1911].value <= threshold=0.5327437818050385
node_123: feature_name=cg16863382	feature_id[2922].value > threshold=0.7200668156147003
node_133: feature_name=cg06989443	feature_id[2327].value > threshold=0.6579365730285645
node_355: feature_name=cg27470978	feature_id[4804].value > threshold=0.6264137625694275
node_361: feature_name=cg20252837	feature_id[420].value > threshold=0.53105828166008
node_363: feature_name=cg27437304	feature_id[1051].value > threshold=0.7641371190547943

node_365: feature_name=cg08215532	feature_id[3203].value <= threshold=0.45234426856040955
node_366: feature_name=cg12486498	feature_id[1977].value > threshold=0.237603098154068
node_368: feature_name=cg16892393	feature_id[3098].value > threshold=0.13003550469875336
node_370: feature_name=cg10982692	feature_id[4136].value > threshold=0.8049887418746948
node_372: feature_name=cg09996971	feature_id[4345].value > threshold=0.5518785715103149
node_374: feature_name=cg07083272	feature_id[4197].value > threshold=0.7356340289115906
node_376: feature_name=cg26607528	feature_id[4315].value > threshold=0.8817691504955292
node_378: feature_name=cg27492839	feature_id[3954].value <= threshold=0.8696597218513489
Class: sclerosing epithelioid fibrosarcoma(SEF)	
Rules_112	passed counts:1
node_0: feature_name=cg11915444	feature_id[2002].value > threshold=0.3402601182460785
node_10: feature_name=cg26016985	feature_id[4407].value > threshold=0.6493876278400421
node_16: feature_name=cg23157618	feature_id[5511].value > threshold=0.5759606957435608
node_20: feature_name=cg10480329	feature_id[1439].value > threshold=0.5518321692943573
node_48: feature_name=cg17843418	feature_id[4868].value > threshold=0.34969255328178406
node_52: feature_name=cg24407065	feature_id[4522].value > threshold=0.5364363789558411
node_56: feature_name=cg07281938	feature_id[249].value > threshold=0.7506992518901825
node_78: feature_name=cg21759907	feature_id[1650].value > threshold=0.46823520958423615
node_98: feature_name=cg10886334	feature_id[3975].value > threshold=0.43698127567768097
node_102: feature_name=cg09373983	feature_id[5025].value > threshold=0.5575815737247467
node_106: feature_name=cg12948116	feature_id[5360].value <= threshold=0.5658791363239288
node_107: feature_name=cg21906519	feature_id[5251].value > threshold=0.6089984774589539

node_111: feature_name=cg17537493	feature_id[4217].value > threshold=0.5825372636318207
node_117: feature_name=cg15720017	feature_id[4443].value > threshold=0.7009969055652618
node_119: feature_name=cg06038180	feature_id[3480].value > threshold=0.4583848565816879
node_121: feature_name=cg08619651	feature_id[4882].value <= threshold=0.2105495035648346
node_122: feature_name=cg01014262	feature_id[1911].value <= threshold=0.5327437818050385
node_123: feature_name=cg16863382	feature_id[2922].value > threshold=0.7200668156147003
node_133: feature_name=cg06989443	feature_id[2327].value > threshold=0.6579365730285645
node_355: feature_name=cg27470978	feature_id[4804].value > threshold=0.6264137625694275
node_361: feature_name=cg20252837	feature_id[420].value > threshold=0.53105828166008
node_363: feature_name=cg27437304	feature_id[1051].value > threshold=0.7641371190547943
node_365: feature_name=cg08215532	feature_id[3203].value <= threshold=0.45234426856040955
node_366: feature_name=cg12486498	feature_id[1977].value > threshold=0.237603098154068
node_368: feature_name=cg16892393	feature_id[3098].value > threshold=0.13003550469875336
node_370: feature_name=cg10982692	feature_id[4136].value > threshold=0.8049887418746948
node_372: feature_name=cg09996971	feature_id[4345].value > threshold=0.5518785715103149
node_374: feature_name=cg07083272	feature_id[4197].value > threshold=0.7356340289115906
node_376: feature_name=cg26607528	feature_id[4315].value <= threshold=0.8817691504955292
Class: malignant rhabdoid tumour (MRT)	
Rules_113	passed counts:1
node_0: feature_name=cg11915444	feature_id[2002].value > threshold=0.3402601182460785
node_10: feature_name=cg26016985	feature_id[4407].value > threshold=0.6493876278400421
node_16: feature_name=cg23157618	feature_id[5511].value > threshold=0.5759606957435608

node_20: feature_name=cg10480329	feature_id[1439].value > threshold=0.5518321692943573
node_48: feature_name=cg17843418	feature_id[4868].value > threshold=0.34969255328178406
node_52: feature_name=cg24407065	feature_id[4522].value > threshold=0.5364363789558411
node_56: feature_name=cg07281938	feature_id[249].value > threshold=0.7506992518901825
node_78: feature_name=cg21759907	feature_id[1650].value > threshold=0.46823520958423615
node_98: feature_name=cg10886334	feature_id[3975].value > threshold=0.43698127567768097
node_102: feature_name=cg09373983	feature_id[5025].value > threshold=0.5575815737247467
node_106: feature_name=cg12948116	feature_id[5360].value <= threshold=0.5658791363239288
node_107: feature_name=cg21906519	feature_id[5251].value > threshold=0.6089984774589539
node_111: feature_name=cg17537493	feature_id[4217].value > threshold=0.5825372636318207
node_117: feature_name=cg15720017	feature_id[4443].value > threshold=0.7009969055652618
node_119: feature_name=cg06038180	feature_id[3480].value > threshold=0.4583848565816879
node_121: feature_name=cg08619651	feature_id[4882].value <= threshold=0.2105495035648346
node_122: feature_name=cg01014262	feature_id[1911].value <= threshold=0.5327437818050385
node_123: feature_name=cg16863382	feature_id[2922].value > threshold=0.7200668156147003
node_133: feature_name=cg06989443	feature_id[2327].value > threshold=0.6579365730285645
node_355: feature_name=cg27470978	feature_id[4804].value > threshold=0.6264137625694275
node_361: feature_name=cg20252837	feature_id[420].value > threshold=0.53105828166008
node_363: feature_name=cg27437304	feature_id[1051].value > threshold=0.7641371190547943
node_365: feature_name=cg08215532	feature_id[3203].value <= threshold=0.45234426856040955
node_366: feature_name=cg12486498	feature_id[1977].value > threshold=0.237603098154068
node_368: feature_name=cg16892393	feature_id[3098].value > threshold=0.13003550469875336

node_370: feature_name=cg10982692	feature_id[4136].value > threshold=0.8049887418746948
node_372: feature_name=cg09996971	feature_id[4345].value > threshold=0.5518785715103149
node_374: feature_name=cg07083272	feature_id[4197].value <= threshold=0.7356340289115906
Class: malignant peripheral nerve sheath tumour (MPNST)	
Rules_114	passed counts:1
node_0: feature_name=cg11915444	feature_id[2002].value > threshold=0.3402601182460785
node_10: feature_name=cg26016985	feature_id[4407].value > threshold=0.6493876278400421
node_16: feature_name=cg23157618	feature_id[5511].value > threshold=0.5759606957435608
node_20: feature_name=cg10480329	feature_id[1439].value > threshold=0.5518321692943573
node_48: feature_name=cg17843418	feature_id[4868].value > threshold=0.34969255328178406
node_52: feature_name=cg24407065	feature_id[4522].value > threshold=0.5364363789558411
node_56: feature_name=cg07281938	feature_id[249].value > threshold=0.7506992518901825
node_78: feature_name=cg21759907	feature_id[1650].value > threshold=0.46823520958423615
node_98: feature_name=cg10886334	feature_id[3975].value > threshold=0.43698127567768097
node_102: feature_name=cg09373983	feature_id[5025].value > threshold=0.5575815737247467
node_106: feature_name=cg12948116	feature_id[5360].value <= threshold=0.5658791363239288
node_107: feature_name=cg21906519	feature_id[5251].value > threshold=0.6089984774589539
node_111: feature_name=cg17537493	feature_id[4217].value > threshold=0.5825372636318207
node_117: feature_name=cg15720017	feature_id[4443].value > threshold=0.7009969055652618
node_119: feature_name=cg06038180	feature_id[3480].value > threshold=0.4583848565816879
node_121: feature_name=cg08619651	feature_id[4882].value <= threshold=0.2105495035648346

node_122: feature_name=cg01014262	feature_id[1911].value <= threshold=0.5327437818050385
node_123: feature_name=cg16863382	feature_id[2922].value > threshold=0.7200668156147003
node_133: feature_name=cg06989443	feature_id[2327].value > threshold=0.6579365730285645
node_355: feature_name=cg27470978	feature_id[4804].value > threshold=0.6264137625694275
node_361: feature_name=cg20252837	feature_id[420].value > threshold=0.53105828166008
node_363: feature_name=cg27437304	feature_id[1051].value > threshold=0.7641371190547943
node_365: feature_name=cg08215532	feature_id[3203].value <= threshold=0.45234426856040955
node_366: feature_name=cg12486498	feature_id[1977].value > threshold=0.237603098154068
node_368: feature_name=cg16892393	feature_id[3098].value > threshold=0.13003550469875336
node_370: feature_name=cg10982692	feature_id[4136].value > threshold=0.8049887418746948
node_372: feature_name=cg09996971	feature_id[4345].value <= threshold=0.5518785715103149
Class: atypical fibroxanthoma (AFX)/pleomorphic dermal sarcoma (PDS)	
Rules_115	passed counts:1
node_0: feature_name=cg11915444	feature_id[2002].value > threshold=0.3402601182460785
node_10: feature_name=cg26016985	feature_id[4407].value > threshold=0.6493876278400421
node_16: feature_name=cg23157618	feature_id[5511].value > threshold=0.5759606957435608
node_20: feature_name=cg10480329	feature_id[1439].value > threshold=0.5518321692943573
node_48: feature_name=cg17843418	feature_id[4868].value > threshold=0.34969255328178406
node_52: feature_name=cg24407065	feature_id[4522].value > threshold=0.5364363789558411
node_56: feature_name=cg07281938	feature_id[249].value > threshold=0.7506992518901825
node_78: feature_name=cg21759907	feature_id[1650].value > threshold=0.46823520958423615

node_98: feature_name=cg10886334	feature_id[3975].value > threshold=0.43698127567768097
node_102: feature_name=cg09373983	feature_id[5025].value > threshold=0.5575815737247467
node_106: feature_name=cg12948116	feature_id[5360].value <= threshold=0.5658791363239288
node_107: feature_name=cg21906519	feature_id[5251].value > threshold=0.6089984774589539
node_111: feature_name=cg17537493	feature_id[4217].value > threshold=0.5825372636318207
node_117: feature_name=cg15720017	feature_id[4443].value > threshold=0.7009969055652618
node_119: feature_name=cg06038180	feature_id[3480].value > threshold=0.4583848565816879
node_121: feature_name=cg08619651	feature_id[4882].value <= threshold=0.2105495035648346
node_122: feature_name=cg01014262	feature_id[1911].value <= threshold=0.5327437818050385
node_123: feature_name=cg16863382	feature_id[2922].value > threshold=0.7200668156147003
node_133: feature_name=cg06989443	feature_id[2327].value > threshold=0.6579365730285645
node_355: feature_name=cg27470978	feature_id[4804].value > threshold=0.6264137625694275
node_361: feature_name=cg20252837	feature_id[420].value > threshold=0.53105828166008
node_363: feature_name=cg27437304	feature_id[1051].value > threshold=0.7641371190547943
node_365: feature_name=cg08215532	feature_id[3203].value <= threshold=0.45234426856040955
node_366: feature_name=cg12486498	feature_id[1977].value > threshold=0.237603098154068
node_368: feature_name=cg16892393	feature_id[3098].value > threshold=0.13003550469875336
node_370: feature_name=cg10982692	feature_id[4136].value <= threshold=0.8049887418746948
Class: melanoma (MEL)	
Rules_116	passed counts:1
node_0: feature_name=cg11915444	feature_id[2002].value > threshold=0.3402601182460785
node_10: feature_name=cg26016985	feature_id[4407].value > threshold=0.6493876278400421

node_16: feature_name=cg23157618	feature_id[5511].value > threshold=0.5759606957435608
node_20: feature_name=cg10480329	feature_id[1439].value > threshold=0.5518321692943573
node_48: feature_name=cg17843418	feature_id[4868].value > threshold=0.34969255328178406
node_52: feature_name=cg24407065	feature_id[4522].value > threshold=0.5364363789558411
node_56: feature_name=cg07281938	feature_id[249].value > threshold=0.7506992518901825
node_78: feature_name=cg21759907	feature_id[1650].value > threshold=0.46823520958423615
node_98: feature_name=cg10886334	feature_id[3975].value > threshold=0.43698127567768097
node_102: feature_name=cg09373983	feature_id[5025].value > threshold=0.5575815737247467
node_106: feature_name=cg12948116	feature_id[5360].value <= threshold=0.5658791363239288
node_107: feature_name=cg21906519	feature_id[5251].value > threshold=0.6089984774589539
node_111: feature_name=cg17537493	feature_id[4217].value > threshold=0.5825372636318207
node_117: feature_name=cg15720017	feature_id[4443].value > threshold=0.7009969055652618
node_119: feature_name=cg06038180	feature_id[3480].value > threshold=0.4583848565816879
node_121: feature_name=cg08619651	feature_id[4882].value <= threshold=0.2105495035648346
node_122: feature_name=cg01014262	feature_id[1911].value <= threshold=0.5327437818050385
node_123: feature_name=cg16863382	feature_id[2922].value > threshold=0.7200668156147003
node_133: feature_name=cg06989443	feature_id[2327].value > threshold=0.6579365730285645
node_355: feature_name=cg27470978	feature_id[4804].value > threshold=0.6264137625694275
node_361: feature_name=cg20252837	feature_id[420].value > threshold=0.53105828166008
node_363: feature_name=cg27437304	feature_id[1051].value > threshold=0.7641371190547943
node_365: feature_name=cg08215532	feature_id[3203].value <= threshold=0.45234426856040955
node_366: feature_name=cg12486498	feature_id[1977].value > threshold=0.237603098154068

node_368: feature_name=cg16892393	feature_id[3098].value <= threshold=0.13003550469875336
Class: Ewing sarcoma (EWING)	
Rules_117	passed counts:1
node_0: feature_name=cg11915444	feature_id[2002].value > threshold=0.3402601182460785
node_10: feature_name=cg26016985	feature_id[4407].value > threshold=0.6493876278400421
node_16: feature_name=cg23157618	feature_id[5511].value > threshold=0.5759606957435608
node_20: feature_name=cg10480329	feature_id[1439].value > threshold=0.5518321692943573
node_48: feature_name=cg17843418	feature_id[4868].value > threshold=0.34969255328178406
node_52: feature_name=cg24407065	feature_id[4522].value > threshold=0.5364363789558411
node_56: feature_name=cg07281938	feature_id[249].value > threshold=0.7506992518901825
node_78: feature_name=cg21759907	feature_id[1650].value > threshold=0.46823520958423615
node_98: feature_name=cg10886334	feature_id[3975].value > threshold=0.43698127567768097
node_102: feature_name=cg09373983	feature_id[5025].value > threshold=0.5575815737247467
node_106: feature_name=cg12948116	feature_id[5360].value <= threshold=0.5658791363239288
node_107: feature_name=cg21906519	feature_id[5251].value > threshold=0.6089984774589539
node_111: feature_name=cg17537493	feature_id[4217].value > threshold=0.5825372636318207
node_117: feature_name=cg15720017	feature_id[4443].value > threshold=0.7009969055652618
node_119: feature_name=cg06038180	feature_id[3480].value > threshold=0.4583848565816879
node_121: feature_name=cg08619651	feature_id[4882].value <= threshold=0.2105495035648346
node_122: feature_name=cg01014262	feature_id[1911].value <= threshold=0.5327437818050385
node_123: feature_name=cg16863382	feature_id[2922].value > threshold=0.7200668156147003
node_133: feature_name=cg06989443	feature_id[2327].value <= threshold=0.6579365730285645

node_134: feature_name=cg07344990	feature_id[2398].value > threshold=0.5562876164913177
node_138: feature_name=cg02966841	feature_id[2199].value > threshold=0.7744501233100891
node_204: feature_name=cg12120430	feature_id[3727].value > threshold=0.5775820016860962
node_206: feature_name=cg18386876	feature_id[4066].value > threshold=0.6847257018089294
node_208: feature_name=cg02053092	feature_id[3610].value > threshold=0.39507485926151276
node_210: feature_name=cg10298992	feature_id[3603].value > threshold=0.5707973837852478
node_228: feature_name=cg03840920	feature_id[4524].value > threshold=0.5365143716335297
node_234: feature_name=cg25364972	feature_id[1792].value > threshold=0.2418053299188614
node_238: feature_name=cg23485307	feature_id[221].value > threshold=0.14884746074676514
node_242: feature_name=cg04873169	feature_id[4696].value > threshold=0.6058756709098816
node_244: feature_name=cg17009731	feature_id[3218].value > threshold=0.6602252125740051
node_246: feature_name=cg18253910	feature_id[4458].value > threshold=0.6904322504997253
node_250: feature_name=cg04774476	feature_id[1257].value > threshold=0.5675267577171326
node_256: feature_name=cg19259111	feature_id[63].value > threshold=0.40771469473838806
node_264: feature_name=cg21483216	feature_id[5253].value > threshold=0.6332719326019287
node_266: feature_name=cg04026354	feature_id[3502].value > threshold=0.42332448065280914
node_336: feature_name=cg05824594	feature_id[1431].value <= threshold=0.629401296377182
node_337: feature_name=cg18475969	feature_id[2117].value > threshold=0.3116971254348755
node_351: feature_name=cg22286382	feature_id[2821].value > threshold=0.6607459187507629
Class: well differentiated liposarcoma (WDLS)/dedifferentiated liposarcoma (DDLS)	
Rules_118	passed counts:1

node_0: feature_name=cg11915444	feature_id[2002].value > threshold=0.3402601182460785
node_10: feature_name=cg26016985	feature_id[4407].value > threshold=0.6493876278400421
node_16: feature_name=cg23157618	feature_id[5511].value > threshold=0.5759606957435608
node_20: feature_name=cg10480329	feature_id[1439].value > threshold=0.5518321692943573
node_48: feature_name=cg17843418	feature_id[4868].value > threshold=0.34969255328178406
node_52: feature_name=cg24407065	feature_id[4522].value > threshold=0.5364363789558411
node_56: feature_name=cg07281938	feature_id[249].value > threshold=0.7506992518901825
node_78: feature_name=cg21759907	feature_id[1650].value > threshold=0.46823520958423615
node_98: feature_name=cg10886334	feature_id[3975].value > threshold=0.43698127567768097
node_102: feature_name=cg09373983	feature_id[5025].value > threshold=0.5575815737247467
node_106: feature_name=cg12948116	feature_id[5360].value <= threshold=0.5658791363239288
node_107: feature_name=cg21906519	feature_id[5251].value > threshold=0.6089984774589539
node_111: feature_name=cg17537493	feature_id[4217].value > threshold=0.5825372636318207
node_117: feature_name=cg15720017	feature_id[4443].value > threshold=0.7009969055652618
node_119: feature_name=cg06038180	feature_id[3480].value > threshold=0.4583848565816879
node_121: feature_name=cg08619651	feature_id[4882].value <= threshold=0.2105495035648346
node_122: feature_name=cg01014262	feature_id[1911].value <= threshold=0.5327437818050385
node_123: feature_name=cg16863382	feature_id[2922].value > threshold=0.7200668156147003
node_133: feature_name=cg06989443	feature_id[2327].value <= threshold=0.6579365730285645
node_134: feature_name=cg07344990	feature_id[2398].value > threshold=0.5562876164913177
node_138: feature_name=cg02966841	feature_id[2199].value > threshold=0.7744501233100891
node_204: feature_name=cg12120430	feature_id[3727].value > threshold=0.5775820016860962

node_206: feature_name=cg18386876	feature_id[4066].value > threshold=0.6847257018089294
node_208: feature_name=cg02053092	feature_id[3610].value > threshold=0.39507485926151276
node_210: feature_name=cg10298992	feature_id[3603].value > threshold=0.5707973837852478
node_228: feature_name=cg03840920	feature_id[4524].value > threshold=0.5365143716335297
node_234: feature_name=cg25364972	feature_id[1792].value > threshold=0.2418053299188614
node_238: feature_name=cg23485307	feature_id[221].value > threshold=0.14884746074676514
node_242: feature_name=cg04873169	feature_id[4696].value > threshold=0.6058756709098816
node_244: feature_name=cg17009731	feature_id[3218].value > threshold=0.6602252125740051
node_246: feature_name=cg18253910	feature_id[4458].value > threshold=0.6904322504997253
node_250: feature_name=cg04774476	feature_id[1257].value > threshold=0.5675267577171326
node_256: feature_name=cg19259111	feature_id[63].value > threshold=0.40771469473838806
node_264: feature_name=cg21483216	feature_id[5253].value > threshold=0.6332719326019287
node_266: feature_name=cg04026354	feature_id[3502].value > threshold=0.42332448065280914
node_336: feature_name=cg05824594	feature_id[1431].value <= threshold=0.629401296377182
node_337: feature_name=cg18475969	feature_id[2117].value <= threshold=0.3116971254348755
node_338: feature_name=cg19494591	feature_id[2196].value > threshold=0.7268106639385223
node_340: feature_name=cg09075515	feature_id[1529].value > threshold=0.5144184827804565
node_342: feature_name=cg09322899	feature_id[2281].value <= threshold=0.9401540160179138
node_343: feature_name=cg19382919	feature_id[4392].value > threshold=0.0787786990404129
node_345: feature_name=cg06371044	feature_id[2455].value > threshold=0.7931062579154968
node_347: feature_name=cg27470978	feature_id[4804].value > threshold=0.8740440309047699
Class: angiomatoid fibrous histiocytoma (AFH)	

Rules_119	passed counts:1
node_0: feature_name=cg11915444	feature_id[2002].value > threshold=0.3402601182460785
node_10: feature_name=cg26016985	feature_id[4407].value > threshold=0.6493876278400421
node_16: feature_name=cg23157618	feature_id[5511].value > threshold=0.5759606957435608
node_20: feature_name=cg10480329	feature_id[1439].value > threshold=0.5518321692943573
node_48: feature_name=cg17843418	feature_id[4868].value > threshold=0.34969255328178406
node_52: feature_name=cg24407065	feature_id[4522].value > threshold=0.5364363789558411
node_56: feature_name=cg07281938	feature_id[249].value > threshold=0.7506992518901825
node_78: feature_name=cg21759907	feature_id[1650].value > threshold=0.46823520958423615
node_98: feature_name=cg10886334	feature_id[3975].value > threshold=0.43698127567768097
node_102: feature_name=cg09373983	feature_id[5025].value > threshold=0.5575815737247467
node_106: feature_name=cg12948116	feature_id[5360].value <= threshold=0.5658791363239288
node_107: feature_name=cg21906519	feature_id[5251].value > threshold=0.6089984774589539
node_111: feature_name=cg17537493	feature_id[4217].value > threshold=0.5825372636318207
node_117: feature_name=cg15720017	feature_id[4443].value > threshold=0.7009969055652618
node_119: feature_name=cg06038180	feature_id[3480].value > threshold=0.4583848565816879
node_121: feature_name=cg08619651	feature_id[4882].value <= threshold=0.2105495035648346
node_122: feature_name=cg01014262	feature_id[1911].value <= threshold=0.5327437818050385
node_123: feature_name=cg16863382	feature_id[2922].value > threshold=0.7200668156147003
node_133: feature_name=cg06989443	feature_id[2327].value <= threshold=0.6579365730285645
node_134: feature_name=cg07344990	feature_id[2398].value > threshold=0.5562876164913177

node_138: feature_name=cg02966841	feature_id[2199].value > threshold=0.7744501233100891
node_204: feature_name=cg12120430	feature_id[3727].value > threshold=0.5775820016860962
node_206: feature_name=cg18386876	feature_id[4066].value > threshold=0.6847257018089294
node_208: feature_name=cg02053092	feature_id[3610].value > threshold=0.39507485926151276
node_210: feature_name=cg10298992	feature_id[3603].value > threshold=0.5707973837852478
node_228: feature_name=cg03840920	feature_id[4524].value > threshold=0.5365143716335297
node_234: feature_name=cg25364972	feature_id[1792].value > threshold=0.2418053299188614
node_238: feature_name=cg23485307	feature_id[221].value > threshold=0.14884746074676514
node_242: feature_name=cg04873169	feature_id[4696].value > threshold=0.6058756709098816
node_244: feature_name=cg17009731	feature_id[3218].value > threshold=0.6602252125740051
node_246: feature_name=cg18253910	feature_id[4458].value > threshold=0.6904322504997253
node_250: feature_name=cg04774476	feature_id[1257].value > threshold=0.5675267577171326
node_256: feature_name=cg19259111	feature_id[63].value > threshold=0.40771469473838806
node_264: feature_name=cg21483216	feature_id[5253].value > threshold=0.6332719326019287
node_266: feature_name=cg04026354	feature_id[3502].value > threshold=0.42332448065280914
node_336: feature_name=cg05824594	feature_id[1431].value <= threshold=0.629401296377182
node_337: feature_name=cg18475969	feature_id[2117].value <= threshold=0.3116971254348755
node_338: feature_name=cg19494591	feature_id[2196].value > threshold=0.7268106639385223
node_340: feature_name=cg09075515	feature_id[1529].value > threshold=0.5144184827804565
node_342: feature_name=cg09322899	feature_id[2281].value <= threshold=0.9401540160179138
node_343: feature_name=cg19382919	feature_id[4392].value > threshold=0.0787786990404129
node_345: feature_name=cg06371044	feature_id[2455].value > threshold=0.7931062579154968

node_347: feature_name=cg27470978	feature_id[4804].value <= threshold=0.8740440309047699
Class: giant cell tumour of bone (GCTB)	
Rules_120	passed counts:1
node_0: feature_name=cg11915444	feature_id[2002].value > threshold=0.3402601182460785
node_10: feature_name=cg26016985	feature_id[4407].value > threshold=0.6493876278400421
node_16: feature_name=cg23157618	feature_id[5511].value > threshold=0.5759606957435608
node_20: feature_name=cg10480329	feature_id[1439].value > threshold=0.5518321692943573
node_48: feature_name=cg17843418	feature_id[4868].value > threshold=0.34969255328178406
node_52: feature_name=cg24407065	feature_id[4522].value > threshold=0.5364363789558411
node_56: feature_name=cg07281938	feature_id[249].value > threshold=0.7506992518901825
node_78: feature_name=cg21759907	feature_id[1650].value > threshold=0.46823520958423615
node_98: feature_name=cg10886334	feature_id[3975].value > threshold=0.43698127567768097
node_102: feature_name=cg09373983	feature_id[5025].value > threshold=0.5575815737247467
node_106: feature_name=cg12948116	feature_id[5360].value <= threshold=0.5658791363239288
node_107: feature_name=cg21906519	feature_id[5251].value > threshold=0.6089984774589539
node_111: feature_name=cg17537493	feature_id[4217].value > threshold=0.5825372636318207
node_117: feature_name=cg15720017	feature_id[4443].value > threshold=0.7009969055652618
node_119: feature_name=cg06038180	feature_id[3480].value > threshold=0.4583848565816879
node_121: feature_name=cg08619651	feature_id[4882].value <= threshold=0.2105495035648346
node_122: feature_name=cg01014262	feature_id[1911].value <= threshold=0.5327437818050385
node_123: feature_name=cg16863382	feature_id[2922].value > threshold=0.7200668156147003
node_133: feature_name=cg06989443	feature_id[2327].value <= threshold=0.6579365730285645

node_134: feature_name=cg07344990	feature_id[2398].value > threshold=0.5562876164913177
node_138: feature_name=cg02966841	feature_id[2199].value > threshold=0.7744501233100891
node_204: feature_name=cg12120430	feature_id[3727].value > threshold=0.5775820016860962
node_206: feature_name=cg18386876	feature_id[4066].value > threshold=0.6847257018089294
node_208: feature_name=cg02053092	feature_id[3610].value > threshold=0.39507485926151276
node_210: feature_name=cg10298992	feature_id[3603].value > threshold=0.5707973837852478
node_228: feature_name=cg03840920	feature_id[4524].value > threshold=0.5365143716335297
node_234: feature_name=cg25364972	feature_id[1792].value > threshold=0.2418053299188614
node_238: feature_name=cg23485307	feature_id[221].value > threshold=0.14884746074676514
node_242: feature_name=cg04873169	feature_id[4696].value > threshold=0.6058756709098816
node_244: feature_name=cg17009731	feature_id[3218].value > threshold=0.6602252125740051
node_246: feature_name=cg18253910	feature_id[4458].value > threshold=0.6904322504997253
node_250: feature_name=cg04774476	feature_id[1257].value > threshold=0.5675267577171326
node_256: feature_name=cg19259111	feature_id[63].value > threshold=0.40771469473838806
node_264: feature_name=cg21483216	feature_id[5253].value > threshold=0.6332719326019287
node_266: feature_name=cg04026354	feature_id[3502].value > threshold=0.42332448065280914
node_336: feature_name=cg05824594	feature_id[1431].value <= threshold=0.629401296377182
node_337: feature_name=cg18475969	feature_id[2117].value <= threshold=0.3116971254348755
node_338: feature_name=cg19494591	feature_id[2196].value > threshold=0.7268106639385223
node_340: feature_name=cg09075515	feature_id[1529].value > threshold=0.5144184827804565
node_342: feature_name=cg09322899	feature_id[2281].value <= threshold=0.9401540160179138
node_343: feature_name=cg19382919	feature_id[4392].value > threshold=0.0787786990404129

node_345: feature_name=cg06371044	feature_id[2455].value <= threshold=0.7931062579154968
Class: sarcoma (SARC)	
Rules_121	passed counts:1
node_0: feature_name=cg11915444	feature_id[2002].value > threshold=0.3402601182460785
node_10: feature_name=cg26016985	feature_id[4407].value > threshold=0.6493876278400421
node_16: feature_name=cg23157618	feature_id[5511].value > threshold=0.5759606957435608
node_20: feature_name=cg10480329	feature_id[1439].value > threshold=0.5518321692943573
node_48: feature_name=cg17843418	feature_id[4868].value > threshold=0.34969255328178406
node_52: feature_name=cg24407065	feature_id[4522].value > threshold=0.5364363789558411
node_56: feature_name=cg07281938	feature_id[249].value > threshold=0.7506992518901825
node_78: feature_name=cg21759907	feature_id[1650].value > threshold=0.46823520958423615
node_98: feature_name=cg10886334	feature_id[3975].value > threshold=0.43698127567768097
node_102: feature_name=cg09373983	feature_id[5025].value > threshold=0.5575815737247467
node_106: feature_name=cg12948116	feature_id[5360].value <= threshold=0.5658791363239288
node_107: feature_name=cg21906519	feature_id[5251].value > threshold=0.6089984774589539
node_111: feature_name=cg17537493	feature_id[4217].value > threshold=0.5825372636318207
node_117: feature_name=cg15720017	feature_id[4443].value > threshold=0.7009969055652618
node_119: feature_name=cg06038180	feature_id[3480].value > threshold=0.4583848565816879
node_121: feature_name=cg08619651	feature_id[4882].value <= threshold=0.2105495035648346
node_122: feature_name=cg01014262	feature_id[1911].value <= threshold=0.5327437818050385
node_123: feature_name=cg16863382	feature_id[2922].value > threshold=0.7200668156147003
node_133: feature_name=cg06989443	feature_id[2327].value <= threshold=0.6579365730285645

node_134: feature_name=cg07344990	feature_id[2398].value > threshold=0.5562876164913177
node_138: feature_name=cg02966841	feature_id[2199].value > threshold=0.7744501233100891
node_204: feature_name=cg12120430	feature_id[3727].value > threshold=0.5775820016860962
node_206: feature_name=cg18386876	feature_id[4066].value > threshold=0.6847257018089294
node_208: feature_name=cg02053092	feature_id[3610].value > threshold=0.39507485926151276
node_210: feature_name=cg10298992	feature_id[3603].value > threshold=0.5707973837852478
node_228: feature_name=cg03840920	feature_id[4524].value > threshold=0.5365143716335297
node_234: feature_name=cg25364972	feature_id[1792].value > threshold=0.2418053299188614
node_238: feature_name=cg23485307	feature_id[221].value > threshold=0.14884746074676514
node_242: feature_name=cg04873169	feature_id[4696].value > threshold=0.6058756709098816
node_244: feature_name=cg17009731	feature_id[3218].value > threshold=0.6602252125740051
node_246: feature_name=cg18253910	feature_id[4458].value > threshold=0.6904322504997253
node_250: feature_name=cg04774476	feature_id[1257].value > threshold=0.5675267577171326
node_256: feature_name=cg19259111	feature_id[63].value > threshold=0.40771469473838806
node_264: feature_name=cg21483216	feature_id[5253].value > threshold=0.6332719326019287
node_266: feature_name=cg04026354	feature_id[3502].value > threshold=0.42332448065280914
node_336: feature_name=cg05824594	feature_id[1431].value <= threshold=0.629401296377182
node_337: feature_name=cg18475969	feature_id[2117].value <= threshold=0.3116971254348755
node_338: feature_name=cg19494591	feature_id[2196].value > threshold=0.7268106639385223
node_340: feature_name=cg09075515	feature_id[1529].value > threshold=0.5144184827804565
node_342: feature_name=cg09322899	feature_id[2281].value <= threshold=0.9401540160179138
node_343: feature_name=cg19382919	feature_id[4392].value <= threshold=0.0787786990404129

Class: angiosarcoma (AS)	
Rules_122	passed counts:1
node_0: feature_name=cg11915444	feature_id[2002].value > threshold=0.3402601182460785
node_10: feature_name=cg26016985	feature_id[4407].value > threshold=0.6493876278400421
node_16: feature_name=cg23157618	feature_id[5511].value > threshold=0.5759606957435608
node_20: feature_name=cg10480329	feature_id[1439].value > threshold=0.5518321692943573
node_48: feature_name=cg17843418	feature_id[4868].value > threshold=0.34969255328178406
node_52: feature_name=cg24407065	feature_id[4522].value > threshold=0.5364363789558411
node_56: feature_name=cg07281938	feature_id[249].value > threshold=0.7506992518901825
node_78: feature_name=cg21759907	feature_id[1650].value > threshold=0.46823520958423615
node_98: feature_name=cg10886334	feature_id[3975].value > threshold=0.43698127567768097
node_102: feature_name=cg09373983	feature_id[5025].value > threshold=0.5575815737247467
node_106: feature_name=cg12948116	feature_id[5360].value <= threshold=0.5658791363239288
node_107: feature_name=cg21906519	feature_id[5251].value > threshold=0.6089984774589539
node_111: feature_name=cg17537493	feature_id[4217].value > threshold=0.5825372636318207
node_117: feature_name=cg15720017	feature_id[4443].value > threshold=0.7009969055652618
node_119: feature_name=cg06038180	feature_id[3480].value > threshold=0.4583848565816879
node_121: feature_name=cg08619651	feature_id[4882].value <= threshold=0.2105495035648346
node_122: feature_name=cg01014262	feature_id[1911].value <= threshold=0.5327437818050385
node_123: feature_name=cg16863382	feature_id[2922].value > threshold=0.7200668156147003
node_133: feature_name=cg06989443	feature_id[2327].value <= threshold=0.6579365730285645
node_134: feature_name=cg07344990	feature_id[2398].value > threshold=0.5562876164913177

node_138: feature_name=cg02966841	feature_id[2199].value > threshold=0.7744501233100891
node_204: feature_name=cg12120430	feature_id[3727].value > threshold=0.5775820016860962
node_206: feature_name=cg18386876	feature_id[4066].value > threshold=0.6847257018089294
node_208: feature_name=cg02053092	feature_id[3610].value > threshold=0.39507485926151276
node_210: feature_name=cg10298992	feature_id[3603].value > threshold=0.5707973837852478
node_228: feature_name=cg03840920	feature_id[4524].value > threshold=0.5365143716335297
node_234: feature_name=cg25364972	feature_id[1792].value > threshold=0.2418053299188614
node_238: feature_name=cg23485307	feature_id[221].value > threshold=0.14884746074676514
node_242: feature_name=cg04873169	feature_id[4696].value > threshold=0.6058756709098816
node_244: feature_name=cg17009731	feature_id[3218].value > threshold=0.6602252125740051
node_246: feature_name=cg18253910	feature_id[4458].value > threshold=0.6904322504997253
node_250: feature_name=cg04774476	feature_id[1257].value > threshold=0.5675267577171326
node_256: feature_name=cg19259111	feature_id[63].value > threshold=0.40771469473838806
node_264: feature_name=cg21483216	feature_id[5253].value > threshold=0.6332719326019287
node_266: feature_name=cg04026354	feature_id[3502].value <= threshold=0.42332448065280914
node_267: feature_name=cg11444072	feature_id[116].value > threshold=0.4940422773361206
node_273: feature_name=cg08571020	feature_id[381].value > threshold=0.4616774618625641
node_275: feature_name=cg08331427	feature_id[2081].value > threshold=0.5443233847618103
node_281: feature_name=cg00216961	feature_id[945].value <= threshold=0.4166988283395767
node_282: feature_name=cg04716580	feature_id[1390].value > threshold=0.4204266667366028
node_286: feature_name=cg07891483	feature_id[1877].value > threshold=0.829958975315094
node_288: feature_name=cg01475325	feature_id[3969].value > threshold=0.45323844254016876

node_292: feature_name=cg19702397	feature_id[2066].value > threshold=0.3536848723888397
node_294: feature_name=cg21936959	feature_id[595].value > threshold=0.5567200481891632
node_308: feature_name=cg18645642	feature_id[262].value > threshold=0.42608243227005005
node_310: feature_name=cg27351239	feature_id[1874].value > threshold=0.6790103316307068
node_330: feature_name=cg18252102	feature_id[1205].value > threshold=0.5534502267837524
Class: Ewing sarcoma (EWING)	
Rules_123	passed counts:1
node_0: feature_name=cg11915444	feature_id[2002].value > threshold=0.3402601182460785
node_10: feature_name=cg26016985	feature_id[4407].value > threshold=0.6493876278400421
node_16: feature_name=cg23157618	feature_id[5511].value > threshold=0.5759606957435608
node_20: feature_name=cg10480329	feature_id[1439].value > threshold=0.5518321692943573
node_48: feature_name=cg17843418	feature_id[4868].value > threshold=0.34969255328178406
node_52: feature_name=cg24407065	feature_id[4522].value > threshold=0.5364363789558411
node_56: feature_name=cg07281938	feature_id[249].value > threshold=0.7506992518901825
node_78: feature_name=cg21759907	feature_id[1650].value > threshold=0.46823520958423615
node_98: feature_name=cg10886334	feature_id[3975].value > threshold=0.43698127567768097
node_102: feature_name=cg09373983	feature_id[5025].value > threshold=0.5575815737247467
node_106: feature_name=cg12948116	feature_id[5360].value <= threshold=0.5658791363239288
node_107: feature_name=cg21906519	feature_id[5251].value > threshold=0.6089984774589539
node_111: feature_name=cg17537493	feature_id[4217].value > threshold=0.5825372636318207
node_117: feature_name=cg15720017	feature_id[4443].value > threshold=0.7009969055652618
node_119: feature_name=cg06038180	feature_id[3480].value > threshold=0.4583848565816879

node_121: feature_name=cg08619651	feature_id[4882].value <= threshold=0.2105495035648346
node_122: feature_name=cg01014262	feature_id[1911].value <= threshold=0.5327437818050385
node_123: feature_name=cg16863382	feature_id[2922].value > threshold=0.7200668156147003
node_133: feature_name=cg06989443	feature_id[2327].value <= threshold=0.6579365730285645
node_134: feature_name=cg07344990	feature_id[2398].value > threshold=0.5562876164913177
node_138: feature_name=cg02966841	feature_id[2199].value > threshold=0.7744501233100891
node_204: feature_name=cg12120430	feature_id[3727].value > threshold=0.5775820016860962
node_206: feature_name=cg18386876	feature_id[4066].value > threshold=0.6847257018089294
node_208: feature_name=cg02053092	feature_id[3610].value > threshold=0.39507485926151276
node_210: feature_name=cg10298992	feature_id[3603].value > threshold=0.5707973837852478
node_228: feature_name=cg03840920	feature_id[4524].value > threshold=0.5365143716335297
node_234: feature_name=cg25364972	feature_id[1792].value > threshold=0.2418053299188614
node_238: feature_name=cg23485307	feature_id[221].value > threshold=0.14884746074676514
node_242: feature_name=cg04873169	feature_id[4696].value > threshold=0.6058756709098816
node_244: feature_name=cg17009731	feature_id[3218].value > threshold=0.6602252125740051
node_246: feature_name=cg18253910	feature_id[4458].value > threshold=0.6904322504997253
node_250: feature_name=cg04774476	feature_id[1257].value > threshold=0.5675267577171326
node_256: feature_name=cg19259111	feature_id[63].value > threshold=0.40771469473838806
node_264: feature_name=cg21483216	feature_id[5253].value > threshold=0.6332719326019287
node_266: feature_name=cg04026354	feature_id[3502].value <= threshold=0.42332448065280914
node_267: feature_name=cg11444072	feature_id[116].value > threshold=0.4940422773361206
node_273: feature_name=cg08571020	feature_id[381].value > threshold=0.4616774618625641

node_275: feature_name=cg08331427	feature_id[2081].value > threshold=0.5443233847618103
node_281: feature_name=cg00216961	feature_id[945].value <= threshold=0.4166988283395767
node_282: feature_name=cg04716580	feature_id[1390].value > threshold=0.4204266667366028
node_286: feature_name=cg07891483	feature_id[1877].value > threshold=0.829958975315094
node_288: feature_name=cg01475325	feature_id[3969].value > threshold=0.45323844254016876
node_292: feature_name=cg19702397	feature_id[2066].value > threshold=0.3536848723888397
node_294: feature_name=cg21936959	feature_id[595].value > threshold=0.5567200481891632
node_308: feature_name=cg18645642	feature_id[262].value > threshold=0.42608243227005005
node_310: feature_name=cg27351239	feature_id[1874].value <= threshold=0.6790103316307068
node_311: feature_name=cg13107973	feature_id[1326].value <= threshold=0.6562457382678986
node_312: feature_name=cg10024583	feature_id[1175].value <= threshold=0.518094539642334
node_313: feature_name=cg18259342	feature_id[4480].value > threshold=0.19059929996728897
node_315: feature_name=cg10979364	feature_id[992].value > threshold=0.44472505152225494
node_317: feature_name=cg14406727	feature_id[815].value > threshold=0.36684730648994446
node_319: feature_name=cg24926689	feature_id[2976].value > threshold=0.822247177362442
node_321: feature_name=cg11535638	feature_id[2812].value <= threshold=0.7990793883800507
node_322: feature_name=cg13039539	feature_id[4333].value > threshold=0.8452905118465424
node_324: feature_name=cg09956907	feature_id[5470].value > threshold=0.16951685398817062
Class: alveolar soft part sarcoma (ASPS)	
Rules_124	passed counts:1
node_0: feature_name=cg11915444	feature_id[2002].value > threshold=0.3402601182460785
node_10: feature_name=cg26016985	feature_id[4407].value > threshold=0.6493876278400421

node_16: feature_name=cg23157618	feature_id[5511].value > threshold=0.5759606957435608
node_20: feature_name=cg10480329	feature_id[1439].value > threshold=0.5518321692943573
node_48: feature_name=cg17843418	feature_id[4868].value > threshold=0.34969255328178406
node_52: feature_name=cg24407065	feature_id[4522].value > threshold=0.5364363789558411
node_56: feature_name=cg07281938	feature_id[249].value > threshold=0.7506992518901825
node_78: feature_name=cg21759907	feature_id[1650].value > threshold=0.46823520958423615
node_98: feature_name=cg10886334	feature_id[3975].value > threshold=0.43698127567768097
node_102: feature_name=cg09373983	feature_id[5025].value > threshold=0.5575815737247467
node_106: feature_name=cg12948116	feature_id[5360].value <= threshold=0.5658791363239288
node_107: feature_name=cg21906519	feature_id[5251].value > threshold=0.6089984774589539
node_111: feature_name=cg17537493	feature_id[4217].value > threshold=0.5825372636318207
node_117: feature_name=cg15720017	feature_id[4443].value > threshold=0.7009969055652618
node_119: feature_name=cg06038180	feature_id[3480].value > threshold=0.4583848565816879
node_121: feature_name=cg08619651	feature_id[4882].value <= threshold=0.2105495035648346
node_122: feature_name=cg01014262	feature_id[1911].value <= threshold=0.5327437818050385
node_123: feature_name=cg16863382	feature_id[2922].value > threshold=0.7200668156147003
node_133: feature_name=cg06989443	feature_id[2327].value <= threshold=0.6579365730285645
node_134: feature_name=cg07344990	feature_id[2398].value > threshold=0.5562876164913177
node_138: feature_name=cg02966841	feature_id[2199].value > threshold=0.7744501233100891
node_204: feature_name=cg12120430	feature_id[3727].value > threshold=0.5775820016860962
node_206: feature_name=cg18386876	feature_id[4066].value > threshold=0.6847257018089294
node_208: feature_name=cg02053092	feature_id[3610].value > threshold=0.39507485926151276

node_210: feature_name=cg10298992	feature_id[3603].value > threshold=0.5707973837852478
node_228: feature_name=cg03840920	feature_id[4524].value > threshold=0.5365143716335297
node_234: feature_name=cg25364972	feature_id[1792].value > threshold=0.2418053299188614
node_238: feature_name=cg23485307	feature_id[221].value > threshold=0.14884746074676514
node_242: feature_name=cg04873169	feature_id[4696].value > threshold=0.6058756709098816
node_244: feature_name=cg17009731	feature_id[3218].value > threshold=0.6602252125740051
node_246: feature_name=cg18253910	feature_id[4458].value > threshold=0.6904322504997253
node_250: feature_name=cg04774476	feature_id[1257].value > threshold=0.5675267577171326
node_256: feature_name=cg19259111	feature_id[63].value > threshold=0.40771469473838806
node_264: feature_name=cg21483216	feature_id[5253].value > threshold=0.6332719326019287
node_266: feature_name=cg04026354	feature_id[3502].value <= threshold=0.42332448065280914
node_267: feature_name=cg11444072	feature_id[116].value > threshold=0.4940422773361206
node_273: feature_name=cg08571020	feature_id[381].value > threshold=0.4616774618625641
node_275: feature_name=cg08331427	feature_id[2081].value > threshold=0.5443233847618103
node_281: feature_name=cg00216961	feature_id[945].value <= threshold=0.4166988283395767
node_282: feature_name=cg04716580	feature_id[1390].value > threshold=0.4204266667366028
node_286: feature_name=cg07891483	feature_id[1877].value > threshold=0.829958975315094
node_288: feature_name=cg01475325	feature_id[3969].value > threshold=0.45323844254016876
node_292: feature_name=cg19702397	feature_id[2066].value > threshold=0.3536848723888397
node_294: feature_name=cg21936959	feature_id[595].value > threshold=0.5567200481891632
node_308: feature_name=cg18645642	feature_id[262].value > threshold=0.42608243227005005
node_310: feature_name=cg27351239	feature_id[1874].value <= threshold=0.6790103316307068

node_311: feature_name=cg13107973	feature_id[1326].value <= threshold=0.6562457382678986
node_312: feature_name=cg10024583	feature_id[1175].value <= threshold=0.518094539642334
node_313: feature_name=cg18259342	feature_id[4480].value > threshold=0.19059929996728897
node_315: feature_name=cg10979364	feature_id[992].value > threshold=0.44472505152225494
node_317: feature_name=cg14406727	feature_id[815].value > threshold=0.36684730648994446
node_319: feature_name=cg24926689	feature_id[2976].value > threshold=0.822247177362442
node_321: feature_name=cg11535638	feature_id[2812].value <= threshold=0.7990793883800507
node_322: feature_name=cg13039539	feature_id[4333].value > threshold=0.8452905118465424
node_324: feature_name=cg09956907	feature_id[5470].value <= threshold=0.16951685398817062
Class: infantile fibrosarcoma (IFS)	
Rules_125	passed counts:1
node_0: feature_name=cg11915444	feature_id[2002].value > threshold=0.3402601182460785
node_10: feature_name=cg26016985	feature_id[4407].value > threshold=0.6493876278400421
node_16: feature_name=cg23157618	feature_id[5511].value > threshold=0.5759606957435608
node_20: feature_name=cg10480329	feature_id[1439].value > threshold=0.5518321692943573
node_48: feature_name=cg17843418	feature_id[4868].value > threshold=0.34969255328178406
node_52: feature_name=cg24407065	feature_id[4522].value > threshold=0.5364363789558411
node_56: feature_name=cg07281938	feature_id[249].value > threshold=0.7506992518901825
node_78: feature_name=cg21759907	feature_id[1650].value > threshold=0.46823520958423615
node_98: feature_name=cg10886334	feature_id[3975].value > threshold=0.43698127567768097
node_102: feature_name=cg09373983	feature_id[5025].value > threshold=0.5575815737247467
node_106: feature_name=cg12948116	feature_id[5360].value <= threshold=0.5658791363239288

node_107: feature_name=cg21906519	feature_id[5251].value > threshold=0.6089984774589539
node_111: feature_name=cg17537493	feature_id[4217].value > threshold=0.5825372636318207
node_117: feature_name=cg15720017	feature_id[4443].value > threshold=0.7009969055652618
node_119: feature_name=cg06038180	feature_id[3480].value > threshold=0.4583848565816879
node_121: feature_name=cg08619651	feature_id[4882].value <= threshold=0.2105495035648346
node_122: feature_name=cg01014262	feature_id[1911].value <= threshold=0.5327437818050385
node_123: feature_name=cg16863382	feature_id[2922].value > threshold=0.7200668156147003
node_133: feature_name=cg06989443	feature_id[2327].value <= threshold=0.6579365730285645
node_134: feature_name=cg07344990	feature_id[2398].value > threshold=0.5562876164913177
node_138: feature_name=cg02966841	feature_id[2199].value > threshold=0.7744501233100891
node_204: feature_name=cg12120430	feature_id[3727].value > threshold=0.5775820016860962
node_206: feature_name=cg18386876	feature_id[4066].value > threshold=0.6847257018089294
node_208: feature_name=cg02053092	feature_id[3610].value > threshold=0.39507485926151276
node_210: feature_name=cg10298992	feature_id[3603].value > threshold=0.5707973837852478
node_228: feature_name=cg03840920	feature_id[4524].value > threshold=0.5365143716335297
node_234: feature_name=cg25364972	feature_id[1792].value > threshold=0.2418053299188614
node_238: feature_name=cg23485307	feature_id[221].value > threshold=0.14884746074676514
node_242: feature_name=cg04873169	feature_id[4696].value > threshold=0.6058756709098816
node_244: feature_name=cg17009731	feature_id[3218].value > threshold=0.6602252125740051
node_246: feature_name=cg18253910	feature_id[4458].value > threshold=0.6904322504997253
node_250: feature_name=cg04774476	feature_id[1257].value > threshold=0.5675267577171326
node_256: feature_name=cg19259111	feature_id[63].value > threshold=0.40771469473838806

node_264: feature_name=cg21483216	feature_id[5253].value > threshold=0.6332719326019287
node_266: feature_name=cg04026354	feature_id[3502].value <= threshold=0.42332448065280914
node_267: feature_name=cg11444072	feature_id[116].value > threshold=0.4940422773361206
node_273: feature_name=cg08571020	feature_id[381].value > threshold=0.4616774618625641
node_275: feature_name=cg08331427	feature_id[2081].value > threshold=0.5443233847618103
node_281: feature_name=cg00216961	feature_id[945].value <= threshold=0.4166988283395767
node_282: feature_name=cg04716580	feature_id[1390].value > threshold=0.4204266667366028
node_286: feature_name=cg07891483	feature_id[1877].value > threshold=0.829958975315094
node_288: feature_name=cg01475325	feature_id[3969].value > threshold=0.45323844254016876
node_292: feature_name=cg19702397	feature_id[2066].value > threshold=0.3536848723888397
node_294: feature_name=cg21936959	feature_id[595].value > threshold=0.5567200481891632
node_308: feature_name=cg18645642	feature_id[262].value > threshold=0.42608243227005005
node_310: feature_name=cg27351239	feature_id[1874].value <= threshold=0.6790103316307068
node_311: feature_name=cg13107973	feature_id[1326].value <= threshold=0.6562457382678986
node_312: feature_name=cg10024583	feature_id[1175].value <= threshold=0.518094539642334
node_313: feature_name=cg18259342	feature_id[4480].value > threshold=0.19059929996728897
node_315: feature_name=cg10979364	feature_id[992].value > threshold=0.44472505152225494
node_317: feature_name=cg14406727	feature_id[815].value > threshold=0.36684730648994446
node_319: feature_name=cg24926689	feature_id[2976].value > threshold=0.822247177362442
node_321: feature_name=cg11535638	feature_id[2812].value <= threshold=0.7990793883800507
node_322: feature_name=cg13039539	feature_id[4333].value <= threshold=0.8452905118465424
Class: synovial sarcoma (SYSA)	

Rules_126	passed counts:1
node_0: feature_name=cg11915444	feature_id[2002].value > threshold=0.3402601182460785
node_10: feature_name=cg26016985	feature_id[4407].value > threshold=0.6493876278400421
node_16: feature_name=cg23157618	feature_id[5511].value > threshold=0.5759606957435608
node_20: feature_name=cg10480329	feature_id[1439].value > threshold=0.5518321692943573
node_48: feature_name=cg17843418	feature_id[4868].value > threshold=0.34969255328178406
node_52: feature_name=cg24407065	feature_id[4522].value > threshold=0.5364363789558411
node_56: feature_name=cg07281938	feature_id[249].value > threshold=0.7506992518901825
node_78: feature_name=cg21759907	feature_id[1650].value > threshold=0.46823520958423615
node_98: feature_name=cg10886334	feature_id[3975].value > threshold=0.43698127567768097
node_102: feature_name=cg09373983	feature_id[5025].value > threshold=0.5575815737247467
node_106: feature_name=cg12948116	feature_id[5360].value <= threshold=0.5658791363239288
node_107: feature_name=cg21906519	feature_id[5251].value > threshold=0.6089984774589539
node_111: feature_name=cg17537493	feature_id[4217].value > threshold=0.5825372636318207
node_117: feature_name=cg15720017	feature_id[4443].value > threshold=0.7009969055652618
node_119: feature_name=cg06038180	feature_id[3480].value > threshold=0.4583848565816879
node_121: feature_name=cg08619651	feature_id[4882].value <= threshold=0.2105495035648346
node_122: feature_name=cg01014262	feature_id[1911].value <= threshold=0.5327437818050385
node_123: feature_name=cg16863382	feature_id[2922].value > threshold=0.7200668156147003
node_133: feature_name=cg06989443	feature_id[2327].value <= threshold=0.6579365730285645
node_134: feature_name=cg07344990	feature_id[2398].value > threshold=0.5562876164913177

node_138: feature_name=cg02966841	feature_id[2199].value > threshold=0.7744501233100891
node_204: feature_name=cg12120430	feature_id[3727].value > threshold=0.5775820016860962
node_206: feature_name=cg18386876	feature_id[4066].value > threshold=0.6847257018089294
node_208: feature_name=cg02053092	feature_id[3610].value > threshold=0.39507485926151276
node_210: feature_name=cg10298992	feature_id[3603].value > threshold=0.5707973837852478
node_228: feature_name=cg03840920	feature_id[4524].value > threshold=0.5365143716335297
node_234: feature_name=cg25364972	feature_id[1792].value > threshold=0.2418053299188614
node_238: feature_name=cg23485307	feature_id[221].value > threshold=0.14884746074676514
node_242: feature_name=cg04873169	feature_id[4696].value > threshold=0.6058756709098816
node_244: feature_name=cg17009731	feature_id[3218].value > threshold=0.6602252125740051
node_246: feature_name=cg18253910	feature_id[4458].value > threshold=0.6904322504997253
node_250: feature_name=cg04774476	feature_id[1257].value > threshold=0.5675267577171326
node_256: feature_name=cg19259111	feature_id[63].value > threshold=0.40771469473838806
node_264: feature_name=cg21483216	feature_id[5253].value > threshold=0.6332719326019287
node_266: feature_name=cg04026354	feature_id[3502].value <= threshold=0.42332448065280914
node_267: feature_name=cg11444072	feature_id[116].value > threshold=0.4940422773361206
node_273: feature_name=cg08571020	feature_id[381].value > threshold=0.4616774618625641
node_275: feature_name=cg08331427	feature_id[2081].value > threshold=0.5443233847618103
node_281: feature_name=cg00216961	feature_id[945].value <= threshold=0.4166988283395767
node_282: feature_name=cg04716580	feature_id[1390].value > threshold=0.4204266667366028
node_286: feature_name=cg07891483	feature_id[1877].value > threshold=0.829958975315094
node_288: feature_name=cg01475325	feature_id[3969].value > threshold=0.45323844254016876

node_292: feature_name=cg19702397	feature_id[2066].value > threshold=0.3536848723888397
node_294: feature_name=cg21936959	feature_id[595].value <= threshold=0.5567200481891632
node_295: feature_name=cg05251593	feature_id[515].value <= threshold=0.5597732663154602
node_296: feature_name=cg01343045	feature_id[1653].value <= threshold=0.9222070276737213
node_297: feature_name=cg03701759	feature_id[3914].value <= threshold=0.8587441444396973
node_298: feature_name=cg12497581	feature_id[1864].value > threshold=0.37188364565372467
node_300: feature_name=cg04254198	feature_id[4941].value > threshold=0.6460673660039902
node_302: feature_name=cg18761894	feature_id[3126].value > threshold=0.6353314220905304
Class: low-grade endometrial stromal sarcoma (ESS (LG))	
Rules_127	passed counts:1
node_0: feature_name=cg11915444	feature_id[2002].value > threshold=0.3402601182460785
node_10: feature_name=cg26016985	feature_id[4407].value > threshold=0.6493876278400421
node_16: feature_name=cg23157618	feature_id[5511].value > threshold=0.5759606957435608
node_20: feature_name=cg10480329	feature_id[1439].value > threshold=0.5518321692943573
node_48: feature_name=cg17843418	feature_id[4868].value > threshold=0.34969255328178406
node_52: feature_name=cg24407065	feature_id[4522].value > threshold=0.5364363789558411
node_56: feature_name=cg07281938	feature_id[249].value > threshold=0.7506992518901825
node_78: feature_name=cg21759907	feature_id[1650].value > threshold=0.46823520958423615
node_98: feature_name=cg10886334	feature_id[3975].value > threshold=0.43698127567768097
node_102: feature_name=cg09373983	feature_id[5025].value > threshold=0.5575815737247467
node_106: feature_name=cg12948116	feature_id[5360].value <= threshold=0.5658791363239288

node_107: feature_name=cg21906519	feature_id[5251].value > threshold=0.6089984774589539
node_111: feature_name=cg17537493	feature_id[4217].value > threshold=0.5825372636318207
node_117: feature_name=cg15720017	feature_id[4443].value > threshold=0.7009969055652618
node_119: feature_name=cg06038180	feature_id[3480].value > threshold=0.4583848565816879
node_121: feature_name=cg08619651	feature_id[4882].value <= threshold=0.2105495035648346
node_122: feature_name=cg01014262	feature_id[1911].value <= threshold=0.5327437818050385
node_123: feature_name=cg16863382	feature_id[2922].value > threshold=0.7200668156147003
node_133: feature_name=cg06989443	feature_id[2327].value <= threshold=0.6579365730285645
node_134: feature_name=cg07344990	feature_id[2398].value > threshold=0.5562876164913177
node_138: feature_name=cg02966841	feature_id[2199].value > threshold=0.7744501233100891
node_204: feature_name=cg12120430	feature_id[3727].value > threshold=0.5775820016860962
node_206: feature_name=cg18386876	feature_id[4066].value > threshold=0.6847257018089294
node_208: feature_name=cg02053092	feature_id[3610].value > threshold=0.39507485926151276
node_210: feature_name=cg10298992	feature_id[3603].value > threshold=0.5707973837852478
node_228: feature_name=cg03840920	feature_id[4524].value > threshold=0.5365143716335297
node_234: feature_name=cg25364972	feature_id[1792].value > threshold=0.2418053299188614
node_238: feature_name=cg23485307	feature_id[221].value > threshold=0.14884746074676514
node_242: feature_name=cg04873169	feature_id[4696].value > threshold=0.6058756709098816
node_244: feature_name=cg17009731	feature_id[3218].value > threshold=0.6602252125740051
node_246: feature_name=cg18253910	feature_id[4458].value > threshold=0.6904322504997253
node_250: feature_name=cg04774476	feature_id[1257].value > threshold=0.5675267577171326
node_256: feature_name=cg19259111	feature_id[63].value > threshold=0.40771469473838806

node_264: feature_name=cg21483216	feature_id[5253].value > threshold=0.6332719326019287
node_266: feature_name=cg04026354	feature_id[3502].value <= threshold=0.42332448065280914
node_267: feature_name=cg11444072	feature_id[116].value > threshold=0.4940422773361206
node_273: feature_name=cg08571020	feature_id[381].value > threshold=0.4616774618625641
node_275: feature_name=cg08331427	feature_id[2081].value > threshold=0.5443233847618103
node_281: feature_name=cg00216961	feature_id[945].value <= threshold=0.4166988283395767
node_282: feature_name=cg04716580	feature_id[1390].value > threshold=0.4204266667366028
node_286: feature_name=cg07891483	feature_id[1877].value > threshold=0.829958975315094
node_288: feature_name=cg01475325	feature_id[3969].value > threshold=0.45323844254016876
node_292: feature_name=cg19702397	feature_id[2066].value > threshold=0.3536848723888397
node_294: feature_name=cg21936959	feature_id[595].value <= threshold=0.5567200481891632
node_295: feature_name=cg05251593	feature_id[515].value <= threshold=0.5597732663154602
node_296: feature_name=cg01343045	feature_id[1653].value <= threshold=0.9222070276737213
node_297: feature_name=cg03701759	feature_id[3914].value <= threshold=0.8587441444396973
node_298: feature_name=cg12497581	feature_id[1864].value > threshold=0.37188364565372467
node_300: feature_name=cg04254198	feature_id[4941].value > threshold=0.6460673660039902
node_302: feature_name=cg18761894	feature_id[3126].value <= threshold=0.6353314220905304
Class: mesenchymal chondrosarcoma (CSA (MES))	
Rules_128	passed counts:1
node_0: feature_name=cg11915444	feature_id[2002].value > threshold=0.3402601182460785
node_10: feature_name=cg26016985	feature_id[4407].value > threshold=0.6493876278400421

node_16: feature_name=cg23157618	feature_id[5511].value > threshold=0.5759606957435608
node_20: feature_name=cg10480329	feature_id[1439].value > threshold=0.5518321692943573
node_48: feature_name=cg17843418	feature_id[4868].value > threshold=0.34969255328178406
node_52: feature_name=cg24407065	feature_id[4522].value > threshold=0.5364363789558411
node_56: feature_name=cg07281938	feature_id[249].value > threshold=0.7506992518901825
node_78: feature_name=cg21759907	feature_id[1650].value > threshold=0.46823520958423615
node_98: feature_name=cg10886334	feature_id[3975].value > threshold=0.43698127567768097
node_102: feature_name=cg09373983	feature_id[5025].value > threshold=0.5575815737247467
node_106: feature_name=cg12948116	feature_id[5360].value <= threshold=0.5658791363239288
node_107: feature_name=cg21906519	feature_id[5251].value > threshold=0.6089984774589539
node_111: feature_name=cg17537493	feature_id[4217].value > threshold=0.5825372636318207
node_117: feature_name=cg15720017	feature_id[4443].value > threshold=0.7009969055652618
node_119: feature_name=cg06038180	feature_id[3480].value > threshold=0.4583848565816879
node_121: feature_name=cg08619651	feature_id[4882].value <= threshold=0.2105495035648346
node_122: feature_name=cg01014262	feature_id[1911].value <= threshold=0.5327437818050385
node_123: feature_name=cg16863382	feature_id[2922].value > threshold=0.7200668156147003
node_133: feature_name=cg06989443	feature_id[2327].value <= threshold=0.6579365730285645
node_134: feature_name=cg07344990	feature_id[2398].value > threshold=0.5562876164913177
node_138: feature_name=cg02966841	feature_id[2199].value > threshold=0.7744501233100891
node_204: feature_name=cg12120430	feature_id[3727].value > threshold=0.5775820016860962
node_206: feature_name=cg18386876	feature_id[4066].value > threshold=0.6847257018089294
node_208: feature_name=cg02053092	feature_id[3610].value > threshold=0.39507485926151276

node_210: feature_name=cg10298992	feature_id[3603].value > threshold=0.5707973837852478
node_228: feature_name=cg03840920	feature_id[4524].value > threshold=0.5365143716335297
node_234: feature_name=cg25364972	feature_id[1792].value > threshold=0.2418053299188614
node_238: feature_name=cg23485307	feature_id[221].value > threshold=0.14884746074676514
node_242: feature_name=cg04873169	feature_id[4696].value > threshold=0.6058756709098816
node_244: feature_name=cg17009731	feature_id[3218].value > threshold=0.6602252125740051
node_246: feature_name=cg18253910	feature_id[4458].value > threshold=0.6904322504997253
node_250: feature_name=cg04774476	feature_id[1257].value > threshold=0.5675267577171326
node_256: feature_name=cg19259111	feature_id[63].value > threshold=0.40771469473838806
node_264: feature_name=cg21483216	feature_id[5253].value > threshold=0.6332719326019287
node_266: feature_name=cg04026354	feature_id[3502].value <= threshold=0.42332448065280914
node_267: feature_name=cg11444072	feature_id[116].value > threshold=0.4940422773361206
node_273: feature_name=cg08571020	feature_id[381].value > threshold=0.4616774618625641
node_275: feature_name=cg08331427	feature_id[2081].value > threshold=0.5443233847618103
node_281: feature_name=cg00216961	feature_id[945].value <= threshold=0.4166988283395767
node_282: feature_name=cg04716580	feature_id[1390].value > threshold=0.4204266667366028
node_286: feature_name=cg07891483	feature_id[1877].value > threshold=0.829958975315094
node_288: feature_name=cg01475325	feature_id[3969].value > threshold=0.45323844254016876
node_292: feature_name=cg19702397	feature_id[2066].value > threshold=0.3536848723888397
node_294: feature_name=cg21936959	feature_id[595].value <= threshold=0.5567200481891632
node_295: feature_name=cg05251593	feature_id[515].value <= threshold=0.5597732663154602
node_296: feature_name=cg01343045	feature_id[1653].value <= threshold=0.9222070276737213

node_297: feature_name=cg03701759	feature_id[3914].value <= threshold=0.8587441444396973
node_298: feature_name=cg12497581	feature_id[1864].value > threshold=0.37188364565372467
node_300: feature_name=cg04254198	feature_id[4941].value <= threshold=0.6460673660039902
Class: synovial sarcoma (SYSA)	
Rules_129	passed counts:1
node_0: feature_name=cg11915444	feature_id[2002].value > threshold=0.3402601182460785
node_10: feature_name=cg26016985	feature_id[4407].value > threshold=0.6493876278400421
node_16: feature_name=cg23157618	feature_id[5511].value > threshold=0.5759606957435608
node_20: feature_name=cg10480329	feature_id[1439].value > threshold=0.5518321692943573
node_48: feature_name=cg17843418	feature_id[4868].value > threshold=0.34969255328178406
node_52: feature_name=cg24407065	feature_id[4522].value > threshold=0.5364363789558411
node_56: feature_name=cg07281938	feature_id[249].value > threshold=0.7506992518901825
node_78: feature_name=cg21759907	feature_id[1650].value > threshold=0.46823520958423615
node_98: feature_name=cg10886334	feature_id[3975].value > threshold=0.43698127567768097
node_102: feature_name=cg09373983	feature_id[5025].value > threshold=0.5575815737247467
node_106: feature_name=cg12948116	feature_id[5360].value <= threshold=0.5658791363239288
node_107: feature_name=cg21906519	feature_id[5251].value > threshold=0.6089984774589539
node_111: feature_name=cg17537493	feature_id[4217].value > threshold=0.5825372636318207
node_117: feature_name=cg15720017	feature_id[4443].value > threshold=0.7009969055652618
node_119: feature_name=cg06038180	feature_id[3480].value > threshold=0.4583848565816879
node_121: feature_name=cg08619651	feature_id[4882].value <= threshold=0.2105495035648346
node_122: feature_name=cg01014262	feature_id[1911].value <= threshold=0.5327437818050385

node_123: feature_name=cg16863382	feature_id[2922].value > threshold=0.7200668156147003
node_133: feature_name=cg06989443	feature_id[2327].value <= threshold=0.6579365730285645
node_134: feature_name=cg07344990	feature_id[2398].value > threshold=0.5562876164913177
node_138: feature_name=cg02966841	feature_id[2199].value > threshold=0.7744501233100891
node_204: feature_name=cg12120430	feature_id[3727].value > threshold=0.5775820016860962
node_206: feature_name=cg18386876	feature_id[4066].value > threshold=0.6847257018089294
node_208: feature_name=cg02053092	feature_id[3610].value > threshold=0.39507485926151276
node_210: feature_name=cg10298992	feature_id[3603].value > threshold=0.5707973837852478
node_228: feature_name=cg03840920	feature_id[4524].value > threshold=0.5365143716335297
node_234: feature_name=cg25364972	feature_id[1792].value > threshold=0.2418053299188614
node_238: feature_name=cg23485307	feature_id[221].value > threshold=0.14884746074676514
node_242: feature_name=cg04873169	feature_id[4696].value > threshold=0.6058756709098816
node_244: feature_name=cg17009731	feature_id[3218].value > threshold=0.6602252125740051
node_246: feature_name=cg18253910	feature_id[4458].value > threshold=0.6904322504997253
node_250: feature_name=cg04774476	feature_id[1257].value > threshold=0.5675267577171326
node_256: feature_name=cg19259111	feature_id[63].value > threshold=0.40771469473838806
node_264: feature_name=cg21483216	feature_id[5253].value > threshold=0.6332719326019287
node_266: feature_name=cg04026354	feature_id[3502].value <= threshold=0.42332448065280914
node_267: feature_name=cg11444072	feature_id[116].value > threshold=0.4940422773361206
node_273: feature_name=cg08571020	feature_id[381].value > threshold=0.4616774618625641
node_275: feature_name=cg08331427	feature_id[2081].value > threshold=0.5443233847618103
node_281: feature_name=cg00216961	feature_id[945].value <= threshold=0.4166988283395767

node_282: feature_name=cg04716580	feature_id[1390].value > threshold=0.4204266667366028
node_286: feature_name=cg07891483	feature_id[1877].value > threshold=0.829958975315094
node_288: feature_name=cg01475325	feature_id[3969].value <= threshold=0.45323844254016876
node_289: feature_name=cg21925748	feature_id[5244].value > threshold=0.7208462655544281
Class: undifferentiated sarcoma (USARC)	
Rules_130	passed counts:1
node_0: feature_name=cg11915444	feature_id[2002].value > threshold=0.3402601182460785
node_10: feature_name=cg26016985	feature_id[4407].value > threshold=0.6493876278400421
node_16: feature_name=cg23157618	feature_id[5511].value > threshold=0.5759606957435608
node_20: feature_name=cg10480329	feature_id[1439].value > threshold=0.5518321692943573
node_48: feature_name=cg17843418	feature_id[4868].value > threshold=0.34969255328178406
node_52: feature_name=cg24407065	feature_id[4522].value > threshold=0.5364363789558411
node_56: feature_name=cg07281938	feature_id[249].value > threshold=0.7506992518901825
node_78: feature_name=cg21759907	feature_id[1650].value > threshold=0.46823520958423615
node_98: feature_name=cg10886334	feature_id[3975].value > threshold=0.43698127567768097
node_102: feature_name=cg09373983	feature_id[5025].value > threshold=0.5575815737247467
node_106: feature_name=cg12948116	feature_id[5360].value <= threshold=0.5658791363239288
node_107: feature_name=cg21906519	feature_id[5251].value > threshold=0.6089984774589539
node_111: feature_name=cg17537493	feature_id[4217].value > threshold=0.5825372636318207
node_117: feature_name=cg15720017	feature_id[4443].value > threshold=0.7009969055652618
node_119: feature_name=cg06038180	feature_id[3480].value > threshold=0.4583848565816879
node_121: feature_name=cg08619651	feature_id[4882].value <= threshold=0.2105495035648346

node_122: feature_name=cg01014262	feature_id[1911].value <= threshold=0.5327437818050385
node_123: feature_name=cg16863382	feature_id[2922].value > threshold=0.7200668156147003
node_133: feature_name=cg06989443	feature_id[2327].value <= threshold=0.6579365730285645
node_134: feature_name=cg07344990	feature_id[2398].value > threshold=0.5562876164913177
node_138: feature_name=cg02966841	feature_id[2199].value > threshold=0.7744501233100891
node_204: feature_name=cg12120430	feature_id[3727].value > threshold=0.5775820016860962
node_206: feature_name=cg18386876	feature_id[4066].value > threshold=0.6847257018089294
node_208: feature_name=cg02053092	feature_id[3610].value > threshold=0.39507485926151276
node_210: feature_name=cg10298992	feature_id[3603].value > threshold=0.5707973837852478
node_228: feature_name=cg03840920	feature_id[4524].value > threshold=0.5365143716335297
node_234: feature_name=cg25364972	feature_id[1792].value > threshold=0.2418053299188614
node_238: feature_name=cg23485307	feature_id[221].value > threshold=0.14884746074676514
node_242: feature_name=cg04873169	feature_id[4696].value > threshold=0.6058756709098816
node_244: feature_name=cg17009731	feature_id[3218].value > threshold=0.6602252125740051
node_246: feature_name=cg18253910	feature_id[4458].value > threshold=0.6904322504997253
node_250: feature_name=cg04774476	feature_id[1257].value > threshold=0.5675267577171326
node_256: feature_name=cg19259111	feature_id[63].value > threshold=0.40771469473838806
node_264: feature_name=cg21483216	feature_id[5253].value > threshold=0.6332719326019287
node_266: feature_name=cg04026354	feature_id[3502].value <= threshold=0.42332448065280914
node_267: feature_name=cg11444072	feature_id[116].value > threshold=0.4940422773361206
node_273: feature_name=cg08571020	feature_id[381].value > threshold=0.4616774618625641
node_275: feature_name=cg08331427	feature_id[2081].value > threshold=0.5443233847618103

node_281: feature_name=cg00216961	feature_id[945].value <= threshold=0.4166988283395767
node_282: feature_name=cg04716580	feature_id[1390].value <= threshold=0.4204266667366028
node_283: feature_name=cg09180239	feature_id[2620].value <= threshold=0.7707420885562897
Class: synovial sarcoma (SYSA)	
Rules_131	passed counts:1
node_0: feature_name=cg11915444	feature_id[2002].value > threshold=0.3402601182460785
node_10: feature_name=cg26016985	feature_id[4407].value > threshold=0.6493876278400421
node_16: feature_name=cg23157618	feature_id[5511].value > threshold=0.5759606957435608
node_20: feature_name=cg10480329	feature_id[1439].value > threshold=0.5518321692943573
node_48: feature_name=cg17843418	feature_id[4868].value > threshold=0.34969255328178406
node_52: feature_name=cg24407065	feature_id[4522].value > threshold=0.5364363789558411
node_56: feature_name=cg07281938	feature_id[249].value > threshold=0.7506992518901825
node_78: feature_name=cg21759907	feature_id[1650].value > threshold=0.46823520958423615
node_98: feature_name=cg10886334	feature_id[3975].value > threshold=0.43698127567768097
node_102: feature_name=cg09373983	feature_id[5025].value > threshold=0.5575815737247467
node_106: feature_name=cg12948116	feature_id[5360].value <= threshold=0.5658791363239288
node_107: feature_name=cg21906519	feature_id[5251].value > threshold=0.6089984774589539
node_111: feature_name=cg17537493	feature_id[4217].value > threshold=0.5825372636318207
node_117: feature_name=cg15720017	feature_id[4443].value > threshold=0.7009969055652618
node_119: feature_name=cg06038180	feature_id[3480].value > threshold=0.4583848565816879
node_121: feature_name=cg08619651	feature_id[4882].value <= threshold=0.2105495035648346
node_122: feature_name=cg01014262	feature_id[1911].value <= threshold=0.5327437818050385

node_123: feature_name=cg16863382	feature_id[2922].value > threshold=0.7200668156147003
node_133: feature_name=cg06989443	feature_id[2327].value <= threshold=0.6579365730285645
node_134: feature_name=cg07344990	feature_id[2398].value > threshold=0.5562876164913177
node_138: feature_name=cg02966841	feature_id[2199].value > threshold=0.7744501233100891
node_204: feature_name=cg12120430	feature_id[3727].value > threshold=0.5775820016860962
node_206: feature_name=cg18386876	feature_id[4066].value > threshold=0.6847257018089294
node_208: feature_name=cg02053092	feature_id[3610].value > threshold=0.39507485926151276
node_210: feature_name=cg10298992	feature_id[3603].value > threshold=0.5707973837852478
node_228: feature_name=cg03840920	feature_id[4524].value > threshold=0.5365143716335297
node_234: feature_name=cg25364972	feature_id[1792].value > threshold=0.2418053299188614
node_238: feature_name=cg23485307	feature_id[221].value > threshold=0.14884746074676514
node_242: feature_name=cg04873169	feature_id[4696].value > threshold=0.6058756709098816
node_244: feature_name=cg17009731	feature_id[3218].value > threshold=0.6602252125740051
node_246: feature_name=cg18253910	feature_id[4458].value > threshold=0.6904322504997253
node_250: feature_name=cg04774476	feature_id[1257].value > threshold=0.5675267577171326
node_256: feature_name=cg19259111	feature_id[63].value > threshold=0.40771469473838806
node_264: feature_name=cg21483216	feature_id[5253].value > threshold=0.6332719326019287
node_266: feature_name=cg04026354	feature_id[3502].value <= threshold=0.42332448065280914
node_267: feature_name=cg11444072	feature_id[116].value > threshold=0.4940422773361206
node_273: feature_name=cg08571020	feature_id[381].value > threshold=0.4616774618625641
node_275: feature_name=cg08331427	feature_id[2081].value <= threshold=0.5443233847618103
node_276: feature_name=cg23936766	feature_id[683].value > threshold=0.3338508605957031

node_278: feature_name=cg14178043	feature_id[915].value > threshold=0.42369575798511505
Class: well differentiated liposarcoma (WDLS)/dedifferentiated liposarcoma (DDLS)	
Rules_132	passed counts:1
node_0: feature_name=cg11915444	feature_id[2002].value > threshold=0.3402601182460785
node_10: feature_name=cg26016985	feature_id[4407].value > threshold=0.6493876278400421
node_16: feature_name=cg23157618	feature_id[5511].value > threshold=0.5759606957435608
node_20: feature_name=cg10480329	feature_id[1439].value > threshold=0.5518321692943573
node_48: feature_name=cg17843418	feature_id[4868].value > threshold=0.34969255328178406
node_52: feature_name=cg24407065	feature_id[4522].value > threshold=0.5364363789558411
node_56: feature_name=cg07281938	feature_id[249].value > threshold=0.7506992518901825
node_78: feature_name=cg21759907	feature_id[1650].value > threshold=0.46823520958423615
node_98: feature_name=cg10886334	feature_id[3975].value > threshold=0.43698127567768097
node_102: feature_name=cg09373983	feature_id[5025].value > threshold=0.5575815737247467
node_106: feature_name=cg12948116	feature_id[5360].value <= threshold=0.5658791363239288
node_107: feature_name=cg21906519	feature_id[5251].value > threshold=0.6089984774589539
node_111: feature_name=cg17537493	feature_id[4217].value > threshold=0.5825372636318207
node_117: feature_name=cg15720017	feature_id[4443].value > threshold=0.7009969055652618
node_119: feature_name=cg06038180	feature_id[3480].value > threshold=0.4583848565816879
node_121: feature_name=cg08619651	feature_id[4882].value <= threshold=0.2105495035648346
node_122: feature_name=cg01014262	feature_id[1911].value <= threshold=0.5327437818050385
node_123: feature_name=cg16863382	feature_id[2922].value > threshold=0.7200668156147003

node_133: feature_name=cg06989443	feature_id[2327].value <= threshold=0.6579365730285645
node_134: feature_name=cg07344990	feature_id[2398].value > threshold=0.5562876164913177
node_138: feature_name=cg02966841	feature_id[2199].value > threshold=0.7744501233100891
node_204: feature_name=cg12120430	feature_id[3727].value > threshold=0.5775820016860962
node_206: feature_name=cg18386876	feature_id[4066].value > threshold=0.6847257018089294
node_208: feature_name=cg02053092	feature_id[3610].value > threshold=0.39507485926151276
node_210: feature_name=cg10298992	feature_id[3603].value > threshold=0.5707973837852478
node_228: feature_name=cg03840920	feature_id[4524].value > threshold=0.5365143716335297
node_234: feature_name=cg25364972	feature_id[1792].value > threshold=0.2418053299188614
node_238: feature_name=cg23485307	feature_id[221].value > threshold=0.14884746074676514
node_242: feature_name=cg04873169	feature_id[4696].value > threshold=0.6058756709098816
node_244: feature_name=cg17009731	feature_id[3218].value > threshold=0.6602252125740051
node_246: feature_name=cg18253910	feature_id[4458].value > threshold=0.6904322504997253
node_250: feature_name=cg04774476	feature_id[1257].value > threshold=0.5675267577171326
node_256: feature_name=cg19259111	feature_id[63].value > threshold=0.40771469473838806
node_264: feature_name=cg21483216	feature_id[5253].value > threshold=0.6332719326019287
node_266: feature_name=cg04026354	feature_id[3502].value <= threshold=0.42332448065280914
node_267: feature_name=cg11444072	feature_id[116].value > threshold=0.4940422773361206
node_273: feature_name=cg08571020	feature_id[381].value > threshold=0.4616774618625641
node_275: feature_name=cg08331427	feature_id[2081].value <= threshold=0.5443233847618103
node_276: feature_name=cg23936766	feature_id[683].value > threshold=0.3338508605957031
node_278: feature_name=cg14178043	feature_id[915].value <= threshold=0.42369575798511505

Class: angiosarcoma (AS)	
Rules_133	passed counts:1
node_0: feature_name=cg11915444	feature_id[2002].value > threshold=0.3402601182460785
node_10: feature_name=cg26016985	feature_id[4407].value > threshold=0.6493876278400421
node_16: feature_name=cg23157618	feature_id[5511].value > threshold=0.5759606957435608
node_20: feature_name=cg10480329	feature_id[1439].value > threshold=0.5518321692943573
node_48: feature_name=cg17843418	feature_id[4868].value > threshold=0.34969255328178406
node_52: feature_name=cg24407065	feature_id[4522].value > threshold=0.5364363789558411
node_56: feature_name=cg07281938	feature_id[249].value > threshold=0.7506992518901825
node_78: feature_name=cg21759907	feature_id[1650].value > threshold=0.46823520958423615
node_98: feature_name=cg10886334	feature_id[3975].value > threshold=0.43698127567768097
node_102: feature_name=cg09373983	feature_id[5025].value > threshold=0.5575815737247467
node_106: feature_name=cg12948116	feature_id[5360].value <= threshold=0.5658791363239288
node_107: feature_name=cg21906519	feature_id[5251].value > threshold=0.6089984774589539
node_111: feature_name=cg17537493	feature_id[4217].value > threshold=0.5825372636318207
node_117: feature_name=cg15720017	feature_id[4443].value > threshold=0.7009969055652618
node_119: feature_name=cg06038180	feature_id[3480].value > threshold=0.4583848565816879
node_121: feature_name=cg08619651	feature_id[4882].value <= threshold=0.2105495035648346
node_122: feature_name=cg01014262	feature_id[1911].value <= threshold=0.5327437818050385
node_123: feature_name=cg16863382	feature_id[2922].value > threshold=0.7200668156147003
node_133: feature_name=cg06989443	feature_id[2327].value <= threshold=0.6579365730285645
node_134: feature_name=cg07344990	feature_id[2398].value > threshold=0.5562876164913177

node_138: feature_name=cg02966841	feature_id[2199].value > threshold=0.7744501233100891
node_204: feature_name=cg12120430	feature_id[3727].value > threshold=0.5775820016860962
node_206: feature_name=cg18386876	feature_id[4066].value > threshold=0.6847257018089294
node_208: feature_name=cg02053092	feature_id[3610].value > threshold=0.39507485926151276
node_210: feature_name=cg10298992	feature_id[3603].value > threshold=0.5707973837852478
node_228: feature_name=cg03840920	feature_id[4524].value > threshold=0.5365143716335297
node_234: feature_name=cg25364972	feature_id[1792].value > threshold=0.2418053299188614
node_238: feature_name=cg23485307	feature_id[221].value > threshold=0.14884746074676514
node_242: feature_name=cg04873169	feature_id[4696].value > threshold=0.6058756709098816
node_244: feature_name=cg17009731	feature_id[3218].value > threshold=0.6602252125740051
node_246: feature_name=cg18253910	feature_id[4458].value > threshold=0.6904322504997253
node_250: feature_name=cg04774476	feature_id[1257].value > threshold=0.5675267577171326
node_256: feature_name=cg19259111	feature_id[63].value > threshold=0.40771469473838806
node_264: feature_name=cg21483216	feature_id[5253].value > threshold=0.6332719326019287
node_266: feature_name=cg04026354	feature_id[3502].value <= threshold=0.42332448065280914
node_267: feature_name=cg11444072	feature_id[116].value <= threshold=0.4940422773361206
node_268: feature_name=cg25424742	feature_id[2698].value > threshold=0.762436032295227
node_270: feature_name=cg23632333	feature_id[4343].value <= threshold=0.88172447681427
Class: myositis proliferans (MP)	
Rules_134	passed counts:1
node_0: feature_name=cg11915444	feature_id[2002].value > threshold=0.3402601182460785
node_10: feature_name=cg26016985	feature_id[4407].value > threshold=0.6493876278400421

node_16: feature_name=cg23157618	feature_id[5511].value > threshold=0.5759606957435608
node_20: feature_name=cg10480329	feature_id[1439].value > threshold=0.5518321692943573
node_48: feature_name=cg17843418	feature_id[4868].value > threshold=0.34969255328178406
node_52: feature_name=cg24407065	feature_id[4522].value > threshold=0.5364363789558411
node_56: feature_name=cg07281938	feature_id[249].value > threshold=0.7506992518901825
node_78: feature_name=cg21759907	feature_id[1650].value > threshold=0.46823520958423615
node_98: feature_name=cg10886334	feature_id[3975].value > threshold=0.43698127567768097
node_102: feature_name=cg09373983	feature_id[5025].value > threshold=0.5575815737247467
node_106: feature_name=cg12948116	feature_id[5360].value <= threshold=0.5658791363239288
node_107: feature_name=cg21906519	feature_id[5251].value > threshold=0.6089984774589539
node_111: feature_name=cg17537493	feature_id[4217].value > threshold=0.5825372636318207
node_117: feature_name=cg15720017	feature_id[4443].value > threshold=0.7009969055652618
node_119: feature_name=cg06038180	feature_id[3480].value > threshold=0.4583848565816879
node_121: feature_name=cg08619651	feature_id[4882].value <= threshold=0.2105495035648346
node_122: feature_name=cg01014262	feature_id[1911].value <= threshold=0.5327437818050385
node_123: feature_name=cg16863382	feature_id[2922].value > threshold=0.7200668156147003
node_133: feature_name=cg06989443	feature_id[2327].value <= threshold=0.6579365730285645
node_134: feature_name=cg07344990	feature_id[2398].value > threshold=0.5562876164913177
node_138: feature_name=cg02966841	feature_id[2199].value > threshold=0.7744501233100891
node_204: feature_name=cg12120430	feature_id[3727].value > threshold=0.5775820016860962
node_206: feature_name=cg18386876	feature_id[4066].value > threshold=0.6847257018089294
node_208: feature_name=cg02053092	feature_id[3610].value > threshold=0.39507485926151276

node_210: feature_name=cg10298992	feature_id[3603].value > threshold=0.5707973837852478
node_228: feature_name=cg03840920	feature_id[4524].value > threshold=0.5365143716335297
node_234: feature_name=cg25364972	feature_id[1792].value > threshold=0.2418053299188614
node_238: feature_name=cg23485307	feature_id[221].value > threshold=0.14884746074676514
node_242: feature_name=cg04873169	feature_id[4696].value > threshold=0.6058756709098816
node_244: feature_name=cg17009731	feature_id[3218].value > threshold=0.6602252125740051
node_246: feature_name=cg18253910	feature_id[4458].value > threshold=0.6904322504997253
node_250: feature_name=cg04774476	feature_id[1257].value > threshold=0.5675267577171326
node_256: feature_name=cg19259111	feature_id[63].value <= threshold=0.40771469473838806
node_257: feature_name=cg10803714	feature_id[856].value > threshold=0.4142659604549408
node_259: feature_name=cg23350812	feature_id[1133].value > threshold=0.8047192692756653
node_261: feature_name=cg20464155	feature_id[1845].value > threshold=0.6415376514196396
Class: Ewing sarcoma (EWING)	
Rules_135	passed counts:1
node_0: feature_name=cg11915444	feature_id[2002].value > threshold=0.3402601182460785
node_10: feature_name=cg26016985	feature_id[4407].value > threshold=0.6493876278400421
node_16: feature_name=cg23157618	feature_id[5511].value > threshold=0.5759606957435608
node_20: feature_name=cg10480329	feature_id[1439].value > threshold=0.5518321692943573
node_48: feature_name=cg17843418	feature_id[4868].value > threshold=0.34969255328178406
node_52: feature_name=cg24407065	feature_id[4522].value > threshold=0.5364363789558411
node_56: feature_name=cg07281938	feature_id[249].value > threshold=0.7506992518901825
node_78: feature_name=cg21759907	feature_id[1650].value > threshold=0.46823520958423615

node_98: feature_name=cg10886334	feature_id[3975].value > threshold=0.43698127567768097
node_102: feature_name=cg09373983	feature_id[5025].value > threshold=0.5575815737247467
node_106: feature_name=cg12948116	feature_id[5360].value <= threshold=0.5658791363239288
node_107: feature_name=cg21906519	feature_id[5251].value > threshold=0.6089984774589539
node_111: feature_name=cg17537493	feature_id[4217].value > threshold=0.5825372636318207
node_117: feature_name=cg15720017	feature_id[4443].value > threshold=0.7009969055652618
node_119: feature_name=cg06038180	feature_id[3480].value > threshold=0.4583848565816879
node_121: feature_name=cg08619651	feature_id[4882].value <= threshold=0.2105495035648346
node_122: feature_name=cg01014262	feature_id[1911].value <= threshold=0.5327437818050385
node_123: feature_name=cg16863382	feature_id[2922].value > threshold=0.7200668156147003
node_133: feature_name=cg06989443	feature_id[2327].value <= threshold=0.6579365730285645
node_134: feature_name=cg07344990	feature_id[2398].value > threshold=0.5562876164913177
node_138: feature_name=cg02966841	feature_id[2199].value > threshold=0.7744501233100891
node_204: feature_name=cg12120430	feature_id[3727].value > threshold=0.5775820016860962
node_206: feature_name=cg18386876	feature_id[4066].value > threshold=0.6847257018089294
node_208: feature_name=cg02053092	feature_id[3610].value > threshold=0.39507485926151276
node_210: feature_name=cg10298992	feature_id[3603].value > threshold=0.5707973837852478
node_228: feature_name=cg03840920	feature_id[4524].value > threshold=0.5365143716335297
node_234: feature_name=cg25364972	feature_id[1792].value > threshold=0.2418053299188614
node_238: feature_name=cg23485307	feature_id[221].value > threshold=0.14884746074676514
node_242: feature_name=cg04873169	feature_id[4696].value > threshold=0.6058756709098816
node_244: feature_name=cg17009731	feature_id[3218].value > threshold=0.6602252125740051

node_246: feature_name=cg18253910	feature_id[4458].value > threshold=0.6904322504997253
node_250: feature_name=cg04774476	feature_id[1257].value > threshold=0.5675267577171326
node_256: feature_name=cg19259111	feature_id[63].value <= threshold=0.40771469473838806
node_257: feature_name=cg10803714	feature_id[856].value > threshold=0.4142659604549408
node_259: feature_name=cg23350812	feature_id[1133].value > threshold=0.8047192692756653
node_261: feature_name=cg20464155	feature_id[1845].value <= threshold=0.6415376514196396
Class: leiomyosarcoma (LMS)	
Rules_136	passed counts:1
node_0: feature_name=cg11915444	feature_id[2002].value > threshold=0.3402601182460785
node_10: feature_name=cg26016985	feature_id[4407].value > threshold=0.6493876278400421
node_16: feature_name=cg23157618	feature_id[5511].value > threshold=0.5759606957435608
node_20: feature_name=cg10480329	feature_id[1439].value > threshold=0.5518321692943573
node_48: feature_name=cg17843418	feature_id[4868].value > threshold=0.34969255328178406
node_52: feature_name=cg24407065	feature_id[4522].value > threshold=0.5364363789558411
node_56: feature_name=cg07281938	feature_id[249].value > threshold=0.7506992518901825
node_78: feature_name=cg21759907	feature_id[1650].value > threshold=0.46823520958423615
node_98: feature_name=cg10886334	feature_id[3975].value > threshold=0.43698127567768097
node_102: feature_name=cg09373983	feature_id[5025].value > threshold=0.5575815737247467
node_106: feature_name=cg12948116	feature_id[5360].value <= threshold=0.5658791363239288
node_107: feature_name=cg21906519	feature_id[5251].value > threshold=0.6089984774589539
node_111: feature_name=cg17537493	feature_id[4217].value > threshold=0.5825372636318207
node_117: feature_name=cg15720017	feature_id[4443].value > threshold=0.7009969055652618

node_119: feature_name=cg06038180	feature_id[3480].value > threshold=0.4583848565816879
node_121: feature_name=cg08619651	feature_id[4882].value <= threshold=0.2105495035648346
node_122: feature_name=cg01014262	feature_id[1911].value <= threshold=0.5327437818050385
node_123: feature_name=cg16863382	feature_id[2922].value > threshold=0.7200668156147003
node_133: feature_name=cg06989443	feature_id[2327].value <= threshold=0.6579365730285645
node_134: feature_name=cg07344990	feature_id[2398].value > threshold=0.5562876164913177
node_138: feature_name=cg02966841	feature_id[2199].value > threshold=0.7744501233100891
node_204: feature_name=cg12120430	feature_id[3727].value > threshold=0.5775820016860962
node_206: feature_name=cg18386876	feature_id[4066].value > threshold=0.6847257018089294
node_208: feature_name=cg02053092	feature_id[3610].value > threshold=0.39507485926151276
node_210: feature_name=cg10298992	feature_id[3603].value > threshold=0.5707973837852478
node_228: feature_name=cg03840920	feature_id[4524].value > threshold=0.5365143716335297
node_234: feature_name=cg25364972	feature_id[1792].value > threshold=0.2418053299188614
node_238: feature_name=cg23485307	feature_id[221].value > threshold=0.14884746074676514
node_242: feature_name=cg04873169	feature_id[4696].value > threshold=0.6058756709098816
node_244: feature_name=cg17009731	feature_id[3218].value > threshold=0.6602252125740051
node_246: feature_name=cg18253910	feature_id[4458].value <= threshold=0.6904322504997253
node_247: feature_name=cg01559356	feature_id[3236].value <= threshold=0.089854396879673
Class: osteoblastoma (OB)	
Rules_137	passed counts:1
node_0: feature_name=cg11915444	feature_id[2002].value > threshold=0.3402601182460785
node_10: feature_name=cg26016985	feature_id[4407].value > threshold=0.6493876278400421

node_16: feature_name=cg23157618	feature_id[5511].value > threshold=0.5759606957435608
node_20: feature_name=cg10480329	feature_id[1439].value > threshold=0.5518321692943573
node_48: feature_name=cg17843418	feature_id[4868].value > threshold=0.34969255328178406
node_52: feature_name=cg24407065	feature_id[4522].value > threshold=0.5364363789558411
node_56: feature_name=cg07281938	feature_id[249].value > threshold=0.7506992518901825
node_78: feature_name=cg21759907	feature_id[1650].value > threshold=0.46823520958423615
node_98: feature_name=cg10886334	feature_id[3975].value > threshold=0.43698127567768097
node_102: feature_name=cg09373983	feature_id[5025].value > threshold=0.5575815737247467
node_106: feature_name=cg12948116	feature_id[5360].value <= threshold=0.5658791363239288
node_107: feature_name=cg21906519	feature_id[5251].value > threshold=0.6089984774589539
node_111: feature_name=cg17537493	feature_id[4217].value > threshold=0.5825372636318207
node_117: feature_name=cg15720017	feature_id[4443].value > threshold=0.7009969055652618
node_119: feature_name=cg06038180	feature_id[3480].value > threshold=0.4583848565816879
node_121: feature_name=cg08619651	feature_id[4882].value <= threshold=0.2105495035648346
node_122: feature_name=cg01014262	feature_id[1911].value <= threshold=0.5327437818050385
node_123: feature_name=cg16863382	feature_id[2922].value > threshold=0.7200668156147003
node_133: feature_name=cg06989443	feature_id[2327].value <= threshold=0.6579365730285645
node_134: feature_name=cg07344990	feature_id[2398].value > threshold=0.5562876164913177
node_138: feature_name=cg02966841	feature_id[2199].value > threshold=0.7744501233100891
node_204: feature_name=cg12120430	feature_id[3727].value > threshold=0.5775820016860962
node_206: feature_name=cg18386876	feature_id[4066].value > threshold=0.6847257018089294
node_208: feature_name=cg02053092	feature_id[3610].value > threshold=0.39507485926151276

node_210: feature_name=cg10298992	feature_id[3603].value > threshold=0.5707973837852478
node_228: feature_name=cg03840920	feature_id[4524].value > threshold=0.5365143716335297
node_234: feature_name=cg25364972	feature_id[1792].value <= threshold=0.2418053299188614
node_235: feature_name=cg07612468	feature_id[803].value > threshold=0.8302715420722961
Class: mesenchymal chondrosarcoma (CSA (MES))	
Rules_138	passed counts:1
node_0: feature_name=cg11915444	feature_id[2002].value > threshold=0.3402601182460785
node_10: feature_name=cg26016985	feature_id[4407].value > threshold=0.6493876278400421
node_16: feature_name=cg23157618	feature_id[5511].value > threshold=0.5759606957435608
node_20: feature_name=cg10480329	feature_id[1439].value > threshold=0.5518321692943573
node_48: feature_name=cg17843418	feature_id[4868].value > threshold=0.34969255328178406
node_52: feature_name=cg24407065	feature_id[4522].value > threshold=0.5364363789558411
node_56: feature_name=cg07281938	feature_id[249].value > threshold=0.7506992518901825
node_78: feature_name=cg21759907	feature_id[1650].value > threshold=0.46823520958423615
node_98: feature_name=cg10886334	feature_id[3975].value > threshold=0.43698127567768097
node_102: feature_name=cg09373983	feature_id[5025].value > threshold=0.5575815737247467
node_106: feature_name=cg12948116	feature_id[5360].value <= threshold=0.5658791363239288
node_107: feature_name=cg21906519	feature_id[5251].value > threshold=0.6089984774589539
node_111: feature_name=cg17537493	feature_id[4217].value > threshold=0.5825372636318207
node_117: feature_name=cg15720017	feature_id[4443].value > threshold=0.7009969055652618
node_119: feature_name=cg06038180	feature_id[3480].value > threshold=0.4583848565816879

node_121: feature_name=cg08619651	feature_id[4882].value <= threshold=0.2105495035648346
node_122: feature_name=cg01014262	feature_id[1911].value <= threshold=0.5327437818050385
node_123: feature_name=cg16863382	feature_id[2922].value > threshold=0.7200668156147003
node_133: feature_name=cg06989443	feature_id[2327].value <= threshold=0.6579365730285645
node_134: feature_name=cg07344990	feature_id[2398].value > threshold=0.5562876164913177
node_138: feature_name=cg02966841	feature_id[2199].value > threshold=0.7744501233100891
node_204: feature_name=cg12120430	feature_id[3727].value > threshold=0.5775820016860962
node_206: feature_name=cg18386876	feature_id[4066].value > threshold=0.6847257018089294
node_208: feature_name=cg02053092	feature_id[3610].value > threshold=0.39507485926151276
node_210: feature_name=cg10298992	feature_id[3603].value > threshold=0.5707973837852478
node_228: feature_name=cg03840920	feature_id[4524].value <= threshold=0.5365143716335297
node_229: feature_name=cg21647035	feature_id[2479].value > threshold=0.3486105799674988
node_231: feature_name=cg19849478	feature_id[105].value > threshold=0.83695188164711
Class: malignant peripheral nerve sheath tumour (MPNST)	
Rules_139	passed counts:1
node_0: feature_name=cg11915444	feature_id[2002].value > threshold=0.3402601182460785
node_10: feature_name=cg26016985	feature_id[4407].value > threshold=0.6493876278400421
node_16: feature_name=cg23157618	feature_id[5511].value > threshold=0.5759606957435608
node_20: feature_name=cg10480329	feature_id[1439].value > threshold=0.5518321692943573
node_48: feature_name=cg17843418	feature_id[4868].value > threshold=0.34969255328178406
node_52: feature_name=cg24407065	feature_id[4522].value > threshold=0.5364363789558411

node_56: feature_name=cg07281938	feature_id[249].value > threshold=0.7506992518901825
node_78: feature_name=cg21759907	feature_id[1650].value > threshold=0.46823520958423615
node_98: feature_name=cg10886334	feature_id[3975].value > threshold=0.43698127567768097
node_102: feature_name=cg09373983	feature_id[5025].value > threshold=0.5575815737247467
node_106: feature_name=cg12948116	feature_id[5360].value <= threshold=0.5658791363239288
node_107: feature_name=cg21906519	feature_id[5251].value > threshold=0.6089984774589539
node_111: feature_name=cg17537493	feature_id[4217].value > threshold=0.5825372636318207
node_117: feature_name=cg15720017	feature_id[4443].value > threshold=0.7009969055652618
node_119: feature_name=cg06038180	feature_id[3480].value > threshold=0.4583848565816879
node_121: feature_name=cg08619651	feature_id[4882].value <= threshold=0.2105495035648346
node_122: feature_name=cg01014262	feature_id[1911].value <= threshold=0.5327437818050385
node_123: feature_name=cg16863382	feature_id[2922].value > threshold=0.7200668156147003
node_133: feature_name=cg06989443	feature_id[2327].value <= threshold=0.6579365730285645
node_134: feature_name=cg07344990	feature_id[2398].value > threshold=0.5562876164913177
node_138: feature_name=cg02966841	feature_id[2199].value > threshold=0.7744501233100891
node_204: feature_name=cg12120430	feature_id[3727].value > threshold=0.5775820016860962
node_206: feature_name=cg18386876	feature_id[4066].value > threshold=0.6847257018089294
node_208: feature_name=cg02053092	feature_id[3610].value > threshold=0.39507485926151276
node_210: feature_name=cg10298992	feature_id[3603].value > threshold=0.5707973837852478
node_228: feature_name=cg03840920	feature_id[4524].value <= threshold=0.5365143716335297
node_229: feature_name=cg21647035	feature_id[2479].value > threshold=0.3486105799674988
node_231: feature_name=cg19849478	feature_id[105].value <= threshold=0.83695188164711

Class: low-grade fibromyxoid sarcoma (LGFMS)	
Rules_140	passed counts:1
node_0: feature_name=cg11915444	feature_id[2002].value > threshold=0.3402601182460785
node_10: feature_name=cg26016985	feature_id[4407].value > threshold=0.6493876278400421
node_16: feature_name=cg23157618	feature_id[5511].value > threshold=0.5759606957435608
node_20: feature_name=cg10480329	feature_id[1439].value > threshold=0.5518321692943573
node_48: feature_name=cg17843418	feature_id[4868].value > threshold=0.34969255328178406
node_52: feature_name=cg24407065	feature_id[4522].value > threshold=0.5364363789558411
node_56: feature_name=cg07281938	feature_id[249].value > threshold=0.7506992518901825
node_78: feature_name=cg21759907	feature_id[1650].value > threshold=0.46823520958423615
node_98: feature_name=cg10886334	feature_id[3975].value > threshold=0.43698127567768097
node_102: feature_name=cg09373983	feature_id[5025].value > threshold=0.5575815737247467
node_106: feature_name=cg12948116	feature_id[5360].value <= threshold=0.5658791363239288
node_107: feature_name=cg21906519	feature_id[5251].value > threshold=0.6089984774589539
node_111: feature_name=cg17537493	feature_id[4217].value > threshold=0.5825372636318207
node_117: feature_name=cg15720017	feature_id[4443].value > threshold=0.7009969055652618
node_119: feature_name=cg06038180	feature_id[3480].value > threshold=0.4583848565816879
node_121: feature_name=cg08619651	feature_id[4882].value <= threshold=0.2105495035648346
node_122: feature_name=cg01014262	feature_id[1911].value <= threshold=0.5327437818050385
node_123: feature_name=cg16863382	feature_id[2922].value > threshold=0.7200668156147003
node_133: feature_name=cg06989443	feature_id[2327].value <= threshold=0.6579365730285645
node_134: feature_name=cg07344990	feature_id[2398].value > threshold=0.5562876164913177

node_138: feature_name=cg02966841	feature_id[2199].value > threshold=0.7744501233100891
node_204: feature_name=cg12120430	feature_id[3727].value > threshold=0.5775820016860962
node_206: feature_name=cg18386876	feature_id[4066].value > threshold=0.6847257018089294
node_208: feature_name=cg02053092	feature_id[3610].value > threshold=0.39507485926151276
node_210: feature_name=cg10298992	feature_id[3603].value <= threshold=0.5707973837852478
node_211: feature_name=cg02436098	feature_id[1712].value > threshold=0.27362556010484695
node_225: feature_name=cg01794853	feature_id[1531].value <= threshold=0.46636733412742615
Class: lipoma (LIPO)	
Rules_141	passed counts:1
node_0: feature_name=cg11915444	feature_id[2002].value > threshold=0.3402601182460785
node_10: feature_name=cg26016985	feature_id[4407].value > threshold=0.6493876278400421
node_16: feature_name=cg23157618	feature_id[5511].value > threshold=0.5759606957435608
node_20: feature_name=cg10480329	feature_id[1439].value > threshold=0.5518321692943573
node_48: feature_name=cg17843418	feature_id[4868].value > threshold=0.34969255328178406
node_52: feature_name=cg24407065	feature_id[4522].value > threshold=0.5364363789558411
node_56: feature_name=cg07281938	feature_id[249].value > threshold=0.7506992518901825
node_78: feature_name=cg21759907	feature_id[1650].value > threshold=0.46823520958423615
node_98: feature_name=cg10886334	feature_id[3975].value > threshold=0.43698127567768097
node_102: feature_name=cg09373983	feature_id[5025].value > threshold=0.5575815737247467
node_106: feature_name=cg12948116	feature_id[5360].value <= threshold=0.5658791363239288
node_107: feature_name=cg21906519	feature_id[5251].value > threshold=0.6089984774589539
node_111: feature_name=cg17537493	feature_id[4217].value > threshold=0.5825372636318207

node_117: feature_name=cg15720017	feature_id[4443].value > threshold=0.7009969055652618
node_119: feature_name=cg06038180	feature_id[3480].value > threshold=0.4583848565816879
node_121: feature_name=cg08619651	feature_id[4882].value <= threshold=0.2105495035648346
node_122: feature_name=cg01014262	feature_id[1911].value <= threshold=0.5327437818050385
node_123: feature_name=cg16863382	feature_id[2922].value > threshold=0.7200668156147003
node_133: feature_name=cg06989443	feature_id[2327].value <= threshold=0.6579365730285645
node_134: feature_name=cg07344990	feature_id[2398].value > threshold=0.5562876164913177
node_138: feature_name=cg02966841	feature_id[2199].value > threshold=0.7744501233100891
node_204: feature_name=cg12120430	feature_id[3727].value > threshold=0.5775820016860962
node_206: feature_name=cg18386876	feature_id[4066].value > threshold=0.6847257018089294
node_208: feature_name=cg02053092	feature_id[3610].value > threshold=0.39507485926151276
node_210: feature_name=cg10298992	feature_id[3603].value <= threshold=0.5707973837852478
node_211: feature_name=cg02436098	feature_id[1712].value <= threshold=0.27362556010484695
node_212: feature_name=cg15464763	feature_id[2786].value > threshold=0.6618955731391907
node_214: feature_name=cg15264991	feature_id[2805].value <= threshold=0.701563149690628
node_215: feature_name=cg04585669	feature_id[4112].value > threshold=0.2930298447608948
node_217: feature_name=cg11482422	feature_id[2778].value <= threshold=0.12514593824744225
node_218: feature_name=cg04046364	feature_id[1498].value > threshold=0.21376027166843414
node_220: feature_name=cg15420720	feature_id[1200].value > threshold=0.3182992488145828
Class: Langerhans cell histiocytosis (LCH)	
Rules_142	passed counts:1
node_0: feature_name=cg11915444	feature_id[2002].value > threshold=0.3402601182460785

node_10: feature_name=cg26016985	feature_id[4407].value > threshold=0.6493876278400421
node_16: feature_name=cg23157618	feature_id[5511].value > threshold=0.5759606957435608
node_20: feature_name=cg10480329	feature_id[1439].value > threshold=0.5518321692943573
node_48: feature_name=cg17843418	feature_id[4868].value > threshold=0.34969255328178406
node_52: feature_name=cg24407065	feature_id[4522].value > threshold=0.5364363789558411
node_56: feature_name=cg07281938	feature_id[249].value > threshold=0.7506992518901825
node_78: feature_name=cg21759907	feature_id[1650].value > threshold=0.46823520958423615
node_98: feature_name=cg10886334	feature_id[3975].value > threshold=0.43698127567768097
node_102: feature_name=cg09373983	feature_id[5025].value > threshold=0.5575815737247467
node_106: feature_name=cg12948116	feature_id[5360].value <= threshold=0.5658791363239288
node_107: feature_name=cg21906519	feature_id[5251].value > threshold=0.6089984774589539
node_111: feature_name=cg17537493	feature_id[4217].value > threshold=0.5825372636318207
node_117: feature_name=cg15720017	feature_id[4443].value > threshold=0.7009969055652618
node_119: feature_name=cg06038180	feature_id[3480].value > threshold=0.4583848565816879
node_121: feature_name=cg08619651	feature_id[4882].value <= threshold=0.2105495035648346
node_122: feature_name=cg01014262	feature_id[1911].value <= threshold=0.5327437818050385
node_123: feature_name=cg16863382	feature_id[2922].value > threshold=0.7200668156147003
node_133: feature_name=cg06989443	feature_id[2327].value <= threshold=0.6579365730285645
node_134: feature_name=cg07344990	feature_id[2398].value > threshold=0.5562876164913177
node_138: feature_name=cg02966841	feature_id[2199].value > threshold=0.7744501233100891
node_204: feature_name=cg12120430	feature_id[3727].value > threshold=0.5775820016860962
node_206: feature_name=cg18386876	feature_id[4066].value > threshold=0.6847257018089294

node_208: feature_name=cg02053092	feature_id[3610].value > threshold=0.39507485926151276
node_210: feature_name=cg10298992	feature_id[3603].value <= threshold=0.5707973837852478
node_211: feature_name=cg02436098	feature_id[1712].value <= threshold=0.27362556010484695
node_212: feature_name=cg15464763	feature_id[2786].value > threshold=0.6618955731391907
node_214: feature_name=cg15264991	feature_id[2805].value <= threshold=0.701563149690628
node_215: feature_name=cg04585669	feature_id[4112].value > threshold=0.2930298447608948
node_217: feature_name=cg11482422	feature_id[2778].value <= threshold=0.12514593824744225
node_218: feature_name=cg04046364	feature_id[1498].value > threshold=0.21376027166843414
node_220: feature_name=cg15420720	feature_id[1200].value <= threshold=0.3182992488145828
Class: embryonal rhabdomyosarcoma (RMS (EMB))	
Rules_143	passed counts:1
node_0: feature_name=cg11915444	feature_id[2002].value > threshold=0.3402601182460785
node_10: feature_name=cg26016985	feature_id[4407].value > threshold=0.6493876278400421
node_16: feature_name=cg23157618	feature_id[5511].value > threshold=0.5759606957435608
node_20: feature_name=cg10480329	feature_id[1439].value > threshold=0.5518321692943573
node_48: feature_name=cg17843418	feature_id[4868].value > threshold=0.34969255328178406
node_52: feature_name=cg24407065	feature_id[4522].value > threshold=0.5364363789558411
node_56: feature_name=cg07281938	feature_id[249].value > threshold=0.7506992518901825
node_78: feature_name=cg21759907	feature_id[1650].value > threshold=0.46823520958423615
node_98: feature_name=cg10886334	feature_id[3975].value > threshold=0.43698127567768097
node_102: feature_name=cg09373983	feature_id[5025].value > threshold=0.5575815737247467

node_106: feature_name=cg12948116	feature_id[5360].value <= threshold=0.5658791363239288
node_107: feature_name=cg21906519	feature_id[5251].value > threshold=0.6089984774589539
node_111: feature_name=cg17537493	feature_id[4217].value > threshold=0.5825372636318207
node_117: feature_name=cg15720017	feature_id[4443].value > threshold=0.7009969055652618
node_119: feature_name=cg06038180	feature_id[3480].value > threshold=0.4583848565816879
node_121: feature_name=cg08619651	feature_id[4882].value <= threshold=0.2105495035648346
node_122: feature_name=cg01014262	feature_id[1911].value <= threshold=0.5327437818050385
node_123: feature_name=cg16863382	feature_id[2922].value > threshold=0.7200668156147003
node_133: feature_name=cg06989443	feature_id[2327].value <= threshold=0.6579365730285645
node_134: feature_name=cg07344990	feature_id[2398].value > threshold=0.5562876164913177
node_138: feature_name=cg02966841	feature_id[2199].value > threshold=0.7744501233100891
node_204: feature_name=cg12120430	feature_id[3727].value > threshold=0.5775820016860962
node_206: feature_name=cg18386876	feature_id[4066].value > threshold=0.6847257018089294
node_208: feature_name=cg02053092	feature_id[3610].value > threshold=0.39507485926151276
node_210: feature_name=cg10298992	feature_id[3603].value <= threshold=0.5707973837852478
node_211: feature_name=cg02436098	feature_id[1712].value <= threshold=0.27362556010484695
node_212: feature_name=cg15464763	feature_id[2786].value > threshold=0.6618955731391907
node_214: feature_name=cg15264991	feature_id[2805].value <= threshold=0.701563149690628
node_215: feature_name=cg04585669	feature_id[4112].value > threshold=0.2930298447608948
node_217: feature_name=cg11482422	feature_id[2778].value <= threshold=0.12514593824744225
node_218: feature_name=cg04046364	feature_id[1498].value <= threshold=0.21376027166843414
Class: malignant peripheral nerve sheath tumour (MPNST)	

Rules_144	passed counts:1
node_0: feature_name=cg11915444	feature_id[2002].value > threshold=0.3402601182460785
node_10: feature_name=cg26016985	feature_id[4407].value > threshold=0.6493876278400421
node_16: feature_name=cg23157618	feature_id[5511].value > threshold=0.5759606957435608
node_20: feature_name=cg10480329	feature_id[1439].value > threshold=0.5518321692943573
node_48: feature_name=cg17843418	feature_id[4868].value > threshold=0.34969255328178406
node_52: feature_name=cg24407065	feature_id[4522].value > threshold=0.5364363789558411
node_56: feature_name=cg07281938	feature_id[249].value > threshold=0.7506992518901825
node_78: feature_name=cg21759907	feature_id[1650].value > threshold=0.46823520958423615
node_98: feature_name=cg10886334	feature_id[3975].value > threshold=0.43698127567768097
node_102: feature_name=cg09373983	feature_id[5025].value > threshold=0.5575815737247467
node_106: feature_name=cg12948116	feature_id[5360].value <= threshold=0.5658791363239288
node_107: feature_name=cg21906519	feature_id[5251].value > threshold=0.6089984774589539
node_111: feature_name=cg17537493	feature_id[4217].value > threshold=0.5825372636318207
node_117: feature_name=cg15720017	feature_id[4443].value > threshold=0.7009969055652618
node_119: feature_name=cg06038180	feature_id[3480].value > threshold=0.4583848565816879
node_121: feature_name=cg08619651	feature_id[4882].value <= threshold=0.2105495035648346
node_122: feature_name=cg01014262	feature_id[1911].value <= threshold=0.5327437818050385
node_123: feature_name=cg16863382	feature_id[2922].value > threshold=0.7200668156147003
node_133: feature_name=cg06989443	feature_id[2327].value <= threshold=0.6579365730285645
node_134: feature_name=cg07344990	feature_id[2398].value > threshold=0.5562876164913177

node_138: feature_name=cg02966841	feature_id[2199].value <= threshold=0.7744501233100891
node_139: feature_name=cg06989253	feature_id[194].value > threshold=0.7899391353130341
node_171: feature_name=cg07041720	feature_id[932].value > threshold=0.5004830807447433
node_189: feature_name=cg19374752	feature_id[196].value <= threshold=0.6096120476722717
node_190: feature_name=cg23085846	feature_id[3499].value <= threshold=0.8292354643344879
node_191: feature_name=cg04508340	feature_id[3427].value <= threshold=0.8703204989433289
node_192: feature_name=cg05951828	feature_id[21].value > threshold=0.8927997946739197
node_194: feature_name=cg16239826	feature_id[5096].value > threshold=0.6628752052783966
node_196: feature_name=cg17616283	feature_id[3747].value > threshold=0.23580453544855118
node_198: feature_name=cg03550233	feature_id[1927].value > threshold=0.27456413209438324
Class: atypical fibroxanthoma (AFX)/pleomorphic dermal sarcoma (PDS)	
Rules_145	passed counts:1
node_0: feature_name=cg11915444	feature_id[2002].value > threshold=0.3402601182460785
node_10: feature_name=cg26016985	feature_id[4407].value > threshold=0.6493876278400421
node_16: feature_name=cg23157618	feature_id[5511].value > threshold=0.5759606957435608
node_20: feature_name=cg10480329	feature_id[1439].value > threshold=0.5518321692943573
node_48: feature_name=cg17843418	feature_id[4868].value > threshold=0.34969255328178406
node_52: feature_name=cg24407065	feature_id[4522].value > threshold=0.5364363789558411
node_56: feature_name=cg07281938	feature_id[249].value > threshold=0.7506992518901825
node_78: feature_name=cg21759907	feature_id[1650].value > threshold=0.46823520958423615
node_98: feature_name=cg10886334	feature_id[3975].value > threshold=0.43698127567768097

node_102: feature_name=cg09373983	feature_id[5025].value > threshold=0.5575815737247467
node_106: feature_name=cg12948116	feature_id[5360].value <= threshold=0.5658791363239288
node_107: feature_name=cg21906519	feature_id[5251].value > threshold=0.6089984774589539
node_111: feature_name=cg17537493	feature_id[4217].value > threshold=0.5825372636318207
node_117: feature_name=cg15720017	feature_id[4443].value > threshold=0.7009969055652618
node_119: feature_name=cg06038180	feature_id[3480].value > threshold=0.4583848565816879
node_121: feature_name=cg08619651	feature_id[4882].value <= threshold=0.2105495035648346
node_122: feature_name=cg01014262	feature_id[1911].value <= threshold=0.5327437818050385
node_123: feature_name=cg16863382	feature_id[2922].value > threshold=0.7200668156147003
node_133: feature_name=cg06989443	feature_id[2327].value <= threshold=0.6579365730285645
node_134: feature_name=cg07344990	feature_id[2398].value > threshold=0.5562876164913177
node_138: feature_name=cg02966841	feature_id[2199].value <= threshold=0.7744501233100891
node_139: feature_name=cg06989253	feature_id[194].value > threshold=0.7899391353130341
node_171: feature_name=cg07041720	feature_id[932].value > threshold=0.5004830807447433
node_189: feature_name=cg19374752	feature_id[196].value <= threshold=0.6096120476722717
node_190: feature_name=cg23085846	feature_id[3499].value <= threshold=0.8292354643344879
node_191: feature_name=cg04508340	feature_id[3427].value <= threshold=0.8703204989433289
node_192: feature_name=cg05951828	feature_id[21].value > threshold=0.8927997946739197
node_194: feature_name=cg16239826	feature_id[5096].value > threshold=0.6628752052783966
node_196: feature_name=cg17616283	feature_id[3747].value > threshold=0.23580453544855118
node_198: feature_name=cg03550233	feature_id[1927].value <= threshold=0.27456413209438324
Class: sclerosing epithelioid fibrosarcoma(SEF)	

Rules_146	passed counts:1
node_0: feature_name=cg11915444	feature_id[2002].value > threshold=0.3402601182460785
node_10: feature_name=cg26016985	feature_id[4407].value > threshold=0.6493876278400421
node_16: feature_name=cg23157618	feature_id[5511].value > threshold=0.5759606957435608
node_20: feature_name=cg10480329	feature_id[1439].value > threshold=0.5518321692943573
node_48: feature_name=cg17843418	feature_id[4868].value > threshold=0.34969255328178406
node_52: feature_name=cg24407065	feature_id[4522].value > threshold=0.5364363789558411
node_56: feature_name=cg07281938	feature_id[249].value > threshold=0.7506992518901825
node_78: feature_name=cg21759907	feature_id[1650].value > threshold=0.46823520958423615
node_98: feature_name=cg10886334	feature_id[3975].value > threshold=0.43698127567768097
node_102: feature_name=cg09373983	feature_id[5025].value > threshold=0.5575815737247467
node_106: feature_name=cg12948116	feature_id[5360].value <= threshold=0.5658791363239288
node_107: feature_name=cg21906519	feature_id[5251].value > threshold=0.6089984774589539
node_111: feature_name=cg17537493	feature_id[4217].value > threshold=0.5825372636318207
node_117: feature_name=cg15720017	feature_id[4443].value > threshold=0.7009969055652618
node_119: feature_name=cg06038180	feature_id[3480].value > threshold=0.4583848565816879
node_121: feature_name=cg08619651	feature_id[4882].value <= threshold=0.2105495035648346
node_122: feature_name=cg01014262	feature_id[1911].value <= threshold=0.5327437818050385
node_123: feature_name=cg16863382	feature_id[2922].value > threshold=0.7200668156147003
node_133: feature_name=cg06989443	feature_id[2327].value <= threshold=0.6579365730285645
node_134: feature_name=cg07344990	feature_id[2398].value > threshold=0.5562876164913177

node_138: feature_name=cg02966841	feature_id[2199].value <= threshold=0.7744501233100891
node_139: feature_name=cg06989253	feature_id[194].value > threshold=0.7899391353130341
node_171: feature_name=cg07041720	feature_id[932].value > threshold=0.5004830807447433
node_189: feature_name=cg19374752	feature_id[196].value <= threshold=0.6096120476722717
node_190: feature_name=cg23085846	feature_id[3499].value <= threshold=0.8292354643344879
node_191: feature_name=cg04508340	feature_id[3427].value <= threshold=0.8703204989433289
node_192: feature_name=cg05951828	feature_id[21].value > threshold=0.8927997946739197
node_194: feature_name=cg16239826	feature_id[5096].value > threshold=0.6628752052783966
node_196: feature_name=cg17616283	feature_id[3747].value <= threshold=0.23580453544855118
Class: inflammatory myofibroblastic tumour (IMT)	
Rules_147	passed counts:1
node_0: feature_name=cg11915444	feature_id[2002].value > threshold=0.3402601182460785
node_10: feature_name=cg26016985	feature_id[4407].value > threshold=0.6493876278400421
node_16: feature_name=cg23157618	feature_id[5511].value > threshold=0.5759606957435608
node_20: feature_name=cg10480329	feature_id[1439].value > threshold=0.5518321692943573
node_48: feature_name=cg17843418	feature_id[4868].value > threshold=0.34969255328178406
node_52: feature_name=cg24407065	feature_id[4522].value > threshold=0.5364363789558411
node_56: feature_name=cg07281938	feature_id[249].value > threshold=0.7506992518901825
node_78: feature_name=cg21759907	feature_id[1650].value > threshold=0.46823520958423615
node_98: feature_name=cg10886334	feature_id[3975].value > threshold=0.43698127567768097
node_102: feature_name=cg09373983	feature_id[5025].value > threshold=0.5575815737247467

node_106: feature_name=cg12948116	feature_id[5360].value <= threshold=0.5658791363239288
node_107: feature_name=cg21906519	feature_id[5251].value > threshold=0.6089984774589539
node_111: feature_name=cg17537493	feature_id[4217].value > threshold=0.5825372636318207
node_117: feature_name=cg15720017	feature_id[4443].value > threshold=0.7009969055652618
node_119: feature_name=cg06038180	feature_id[3480].value > threshold=0.4583848565816879
node_121: feature_name=cg08619651	feature_id[4882].value <= threshold=0.2105495035648346
node_122: feature_name=cg01014262	feature_id[1911].value <= threshold=0.5327437818050385
node_123: feature_name=cg16863382	feature_id[2922].value > threshold=0.7200668156147003
node_133: feature_name=cg06989443	feature_id[2327].value <= threshold=0.6579365730285645
node_134: feature_name=cg07344990	feature_id[2398].value > threshold=0.5562876164913177
node_138: feature_name=cg02966841	feature_id[2199].value <= threshold=0.7744501233100891
node_139: feature_name=cg06989253	feature_id[194].value > threshold=0.7899391353130341
node_171: feature_name=cg07041720	feature_id[932].value > threshold=0.5004830807447433
node_189: feature_name=cg19374752	feature_id[196].value <= threshold=0.6096120476722717
node_190: feature_name=cg23085846	feature_id[3499].value <= threshold=0.8292354643344879
node_191: feature_name=cg04508340	feature_id[3427].value <= threshold=0.8703204989433289
node_192: feature_name=cg05951828	feature_id[21].value > threshold=0.8927997946739197
node_194: feature_name=cg16239826	feature_id[5096].value <= threshold=0.6628752052783966
Class: epithelioid sarcoma (ES)	
Rules_148	passed counts:1
node_0: feature_name=cg11915444	feature_id[2002].value > threshold=0.3402601182460785
node_10: feature_name=cg26016985	feature_id[4407].value > threshold=0.6493876278400421

node_16: feature_name=cg23157618	feature_id[5511].value > threshold=0.5759606957435608
node_20: feature_name=cg10480329	feature_id[1439].value > threshold=0.5518321692943573
node_48: feature_name=cg17843418	feature_id[4868].value > threshold=0.34969255328178406
node_52: feature_name=cg24407065	feature_id[4522].value > threshold=0.5364363789558411
node_56: feature_name=cg07281938	feature_id[249].value > threshold=0.7506992518901825
node_78: feature_name=cg21759907	feature_id[1650].value > threshold=0.46823520958423615
node_98: feature_name=cg10886334	feature_id[3975].value > threshold=0.43698127567768097
node_102: feature_name=cg09373983	feature_id[5025].value > threshold=0.5575815737247467
node_106: feature_name=cg12948116	feature_id[5360].value <= threshold=0.5658791363239288
node_107: feature_name=cg21906519	feature_id[5251].value > threshold=0.6089984774589539
node_111: feature_name=cg17537493	feature_id[4217].value > threshold=0.5825372636318207
node_117: feature_name=cg15720017	feature_id[4443].value > threshold=0.7009969055652618
node_119: feature_name=cg06038180	feature_id[3480].value > threshold=0.4583848565816879
node_121: feature_name=cg08619651	feature_id[4882].value <= threshold=0.2105495035648346
node_122: feature_name=cg01014262	feature_id[1911].value <= threshold=0.5327437818050385
node_123: feature_name=cg16863382	feature_id[2922].value > threshold=0.7200668156147003
node_133: feature_name=cg06989443	feature_id[2327].value <= threshold=0.6579365730285645
node_134: feature_name=cg07344990	feature_id[2398].value > threshold=0.5562876164913177
node_138: feature_name=cg02966841	feature_id[2199].value <= threshold=0.7744501233100891
node_139: feature_name=cg06989253	feature_id[194].value > threshold=0.7899391353130341
node_171: feature_name=cg07041720	feature_id[932].value <= threshold=0.5004830807447433
node_172: feature_name=cg04716580	feature_id[1390].value > threshold=0.8913259208202362

node_182: feature_name=cg24426483	feature_id[3487].value > threshold=0.7537154853343964
node_186: feature_name=cg11847597	feature_id[2502].value <= threshold=0.49640630185604095
Class: giant cell tumour of bone (GCTB)	
Rules_149	passed counts:1
node_0: feature_name=cg11915444	feature_id[2002].value > threshold=0.3402601182460785
node_10: feature_name=cg26016985	feature_id[4407].value > threshold=0.6493876278400421
node_16: feature_name=cg23157618	feature_id[5511].value > threshold=0.5759606957435608
node_20: feature_name=cg10480329	feature_id[1439].value > threshold=0.5518321692943573
node_48: feature_name=cg17843418	feature_id[4868].value > threshold=0.34969255328178406
node_52: feature_name=cg24407065	feature_id[4522].value > threshold=0.5364363789558411
node_56: feature_name=cg07281938	feature_id[249].value > threshold=0.7506992518901825
node_78: feature_name=cg21759907	feature_id[1650].value > threshold=0.46823520958423615
node_98: feature_name=cg10886334	feature_id[3975].value > threshold=0.43698127567768097
node_102: feature_name=cg09373983	feature_id[5025].value > threshold=0.5575815737247467
node_106: feature_name=cg12948116	feature_id[5360].value <= threshold=0.5658791363239288
node_107: feature_name=cg21906519	feature_id[5251].value > threshold=0.6089984774589539
node_111: feature_name=cg17537493	feature_id[4217].value > threshold=0.5825372636318207
node_117: feature_name=cg15720017	feature_id[4443].value > threshold=0.7009969055652618
node_119: feature_name=cg06038180	feature_id[3480].value > threshold=0.4583848565816879
node_121: feature_name=cg08619651	feature_id[4882].value <= threshold=0.2105495035648346
node_122: feature_name=cg01014262	feature_id[1911].value <= threshold=0.5327437818050385
node_123: feature_name=cg16863382	feature_id[2922].value > threshold=0.7200668156147003

node_133: feature_name=cg06989443	feature_id[2327].value <= threshold=0.6579365730285645
node_134: feature_name=cg07344990	feature_id[2398].value > threshold=0.5562876164913177
node_138: feature_name=cg02966841	feature_id[2199].value <= threshold=0.7744501233100891
node_139: feature_name=cg06989253	feature_id[194].value > threshold=0.7899391353130341
node_171: feature_name=cg07041720	feature_id[932].value <= threshold=0.5004830807447433
node_172: feature_name=cg04716580	feature_id[1390].value > threshold=0.8913259208202362
node_182: feature_name=cg24426483	feature_id[3487].value <= threshold=0.7537154853343964
node_183: feature_name=cg14753356	feature_id[385].value > threshold=0.3692520558834076
Class: sarcoma (SARC)	
Rules_150	passed counts:1
node_0: feature_name=cg11915444	feature_id[2002].value > threshold=0.3402601182460785
node_10: feature_name=cg26016985	feature_id[4407].value > threshold=0.6493876278400421
node_16: feature_name=cg23157618	feature_id[5511].value > threshold=0.5759606957435608
node_20: feature_name=cg10480329	feature_id[1439].value > threshold=0.5518321692943573
node_48: feature_name=cg17843418	feature_id[4868].value > threshold=0.34969255328178406
node_52: feature_name=cg24407065	feature_id[4522].value > threshold=0.5364363789558411
node_56: feature_name=cg07281938	feature_id[249].value > threshold=0.7506992518901825
node_78: feature_name=cg21759907	feature_id[1650].value > threshold=0.46823520958423615
node_98: feature_name=cg10886334	feature_id[3975].value > threshold=0.43698127567768097
node_102: feature_name=cg09373983	feature_id[5025].value > threshold=0.5575815737247467
node_106: feature_name=cg12948116	feature_id[5360].value <= threshold=0.5658791363239288
node_107: feature_name=cg21906519	feature_id[5251].value > threshold=0.6089984774589539

node_111: feature_name=cg17537493	feature_id[4217].value > threshold=0.5825372636318207
node_117: feature_name=cg15720017	feature_id[4443].value > threshold=0.7009969055652618
node_119: feature_name=cg06038180	feature_id[3480].value > threshold=0.4583848565816879
node_121: feature_name=cg08619651	feature_id[4882].value <= threshold=0.2105495035648346
node_122: feature_name=cg01014262	feature_id[1911].value <= threshold=0.5327437818050385
node_123: feature_name=cg16863382	feature_id[2922].value > threshold=0.7200668156147003
node_133: feature_name=cg06989443	feature_id[2327].value <= threshold=0.6579365730285645
node_134: feature_name=cg07344990	feature_id[2398].value > threshold=0.5562876164913177
node_138: feature_name=cg02966841	feature_id[2199].value <= threshold=0.7744501233100891
node_139: feature_name=cg06989253	feature_id[194].value > threshold=0.7899391353130341
node_171: feature_name=cg07041720	feature_id[932].value <= threshold=0.5004830807447433
node_172: feature_name=cg04716580	feature_id[1390].value <= threshold=0.8913259208202362
node_173: feature_name=cg12183501	feature_id[1208].value > threshold=0.1898196041584015
node_175: feature_name=cg25066857	feature_id[3535].value > threshold=0.6125359833240509
node_177: feature_name=cg19604110	feature_id[2693].value > threshold=0.193588025867939
node_179: feature_name=cg25413575	feature_id[3277].value > threshold=0.7227720022201538
Class: atypical fibroxanthoma (AFX)/pleomorphic dermal sarcoma (PDS)	
Rules_151	passed counts:1
node_0: feature_name=cg11915444	feature_id[2002].value > threshold=0.3402601182460785
node_10: feature_name=cg26016985	feature_id[4407].value > threshold=0.6493876278400421
node_16: feature_name=cg23157618	feature_id[5511].value > threshold=0.5759606957435608

node_20: feature_name=cg10480329	feature_id[1439].value > threshold=0.5518321692943573
node_48: feature_name=cg17843418	feature_id[4868].value > threshold=0.34969255328178406
node_52: feature_name=cg24407065	feature_id[4522].value > threshold=0.5364363789558411
node_56: feature_name=cg07281938	feature_id[249].value > threshold=0.7506992518901825
node_78: feature_name=cg21759907	feature_id[1650].value > threshold=0.46823520958423615
node_98: feature_name=cg10886334	feature_id[3975].value > threshold=0.43698127567768097
node_102: feature_name=cg09373983	feature_id[5025].value > threshold=0.5575815737247467
node_106: feature_name=cg12948116	feature_id[5360].value <= threshold=0.5658791363239288
node_107: feature_name=cg21906519	feature_id[5251].value > threshold=0.6089984774589539
node_111: feature_name=cg17537493	feature_id[4217].value > threshold=0.5825372636318207
node_117: feature_name=cg15720017	feature_id[4443].value > threshold=0.7009969055652618
node_119: feature_name=cg06038180	feature_id[3480].value > threshold=0.4583848565816879
node_121: feature_name=cg08619651	feature_id[4882].value <= threshold=0.2105495035648346
node_122: feature_name=cg01014262	feature_id[1911].value <= threshold=0.5327437818050385
node_123: feature_name=cg16863382	feature_id[2922].value > threshold=0.7200668156147003
node_133: feature_name=cg06989443	feature_id[2327].value <= threshold=0.6579365730285645
node_134: feature_name=cg07344990	feature_id[2398].value > threshold=0.5562876164913177
node_138: feature_name=cg02966841	feature_id[2199].value <= threshold=0.7744501233100891
node_139: feature_name=cg06989253	feature_id[194].value > threshold=0.7899391353130341
node_171: feature_name=cg07041720	feature_id[932].value <= threshold=0.5004830807447433
node_172: feature_name=cg04716580	feature_id[1390].value <= threshold=0.8913259208202362
node_173: feature_name=cg12183501	feature_id[1208].value > threshold=0.1898196041584015

node_175: feature_name=cg25066857	feature_id[3535].value > threshold=0.6125359833240509
node_177: feature_name=cg19604110	feature_id[2693].value > threshold=0.193588025867939
node_179: feature_name=cg25413575	feature_id[3277].value <= threshold=0.7227720022201538
Class: sarcoma (SARC)	
Rules_152	passed counts:1
node_0: feature_name=cg11915444	feature_id[2002].value > threshold=0.3402601182460785
node_10: feature_name=cg26016985	feature_id[4407].value > threshold=0.6493876278400421
node_16: feature_name=cg23157618	feature_id[5511].value > threshold=0.5759606957435608
node_20: feature_name=cg10480329	feature_id[1439].value > threshold=0.5518321692943573
node_48: feature_name=cg17843418	feature_id[4868].value > threshold=0.34969255328178406
node_52: feature_name=cg24407065	feature_id[4522].value > threshold=0.5364363789558411
node_56: feature_name=cg07281938	feature_id[249].value > threshold=0.7506992518901825
node_78: feature_name=cg21759907	feature_id[1650].value > threshold=0.46823520958423615
node_98: feature_name=cg10886334	feature_id[3975].value > threshold=0.43698127567768097
node_102: feature_name=cg09373983	feature_id[5025].value > threshold=0.5575815737247467
node_106: feature_name=cg12948116	feature_id[5360].value <= threshold=0.5658791363239288
node_107: feature_name=cg21906519	feature_id[5251].value > threshold=0.6089984774589539
node_111: feature_name=cg17537493	feature_id[4217].value > threshold=0.5825372636318207
node_117: feature_name=cg15720017	feature_id[4443].value > threshold=0.7009969055652618
node_119: feature_name=cg06038180	feature_id[3480].value > threshold=0.4583848565816879
node_121: feature_name=cg08619651	feature_id[4882].value <= threshold=0.2105495035648346
node_122: feature_name=cg01014262	feature_id[1911].value <= threshold=0.5327437818050385

node_123: feature_name=cg16863382	feature_id[2922].value > threshold=0.7200668156147003
node_133: feature_name=cg06989443	feature_id[2327].value <= threshold=0.6579365730285645
node_134: feature_name=cg07344990	feature_id[2398].value > threshold=0.5562876164913177
node_138: feature_name=cg02966841	feature_id[2199].value <= threshold=0.7744501233100891
node_139: feature_name=cg06989253	feature_id[194].value > threshold=0.7899391353130341
node_171: feature_name=cg07041720	feature_id[932].value <= threshold=0.5004830807447433
node_172: feature_name=cg04716580	feature_id[1390].value <= threshold=0.8913259208202362
node_173: feature_name=cg12183501	feature_id[1208].value > threshold=0.1898196041584015
node_175: feature_name=cg25066857	feature_id[3535].value > threshold=0.6125359833240509
node_177: feature_name=cg19604110	feature_id[2693].value <= threshold=0.193588025867939
Class: sclerosing epithelioid fibrosarcoma(SEF)	
Rules_153	passed counts:1
node_0: feature_name=cg11915444	feature_id[2002].value > threshold=0.3402601182460785
node_10: feature_name=cg26016985	feature_id[4407].value > threshold=0.6493876278400421
node_16: feature_name=cg23157618	feature_id[5511].value > threshold=0.5759606957435608
node_20: feature_name=cg10480329	feature_id[1439].value > threshold=0.5518321692943573
node_48: feature_name=cg17843418	feature_id[4868].value > threshold=0.34969255328178406
node_52: feature_name=cg24407065	feature_id[4522].value > threshold=0.5364363789558411
node_56: feature_name=cg07281938	feature_id[249].value > threshold=0.7506992518901825
node_78: feature_name=cg21759907	feature_id[1650].value > threshold=0.46823520958423615
node_98: feature_name=cg10886334	feature_id[3975].value > threshold=0.43698127567768097
node_102: feature_name=cg09373983	feature_id[5025].value > threshold=0.5575815737247467

node_106: feature_name=cg12948116	feature_id[5360].value <= threshold=0.5658791363239288
node_107: feature_name=cg21906519	feature_id[5251].value > threshold=0.6089984774589539
node_111: feature_name=cg17537493	feature_id[4217].value > threshold=0.5825372636318207
node_117: feature_name=cg15720017	feature_id[4443].value > threshold=0.7009969055652618
node_119: feature_name=cg06038180	feature_id[3480].value > threshold=0.4583848565816879
node_121: feature_name=cg08619651	feature_id[4882].value <= threshold=0.2105495035648346
node_122: feature_name=cg01014262	feature_id[1911].value <= threshold=0.5327437818050385
node_123: feature_name=cg16863382	feature_id[2922].value > threshold=0.7200668156147003
node_133: feature_name=cg06989443	feature_id[2327].value <= threshold=0.6579365730285645
node_134: feature_name=cg07344990	feature_id[2398].value > threshold=0.5562876164913177
node_138: feature_name=cg02966841	feature_id[2199].value <= threshold=0.7744501233100891
node_139: feature_name=cg06989253	feature_id[194].value > threshold=0.7899391353130341
node_171: feature_name=cg07041720	feature_id[932].value <= threshold=0.5004830807447433
node_172: feature_name=cg04716580	feature_id[1390].value <= threshold=0.8913259208202362
node_173: feature_name=cg12183501	feature_id[1208].value > threshold=0.1898196041584015
node_175: feature_name=cg25066857	feature_id[3535].value <= threshold=0.6125359833240509
Class: leiomyosarcoma (LMS)	
Rules_154	passed counts:1
node_0: feature_name=cg11915444	feature_id[2002].value > threshold=0.3402601182460785
node_10: feature_name=cg26016985	feature_id[4407].value > threshold=0.6493876278400421
node_16: feature_name=cg23157618	feature_id[5511].value > threshold=0.5759606957435608
node_20: feature_name=cg10480329	feature_id[1439].value > threshold=0.5518321692943573

node_48: feature_name=cg17843418	feature_id[4868].value > threshold=0.34969255328178406
node_52: feature_name=cg24407065	feature_id[4522].value > threshold=0.5364363789558411
node_56: feature_name=cg07281938	feature_id[249].value > threshold=0.7506992518901825
node_78: feature_name=cg21759907	feature_id[1650].value > threshold=0.46823520958423615
node_98: feature_name=cg10886334	feature_id[3975].value > threshold=0.43698127567768097
node_102: feature_name=cg09373983	feature_id[5025].value > threshold=0.5575815737247467
node_106: feature_name=cg12948116	feature_id[5360].value <= threshold=0.5658791363239288
node_107: feature_name=cg21906519	feature_id[5251].value > threshold=0.6089984774589539
node_111: feature_name=cg17537493	feature_id[4217].value > threshold=0.5825372636318207
node_117: feature_name=cg15720017	feature_id[4443].value > threshold=0.7009969055652618
node_119: feature_name=cg06038180	feature_id[3480].value > threshold=0.4583848565816879
node_121: feature_name=cg08619651	feature_id[4882].value <= threshold=0.2105495035648346
node_122: feature_name=cg01014262	feature_id[1911].value <= threshold=0.5327437818050385
node_123: feature_name=cg16863382	feature_id[2922].value > threshold=0.7200668156147003
node_133: feature_name=cg06989443	feature_id[2327].value <= threshold=0.6579365730285645
node_134: feature_name=cg07344990	feature_id[2398].value > threshold=0.5562876164913177
node_138: feature_name=cg02966841	feature_id[2199].value <= threshold=0.7744501233100891
node_139: feature_name=cg06989253	feature_id[194].value > threshold=0.7899391353130341
node_171: feature_name=cg07041720	feature_id[932].value <= threshold=0.5004830807447433
node_172: feature_name=cg04716580	feature_id[1390].value <= threshold=0.8913259208202362
node_173: feature_name=cg12183501	feature_id[1208].value <= threshold=0.1898196041584015
Class: angiosarcoma (AS)	

Rules_155	passed counts:1
node_0: feature_name=cg11915444	feature_id[2002].value > threshold=0.3402601182460785
node_10: feature_name=cg26016985	feature_id[4407].value > threshold=0.6493876278400421
node_16: feature_name=cg23157618	feature_id[5511].value > threshold=0.5759606957435608
node_20: feature_name=cg10480329	feature_id[1439].value > threshold=0.5518321692943573
node_48: feature_name=cg17843418	feature_id[4868].value > threshold=0.34969255328178406
node_52: feature_name=cg24407065	feature_id[4522].value > threshold=0.5364363789558411
node_56: feature_name=cg07281938	feature_id[249].value > threshold=0.7506992518901825
node_78: feature_name=cg21759907	feature_id[1650].value > threshold=0.46823520958423615
node_98: feature_name=cg10886334	feature_id[3975].value > threshold=0.43698127567768097
node_102: feature_name=cg09373983	feature_id[5025].value > threshold=0.5575815737247467
node_106: feature_name=cg12948116	feature_id[5360].value <= threshold=0.5658791363239288
node_107: feature_name=cg21906519	feature_id[5251].value > threshold=0.6089984774589539
node_111: feature_name=cg17537493	feature_id[4217].value > threshold=0.5825372636318207
node_117: feature_name=cg15720017	feature_id[4443].value > threshold=0.7009969055652618
node_119: feature_name=cg06038180	feature_id[3480].value > threshold=0.4583848565816879
node_121: feature_name=cg08619651	feature_id[4882].value <= threshold=0.2105495035648346
node_122: feature_name=cg01014262	feature_id[1911].value <= threshold=0.5327437818050385
node_123: feature_name=cg16863382	feature_id[2922].value > threshold=0.7200668156147003
node_133: feature_name=cg06989443	feature_id[2327].value <= threshold=0.6579365730285645
node_134: feature_name=cg07344990	feature_id[2398].value > threshold=0.5562876164913177

node_138: feature_name=cg02966841	feature_id[2199].value <= threshold=0.7744501233100891
node_139: feature_name=cg06989253	feature_id[194].value <= threshold=0.7899391353130341
node_140: feature_name=cg24617568	feature_id[3282].value <= threshold=0.26932457089424133
node_141: feature_name=cg12614090	feature_id[2765].value <= threshold=0.1813211366534233
node_142: feature_name=cg10917602	feature_id[4685].value <= threshold=0.5744736790657043
node_143: feature_name=cg23723410	feature_id[34].value <= threshold=0.3201078921556473
node_144: feature_name=cg26804423	feature_id[1725].value > threshold=0.39587317407131195
node_146: feature_name=cg20080282	feature_id[1150].value <= threshold=0.856019914150238
node_147: feature_name=cg08924374	feature_id[4125].value > threshold=0.07863279432058334
node_149: feature_name=cg08535779	feature_id[2187].value > threshold=0.24677357077598572
node_151: feature_name=cg00290626	feature_id[4663].value > threshold=0.6112961769104004
node_153: feature_name=cg20829550	feature_id[1054].value > threshold=0.15552543848752975
node_155: feature_name=cg25841625	feature_id[555].value > threshold=0.20218250900506973
node_157: feature_name=cg11724511	feature_id[1465].value > threshold=0.548207238316536
node_159: feature_name=cg12549211	feature_id[3192].value > threshold=0.6682310700416565
node_161: feature_name=cg25431366	feature_id[136].value > threshold=0.5233770608901978
node_163: feature_name=cg23344121	feature_id[799].value > threshold=0.7573113739490509
Class: epithelioid haemangioendothelioma (EHE)	
Rules_156	passed counts:1
node_0: feature_name=cg11915444	feature_id[2002].value > threshold=0.3402601182460785
node_10: feature_name=cg26016985	feature_id[4407].value > threshold=0.6493876278400421
node_16: feature_name=cg23157618	feature_id[5511].value > threshold=0.5759606957435608

node_20: feature_name=cg10480329	feature_id[1439].value > threshold=0.5518321692943573
node_48: feature_name=cg17843418	feature_id[4868].value > threshold=0.34969255328178406
node_52: feature_name=cg24407065	feature_id[4522].value > threshold=0.5364363789558411
node_56: feature_name=cg07281938	feature_id[249].value > threshold=0.7506992518901825
node_78: feature_name=cg21759907	feature_id[1650].value > threshold=0.46823520958423615
node_98: feature_name=cg10886334	feature_id[3975].value > threshold=0.43698127567768097
node_102: feature_name=cg09373983	feature_id[5025].value > threshold=0.5575815737247467
node_106: feature_name=cg12948116	feature_id[5360].value <= threshold=0.5658791363239288
node_107: feature_name=cg21906519	feature_id[5251].value > threshold=0.6089984774589539
node_111: feature_name=cg17537493	feature_id[4217].value > threshold=0.5825372636318207
node_117: feature_name=cg15720017	feature_id[4443].value > threshold=0.7009969055652618
node_119: feature_name=cg06038180	feature_id[3480].value > threshold=0.4583848565816879
node_121: feature_name=cg08619651	feature_id[4882].value <= threshold=0.2105495035648346
node_122: feature_name=cg01014262	feature_id[1911].value <= threshold=0.5327437818050385
node_123: feature_name=cg16863382	feature_id[2922].value > threshold=0.7200668156147003
node_133: feature_name=cg06989443	feature_id[2327].value <= threshold=0.6579365730285645
node_134: feature_name=cg07344990	feature_id[2398].value > threshold=0.5562876164913177
node_138: feature_name=cg02966841	feature_id[2199].value <= threshold=0.7744501233100891
node_139: feature_name=cg06989253	feature_id[194].value <= threshold=0.7899391353130341
node_140: feature_name=cg24617568	feature_id[3282].value <= threshold=0.26932457089424133
node_141: feature_name=cg12614090	feature_id[2765].value <= threshold=0.1813211366534233
node_142: feature_name=cg10917602	feature_id[4685].value <= threshold=0.5744736790657043

node_143: feature_name=cg23723410	feature_id[34].value <= threshold=0.3201078921556473
node_144: feature_name=cg26804423	feature_id[1725].value > threshold=0.39587317407131195
node_146: feature_name=cg20080282	feature_id[1150].value <= threshold=0.856019914150238
node_147: feature_name=cg08924374	feature_id[4125].value > threshold=0.07863279432058334
node_149: feature_name=cg08535779	feature_id[2187].value > threshold=0.24677357077598572
node_151: feature_name=cg00290626	feature_id[4663].value > threshold=0.6112961769104004
node_153: feature_name=cg20829550	feature_id[1054].value > threshold=0.15552543848752975
node_155: feature_name=cg25841625	feature_id[555].value > threshold=0.20218250900506973
node_157: feature_name=cg11724511	feature_id[1465].value > threshold=0.548207238316536
node_159: feature_name=cg12549211	feature_id[3192].value > threshold=0.6682310700416565
node_161: feature_name=cg25431366	feature_id[136].value > threshold=0.5233770608901978
node_163: feature_name=cg23344121	feature_id[799].value <= threshold=0.7573113739490509
Class: atypical fibroxanthoma (AFX)/pleomorphic dermal sarcoma (PDS)	
Rules_157	passed counts:1
node_0: feature_name=cg11915444	feature_id[2002].value > threshold=0.3402601182460785
node_10: feature_name=cg26016985	feature_id[4407].value > threshold=0.6493876278400421
node_16: feature_name=cg23157618	feature_id[5511].value > threshold=0.5759606957435608
node_20: feature_name=cg10480329	feature_id[1439].value > threshold=0.5518321692943573
node_48: feature_name=cg17843418	feature_id[4868].value > threshold=0.34969255328178406
node_52: feature_name=cg24407065	feature_id[4522].value > threshold=0.5364363789558411
node_56: feature_name=cg07281938	feature_id[249].value > threshold=0.7506992518901825

node_78: feature_name=cg21759907	feature_id[1650].value > threshold=0.46823520958423615
node_98: feature_name=cg10886334	feature_id[3975].value > threshold=0.43698127567768097
node_102: feature_name=cg09373983	feature_id[5025].value > threshold=0.5575815737247467
node_106: feature_name=cg12948116	feature_id[5360].value <= threshold=0.5658791363239288
node_107: feature_name=cg21906519	feature_id[5251].value > threshold=0.6089984774589539
node_111: feature_name=cg17537493	feature_id[4217].value > threshold=0.5825372636318207
node_117: feature_name=cg15720017	feature_id[4443].value > threshold=0.7009969055652618
node_119: feature_name=cg06038180	feature_id[3480].value > threshold=0.4583848565816879
node_121: feature_name=cg08619651	feature_id[4882].value <= threshold=0.2105495035648346
node_122: feature_name=cg01014262	feature_id[1911].value <= threshold=0.5327437818050385
node_123: feature_name=cg16863382	feature_id[2922].value > threshold=0.7200668156147003
node_133: feature_name=cg06989443	feature_id[2327].value <= threshold=0.6579365730285645
node_134: feature_name=cg07344990	feature_id[2398].value > threshold=0.5562876164913177
node_138: feature_name=cg02966841	feature_id[2199].value <= threshold=0.7744501233100891
node_139: feature_name=cg06989253	feature_id[194].value <= threshold=0.7899391353130341
node_140: feature_name=cg24617568	feature_id[3282].value <= threshold=0.26932457089424133
node_141: feature_name=cg12614090	feature_id[2765].value <= threshold=0.1813211366534233
node_142: feature_name=cg10917602	feature_id[4685].value <= threshold=0.5744736790657043
node_143: feature_name=cg23723410	feature_id[34].value <= threshold=0.3201078921556473
node_144: feature_name=cg26804423	feature_id[1725].value > threshold=0.39587317407131195
node_146: feature_name=cg20080282	feature_id[1150].value <= threshold=0.856019914150238
node_147: feature_name=cg08924374	feature_id[4125].value > threshold=0.07863279432058334

node_149: feature_name=cg08535779	feature_id[2187].value > threshold=0.24677357077598572
node_151: feature_name=cg00290626	feature_id[4663].value > threshold=0.6112961769104004
node_153: feature_name=cg20829550	feature_id[1054].value > threshold=0.15552543848752975
node_155: feature_name=cg25841625	feature_id[555].value > threshold=0.20218250900506973
node_157: feature_name=cg11724511	feature_id[1465].value > threshold=0.548207238316536
node_159: feature_name=cg12549211	feature_id[3192].value > threshold=0.6682310700416565
node_161: feature_name=cg25431366	feature_id[136].value <= threshold=0.5233770608901978
Class: alveolar soft part sarcoma (ASPS)	
Rules_158	passed counts:1
node_0: feature_name=cg11915444	feature_id[2002].value > threshold=0.3402601182460785
node_10: feature_name=cg26016985	feature_id[4407].value > threshold=0.6493876278400421
node_16: feature_name=cg23157618	feature_id[5511].value > threshold=0.5759606957435608
node_20: feature_name=cg10480329	feature_id[1439].value > threshold=0.5518321692943573
node_48: feature_name=cg17843418	feature_id[4868].value > threshold=0.34969255328178406
node_52: feature_name=cg24407065	feature_id[4522].value > threshold=0.5364363789558411
node_56: feature_name=cg07281938	feature_id[249].value > threshold=0.7506992518901825
node_78: feature_name=cg21759907	feature_id[1650].value > threshold=0.46823520958423615
node_98: feature_name=cg10886334	feature_id[3975].value > threshold=0.43698127567768097
node_102: feature_name=cg09373983	feature_id[5025].value > threshold=0.5575815737247467
node_106: feature_name=cg12948116	feature_id[5360].value <= threshold=0.5658791363239288
node_107: feature_name=cg21906519	feature_id[5251].value > threshold=0.6089984774589539
node_111: feature_name=cg17537493	feature_id[4217].value > threshold=0.5825372636318207

node_117: feature_name=cg15720017	feature_id[4443].value > threshold=0.7009969055652618
node_119: feature_name=cg06038180	feature_id[3480].value > threshold=0.4583848565816879
node_121: feature_name=cg08619651	feature_id[4882].value <= threshold=0.2105495035648346
node_122: feature_name=cg01014262	feature_id[1911].value <= threshold=0.5327437818050385
node_123: feature_name=cg16863382	feature_id[2922].value > threshold=0.7200668156147003
node_133: feature_name=cg06989443	feature_id[2327].value <= threshold=0.6579365730285645
node_134: feature_name=cg07344990	feature_id[2398].value > threshold=0.5562876164913177
node_138: feature_name=cg02966841	feature_id[2199].value <= threshold=0.7744501233100891
node_139: feature_name=cg06989253	feature_id[194].value <= threshold=0.7899391353130341
node_140: feature_name=cg24617568	feature_id[3282].value <= threshold=0.26932457089424133
node_141: feature_name=cg12614090	feature_id[2765].value <= threshold=0.1813211366534233
node_142: feature_name=cg10917602	feature_id[4685].value <= threshold=0.5744736790657043
node_143: feature_name=cg23723410	feature_id[34].value <= threshold=0.3201078921556473
node_144: feature_name=cg26804423	feature_id[1725].value > threshold=0.39587317407131195
node_146: feature_name=cg20080282	feature_id[1150].value <= threshold=0.856019914150238
node_147: feature_name=cg08924374	feature_id[4125].value > threshold=0.07863279432058334
node_149: feature_name=cg08535779	feature_id[2187].value > threshold=0.24677357077598572
node_151: feature_name=cg00290626	feature_id[4663].value > threshold=0.6112961769104004
node_153: feature_name=cg20829550	feature_id[1054].value > threshold=0.15552543848752975
node_155: feature_name=cg25841625	feature_id[555].value > threshold=0.20218250900506973
node_157: feature_name=cg11724511	feature_id[1465].value > threshold=0.548207238316536
node_159: feature_name=cg12549211	feature_id[3192].value <= threshold=0.6682310700416565

Class: fibrous dysplasia (FDY)	
Rules_159	passed counts:1
node_0: feature_name=cg11915444	feature_id[2002].value > threshold=0.3402601182460785
node_10: feature_name=cg26016985	feature_id[4407].value > threshold=0.6493876278400421
node_16: feature_name=cg23157618	feature_id[5511].value > threshold=0.5759606957435608
node_20: feature_name=cg10480329	feature_id[1439].value > threshold=0.5518321692943573
node_48: feature_name=cg17843418	feature_id[4868].value > threshold=0.34969255328178406
node_52: feature_name=cg24407065	feature_id[4522].value > threshold=0.5364363789558411
node_56: feature_name=cg07281938	feature_id[249].value > threshold=0.7506992518901825
node_78: feature_name=cg21759907	feature_id[1650].value > threshold=0.46823520958423615
node_98: feature_name=cg10886334	feature_id[3975].value > threshold=0.43698127567768097
node_102: feature_name=cg09373983	feature_id[5025].value > threshold=0.5575815737247467
node_106: feature_name=cg12948116	feature_id[5360].value <= threshold=0.5658791363239288
node_107: feature_name=cg21906519	feature_id[5251].value > threshold=0.6089984774589539
node_111: feature_name=cg17537493	feature_id[4217].value > threshold=0.5825372636318207
node_117: feature_name=cg15720017	feature_id[4443].value > threshold=0.7009969055652618
node_119: feature_name=cg06038180	feature_id[3480].value > threshold=0.4583848565816879
node_121: feature_name=cg08619651	feature_id[4882].value <= threshold=0.2105495035648346
node_122: feature_name=cg01014262	feature_id[1911].value <= threshold=0.5327437818050385
node_123: feature_name=cg16863382	feature_id[2922].value > threshold=0.7200668156147003
node_133: feature_name=cg06989443	feature_id[2327].value <= threshold=0.6579365730285645
node_134: feature_name=cg07344990	feature_id[2398].value > threshold=0.5562876164913177

node_138: feature_name=cg02966841	feature_id[2199].value <= threshold=0.7744501233100891
node_139: feature_name=cg06989253	feature_id[194].value <= threshold=0.7899391353130341
node_140: feature_name=cg24617568	feature_id[3282].value <= threshold=0.26932457089424133
node_141: feature_name=cg12614090	feature_id[2765].value <= threshold=0.1813211366534233
node_142: feature_name=cg10917602	feature_id[4685].value <= threshold=0.5744736790657043
node_143: feature_name=cg23723410	feature_id[34].value <= threshold=0.3201078921556473
node_144: feature_name=cg26804423	feature_id[1725].value > threshold=0.39587317407131195
node_146: feature_name=cg20080282	feature_id[1150].value <= threshold=0.856019914150238
node_147: feature_name=cg08924374	feature_id[4125].value > threshold=0.07863279432058334
node_149: feature_name=cg08535779	feature_id[2187].value > threshold=0.24677357077598572
node_151: feature_name=cg00290626	feature_id[4663].value > threshold=0.6112961769104004
node_153: feature_name=cg20829550	feature_id[1054].value > threshold=0.15552543848752975
node_155: feature_name=cg25841625	feature_id[555].value > threshold=0.20218250900506973
node_157: feature_name=cg11724511	feature_id[1465].value <= threshold=0.548207238316536
Class: malignant peripheral nerve sheath tumour (MPNST)	
Rules_160	passed counts:1
node_0: feature_name=cg11915444	feature_id[2002].value > threshold=0.3402601182460785
node_10: feature_name=cg26016985	feature_id[4407].value > threshold=0.6493876278400421
node_16: feature_name=cg23157618	feature_id[5511].value > threshold=0.5759606957435608
node_20: feature_name=cg10480329	feature_id[1439].value > threshold=0.5518321692943573
node_48: feature_name=cg17843418	feature_id[4868].value > threshold=0.34969255328178406

node_52: feature_name=cg24407065	feature_id[4522].value > threshold=0.5364363789558411
node_56: feature_name=cg07281938	feature_id[249].value > threshold=0.7506992518901825
node_78: feature_name=cg21759907	feature_id[1650].value > threshold=0.46823520958423615
node_98: feature_name=cg10886334	feature_id[3975].value > threshold=0.43698127567768097
node_102: feature_name=cg09373983	feature_id[5025].value > threshold=0.5575815737247467
node_106: feature_name=cg12948116	feature_id[5360].value <= threshold=0.5658791363239288
node_107: feature_name=cg21906519	feature_id[5251].value > threshold=0.6089984774589539
node_111: feature_name=cg17537493	feature_id[4217].value > threshold=0.5825372636318207
node_117: feature_name=cg15720017	feature_id[4443].value > threshold=0.7009969055652618
node_119: feature_name=cg06038180	feature_id[3480].value > threshold=0.4583848565816879
node_121: feature_name=cg08619651	feature_id[4882].value <= threshold=0.2105495035648346
node_122: feature_name=cg01014262	feature_id[1911].value <= threshold=0.5327437818050385
node_123: feature_name=cg16863382	feature_id[2922].value > threshold=0.7200668156147003
node_133: feature_name=cg06989443	feature_id[2327].value <= threshold=0.6579365730285645
node_134: feature_name=cg07344990	feature_id[2398].value > threshold=0.5562876164913177
node_138: feature_name=cg02966841	feature_id[2199].value <= threshold=0.7744501233100891
node_139: feature_name=cg06989253	feature_id[194].value <= threshold=0.7899391353130341
node_140: feature_name=cg24617568	feature_id[3282].value <= threshold=0.26932457089424133
node_141: feature_name=cg12614090	feature_id[2765].value <= threshold=0.1813211366534233
node_142: feature_name=cg10917602	feature_id[4685].value <= threshold=0.5744736790657043
node_143: feature_name=cg23723410	feature_id[34].value <= threshold=0.3201078921556473
node_144: feature_name=cg26804423	feature_id[1725].value > threshold=0.39587317407131195

node_146: feature_name=cg20080282	feature_id[1150].value <= threshold=0.856019914150238
node_147: feature_name=cg08924374	feature_id[4125].value > threshold=0.07863279432058334
node_149: feature_name=cg08535779	feature_id[2187].value > threshold=0.24677357077598572
node_151: feature_name=cg00290626	feature_id[4663].value > threshold=0.6112961769104004
node_153: feature_name=cg20829550	feature_id[1054].value > threshold=0.15552543848752975
node_155: feature_name=cg25841625	feature_id[555].value <= threshold=0.20218250900506973
Class: ossifying fibromyxoid tumour (OFMT)	
Rules_161	passed counts:1
node_0: feature_name=cg11915444	feature_id[2002].value > threshold=0.3402601182460785
node_10: feature_name=cg26016985	feature_id[4407].value > threshold=0.6493876278400421
node_16: feature_name=cg23157618	feature_id[5511].value > threshold=0.5759606957435608
node_20: feature_name=cg10480329	feature_id[1439].value > threshold=0.5518321692943573
node_48: feature_name=cg17843418	feature_id[4868].value > threshold=0.34969255328178406
node_52: feature_name=cg24407065	feature_id[4522].value > threshold=0.5364363789558411
node_56: feature_name=cg07281938	feature_id[249].value > threshold=0.7506992518901825
node_78: feature_name=cg21759907	feature_id[1650].value > threshold=0.46823520958423615
node_98: feature_name=cg10886334	feature_id[3975].value > threshold=0.43698127567768097
node_102: feature_name=cg09373983	feature_id[5025].value > threshold=0.5575815737247467
node_106: feature_name=cg12948116	feature_id[5360].value <= threshold=0.5658791363239288
node_107: feature_name=cg21906519	feature_id[5251].value > threshold=0.6089984774589539
node_111: feature_name=cg17537493	feature_id[4217].value > threshold=0.5825372636318207
node_117: feature_name=cg15720017	feature_id[4443].value > threshold=0.7009969055652618

node_119: feature_name=cg06038180	feature_id[3480].value > threshold=0.4583848565816879
node_121: feature_name=cg08619651	feature_id[4882].value <= threshold=0.2105495035648346
node_122: feature_name=cg01014262	feature_id[1911].value <= threshold=0.5327437818050385
node_123: feature_name=cg16863382	feature_id[2922].value > threshold=0.7200668156147003
node_133: feature_name=cg06989443	feature_id[2327].value <= threshold=0.6579365730285645
node_134: feature_name=cg07344990	feature_id[2398].value > threshold=0.5562876164913177
node_138: feature_name=cg02966841	feature_id[2199].value <= threshold=0.7744501233100891
node_139: feature_name=cg06989253	feature_id[194].value <= threshold=0.7899391353130341
node_140: feature_name=cg24617568	feature_id[3282].value <= threshold=0.26932457089424133
node_141: feature_name=cg12614090	feature_id[2765].value <= threshold=0.1813211366534233
node_142: feature_name=cg10917602	feature_id[4685].value <= threshold=0.5744736790657043
node_143: feature_name=cg23723410	feature_id[34].value <= threshold=0.3201078921556473
node_144: feature_name=cg26804423	feature_id[1725].value > threshold=0.39587317407131195
node_146: feature_name=cg20080282	feature_id[1150].value <= threshold=0.856019914150238
node_147: feature_name=cg08924374	feature_id[4125].value > threshold=0.07863279432058334
node_149: feature_name=cg08535779	feature_id[2187].value > threshold=0.24677357077598572
node_151: feature_name=cg00290626	feature_id[4663].value > threshold=0.6112961769104004
node_153: feature_name=cg20829550	feature_id[1054].value <= threshold=0.15552543848752975
Class: extraskeletal myxoid chondrosarcoma (EMCS)	
Rules_162	passed counts:1
node_0: feature_name=cg11915444	feature_id[2002].value > threshold=0.3402601182460785

node_10: feature_name=cg26016985	feature_id[4407].value > threshold=0.6493876278400421
node_16: feature_name=cg23157618	feature_id[5511].value > threshold=0.5759606957435608
node_20: feature_name=cg10480329	feature_id[1439].value > threshold=0.5518321692943573
node_48: feature_name=cg17843418	feature_id[4868].value > threshold=0.34969255328178406
node_52: feature_name=cg24407065	feature_id[4522].value > threshold=0.5364363789558411
node_56: feature_name=cg07281938	feature_id[249].value > threshold=0.7506992518901825
node_78: feature_name=cg21759907	feature_id[1650].value > threshold=0.46823520958423615
node_98: feature_name=cg10886334	feature_id[3975].value > threshold=0.43698127567768097
node_102: feature_name=cg09373983	feature_id[5025].value > threshold=0.5575815737247467
node_106: feature_name=cg12948116	feature_id[5360].value <= threshold=0.5658791363239288
node_107: feature_name=cg21906519	feature_id[5251].value > threshold=0.6089984774589539
node_111: feature_name=cg17537493	feature_id[4217].value > threshold=0.5825372636318207
node_117: feature_name=cg15720017	feature_id[4443].value > threshold=0.7009969055652618
node_119: feature_name=cg06038180	feature_id[3480].value > threshold=0.4583848565816879
node_121: feature_name=cg08619651	feature_id[4882].value <= threshold=0.2105495035648346
node_122: feature_name=cg01014262	feature_id[1911].value <= threshold=0.5327437818050385
node_123: feature_name=cg16863382	feature_id[2922].value > threshold=0.7200668156147003
node_133: feature_name=cg06989443	feature_id[2327].value <= threshold=0.6579365730285645
node_134: feature_name=cg07344990	feature_id[2398].value > threshold=0.5562876164913177
node_138: feature_name=cg02966841	feature_id[2199].value <= threshold=0.7744501233100891
node_139: feature_name=cg06989253	feature_id[194].value <= threshold=0.7899391353130341
node_140: feature_name=cg24617568	feature_id[3282].value <= threshold=0.26932457089424133

node_141: feature_name=cg12614090	feature_id[2765].value <= threshold=0.1813211366534233
node_142: feature_name=cg10917602	feature_id[4685].value <= threshold=0.5744736790657043
node_143: feature_name=cg23723410	feature_id[34].value <= threshold=0.3201078921556473
node_144: feature_name=cg26804423	feature_id[1725].value > threshold=0.39587317407131195
node_146: feature_name=cg20080282	feature_id[1150].value <= threshold=0.856019914150238
node_147: feature_name=cg08924374	feature_id[4125].value > threshold=0.07863279432058334
node_149: feature_name=cg08535779	feature_id[2187].value > threshold=0.24677357077598572
node_151: feature_name=cg00290626	feature_id[4663].value <= threshold=0.6112961769104004
Class: epithelioid sarcoma (ES)	
Rules_163	passed counts:1
node_0: feature_name=cg11915444	feature_id[2002].value > threshold=0.3402601182460785
node_10: feature_name=cg26016985	feature_id[4407].value > threshold=0.6493876278400421
node_16: feature_name=cg23157618	feature_id[5511].value > threshold=0.5759606957435608
node_20: feature_name=cg10480329	feature_id[1439].value > threshold=0.5518321692943573
node_48: feature_name=cg17843418	feature_id[4868].value > threshold=0.34969255328178406
node_52: feature_name=cg24407065	feature_id[4522].value > threshold=0.5364363789558411
node_56: feature_name=cg07281938	feature_id[249].value > threshold=0.7506992518901825
node_78: feature_name=cg21759907	feature_id[1650].value > threshold=0.46823520958423615
node_98: feature_name=cg10886334	feature_id[3975].value > threshold=0.43698127567768097
node_102: feature_name=cg09373983	feature_id[5025].value > threshold=0.5575815737247467
node_106: feature_name=cg12948116	feature_id[5360].value <= threshold=0.5658791363239288
node_107: feature_name=cg21906519	feature_id[5251].value > threshold=0.6089984774589539

node_111: feature_name=cg17537493	feature_id[4217].value > threshold=0.5825372636318207
node_117: feature_name=cg15720017	feature_id[4443].value > threshold=0.7009969055652618
node_119: feature_name=cg06038180	feature_id[3480].value > threshold=0.4583848565816879
node_121: feature_name=cg08619651	feature_id[4882].value <= threshold=0.2105495035648346
node_122: feature_name=cg01014262	feature_id[1911].value <= threshold=0.5327437818050385
node_123: feature_name=cg16863382	feature_id[2922].value > threshold=0.7200668156147003
node_133: feature_name=cg06989443	feature_id[2327].value <= threshold=0.6579365730285645
node_134: feature_name=cg07344990	feature_id[2398].value > threshold=0.5562876164913177
node_138: feature_name=cg02966841	feature_id[2199].value <= threshold=0.7744501233100891
node_139: feature_name=cg06989253	feature_id[194].value <= threshold=0.7899391353130341
node_140: feature_name=cg24617568	feature_id[3282].value <= threshold=0.26932457089424133
node_141: feature_name=cg12614090	feature_id[2765].value <= threshold=0.1813211366534233
node_142: feature_name=cg10917602	feature_id[4685].value <= threshold=0.5744736790657043
node_143: feature_name=cg23723410	feature_id[34].value <= threshold=0.3201078921556473
node_144: feature_name=cg26804423	feature_id[1725].value > threshold=0.39587317407131195
node_146: feature_name=cg20080282	feature_id[1150].value <= threshold=0.856019914150238
node_147: feature_name=cg08924374	feature_id[4125].value > threshold=0.07863279432058334
node_149: feature_name=cg08535779	feature_id[2187].value <= threshold=0.24677357077598572
Class: osteoblastoma (OB)	
Rules_164	passed counts:1
node_0: feature_name=cg11915444	feature_id[2002].value > threshold=0.3402601182460785
node_10: feature_name=cg26016985	feature_id[4407].value > threshold=0.6493876278400421

node_16: feature_name=cg23157618	feature_id[5511].value > threshold=0.5759606957435608
node_20: feature_name=cg10480329	feature_id[1439].value > threshold=0.5518321692943573
node_48: feature_name=cg17843418	feature_id[4868].value > threshold=0.34969255328178406
node_52: feature_name=cg24407065	feature_id[4522].value > threshold=0.5364363789558411
node_56: feature_name=cg07281938	feature_id[249].value > threshold=0.7506992518901825
node_78: feature_name=cg21759907	feature_id[1650].value > threshold=0.46823520958423615
node_98: feature_name=cg10886334	feature_id[3975].value > threshold=0.43698127567768097
node_102: feature_name=cg09373983	feature_id[5025].value > threshold=0.5575815737247467
node_106: feature_name=cg12948116	feature_id[5360].value <= threshold=0.5658791363239288
node_107: feature_name=cg21906519	feature_id[5251].value > threshold=0.6089984774589539
node_111: feature_name=cg17537493	feature_id[4217].value > threshold=0.5825372636318207
node_117: feature_name=cg15720017	feature_id[4443].value > threshold=0.7009969055652618
node_119: feature_name=cg06038180	feature_id[3480].value > threshold=0.4583848565816879
node_121: feature_name=cg08619651	feature_id[4882].value <= threshold=0.2105495035648346
node_122: feature_name=cg01014262	feature_id[1911].value <= threshold=0.5327437818050385
node_123: feature_name=cg16863382	feature_id[2922].value > threshold=0.7200668156147003
node_133: feature_name=cg06989443	feature_id[2327].value <= threshold=0.6579365730285645
node_134: feature_name=cg07344990	feature_id[2398].value <= threshold=0.5562876164913177
node_135: feature_name=cg01329151	feature_id[1681].value <= threshold=0.20543088763952255
Class: desmoplastic small round cell tumour (DSRCT)	
Rules_165	passed counts:1

node_0: feature_name=cg11915444	feature_id[2002].value > threshold=0.3402601182460785
node_10: feature_name=cg26016985	feature_id[4407].value > threshold=0.6493876278400421
node_16: feature_name=cg23157618	feature_id[5511].value > threshold=0.5759606957435608
node_20: feature_name=cg10480329	feature_id[1439].value > threshold=0.5518321692943573
node_48: feature_name=cg17843418	feature_id[4868].value > threshold=0.34969255328178406
node_52: feature_name=cg24407065	feature_id[4522].value > threshold=0.5364363789558411
node_56: feature_name=cg07281938	feature_id[249].value > threshold=0.7506992518901825
node_78: feature_name=cg21759907	feature_id[1650].value > threshold=0.46823520958423615
node_98: feature_name=cg10886334	feature_id[3975].value > threshold=0.43698127567768097
node_102: feature_name=cg09373983	feature_id[5025].value > threshold=0.5575815737247467
node_106: feature_name=cg12948116	feature_id[5360].value <= threshold=0.5658791363239288
node_107: feature_name=cg21906519	feature_id[5251].value > threshold=0.6089984774589539
node_111: feature_name=cg17537493	feature_id[4217].value > threshold=0.5825372636318207
node_117: feature_name=cg15720017	feature_id[4443].value > threshold=0.7009969055652618
node_119: feature_name=cg06038180	feature_id[3480].value > threshold=0.4583848565816879
node_121: feature_name=cg08619651	feature_id[4882].value <= threshold=0.2105495035648346
node_122: feature_name=cg01014262	feature_id[1911].value <= threshold=0.5327437818050385
node_123: feature_name=cg16863382	feature_id[2922].value <= threshold=0.7200668156147003
node_124: feature_name=cg11225330	feature_id[3185].value > threshold=0.7814575433731079
node_126: feature_name=cg15543534	feature_id[423].value <= threshold=0.86179119348526
node_127: feature_name=cg18809729	feature_id[5184].value <= threshold=0.8457489907741547
node_128: feature_name=cg20000539	feature_id[5329].value <= threshold=0.7002188265323639

Class: rhabdomyosarcoma (RMS)	
Rules_166	passed counts:1
node_0: feature_name=cg11915444	feature_id[2002].value > threshold=0.3402601182460785
node_10: feature_name=cg26016985	feature_id[4407].value > threshold=0.6493876278400421
node_16: feature_name=cg23157618	feature_id[5511].value > threshold=0.5759606957435608
node_20: feature_name=cg10480329	feature_id[1439].value > threshold=0.5518321692943573
node_48: feature_name=cg17843418	feature_id[4868].value > threshold=0.34969255328178406
node_52: feature_name=cg24407065	feature_id[4522].value > threshold=0.5364363789558411
node_56: feature_name=cg07281938	feature_id[249].value > threshold=0.7506992518901825
node_78: feature_name=cg21759907	feature_id[1650].value > threshold=0.46823520958423615
node_98: feature_name=cg10886334	feature_id[3975].value > threshold=0.43698127567768097
node_102: feature_name=cg09373983	feature_id[5025].value > threshold=0.5575815737247467
node_106: feature_name=cg12948116	feature_id[5360].value <= threshold=0.5658791363239288
node_107: feature_name=cg21906519	feature_id[5251].value > threshold=0.6089984774589539
node_111: feature_name=cg17537493	feature_id[4217].value <= threshold=0.5825372636318207
node_112: feature_name=cg07260789	feature_id[1074].value > threshold=0.8945248425006866
node_114: feature_name=cg11251006	feature_id[4182].value <= threshold=0.7591733336448669
Class: epithelioid haemangioendothelioma (EHE)	
Rules_167	passed counts:1
node_0: feature_name=cg11915444	feature_id[2002].value > threshold=0.3402601182460785
node_10: feature_name=cg26016985	feature_id[4407].value > threshold=0.6493876278400421
node_16: feature_name=cg23157618	feature_id[5511].value > threshold=0.5759606957435608

node_20: feature_name=cg10480329	feature_id[1439].value > threshold=0.5518321692943573
node_48: feature_name=cg17843418	feature_id[4868].value > threshold=0.34969255328178406
node_52: feature_name=cg24407065	feature_id[4522].value > threshold=0.5364363789558411
node_56: feature_name=cg07281938	feature_id[249].value > threshold=0.7506992518901825
node_78: feature_name=cg21759907	feature_id[1650].value > threshold=0.46823520958423615
node_98: feature_name=cg10886334	feature_id[3975].value > threshold=0.43698127567768097
node_102: feature_name=cg09373983	feature_id[5025].value > threshold=0.5575815737247467
node_106: feature_name=cg12948116	feature_id[5360].value <= threshold=0.5658791363239288
node_107: feature_name=cg21906519	feature_id[5251].value <= threshold=0.6089984774589539
node_108: feature_name=cg05288253	feature_id[559].value <= threshold=0.28334617614746094
Class: solitary fibrous tumour (SFT)	
Rules_168	passed counts:1
node_0: feature_name=cg11915444	feature_id[2002].value > threshold=0.3402601182460785
node_10: feature_name=cg26016985	feature_id[4407].value > threshold=0.6493876278400421
node_16: feature_name=cg23157618	feature_id[5511].value > threshold=0.5759606957435608
node_20: feature_name=cg10480329	feature_id[1439].value > threshold=0.5518321692943573
node_48: feature_name=cg17843418	feature_id[4868].value > threshold=0.34969255328178406
node_52: feature_name=cg24407065	feature_id[4522].value > threshold=0.5364363789558411
node_56: feature_name=cg07281938	feature_id[249].value > threshold=0.7506992518901825
node_78: feature_name=cg21759907	feature_id[1650].value > threshold=0.46823520958423615
node_98: feature_name=cg10886334	feature_id[3975].value > threshold=0.43698127567768097
node_102: feature_name=cg09373983	feature_id[5025].value <= threshold=0.5575815737247467

node_103: feature_name=cg07642499	feature_id[3026].value <= threshold=0.7077974379062653
Class: myxoid liposarcoma (MLS)	
Rules_169	passed counts:1
node_0: feature_name=cg11915444	feature_id[2002].value > threshold=0.3402601182460785
node_10: feature_name=cg26016985	feature_id[4407].value > threshold=0.6493876278400421
node_16: feature_name=cg23157618	feature_id[5511].value > threshold=0.5759606957435608
node_20: feature_name=cg10480329	feature_id[1439].value > threshold=0.5518321692943573
node_48: feature_name=cg17843418	feature_id[4868].value > threshold=0.34969255328178406
node_52: feature_name=cg24407065	feature_id[4522].value > threshold=0.5364363789558411
node_56: feature_name=cg07281938	feature_id[249].value > threshold=0.7506992518901825
node_78: feature_name=cg21759907	feature_id[1650].value > threshold=0.46823520958423615
node_98: feature_name=cg10886334	feature_id[3975].value <= threshold=0.43698127567768097
node_99: feature_name=cg08091706	feature_id[4506].value <= threshold=0.7506741881370544
Class: undifferentiated sarcoma (USARC)	
Rules_170	passed counts:1
node_0: feature_name=cg11915444	feature_id[2002].value > threshold=0.3402601182460785
node_10: feature_name=cg26016985	feature_id[4407].value > threshold=0.6493876278400421
node_16: feature_name=cg23157618	feature_id[5511].value > threshold=0.5759606957435608
node_20: feature_name=cg10480329	feature_id[1439].value > threshold=0.5518321692943573
node_48: feature_name=cg17843418	feature_id[4868].value > threshold=0.34969255328178406
node_52: feature_name=cg24407065	feature_id[4522].value > threshold=0.5364363789558411
node_56: feature_name=cg07281938	feature_id[249].value > threshold=0.7506992518901825

node_78: feature_name=cg21759907	feature_id[1650].value <= threshold=0.46823520958423615
node_79: feature_name=cg25476129	feature_id[2302].value > threshold=0.1687404215335846
node_93: feature_name=cg16318412	feature_id[1590].value > threshold=0.9159156084060669
node_95: feature_name=cg07577934	feature_id[3731].value > threshold=0.7088948488235474
Class: angioleiomyoma (ALMO)/myopericytoma (MPC)	
Rules_171	passed counts:1
node_0: feature_name=cg11915444	feature_id[2002].value > threshold=0.3402601182460785
node_10: feature_name=cg26016985	feature_id[4407].value > threshold=0.6493876278400421
node_16: feature_name=cg23157618	feature_id[5511].value > threshold=0.5759606957435608
node_20: feature_name=cg10480329	feature_id[1439].value > threshold=0.5518321692943573
node_48: feature_name=cg17843418	feature_id[4868].value > threshold=0.34969255328178406
node_52: feature_name=cg24407065	feature_id[4522].value > threshold=0.5364363789558411
node_56: feature_name=cg07281938	feature_id[249].value > threshold=0.7506992518901825
node_78: feature_name=cg21759907	feature_id[1650].value <= threshold=0.46823520958423615
node_79: feature_name=cg25476129	feature_id[2302].value > threshold=0.1687404215335846
node_93: feature_name=cg16318412	feature_id[1590].value > threshold=0.9159156084060669
node_95: feature_name=cg07577934	feature_id[3731].value <= threshold=0.7088948488235474
Class: undifferentiated sarcoma (USARC)	
Rules_172	passed counts:1
node_0: feature_name=cg11915444	feature_id[2002].value > threshold=0.3402601182460785
node_10: feature_name=cg26016985	feature_id[4407].value > threshold=0.6493876278400421
node_16: feature_name=cg23157618	feature_id[5511].value > threshold=0.5759606957435608

node_20: feature_name=cg10480329	feature_id[1439].value > threshold=0.5518321692943573
node_48: feature_name=cg17843418	feature_id[4868].value > threshold=0.34969255328178406
node_52: feature_name=cg24407065	feature_id[4522].value > threshold=0.5364363789558411
node_56: feature_name=cg07281938	feature_id[249].value > threshold=0.7506992518901825
node_78: feature_name=cg21759907	feature_id[1650].value <= threshold=0.46823520958423615
node_79: feature_name=cg25476129	feature_id[2302].value <= threshold=0.1687404215335846
node_80: feature_name=cg26362368	feature_id[414].value <= threshold=0.834072083234787
node_81: feature_name=cg15881332	feature_id[154].value <= threshold=0.7577665150165558
node_82: feature_name=cg14651518	feature_id[1456].value <= threshold=0.7856765985488892
node_83: feature_name=cg08132573	feature_id[40].value > threshold=0.740606278181076
node_85: feature_name=cg09464883	feature_id[3924].value > threshold=0.7645087838172913
node_87: feature_name=cg19509663	feature_id[2011].value > threshold=0.4913187026977539
Class: angioleiomyoma (ALMO)/myopericytoma (MPC)	
Rules_173	passed counts:1
node_0: feature_name=cg11915444	feature_id[2002].value > threshold=0.3402601182460785
node_10: feature_name=cg26016985	feature_id[4407].value > threshold=0.6493876278400421
node_16: feature_name=cg23157618	feature_id[5511].value > threshold=0.5759606957435608
node_20: feature_name=cg10480329	feature_id[1439].value > threshold=0.5518321692943573
node_48: feature_name=cg17843418	feature_id[4868].value > threshold=0.34969255328178406
node_52: feature_name=cg24407065	feature_id[4522].value > threshold=0.5364363789558411
node_56: feature_name=cg07281938	feature_id[249].value > threshold=0.7506992518901825

node_78: feature_name=cg21759907	feature_id[1650].value <= threshold=0.46823520958423615
node_79: feature_name=cg25476129	feature_id[2302].value <= threshold=0.1687404215335846
node_80: feature_name=cg26362368	feature_id[414].value <= threshold=0.834072083234787
node_81: feature_name=cg15881332	feature_id[154].value <= threshold=0.7577665150165558
node_82: feature_name=cg14651518	feature_id[1456].value <= threshold=0.7856765985488892
node_83: feature_name=cg08132573	feature_id[40].value > threshold=0.740606278181076
node_85: feature_name=cg09464883	feature_id[3924].value > threshold=0.7645087838172913
node_87: feature_name=cg19509663	feature_id[2011].value <= threshold=0.4913187026977539
Class: low-grade endometrial stromal sarcoma (ESS (LG))	
Rules_174	passed counts:1
node_0: feature_name=cg11915444	feature_id[2002].value > threshold=0.3402601182460785
node_10: feature_name=cg26016985	feature_id[4407].value > threshold=0.6493876278400421
node_16: feature_name=cg23157618	feature_id[5511].value > threshold=0.5759606957435608
node_20: feature_name=cg10480329	feature_id[1439].value > threshold=0.5518321692943573
node_48: feature_name=cg17843418	feature_id[4868].value > threshold=0.34969255328178406
node_52: feature_name=cg24407065	feature_id[4522].value > threshold=0.5364363789558411
node_56: feature_name=cg07281938	feature_id[249].value > threshold=0.7506992518901825
node_78: feature_name=cg21759907	feature_id[1650].value <= threshold=0.46823520958423615
node_79: feature_name=cg25476129	feature_id[2302].value <= threshold=0.1687404215335846
node_80: feature_name=cg26362368	feature_id[414].value <= threshold=0.834072083234787
node_81: feature_name=cg15881332	feature_id[154].value <= threshold=0.7577665150165558

node_82: feature_name=cg14651518	feature_id[1456].value <= threshold=0.7856765985488892
node_83: feature_name=cg08132573	feature_id[40].value > threshold=0.740606278181076
node_85: feature_name=cg09464883	feature_id[3924].value <= threshold=0.7645087838172913
Class: schwannoma (SWN)	
Rules_175	passed counts:1
node_0: feature_name=cg11915444	feature_id[2002].value > threshold=0.3402601182460785
node_10: feature_name=cg26016985	feature_id[4407].value > threshold=0.6493876278400421
node_16: feature_name=cg23157618	feature_id[5511].value > threshold=0.5759606957435608
node_20: feature_name=cg10480329	feature_id[1439].value > threshold=0.5518321692943573
node_48: feature_name=cg17843418	feature_id[4868].value > threshold=0.34969255328178406
node_52: feature_name=cg24407065	feature_id[4522].value > threshold=0.5364363789558411
node_56: feature_name=cg07281938	feature_id[249].value > threshold=0.7506992518901825
node_78: feature_name=cg21759907	feature_id[1650].value <= threshold=0.46823520958423615
node_79: feature_name=cg25476129	feature_id[2302].value <= threshold=0.1687404215335846
node_80: feature_name=cg26362368	feature_id[414].value <= threshold=0.834072083234787
node_81: feature_name=cg15881332	feature_id[154].value <= threshold=0.7577665150165558
node_82: feature_name=cg14651518	feature_id[1456].value <= threshold=0.7856765985488892
node_83: feature_name=cg08132573	feature_id[40].value <= threshold=0.740606278181076
Class: malignant rhabdoid tumour (MRT)	
Rules_176	passed counts:1
node_0: feature_name=cg11915444	feature_id[2002].value > threshold=0.3402601182460785
node_10: feature_name=cg26016985	feature_id[4407].value > threshold=0.6493876278400421

node_16: feature_name=cg23157618	feature_id[5511].value > threshold=0.5759606957435608
node_20: feature_name=cg10480329	feature_id[1439].value > threshold=0.5518321692943573
node_48: feature_name=cg17843418	feature_id[4868].value > threshold=0.34969255328178406
node_52: feature_name=cg24407065	feature_id[4522].value > threshold=0.5364363789558411
node_56: feature_name=cg07281938	feature_id[249].value <= threshold=0.7506992518901825
node_57: feature_name=cg21189849	feature_id[1261].value > threshold=0.7545044124126434
node_63: feature_name=cg18048309	feature_id[2794].value > threshold=0.36701037734746933
node_65: feature_name=cg05851887	feature_id[4211].value <= threshold=0.7704950571060181
node_66: feature_name=cg22575379	feature_id[2711].value > threshold=0.4320530295372009
node_68: feature_name=cg02053092	feature_id[3610].value > threshold=0.7796373069286346
node_70: feature_name=cg12129983	feature_id[4781].value > threshold=0.649698406457901
node_72: feature_name=cg12011299	feature_id[4765].value > threshold=0.49161551892757416
node_74: feature_name=cg04847386	feature_id[2852].value > threshold=0.8453048467636108
Class: leiomyosarcoma (LMS)	
Rules_177	passed counts:1
node_0: feature_name=cg11915444	feature_id[2002].value > threshold=0.3402601182460785
node_10: feature_name=cg26016985	feature_id[4407].value > threshold=0.6493876278400421
node_16: feature_name=cg23157618	feature_id[5511].value > threshold=0.5759606957435608
node_20: feature_name=cg10480329	feature_id[1439].value > threshold=0.5518321692943573
node_48: feature_name=cg17843418	feature_id[4868].value > threshold=0.34969255328178406
node_52: feature_name=cg24407065	feature_id[4522].value > threshold=0.5364363789558411
node_56: feature_name=cg07281938	feature_id[249].value <= threshold=0.7506992518901825

node_57: feature_name=cg21189849	feature_id[1261].value > threshold=0.7545044124126434
node_63: feature_name=cg18048309	feature_id[2794].value > threshold=0.36701037734746933
node_65: feature_name=cg05851887	feature_id[4211].value <= threshold=0.7704950571060181
node_66: feature_name=cg22575379	feature_id[2711].value > threshold=0.4320530295372009
node_68: feature_name=cg02053092	feature_id[3610].value > threshold=0.7796373069286346
node_70: feature_name=cg12129983	feature_id[4781].value > threshold=0.649698406457901
node_72: feature_name=cg12011299	feature_id[4765].value > threshold=0.49161551892757416
node_74: feature_name=cg04847386	feature_id[2852].value <= threshold=0.8453048467636108
Class: angiomatoid fibrous histiocytoma (AFH)	
Rules_178	passed counts:1
node_0: feature_name=cg11915444	feature_id[2002].value > threshold=0.3402601182460785
node_10: feature_name=cg26016985	feature_id[4407].value > threshold=0.6493876278400421
node_16: feature_name=cg23157618	feature_id[5511].value > threshold=0.5759606957435608
node_20: feature_name=cg10480329	feature_id[1439].value > threshold=0.5518321692943573
node_48: feature_name=cg17843418	feature_id[4868].value > threshold=0.34969255328178406
node_52: feature_name=cg24407065	feature_id[4522].value > threshold=0.5364363789558411
node_56: feature_name=cg07281938	feature_id[249].value <= threshold=0.7506992518901825
node_57: feature_name=cg21189849	feature_id[1261].value > threshold=0.7545044124126434
node_63: feature_name=cg18048309	feature_id[2794].value > threshold=0.36701037734746933
node_65: feature_name=cg05851887	feature_id[4211].value <= threshold=0.7704950571060181
node_66: feature_name=cg22575379	feature_id[2711].value > threshold=0.4320530295372009
node_68: feature_name=cg02053092	feature_id[3610].value > threshold=0.7796373069286346

node_70: feature_name=cg12129983	feature_id[4781].value > threshold=0.649698406457901
node_72: feature_name=cg12011299	feature_id[4765].value <= threshold=0.49161551892757416
Class: angioleiomyoma (ALMO)/myopericytoma (MPC)	
Rules_179	passed counts:1
node_0: feature_name=cg11915444	feature_id[2002].value > threshold=0.3402601182460785
node_10: feature_name=cg26016985	feature_id[4407].value > threshold=0.6493876278400421
node_16: feature_name=cg23157618	feature_id[5511].value > threshold=0.5759606957435608
node_20: feature_name=cg10480329	feature_id[1439].value > threshold=0.5518321692943573
node_48: feature_name=cg17843418	feature_id[4868].value > threshold=0.34969255328178406
node_52: feature_name=cg24407065	feature_id[4522].value > threshold=0.5364363789558411
node_56: feature_name=cg07281938	feature_id[249].value <= threshold=0.7506992518901825
node_57: feature_name=cg21189849	feature_id[1261].value > threshold=0.7545044124126434
node_63: feature_name=cg18048309	feature_id[2794].value > threshold=0.36701037734746933
node_65: feature_name=cg05851887	feature_id[4211].value <= threshold=0.7704950571060181
node_66: feature_name=cg22575379	feature_id[2711].value > threshold=0.4320530295372009
node_68: feature_name=cg02053092	feature_id[3610].value > threshold=0.7796373069286346
node_70: feature_name=cg12129983	feature_id[4781].value <= threshold=0.649698406457901
Class: solitary fibrous tumour (SFT)	
Rules_180	passed counts:1
node_0: feature_name=cg11915444	feature_id[2002].value > threshold=0.3402601182460785
node_10: feature_name=cg26016985	feature_id[4407].value > threshold=0.6493876278400421
node_16: feature_name=cg23157618	feature_id[5511].value > threshold=0.5759606957435608

node_20: feature_name=cg10480329	feature_id[1439].value > threshold=0.5518321692943573
node_48: feature_name=cg17843418	feature_id[4868].value > threshold=0.34969255328178406
node_52: feature_name=cg24407065	feature_id[4522].value > threshold=0.5364363789558411
node_56: feature_name=cg07281938	feature_id[249].value <= threshold=0.7506992518901825
node_57: feature_name=cg21189849	feature_id[1261].value > threshold=0.7545044124126434
node_63: feature_name=cg18048309	feature_id[2794].value > threshold=0.36701037734746933
node_65: feature_name=cg05851887	feature_id[4211].value <= threshold=0.7704950571060181
node_66: feature_name=cg22575379	feature_id[2711].value > threshold=0.4320530295372009
node_68: feature_name=cg02053092	feature_id[3610].value <= threshold=0.7796373069286346
Class: chondroblastoma (CB)	
Rules_181	passed counts:1
node_0: feature_name=cg11915444	feature_id[2002].value > threshold=0.3402601182460785
node_10: feature_name=cg26016985	feature_id[4407].value > threshold=0.6493876278400421
node_16: feature_name=cg23157618	feature_id[5511].value > threshold=0.5759606957435608
node_20: feature_name=cg10480329	feature_id[1439].value > threshold=0.5518321692943573
node_48: feature_name=cg17843418	feature_id[4868].value > threshold=0.34969255328178406
node_52: feature_name=cg24407065	feature_id[4522].value > threshold=0.5364363789558411
node_56: feature_name=cg07281938	feature_id[249].value <= threshold=0.7506992518901825
node_57: feature_name=cg21189849	feature_id[1261].value <= threshold=0.7545044124126434
node_58: feature_name=cg11960033	feature_id[260].value <= threshold=0.7065970599651337
node_59: feature_name=cg18148375	feature_id[1524].value > threshold=0.6905054450035095
Class: fibrous dysplasia (FDY)	

Rules_182	passed counts:1
node_0: feature_name=cg11915444	feature_id[2002].value > threshold=0.3402601182460785
node_10: feature_name=cg26016985	feature_id[4407].value > threshold=0.6493876278400421
node_16: feature_name=cg23157618	feature_id[5511].value > threshold=0.5759606957435608
node_20: feature_name=cg10480329	feature_id[1439].value > threshold=0.5518321692943573
node_48: feature_name=cg17843418	feature_id[4868].value > threshold=0.34969255328178406
node_52: feature_name=cg24407065	feature_id[4522].value <= threshold=0.5364363789558411
node_53: feature_name=cg27049344	feature_id[5181].value > threshold=0.9606902599334717
Class: embryonal rhabdomyosarcoma (RMS (EMB))	
Rules_183	passed counts:1
node_0: feature_name=cg11915444	feature_id[2002].value > threshold=0.3402601182460785
node_10: feature_name=cg26016985	feature_id[4407].value > threshold=0.6493876278400421
node_16: feature_name=cg23157618	feature_id[5511].value > threshold=0.5759606957435608
node_20: feature_name=cg10480329	feature_id[1439].value > threshold=0.5518321692943573
node_48: feature_name=cg17843418	feature_id[4868].value <= threshold=0.34969255328178406
node_49: feature_name=cg06551007	feature_id[1675].value <= threshold=0.44745437800884247
Class: melanoma (MEL)	
Rules_184	passed counts:1
node_0: feature_name=cg11915444	feature_id[2002].value > threshold=0.3402601182460785
node_10: feature_name=cg26016985	feature_id[4407].value > threshold=0.6493876278400421
node_16: feature_name=cg23157618	feature_id[5511].value > threshold=0.5759606957435608
node_20: feature_name=cg10480329	feature_id[1439].value <= threshold=0.5518321692943573

node_21: feature_name=cg25510609	feature_id[149].value > threshold=0.08151235431432724
node_39: feature_name=cg08726900	feature_id[250].value > threshold=0.5245974063873291
node_41: feature_name=cg09040552	feature_id[1285].value > threshold=0.5485720634460449
node_43: feature_name=cg18888464	feature_id[4555].value > threshold=0.0659504197537899
node_45: feature_name=cg16461139	feature_id[25].value > threshold=0.7099440097808838
Class: giant cell tumour of bone (GCTB)	
Rules_185	passed counts:1
node_0: feature_name=cg11915444	feature_id[2002].value > threshold=0.3402601182460785
node_10: feature_name=cg26016985	feature_id[4407].value > threshold=0.6493876278400421
node_16: feature_name=cg23157618	feature_id[5511].value > threshold=0.5759606957435608
node_20: feature_name=cg10480329	feature_id[1439].value <= threshold=0.5518321692943573
node_21: feature_name=cg25510609	feature_id[149].value > threshold=0.08151235431432724
node_39: feature_name=cg08726900	feature_id[250].value > threshold=0.5245974063873291
node_41: feature_name=cg09040552	feature_id[1285].value > threshold=0.5485720634460449
node_43: feature_name=cg18888464	feature_id[4555].value > threshold=0.0659504197537899
node_45: feature_name=cg16461139	feature_id[25].value <= threshold=0.7099440097808838
Class: angiosarcoma (AS)	
Rules_186	passed counts:1
node_0: feature_name=cg11915444	feature_id[2002].value > threshold=0.3402601182460785
node_10: feature_name=cg26016985	feature_id[4407].value > threshold=0.6493876278400421
node_16: feature_name=cg23157618	feature_id[5511].value > threshold=0.5759606957435608
node_20: feature_name=cg10480329	feature_id[1439].value <= threshold=0.5518321692943573

node_21: feature_name=cg25510609	feature_id[149].value > threshold=0.08151235431432724
node_39: feature_name=cg08726900	feature_id[250].value > threshold=0.5245974063873291
node_41: feature_name=cg09040552	feature_id[1285].value > threshold=0.5485720634460449
node_43: feature_name=cg18888464	feature_id[4555].value <= threshold=0.0659504197537899
Class: chondrosarcoma (CSA)	
Rules_187	passed counts:1
node_0: feature_name=cg11915444	feature_id[2002].value > threshold=0.3402601182460785
node_10: feature_name=cg26016985	feature_id[4407].value > threshold=0.6493876278400421
node_16: feature_name=cg23157618	feature_id[5511].value > threshold=0.5759606957435608
node_20: feature_name=cg10480329	feature_id[1439].value <= threshold=0.5518321692943573
node_21: feature_name=cg25510609	feature_id[149].value > threshold=0.08151235431432724
node_39: feature_name=cg08726900	feature_id[250].value > threshold=0.5245974063873291
node_41: feature_name=cg09040552	feature_id[1285].value <= threshold=0.5485720634460449
Class: undifferentiated sarcoma (USARC)	
Rules_188	passed counts:1
node_0: feature_name=cg11915444	feature_id[2002].value > threshold=0.3402601182460785
node_10: feature_name=cg26016985	feature_id[4407].value > threshold=0.6493876278400421
node_16: feature_name=cg23157618	feature_id[5511].value > threshold=0.5759606957435608
node_20: feature_name=cg10480329	feature_id[1439].value <= threshold=0.5518321692943573
node_21: feature_name=cg25510609	feature_id[149].value <= threshold=0.08151235431432724
node_22: feature_name=cg23850277	feature_id[2159].value <= threshold=0.5682950615882874
node_23: feature_name=cg01259126	feature_id[3146].value > threshold=0.34355829656124115

node_25: feature_name=cg07034004	feature_id[3345].value <= threshold=0.20594919472932816
node_26: feature_name=cg12895304	feature_id[651].value > threshold=0.9125175774097443
node_28: feature_name=cg02235663	feature_id[785].value > threshold=0.4301222711801529
node_30: feature_name=cg05242371	feature_id[687].value > threshold=0.46140024065971375
node_32: feature_name=cg20668718	feature_id[4160].value > threshold=0.3259790688753128
node_34: feature_name=cg21740826	feature_id[2704].value > threshold=0.7765077352523804
Class: inflammatory myofibroblastic tumour (IMT)	
Rules_189	passed counts:1
node_0: feature_name=cg11915444	feature_id[2002].value > threshold=0.3402601182460785
node_10: feature_name=cg26016985	feature_id[4407].value > threshold=0.6493876278400421
node_16: feature_name=cg23157618	feature_id[5511].value > threshold=0.5759606957435608
node_20: feature_name=cg10480329	feature_id[1439].value <= threshold=0.5518321692943573
node_21: feature_name=cg25510609	feature_id[149].value <= threshold=0.08151235431432724
node_22: feature_name=cg23850277	feature_id[2159].value <= threshold=0.5682950615882874
node_23: feature_name=cg01259126	feature_id[3146].value > threshold=0.34355829656124115
node_25: feature_name=cg07034004	feature_id[3345].value <= threshold=0.20594919472932816
node_26: feature_name=cg12895304	feature_id[651].value > threshold=0.9125175774097443
node_28: feature_name=cg02235663	feature_id[785].value > threshold=0.4301222711801529
node_30: feature_name=cg05242371	feature_id[687].value > threshold=0.46140024065971375
node_32: feature_name=cg20668718	feature_id[4160].value > threshold=0.3259790688753128
node_34: feature_name=cg21740826	feature_id[2704].value <= threshold=0.7765077352523804
Class: undifferentiated sarcoma (USARC)	

Rules_190	passed counts:1
node_0: feature_name=cg11915444	feature_id[2002].value > threshold=0.3402601182460785
node_10: feature_name=cg26016985	feature_id[4407].value > threshold=0.6493876278400421
node_16: feature_name=cg23157618	feature_id[5511].value > threshold=0.5759606957435608
node_20: feature_name=cg10480329	feature_id[1439].value <= threshold=0.5518321692943573
node_21: feature_name=cg25510609	feature_id[149].value <= threshold=0.08151235431432724
node_22: feature_name=cg23850277	feature_id[2159].value <= threshold=0.5682950615882874
node_23: feature_name=cg01259126	feature_id[3146].value > threshold=0.34355829656124115
node_25: feature_name=cg07034004	feature_id[3345].value <= threshold=0.20594919472932816
node_26: feature_name=cg12895304	feature_id[651].value > threshold=0.9125175774097443
node_28: feature_name=cg02235663	feature_id[785].value > threshold=0.4301222711801529
node_30: feature_name=cg05242371	feature_id[687].value > threshold=0.46140024065971375
node_32: feature_name=cg20668718	feature_id[4160].value <= threshold=0.3259790688753128
Class: clear cell sarcoma of soft parts (CCS)	
Rules_191	passed counts:1
node_0: feature_name=cg11915444	feature_id[2002].value > threshold=0.3402601182460785
node_10: feature_name=cg26016985	feature_id[4407].value > threshold=0.6493876278400421
node_16: feature_name=cg23157618	feature_id[5511].value > threshold=0.5759606957435608
node_20: feature_name=cg10480329	feature_id[1439].value <= threshold=0.5518321692943573
node_21: feature_name=cg25510609	feature_id[149].value <= threshold=0.08151235431432724
node_22: feature_name=cg23850277	feature_id[2159].value <= threshold=0.5682950615882874
node_23: feature_name=cg01259126	feature_id[3146].value > threshold=0.34355829656124115

node_25: feature_name=cg07034004	feature_id[3345].value <= threshold=0.20594919472932816
node_26: feature_name=cg12895304	feature_id[651].value > threshold=0.9125175774097443
node_28: feature_name=cg02235663	feature_id[785].value > threshold=0.4301222711801529
node_30: feature_name=cg05242371	feature_id[687].value <= threshold=0.46140024065971375
Class: epithelioid sarcoma (ES)	
Rules_192	passed counts:1
node_0: feature_name=cg11915444	feature_id[2002].value > threshold=0.3402601182460785
node_10: feature_name=cg26016985	feature_id[4407].value > threshold=0.6493876278400421
node_16: feature_name=cg23157618	feature_id[5511].value <= threshold=0.5759606957435608
node_17: feature_name=cg23477348	feature_id[3774].value <= threshold=0.19775588810443878
Class: sarcoma (SARC)	
Rules_193	passed counts:1
node_0: feature_name=cg11915444	feature_id[2002].value > threshold=0.3402601182460785
node_10: feature_name=cg26016985	feature_id[4407].value <= threshold=0.6493876278400421
node_11: feature_name=cg14769121	feature_id[1178].value > threshold=0.8216802477836609
node_13: feature_name=cg01254644	feature_id[4106].value > threshold=0.5698081851005554
Class: Kaposi sarcoma (KS)	
Rules_194	passed counts:1
node_0: feature_name=cg11915444	feature_id[2002].value > threshold=0.3402601182460785
node_10: feature_name=cg26016985	feature_id[4407].value <= threshold=0.6493876278400421
node_11: feature_name=cg14769121	feature_id[1178].value > threshold=0.8216802477836609
node_13: feature_name=cg01254644	feature_id[4106].value <= threshold=0.5698081851005554
Class: dermatofibrosarcoma protuberans (DFSP)	

Rules_195	passed counts:1
node_0: feature_name=cg11915444	feature_id[2002].value <= threshold=0.3402601182460785
node_1: feature_name=cg17156862	feature_id[2598].value <= threshold=0.6409126222133636
node_2: feature_name=cg08947774	feature_id[1302].value <= threshold=0.6231959313154221
node_3: feature_name=cg10370025	feature_id[4047].value > threshold=0.7934649586677551
node_5: feature_name=cg18653451	feature_id[4856].value > threshold=0.18988156504929066
Class: angiosarcoma (AS)	
Rules_196	passed counts:1
node_0: feature_name=cg11915444	feature_id[2002].value <= threshold=0.3402601182460785
node_1: feature_name=cg17156862	feature_id[2598].value <= threshold=0.6409126222133636
node_2: feature_name=cg08947774	feature_id[1302].value <= threshold=0.6231959313154221
node_3: feature_name=cg10370025	feature_id[4047].value > threshold=0.7934649586677551
node_5: feature_name=cg18653451	feature_id[4856].value <= threshold=0.18988156504929066
Class: chordoma (CHORD)	
Rules_197	passed counts:1
node_0: feature_name=cg11915444	feature_id[2002].value <= threshold=0.3402601182460785
node_1: feature_name=cg17156862	feature_id[2598].value <= threshold=0.6409126222133636
node_2: feature_name=cg08947774	feature_id[1302].value <= threshold=0.6231959313154221
node_3: feature_name=cg10370025	feature_id[4047].value <= threshold=0.7934649586677551
Class: infantile fibrosarcoma (IFS)	
